# Site-specific *O*-Glycosylation Analysis of Human Blood Plasma Proteins*[Fn FN2]

**DOI:** 10.1074/mcp.M115.053546

**Published:** 2015-11-23

**Authors:** Marcus Hoffmann, Kristina Marx, Udo Reichl, Manfred Wuhrer, Erdmann Rapp

**Affiliations:** From the ‡Max Planck Institute for Dynamics of Complex Technical Systems, Bioprocess Engineering, 39106 Magdeburg, Germany;; §Bruker Daltonik GmbH, 28359 Bremen, Germany;; ¶Otto von Guericke University Magdeburg, Chair of Bioprocess Engineering, 39106 Magdeburg, Germany;; ‖Center for Proteomics and Metabolomics, Department of Rheumatology, Leiden University Medical Center, 2300 RC Leiden, The Netherlands;; **glyXera GmbH, Leipziger Strasse 44 (Zenit), 39120 Magdeburg, Germany

## Abstract

Site-specific glycosylation analysis is key to investigate structure-function relationships of glycoproteins, *e.g.* in the context of antigenicity and disease progression. The analysis, though, is quite challenging and time consuming, in particular for *O*-glycosylated proteins. In consequence, despite their clinical and biopharmaceutical importance, many human blood plasma glycoproteins have not been characterized comprehensively with respect to their *O*-glycosylation. Here, we report on the site-specific *O*-glycosylation analysis of human blood plasma glycoproteins. To this end pooled human blood plasma of healthy donors was proteolytically digested using a broad-specific enzyme (Proteinase K), followed by a precipitation step, as well as a glycopeptide enrichment and fractionation step via hydrophilic interaction liquid chromatography, the latter being optimized for intact *O*-glycopeptides carrying short mucin-type core-1 and -2 *O*-glycans, which represent the vast majority of *O*-glycans on human blood plasma proteins. Enriched *O*-glycopeptide fractions were subjected to mass spectrometric analysis using reversed-phase liquid chromatography coupled online to an ion trap mass spectrometer operated in positive-ion mode. Peptide identity and glycan composition were derived from low-energy collision-induced dissociation fragment spectra acquired in multistage mode. To pinpoint the *O*-glycosylation sites glycopeptides were fragmented using electron transfer dissociation. Spectra were annotated by database searches as well as manually. Overall, 31 *O*-glycosylation sites and regions belonging to 22 proteins were identified, the majority being acute-phase proteins. Strikingly, also 11 novel *O*-glycosylation sites and regions were identified. In total 23 *O*-glycosylation sites could be pinpointed. Interestingly, the use of Proteinase K proved to be particularly beneficial in this context. The identified *O*-glycan compositions most probably correspond to mono- and disialylated core-1 mucin-type *O*-glycans (T-antigen). The developed workflow allows the identification and characterization of the major population of the human blood plasma *O*-glycoproteome and our results provide new insights, which can help to unravel structure-function relationships. The data were deposited to ProteomeXchange PXD003270.

Human blood plasma harbors arguably the most complex yet also the most informative proteome present in the human body (1). A significant impact on its clinical relevance and diagnostic potential is attributed to the features and functions of a plethora of proteins (60–80 mg protein per ml plasma), covering a dynamic concentration range of more than ten orders of magnitude (2). The majority, that is 99%, of these proteins are classical blood plasma proteins, like albumins, (immuno)globulins, clotting factors, and proteins of the complement system; however, also a lower abundant but—no less meaningful—fraction of nonclassical proteins is present that comprises a multitude of cytokines as well as tissue leakage proteins. Several clinical studies could show that qualitative and quantitative alterations of these proteins (and peptides)—analyzed individually or in their entirety as a proteome (or peptidome)—can directly reflect pathophysiological states, and can serve as biomarkers for the onset and progression of a number of diseases (3–5). In recent years the focus of in-depth analyses of the human blood plasma proteome has evolved from the identification and quantification of the entire proteome (or peptidome) (6–10) toward the analysis of subproteomes like the interactome (11), phosphoproteome (12, 13) or the glycoproteome (14). The latter has received particular interest in recent years, because the majority of blood plasma proteins is *N*- and/or *O*-glycosylated (2). Although the comprehensive analysis of the *N*-glycoproteome is already quite advanced (15), even in complex samples like human blood plasma (16, 17), similar analyses of the *O*-glycoproteome - though arguably equally important and relevant - are still lagging behind. The most ubiquitously found and functionally relevant form of *O*-glycosylation, as shown by a number of *O*-glycan-related (clinical) studies (18–23), is the mucin-type *O*-glycosyation (*O*-GalNAc), in particular the core-1 and core-2 types (24, 25). The predominantly clustered occurrence of mucin-type *O*-glycans on proteins is described to confer overall stability and proteolytic protection (26). Apart from this global impact, recent studies could link the presence of *O*-glycans in the proximity of regulatory domains to proteolysis events involved in protein maturation (proprotein-convertase-processing) (27). To better understand these protective and regulatory capabilities and to move the mucin-type *O*-glycoproteome from form to function comprehensive site-specific *O*-glycosylation analyses are required.

One of the main obstacles in site-specific mucin-type *O*-glycosylation analyses relates to the lack of a predictable *O*-glycan consensus-motif within the peptide backbone as it can be found for *N*-glycans (28). The initial attachment of the *N*-acetylgalactosamine monosaccharide to the hydroxyl group of either serine or threonine, but also to tyrosine or hydroxylysine, is governed by a family of 20 distinct polypeptide GalNAc-transferase isoenzymes (GalNAc-Ts) with different but partially overlapping peptide specificities and tissue expression patterns. This dynamic regulation, in turn, contributes to the complexity of the mucin-type *O*-glycoproteome. However, previous studies could show that mucin-type *O*-glycans are primarily attached to serine or threonine in regions with a high content of serine, threonine and proline (Ser/Thr-X-X-Pro, Ser/Thr-P and Pro-Ser/Thr) (29, 30). As *O*-glycosylation is a postfolding event, taking place in the Golgi apparatus, the attachment is depended on protein surface accessibility and is thus predominantly found in coil, turn, and linker regions (31). Additional confounding factors during mucin-type *O*-glycosylation analyses are the clustered occurrence of *O*-glycans and the lack of a universal endo-*O*-glycosidase that enables the release of intact *O*-glycans from the proteins; though, chemical *O*-glycan release methods do exist (28).

Mass spectrometry has proven to be the core technique in site-specific *N*- and *O*-glycosylation analyses. A generic *O*-glycoproteomic workflow usually starts with the isolation, enrichment or prefractionation of a single glycoprotein or a group of glycoproteins. In subsequent steps, (glyco)peptides are generated by proteolytic digestion primarily using specific proteases like trypsin. Apart from this, also broad- and nonspecific proteases like Proteinase K or Pronase E were successfully employed in recent years (32–34). Essential to nearly every glycoproteomic approach is the removal of high-abundant and interfering nonglycosylated peptides by selective enrichment of the usually lower abundant glycopeptides. The repertoire of glycopeptide enrichment and separation techniques covers different solid phase extraction and chromatography based methods such as hydrophilic liquid interaction chromatography (HILIC) (35, 36). The most frequently used setup for the measurement of enriched (glyco)peptides is liquid chromatography (LC)^1^ coupled online to electrospray ionization tandem mass spectrometry (LC-ESI-MS/MS). Recent advances in instrumentation, in particular the development of electron-transfer/electron-capture dissociation (ETD/ECD) (37, 38), and high resolution orbital mass analyzers, have paved the way for the mapping of thousands of occupied *N*- and *O*-glycosylation sites as recently shown (17, 27). Combined workflows using ETD/ECD fragmentation along with (multistage, MS^n^) fragmentation with high- and/or low collisional induced dissociation energy (HCD/CID) can provide compositional (structural) information on the glycan moiety as well as information on the peptide sequence and the glycosylation site (39, 40). Recent advances in mass spectrometry driven *O*-glycoproteomics have been reviewed in detail elsewhere (41, 42). Owing to the amount and complexity of *O*-glycoproteomic data a number of bioinformatic software tools for the prediction of mucin-type *O*-glycosylation sites (27) as well as for the database assisted interpretation and annotation of glycan and glycopeptide fragment spectra have been developed (43, 44). Moreover, reporting guidelines for collecting, sharing, integrating, and interpreting mass spectrometry based glycomics data have been specified by the MIRAGE consortium (minimum information required for a glycomics experiment) (45, 46).

The aim of our study was to develop a glycoproteomic workflow that allows the explorative nontargeted analysis of *O*-glycosylated human blood plasma proteins, which are known to carry mainly short mono- and disialylated mucin-type core-1 and -2 *O*-glycans. To achieve this, we have combined *O*-glycopeptide selective offline-HILIC fractionation of Proteinase K digested peptides with nano-reversed-phase liquid chromatography coupled online to multistage ion-trap mass spectrometry (nanoRP-LC-ESI-IT-MS: CID-MS^2^/-MS^3^, ETD-MS^2^). The workflow has been applied to investigate the mucin-type *O*-glycoproteome of a pooled blood plasma sample derived from 20 healthy donors. Based on the mass spectrometric analysis of intact *O*-glycopeptides, we were able to characterize the *O*-glycosylation (*i.e.* peptide, site, and attached *O*-glycans) of a number of major human blood glycoproteins, including many acute phase proteins such as fibrinogen and plasminogen. Overall, the site-specific glycosylation analysis of human blood plasma glycopeptides revealed exclusively mono- and disialylated core-1 mucin-type *O*-glycopeptides. Interestingly, also a few novel *O*-glycosylation sites could be identified.

## EXPERIMENTAL PROCEDURES

All chemicals and solvents were of the highest purity available. Purified water used for sample preparation and HILIC fractionation was freshly prepared using a Milli-Q water purification system (referred to as “Milli-Q water”, 18.2 mΩ × cm^−1^ at 25 °C, Total Organic Carbon 3 ppb; Merck Millipore, Darmstadt, Germany). For preparation of LC-MS solvents ultrapure water was used, which was freshly prepared using the same system but equipped with an additional filter (referred to as “Milli-Q water MS”; LC-Pak Polisher, Merck Millipore).

### 

#### 

##### Sample Preparation

Human blood plasma (pooled sample, derived from 20 healthy donors) was purchased from Affinity Biologicals Inc. (VisuCon-F, Frozen Normal Control Blood, FRNCP0125; Ancaster, ON, Canada). To 25 μl of the sample (about 2 mg protein) 25 μl 100 mm ammonium bicarbonate_(aq)_ (NH_4_HCO_3_, pH 8.0) (Sigma Aldrich, Steinheim, Germany) was added to obtain a final concentration of 50 mm NH_4_HCO_3(aq)_ (pH 8.0). Disulfide bonds were reduced by addition of 6.25 μl 100 mm 1,4-dithiothreitol (DTT; Sigma Aldrich) dissolved in 50 mm NH_4_HCO_3(aq)_ (pH 8.0), to a final concentration of 10 mm DTT. The sample was incubated for 45 min at 60 °C, and subsequently allowed to cool down to room temperature. Cystein alkylation was achieved by addition of 12.5 μl 100 mm iodoacetamide (IAA; Sigma Aldrich) dissolved in 50 mm NH_4_HCO_3(aq)_ (pH 8.0), to a final concentration of 16.67 mm IAA. The sample was incubated at room temperature for 20 min under light exclusion. The alkylation reaction was quenched by addition of 2.5 μl 100 mm DTT dissolved in 50 mm NH_4_HCO_3(aq)_ (pH 8.0), followed by addition of 3.75 μl 50 mm NH_4_HCO_3(aq)_ (pH 8.0), before placing the sample under a fluorescent lamp (gas-discharge lamp) for 15 min to decompose the light-sensitive IAA. By adding 169 μl 50 mm NH_4_HCO_3(aq)_ (pH 8.0) the sample was brought to a final volume of 250 μl.

##### Proteinase K Digestion

Proteolytic digestion was achieved by addition of Proteinase K (Sigma Aldrich), a serine protease with a broad specificity that cleaves primarily after aliphatic, aromatic and hydrophobic amino acids. The pooled blood plasma sample (about 2 mg protein in 250 μl buffer) was supplemented with 66 μg Proteinase K dissolved in 122 μl 50 mm NH_4_HCO_3(aq)_ (pH 8.0) in order to obtain a final enzyme/protein ratio of 1:30 (w/w, 0.033 mg enzyme per mg protein). The sample was incubated for 16 h at 37 °C with gentle agitation (200 rpm).

##### Acetonitrile Precipitation

For post-digestion cleanup the sample was precipitated using acetonitrile (ACN; Sigma Aldrich). To this end four volumes of ACN were added and the sample was centrifuged for 10 min at 2880 × *g* (Centrifuge 5804 R; Eppendorf, Hamburg, Germany). The supernatant was transferred and dried by vacuum centrifugation (RVC 2–33 CDplus, ALPHA 2–4 LDplus; Martin Christ GmbH, Osterode am Harz, Germany).

##### Glycopeptide Enrichment and Fractionation via HILIC-HPLC

The dried Proteinase K digest was resuspended in 500 μl 80% ACN in 50 mm NH_4_HCO_3(aq)_ (v/v, pH 8.0) and subsequently centrifuged for 10 min at 20,238 × *g* to remove any particles (Centrifuge 5424; Eppendorf). The supernatant, containing about 2 mg peptides and glycopeptides, was subjected to HILIC-HPLC (UltiMate™ Nano HPLC-System: Thermo Scientific/Dionex, Dreieich, Germany; HILIC Column: ACQUITY UPLC BEH HILIC Column, 130Å, 1.7 μm, 2.1 mm X 100 mm; Waters, Manchester, UK) for fractionation and glycopeptide enrichment.

The HPLC system was operated using a binary gradient of 100% ACN (v/v; solvent A) and 50 mm ammonium formate_(aq)_ (NH_4_FA, pH 4.4; solvent B, Sigma Aldrich). After sample injection (500 μl) 20% solvent B was applied isocratically for 5 min, followed by a linear gradient to 50% solvent B within 25 min, both using a constant flow rate of 250 μl/min. Subsequently, a linear gradient went to 90% solvent B within 1 min, while reducing the flow rate to 150 μl/min. To wash the column solvent B was kept at 90% for 9 min. Column re-equilibration was achieved by isocratic elution with 20% solvent B for 20 min; (the flow rate was increased to 250 μl/min after 10 min). During the separation the column temperature was kept constant at 40 °C. The elution profile was monitored by UV absorption at 214 nm. Fractions were collected every 2 mins from 0 min to 34 min. The fractions were dried by vacuum centrifugation and reconstituted in 50 μl Milli-Q water.

##### nanoRP-LC-ESI-IT-MS^n^ (CID,ETD)

HILIC fractions were analyzed by reversed-phase nano-LC-MS^n^ using an Ultimate3000 nanoHPLC system (Thermo Scientific/Dionex) coupled online to an ion trap mass spectrometer (AmaZon ETD, Bruker Daltonics, Bremen, Germany). Within the first 2 mins after sample injection, (glyco)peptides were loaded isocratically on a C18 μ-precolumn (Acclaim PepMap100, C18, 5 μm, 100 Å, 300 μm i.d. × 5 mm; Thermo Scientific/Dionex). During this pre-concentration and desalting step, “loading pump solvent 1” (98% Milli-Q water MS, 2% ACN, 0.05% trifluoroacetic acid (Sigma Aldrich)) was used at a flow rate of 7 μl/min. Subsequently, the C18 μ-precolumn was switched in line with the C18 nano-separation column (Acclaim PepMap RSLC, C18, 2 μm, 100 Å, 75 μm i.d. × 15 cm; Thermo Scientific/Dionex) for gradient elution. Here, the following solvents were used at a constant flow rate of 300 nL/min: “A” (98% Milli-Q water MS, 2% ACN, 0.1% formic acid (Sigma Aldrich)); “B” (10% Milli-Q water MS, 10% 2,2,2-trifluoroethanol (Merck), 80% ACN, 0.1% formic acid (Sigma Aldrich)). A binary gradient was applied as follows: 4% B for 2 min; linear gradient to 30% B within 30 min; isocratic washing step at 90% B for 5 min, finally 20 min re-equilibration at 4% B. After 42 min the C18 μ-precolumn was switched back into loading-pump flow, in order to be washed for 3 min at 100% “loading pump solvent 2” (20% Milli-Q water MS, 80% ACN, 0.05% trifluoroacetic acid (Sigma Aldrich)), and eventually to be re-equilibrated for 15 min at 100% “loading pump solvent 1,” both at 7 μl/min flow rate.

The ion trap mass spectrometer was interfaced with a nanoFlow ESI Sprayer (Bruker Daltonics) and was operated in positive ion mode. For electrospray ionization the following parameters were used: capillary voltage (-4,500 V), end plate offset (-500 V), N_2_ dry gas (5 L/min), nebulizer (8 psi), dry gas temperature (220 °C). The (glyco)peptides were fragmented via CID using multistage fragmentation (CID-MS^2^, CID-MS^3^ experiments) and ETD-MS^2^. For negative-mode chemical ionization during ETD measurements methane was supplied at 4 bar.

CID experiments were carried out using the following precursor scan settings: precursor scan mass range (*m*/*z* 100–2500); ion charge control (ICC) target (300, 000); maximum accumulation time (200 ms); averages (5); rolling averaging (off); target mass for smart parameter settings (*m*/*z* 850). CID-MS^2^ experiments were conducted using a data dependent fragmentation routine. The top four most intense precursor ions, in the range of *m*/*z* 500–1500, were subjected to CID fragmentation in the ion trap mass analyzer (MS/MS fragmentation amplitude 1.20 V). The relative intensity threshold for fragmentation was set to 5%. Singly charged ions were excluded and selected precursors were actively excluded for 0.15 min after acquiring two fragment spectra. Charge state preference was set to “none.” Recorded scan range, ICC target and maximum accumulation time were the same as for the precursor scan. In CID-MS^3^ experiments precursor selection and fragmentation was applied manually. The fragmentation amplitude was set to 1.20 V. The recorded scan range was set individually with respect to the *m*/*z* of the precursor. ICC target and maximum accumulation time were the same as for the precursor scan. In both CID-MS^2^ and CID-MS^3^ experiments the following CID parameters were used: cut-off selection (default); smart fragmentation (on); start amplitude (30%); end amplitude (200%); reaction time (40 ms). All CID experiments were carried out using the enhanced resolution mode. For CID-MS^2^ measurements 1 μl of each HILIC fraction was injected. For CID-MS^3^ measurements 5 μl were used, respectively.

ETD experiments were carried out using the following precursor scan settings: precursor scan mass range (*m*/*z* 400–2500); ICC target (200, 000); max. accu. Time (50 ms); averages (5); rolling averaging (on, number: 1); target mass for smart parameter settings (*m*/*z* 850), enhanced resolution mode. Fragment spectra were acquired using a data dependent fragmentation routine in the ultrascan mode. The top three most intense precursor ions were subjected to ETD fragmentation in the ion trap mass analyzer. The relative intensity threshold for fragmentation was set to 1%. Singly charged ions were excluded and selected precursors were actively excluded for 0.15 min after acquiring two fragment spectra. Charge state preference was set to “none”. Fragment ions between *m*/*z* 100–3000 were detected. ICC target was set to 400,000 and max. accu. time was set to 100 ms. The following parameters were used for the EDT reagent: ICC target (500,000); max. accu. time (10 ms); Remove ≤ *m*/*z* 210 (On); Max. ETD Precursor (*m*/*z* 1200), cut-off (*m*/*z* 160); reaction time (160 ms); smart decomposition (auto). For ETD measurements 1 μl of each HILIC fraction was injected.

All MS parameters were tested and optimized using *N*-glycopeptides derived from human IgG (sample preparation according to Selman *et al.* (47)) as well as *O*-glycopeptides from erythropoietin (Protea Biosciences, Morgantown, WV) (data not shown).

##### Data Analysis

Two separate strategies were followed for the spectra analysis. The first approach focuses exclusively on the identification of nonglycosylated peptides, whereas the second approach aims for the characterization and identification of the glycopeptides.

##### Analysis of Nonglycosylated Peptides

Fragment spectra (MS^2^) acquired with CID and ETD were searched for nonglycosylated peptides. To this end spectra were processed in DataAnalysis software 4.0 (Bruker Daltonics) using a built-in function for MS^n^ spectra processing (“processautomsn”; compound detection: standard settings). Processed spectra were imported into ProteinScape 3.1 (Bruker Daltonics) and were searched against a UniProtKB/Swiss-Prot database (SwissProt 51.6; 257964 sequences; 93947433 residues; downloaded February, 2013) using MASCOT version 2.2.04 (Matrix Science, London, UK). The following search parameters were applied: taxonomy (human); enzyme (none); fixed modifications (carbamidomethylation of cysteine residues); variable modifications (deamidation of asparagine and/or glutamine; methionine oxidation); precursor ion mass tolerance (±0.3 Da, with #^13^C = 1; monoisotopic mass); fragment ion mass tolerance (CID: ±0.5 Da; ETD: ±1.3 Da); preferred charge state (2^+^/3^+^); peptide decoy search (1% FDR). Proteins and peptides with a MASCOT ion score higher than 50 and 25 were accepted, respectively.

##### Analysis of Glycopeptides

CID and ETD fragment spectra (MS^2^/MS^3^) were manually analyzed assisted by the DataAnalysis software 4.0 (Bruker Daltonics) without any pre-processing. Fragmentation of glycopeptides using low-energy CID almost exclusively yields fragment ions derived from the glycan moiety. This allows filtering of CID-MS^2^ spectra for the presence of low-molecular weight fragment ions derived from the nonreducing end of the glycan (48) (B-ions, oxonium ions; [M+H]^+^; *e.g.* Hex {*m*/*z* 163.06}; NeuAc -H_2_O {*m*/*z* 274.09}; NeuAc {*m*/*z* 292.10}; Hex_1_NeuAc_1_ {*m*/*z* 454.16}; HexNAc_1_Hex_1_NeuAc_1_ {*m*/*z* 657.24}; tolerance: *m*/*z* ±0.3) using dedicated extracted ion chromatograms (EICs). In addition to this CID-MS^2^ glycopeptide spectra feature multiply charged fragment ions (Y-ions) that show characteristic mono(oligo)-saccharide mass differences caused by the consecutive fragmentation of the glycan moiety down to the deglycosylated peptide. Both features were used to deduce the glycan composition along with the putative peptide mass in CID-MS^2^ glycopeptide spectra. To identify the peptide moiety the putative peptide mass was used to trigger manual CID-MS^3^ fragmentation in a separate run. In rare cases the peptide mass with an additional HexNAc had to be used for CID-MS^3^ fragmentation. CID-MS^3^ fragment spectra were exported to BioTools software 3.2 (Bruker Daltonics). Subsequent peptide identification was conducted using MASCOT. The spectra were searched against a UniProtKB/Swiss-Prot database using the following parameters: taxonomy (human); enzyme (none); fixed modifications (carbamidomethylation of cysteine residues); variable modifications (deamidation of asparagine and/or glutamine; methionine oxidation); precursor ion mass tolerance (±0.3 Da, with #^13^C = 1; monoisotopic mass); fragment ion mass tolerance (CID: ±0.35 Da); preferred charge state (2^+^/3^+^); MASCOT significance threshold (0.05); maximum number of reported hits: 10.

**Fig. 1.**
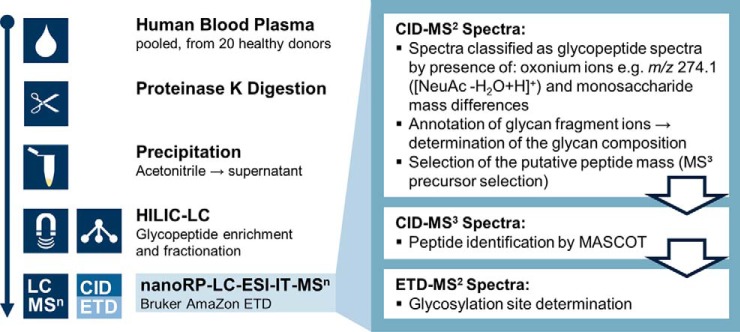
**Left: *O*-Glycoproteomic workflow for the analysis of human blood plasma glycoproteins.** Right: LC-MS^n^ measurement and data analysis workflow. RP = reversed-phase; CID = Collision induced dissociation; ETD = Electron transfer dissociation.

Peptides with a MASCOT ion score greater than 20 were considered; in very rare cases also lower scored peptides were accepted. Peptide identification was supported by the presence of a glycosylation consensus motif within the putative peptide sequence (*N*-glycosylation: Asn-X-Ser/Thr; *O*-glycosylation: Ser/Thr). Furthermore, knowledge, derived from public databases (UniProtKB and UniCarbKB) on already described *N*-/*O*-glycosylation sites within the putative peptide sequence or within the entire protein, was used to validate a peptide/protein hit. ETD-MS^2^ fragment spectra of identified and characterized glycopeptides were annotated manually with respect to the presence of glycan fragment ions (Y-ions). Subsequently, the spectra were exported to BioTools software 3.2 (Bruker Daltonics) to identify the glycosylation site(s). The peptide sequences, proposed by CID-MS^3^ measurements, were modified *in silico* with the corresponding glycan compositions inferred from CID-MS^2^, taking into account all the potential glycosylation sites. Fragment ions (c- and z-type ions) derived from these *in silico* glycopeptide sequences were then matched to their counterparts in the measured ETD-MS^2^ spectra. The accuracy of this annotation was validated using the BioTools score along with manual inspection of the respective spectra. The entire glycopeptide data analysis workflow is briefly summarized in Fig. 1.

All mass spectrometry raw data as well as (glyco)peptide identifications and spectra annotations have been deposited to the ProteomeXchange Consortium (http://proteomecentral.proteomexchange.org)via the MassIVE repository with the dataset identifier PXD002315or MSV000079141, respectively.

## RESULTS

Central to this study is the explorative, nontargeted analysis of *O*-glycosylated blood plasma glycoproteins. To this end a glycoproteomics approach was applied, that includes the identification of the peptide moiety as well as a characterization and localization of the *O*-glycosylation sites with the characterization of the corresponding *O*-glycans. HILIC enriched glycopeptides derived from a broad-specific proteolytic digest of human blood plasma proteins were analyzed by reversed-phase liquid chromatography combined with multistage mass spectrometry (CID-MS^2^/-MS^3^, ETD-MS^2^ fragmentation).

### 

#### 

##### Reproducibility of the Proteinase K Digest

Previous studies on single glycoproteins could show the successful application of Proteinase K in the context of *N*- and *O*-glycoproteomics (32, 49–52). However, its application on complex samples, like human blood plasma, has not been described so far. Here, we have employed Proteinase K to generate (glyco)peptides from the entire (glyco)proteome of a pooled human blood plasma sample that was derived from 20 healthy donors. To assess the reproducibility of such a digest, five independent Proteinase K treated blood plasma samples (technical replicates) were measured with nanoRP-LC-ESI-IT-MS/MS in preliminary experiments. A comparison of the resulting base peak chromatograms revealed a high reproducibility of these digests, as shown in supplemental Fig. S1.

##### Glycopeptide Enrichment and Fractionation via HILIC-HPLC

**Fig. 2.**
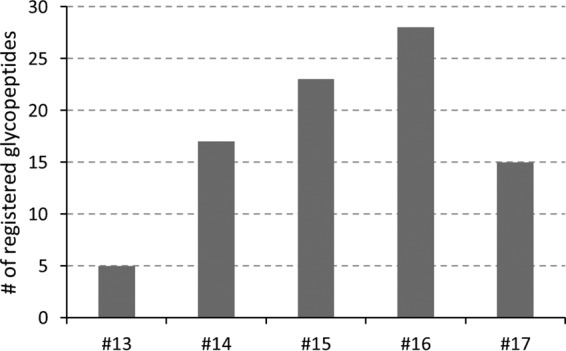
**Number of detected glycopeptides in HILIC fractions #13-#17.**

**Fig. 3.**
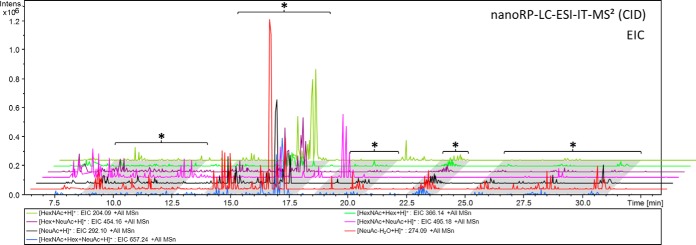
**Extracted ion chromatograms (EICs) of diagnostic glycan oxonium ions (*e. g*.** [HexNAc+Hex+NeuAc+H]^+^: EIC 657.24) reveal the clustered elution of *O*-glycopeptides (*) on a C18 reversed-phase column. EICs of HILIC fraction #15 are shown as an example.

The HILIC-HPLC fractionation carried out in the present study was optimized for the enrichment of *O*-glycosylated peptides (data not shown). In total 17 HILIC fractions were collected and were analyzed by nanoRP-LC-ESI-IT-MS^2^ (CID). The acquired fragment spectra were manually screened for the presence of *N*- and *O*-glycopeptides—relying on the detection of diagnostic oxonium ions (B-ions, *e.g.* HexNAc_1_Hex_1_NeuAc_1_; *m*/*z* 657.24) and characteristic mono(oligo)-saccharide neutral loss fragment ions (Y-ions). Glycopeptides were detected in five HILIC fractions (#13-#17) (Fig. 2). The glycopeptides eluted in the range of 9–32 min in RP-LC-MS and clusters of glycopeptides were registered between 12–18 min, 20–22 min, and 25–29 min (exemplarily shown for fraction #15, Fig. 3).

##### Determination of the Glycan Composition

**Fig. 4.**
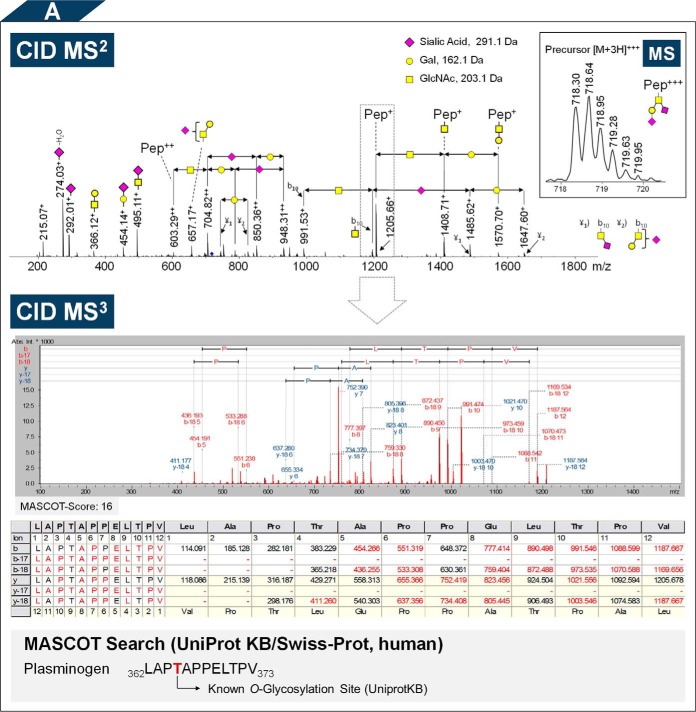

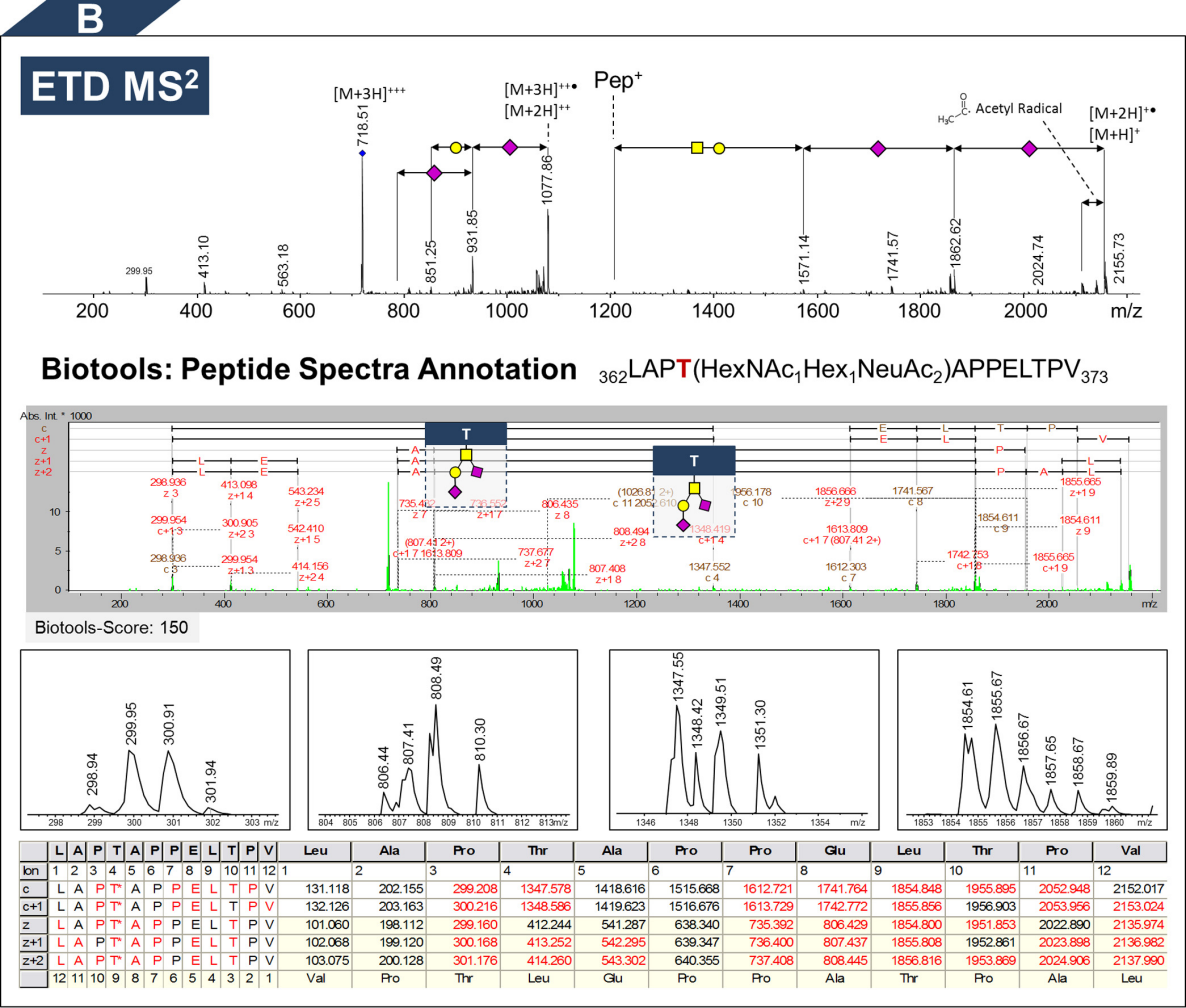
**Fragment ion spectra of the Proteinase K generated plasminogen *O*-glycopeptide _362_LAP*T***APPELTPV_373_ measured with nanoRP-LC-ESI MS^n^ (positive ion mode, CID and ETD). *A (top),* For the given *O*-glycopeptide the CID-MS^2^ spectrum is shown together with its corresponding precursor ion *m*/*z* 718.30 [M+3H]^3+^ (inset). The spectrum allows the elucidation of the *O*-glycan composition (here disialylated T-antigen). In addition, also some internal glycopeptide fragments have been detected (*e.g.* b_10_+HexNAc). *A (bottom):* The putative peptide mass (*m*/*z* 1205.66 [M+H]^+^) of the given *O*-glycopeptide was subjected to CID-MS^3^ fragmentation. The peptide was identified by MASCOT search (Score: 16, UniProt KB/Swiss-Prot, human). *B,* The *O*-glycosylation site (here Thr_365_) was pinpointed by means of ETD (Biotools-Score: 150). Magnified regions show the isotope pattern of selected peptide fragment ions, confirming the annotation. In addition to peptide fragment ions also fragment ions derived from the glycan moiety were detected, allowing a verification of the glycan composition. Furthermore, a neutral loss of an acetyl radical from the intact *O*-glycopeptide was observed, which is typically seen in ETD spectra of glycopeptides.

CID-MS^2^ spectra were carefully inspected and manually annotated with respect to the glycan composition. Major signals in these spectra resulted from consecutive neutral losses (singly and doubly charged species) of the monosaccharides hexose, *N*-acetylhexosamine and *N*-acetylneuraminic acid from the intact glycopeptide and most of the time the applied collision energy induced the complete fragmentation of the glycan moiety while leaving the de-glycosylated peptide intact. These fragment ions along with corresponding oxonium ions, allowed inferring the glycan composition and the putative peptide mass (Fig. 4*A*). Detailed analysis revealed, that exclusively mucin-type core 1 mono- and disialylated *O*-linked glycopeptides ((di)sialyl-T-antigen) were present. For the glycan annotation a mass error of ±0.3 Da was accepted. This parameter was justified as the observed mass errors were about 0.07 Da (median value). In total 88 *O*-glycopeptides were detected and characterized with respect to their glycan composition. The registered glycopeptides covered an *m*/*z* range of 507–945 (average *m*/*z* 728) and were either doubly (55 peptides) or triply charged (33 peptides).

##### Identification of the Peptide Moiety

To complement the deduced glycan composition with peptide sequence information, CID-MS^3^ experiments were conducted on putative peptide masses, which were derived from CID-MS^2^ spectra (Fig. 4*A*). In separate LC-MS runs the selected peptide precursor masses (predominantly singly charged) were used to trigger manual CID-MS^3^ fragmentation. In rare cases peptide+HexNAc was selected for fragmentation, because of low signal intensity of the peptide species in MS^2^. CID-MS^3^ spectra were searched against the human subset of the highly curated and nonredundant protein database UniProtKB/Swiss-Prot. Notably, also in some CID-MS^2^ spectra b- and y-ions derived from peptide backbone cleavages were detected, which enabled peptide identification (*e.g.* supplemental Fig. S5: α-2-HS-glycoprotein *m*/*z* 623.23^3+^).

For 88 detected glycopeptides, 60 corresponding peptides could be identified unambiguously (Table I, Table II). These 60 peptides could be linked to 22 different proteins, most of them being acute phase proteins. As the protein identification is based on a single peptide, validation of the potential peptide hits is of utmost importance. Here, in particular, the protein inference problem (53), which is intrinsic to bottom-up proteomic approaches, had to be considered. To cope with this, peptide spectra were manually revised and only peptide hits with a MASCOT ion score of greater than 20 were considered; only in rare cases, and supported by other evidences, also lower scored peptides were accepted. Furthermore, peptide hits needed to exhibit at least one potential *O*-glycosylation site (Ser/Thr). If available, knowledge derived from public databases (UniProtKB and UniCarbKB) on already described *O*-glycosylation sites within the putative peptides or within the entire protein was used to support a potential hit. The peptide identification was further corroborated by redundant identifications, that is the multiple occurrence of: (1) the same glycopeptide in different HILIC fractions, (2) or the same peptide but with a different glycan moiety, (3) or the identification of a peptide harboring the same glycosylation site, but differing in peptide length; the latter being attributed to the broad-specific proteolysis (*e.g.* alpha-2-HS-glycoprotein, _341_TVVQP***S***[HexNAc_1_Hex_1_NeuAc_1_]VG_348_ derived from HILIC fraction #13 and _342_VVQP***S***[HexNAc_1_Hex_1_NeuAc_1_]VG_348_ from fraction #14). In some cases, though, peptide identification was hampered or inconclusive. One of the main obstacles here was the frequent occurrence of prolines within the (glyco)peptide sequence, which was also described in literature. The cyclic structure of proline, gives rise to a high signal of the preceding y-ion but precludes in most cases the generation of a subsequent b-ion—thus introducing a sequence gap (54). This in turn leads to incomplete peptide fragment ion series and the occurrence of dipeptide fragment ions (*e.g.* PS and SP), which may result in ambiguity in peptide identification. This effect is particularly critical for short peptide sequences, as usually obtained by a broad- or nonspecific digest. The average peptide length of glycopeptides identified in this study is 10 amino acids (aa). This is significantly shorter than the average length of tryptic peptides (14 aa, based on an *in-silico* digestion of the human UniProtKB database (55), supplemental Fig. S2). All this—in conjunction with a nonspecific peptide search—makes a reliable peptide identification challenging.

**Table I:**
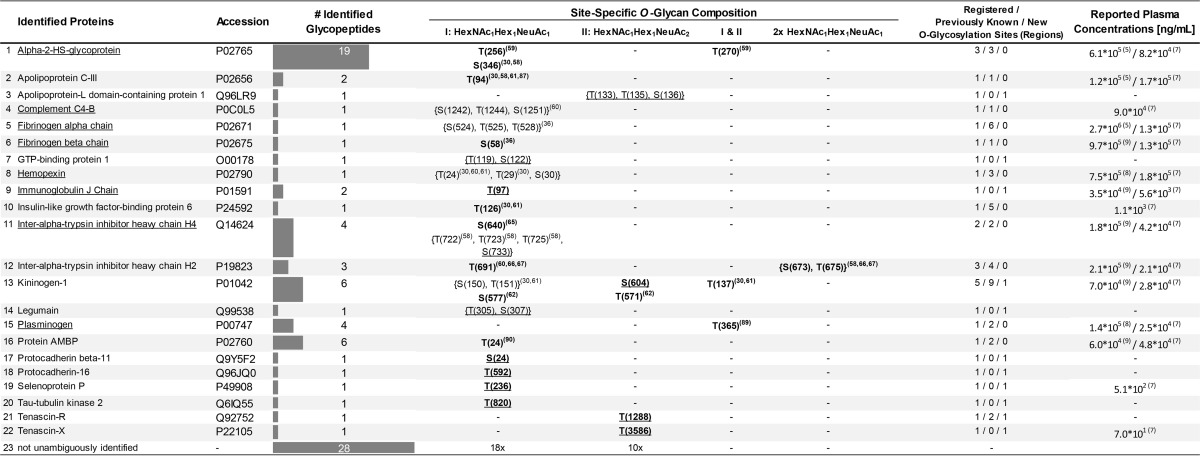
Site-specific O-glycan composition of identified human blood plasma glycoproteins. Glycoproteins are listed with their UniprotKB accession number as well as the number of identified glycopeptides. O-glycosylated sites or regions are indexed with respect to the attached O-glycans (mono- and/or disialylated mucin-type core 1 O-glycans). O-glycosylation sites in bold have been pinpointed within this study. Previously unknown sites und regions are indicated by underlining. Curled brackets mark regions with several possible O-glycosylation sites. Superscript numbers indicate literature references. For every protein the number of registered, previously known, as well as new O-glycosylation sites and regions are given. For underlined proteins, glycosylated as well as non-glycosylated peptides were identified (supplemental Table S1). In addition previously reported plasma concentrations are given. HexNAc (N-acetylhexosamine), Hex (hexose), NeuAc (N-acetylneuraminic acid, sialic acid)

**Table II:**
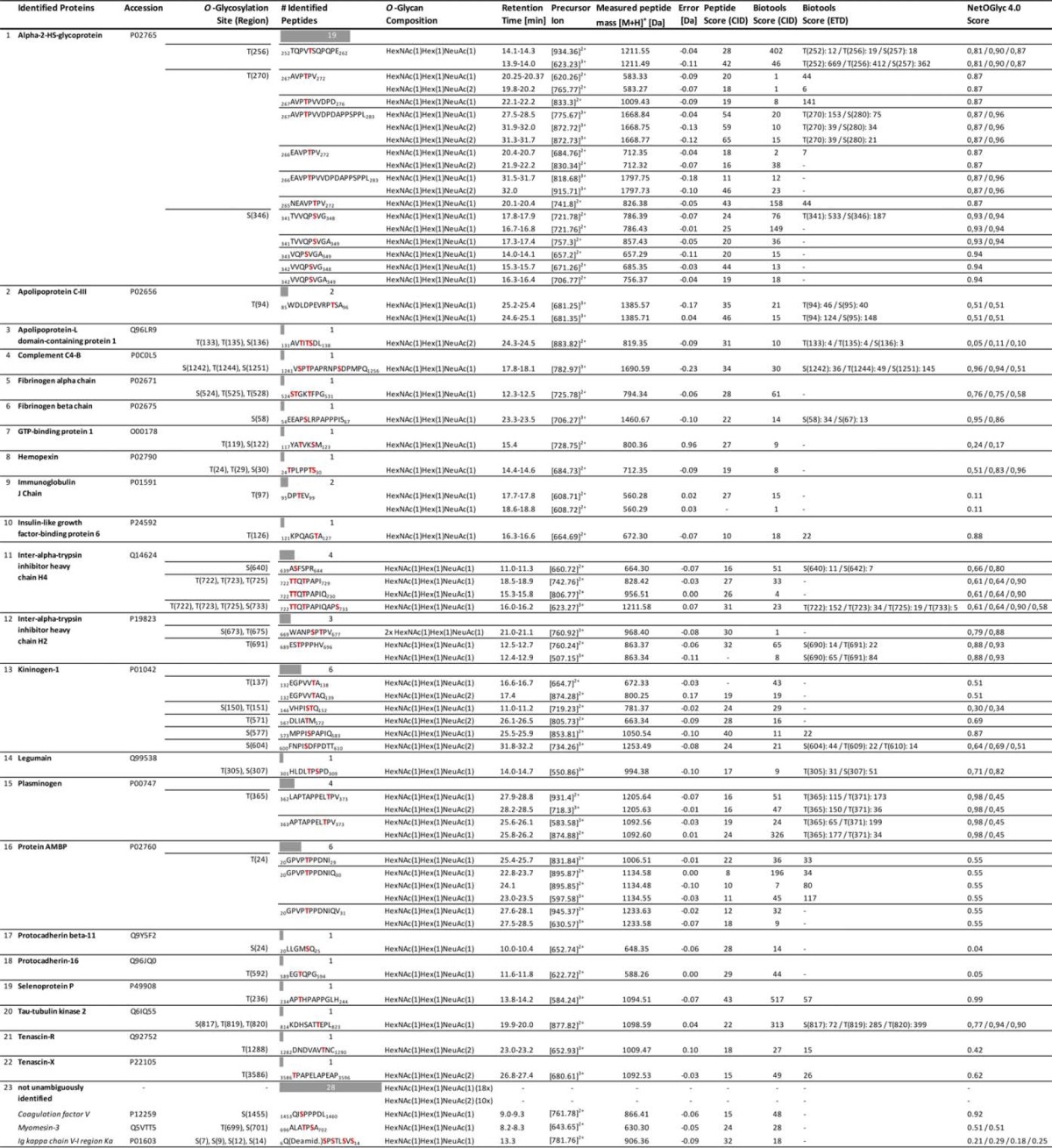
Detailed overview of all identified human blood plasma O-glycopeptides. For each O-glycopeptide, the corresponding glycoprotein including the UniProtKB accession number, the identified O-glycosylation site(s)/regions as well as the O-glycan composition are given, respectively. Likewise, the LC-MS retention time, the mass of the intact glycopeptide precursor, the measured peptide mass as well as the corresponding error is listed. The peptide identification using CID-MS^3^ was validated by the MASCOT peptide score and the Biotools score (CID). ETD based determination of the O-glycosylation site(s) was validated by the Biotools score (CID) as well as the NetOGlyc 4.0 score

To complement the identified *O*-glycopeptides with nonglycosylated peptides that are also present in blood plasma, CID und ETD fragment spectra of the corresponding HILIC fractions (#1–17) were searched against the human subset of the UniProtKB/Swiss-Prot protein database. In total 111 proteins were identified. CID and ETD spectra provided complementary results; 54 and 45 proteins were identified, respectively, and only 12 proteins were identified with both modes. Compared with ETD, significantly more peptides were identified with CID (321 *versus* 150), though. The majority of peptides were derived from immunoglobulins, serotransferrin, haptoglobin and serumalbumin (supplemental Table S1). Notably, also nonglycosylated peptides corresponding to previously identified *O*-glycopeptides, *e.g.* of plasminogen and hemopexin, were identified (Table I).

##### Localization of the O-Glycosylation Sites

To further characterize the identified *O*-glycopeptides, the corresponding *O*-glycosylation sites needed to be localized. In a few cases the use of Proteinase K, already generated glycopeptides that exhibit only one possible *O*-glycosylation site, *e.g.*_132_EGPVV***T***[HexNAc_1_Hex_1_NeuAc_1_]A_138_ and _567_DLIA***T***[HexNAc_1_Hex_1_NeuAc_2_]M_572_ from kininogen-1 or _234_AP***T***[HexNAc_1_Hex_1_NeuAc_1_]HPAPPGLH_244_ from selenoprotein P. Noteworthy, in the first example a tryptic digest would have generated a peptide with a length of 43 aa (_119_F***S***VA***T***Q***T***CQI***T***PA*EGPVV**T**A*QYDCLGCVHPI***ST***Q***S***PDLEPIL**R**_161_), harboring 8 potential *O*-glycosylation sites. This clearly illustrates a benefit of the Proteinase K digest for the *O*-glycan site identification.

When the *O*-glycosylation sites could not be inferred directly, glycopeptides were subjected to ETD fragmentation in a separate LC-MS run (Fig. 4*B*). The most prominent peaks in the acquired ETD glycopeptide spectra were the unfragmented precursor ion along with charge-reduced species; minor peaks were derived from c- and z-type peptide backbone cleavages. Furthermore, fragment ions indicating either the loss of 43.018 Da (C_2_H_3_O·) from the radical cationic species or 42.016 Da (C_2_H_2_O) from the even electron species [M+H]^+^ were consistently detected. In the literature this spectral feature was attributed to the loss of an acetyl-radical from the *N*-acetyl group of a HexNAc (56, 57). This in turn can support the discrimination of ETD spectra derived from glycosylated and nonglycosylated species. Strikingly, and in contrast to the general mode of action of ETD, also fragmentations of the glycan moiety along the intact peptide backbone were observed, leading to a complete loss of the *O*-glycosylated Ser/Thr side-chain. Nevertheless, the resulting fragment ions enabled a verification of the glycan composition as well as the peptide mass.

At first, ETD generated glycopeptide spectra were searched against the human subset of the UniProtKB/Swiss-Prot database using MASCOT, under consideration of the *O*-glycan modification (theoretical glycan mass used as variable modification of Ser/Thr). However, this strategy failed because of the presence of intense signals in the ETD spectrum, which correspond to: (I) the precursor ion, (II) the charge reduced precursor ion, (III) acetyl radicals ions, (IV) or glycan fragment ions. These ions might be erroneously interpreted as peptide derived fragment ions by the search engine, because ETD is supposed to solely produce peptide fragment ions while keeping fragile side-chain modifications, like the glycosylation, intact. To overcome this, glycopeptide spectra were exported to Bruker BioTools for manual spectra annotation. Here, the identified glycopeptides were built *in silico*, taking into account the corresponding *O*-glycan moieties as well as all possible *O*-glycosylation sites. Subsequently, the resulting *in silico* fragment ions (c- and z-type ions) were matched to their counterparts in the measured ETD-MS^2^ spectra. To evaluate the spectra annotation and to discern the correct *O*-glycosylation site, the BioTools spectra matching score along with manual inspection of the respective spectra were considered. Furthermore, public repositories, namely UniProtKB and UniCarbKB, were queried with respect to known *O*-glycosylation sites within the peptide in question. To further asses the validity of the *O*-glycosylation site annotation, the site occupancy was predicted using NetOGlyc—an online tool, based on machine-learning algorithms, which allows the prediction of mucin-type *O*-glycosylation sites (27). For 36 of 60 identified glycopeptides the quality of the corresponding ETD spectra was acceptable - in terms of signal intensity and the number of fragment ions. Overall, 31 *O*-glycosylation sites and regions were detected, of which 23 sites could be pinpointed (Tables I and II). Strikingly, 11 previously unknown *O*-glycosylation sites and regions were registered, of which 8 sites could be pinpointed. Generally, *O*-glycosylation on threonine residues was observed more frequently than on serine (16× Thr, 7× Ser). In accordance with literature, prolines were frequently found in close vicinity to the *O*-glycosylation site (positions *n* - 1, *n* + 1, *n* + 3), *e.g.*_267_AVP***T***[HexNAc_1_Hex_1_NeuAc_1_]PV_272_, _343_VQP***S***[HexNAc_1_Hex_1_NeuAc_1_]VGA_349_ from alpha-2-HS-glycoprotein (30). In addition also prolines in position *n* + 2 were found occasionally, *e.g.*_20_GPVP***T***[HexNAc_1_Hex_1_NeuAc_1_]PPDNI_29_ from alpha-1 microglycoprotein (protein AMBP).

##### Identified Glycoproteins: Selected Examples

In the following selected examples of identified *O*-glycopeptides are detailed, that feature novel *O*-glycosylation sites or exhibit remarkable fragmentation characteristics.

##### α-2-HS-glycoprotein

In this study, the majority of identified *O*-glycopeptides were derived from α-2-HS-glycoprotein, also known as fetuin-A. Fetuin-A is a negative acute phase glycoprotein that is highly abundant in fetal blood plasma. It is involved in transport and storage of substances and features three *O*-glycosylation sites (Thr_256_, Thr_270_, Ser_346_), which are decorated with sialylated mucin-type core 1 *O*-glycan structures (30, 58, 59). In contrast to previous reports (30, 58), intact *O*-glycopeptides identified and characterized in the present study describe all three known fetuin-A *O*-glycosylation sites including the attached *O*-glycans (mono- and disialylated mucin-type core 1 *O*-glycans), respectively. By pinpointing *O*-glycosylation sites using ETD, the reported ETD Biotools scores can be misleading. This for instance holds true for the fetuin-A *O*-glycopeptide _252_***T***QPV**TS**QPQPE_262_ (*m*/*z* 623.23^3+^) and its three potential *O*-glycosylation sites: Thr_252_ (669), Thr_256_ (412), Thr_257_ (362) (supplemental Fig. S7). According to the score values T_252_ would be the occupied site; the presence of characteristic ETD fragment ions at *m*/*z* 344.01^1+^ (c_3_), 1200.45^1+^ (c_5_), 1287.47^1+^ (c_6_), 1525.57^1+^ (z+1_8_), 1751.51^1+^ (z+2_10_), though, clearly indicates the occupancy of Thr_256_, which is in agreement with literature findings. For the two other described fetuin-A *O*-glycosylation sites Thr_270_ and Ser_346_ ETD fragmentation was actually not mandatory, because corresponding *O*-glycopeptides were identified that solely harbor one *O*-glycosylation site (*e.g.* Thr_270_: _267_AVP***T***PV_272_, Ser_346_: _342_VVQP***S***VG_348)_, respectively. Also of note, with respect to the peptide identification, b- and y-ions were detected in the CID-MS^2^ fragment spectra of the fetuin-A *O*-glycopeptides _252_TQPV***T***SQPQPE_262_ (*m/z* 623.23^3+^), _267_AVP***T***PVVDPDAPPSPPL_283_ (*m*/*z* 872.72^3+^) and _266_EAVP***T***PVVDPDAPPSPPL_283_ (*m/z* 915.71^3+^), which already permit the unambiguous peptide identification without consideration of CID-MS^3^ spectra (supplemental Figs. S5–S7). Furthermore, internal glycopeptide fragment ions resulting from concerted fragmentations along the peptide backbone and along the glycan moiety were detected in the same CID-MS^2^ spectra - a low-energy CID glycopeptide fragmentation event that is rarely described in literature (*e.g.*_252_TQPV***T***(HexNAc_1_Hex_1_NeuAc_1_)SQPQPE_262_ → _252_TQPV***T***(HexNAc)SQ_258_ *m/z* 945.35^1+^) (supplemental Fig. S7).

##### Kininogen-1

The human KNG1 gene codes for two splicing variants of kininogen, namely low-molecular and high-molecular weight kininogen. The latter is involved in blood coagulation and the assembly of the kallikrein-kinin system and was identified in the present study by six *O*-glycopeptides. Currently nine *O*-glycosylation sites/regions are described in literature for kininiogen-1—presumably all being decorated with mucin-type core 1 or possibly core 8 *O*-glycans (30, 60–62). Experimental glycoproteomic evidence on the macro and microheterogeneity of kininogen-1 is still missing, though. In the present study, four kininogen-1 *O*-glycosylation sites, including one novel site (Ser_604_), could be pinpointed and described with respected to the composition of the attached *O*-glycans (Table I). The identified *O*-glycopeptide _600_FNPI***S***DFPD***TT***_610_ (*m*/*z* 734.26^3+^) carries a disialylated T-antigen and harbors three potential *O*-glycosylation sites. ETD analysis implies the occupancy of Ser_604_, because of the presence of a signal at *m*/*z* 1638.30^1+^, corresponding to a c_6_ ion (supplemental Fig. S6). Also of note, in previous studies the use of trypsin did not allow to pinpoint occupied *O*-glycosylation sites in the region aa119–161 (30, 61). Proteinase K, however, generated two distinct *O*-glycopeptides (_132_EGPVV***T***A_138_ *m*/*z* 664.70^2+^, _146_VHPI***ST***Q_152_ *m*/*z* 719.23^2+^) that allowed pinpointing the site Thr_137_ and the region Ser_150_/Thr_151_. For the latter, unfortunately, the ETD spectrum quality did not allow localizing the exact site. The peptide _132_EGPVV***T***A_138_ (*m*/*z* 664.70^2+^) could not be identified correctly by MASCOT database search, because of missing fragment ions. However, the peptide could be identified via manual *de novo* annotation supported by mass tag ([283.0 Da]VVTA) assisted *de novo* sequencing using the tool MS-Homology (http://prospector.ucsf.edu/prospector) (supplemental Fig. S5). The peptide identity was further verified by the identification of the glycosylated peptide _132_EGPVV***T***AQ_138_ (*m*/*z* 874.28^2+^) in a subsequent HILIC fraction (supplemental Fig. S7).

##### Immunoglobulin J Chain

The immunoglobulin J chain (joining chain) participates in the effective di-/polymerization of either IgA or IgM and is essential for the secretion of these immunoglobulins into the mucosa. In literature the J chain was reported to be *N*-glycosylated at Asn_49_ (60, 63, 64); however, *O*-glycosylation has hitherto not been described for the molecule. Interestingly, two *O*-glycopeptides detected in HILIC fractions #13 and #14 might correspond to the J chain and suggest *O*-glycosylation at Thr_97_ (_95_DP***T***EV_99_ *m/z* 608.72^2+^, *m*/*z* 608.71^2+^) (supplemental Figs. S3 and S5). This potentially new *O*-glycosylation site is in close vicinity to a cysteine (Cys_91_) that can form a disulfide-bridge to IgM molecules. Hence, one might speculate that an occupied *O*-glycosylation site in this region might function in the establishment/preservation of this inter-molecular bond. However, the number of present fragment ions in the corresponding CID-MS^3^ spectra did not allow an unambiguous identification of the peptide, as evidenced by several potential peptide hits being equally scored by the search engine. Manual fragment spectra annotation, though, suggest the identification of immunoglobulin J chain—nevertheless, this identification deserves further validation. Both identified *O*-glycopeptides were found to be decorated with monosialylated T-antigens.

##### Inter-α-trypsin Inhibitor Heavy Chain H4

For the protease inhibitor inter-alpha-trypsin inhibitor heavy chain H4 two *O*-glycosylation sites/regions, Ser_640_ and Thr_722/723_ have been described in literature (58, 65). In agreement with recent findings by Chandler *et al.*, Ser_640_ was found to be *O*-glycosylated. The *O*-glycopeptide _639_A***S***F***S***PR_644_ (*m*/*z* 660.72^2+^) harbors two potential *O*-glycosylation sites and the occupied site could be clearly inferred from the ETD spectra by the presence of a signal at *m*/*z* 490.22^1+^, corresponding to a z+1_4_ ion (supplemental Fig. S5). In contrast to Chandler *et al.*, but in agreement with Halim *et al.*, ETD data of the *O*-glycopeptide _722_***TT***QTPAPIQAPS_733_ (*m/z* 623.27^3+^) suggested the occupancy of the sites Thr_722/723_ (58, 65) (supplemental Fig. S5). Unfortunately, none of the two potential *O*-glycosylation sites could be clearly ruled out by the detected fragment ions. Both sites/regions Ser_640_ and Thr_722/723_ were decorated with a monosialylated T-antigen. This contrasts previous findings by Chandler *et al.* who also observed a disialylated T-antigen on S_640_.

##### Inter-α-trypsin Inhibitor Heavy Chain H2

For the H2 heavy chain of the Inter-alpha-trypsin inhibitor a c-terminal cluster of mono- and disialylated mucin-type core 1 *O*-glycans (Thr_666_, Ser_673_, Thr_675_ and Thr_691_) has been described in literature (58, 60, 66, 67). These previously reported *O*-glycosylated sites, except for the site T_666_, could be confirmed by the present study, albeit solely with monosialylated T-antigens. ETD spectra of the *O*-glycopeptide _689_ES***T***PPPHV_696_ (*m*/*z* 507.15^3+^/760.24^2+^) enabled a clear identification of the occupied *O*-glycosylation site Thr_666_. This finding is supported, in particular, by a signal detected in the doubly charged species at *m*/*z* 1287.41^1+^ which corresponds to a z+1_6_ ion (supplemental Fig. S6). Remarkably, the CID-MS^2^ spectrum of the *O*-glycopeptide _669_WANP***S***P***T***PV_677_ (*m*/*z* 760.92^3+^) revealed that both *O*-glycosylation sites, Ser_673_ and Thr_675,_ are occupied by a monosialylated T-antigen (supplemental Fig. S7). Moreover, the spectrum features signals indicating the presence of hexose rearrangement products, that is the transfer of an additional hexose either to the glycan or the peptide moiety, as described earlier (68, 69). The occurrence of these artifacts necessitates the careful interpretation of CID glycopeptide fragment spectra.

##### τ-Tubulin Kinase 2

The τ-tubulin kinase 2 (TTBK2) phosphorylates τ and tubulin, preferably in the nervous system. Aberrant TTBK2 activity was linked to the progression of the Alzheimer's disease (70, 71). The protein resides primarily in the cytosol; however, Böhm *et al.* could also detect TTBK2 in a secreted form in human tears (72, 73). Hitherto, no glycosylation of this protein has been described. CID-MS^3^ as well as ETD spectra of the *O*-glycopeptide _814_KDHSAT***T***EPL_823_ +HexNAc_1_Hex_1_NeuAc_1_ (*m*/*z* 877.82^2+^), though, suggest the *O*-glycosylation of Thr_820_. ETD fragment ions at *m*/*z* 485.22^1+^, 557.43^1+^, and 1098.63^1+^, corresponding to c_4_, c+1_5_, and z_4_ ions, allowed discerning the exact glycosylation site. As TTBK2 is involved in ciliogenesis (74, 75), a process which requires the vesicle transport from the Golgi to the basal bodies and cilia, we speculate that TTBK2 might become *O*-glycosylated during this process.

##### Fibrinogen α and β Chain

The blood clotting protein fibrinogen is known to be *N*-glycosylated at the β- and γ-chain. Interestingly, a recent study by Zauner *et al.* could also show *O*-glycosylated sites and regions, seven in total, within the molecule (51). In the present study *O*-glycosylation of the fibrinogen alpha region aa524–528 could be confirmed; pinpointing the exact *O*-glycosylation site was not possible, though (supplemental Fig. S5, _524_***ST***GK***T***FPG_531_, *m*/*z* 725.78^2+^). Nevertheless, *O*-glycosylation within the fibrinogen beta region aa58–67 could be confirmed and pinpointed. Here, the presence of the ETD fragment ions *m*/*z* 931.54^1+^, 1300.54^1+^, and 1915.50^1+^, corresponding to z+1_9_, c_6_, and c_12_ ions (supplemental Fig. S6), _54_EEAP***S***LRPAPPPIS_67_, *m*/*z* 706.27^3+^) indicates *O*-glycosylation at the site Ser_58_. This contrasts recent findings by Bai *et al.* who reported the site Ser_67_ to be *O*-glycosylated, but not the site Ser_58_ (76). In agreement with previous findings, both fibrinogen *O*-glycopeptides (_524_***ST***GK***T***FPG_531_, *m*/*z* 725.78^2+^, _54_EEAP***S***LRPAPPPIS_67_, *m*/*z* 706.27^3+^), detected in the present study, were found to be decorated with monosialylated T-antigens. Interestingly, the peptide _54_EEAP***S***LRPAPPPIS_67_ was also found in its nonglycosylated form (HILIC fractions #12-#15, CID, see supplemental Table S2), which suggests only a partial occupation of the *O*-glycosylation site Ser_58_.

## DISCUSSION

Over the last few years mass spectrometry based glycoproteomics has experienced significant advances in terms of instrumentation, methodology and bioinformatics; resulting in a variety of excellent glycoproteomic publications that highlight the merits of high resolution mass spectra, complementary fragmentation techniques, improved multidimensional glycopeptide enrichment and separation techniques as well as sophisticated software tools (41). However, despite these advances—and despite its enormous clinical and pharmaceutical relevance as well as diagnostic potential—our knowledge about the human blood plasma glycoproteome is still very limited. This holds particularly true for the human blood plasma *O*-glycoproteome. Here several important questions can be raised: Which proteins are *O*-glycosylated?, Which *O*-glycans are attached to which sites?, Which dynamics in terms of the *O*-glycan micro- and macroheterogeneity can be observed in a certain biological context?, What are the biological and biotechnological implications of *O*-glycosylation?

In the present study we have developed and employed an analytical workflow that allows the explorative, nontargeted analysis of the human blood plasma *O*-glycoproteome in a site-specific manner. To this end intact human blood plasma *O*-glycopeptides, generated by a broad-specific proteolytic digest via Proteinase K, were selectively enriched using HILIC fractionation in order to be analyzed by multistage nanoRP-LC-ESI-IT-MS using low-energy CID as well as ETD (CID-MS^2^/MS^3^, ETD-MS^2^). This combined workflow was applied on a pooled blood plasma sample derived from 20 healthy donors and allowed for the identification of 31 *O*-glycosylation sites in 22 proteins, including the detection of 11 previously unknown *O*-glycosylation sites. We were able to pinpoint 23 *O*-glycosylation sites, of which eight sites have been described for the first time. The identified *O*-glycan compositions most probably correspond to mono- and disialylated core-1 mucin-type *O*-glycans (T-antigen).

### 

#### 

##### Other O-glycoprotomic Studies on Complex Biofluids

In the recent past efforts have been made to investigate the *O*-glycoproteome of different complex biological samples. Halim *et al.*, for instance, analyzed the *O*-glycoproteome of cerebrospinal fluid (CSF) using a sialic-acid capture-and-release protocol (30). This protocol is based on the sialic acid specific hydrazide capturing of periodate oxidized glycoproteins. Upon tryptic digestion the protocol allows the acid hydrolysis of sialic acid glycosidic bonds in order to release and analyze (formerly) sialylated glycopeptides. To focus on *O*-glycosylations the authors included a peptide *N*-glycosidase F (PNGase F) sample pretreatment step to remove *N*-glycans. The authors have used an automated CID-MS^2^/-MS^3^ spectra search protocol for glycopeptide identification (Peptide-GalNAc-Gal) and have employed ECD and ETD to pinpoint the glycosylation sites. In total they have identified 106 *O*-glycosylation sites and could pinpoint 67 of these. The identified CSF *O*-glycopeptides belong to 49 different proteins and were predominately decorated with structures corresponding to core-1 mucin-type *O*-glycans. In a previous study the same group has also investigated the human urinary *N*-and *O*-glycoproteome using the sialic-acid capture-and-release protocol (58). Unfortunately, the applied protocol does not allow the enrichment of nonsialylated glycoproteins nor does it give any information on the degree of sialylation of the attached *O*-glycan moieties. This limits the applicability of this procedure, as the degree of *O*-glycan sialylation is a crucial determinant in the pathogenesis of a number of diseases (22).

In another large-scale glycoproteomics study conducted by Hägglund *et al.* in 2007 human plasma proteins, derived from Cohn fraction IV of a plasma fractionation, were analyzed with respect to occupied *N*- and *O*-glycosylation sites (60). The analyzed Cohn fraction is supposed to contain mainly α-globulins, like plasminogen and haptoglobin, and is depleted from γ-globulins and serum albumin. The authors have employed two different enzymatic deglycosylation strategies to pinpoint occupied *N*-glycosylation sites: (1) PNGase F + H_2_^18^O; (2) endo-β-*N*-acetylglucosaminidases (Endo D and Endo H) + different exoglycosidases. These two strategies were applied on HILIC enriched tryptic (glyco)peptides, that were fractionated by strong cation exchange chromatography and eventually measured by LC-ESI-MS/MS using high-energy CID. The authors were able to identify 103 *N*-glycosylation sites as well as 23 *O*-glycosylation sites/regions derived from 61 and 11 human blood plasma proteins, respectively. Unfortunately, the occupied *O*-glycosylation sites could not be pinpointed and no information on the glycan moiety could be deduced.

In 2012 Darula *et al.* reported on the *O*-glycoproteomic analysis of bovine serum (77). In this study the authors have combined different protein- and peptide-level prefractionation and enrichment strategies, including jacalin lectin affinity chromatography, mixed-mode chromatography, and electrostatic repulsion hydrophilic interaction chromatography (ERLIC) to enrich tryptic mucin-type *O*-glycopeptides. After additional use of exoglycosidases to improve glycopeptide characterization, truncated glycopeptides were subjected to LC-ESI-MS/MS with HCD and ETD for automated peptide identification and glycosylation site determination. Overall, the authors could identify and pinpoint 124 glycosylation sites in 51 proteins, including many *O*-glycosylation sites that have not been described before—unfortunately, though, at the expense of the intact glycan structure.

In a recent publication from Bai *et al.* an analytical workflow is presented, which allows the mapping of mucin-type *O*-glycosylation sites on glycoproteins present in human blood plasma (76). The authors have used jacalin lectin affinity chromatography to enriched tryptic *O*-glycopeptides (peptide+GalNAc), which were treated with PNGase F and different exoglycosidases. In this study 49 *O*-glycopeptides, belonging to 36 human blood plasma glycoproteins, were identified by LC-ESI-MS/MS (CID). Overall, the authors could assign 13 *O*-glycosylation sites unambiguously, of which nine sites have not been described before.

##### Proteinase K Digest

The majority of large-scale glycoproteomic studies features trypsin for the generation of (glyco)peptides. Trypsin is the proteolytic gold standard in LC-MS/MS based peptide identification and quantification, as it reproducibly generates predictable peptides that can be readily retained on reversed-phase column and that give enough fragment ions for an unambiguous peptide identification, in most cases. In terms of glycoproteomics, though, the cleavage specificity of trypsin can be a limiting factor for the identification and the localization of certain glycosylation sites, in particular for densely clustered *O*-glycosylation sites. Hence, the use of broad- and nonspecific proteases, like Pronase E or Proteinase K was proposed, to reduce the number of nonglycosylated peptides and to make certain glycosylation sites analytically amenable (34). Proteinase K, for instance, has been successfully used in a number of publications that are centered on the *O*-glycoproteomic analysis of single proteins; though, the use of Proteinase K in large-scale glycoproteomic studies on complex samples has not been described so far. In the present study we could show that Proteinase K generates (glyco)peptides from a complex sample, like human blood plasma, in a reproducible and nonrandom manner, which is in agreement with a report from Hua *et al.* (34). We could show that, most of the time, Proteinase K generates shorter peptides compared with trypsin (supplemental Fig. S7), and that Proteinase K cleaves effectively in-between densely *O*-glycosylated regions—thus, rendering the determination of the occupied *O*-glycosylation site(s) less difficult. In fact we could show that Proteinase K can generate *O*-glycopeptides that exhibit only one potential *O*-glycosylation site, thus allowing for an unambiguous localization of the occupied site. We could clearly show that some *O*-glycosylation sites could only be identified and pinpointed by the use of Proteinase K, because tryptic peptides would have been too long and would have harbored too many potential *O*-glycosylation sites.

##### Glycopeptide Enrichment Via HILIC

Glycopeptides are usually under-represented in a peptide mixture, because of the glycan microheterogeneity. In a tryptic digest of a typical glycoprotein only about 2% to 5% of the peptides are glycopeptides (78). In addition, the ionization efficiency of glycopeptides is significantly lower compared with their nonglycosylated counterparts, thus making the efficient and selective enrichment of glycopeptides key to most glycoproteomics workflows. The use of HILIC based glycopeptide enrichment methods has proven to be a vital tool in glycoproteomics because of their broad glycan specificity, reproducibility and compatibility with mass spectrometry. In a previous report by Zauner *et al.* it could be shown, that Proteinase K-generated glycopeptides can be separated into earlier eluting *O*-glycopeptides and later eluting *N*-glycopeptides using HILIC (32). Based on this publication we have employed HILIC for the selective enrichment and fractionation of human blood plasma *O*-glycopeptides. Here of particular importance is the removal of highly abundant nonglycosylated peptides derived from albumin and other major (glyco-)proteins. Careful manual inspection of CID-MS^2^ fragment spectra of the acquired HILIC fractions revealed the efficient enrichment of glycopeptides - and indeed the presence of solely mucin-type core-1 *O*-glycosylated glycopeptides. *N*-glycopeptides were not detected, as they were expected to be present in the late eluting HILIC wash fractions because of their generally higher hydrophilicity compared with the most commonly found forms of mucin-type *O*-glycopeptides (non-, mono- and disialylated core-1 and -2 *O*-glycopeptides).

##### Identification of the O-glycan Composition

For an automated glycopeptide spectra filtering and glycan fragment annotation the use of commercial software tools was considered, but turned out to be too error-prone in our case (data not shown). Hence, in the present work we relied on manual annotation and interpretation of low-energy CID-MS^2^ fragment spectra in order to elucidate the *O*-glycan composition-however, at the expense of throughput and the possibility to report false discovery rates. In total we were able to characterize 88 *O*-glycopeptides with respect to their *O*-glycan composition. The detected *O*-glycan compositions most likely correspond to mucin-type core-1 mono- and disialylated *O*-glycans ((di)sialyl-T-antigen). In agreement with literature, glycopeptides carrying disialylated *O*-glycans, were found in later eluting HILIC fractions (#15–#17), as the additional sialic acid renders the molecule more hydrophilic. Mono- and disialylated glycoforms could be usually discriminated by the presence of distinct oxonium ions: whereas fragmentation of monosialylated *O*-glycans generated a characteristic oxonium ion at *m*/*z* 454.16 (Hex_1_NeuAc_1_), disialylated *O*-glycans gave rise to an additional intense peak at *m*/*z* 495.18 (HexNAc_1_NeuAc_1_) (supplemental Fig. S6, _266_EAVP***T***PVVDPDAPPSPPL_283_, *m*/*z* 818.68^3+^, _267_AVP***T***PVVDPDAPPSPPL_283_, *m*/*z* 872.73^3+^). Furthermore, in disialylated species characteristic fragment ions of the peptide+HexNAc+NeuAc were observed. In a few cases the glycan annotation was compromised by the presence of fragment ions corresponding to hexose rearrangement products (68, 69). Generally, it is important to note, that low-energy CID-MS^2^ fragmentation of glycopeptides does usually not produce fragment ions that relate to the linkage of the attached monosaccharides. Therefore, validation of the inferred *O*-glycan structures using dedicated *O*-glycomics approaches, including for instance (reductive) beta-elimination or hydrazinolysis, is recommended. However, our findings are in good agreement with literature, as mono- and disialylated mucin-type core-1 *O*-glycans are known to be present on the majority of secreted blood plasma glycoproteins, produced by hepatic cells of healthy individuals (79). Notably, a study on plasma-derived von Willebrand factor could show, that apart from mucin-type core 1 *O*-glycans (T-antigen), more complex *O*-glycan structures including ABH blood group antigen containing mucin-type core-2 ([GalNAcβ1–6-(Galβ1–3)-GalNAcα-*O*-Ser/Thr]), can be present on human blood plasma glycoproteins, too (80). In the present work, analyzing the total human blood plasma *O*-glycoproteome, we could not detect any (glyco)peptide derived from von Willebrand factor, nor could we find any indication for the presence of fucosylated (ABH blood group antigens) and/or LacNAc extended mucin-type core-2 *O*-glycans.

##### Glycopeptide Identification

Low-energy CID-MS^2^ fragmentation of glycopeptides, as employed in the present work, almost exclusively generates fragment ions corresponding to the fragmentation of the glycan moiety, while leaving the peptide backbone mainly intact. Thus, this type of fragmentation does usually not provide any information on the sequence of the peptide backbone nor on the occupied glycosylation site. To identify the peptide we have employed manual CID-MS^3^ fragmentation on the putative peptide mass, which has been inferred from the annotation of the corresponding CID-MS^2^ spectra before. In a few of cases the signal of the putative peptide mass was too low to yield sufficient fragment ions. Consequently, the putative peptide+HexNAc ion was subjected to CID-MS^3^ fragmentation instead. We did not employ an automated CID-MS^3^ fragmentation procedure, *e.g.* fragmentation of the three most intense precursor ions in the CID-MS^2^ spectrum, because we wanted to generate and sum up as many fragment spectra as possible from the selected putative peptide mass, in order to increase spectra quality and therefore the chance of successful peptide identification. By searching the acquired CID-MS^3^ fragment spectra against the human subset of the UniProtKB/Swiss-Prot protein database, a total of 60 peptides (of 88 detected *O*-glycopeptides) could be identified unambiguously. Notably, in a few cases also peptide fragment ions present in CID-MS^2^ spectra allowed for an unambiguous peptide identification (supplemental Fig. S4, _267_AVP***T***PVVDPDAPPSPPL_283_, *m*/*z* 872.73^3+^). Overall, the identified peptides belong to 22 different proteins—primarily acute phase proteins. This constantly growing group of blood plasma proteins fulfills essential functions during inflammation (*e.g.* coagulation, anti-inflammatory and anti-pathogenic activity), and, accordingly, their expression is known to be either significantly up- or downregulated (positive and negative acute phase proteins) in this context. As a result, this group of proteins attracted a lot of attention as potential cancer biomarkers in recent years (5). Noteworthy, the identified proteins span a concentration range of 5 orders of magnitude. Therefore, the applied approach seems to be suitable to also detect lower abundant proteins or peptides.

A group of *O*-glycosylated proteins that have frequently been identified in other large-scale glycoproteomic studies are Coagulation factors (30, 58, 60, 77). In our study there is an indication for the presence of an *O*-glycosylated peptide derived from Coagulation factor V (HILIC fraction #15, *m*/*z* 761.78^2+^, _1453_QI***S***PPPDL_1460_+HexNAc_1_Hex_1_NeuAc_1_, Table II, supplemental Fig. S5). Interestingly, the detected Coagulation factor V *O*-glycosylation site (Ser_1455_) has not been described so far. Unfortunately, our data do not allow an unambiguous identification of this protein.

##### General Remarks on Immunoglobulin O-glycoproteomics

Another *O*-glycosylated protein that could not be identified in our study is Ig α-1 (IgA1). IgA1 is a high abundant human blood plasma glycoprotein that features a cluster of three to five mucin-type *O*-glycans in the hinge region of the heavy chain (81). This cluster harbors many prolines, hence corresponding Proteinase K generated peptides might have been not unambiguously identified (the tryptic IgA1 hinge region *O*-glycopeptide looks as follows: (K)_89_HYTNPSQDVTVPCPVP***S***TPPTP***S***P***S***TPPTP***S***P***S***CCHP**R**_126)_. Furthermore, because of the densely clustered *O*-glycans a potential IgA1 *O*-glycopeptide carrying mucin-type *O*-glycans at each potential site, such as P***S***TPPTP***S***P***S***TPPTP***S***P***S***CC, might be too hydrophilic and consequently might have been among the (glyco)peptides present in the late eluting HILIC wash fraction. Worth mentioning, in our study we could detect the IgA1 peptide _95_QDVTVPCPVP***S***_105_ in its nonglycosylated form (HILIC Fraction #11, CID, supplemental Table S2). Therefore, the *O*-glycosylation site S_105_ seems to be only partially occupied. Surprisingly, human IgA1 *O*-glycopeptides have not been identified in any other large-scale glycoproteomic studies (30, 58, 60, 76, 77, 82). However, there is a targeted glycoproteomic study from Takahashi *et al.* focusing on IgA1 *O*-glycosylation (81). In this study the authors analyzed human plasma derived IgA1 *O*-glycopeptides (tryptic and nontryptic) with ESI-FT-ICR-MS/MS as well as ESI-LTQ-FT-MS/MS, both in online- and offline-Mode. To pinpoint the *O*-glycosylation sites the authors have employed activated ion-electron capture dissociation (AI-ECD) and ETD. Another immunoglobulin that is reported to carry mucin-type *O*-glycans in the hinge region is Ig delta (IgD) (83). The plasma concentration of IgD is much lower than the concentration of IgA, IgG, and IgM but higher than that of IgE (IgD represents 0.25% of total plasma immunoglobulins). Apart from the study conducted by Takayasu *et al.* from 1982 (83) on truncated *O*-glycopeptides (peptide+GalNAc), at present no *O*-glycoproteomic data do exist for intact human IgD *O*-glycopeptides. Also of particular interest is a recent finding by Plomp *et al.*: using a targeted glycoproteomics approach these authors could demonstrate, for the first time, that IgG3 is partially *O*-glycosylated in its hinge region (mucin-type core-1 *O*-glycans) (84).

##### Pinpointing of O-glycosylation Sites

Pinpointing the correct *O*-glycosylation sites is a crucial but very challenging task. Proteinase K, in this regard, proved to be beneficial as it can generate short glycopeptides, which exhibit only one potential *O*-glycosylation site. In case the occupied *O*-glycosylation site could not be inferred directly, we have employed ETD-MS^2^ fragmentation. In first attempts database-assisted peptide identification via MASCOT was tested on the acquired ETD glycopeptide spectra, but turned out to be not successful. One reason for this is the presence of intense signals in the ETD-MS^2^ spectrum, which do not correspond to peptide fragment ions (*e.g.* unfragmented precursor ions, glycan fragment ions), and which thus compromise automated peptide identification (85). A possible solution for this is the (manual) removal of these additional *m*/*z*-values from the ETD-spectra before running the search algorithm. In the present study, however, this procedure did not improve the database-assisted peptide identification. For these reasons we relied on manual spectra annotation and interpretation using *DataAnalysis, Biotools* as well as public repositories (UniProtKB and UniCarbKB). Furthermore, NetOGlyc 4.0 was employed to predict *O*-glycosylation sites and to support experimental findings. Predicted and experimentally determined *O*-glycosylation sites were mostly in good agreement for already known *O*-glycosylation sites—however, support for potentially novel sites could only be found in a few cases. A general shortcoming of glycopeptide enrichment methods is that they are biased toward glycosylated peptides, while underrepresenting potential corresponding aglyosylated counterparts. Hence, in the present study no conclusions with respect to the macro-heterogeneity of the glycoproteins (site-occupancy) can be drawn.

##### Caveats of the Approach

In contrast to tryptic (glyco)peptides, Proteinase K generated peptides and glycopeptides cannot be predicted because of the broad cleavage specificity of the enzyme. More importantly, though, is the reduced peptide length compared with a tryptic digest, as this can lead to an insufficient number of detected fragment ions to allow for unambiguous peptide identifications. This problem can be even more intensified by the frequent occurrence of prolines within mucin-type *O*-glycopeptide sequences, as prolines can introduce additional sequence gaps during mass spectrometry-based peptide sequencing. Also important to note is the increased search space of the search engine because of the use of a nonspecific enzyme, which results in an increased ambiguity with respect to the peptide identification (lower identification scores) and longer search times. A confounding factor that relates to the ETD analysis is the predominance of charge state 2^+^ among the measured *O*-glycopeptide precursor ions, because ETD fragmentation is more efficient for precursor charge states greater than 2^+^ (86). The predominance of charge state 2^+^ can be explained by a lack of ionizable/basic amino acids (lack of Arg, Lys, His) within the glycopeptides—a characteristic that can be linked to the broad-specific proteolytic digest by Proteinase K (87). Another caveat is related to the HILIC glycopeptide enrichment: this step was optimized to enrich *O*-glycopeptides carrying short mucin-type core-1 and -2 *O*-glycans, as they represent the vast majority of *O*-glycans on human blood plasma proteins (25). Hence, *O*-glycopeptides carrying bigger and thus more hydrophilic *O*-glycans, such as *N*-acetyl-lactosamine (LacNAc) extended mucin-type core-2 *O*-glycans, or O-glycopeptides carrying multiple mucin-type *O*-glycans, might elute in the subsequent washing phase of the HILIC fractionation and as a consequence cannot be found during the analysis.

##### Summary and Outlook

In the present study we have investigated the human blood plasma mucin-type *O*-glycoproteome of healthy individuals in an explorative and nontargeted manner. To this end, we have conducted a site-specific large-scale *O*-glycoproteomic analysis, which combines a broad-specific proteolytic digest, with HILIC enrichment/fractionation and subsequent multistage mass spectrometry measurement (nano-RPLC-ESI-IT-MS^n^) with CID and ETD. Centered on the characterization and identification of intact glycopeptides, we could demonstrate the in-depth *O*-glycoproteomic analysis of a number of important human blood plasma glycoproteins (mainly acute phase proteins), including alpha-2-HS-glycoprotein, fibrinogen, plasminogen and kininogen-1. Our results are in good agreement with previous findings by other research groups, but also add new aspects to the field, *e.g.* the identification of a couple of novel *O*-glycosylation site as well as the benefits and drawbacks of using Proteinase K in large-scale mass spectrometric glycoproteomic studies.

Explorative site-specific *N*- and *O*-glycoproteomic studies of biofluids, like human blood plasma, human milk, urine or cerebrospinal fluid hold an enormous potential to better understand the implications of protein glycosylation under normal physiological conditions, but also under pathophysiological conditions. By serving as a diagnostic tool, the detection/discovery of relevant glycopeptides (biomarker candidates) can be the basis for targeted quantitative glycoproteomic analyses, which allow for a site-specific monitoring of glycosylation alterations, *e.g.* during disease progression. Site-specific glycosylation analyses are, moreover, important to produce biopharmaceuticals according to quality by design requirements, in particular if these biopharmaceuticals are produced in heterologous expression systems. In this regard site-specific glycosylation analyses might also enable understanding/controlling important glycan-related features of the final product including its efficacy, half-life, or antigenicity.

## Supplementary Material

Supplemental Data

## Footnotes

^1^ The abbreviations used are:

LC: liquid chromatography
ECD: Electron-capture dissociation
ETD: Electron-transfer dissociation
FT-ICR: Fourier transform ion cyclotron resonance
HILIC: Hydrophilic interaction liquid chromatography
IAA: Iodoacetamide
ICC: Ion charge control
LTQ: Linear trap quadrupole.
