# Supplementary material for: Site-specific O-Glycosylation Analysis of Human Blood Plasma Proteins
Source: Mol Cell Proteomics. 2015 Nov 23;15(2):624–41. doi: 10.1074/mcp.M115.053546 (PMC4739677; doi:10.1074/mcp.M115.053546)
Supplement: Supplemental Data [file supp_15_2_624__index.html]

Site-specific O-Glycosylation Analysis of Human Blood Plasma Proteins — Site-specific O-Glycosylation Analysis of Human Blood Plasma Proteins — Site-specific O-Glycosylation Analysis of Human Blood Plasma Proteins — Supplemental Data 

# Site-specific *O*-Glycosylation Analysis of Human Blood Plasma Proteins

## Supplemental Data

- Supplemental Figure 1 (.pdf, 355 KB) - Proteinase K Digest Reproducibility
- Supplemental Figure 2 (.pdf, 292 KB) - Peptide Length Obtained with Proteinase K
- Supplemental Figure 3 (.pdf, 1.3 MB) - O-Glycoproteomic Analysis: HILIC Fraction #13
- Supplemental Figure 4 (.pdf, 2.5 MB) - O-Glycoproteomic Analysis: HILIC Fraction #14
- Supplemental Figure 5 (.pdf, 4.6 MB) - O-Glycoproteomic Analysis: HILIC Fraction #15
- Supplemental Figure 6 (.pdf, 4.2 MB) - O-Glycoproteomic Analysis: HILIC Fraction #16
- Supplemental Figure 7 (.pdf, 2.9 MB) - O-Glycoproteomic Analysis: HILIC Fraction #17
- Supplemental Table 1 (.xlsx, 55 KB) - Protein Identification: CID vs ETD
- Supplemental Table 2 (.xlsx, 275 KB) - Protein Identification: CID
- Supplemental Table 3 (.xlsx, 239 KB) - Protein Identification: ETD
