# Supplementary material for: Site-specific O-Glycosylation Analysis of Human Blood Plasma Proteins
Source: Mol Cell Proteomics. 2015 Nov 23;15(2):624–41. doi: 10.1074/mcp.M115.053546 (PMC4739677; doi:10.1074/mcp.M115.053546)
Supplement: Supplemental Data [file 10.1074_M115.053546_mcp.M115.053546-1.pdf]

Reproducibility of the Proteinase K Digest

- Proteinase K digest of human blood plasma: 5 independent replicates
- Same digest conditions as for the analytical samples
- No post-digest clean-up
- Samples measured with nanoRP-LC-ESI IT MS/MS

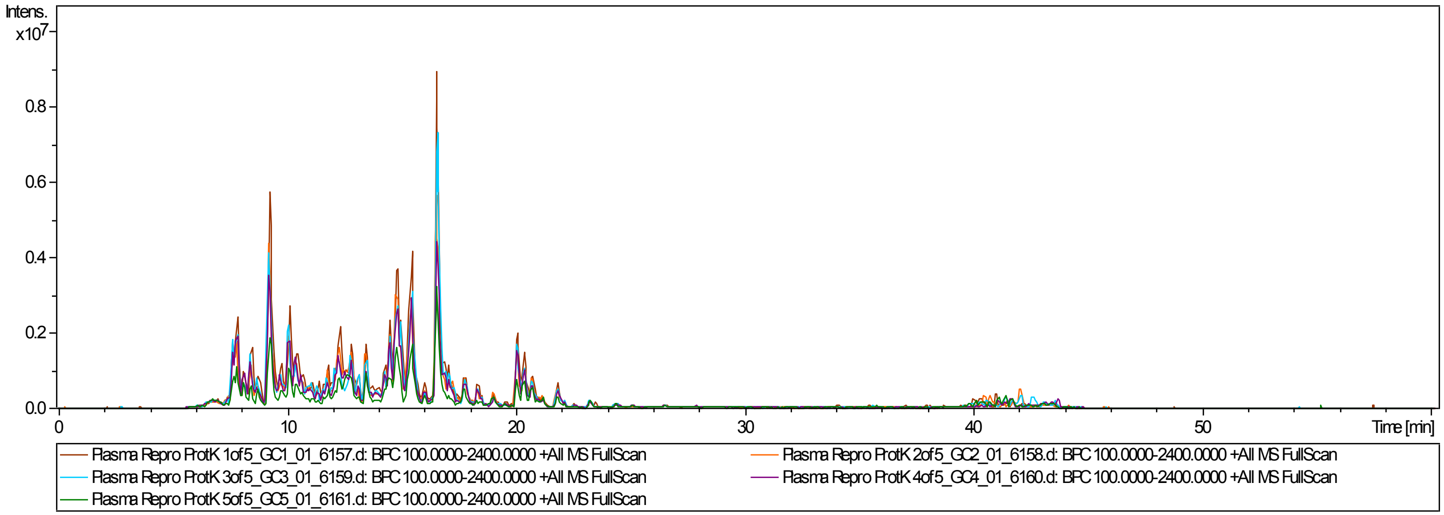

Fig.: Base peak chromatograms (MS) of five proteinase K digests measured with nanoRP-LC-ESI IT MS/MS.

Reproducibility of the Proteinase K Digest

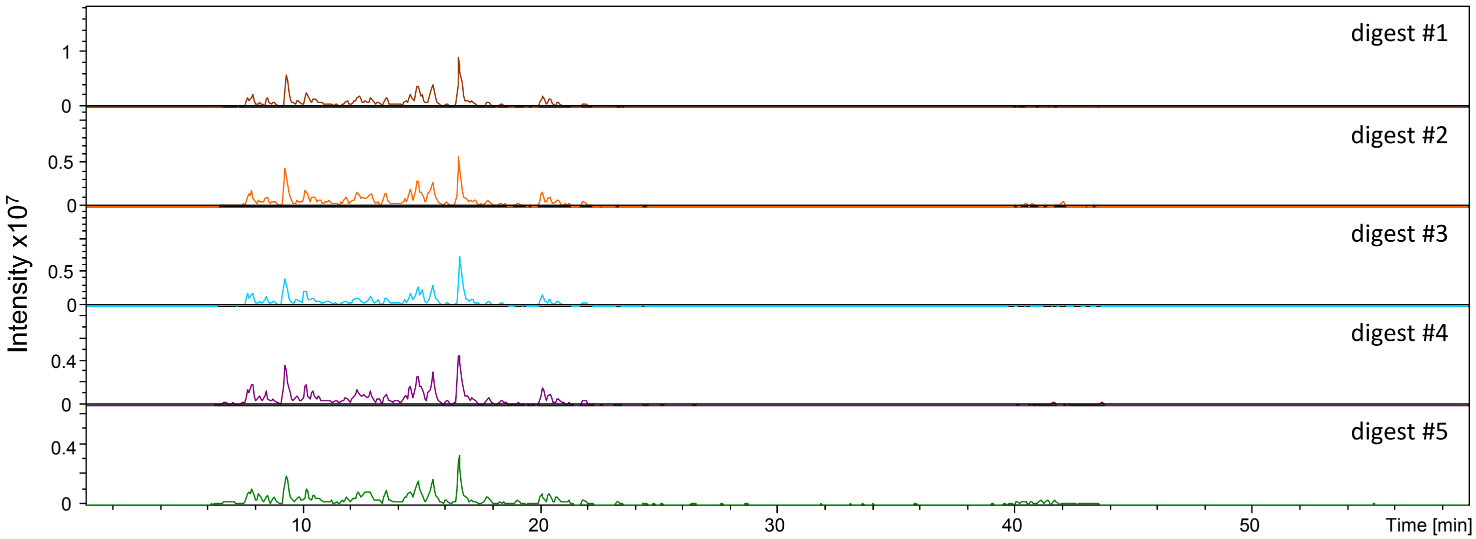

Fig.: Base peak chromatograms (MS) of five proteinase K digests measured with nanoRP-LC-ESI IT MS/MS.
