# Supplementary material for: Site-specific O-Glycosylation Analysis of Human Blood Plasma Proteins
Source: Mol Cell Proteomics. 2015 Nov 23;15(2):624–41. doi: 10.1074/mcp.M115.053546 (PMC4739677; doi:10.1074/mcp.M115.053546)
Supplement: Supplemental Data [file 10.1074_M115.053546_mcp.M115.053546-2.pdf]

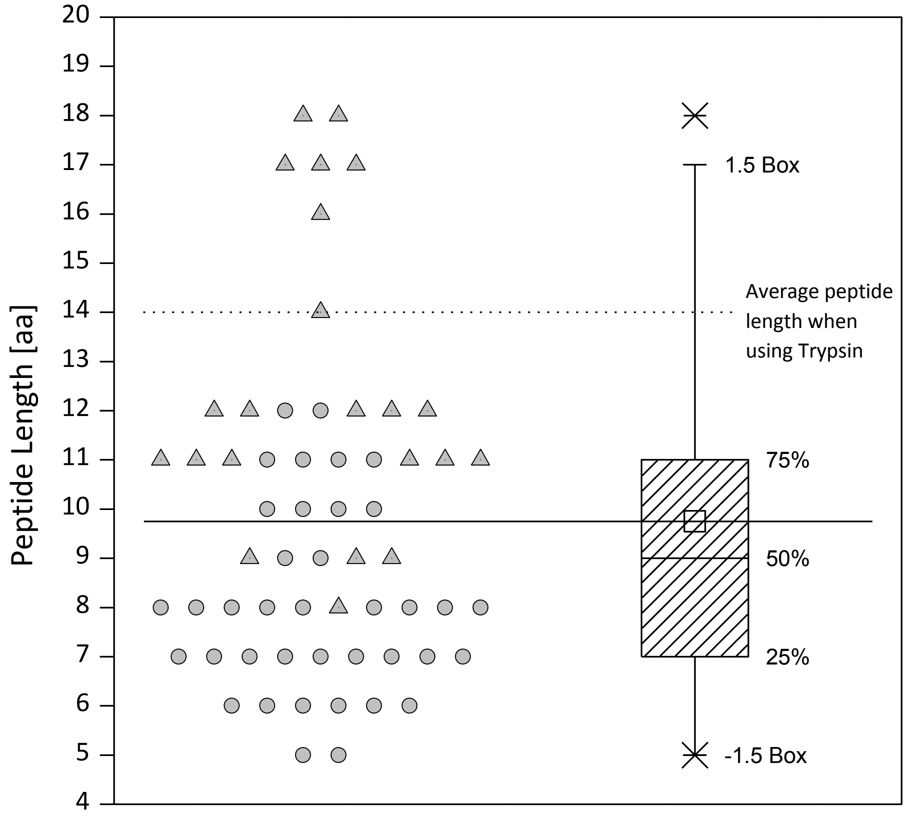

Figure: Box-and-whisker plot of the peptide length obtained with Proteinase K (in total 60 peptides). The horizontal line within the box indicates the median, boundaries of the box mark the upper and lower quartile (Q_1, Q_2, Box=interquartile range (IQR)). The whiskers indicate Q_1-(1.5xIQR) and Q_3+(1.5xIQR). The mean value is illustrated by square within the box. Outliers are marked with an "X". Data points plotted next to the Box-and-whisker plot are indexed with respect to the peptide charge state (circle=doubly charged, triangle=triply charged). Dotted line: average peptide length for a tryptic digest (based on an in-silico digestion of the human Uniprot database, (54)).
