# Supplementary material for: Site-specific O-Glycosylation Analysis of Human Blood Plasma Proteins
Source: Mol Cell Proteomics. 2015 Nov 23;15(2):624–41. doi: 10.1074/mcp.M115.053546 (PMC4739677; doi:10.1074/mcp.M115.053546)
Supplement: Supplemental Data [file 10.1074_M115.053546_mcp.M115.053546-3.pdf]

Site-Specific *O*-Glycosylation Analysis of Human Blood Plasma Proteins

Proteinase K Digest

HILIC Fraction 13

Search Parameters For Protein Identification

Project: Blood Plasma Glycoprotomics (2013), ProtK-Digest
Glycopeptides measured on Bruker ESI-Ion Trap MS (CID-MS^3)

Mascot version 2.2.07

Database:	SwissProt
Fasta file:	SwissProt_51.6.fasta
Total sequences:	257964
Total residues:	93947433
Sequences after taxonomy filter:	15720
Number of queries:	1

Variable modifications ---

Identifier	*Name*	*Delta*	*Neutral loss(es)*
1	Deamidated (NQ)	0.984009	0
2	Oxidation (M)	15.994919	63.998285

Search Parameters ---

Taxonomy filter:	Homo sapiens (human)
Enzyme:	None
Maximum Missed Cleavages:	0
Fixed modifications	Carbamidomethyl (C)
ICAT experiment	0
Variable modifications	Deamidated (NQ), Oxidation (M)
Peptide Mass Tolerance	0.3
Peptide Mass Tolerance Units	Da
Fragment Mass Tolerance	0.35
Fragment Mass Tolerance Units	Da
Mass values	Monoisotopic
Instrument type	ESI-TRAP
Isotope error mode	1

Format parameters ---

Significance threshold	0.05
Max. number of hits	20
Use MudPIT protein scoring	0
Ions score cut-off	0
Include same-set proteins	0
Include sub-set proteins	0
Include unassigned	0
Require bold red	0

Extracted ion chromatograms of glycan-specific oxonium ions

Oxonium Ions	$[M+H]^+$ m/z
Fuc	147.08
Hex	163.06
HexNAc	204.09
NeuAc -H_2O	274.09
NeuAc	292.10
HexNAc(1)Hex(1)	366.14
Hex(1)NeuAc(1)	454.16
HexNAc(1)NeuAc(1)	495.18
HexNAc(1)Hex(1)Fuc(1)	512.21
HexNAc(1)Hex(2)	528.19
HexNAc(1)Hex(1)NeuAc(1)	657.24

Supplementary Figure 3: Human Blood Plasma O-Glycoproteomics, HILIC Fraction 13

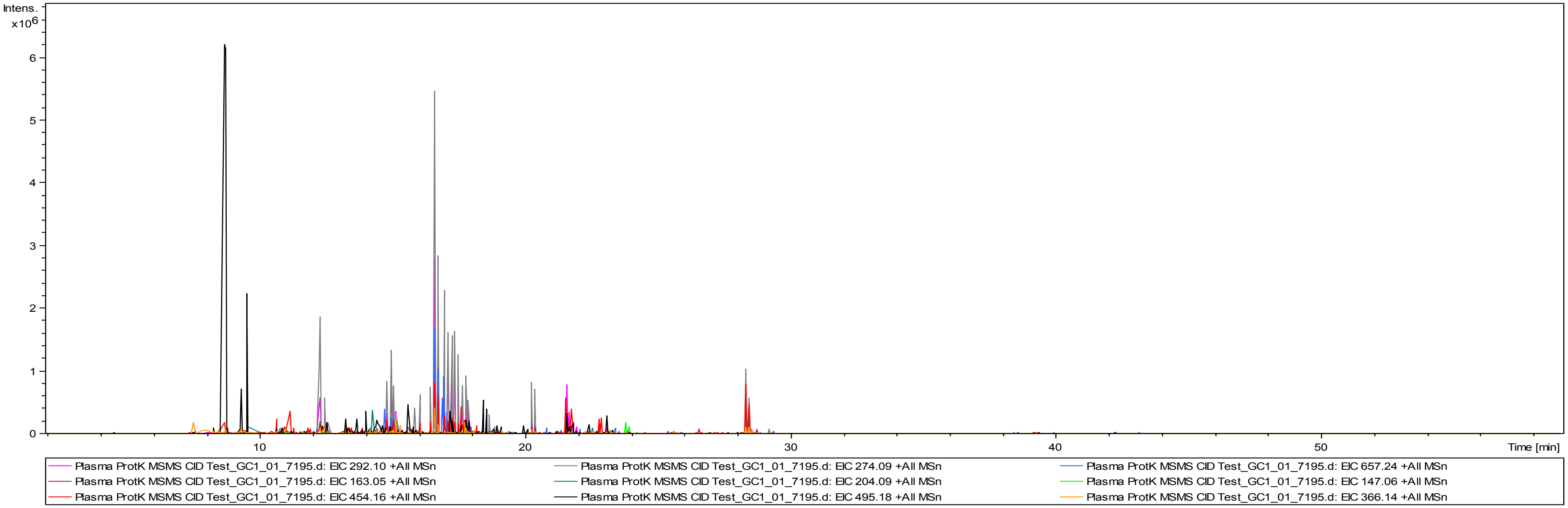

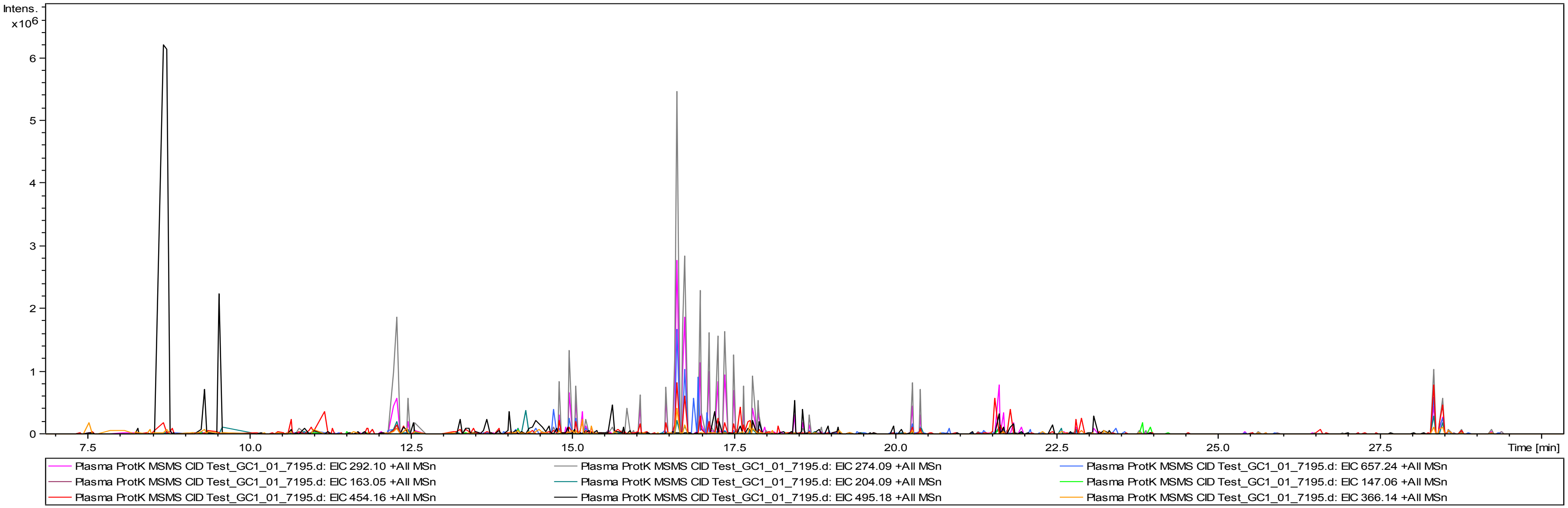

Extracted ion chromatograms of glycan-specific oxonium ions

Supplementary Figure 3: Human Blood Plasma O-Glycoproteomics, HILIC Fraction 13

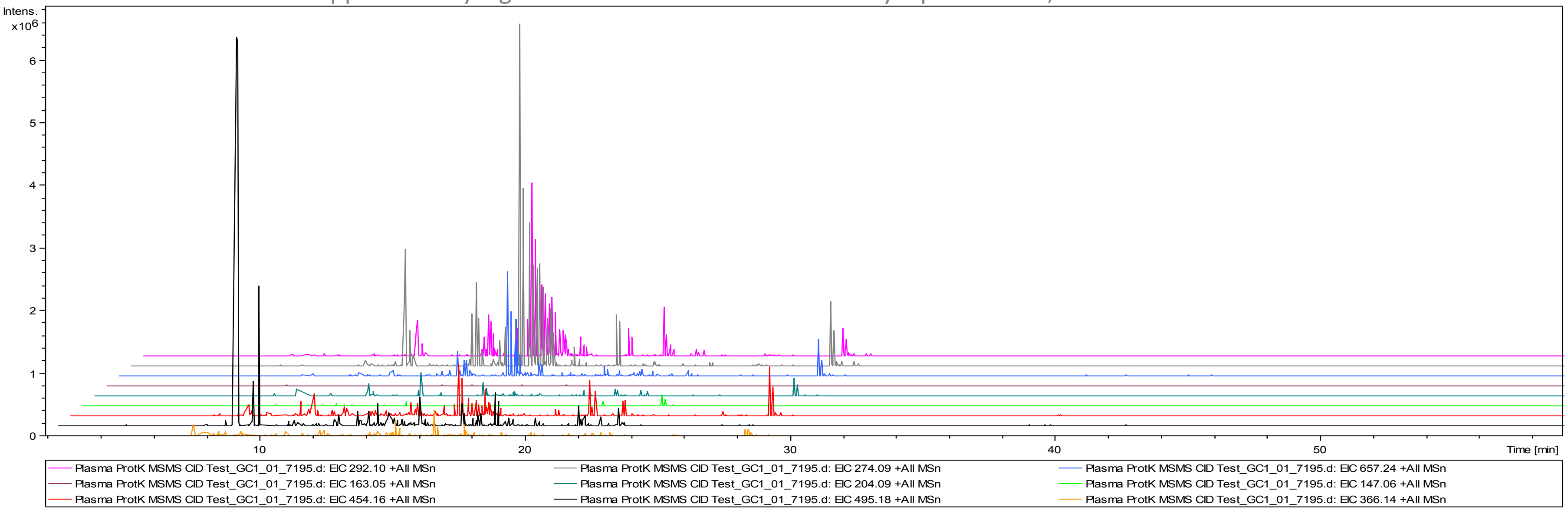

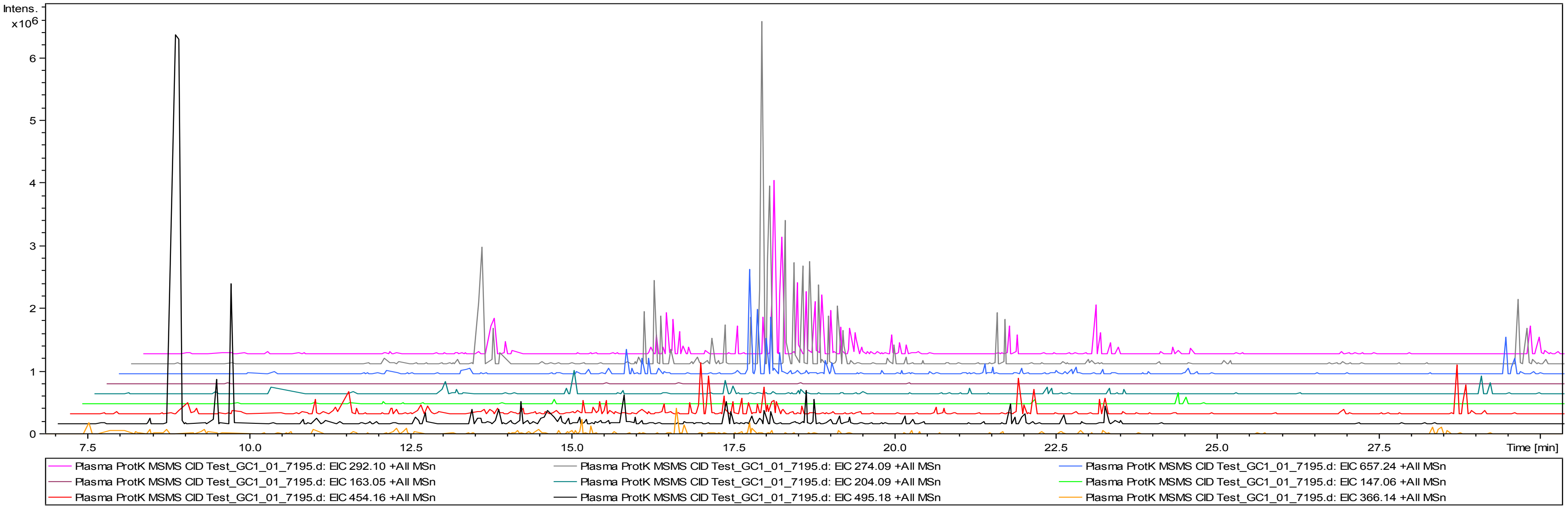

Extracted ion chromatograms of glycan-specific oxonium ions

Supplementary Figure 3: Human Blood Plasma O-Glycoproteomics, HILIC Fraction 13

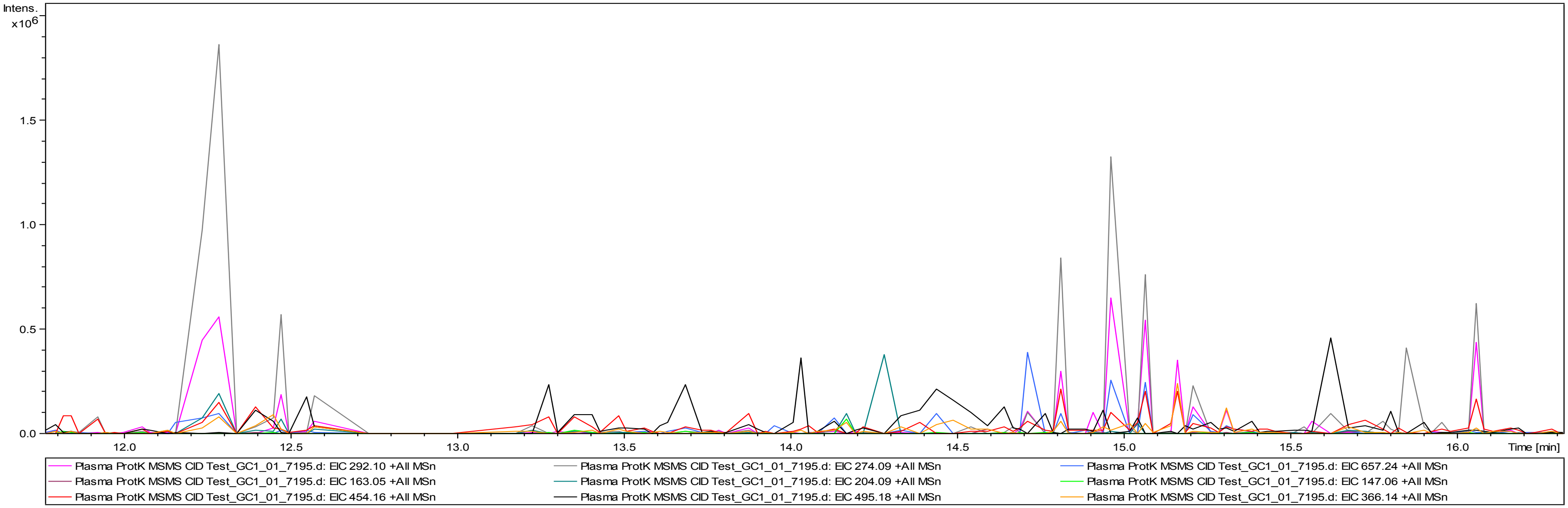

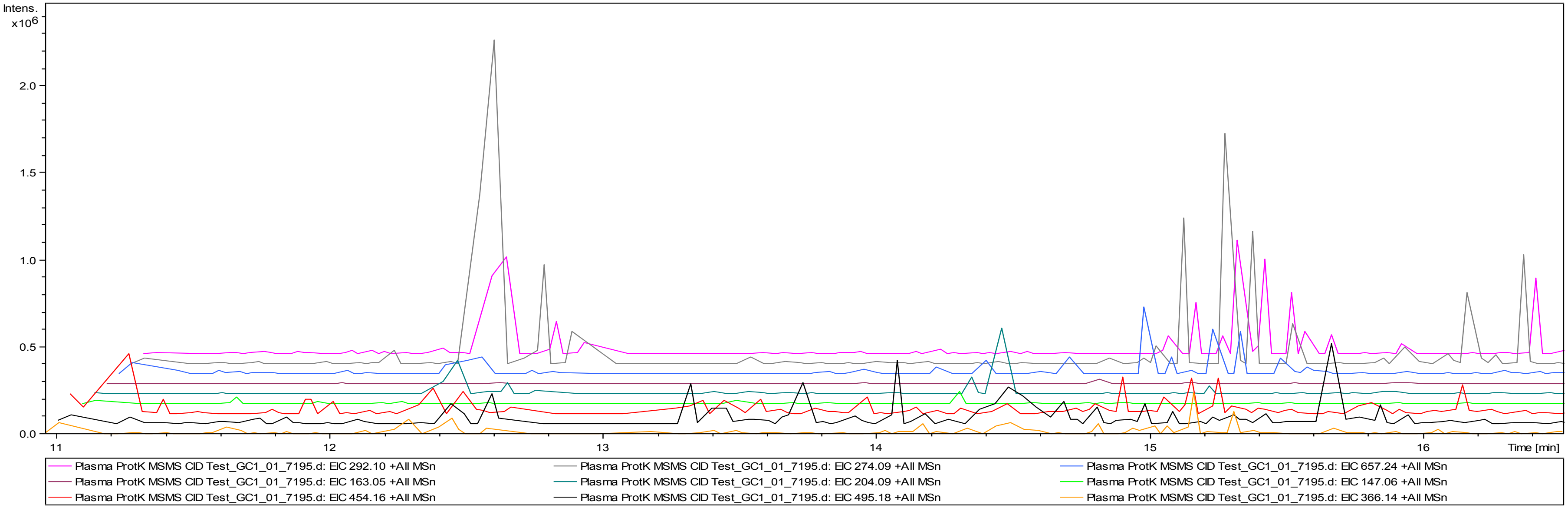

Extracted ion chromatograms of glycan-specific oxonium ions

Supplementary Figure 3: Human Blood Plasma O-Glycoproteomics, HILIC Fraction 13

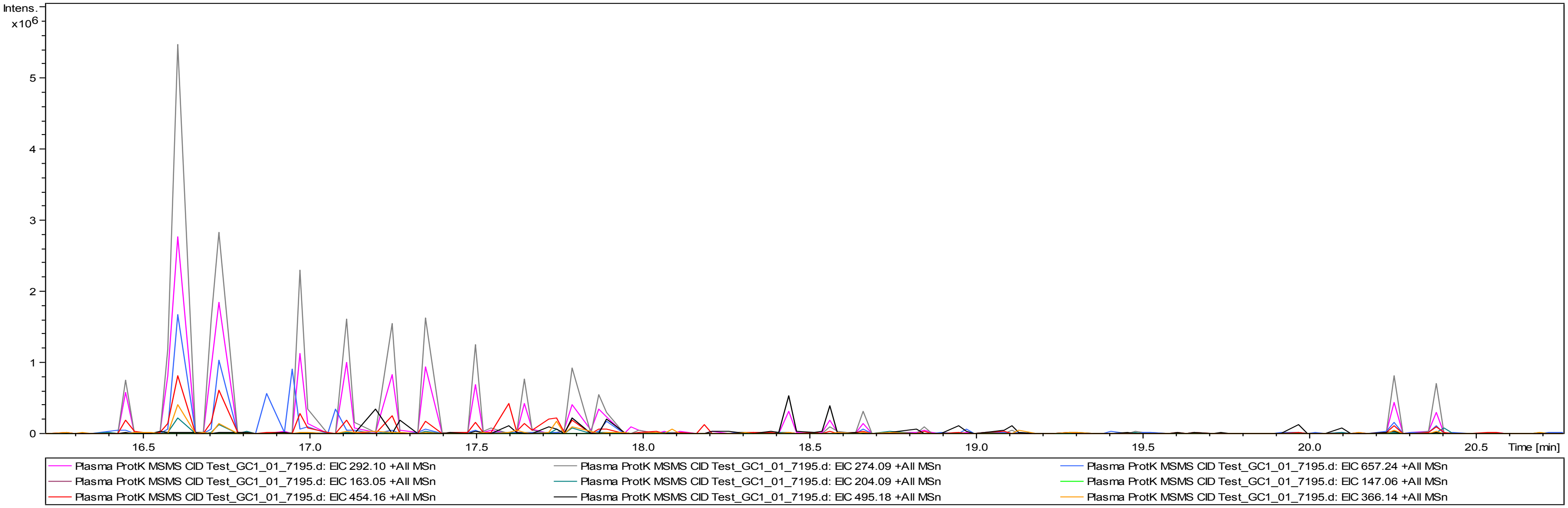

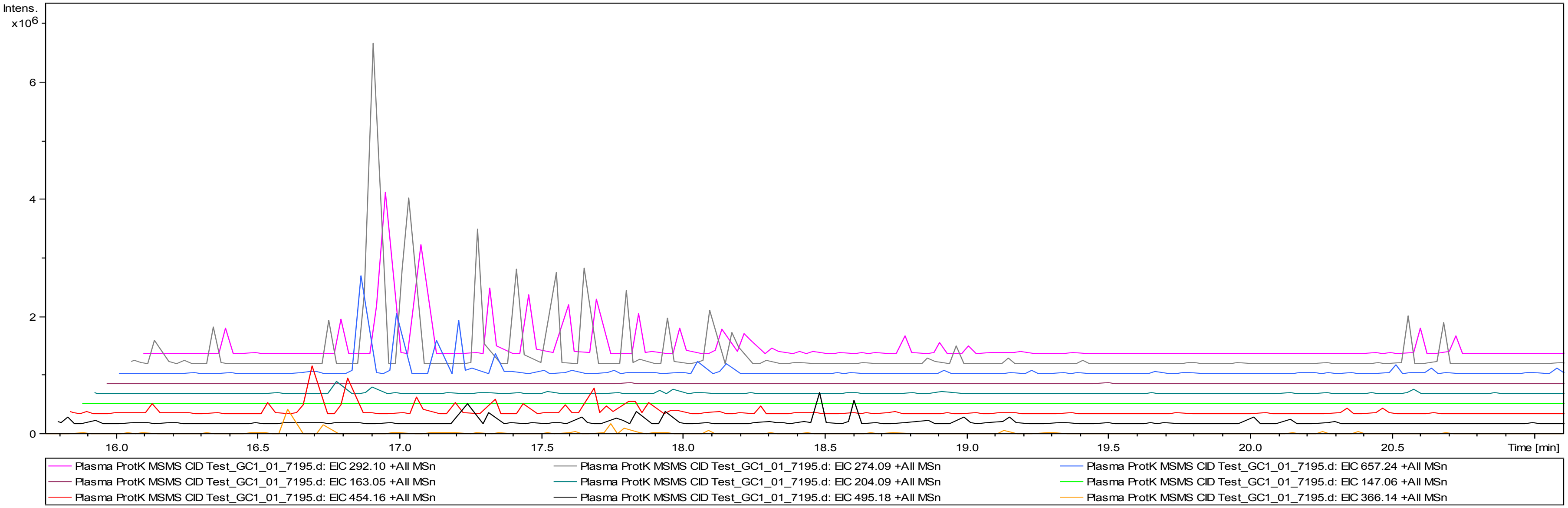

Extracted ion chromatograms of glycan-specific oxonium ions

Supplementary Figure 3: Human Blood Plasma O-Glycoproteomics, HILIC Fraction 13

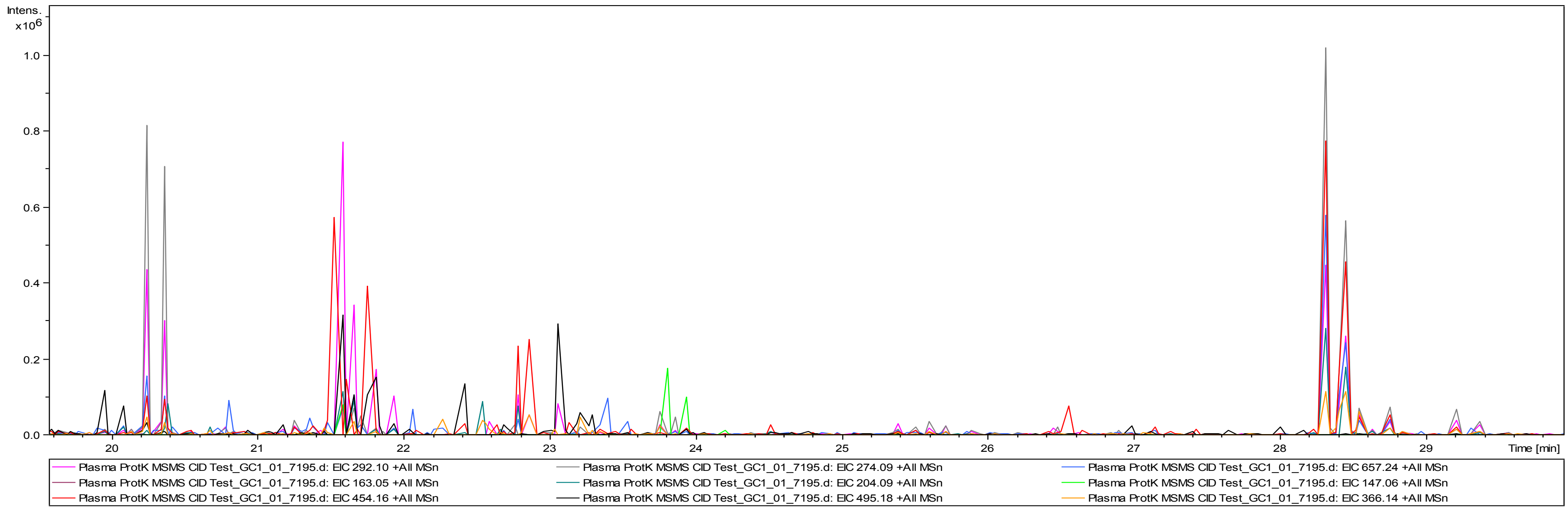

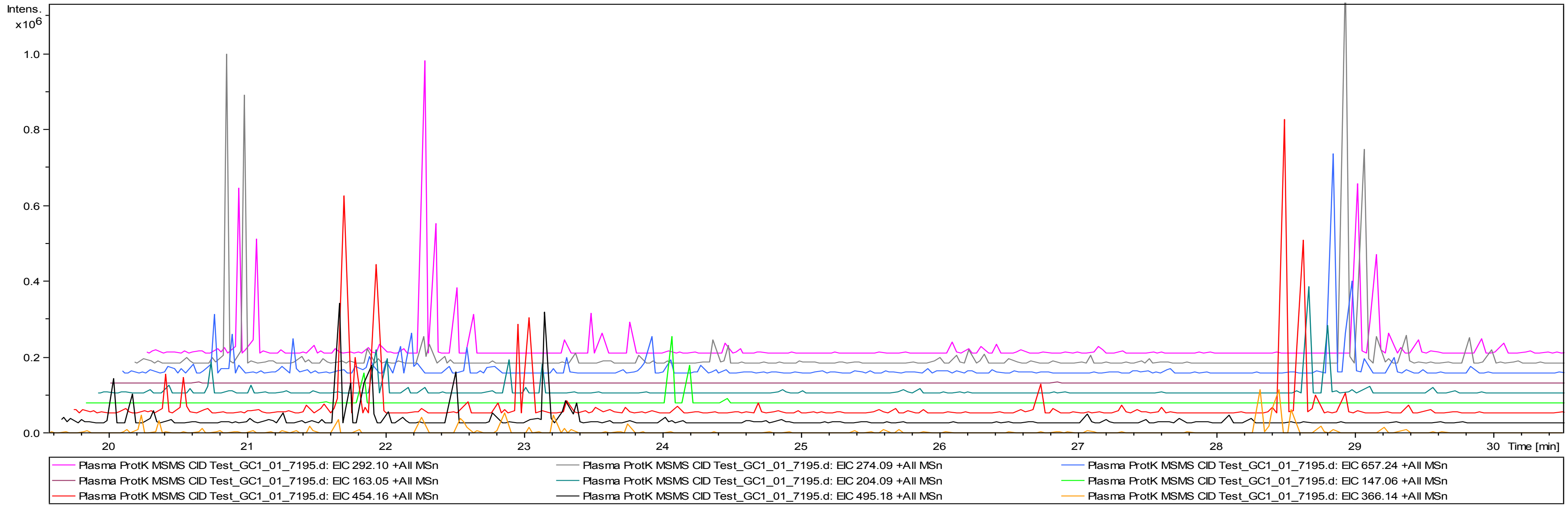

Extracted ion chromatograms of glycan-specific oxonium ions

Fraction 13

721.78++ → Pep [M+H]+ 786.39+ [17.8-17.9 min]

CID-MS Precursor

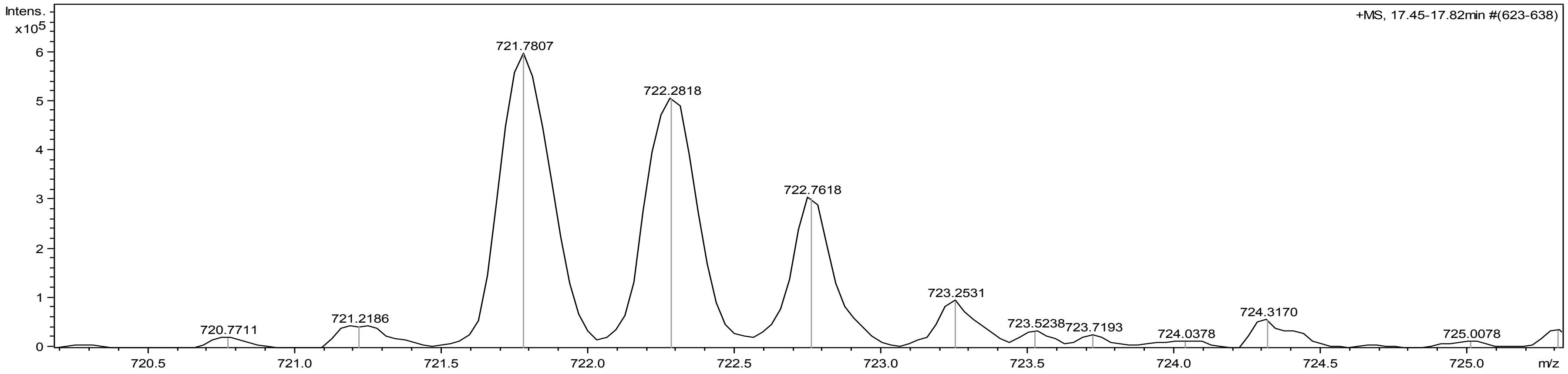

Fraction 13

721.78++ → Pep [M+H]+ 786.39+ [17.8-17.9 min] CID-MS2

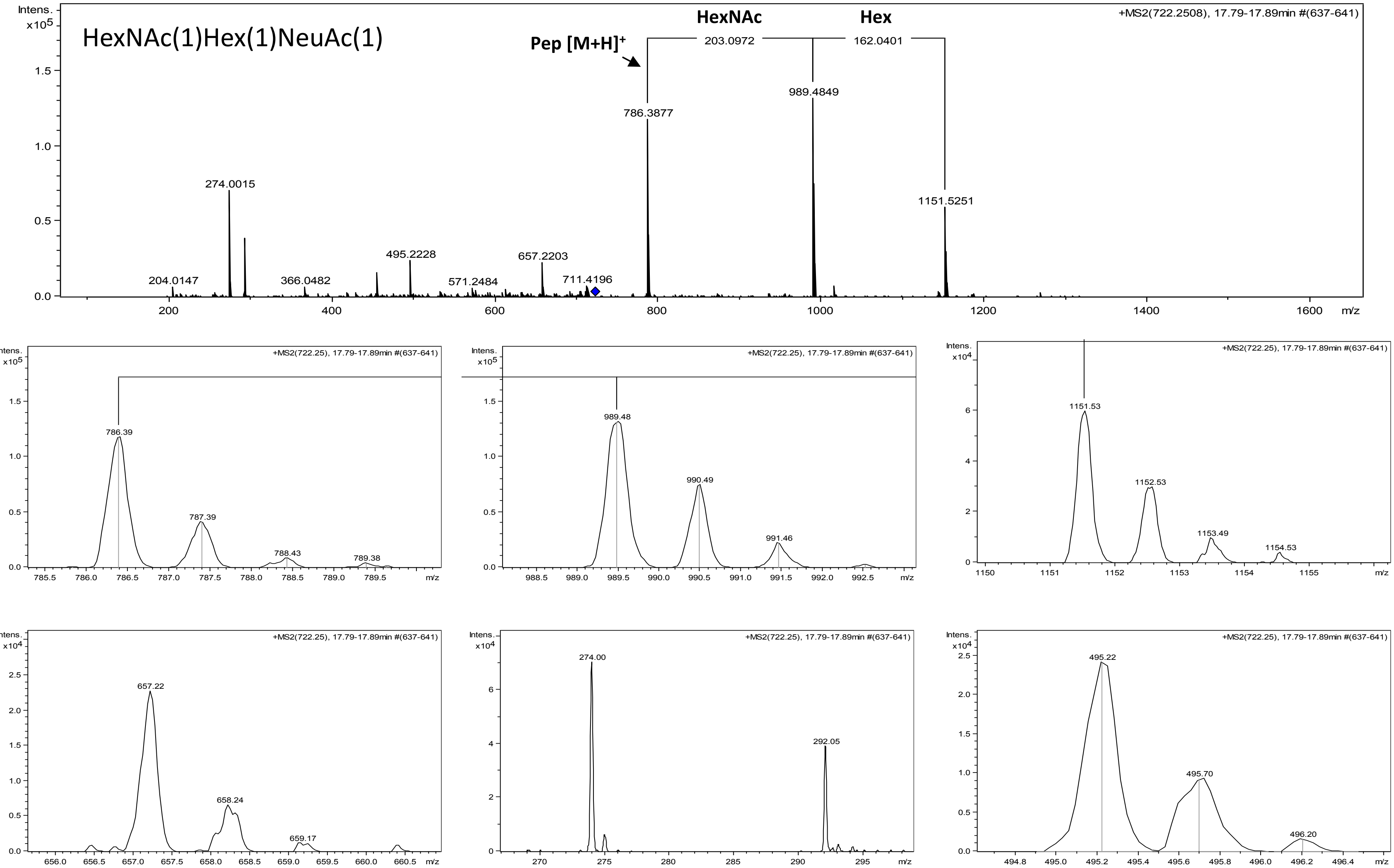

Fraction 13

721.78++ → Pep [M+H]+ 786.39+ [17.8-17.9 min] CID-MS3 Manual DeNovo

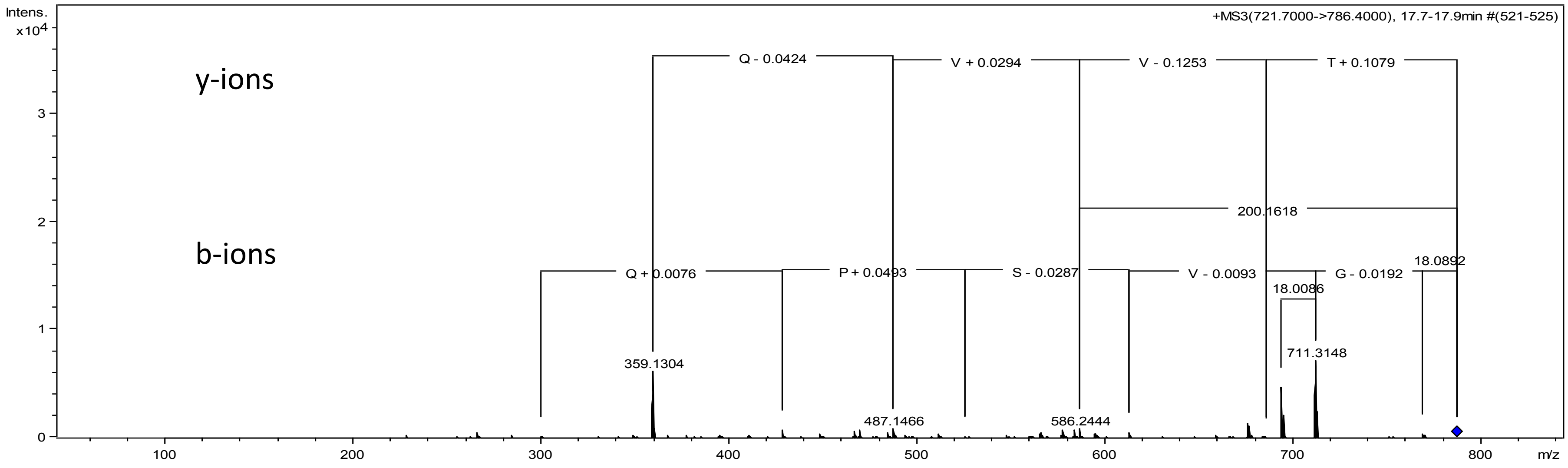

known O-glycosylation site

Alpha-2-HS-glycoprotein

$_{341}$**TVVQPSVG**$_{348}$

Fraction 13

721.78++ → Pep [M+H]+ 786.39+ [17.8-17.9 min] CID-MS3

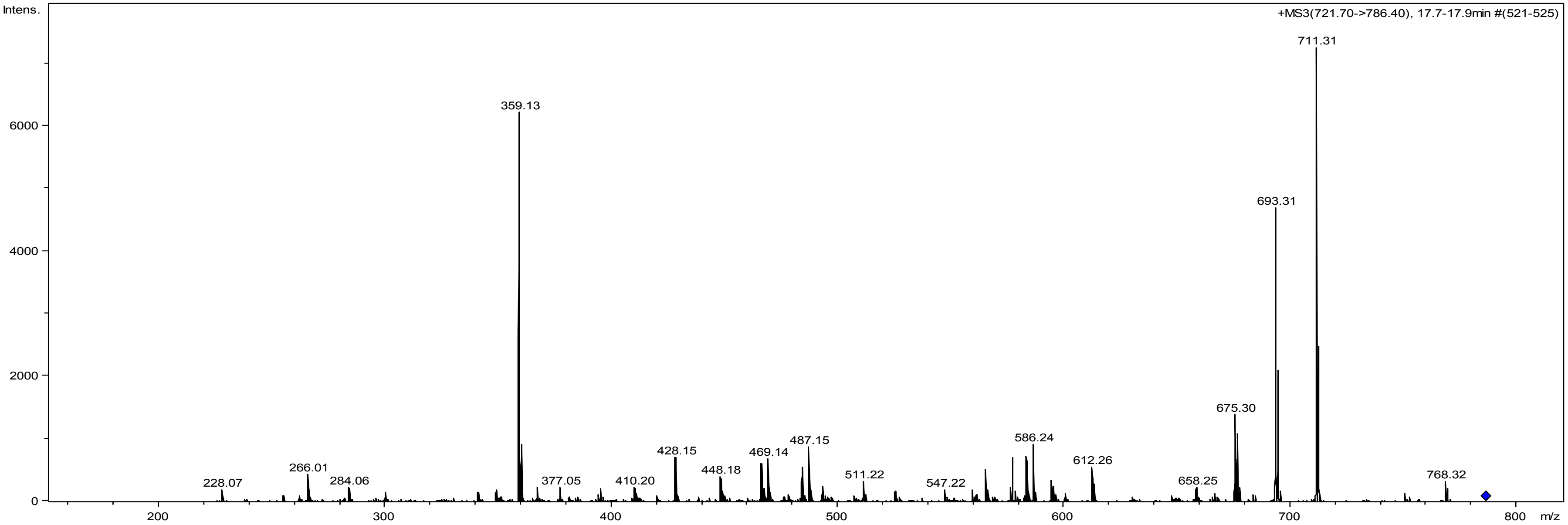

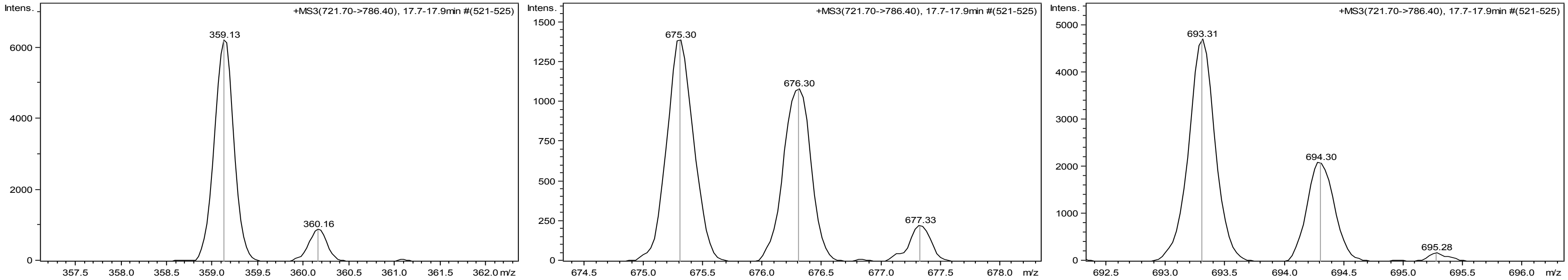

known O-glycosylation site

Alpha-2-HS-glycoprotein

$_{341}$**TVVQPSVG**$_{348}$

Fraction 13

721.78++ → Pep [M+H]+ 786.39+ [17.8-17.9 min] CID-MS3

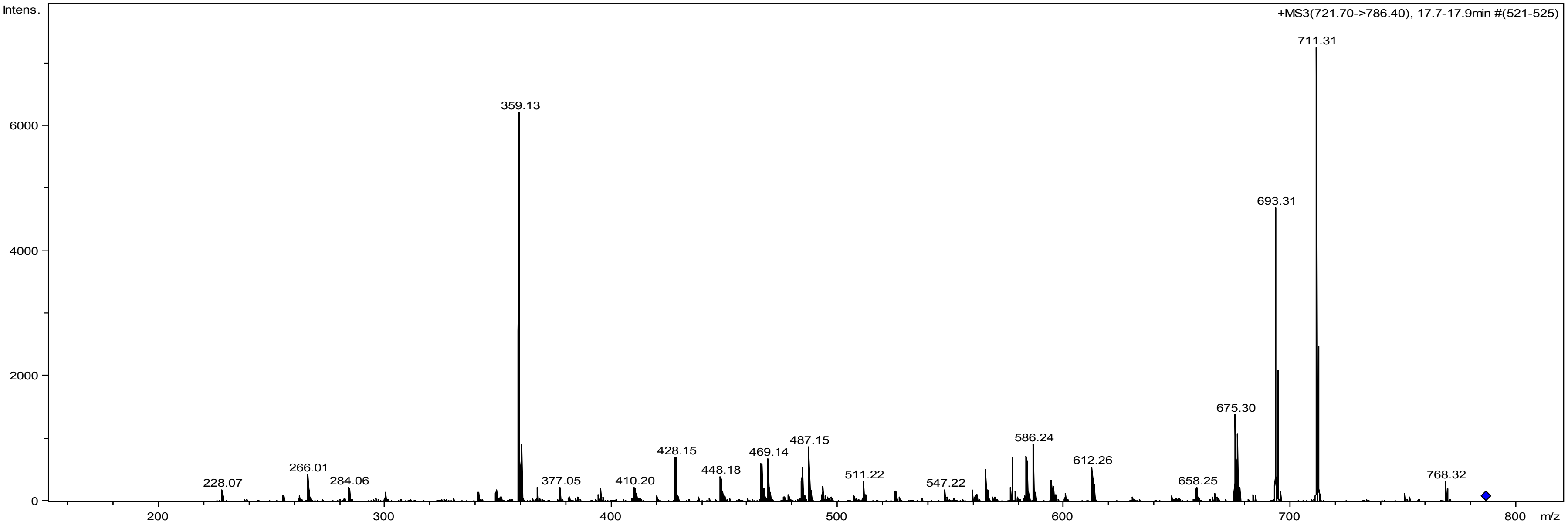

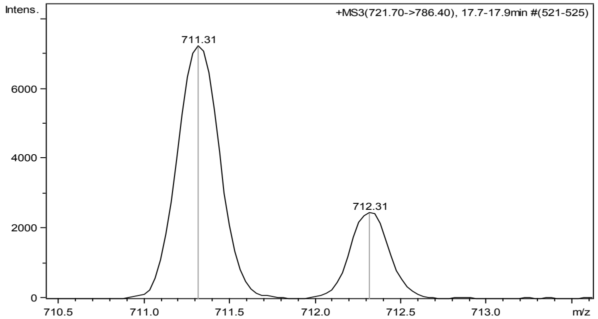

known O-glycosylation site

Alpha-2-HS-glycoprotein

$_{341}$**TVVQPSVG**$_{348}$

Fraction 13

721.78++ → Pep [M+H]+ 786.39+ [17.8-17.9 min]   CID-MS3   MASCOT Search

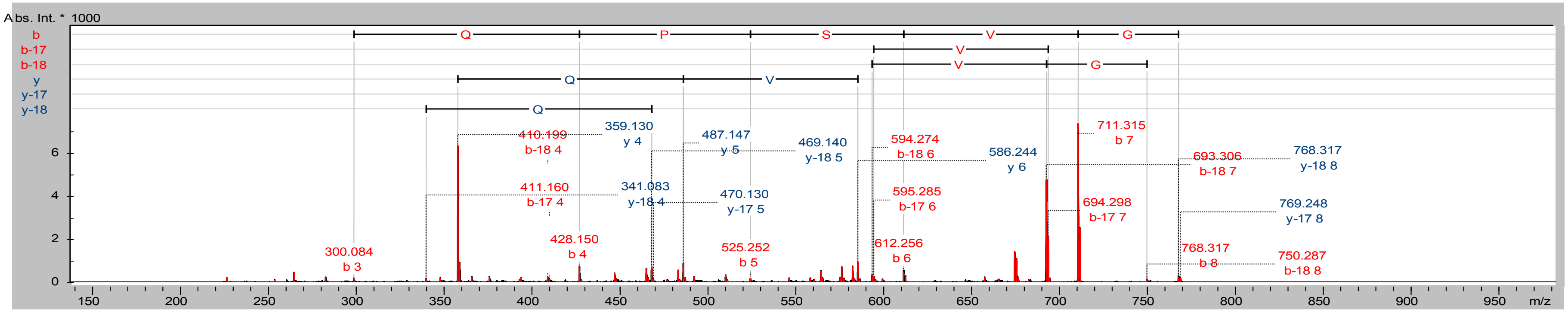

	T	V	V	Q	P	S	V	G	Thr	Val	Val	Gln	Pro	Ser	Val	Gly
Ion	1	2	3	4	5	6	7	8	1	2	3	4	5	6	7	8
b	T	V	V	Q	P	S	V	G	102.055	201.123	300.192	428.250	525.303	612.335	711.404	768.425
b-17	T	V	V	Q	P	S	V	G	-	-	-	411.224	508.277	595.309	694.377	751.398
b-18	T	V	V	Q	P	S	V	G	84.044	183.113	282.181	410.240	507.293	594.325	693.393	750.414
y	T	V	V	Q	P	S	V	G	76.039	175.108	262.140	359.193	487.251	586.320	685.388	786.436
y-17	T	V	V	Q	P	S	V	G	-	-	-	-	470.225	569.293	668.361	769.409
y-18	T	V	V	Q	P	S	V	G	-	-	244.129	341.182	469.241	568.309	667.377	768.425
	8	7	6	5	4	3	2	1	Gly	Val	Ser	Pro	Gln	Val	Val	Thr

known O-glycosylation site

Alpha-2-HS-glycoprotein

$_{341}$**TVVQPSVG**$_{348}$

Fraction 13

721.78++ → Pep [M+H]+ 786.39+ [17.8-17.9 min] CID-MS3 MASCOT Search

prot_hit_nur	prot_acc	prot_desc	prot_score	prot_mass	prot_matche	pep_query	pep_rank	pep_isbold	pep_exp_mz	pep_exp_mr	pep_exp_z	pep_calc_mr	pep_delta	pep_miss	pep_score	pep_expect	pep_res_bef	pep_seq
1	FETUA_HUM	Alpha-2-HS-g	18	40098	1	1	1	1	786.37	785.3627	1	785.4283	-0.0656	0	23.6	54	R	TVVQPSVG
2	ITBP2_HUMA	Integrin beta	18	39213	1	1	2	0	786.37	785.3627	1	785.4283	-0.0656	0	22.7	67	I	SLVKADPG
3	SMC6_HUMA	Structural ma	17	127216	1	1	2	0	786.37	785.3627	1	785.3919	-0.0292	0	22.7	67	S	ISVQPGEG
4	GRP78_HUM	78 kDa gluco	16	72402	1	1	5	0	786.37	785.3627	1	784.4443	0.9184	0	21.95	79	Y	GAAVQAGVL
5	DEN4C_HUM	DENN domai	16	182536	1	1	2	0	786.37	785.3627	1	785.4647	-0.102	0	22.7	67	S	SIVKVPSG
6	CNKR1_HUM	Connector er	15	80512	1	1	6	0	786.37	785.3627	1	785.3919	-0.0292	0	21.24	93	E	SPDKSPVG
7	ZN592_HUM	Zinc finger p	14	140148	1	1	6	0	786.37	785.3627	1	785.3919	-0.0292	0	21.24	93	D	DPSKSPVG
8	CRSP7_HUM	CRSP comple	13	65897	1	1	9	0	786.37	785.3627	1	785.3304	0.0323	0	19.28	1.50E+02	D	ENDKHSG
9	TAB2_HUMA	Mitogen-acti	13	77017	1	1	9	0	786.37	785.3627	1	785.3304	0.0323	0	19.28	1.50E+02	R	QTQQHSG
10	NFAT5_HUM	Nuclear facto	13	166629	1	1	8	0	786.37	785.3627	1	785.349	0.0137	0	19.87	1.30E+02	M	VQMQHSG

Biotools-Score: 76

MASCOT-Score: 24

known O-glycosylation site

Alpha-2-HS-glycoprotein

$_{341}$**TVVQPSVG**$_{348}$

Fraction 13

721.78++ → Pep [M+H]+ 786.39+ [17.8-17.9 min] ETD

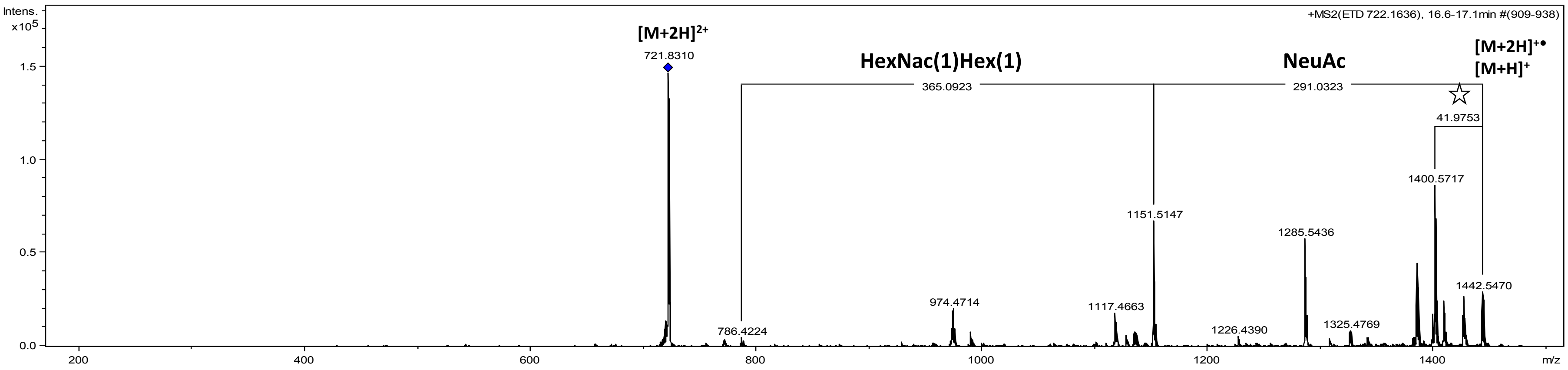

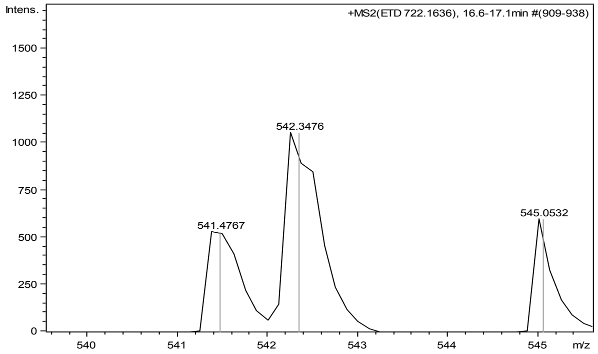

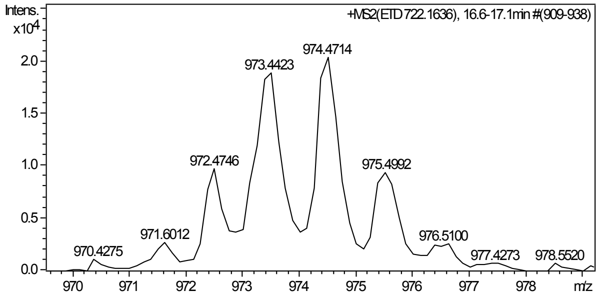

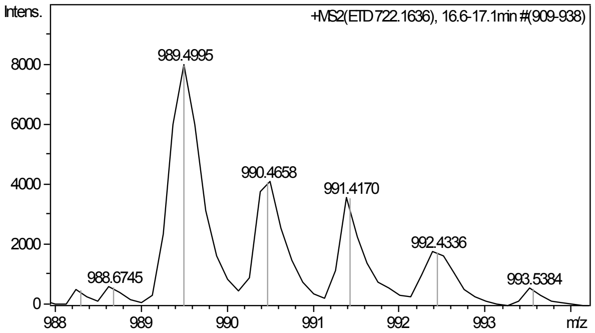

☆ The Combination of Electron Capture Dissociation and Fixed Charge Derivatization Increases Sequence Coverage for O-Glycosylated and O-Phosphorylated Peptides, Chamot-Rooke et al., American Society for Mass Spectrometry, 2007

Mormann, M.; Paulsen, H.; Peter-Katalinic, J. Electron Capture Dissociation of O-Glycosylated Peptides: Radical Site-Induced Fragmentation of Glycosidic Bond. Eur. J. Mass Spectrom. 11 2005 497 –511.

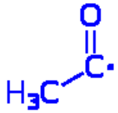

Acetyl Radical ☆

known O-glycosylation site

Alpha-2-HS-glycoprotein

$_{341}$**TVVQPSVG**$_{348}$

Fraction 13

721.78++ → Pep [M+H]+ 786.39+ [17.8-17.9 min] ETD

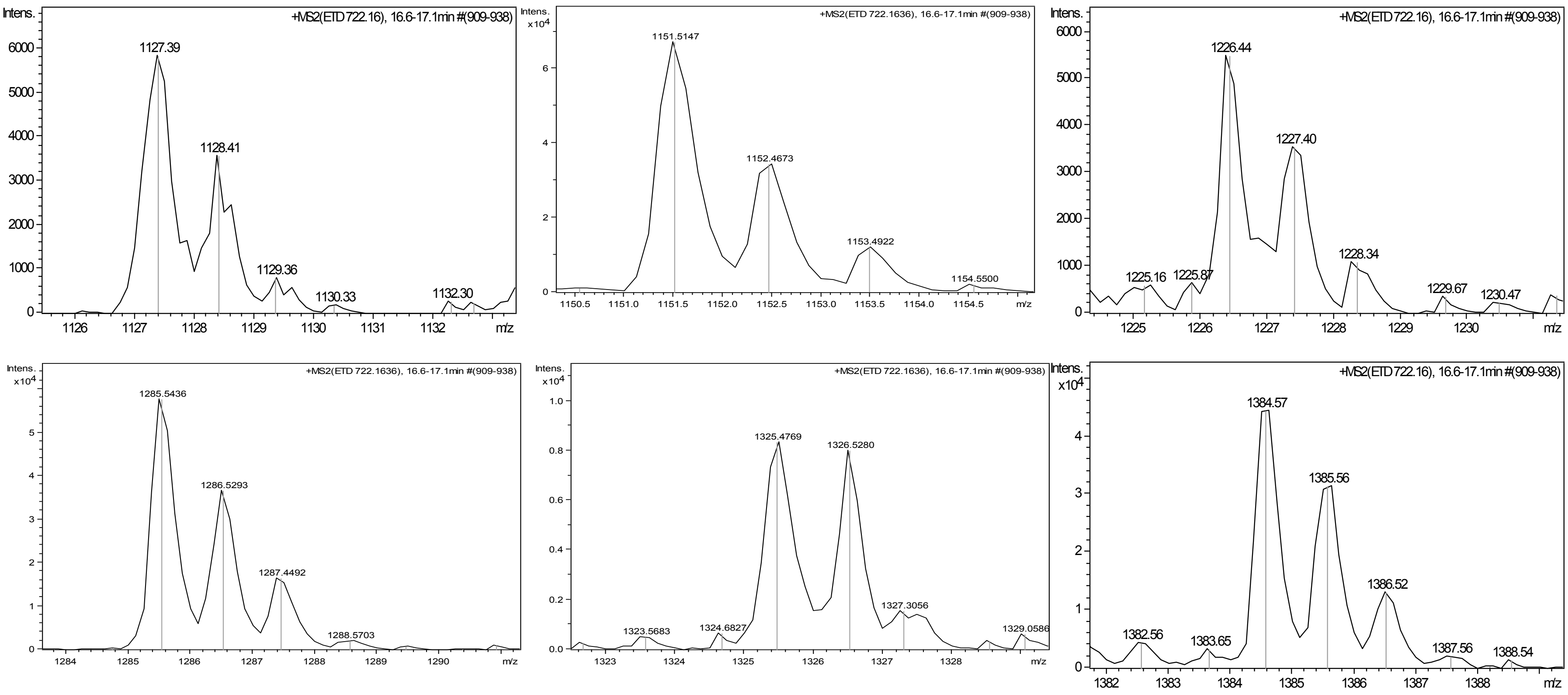

known O-glycosylation site

Alpha-2-HS-glycoprotein

$_{341}$**TVVQPSVG**$_{348}$

Fraction 13

721.78++ → Pep [M+H]+ 786.39+ [17.8-17.9 min] ETD

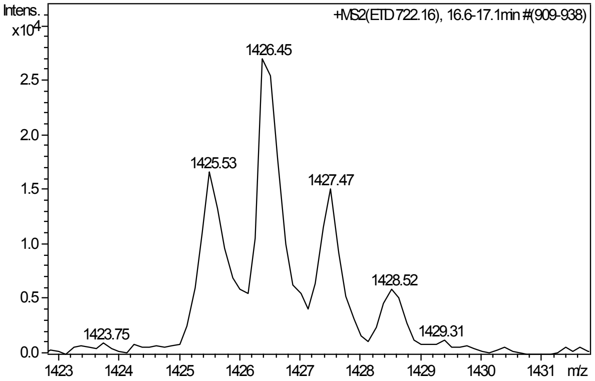

known O-glycosylation site

Alpha-2-HS-glycoprotein $_{341}$**TVVQPSVG**$_{348}$

Fraction 13

721.78++ → Pep [M+H]+ 786.39+ [17.8-17.9 min] ETD

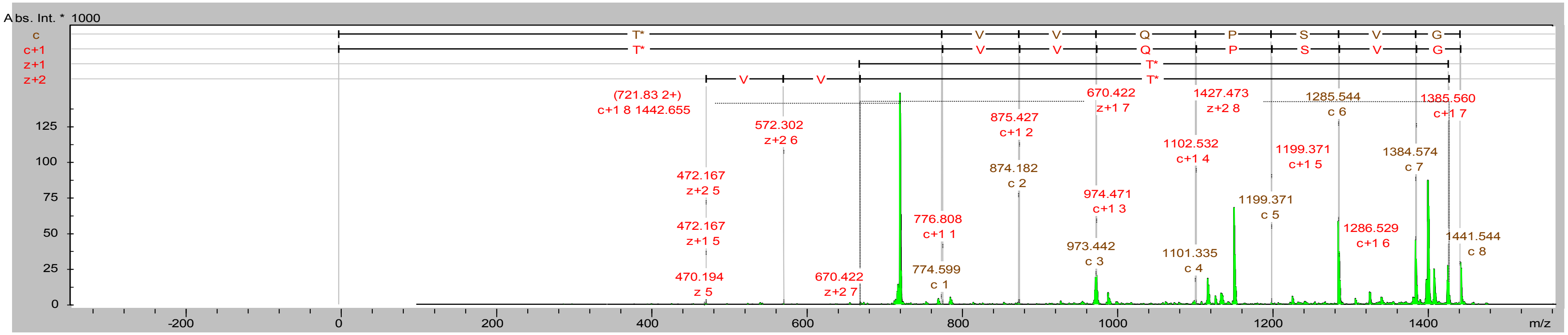

<u>T</u>VVQPSVG

	T	V	V	Q	P	S	V	G	Thr	Val	Val	Gln	Pro	Ser	Val	Gly
Ion	1	2	3	4	5	6	7	8	1	2	3	4	5	6	7	8
c	T*	V	V	Q	P	S	V	G	775.309	874.378	973.446	1101.505	1198.557	1285.589	1384.658	1441.679
c+1	T*	V	V	Q	P	S	V	G	776.317	875.385	974.454	1102.512	1199.565	1286.597	1385.666	1442.687
z	T*	V	V	Q	P	S	V	G	59.013	158.081	245.113	342.166	470.225	569.293	668.361	1425.637
z+1	T*	V	V	Q	P	S	V	G	60.021	159.089	246.121	343.174	471.232	570.301	669.369	1426.644
z+2	T*	V	V	Q	P	S	V	G	61.028	160.097	247.129	344.182	472.240	571.309	670.377	1427.652
	8	7	6	5	4	3	2	1	Gly	Val	Ser	Pro	Gln	Val	Val	Thr

Biotools-Score: 533

Annotation is not accurate – T is not modified

known O-glycosylation site

Alpha-2-HS-glycoprotein

341**TVVQPSVG**348

Fraction 13

721.78++ → Pep [M+H]+ 786.39+ [17.8-17.9 min] ETD

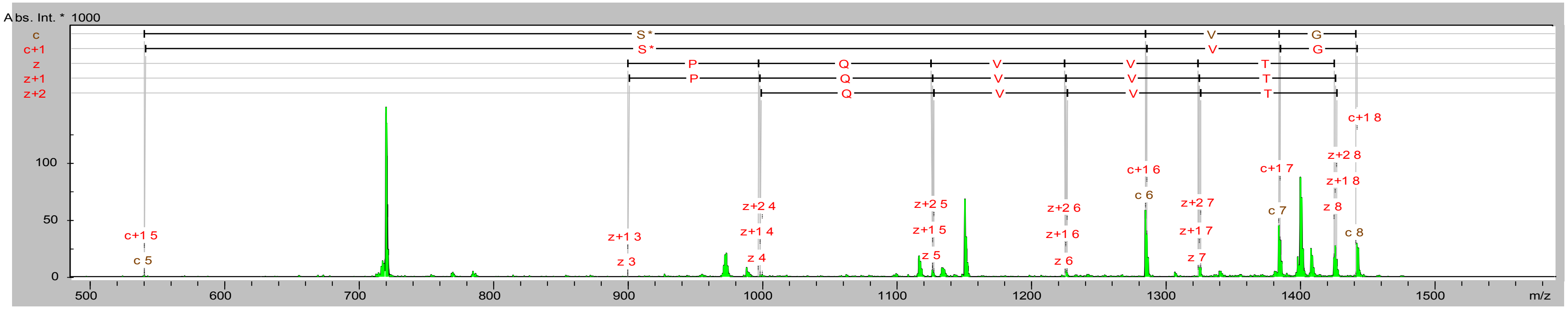

TVVQPSVG

	T	V	V	Q	P	S	V	G	Thr	Val	Val	Gln	Pro	Ser	Val	Gly
Ion	1	2	3	4	5	6	7	8	1	2	3	4	5	6	7	8
c	T	V	V	Q	P	S*	V	G	119.082	218.150	317.218	445.277	542.330	1285.589	1384.658	1441.679
c+1	T	V	V	Q	P	S*	V	G	120.089	219.158	318.226	446.285	543.337	1286.597	1385.666	1442.687
z	T	V	V	Q	P	S*	V	G	59.013	158.081	901.341	998.394	1126.452	1225.521	1324.589	1425.637
z+1	T	V	V	Q	P	S*	V	G	60.021	159.089	902.349	999.401	1127.460	1226.528	1325.597	1426.644
z+2	T	V	V	Q	P	S*	V	G	61.028	160.097	903.356	1000.409	1128.468	1227.536	1326.605	1427.652
	8	7	6	5	4	3	2	1	Gly	Val	Ser	Pro	Gln	Val	Val	Thr

The presence of the highlighted fragment ions indicates the occupancy of S_{346}

Biotools-Score: 187

known O-glycosylation site

Alpha-2-HS-glycoprotein

$_{341}$**TVVQPSVG**$_{348}$

Fraction 13

620.26++ → Pep [M+H]+ 583.33+ [20.25-20.37 min] CID-MS Precursor

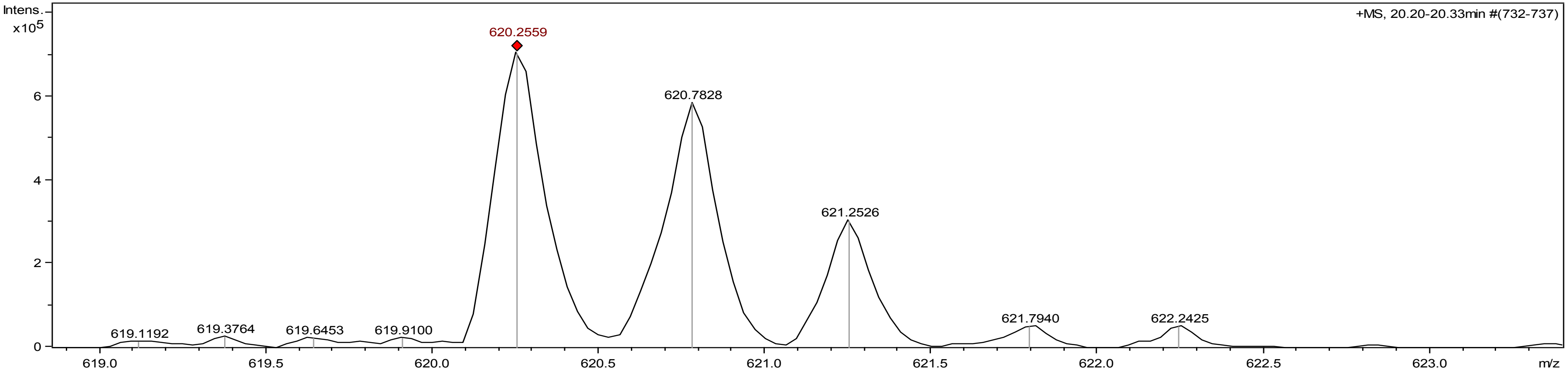

Fraction 13

620.26++ → Pep [M+H]+ 583.33+ [20.25-20.37 min] CID-MS2

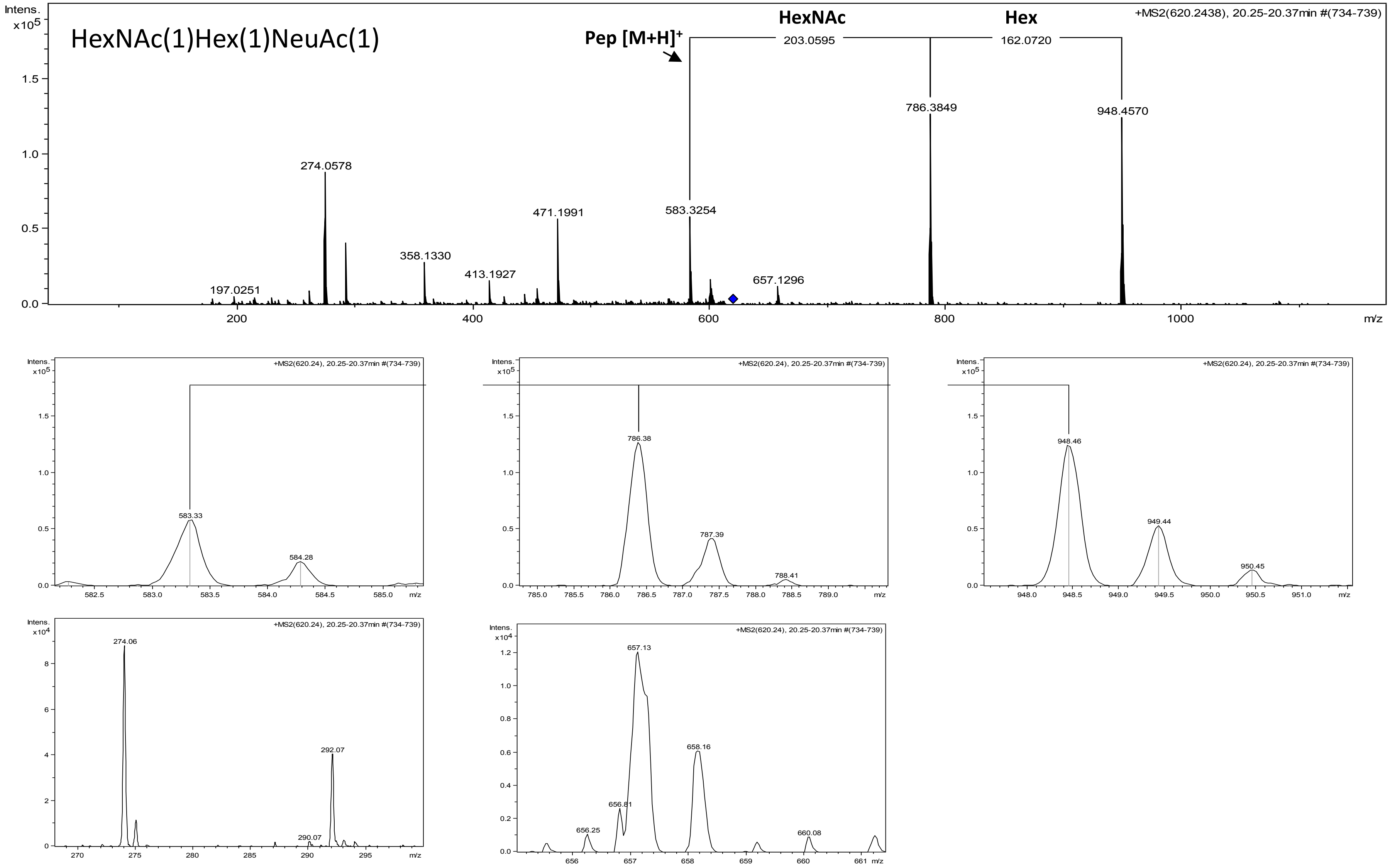

Fraction 13

620.26++ → Pep [M+H]+ 583.33+ [20.25-20.37 min] CID-MS2

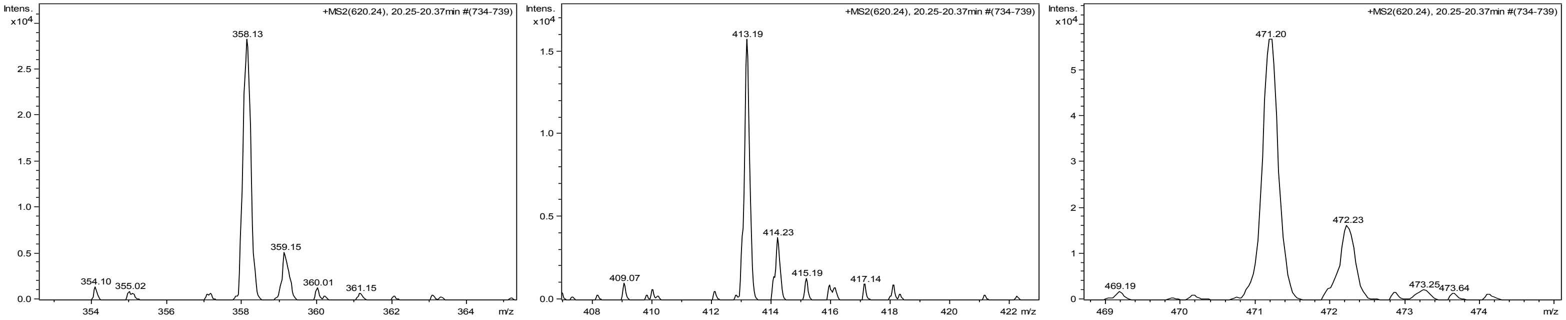

Fraction 13

620.26++ → Pep [M+H]+ 583.33+ [20.25-20.37 min]   CID-MS3   Manual DeNovo

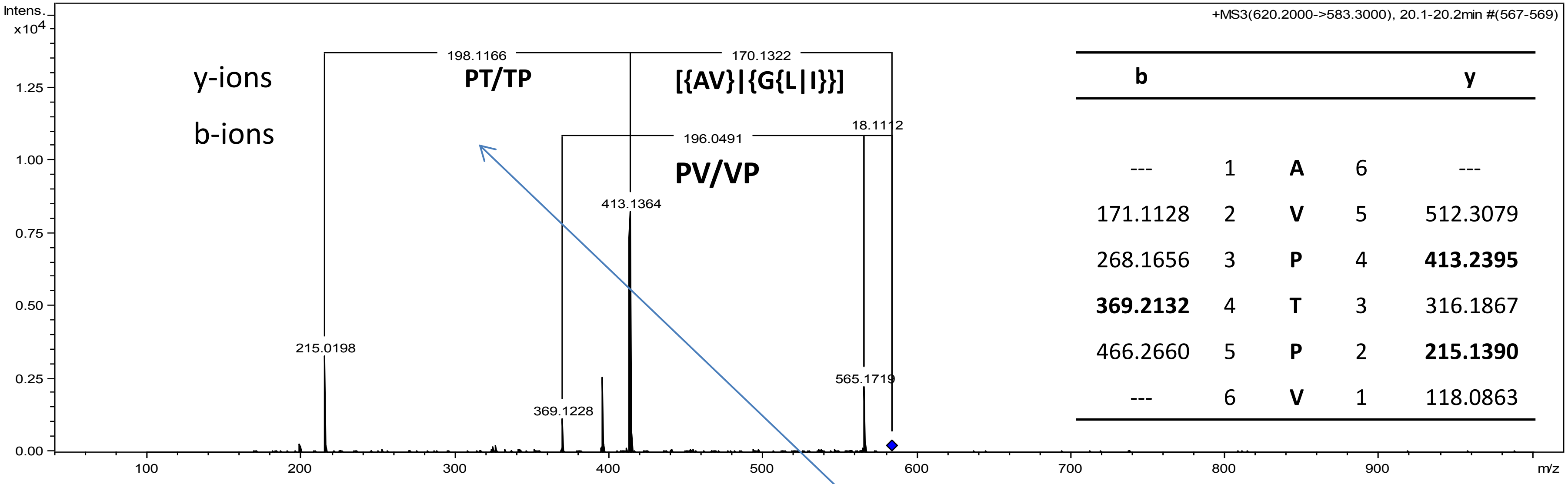

b				y
---	1	**A**	6	---
171.1128	2	**V**	5	512.3079
268.1656	3	**P**	4	**413.2395**
**369.2132**	4	**T**	3	316.1867
466.2660	5	**P**	2	**215.1390**
---	6	**V**	1	118.0863

267**AVPTPV**272   HexNAc(1)Hex(1)NeuAc(1)

Alpha-2-HS-glycoprotein

It should be PT not TP,
due to Proline-Gap
→ hindered Pro-Xxx

Fraction 13

620.26++ → Pep [M+H]+ 583.33+ [20.25-20.37 min] CID-MS3

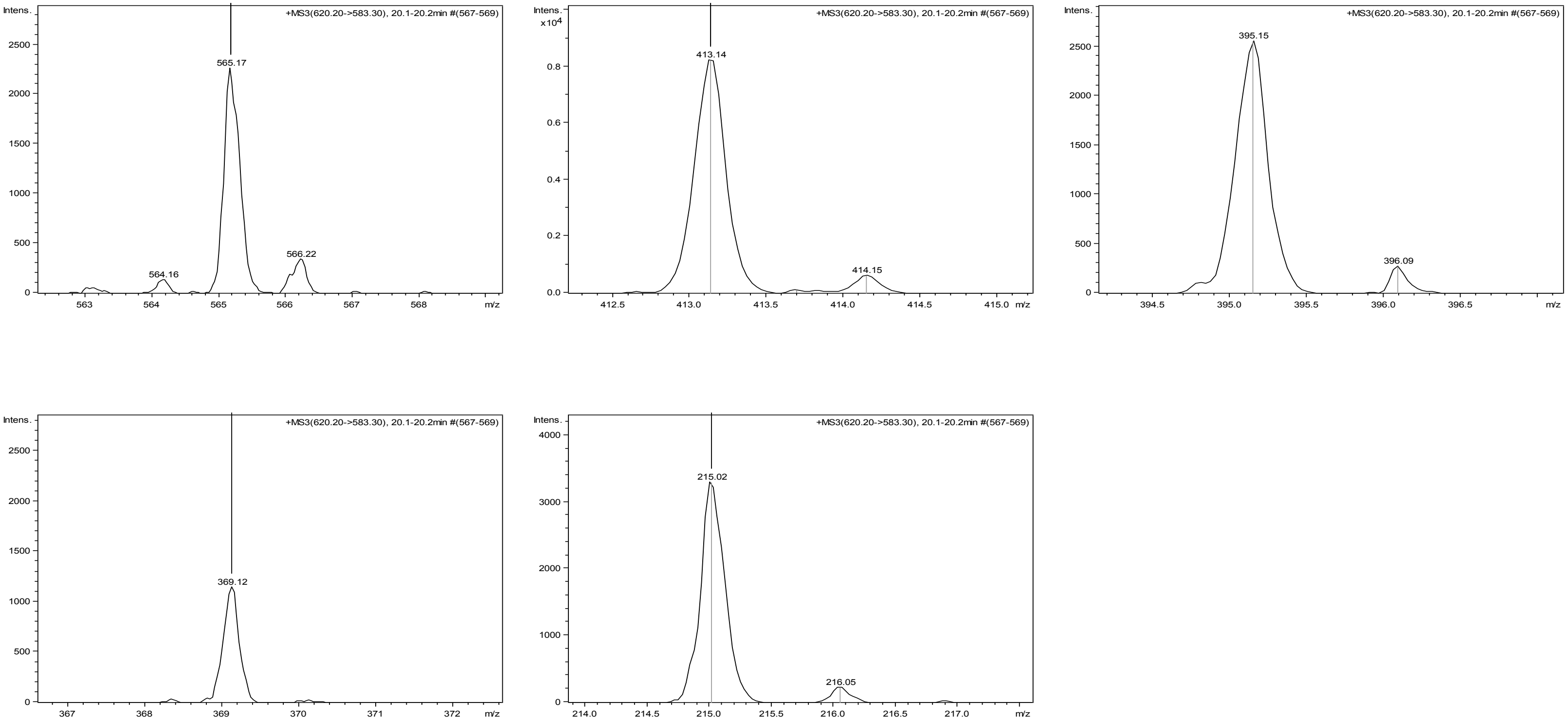

Fraction 13

620.26++ → Pep [M+H]+ 583.33+ [20.25-20.37 min]

CID-MS3 MASCOT Search

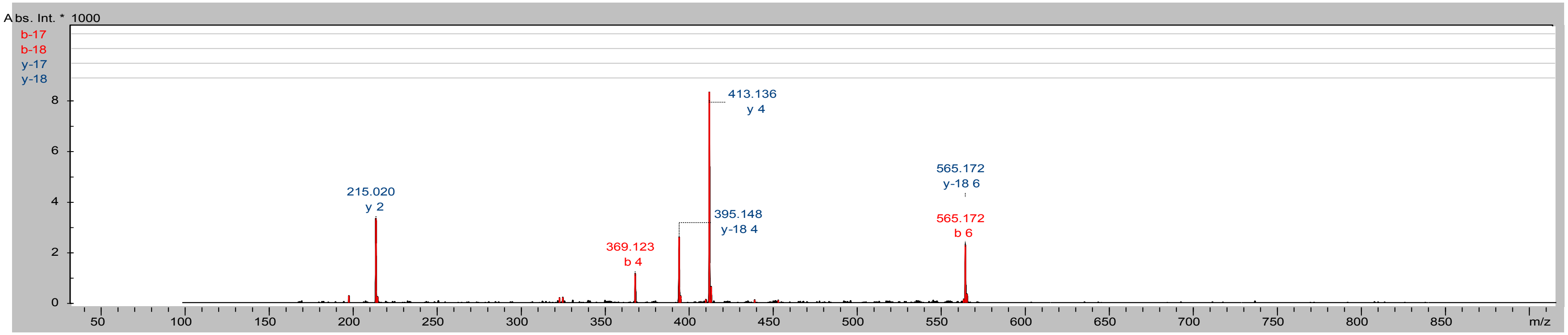

	A	V	P	T	P	V	Ala	Val	Pro	Thr	Pro	Val
Ion	1	2	3	4	5	6	1	2	3	4	5	6
b	A	V	P	T	P	V	72.044	171.113	268.166	369.213	466.266	565.334
b-17	A	V	P	T	P	V	-	-	-	-	-	-
b-18	A	V	P	T	P	V	-	-	-	351.203	448.255	547.324
y	A	V	P	T	P	V	118.086	215.139	316.187	413.239	512.308	583.345
y-17	A	V	P	T	P	V	-	-	-	-	-	-
y-18	A	V	P	T	P	V	-	-	298.176	395.229	494.297	565.334
	6	5	4	3	2	1	Val	Pro	Thr	Pro	Val	Ala

known O-glycosylation site

Alpha-2-HS-glycoprotein

$_{267}$**AVPTPV**$_{272}$

Fraction 13

620.26++ → Pep [M+H]+ 583.33+ [20.25-20.37 min]

CID-MS3 MASCOT Search

prot_hit_nur	prot_acc	prot_desc	prot_score	prot_mass	prot_matche	pep_query	pep_rank	pep_isbold	pep_exp_mz	pep_exp_mr	pep_exp_z	pep_calc_mr	pep_delta	pep_miss	pep_score	pep_expect	pep_res_bef	pep_seq
1	CT165_HUMA	Uncharacteri	17	24161	1	1	1	1	583.26	582.2527	1	582.3377	-0.085	0	19.58	85	A	GIPTVP
2	TLX1_HUMAI	T-cell leuker	16	34685	1	1	1	0	583.26	582.2527	1	582.3377	-0.085	0	19.58	85	T	GLPTVP
3	IBP7_HUMAI	Insulin-like g	16	30138	1	1	1	0	583.26	582.2527	1	582.3377	-0.085	0	19.58	85	I	GIPTPV
4	FETUA_HUM	Alpha-2-HS-g	15	40098	1	1	1	0	583.26	582.2527	1	582.3377	-0.085	0	19.58	85	E	AVPTPV
5	FRS2_HUMAI	Fibroblast gr	15	57892	1	1	1	0	583.26	582.2527	1	582.3377	-0.085	0	19.58	85	V	LGPTPV
6	F111A_HUMA	Protein FAM	15	71007	1	1	1	0	583.26	582.2527	1	582.3377	-0.085	0	19.58	85	N	GITPVP
7	LARP1_HUMA	La-related pr	14	123833	1	1	1	0	583.26	582.2527	1	582.3377	-0.085	0	19.58	85	R	AVTPVP
8	POL_HTL1A	Gag-Pro-Pol	13	163893	1	1	1	0	583.26	582.2527	1	582.3377	-0.085	0	19.58	85	D	AVPTVP
9	BMPR2_HUM	Bone morph	13	116440	1	1	1	0	583.26	582.2527	1	582.3377	-0.085	0	19.58	85	S	IGPTPV
10	CEP35_HUMA	Centrosome	12	352312	1	1	1	0	583.26	582.2527	1	582.3377	-0.085	0	19.58	85	K	AVTPPV

Biotools-Score: 1

MASCOT-Score: 20

known O-glycosylation site

Alpha-2-HS-glycoprotein

$_{267}$**AVPTPV**$_{272}$

Fraction 13

620.26++ → Pep [M+H]+ 583.33+ [20.25-20.37 min] ETD

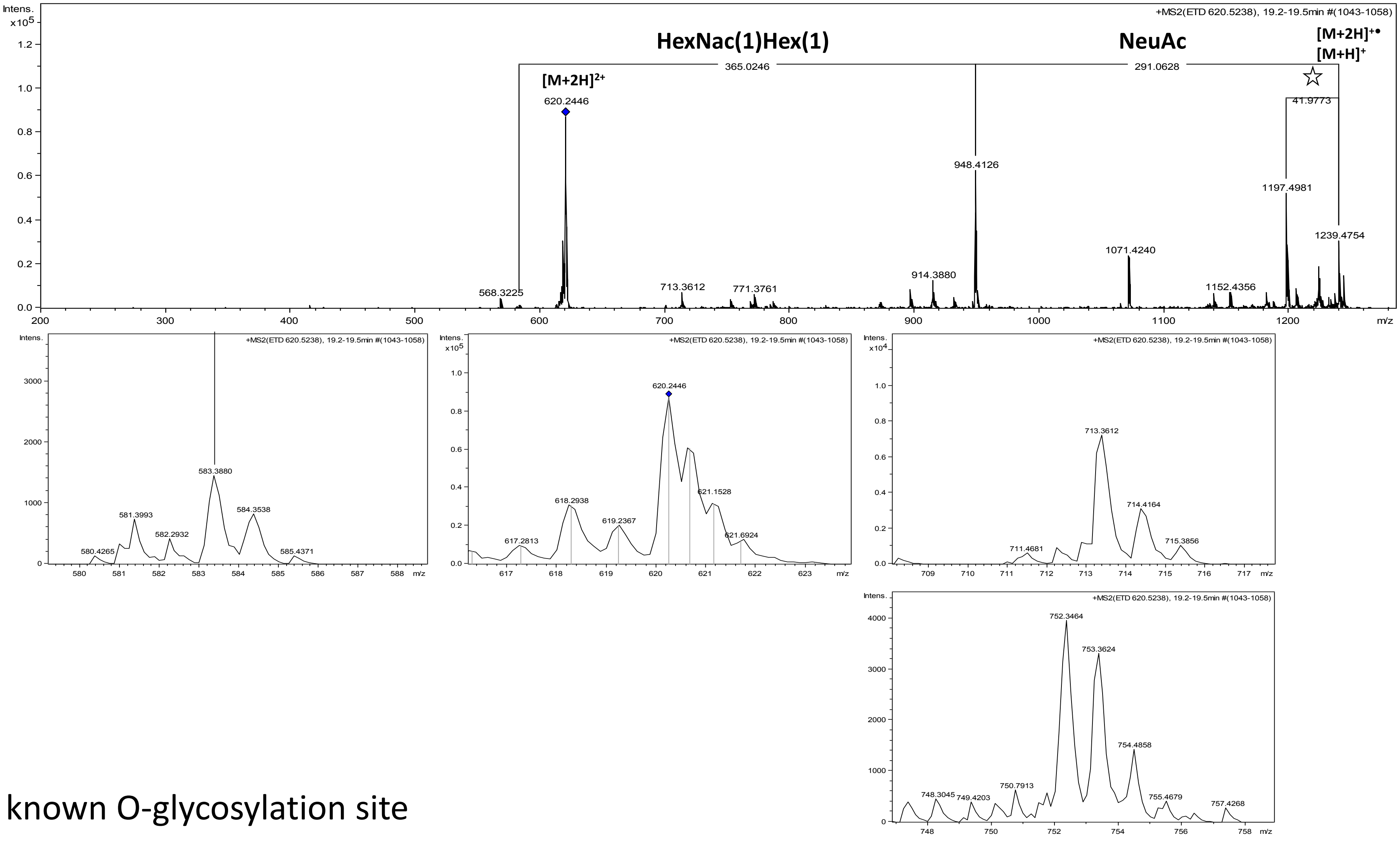

known O-glycosylation site

Alpha-2-HS-glycoprotein

$_{267}$**AVPTPV**$_{272}$

Fraction 13

620.26++ → Pep [M+H]+ 583.33+ [20.25-20.37 min]

ETD

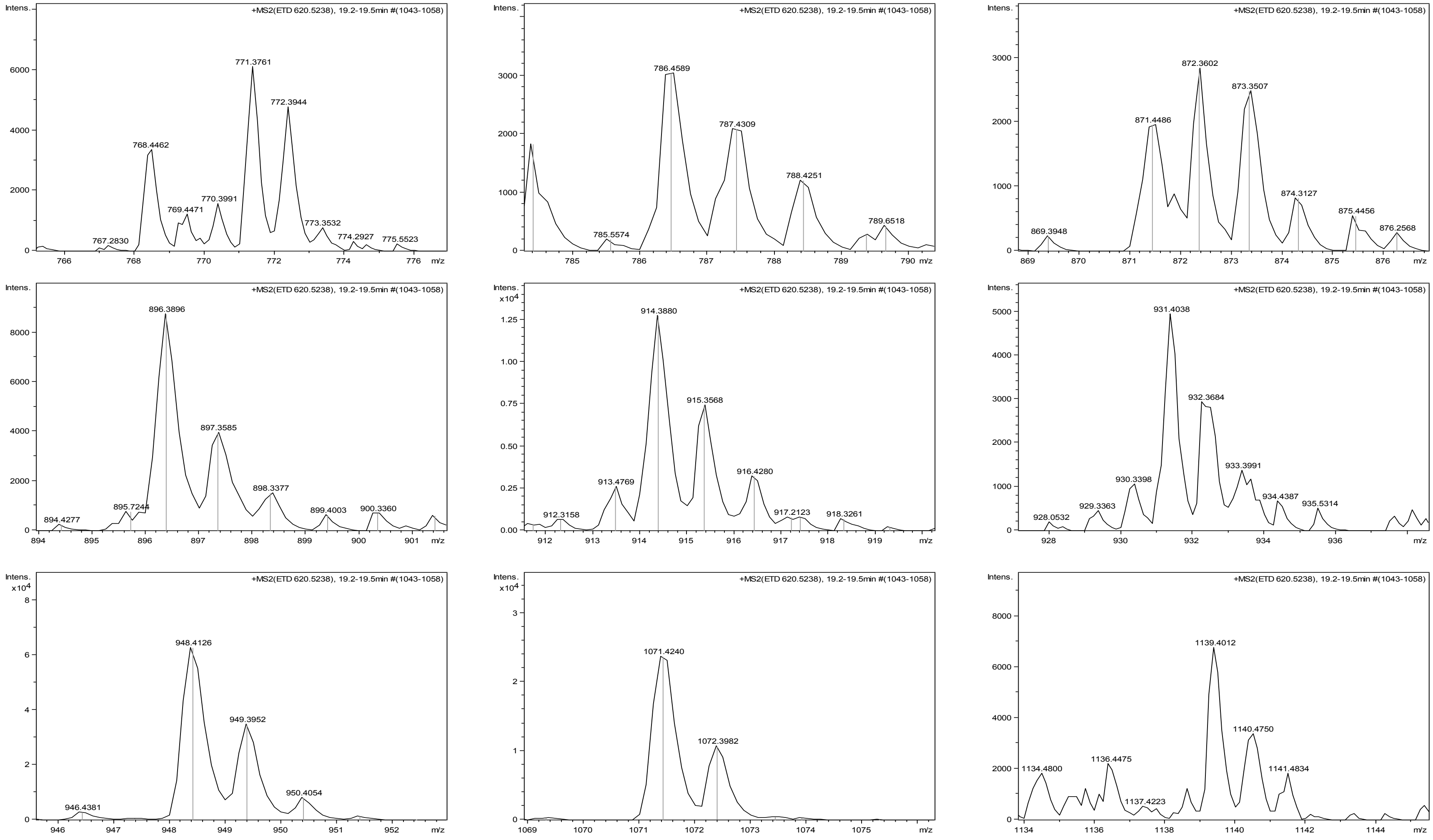

known O-glycosylation site

Alpha-2-HS-glycoprotein

$_{267}$**AVPTPV**$_{272}$

Fraction 13

620.26++ → Pep [M+H]+ 583.33+ [20.25-20.37 min] ETD

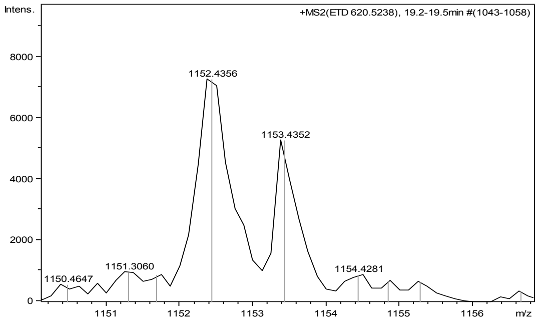

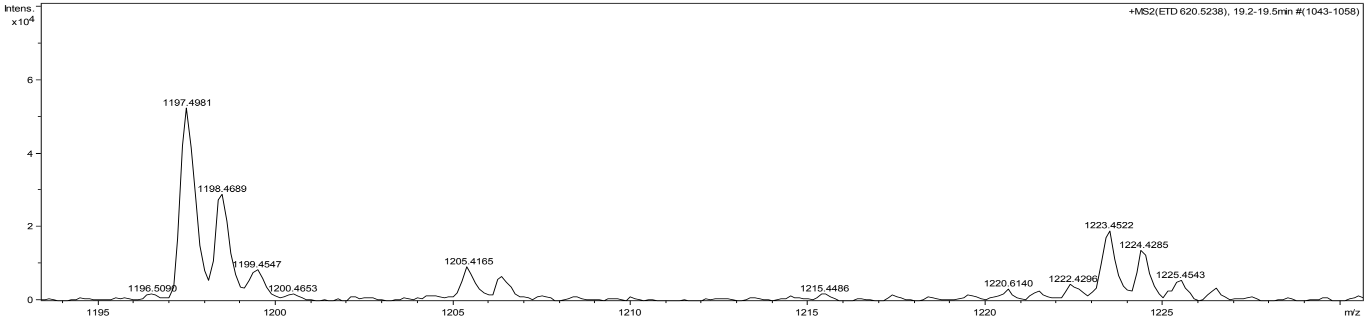

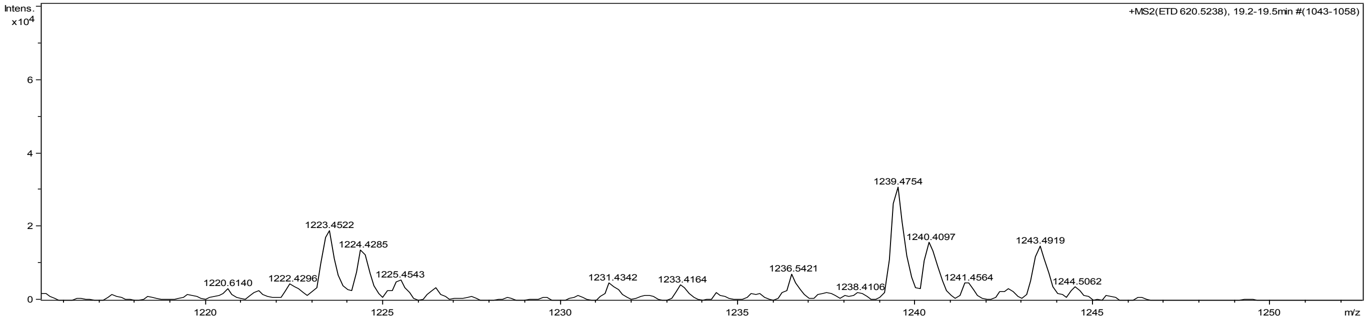

known O-glycosylation site

Alpha-2-HS-glycoprotein

$_{267}$**AVPTPV**$_{272}$

Fraction 13

620.26++ → Pep [M+H]+ 583.33+ [20.25-20.37 min] ETD

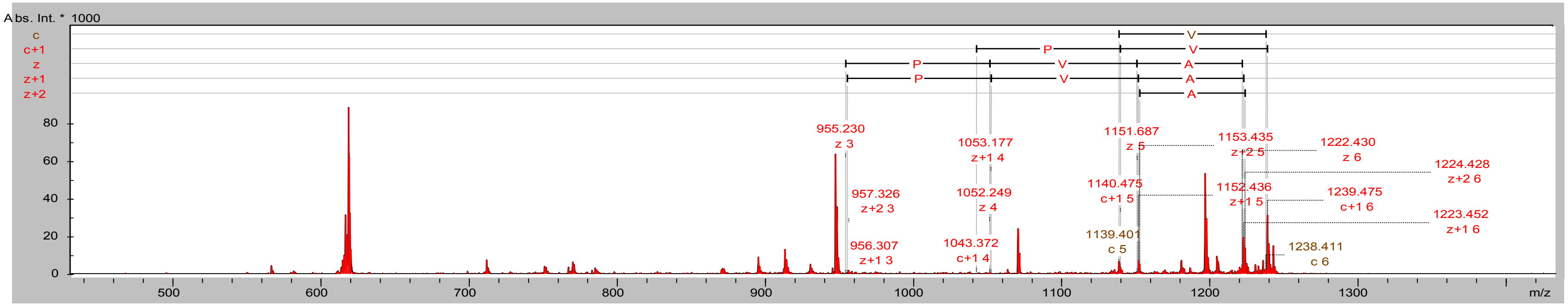

	A	V	P	T	P	V	Ala	Val	Pro	Thr	Pro	Val
Ion	1	2	3	4	5	6	1	2	3	4	5	6
c	A	V	P	T*	P	V	89.071	188.139	285.192	1042.467	1139.520	1238.589
c+1	A	V	P	T*	P	V	90.079	189.147	286.200	1043.475	1140.528	1239.596
z	A	V	P	T*	P	V	101.060	198.112	955.388	1052.441	1151.509	1222.546
z+1	A	V	P	T*	P	V	102.068	199.120	956.396	1053.448	1152.517	1223.554
z+2	A	V	P	T*	P	V	103.075	200.128	957.403	1054.456	1153.525	1224.562
	6	5	4	3	2	1	Val	Pro	Thr	Pro	Val	Ala

Biotools-Score: 44

known O-glycosylation site

Alpha-2-HS-glycoprotein

267**AVPTPV**272

Fraction 13

608.72++ → Pep [M+H]+ 560.29+ [18.6-18.8 min]

CID-MS Precursor

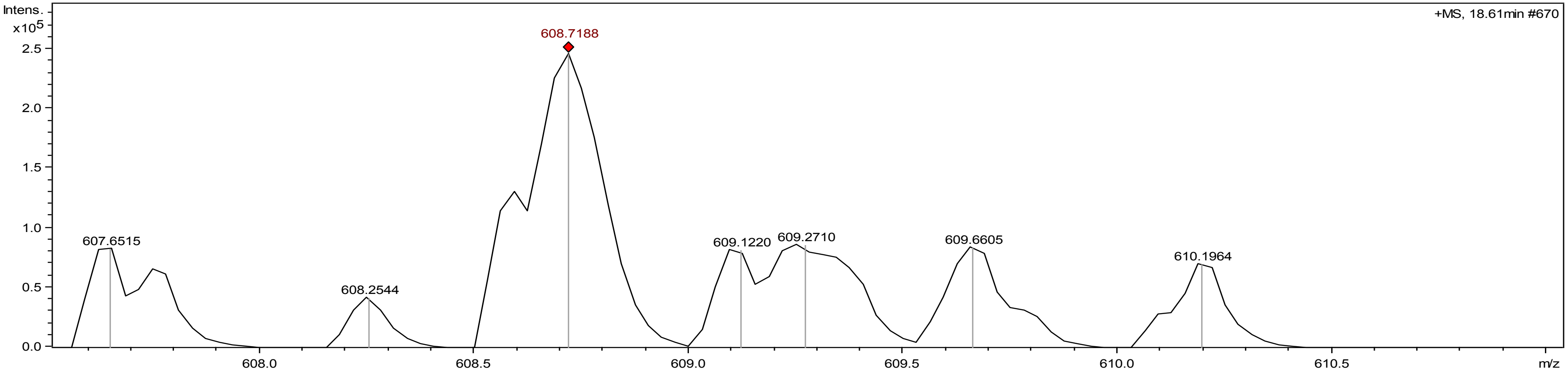

ETD spectrum not available

Fraction 13

608.72++ → Pep [M+H]+ 560.29+ [18.6-18.8 min] CID-MS2

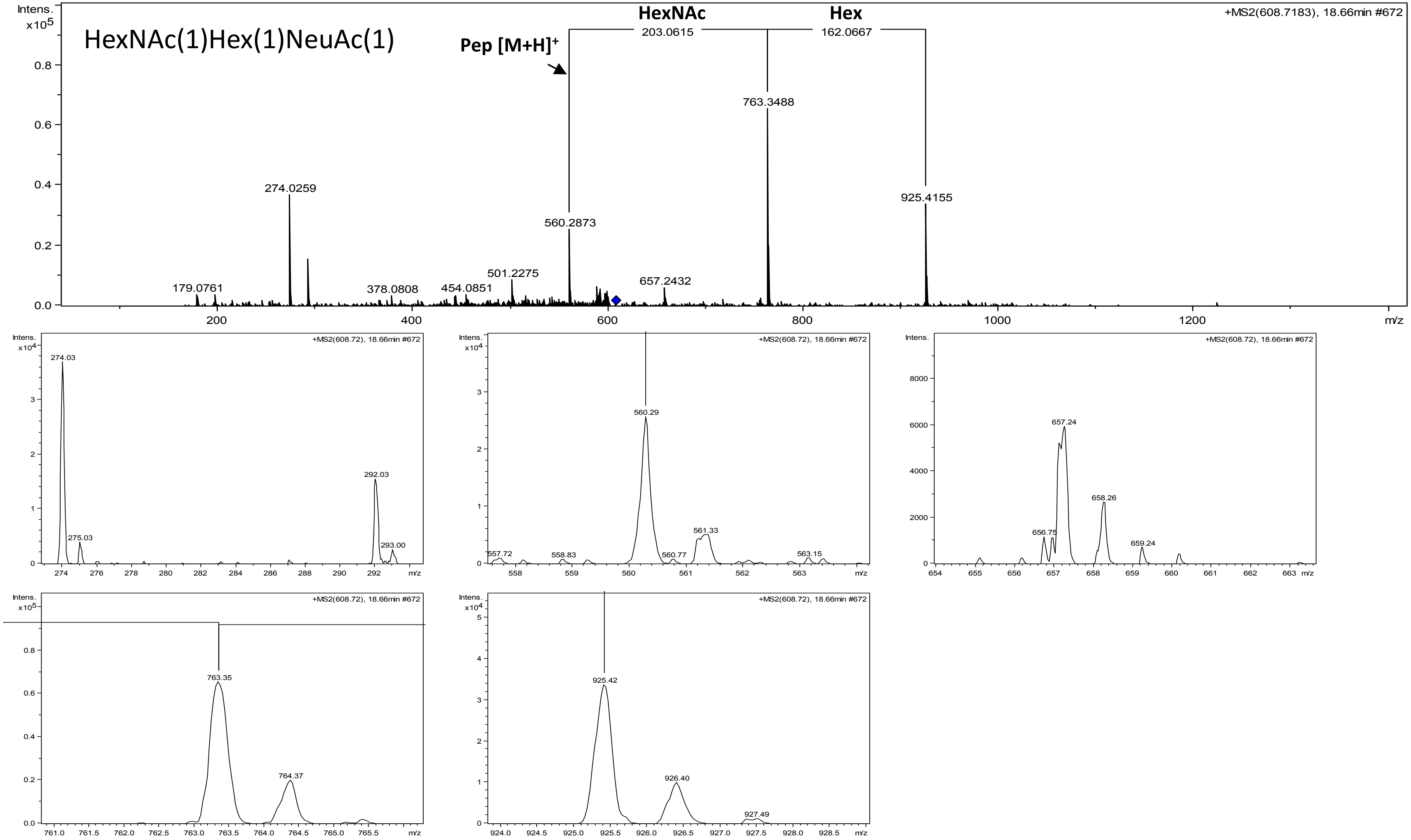

ETD spectrum not available

Fraction 13

608.72++ → Pep [M+H]+ 560.29+ [18.6-18.8 min]

CID-MS3 Manual DeNovo

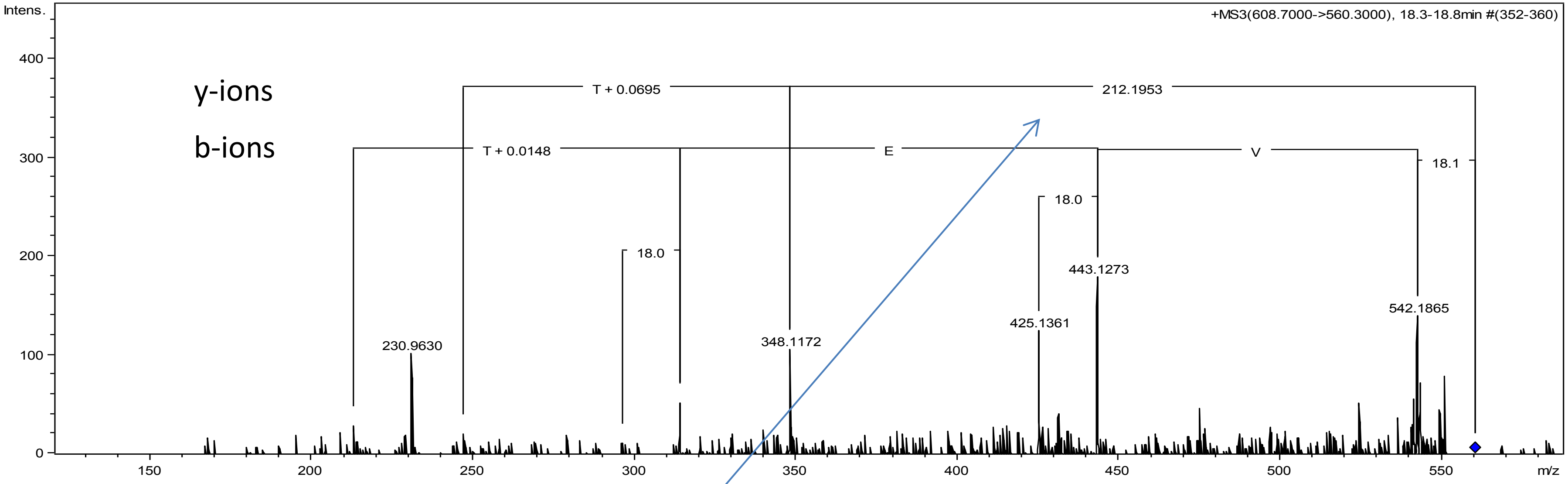

Possible sequence variants:
DPTEV
PDTEV
V(L/I)TEV
(L/I)VTEV

It should be PD not DP,
due to Proline-Gap
→ hindered Pro-Xxx

Fraction 13

608.72++ → Pep [M+H]+ 560.29+ [18.6-18.8 min]

CID-MS3 Manual DeNovo

Download GenPept Graphics

Ig J-chain, partial [Homo sapiens]

Sequence ID: gb|AAA58902.1| **Length:** 137 **Number of Matches:** 1

Range 1: 73 to 77 GenPept Graphics Next Match Previous Match

Score	Expect	Identities	Positives	Gaps
19.3 bits(38)	130	5/5(100%)	5/5(100%)	0/5(0%)

```
Query  1   DPTEV  5
           DPTEV
Sbjct  73  DPTEV  77
```

BLASTp

← Lowest E-Value among the sequence variants, most likely sequence

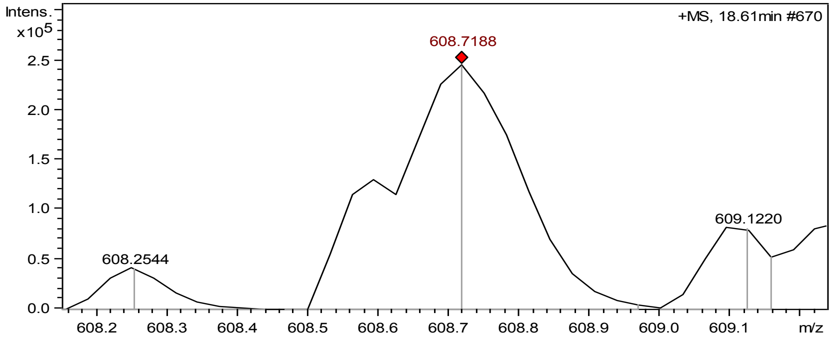

Possible sequence variants:

DPTEV
PDTEV
V(L/I)TEV
(L/I)VTEV

DPT(HexNAc(1)Hex(1)NeuAc(1))EV= 608.7419++
VLT(HexNAc(1)Hex(1)NeuAc(1))EV= 608.7783++

DP/PD = 212.0797 Da
(L/I)V/V(L/I) = 212.1525 Da

Due to mass accuracy DPTEV/PDTEV more likely to be the correct sequence

unknown O-glycosylation site

Immunoglobulin J chain precursor

$_{95}$**DPTEV**$_{99}$

Fraction 13

608.72++ → Pep [M+H]+ 560.29+ [18.6-18.8 min] CID-MS3 RapiDenovo DeNovo

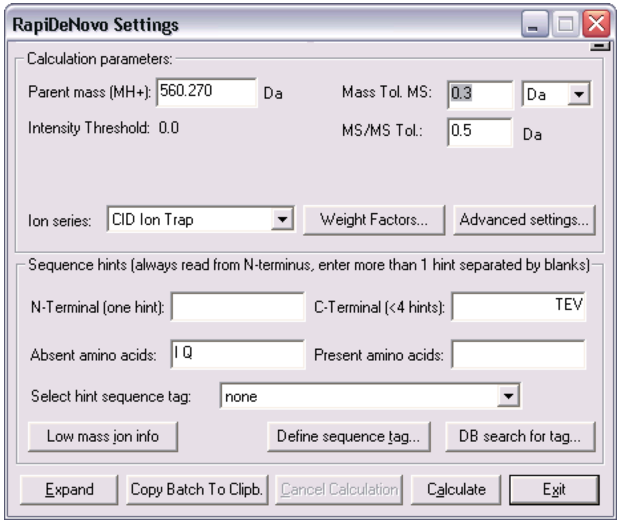

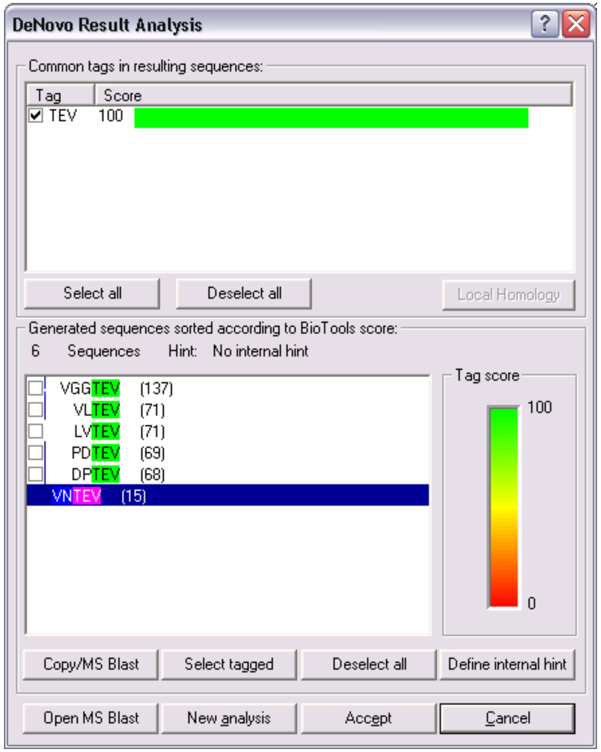

MASCOT Search did not give any reasonable result

unknown O-glycosylation site

Immunoglobulin J chain precursor $_{95}$**DPTEV**$_{99}$

Fraction 13

853.81++ → Pep [M+H]+ 1050.54+ [25.5-25.9 min]   CID-MS Precursor

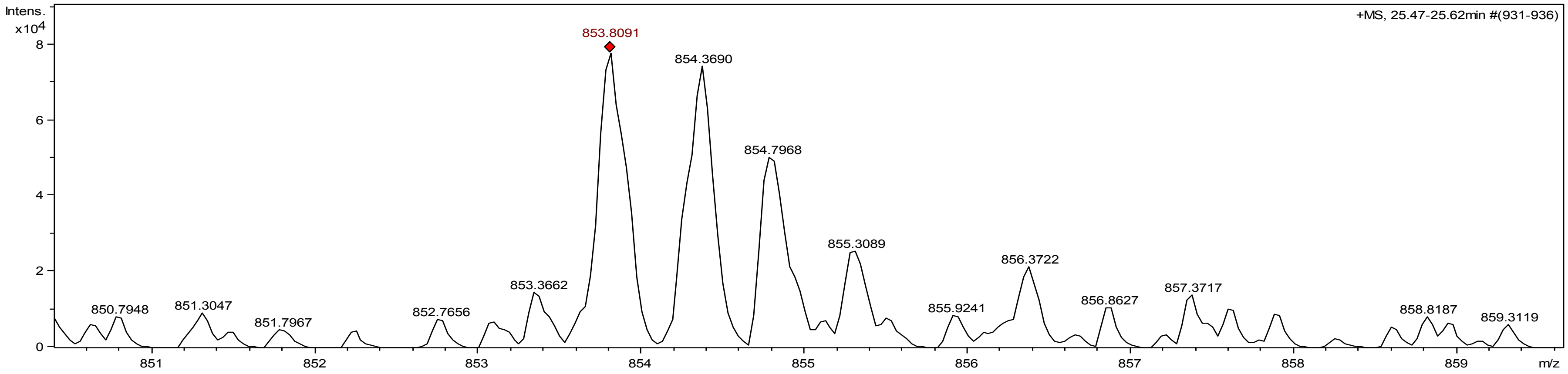

Fraction 13

853.81++ → Pep [M+H]+ 1050.54+ [25.5-25.9 min] CID-MS2

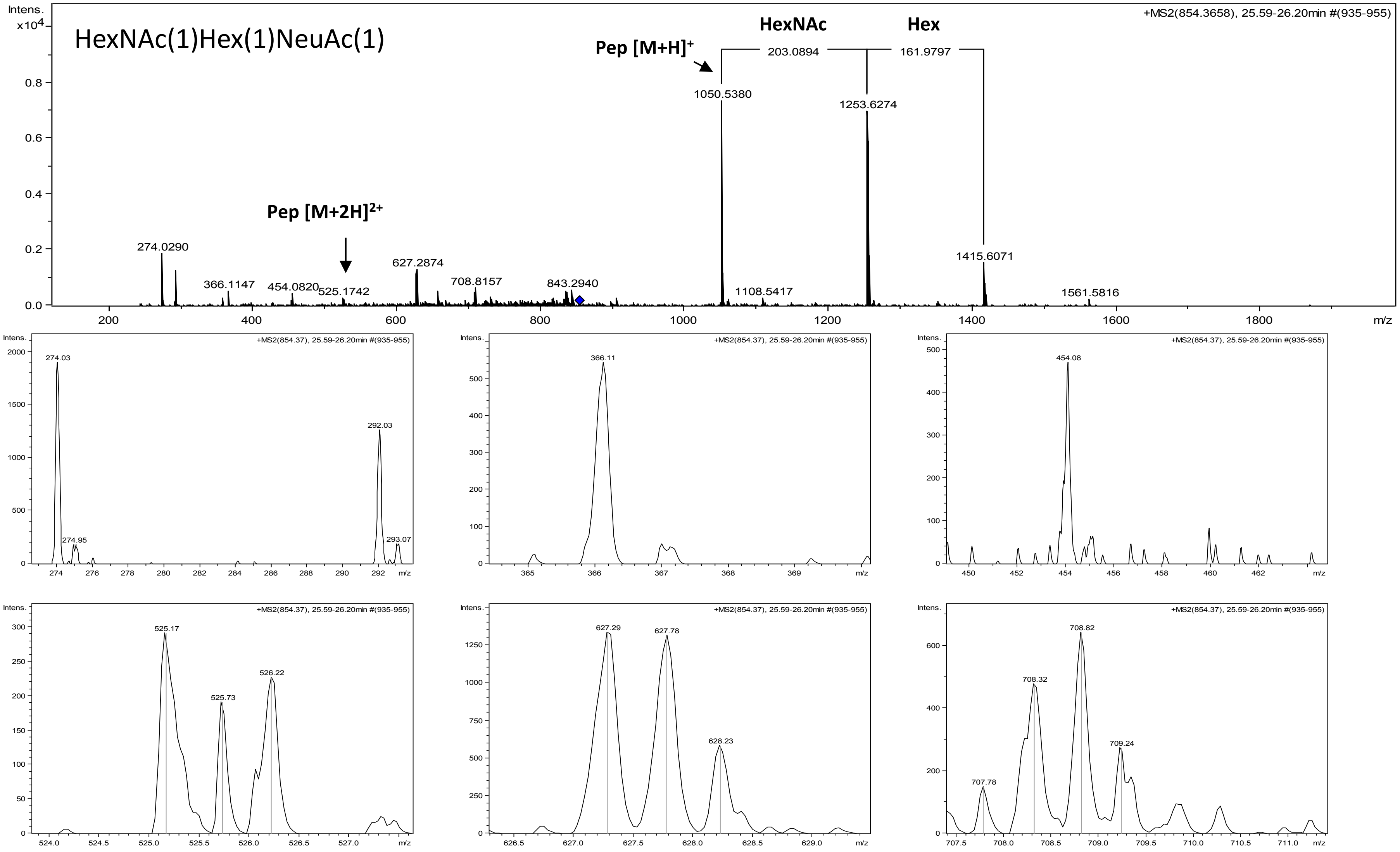

Fraction 13

853.81++ → Pep [M+H]+ 1050.54+ [25.5-25.9 min] CID-MS2

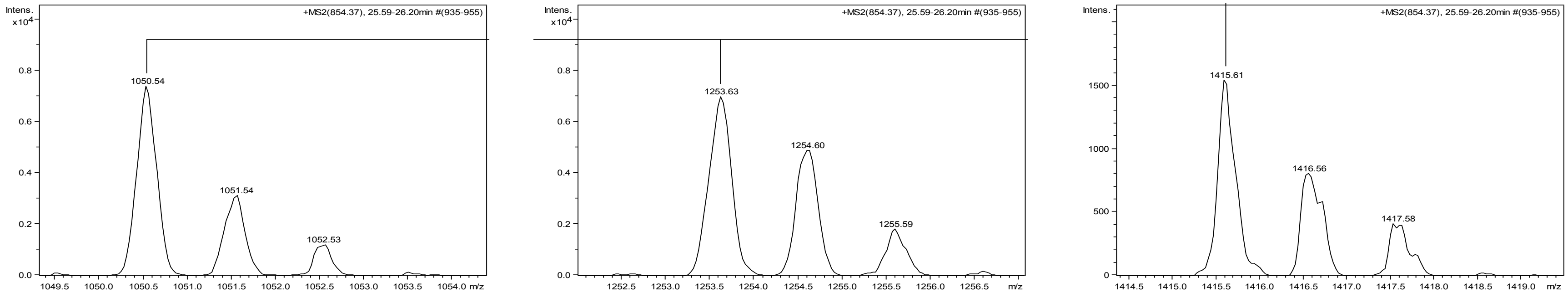

Fraction 13

853.81++ → Pep [M+H]+ 1050.54+ [25.5-25.9 min] CID-MS3 Manual DeNovo

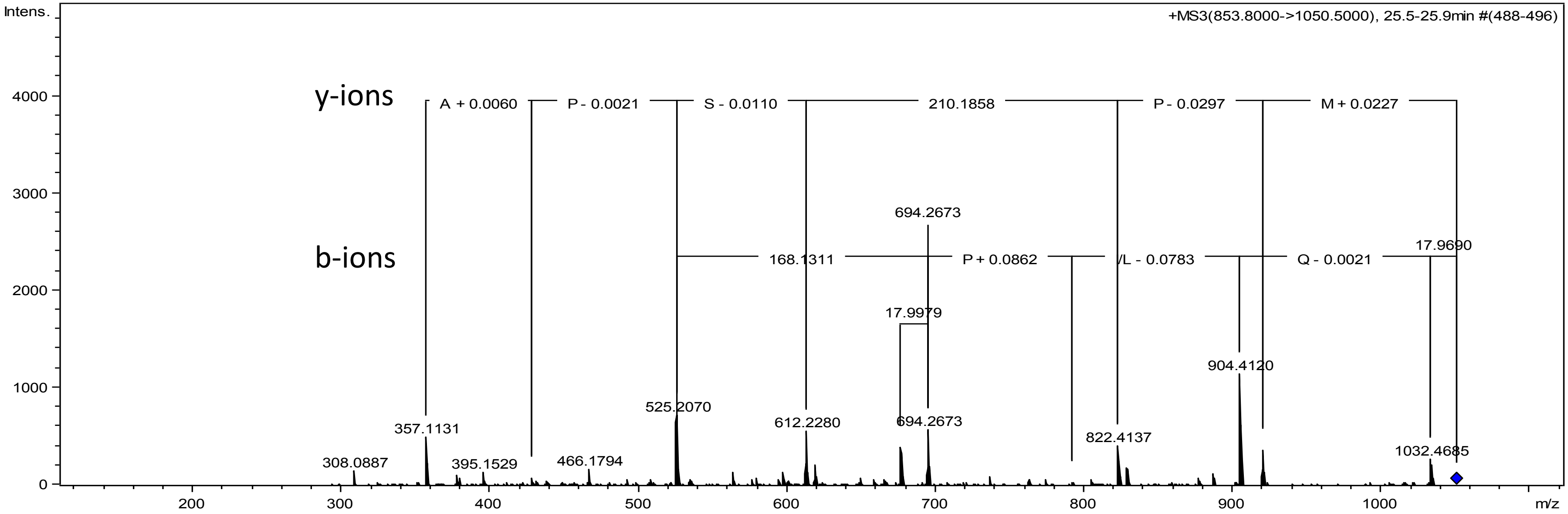

	M	P	P	I	S	P	A	P	I	Q	Met	Pro	Pro	Ile	Ser	Pro	Ala	Pro	Ile	Gln
Ion	1	2	3	4	5	6	7	8	9	10	1	2	3	4	5	6	7	8	9	10
b	M	P	P	I	S	P	A	P	I	Q	132.048	229.101	326.153	439.237	526.269	623.322	694.359	791.412	904.496	1032.555
b-17	M	P	P	I	S	P	A	P	I	Q	-	-	-	-	-	-	-	-	-	1015.528
b-18	M	P	P	I	S	P	A	P	I	Q	-	-	-	-	508.259	605.312	676.349	773.401	886.486	1014.544
y	M	P	P	I	S	P	A	P	I	Q	147.076	260.160	357.213	428.250	525.303	612.335	725.419	822.472	919.525	1050.565
y-17	M	P	P	I	S	P	A	P	I	Q	130.050	243.134	340.187	411.224	508.277	595.309	708.393	805.445	902.498	1033.539
y-18	M	P	P	I	S	P	A	P	I	Q	-	-	-	-	-	594.325	707.409	804.461	901.514	1032.555
	10	9	8	7	6	5	4	3	2	1	Gln	Ile	Pro	Ala	Pro	Ser	Ile	Pro	Pro	Met

known O-glycosylation site

Kininogen-1

$_{573}$MPPISPAPIQ$_{582}$

Fraction 13

853.81++ → Pep [M+H]+ 1050.54+ [25.5-25.9 min]

CID-MS3 MASCOT Search

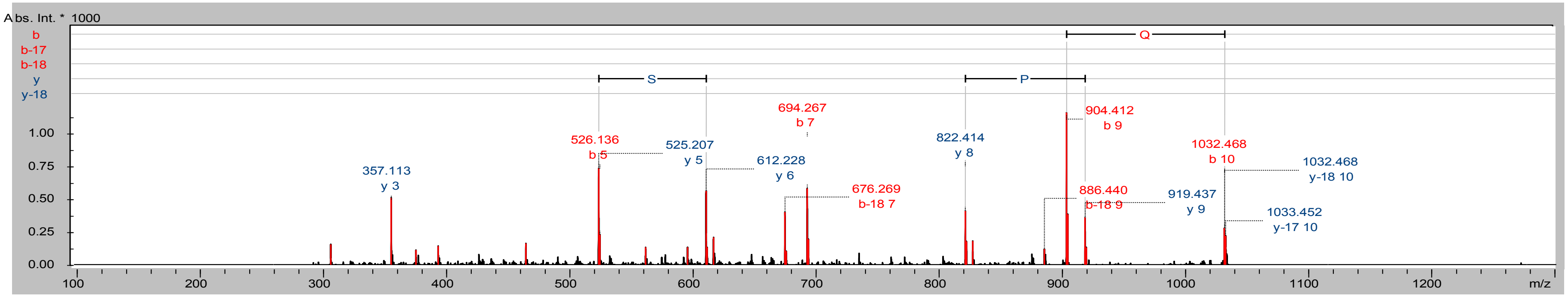

	M	P	P	I	S	P	A	P	I	Q	Met	Pro	Pro	Ile	Ser	Pro	Ala	Pro	Ile	Gln
Ion	1	2	3	4	5	6	7	8	9	10	1	2	3	4	5	6	7	8	9	10
b	M	P	P	I	S	P	A	P	I	Q	132.048	229.101	326.153	439.237	526.269	623.322	694.359	791.412	904.496	1032.555
b-17	M	P	P	I	S	P	A	P	I	Q	-	-	-	-	-	-	-	-	-	1015.528
b-18	M	P	P	I	S	P	A	P	I	Q	-	-	-	-	508.259	605.312	676.349	773.401	886.486	1014.544
y	M	P	P	I	S	P	A	P	I	Q	147.076	260.160	357.213	428.250	525.303	612.335	725.419	822.472	919.525	1050.565
y-17	M	P	P	I	S	P	A	P	I	Q	130.050	243.134	340.187	411.224	508.277	595.309	708.393	805.445	902.498	1033.539
y-18	M	P	P	I	S	P	A	P	I	Q	-	-	-	-	-	594.325	707.409	804.461	901.514	1032.555
	10	9	8	7	6	5	4	3	2	1	Gln	Ile	Pro	Ala	Pro	Ser	Ile	Pro	Pro	Met

known O-glycosylation site

Kininogen-1

$_{573}$MPPI**S**PAPIQ$_{582}$

Fraction 13

853.81++ → Pep [M+H]+ 1050.54+ [25.5-25.9 min]

CID-MS3 MASCOT Search

prot_hit_nur	prot_acc	prot_desc	prot_score	prot_mass	prot_matche	pep_query	pep_rank	pep_isbold	pep_exp_mz	pep_exp_mr	pep_exp_z	pep_calc_mr	pep_delta	pep_miss	pep_score	pep_expect	pep_res_bef	pep_seq
1	KNG1_HUMA	Kininogen-1	34	72996	1	1	1	1	1050.47	1049.4627	1	1049.5579	-0.0952	0	40.4	1.5	M	MPPISPAPIQ
2	PHLPP_HUM	PH domain le	21	186951	1	1	2	0	1050.47	1049.4627	1	1049.6206	-0.1579	0	28.8	21	R	RRRGAPQPI
3	Y0125_HUMA	Very putativ	21	7876	1	1	4	0	1050.47	1049.4627	1	1049.5982	-0.1354	0	22.68	86	M	SRAPGGPAKI
4	TCOF_HUMA	Treacle prote	20	152241	1	1	3	0	1050.47	1049.4627	1	1049.4885	-0.0258	0	26.27	38	P	QVKPASTMG
5	MTG8_HUMA	Protein CBFA	16	68323	1	1	6	0	1050.47	1049.4627	1	1048.5288	0.9339	0	22.09	99	E	QLLLDASTTS
6	STIP1_HUMA	Stress-induc	16	63227	1	1	8	0	1050.47	1049.4627	1	1049.5254	-0.0627	0	21.41	1.20E+02	L	KHEANNPQL
7	TARB1_HUM	Probable me	14	183840	1	1	7	0	1050.47	1049.4627	1	1049.5506	-0.0878	0	21.74	1.10E+02	L	QLDSLHAGPL
8	MAP2_HUMA	Microtubule-	14	199873	1	1	8	0	1050.47	1049.4627	1	1049.6485	-0.1858	0	21.41	1.20E+02	S	LVVPGIDLPK
9	HRX_HUMAN	Zinc finger p	13	436044	1	1	5	0	1050.47	1049.4627	1	1049.5757	-0.113	0	22.48	90	E	DAEPLAPPIK
10	GOGA4_HUM	Golgin subfa	13	261892	1	1	8	0	1050.47	1049.4627	1	1049.6233	-0.1606	0	21.41	1.20E+02	S	KSHLVQPKL

Biotools-Score: 11

MASCOT-Score: 40

known O-glycosylation site

Kininogen-1

$_{573}$MPPI**S**PAPIQ$_{582}$

Fraction 13

853.81++ → Pep [M+H]+ 1050.54+ [25.5-25.9 min] ETD

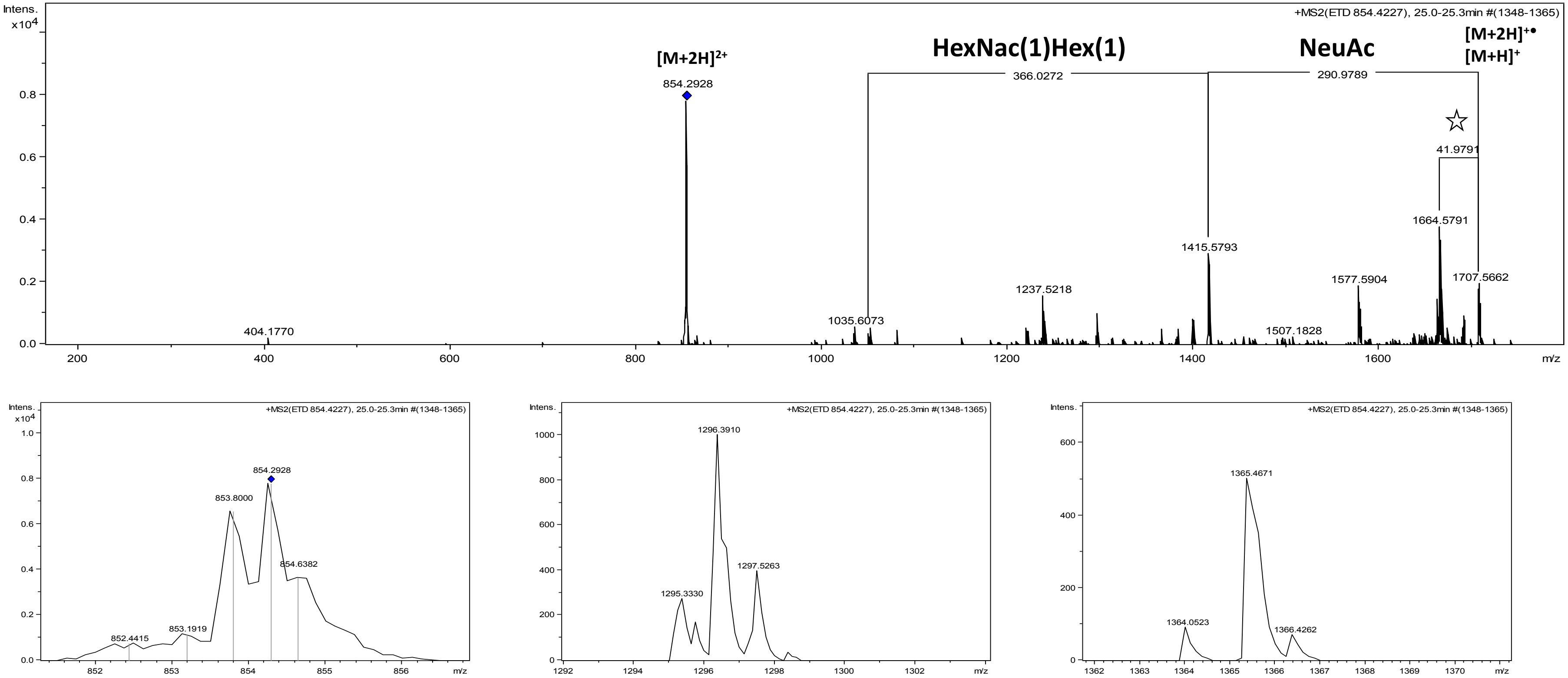

known O-glycosylation site

Kininogen-1

$_{573}$MPPI**S**PAPIQ$_{582}$

Fraction 13

853.81++ → Pep [M+H]+ 1050.54+ [25.5-25.9 min] ETD

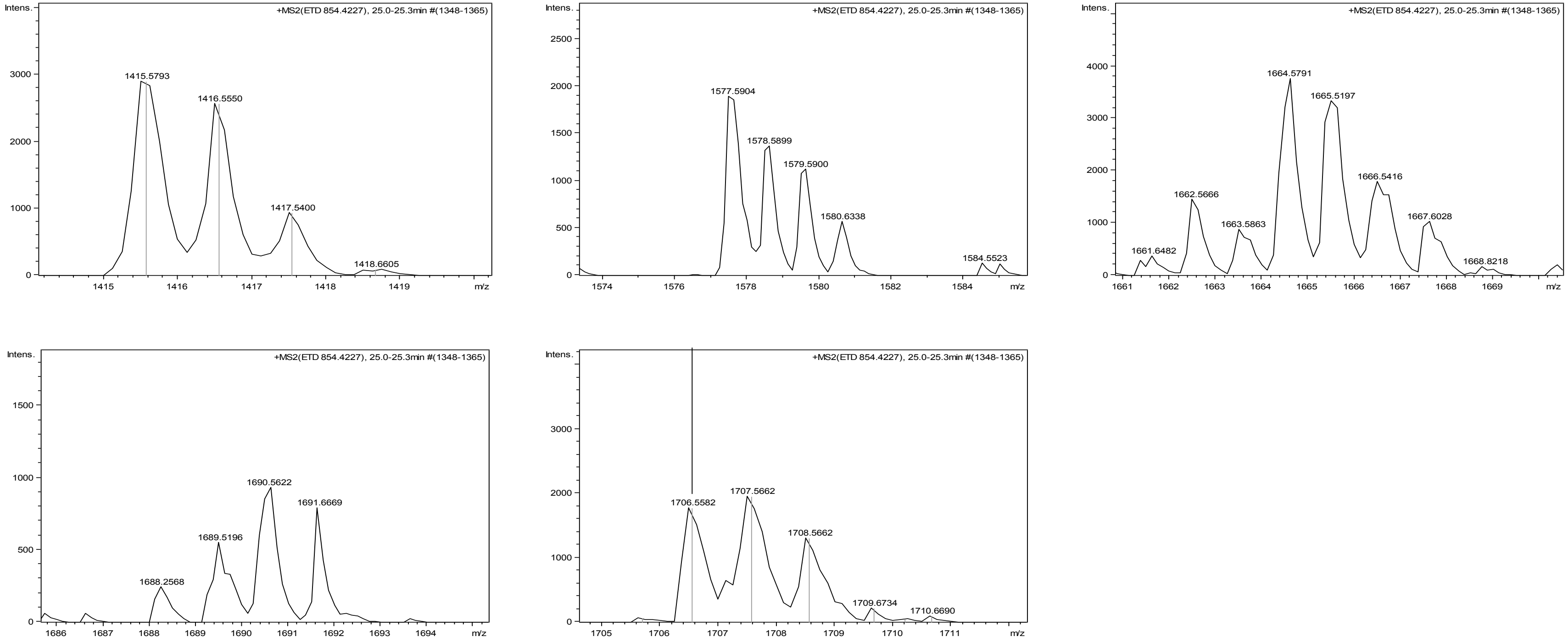

known O-glycosylation site

Kininogen-1

$_{573}$MPPI**S**PAPIQ$_{582}$

Fraction 13

853.81++ → Pep [M+H]+ 1050.54+ [25.5-25.9 min] ETD

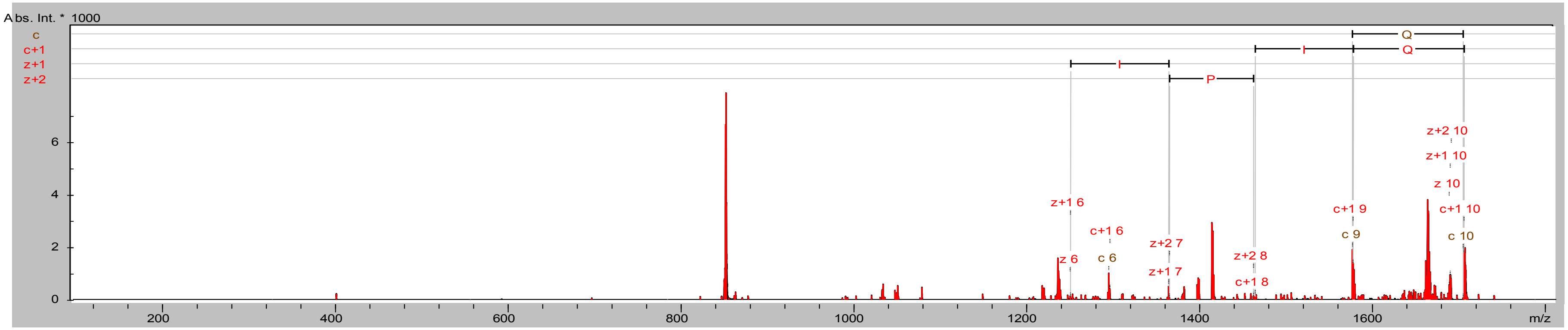

	M	P	P	I	S	P	A	P	I	Q	Met	Pro	Pro	Ile	Ser	Pro	Ala	Pro	Ile	Gln
Ion	1	2	3	4	5	6	7	8	9	10	1	2	3	4	5	6	7	8	9	10
c	M	P	P	I	S*	P	A	P	I	Q	149.074	246.127	343.180	456.264	1199.524	1296.576	1367.613	1464.666	1577.750	1705.809
c+1	M	P	P	I	S*	P	A	P	I	Q	150.082	247.135	344.188	457.272	1200.531	1297.584	1368.621	1465.674	1578.758	1706.817
z	M	P	P	I	S*	P	A	P	I	Q	130.050	243.134	340.187	411.224	508.277	1251.536	1364.620	1461.673	1558.726	1689.766
z+1	M	P	P	I	S*	P	A	P	I	Q	131.058	244.142	341.195	412.232	509.284	1252.544	1365.628	1462.681	1559.734	1690.774
z+2	M	P	P	I	S*	P	A	P	I	Q	132.066	245.150	342.202	413.239	510.292	1253.552	1366.636	1463.689	1560.741	1691.782
	10	9	8	7	6	5	4	3	2	1	Gln	Ile	Pro	Ala	Pro	Ser	Ile	Pro	Pro	Met

Biotools-Score: 22

known O-glycosylation site

Kininogen-1

$_{573}$MPPI**S**PAPIQ$_{582}$

Fraction 13

931.40++ → Pep [M+H]+ 1205.64+ [27.9-28.8 min] CID-MS Precursor

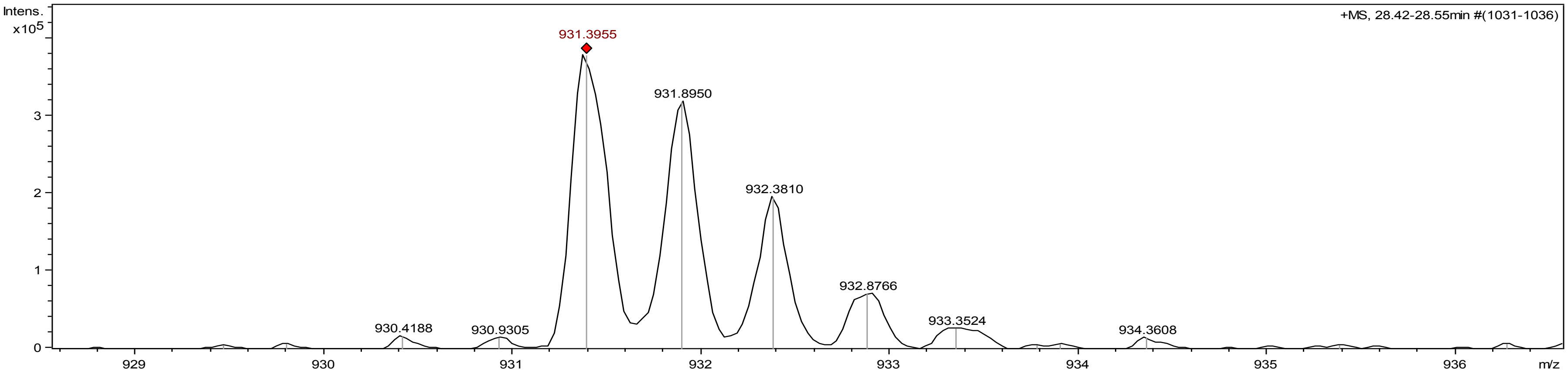

Fraction 13

931.40++ → Pep [M+H]+ 1205.64+ [27.9-28.8 min] CID-MS2

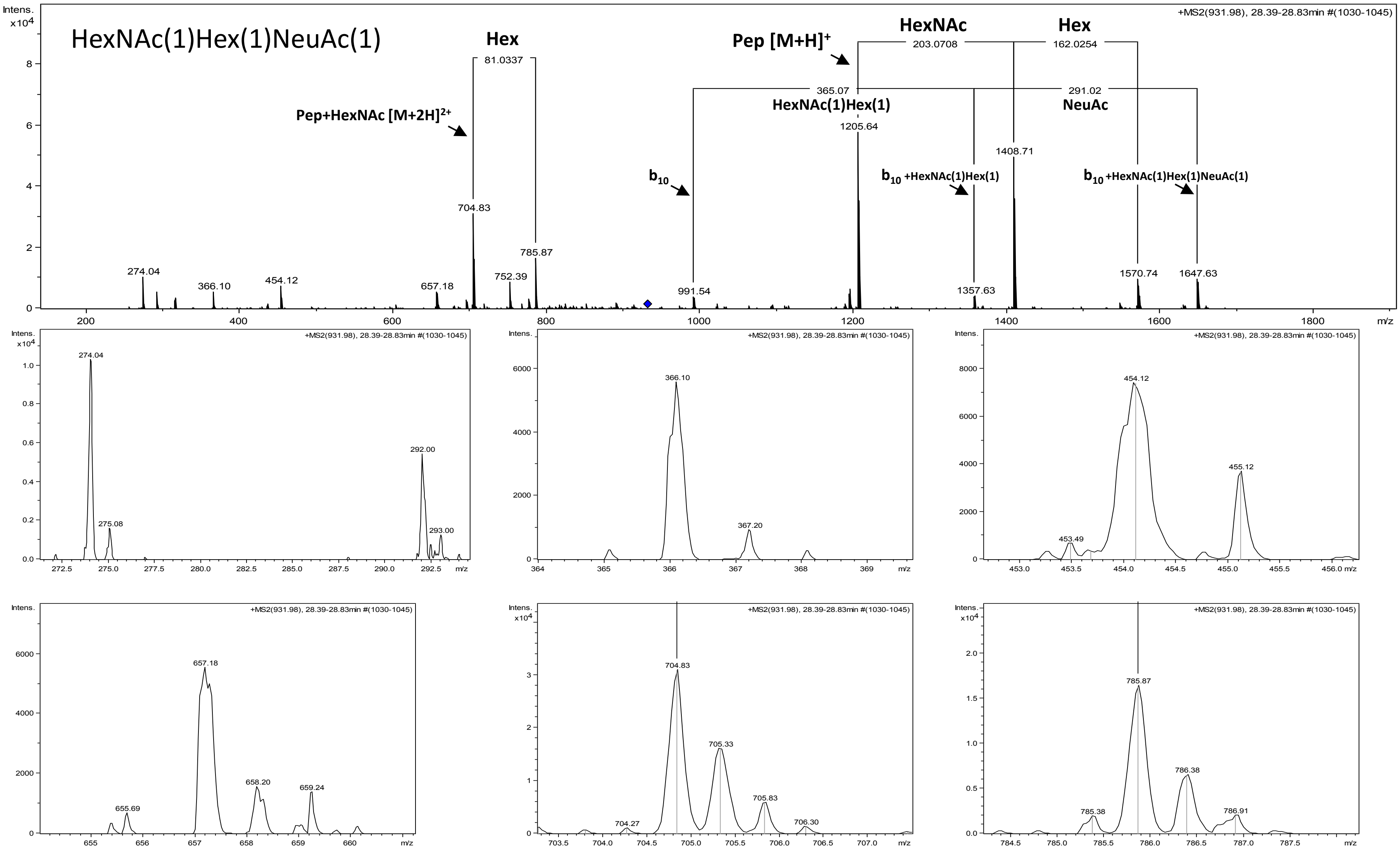

Fraction 13

931.40++ → Pep [M+H]+ 1205.64+ [27.9-28.8 min] CID-MS2

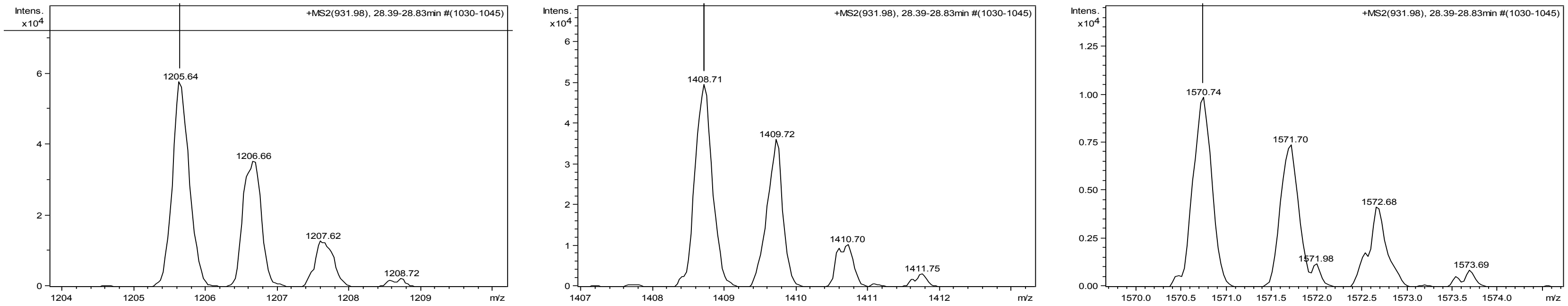

Fraction 13

931.40++ → Pep [M+H]+ 1205.64+ [27.9-28.8 min]

CID-MS3 Manual DeNovo

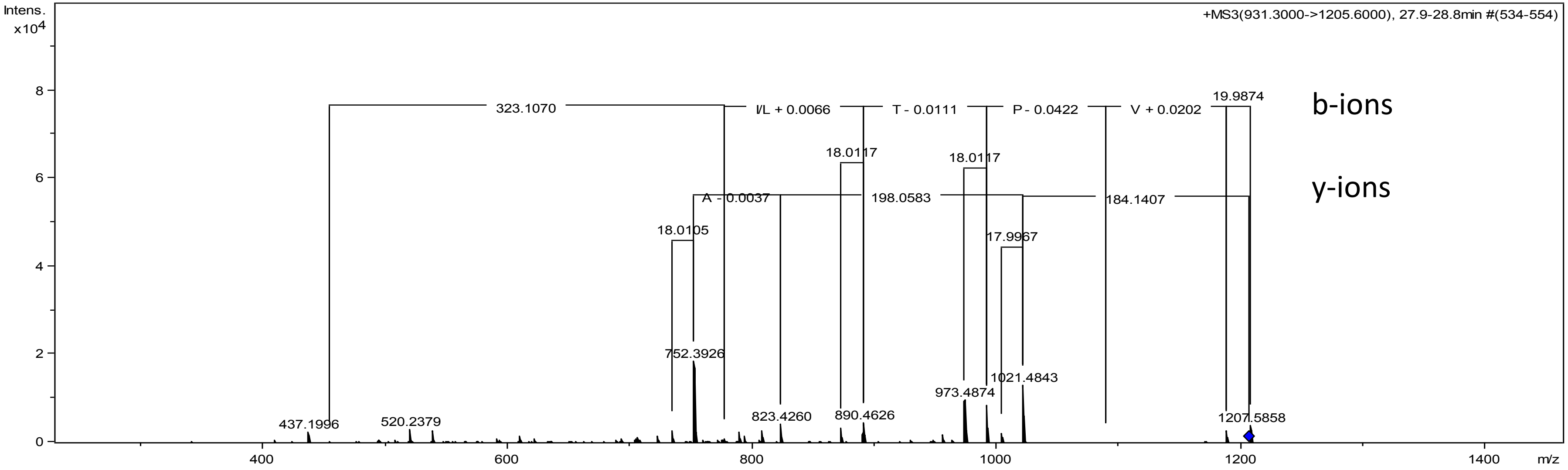

known O-glycosylation site

Plasminogen

$_{362}$LAP**T**APPELTPV$_{373}$

Fraction 13

931.40++ → Pep [M+H]+ 1205.64+ [27.9-28.8 min]

CID-MS3 MASCOT Search

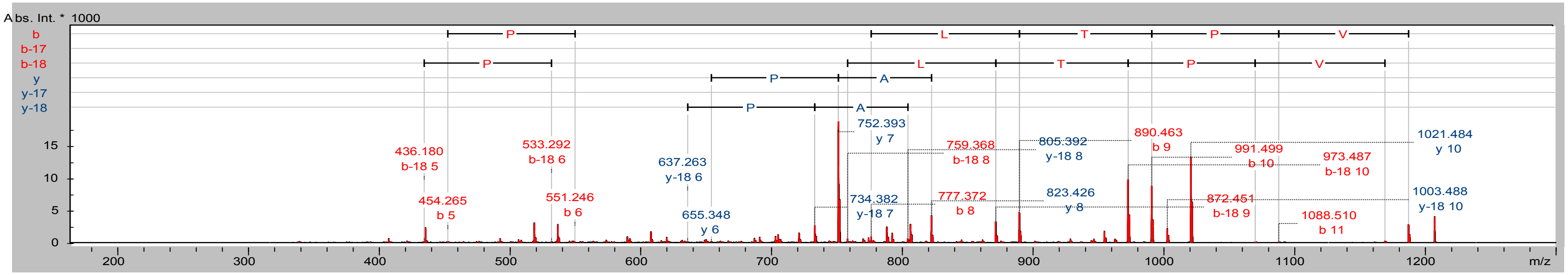

	L	A	P	T	A	P	P	E	L	T	P	V	Leu	Ala	Pro	Thr	Ala	Pro	Pro	Glu	Leu	Thr	Pro	Val
Ion	1	2	3	4	5	6	7	8	9	10	11	12	1	2	3	4	5	6	7	8	9	10	11	12
b	L	A	P	T	A	P	P	E	L	T	P	V	114.091	185.128	282.181	383.229	454.266	551.319	648.372	777.414	890.498	991.546	1088.599	1187.667
b-17	L	A	P	T	A	P	P	E	L	T	P	V	-	-	-	-	-	-	-	-	-	-	-	-
b-18	L	A	P	T	A	P	P	E	L	T	P	V	-	-	-	365.218	436.255	533.308	630.361	759.404	872.488	973.535	1070.588	1169.656
y	L	A	P	T	A	P	P	E	L	T	P	V	118.086	215.139	316.187	429.271	558.313	655.366	752.419	823.456	924.504	1021.556	1092.594	1205.678
y-17	L	A	P	T	A	P	P	E	L	T	P	V	-	-	-	-	-	-	-	-	-	-	-	-
y-18	L	A	P	T	A	P	P	E	L	T	P	V	-	-	298.176	411.260	540.303	637.356	734.408	805.445	906.493	1003.546	1074.583	1187.667
	12	11	10	9	8	7	6	5	4	3	2	1	Val	Pro	Thr	Leu	Glu	Pro	Pro	Ala	Thr	Pro	Ala	Leu

known O-glycosylation site

Plasminogen

$_{362}$LAP**T**APPELTPV$_{373}$

Fraction 13

931.40++ → Pep [M+H]+ 1205.64+ [27.9-28.8 min]

CID-MS3 MASCOT Search

prot_hit_nur	prot_acc	prot_desc	prot_score	prot_mass	prot_matche	pep_query	pep_rank	pep_isbold	pep_exp_mz	pep_exp_mr	pep_exp_z	pep_calc_mr	pep_delta	pep_miss	pep_score	pep_expect	pep_res_bef	pep_seq
1	COPZ1_HUM	Coatomer su	10	20242	1	1	3	1	1205.61	1204.6027	1	1204.7067	-0.104	0	13.02	8.80E+02	I	ALLEGLTVVYI
2	PLMN_HUMA	Plasminoger	10	93247	1	1	1	0	1205.61	1204.6027	1	1204.6703	-0.0676	0	15.76	4.70E+02	Q	LAPTAPPELTI
3	MAP7_HUMA	Ensconsin (M	9	84116	1	1	2	0	1205.61	1204.6027	1	1204.5571	0.0456	0	15.46	5.00E+02	K	PSRLDVTNSE
4	TECT1_HUMA	Tectonic-1 pr	6	64613	1	1	5	0	1205.61	1204.6027	1	1204.6048	-0.0021	0	12.51	9.90E+02	K	TLTRREDTDV
5	STABP_HUM	STAM-bindir	6	48617	1	1	6	0	1205.61	1204.6027	1	1204.6928	-0.0901	0	11.35	1.30E+03	I	PTIDGLRHVV
6	MCAR6_HUN	Mitochondri	6	34573	1	1	10	0	1205.61	1204.6027	1	1204.5434	0.0593	0	10.71	1.50E+03	N	MPSLWASAC
7	G3BP1_HUM	Ras GTPase-a	6	52189	1	1	6	0	1205.61	1204.6027	1	1204.5401	0.0627	0	11.35	1.30E+03	L	KDFFQSYGN\
8	RPGF2_HUM	Rap guanine	5	168168	1	1	4	0	1205.61	1204.6027	1	1204.6816	-0.0788	0	12.95	8.90E+02	R	QTKHIPTALP'
9	WTAP_HUMA	Wilms~ tumc	5	44388	1	1	8	0	1205.61	1204.6027	1	1204.6122	-0.0094	0	11.07	1.40E+03	Q	PSVAQLRSTN
10	PHF14_HUMA	PHD finger p	4	101843	1	1	9	0	1205.61	1204.6027	1	1204.5823	0.0204	0	10.78	1.50E+03	A	VNTSPSVPTT

Biotools-Score: 51

MASCOT-Score: 16

known O-glycosylation site

Plasminogen

$_{362}$LAP**T**APPELTPV$_{373}$

Fraction 13

931.40++ → Pep [M+H]+ 1205.64+ [27.9-28.8 min] ETD

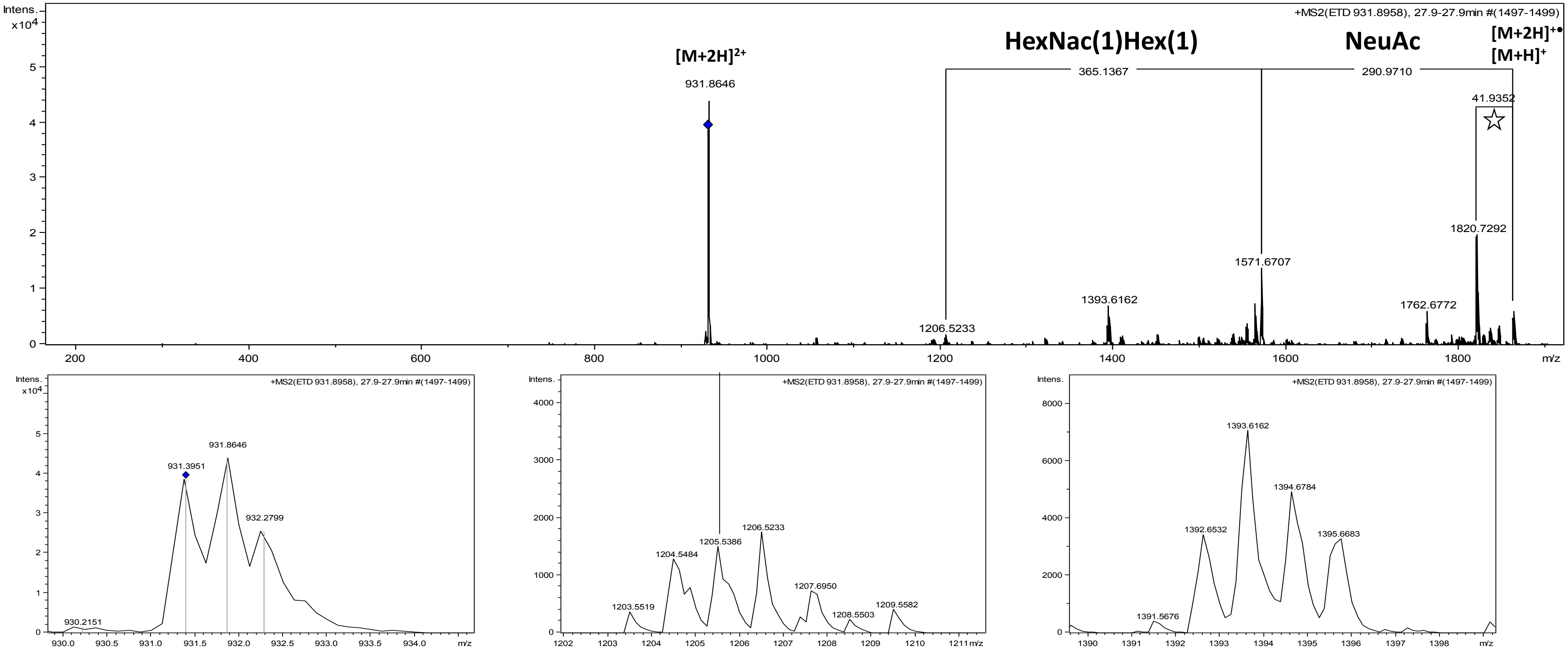

known O-glycosylation site

Plasminogen

$_{362}$LAP**T**APPELTPV$_{373}$

Fraction 13

931.40++ → Pep [M+H]+ 1205.64+ [27.9-28.8 min]

ETD

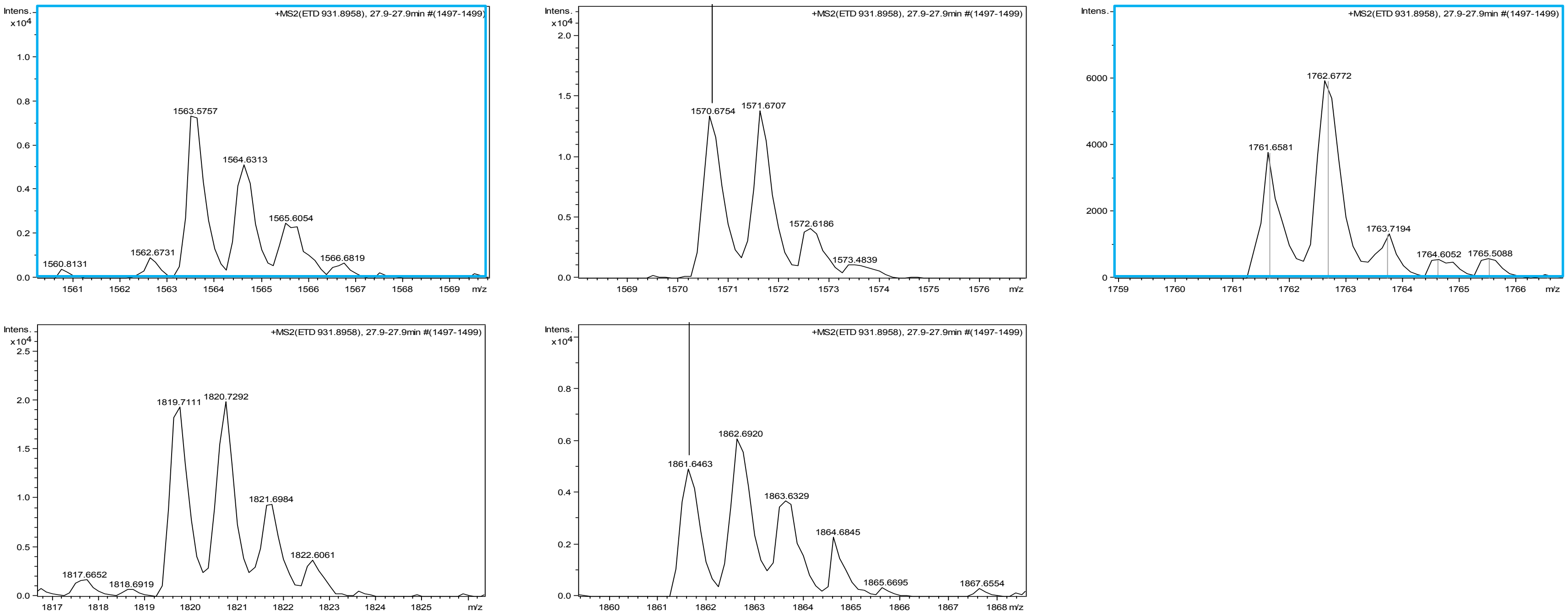

known O-glycosylation site

Plasminogen

$_{362}$LAPTAPPELTPV$_{373}$

Fraction 13

931.40++ → Pep [M+H]+ 1205.64+ [27.9-28.8 min] ETD

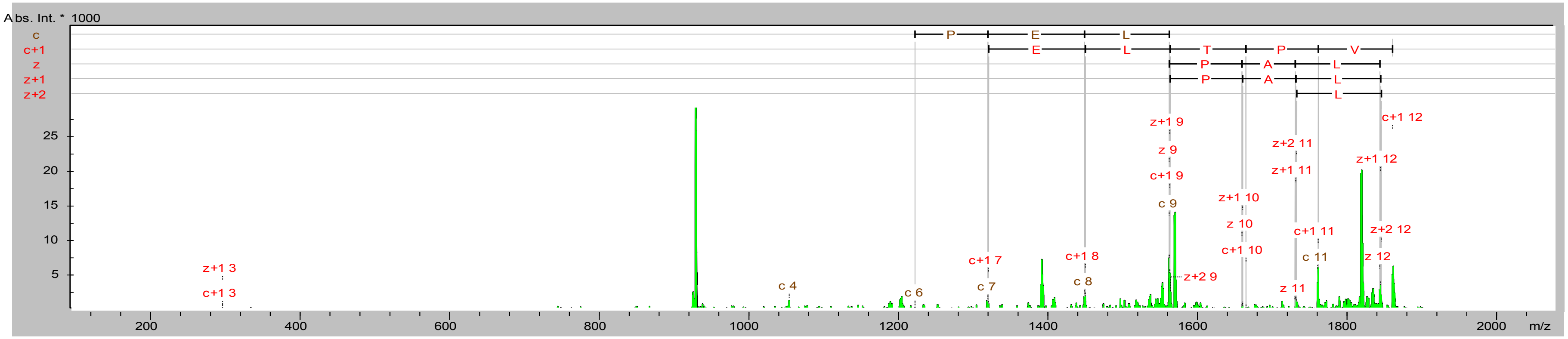

LAPTAPPELTPV

	L	A	P	T	A	P	P	E	L	T	P	V	Leu	Ala	Pro	Thr	Ala	Pro	Pro	Glu	Leu	Thr	Pro	Val
Ion	1	2	3	4	5	6	7	8	9	10	11	12	1	2	3	4	5	6	7	8	9	10	11	12
c	L	A	P	T*	A	P	P	E	L	T	P	V	131.118	202.155	299.208	1056.483	1127.520	1224.573	1321.626	1450.668	1563.752	1664.800	1761.853	1860.921
c+1	L	A	P	T*	A	P	P	E	L	T	P	V	132.126	203.163	300.216	1057.491	1128.528	1225.581	1322.634	1451.676	1564.760	1665.808	1762.861	1861.929
z	L	A	P	T*	A	P	P	E	L	T	P	V	101.060	198.112	299.160	412.244	541.287	638.340	735.392	806.429	1563.705	1660.758	1731.795	1844.879
z+1	L	A	P	T*	A	P	P	E	L	T	P	V	102.068	199.120	300.168	413.252	542.295	639.347	736.400	807.437	1564.713	1661.765	1732.802	1845.887
z+2	L	A	P	T*	A	P	P	E	L	T	P	V	103.075	200.128	301.176	414.260	543.302	640.355	737.408	808.445	1565.720	1662.773	1733.810	1846.894
	12	11	10	9	8	7	6	5	4	3	2	1	Val	Pro	Thr	Leu	Glu	Pro	Pro	Ala	Thr	Pro	Ala	Leu

Biotools-Score: 115

known O-glycosylation site

Plasminogen $_{362}$LAP**T**APPELTPV$_{373}$

Fraction 13

931.40++ → Pep [M+H]+ 1205.64+ [27.9-28.8 min] ETD

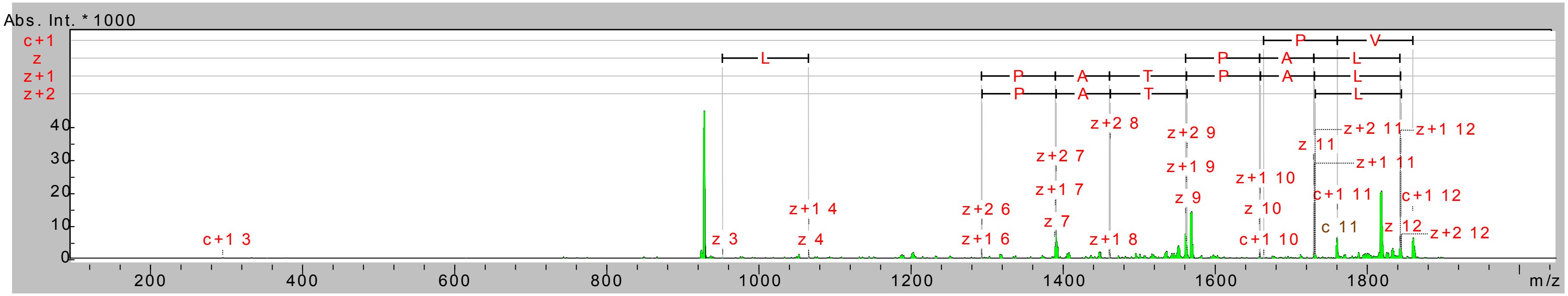

LAPTAPPELTPV

	L	A	P	T	A	P	P	E	L	T	P	V	Leu	Ala	Pro	Thr	Ala	Pro	Pro	Glu	Leu	Thr	Pro	Val
Ion	1	2	3	4	5	6	7	8	9	10	11	12	1	2	3	4	5	6	7	8	9	10	11	12
c	L	A	P	T	A	P	P	E	L	T*	P	V	131.118	202.155	299.208	400.255	471.293	568.345	665.398	794.441	907.525	1664.800	1761.853	1860.921
c+1	L	A	P	T	A	P	P	E	L	T*	P	V	132.126	203.163	300.216	401.263	472.300	569.353	666.406	795.449	908.533	1665.808	1762.861	1861.929
z	L	A	P	T	A	P	P	E	L	T*	P	V	101.060	198.112	955.388	1068.472	1197.514	1294.567	1391.620	1462.657	1563.705	1660.758	1731.795	1844.879
z+1	L	A	P	T	A	P	P	E	L	T*	P	V	102.068	199.120	956.396	1069.480	1198.522	1295.575	1392.628	1463.665	1564.713	1661.765	1732.802	1845.887
z+2	L	A	P	T	A	P	P	E	L	T*	P	V	103.075	200.128	957.403	1070.487	1199.530	1296.583	1393.636	1464.673	1565.720	1662.773	1733.810	1846.894
	12	11	10	9	8	7	6	5	4	3	2	1	Val	Pro	Thr	Leu	Glu	Pro	Pro	Ala	Thr	Pro	Ala	Leu

Biotools-Score: 173

known O-glycosylation site

Plasminogen

$_{362}$LAP**T**APPELTPV$_{373}$
