# Supplementary material for: Site-specific O-Glycosylation Analysis of Human Blood Plasma Proteins
Source: Mol Cell Proteomics. 2015 Nov 23;15(2):624–41. doi: 10.1074/mcp.M115.053546 (PMC4739677; doi:10.1074/mcp.M115.053546)
Supplement: Supplemental Data [file 10.1074_M115.053546_mcp.M115.053546-4.pdf]

# Site-Specific *O*-Glycosylation Analysis of Human Blood Plasma Proteins

## Proteinase K Digest

**Fraction 14**

## Search Parameters For Protein Identification

Project: Blood Plasma Glycoprotomics (2013), ProtK-Digest
Glycopeptides measured on Bruker ESI-Ion Trap MS (CID-$MS^3$)

| | |
|---|---|
| Mascot version 2.2.07 | |
| Database: | SwissProt |
| Fasta file: | SwissProt_51.6.fasta |
| Total sequences: | 257964 |
| Total residues: | 93947433 |
| Sequences after taxonomy filter: | 15720 |
| Number of queries: | 1 |

**Variable modifications** -------------------------------------------------------

| *Identifier* | *Name* | *Delta* | *Neutral loss(es)* |
|---|---|---|---|
| 1 | Deamidated (NQ) | 0.984009 | 0 |
| 2 | Oxidation (M) | 15.994919 | 63.998285 |

**Search Parameters** -------------------------------------------------------

| | |
|---|---|
| Taxonomy filter: | Homo sapiens (human) |
| Enzyme: | None |
| Maximum Missed Cleavages: | 0 |
| Fixed modifications | Carbamidomethyl (C) |
| ICAT experiment | 0 |
| Variable modifications | Deamidated (NQ), Oxidation (M) |
| Peptide Mass Tolerance | 0.3 |
| Peptide Mass Tolerance Units | Da |
| Fragment Mass Tolerance | 0.35 |
| Fragment Mass Tolerance Units | Da |
| Mass values | Monoisotopic |
| Instrument type | ESI-TRAP |
| Isotope error mode | 1 |

**Format parameters** -------------------------------------------------------

| | |
|---|---|
| Significance threshold | 0.05 |
| Max. number of hits | 20 |
| Use MudPIT protein scoring | 0 |
| Ions score cut-off | 0 |
| Include same-set proteins | 0 |
| Include sub-set proteins | 0 |
| Include unassigned | 0 |
| Require bold red | 0 |

# Extracted ion chromatograms of glycan-specific oxonium ions

| **Oxonium Ions** | **$[M+H]^+$ m/z** |
|---|---|
| Fuc | 147.08 |
| Hex | 163.06 |
| HexNAc | 204.09 |
| NeuAc -$H_2O$ | 274.09 |
| NeuAc | 292.10 |
| HexNAc(1)Hex(1) | 366.14 |
| Hex(1)NeuAc(1) | 454.16 |
| HexNAc(1)NeuAc(1) | 495.18 |
| HexNAc(1)Hex(1)Fuc(1) | 512.21 |
| HexNAc(1)Hex(2) | 528.19 |
| HexNAc(1)Hex(1)NeuAc(1) | 657.24 |

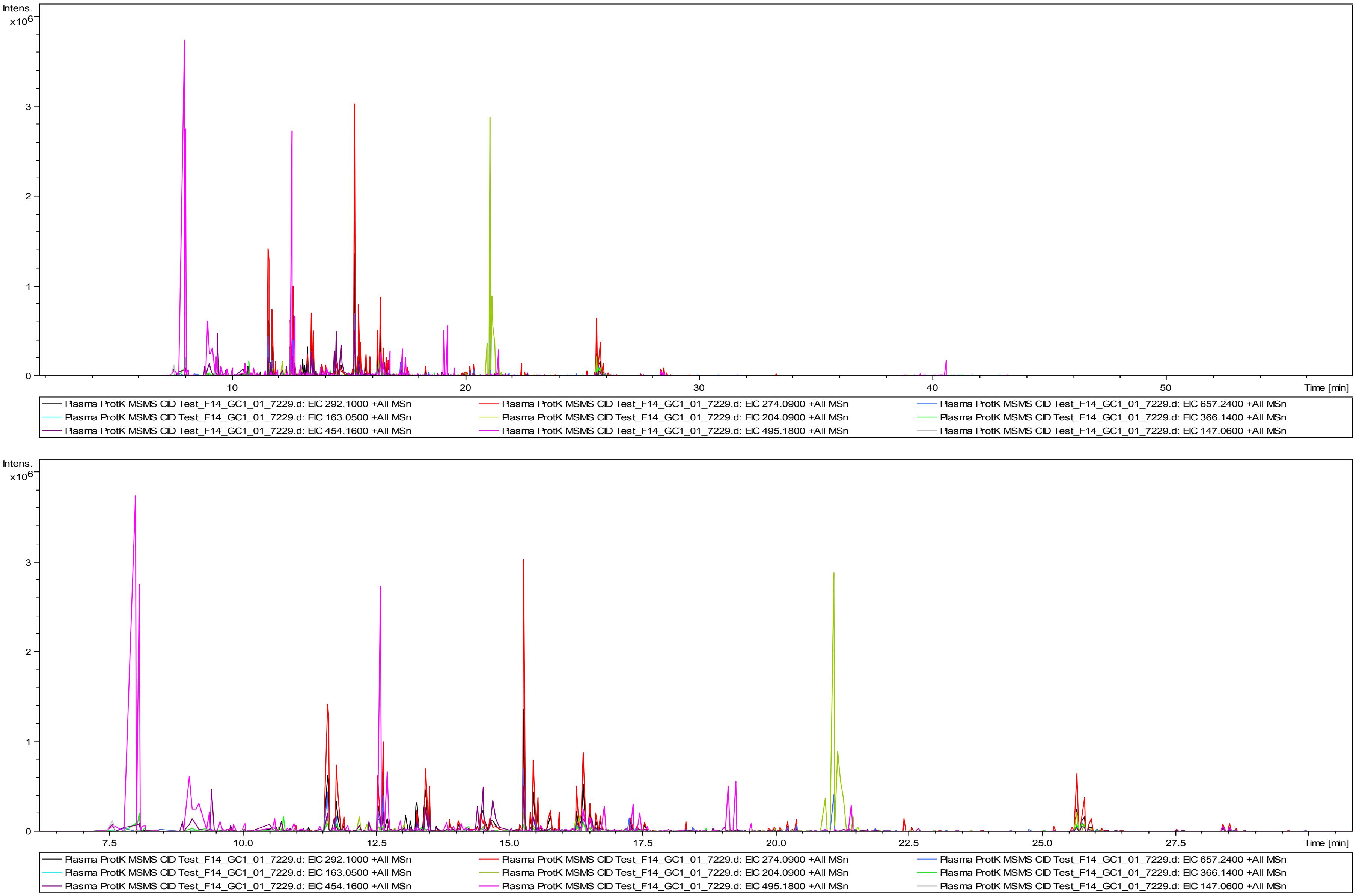


**Extracted ion chromatograms of glycan-specific oxonium ions**

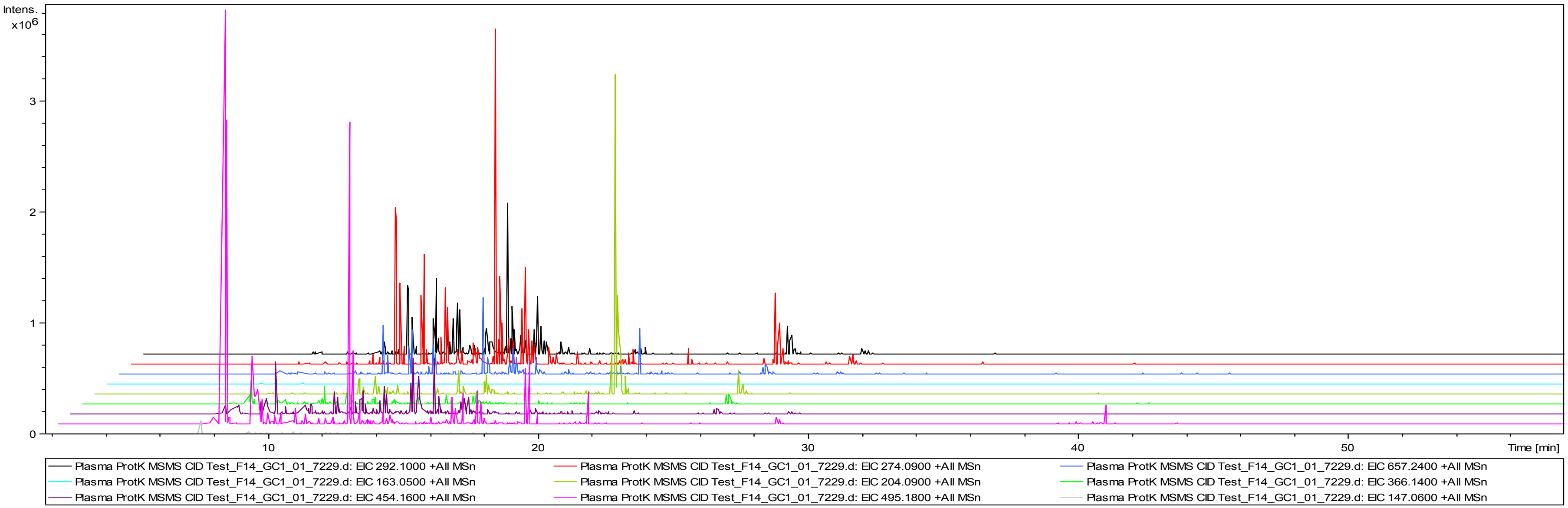


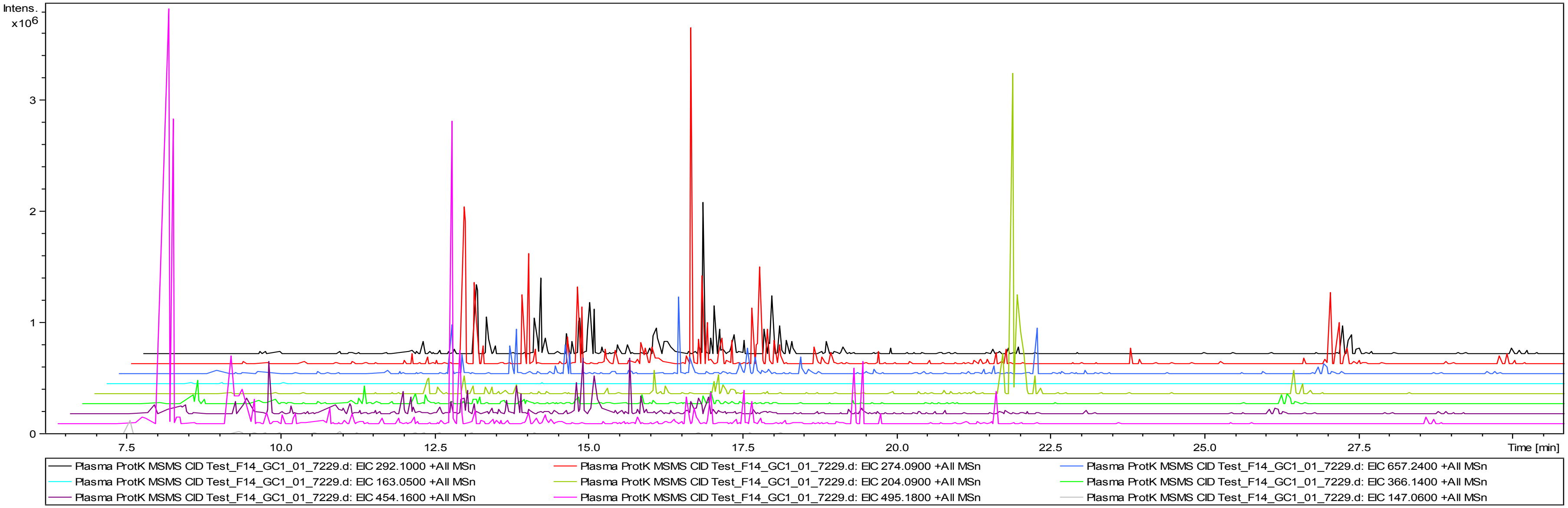


**Extracted ion chromatograms of glycan-specific oxonium ions**

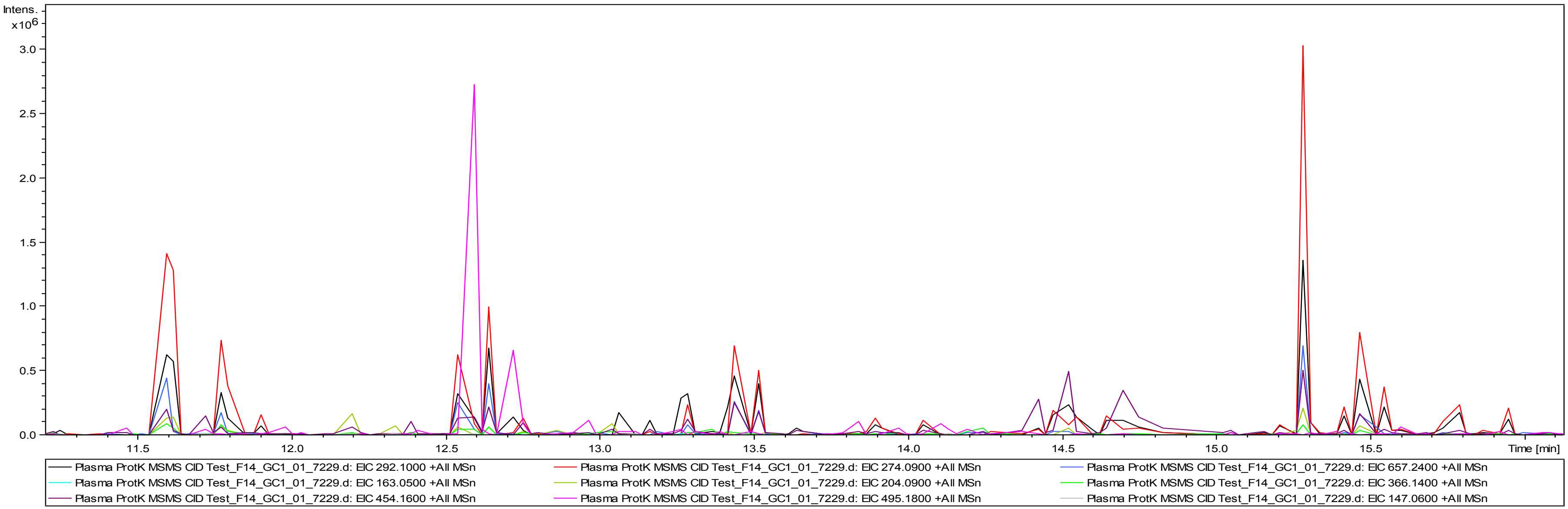


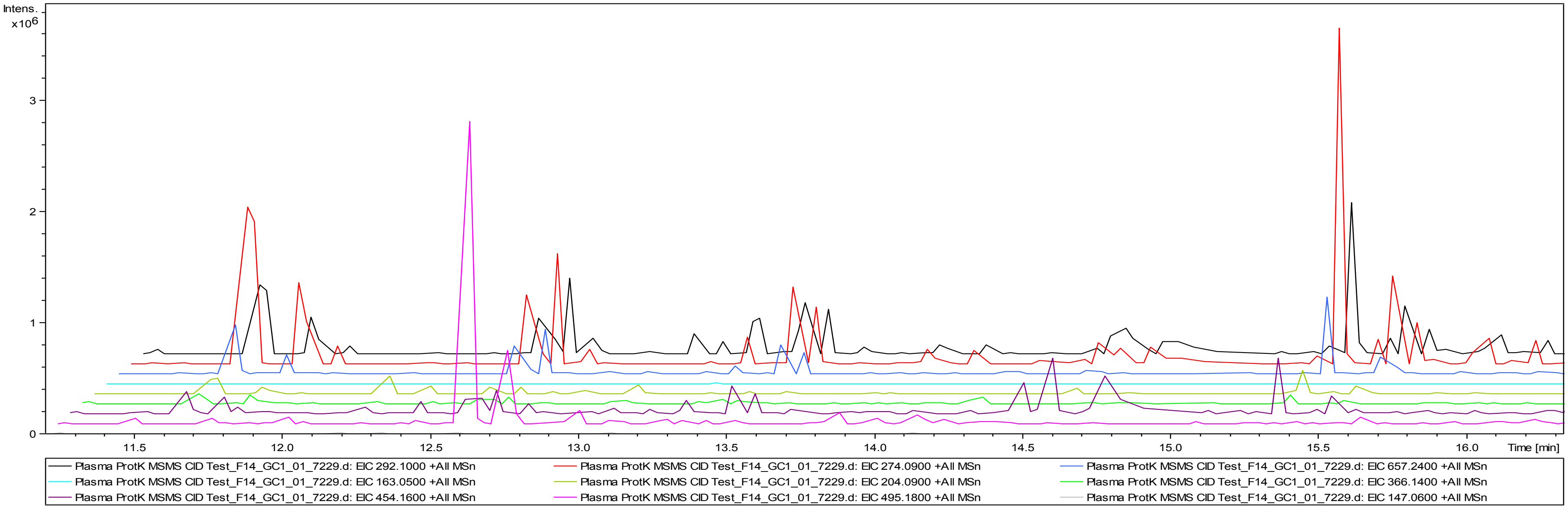


**Extracted ion chromatograms of glycan-specific oxonium ions**

# Supplementary Figure 4: Human Blood Plasma O-Glycoproteomics, HILIC Fraction 14

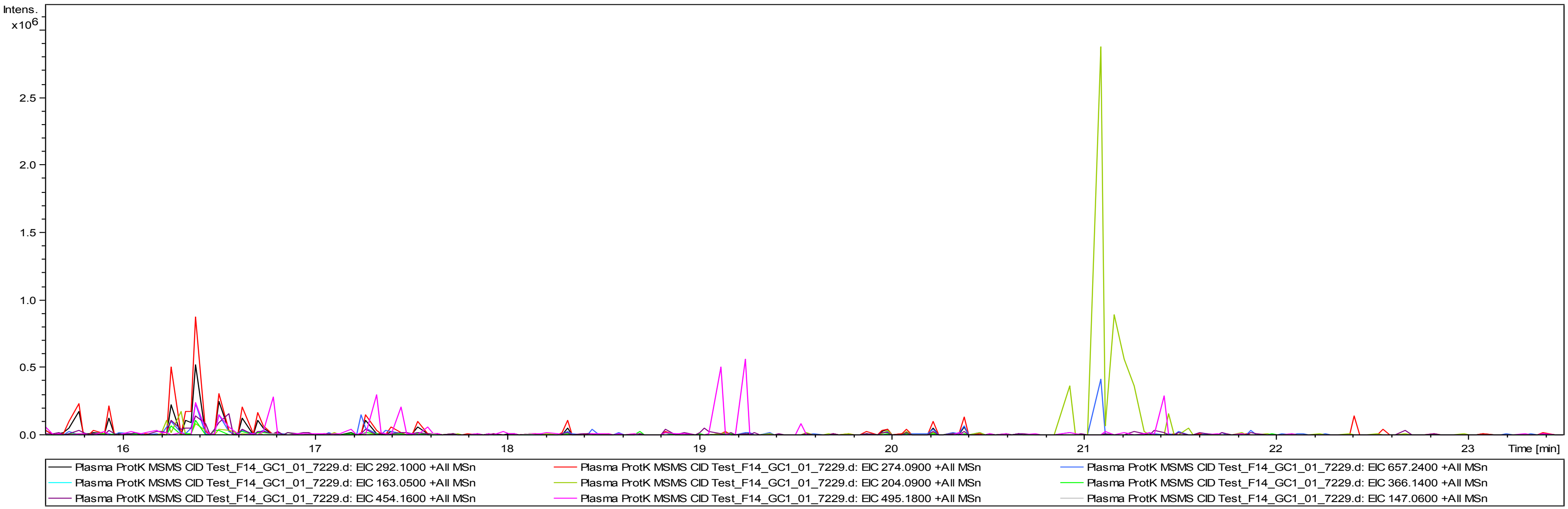


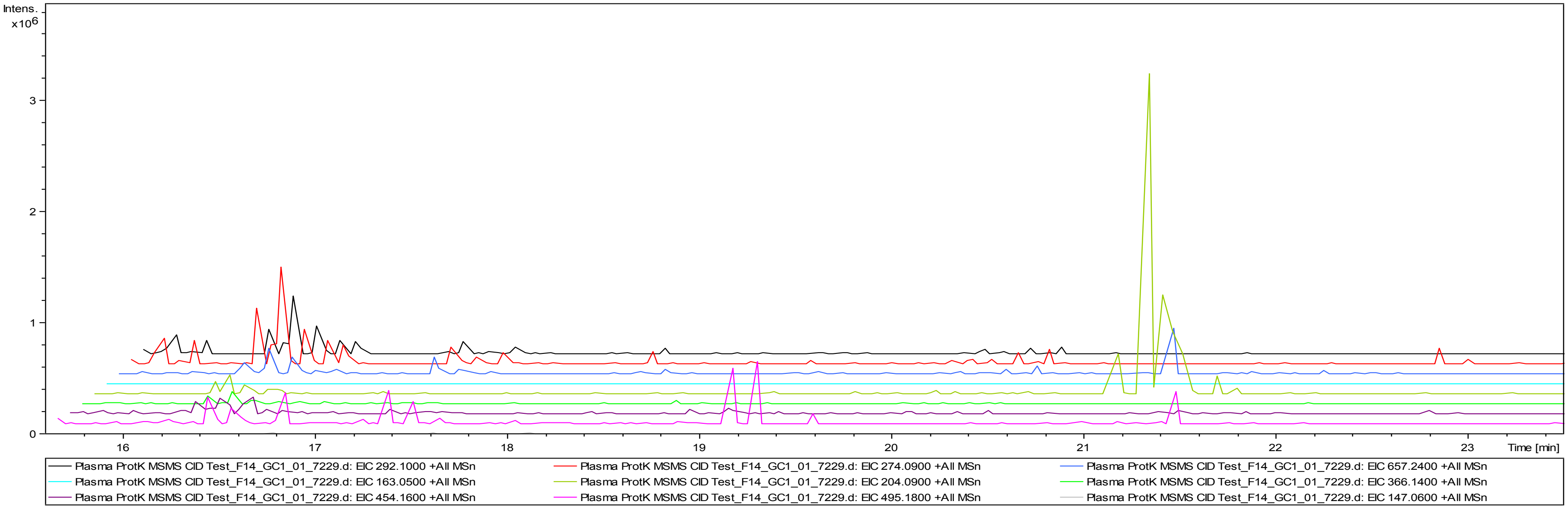


**Extracted ion chromatograms of glycan-specific oxonium ions**

## Supplementary Figure 4: Human Blood Plasma O-Glycoproteomics, HILIC Fraction 14

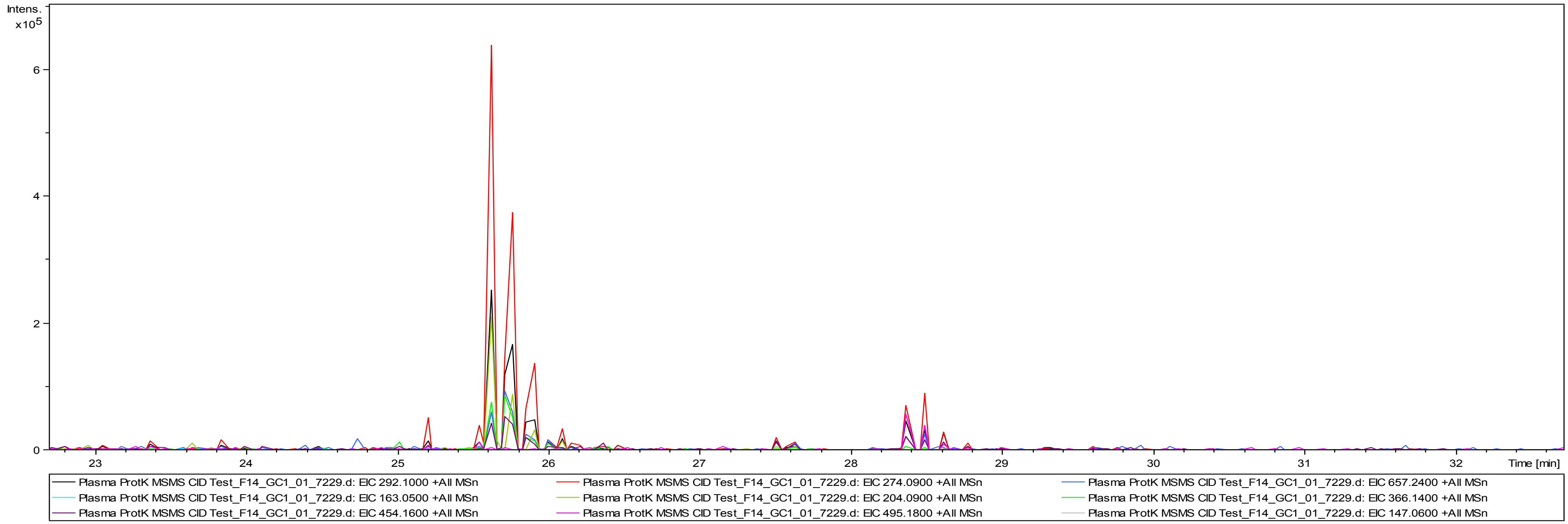


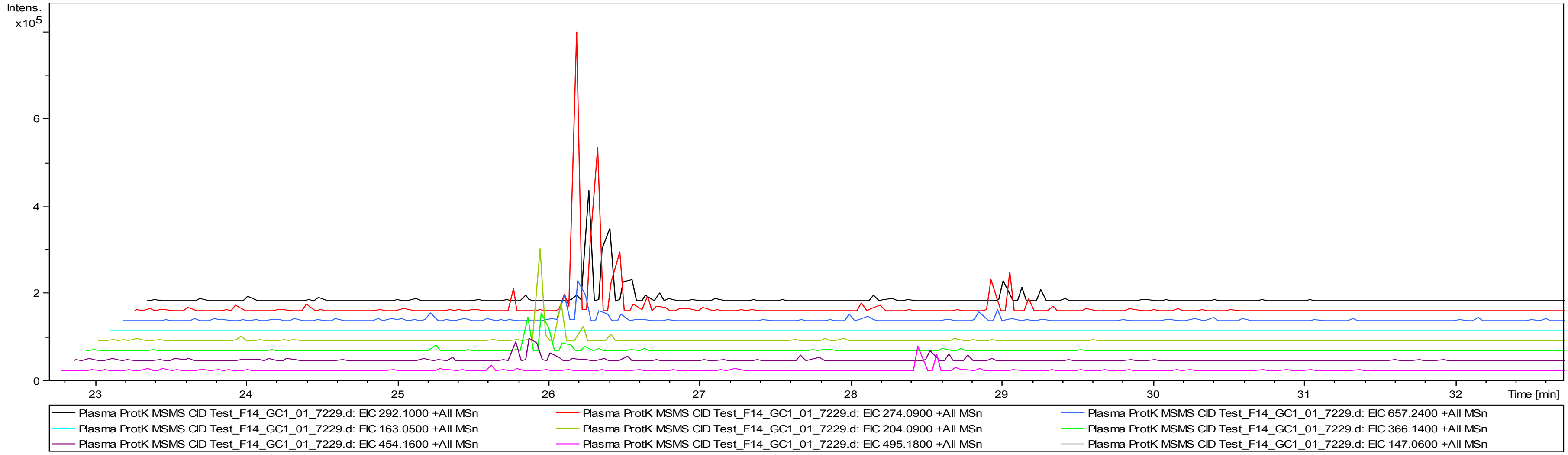


**Extracted ion chromatograms of glycan-specific oxonium ions**

# Fraction 14

719.23++ → Pep [M+H]+ 781.37+ [11.0-11.2 min] CID-MS Precursor

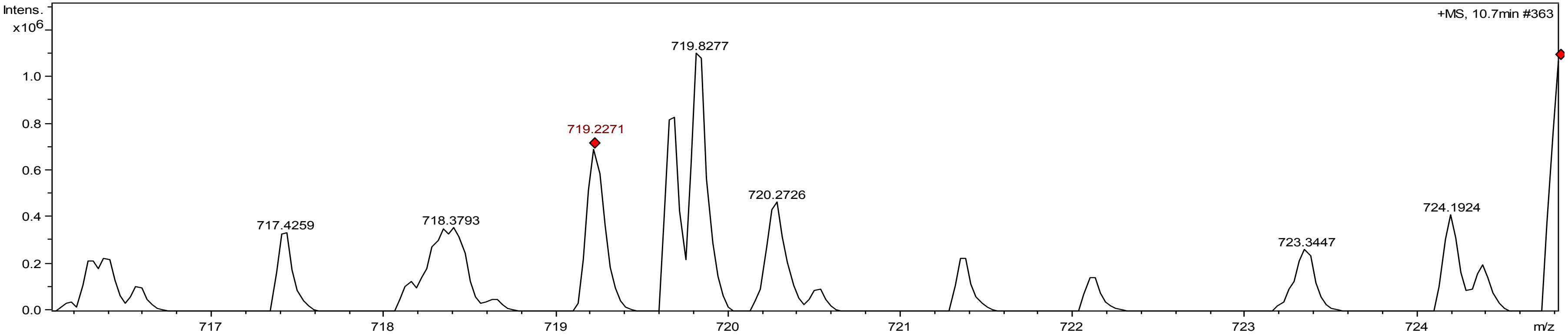


ETD spectrum of poor quality

# Fraction 14

## 719.23++ → Pep [M+H]+ 781.37+ [11.0-11.2 min] CID-MS2

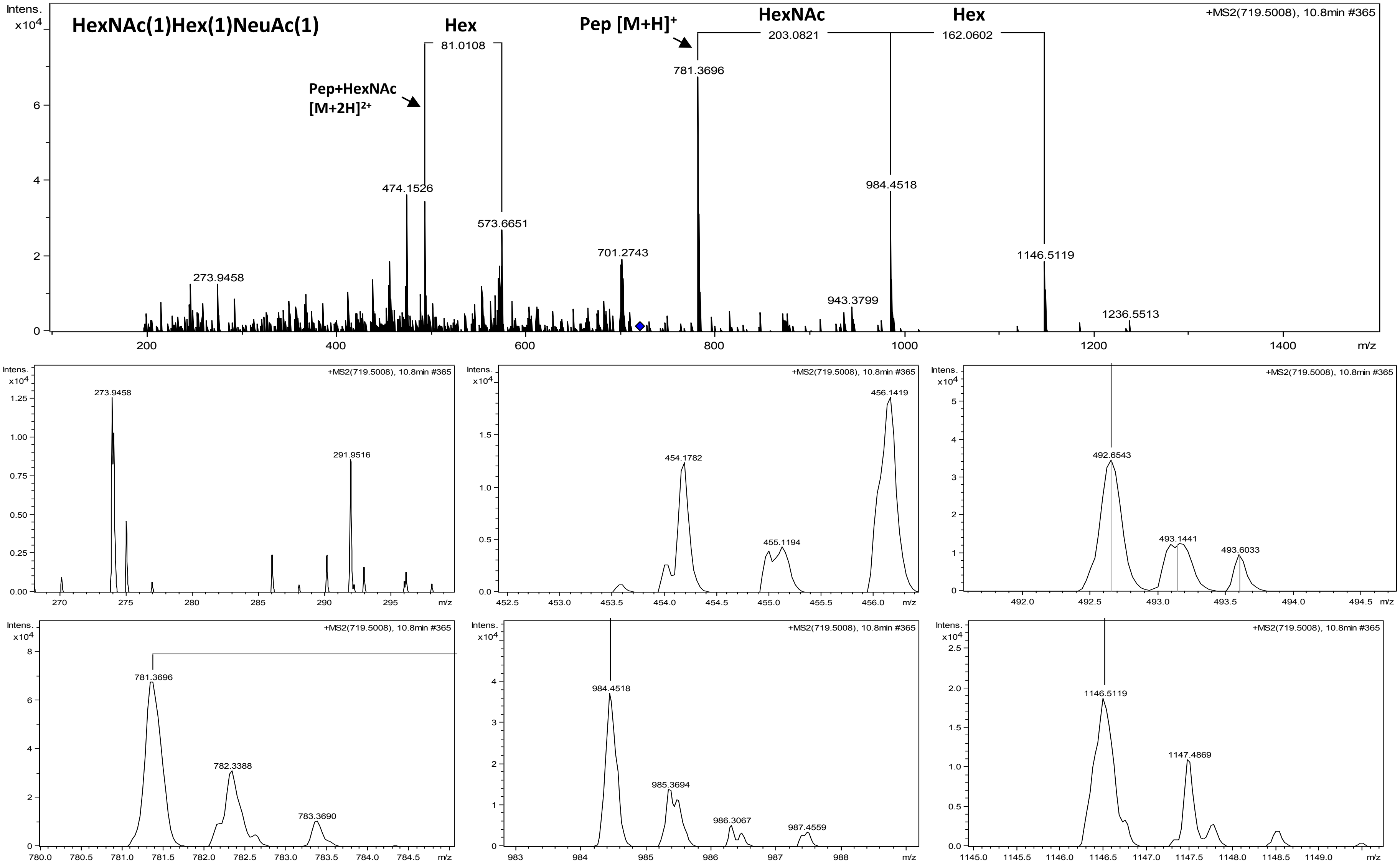

# Fraction 14

719.23++ → Pep [M+H]+ 781.37+ [11.0-11.2 min] CID-MS3

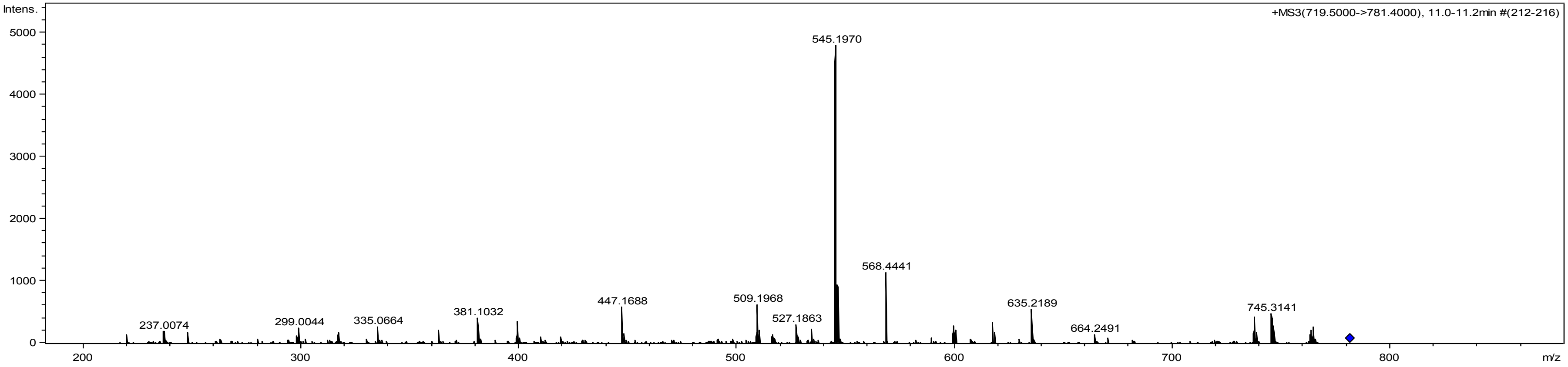


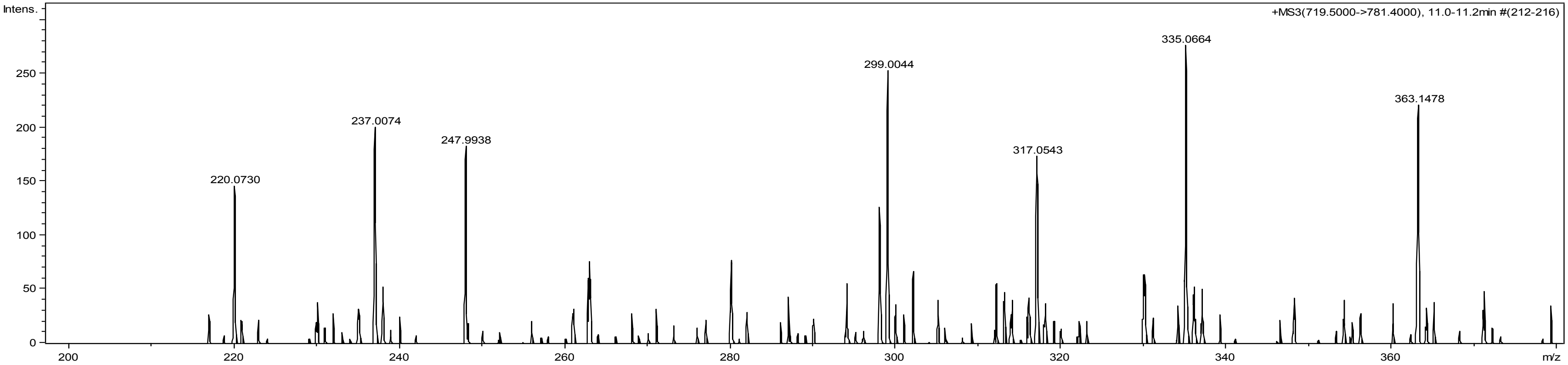

# Fraction 14

## 719.23++ → Pep [M+H]+ 781.37+ [11.0-11.2 min]

## CID-MS3

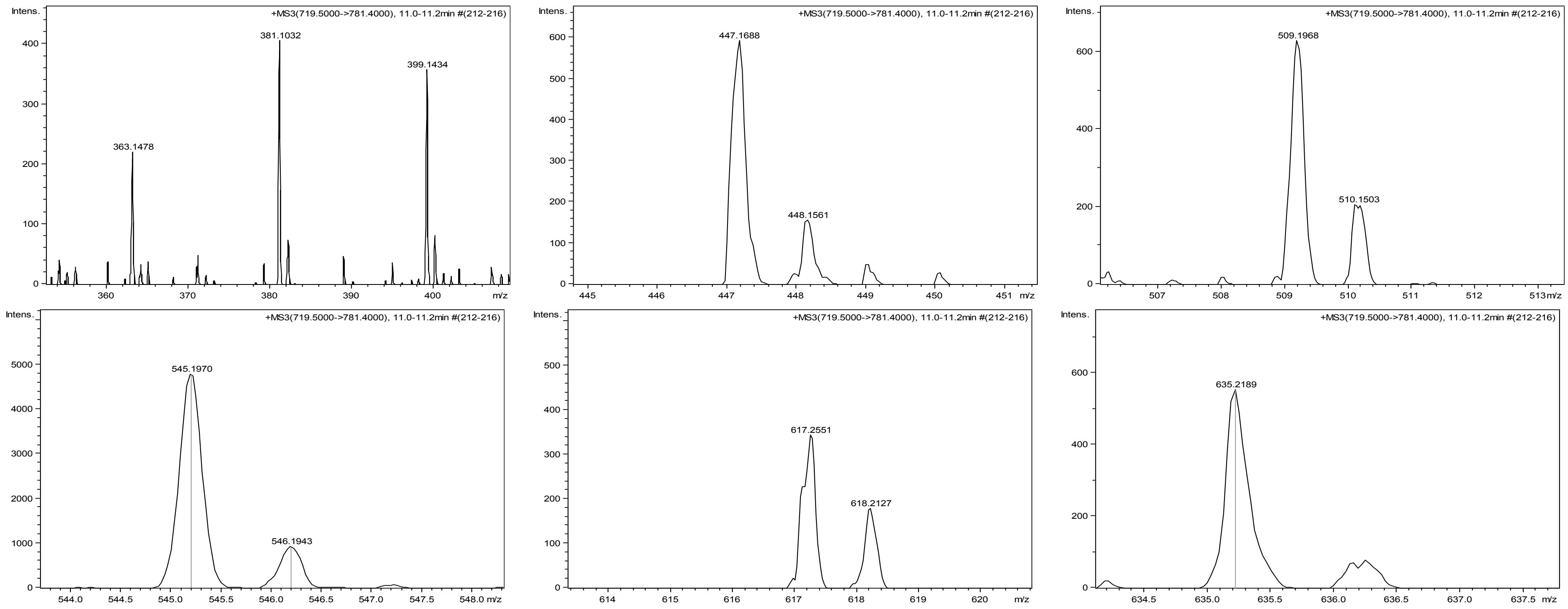

# Fraction 14

## 719.23++ → Pep [M+H]+ 781.37+ [11.0-11.2 min] CID-MS3

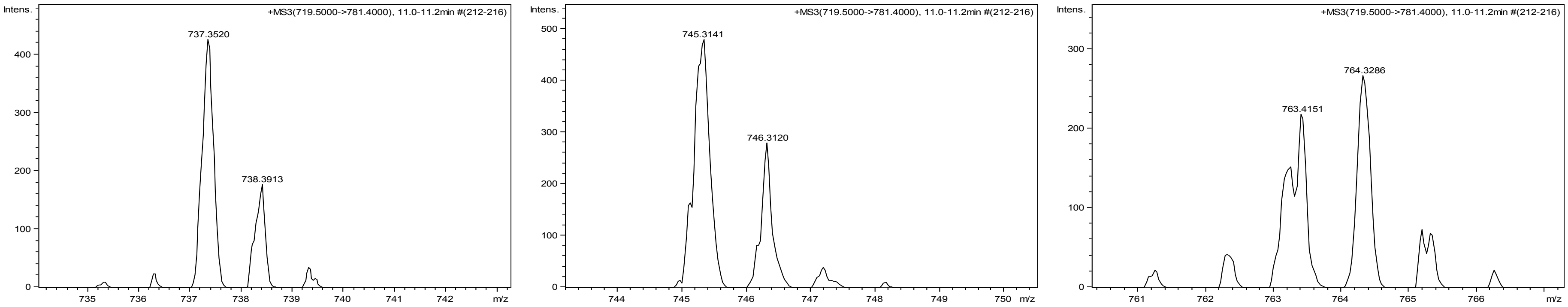

# Fraction 14

719.23++ → Pep [M+H]+ 781.37+ [11.0-11.2 min]

CID-MS3 MASCOT Search

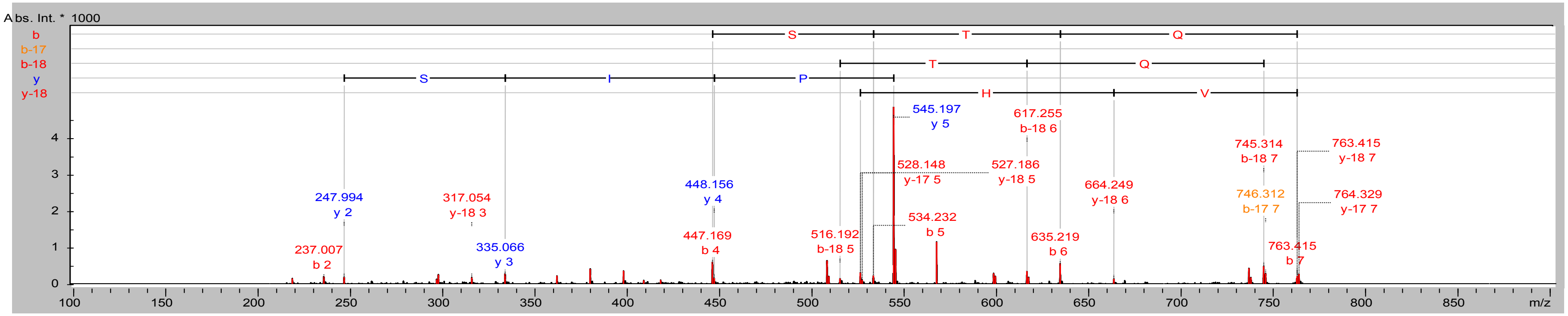


| | V | H | P | I | S | T | Q | Val | His | Pro | Ile | Ser | Thr | Gln |
|---|---|---|---|---|---|---|---|---|---|---|---|---|---|---|
| Ion | 1 | 2 | 3 | 4 | 5 | 6 | 7 | 1 | 2 | 3 | 4 | 5 | 6 | 7 |
| b | V | H | P | I | S | T | Q | 100.076 | 237.135 | 334.187 | 447.271 | 534.303 | 635.351 | 763.410 |
| b-17 | V | H | P | I | S | T | Q | - | - | - | - | - | - | 746.383 |
| b-18 | V | H | P | I | S | T | Q | - | - | - | - | 516.293 | 617.341 | 745.399 |
| y | V | H | P | I | S | T | Q | 147.076 | 248.124 | 335.156 | 448.240 | 545.293 | 682.352 | 781.420 |
| y-17 | V | H | P | I | S | T | Q | 130.050 | 231.098 | 318.130 | 431.214 | 528.266 | 665.325 | 764.394 |
| y-18 | V | H | P | I | S | T | Q | - | 230.114 | 317.146 | 430.230 | 527.282 | 664.341 | 763.410 |
| | 7 | 6 | 5 | 4 | 3 | 2 | 1 | Gln | Thr | Ser | Ile | Pro | His | Val |

unknown O-glycosylation region

Kininogen-1

$_{146}$VHPI**ST**Q$_{152}$

# Fraction 14

719.23++ → Pep [M+H]+ 781.37+ [11.0-11.2 min]

CID-MS3 MASCOT Search

| prot_hit_nur | prot_acc | prot_desc | prot_score | prot_mass | prot_matche | pep_query | pep_rank | pep_isbold | pep_exp_mz | pep_exp_mr | pep_exp_z | pep_calc_mr | pep_delta | pep_miss | pep_score | pep_expect | pep_res_bef | pep_seq |
|---|---|---|---|---|---|---|---|---|---|---|---|---|---|---|---|---|---|---|
| 1 | KNG1_HUMA | Kininogen-1 | 19 | 72996 | 1 | 1 | 1 | 1 | 781.4 | 780.3927 | 1 | 780.413 | -0.0203 | 0 | 24.09 | 49 | C | VHPISTQ |
| 2 | F125A_HUM/ | Protein FAM | 8 | 29107 | 1 | 1 | 6 | 0 | 781.4 | 780.3927 | 1 | 780.3476 | 0.0451 | 0 | 11.14 | 9.60E+02 | K | SCSPLAF |
| 3 | STX5_HUMAI | Syntaxin-5 - | 7 | 39762 | 1 | 1 | 7 | 0 | 781.4 | 780.3927 | 1 | 780.4381 | -0.0454 | 0 | 10.99 | 9.90E+02 | V | YLGLSKT |
| 4 | S18L1_HUMA | SS18-like prc | 7 | 43078 | 1 | 1 | 3 | 0 | 781.4 | 780.3927 | 1 | 780.3072 | 0.0855 | 0 | 12.92 | 6.40E+02 | G | QGSQGSSM |
| 5 | MXRA5_HUN | Matrix-remo | 6 | 314195 | 1 | 1 | 2 | 0 | 781.4 | 780.3927 | 1 | 780.4017 | -0.009 | 0 | 14.78 | 4.20E+02 | D | AFNLSTK |
| 6 | PPB1_HUMA | Alkaline pho | 6 | 58259 | 1 | 1 | 7 | 0 | 781.4 | 780.3927 | 1 | 780.4381 | -0.0454 | 0 | 10.99 | 9.90E+02 | P | YVALSKT |
| 7 | ZN181_HUM/ | Zinc finger p | 6 | 67339 | 1 | 1 | 4 | 0 | 781.4 | 780.3927 | 1 | 780.3324 | 0.0604 | 0 | 11.67 | 8.50E+02 | S | CSSNLTV |
| 8 | RXFP1_HUM/ | Relaxin rece | 5 | 88475 | 1 | 1 | 4 | 0 | 781.4 | 780.3927 | 1 | 780.3324 | 0.0604 | 0 | 11.67 | 8.50E+02 | I | SCSNLTV |
| 9 | GOGA3_HUN | Golgin subfa | 4 | 167765 | 1 | 1 | 7 | 0 | 781.4 | 780.3927 | 1 | 780.4381 | -0.0454 | 0 | 10.99 | 9.90E+02 | T | YGILSKT |
| 10 | SMC3_HUMA | Structural ma | 3 | 141853 | 1 | 1 | 10 | 0 | 781.4 | 780.3927 | 1 | 780.3324 | 0.0604 | 0 | 10.46 | 1.10E+03 | R | SMEVSTQ |

Biotools-Score: 29

MASCOT-Score: 24

unknown O-glycosylation region

Kininogen-1

$_{146}$VHPI**ST**Q$_{152}$

# Fraction 14

622.72++ → Pep [M+H]+ 588.26+ [11.6-11.8 min] CID-MS Precursor

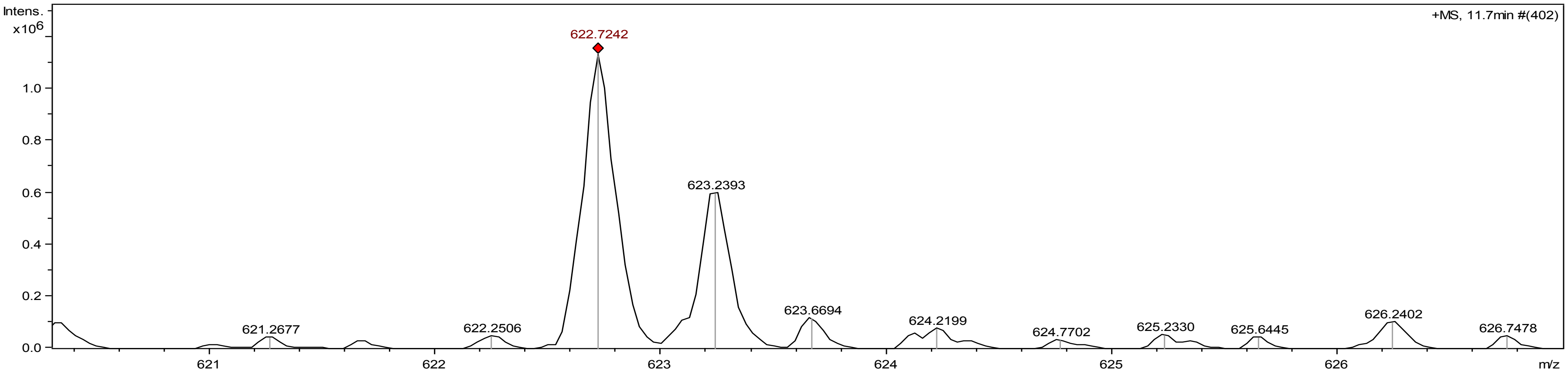


ETD spectrum not available

# Fraction 14

622.72++ → Pep [M+H]+ 588.26+ [11.6-11.8 min] CID-MS2

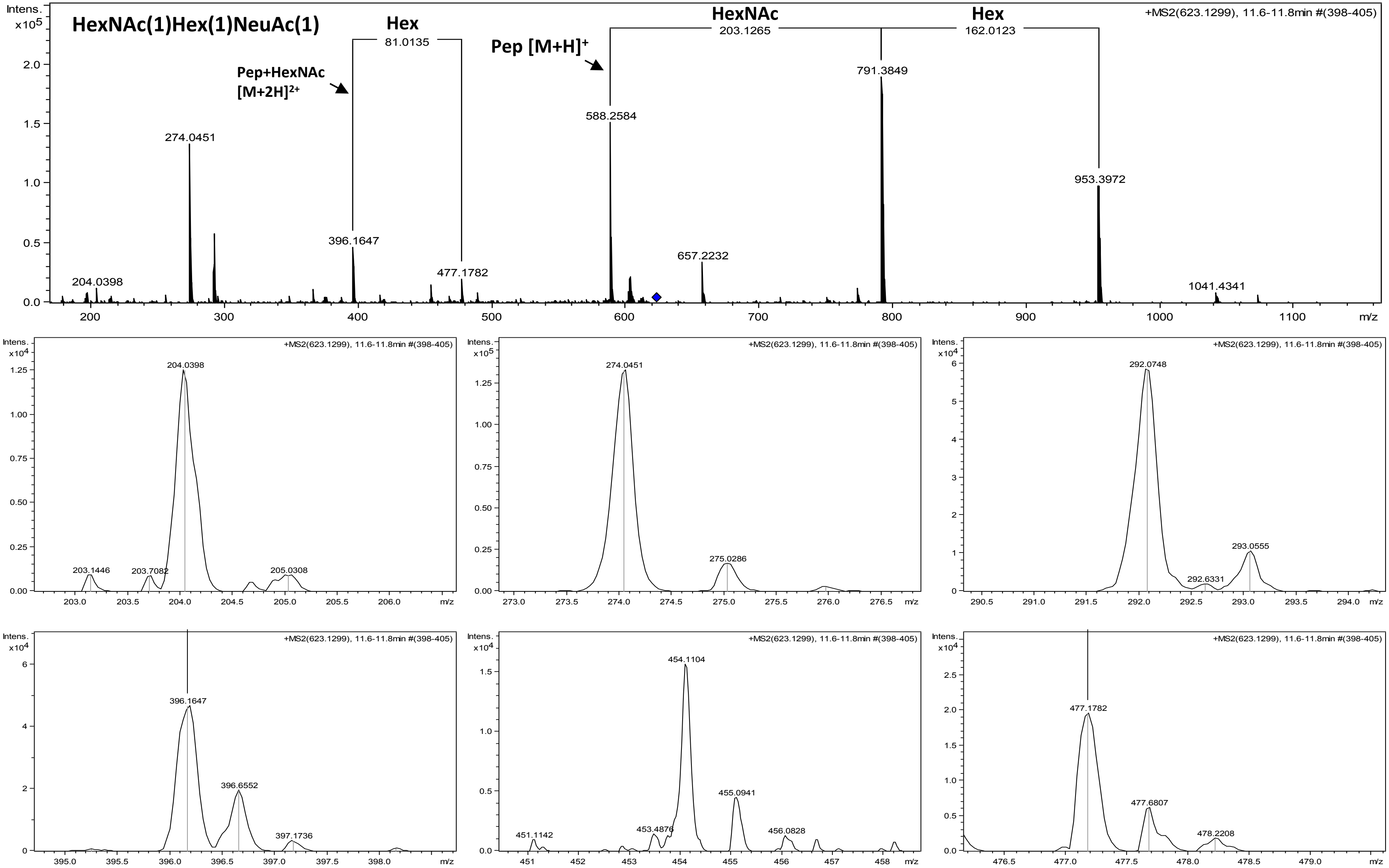

# Fraction 14

622.72++ → Pep [M+H]+ 588.26+ [11.6-11.8 min]

CID-MS2

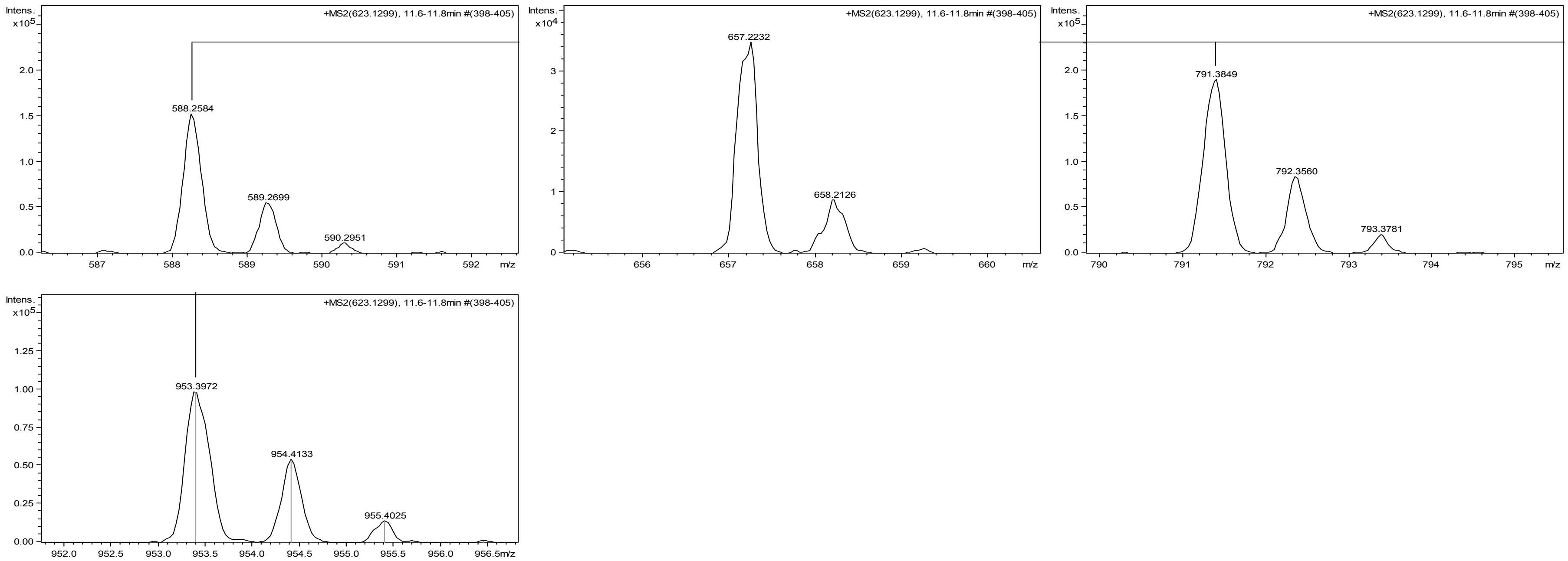

# Fraction 14

622.72++ → Pep [M+H]+ 588.26+ [11.6-11.8 min]

CID-MS3 MASCOT Search

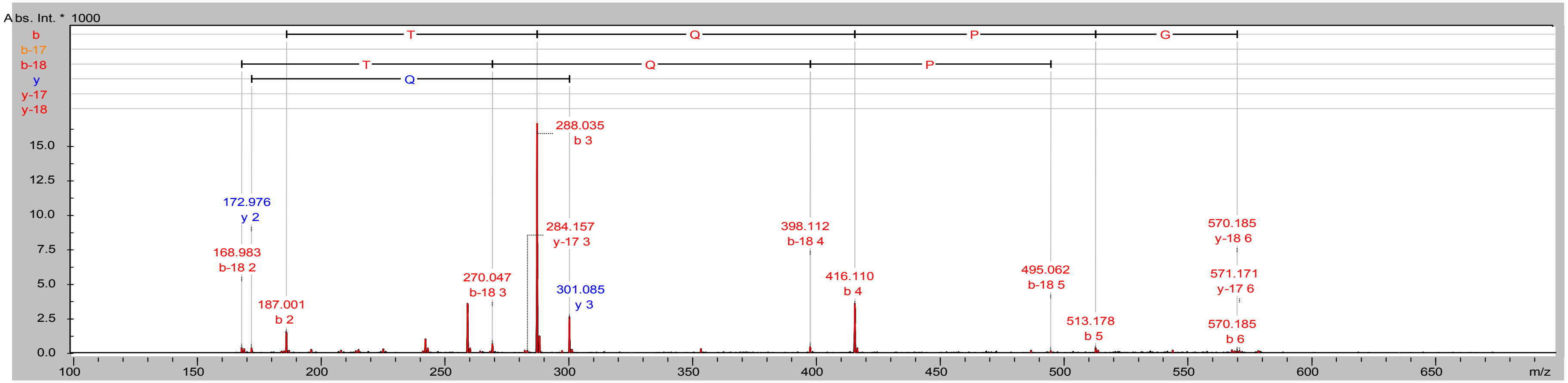


| | E | G | T | Q | P | G | Glu | Gly | Thr | Gln | Pro | Gly |
|---|---|---|---|---|---|---|---|---|---|---|---|---|
| Ion | 1 | 2 | 3 | 4 | 5 | 6 | 1 | 2 | 3 | 4 | 5 | 6 |
| b | E | G | T | Q | P | G | 130.050 | 187.071 | 288.119 | 416.178 | 513.230 | 570.252 |
| b-17 | E | G | T | Q | P | G | - | - | - | 399.151 | 496.204 | 553.225 |
| b-18 | E | G | T | Q | P | G | 112.039 | 169.061 | 270.108 | 398.167 | 495.220 | 552.241 |
| y | E | G | T | Q | P | G | 76.039 | 173.092 | 301.151 | 402.198 | 459.220 | 588.262 |
| y-17 | E | G | T | Q | P | G | - | - | 284.124 | 385.172 | 442.193 | 571.236 |
| y-18 | E | G | T | Q | P | G | - | - | - | 384.188 | 441.209 | 570.252 |
| | 6 | 5 | 4 | 3 | 2 | 1 | Gly | Pro | Gln | Thr | Gly | Glu |

# Fraction 14

622.72++ → Pep [M+H]+ 588.26+ [11.6-11.8 min]

CID-MS3 MASCOT Search

| prot_hit_nur | prot_acc | prot_desc | prot_score | prot_mass | prot_matche | pep_query | pep_rank | pep_isbold | pep_exp_mz | pep_exp_mr | pep_exp_z | pep_calc_mr | pep_delta | pep_miss | pep_score | pep_expect | pep_res_bef | pep_seq |
|---|---|---|---|---|---|---|---|---|---|---|---|---|---|---|---|---|---|---|
| 1 | ELAF_HUMAI | Elafin precursor (Elastase-specific inhibitor) (ESI) ( | 25 | 12718 | 1 | 1 | 3 | 1 | 588,3 | 587,2927 | 1 | 587,3279 | -0,0352 | 0 | 28,88 | 18 | P | VSTKPG |
| 2 | CD1D_HUMA | T-cell surface glycoprotein CD1d precursor (CD1d a | 25 | 38092 | 1 | 1 | 3 | 0 | 588,3 | 587,2927 | 1 | 587,2551 | 0,0376 | 0 | 28,88 | 18 | Q | QGTQPG |
| 3 | YBOX1_HUM | Nuclease sensitive element-binding protein 1 (Y-I | 25 | 35903 | 1 | 1 | 3 | 0 | 588,3 | 587,2927 | 1 | 587,2915 | 0,0012 | 0 | 28,88 | 18 | A | ADTKPG |
| 4 | GNAQ_HUM/ | Guanine nucleotide-binding protein G(q) subunit | 24 | 41726 | 1 | 1 | 3 | 0 | 588,3 | 587,2927 | 1 | 587,234 | 0,0587 | 0 | 28,88 | 18 | L | WNDPG |
| 5 | GBRB3_HUM | Gamma-aminobutyric-acid receptor subunit beta- | 23 | 54423 | 1 | 1 | 3 | 0 | 588,3 | 587,2927 | 1 | 587,2551 | 0,0376 | 0 | 28,88 | 18 | Q | SVNDPG |
| 6 | ACE2_HUMA | Angiotensin-converting enzyme 2 precursor (EC 3. | 23 | 92860 | 1 | 1 | 1 | 0 | 588,3 | 587,2927 | 1 | 587,2187 | 0,074 | 0 | 28,91 | 18 | K | GENNPG |
| 7 | SP2_HUMAN | Transcription factor Sp2 - Homo sapiens (Human) | 22 | 65487 | 1 | 1 | 1 | 0 | 588,3 | 587,2927 | 1 | 587,2551 | 0,0376 | 0 | 28,91 | 18 | A | GETQPG |
| 8 | PCF11_HUM/ | Pre-mRNA cleavage complex 2 protein Pcf11 (Pre- | 22 | 184666 | 1 | 1 | 3 | 0 | 588,3 | 587,2927 | 1 | 587,2915 | 0,0012 | 0 | 28,88 | 18 | K | QGTKPG |
| 9 | LRBA_HUMA | Lipopolysaccharide-responsive and beige-like anc | 20 | 321639 | 1 | 1 | 3 | 0 | 588,3 | 587,2927 | 1 | 587,2551 | 0,0376 | 0 | 28,88 | 18 | A | ANTQPG |
| 10 | PCD16_HUM. | Protocadherin-16 precursor (Dachsous-1) (Cadher | 20 | 346712 | 1 | 1 | 3 | 0 | 588,3 | 587,2927 | 1 | 587,2551 | 0,0376 | 0 | 28,88 | 18 | P | EGTQPG |

**No unambiguous result**

# Fraction 14

622.72++ → Pep [M+H]+ 588.26+ [11.6-11.8 min]    CID-MS3    MS-Homology Search

UCSF University of California, San Francisco | About UCSF | Search UCSF | UCSF Medical Center



## MS-Homology Search Results

Search completed. 3 sec elapsed. 0 sec remaining.

[–] **Parameters**

Database searched: **SwissProt.2012.12.3**
Digest Used: **No enzyme**
Max. # Missed Cleavages: **1**
Min matches: **1**
Score matrix: **BLOSUM62**
List of Sequences: **1 [{GE}|{DA}|{VS}]T[K|Q]PG 0**
Mass Tolerance: **0.3 Da**

Missing fragment ion: unable to deduce correct sequence

MS resolution not adequate to discriminate between Q/K

[–] **Pre Search Results (SwissProt.2012.12.3)**

Number of entries in the database: **453850**
Full Molecular Weight range: **453850** entries.
Full pI range: **453850** entries.
Taxonomy search **HOMO SAPIENS** selects **20479** entries.
Pre searches select **20479** entries.

MS-Homology search selects **5** entries.

Number of Peptide Hits: **5**

Elafin is reported to be non-glycosylated

| Protein Score | Peptide Score | Peptide Sequence | Matching Sequence | Start AA | MS-Digest Index # | Protein MW (Da)/pI | Accession # | Species | Protein Name |
|---|---|---|---|---|---|---|---|---|---|
| 34 | 34 | GETQPG | (A)GETQPG(E) | 492 | 167152 | 64901/10.0 | Q02086 | HUMAN | Transcription factor Sp2 |
| 34 | 34 | GETQPG | (R)GETQPG(V) | 143 | 248229 | 18106/10.0 | Q6ZS92 | HUMAN | Putative uncharacterized protein FLJ45721 |
| 34 | 34 | EGTQPG | (P)EGTQPG(T) | 589 | 407896 | 346184/4.8 | Q96JQ0 | HUMAN | Protocadherin-16 |
| 33 | 33 | ADTKPG | (A)ADTKPG(T) | 23 | 317990 | 35924/9.9 | P67808 M | BOVIN | Nuclease-sensitive element-binding protein 1 |
| 31 | 31 | VSTKPG | (P)VSTKPG(S) | 69 | 52981 | 12270/9.1 | P19957 | HUMAN | Elafin |

MS-Homology in ProteinProspector 5.10.11 Basic
© Copyright (1999-2013) The Regents of the University of California.

# Fraction 14

622.72++ → Pep [M+H]+ 588.26+ [11.6-11.8 min]

CID-MS3 BioTools

| | G | E | T | Q | P | G | Gly | Glu | Thr | Gln | Pro | Gly |
|---|---|---|---|---|---|---|---|---|---|---|---|---|
| Ion | 1 | 2 | 3 | 4 | 5 | 6 | 1 | 2 | 3 | 4 | 5 | 6 |
| b | G | E | T | Q | P | G | 58.029 | 187.071 | 288.119 | 416.178 | 513.230 | 570.252 |
| b-17 | G | E | T | Q | P | G | - | - | - | 399.151 | 496.204 | 553.225 |
| b-18 | G | E | T | Q | P | G | - | 169.061 | 270.108 | 398.167 | 495.220 | 552.241 |
| y | G | E | T | Q | P | G | 76.039 | 173.092 | 301.151 | 402.198 | 531.241 | 588.262 |
| y-17 | G | E | T | Q | P | G | - | - | 284.124 | 385.172 | 514.214 | 571.236 |
| y-18 | G | E | T | Q | P | G | - | - | - | 384.188 | 513.230 | 570.252 |
| | 6 | 5 | 4 | 3 | 2 | 1 | Gly | Pro | Gln | Thr | Glu | Gly |

**Transcription factor Sp2, BioTools-Score: 29**

| | A | D | T | K | P | G | Ala | Asp | Thr | Lys | Pro | Gly |
|---|---|---|---|---|---|---|---|---|---|---|---|---|
| Ion | 1 | 2 | 3 | 4 | 5 | 6 | 1 | 2 | 3 | 4 | 5 | 6 |
| b | A | D | T | K | P | G | 72.044 | 187.071 | 288.119 | 416.214 | 513.267 | 570.288 |
| b-17 | A | D | T | K | P | G | - | - | - | 399.187 | 496.240 | 553.262 |
| b-18 | A | D | T | K | P | G | - | 169.061 | 270.108 | 398.203 | 495.256 | 552.278 |
| y | A | D | T | K | P | G | 76.039 | 173.092 | 301.187 | 402.235 | 517.262 | 588.299 |
| y-17 | A | D | T | K | P | G | - | - | 284.160 | 385.208 | 500.235 | 571.272 |
| y-18 | A | D | T | K | P | G | - | - | - | 384.224 | 499.251 | 570.288 |
| | 6 | 5 | 4 | 3 | 2 | 1 | Gly | Pro | Lys | Thr | Asp | Ala |

**Nuclease-sensitive element-binding protein 1, BioTools-Score: 31**

| | V | S | T | K | P | G | Val | Ser | Thr | Lys | Pro | Gly |
|---|---|---|---|---|---|---|---|---|---|---|---|---|
| Ion | 1 | 2 | 3 | 4 | 5 | 6 | 1 | 2 | 3 | 4 | 5 | 6 |
| b | V | S | T | K | P | G | 100.076 | 187.108 | 288.155 | 416.250 | 513.303 | 570.325 |
| b-17 | V | S | T | K | P | G | - | - | - | 399.224 | 496.277 | 553.298 |
| b-18 | V | S | T | K | P | G | - | 169.097 | 270.145 | 398.240 | 495.293 | 552.314 |
| y | V | S | T | K | P | G | 76.039 | 173.092 | 301.187 | 402.235 | 489.267 | 588.335 |
| y-17 | V | S | T | K | P | G | - | - | 284.160 | 385.208 | 472.240 | 571.309 |
| y-18 | V | S | T | K | P | G | - | - | - | 384.224 | 471.256 | 570.325 |
| | 6 | 5 | 4 | 3 | 2 | 1 | Gly | Pro | Lys | Thr | Ser | Val |

**Elafin, BioTools-Score: 31**

| | E | G | T | Q | P | G | Glu | Gly | Thr | Gln | Pro | Gly |
|---|---|---|---|---|---|---|---|---|---|---|---|---|
| Ion | 1 | 2 | 3 | 4 | 5 | 6 | 1 | 2 | 3 | 4 | 5 | 6 |
| b | E | G | T | Q | P | G | 130.050 | 187.071 | 288.119 | 416.178 | 513.230 | 570.252 |
| b-17 | E | G | T | Q | P | G | - | - | - | 399.151 | 496.204 | 553.225 |
| b-18 | E | G | T | Q | P | G | 112.039 | 169.061 | 270.108 | 398.167 | 495.220 | 552.241 |
| y | E | G | T | Q | P | G | 76.039 | 173.092 | 301.151 | 402.198 | 459.220 | 588.262 |
| y-17 | E | G | T | Q | P | G | - | - | 284.124 | 385.172 | 442.193 | 571.236 |
| y-18 | E | G | T | Q | P | G | - | - | - | 384.188 | 441.209 | 570.252 |
| | 6 | 5 | 4 | 3 | 2 | 1 | Gly | Pro | Gln | Thr | Gly | Glu |

**Protocadherin-16, BioTools-Score: 44**

Most likely: **Protocadherin-16**, N-Glycans have already been described

$_{589}$EG**T**QPG$_{594}$

| Protein Score | Peptide Score | Peptide Sequence | Matching Sequence | Start AA | MS-Digest Index # | Protein MW (Da)/pI | Accession # | Species | Protein Name |
|---|---|---|---|---|---|---|---|---|---|
| 34 | 34 | GETQPG | (A)GETQPG(E) | 492 | 167152 | 64901/10.0 | Q02086 | HUMAN | Transcription factor Sp2 |
| 34 | 34 | GETQPG | (R)GETQPG(V) | 143 | 248229 | 18106/10.0 | Q6ZS92 | HUMAN | Putative uncharacterized protein FLJ45721 |
| 34 | 34 | EGTQPG | (P)EGTQPG(T) | 589 | 407896 | 346184/4.8 | Q96JQ0 | HUMAN | Protocadherin-16 |

# Fraction 14

621.73++ → Pep [M+H]+ 586.28+ [12.5-12.6 min] CID-MS Precursor

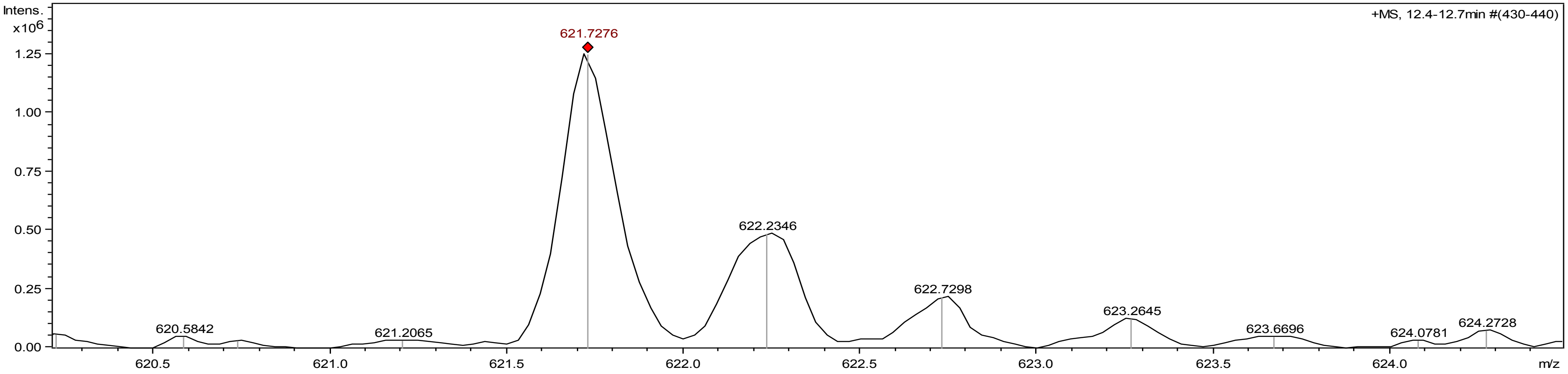


ETD spectrum not available

# Fraction 14

## 621.73++ → Pep [M+H]+ 586.28+ [12.5-12.6 min] CID-MS2

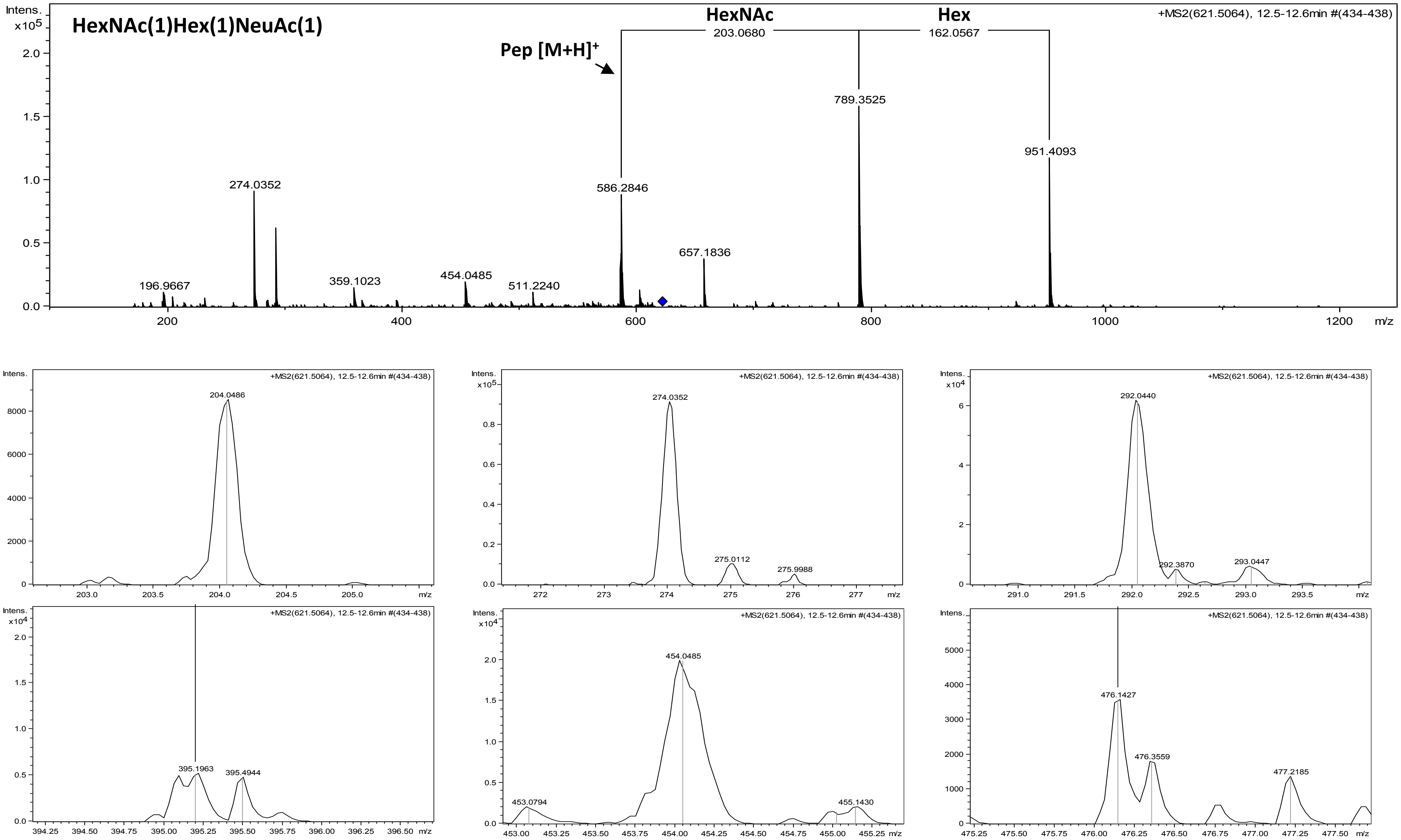

# Fraction 14

## 621.73++ → Pep [M+H]+ 586.28+ [12.5-12.6 min]

CID-MS2

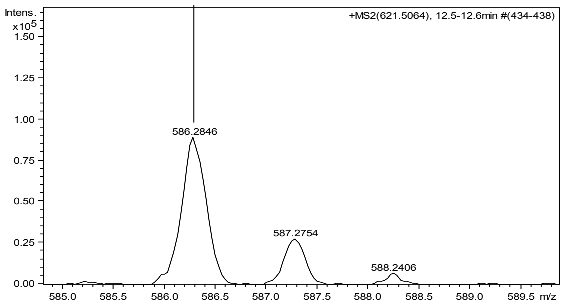


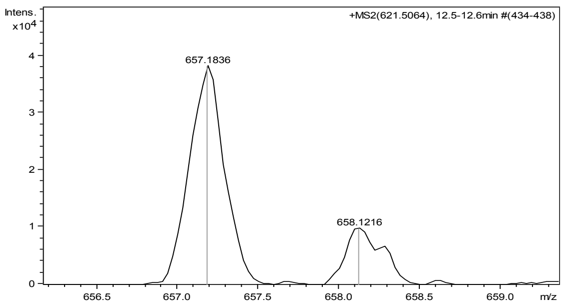


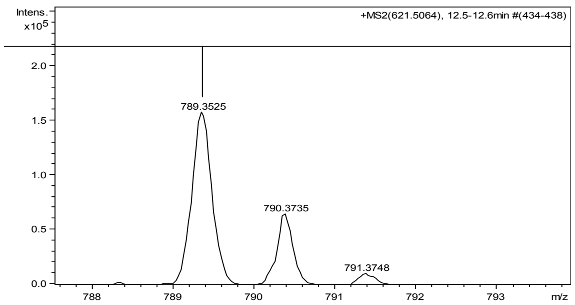


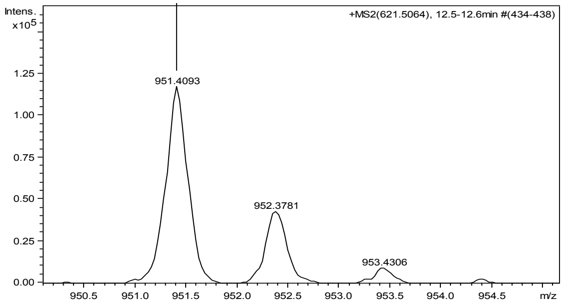

# Fraction 14

621.73++ → Pep [M+H]+ 586.28+ [12.5-12.6 min]

CID-MS3 MASCOT Search

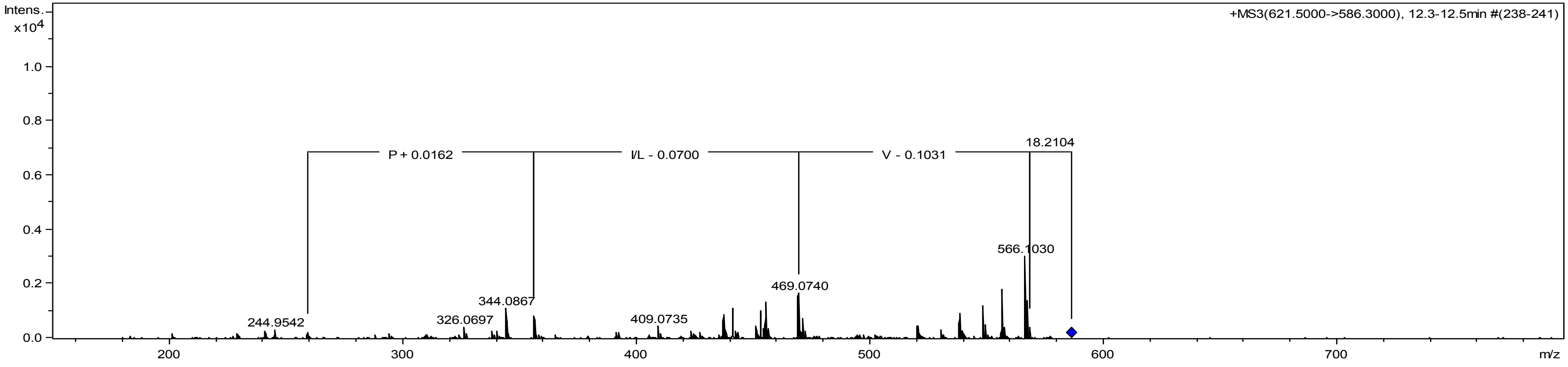


## MASCOT Search results

| prot_hit_nur | prot_acc | prot_desc | prot_score | prot_mass | prot_matche | pep_query | pep_rank | pep_isbold | pep_exp_mz | pep_exp_mr | pep_exp_z | pep_calc_mr | pep_delta | pep_miss | pep_score | pep_expect | pep_res_bef | pep_seq |
|---|---|---|---|---|---|---|---|---|---|---|---|---|---|---|---|---|---|---|
| 1 | BASI_HUMAI | Basigin precu | 6 | 42573 | 1 | 1 | 2 | 1 | 586,2846 | 585,2773 | 1 | 584,2806 | 0,9967 | 0 | 10,42 | 8,60E+02 | V | GSPVPE |
| 2 | TIAF1_HUMA | TGFB1-induc | 6 | 12691 | 1 | 1 | 5 | 0 | 586,2846 | 585,2773 | 1 | 584,3534 | 0,924 | 0 | 8,19 | 1,40E+03 | R | GSPVLL |
| 3 | PLSB_HUMAI | Glycerol-3-p | 6 | 94762 | 1 | 1 | 2 | 0 | 586,2846 | 585,2773 | 1 | 584,2806 | 0,9967 | 0 | 10,42 | 8,60E+02 | F | SGPVPE |
| 4 | K0329_HUM/ | Uncharacteri | 5 | 155761 | 1 | 1 | 2 | 0 | 586,2846 | 585,2773 | 1 | 584,2806 | 0,9967 | 0 | 10,42 | 8,60E+02 | E | GSPVEP |
| 5 | PP1RA_HUM | Serine/threc | 5 | 99338 | 1 | 1 | 1 | 0 | 586,2846 | 585,2773 | 1 | 584,2554 | 1,0219 | 0 | 10,53 | 8,40E+02 | R | SGGGPPN |
| 6 | ARFP1_HUM | Arfaptin-1 (A | 4 | 41770 | 1 | 1 | 5 | 0 | 586,2846 | 585,2773 | 1 | 584,3534 | 0,924 | 0 | 8,19 | 1,40E+03 | K | SGPVIL |
| 7 | GLTL1_HUM/ | Putative poly | 4 | 63776 | 1 | 1 | 5 | 0 | 586,2846 | 585,2773 | 1 | 584,3534 | 0,924 | 0 | 8,19 | 1,40E+03 | P | GSPVIL |
| 8 | ULK2_HUMA | Serine/threc | 2 | 114308 | 1 | 1 | 5 | 0 | 586,2846 | 585,2773 | 1 | 584,2806 | 0,9967 | 0 | 8,19 | 1,40E+03 | S | GSPVPQ |
| 9 | CXXC6_HUM | CXXC-type zi | 1 | 237956 | 1 | 1 | 5 | 0 | 586,2846 | 585,2773 | 1 | 584,2806 | 0,9967 | 0 | 8,19 | 1,40E+03 | D | GSPVQP |
| 10 | TARA_HUMA | TRIO and F-a | 1 | 264125 | 1 | 1 | 5 | 0 | 586,2846 | 585,2773 | 1 | 584,3534 | 0,924 | 0 | 8,19 | 1,40E+03 | H | GSPVLI |

unambiguous identification not possible

# Fraction 14

684.73++ → Pep+HexNAc [M+H]+ 915.43+ [14.4-14.6 min]

CID-MS Precursor

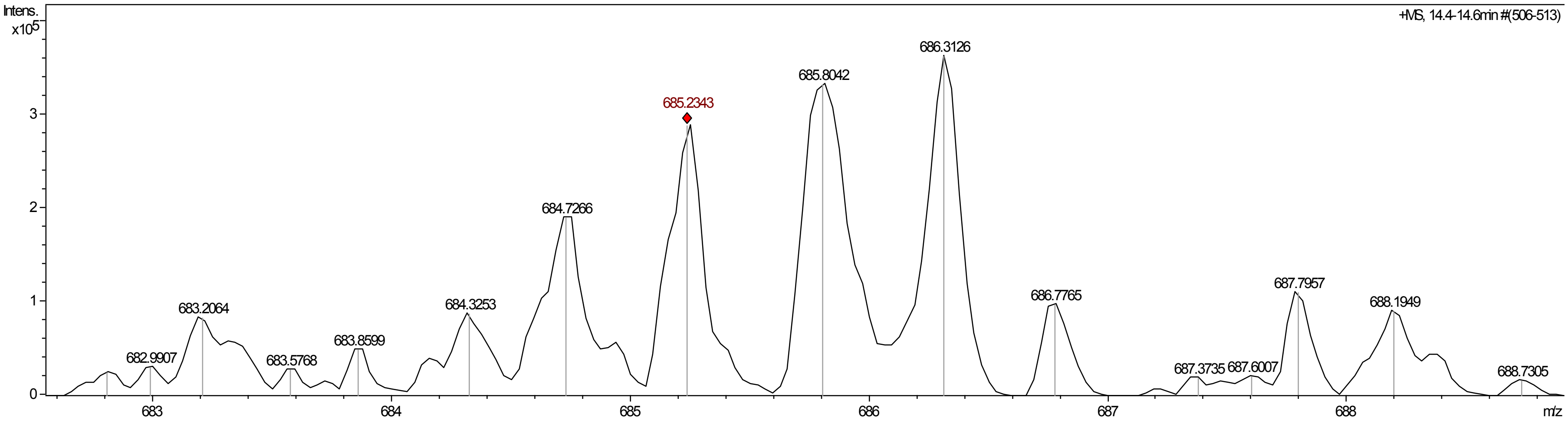


ETD spectrum of poor quality

# Fraction 14

684.73++ → Pep+HexNAc [M+H]+ 915.43+ [14.4-14.6 min]

CID-MS2

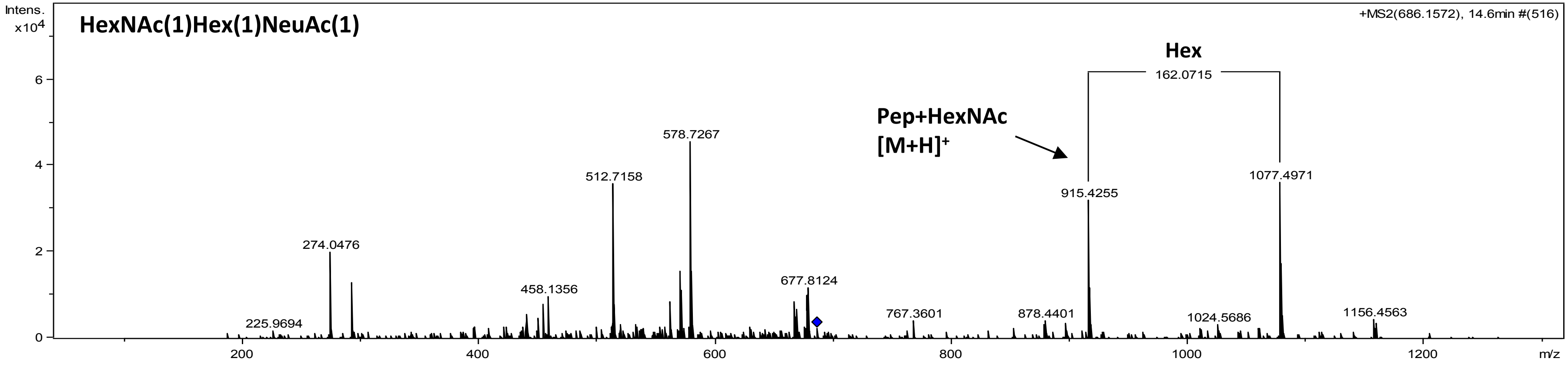


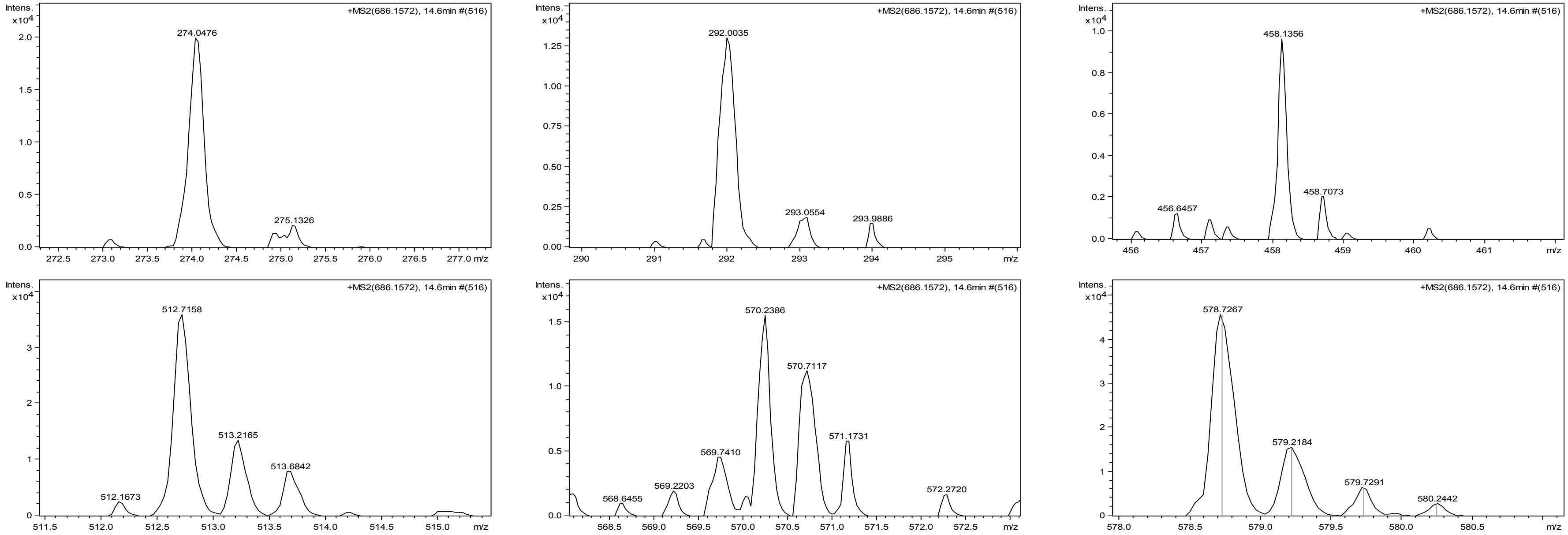

# Fraction 14

684.73++ → Pep+HexNAc [M+H]+ 915.43+ [14.4-14.6 min]

CID-MS2

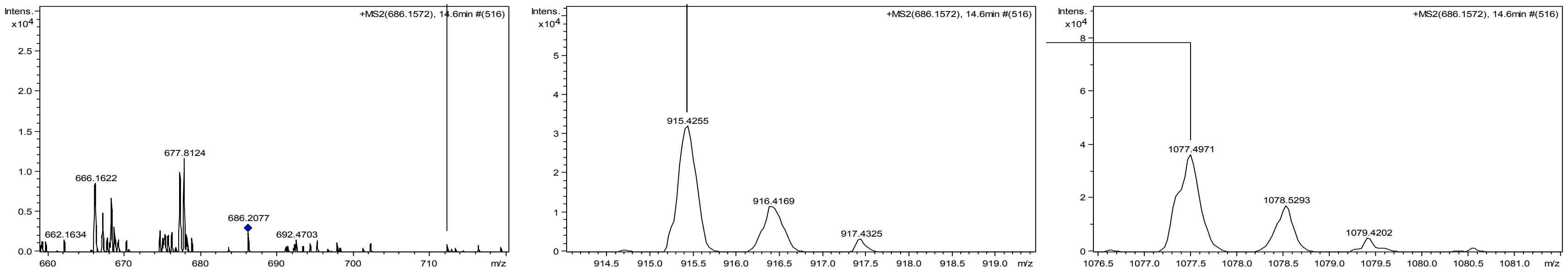

# Fraction 14

684.73++ → Pep+HexNAc [M+H]+ 915.43+ [14.4-14.6 min]

CID-MS3 Manual DeNovo

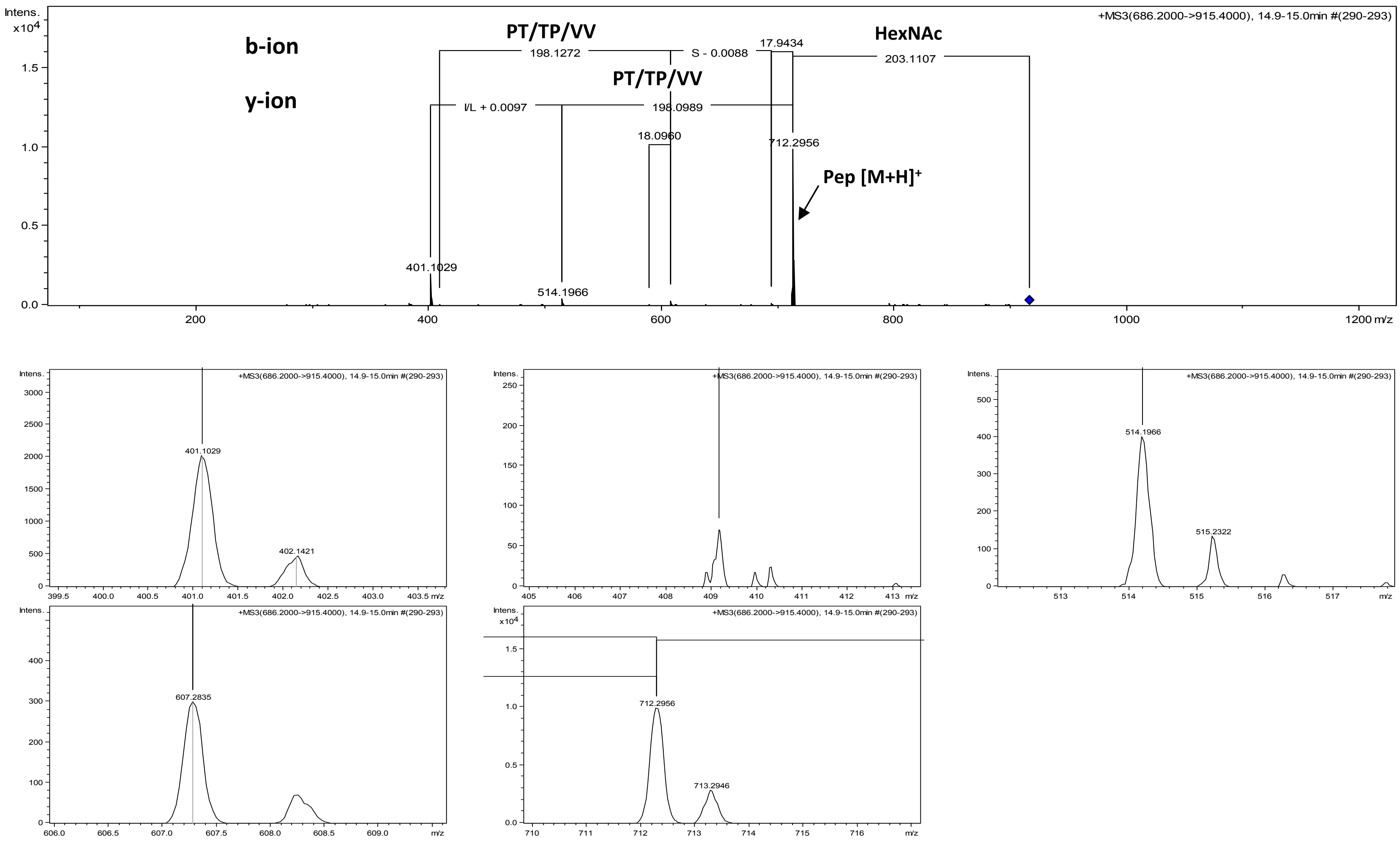

# Fraction 14

684.73++ → Pep+HexNAc [M+H]+ 915.43+ [14.4-14.6 min]

CID-MS3 MASCOT Search

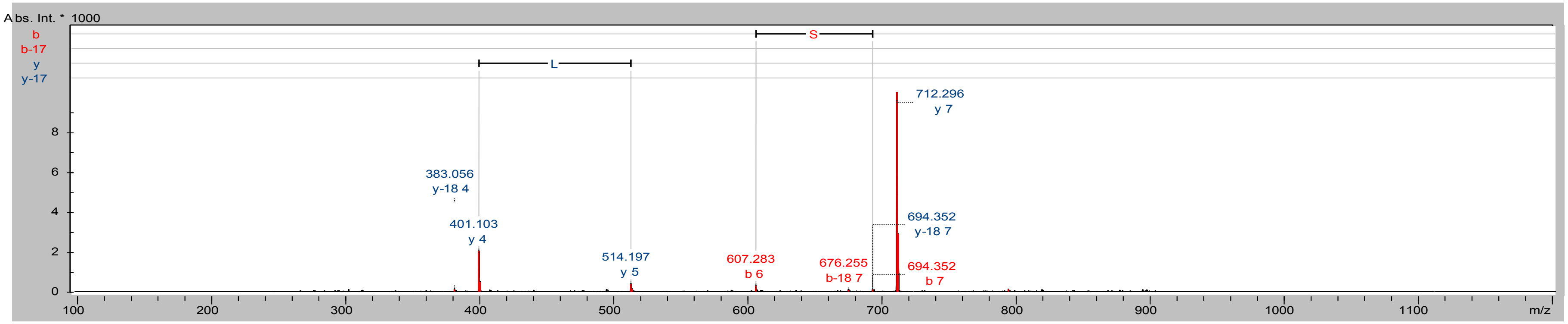


| | T | P | L | P | P | T | S | Thr | Pro | Leu | Pro | Pro | Thr | Ser |
|---|---|---|---|---|---|---|---|---|---|---|---|---|---|---|
| Ion | 1 | 2 | 3 | 4 | 5 | 6 | 7 | 1 | 2 | 3 | 4 | 5 | 6 | 7 |
| b | T | P | L | P | P | T | S | 102.055 | 199.108 | 312.192 | 409.245 | 506.297 | 607.345 | 694.377 |
| b-17 | T | P | L | P | P | T | S | - | - | - | - | - | - | - |
| b-18 | T | P | L | P | P | T | S | 84.044 | 181.097 | 294.181 | 391.234 | 488.287 | 589.334 | 676.366 |
| y | T | P | L | P | P | T | S | 106.050 | 207.098 | 304.150 | 401.203 | 514.287 | 611.340 | 712.388 |
| y-17 | T | P | L | P | P | T | S | - | - | - | - | - | - | - |
| y-18 | T | P | L | P | P | T | S | 88.039 | 189.087 | 286.140 | 383.193 | 496.277 | 593.329 | 694.377 |
| | 7 | 6 | 5 | 4 | 3 | 2 | 1 | Ser | Thr | Pro | Pro | Leu | Pro | Thr |

known O-glycosylation region

Hemopexin precursor (Beta-1B-glycoprotein)

$_{24}$TPLPP**TS**$_{30}$

# Fraction 14

684.73++ → Pep+HexNAc [M+H]+ 915.43+ [14.4-14.6 min]

CID-MS3 MASCOT Search

| prot_hit_nur | prot_acc | prot_desc | prot_score | prot_mass | prot_matche | pep_query | pep_rank | pep_isbold | pep_exp_mz | pep_exp_mr | pep_exp_z | pep_calc_mr | pep_delta | pep_miss | pep_score | pep_expect | pep_res_bef | pep_seq |
|---|---|---|---|---|---|---|---|---|---|---|---|---|---|---|---|---|---|---|
| 1 | AMPH_HUM | Amphiphysir | 15 | 76381 | 1 | 1 | 1 | 1 | 712.2813 | 711.274 | 1 | 711.4531 | -0.1791 | 0 | 18.78 | 1.30E+02 | D | VVLVVPS |
| 2 | HEMO_HUM | Hemopexin | 14 | 52385 | 1 | 1 | 1 | 0 | 712.2813 | 711.274 | 1 | 711.3803 | -0.1063 | 0 | 18.78 | 1.30E+02 | A | TPLPPTS |
| 3 | TAF10_HUM | Transcriptior | 14 | 21812 | 1 | 1 | 4 | 0 | 712.2813 | 711.274 | 1 | 711.3803 | -0.1063 | 0 | 14.97 | 3.10E+02 | T | PTIPDAV |
| 4 | PART1_HUM | Prostate-spe | 13 | 6756 | 1 | 1 | 4 | 0 | 712.2813 | 711.274 | 1 | 711.4531 | -0.1791 | 0 | 14.97 | 3.10E+02 | T | PTIGLVL |
| 5 | PRP16_HUM | Pre-mRNA-s | 12 | 141270 | 1 | 1 | 1 | 0 | 712.2813 | 711.274 | 1 | 711.3803 | -0.1063 | 0 | 18.78 | 1.30E+02 | D | TPLPTPS |
| 6 | RMP_HUMA | RNA polyme | 10 | 57185 | 1 | 1 | 4 | 0 | 712.2813 | 711.274 | 1 | 711.3915 | -0.1175 | 0 | 14.97 | 3.10E+02 | L | PTIPER |
| 7 | 1C17_HUMA | HLA class I hi | 10 | 41612 | 1 | 1 | 4 | 0 | 712.2813 | 711.274 | 1 | 711.3803 | -0.1063 | 0 | 14.97 | 3.10E+02 | Q | PTIPNLG |
| 8 | ULK1_HUMA | Serine/threc | 9 | 113557 | 1 | 1 | 4 | 0 | 712.2813 | 711.274 | 1 | 711.3915 | -0.1175 | 0 | 14.97 | 3.10E+02 | V | PTIPRE |
| 9 | PLXA1_HUM | Plexin-A1 pr | 8 | 214089 | 1 | 1 | 4 | 0 | 712.2813 | 711.274 | 1 | 711.4643 | -0.1903 | 0 | 14.97 | 3.10E+02 | D | PTILRI |
| 10 | CAC1I_HUM | Voltage-dep | 8 | 248310 | 1 | 1 | 4 | 0 | 712.2813 | 711.274 | 1 | 711.4643 | -0.1903 | 0 | 14.97 | 3.10E+02 | N | PTIIRI |

Biotools-Score: 8

MASCOT-Score: 19

known O-glycosylation region

Hemopexin precursor (Beta-1B-glycoprotein)

$_{24}$**T**PLPP**TS**$_{30}$

# Fraction 14

671.26++ → Pep [M+H]+ 685.35+ [15.3-15.7 min] CID-MS Precursor

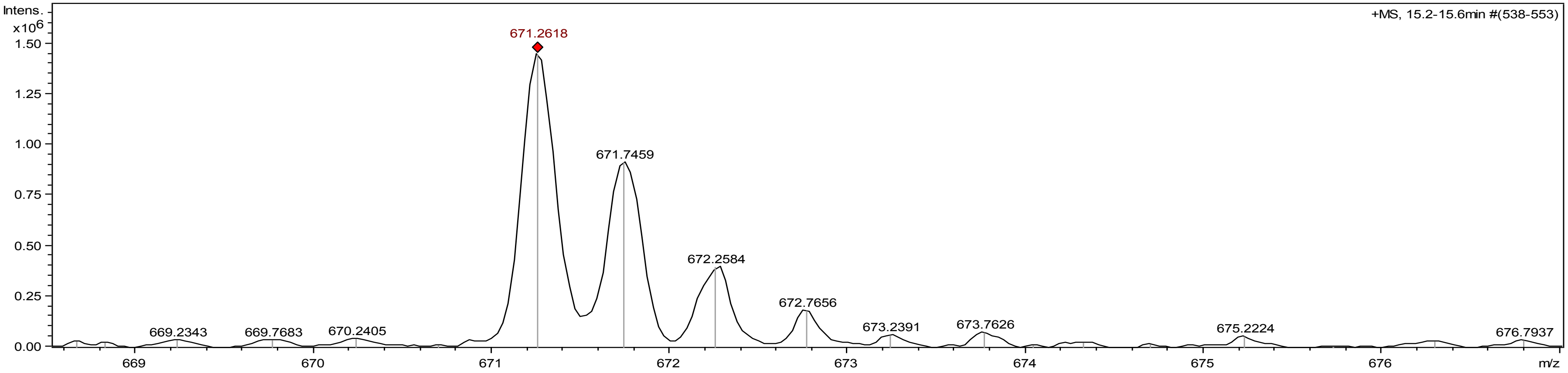


ETD spectrum of poor quality

# Fraction 14

671.26++ → Pep [M+H]+ 685.35+ [15.3-15.7 min] CID-MS2

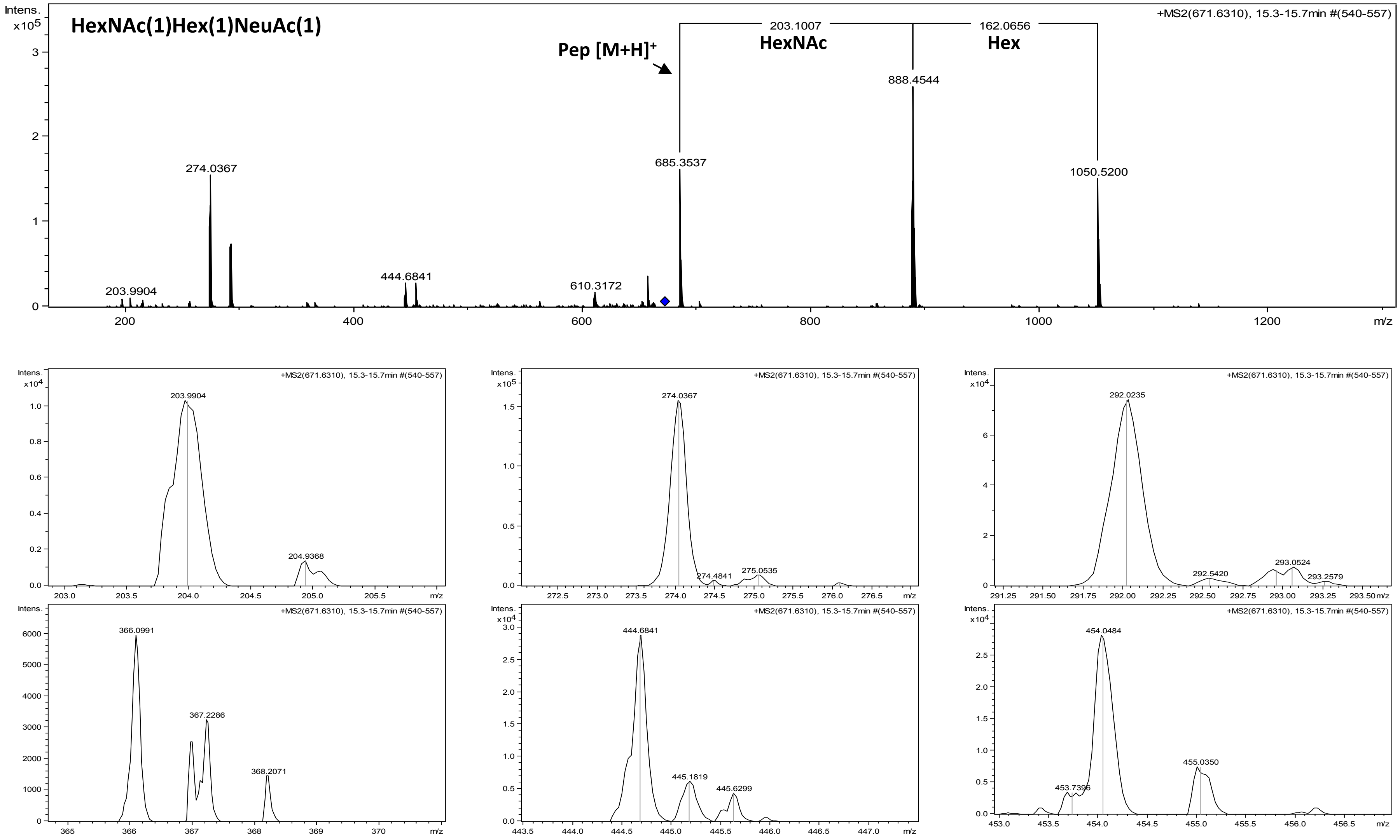

# Fraction 14

## 671.26++ → Pep [M+H]+ 685.35+ [15.3-15.7 min]

CID-MS2

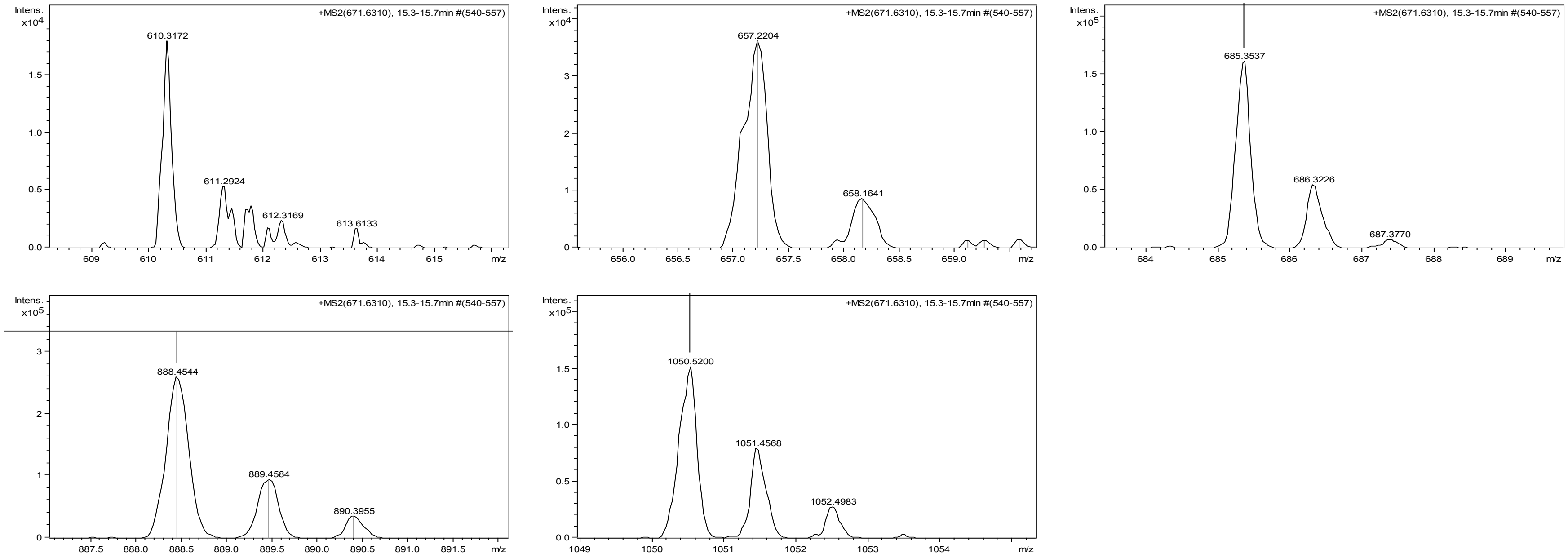

# Fraction 14

671.26++ → Pep [M+H]+ 685.35+ [15.3-15.7 min]

CID-MS3 MASCOT Search

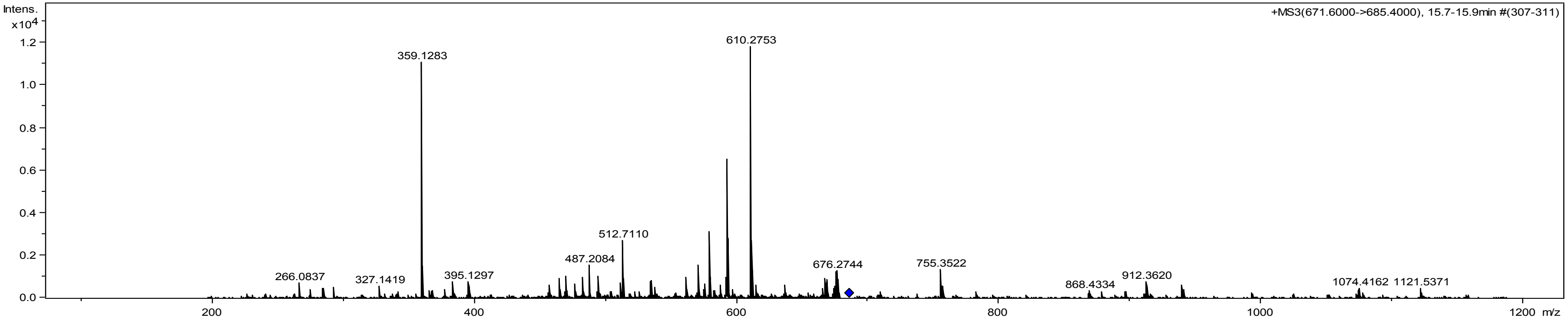


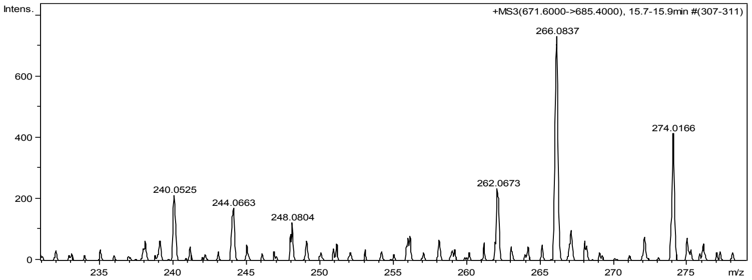


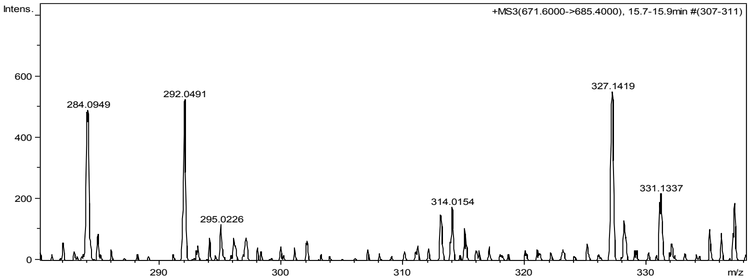


known O-glycosylation site

Alpha-2-HS-glycoprotein precursor

$_{342}$VVQPSVG$_{348}$

# Fraction 14

671.26++ → Pep [M+H]+ 685.35+ [15.3-15.7 min]

CID-MS3 MASCOT Search

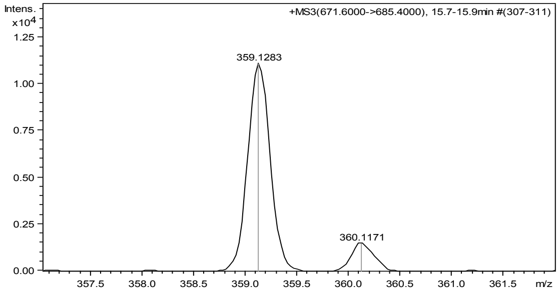


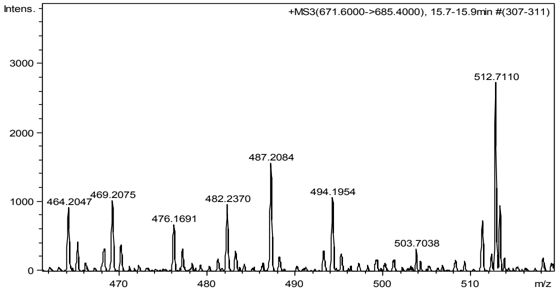


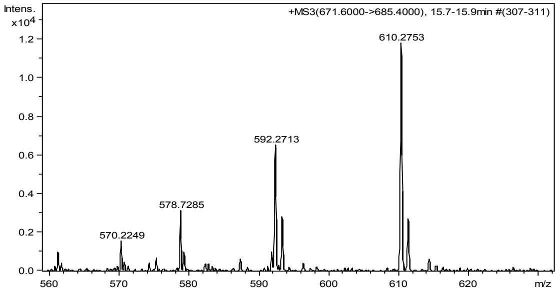


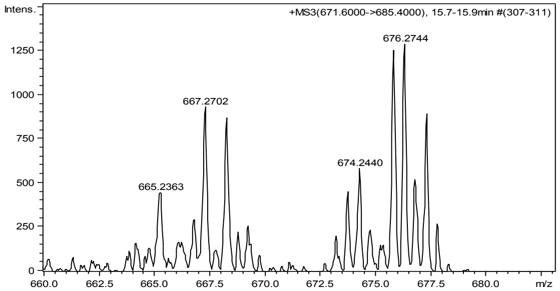


known O-glycosylation site

Alpha-2-HS-glycoprotein precursor

$_{342}$VVQP**S**VG$_{348}$

# Fraction 14

671.26++ → Pep [M+H]+ 685.35+ [15.3-15.7 min] CID-MS3 MASCOT Search

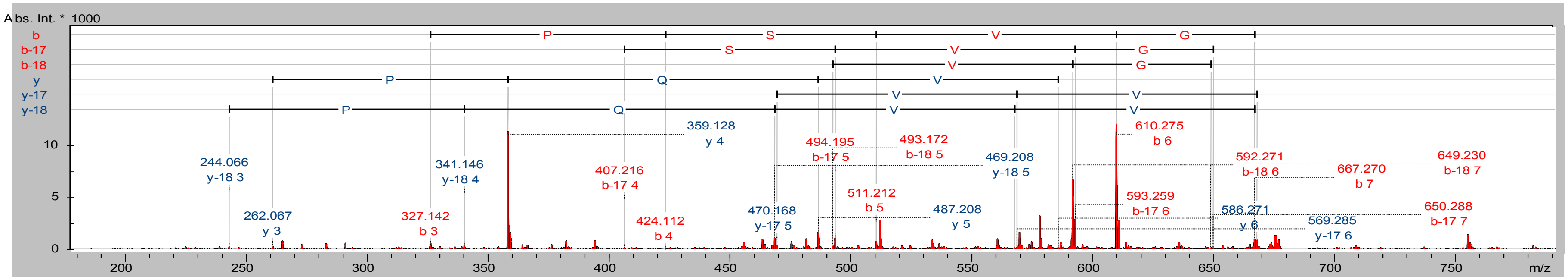


| | V | V | Q | P | S | V | G | Val | Val | Gln | Pro | Ser | Val | Gly |
|---|---|---|---|---|---|---|---|---|---|---|---|---|---|---|
| Ion | 1 | 2 | 3 | 4 | 5 | 6 | 7 | 1 | 2 | 3 | 4 | 5 | 6 | 7 |
| b | V | V | Q | P | S | V | G | 100.076 | 199.144 | 327.203 | 424.255 | 511.287 | 610.356 | 667.377 |
| b-17 | V | V | Q | P | S | V | G | - | - | 310.176 | 407.229 | 494.261 | 593.329 | 650.351 |
| b-18 | V | V | Q | P | S | V | G | - | - | - | - | 493.277 | 592.345 | 649.367 |
| y | V | V | Q | P | S | V | G | 76.039 | 175.108 | 262.140 | 359.193 | 487.251 | 586.320 | 685.388 |
| y-17 | V | V | Q | P | S | V | G | - | - | - | - | 470.225 | 569.293 | 668.361 |
| y-18 | V | V | Q | P | S | V | G | - | - | 244.129 | 341.182 | 469.241 | 568.309 | 667.377 |
| | 7 | 6 | 5 | 4 | 3 | 2 | 1 | Gly | Val | Ser | Pro | Gln | Val | Val |

known O-glycosylation site

Alpha-2-HS-glycoprotein precursor

$_{342}$VVQP**S**VG$_{348}$

# Fraction 14

671.26++ → Pep [M+H]+ 685.35+ [15.3-15.7 min]

CID-MS3 MASCOT Search

| prot_hit_nur | prot_acc | prot_desc | prot_score | prot_mass | prot_matche | pep_query | pep_rank | pep_isbold | pep_exp_mz | pep_exp_mr | pep_exp_z | pep_calc_mr | pep_delta | pep_miss | pep_score | pep_expect | pep_res_bef | pep_seq |
|---|---|---|---|---|---|---|---|---|---|---|---|---|---|---|---|---|---|---|
| 1 | FETUA_HUM | Alpha-2-HS-g | 9 | 40098 | 1 | 1 | 3 | 1 | 685.3537 | 684.3464 | 1 | 684.3807 | -0.0342 | 0 | 12.59 | 5.50E+02 | T | VVQPSVG |
| 2 | CHODL_HUM | Chondrolect | 9 | 31095 | 1 | 1 | 6 | 0 | 685.3537 | 684.3464 | 1 | 684.3079 | 0.0386 | 0 | 11.49 | 7.00E+02 | H | QPTANPG |
| 3 | ARHG2_HUM | Rho/Rac gua | 9 | 102080 | 1 | 1 | 1 | 0 | 685.3537 | 684.3464 | 1 | 684.3443 | 0.0022 | 0 | 13.87 | 4.10E+02 | L | EPGVVQG |
| 4 | LRBA_HUMA | Lipopolysacc | 6 | 321639 | 1 | 1 | 1 | 0 | 685.3537 | 684.3464 | 1 | 684.417 | -0.0706 | 0 | 13.87 | 4.10E+02 | A | PSVVVKG |
| 5 | NCOR1_HUM | Nuclear rece | 5 | 270957 | 1 | 1 | 3 | 0 | 685.3537 | 684.3464 | 1 | 684.3806 | -0.0342 | 0 | 12.59 | 5.50E+02 | E | TPKPSVG |
| 6 | FUS_HUMAN | RNA-binding | 5 | 53622 | 1 | 1 | 8 | 0 | 685.3537 | 684.3464 | 1 | 684.3079 | 0.0385 | 0 | 10.75 | 8.40E+02 | Y | PTQPGQG |
| 7 | SC23A_HUM | Protein trans | 4 | 87004 | 1 | 1 | 7 | 0 | 685.3537 | 684.3464 | 1 | 684.3079 | 0.0385 | 0 | 10.83 | 8.20E+02 | G | GPATQGPG |
| 8 | PCNT_HUMA | Pericentrin ( | 4 | 380644 | 1 | 1 | 5 | 0 | 685.3537 | 684.3464 | 1 | 684.3079 | 0.0385 | 0 | 11.67 | 6.80E+02 | L | PQTQGPG |
| 9 | BAZ2B_HUM | Bromodoma | 3 | 222285 | 1 | 1 | 8 | 0 | 685.3537 | 684.3464 | 1 | 684.3442 | 0.0022 | 0 | 10.75 | 8.40E+02 | M | TPKPNAG |
| 10 | CO5A3_HUM | Collagen alp | 3 | 172631 | 1 | 1 | 10 | 0 | 685.3537 | 684.3464 | 1 | 684.3079 | 0.0385 | 0 | 10.7 | 8.50E+02 | Q | PGATGQPG |

Biotools-Score: 44

MASCOT-Score: 13

known O-glycosylation site

Alpha-2-HS-glycoprotein precursor

$_{342}$VVQP**S**VG$_{348}$

# Fraction 14

706.77++ → Pep [M+H]+ 756.37+ [16.3-16.4 min] CID-MS Precursor

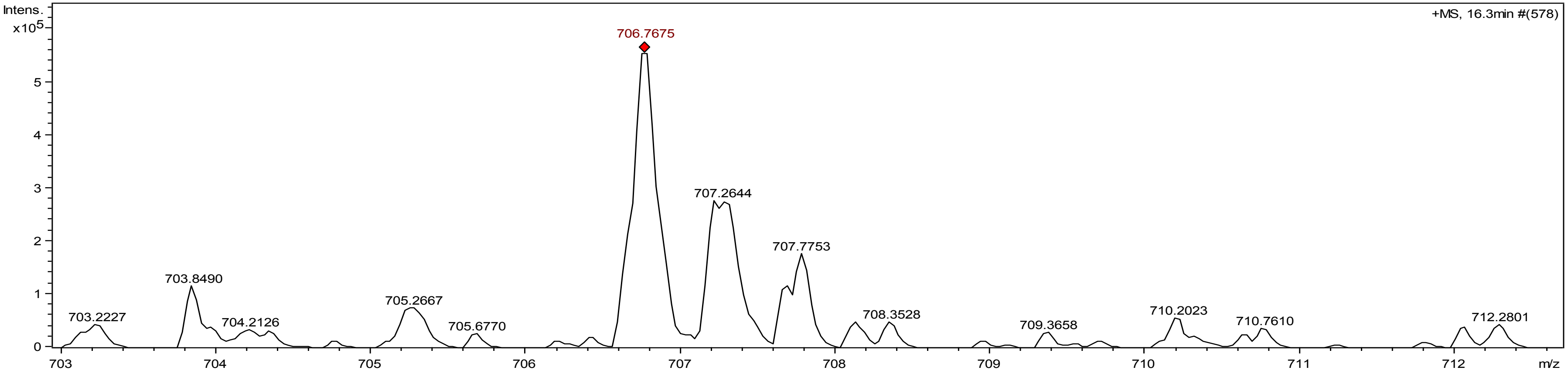


ETD spectrum of poor quality

# Fraction 14

706.77++ → Pep [M+H]+ 756.37+ [16.3-16.4 min] CID-MS2

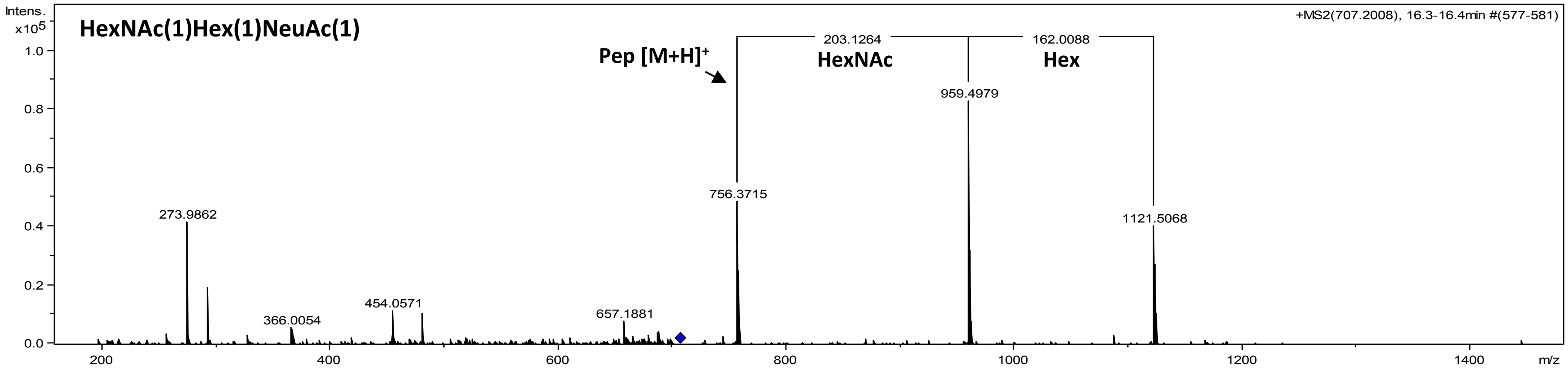


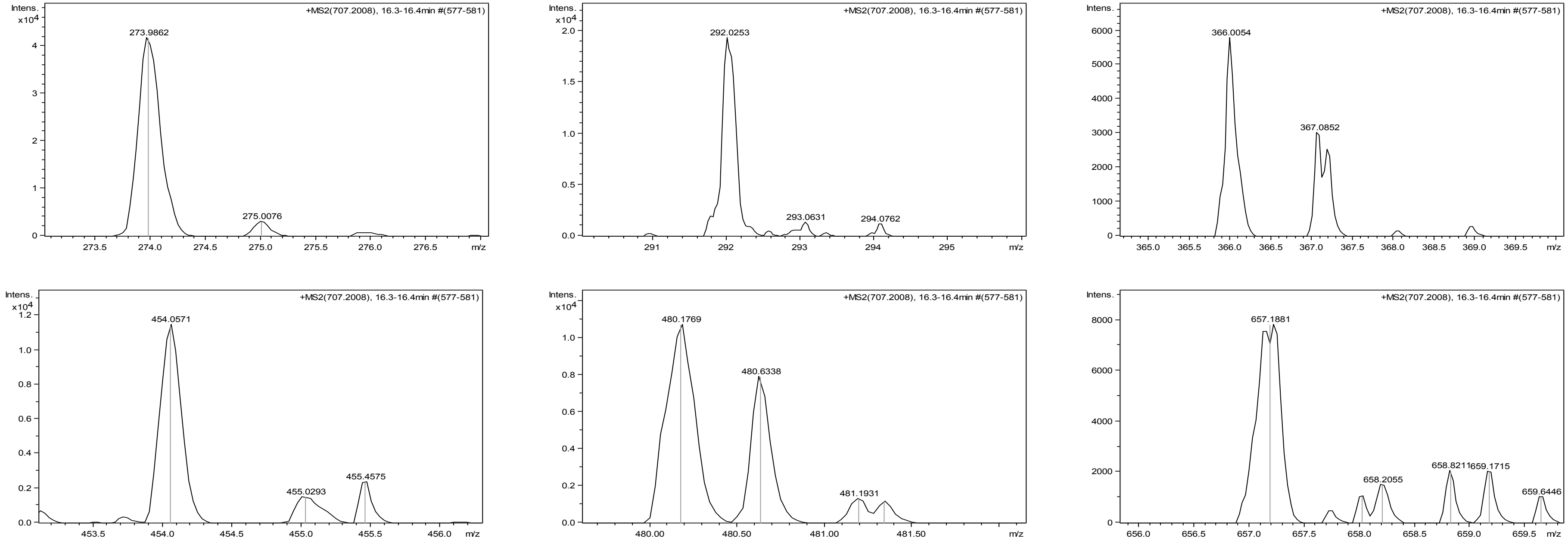

# Fraction 14

706.77++ → Pep [M+H]+ 756.37+ [16.3-16.4 min] CID-MS2

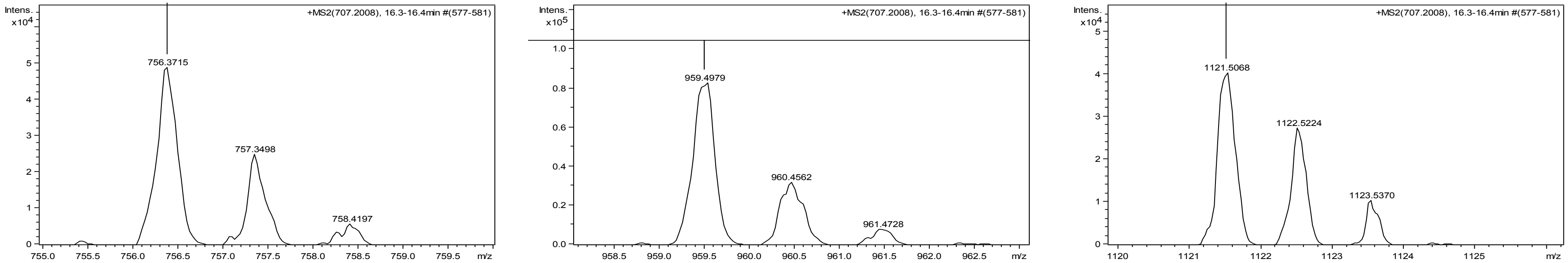

# Fraction 14

706.77++ → Pep [M+H]+ 756.37+ [16.3-16.4 min]    CID-MS3    Manual DeNovo

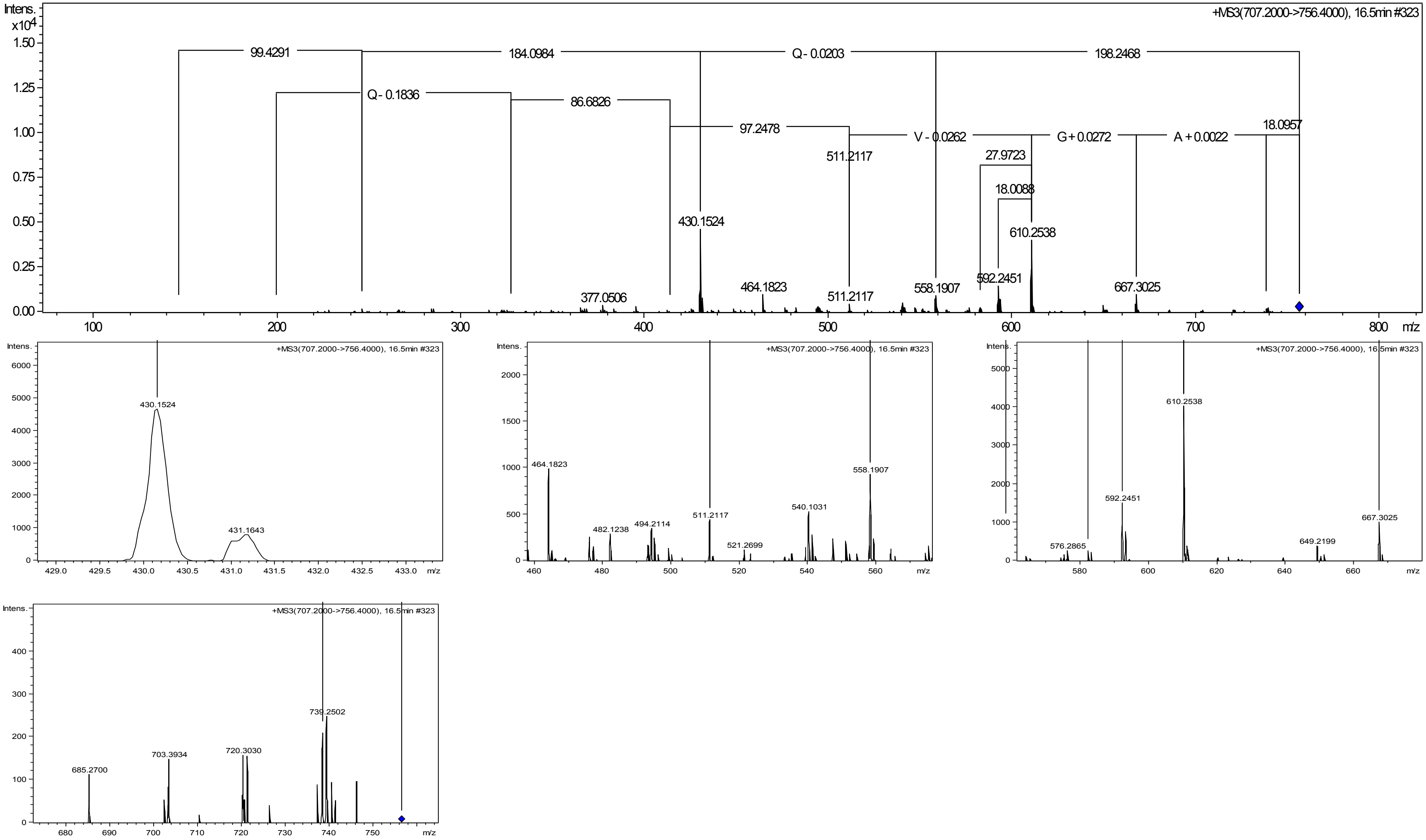

# Fraction 14

706.77++ → Pep [M+H]+ 756.37+ [16.3-16.4 min]    CID-MS3    MS-Homology Search

UCSF University of California, San Francisco | About UCSF | Search UCSF | UCSF Medical Center

Home | MS-Fit | MS-Tag | MS-Seq | MS-Pattern | MS-Bridge | MS-Digest | MS-Product | MS-Comp | DB-Stat | MS-Isotope | MS-Homology

## MS-Homology Search Results

Search completed. 3 sec elapsed. 0 sec remaining.

[–] **Parameters**

Database searched: **SwissProt.2012.12.3**
Digest Used: **No enzyme**
Max. # Missed Cleavages: **1**
Min matches: **1**
Score matrix: **BLOSUM62**
List of Sequences: **1 [VV|{PT}][K|Q][{LA}|{SP}]VGA 0**
Mass Tolerance: **0.5 Da**

[–] **Pre Search Results (SwissProt.2012.12.3)**

Number of entries in the database: **453850**
Full Molecular Weight range: **453850** entries.
Full pI range: **453850** entries.
Taxonomy search **HOMO SAPIENS** selects **20479** entries.
Pre searches select **20479** entries.

MS-Homology search selects **1** entry.

Number of Peptide Hits: **1**

| Protein Score | Peptide Score | Peptide Sequence | Matching Sequence | Start AA | MS-Digest Index # | Protein MW (Da)/pI | Accession # | Species | Protein Name |
|---|---|---|---|---|---|---|---|---|---|
| 38 | 38 | VVQPSVGA | (T)VVQPSVGA(A) | 342 | 69033 | 39325/5.4 | P02765 | HUMAN | Alpha-2-HS-glycoprotein |

known O-glycosylation site

Alpha-2-HS-glycoprotein precursor

$_{342}$VVQP**S**VGA$_{349}$

# Fraction 14

706.77++ → Pep [M+H]+ 756.37+ [16.3-16.4 min]

CID-MS3 MASCOT Search

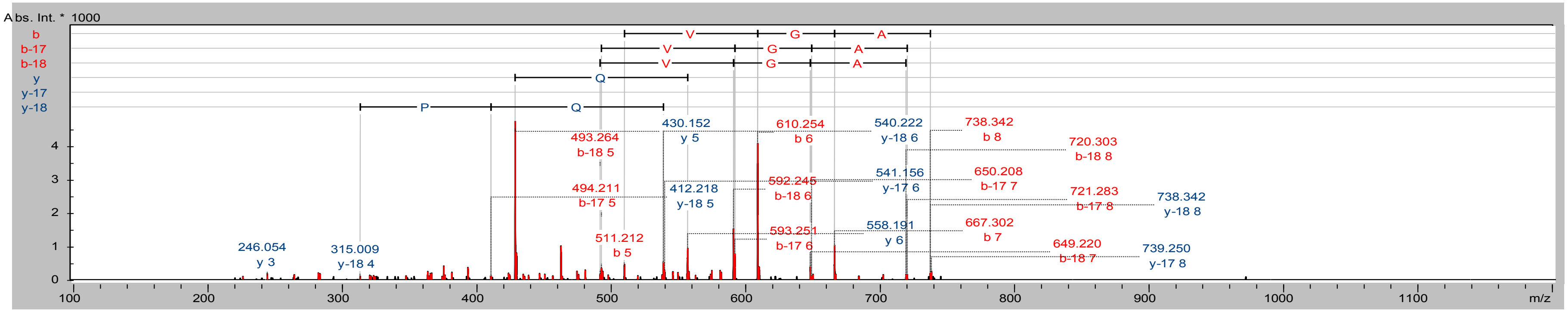


| | V | V | Q | P | S | V | G | A | Val | Val | Gln | Pro | Ser | Val | Gly | Ala |
|---|---|---|---|---|---|---|---|---|---|---|---|---|---|---|---|---|
| Ion | 1 | 2 | 3 | 4 | 5 | 6 | 7 | 8 | 1 | 2 | 3 | 4 | 5 | 6 | 7 | 8 |
| b | V | V | Q | P | S | V | G | A | 100.076 | 199.144 | 327.203 | 424.255 | 511.287 | 610.356 | 667.377 | 738.414 |
| b-17 | V | V | Q | P | S | V | G | A | - | - | 310.176 | 407.229 | 494.261 | 593.329 | 650.351 | 721.388 |
| b-18 | V | V | Q | P | S | V | G | A | - | - | - | - | 493.277 | 592.345 | 649.367 | 720.404 |
| y | V | V | Q | P | S | V | G | A | 90.055 | 147.076 | 246.145 | 333.177 | 430.230 | 558.288 | 657.357 | 756.425 |
| y-17 | V | V | Q | P | S | V | G | A | - | - | - | - | - | 541.262 | 640.330 | 739.398 |
| y-18 | V | V | Q | P | S | V | G | A | - | - | - | 315.166 | 412.219 | 540.278 | 639.346 | 738.414 |
| | 8 | 7 | 6 | 5 | 4 | 3 | 2 | 1 | Ala | Gly | Val | Ser | Pro | Gln | Val | Val |

known O-glycosylation site

Alpha-2-HS-glycoprotein precursor

$_{342}$VVQP**S**VGA$_{349}$

# Fraction 14

706.77++ → Pep [M+H]+ 756.37+ [16.3-16.4 min]

CID-MS3 MASCOT Search

| prot_hit_nur | prot_acc | prot_desc | prot_score | prot_mass | prot_matche | pep_query | pep_rank | pep_isbold | pep_exp_mz | pep_exp_mr | pep_exp_z | pep_calc_mr | pep_delta | pep_miss | pep_score | pep_expect | pep_res_bef | pep_seq |
|---|---|---|---|---|---|---|---|---|---|---|---|---|---|---|---|---|---|---|
| 1 | SPHK1_HUM. | Sphingosine | 14 | 42889 | 1 | 1 | 3 | 1 | 756.3715 | 755.3642 | 1 | 755.4178 | -0.0535 | 0 | 17.04 | 2.10E+02 | A | SPVVVQQ |
| 2 | FETUA_HUM. | Alpha-2-HS-g | 14 | 40098 | 1 | 1 | 2 | 0 | 756.3715 | 755.3642 | 1 | 755.4178 | -0.0535 | 0 | 18.99 | 1.30E+02 | T | VVQPSVGA |
| 3 | LAMA5_HUM | Laminin subu | 13 | 411997 | 1 | 1 | 1 | 0 | 756.3715 | 755.3642 | 1 | 755.3198 | 0.0444 | 0 | 21.37 | 77 | C | QHNTEGA |
| 4 | CBX7_HUMA | Chromobox p | 12 | 28437 | 1 | 1 | 9 | 0 | 756.3715 | 755.3642 | 1 | 755.4178 | -0.0535 | 0 | 15.82 | 2.80E+02 | S | PEGVVKAG |
| 5 | DHB4_HUMA | Peroxisomal | 12 | 80092 | 1 | 1 | 3 | 0 | 756.3715 | 755.3642 | 1 | 755.4541 | -0.0899 | 0 | 17.04 | 2.10E+02 | I | GPEVVKK |
| 6 | PO3F2_HUM. | POU domain | 11 | 47092 | 1 | 1 | 3 | 0 | 756.3715 | 755.3642 | 1 | 755.4178 | -0.0535 | 0 | 17.04 | 2.10E+02 | K | PSVVVQQ |
| 7 | MKL1_HUMA | MKL/myocar | 11 | 99257 | 1 | 1 | 3 | 0 | 756.3715 | 755.3642 | 1 | 755.4541 | -0.0899 | 0 | 17.04 | 2.10E+02 | P | PSVVVKQ |
| 8 | RBM15_HUM | Putative RNA | 11 | 107352 | 1 | 1 | 8 | 0 | 756.3715 | 755.3642 | 1 | 755.3926 | -0.0284 | 0 | 16.27 | 2.50E+02 | L | EPRVGAGA |
| 9 | RAPH1_HUM | Ras-associate | 10 | 141762 | 1 | 1 | 3 | 0 | 756.3715 | 755.3642 | 1 | 755.4541 | -0.0899 | 0 | 17.04 | 2.10E+02 | P | VPSVVKQ |
| 10 | SRC8_HUMA | Src substrate | 10 | 61770 | 1 | 1 | 10 | 0 | 756.3715 | 755.3642 | 1 | 755.3926 | -0.0284 | 0 | 15.67 | 2.90E+02 | E | KHESQK |

Biotools-Score: 18

MASCOT-Score: 19

known O-glycosylation site

Alpha-2-HS-glycoprotein precursor

$_{342}$VVQP**S**VGA$_{349}$

# Fraction 14

664.70++ → Pep [M+H]+ 672.33+ [16.6-16.7 min]    CID-MS Precursor

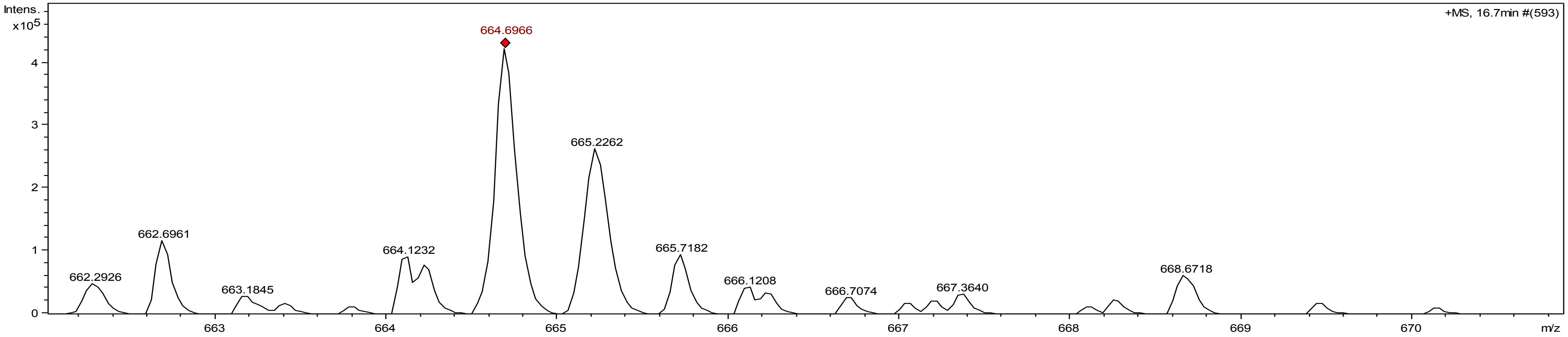


ETD spectrum with poor quality

# Fraction 14

## 664.70++ → Pep [M+H]+ 672.33+ [16.6-16.7 min] CID-MS2

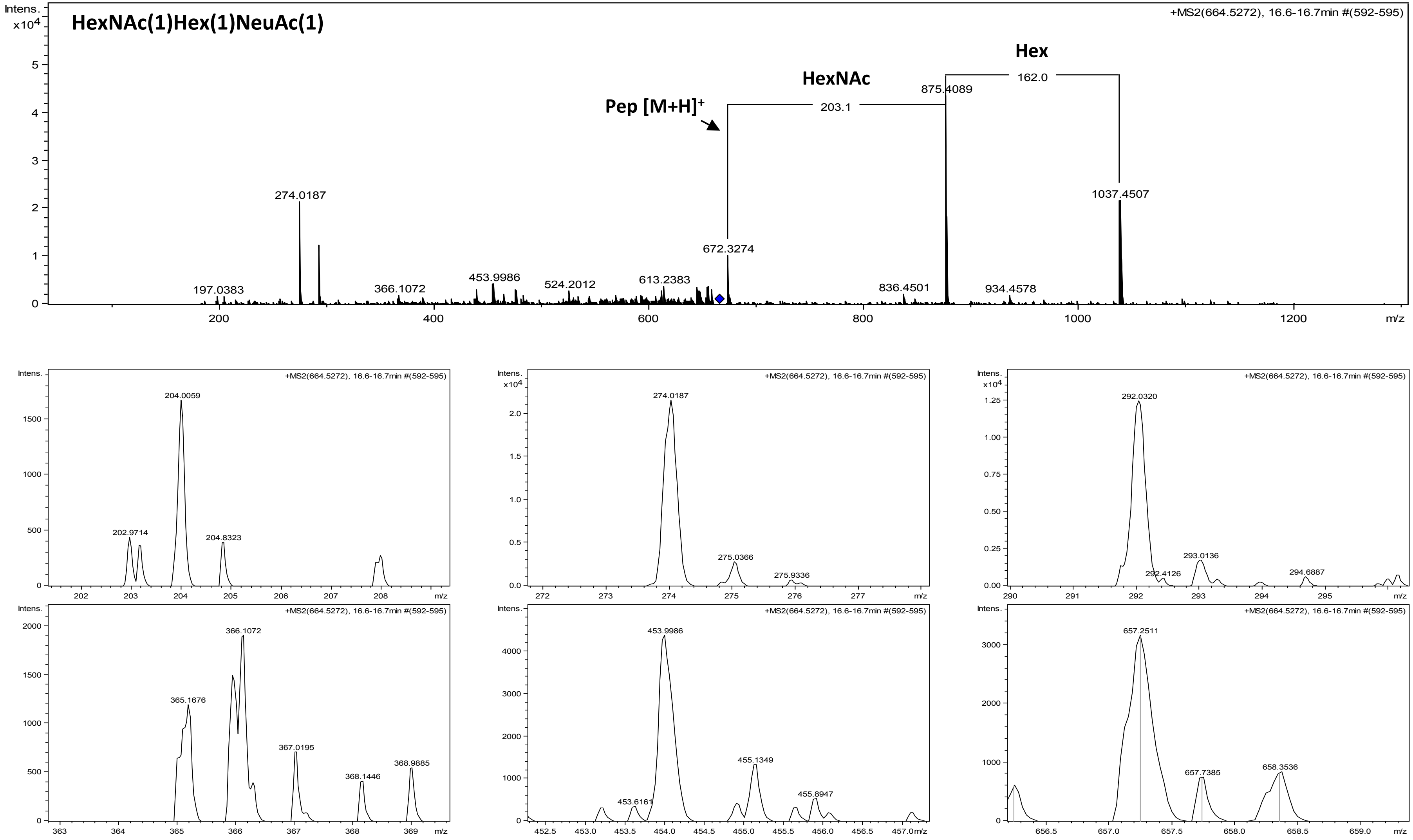

# Fraction 14

## 664.70++ → Pep [M+H]+ 672.33+ [16.6-16.7 min] CID-MS2

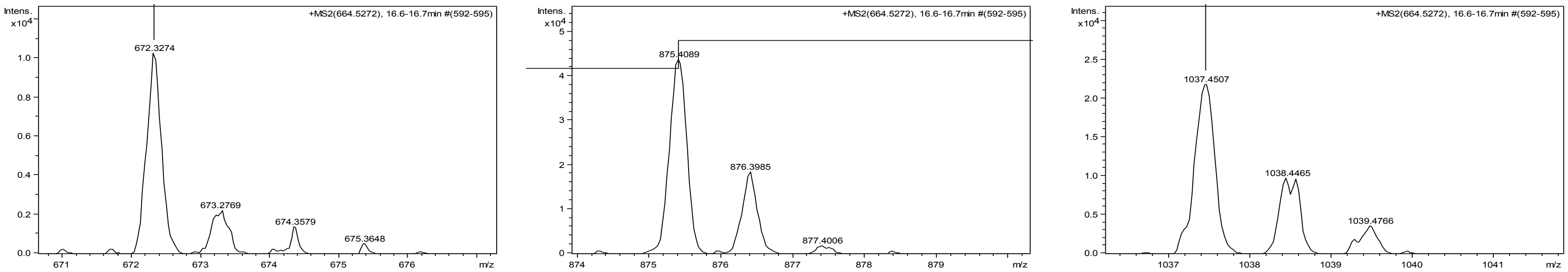

# Fraction 14

## 664.70++ → Pep [M+H]+ 672.33+ [16.6-16.7 min] Manual DeNovo

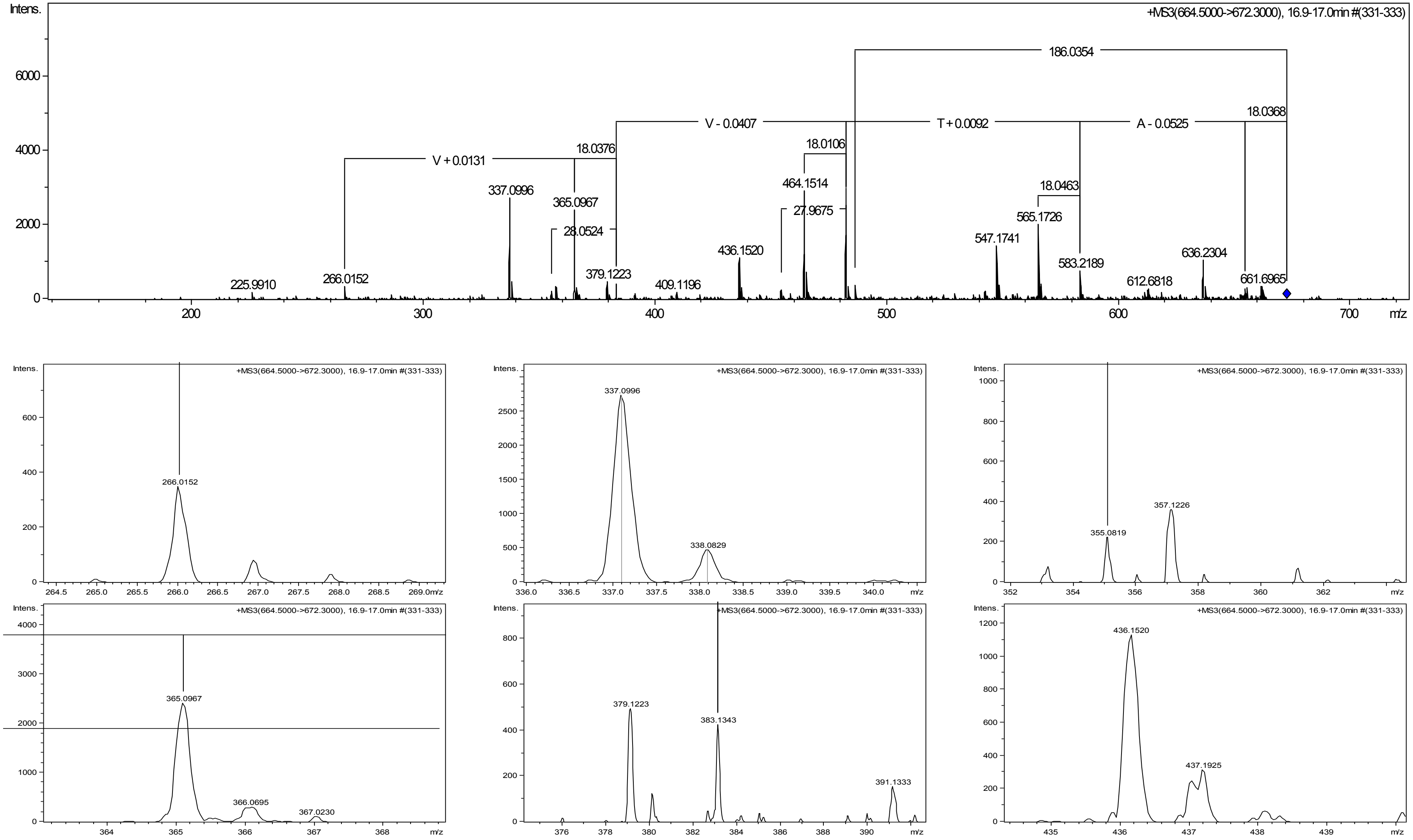

# Fraction 14

## 664.70++ → Pep [M+H]+ 672.33+ [16.6-16.7 min]    CID-MS3

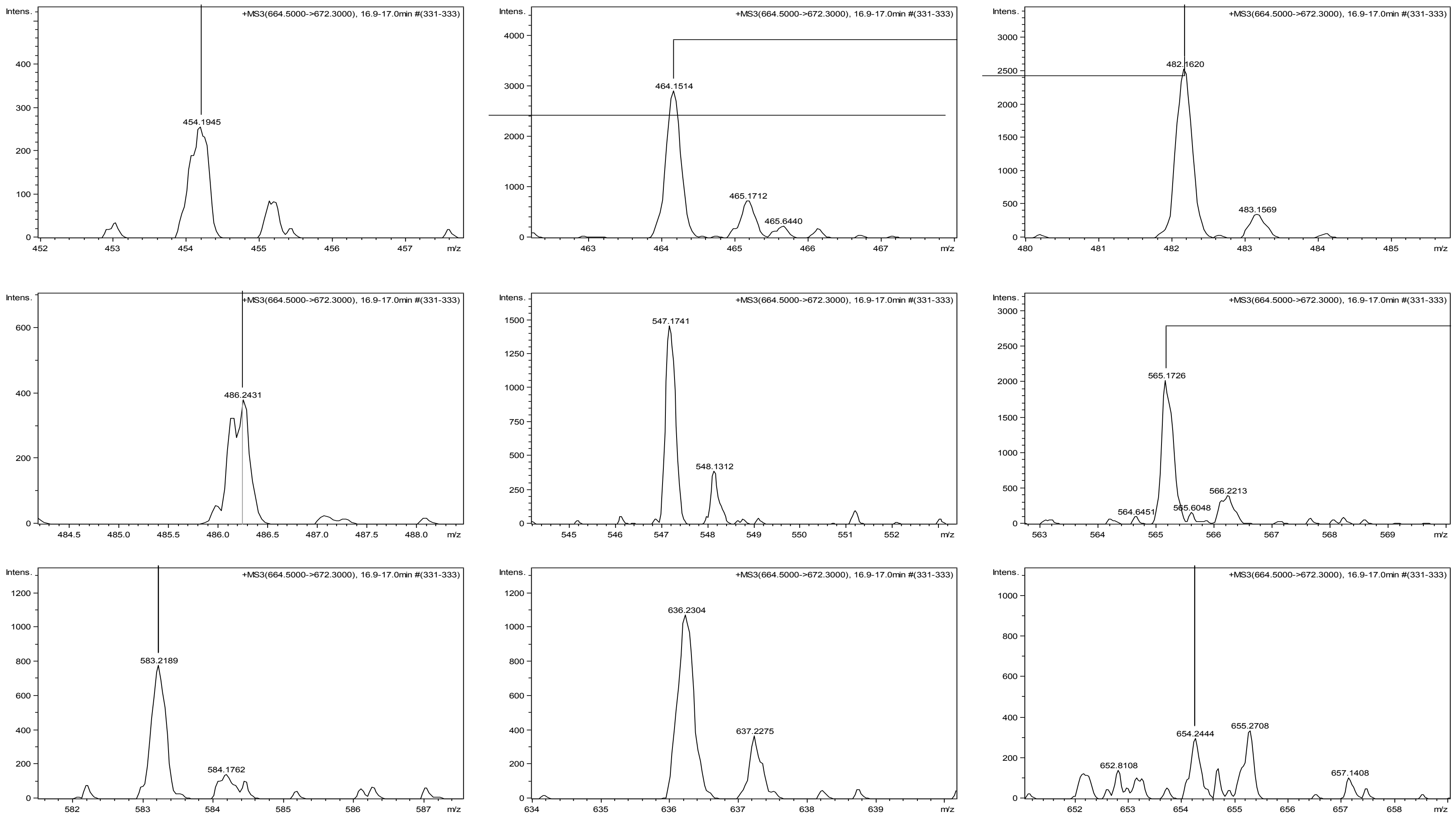

# Fraction 14

664.70++ → Pep [M+H]+ 672.33+ [16.6-16.7 min]

CID-MS3

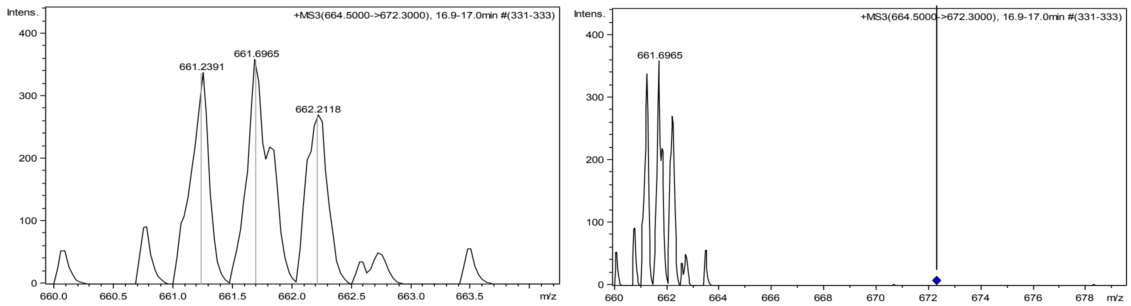

# Fraction 14

664.70++ → Pep [M+H]+ 672.33+ [16.6-16.7 min]

CID-MS3 MASCOT Search

| prot_hit_nur | prot_acc | prot_desc | prot_score | prot_mass | prot_matche | pep_query | pep_rank | pep_isbold | pep_exp_mz | pep_exp_mr | pep_exp_z | pep_calc_mr | pep_delta | pep_miss | pep_score | pep_expect | pep_res_bef | pep_seq |
|---|---|---|---|---|---|---|---|---|---|---|---|---|---|---|---|---|---|---|
| 1 | FA96B_HUMA | Protein FAM | 17 | 17766 | 1 | 1 | 1 | 1 | 672.3274 | 671.3201 | 1 | 671.3602 | -0.0401 | 0 | 18.94 | 1.40E+02 | G | ERPVTA |
| 2 | CN021_HUM | Uncharacteri | 9 | 70136 | 1 | 1 | 3 | 0 | 672.3274 | 671.3201 | 1 | 671.3061 | 0.014 | 0 | 14.42 | 4.10E+02 | E | HQVAMA |
| 3 | IBP6_HUMAI | Insulin-like g | 9 | 26219 | 1 | 1 | 9 | 0 | 672.3274 | 671.3201 | 1 | 671.3602 | -0.0401 | 0 | 12.47 | 6.40E+02 | S | KPQAGTA |
| 4 | FA59A_HUM | Protein FAM | 9 | 98550 | 1 | 1 | 2 | 0 | 672.3274 | 671.3201 | 1 | 671.349 | -0.0289 | 0 | 14.88 | 3.60E+02 | S | PTSPVTA |
| 5 | ELOV4_HUM | Elongation o | 8 | 36976 | 1 | 1 | 9 | 0 | 672.3274 | 671.3201 | 1 | 671.3966 | -0.0765 | 0 | 12.47 | 6.40E+02 | K | PKAGKTA |
| 6 | TXND3_HUM | Thioredoxin | 8 | 67683 | 1 | 1 | 4 | 0 | 672.3274 | 671.3201 | 1 | 671.3126 | 0.0075 | 0 | 13.03 | 5.60E+02 | D | QPEVEA |
| 7 | VAV2_HUMA | Protein vav-: | 7 | 102446 | 1 | 1 | 5 | 0 | 672.3274 | 671.3201 | 1 | 671.3238 | -0.0037 | 0 | 12.88 | 5.80E+02 | R | ERPAEA |
| 8 | NUPL_HUMA | Nucleoporin | 6 | 58509 | 1 | 1 | 7 | 0 | 672.3274 | 671.3201 | 1 | 671.3126 | 0.0075 | 0 | 12.62 | 6.10E+02 | A | PAPQSTA |
| 9 | K1219_HUM | Protein KIAA | 6 | 168460 | 1 | 1 | 5 | 0 | 672.3274 | 671.3201 | 1 | 671.3238 | -0.0037 | 0 | 12.88 | 5.80E+02 | S | ERPAQA |
| 10 | DOCK9_HUM | Dedicator of | 5 | 238519 | 1 | 1 | 7 | 0 | 672.3274 | 671.3201 | 1 | 671.2875 | 0.0327 | 0 | 12.62 | 6.10E+02 | K | HQSETA |

MASCOT does not give the correct result:
most likely protein: Insulin-like growth factor-binding protein 6 precursor
→ fragment ions do not match!

# Fraction 14

664.70++ → Pep [M+H]+ 672.33+ [16.6-16.7 min]

CID-MS3 MS-Homology-Search

University of California, San Francisco | About UCSF | Search UCSF | UCSF Medical Center

Home | MS-Fit | MS-Tag | MS-Seq | MS-Pattern | MS-Bridge | MS-Digest | MS-Product | MS-Comp | DB-Stat | MS-Isotope | MS-Homology

## MS-Homology Search Results

Search completed. 11 sec elapsed. 0 sec remaining.

[–] Parameters

Database searched: **SwissProt.2012.12.3**
Digest Used: **No enzyme**
Max. # Missed Cleavages: **1**
Min matches: **1**
Score matrix: **BLOSUM62**
List of Sequences: **1 [283.0]VVTA 0**
Mass Tolerance: **0.5 Da**

266.02+17=283.02

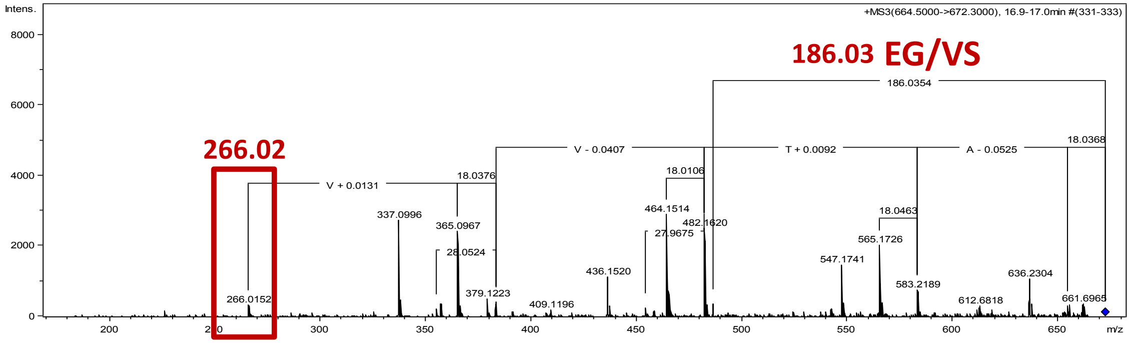


[–] Pre Search Results (SwissProt.2012.12.3)

Number of entries in the database: **453850**
Full Molecular Weight range: **453850** entries.
Full pI range: **453850** entries.
Taxonomy search **HOMO SAPIENS** selects **20479** entries.
Pre searches select **20479** entries.

MS-Homology search selects **3** entries.

Number of Peptide Hits: **3**

| Protein Score | Peptide Score | Peptide Sequence | Matching Sequence | Start AA | MS-Digest Index # | Protein MW (Da)/pI | Accession # | Species | Protein Name |
|---|---|---|---|---|---|---|---|---|---|
| 35 | 35 | EGPVVTA | (A)EGPVVTA(Q) | 132 | 120339 | 71958/6.3 | P01042 | HUMAN | Kininogen-1 ✓ |
| 31 | 31 | ILGVVTA | (Y)ILGVVTA(M) | 441 | 112255 | 64615/8.4 | Q86VW1 | HUMAN | Solute carrier family 22 member 16 |
| 29 | 29 | AVLVVTA | (L)AVLVVTA(T) | 14 | 154464 | 68392/5.3 | Q6P5W5 | HUMAN | Zinc transporter ZIP4 |

MS-Homology in ProteinProspector 5.10.11 Basic
© Copyright (1999-2013) The Regents of the University of California.

# Fraction 14

664.70++ → Pep [M+H]+ 672.33+ [16.6-16.7 min]

CID-MS3 MS-Homology-Search

UCSF University of California, San Francisco | About UCSF | Search UCSF | UCSF Medical Center

Home | MS-Fit | MS-Tag | MS-Seq | MS-Pattern | MS-Bridge | MS-Digest | MS-Product | MS-Comp | Di

## EGPVVTA

User AA Formula 1: **C2 H3 N1 O1**

Elemental Composition: C29 H50 N7 O11

| $MH^{+1}$(av) | $MH^{+1}$(mono) |
|---|---|
| 672.7605 | 672.3563 |

[–] Main Sequence Ions

| b | | | | y |
|---|---|---|---|---|
| --- | 1 | E | 7 | --- |
| 187.0713 | 2 | G | 6 | 543.3137 |
| 284.1241 | 3 | P | 5 | 486.2922 |
| 383.1925 | 4 | V | 4 | 389.2395 |
| 482.2609 | 5 | V | 3 | 290.1710 |
| 583.3086 | 6 | T | 2 | 191.1026 |
| --- | 7 | A | 1 | 90.0550 |

[+] All Sequence Ions

[–] Theoretical Peak Table

| | | | | | | | | | |
|---|---|---|---|---|---|---|---|---|---|
| 70.0651 | P | 173.0921 | y2-H2O | 290.1710 | y3 | 454.2660 | a5 | 555.3137 | a6 |
| 72.0808 | V | 187.0713 | b2 | 302.1347 | Cladder3 | 464.2504 | b5-H2O | 565.2980 | b6-H2O |
| 74.0600 | T | 191.1026 | y2 | 355.1976 | a4 | 468.2817 | y5-H2O | 583.3086 | b6 |
| 90.0550 | y1 | 205.0819 | Cladder2 | 365.1819 | b4-H2O | 482.2609 | b5 | 601.3192 | Cladder6 |
| 102.0550 | E | 256.1292 | a3 | 371.2289 | y4-H2O | 486.2922 | y5 | 654.3457 | MH-H2O |
| 126.0550 | P | 266.1135 | b3-H2O | 383.1925 | b4 | 500.2715 | Cladder5 | 672.3563 | MH |
| 159.0764 | a2 | 272.1605 | y3-H2O | 389.2395 | y4 | 525.3031 | y6-H2O | | |
| 169.0608 | b2-H2O | 284.1241 | b3 | 401.2031 | Cladder4 | 543.3137 | y6 | | |

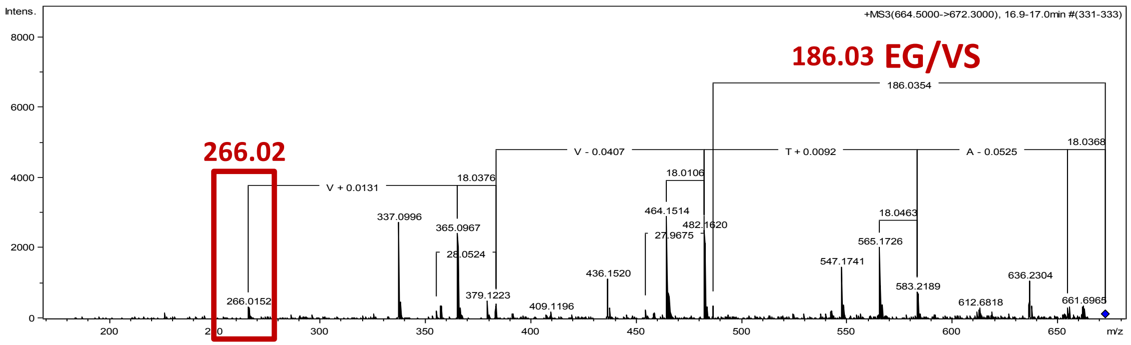


Kininogen-1

$_{132}$EGPVV**T**A$_{138}$

known O-glycosylation site

# Fraction 14

664.70++ → Pep [M+H]+ 672.33+ [16.6-16.7 min] CID-MS3 BioTools

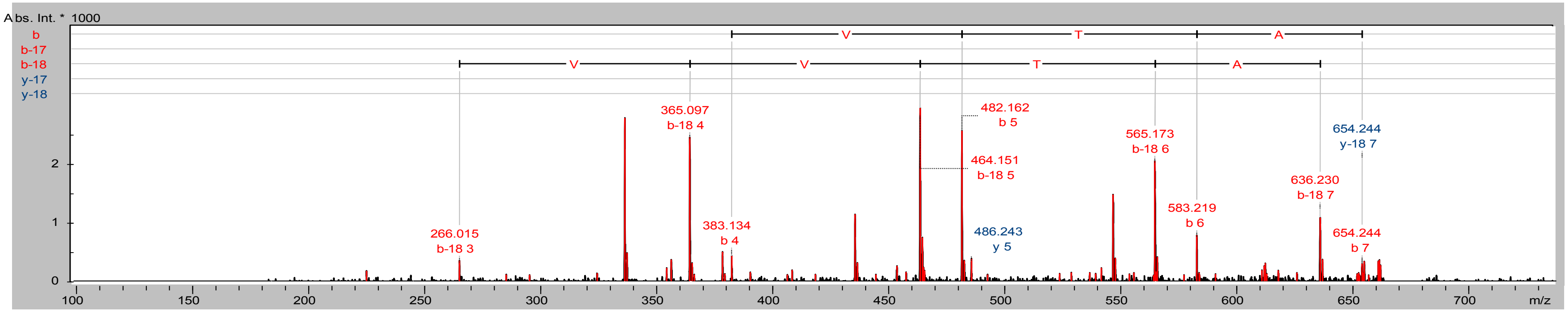


| | E | G | P | V | V | T | A | Glu | Gly | Pro | Val | Val | Thr | Ala |
|---|---|---|---|---|---|---|---|---|---|---|---|---|---|---|
| Ion | 1 | 2 | 3 | 4 | 5 | 6 | 7 | 1 | 2 | 3 | 4 | 5 | 6 | 7 |
| b | E | G | P | V | V | T | A | 130.050 | 187.071 | 284.124 | 383.193 | 482.261 | 583.309 | 654.346 |
| b-17 | E | G | P | V | V | T | A | - | - | - | - | - | - | - |
| b-18 | E | G | P | V | V | T | A | 112.039 | 169.061 | 266.114 | 365.182 | 464.250 | 565.298 | 636.335 |
| y | E | G | P | V | V | T | A | 90.055 | 191.103 | 290.171 | 389.239 | 486.292 | 543.314 | 672.356 |
| y-17 | E | G | P | V | V | T | A | - | - | - | - | - | - | - |
| y-18 | E | G | P | V | V | T | A | - | 173.092 | 272.160 | 371.229 | 468.282 | 525.303 | 654.346 |
| | 7 | 6 | 5 | 4 | 3 | 2 | 1 | Ala | Thr | Val | Val | Pro | Gly | Glu |

Biotools-Score: 43

known O-glycosylation site

Kininogen-1

$_{132}$EGPVV**T**A$_{138}$

# Fraction 14

757.30++ → Pep [M+H]+ 857.43+ [17.3-17.4 min]    CID-MS Precursor

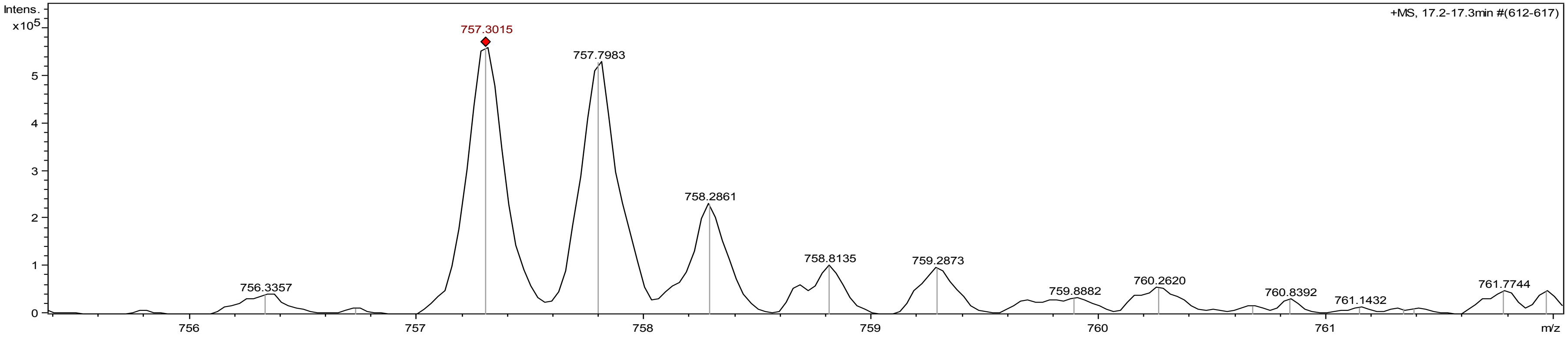


ETD spectrum of poor quality

# Fraction 14

757.30++ → Pep [M+H]+ 857.43+ [17.3-17.4 min] CID-MS2

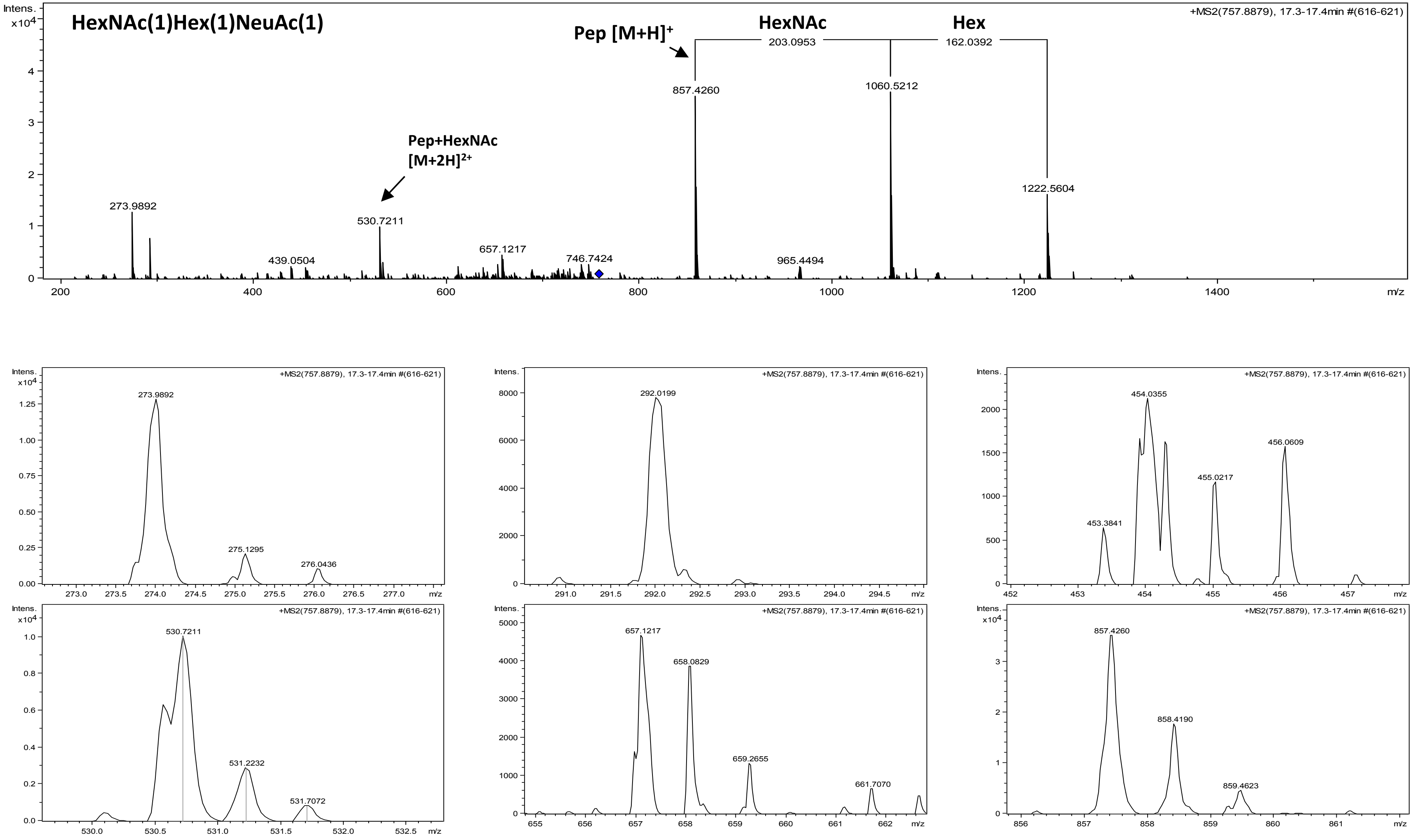

# Fraction 14

757.30++ → Pep [M+H]+ 857.43+ [17.3-17.4 min] CID-MS2

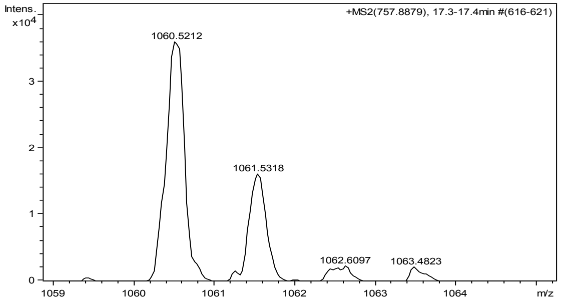


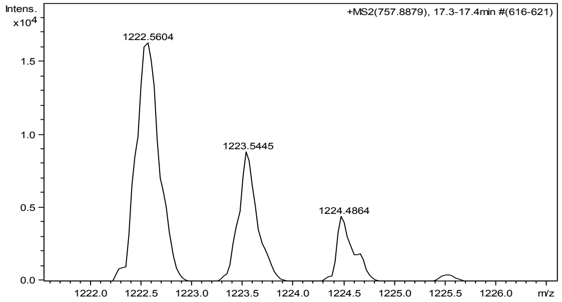

# Fraction 14

757.30++ → Pep [M+H]+ 857.43+ [17.3-17.4 min]

CID-MS3 Manual DeNovo

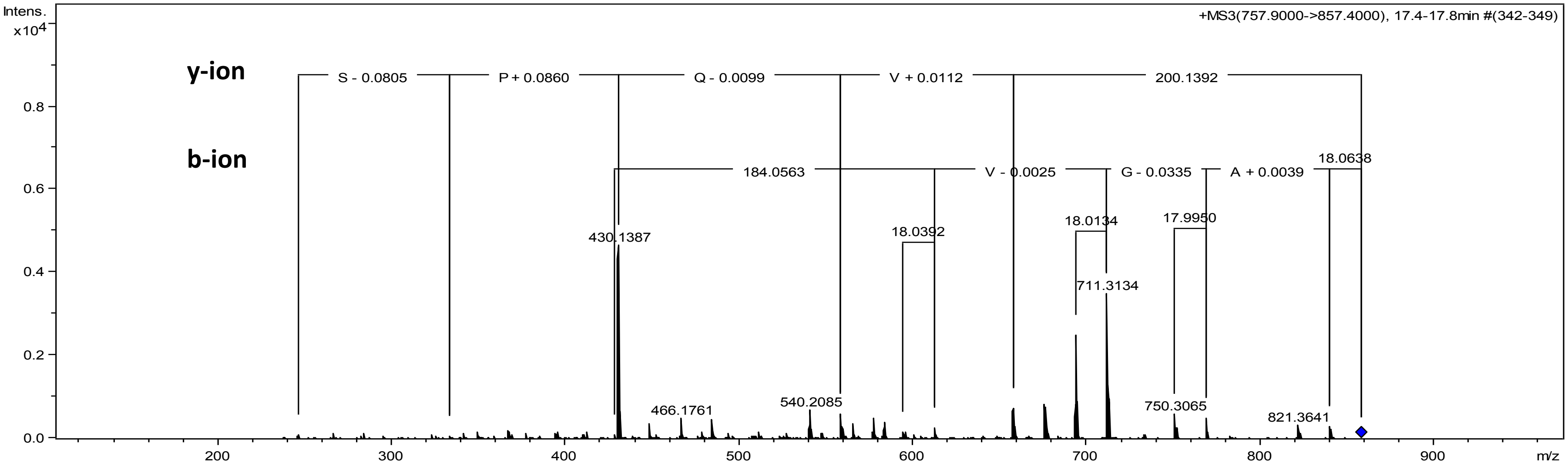


known O-glycosylation site

Alpha-2-HS-glycoprotein precursor

$_{341}$TVVQP**S**VGA$_{349}$

# Fraction 14

757.30++ → Pep [M+H]+ 857.43+ [17.3-17.4 min]

CID-MS3

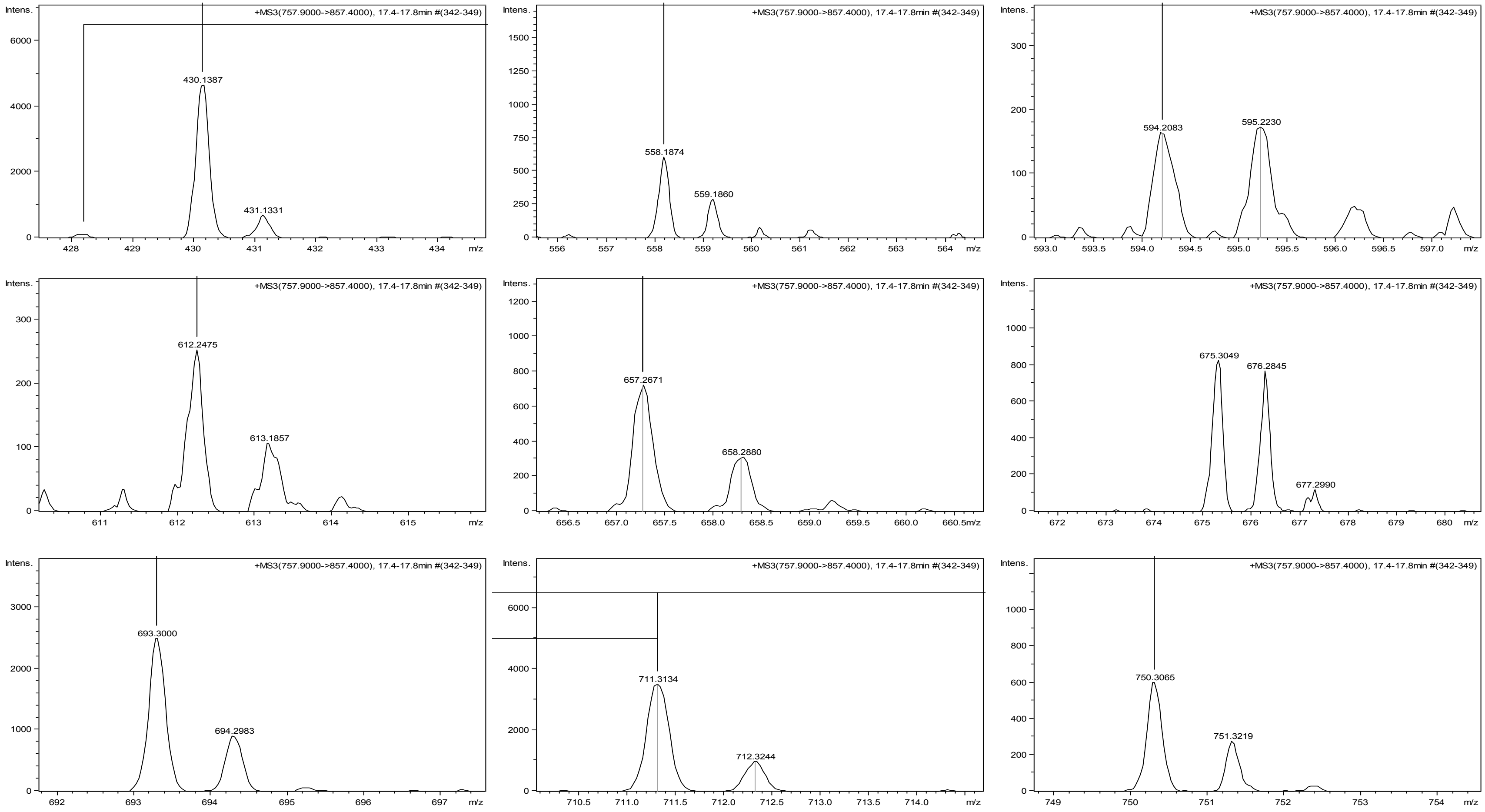


known O-glycosylation site

Alpha-2-HS-glycoprotein precursor

$_{341}$TVVQP**S**VGA$_{349}$

# Fraction 14

757.30++ → Pep [M+H]+ 857.43+ [17.3-17.4 min]

CID-MS3

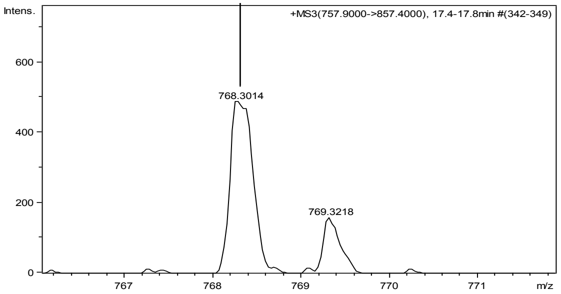


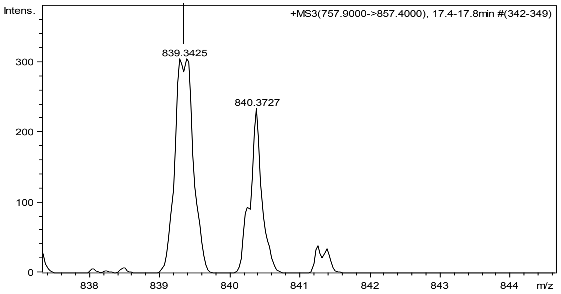


known O-glycosylation site

Alpha-2-HS-glycoprotein precursor

$_{341}$TVVQP**S**VGA$_{349}$

# Fraction 14

757.30++ → Pep [M+H]+ 857.43+ [17.3-17.4 min] CID-MS3 MASCOT Search

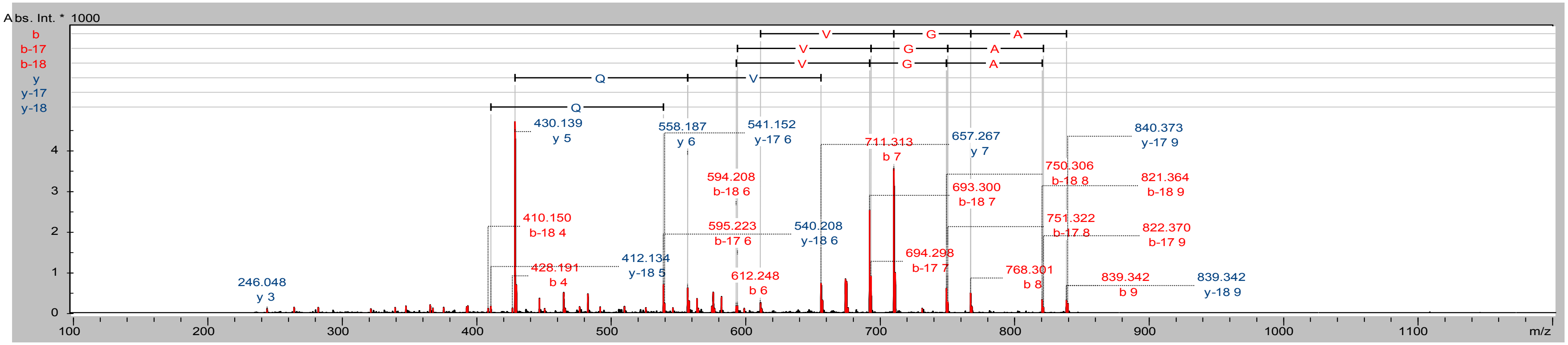


| | T | V | V | Q | P | S | V | G | A | Thr | Val | Val | Gln | Pro | Ser | Val | Gly | Ala |
|---|---|---|---|---|---|---|---|---|---|---|---|---|---|---|---|---|---|---|
| Ion | 1 | 2 | 3 | 4 | 5 | 6 | 7 | 8 | 9 | 1 | 2 | 3 | 4 | 5 | 6 | 7 | 8 | 9 |
| b | T | V | V | Q | P | S | V | G | A | 102.055 | 201.123 | 300.192 | 428.250 | 525.303 | 612.335 | 711.404 | 768.425 | 839.462 |
| b-17 | T | V | V | Q | P | S | V | G | A | - | - | - | 411.224 | 508.277 | 595.309 | 694.377 | 751.398 | 822.436 |
| b-18 | T | V | V | Q | P | S | V | G | A | 84.044 | 183.113 | 282.181 | 410.240 | 507.293 | 594.325 | 693.393 | 750.414 | 821.452 |
| y | T | V | V | Q | P | S | V | G | A | 90.055 | 147.076 | 246.145 | 333.177 | 430.230 | 558.288 | 657.357 | 756.425 | 857.473 |
| y-17 | T | V | V | Q | P | S | V | G | A | - | - | - | - | - | 541.262 | 640.330 | 739.398 | 840.446 |
| y-18 | T | V | V | Q | P | S | V | G | A | - | - | - | 315.166 | 412.219 | 540.278 | 639.346 | 738.414 | 839.462 |
| | 9 | 8 | 7 | 6 | 5 | 4 | 3 | 2 | 1 | Ala | Gly | Val | Ser | Pro | Gln | Val | Val | Thr |

known O-glycosylation site

Alpha-2-HS-glycoprotein precursor

$_{341}$TVVQP**S**VGA$_{349}$

# Fraction 14

## 757.30++ → Pep [M+H]+ 857.43+ [17.3-17.4 min] CID-MS3 MASCOT Search

| prot_hit_nur | prot_acc | prot_desc | prot_score | prot_mass | prot_matche | pep_query | pep_rank | pep_isbold | pep_exp_mz | pep_exp_mr | pep_exp_z | pep_calc_mr | pep_delta | pep_miss | pep_score | pep_expect | pep_res_bef | pep_seq |
|---|---|---|---|---|---|---|---|---|---|---|---|---|---|---|---|---|---|---|
| 1 | REL3_HUMAI | Relaxin-3 pre | 17 | 15783 | 1 | 1 | 2 | 1 | 857.426 | 856.4187 | 1 | 856.4191 | -0.0004 | 0 | 19.19 | 1.50E+02 | L | WPGAEARA |
| 2 | FETUA_HUM | Alpha-2-HS-g | 14 | 40098 | 1 | 1 | 1 | 0 | 857.426 | 856.4187 | 1 | 856.4655 | -0.0467 | 0 | 19.59 | 1.40E+02 | R | TVVQPSVGA |
| 3 | PP2CA_HUM | Protein phos | 14 | 43048 | 1 | 1 | 4 | 0 | 857.426 | 856.4187 | 1 | 856.5018 | -0.0831 | 0 | 18.27 | 1.90E+02 | K | VSPEAVKK |
| 4 | LU_HUMAN | Lutheran blo | 14 | 68161 | 1 | 1 | 3 | 0 | 857.426 | 856.4187 | 1 | 856.4039 | 0.0149 | 0 | 18.43 | 1.80E+02 | P | DAPAQARGA |
| 5 | OR3A2_HUM | Olfactory rec | 13 | 35053 | 1 | 1 | 5 | 0 | 857.426 | 856.4187 | 1 | 856.4039 | 0.0148 | 0 | 18.02 | 2.00E+02 | L | RNPDVQGA |
| 6 | NOG2_HUMA | Nucleolar GT | 12 | 83831 | 1 | 1 | 5 | 0 | 857.426 | 856.4187 | 1 | 856.4039 | 0.0148 | 0 | 18.02 | 2.00E+02 | T | NPDRVQGA |
| 7 | COQ9_HUMA | Ubiquinone | 11 | 35658 | 1 | 1 | 10 | 0 | 857.426 | 856.4187 | 1 | 856.3675 | 0.0512 | 0 | 16.03 | 3.20E+02 | Q | QHSETQGA |
| 8 | ALO17_HUM | Protein ALO1 | 11 | 176839 | 1 | 1 | 7 | 0 | 857.426 | 856.4187 | 1 | 856.429 | -0.0103 | 0 | 17.58 | 2.20E+02 | A | EPANAVKGA |
| 9 | NOL6_HUMA | Nucleolar pr | 10 | 128368 | 1 | 1 | 8 | 0 | 857.426 | 856.4187 | 1 | 856.3927 | 0.0261 | 0 | 17.21 | 2.40E+02 | W | PQDAEAVQ |
| 10 | PK1L1_HUMA | Polycystic ki | 9 | 319453 | 1 | 1 | 9 | 0 | 857.426 | 856.4187 | 1 | 856.5018 | -0.0831 | 0 | 16.95 | 2.60E+02 | V | GLLGSLGLGA |

Biotools-Score: 36

MASCOT-Score: 20

known O-glycosylation site

Alpha-2-HS-glycoprotein precursor

$_{341}$TVVQP**S**VGA$_{349}$

# Fraction 14

742.76++ → Pep [M+H]+ 828.42+ [18.5-18.9 min] CID-MS Precursor

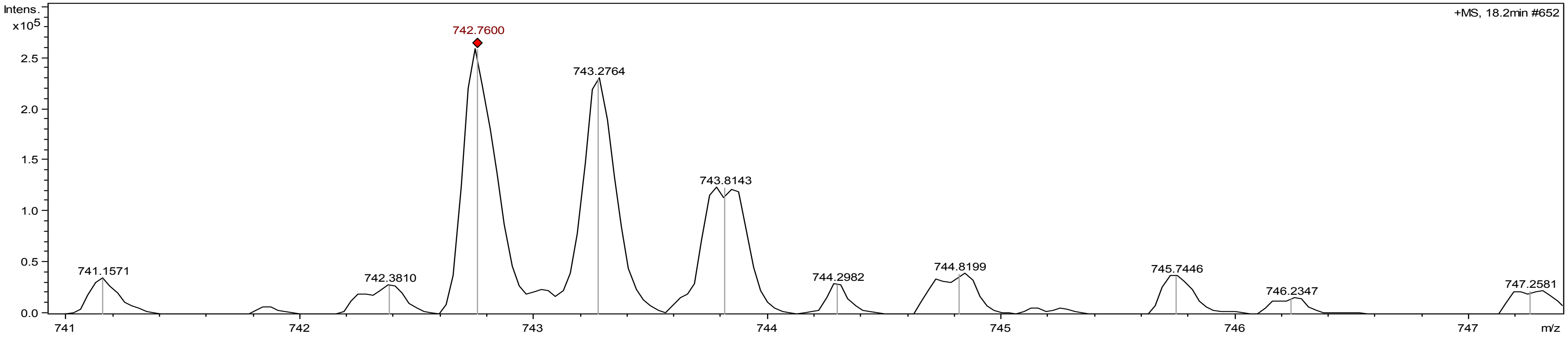


ETD spectrum of poor quality

# Fraction 14

## 742.76++ → Pep [M+H]+ 828.42+ [18.5-18.9 min] CID-MS2

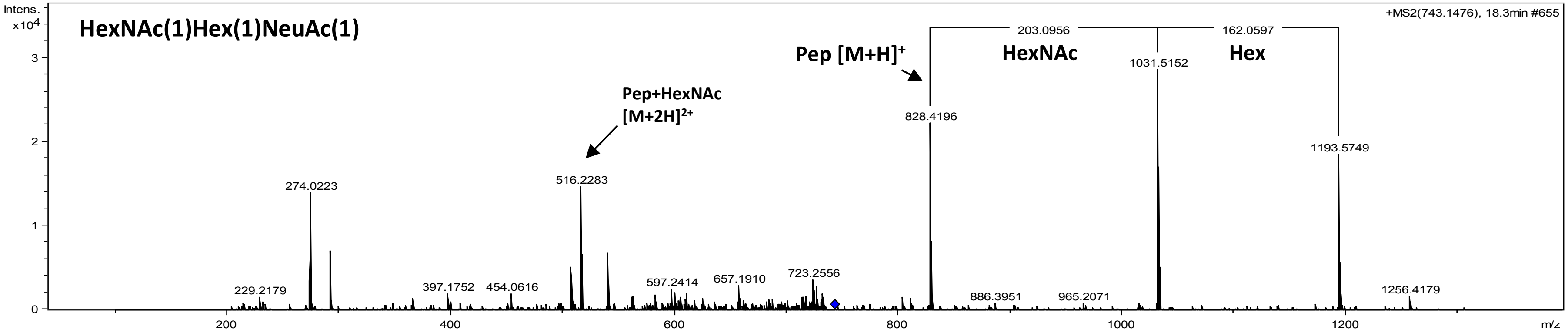


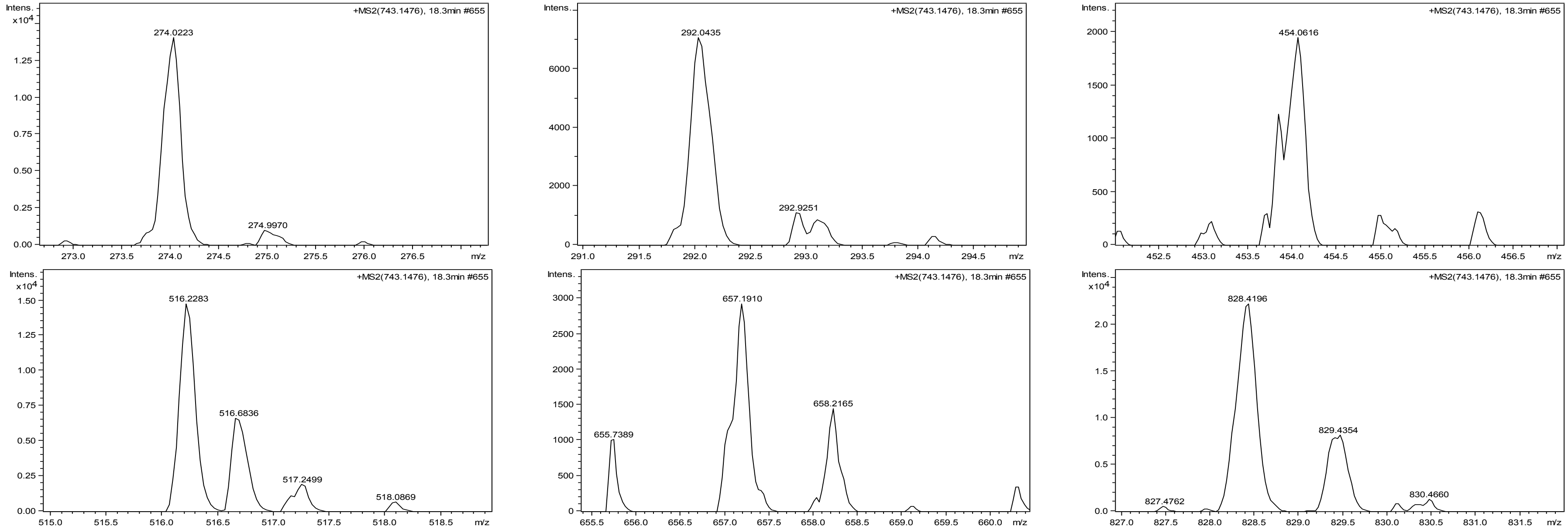

# Fraction 14

742.76++ → Pep [M+H]+ 828.42+ [18.5-18.9 min] CID-MS2

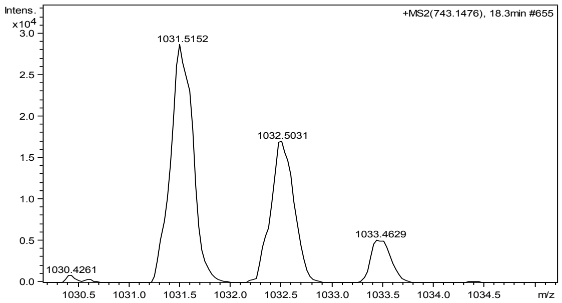


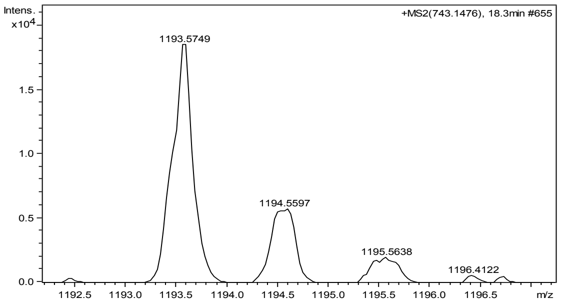

# Fraction 14

742.76++ → Pep [M+H]+ 828.42+ [18.5-18.9 min]

CID-MS3 Manual DeNovo

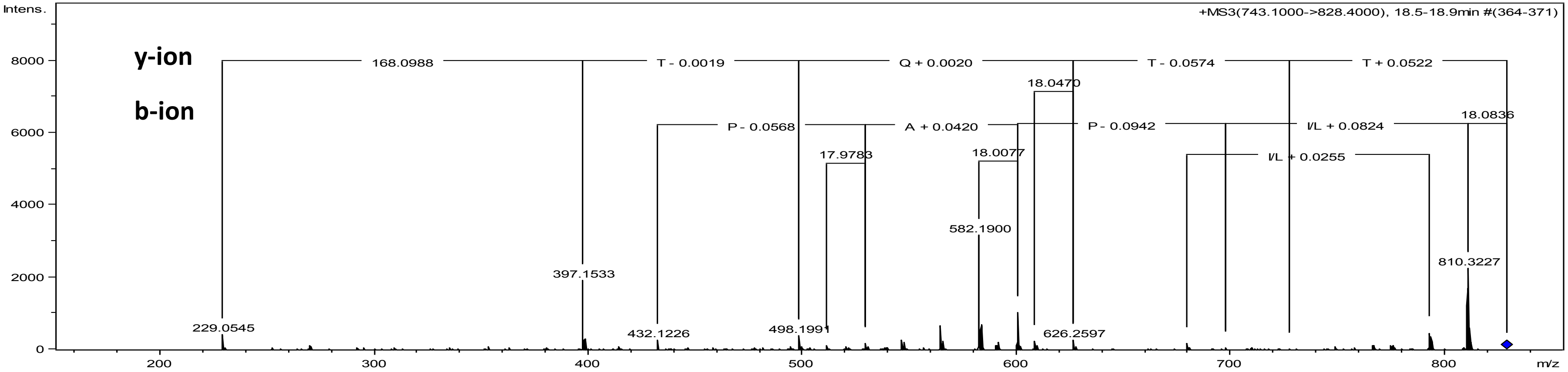

# Fraction 14

742.76++ → Pep [M+H]+ 828.42+ [18.5-18.9 min]

CID-MS3

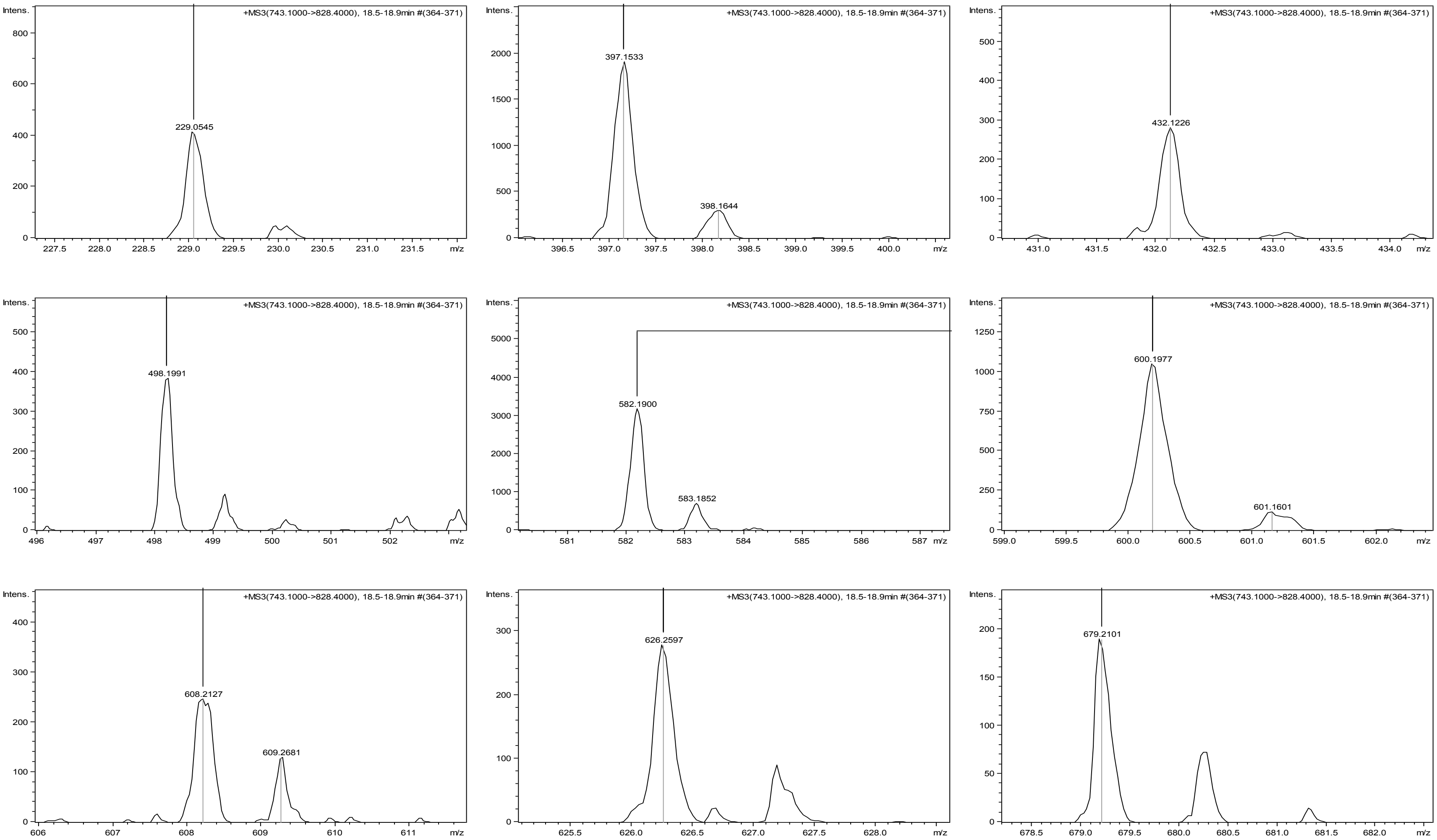

# Fraction 14

742.76++ → Pep [M+H]+ 828.42+ [18.5-18.9 min] CID-MS3

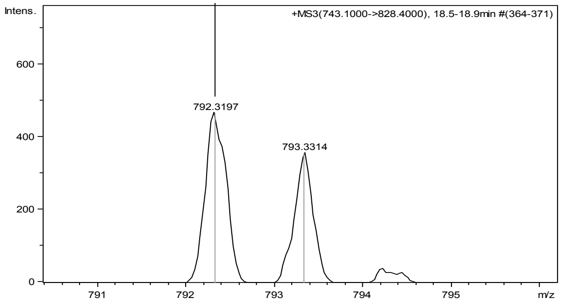


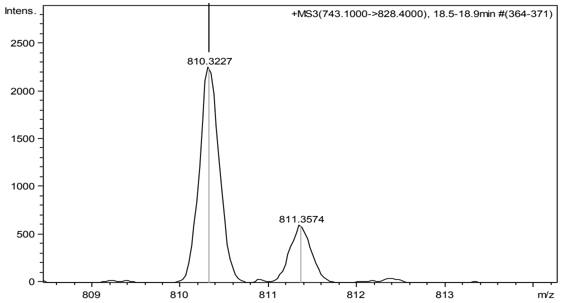

# Fraction 14

742.76++ → Pep [M+H]+ 828.42+ [18.5-18.9 min]

CID-MS3 MASCOT-Search

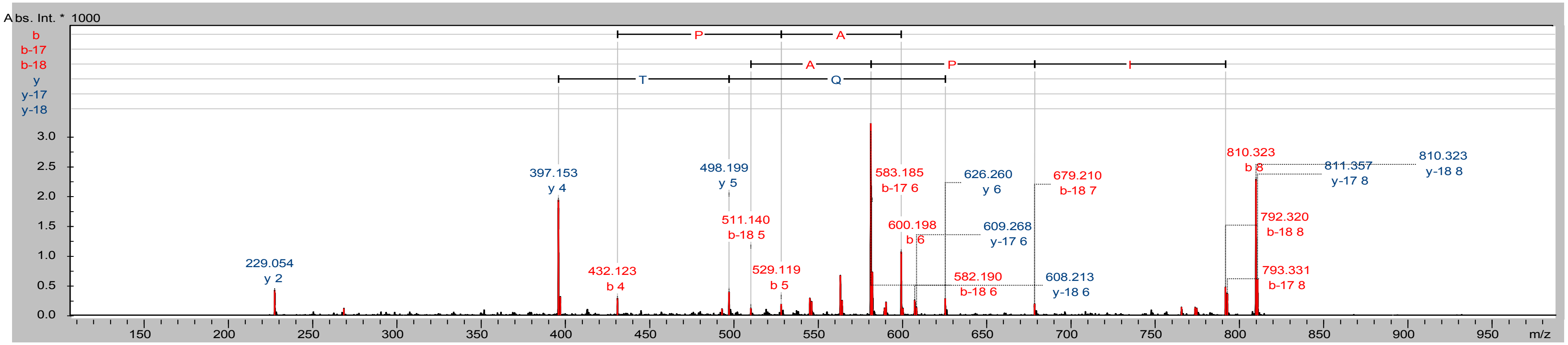


| | T | T | Q | T | P | A | P | I | Thr | Thr | Gln | Thr | Pro | Ala | Pro | Ile |
|---|---|---|---|---|---|---|---|---|---|---|---|---|---|---|---|---|
| Ion | 1 | 2 | 3 | 4 | 5 | 6 | 7 | 8 | 1 | 2 | 3 | 4 | 5 | 6 | 7 | 8 |
| b | T | T | Q | T | P | A | P | I | 102.055 | 203.103 | 331.161 | 432.209 | 529.262 | 600.299 | 697.352 | 810.436 |
| b-17 | T | T | Q | T | P | A | P | I | - | - | 314.135 | 415.182 | 512.235 | 583.272 | 680.325 | 793.409 |
| b-18 | T | T | Q | T | P | A | P | I | 84.044 | 185.092 | 313.151 | 414.198 | 511.251 | 582.288 | 679.341 | 792.425 |
| y | T | T | Q | T | P | A | P | I | 132.102 | 229.155 | 300.192 | 397.245 | 498.292 | 626.351 | 727.398 | 828.446 |
| y-17 | T | T | Q | T | P | A | P | I | - | - | - | - | - | 609.324 | 710.372 | 811.420 |
| y-18 | T | T | Q | T | P | A | P | I | - | - | - | - | 480.282 | 608.340 | 709.388 | 810.436 |
| | 8 | 7 | 6 | 5 | 4 | 3 | 2 | 1 | Ile | Pro | Ala | Pro | Thr | Gln | Thr | Thr |

unknown O-glycosylation region

Inter-alpha-trypsin inhibitor heavy chain H4 precursor (ITI heavy chain H4)

$_{722}$**TT**Q**T**PAPI$_{729}$

# Fraction 14

742.76++ → Pep [M+H]+ 828.42+ [18.5-18.9 min]

CID-MS3 MASCOT-Search

| prot_hit_nur | prot_acc | prot_desc | prot_score | prot_mass | prot_matche | pep_query | pep_rank | pep_isbold | pep_exp_mz | pep_exp_mr | pep_exp_z | pep_calc_mr | pep_delta | pep_miss |
|---|---|---|---|---|---|---|---|---|---|---|---|---|---|---|
| 1 | COG5_HUMA | Conserved oligomeric Gol | 21 | 93117 | 1 | 1 | 1 | 1 | 828.4196 | 827.4123 | 1 | 827.3847 | 0.0276 | 0 |
| 2 | ITIH4_HUMA | Inter-alpha-trypsin inhibit | 20 | 103489 | 1 | 1 | 2 | 0 | 828.4196 | 827.4123 | 1 | 827.4389 | -0.0266 | 0 |
| 3 | SPA13_HUM | Serpin A13 precursor - Hor | 19 | 35070 | 1 | 1 | 4 | 0 | 828.4196 | 827.4123 | 1 | 827.4025 | 0.0099 | 0 |
| 4 | TBA2_HUMA | Tubulin alpha-2 chain (Alp | 18 | 50612 | 1 | 1 | 3 | 0 | 828.4196 | 827.4123 | 1 | 827.5229 | -0.1105 | 0 |
| 5 | ATPBB_HUM | ATP-binding domain 1 fam | 17 | 34994 | 1 | 1 | 4 | 0 | 828.4196 | 827.4123 | 1 | 827.4025 | 0.0098 | 0 |
| 6 | NIT1_HUMAI | Nitrilase homolog 1 (EC 3.5 | 16 | 36728 | 1 | 1 | 6 | 0 | 828.4196 | 827.4123 | 1 | 827.3847 | 0.0276 | 0 |
| 7 | COG2_HUMA | Conserved oligomeric Gol | 14 | 83726 | 1 | 1 | 8 | 0 | 828.4196 | 827.4123 | 1 | 827.4865 | -0.0742 | 0 |
| 8 | RIPK5_HUMA | Receptor-interacting serin | 14 | 106617 | 1 | 1 | 8 | 0 | 828.4196 | 827.4123 | 1 | 827.4865 | -0.0742 | 0 |
| 9 | CXXC6_HUM | CXXC-type zinc finger prot | 12 | 237956 | 1 | 1 | 10 | 0 | 828.4196 | 827.4123 | 1 | 827.4389 | -0.0265 | 0 |
| 10 | PCLO_HUMA | Protein piccolo (Aczonin) - | 11 | 568304 | 1 | 1 | 6 | 0 | 828.4196 | 827.4123 | 1 | 827.4211 | -0.0088 | 0 |

Biotools-Score: 33

MASCOT-Score: 27

## unknown O-glycosylation region

Inter-alpha-trypsin inhibitor heavy chain H4 precursor (ITI heavy chain H4)

$_{722}$TTQTPAPI$_{729}$

# Fraction 14

583.58+++ → Pep [M+H]+ 1092.56+ [25.6-26.1 min] CID-MS Precursor

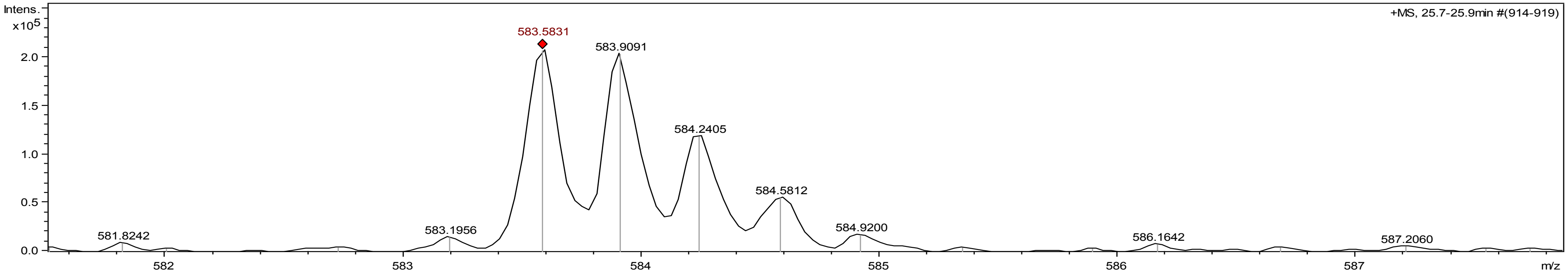

# Fraction 14

583.58+++ → Pep [M+H]+ 1092.56+ [25.6-26.1 min] CID-MS2

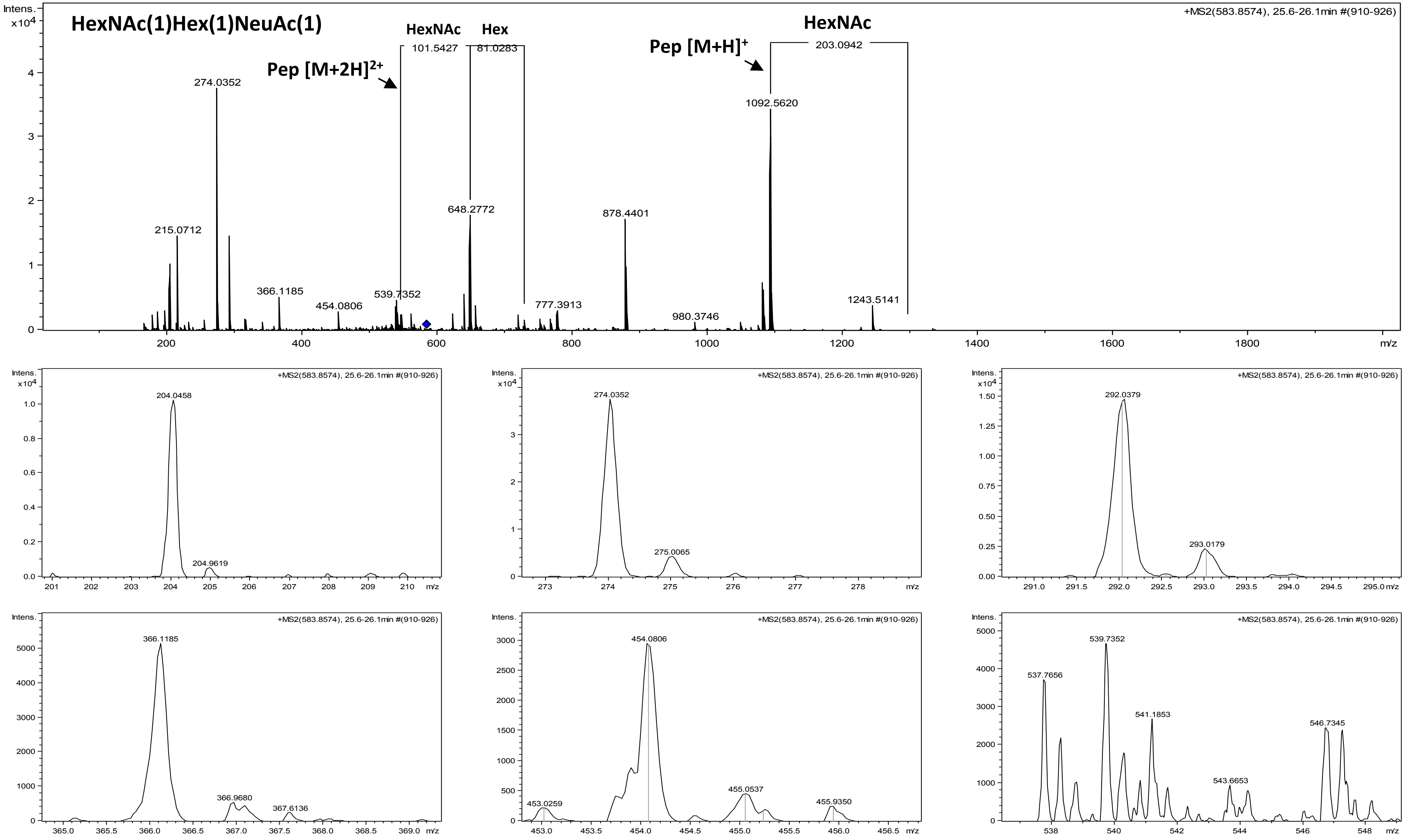

# Fraction 14

583.58+++ → Pep [M+H]+ 1092.56+ [25.6-26.1 min] CID-MS2

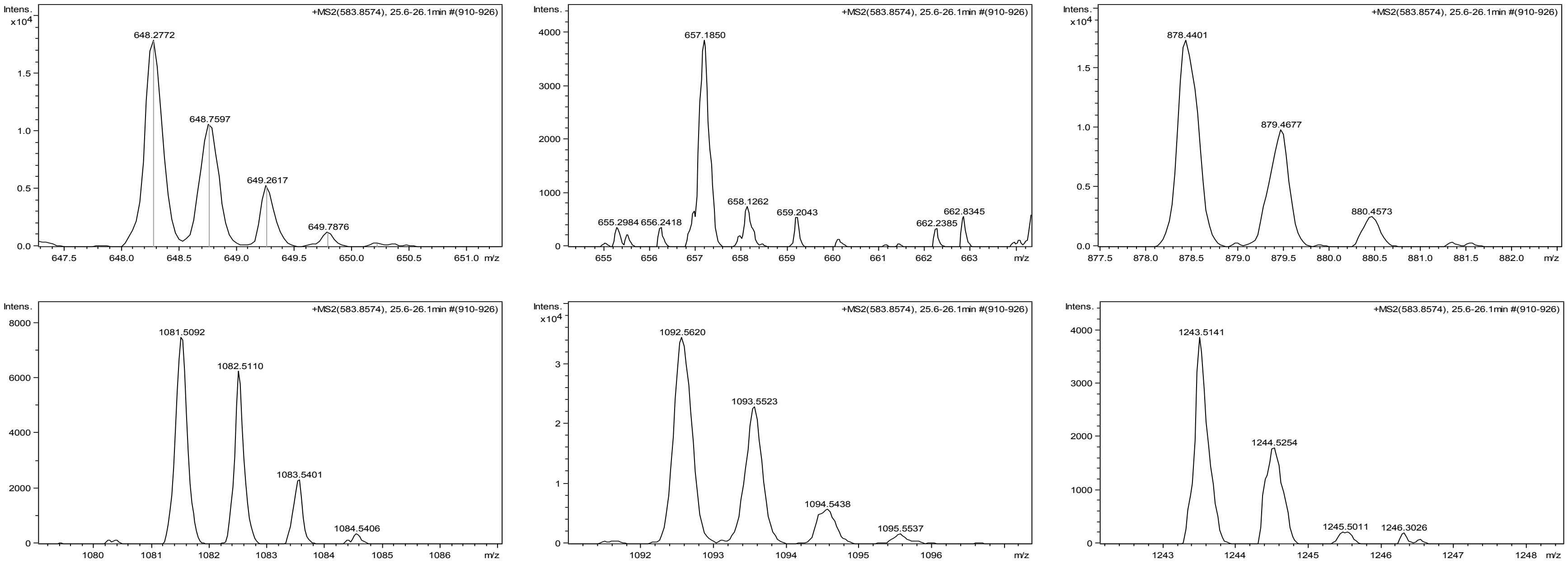

# Fraction 14

583.58+++ → Pep [M+H]+ 1092.56+ [25.6-26.1 min] CID-MS3

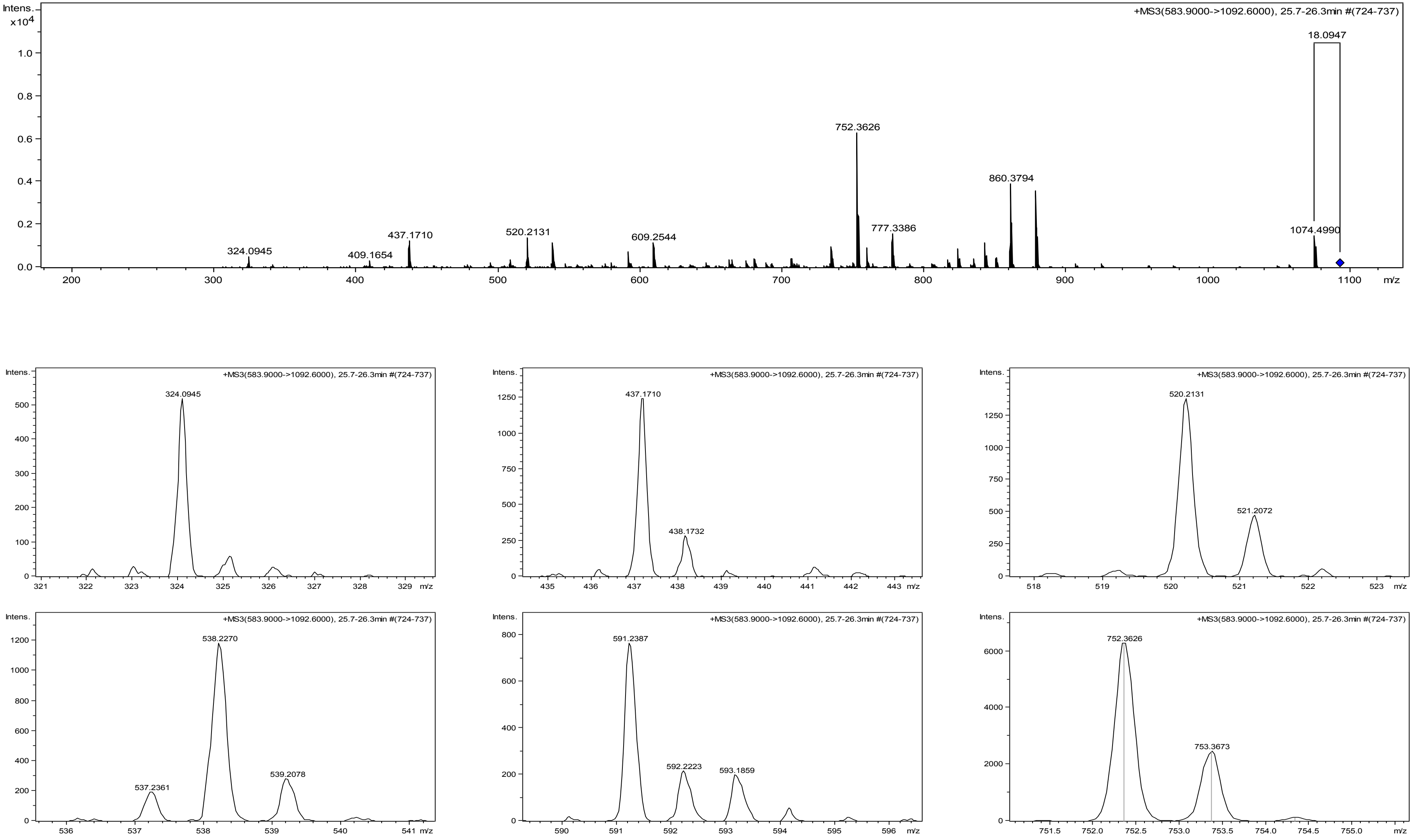

# Fraction 14

583.58+++ → Pep [M+H]+ 1092.56+ [25.6-26.1 min] CID-MS3

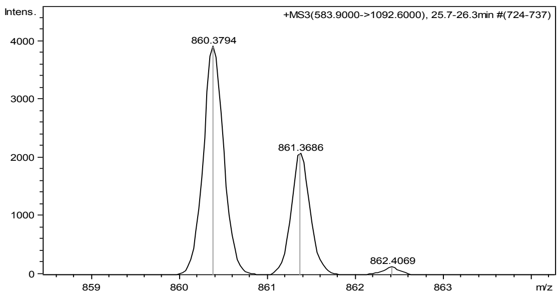


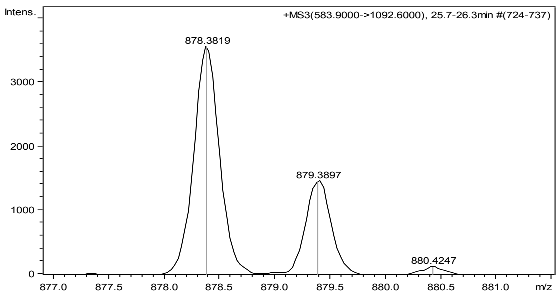


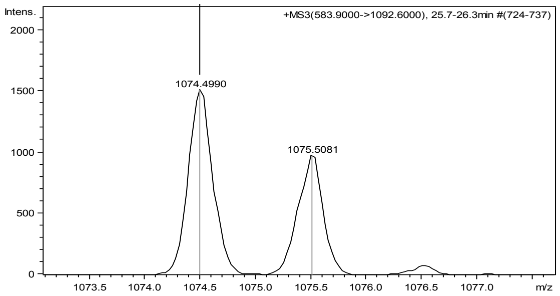

# Fraction 14

583.58+++ → Pep [M+H]+ 1092.56+ [25.6-26.1 min]    CID-MS3    MASCOT-Search

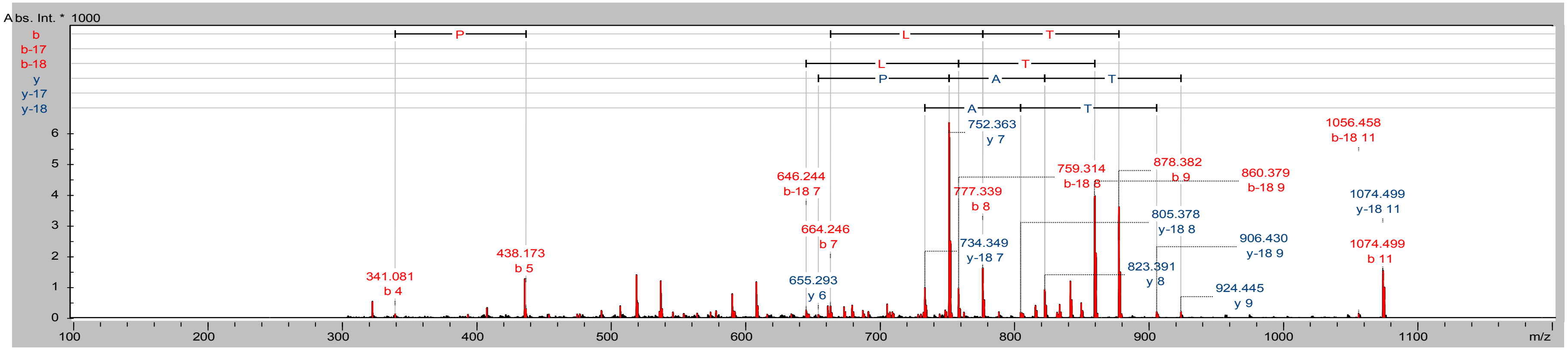


| | A | P | T | A | P | P | E | L | T | P | V | Ala | Pro | Thr | Ala | Pro | Pro | Glu | Leu | Thr | Pro | Val |
|---|---|---|---|---|---|---|---|---|---|---|---|---|---|---|---|---|---|---|---|---|---|---|
| Ion | 1 | 2 | 3 | 4 | 5 | 6 | 7 | 8 | 9 | 10 | 11 | 1 | 2 | 3 | 4 | 5 | 6 | 7 | 8 | 9 | 10 | 11 |
| b | A | P | T | A | P | P | E | L | T | P | V | 72.044 | 169.097 | 270.145 | 341.182 | 438.235 | 535.287 | 664.330 | 777.414 | 878.462 | 975.515 | 1074.583 |
| b-17 | A | P | T | A | P | P | E | L | T | P | V | - | - | - | - | - | - | - | - | - | - | - |
| b-18 | A | P | T | A | P | P | E | L | T | P | V | - | - | 252.134 | 323.171 | 420.224 | 517.277 | 646.320 | 759.404 | 860.451 | 957.504 | 1056.572 |
| y | A | P | T | A | P | P | E | L | T | P | V | 118.086 | 215.139 | 316.187 | 429.271 | 558.313 | 655.366 | 752.419 | 823.456 | 924.504 | 1021.556 | 1092.594 |
| y-17 | A | P | T | A | P | P | E | L | T | P | V | - | - | - | - | - | - | - | - | - | - | - |
| y-18 | A | P | T | A | P | P | E | L | T | P | V | - | - | 298.176 | 411.260 | 540.303 | 637.356 | 734.408 | 805.445 | 906.493 | 1003.546 | 1074.583 |
| | 11 | 10 | 9 | 8 | 7 | 6 | 5 | 4 | 3 | 2 | 1 | Val | Pro | Thr | Leu | Glu | Pro | Pro | Ala | Thr | Pro | Ala |

known O-glycosylation site

Plasminogen

$_{363}$AP**T**APPELTPV$_{373}$

# Fraction 14

583.58+++ → Pep [M+H]+ 1092.56+ [25.6-26.1 min]

CID-MS3 MASCOT-Search

| prot_hit_nur | prot_acc | prot_desc | prot_score | prot_mass | prot_matche | pep_query | pep_rank | pep_isbold | pep_exp_mz | pep_exp_mr | pep_exp_z | pep_calc_mr | pep_delta | pep_miss | pep_score | pep_expect | pep_res_bef | pep_seq |
|---|---|---|---|---|---|---|---|---|---|---|---|---|---|---|---|---|---|---|
| 1 | PLMN_HUM | Plasminogen | 13 | 93247 | 1 | 1 | 2 | 1 | 1092.562 | 1091.5547 | 1 | 1091.5863 | -0.0315 | 0 | 19.03 | 2.10E+02 | L | APTAPPELTP |
| 2 | PLXA4_HUM | Plexin-A4 pr | 13 | 215682 | 1 | 1 | 1 | 0 | 1092.562 | 1091.5547 | 1 | 1091.4991 | 0.0556 | 0 | 20.95 | 1.30E+02 | E | MTCQAPALA |
| 3 | BIRC4_HUM | Baculoviral I | 10 | 57789 | 1 | 1 | 6 | 0 | 1092.562 | 1091.5547 | 1 | 1090.5151 | 1.0396 | 0 | 14.9 | 5.40E+02 | N | PMVQEAIRM |
| 4 | ABCA4_HUM | Retinal-spec | 10 | 258232 | 1 | 1 | 3 | 0 | 1092.562 | 1091.5547 | 1 | 1091.5499 | 0.0048 | 0 | 17.98 | 2.60E+02 | D | TLGNPTVKDF |
| 5 | CERU_HUMA | Ceruloplasm | 9 | 122983 | 1 | 1 | 4 | 0 | 1092.562 | 1091.5547 | 1 | 1091.5499 | 0.0048 | 0 | 16.89 | 3.40E+02 | A | VDPTKDIFTG |
| 6 | PSDE_HUMA | 26S proteaso | 9 | 34726 | 1 | 1 | 9 | 0 | 1092.562 | 1091.5547 | 1 | 1091.5135 | 0.0412 | 0 | 14.32 | 6.10E+02 | A | PAVDTAEQV |
| 7 | NPAS2_HUM | Neuronal PA | 8 | 92501 | 1 | 1 | 6 | 0 | 1092.562 | 1091.5547 | 1 | 1091.4982 | 0.0565 | 0 | 14.89 | 5.40E+02 | N | LTTPASTSQD |
| 8 | FLII_HUMAN | Protein fligh | 7 | 146142 | 1 | 1 | 8 | 0 | 1092.562 | 1091.5547 | 1 | 1091.5571 | -0.0024 | 0 | 14.85 | 5.40E+02 | W | RGAQATLSST |
| 9 | PHF20_HUM | PHD finger p | 7 | 116739 | 1 | 1 | 10 | 0 | 1092.562 | 1091.5547 | 1 | 1091.4619 | 0.0929 | 0 | 13.76 | 7.00E+02 | I | APTAVDSNS |
| 10 | VP13C_HUM | Vacuolar pro | 7 | 424462 | 1 | 1 | 5 | 0 | 1092.562 | 1091.5547 | 1 | 1090.5659 | 0.9889 | 0 | 16.53 | 3.70E+02 | T | KADAPALTAS |

Biotools-Score: 24

MASCOT-Score: 19

known O-glycosylation site

Plasminogen

$_{363}$APTAPPELTPV$_{373}$

# Fraction 14

583.58+++ → Pep [M+H]+ 1092.56+ [25.6-26.1 min] ETD

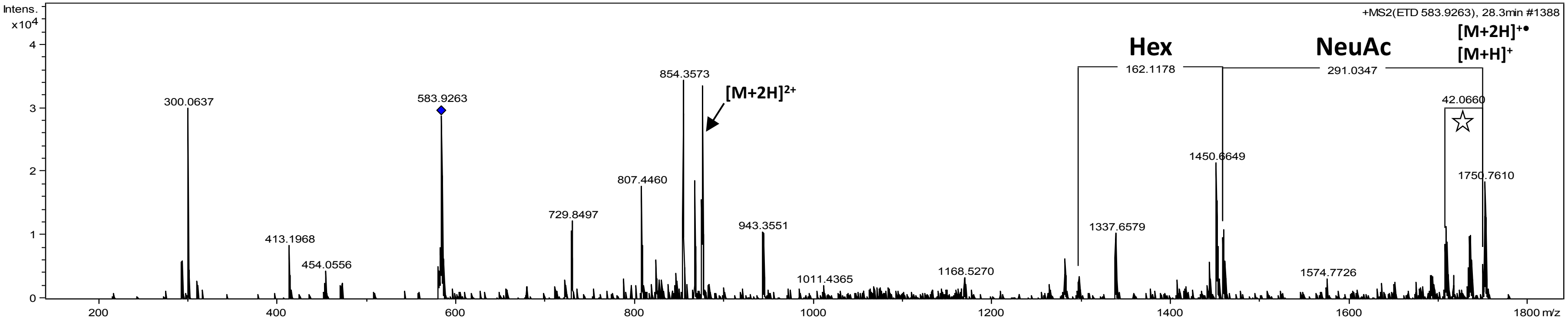


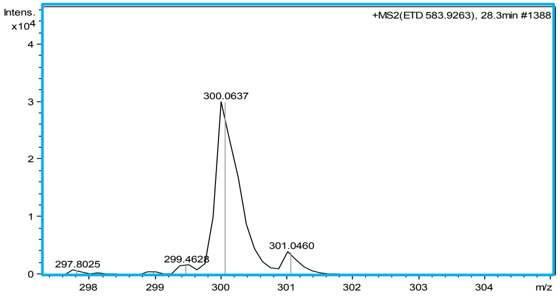


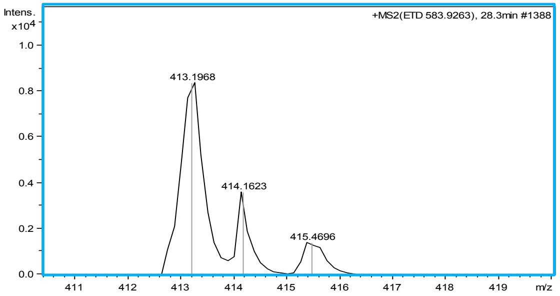


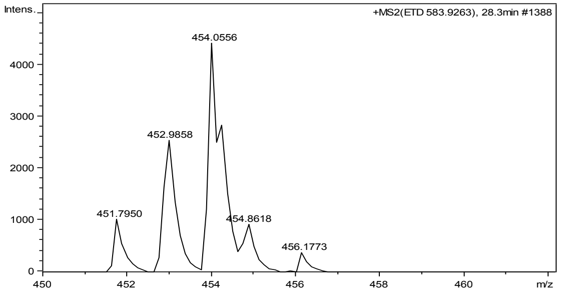


known O-glycosylation site

Plasminogen

$_{363}$APTAPPELTPV$_{373}$

# Fraction 14

## 583.58+++ → Pep [M+H]+ 1092.56+ [25.6-26.1 min] ETD

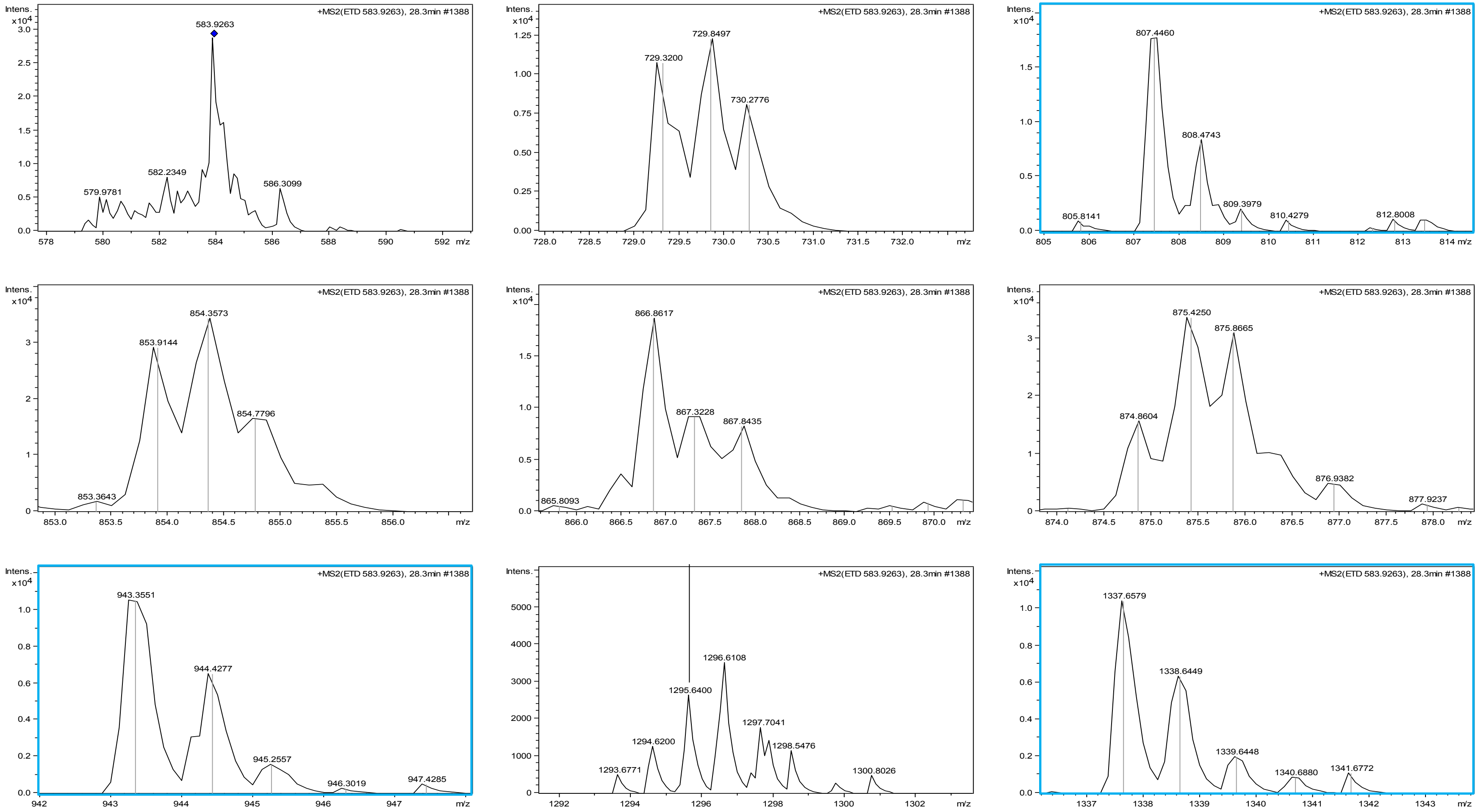


known O-glycosylation site

Plasminogen

$_{363}$AP**T**APPELTPV$_{373}$

# Fraction 14

583.58+++ → Pep [M+H]+ 1092.56+ [25.6-26.1 min] ETD

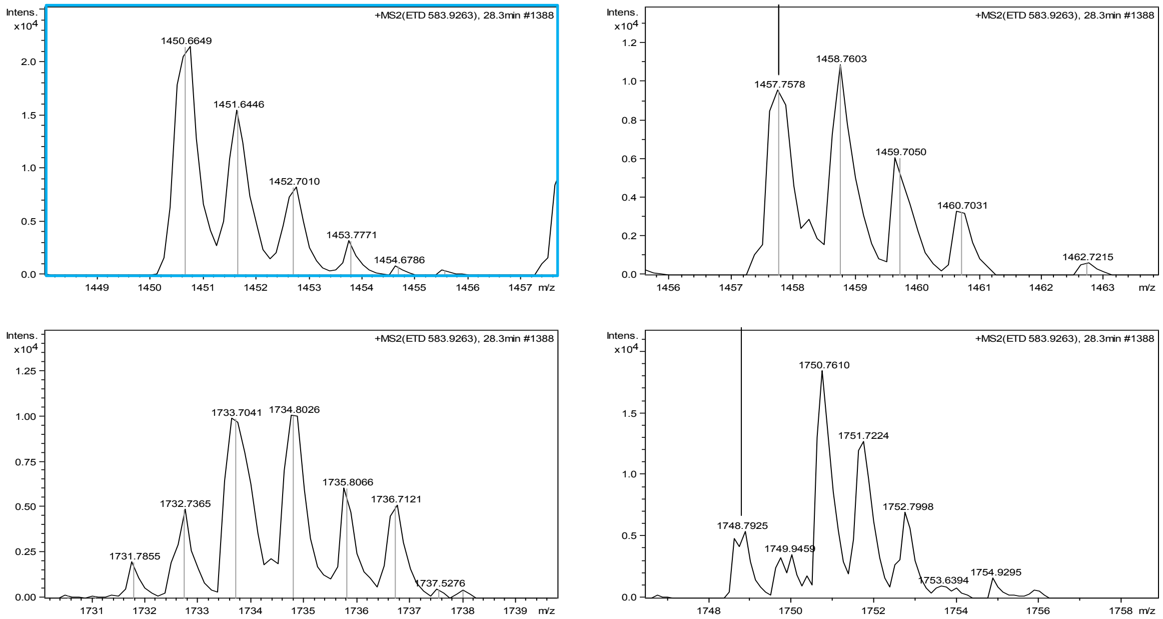


known O-glycosylation site

Plasminogen

$_{363}$AP**T**APPELTPV$_{373}$

# Fraction 14

583.58+++ → Pep [M+H]+ 1092.56+ [25.6-26.1 min] ETD

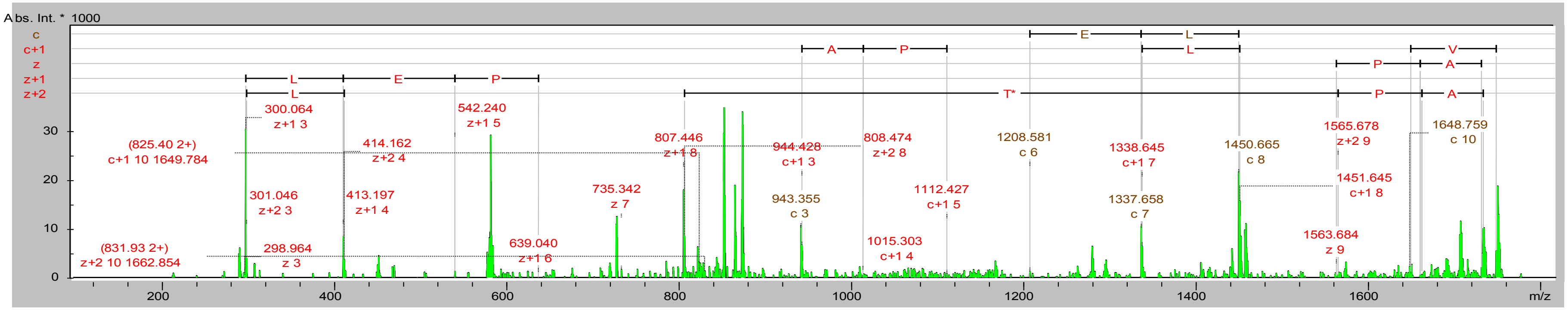


APTAPPELTPV

| | A | P | T | A | P | P | E | L | T | P | V | Ala | Pro | Thr | Ala | Pro | Pro | Glu | Leu | Thr | Pro | Val |
|---|---|---|---|---|---|---|---|---|---|---|---|---|---|---|---|---|---|---|---|---|---|---|
| Ion | 1 | 2 | 3 | 4 | 5 | 6 | 7 | 8 | 9 | 10 | 11 | 1 | 2 | 3 | 4 | 5 | 6 | 7 | 8 | 9 | 10 | 11 |
| c | A | P | T* | A | P | P | E | L | T | P | V | 89.071 | 186.124 | 943.399 | 1014.436 | 1111.489 | 1208.542 | 1337.584 | 1450.668 | 1551.716 | 1648.769 | 1747.837 |
| c+1 | A | P | T* | A | P | P | E | L | T | P | V | 90.079 | 187.132 | 944.407 | 1015.444 | 1112.497 | 1209.549 | 1338.592 | 1451.676 | 1552.724 | 1649.777 | 1748.845 |
| z | A | P | T* | A | P | P | E | L | T | P | V | 101.060 | 198.112 | 299.160 | 412.244 | 541.287 | 638.340 | 735.392 | 806.429 | 1563.705 | 1660.758 | 1731.795 |
| z+1 | A | P | T* | A | P | P | E | L | T | P | V | 102.068 | 199.120 | 300.168 | 413.252 | 542.295 | 639.347 | 736.400 | 807.437 | 1564.713 | 1661.765 | 1732.802 |
| z+2 | A | P | T* | A | P | P | E | L | T | P | V | 103.075 | 200.128 | 301.176 | 414.260 | 543.302 | 640.355 | 737.408 | 808.445 | 1565.720 | 1662.773 | 1733.810 |
| | 11 | 10 | 9 | 8 | 7 | 6 | 5 | 4 | 3 | 2 | 1 | Val | Pro | Thr | Leu | Glu | Pro | Pro | Ala | Thr | Pro | Ala |

Biotools-Score: 65

known O-glycosylation site

Plasminogen

$_{363}$APTAPPELTPV$_{373}$

# Fraction 14

583.58+++ → Pep [M+H]+ 1092.56+ [25.6-26.1 min] ETD

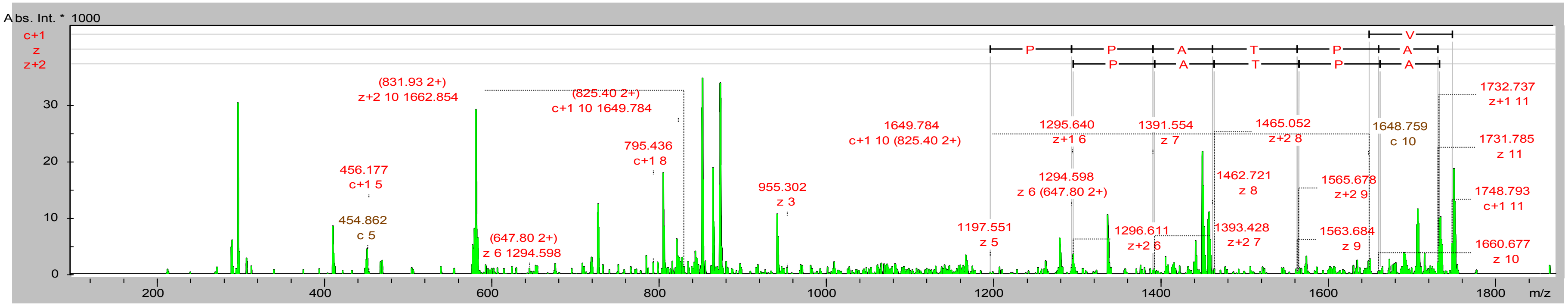


APTAPPELTPV

| | A | P | T | A | P | P | E | L | T | P | V | Ala | Pro | Thr | Ala | Pro | Pro | Glu | Leu | Thr | Pro | Val |
|---|---|---|---|---|---|---|---|---|---|---|---|---|---|---|---|---|---|---|---|---|---|---|
| Ion | 1 | 2 | 3 | 4 | 5 | 6 | 7 | 8 | 9 | 10 | 11 | 1 | 2 | 3 | 4 | 5 | 6 | 7 | 8 | 9 | 10 | 11 |
| c | A | P | T | A | P | P | E | L | T* | P | V | 89.071 | 186.124 | 287.171 | 358.208 | 455.261 | 552.314 | 681.357 | 794.441 | 1551.716 | 1648.769 | 1747.837 |
| c+1 | A | P | T | A | P | P | E | L | T* | P | V | 90.079 | 187.132 | 288.179 | 359.216 | 456.269 | 553.322 | 682.364 | 795.449 | 1552.724 | 1649.777 | 1748.845 |
| z | A | P | T | A | P | P | E | L | T* | P | V | 101.060 | 198.112 | 955.388 | 1068.472 | 1197.514 | 1294.567 | 1391.620 | 1462.657 | 1563.705 | 1660.758 | 1731.795 |
| z+1 | A | P | T | A | P | P | E | L | T* | P | V | 102.068 | 199.120 | 956.396 | 1069.480 | 1198.522 | 1295.575 | 1392.628 | 1463.665 | 1564.713 | 1661.765 | 1732.802 |
| z+2 | A | P | T | A | P | P | E | L | T* | P | V | 103.075 | 200.128 | 957.403 | 1070.487 | 1199.530 | 1296.583 | 1393.636 | 1464.673 | 1565.720 | 1662.773 | 1733.810 |
| | 11 | 10 | 9 | 8 | 7 | 6 | 5 | 4 | 3 | 2 | 1 | Val | Pro | Thr | Leu | Glu | Pro | Pro | Ala | Thr | Pro | Ala |

Biotools-Score: 199

Annotation is not accurate

known O-glycosylation site

Plasminogen

$_{363}$AP**T**APPELTPV$_{373}$

# Fraction 14

650.71++ → Pep [M+H]+ 644.35+ [11.7-11.9 min]

CID-MS Precursor

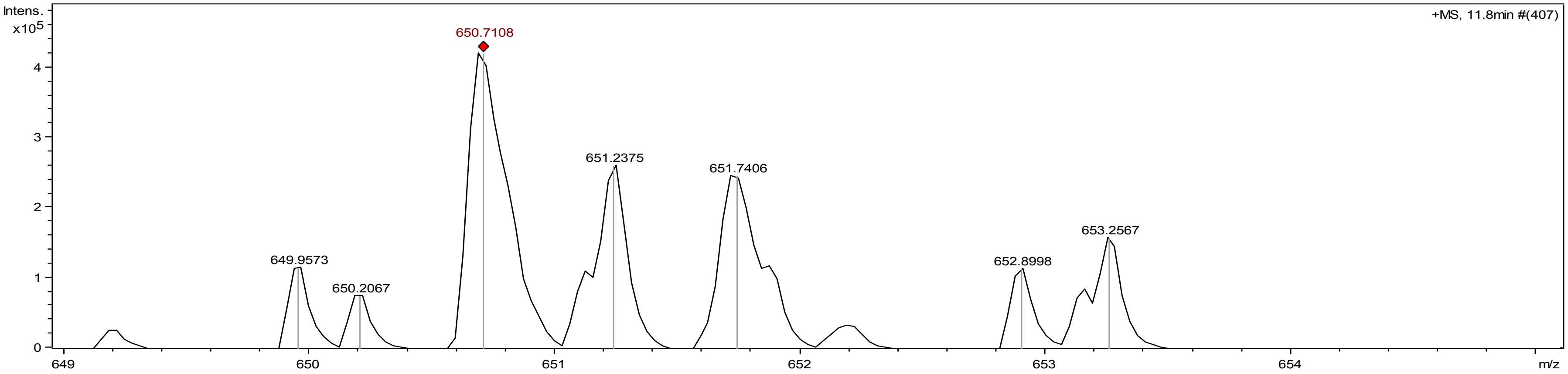


CID-MS3³ spectrum of poor quality

ETD spectrum of poor quality

# Fraction 14

650.71++ → Pep [M+H]+ 644.35+ [11.7-11.9 min] CID-MS2

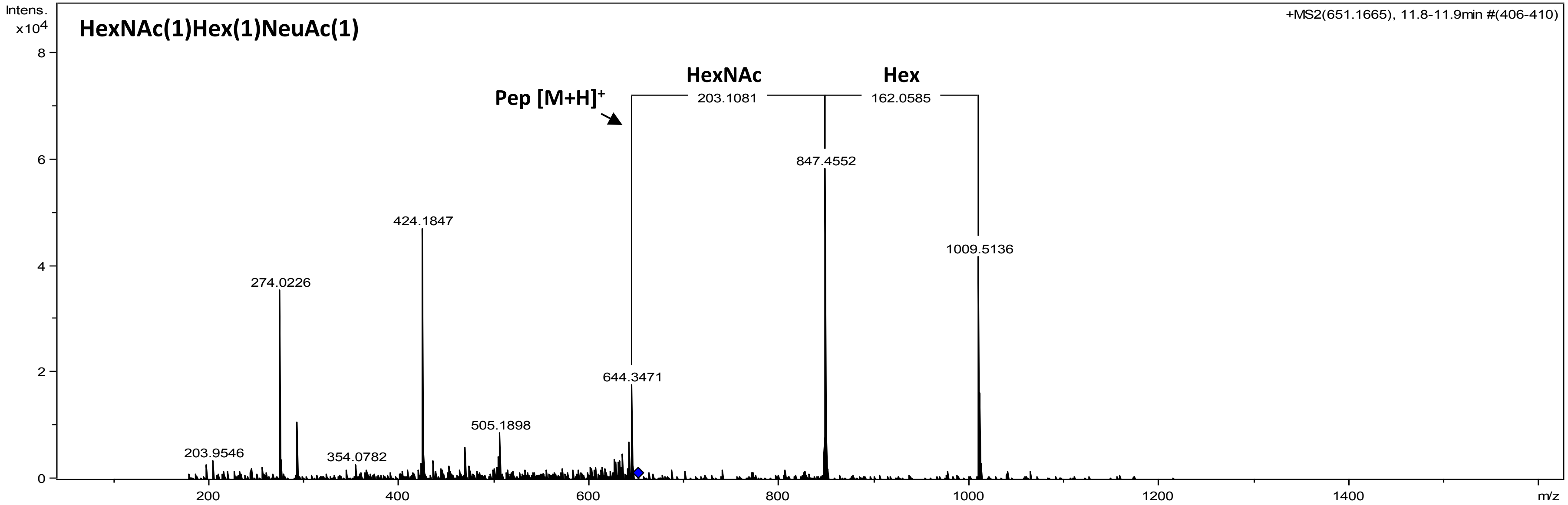

# Fraction 14

650.71++ → Pep [M+H]+ 644.35+ [11.7-11.9 min] CID-MS3

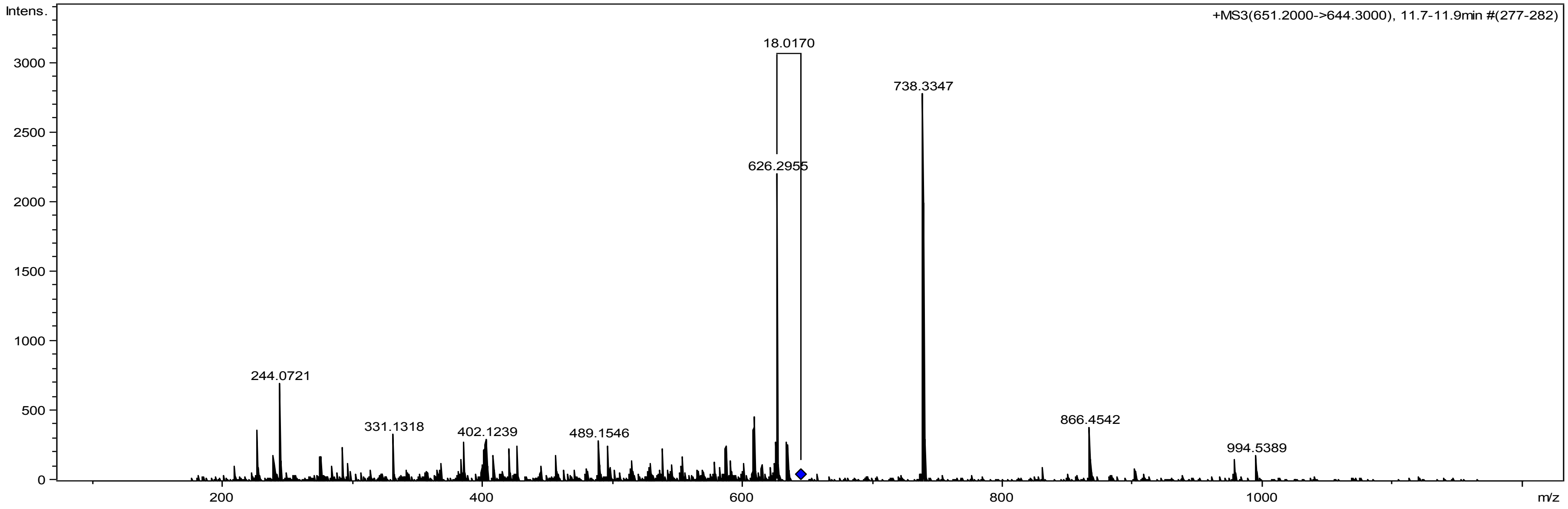

# Fraction 14

657.20++ → Pep+HexNAc [M+H]+ 860.37+ [14.0-14.1 min] CID-MS Precursor

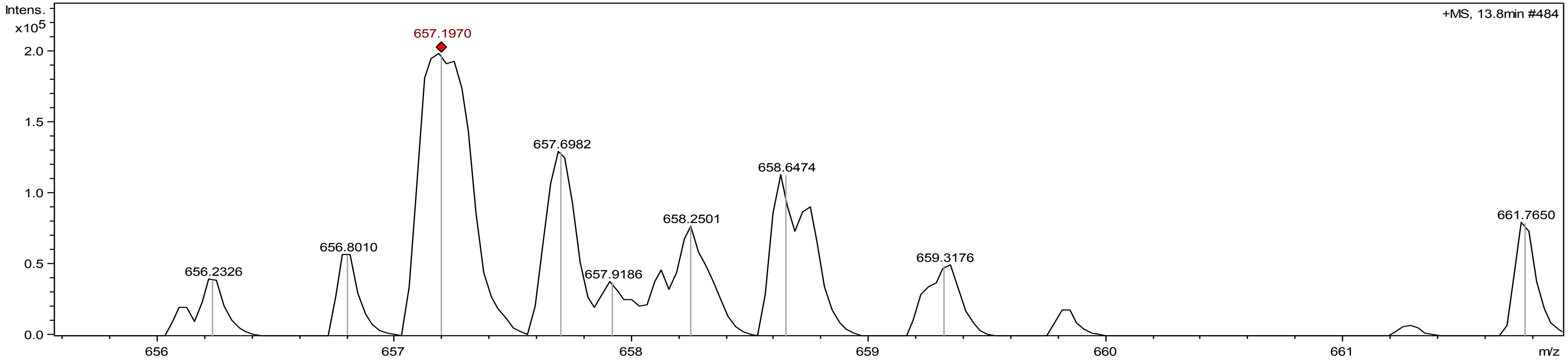


ETD spectrum with poor quality

# Fraction 14

## 657.20++ → Pep+HexNAc [M+H]+ 860.37+ [14.0-14.1 min] CID-MS2

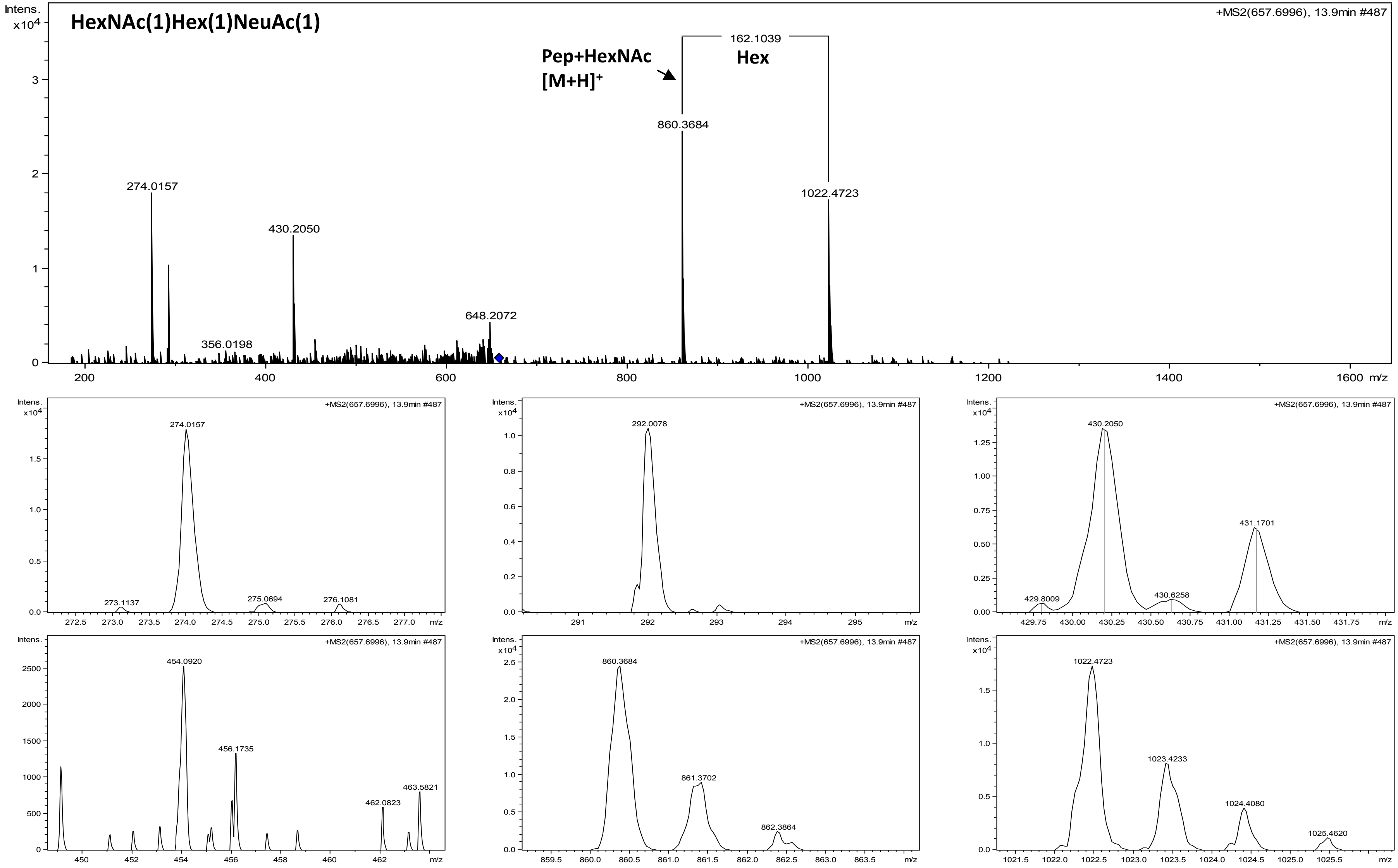

# Fraction 14

657.20++ → Pep+HexNAc [M+H]+ 860.37+ [14.0-14.1 min]

CID-MS3 Manual DeNovo

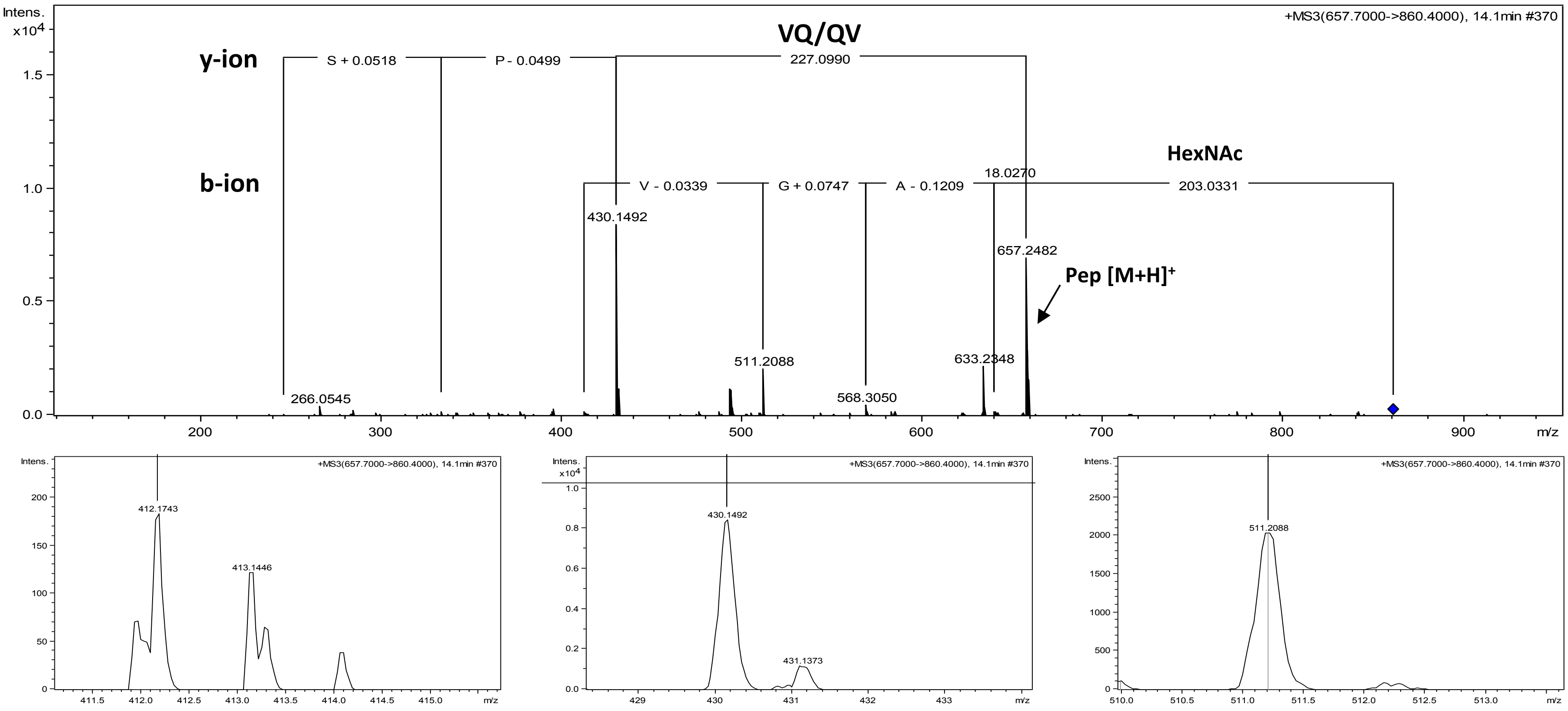


known O-glycosylation site

Alpha-2-HS-glycoprotein precursor

$_{343}$VQP**S**VGA$_{349}$

# Fraction 14

657.20++ → Pep+HexNAc [M+H]+ 860.37+ [14.0-14.1 min] CID-MS3

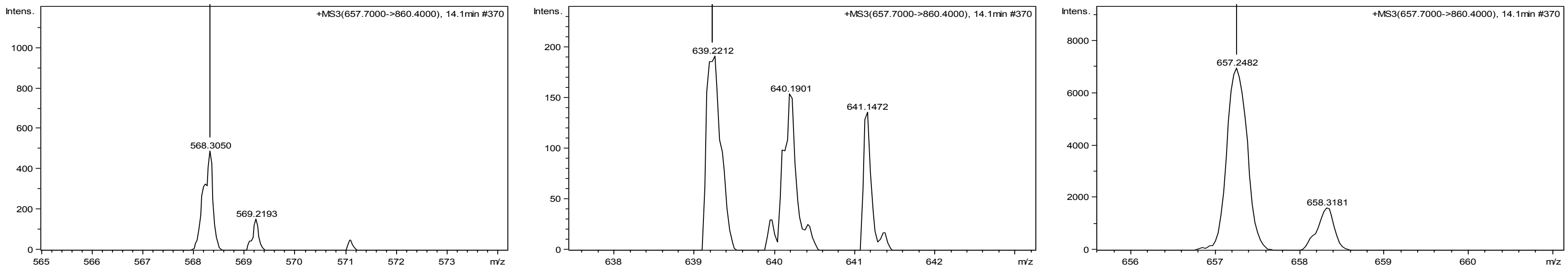


known O-glycosylation site

Alpha-2-HS-glycoprotein precursor $_{343}$VQP**S**VGA$_{349}$

# Fraction 14

657.20++ → Pep+HexNAc [M+H]+ 860.37+ [14.0-14.1 min]

CID-MS3 MASCOT-Search

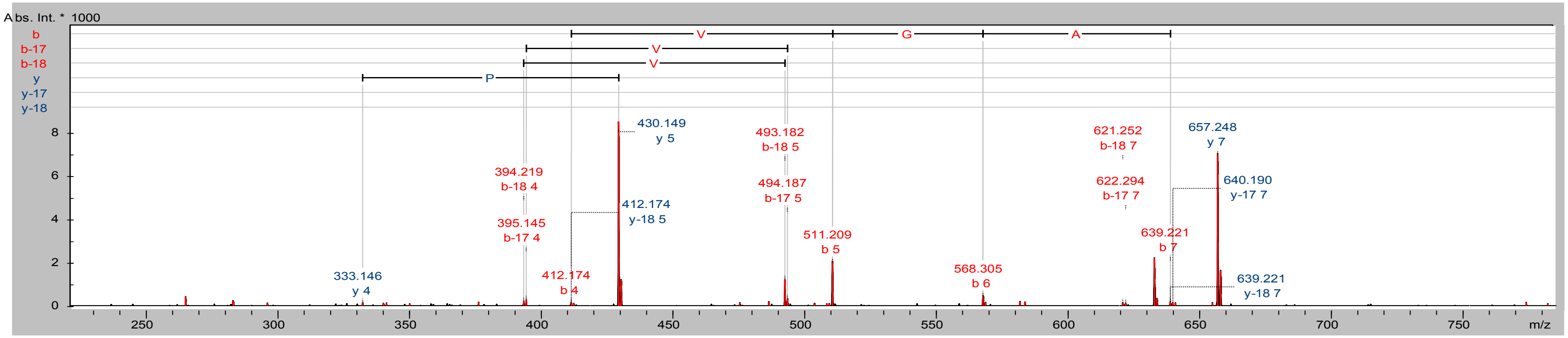


| | V | Q | P | S | V | G | A | Val | Gln | Pro | Ser | Val | Gly | Ala |
|---|---|---|---|---|---|---|---|---|---|---|---|---|---|---|
| Ion | 1 | 2 | 3 | 4 | 5 | 6 | 7 | 1 | 2 | 3 | 4 | 5 | 6 | 7 |
| b | V | Q | P | S | V | G | A | 100.076 | 228.134 | 325.187 | 412.219 | 511.287 | 568.309 | 639.346 |
| b-17 | V | Q | P | S | V | G | A | - | 211.108 | 308.160 | 395.193 | 494.261 | 551.282 | 622.320 |
| b-18 | V | Q | P | S | V | G | A | - | - | - | 394.208 | 493.277 | 550.298 | 621.335 |
| y | V | Q | P | S | V | G | A | 90.055 | 147.076 | 246.145 | 333.177 | 430.230 | 558.288 | 657.357 |
| y-17 | V | Q | P | S | V | G | A | - | - | - | - | - | 541.262 | 640.330 |
| y-18 | V | Q | P | S | V | G | A | - | - | - | 315.166 | 412.219 | 540.278 | 639.346 |
| | 7 | 6 | 5 | 4 | 3 | 2 | 1 | Ala | Gly | Val | Ser | Pro | Gln | Val |

| prot_hit_nur | prot_acc | prot_desc | prot_score | prot_mass | prot_matche | pep_query | pep_rank | pep_isbold | pep_exp_mz | pep_exp_mr | pep_exp_z | pep_calc_mr | pep_delta | pep_miss | pep_score | pep_expect | pep_res_bef | pep_seq |
|---|---|---|---|---|---|---|---|---|---|---|---|---|---|---|---|---|---|---|
| 1 | FETUA_HUM | Alpha-2-HS-g | 15 | 40098 | 1 | 1 | 3 | 1 | 657.2482 | 656.2409 | 1 | 656.3493 | -0.1084 | 0 | 19.55 | 1.10E+02 | V | VQPSVGA |
| 2 | DOT1L_HUM | Histone-lysir | 13 | 185538 | 1 | 1 | 1 | 0 | 657.2482 | 656.2409 | 1 | 656.3493 | -0.1084 | 0 | 20.17 | 94 | G | PEGKVAG |
| 3 | TACC2_HUM | Transforming | 12 | 311632 | 1 | 1 | 1 | 0 | 657.2482 | 656.2409 | 1 | 656.3493 | -0.1084 | 0 | 20.17 | 94 | L | EPGKVAG |
| 4 | TEAD2_HUM | Transcriptior | 12 | 49554 | 1 | 1 | 4 | 0 | 657.2482 | 656.2409 | 1 | 656.3242 | -0.0832 | 0 | 16.12 | 2.40E+02 | M | GEPRAGA |
| 5 | NOXO1_HUN | NADPH oxid | 11 | 41627 | 1 | 1 | 4 | 0 | 657.2482 | 656.2409 | 1 | 656.3493 | -0.1084 | 0 | 16.12 | 2.40E+02 | Y | PVSVQGA |
| 6 | HCN4_HUMA | Potassium/s | 11 | 129645 | 1 | 1 | 4 | 0 | 657.2482 | 656.2409 | 1 | 656.3242 | -0.0832 | 0 | 16.12 | 2.40E+02 | S | EGPARGA |
| 7 | A20A1_HUM | Ankyrin repe | 10 | 94902 | 1 | 1 | 4 | 0 | 657.2482 | 656.2409 | 1 | 656.3493 | -0.1084 | 0 | 16.12 | 2.40E+02 | E | DPAVKGA |
| 8 | GLTL3_HUM | Putative poly | 10 | 68450 | 1 | 1 | 9 | 0 | 657.2482 | 656.2409 | 1 | 656.3857 | -0.1448 | 0 | 15.33 | 2.90E+02 | L | NLIAVAG |
| 9 | PACS2_HUM | Phosphofuri | 10 | 98440 | 1 | 1 | 9 | 0 | 657.2482 | 656.2409 | 1 | 656.4221 | -0.1812 | 0 | 15.33 | 2.90E+02 | P | VKIAVAG |
| 10 | CF167_HUM | Uncharacteri | 10 | 144106 | 1 | 1 | 4 | 0 | 657.2482 | 656.2409 | 1 | 656.3493 | -0.1084 | 0 | 16.12 | 2.40E+02 | L | PSVNIGA |

known O-glycosylation site

Alpha-2-HS-glycoprotein precursor

$_{343}$VQP**S**VGA$_{349}$

# Fraction 14

657.20++ → Pep+HexNAc [M+H]+ 860.37+ [14.0-14.1 min]

CID-MS3 MASCOT-Search

| prot_hit_nur | prot_acc | prot_desc | prot_score | prot_mass | prot_matche | pep_query | pep_rank | pep_isbold | pep_exp_mz | pep_exp_mr | pep_exp_z | pep_calc_mr | pep_delta | pep_miss | pep_score | pep_expect | pep_res_bef | pep_seq |
|---|---|---|---|---|---|---|---|---|---|---|---|---|---|---|---|---|---|---|
| 1 | FETUA_HUM | Alpha-2-HS-g | 15 | 40098 | 1 | 1 | 3 | 1 | 657.2482 | 656.2409 | 1 | 656.3493 | -0.1084 | 0 | 19.55 | 1.10E+02 | V | VQPSVGA |
| 2 | DOT1L_HUM | Histone-lysir | 13 | 185538 | 1 | 1 | 1 | 0 | 657.2482 | 656.2409 | 1 | 656.3493 | -0.1084 | 0 | 20.17 | 94 | G | PEGKVAG |
| 3 | TACC2_HUM | Transforming | 12 | 311632 | 1 | 1 | 1 | 0 | 657.2482 | 656.2409 | 1 | 656.3493 | -0.1084 | 0 | 20.17 | 94 | L | EPGKVAG |
| 4 | TEAD2_HUM | Transcriptior | 12 | 49554 | 1 | 1 | 4 | 0 | 657.2482 | 656.2409 | 1 | 656.3242 | -0.0832 | 0 | 16.12 | 2.40E+02 | M | GEPRAGA |
| 5 | NOXO1_HUN | NADPH oxid | 11 | 41627 | 1 | 1 | 4 | 0 | 657.2482 | 656.2409 | 1 | 656.3493 | -0.1084 | 0 | 16.12 | 2.40E+02 | Y | PVSVQGA |
| 6 | HCN4_HUMA | Potassium/s | 11 | 129645 | 1 | 1 | 4 | 0 | 657.2482 | 656.2409 | 1 | 656.3242 | -0.0832 | 0 | 16.12 | 2.40E+02 | S | EGPARGA |
| 7 | A20A1_HUM | Ankyrin repe | 10 | 94902 | 1 | 1 | 4 | 0 | 657.2482 | 656.2409 | 1 | 656.3493 | -0.1084 | 0 | 16.12 | 2.40E+02 | E | DPAVKGA |
| 8 | GLTL3_HUMA | Putative poly | 10 | 68450 | 1 | 1 | 9 | 0 | 657.2482 | 656.2409 | 1 | 656.3857 | -0.1448 | 0 | 15.33 | 2.90E+02 | L | NLIAVAG |
| 9 | PACS2_HUM | Phosphofuri | 10 | 98440 | 1 | 1 | 9 | 0 | 657.2482 | 656.2409 | 1 | 656.4221 | -0.1812 | 0 | 15.33 | 2.90E+02 | P | VKIAVAG |
| 10 | CF167_HUMA | Uncharacteri | 10 | 144106 | 1 | 1 | 4 | 0 | 657.2482 | 656.2409 | 1 | 656.3493 | -0.1084 | 0 | 16.12 | 2.40E+02 | L | PSVNIGA |

Biotools-Score: 15

MASCOT-Score: 20

known O-glycosylation site

Alpha-2-HS-glycoprotein precursor

$_{343}$VQP**S**VGA$_{349}$

# Fraction 14

641.27++ → Pep+HexNac [M+H]+ 828.42+ [15.5-15.9 min]

CID-MS Precursor

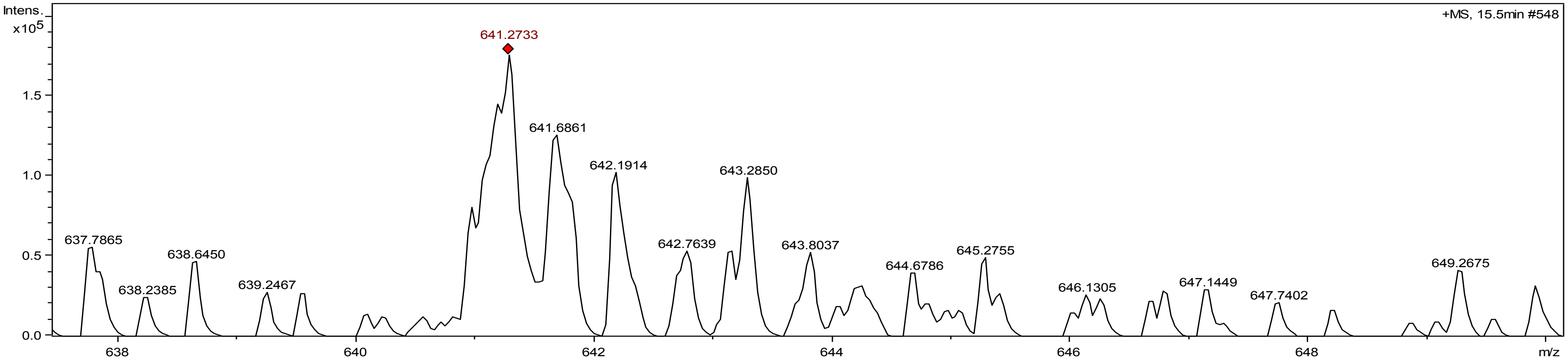


CID-MS³ spectrum of poor quality

ETD spectrum of poor quality

# Fraction 14

641.27++ → Pep+HexNac [M+H]+ 828.42+ [15.5-15.9 min] CID-MS2

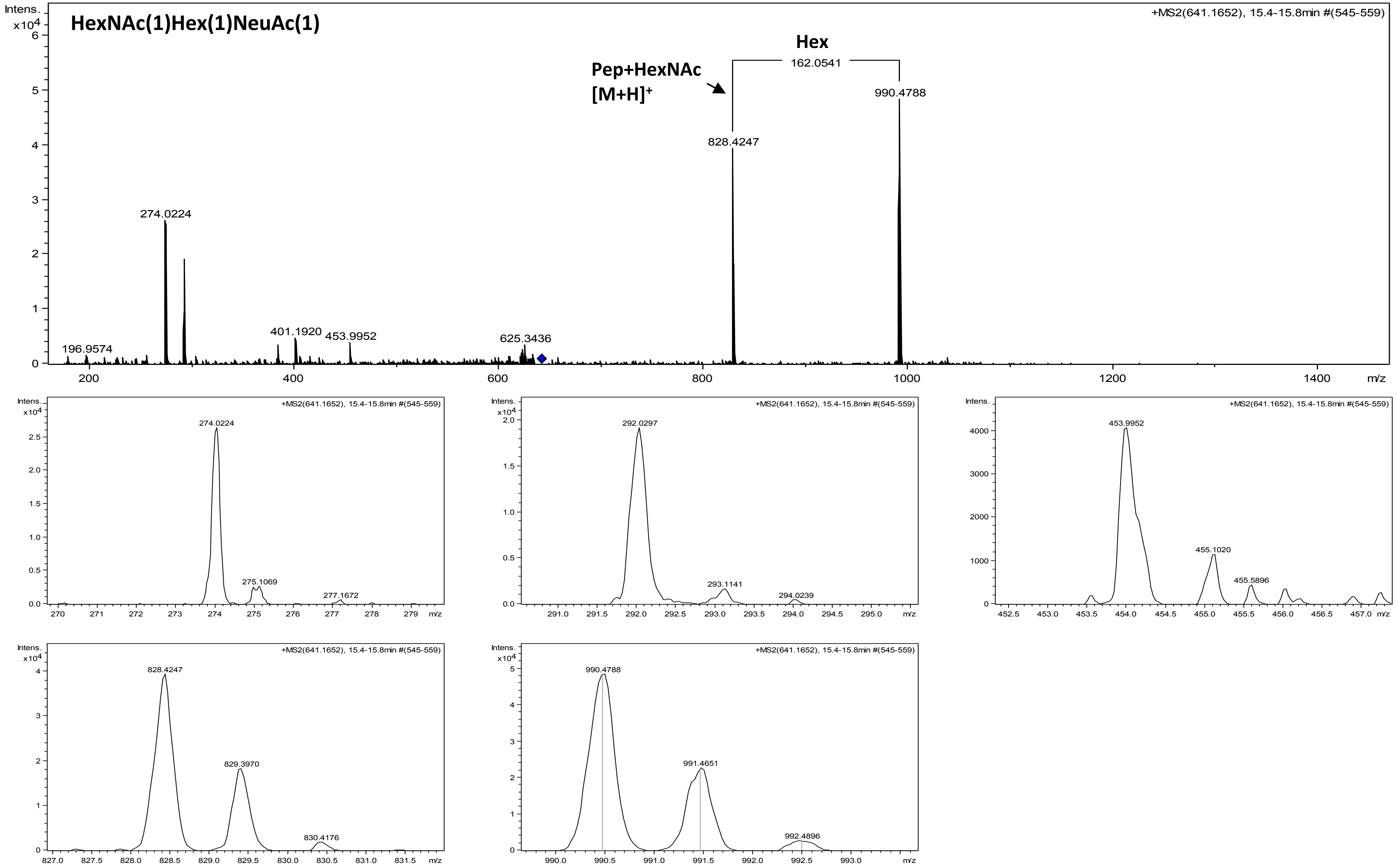

# Fraction 14

641.27++ → Pep+HexNac [M+H]+ 828.42+ [15.5-15.9 min] CID-MS3

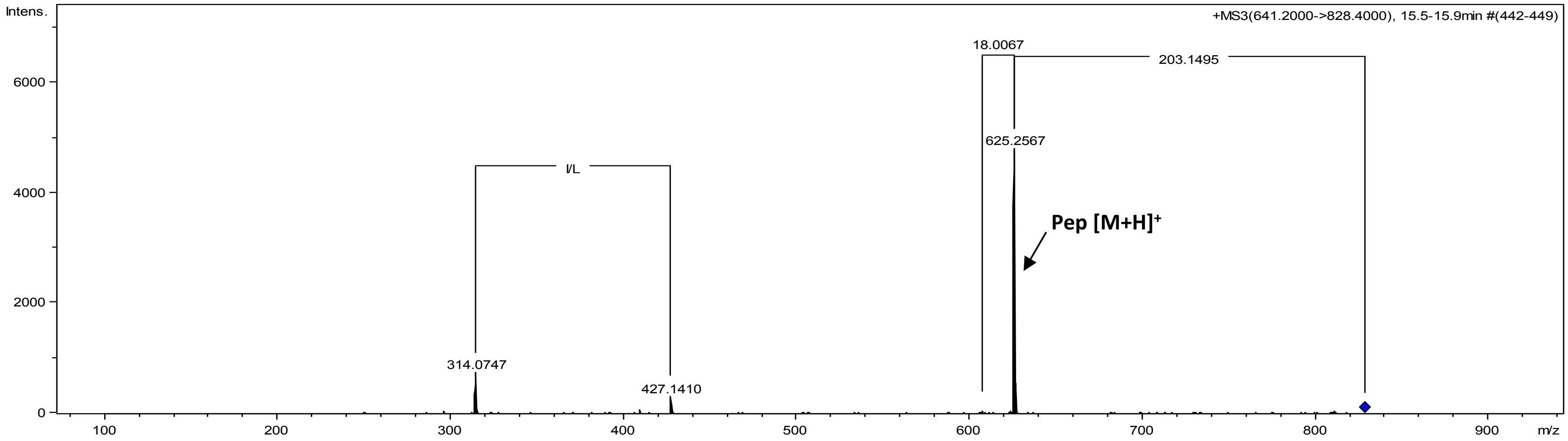


Only a few peptide fragments; major fragment is derived from glycan moiety

# Fraction 14

721.76++ → Pep [M+H]+ 786.43+ [16.7-16.8 min]

CID-MS Precursor

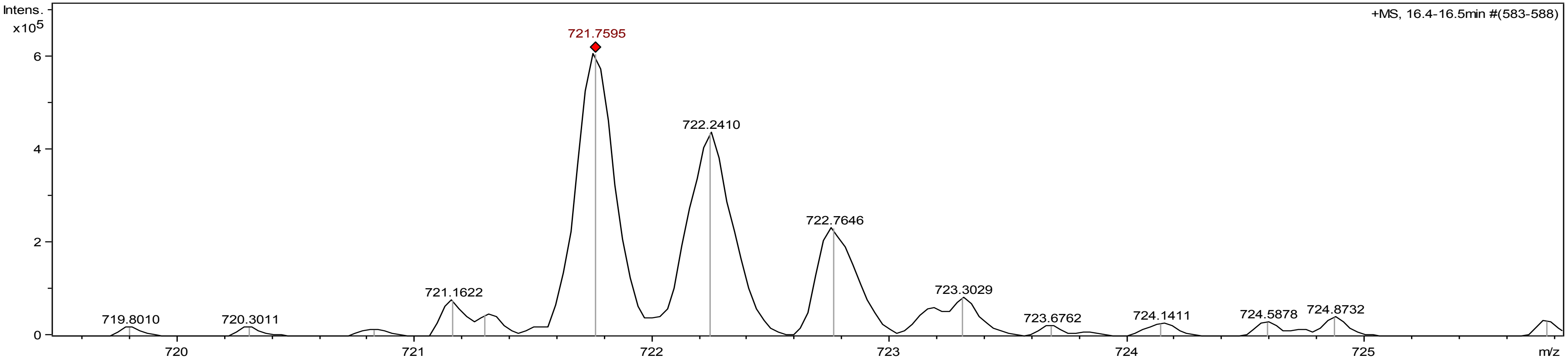


ETD spectrum of poor quality

# Fraction 14

721.76++ → Pep [M+H]+ 786.43+ [16.7-16.8 min] CID-MS2

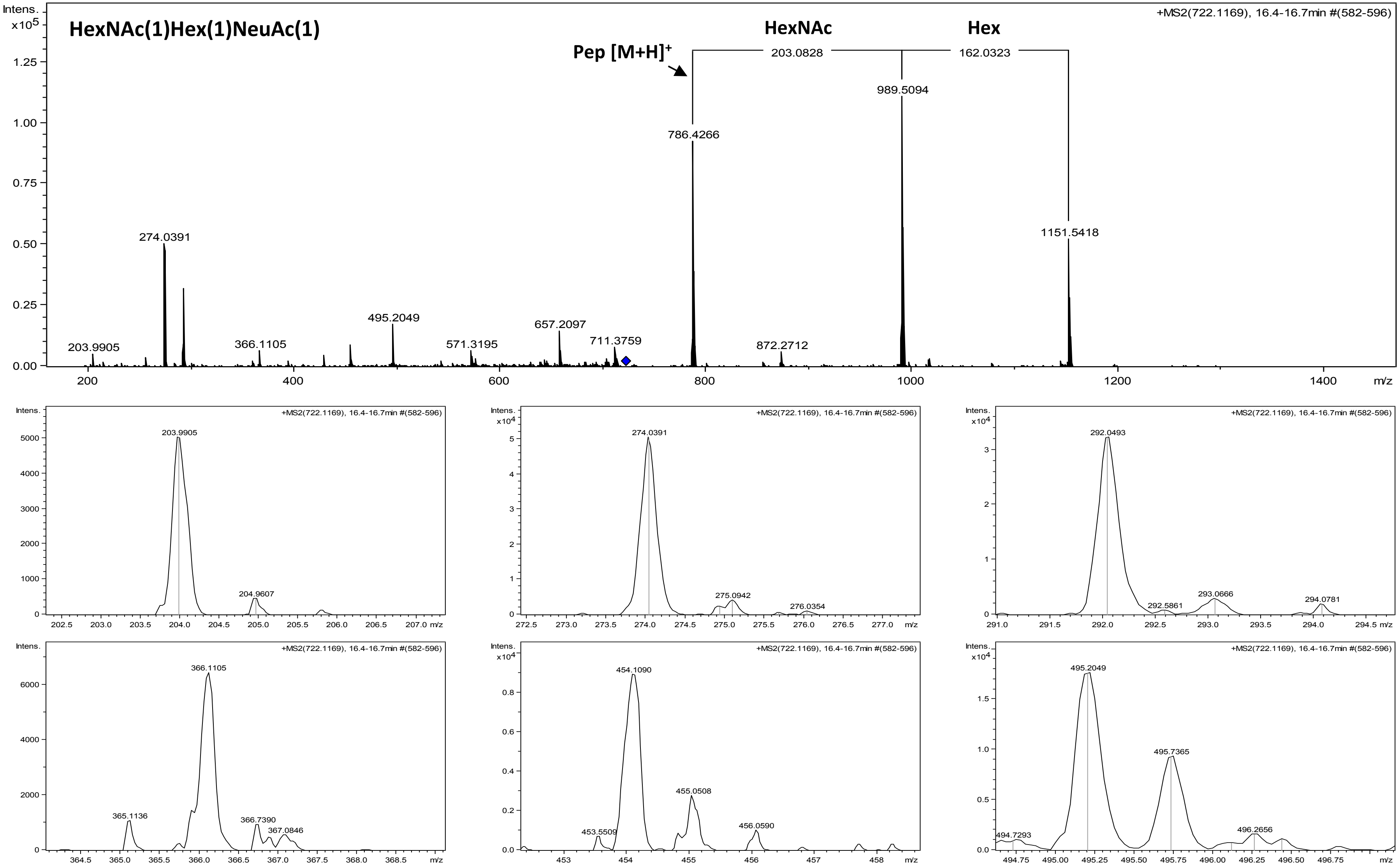

# Fraction 14

## 721.76++ → Pep [M+H]+ 786.43+ [16.7-16.8 min] CID-MS2

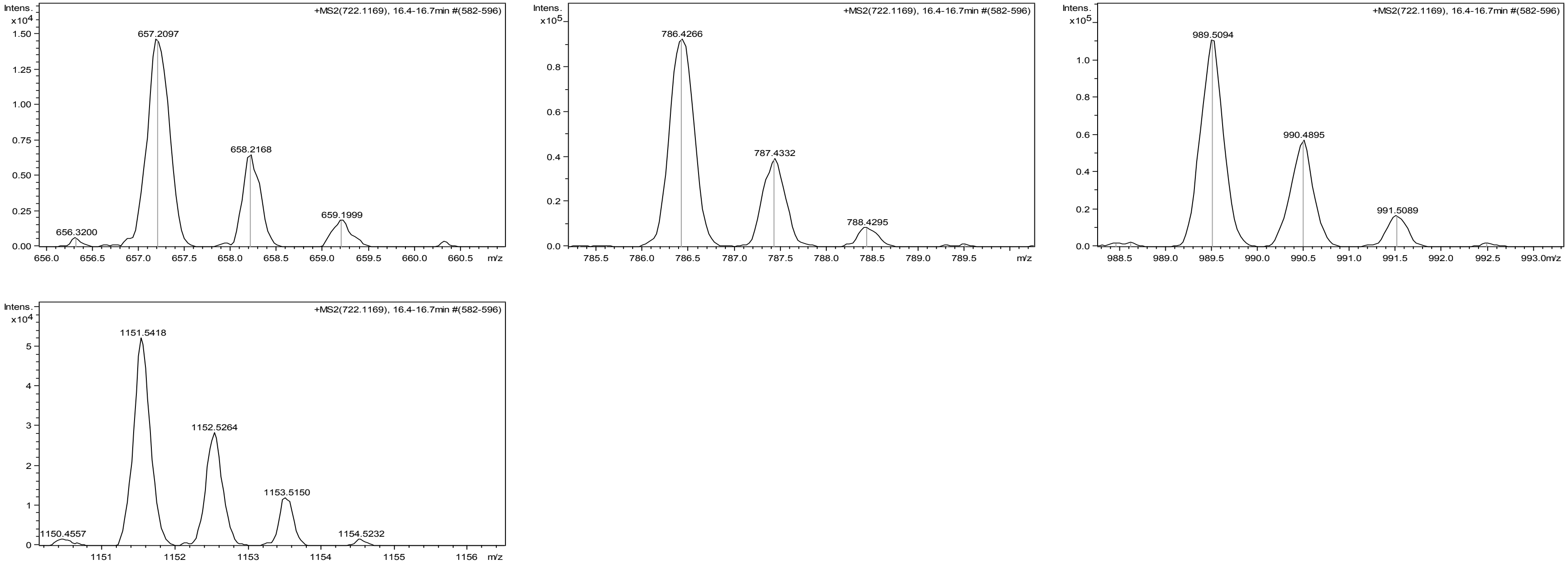

# Fraction 14

721.76++ → Pep [M+H]+ 786.43+ [16.7-16.8 min] Manual DeNovo

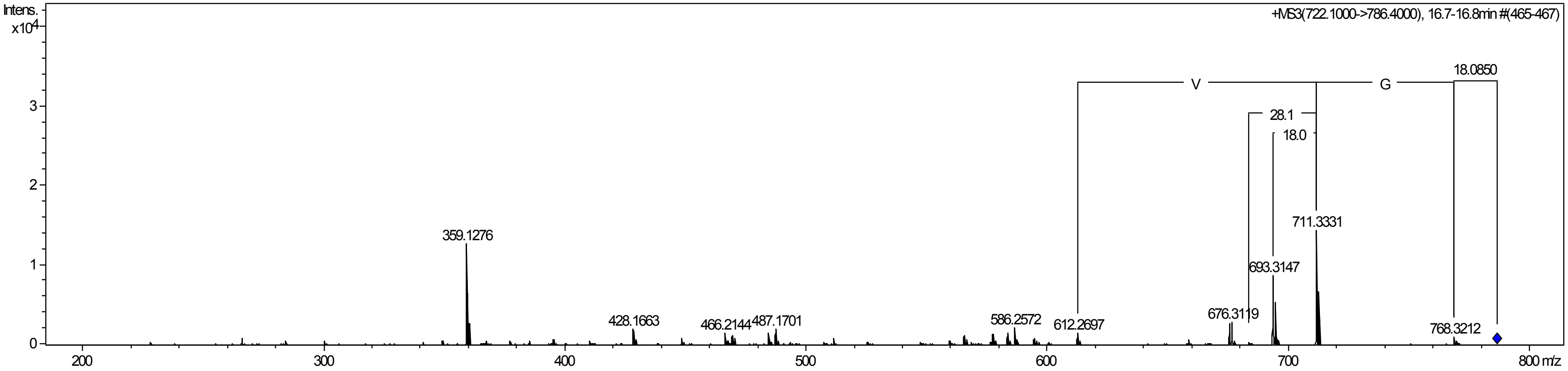

# Fraction 14

721.76++ → Pep [M+H]+ 786.43+ [16.7-16.8 min] CID-MS3

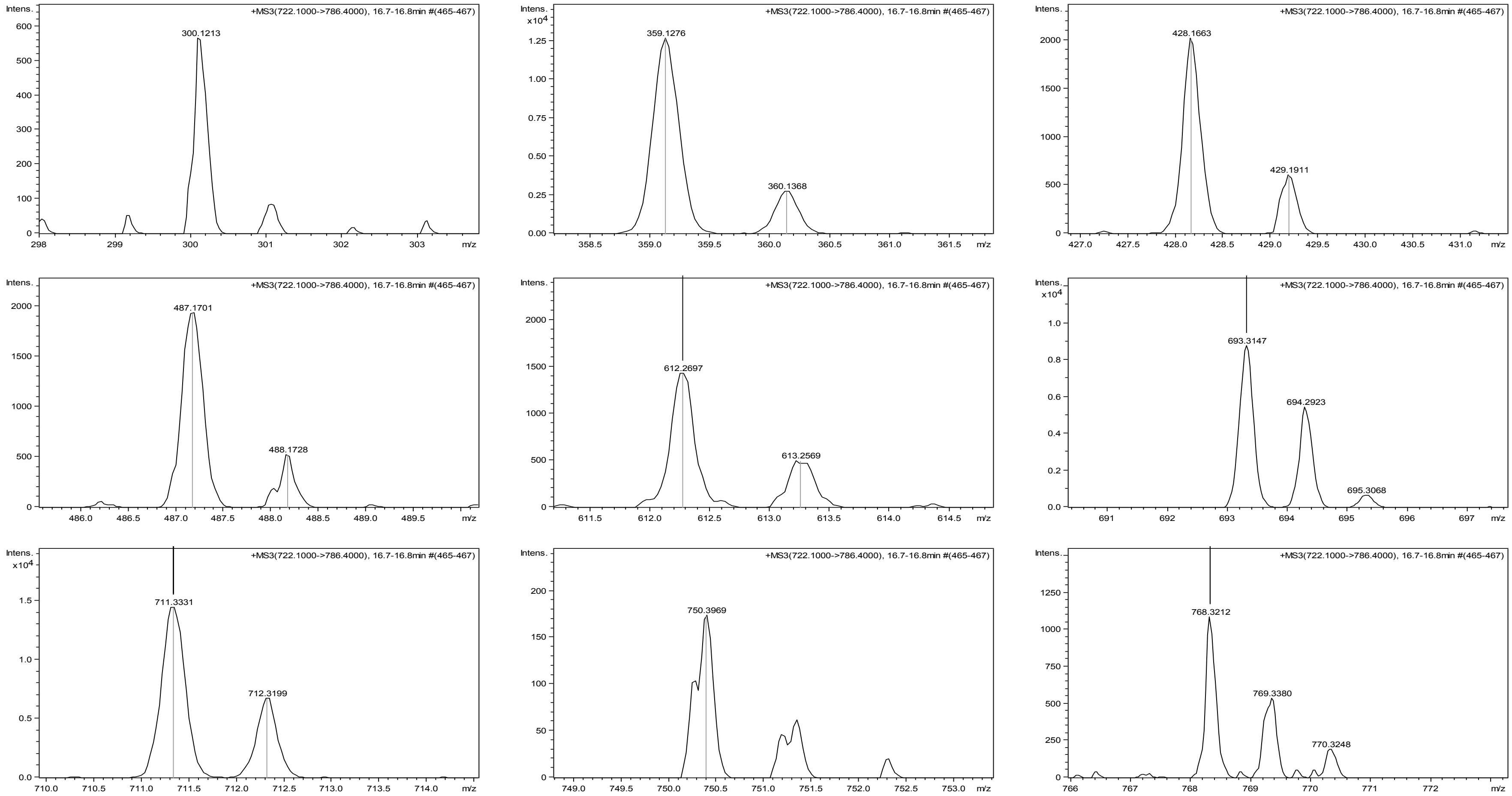

# Fraction 14

721.76++ → Pep [M+H]+ 786.43+ [16.7-16.8 min]

CID-MS3 MASCOT Search

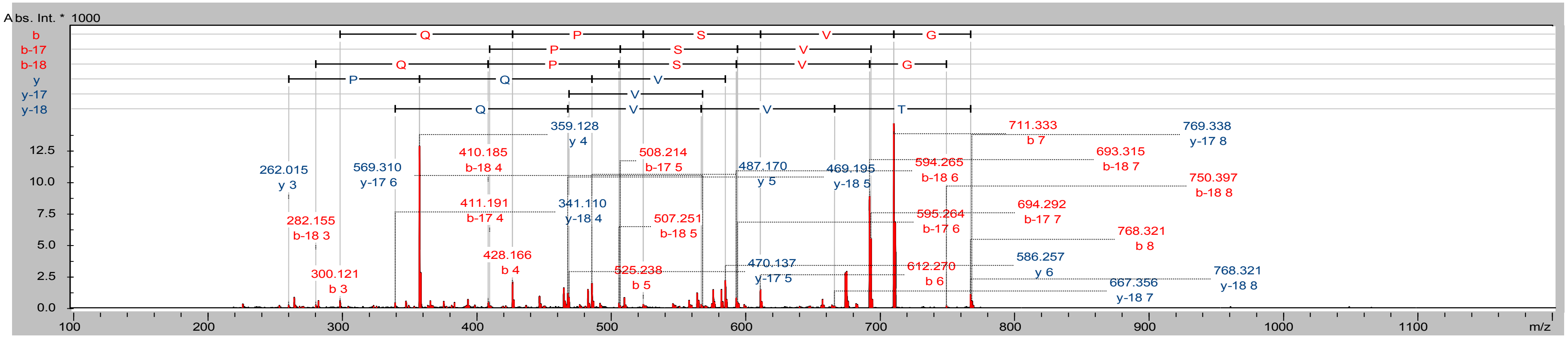


| | T | V | V | Q | P | S | V | G | Thr | Val | Val | Gln | Pro | Ser | Val | Gly |
|---|---|---|---|---|---|---|---|---|---|---|---|---|---|---|---|---|
| Ion | 1 | 2 | 3 | 4 | 5 | 6 | 7 | 8 | 1 | 2 | 3 | 4 | 5 | 6 | 7 | 8 |
| b | T | V | V | Q | P | S | V | G | 102.055 | 201.123 | 300.192 | 428.250 | 525.303 | 612.335 | 711.404 | 768.425 |
| b-17 | T | V | V | Q | P | S | V | G | - | - | - | 411.224 | 508.277 | 595.309 | 694.377 | 751.398 |
| b-18 | T | V | V | Q | P | S | V | G | 84.044 | 183.113 | 282.181 | 410.240 | 507.293 | 594.325 | 693.393 | 750.414 |
| y | T | V | V | Q | P | S | V | G | 76.039 | 175.108 | 262.140 | 359.193 | 487.251 | 586.320 | 685.388 | 786.436 |
| y-17 | T | V | V | Q | P | S | V | G | - | - | - | - | 470.225 | 569.293 | 668.361 | 769.409 |
| y-18 | T | V | V | Q | P | S | V | G | - | - | 244.129 | 341.182 | 469.241 | 568.309 | 667.377 | 768.425 |
| | 8 | 7 | 6 | 5 | 4 | 3 | 2 | 1 | Gly | Val | Ser | Pro | Gln | Val | Val | Thr |

known O-glycosylation site

Alpha-2-HS-glycoprotein precursor

$_{341}$TVVQP**S**VG$_{348}$

# Fraction 14

721.76++ → Pep [M+H]+ 786.43+ [16.7-16.8 min]

CID-MS3 MASCOT Search

| prot_hit_nur | prot_acc | prot_desc | prot_score | prot_mass | prot_matche | pep_query | pep_rank | pep_isbold | pep_exp_mz | pep_exp_mr | pep_exp_z | pep_calc_mr | pep_delta | pep_miss | pep_score | pep_expect | pep_res_bef | pep_seq |
|---|---|---|---|---|---|---|---|---|---|---|---|---|---|---|---|---|---|---|
| 1 | ITBP2_HUMA | Integrin beta | 20 | 39213 | 1 | 1 | 1 | 1 | 786.4266 | 785.4193 | 1 | 785.4283 | -0.009 | 0 | 24.5 | 44 | I | SLVKADPG |
| 2 | FETUA_HUM | Alpha-2-HS-g | 19 | 40098 | 1 | 1 | 1 | 0 | 786.4266 | 785.4193 | 1 | 785.4283 | -0.009 | 0 | 24.5 | 44 | R | TVVQPSVG |
| 3 | SMC6_HUMA | Structural ma | 18 | 127216 | 1 | 1 | 1 | 0 | 786.4266 | 785.4193 | 1 | 785.3919 | 0.0274 | 0 | 24.5 | 44 | S | ISVQPGEG |
| 4 | DEN4C_HUM | DENN domai | 18 | 182536 | 1 | 1 | 1 | 0 | 786.4266 | 785.4193 | 1 | 785.4647 | -0.0454 | 0 | 24.5 | 44 | S | SIVKVPSG |
| 5 | OR3A4_HUM | Olfactory rec | 16 | 37867 | 1 | 1 | 7 | 0 | 786.4266 | 785.4193 | 1 | 785.4283 | -0.009 | 0 | 20.93 | 1.00E+02 | W | GIQQALVG |
| 6 | CNKR1_HUM | Connector en | 15 | 80512 | 1 | 1 | 5 | 0 | 786.4266 | 785.4193 | 1 | 785.3919 | 0.0274 | 0 | 21.13 | 96 | E | SPDKSPVG |
| 7 | UROK_HUMA | Urokinase-ty | 15 | 49919 | 1 | 1 | 9 | 0 | 786.4266 | 785.4193 | 1 | 785.4283 | -0.009 | 0 | 19.71 | 1.30E+02 | S | DALQLGLG |
| 8 | MEFV_HUMA | Pyrin (Maren | 14 | 87359 | 1 | 1 | 9 | 0 | 786.4266 | 785.4193 | 1 | 785.3919 | 0.0274 | 0 | 19.71 | 1.30E+02 | S | ADLKEGPG |
| 9 | ZN592_HUM | Zinc finger p | 14 | 140148 | 1 | 1 | 5 | 0 | 786.4266 | 785.4193 | 1 | 785.3919 | 0.0274 | 0 | 21.13 | 96 | D | DPSKSPVG |
| 10 | TRPC7_HUM | Short transie | 14 | 100239 | 1 | 1 | 8 | 0 | 786.4266 | 785.4193 | 1 | 785.4283 | -0.009 | 0 | 20.33 | 1.10E+02 | Q | NALQLAVG |

Biotools-Score: 149

MASCOT-Score: 25

known O-glycosylation site

Alpha-2-HS-glycoprotein precursor

$_{341}$TVVQP**S**VG$_{348}$

# Fraction 14

608.71++ → Pep [M+H]+ 560.28+ [17.7-17.8 min]

CID-MS Precursor

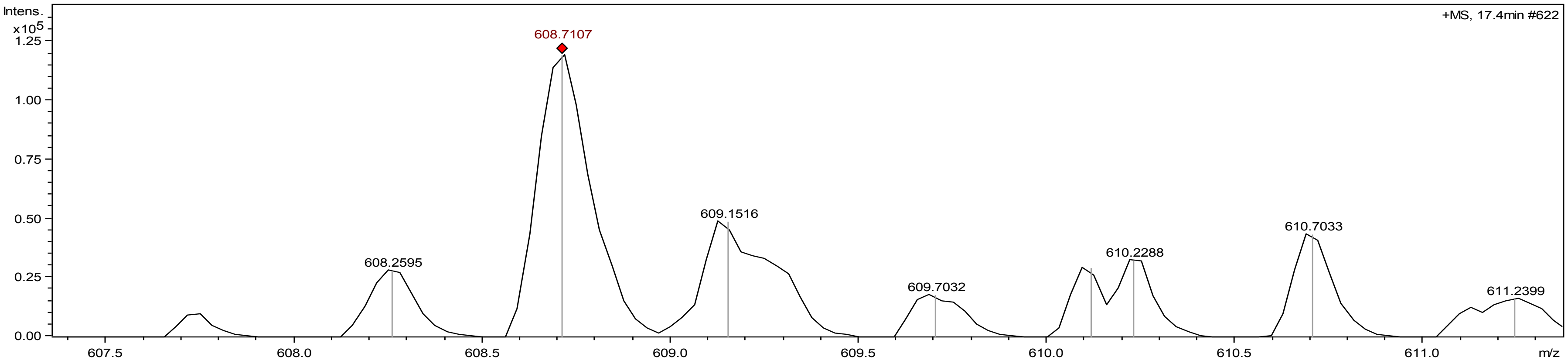


ETD spectrum not available

# Fraction 14

608.71++ → Pep [M+H]+ 560.28+ [17.7-17.8 min] CID-MS2

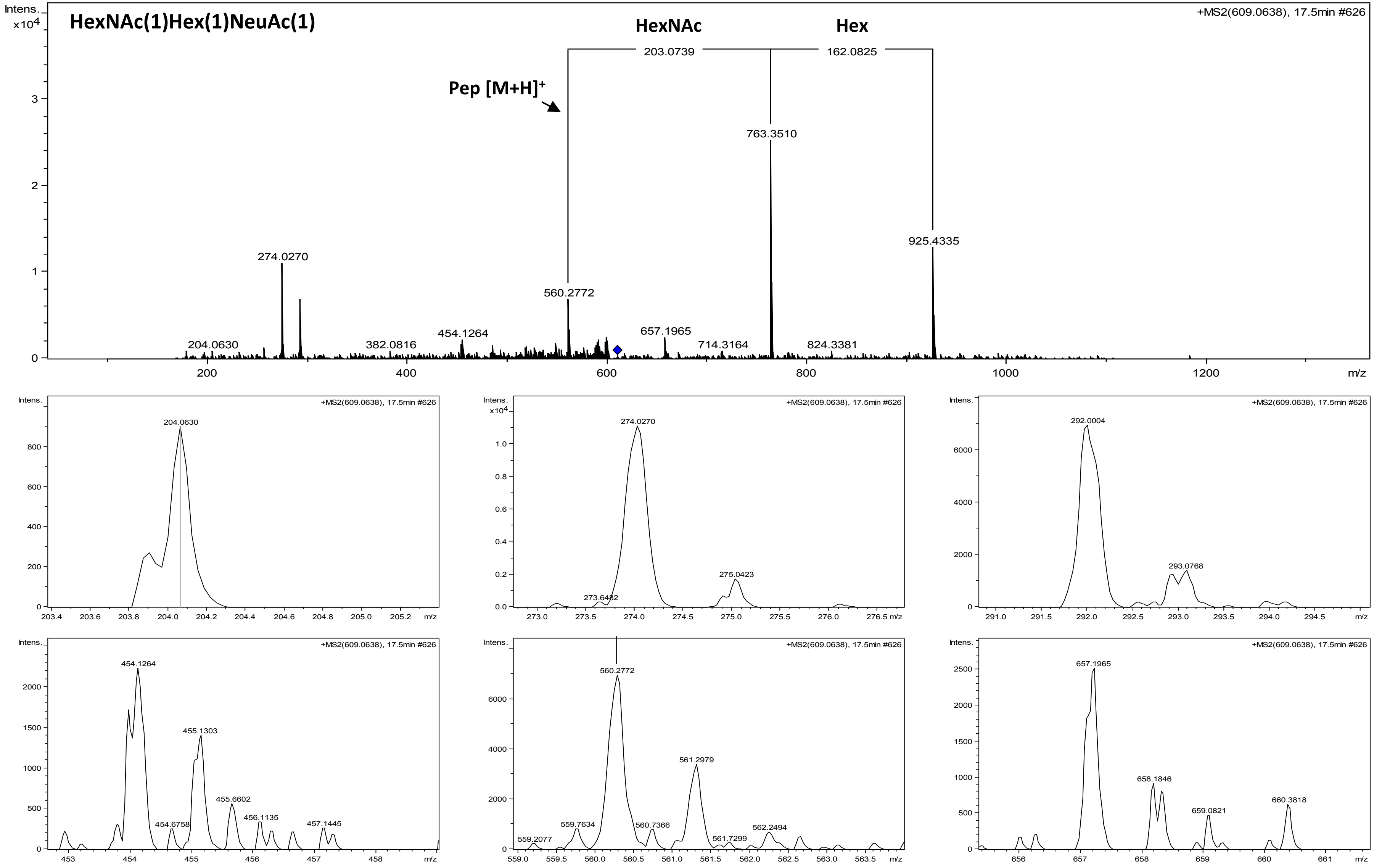

# Fraction 14

608.71++ → Pep [M+H]+ 560.28+ [17.7-17.8 min] CID-MS2

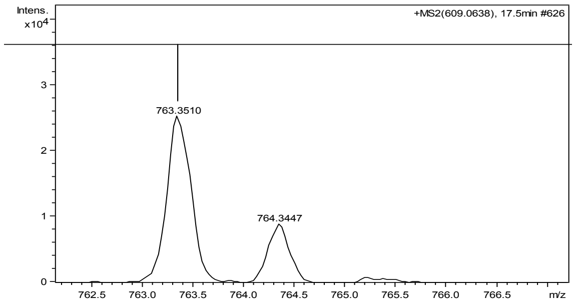


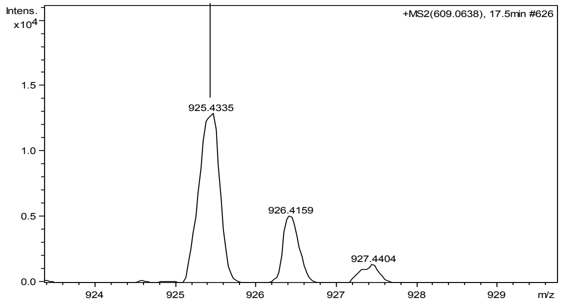

# Fraction 14

608.71++ → Pep [M+H]+ 560.28+ [17.7-17.8 min]    CID-MS3

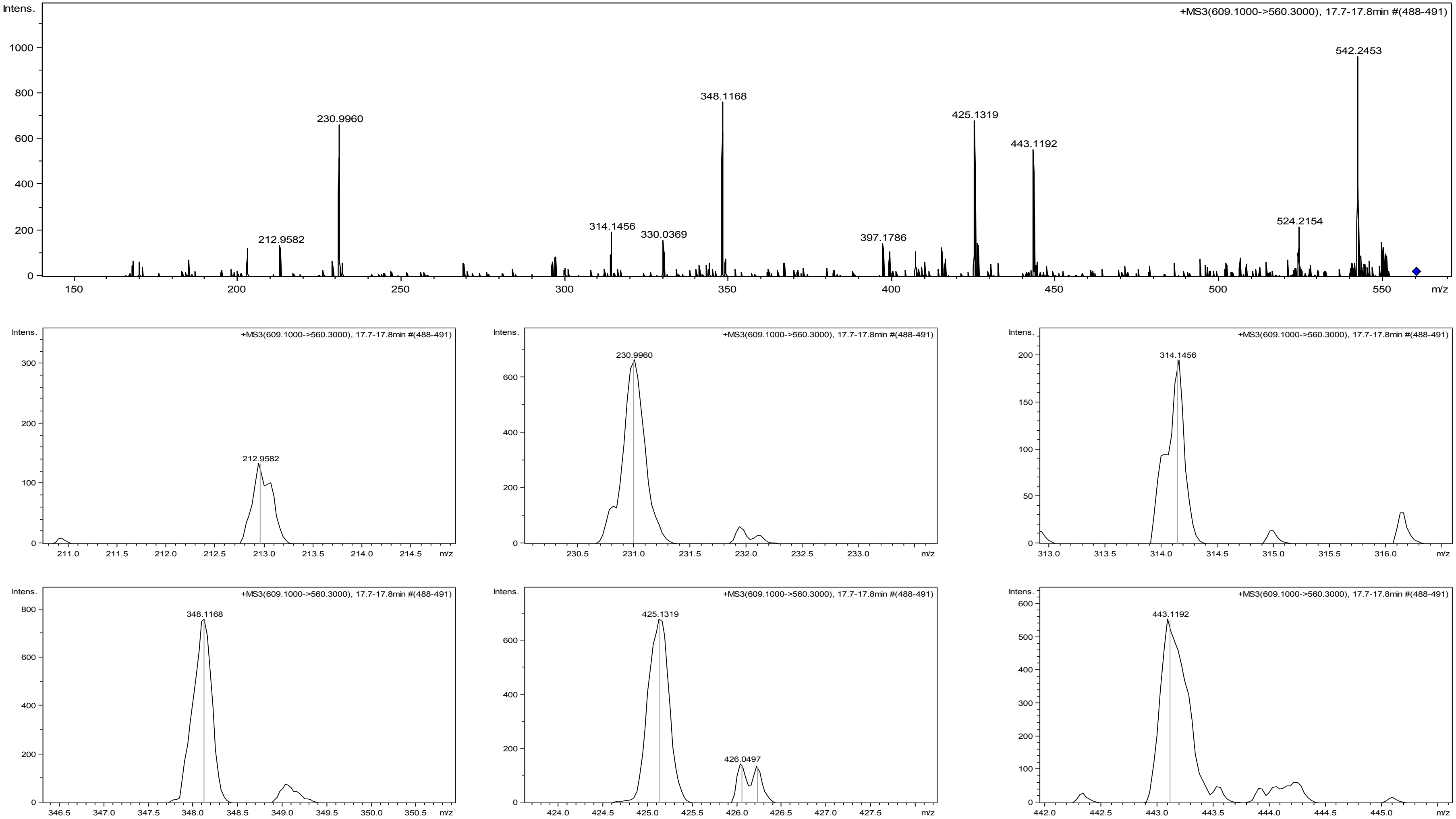

# Fraction 14

608.71++ → Pep [M+H]+ 560.28+ [17.7-17.8 min] CID-MS3

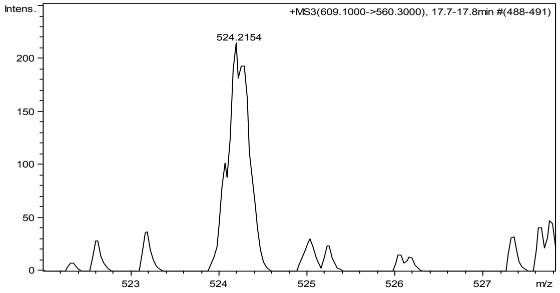


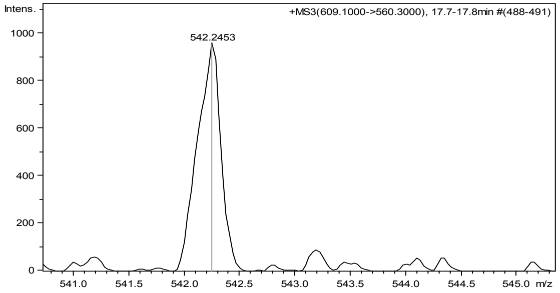

# Fraction 14

608.71++ → Pep [M+H]+ 560.28+ [17.7-17.8 min]

CID-MS3 MASCOT Search

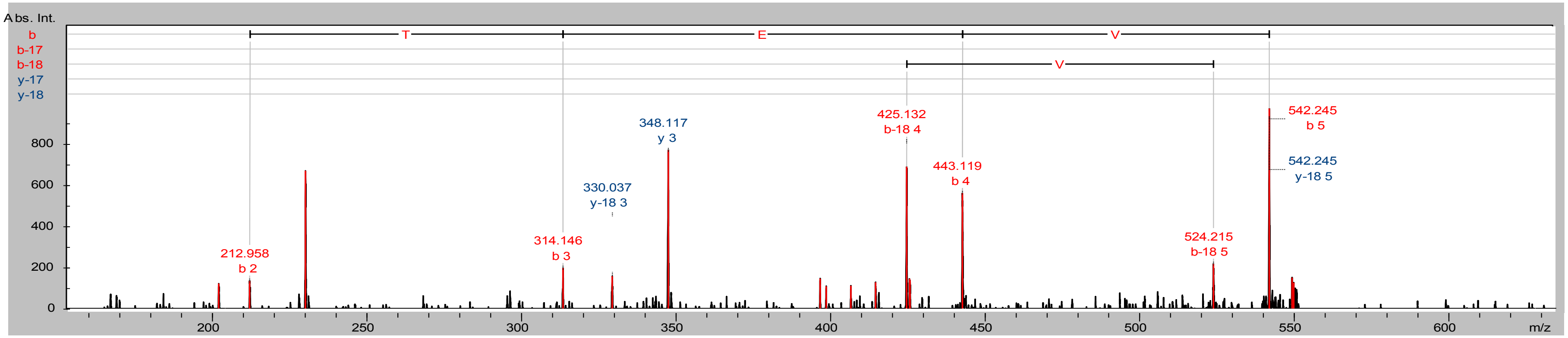


| | D | P | T | E | V | Asp | Pro | Thr | Glu | Val |
|---|---|---|---|---|---|---|---|---|---|---|
| Ion | 1 | 2 | 3 | 4 | 5 | 1 | 2 | 3 | 4 | 5 |
| b | D | P | T | E | V | 116.034 | 213.087 | 314.135 | 443.177 | 542.246 |
| b-17 | D | P | T | E | V | - | - | - | - | - |
| b-18 | D | P | T | E | V | 98.024 | 195.076 | 296.124 | 425.167 | 524.235 |
| y | D | P | T | E | V | 118.086 | 247.129 | 348.177 | 445.229 | 560.256 |
| y-17 | D | P | T | E | V | - | - | - | - | - |
| y-18 | D | P | T | E | V | - | 229.118 | 330.166 | 427.219 | 542.246 |
| | 5 | 4 | 3 | 2 | 1 | Val | Glu | Thr | Pro | Asp |

unknown O-glycosylation site

Immunoglobulin J chain

$_{95}$DP**T**EV$_{99}$

# Fraction 14

608.71++ → Pep [M+H]+ 560.28+ [17.7-17.8 min]

CID-MS3 MASCOT Search

| prot_hit_nur | prot_acc | prot_desc | prot_score | prot_mass | prot_matche | pep_query | pep_rank | pep_isbold | pep_exp_mz | pep_exp_mr | pep_exp_z | pep_calc_mr | pep_delta | pep_miss | pep_score | pep_expect | pep_res_bef | pep_seq |
|---|---|---|---|---|---|---|---|---|---|---|---|---|---|---|---|---|---|---|
| 1 | ILBP_HUMAN | Gastrotropin | 26 | 14362 | 1 | 1 | 1 | 1 | 560.2772 | 559.2699 | 1 | 559.3217 | -0.0518 | 0 | 27.1 | 30 | K | IVTEV |
| 2 | IGJ_HUMAN | Immunoglob | 25 | 16041 | 1 | 1 | 1 | 0 | 560.2772 | 559.2699 | 1 | 559.249 | 0.021 | 0 | 27.1 | 30 | C | DPTEV |
| 3 | CJ031_HUMA | Putative unc | 25 | 19734 | 1 | 1 | 1 | 0 | 560.2772 | 559.2699 | 1 | 559.249 | 0.021 | 0 | 27.1 | 30 | V | PDTQV |
| 4 | RASF5_HUM | Ras associati | 23 | 47688 | 1 | 1 | 1 | 0 | 560.2772 | 559.2699 | 1 | 559.249 | 0.021 | 0 | 27.1 | 30 | G | PDTEV |
| 5 | ERG19_HUM | Diphosphom | 23 | 43890 | 1 | 1 | 1 | 0 | 560.2772 | 559.2699 | 1 | 559.3217 | -0.0518 | 0 | 27.1 | 30 | I | IVTQV |
| 6 | CCR3_HUMA | C-C chemoki | 23 | 41530 | 1 | 1 | 1 | 0 | 560.2772 | 559.2699 | 1 | 559.3217 | -0.0518 | 0 | 27.1 | 30 | M | LVTEV |
| 7 | HSPB1_HUM | Heat-shock p | 22 | 22826 | 1 | 1 | 1 | 0 | 560.2772 | 559.2699 | 1 | 559.249 | 0.021 | 0 | 27.1 | 30 | V | DPTQV |
| 8 | AP2C_HUMA | Transcriptior | 22 | 49602 | 1 | 1 | 1 | 0 | 560.2772 | 559.2699 | 1 | 559.2489 | 0.021 | 0 | 27.1 | 30 | M | NPTEV |
| 9 | RUNX3_HUM | Runt-related | 22 | 44556 | 1 | 1 | 1 | 0 | 560.2772 | 559.2699 | 1 | 559.2489 | 0.021 | 0 | 27.1 | 30 | T | NPTQV |
| 10 | CAN5_HUMA | Calpain-5 (E( | 21 | 74092 | 1 | 1 | 1 | 0 | 560.2772 | 559.2699 | 1 | 559.3217 | -0.0518 | 0 | 27.1 | 30 | Q | LVTQV |

Biotools-Score: 15

MASCOT-Score: 27

unknown O-glycosylation site

Immunoglobulin J chain

$_{95}$DPTEV$_{99}$

# Fraction 14

684.76++ → Pep [M+H]+ 712.35+ [20.4-20.7 min]

CID-MS Precursor

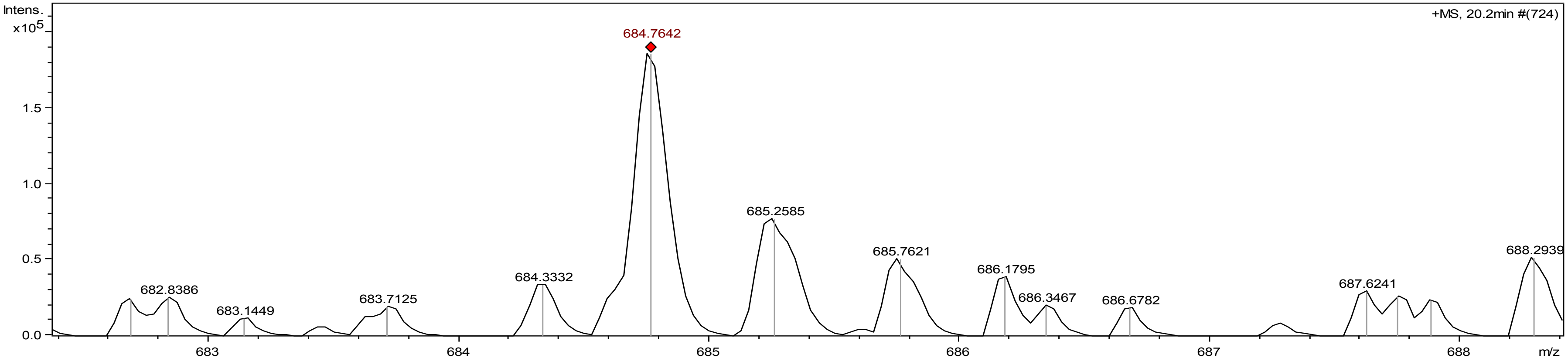


ETD spectrum not available

# Fraction 14

684.76++ → Pep [M+H]+ 712.35+ [20.4-20.7 min] CID-MS2

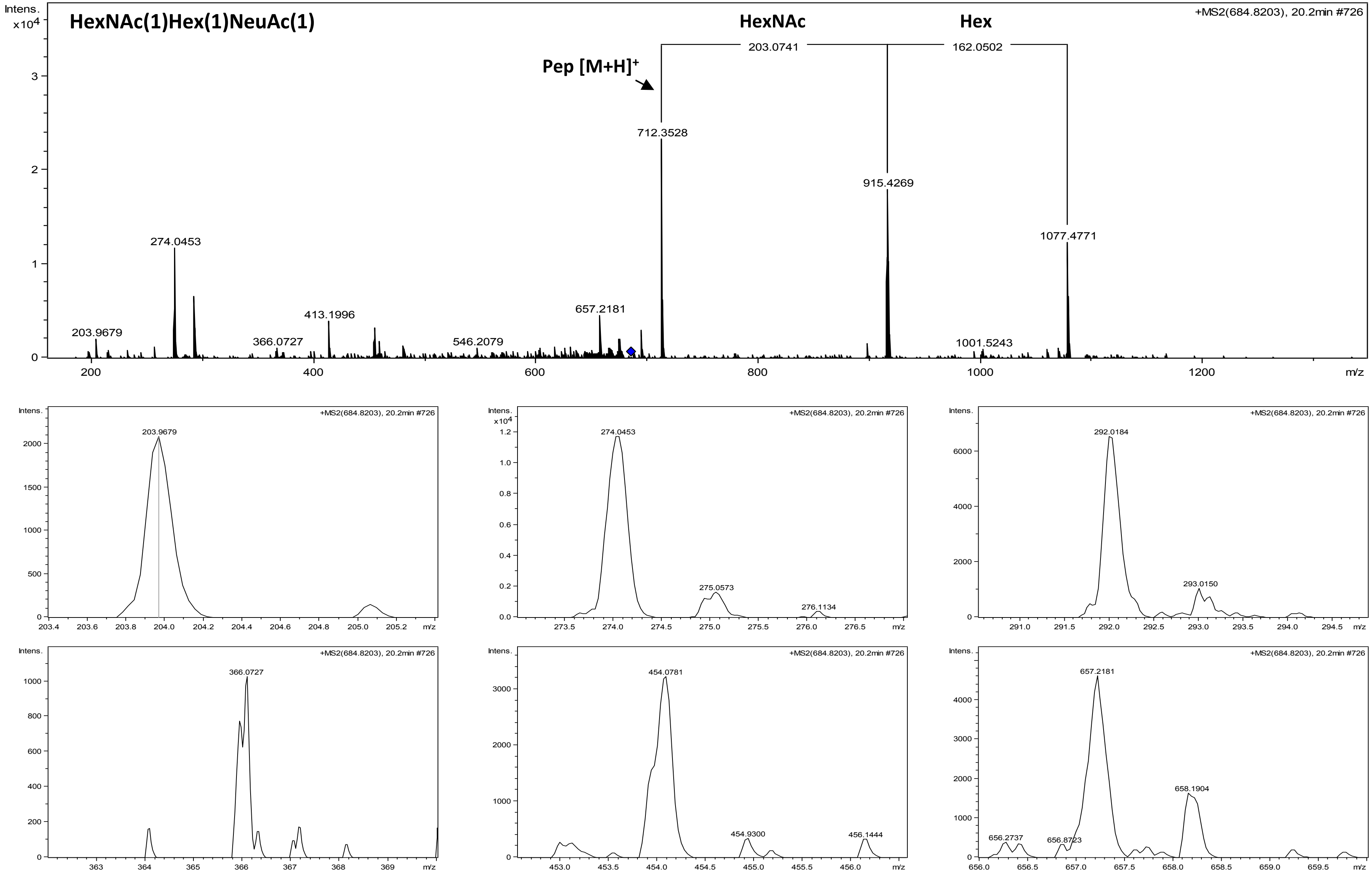

# Fraction 14

684.76++ → Pep [M+H]+ 712.35+ [20.4-20.7 min] CID-MS2

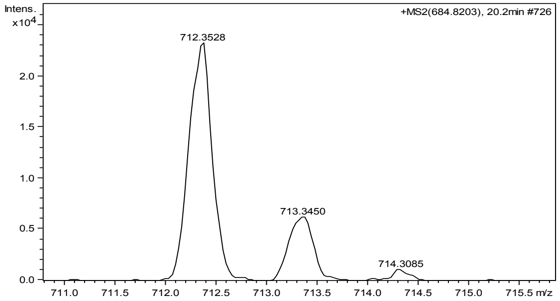


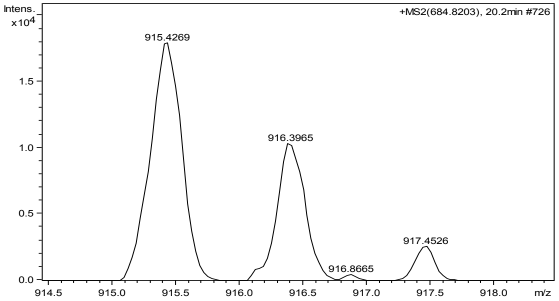


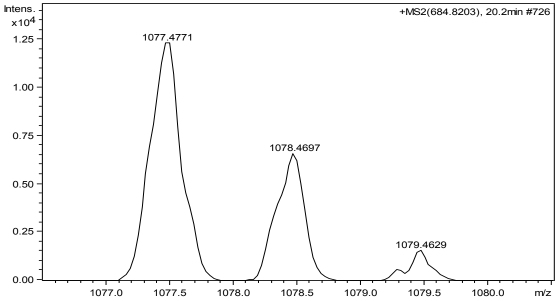

# Fraction 14

## 684.76++ → Pep [M+H]+ 712.35+ [20.4-20.7 min]  CID-MS3  Manual DeNovo

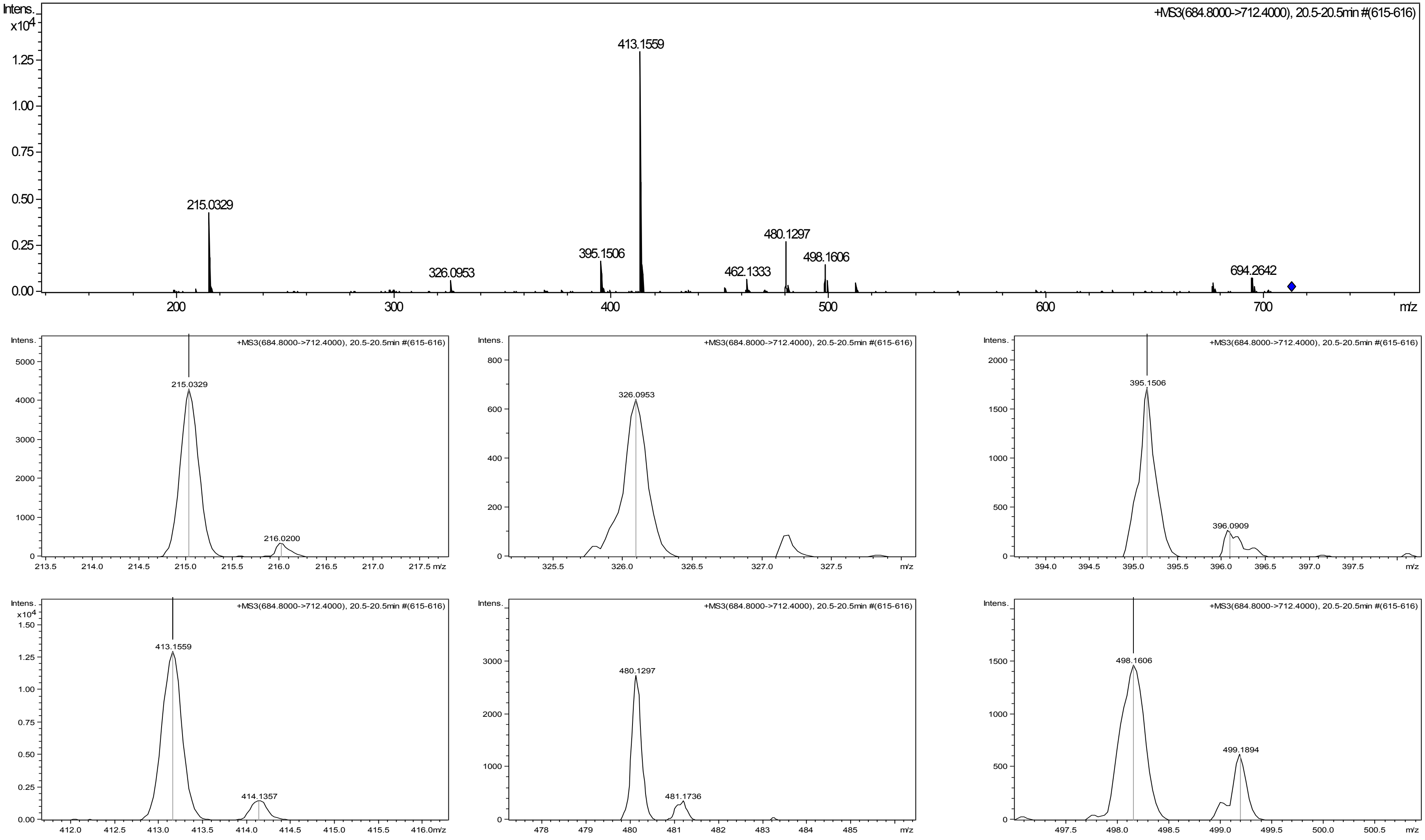

# Fraction 14

684.76++ → Pep [M+H]+ 712.35+ [20.4-20.7 min] CID-MS3

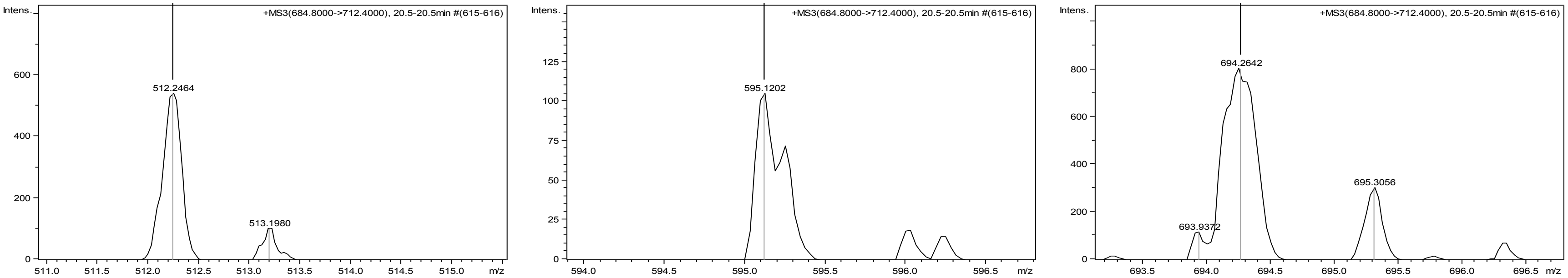

# Fraction 14

684.76++ → Pep [M+H]+ 712.35+ [20.4-20.7 min]

CID-MS3 MASCOT Search

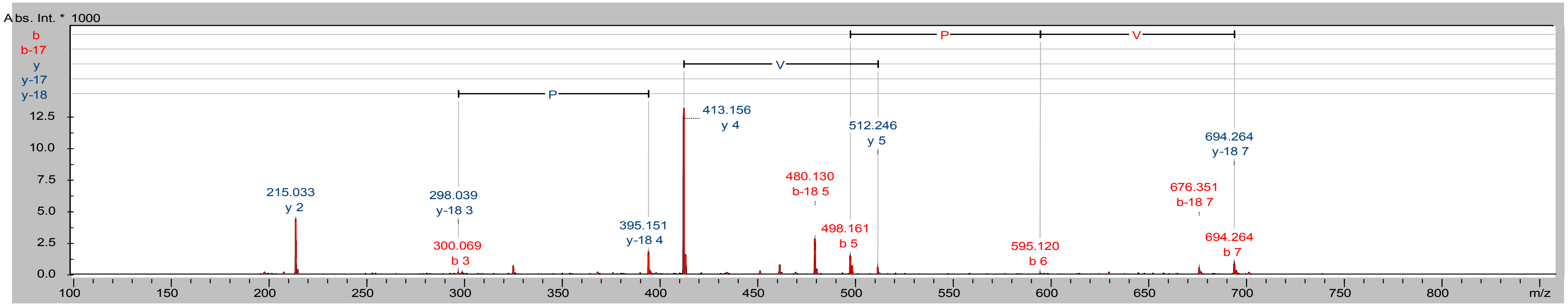


| | EAVPTPV | Glu | Ala | Val | Pro | Thr | Pro | Val |
|---|---|---|---|---|---|---|---|---|
| Ion | 1 2 3 4 5 6 7 | 1 | 2 | 3 | 4 | 5 | 6 | 7 |
| b | EAVPTPV | 130.050 | 201.087 | 300.155 | 397.208 | 498.256 | 595.309 | 694.377 |
| b-17 | EAVPTPV | - | - | - | - | - | - | - |
| b-18 | EAVPTPV | 112.039 | 183.076 | 282.145 | 379.198 | 480.245 | 577.298 | 676.366 |
| y | EAVPTPV | 118.086 | 215.139 | 316.187 | 413.239 | 512.308 | 583.345 | 712.388 |
| y-17 | EAVPTPV | - | - | - | - | - | - | - |
| y-18 | EAVPTPV | - | - | 298.176 | 395.229 | 494.297 | 565.334 | 694.377 |
| | 7 6 5 4 3 2 1 | Val | Pro | Thr | Pro | Val | Ala | Glu |

Known O-glycosylation site

Alpha-2-HS-glycoprotein precursor

$_{266}$EAVP**T**PV$_{272}$

# Fraction 14

684.76++ → Pep [M+H]+ 712.35+ [20.4-20.7 min]

CID-MS3 MASCOT Search

| prot_hit_nur | prot_acc | prot_desc | prot_score | prot_mass | prot_matche | pep_query | pep_rank | pep_isbold | pep_exp_mz | pep_exp_mr | pep_exp_z | pep_calc_mr | pep_delta | pep_miss | pep_score | pep_expect | pep_res_bef | pep_seq |
|---|---|---|---|---|---|---|---|---|---|---|---|---|---|---|---|---|---|---|
| 1 | MK07_HUMA | Mitogen-acti | 17 | 89151 | 1 | 1 | 1 | 1 | 712.3528 | 711.3455 | 1 | 711.3803 | -0.0348 | 0 | 22.9 | 50 | P | TPTPTPV |
| 2 | FETUA_HUM | Alpha-2-HS- | 14 | 40098 | 1 | 1 | 3 | 0 | 712.3528 | 711.3455 | 1 | 711.3803 | -0.0348 | 0 | 18.47 | 1.40E+02 | N | EAVPTPV |
| 3 | CN043_HUM | Uncharacteri | 12 | 115431 | 1 | 1 | 3 | 0 | 712.3528 | 711.3455 | 1 | 711.3803 | -0.0348 | 0 | 18.47 | 1.40E+02 | S | AEVTPPV |
| 4 | NFYC_HUMA | Nuclear tran | 12 | 50556 | 1 | 1 | 6 | 0 | 712.3528 | 711.3455 | 1 | 711.3803 | -0.0348 | 0 | 16.33 | 2.30E+02 | S | VTPAEPV |
| 5 | APOA_HUM | Apolipoprot | 11 | 514737 | 1 | 1 | 2 | 0 | 712.3528 | 711.3455 | 1 | 711.4167 | -0.0712 | 0 | 21.25 | 73 | T | TPTVVPV |
| 6 | ITIH2_HUMA | Inter-alpha-t | 10 | 106826 | 1 | 1 | 10 | 0 | 712.3528 | 711.3455 | 1 | 711.3439 | 0.0016 | 0 | 15.65 | 2.60E+02 | A | NPSPTPV |
| 7 | TRIPC_HUM | Thyroid rece | 9 | 222234 | 1 | 1 | 9 | 0 | 712.3528 | 711.3455 | 1 | 711.2568 | 0.0888 | 0 | 15.9 | 2.50E+02 | T | LTMNGC |
| 8 | WNK2_HUM | Serine/thre | 9 | 244293 | 1 | 1 | 5 | 0 | 712.3528 | 711.3455 | 1 | 711.3803 | -0.0348 | 0 | 16.75 | 2.00E+02 | A | AQVPTVP |
| 9 | CEP35_HUM | Centrosome- | 8 | 352312 | 1 | 1 | 7 | 0 | 712.3528 | 711.3455 | 1 | 710.4327 | 0.9128 | 0 | 16.11 | 2.40E+02 | T | KAVTPPV |

Biotools-Score: 38

MASCOT-Score: 18

Known O-glycosylation site

Alpha-2-HS-glycoprotein precursor

$_{266}$EAVP**T**PV$_{272}$

# Fraction 14

874.88++ → Pep [M+H]+ 1092.60+ [25.8-26.2 min] CID-MS Precursor

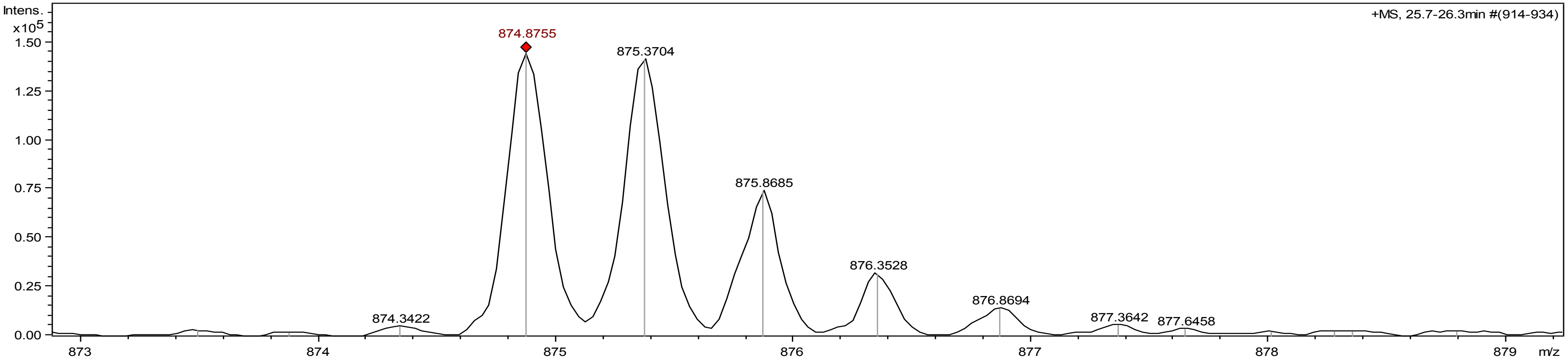

# Fraction 14

874.88++ → Pep [M+H]+ 1092.60+ [25.8-26.2 min] CID-MS2

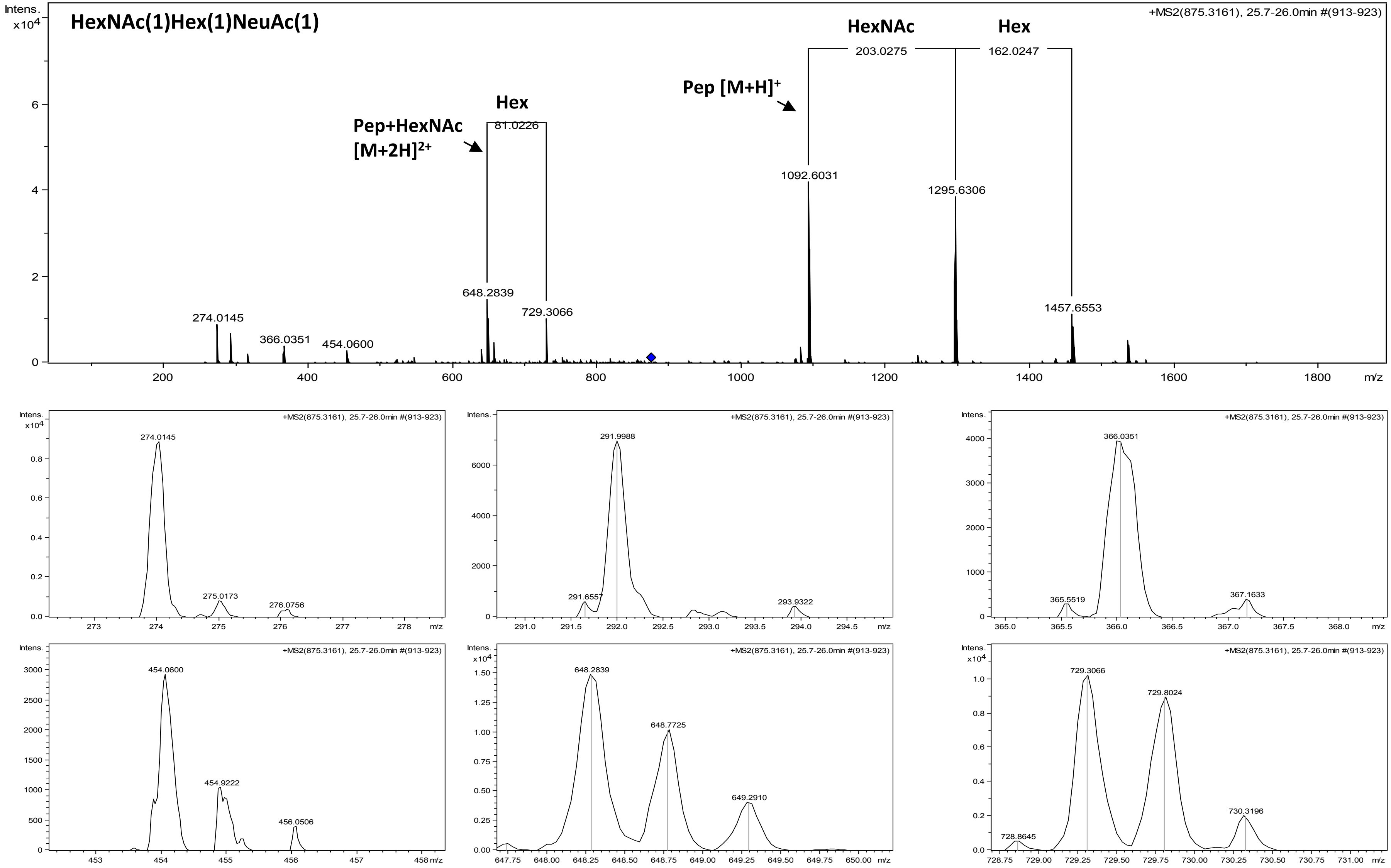

# Fraction 14

## 874.88++ → Pep [M+H]+ 1092.60+ [25.8-26.2 min] CID-MS2

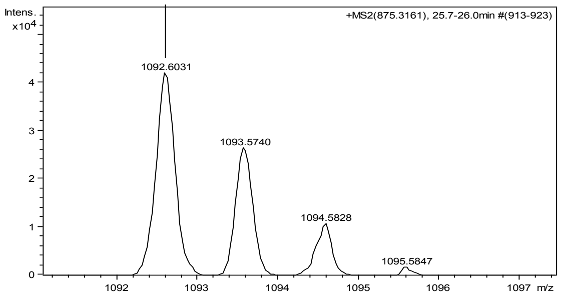


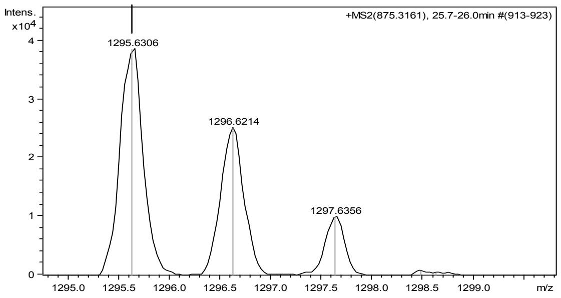


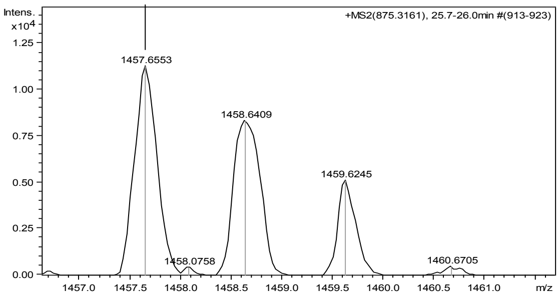


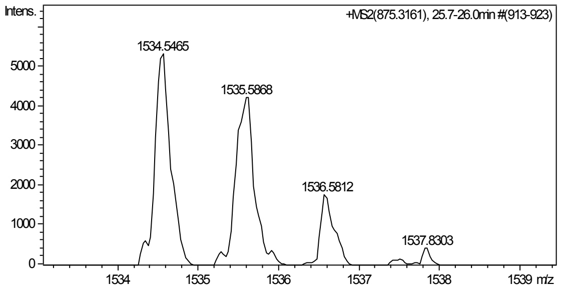

# Fraction 14

874.88++ → Pep [M+H]+ 1092.60+ [25.8-26.2 min] CID-MS3

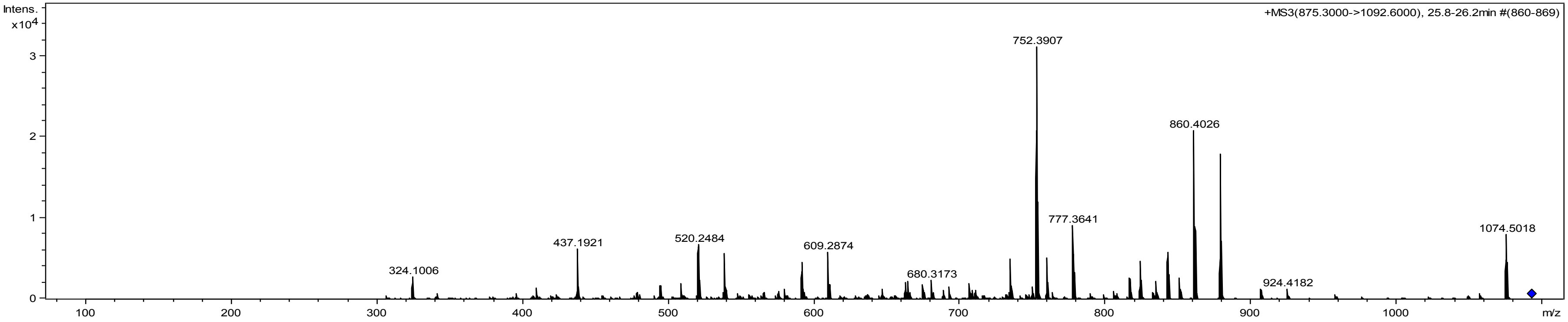


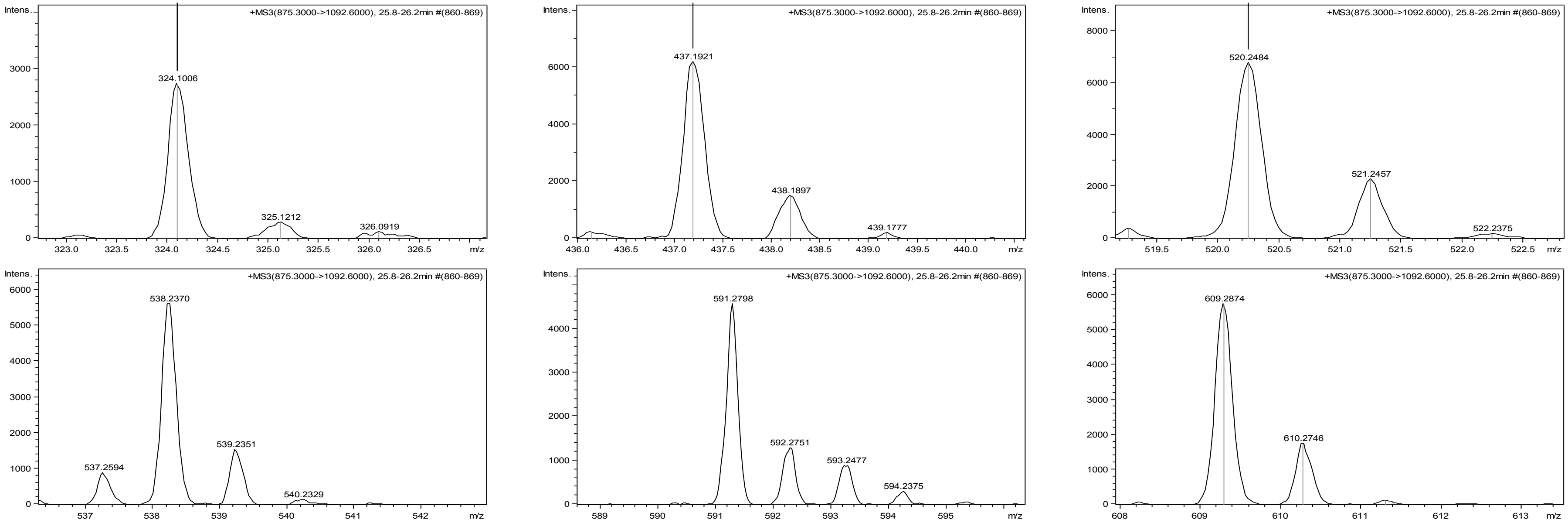

# Fraction 14

## 874.88++ → Pep [M+H]+ 1092.60+ [25.8-26.2 min]

CID-MS3

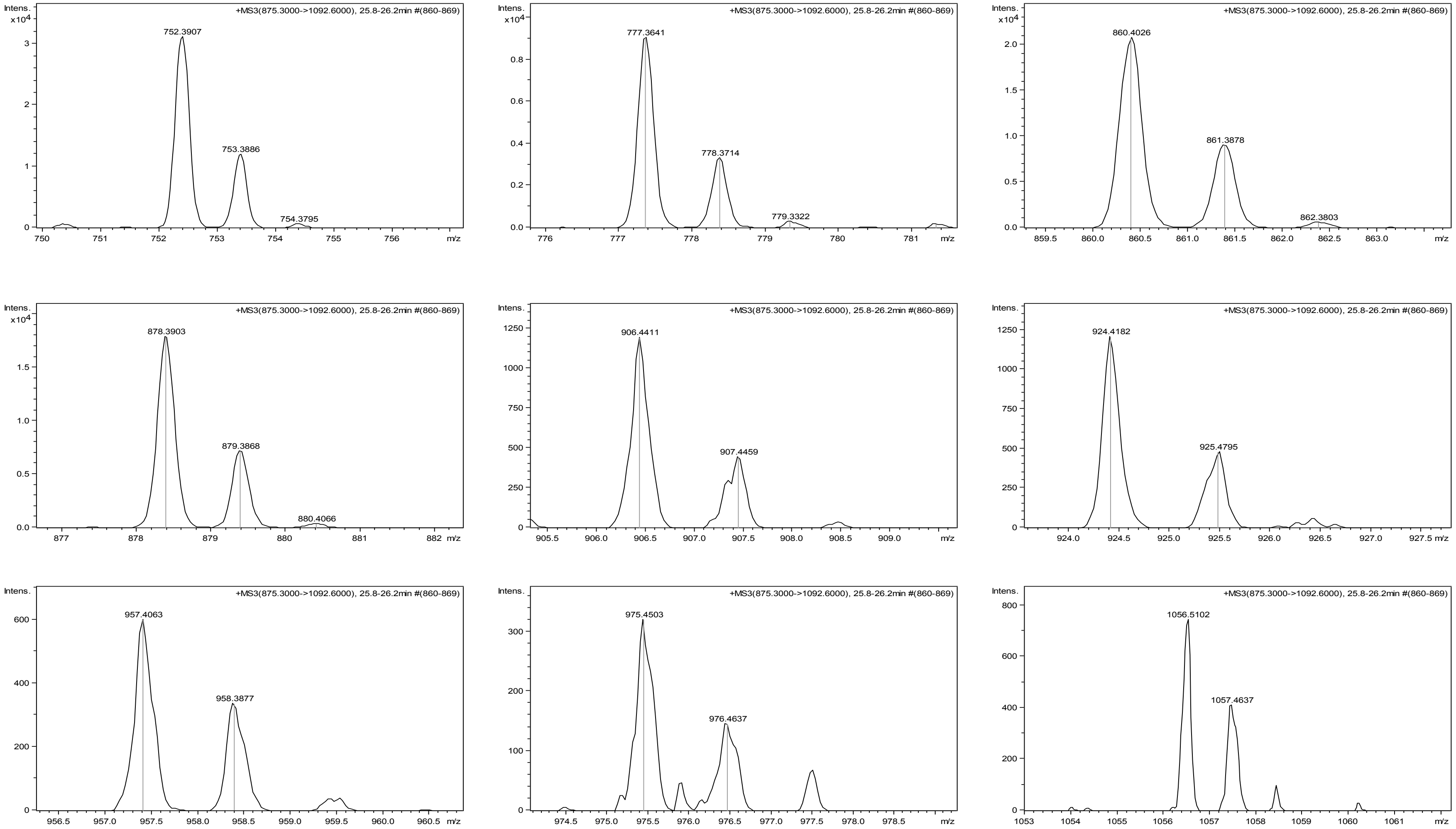

# Fraction 14

874.88++ → Pep [M+H]+ 1092.60+ [25.8-26.2 min] CID-MS3

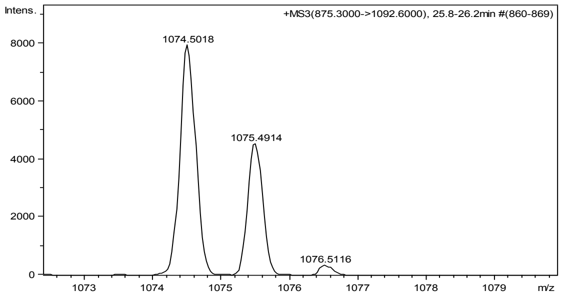

# Fraction 14

874.88++ → Pep [M+H]+ 1092.60+ [25.8-26.2 min]    CID-MS3    MASCOT Search

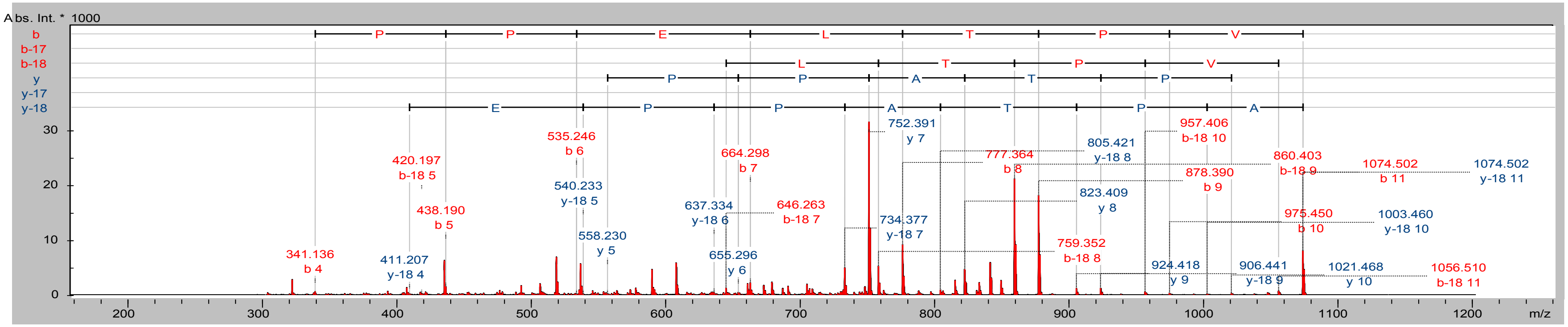


| | A | P | T | A | P | P | E | L | T | P | V | Ala | Pro | Thr | Ala | Pro | Pro | Glu | Leu | Thr | Pro | Val |
|---|---|---|---|---|---|---|---|---|---|---|---|---|---|---|---|---|---|---|---|---|---|---|
| Ion | 1 | 2 | 3 | 4 | 5 | 6 | 7 | 8 | 9 | 10 | 11 | 1 | 2 | 3 | 4 | 5 | 6 | 7 | 8 | 9 | 10 | 11 |
| b | A | P | T | A | P | P | E | L | T | P | V | 72.044 | 169.097 | 270.145 | 341.182 | 438.235 | 535.287 | 664.330 | 777.414 | 878.462 | 975.515 | 1074.583 |
| b-17 | A | P | T | A | P | P | E | L | T | P | V | - | - | - | - | - | - | - | - | - | - | - |
| b-18 | A | P | T | A | P | P | E | L | T | P | V | - | - | 252.134 | 323.171 | 420.224 | 517.277 | 646.320 | 759.404 | 860.451 | 957.504 | 1056.572 |
| y | A | P | T | A | P | P | E | L | T | P | V | 118.086 | 215.139 | 316.187 | 429.271 | 558.313 | 655.366 | 752.419 | 823.456 | 924.504 | 1021.556 | 1092.594 |
| y-17 | A | P | T | A | P | P | E | L | T | P | V | - | - | - | - | - | - | - | - | - | - | - |
| y-18 | A | P | T | A | P | P | E | L | T | P | V | - | - | 298.176 | 411.260 | 540.303 | 637.356 | 734.408 | 805.445 | 906.493 | 1003.546 | 1074.583 |
| | 11 | 10 | 9 | 8 | 7 | 6 | 5 | 4 | 3 | 2 | 1 | Val | Pro | Thr | Leu | Glu | Pro | Pro | Ala | Thr | Pro | Ala |

Known O-glycosylation site

Plasminogen precursor

$_{363}$AP**T**APPELTPV$_{373}$

# Fraction 14

874.88++ → Pep [M+H]+ 1092.60+ [25.8-26.2 min]

CID-MS3 MASCOT Search

| prot_hit_nur | prot_acc | prot_desc | prot_score | prot_mass | prot_matche | pep_query | pep_rank | pep_isbold | pep_exp_mz | pep_exp_mr | pep_exp_z | pep_calc_mr | pep_delta | pep_miss | pep_score | pep_expect | pep_res_bef | pep_seq |
|---|---|---|---|---|---|---|---|---|---|---|---|---|---|---|---|---|---|---|
| 1 | PLMN_HUMA | Plasminoger | 18 | 93247 | 1 | 1 | 1 | 1 | 1092.6031 | 1091.5958 | 1 | 1091.5863 | 0.0096 | 0 | 23.89 | 68 | L | APTAPPELTP |
| 2 | SON_HUMAN | SON protein | 11 | 264079 | 1 | 1 | 2 | 0 | 1092.6031 | 1091.5958 | 1 | 1091.5863 | 0.0095 | 0 | 18.89 | 2.10E+02 | V | PELPGPSVTP |
| 3 | SIAT1_HUMA | CMP-N-acety | 10 | 47088 | 1 | 1 | 5 | 0 | 1092.6031 | 1091.5958 | 1 | 1091.4771 | 0.1187 | 0 | 15.5 | 4.70E+02 | G | APTANFQQD |
| 4 | CERU_HUMA | Ceruloplasm | 8 | 122983 | 1 | 1 | 4 | 0 | 1092.6031 | 1091.5958 | 1 | 1091.5499 | 0.0459 | 0 | 15.66 | 4.50E+02 | A | VDPTKDIFTG |
| 5 | MINT_HUMA | Msx2-interac | 8 | 403030 | 1 | 1 | 3 | 0 | 1092.6031 | 1091.5958 | 1 | 1091.5135 | 0.0823 | 0 | 17.19 | 3.20E+02 | P | EGEPLQPPQF |
| 6 | DDFL1_HUM | Developmer | 8 | 100177 | 1 | 1 | 6 | 0 | 1092.6031 | 1091.5958 | 1 | 1091.5135 | 0.0823 | 0 | 14.02 | 6.60E+02 | G | QPLPGPDAQ |
| 7 | IF39_HUMAN | Eukaryotic tr | 6 | 92833 | 1 | 1 | 8 | 0 | 1092.6031 | 1091.5958 | 1 | 1091.5135 | 0.0823 | 0 | 11.91 | 1.10E+03 | A | EPPVPAQGE |
| 8 | NPAS2_HUM | Neuronal PA | 6 | 92501 | 1 | 1 | 7 | 0 | 1092.6031 | 1091.5958 | 1 | 1091.4982 | 0.0976 | 0 | 12.12 | 1.00E+03 | N | LTTPASTSQD |
| 9 | ABCG4_HUM | ATP-binding | 5 | 72932 | 1 | 1 | 9 | 0 | 1092.6031 | 1091.5958 | 1 | 1090.377 | 1.2189 | 0 | 11.66 | 1.10E+03 | S | CYIMQDDM |
| 10 | SO1B3_HUM | Solute carrie | 5 | 78436 | 1 | 1 | 10 | 0 | 1092.6031 | 1091.5958 | 1 | 1091.5863 | 0.0096 | 0 | 11.44 | 1.20E+03 | A | IQVINSLFSA |

Biotools-Score: 326

MASCOT-Score: 24

Known O-glycosylation site

Plasminogen precursor

$_{363}$APTAPPELTPV$_{373}$

# Fraction 14

874.88++ → Pep [M+H]+ 1092.60+ [25.8-26.2 min] ETD

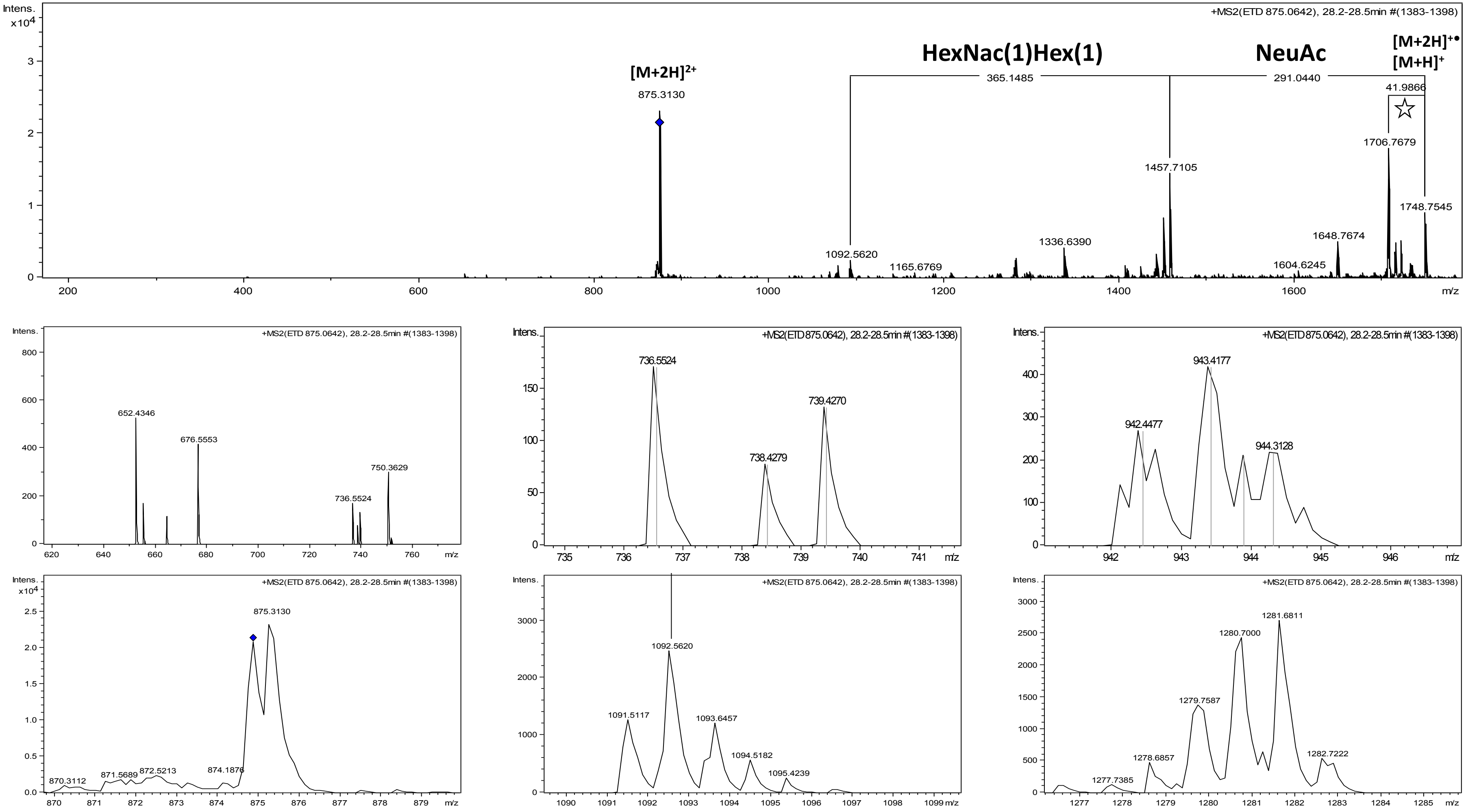


Known O-glycosylation site

Plasminogen precursor

$_{363}$AP**T**APPELTPV$_{373}$

# Fraction 14

## 874.88++ → Pep [M+H]+ 1092.60+ [25.8-26.2 min] ETD

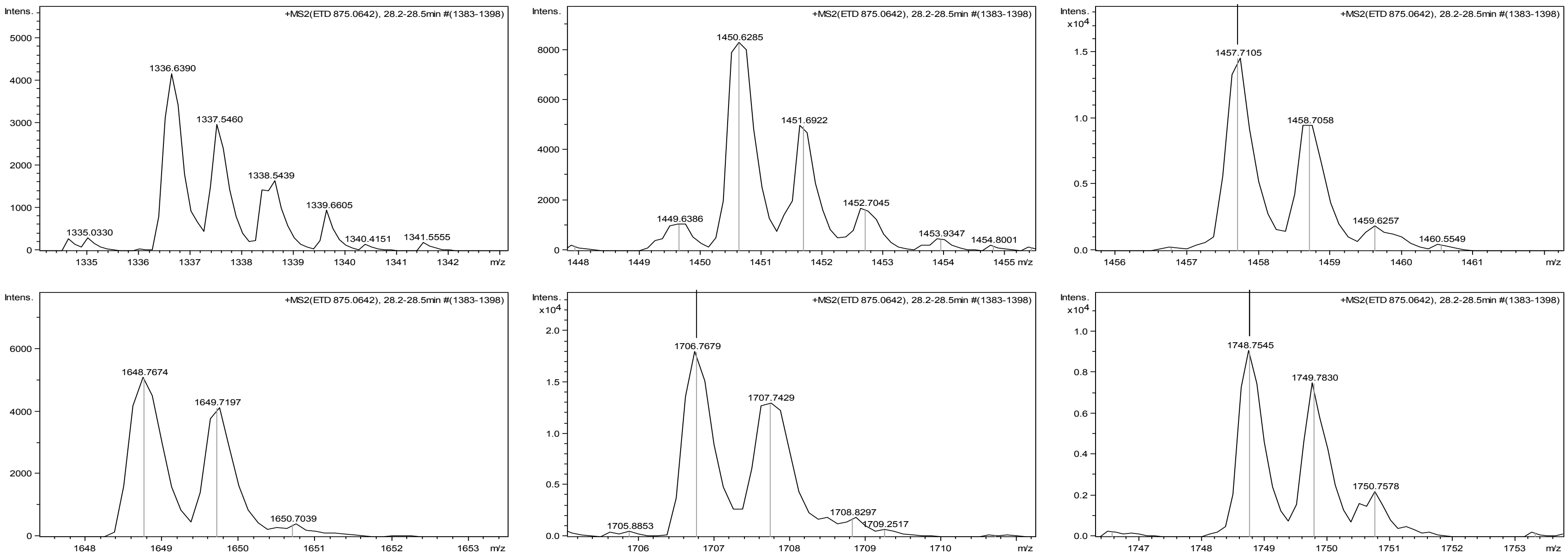


Known O-glycosylation site

Plasminogen precursor

$_{363}$AP**T**APPELTPV$_{373}$

# Fraction 14

874.88++ → Pep [M+H]+ 1092.60+ [25.8-26.2 min] ETD

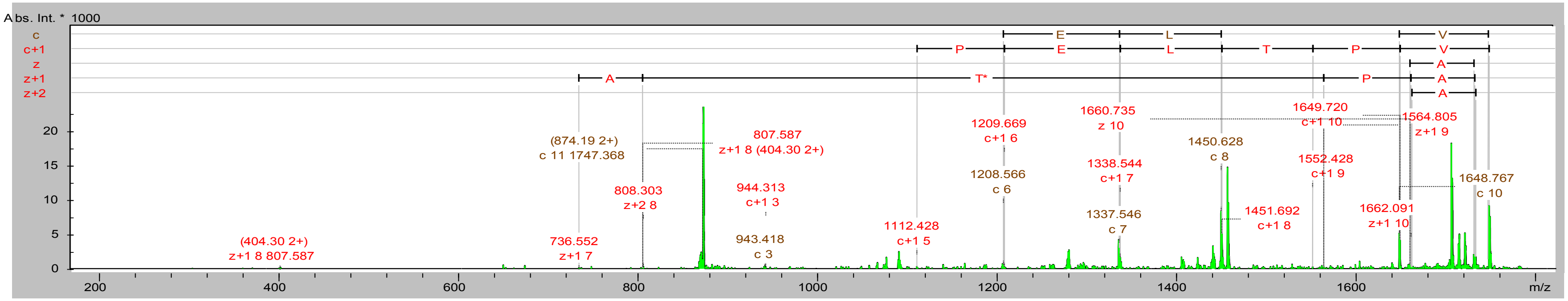


APTAPPELTPV

| | A | P | T | A | P | P | E | L | T | P | V | Ala | Pro | Thr | Ala | Pro | Pro | Glu | Leu | Thr | Pro | Val |
|---|---|---|---|---|---|---|---|---|---|---|---|---|---|---|---|---|---|---|---|---|---|---|
| Ion | 1 | 2 | 3 | 4 | 5 | 6 | 7 | 8 | 9 | 10 | 11 | 1 | 2 | 3 | 4 | 5 | 6 | 7 | 8 | 9 | 10 | 11 |
| c | A | P | T* | A | P | P | E | L | T | P | V | 89.071 | 186.124 | 943.399 | 1014.436 | 1111.489 | 1208.542 | 1337.584 | 1450.668 | 1551.716 | 1648.769 | 1747.837 |
| c+1 | A | P | T* | A | P | P | E | L | T | P | V | 90.079 | 187.132 | 944.407 | 1015.444 | 1112.497 | 1209.549 | 1338.592 | 1451.676 | 1552.724 | 1649.777 | 1748.845 |
| z | A | P | T* | A | P | P | E | L | T | P | V | 101.060 | 198.112 | 299.160 | 412.244 | 541.287 | 638.340 | 735.392 | 806.429 | 1563.705 | 1660.758 | 1731.795 |
| z+1 | A | P | T* | A | P | P | E | L | T | P | V | 102.068 | 199.120 | 300.168 | 413.252 | 542.295 | 639.347 | 736.400 | 807.437 | 1564.713 | 1661.765 | 1732.802 |
| z+2 | A | P | T* | A | P | P | E | L | T | P | V | 103.075 | 200.128 | 301.176 | 414.260 | 543.302 | 640.355 | 737.408 | 808.445 | 1565.720 | 1662.773 | 1733.810 |
| | 11 | 10 | 9 | 8 | 7 | 6 | 5 | 4 | 3 | 2 | 1 | Val | Pro | Thr | Leu | Glu | Pro | Pro | Ala | Thr | Pro | Ala |

Biotools-Score: 177

Known O-glycosylation site

Plasminogen precursor $_{363}$APTAPPELTPV$_{373}$

# Fraction 14

874.88++ → Pep [M+H]+ 1092.60+ [25.8-26.2 min] ETD

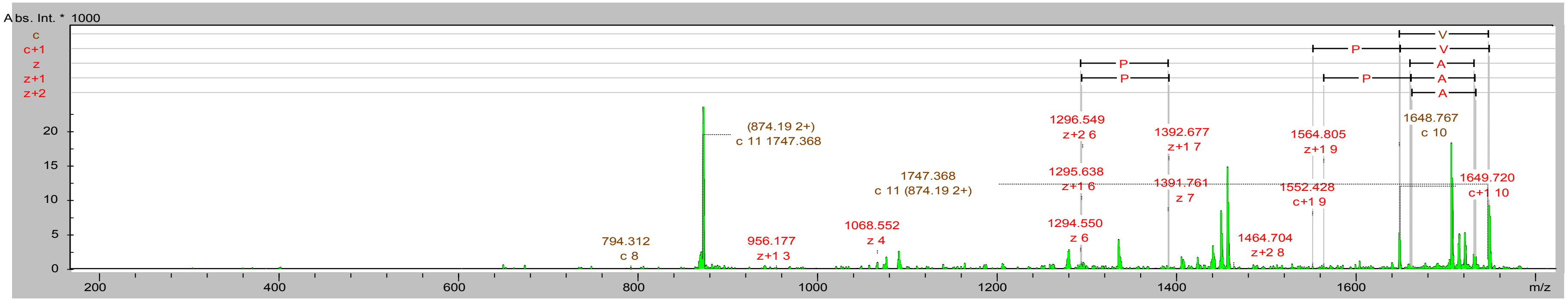


APTAPPEL**T**PV

| | A | P | T | A | P | P | E | L | T | P | V | Ala | Pro | Thr | Ala | Pro | Pro | Glu | Leu | Thr | Pro | Val |
|---|---|---|---|---|---|---|---|---|---|---|---|---|---|---|---|---|---|---|---|---|---|---|
| Ion | 1 | 2 | 3 | 4 | 5 | 6 | 7 | 8 | 9 | 10 | 11 | 1 | 2 | 3 | 4 | 5 | 6 | 7 | 8 | 9 | 10 | 11 |
| c | A | P | T | A | P | P | E | L | T* | P | V | 89.071 | 186.124 | 287.171 | 358.208 | 455.261 | 552.314 | 681.357 | 794.441 | 1551.716 | 1648.769 | 1747.837 |
| c+1 | A | P | T | A | P | P | E | L | T* | P | V | 90.079 | 187.132 | 288.179 | 359.216 | 456.269 | 553.322 | 682.364 | 795.449 | 1552.724 | 1649.777 | 1748.845 |
| z | A | P | T | A | P | P | E | L | T* | P | V | 101.060 | 198.112 | 955.388 | 1068.472 | 1197.514 | 1294.567 | 1391.620 | 1462.657 | 1563.705 | 1660.758 | 1731.795 |
| z+1 | A | P | T | A | P | P | E | L | T* | P | V | 102.068 | 199.120 | 956.396 | 1069.480 | 1198.522 | 1295.575 | 1392.628 | 1463.665 | 1564.713 | 1661.765 | 1732.802 |
| z+2 | A | P | T | A | P | P | E | L | T* | P | V | 103.075 | 200.128 | 957.403 | 1070.487 | 1199.530 | 1296.583 | 1393.636 | 1464.673 | 1565.720 | 1662.773 | 1733.810 |
| | 11 | 10 | 9 | 8 | 7 | 6 | 5 | 4 | 3 | 2 | 1 | Val | Pro | Thr | Leu | Glu | Pro | Pro | Ala | Thr | Pro | Ala |

Biotools-Score: 34

Known O-glycosylation site

Plasminogen precursor

$_{363}$AP**T**APPELTPV$_{373}$
