# Supplementary material for: Site-specific O-Glycosylation Analysis of Human Blood Plasma Proteins
Source: Mol Cell Proteomics. 2015 Nov 23;15(2):624–41. doi: 10.1074/mcp.M115.053546 (PMC4739677; doi:10.1074/mcp.M115.053546)
Supplement: Supplemental Data [file 10.1074_M115.053546_mcp.M115.053546-5.pdf]

Site-Specific *O*-Glycosylation Analysis of Human Blood Plasma Proteins

Proteinase K Digest

Fraction 15

Search Parameters For Protein Identification

Project: Blood Plasma Glycoprotomics (2013), ProtK-Digest
Glycopeptides measured on Bruker ESI-Ion Trap MS (CID-MS³)

Mascot version 2.2.07	
Database:	SwissProt
Fasta file:	SwissProt_51.6.fasta
Total sequences:	257964
Total residues:	93947433
Sequences after taxonomy filter:	15720
Number of queries:	1

Variable modifications --

Identifier	*Name*	*Delta*	*Neutral loss(es)*
1	Deamidated (NQ)	0.984009	0
2	Oxidation (M)	15.994919	63.998285

Search Parameters --

Taxonomy filter:	Homo sapiens (human)
Enzyme:	None
Maximum Missed Cleavages:	0
Fixed modifications	Carbamidomethyl (C)
ICAT experiment	0
Variable modifications	Deamidated (NQ), Oxidation (M)
Peptide Mass Tolerance	0.3
Peptide Mass Tolerance Units	Da
Fragment Mass Tolerance	0.35
Fragment Mass Tolerance Units	Da
Mass values	Monoisotopic
Instrument type	ESI-TRAP
Isotope error mode	1

Format parameters --

Significance threshold	0.05
Max. number of hits	20
Use MudPIT protein scoring	0
Ions score cut-off	0
Include same-set proteins	0
Include sub-set proteins	0
Include unassigned	0
Require bold red	0

Extracted ion chromatograms of glycan-specific oxonium ions

Oxonium Ions	$[M+H]^+$ m/z
Fuc	147.08
Hex	163.06
HexNAc	204.09
NeuAc -H_2O	274.09
NeuAc	292.10
HexNAc(1)Hex(1)	366.14
Hex(1)NeuAc(1)	454.16
HexNAc(1)NeuAc(1)	495.18
HexNAc(1)Hex(1)Fuc(1)	512.21
HexNAc(1)Hex(2)	528.19
HexNAc(1)Hex(1)NeuAc(1)	657.24

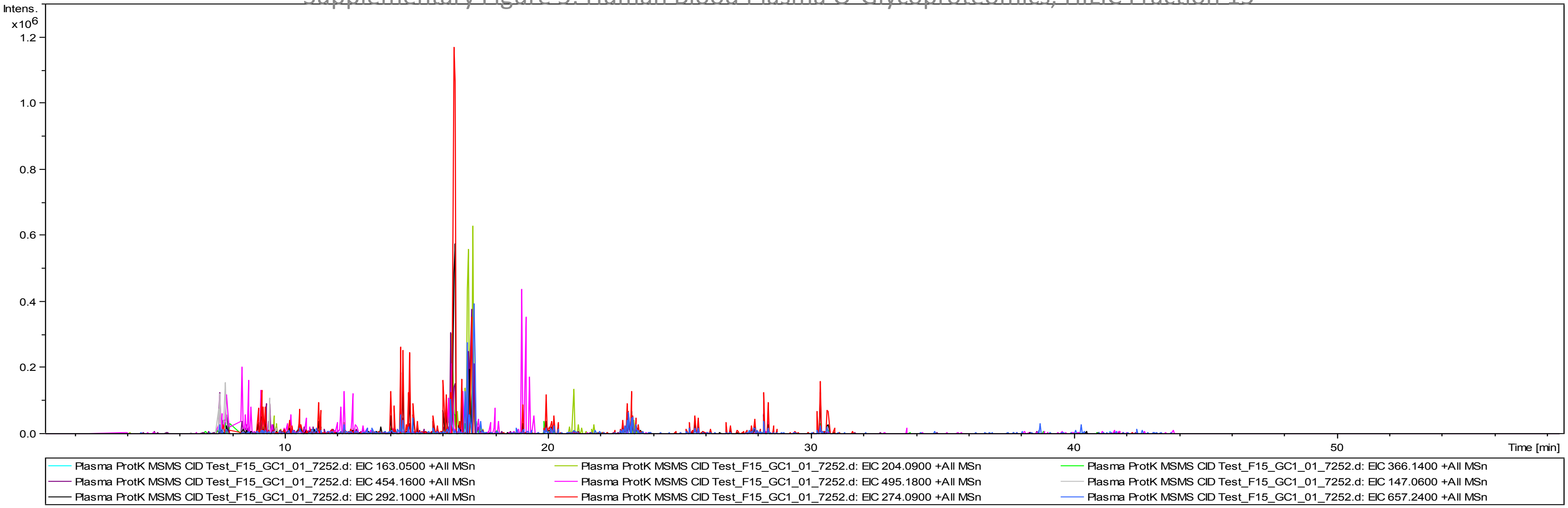

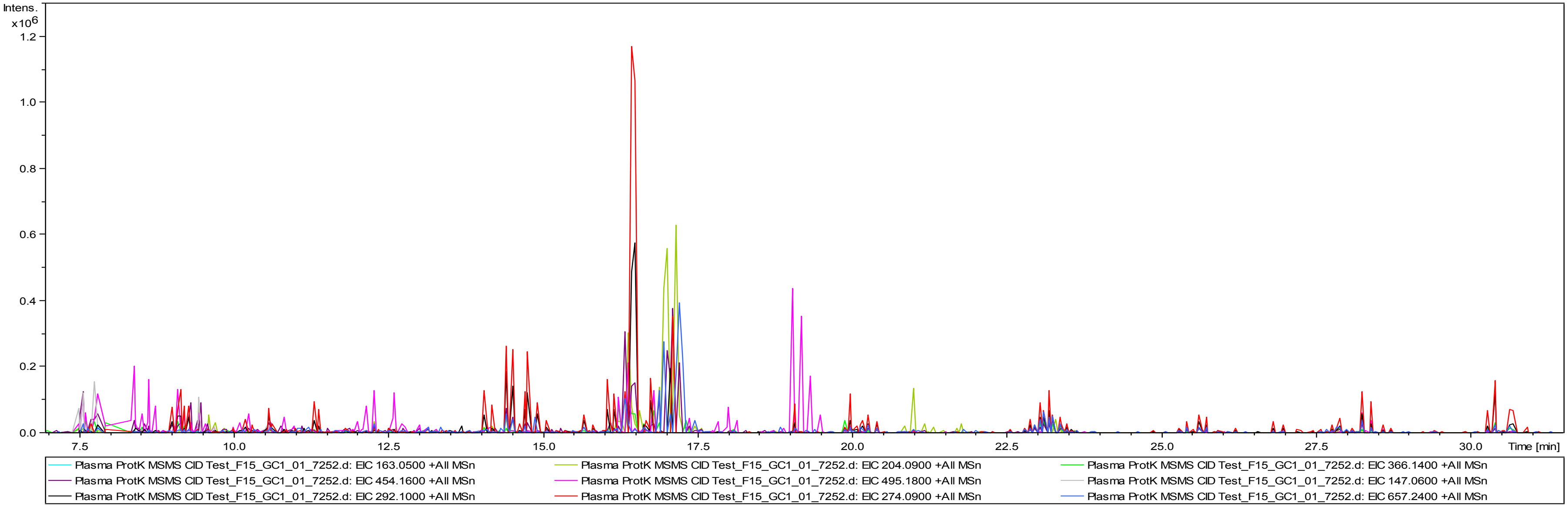

Extracted ion chromatograms of glycan-specific oxonium ions

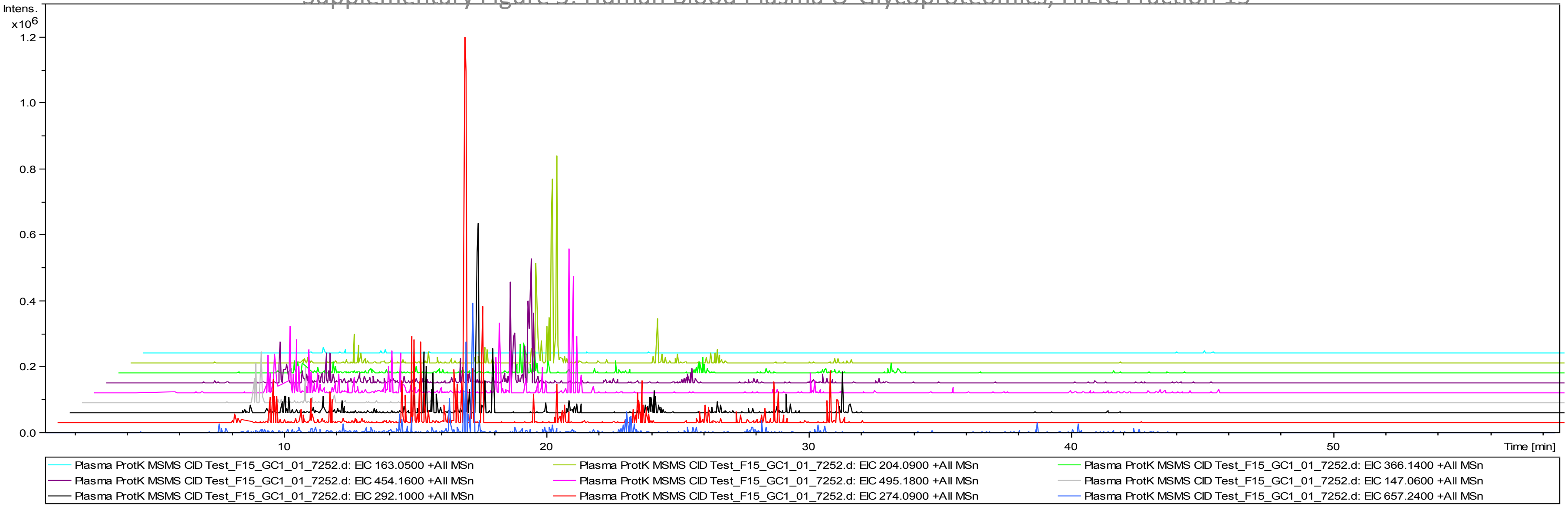

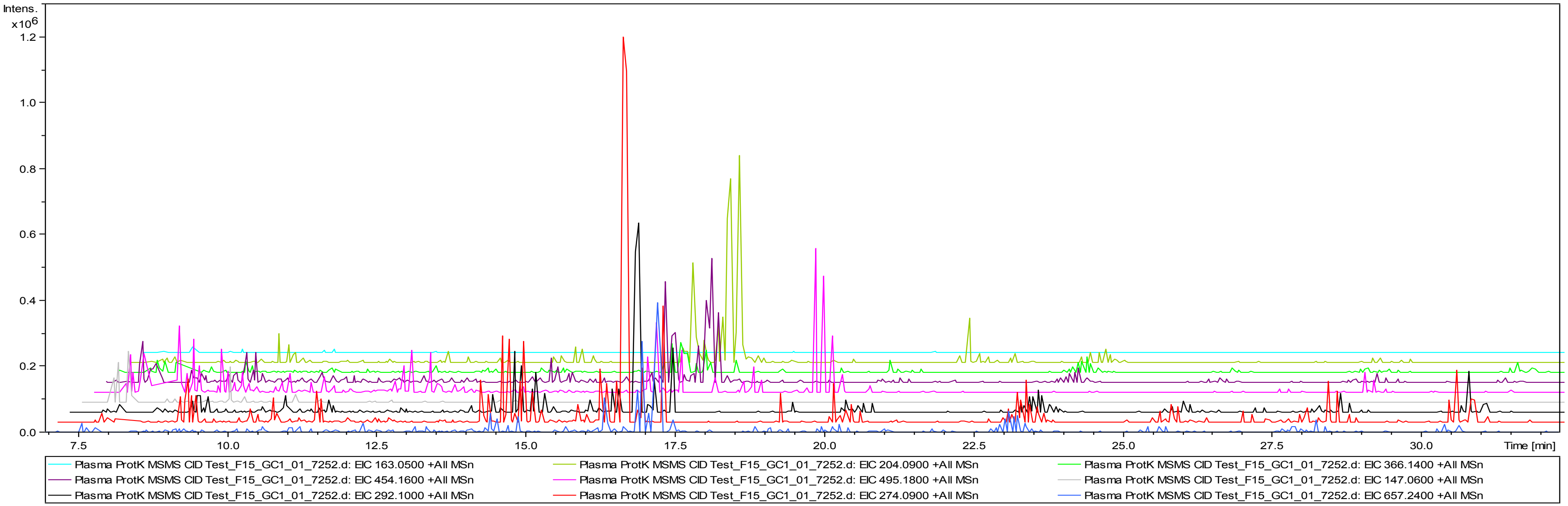

Extracted ion chromatograms of glycan-specific oxonium ions

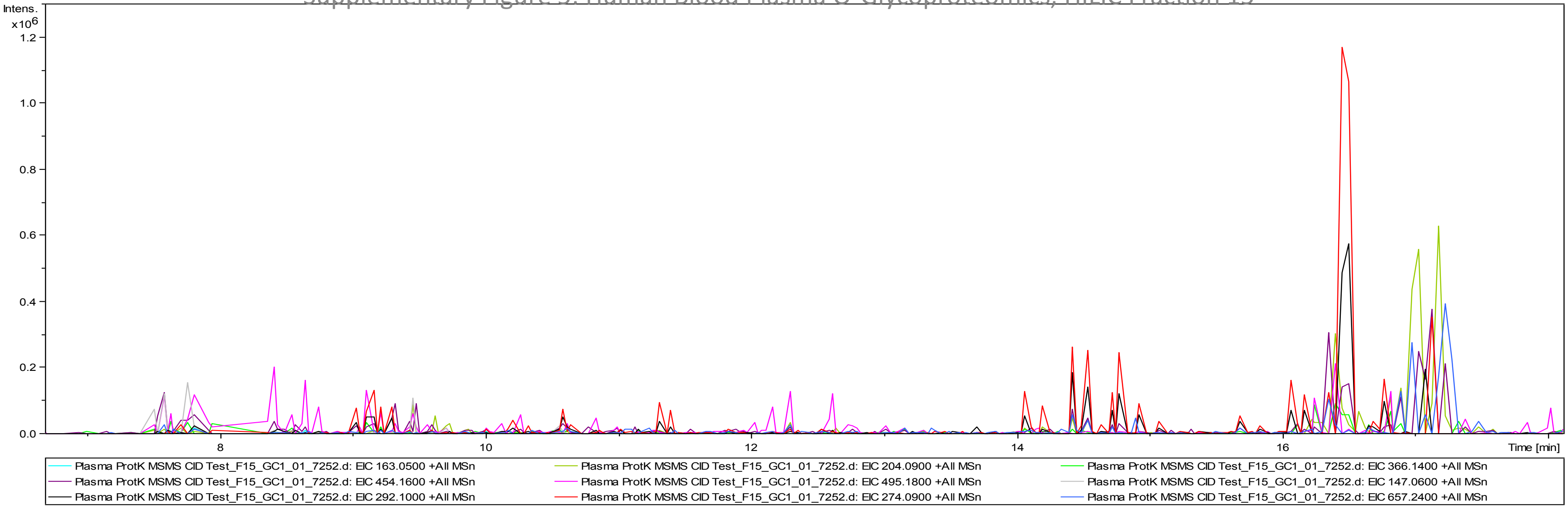

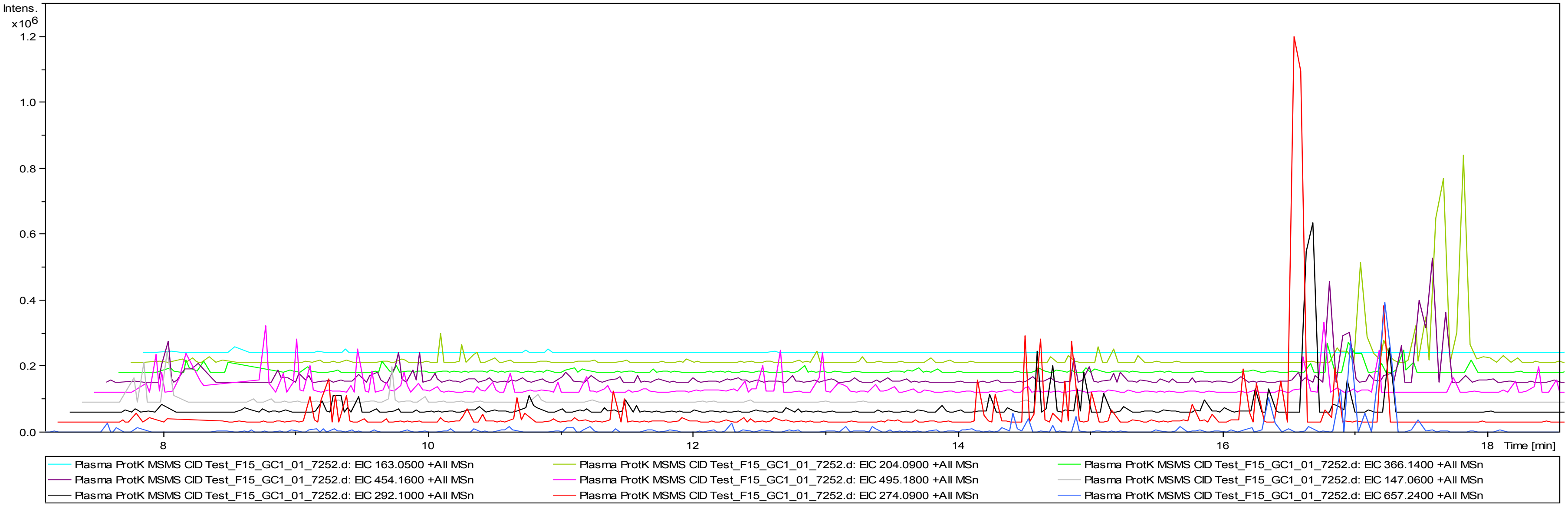

Extracted ion chromatograms of glycan-specific oxonium ions

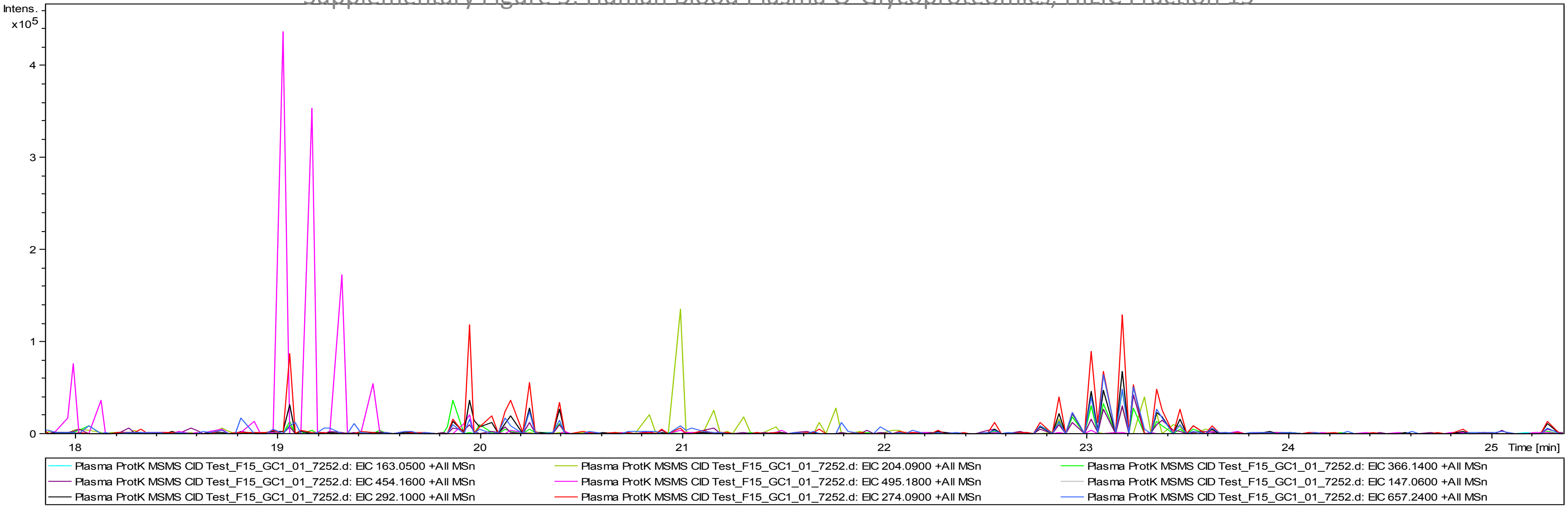

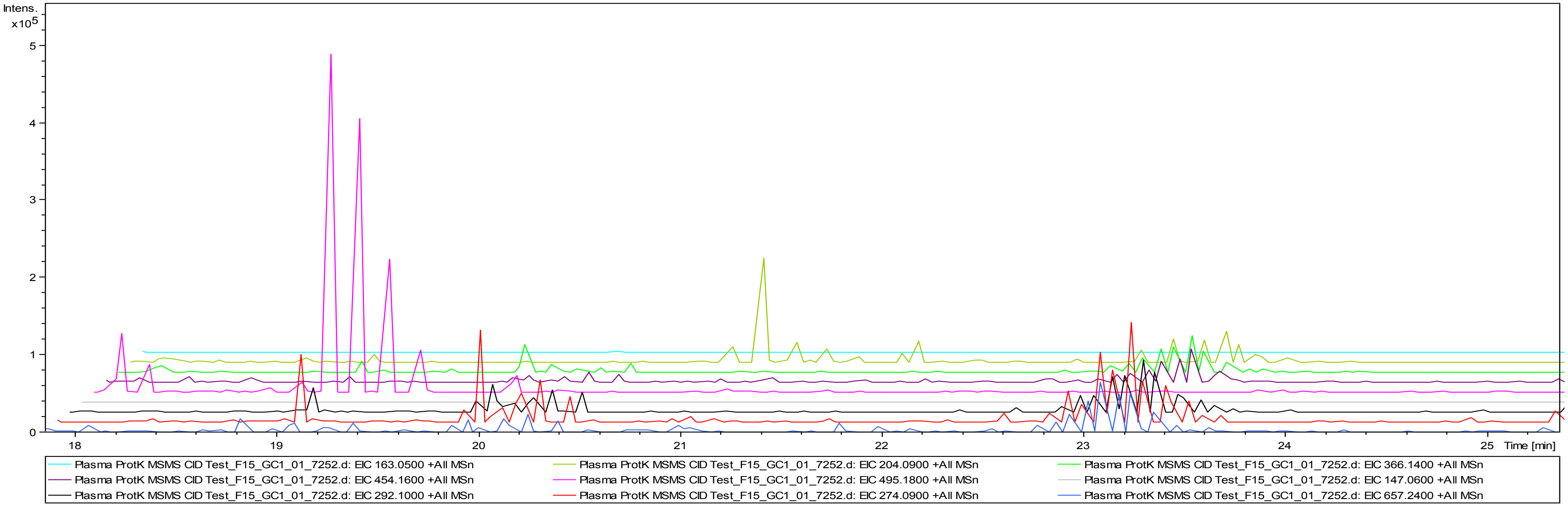

Extracted ion chromatograms of glycan-specific oxonium ions

Supplementary Figure 5: Human Blood Plasma O-Glycoproteomics, HILIC Fraction 15

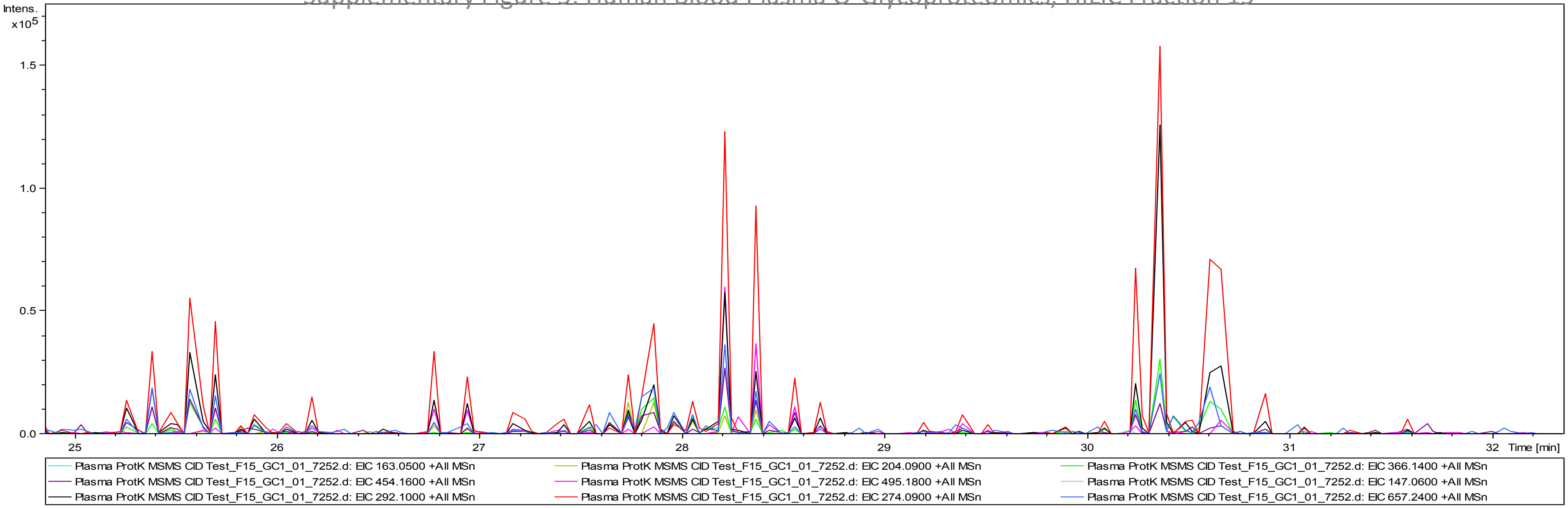

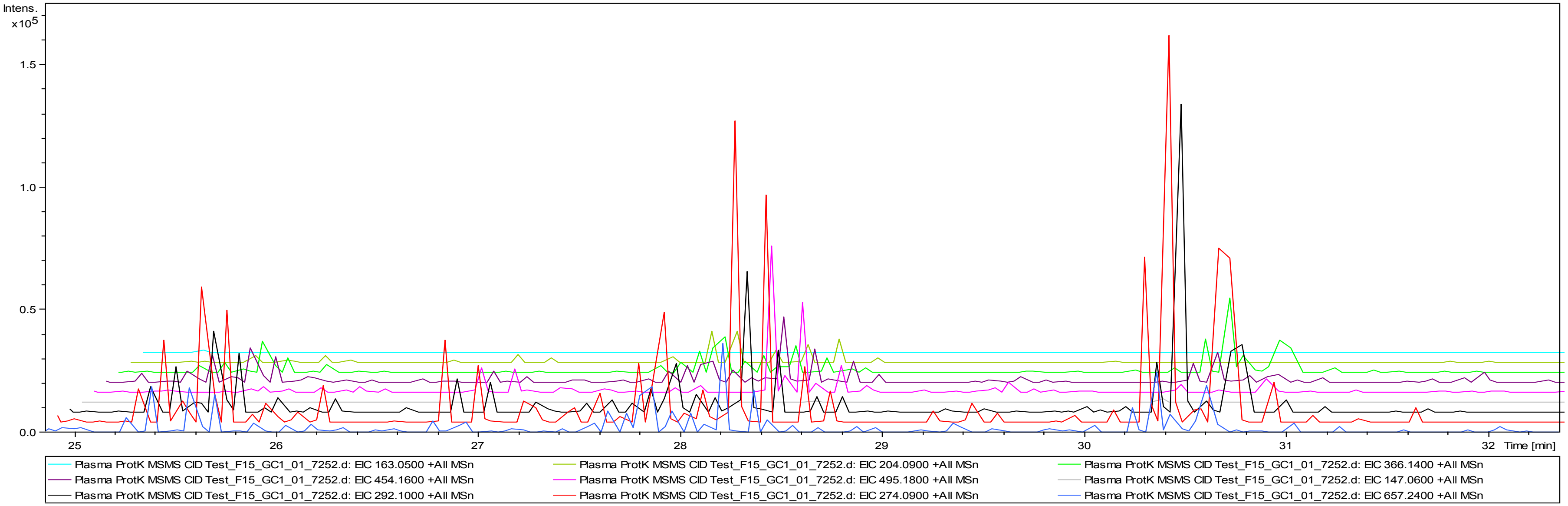

Extracted ion chromatograms of glycan-specific oxonium ions

Fraction 15

761.78++ → Pep [M+H]+ 866.41+ [9.0-9.3 min]

CID-MS Precursor

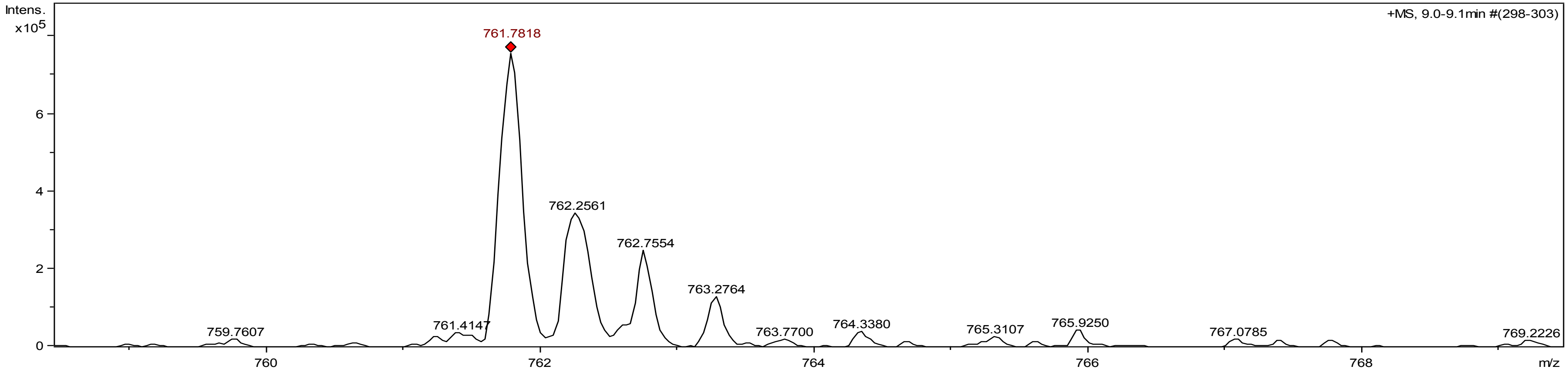

ETD spectrum of poor quality

Fraction 15

761.78++ → Pep [M+H]+ 866.41+ [9.0-9.3 min] CID-MS2

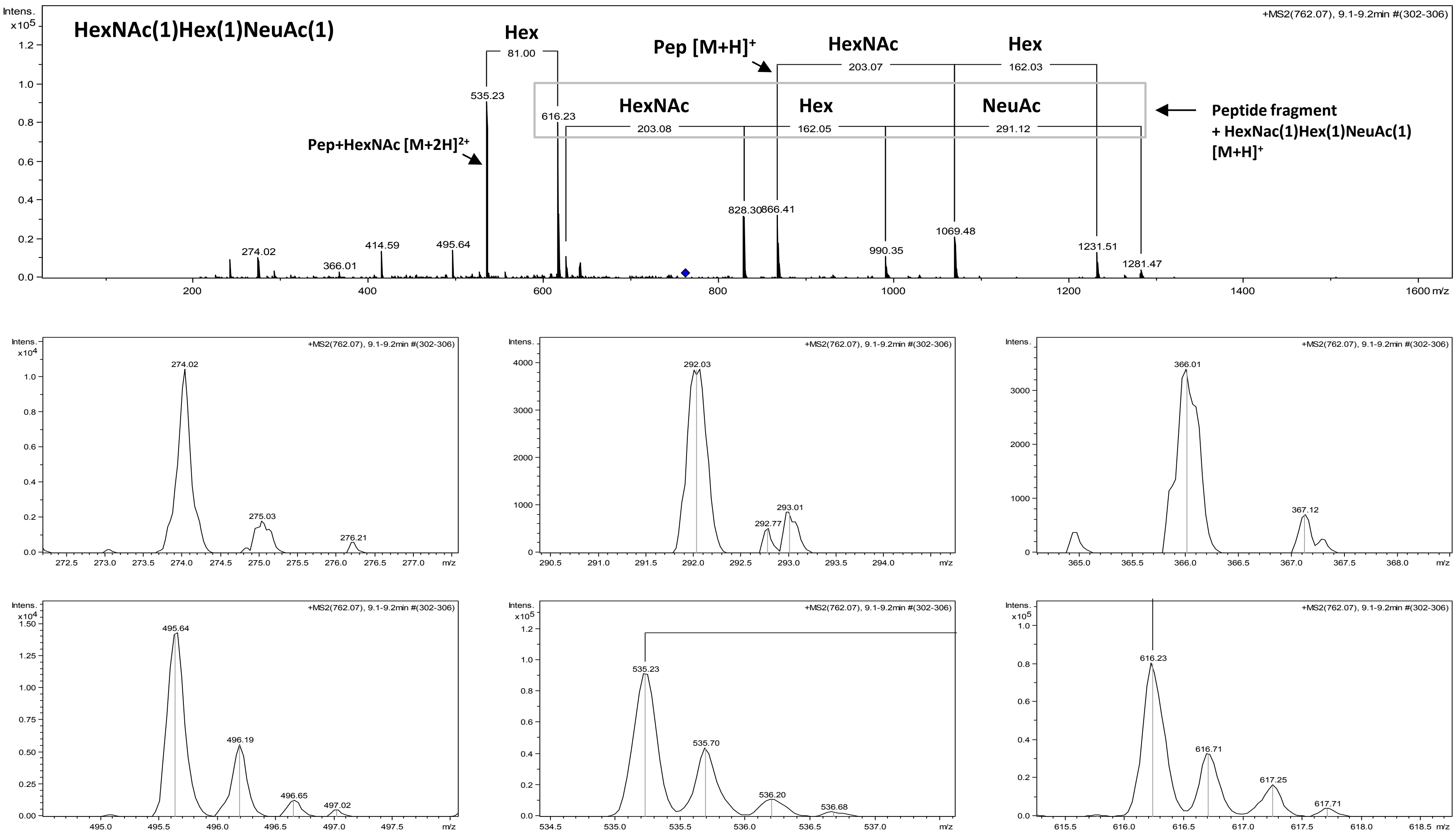

Fraction 15

761.78++ → Pep [M+H]+ 866.41+ [9.0-9.3 min] CID-MS2

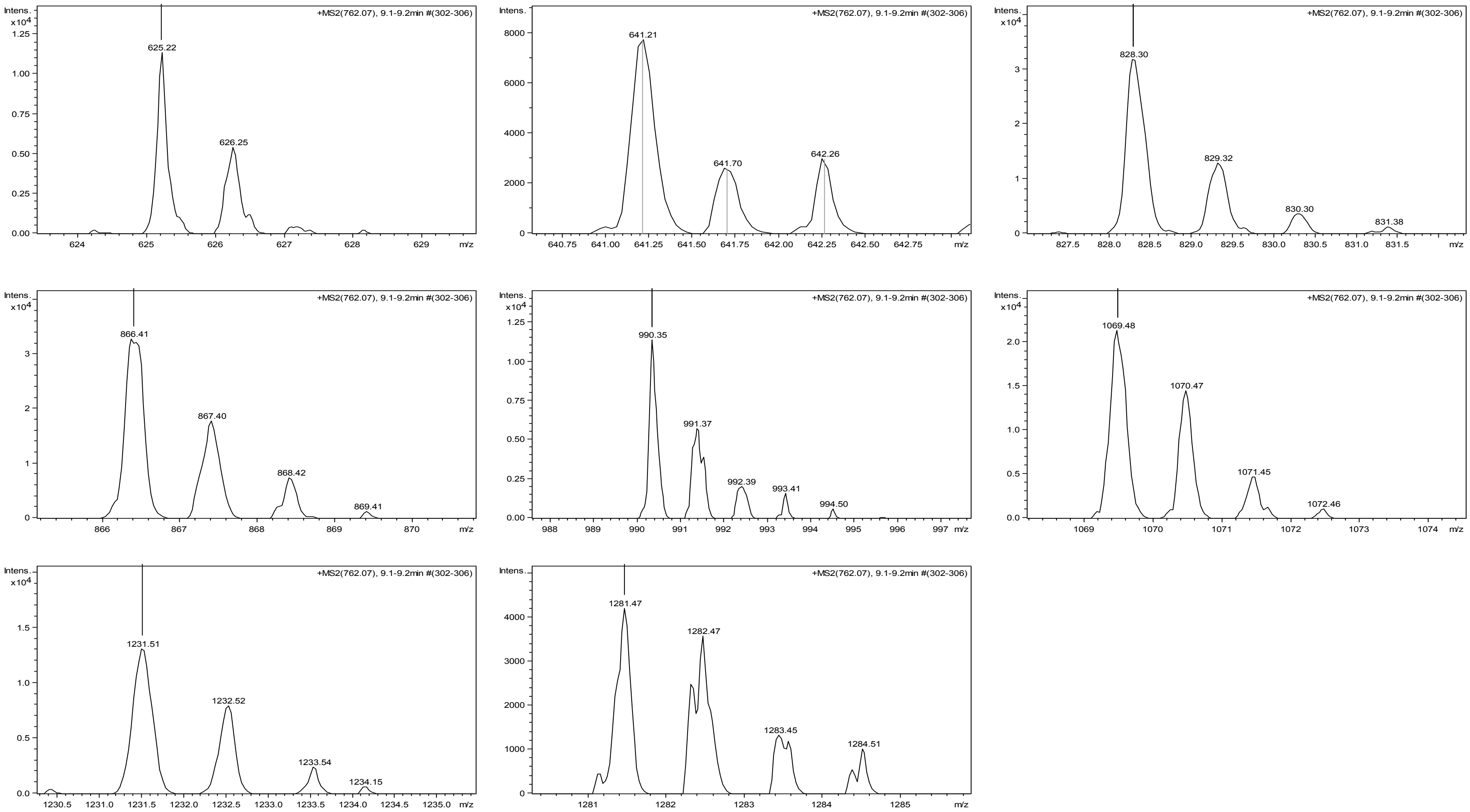

Fraction 15

761.78++ → Pep [M+H]+ 866.41+ [9.0-9.3 min] CID-MS3

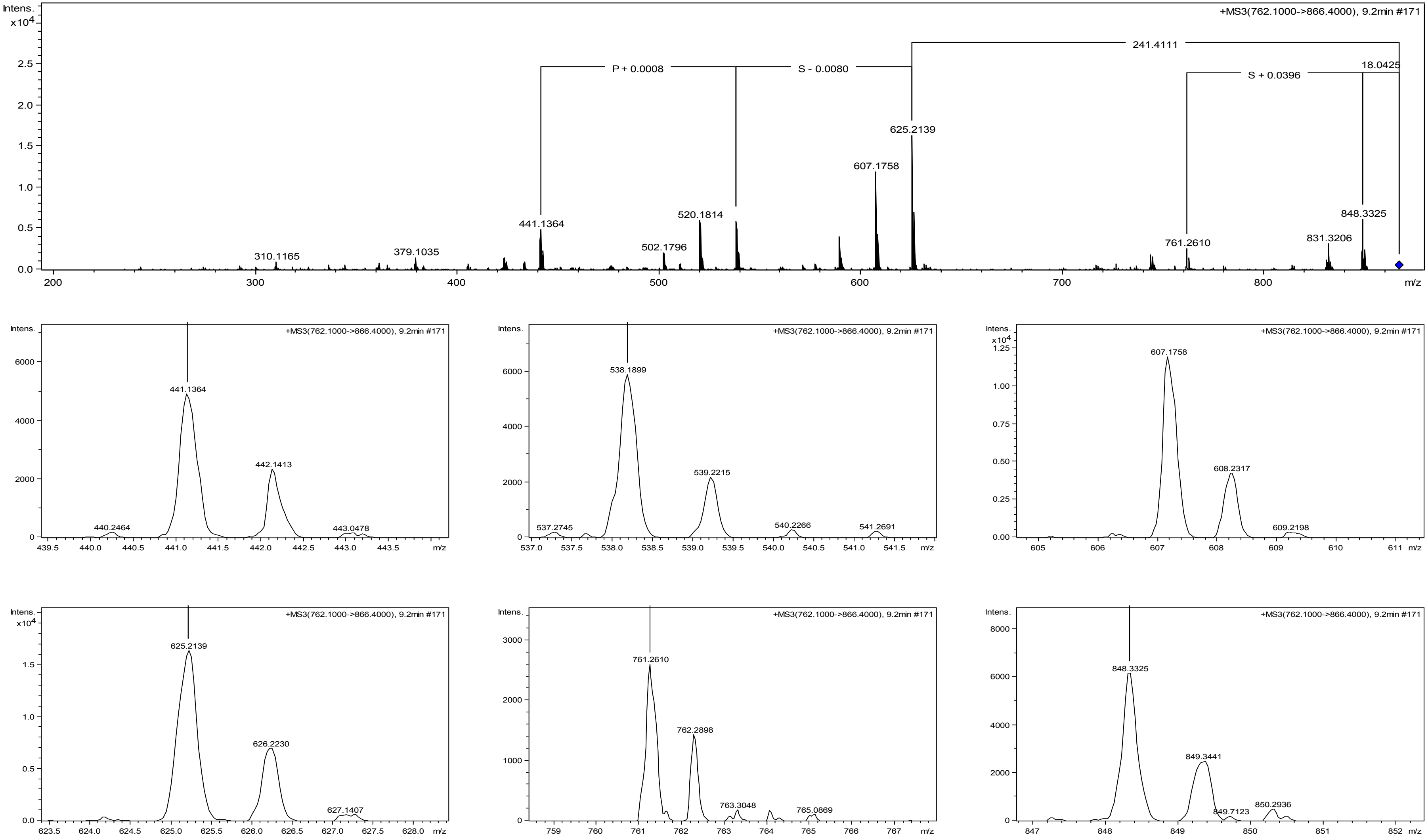

Fraction 15

761.78++ → Pep [M+H]+ 866.41+ [9.0-9.3 min] CID-MS3 MASCOT Search

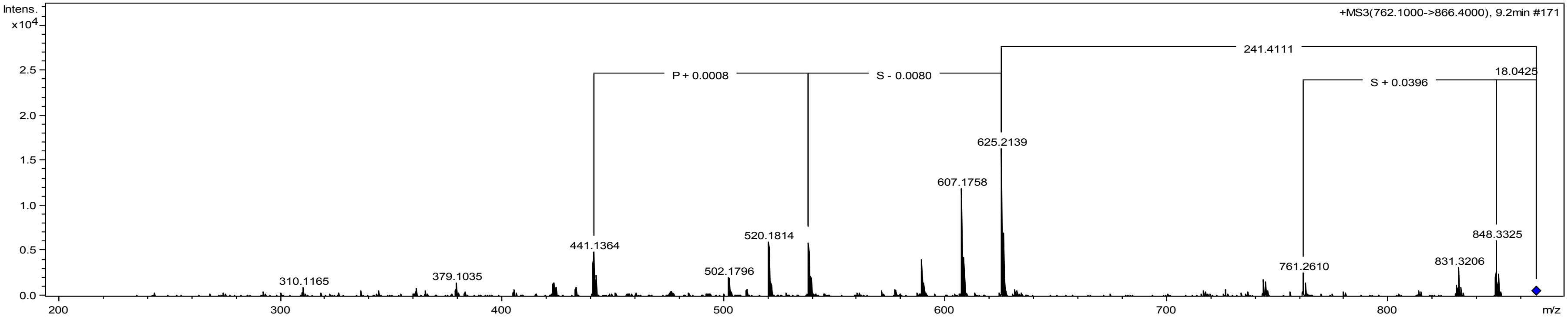

prot_hit_nur	prot_acc	prot_desc	prot_score	prot_mass	prot_matche	pep_query	pep_rank	pep_isbold	pep_exp_mz	pep_exp_mr	pep_exp_z	pep_calc_mr	pep_delta	pep_miss	pep_score	pep_expect	pep_res_bef	pep_seq
1	S3TC2_HUMAN	SH3 domain and tetratricopeptide repeats-containing protein 2	13	146681	1	1	1	1	866,4059	865,3986	1	864,4263	0,9724	0	19,34	1,60E+02	E	LLMDLSTG
2	TVB1_HUMAN	T-cell receptor beta chain V region YT35 precursor - Homo sapie	10	15429	1	1	10	0	866,4059	865,3986	1	865,4406	-0,042	0	13,76	5,80E+02	V	IQSPRHE
3	RABX5_HUMAN	Rab5 GDP/GTP exchange factor (Rabex-5) (Rabaptin-5-associat	8	80575	1	1	6	0	866,4059	865,3986	1	865,4181	-0,0195	0	14,05	5,40E+02	R	LQSNIQY
4	FA5_HUMAN	Coagulation factor V precursor (Activated protein C cofactor) [(	8	252654	1	1	4	0	866,4059	865,3986	1	865,4545	-0,0559	0	15,2	4,10E+02	S	QISPPPDL
5	CHD6_HUMAN	Chromodomain-helicase-DNA-binding protein 6 (EC 3.6.1.-) (AT	7	308071	1	1	3	0	866,4059	865,3986	1	864,3349	1,0638	0	15,42	3,90E+02	F	QESPSTNT
6	SMG1_HUMAN	Serine/threonine-protein kinase SMG1 (EC 2.7.11.1) (SMG-1) (h	7	414051	1	1	2	0	866,4059	865,3986	1	864,4263	0,9723	0	16,43	3,10E+02	N	VLIDMTTG
7	RBM10_HUMAN	RNA-binding protein 10 (RNA-binding motif protein 10) - Homo	7	103738	1	1	8	0	866,4059	865,3986	1	865,4294	-0,0308	0	13,96	5,50E+02	E	LQSPTHPS
8	TCF20_HUMAN	Transcription factor 20 (Stromelysin 1 PDGF-responsive elemer	7	213123	1	1	5	0	866,4059	865,3986	1	865,4294	-0,0307	0	15,09	4,20E+02	K	SPGSLRYS
9	CNTP4_HUMAN	Contactin-associated protein-like 4 precursor (Cell recognition	7	147220	1	1	6	0	866,4059	865,3986	1	865,4545	-0,0559	0	14,05	5,40E+02	K	LKSNLYQ
10	GASP1_HUMAN	G-protein coupled receptor-associated sorting protein 1 (GASP	7	158178	1	1	8	0	866,4059	865,3986	1	864,3349	1,0638	0	13,96	5,50E+02	T	EESNIDGT

No unambiguous result

Fraction 15

652.74++ → Pep+HexNAc [M+H]+ 851.43+ [10.0-10.4 min]

CID-MS Precursor

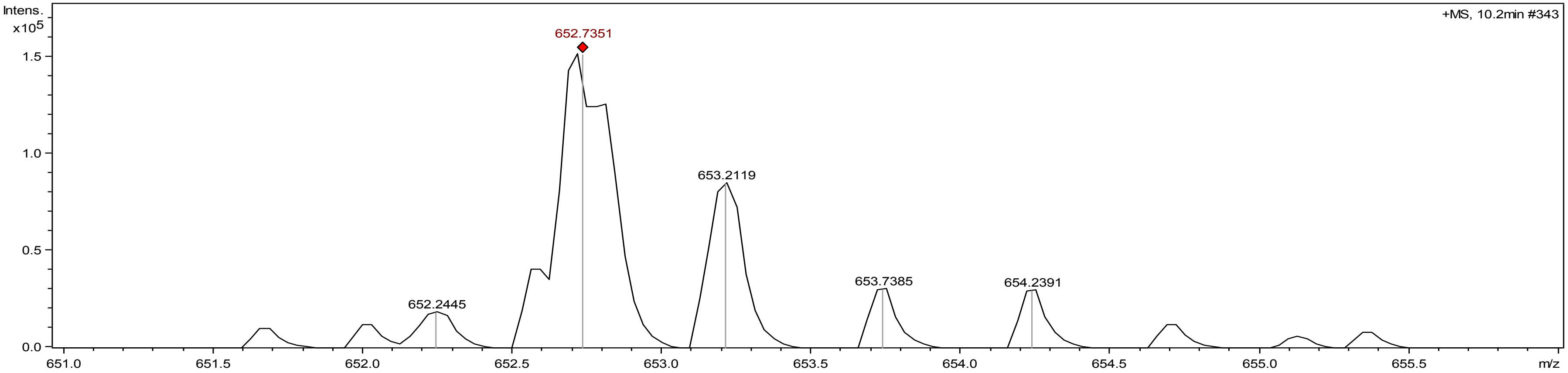

ETD spectrum of poor quality

Fraction 15

652.74++ → Pep+HexNAc [M+H]+ 851.43+ [10.0-10.4 min]

CID-MS2

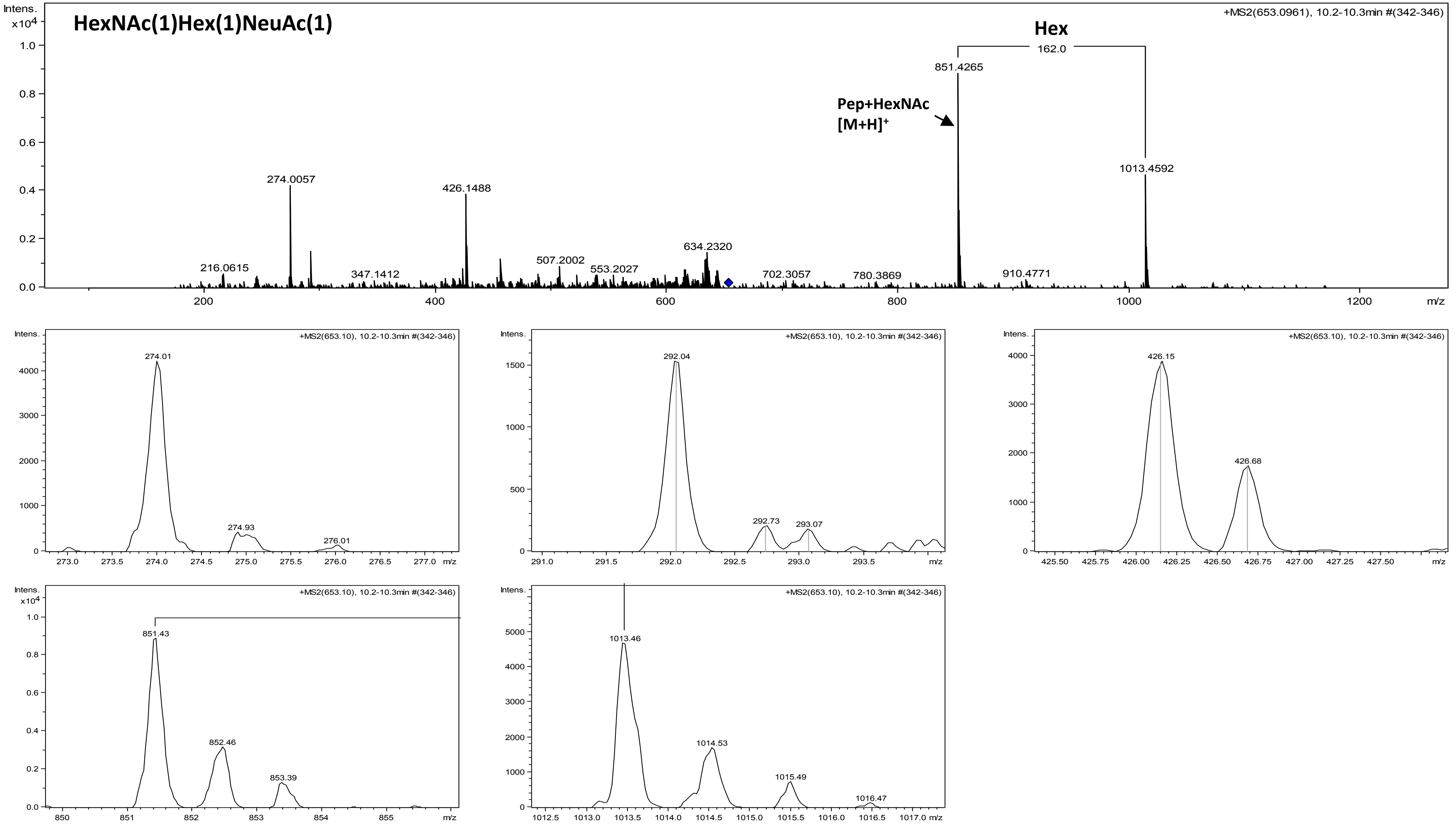

Fraction 15

652.74++ → Pep+HexNAc [M+H]+ 851.43+ [10.0-10.4 min]

CID-MS3 Manual DeNovo

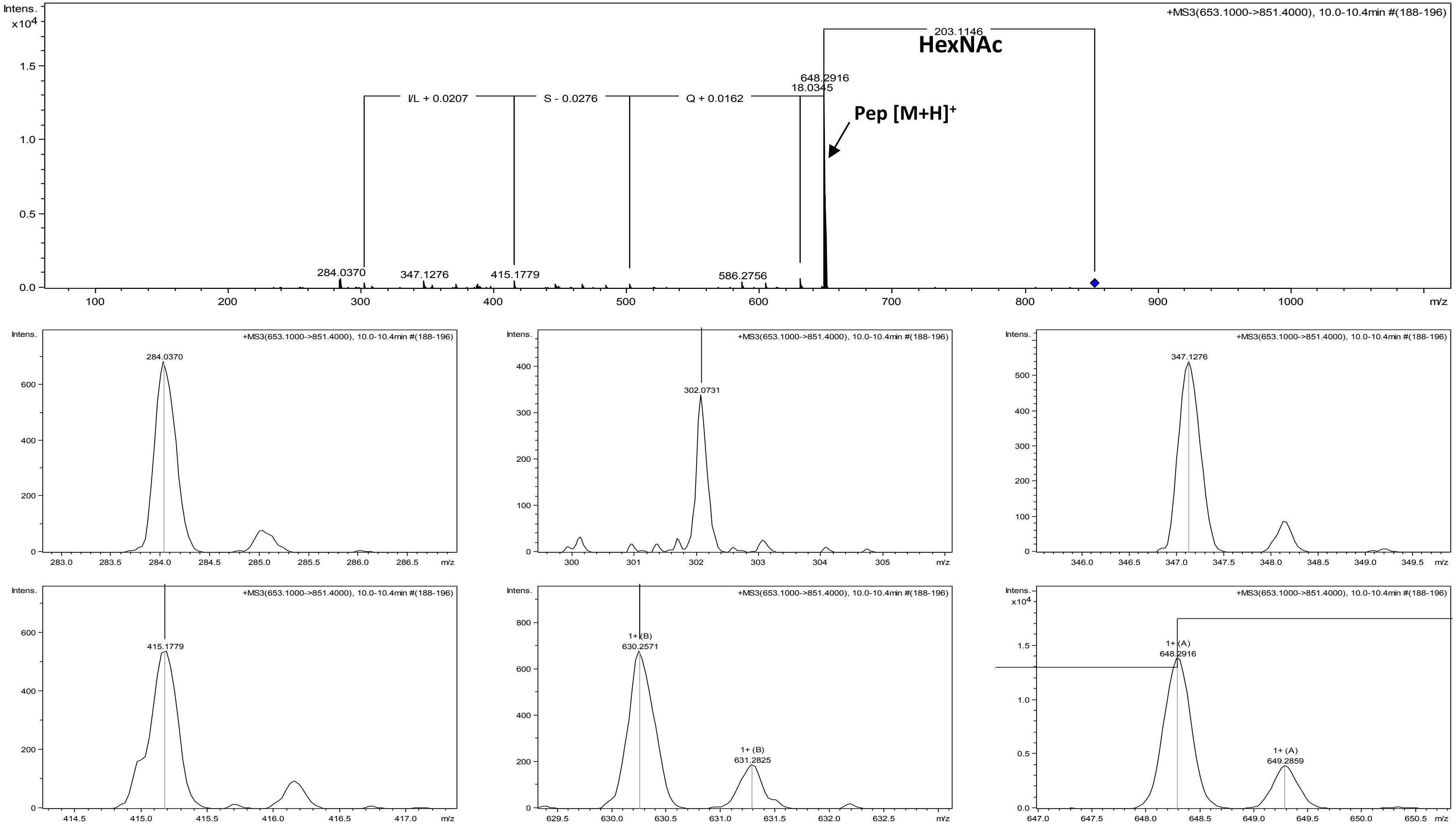

Fraction 15

652.74++ → Pep+HexNAc [M+H]+ 851.43+ [10.0-10.4 min]

CID-MS3 MASCOT Search

prot_hit_nur	prot_acc	prot_desc	prot_score	prot_mass	prot_matche	pep_query	pep_rank	pep_isbold	pep_exp_mz	pep_exp_mr	pep_exp_z	pep_calc_mr	pep_delta	pep_miss	pep_score	pep_expect	pep_res_bef	pep_seq
1	RL4_HUMAN	60S ribosoma	24	47953	1	1	1	1	648.2813	647.274	1	647.3676	-0.0936	0	27.89	25	P	ALVMSK
2	F10A5_HUM	Protein FAM	23	41579	1	1	1	0	648.2813	647.274	1	647.2948	-0.0208	0	27.89	25	N	PANMSK
3	MORF4_HUN	Transcriptior	23	26773	1	1	1	0	648.2813	647.274	1	647.2585	0.0156	0	27.89	25	P	DAPMSQ
4	DAZP1_HUM	DAZ-associat	22	43584	1	1	1	0	648.2813	647.274	1	647.2948	-0.0208	0	27.89	25	A	APDMSK
5	PCDBB_HUM	Protocadheri	21	87491	1	1	1	0	648.2813	647.274	1	647.3312	-0.0572	0	27.89	25	V	LLGMSQ
6	PITM3_HUM	Membrane-a	21	107797	1	1	1	0	648.2813	647.274	1	647.3312	-0.0572	0	27.89	25	I	LIGMSQ
7	CLOCK_HUM	Circadian loc	21	95757	1	1	1	0	648.2813	647.274	1	647.2585	0.0156	0	27.89	25	I	PQGMSQ
8	ZN536_HUM	Zinc finger pı	20	143095	1	1	1	0	648.2813	647.274	1	647.3312	-0.0572	0	27.89	25	N	LGIMSQ
9	TAF1_HUMA	Transcriptior	20	213969	1	1	1	0	648.2813	647.274	1	647.2948	-0.0208	0	27.89	25	G	EGPMSK
10	ARI1B_HUM	AT-rich inter	19	237057	1	1	1	0	648.2813	647.274	1	647.2585	0.0156	0	27.89	25	Y	GPQMSQ

No unambiguous result

Fraction 15

652.74++ → Pep+HexNAc [M+H]+ 851.43+ [10.0-10.4 min]

CID-MS3 MASCOT Search

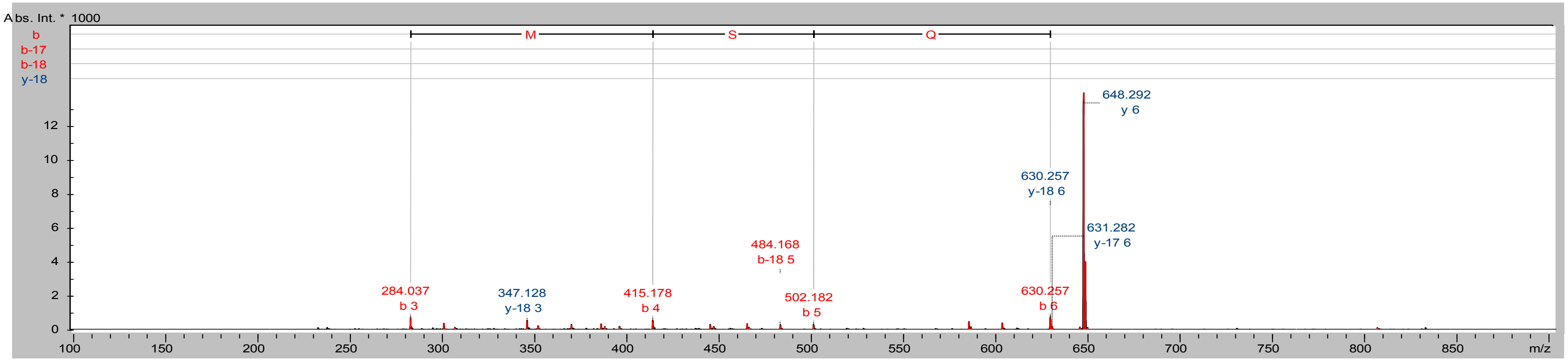

	L	L	G	M	S	Q	Leu	Leu	Gly	Met	Ser	Gln
Ion	1	2	3	4	5	6	1	2	3	4	5	6
b	L	L	G	M	S	Q	114.091	227.175	284.197	415.237	502.269	630.328
b-17	L	L	G	M	S	Q	-	-	-	-	-	613.301
b-18	L	L	G	M	S	Q	-	-	-	-	484.259	612.317
y	L	L	G	M	S	Q	147.076	234.108	365.149	422.170	535.254	648.339
y-17	L	L	G	M	S	Q	130.050	217.082	348.122	405.144	518.228	631.312
y-18	L	L	G	M	S	Q	-	216.098	347.138	404.160	517.244	630.328
	6	5	4	3	2	1	Gln	Ser	Met	Gly	Leu	Leu

Biotools-Score: 14

MASCOT-Score: 28

Most likely

unknown O-glycosylation site

Protocadherin beta 11 precursor

$_{20}$LLGM**S**Q$_{25}$

Fraction 15

660.72++ → Pep+HexNAc [M+H]+ 867.38+ [11.0-11.3 min]

CID-MS Precursor

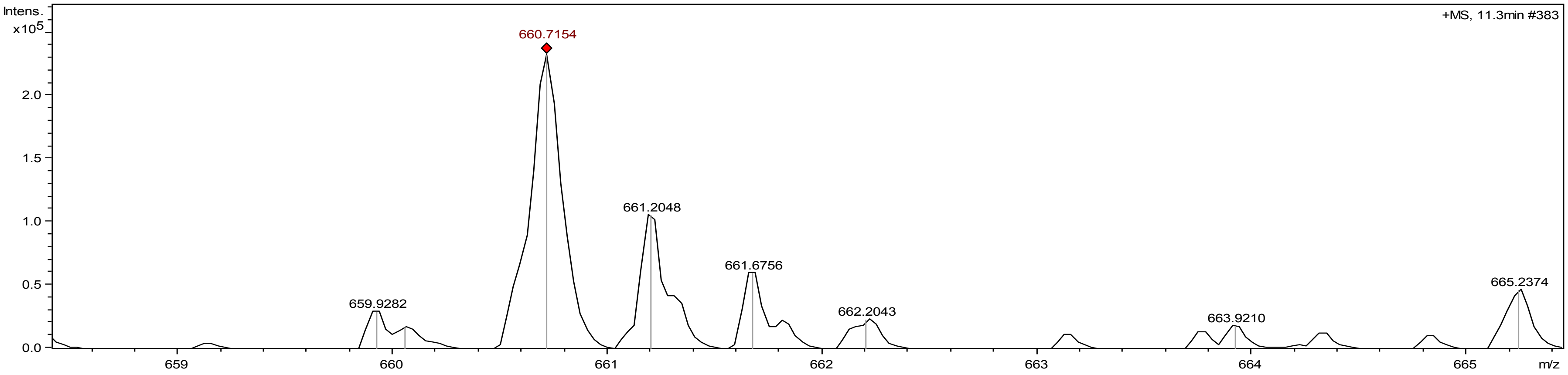

Fraction 15

660.72++ → Pep+HexNAc [M+H]+ 867.38+ [11.0-11.3 min] CID-MS2

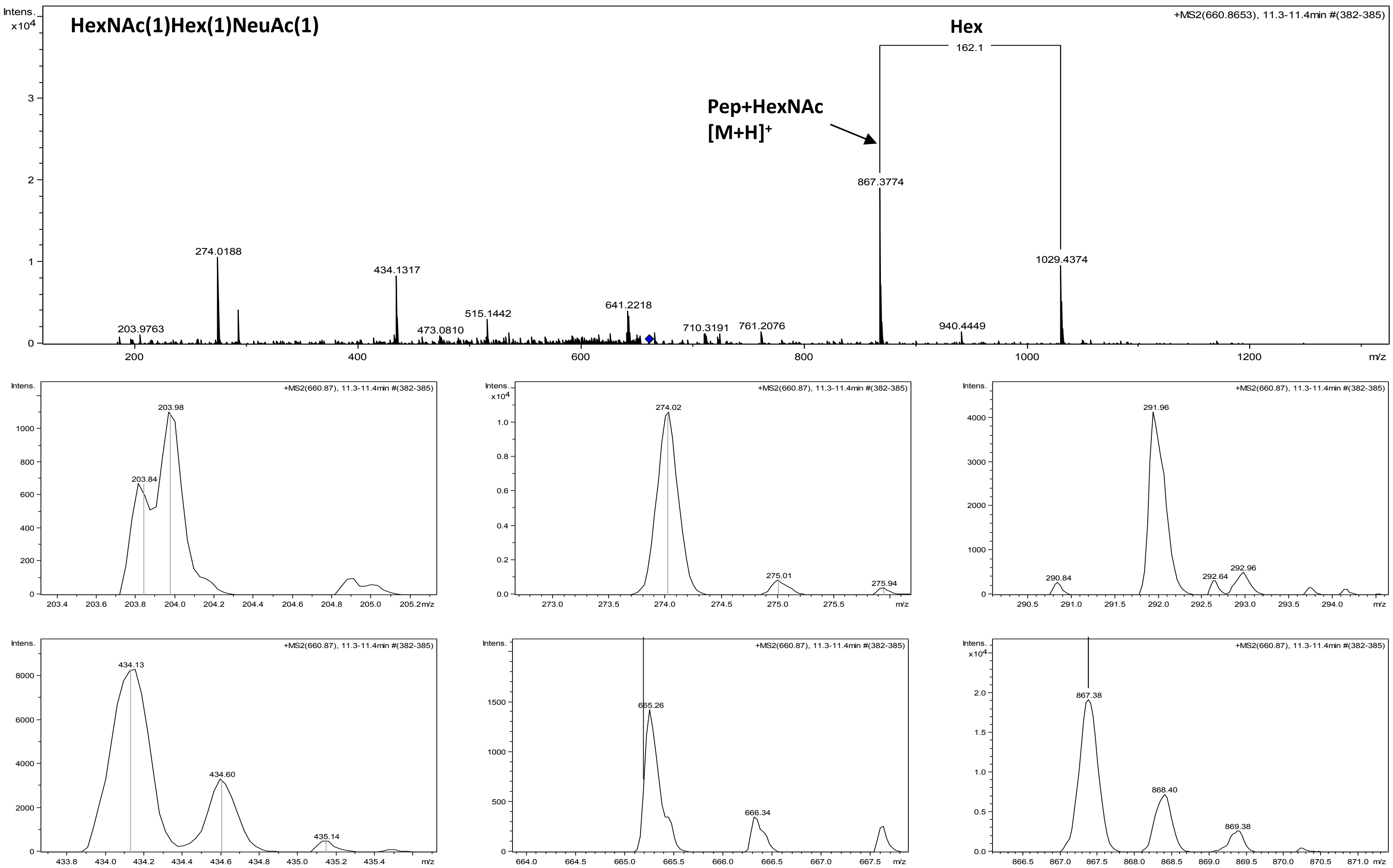

Fraction 15

660.72++ → Pep+HexNAc [M+H]+ 867.38+ [11.0-11.3 min] CID-MS2

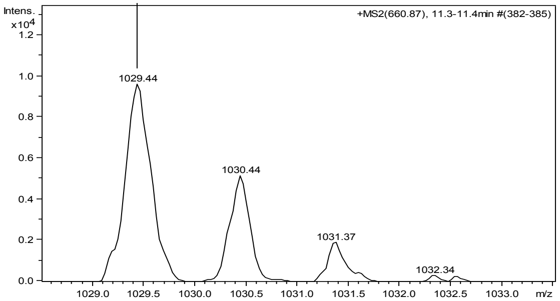

Fraction 15

660.72++ → Pep+HexNAc [M+H]+ 867.38+ [11.0-11.3 min] CID-MS3

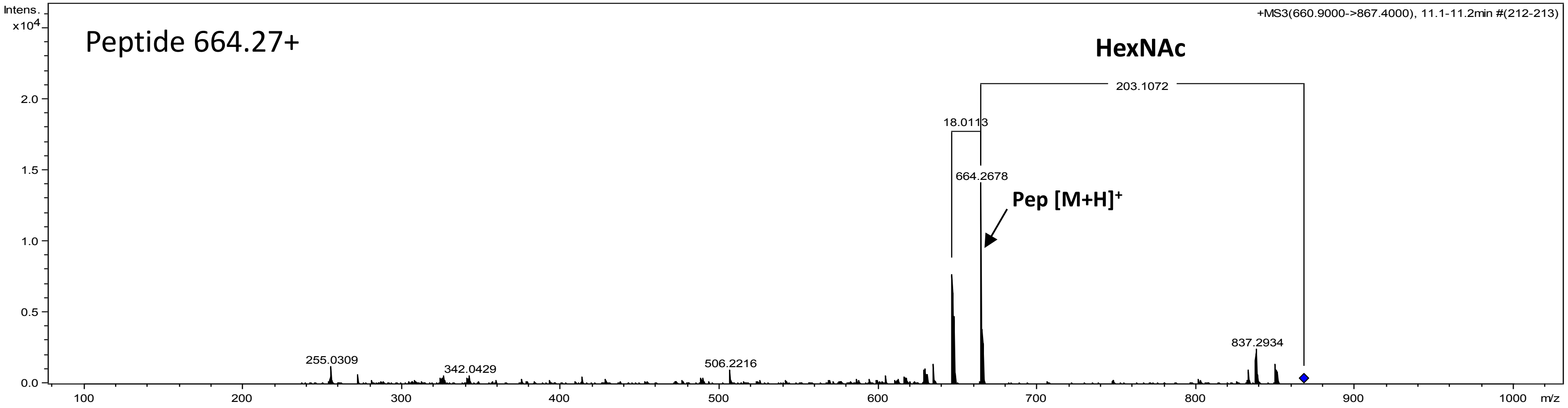

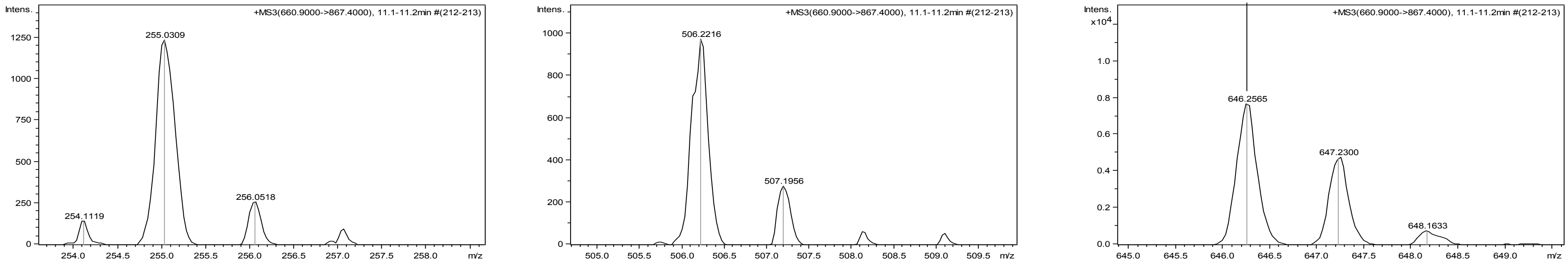

Fraction 15

660.72++ → Pep+HexNAc [M+H]+ 867.38+ [11.0-11.3 min] CID-MS3

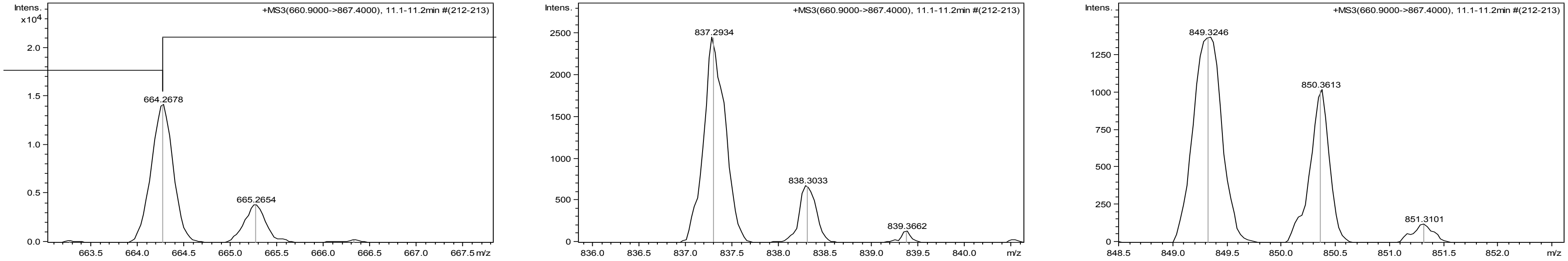

Fraction 15

660.72++ → Pep+HexNAc [M+H]+ 867.38+ [11.0-11.3 min]

CID-MS3 MASCOT Search

prot_hit_nur	prot_acc	prot_desc	prot_score	prot_mass	prot_matche	pep_query	pep_rank	pep_isbold	pep_exp_mz	pep_exp_mr	pep_exp_z	pep_calc_mr	pep_delta	pep_miss	pep_score	pep_expect	pep_res_bef	pep_seq
1	CA147_HUM/	Uncharacteri	12	30620	1	1	2	1	664.2678	663.2605	1	663.334	-0.0735	0	15.7	3.70E+02	T	GTFSPR
2	MUCB_HUM/	Ig mu heavy	12	43543	1	1	2	0	664.2678	663.2605	1	663.334	-0.0735	0	15.7	3.70E+02	A	TGFSPR
3	PRIO_HUMA	Major prion		11	27871	1	1	2	0	664.2678	663.2605	1	663.301	-0.0405	0	15.7	3.70E+02	G	SAMSRP
4	TRI25_HUMA	Tripartite mc	10	72597	1	1	1	0	664.2678	663.2605	1	663.3075	-0.047	0	15.89	3.50E+02	P	ASLSQAS
5	CG027_HUM/	HEAT repeat	10	89674	1	1	9	0	664.2678	663.2605	1	663.3075	-0.047	0	14.95	4.40E+02	L	GTLSQGT
6	ITIH4_HUMA	Inter-alpha-t	9	103489	1	1	2	0	664.2678	663.2605	1	663.334	-0.0735	0	15.7	3.70E+02	E	ASFSPR
7	IRS1_HUMAN	Insulin recep	9	132706	1	1	2	0	664.2678	663.2605	1	663.301	-0.0405	0	15.7	3.70E+02	E	GTMSRP
8	SEM6A_HUN	Semaphorin-	9	115608	1	1	9	0	664.2678	663.2605	1	663.3075	-0.047	0	14.95	4.40E+02	G	ASLSQTG
9	KCNH7_HUN	Potassium vc	9	136239	1	1	2	0	664.2678	663.2605	1	663.334	-0.0735	0	15.7	3.70E+02	M	TGFSRP
10	TTC28_HUM/	Tetratricope	8	187184	1	1	2	0	664.2678	663.2605	1	663.334	-0.0735	0	15.7	3.70E+02	T	SAFSRP

No unambiguous result

Fraction 15

660.72++ → Pep+HexNAc [M+H]+ 867.38+ [11.0-11.3 min]

CID-MS3 MASCOT Search

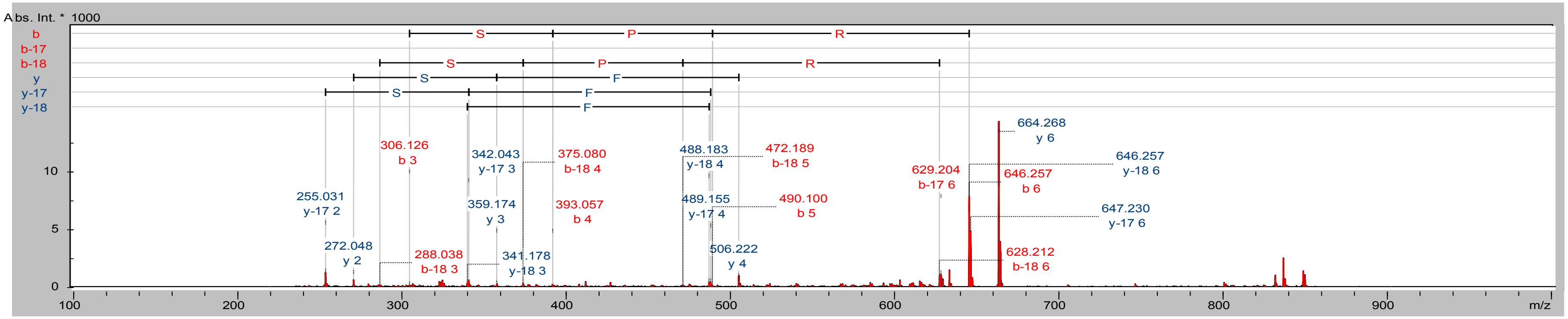

Ig mu heavy chain disease protein (BOT)

$_{75}$TGFSPR$_{80}$

	T	G	F	S	P	R	Thr	Gly	Phe	Ser	Pro	Arg
Ion	1	2	3	4	5	6	1	2	3	4	5	6
b	T	G	F	S	P	R	102.055	159.076	306.145	393.177	490.230	646.331
b-17	T	G	F	S	P	R	-	-	-	-	-	629.304
b-18	T	G	F	S	P	R	84.044	141.066	288.134	375.166	472.219	628.320
y	T	G	F	S	P	R	175.119	272.172	359.204	506.272	563.294	664.341
y-17	T	G	F	S	P	R	158.092	255.145	342.177	489.246	546.267	647.315
y-18	T	G	F	S	P	R	-	-	341.193	488.262	545.283	646.331
	6	5	4	3	2	1	Arg	Pro	Ser	Phe	Gly	Thr

Biotools-Score: 44

MASCOT-Score: 16

Fraction 15

660.72++ → Pep+HexNAc [M+H]+ 867.38+ [11.0-11.3 min]

CID-MS3 MASCOT Search

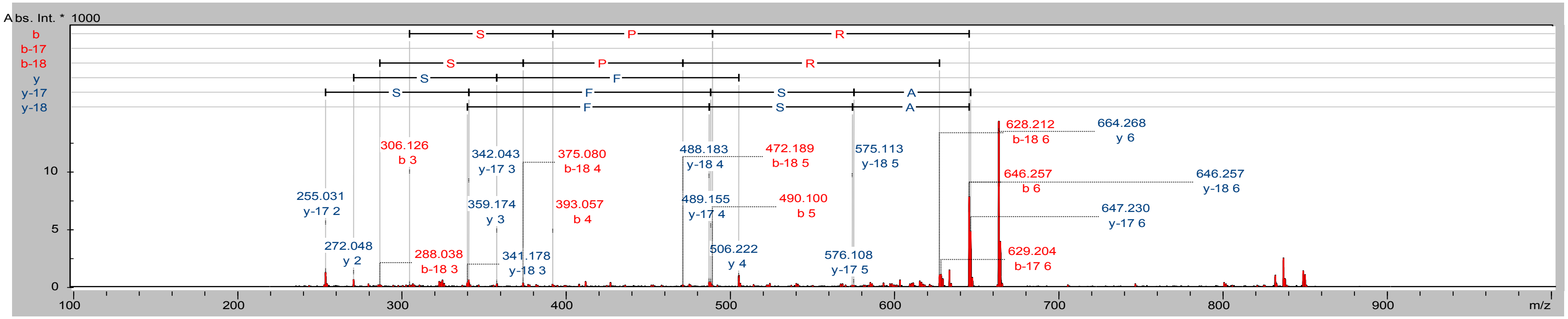

	A	S	F	S	P	R	Ala	Ser	Phe	Ser	Pro	Arg
Ion	1	2	3	4	5	6	1	2	3	4	5	6
b	A	S	F	S	P	R	72.044	159.076	306.145	393.177	490.230	646.331
b-17	A	S	F	S	P	R	-	-	-	-	-	629.304
b-18	A	S	F	S	P	R	-	141.066	288.134	375.166	472.219	628.320
y	A	S	F	S	P	R	175.119	272.172	359.204	506.272	593.304	664.341
y-17	A	S	F	S	P	R	158.092	255.145	342.177	489.246	576.278	647.315
y-18	A	S	F	S	P	R	-	-	341.193	488.262	575.294	646.331
	6	5	4	3	2	1	Arg	Pro	Ser	Phe	Ser	Ala

Biotools-Score: 51

MASCOT-Score: 16

Most likely

Inter-alpha-trypsin inhibitor heavy chain H4 precursor

$_{639}$A**S**F**S**PR$_{644}$

Fraction 15

660.72++ → Pep+HexNAc [M+H]+ 867.38+ [11.0-11.3 min] ETD

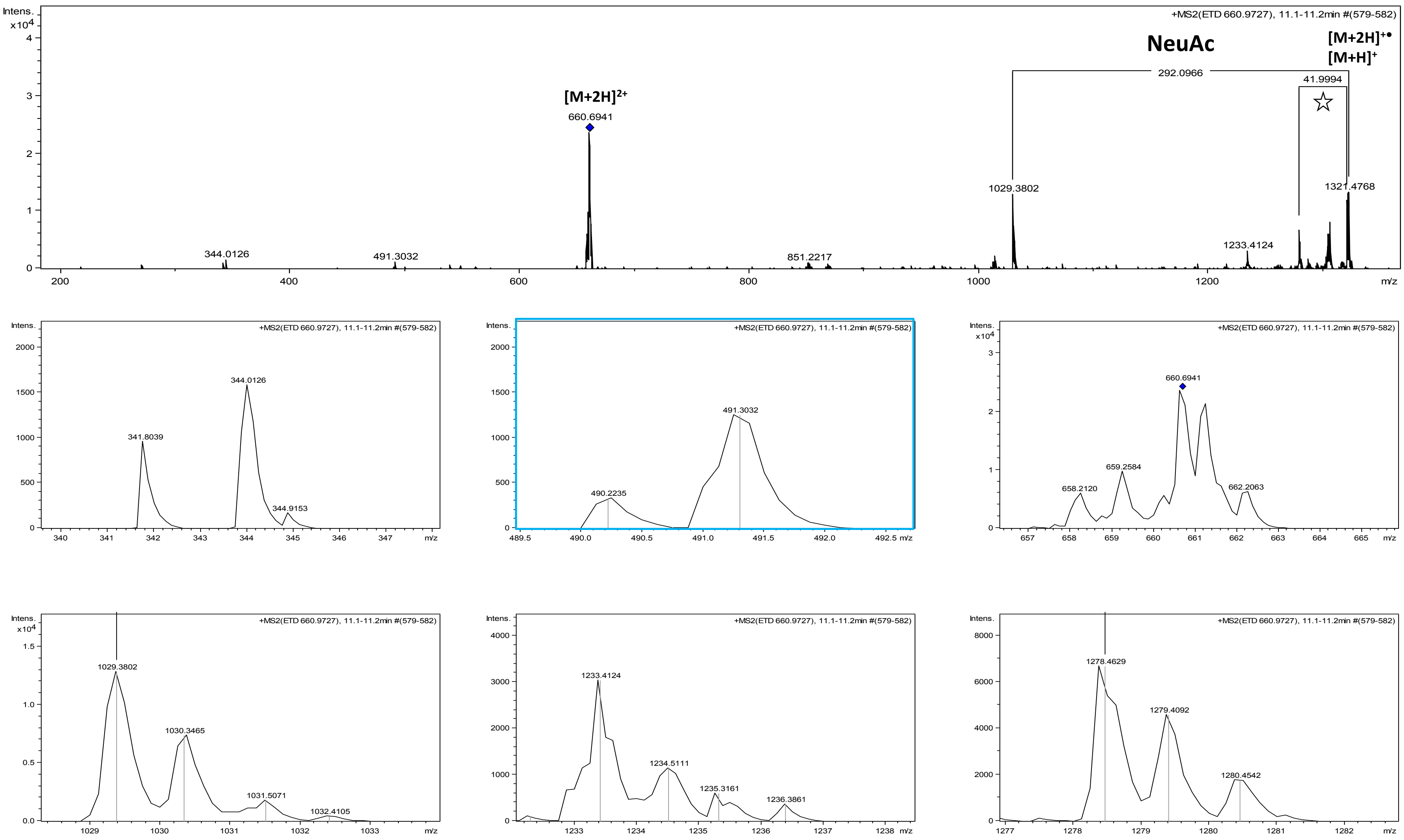

Fraction 15

660.72++ → Pep+HexNAc [M+H]+ 867.38+ [11.0-11.3 min] ETD

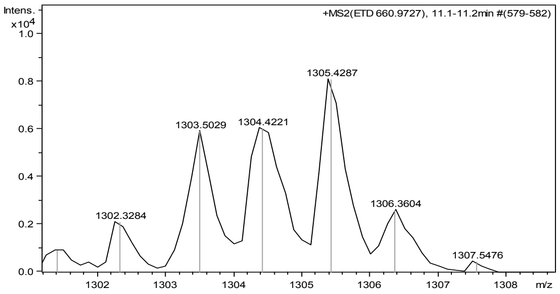

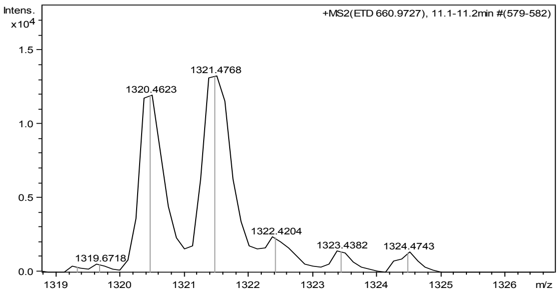

Fraction 15

660.72++ → Pep+HexNAc [M+H]+ 867.38+ [11.0-11.3 min] ETD

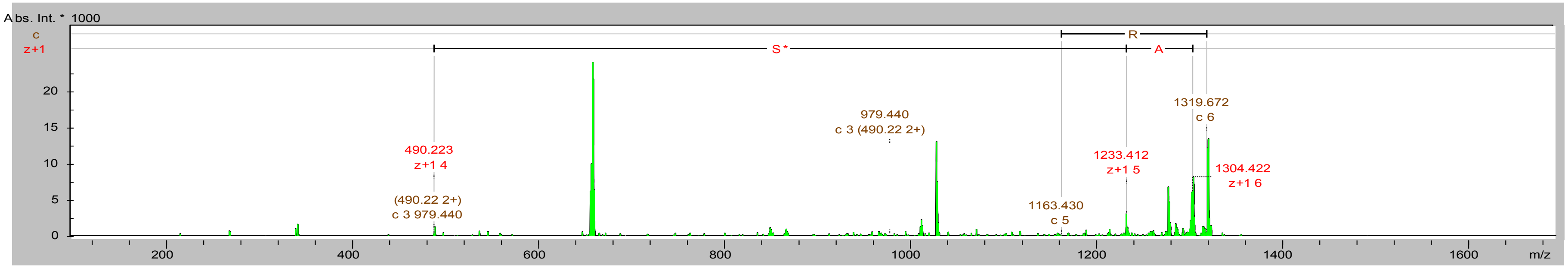

ASFSPR

	A	S	F	S	P	R	Ala	Ser	Phe	Ser	Pro	Arg
Ion	1	2	3	4	5	6	1	2	3	4	5	6
c	A	S*	F	S	P	R	89.071	832.331	979.399	1066.431	1163.484	1319.585
z+1	A	S*	F	S	P	R	159.100	256.153	343.185	490.253	1233.513	1304.550
	6	5	4	3	2	1	Arg	Pro	Ser	Phe	Ser	Ala

Biotools-Score: 11

S(640) is the most likely glycosylation site

unknown O-glycosylation site

Inter-alpha-trypsin inhibitor heavy chain H4 precursor $_{639}$ASFSPR$_{644}$

Fraction 15

660.72++ → Pep+HexNAc [M+H]+ 867.38+ [11.0-11.3 min] ETD

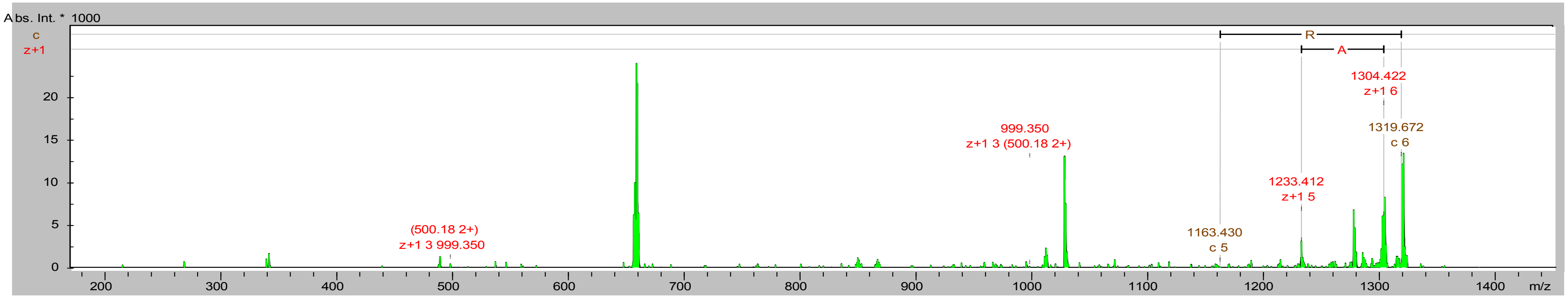

ASFSPR

	A	S	F	S	P	R	Ala	Ser	Phe	Ser	Pro	Arg
Ion	1	2	3	4	5	6	1	2	3	4	5	6
c	A	S	F	S*	P	R	89.071	176.103	323.171	1066.431	1163.484	1319.585
z+1	A	S	F	S*	P	R	159.100	256.153	999.413	1146.481	1233.513	1304.550
	6	5	4	3	2	1	Arg	Pro	Ser	Phe	Ser	Ala

Biotools-Score: 7

unknown O-glycosylation site

Inter-alpha-trypsin inhibitor heavy chain H4 precursor $_{639}$ASFSPR$_{644}$

Fraction 15

725.78++ → Pep [M+H]+ 794.34+ [12.3-12.5 min] CID-MS Precursor

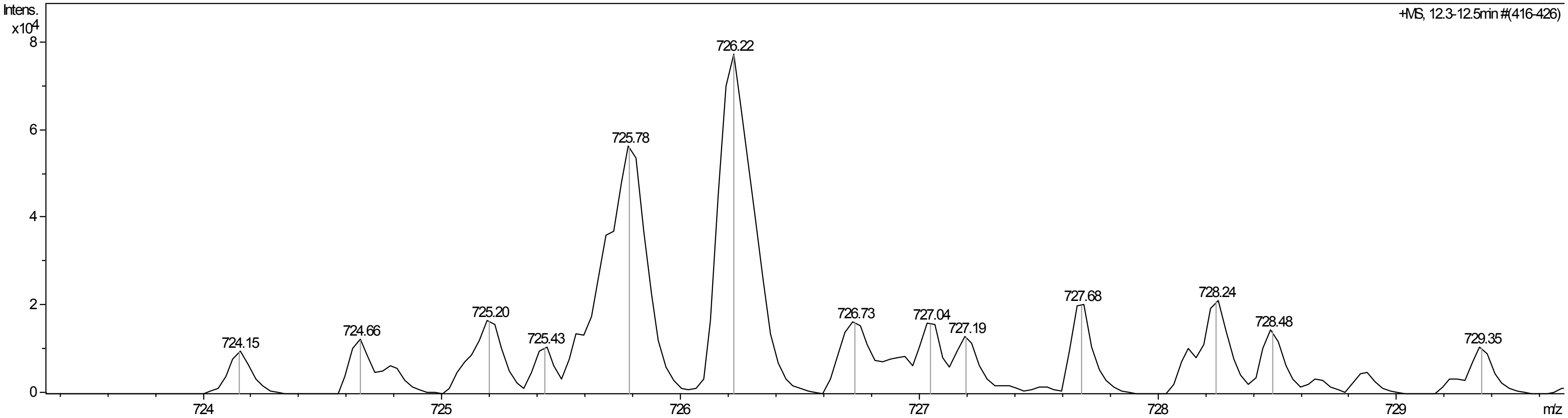

ETD spectrum of poor quality

Fraction 15

725.78++ → Pep [M+H]+ 794.34+ [12.3-12.5 min] CID-MS2

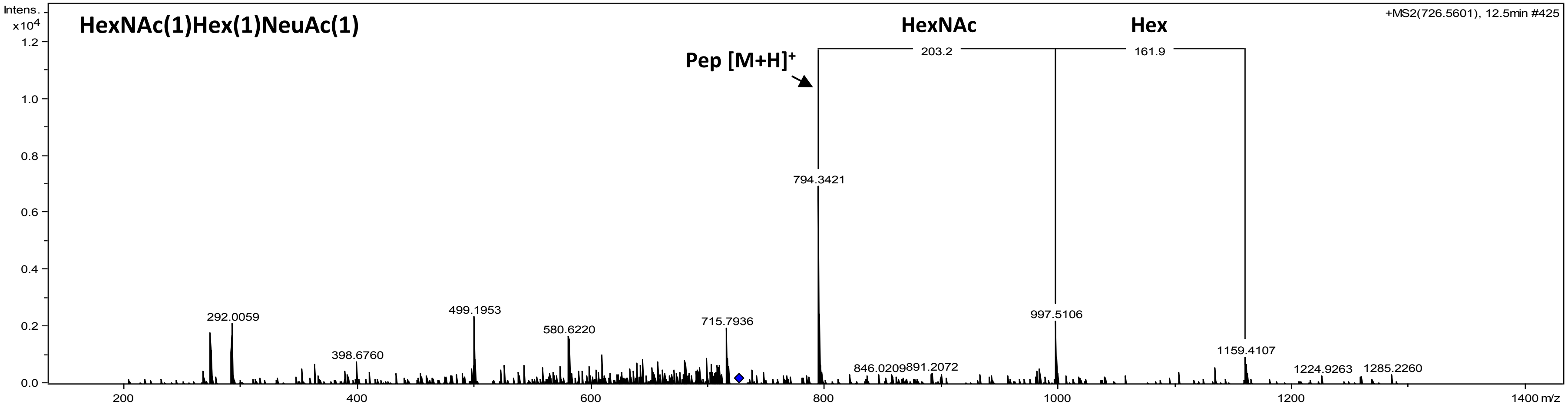

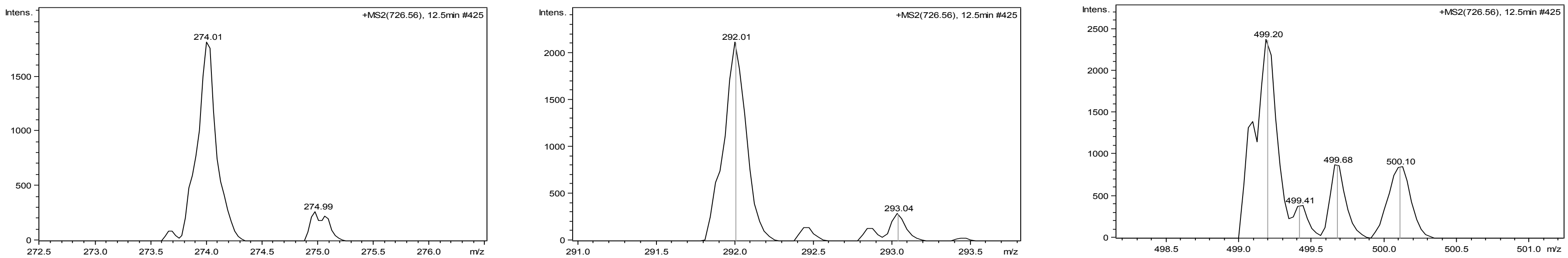

Fraction 15

725.78++ → Pep [M+H]+ 794.34+ [12.3-12.5 min]

CID-MS2

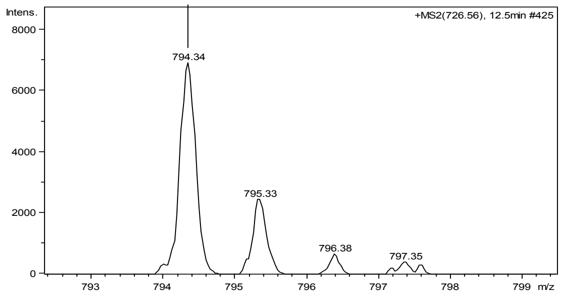

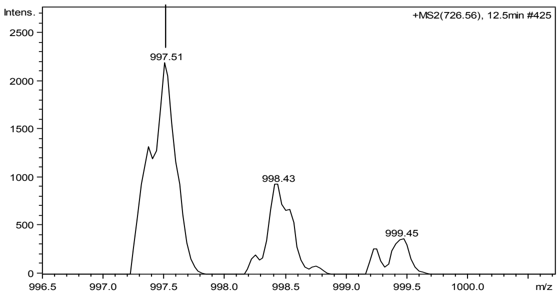

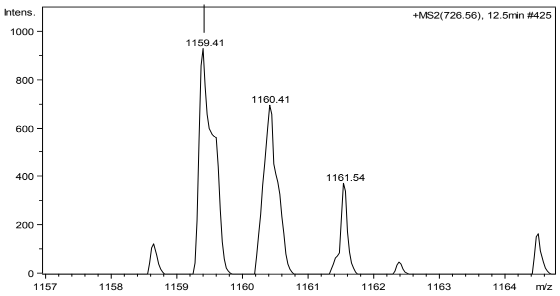

Fraction 15

725.78++ → Pep [M+H]+ 794.34+ [12.3-12.5 min]

CID-MS3 Manual DeNovo

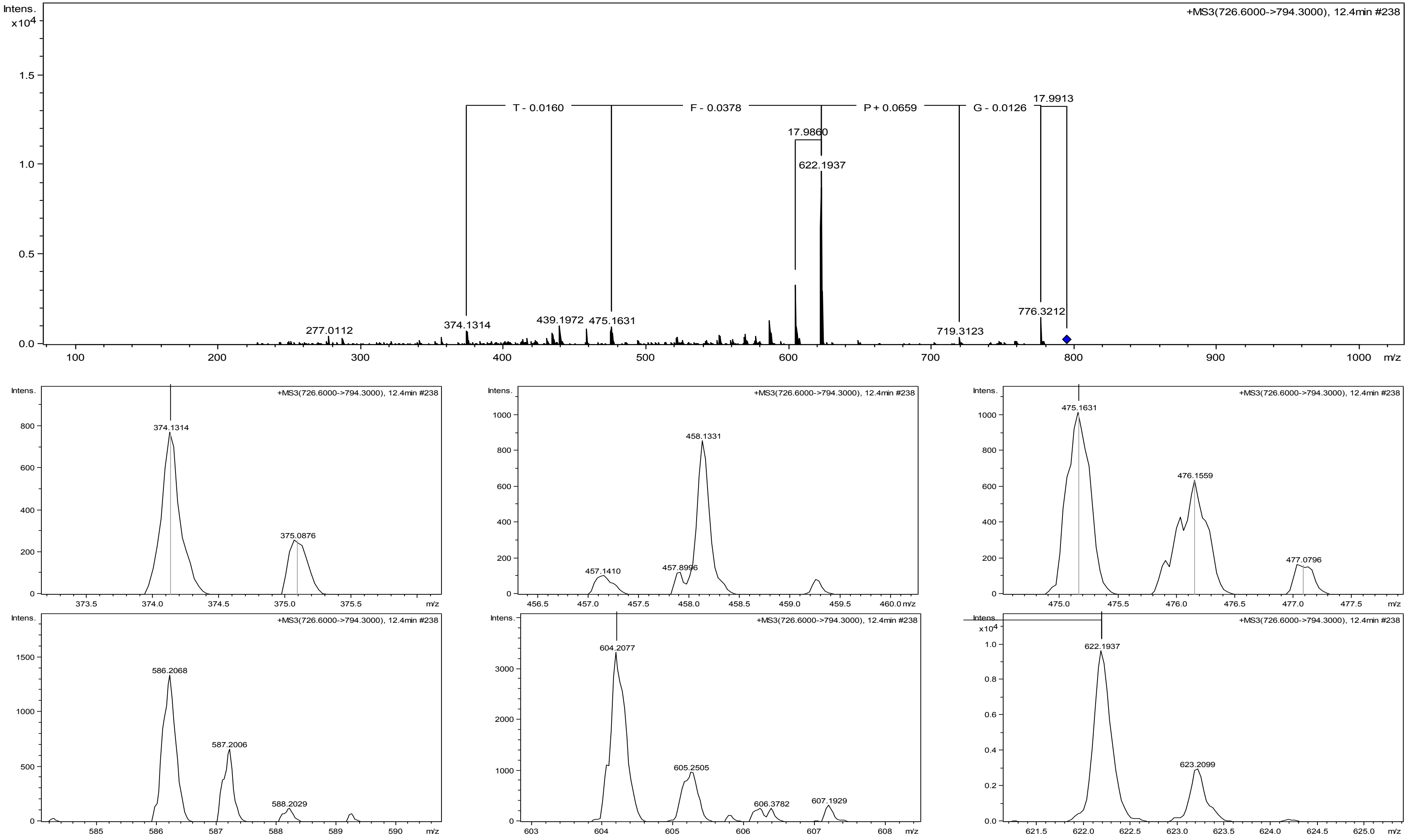

Fraction 15

725.78++ → Pep [M+H]+ 794.34+ [12.3-12.5 min]

CID-MS3 Manual DeNovo

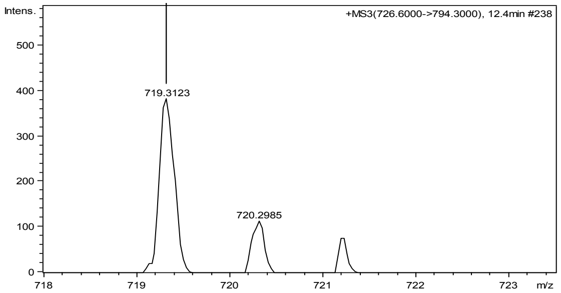

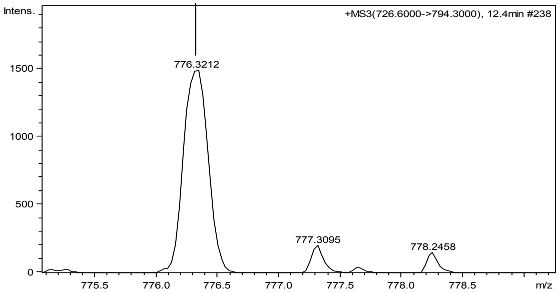

Fraction 15

725.78++ → Pep [M+H]+ 794.34+ [12.3-12.5 min]

CID-MS3 MASCOT Search

prot_hit_nur	prot_acc	prot_desc	prot_score	prot_mass	prot_matche	pep_query	pep_rank	pep_isbold	pep_exp_mz	pep_exp_mr	pep_exp_z	pep_calc_mr	pep_delta	pep_miss	pep_score	pep_expect	pep_res_bef	pep_seq
1	FIBA_HUMAI	Fibrinogen a	22	95656	1	1	1	1	794.3421	793.3348	1	793.397	-0.0622	0	27.97	21	A	STGKTFPG
2	COR2B_HUM	Coronin-2B (	21	54928	1	1	4	0	794.3421	793.3348	1	793.368	-0.0332	0	25.36	39	D	IYPMTPG
3	UN45A_HUM	UNC45 homc	20	104266	1	1	2	0	794.3421	793.3348	1	793.3316	0.0032	0	25.5	38	N	PEMTFPG
4	STAG1_HUM	Cohesin subu	19	145323	1	1	2	0	794.3421	793.3348	1	793.368	-0.0332	0	25.5	38	D	YPLTMPG
5	CHODL_HUM	Chondrolect	18	31095	1	1	8	0	794.3421	793.3348	1	793.3494	-0.0146	0	22.33	78	P	YLTNQPG
6	FLNC_HUMA	Filamin-C (G	18	293344	1	1	4	0	794.3421	793.3348	1	793.401	-0.0662	0	25.36	39	E	YIPFTPG
7	DSPP_HUMA	Dentin sialop	16	126472	1	1	6	0	794.3421	793.3348	1	793.2614	0.0735	0	22.36	78	R	QENTQDG
8	JHD2A_HUM	JmjC domain	16	149458	1	1	6	0	794.3421	793.3348	1	793.3454	-0.0105	0	22.36	78	S	STGKTENG
9	ITSN1_HUM/	Intersectin-1	13	196155	1	1	10	0	794.3421	793.3348	1	793.3647	-0.0298	0	20.51	1.20E+02	A	QFPTPFG
10	FATH_HUMA	Cadherin-rel	13	509384	1	1	8	0	794.3421	793.3348	1	793.313	0.0218	0	22.33	78	V	FENTEPG

known O-glycosylation region

Fibrinogen alpha chain precursor

$_{524}$**STGKTFPG**$_{531}$

Same glycopetide found by Zauner et al. 2012

Fraction 15

725.78++ → Pep [M+H]+ 794.34+ [12.3-12.5 min]   CID-MS3   MASCOT Search

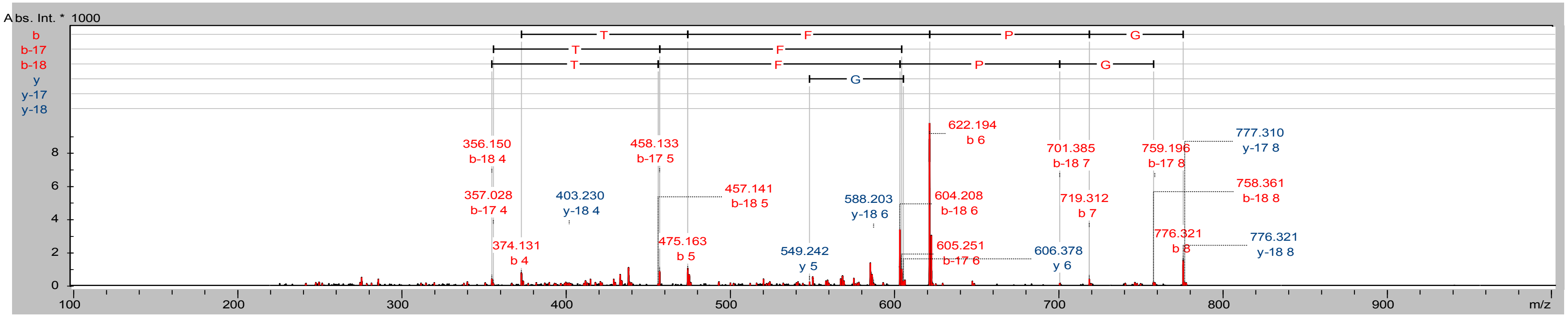

	S	T	G	K	T	F	P	G	Ser	Thr	Gly	Lys	Thr	Phe	Pro	Gly
Ion	1	2	3	4	5	6	7	8	1	2	3	4	5	6	7	8
b	S	T	G	K	T	F	P	G	88.039	189.087	246.108	374.203	475.251	622.320	719.372	776.394
b-17	S	T	G	K	T	F	P	G	-	-	-	357.177	458.225	605.293	702.346	759.367
b-18	S	T	G	K	T	F	P	G	70.029	171.076	228.098	356.193	457.241	604.309	701.362	758.383
y	S	T	G	K	T	F	P	G	76.039	173.092	320.160	421.208	549.303	606.325	707.372	794.404
y-17	S	T	G	K	T	F	P	G	-	-	-	-	532.277	589.298	690.346	777.378
y-18	S	T	G	K	T	F	P	G	-	-	-	403.198	531.293	588.314	689.362	776.394
	8	7	6	5	4	3	2	1	Gly	Pro	Phe	Thr	Lys	Gly	Thr	Ser

Biotools-Score: 61

MASCOT-Score: 28

known O-glycosylation region

Fibrinogen alpha chain precursor

$_{524}$**ST**GK**T**FPG$_{531}$

Same glycopetide found by Zauner et al. 2012

Fraction 15

584.24+++ → Pep [M+2H]++ 547.76++ [13.8-14.2 min] CID-MS Precursor

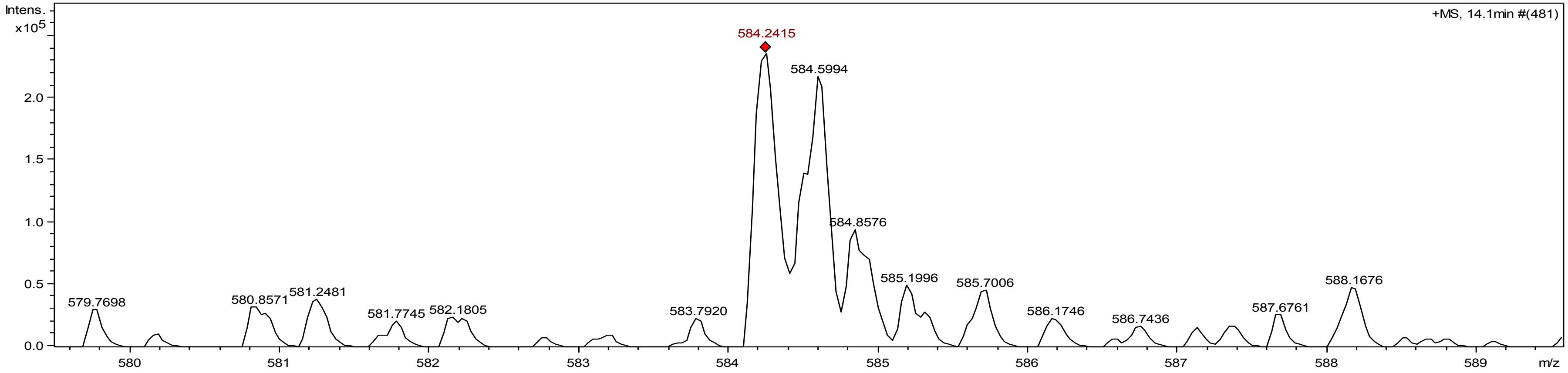

Fraction 15

584.24+++ → Pep [M+2H]++ 547.76++ [13.8-14.2 min] CID-MS2

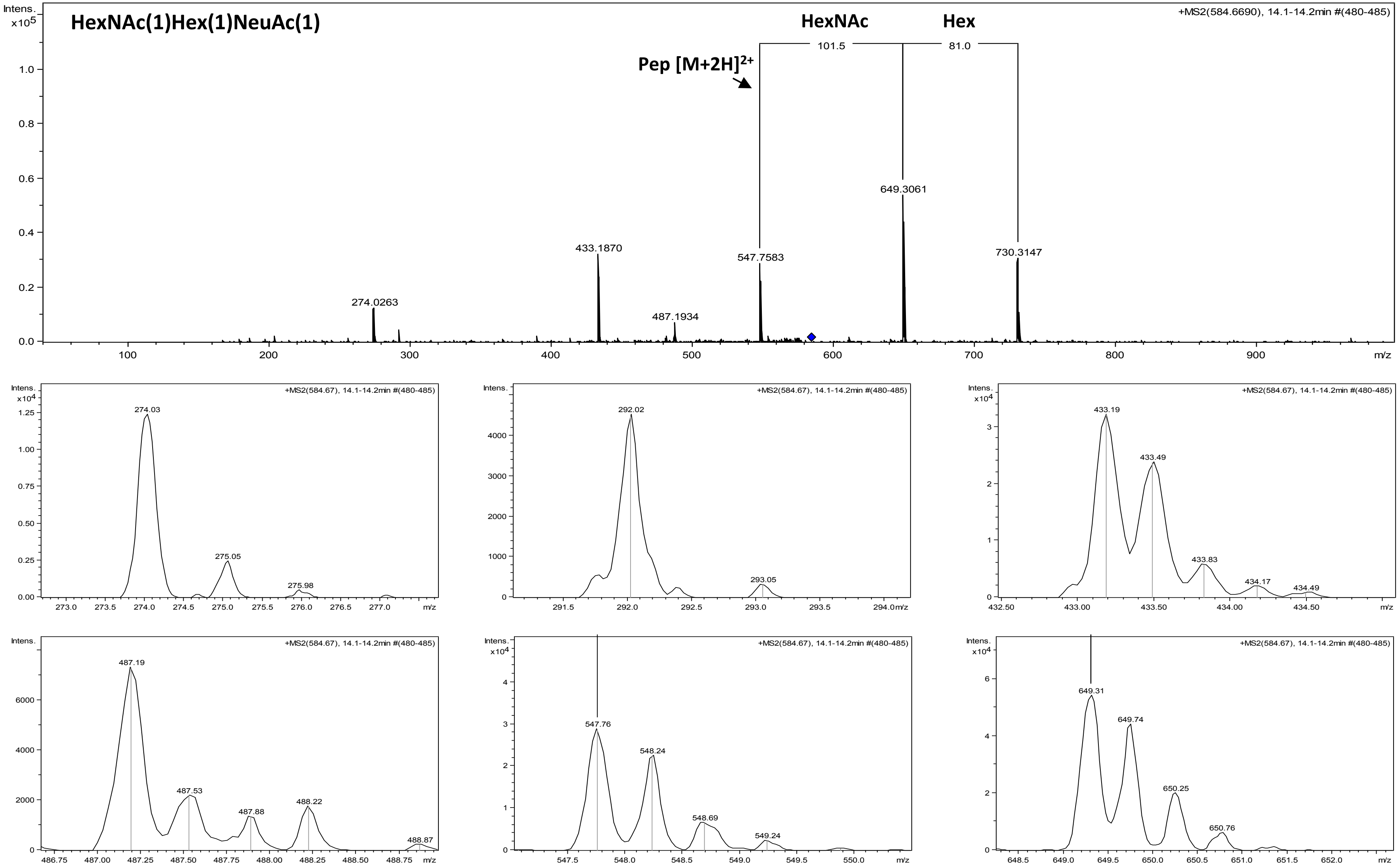

Fraction 15

584.24+++ → Pep [M+2H]++ 547.76++ [13.8-14.2 min] CID-MS2

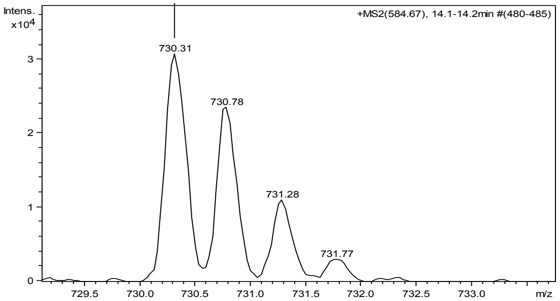

Fraction 15

584.24+++ → Pep [M+2H]++ 547.76++ [13.8-14.2 min] CID-MS3

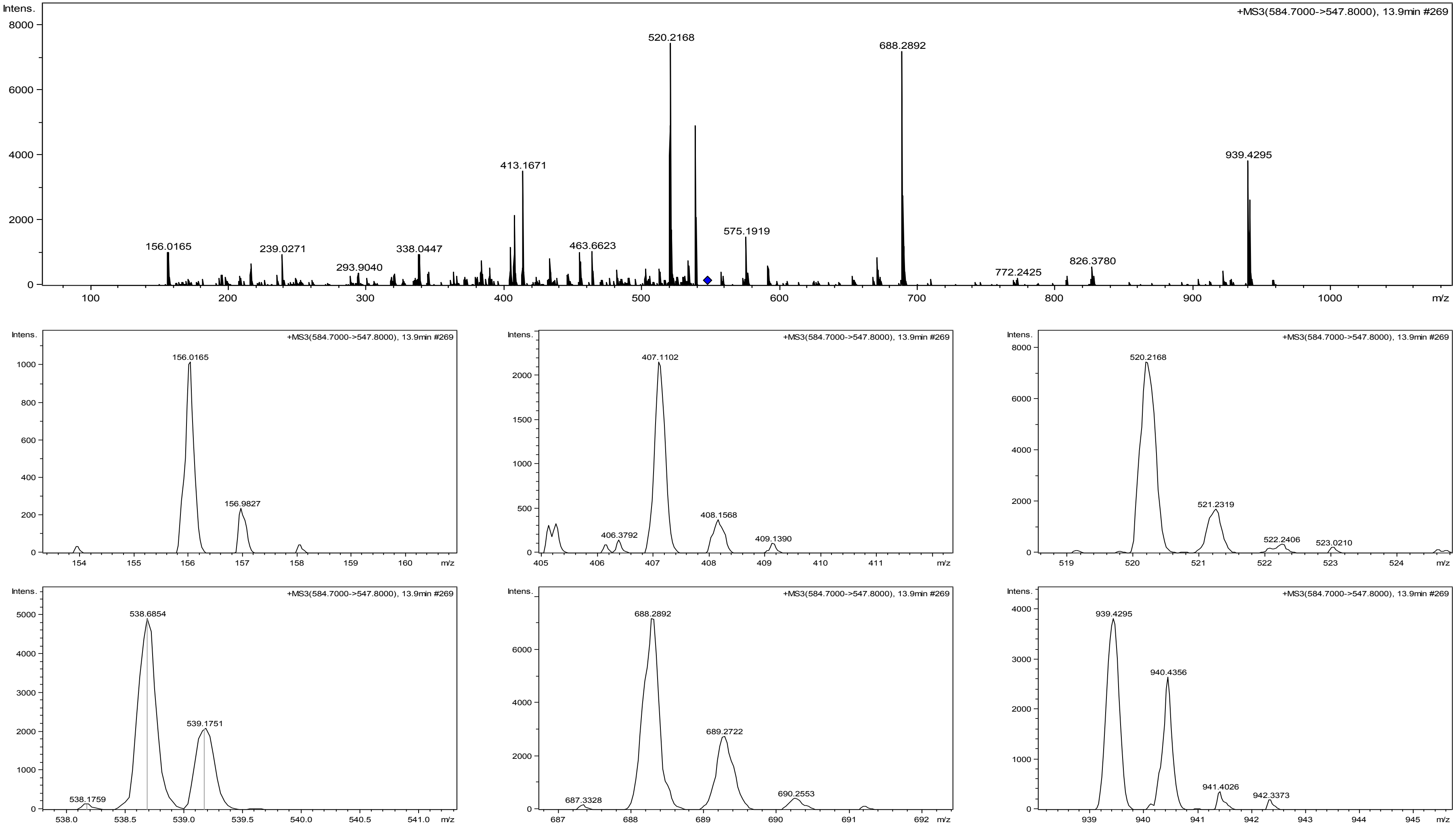

Fraction 15

584.24+++ → Pep [M+2H]++ 547.76++ [13.8-14.2 min]   CID-MS3   MASCOT Search

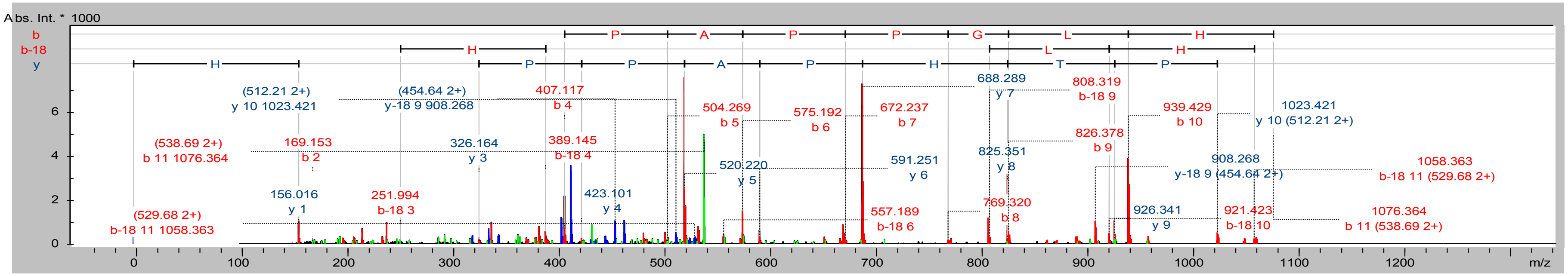

	A	P	T	H	P	A	P	P	G	L	H	Ala	Pro	Thr	His	Pro	Ala	Pro	Pro	Gly	Leu	His
Ion	1	2	3	4	5	6	7	8	9	10	11	1	2	3	4	5	6	7	8	9	10	11
b	A	P	T	H	P	A	P	P	G	L	H	72.044	169.097	270.145	407.204	504.257	575.294	672.346	769.399	826.421	939.505	1076.564
b-17	A	P	T	H	P	A	P	P	G	L	H	-	-	-	-	-	-	-	-	-	-	-
b-18	A	P	T	H	P	A	P	P	G	L	H	-	-	252.134	389.193	486.246	557.283	654.336	751.389	808.410	921.494	1058.553
y	A	P	T	H	P	A	P	P	G	L	H	156.077	269.161	326.182	423.235	520.288	591.325	688.378	825.437	926.484	1023.537	1094.574
y-17	A	P	T	H	P	A	P	P	G	L	H	-	-	-	-	-	-	-	-	-	-	-
y-18	A	P	T	H	P	A	P	P	G	L	H	-	-	-	-	-	-	-	-	908.474	1005.526	1076.564
	11	10	9	8	7	6	5	4	3	2	1	His	Leu	Gly	Pro	Pro	Ala	Pro	His	Thr	Pro	Ala

unknown O-glycosylation site

Selenoprotein P precursor

$_{234}$AP**T**HPAPPGLH$_{244}$

Fraction 15

584.24+++ → Pep [M+2H]++ 547.76++ [13.8-14.2 min]

CID-MS3 MASCOT Search

prot_hit_nur	prot_acc	prot_desc	prot_score	prot_mass	prot_matche	pep_query	pep_rank	pep_isbold	pep_exp_mz	pep_exp_mr	pep_exp_z	pep_calc_mr	pep_delta	pep_miss	pep_score	pep_expect	pep_res_bef	pep_seq
1	SEPP1_HUM/	Selenoprote	51	44216	1	1	1	1	547,7583	1093,502	2	1093,5669	-0,0648	0	55,93	0,04	N	APTHPAPPGLH
2	NRK_HUMAN	Nik-related p	19	179509	1	1	2	0	547,7583	1093,502	2	1093,4312	0,0708	0	25,91	40	S	STQSDFSANH
3	NOL6_HUMA	Nucleolar pr	13	128368	1	1	4	0	547,7583	1093,502	2	1093,5656	-0,0635	0	18,81	2,10E+02	R	SVLQFLATTD
4	EPB42_HUM/	Erythrocyte r	13	77780	1	1	6	0	547,7583	1093,502	2	1093,5292	-0,0271	0	18,28	2,30E+02	D	AQSWTISVTT
5	K0103_HUM/	Tetratricope	13	34982	1	1	6	0	547,7583	1093,502	2	1093,4797	0,0223	0	18,28	2,30E+02	E	LMMTNPHNH
6	KCMA1_HUM	Calcium-acti	12	139467	1	1	5	0	547,7583	1093,502	2	1092,5564	0,9457	0	18,68	2,10E+02	N	PGNHLKIQEG
7	CTR1_HUMA	High-affinity	12	68449	1	1	9	0	547,7583	1093,502	2	1093,5478	-0,0457	0	17,66	2,70E+02	Y	AMAEDGLLFK
8	CXXC6_HUM	CXXC-type zi	12	237956	1	1	3	0	547,7583	1093,502	2	1093,504	-0,0019	0	19,63	1,70E+02	G	ADPVHGEALGE
9	ZN281_HUM/	Zinc finger p	11	97596	1	1	8	0	547,7583	1093,502	2	1093,5742	-0,0722	0	18,01	2,50E+02	S	MKFIQKYH
10	DMN_HUMA	Desmuslin -	10	173005	1	1	10	0	547,7583	1093,502	2	1093,5655	-0,0635	0	17,01	3,10E+02	K	PLDVPAPSLEG

Biotools-Score: 517

MASCOT-Score: 43

unknown O-glycosylation site

Selenoprotein P precursor

$_{234}$APTHPAPPGLH$_{244}$

Fraction 15

584.24+++ → Pep [M+2H]++ 547.76++ [13.8-14.2 min] ETD

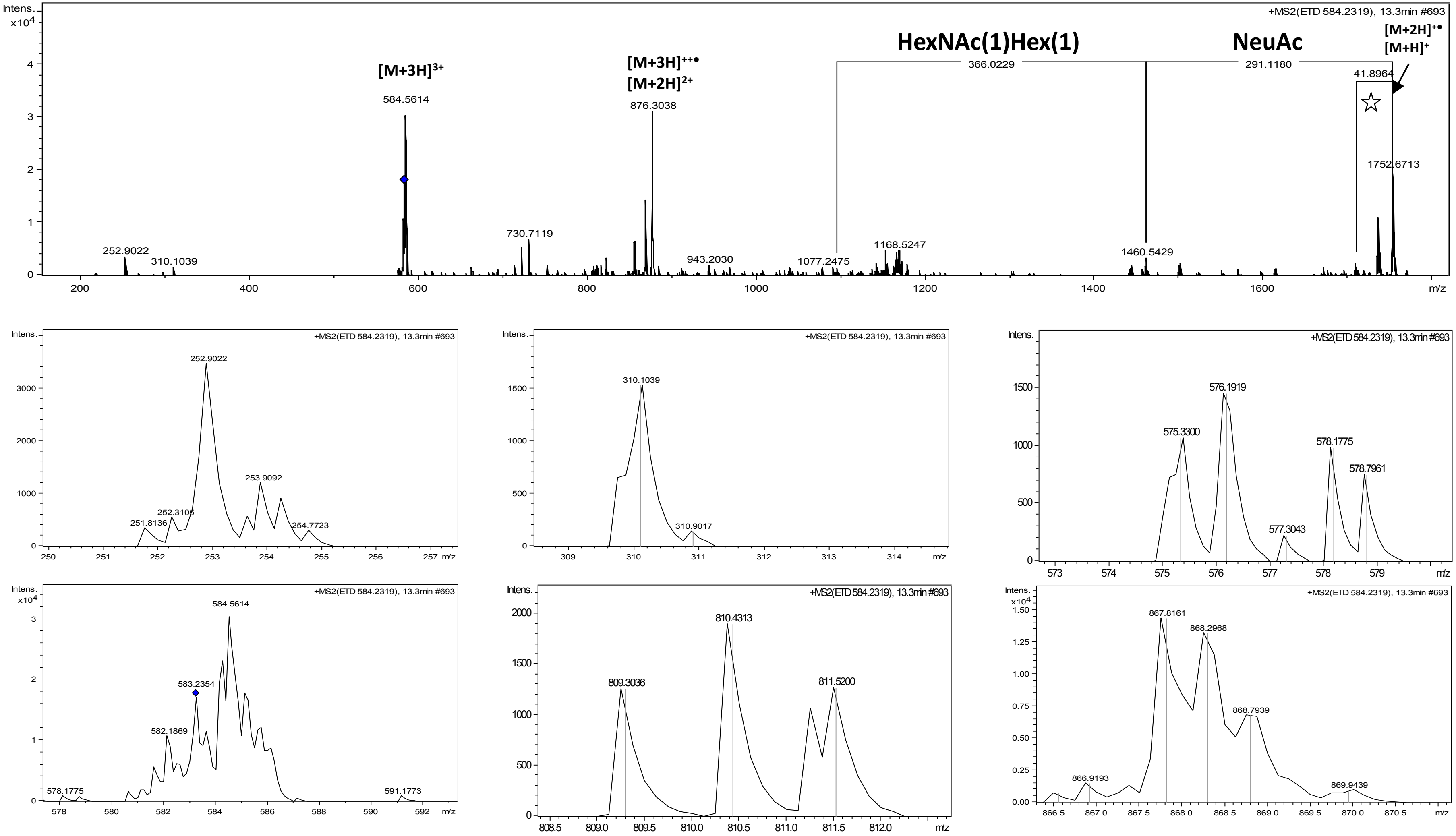

Fraction 15

584.24+++ → Pep [M+2H]++ 547.76++ [13.8-14.2 min] ETD

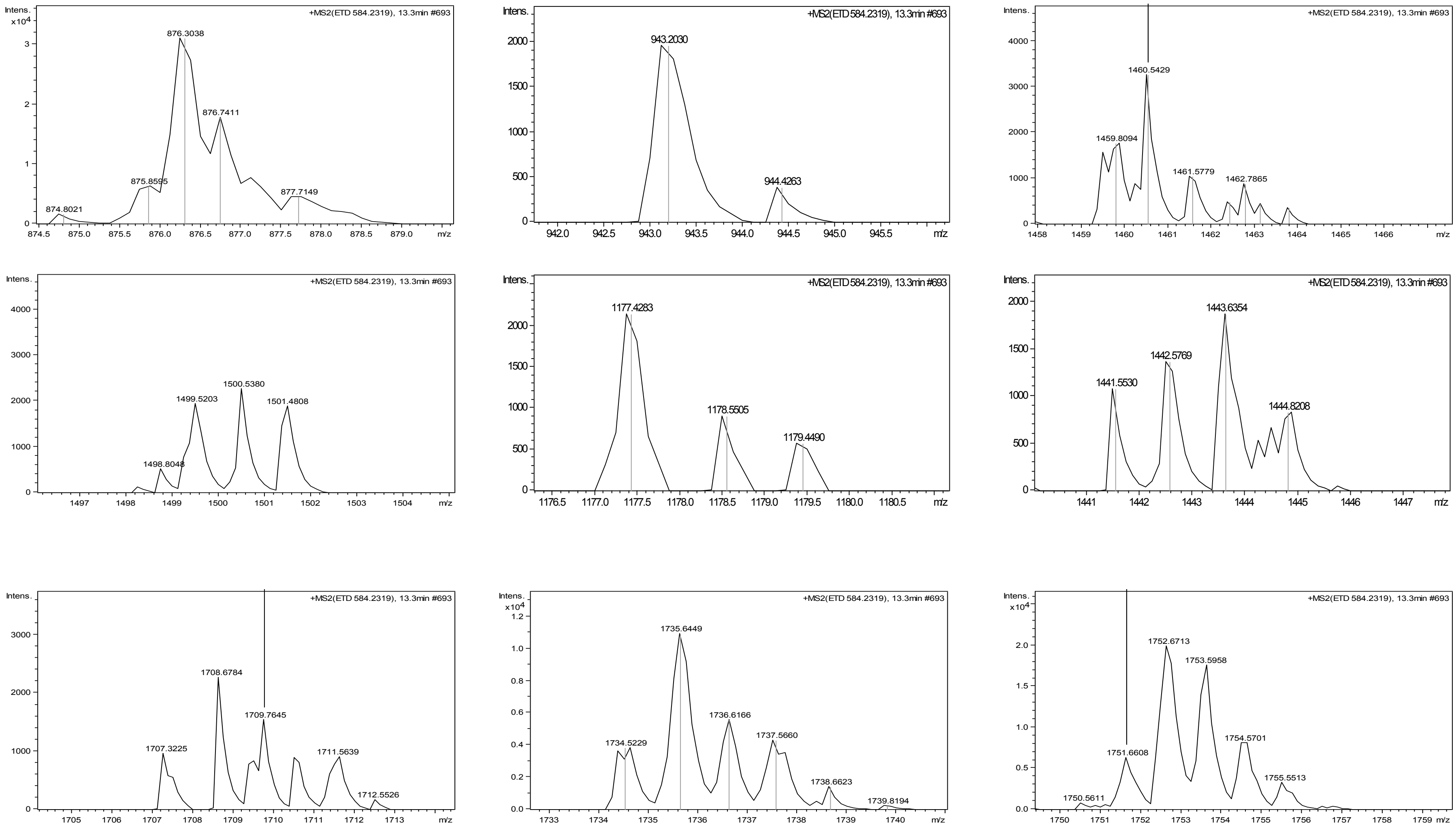

Fraction 15

584.24+++ → Pep [M+2H]++ 547.76++ [13.8-14.2 min] ETD

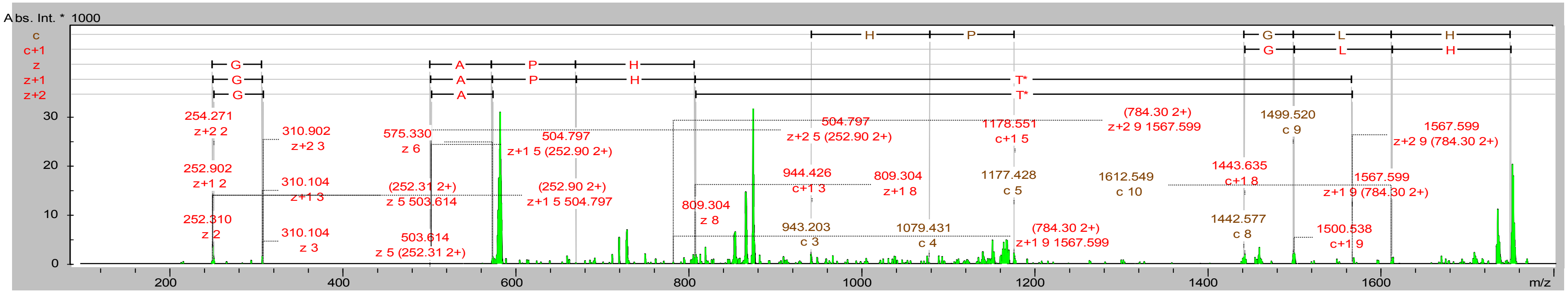

	A	P	T	H	P	A	P	P	G	L	H	Ala	Pro	Thr	His	Pro	Ala	Pro	Pro	Gly	Leu	His
Ion	1	2	3	4	5	6	7	8	9	10	11	1	2	3	4	5	6	7	8	9	10	11
c	A	P	T*	H	P	A	P	P	G	L	H	89.071	186.124	943.399	1080.458	1177.511	1248.548	1345.601	1442.653	1499.675	1612.759	1749.818
c+1	A	P	T*	H	P	A	P	P	G	L	H	90.079	187.132	944.407	1081.466	1178.518	1249.556	1346.608	1443.661	1500.683	1613.767	1750.826
z	A	P	T*	H	P	A	P	P	G	L	H	139.050	252.134	309.156	406.208	503.261	574.298	671.351	808.410	1565.685	1662.738	1733.775
z+1	A	P	T*	H	P	A	P	P	G	L	H	140.058	253.142	310.164	407.216	504.269	575.306	672.359	809.418	1566.693	1663.746	1734.783
z+2	A	P	T*	H	P	A	P	P	G	L	H	141.066	254.150	311.171	408.224	505.277	576.314	673.367	810.426	1567.701	1664.754	1735.791
	11	10	9	8	7	6	5	4	3	2	1	His	Leu	Gly	Pro	Pro	Ala	Pro	His	Thr	Pro	Ala

Biotools-Score: 57

unknown O-glycosylation site

Selenoprotein P precursor

$_{234}$AP**T**HPAPPGLH$_{244}$

Fraction 15

683.74++ → Pep+HexNAc(1)NeuAc(1) [M+H]+ 913.39+ [14.7-15.0 min]

CID-MS Precursor

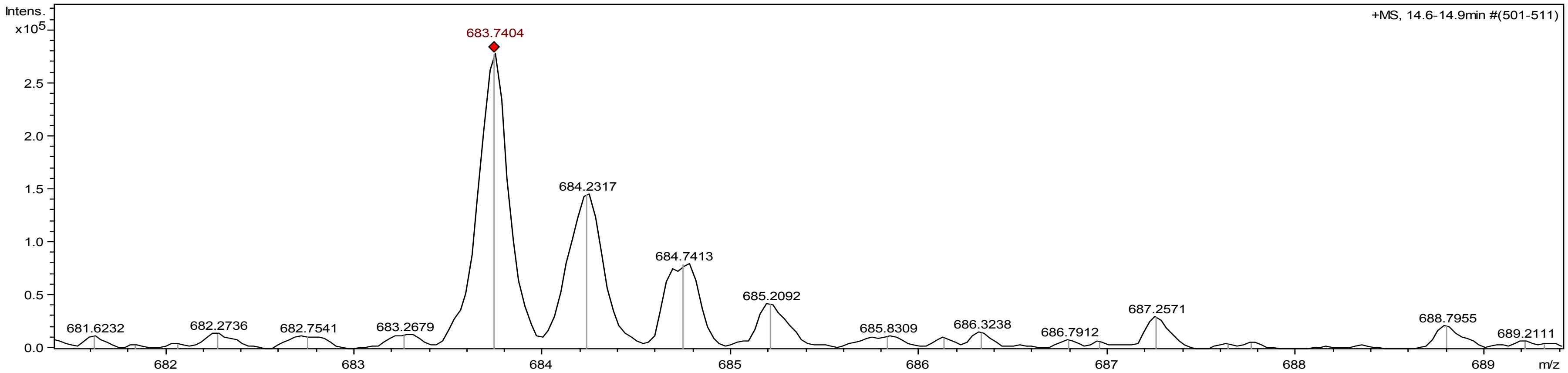

No ETD spectrum available

Fraction 15

683.74++ → Pep+HexNAc(1)NeuAc(1) [M+H]+ 913.39+ [14.7-15.0 min]

CID-MS2

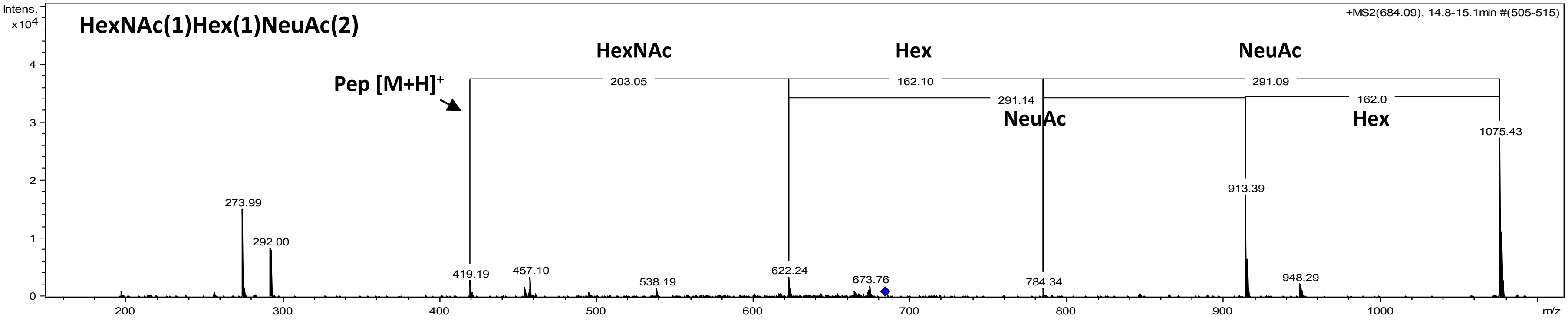

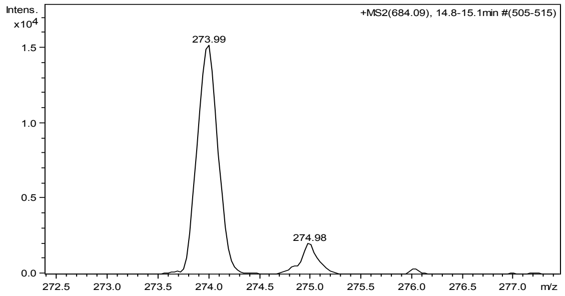

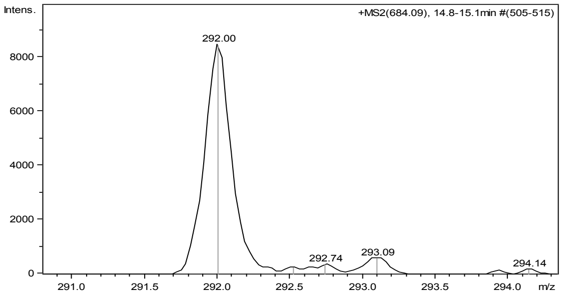

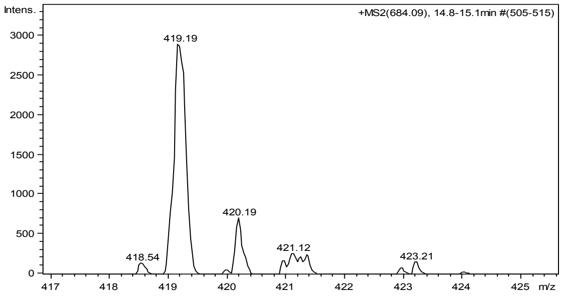

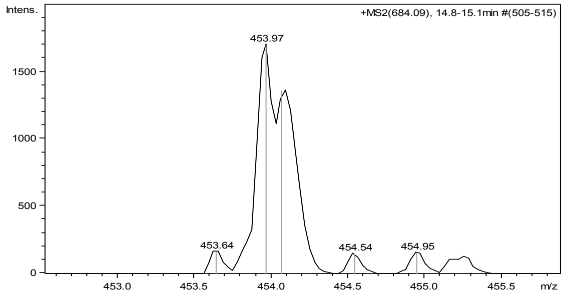

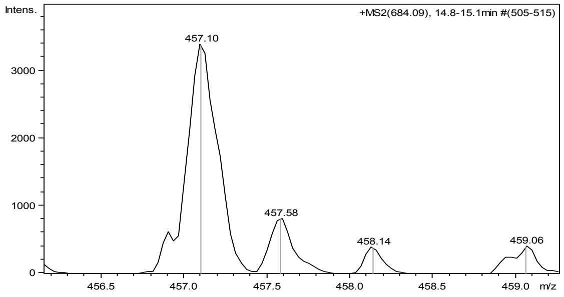

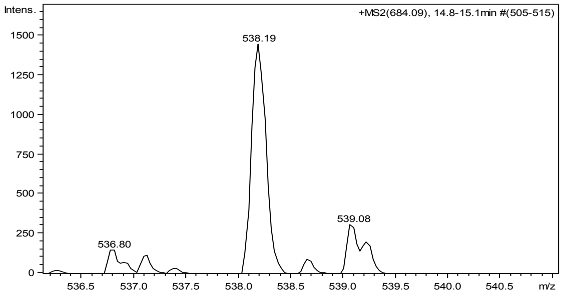

Fraction 15

683.74++ → Pep+HexNAc(1)NeuAc(1) [M+H]+ 913.39+ [14.7-15.0 min] CID-MS3 MASCOT Search

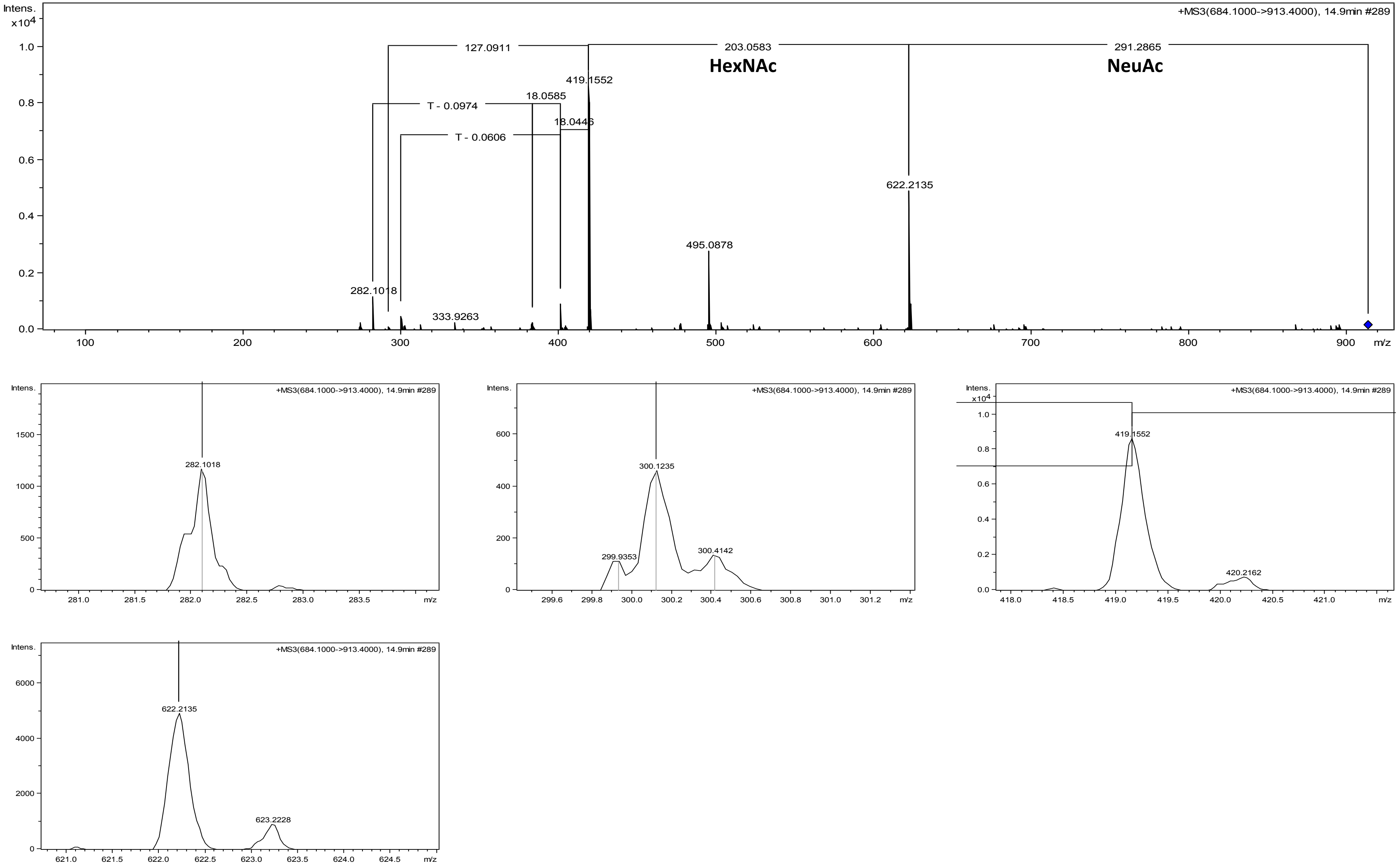

Fraction 15

683.74++ → Pep+HexNAc(1)NeuAc(1) [M+H]+ 913.39+ [14.7-15.0 min]   CID-MS3   MASCOT Search

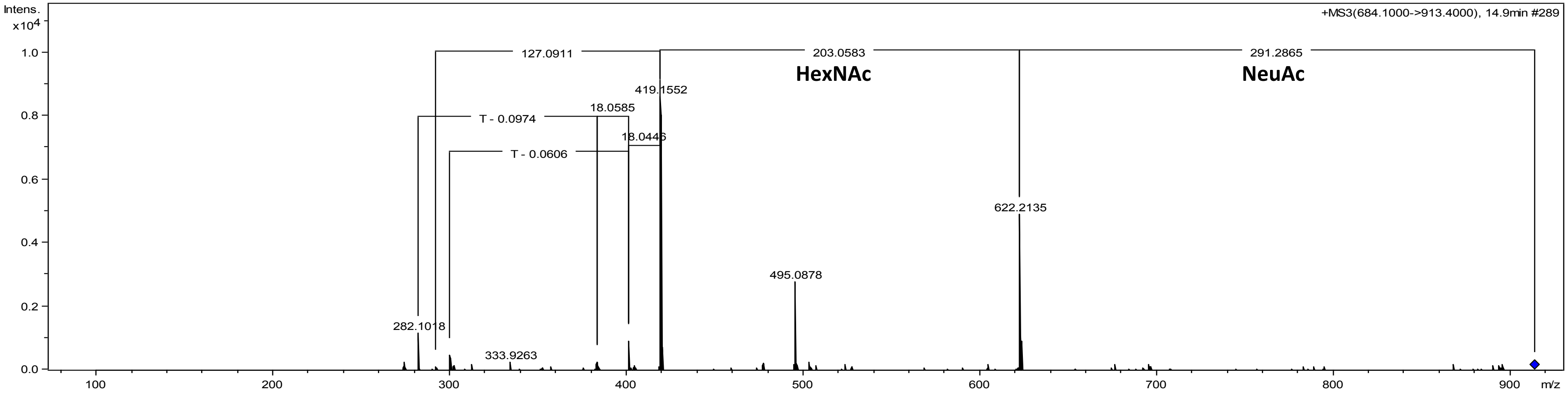

Too little information within the fragment spectrum

Too many possible sequences

Fraction 15

734.25++ → Pep+HexNAc(1)NeuAc(1) [M+H]+ 1014.48+ [16.0-16.2 min]

CID-MS Precursor

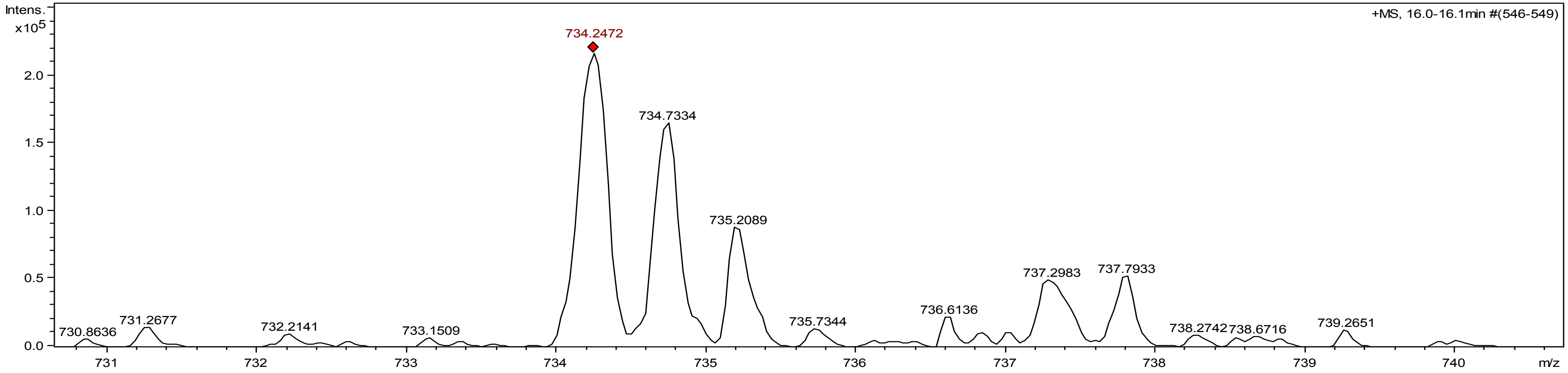

No unambiguous result

Fraction 15

734.25++ → Pep+HexNAc(1)NeuAc(1) [M+H]+ 1014.48+ [16.0-16.2 min]

CID-MS2

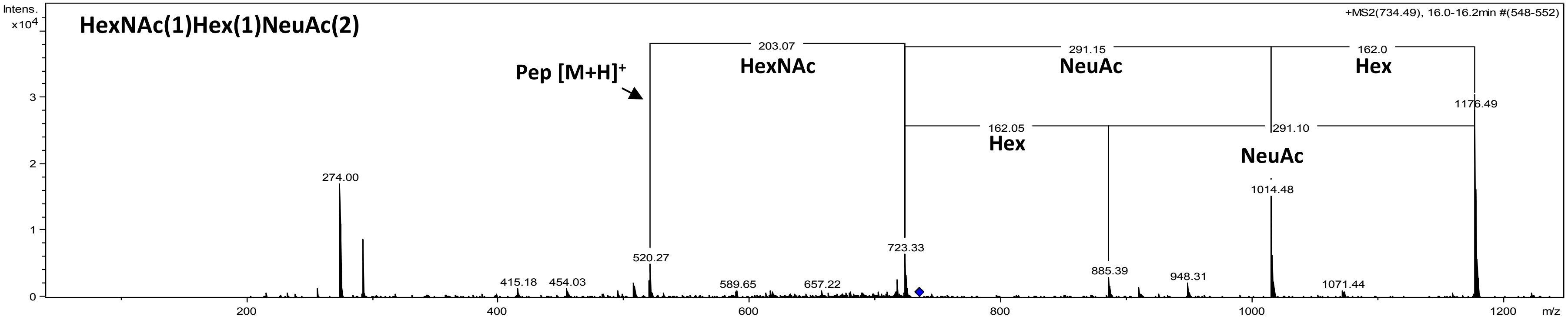

Fraction 15

734.25++ → Pep+HexNAc(1)NeuAc(1) [M+H]+ 1014.48+ [16.0-16.2 min] CID-MS3 MASCOT Search

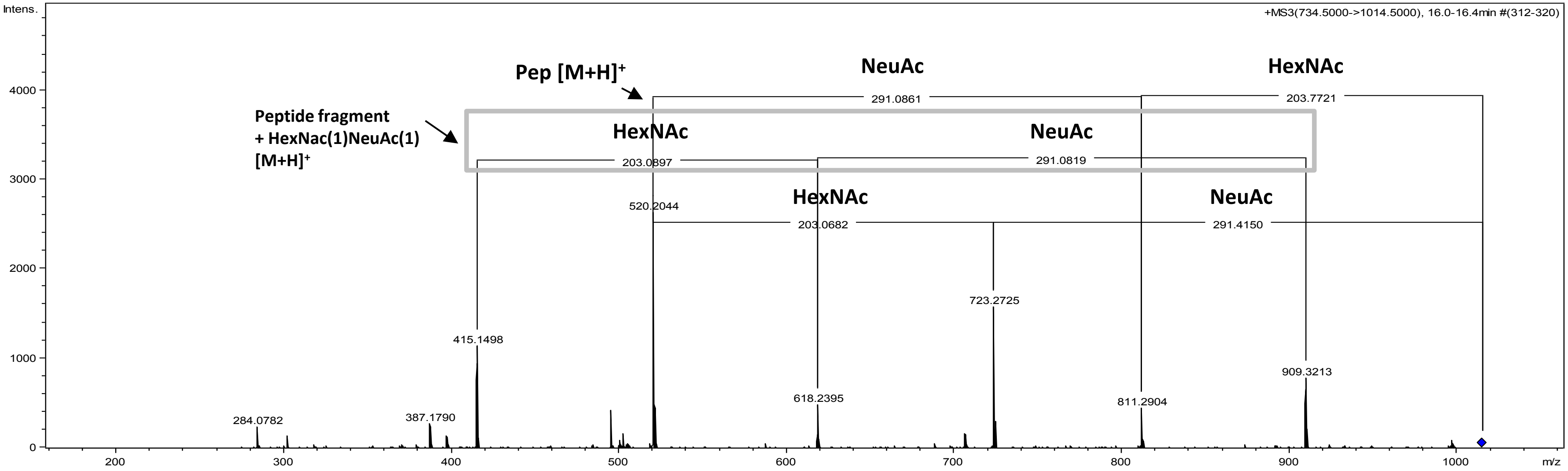

Fraction 15

734.25++ → Pep+HexNAc(1)NeuAc(1) [M+H]+ 1014.48+ [16.0-16.2 min] CID-MS3 MASCOT Search

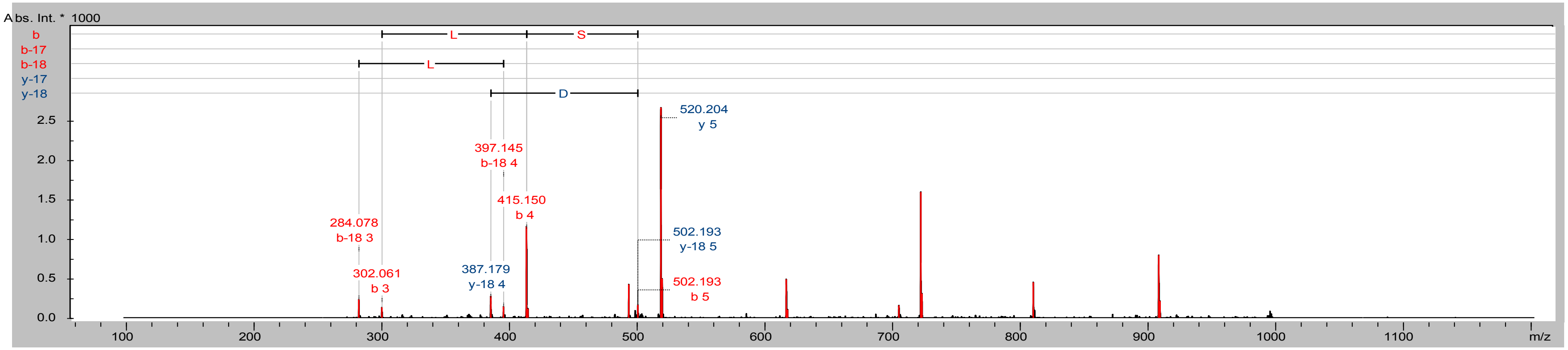

	D	S	V	L	S	Asp	Ser	Val	Leu	Ser
Ion	1	2	3	4	5	1	2	3	4	5
b	D	S	V	L	S	116.034	203.066	302.135	415.219	502.251
b-17	D	S	V	L	S	-	-	-	-	-
b-18	D	S	V	L	S	98.024	185.056	284.124	397.208	484.240
y	D	S	V	L	S	106.050	219.134	318.202	405.234	520.261
y-17	D	S	V	L	S	-	-	-	-	-
y-18	D	S	V	L	S	88.039	201.123	300.192	387.224	502.251
	5	4	3	2	1	Ser	Leu	Val	Ser	Asp

Too little information within the fragment spectrum
Too many possible sequences

Fraction 15

623.27+++ → Pep [M+H]+ 1211.58+ [18.9-19.2 min] CID-MS Precursor

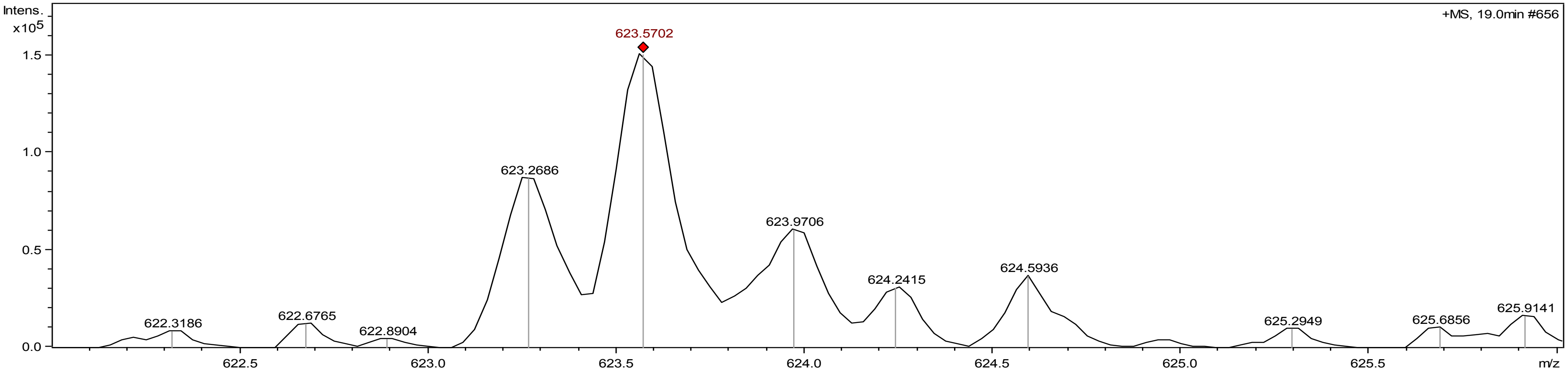

Fraction 15

623.27+++ → Pep [M+H]+ 1211.58+ [18.9-19.2 min] CID-MS2

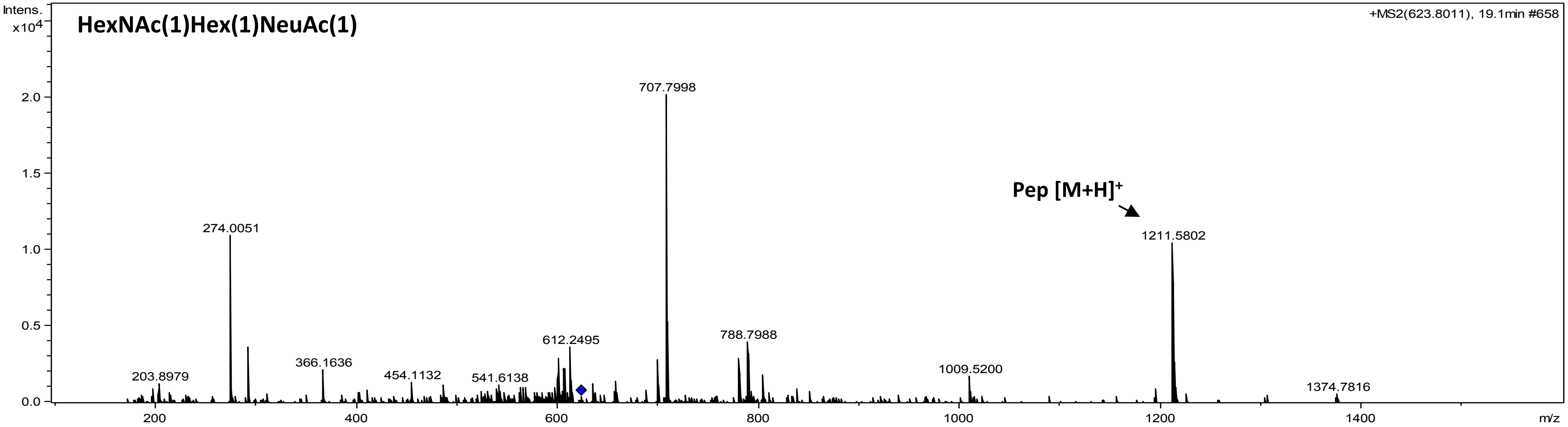

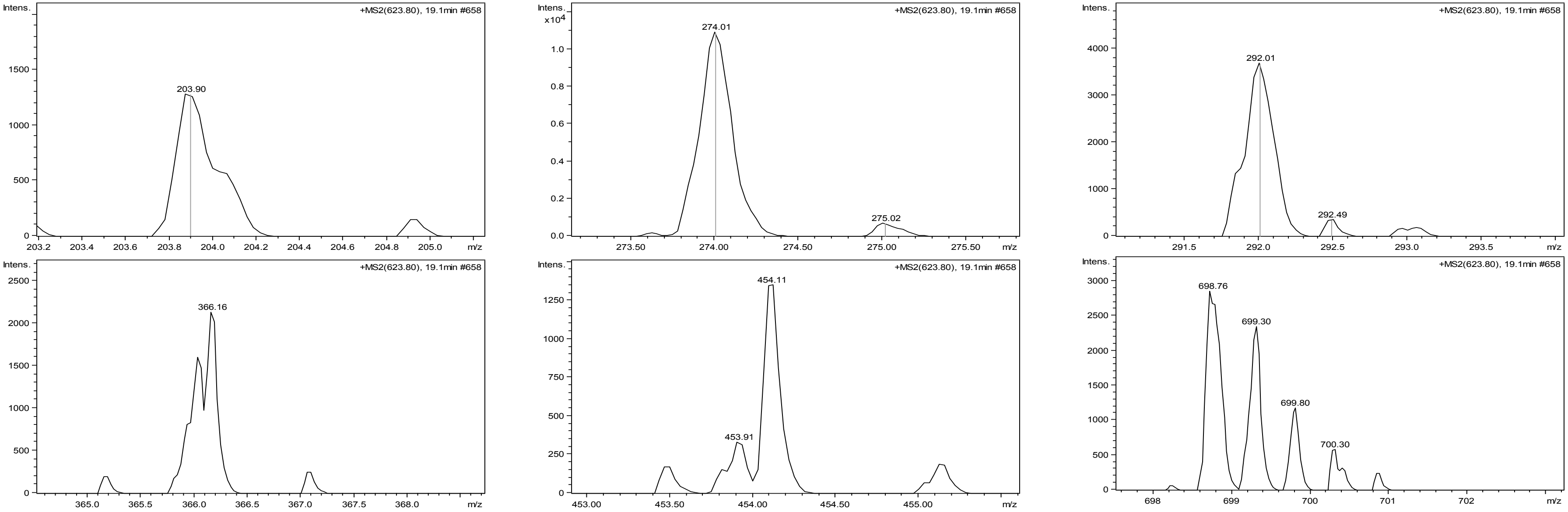

Fraction 15

623.27+++ → Pep [M+H]+ 1211.58+ [18.9-19.2 min] CID-MS2

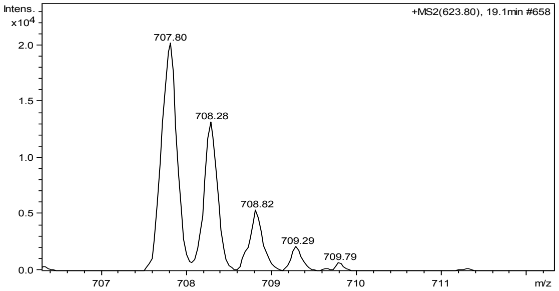

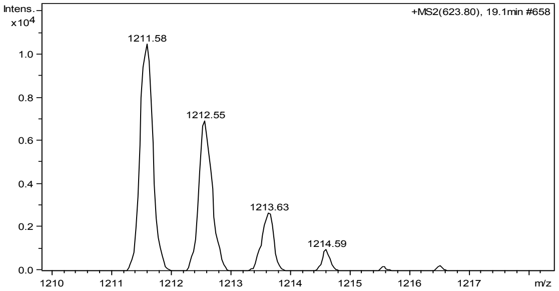

Fraction 15

623.27+++ → Pep [M+H]+ 1211.58+ [18.9-19.2 min]

CID-MS3

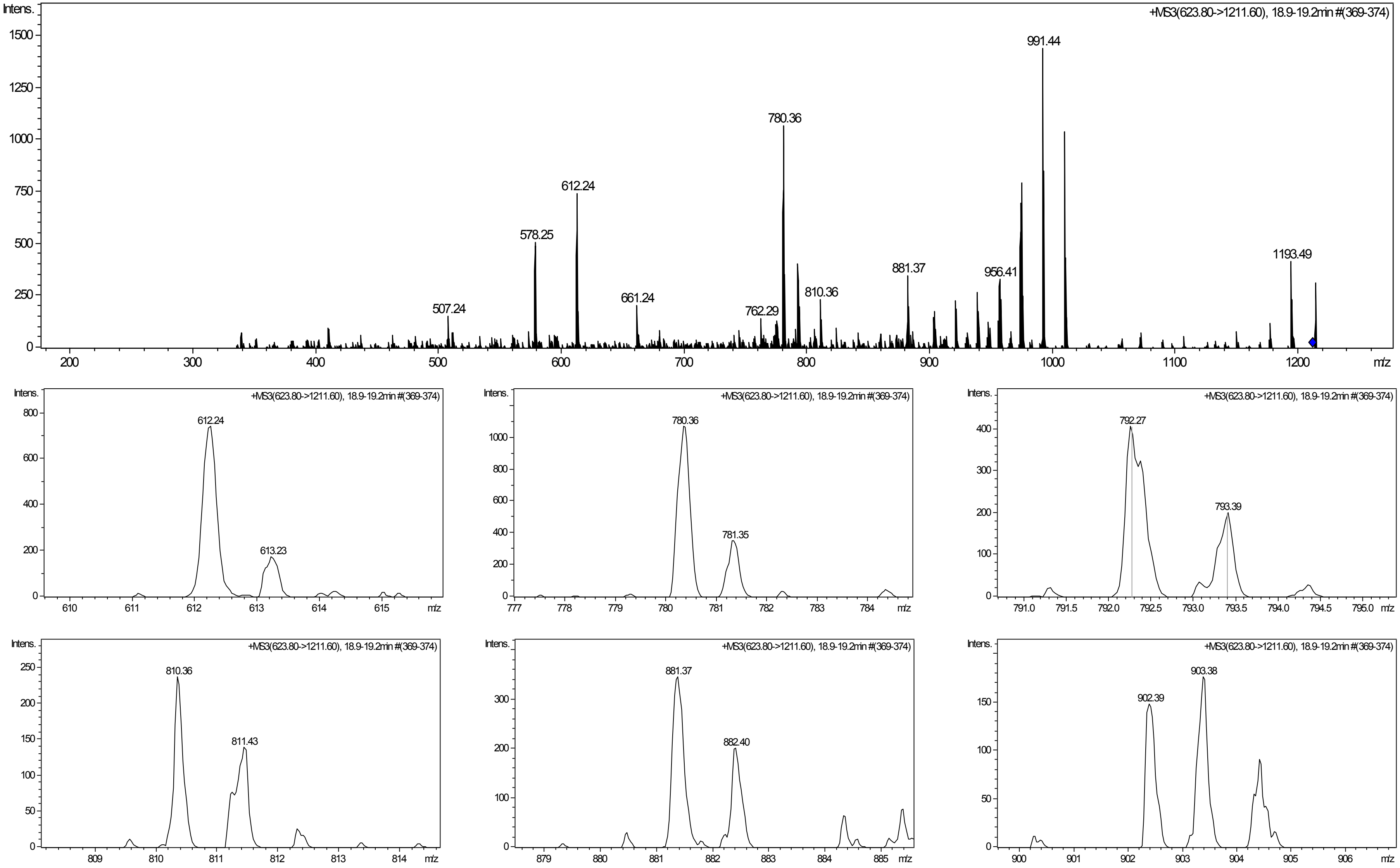

Fraction 15

623.27+++ → Pep [M+H]+ 1211.58+ [18.9-19.2 min]

CID-MS3

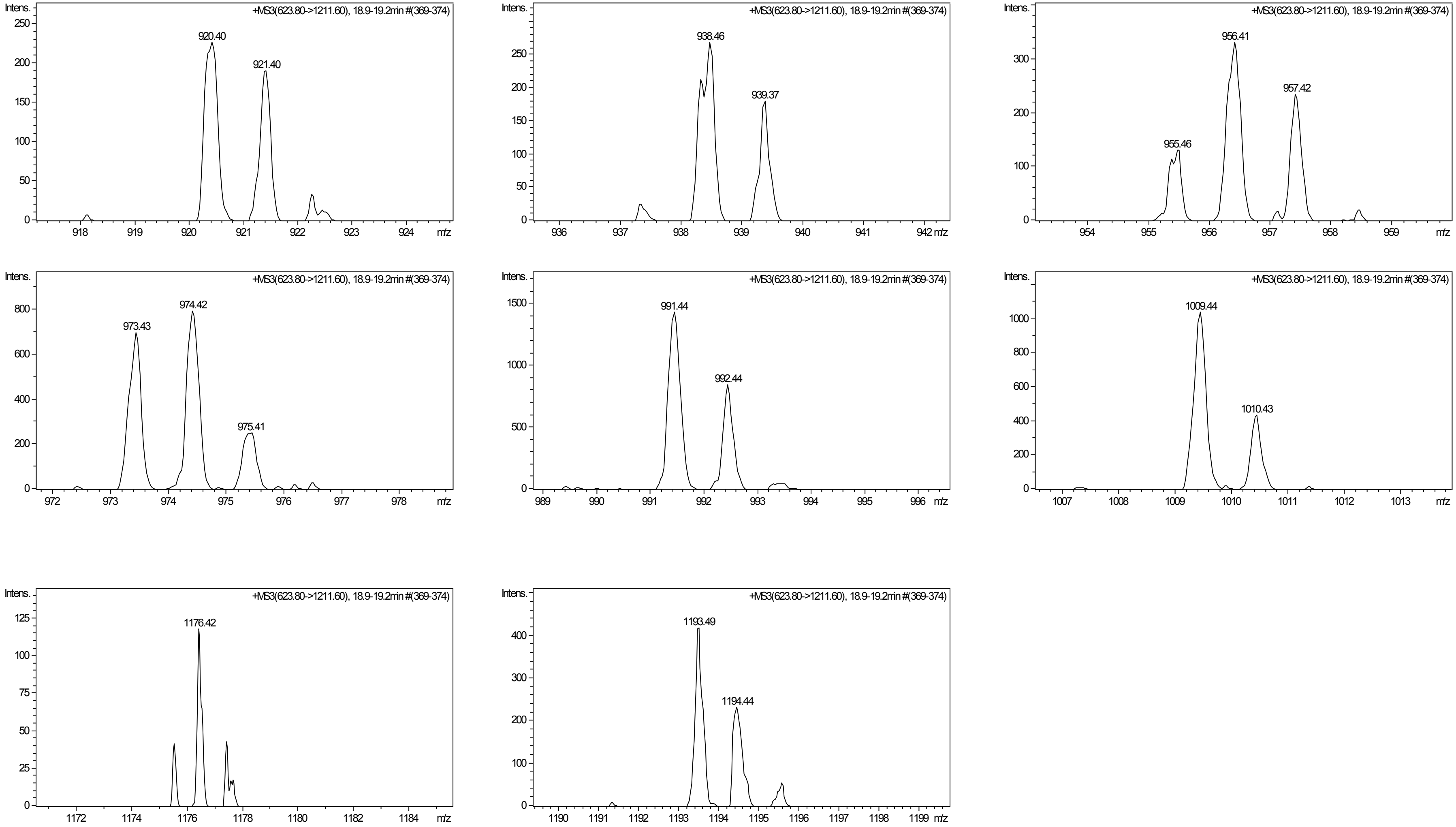

Fraction 15

623.27+++ → Pep [M+H]+ 1211.58+ [18.9-19.2 min]   CID-MS3   MASCOT Search

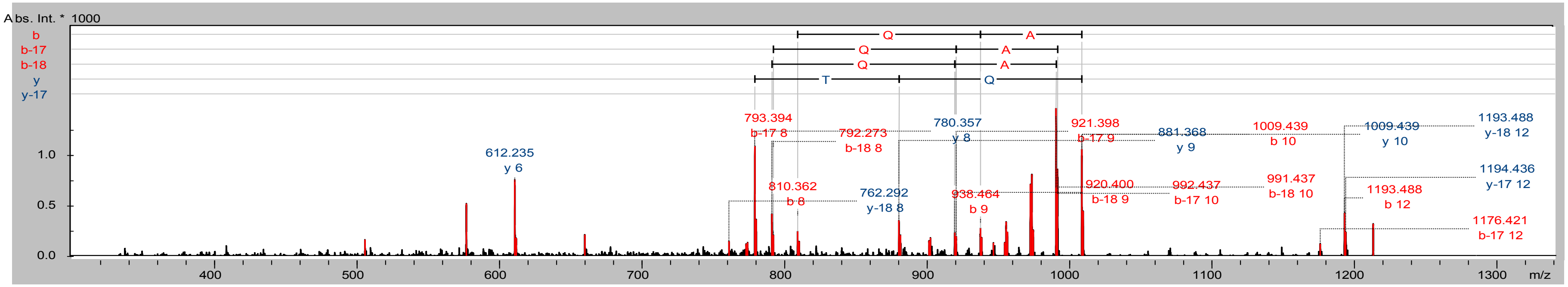

	T	T	Q	T	P	A	P	I	Q	A	P	S	Thr	Thr	Gln	Thr	Pro	Ala	Pro	Ile	Gln	Ala	Pro	Ser
Ion	1	2	3	4	5	6	7	8	9	10	11	12	1	2	3	4	5	6	7	8	9	10	11	12
b	T	T	Q	T	P	A	P	I	Q	A	P	S	102.055	203.103	331.161	432.209	529.262	600.299	697.352	810.436	938.494	1009.531	1106.584	1193.616
b-17	T	T	Q	T	P	A	P	I	Q	A	P	S	-	-	314.135	415.182	512.235	583.272	680.325	793.409	921.468	992.505	1089.557	1176.590
b-18	T	T	Q	T	P	A	P	I	Q	A	P	S	84.044	185.092	313.151	414.198	511.251	582.288	679.341	792.425	920.484	991.521	1088.573	1175.606
y	T	T	Q	T	P	A	P	I	Q	A	P	S	106.050	203.103	274.140	402.198	515.282	612.335	683.372	780.425	881.473	1009.531	1110.579	1211.627
y-17	T	T	Q	T	P	A	P	I	Q	A	P	S	-	-	-	385.172	498.256	595.309	666.346	763.398	864.446	992.505	1093.552	1194.600
y-18	T	T	Q	T	P	A	P	I	Q	A	P	S	88.039	185.092	256.129	384.188	497.272	594.325	665.362	762.414	863.462	991.521	1092.568	1193.616
	12	11	10	9	8	7	6	5	4	3	2	1	Ser	Pro	Ala	Gln	Ile	Pro	Ala	Pro	Thr	Gln	Thr	Thr

known O-glycosylation region

Inter-alpha-trypsin inhibitor heavy chain H4 precursor

$_{722}$**TT**Q**T**PAPIQAP**S**$_{733}$

Halim et al. 2012

Fraction 15

623.27+++ → Pep [M+H]+ 1211.58+ [18.9-19.2 min]

CID-MS3 MASCOT Search

prot_hit_nur	prot_acc	prot_desc	prot_score	prot_mass	prot_matche	pep_query	pep_rank	pep_isbold	pep_exp_mz	pep_exp_mr	pep_exp_z	pep_calc_mr	pep_delta	pep_miss	pep_score	pep_expect	pep_res_bef	pep_seq
1	CX055_HUMA	Putative unc	27	46164	1	1	3	1	1211,5802	1210,5729	1	1210,5023	0,0706	0	30,46	14	V	TTGATAMEEEAT
2	KIF6_HUMAN	Kinesin-like	25	93052	1	1	1	0	1211,5802	1210,5729	1	1210,5354	0,0376	0	31,21	12	G	DSQTDSDIIAF
3	ITIH4_HUMA	Inter-alpha-t	23	103489	1	1	2	0	1211,5802	1210,5729	1	1210,6194	-0,0464	0	30,66	14	M	TTQTPAPIQAPS
4	SRP54_HUMA	Signal recogr	23	55953	1	1	3	0	1211,5802	1210,5729	1	1209,6023	0,9706	0	30,46	14	Q	GSGKTTTCSKLA
5	NAL12_HUM	NACHT, LRR a	16	122490	1	1	5	0	1211,5802	1210,5729	1	1210,4958	0,0771	0	22,85	83	L	DSCGLTAKACE
6	NONO_HUM	Non-POU do	15	54311	1	1	6	0	1211,5802	1210,5729	1	1209,5877	0,9852	0	20,69	1,40E+02	Q	SNKTFNLEKQ
7	DEPD5_HUM	DEP domain-	13	179450	1	1	6	0	1211,5802	1210,5729	1	1209,5262	1,0467	0	20,69	1,40E+02	G	NSQTFGNSQNI
8	LYST_HUMAN	Lysosomal-tr	11	434169	1	1	6	0	1211,5802	1210,5729	1	1209,5271	1,0459	0	20,69	1,40E+02	D	SNKTFCMIGH

Biotools-Score: 23

MASCOT-Score: 31

known O-glycosylation region

Inter-alpha-trypsin inhibitor heavy chain H4 precursor

$_{722}$TTQTPAPIQAPS$_{733}$

Fraction 15

623.27+++ → Pep [M+H]+ 1211.58+ [18.9-19.2 min] ETD

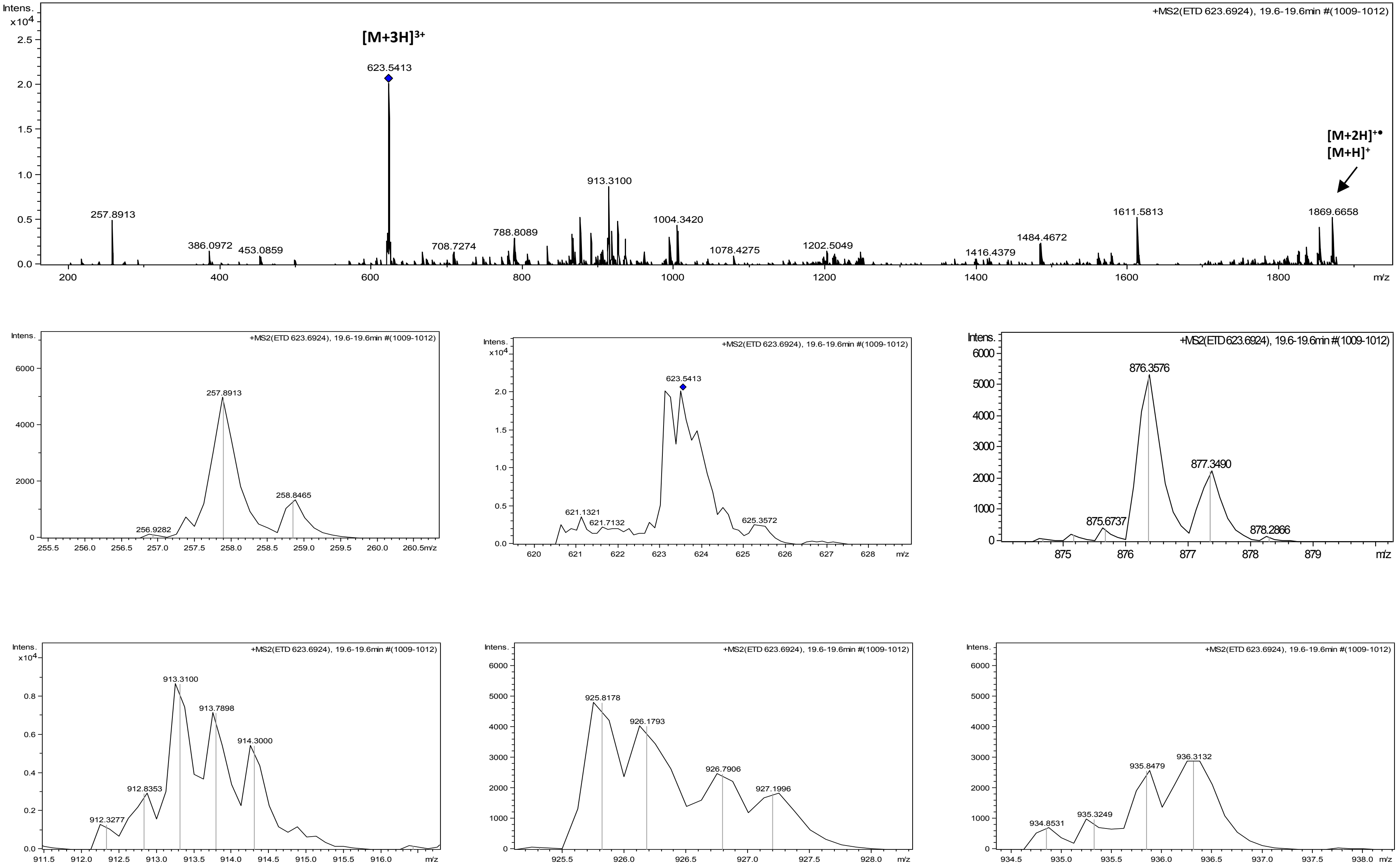

Fraction 15

623.27+++ → Pep [M+H]+ 1211.58+ [18.9-19.2 min] ETD

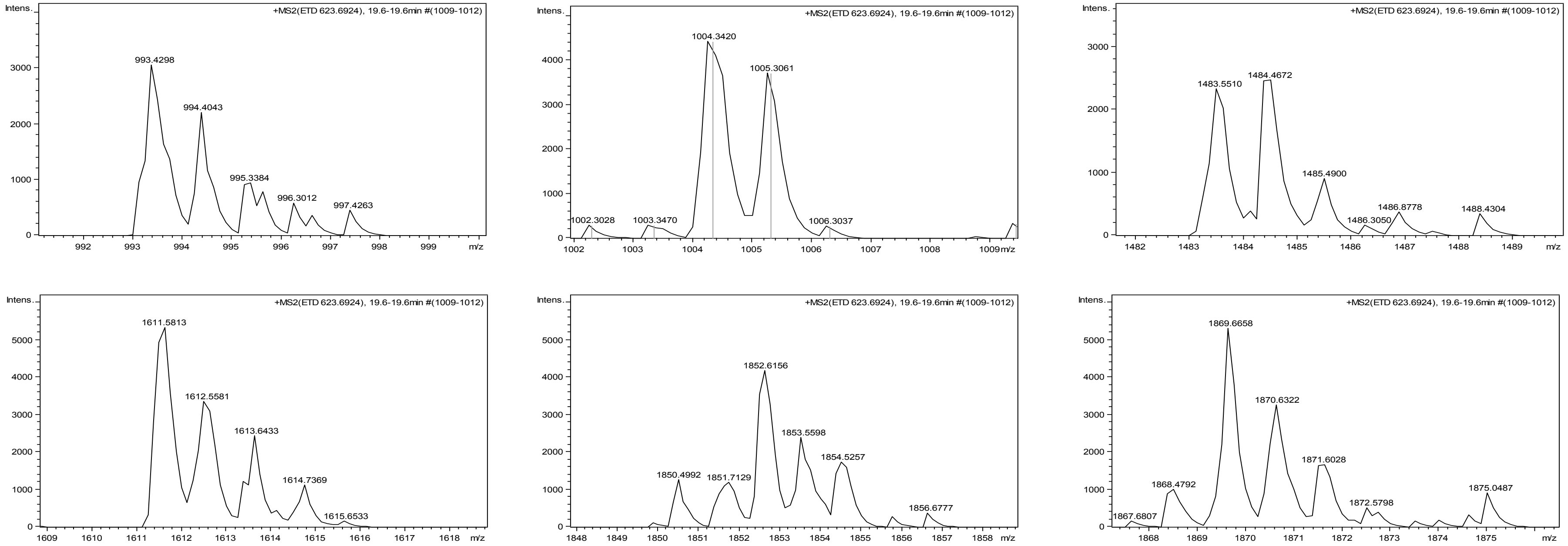

Fraction 15

623.27+++ → Pep [M+H]+ 1211.58+ [18.9-19.2 min] ETD

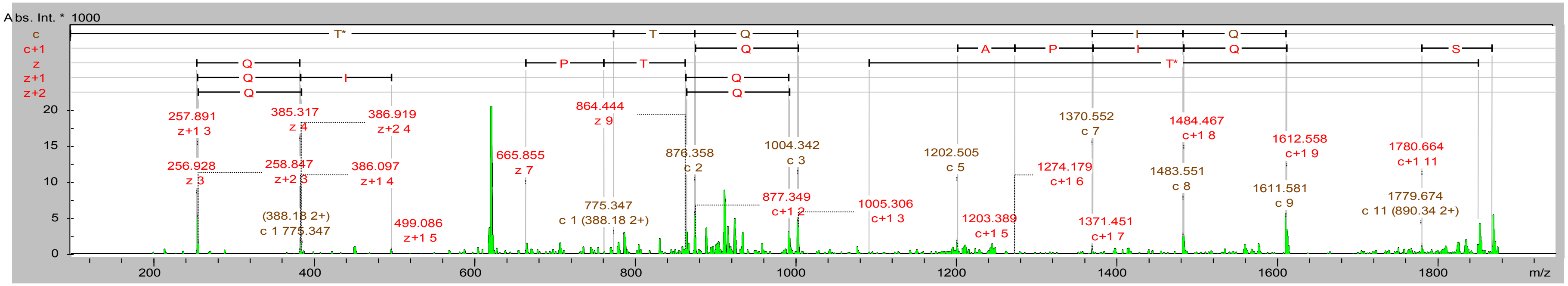

TTQTPAPIQAPS

	T	T	Q	T	P	A	P	I	Q	A	P	S	Thr	Thr	Gln	Thr	Pro	Ala	Pro	Ile	Gln	Ala	Pro	Ser
Ion	1	2	3	4	5	6	7	8	9	10	11	12	1	2	3	4	5	6	7	8	9	10	11	12
c	T*	T	Q	T	P	A	P	I	Q	A	P	S	775.309	876.357	1004.415	1105.463	1202.516	1273.553	1370.606	1483.690	1611.748	1682.785	1779.838	1866.870
c+1	T*	T	Q	T	P	A	P	I	Q	A	P	S	776.317	877.365	1005.423	1106.471	1203.524	1274.561	1371.614	1484.698	1612.756	1683.793	1780.846	1867.878
z	T*	T	Q	T	P	A	P	I	Q	A	P	S	89.023	186.076	257.113	385.172	498.256	595.309	666.346	763.398	864.446	992.505	1093.552	1850.828
z+1	T*	T	Q	T	P	A	P	I	Q	A	P	S	90.031	187.084	258.121	386.180	499.264	596.316	667.354	764.406	865.454	993.513	1094.560	1851.836
z+2	T*	T	Q	T	P	A	P	I	Q	A	P	S	91.039	188.092	259.129	387.187	500.271	597.324	668.361	765.414	866.462	994.520	1095.568	1852.843
	12	11	10	9	8	7	6	5	4	3	2	1	Ser	Pro	Ala	Gln	Ile	Pro	Ala	Pro	Thr	Gln	Thr	Thr

Biotools-Score: 152

Glycosylation site cannot be exactly determined → most likely T(722/723)

known O-glycosylation region Halim et al. 2012

Inter-alpha-trypsin inhibitor heavy chain H4 precursor

$_{722}$**TT**Q**T**PAPIQAP**S**$_{733}$

Fraction 15

623.27+++ → Pep [M+H]+ 1211.58+ [18.9-19.2 min] ETD

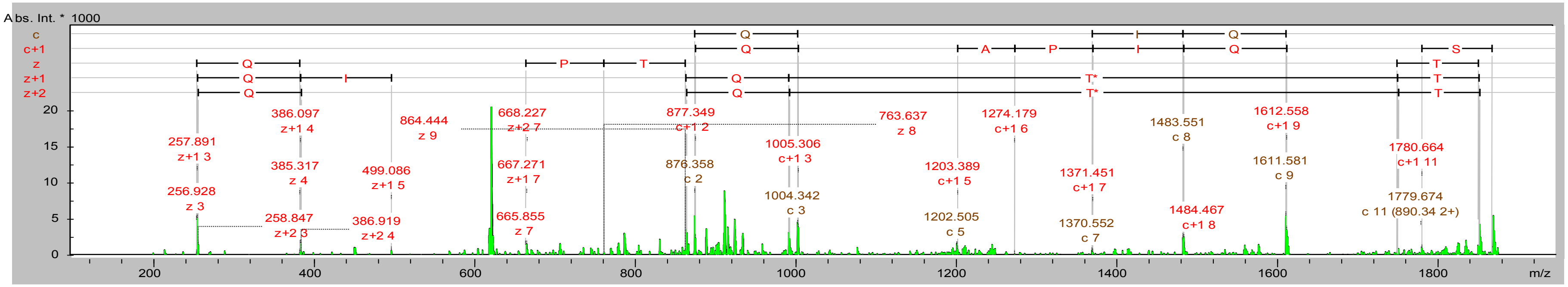

TTQTPAPIQAPS

	T	T	Q	T	P	A	P	I	Q	A	P	S	Thr	Thr	Gln	Thr	Pro	Ala	Pro	Ile	Gln	Ala	Pro	Ser
Ion	1	2	3	4	5	6	7	8	9	10	11	12	1	2	3	4	5	6	7	8	9	10	11	12
c	T	T*	Q	T	P	A	P	I	Q	A	P	S	119.082	876.357	1004.415	1105.463	1202.516	1273.553	1370.606	1483.690	1611.748	1682.785	1779.838	1866.870
c+1	T	T*	Q	T	P	A	P	I	Q	A	P	S	120.089	877.365	1005.423	1106.471	1203.524	1274.561	1371.614	1484.698	1612.756	1683.793	1780.846	1867.878
z	T	T*	Q	T	P	A	P	I	Q	A	P	S	89.023	186.076	257.113	385.172	498.256	595.309	666.346	763.398	864.446	992.505	1749.780	1850.828
z+1	T	T*	Q	T	P	A	P	I	Q	A	P	S	90.031	187.084	258.121	386.180	499.264	596.316	667.354	764.406	865.454	993.513	1750.788	1851.836
z+2	T	T*	Q	T	P	A	P	I	Q	A	P	S	91.039	188.092	259.129	387.187	500.271	597.324	668.361	765.414	866.462	994.520	1751.796	1852.843
	12	11	10	9	8	7	6	5	4	3	2	1	Ser	Pro	Ala	Gln	Ile	Pro	Ala	Pro	Thr	Gln	Thr	Thr

Biotools-Score: 34

Glycosylation site cannot be exactly determined

known O-glycosylation region Halim et al. 2012

Inter-alpha-trypsin inhibitor heavy chain H4 precursor

$_{722}$**TT**Q**T**PAPIQAP**S**$_{733}$

Fraction 15

623.27+++ → Pep [M+H]+ 1211.58+ [18.9-19.2 min] ETD

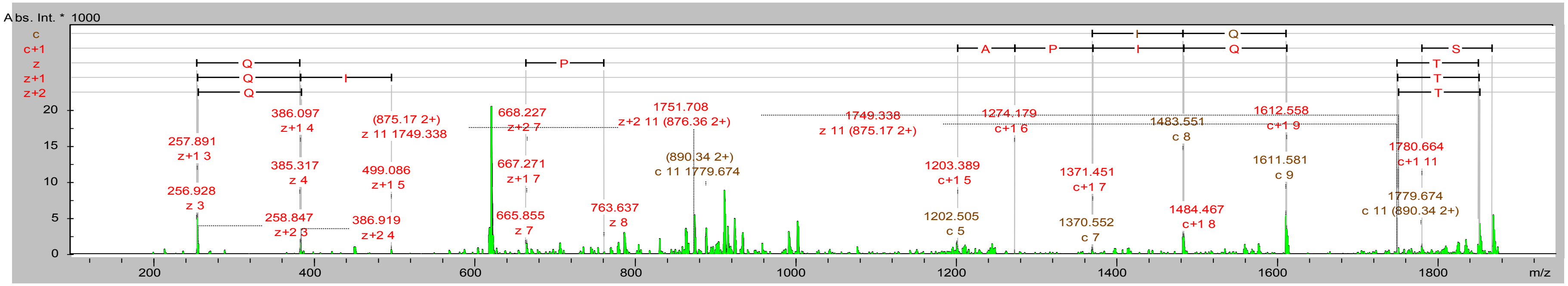

TTQTPAPIQAPS

	T	T	Q	T	P	A	P	I	Q	A	P	S	Thr	Thr	Gln	Thr	Pro	Ala	Pro	Ile	Gln	Ala	Pro	Ser
Ion	1	2	3	4	5	6	7	8	9	10	11	12	1	2	3	4	5	6	7	8	9	10	11	12
c	T	T	Q	T*	P	A	P	I	Q	A	P	S	119.082	220.129	348.188	1105.463	1202.516	1273.553	1370.606	1483.690	1611.748	1682.785	1779.838	1866.870
c+1	T	T	Q	T*	P	A	P	I	Q	A	P	S	120.089	221.137	349.196	1106.471	1203.524	1274.561	1371.614	1484.698	1612.756	1683.793	1780.846	1867.878
z	T	T	Q	T*	P	A	P	I	Q	A	P	S	89.023	186.076	257.113	385.172	498.256	595.309	666.346	763.398	1520.674	1648.732	1749.780	1850.828
z+1	T	T	Q	T*	P	A	P	I	Q	A	P	S	90.031	187.084	258.121	386.180	499.264	596.316	667.354	764.406	1521.682	1649.740	1750.788	1851.836
z+2	T	T	Q	T*	P	A	P	I	Q	A	P	S	91.039	188.092	259.129	387.187	500.271	597.324	668.361	765.414	1522.689	1650.748	1751.796	1852.843
	12	11	10	9	8	7	6	5	4	3	2	1	Ser	Pro	Ala	Gln	Ile	Pro	Ala	Pro	Thr	Gln	Thr	Thr

Biotools-Score: 19

Glycosylation site cannot be exactly determined

known O-glycosylation region Halim et al. 2012

Inter-alpha-trypsin inhibitor heavy chain H4 precursor

$_{722}$**TT**Q**T**PAPIQAP**S**$_{733}$

Fraction 15

623.27+++ → Pep [M+H]+ 1211.58+ [18.9-19.2 min] ETD

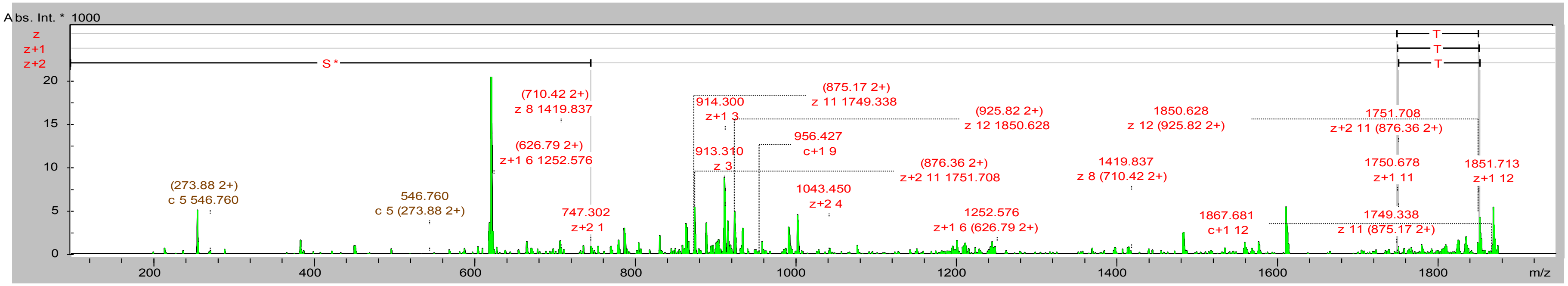

TTQTPAPIQAPS

	T	T	Q	T	P	A	P	I	Q	A	P	S	Thr	Thr	Gln	Thr	Pro	Ala	Pro	Ile	Gln	Ala	Pro	Ser
Ion	1	2	3	4	5	6	7	8	9	10	11	12	1	2	3	4	5	6	7	8	9	10	11	12
c	T	T	Q	T	P	A	P	I	Q	A	P	S*	119.082	220.129	348.188	449.235	546.288	617.325	714.378	827.462	955.521	1026.558	1123.611	1866.870
c+1	T	T	Q	T	P	A	P	I	Q	A	P	S*	120.089	221.137	349.196	450.243	547.296	618.333	715.386	828.470	956.529	1027.566	1124.618	1867.878
z	T	T	Q	T	P	A	P	I	Q	A	P	S*	745.251	842.304	913.341	1041.399	1154.483	1251.536	1322.573	1419.626	1520.674	1648.732	1749.780	1850.828
z+1	T	T	Q	T	P	A	P	I	Q	A	P	S*	746.259	843.312	914.349	1042.407	1155.491	1252.544	1323.581	1420.634	1521.682	1649.740	1750.788	1851.836
z+2	T	T	Q	T	P	A	P	I	Q	A	P	S*	747.267	844.319	915.356	1043.415	1156.499	1253.552	1324.589	1421.642	1522.689	1650.748	1751.796	1852.843
	12	11	10	9	8	7	6	5	4	3	2	1	Ser	Pro	Ala	Gln	Ile	Pro	Ala	Pro	Thr	Gln	Thr	Thr

Biotools-Score: 5

known O-glycosylation region Halim et al. 2012

Inter-alpha-trypsin inhibitor heavy chain H4 precursor

$_{722}$**TT**Q**T**PAPIQAP**S**$_{733}$

Fraction 15

765.77++ → Pep [M+H]+ 583.27+ [19.8-20.2 min] CID-MS Precursor

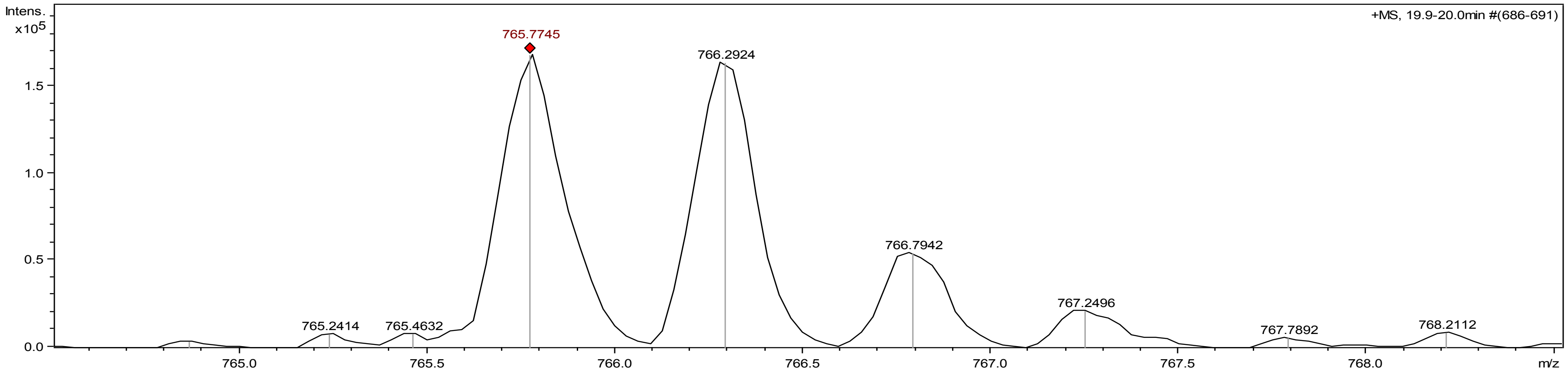

Fraction 15

765.77++ → Pep [M+H]+ 583.27+ [19.8-20.2 min] CID-MS2

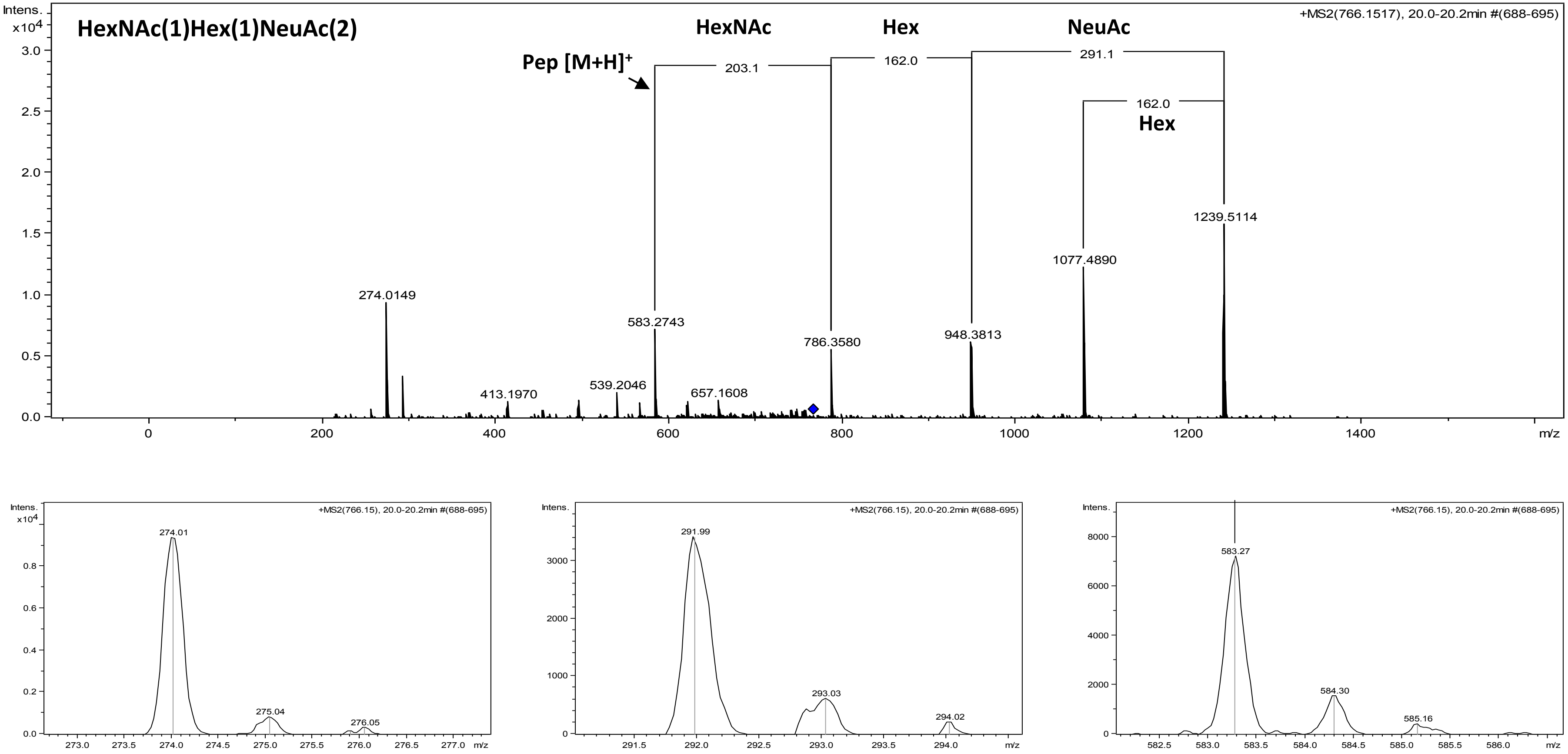

Fraction 15

765.77++ → Pep [M+H]+ 583.27+ [19.8-20.2 min]

CID-MS2

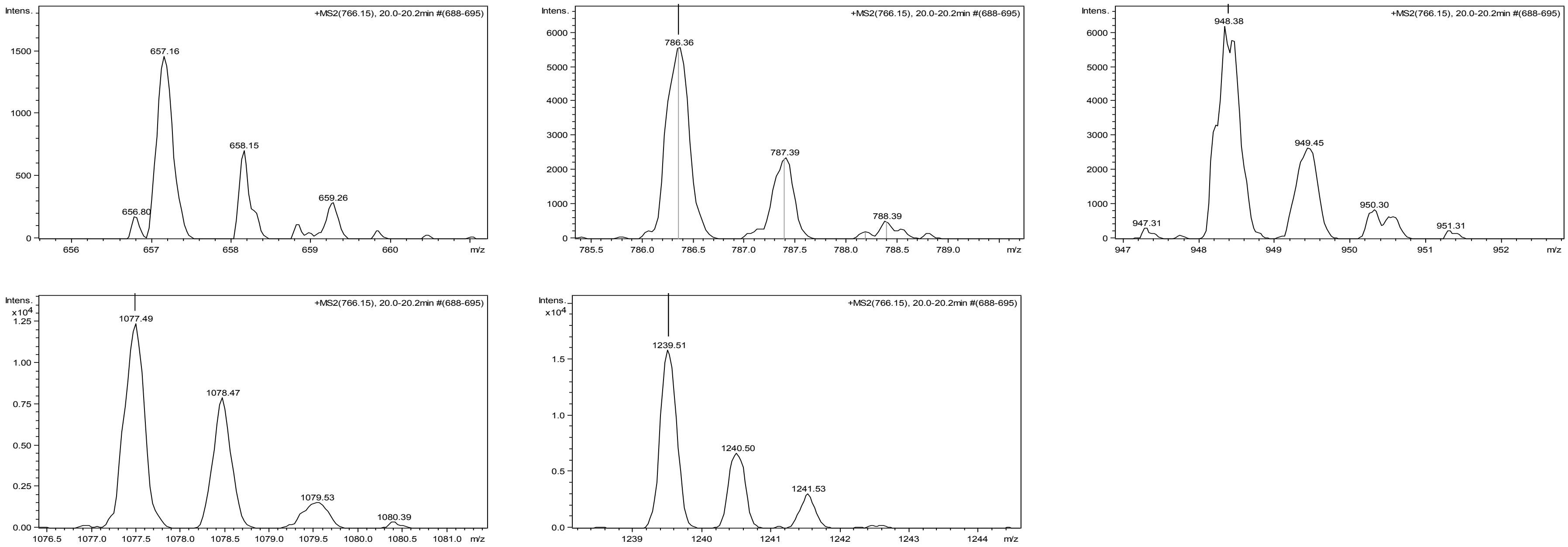

Fraction 15

765.77++ → Pep [M+H]+ 583.27+ [19.8-20.2 min] CID-MS3

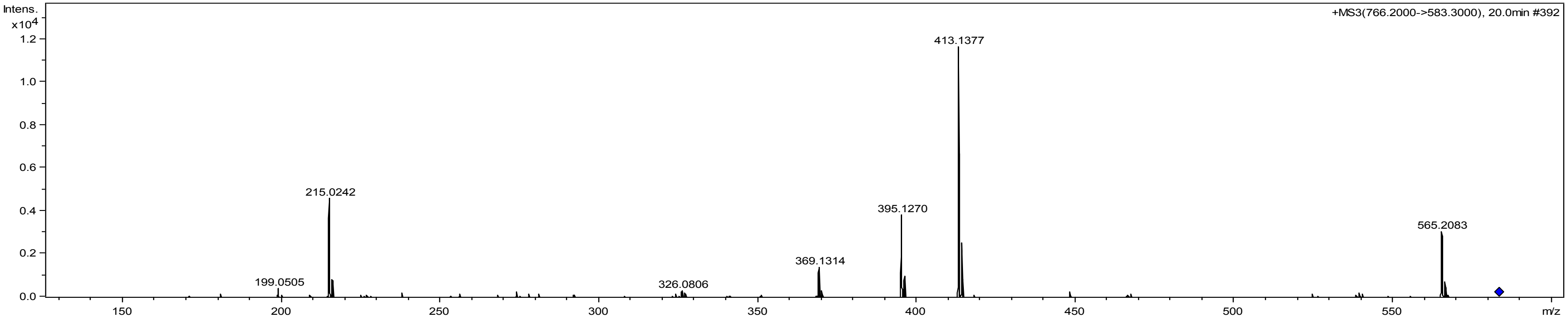

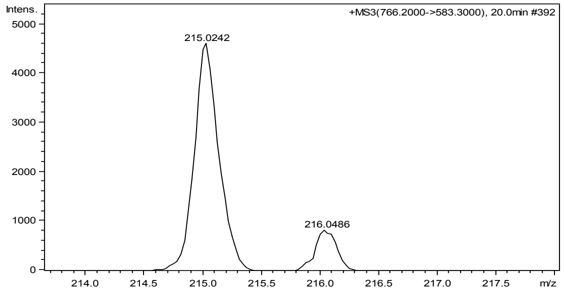

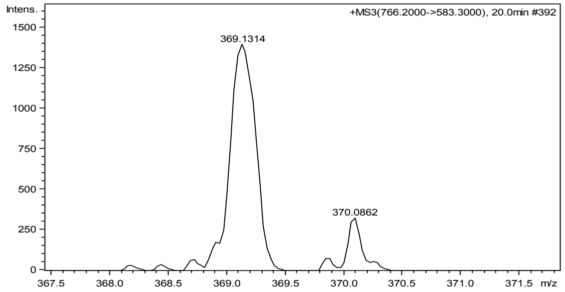

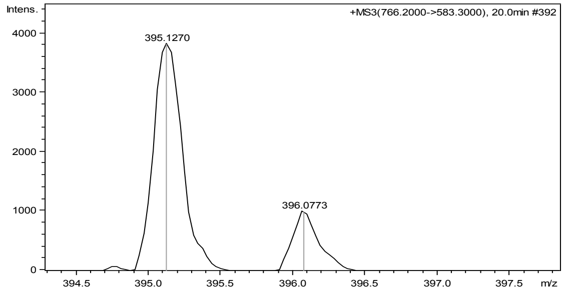

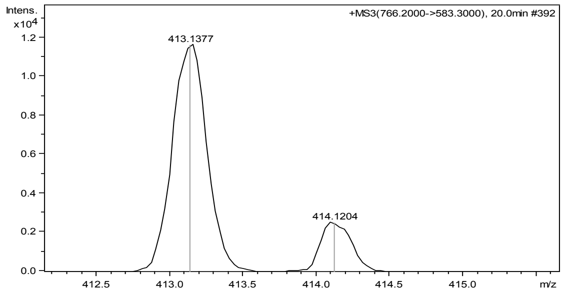

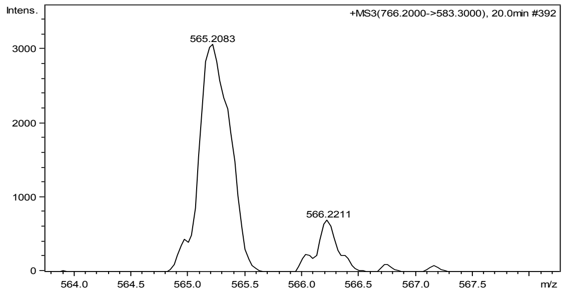

Fraction 15

765.77++ → Pep [M+H]+ 583.27+ [19.8-20.2 min]

CID-MS3 MASCOT Search

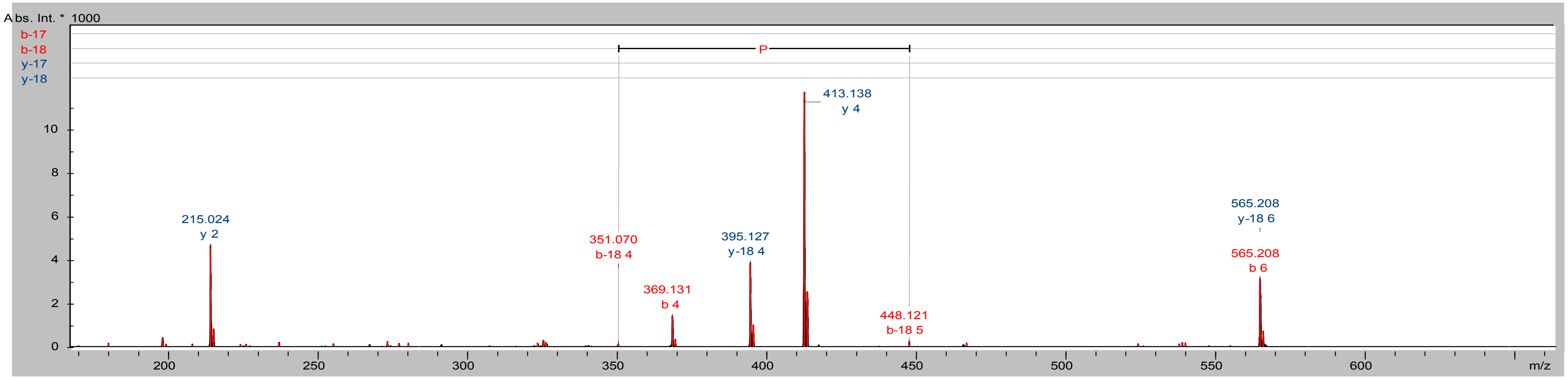

	A	V	P	T	P	V	Ala	Val	Pro	Thr	Pro	Val
Ion	1	2	3	4	5	6	1	2	3	4	5	6
b	A	V	P	T	P	V	72.044	171.113	268.166	369.213	466.266	565.334
b-17	A	V	P	T	P	V	-	-	-	-	-	-
b-18	A	V	P	T	P	V	-	-	-	351.203	448.255	547.324
y	A	V	P	T	P	V	118.086	215.139	316.187	413.239	512.308	583.345
y-17	A	V	P	T	P	V	-	-	-	-	-	-
y-18	A	V	P	T	P	V	-	-	298.176	395.229	494.297	565.334
	6	5	4	3	2	1	Val	Pro	Thr	Pro	Val	Ala

known O-glycosylation site

Alpha-2-HS-glycoprotein precursor

$_{267}$AVP**T**PV$_{272}$

Fraction 15

765.77++ → Pep [M+H]+ 583.27+ [19.8-20.2 min]

CID-MS3 MASCOT Search

prot_hit_nur	prot_acc	prot_desc	prot_score	prot_mass	prot_matche	pep_query	pep_rank	pep_isbold	pep_exp_mz	pep_exp_mr	pep_exp_z	pep_calc_mr	pep_delta	pep_miss	pep_score	pep_expect	pep_res_bef	pep_seq
1	CG013_HUM	Uncharacteri	16	22803	1	1	1	1	583.2743	582.267	1	582.3741	-0.1071	0	18.43	1.10E+02	V	AVVVPV
2	CT165_HUM	Uncharacteri	15	24161	1	1	1	0	583.2743	582.267	1	582.3377	-0.0707	0	18.43	1.10E+02	A	GIPTVP
3	TLX1_HUMA	T-cell leuker	15	34685	1	1	1	0	583.2743	582.267	1	582.3377	-0.0707	0	18.43	1.10E+02	T	GLPTVP
4	IBP7_HUMA	Insulin-like g	15	30138	1	1	1	0	583.2743	582.267	1	582.3377	-0.0707	0	18.43	1.10E+02	I	GIPTPV
5	FETUA_HUM	Alpha-2-HS-g	14	40098	1	1	1	0	583.2743	582.267	1	582.3377	-0.0707	0	18.43	1.10E+02	E	AVPTPV
6	NMNA2_HU	Nicotinamid	14	34930	1	1	1	0	583.2743	582.267	1	582.3741	-0.1071	0	18.43	1.10E+02	F	GIVVVP
7	F111A_HUM	Protein FAM	14	71007	1	1	1	0	583.2743	582.267	1	582.3377	-0.0707	0	18.43	1.10E+02	N	GITPVP
8	LARP1_HUM	La-related pr	13	123833	1	1	1	0	583.2743	582.267	1	582.3377	-0.0707	0	18.43	1.10E+02	R	AVTPVP
9	POL_HTL1A	Gag-Pro-Pol	12	163893	1	1	1	0	583.2743	582.267	1	582.3377	-0.0707	0	18.43	1.10E+02	D	AVPTVP
10	CEP35_HUM	Centrosome-	11	352312	1	1	1	0	583.2743	582.267	1	582.3377	-0.0707	0	18.43	1.10E+02	K	AVTPPV

Biotools-Score: 1

MASCOT-Score: 18

No unambiguous result

known O-glycosylation site

Alpha-2-HS-glycoprotein precursor

$_{267}$AVP**T**PV$_{272}$

Fraction 15

765.77++ → Pep [M+H]+ 583.27+ [19.8-20.2 min]

ETD

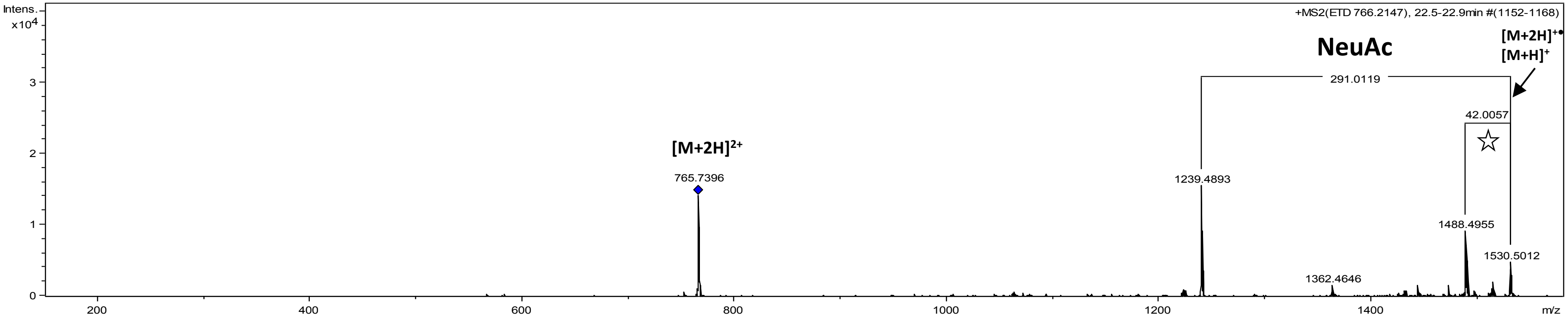

known O-glycosylation site

Alpha-2-HS-glycoprotein precursor

$_{267}$AVP**T**PV$_{272}$

Fraction 15

765.77++ → Pep [M+H]+ 583.27+ [19.8-20.2 min]   ETD

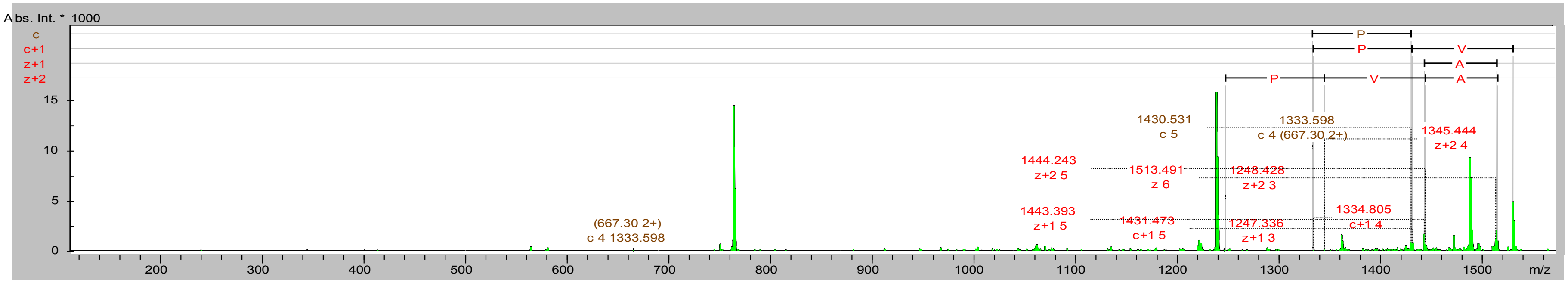

	A	V	P	T	P	V	Ala	Val	Pro	Thr	Pro	Val
Ion	1	2	3	4	5	6	1	2	3	4	5	6
c	A	V	P	T*	P	V	89.071	188.139	285.192	1333.563	1430.616	1529.684
c+1	A	V	P	T*	P	V	90.079	189.147	286.200	1334.571	1431.623	1530.692
z	A	V	P	T*	P	V	101.060	198.112	1246.483	1343.536	1442.604	1513.641
z+1	A	V	P	T*	P	V	102.068	199.120	1247.491	1344.544	1443.612	1514.649
z+2	A	V	P	T*	P	V	103.075	200.128	1248.499	1345.552	1444.620	1515.657
	6	5	4	3	2	1	Val	Pro	Thr	Pro	Val	Ala

Biotools-Score: 6

known O-glycosylation site

Alpha-2-HS-glycoprotein precursor $_{267}$AVP**T**PV$_{272}$

Fraction 15

895.87++ → Pep [M+H]+ 1134.58+ [22.8-23.7 min] CID-MS Precursor

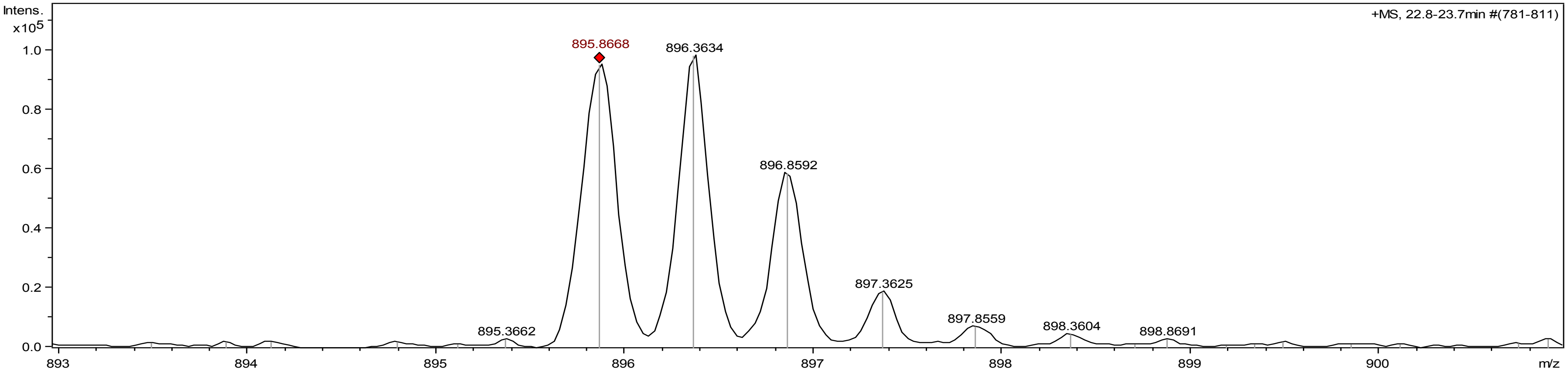

Fraction 15

895.87++ → Pep [M+H]+ 1134.58+ [22.8-23.7 min] CID-MS2

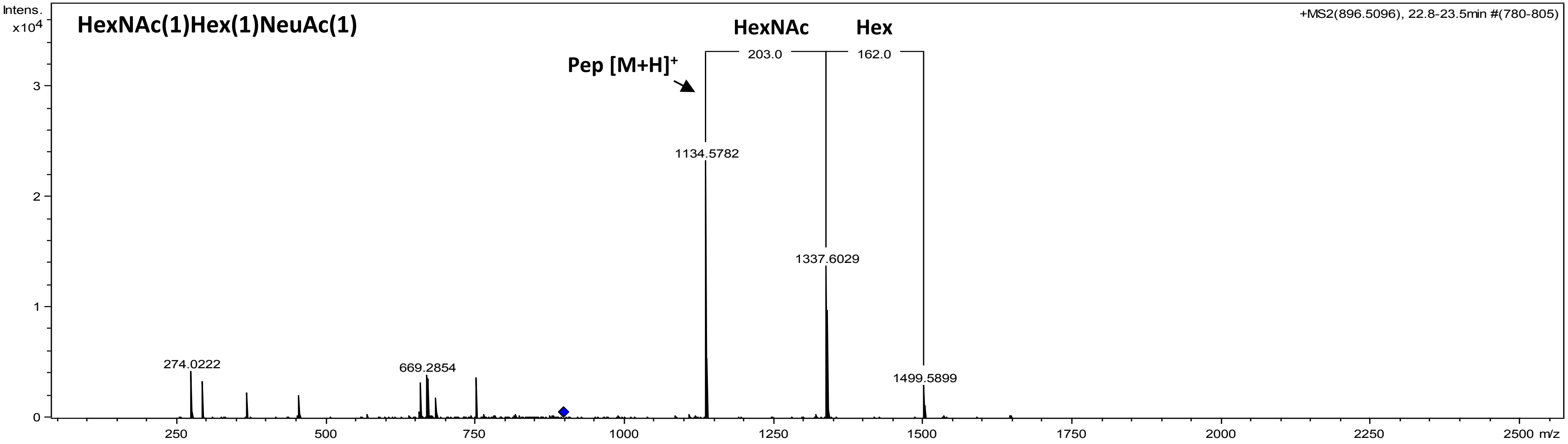

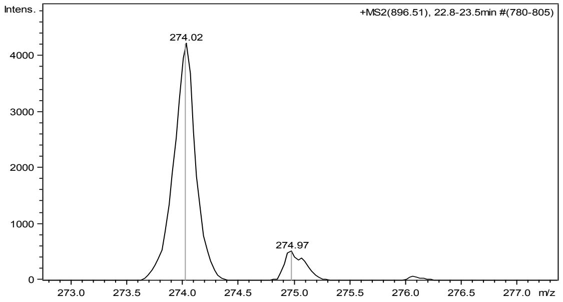

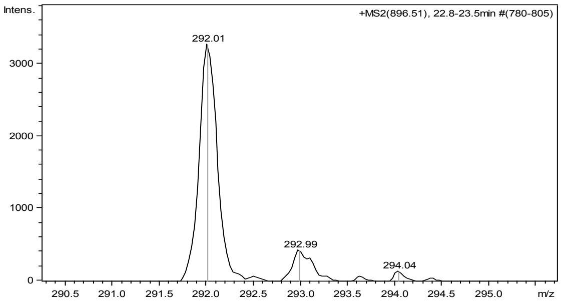

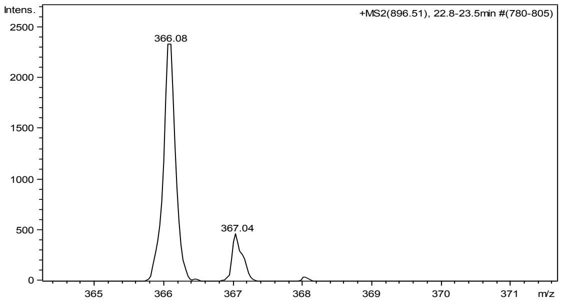

Fraction 15

895.87++ → Pep [M+H]+ 1134.58+ [22.8-23.7 min]

CID-MS2

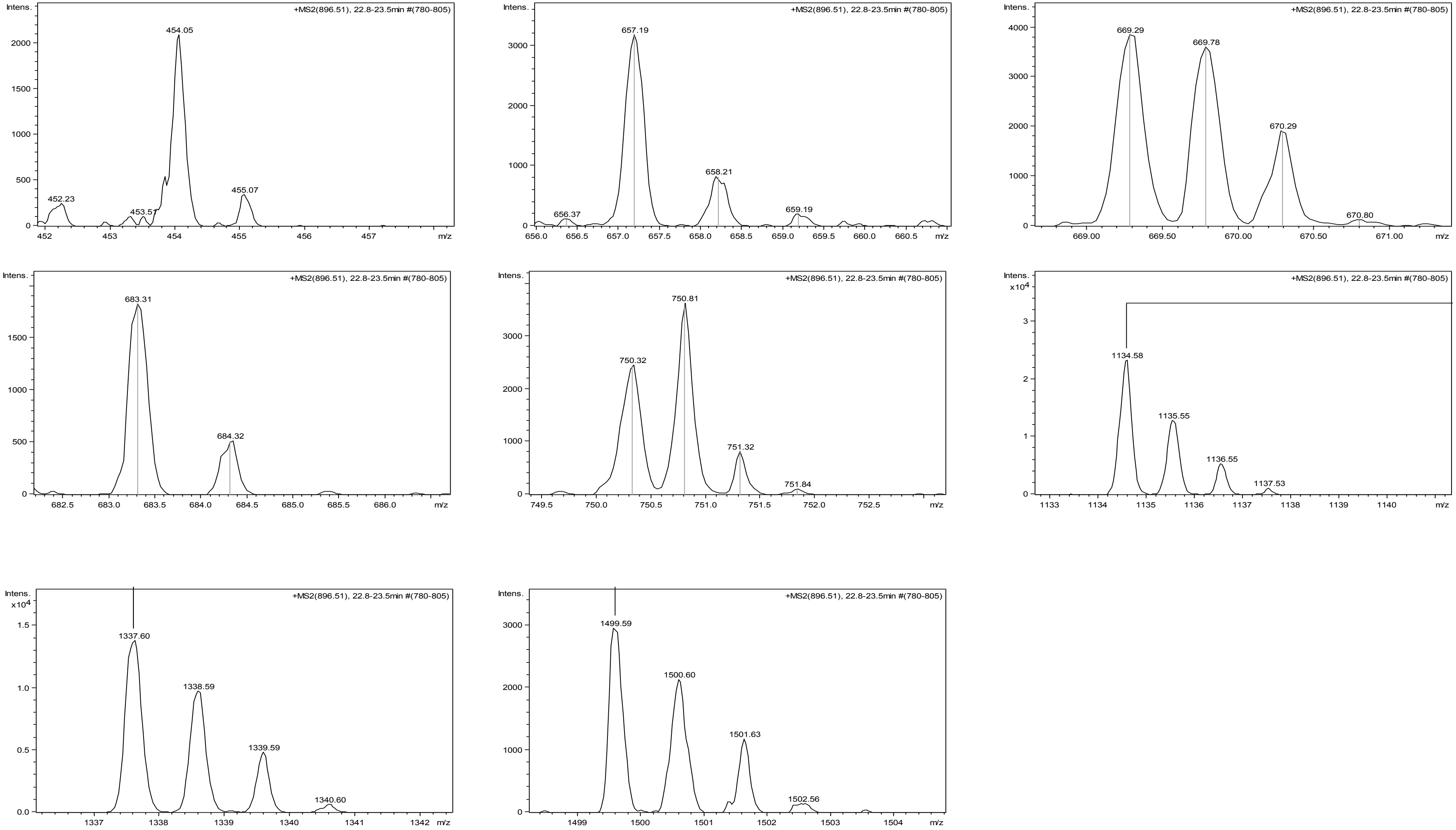

Fraction 15

895.87++ → Pep [M+H]+ 1134.58+ [22.8-23.7 min] CID-MS3

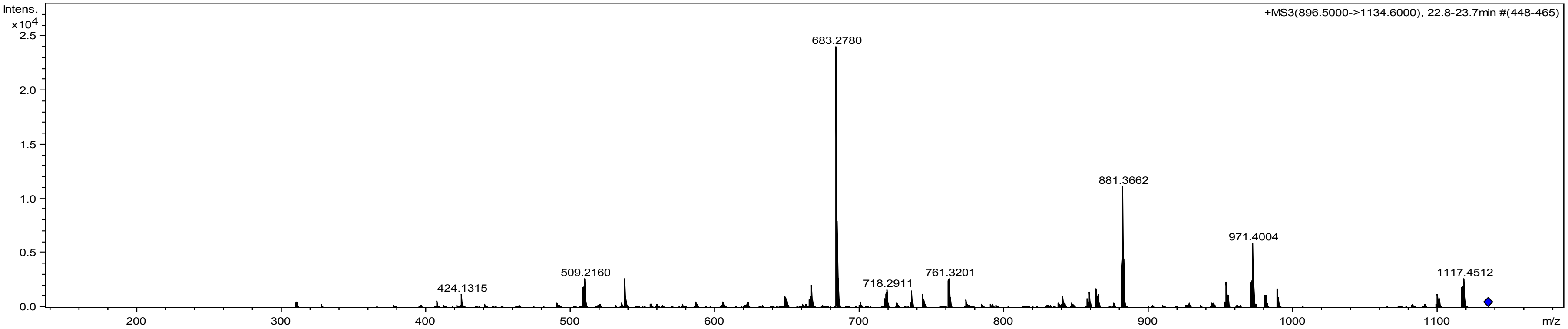

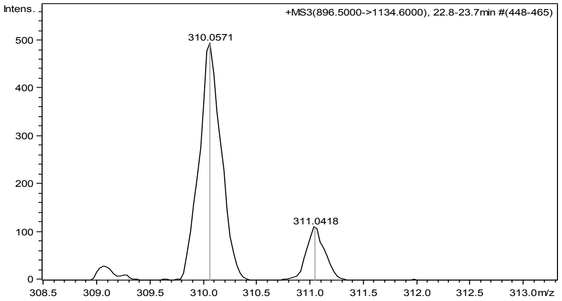

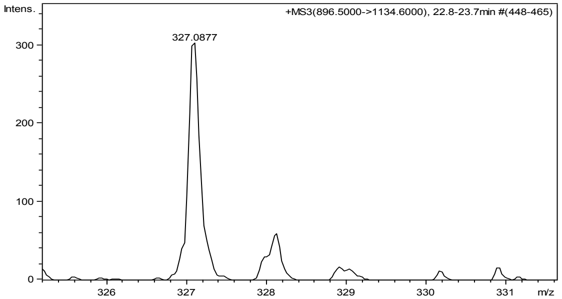

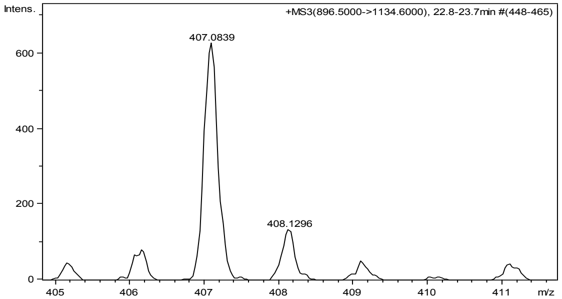

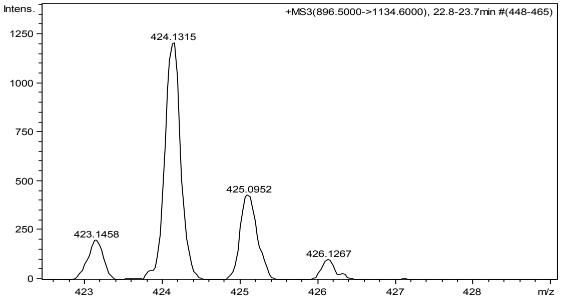

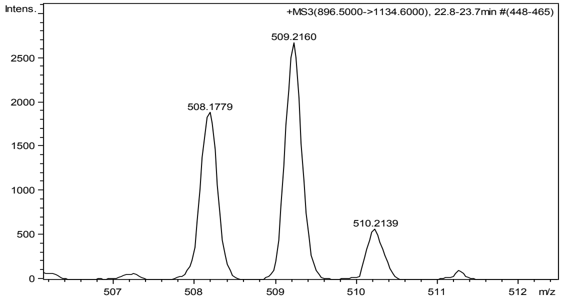

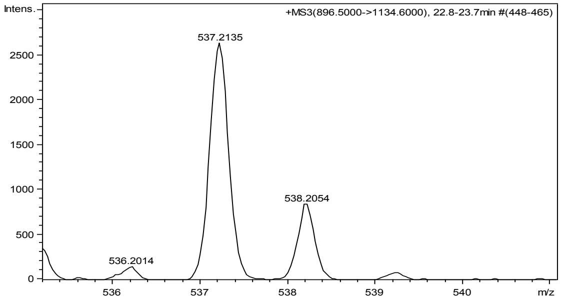

Fraction 15

895.87++ → Pep [M+H]+ 1134.58+ [22.8-23.7 min]

CID-MS3

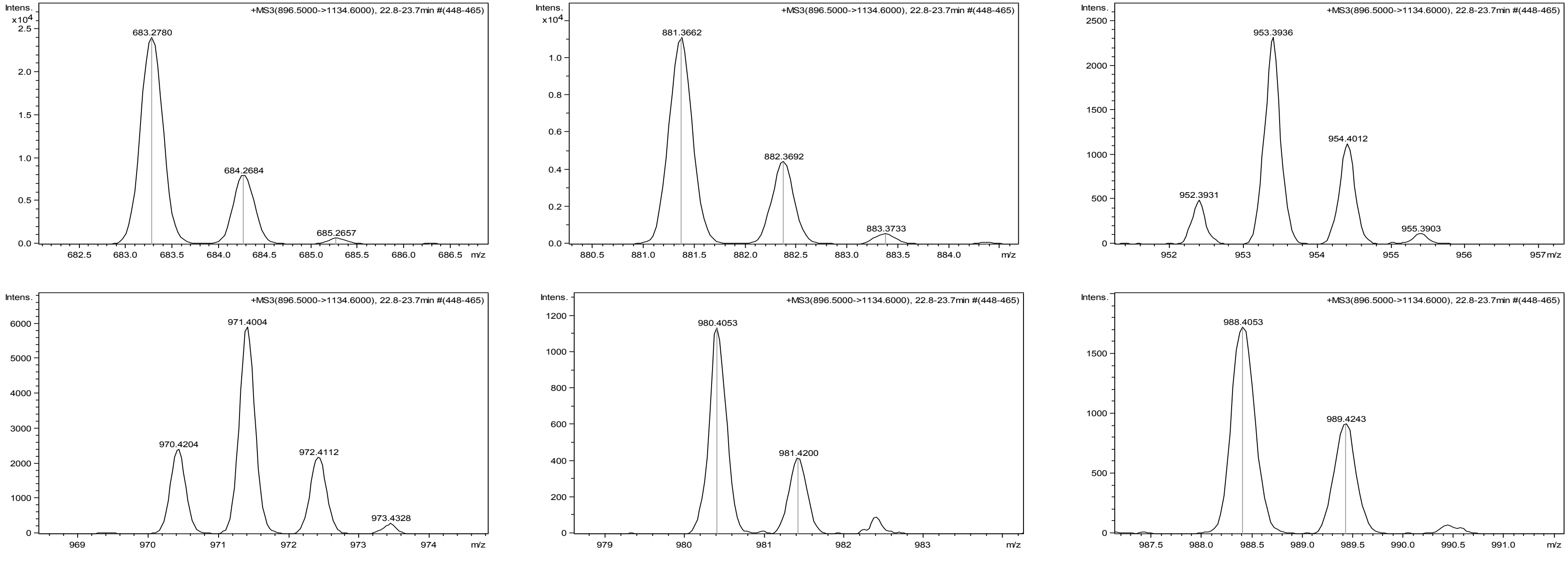

Fraction 15

895.87++ → Pep [M+H]+ 1134.58+ [22.8-23.7 min]   CID-MS3   MASCOT Search

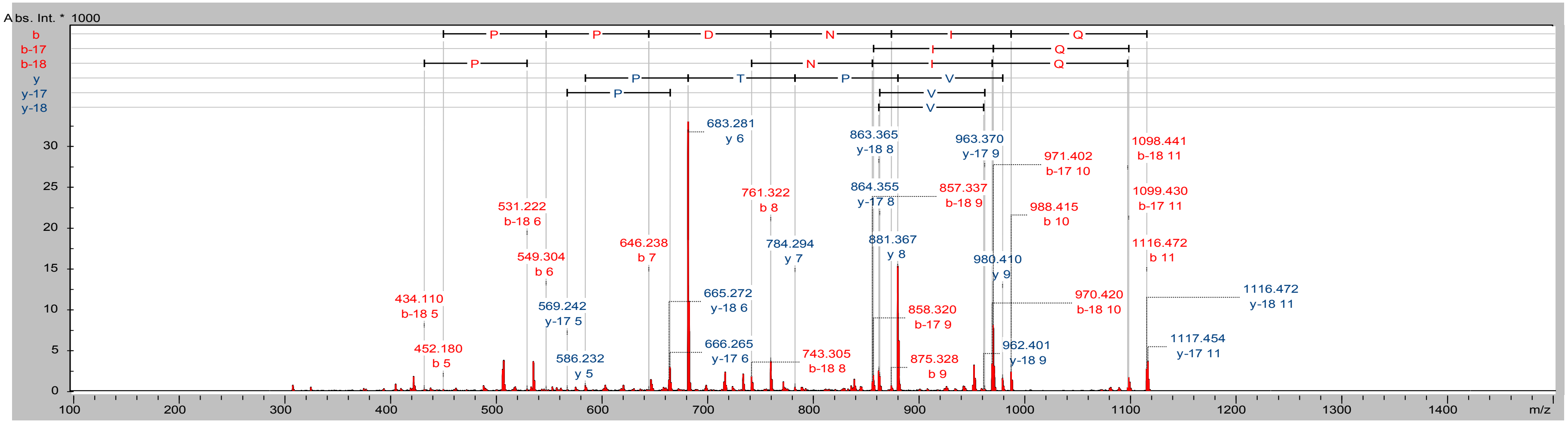

	G	P	V	P	T	P	P	D	N	I	Q	Gly	Pro	Val	Pro	Thr	Pro	Pro	Asp	Asn	Ile	Gln
Ion	1	2	3	4	5	6	7	8	9	10	11	1	2	3	4	5	6	7	8	9	10	11
b	G	P	V	P	T	P	P	D	N	I	Q	58.029	155.082	254.150	351.203	452.250	549.303	646.356	761.383	875.426	988.510	1116.568
b-17	G	P	V	P	T	P	P	D	N	I	Q	-	-	-	-	-	-	-	-	858.399	971.483	1099.542
b-18	G	P	V	P	T	P	P	D	N	I	Q	-	-	-	-	434.240	531.293	628.345	743.372	857.415	970.499	1098.558
y	G	P	V	P	T	P	P	D	N	I	Q	147.076	260.160	374.203	489.230	586.283	683.336	784.384	881.436	980.505	1077.557	1134.579
y-17	G	P	V	P	T	P	P	D	N	I	Q	130.050	243.134	357.177	472.204	569.257	666.309	767.357	864.410	963.478	1060.531	1117.552
y-18	G	P	V	P	T	P	P	D	N	I	Q	-	-	-	471.220	568.273	665.325	766.373	863.426	962.494	1059.547	1116.568
	11	10	9	8	7	6	5	4	3	2	1	Gln	Ile	Asn	Asp	Pro	Pro	Thr	Pro	Val	Pro	Gly

Fraction 15

895.87++ → Pep [M+H]+ 1134.58+ [22.8-23.7 min]

CID-MS3 MASCOT Search

prot_hit_nur	prot_acc	prot_desc	prot_score	prot_mass	prot_matche	pep_query	pep_rank	pep_isbold	pep_exp_mz	pep_exp_mr	pep_exp_z	pep_calc_mr	pep_delta	pep_miss	pep_score	pep_expect	pep_res_bef	pep_seq
1	K2C1_HUMA	Keratin, type	6	66149	1	1	2	1	1134.5782	1133.5709	1	1133.4738	0.0972	0	10.81	1.40E+03	S	YGSGSSSGGY
2	FOSL1_HUM/	Fos-related a	5	29737	1	1	6	0	1134.5782	1133.5709	1	1133.5869	-0.016	0	8.2	2.60E+03	S	YPRPLTYPQ
3	CN101_HUM	Uncharacteri	4	80275	1	1	4	0	1134.5782	1133.5709	1	1133.5393	0.0316	0	9.57	1.90E+03	L	YAQAYDLYK
4	ADO_HUMAI	Aldehyde ox	4	150401	1	1	1	0	1134.5782	1133.5709	1	1132.5441	1.0269	0	11.61	1.20E+03	V	YVSTQFPKY
5	TAF6_HUMA	Transcriptior	3	73250	1	1	3	0	1134.5782	1133.5709	1	1133.6921	-0.1212	0	9.65	1.80E+03	G	PRTPGLLKVP
6	RT05_HUMAI	Mitochondri:	3	48489	1	1	9	0	1134.5782	1133.5709	1	1133.5829	-0.012	0	7.69	2.90E+03	L	VAVGNGKGA
7	AMBP_HUM/	AMBP protei	3	39886	1	1	7	0	1134.5782	1133.5709	1	1133.5717	-0.0008	0	8.07	2.70E+03	A	GPVPTPPDN
8	DRBP1_HUM	Developmer	3	53868	1	1	5	0	1134.5782	1133.5709	1	1132.4495	1.1214	0	8.66	2.30E+03	Q	QFMQFGGSS
9	ABRA_HUMA	Actin-bindin	2	43319	1	1	8	0	1134.5782	1133.5709	1	1133.608	-0.0371	0	7.89	2.80E+03	Q	QKYSPVGNLI
10	KPB2_HUMA	Phosphoryla	0	139404	1	1	10	0	1134.5782	1133.5709	1	1133.5604	0.0105	0	7.63	2.90E+03	S	YLQELLPNSG

Biotools-Score: 196

MASCOT-Score: 8

known O-glycosylation site

Protein AMBP

$_{20}$GPVP**T**PPDNIQ$_{30}$

Fraction 15

895.87++ → Pep [M+H]+ 1134.58+ [22.8-23.7 min] ETD

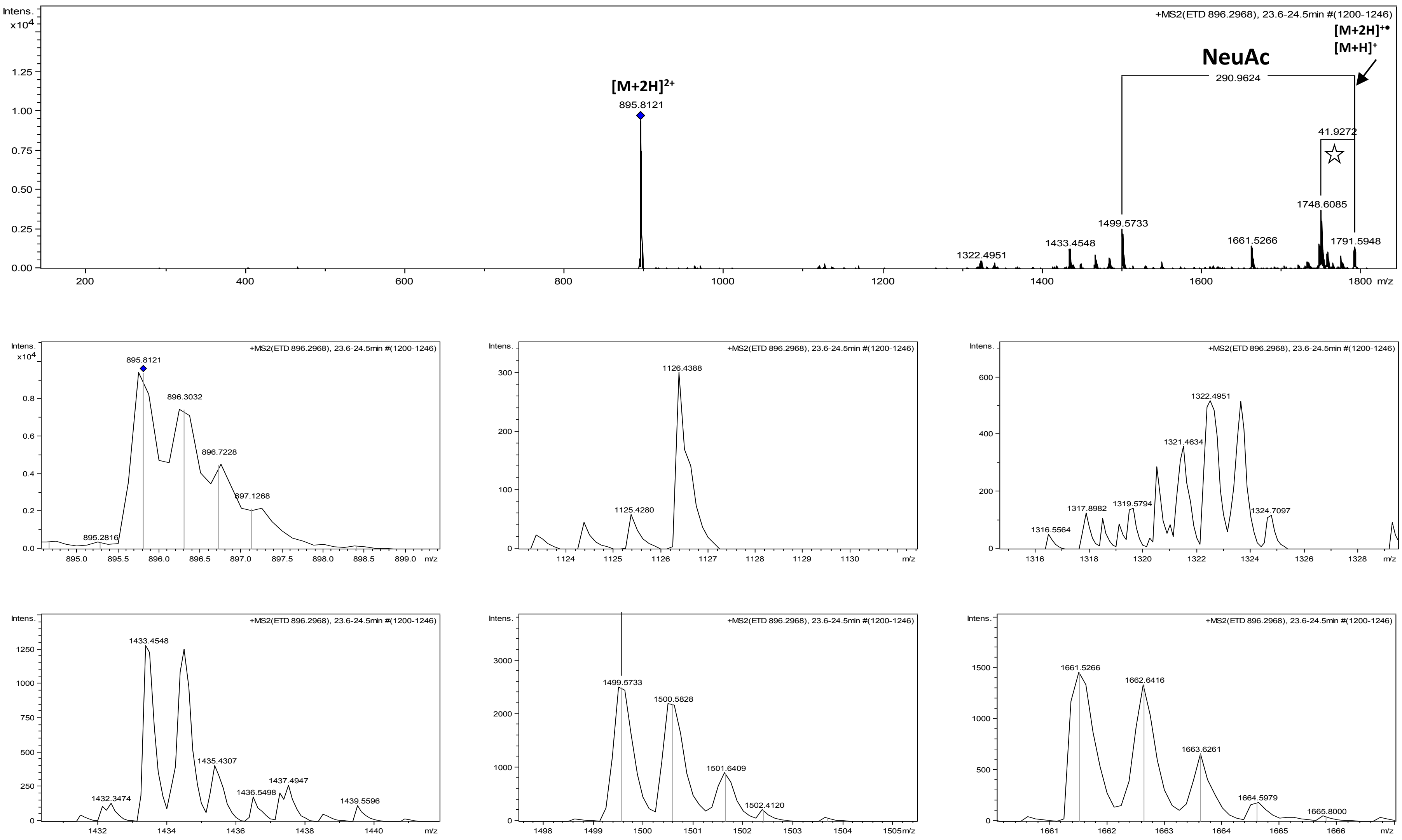

Fraction 15

895.87++ → Pep [M+H]+ 1134.58+ [22.8-23.7 min] ETD

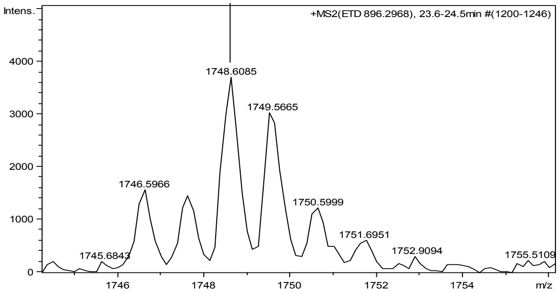

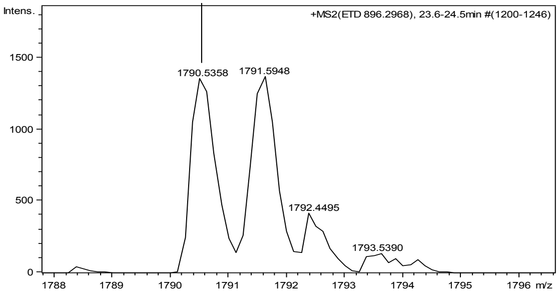

Fraction 15

895.87++ → Pep [M+H]+ 1134.58+ [22.8-23.7 min] ETD

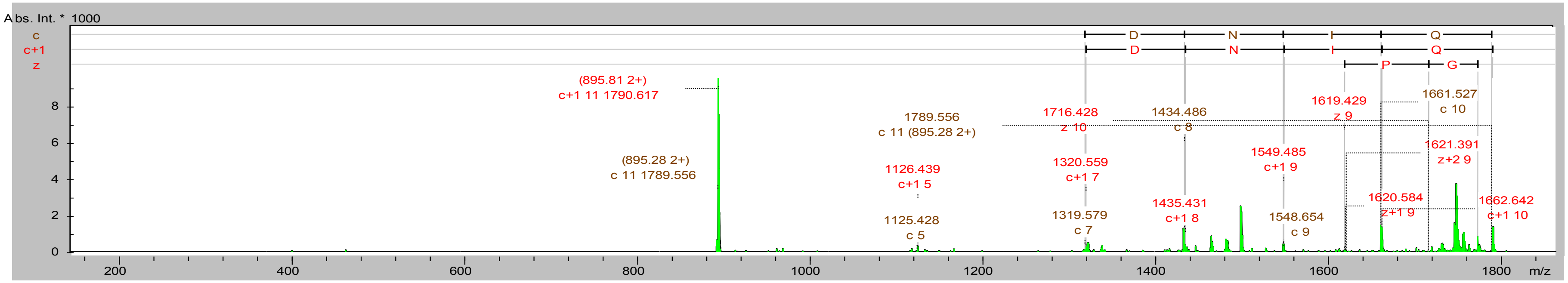

	G	P	V	P	T	P	P	D	N	I	Q	Gly	Pro	Val	Pro	Thr	Pro	Pro	Asp	Asn	Ile	Gln
Ion	1	2	3	4	5	6	7	8	9	10	11	1	2	3	4	5	6	7	8	9	10	11
c	G	P	V	P	T*	P	P	D	N	I	Q	75.055	172.108	271.176	368.229	1125.505	1222.557	1319.610	1434.637	1548.680	1661.764	1789.823
c+1	G	P	V	P	T*	P	P	D	N	I	Q	76.063	173.116	272.184	369.237	1126.512	1223.565	1320.618	1435.645	1549.688	1662.772	1790.830
z	G	P	V	P	T*	P	P	D	N	I	Q	130.050	243.134	357.177	472.204	569.257	666.309	1423.585	1520.637	1619.706	1716.759	1773.780
z+1	G	P	V	P	T*	P	P	D	N	I	Q	131.058	244.142	358.185	473.212	570.264	667.317	1424.592	1521.645	1620.714	1717.766	1774.788
z+2	G	P	V	P	T*	P	P	D	N	I	Q	132.066	245.150	359.193	474.219	571.272	668.325	1425.600	1522.653	1621.721	1718.774	1775.796
	11	10	9	8	7	6	5	4	3	2	1	Gln	Ile	Asn	Asp	Pro	Pro	Thr	Pro	Val	Pro	Gly

Biotools-Score: 34

known O-glycosylation site

Protein AMBP

$_{20}$GPVP**T**PPDNIQ$_{30}$

Fraction 15

831.84++ → Pep [M+H]+ 1006.51+ [25.4-25.7 min] CID-MS Precursor

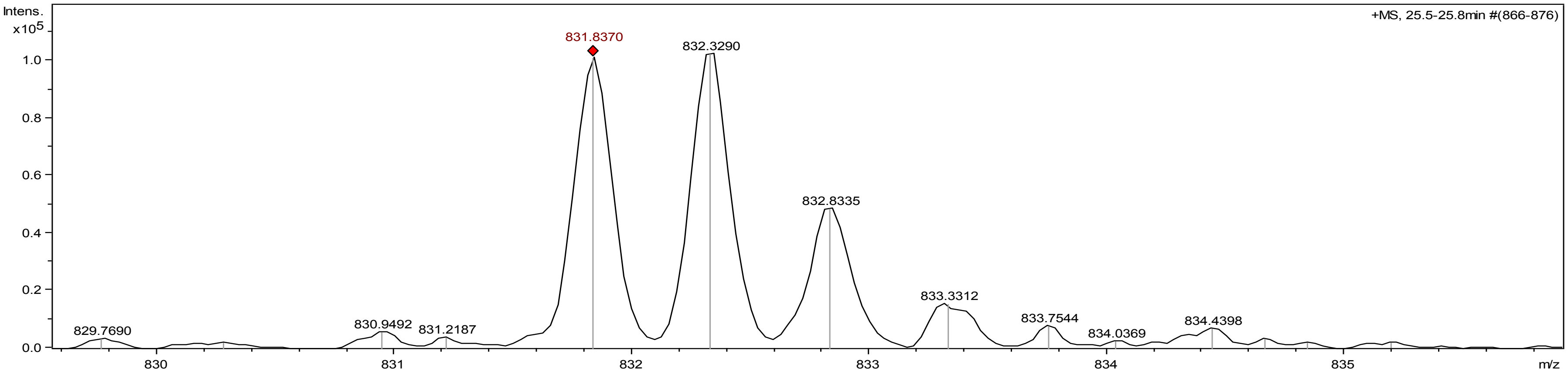

Fraction 15

831.84++ → Pep [M+H]+ 1006.51+ [25.4-25.7 min] CID-MS2

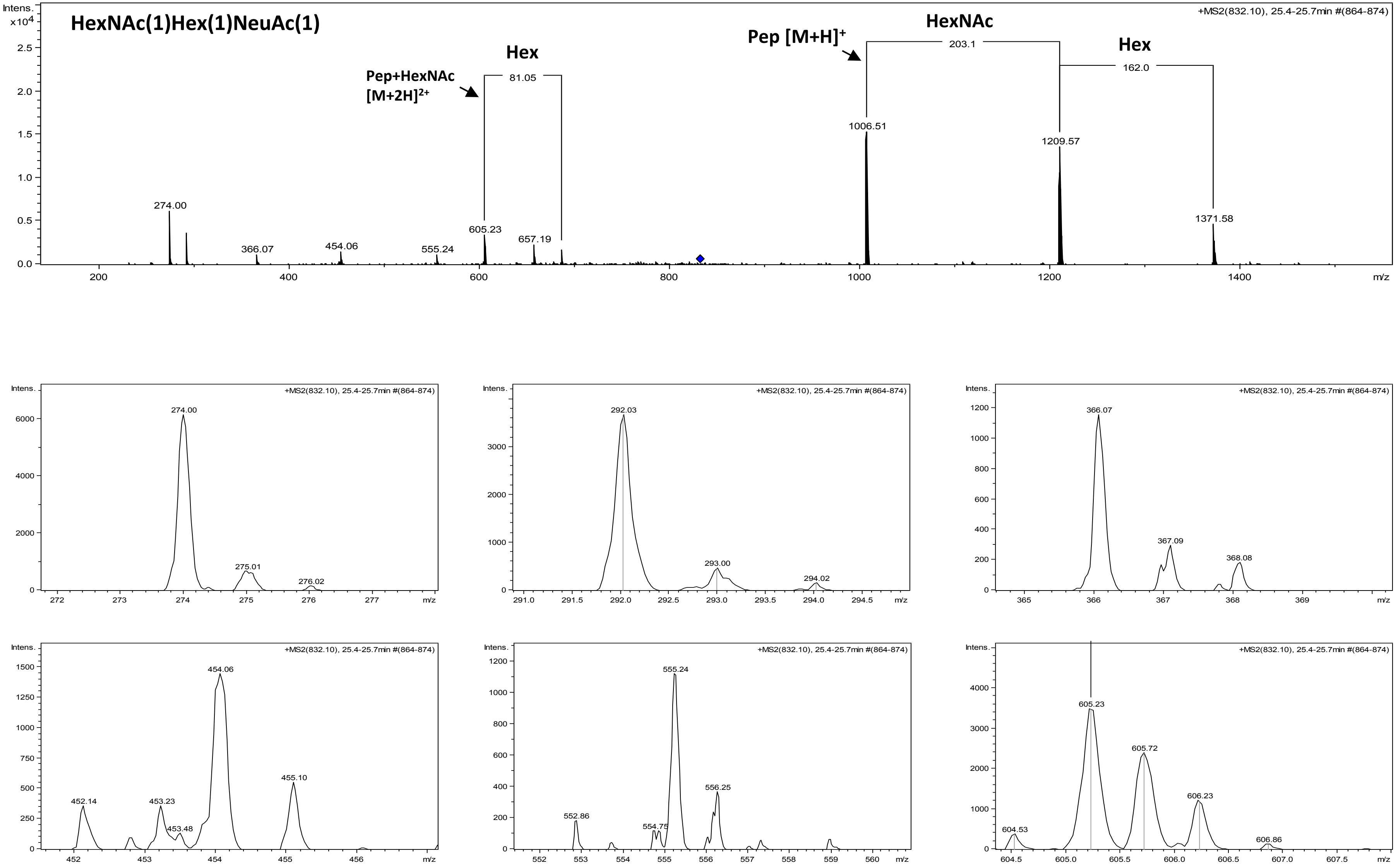

Fraction 15

831.84++ → Pep [M+H]+ 1006.51+ [25.4-25.7 min]

CID-MS2

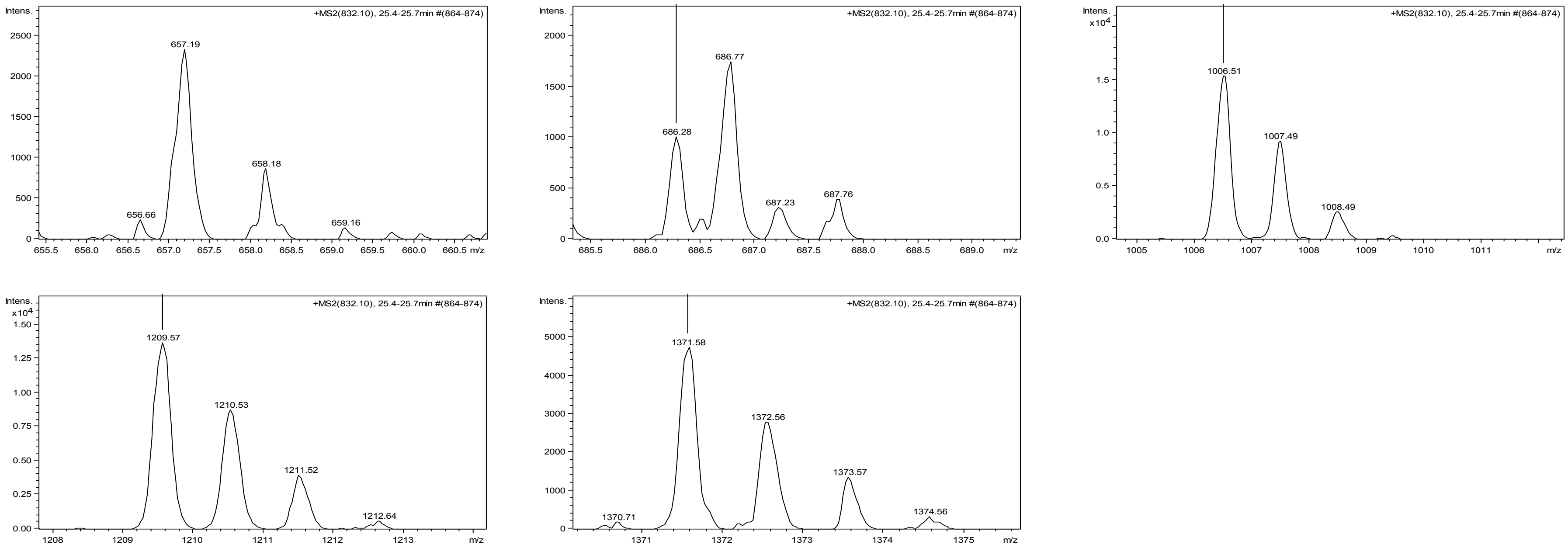

Fraction 15

831.84++ → Pep [M+H]+ 1006.51+ [25.4-25.7 min]

CID-MS3

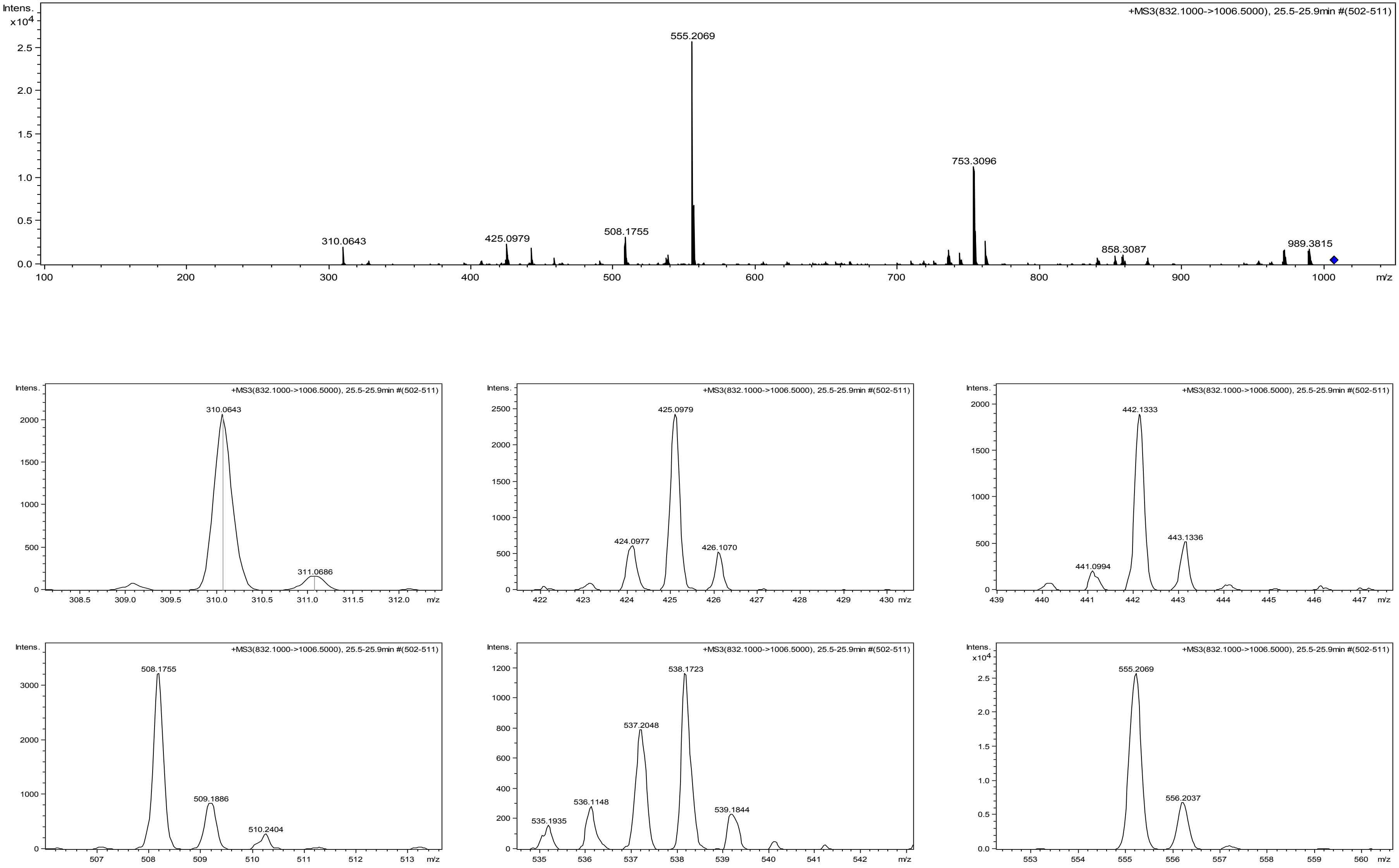

Fraction 15

831.84++ → Pep [M+H]+ 1006.51+ [25.4-25.7 min]

CID-MS3

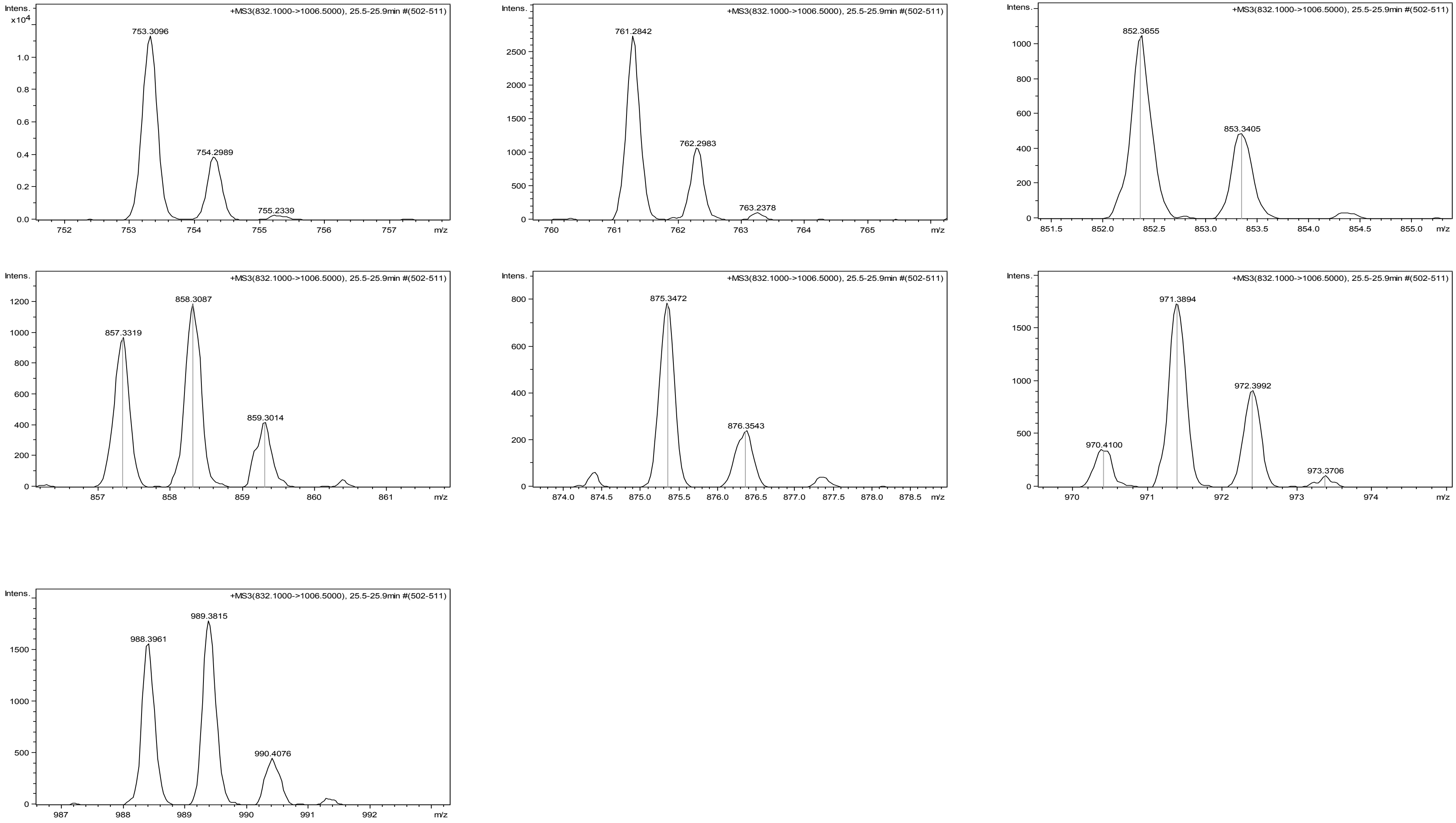

Fraction 15

831.84++ → Pep [M+H]+ 1006.51+ [25.4-25.7 min]   CID-MS3   MASCOT Search

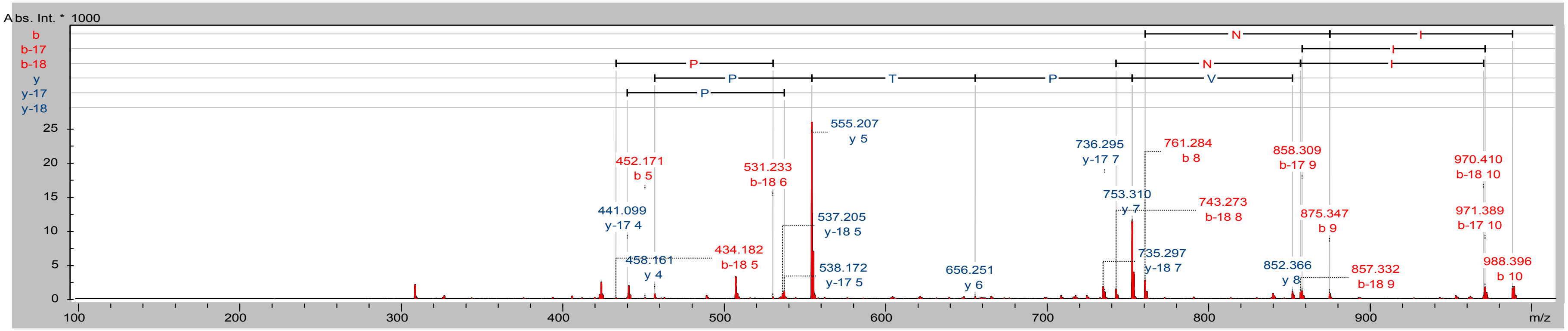

	G	P	V	P	T	P	P	D	N	I	Gly	Pro	Val	Pro	Thr	Pro	Pro	Asp	Asn	Ile
Ion	1	2	3	4	5	6	7	8	9	10	1	2	3	4	5	6	7	8	9	10
b	G	P	V	P	T	P	P	D	N	I	58.029	155.082	254.150	351.203	452.250	549.303	646.356	761.383	875.426	988.510
b-17	G	P	V	P	T	P	P	D	N	I	-	-	-	-	-	-	-	-	858.399	971.483
b-18	G	P	V	P	T	P	P	D	N	I	-	-	-	-	434.240	531.293	628.345	743.372	857.415	970.499
y	G	P	V	P	T	P	P	D	N	I	132.102	246.145	361.172	458.225	555.277	656.325	753.378	852.446	949.499	1006.520
y-17	G	P	V	P	T	P	P	D	N	I	-	229.118	344.145	441.198	538.251	639.298	736.351	835.420	932.472	989.494
y-18	G	P	V	P	T	P	P	D	N	I	-	-	343.161	440.214	537.267	638.314	735.367	834.436	931.488	988.510
	10	9	8	7	6	5	4	3	2	1	Ile	Asn	Asp	Pro	Pro	Thr	Pro	Val	Pro	Gly

known O-glycosylation site

Protein AMBP

$_{20}$GPVP**T**PPDNI$_{29}$

Fraction 15

831.84++ → Pep [M+H]+ 1006.51+ [25.4-25.7 min]

CID-MS3 MASCOT Search

prot_hit_nur	prot_acc	prot_desc	prot_score	prot_mass	prot_matche	pep_query	pep_rank	pep_isbold	pep_exp_mz	pep_exp_mr	pep_exp_z	pep_calc_mr	pep_delta	pep_miss	pep_score	pep_expect	pep_res_bef	pep_seq
1	AMBP_HUM/	AMBP protei	18	39886	1	1	1	1	1006.5099	1005.5026	1	1005.5131	-0.0105	0	22.33	94	A	GPVPTPPDN
2	GBB4_HUMA	Guanine nuc	10	38284	1	1	3	0	1006.5099	1005.5026	1	1005.5165	-0.0139	0	15.7	4.30E+02	A	TLVQITSNM
3	COHA1_HUN	Collagen alp	9	150784	1	1	2	0	1006.5099	1005.5026	1	1005.5131	-0.0105	0	16.76	3.40E+02	P	PGVSGALATY
4	LRRC1_HUM/	Leucine-rich	9	59946	1	1	5	0	1006.5099	1005.5026	1	1004.4298	1.0728	0	14.22	6.10E+02	E	NLVNDVSDE
5	CRLF1_HUM/	Cytokine rec	9	46957	1	1	5	0	1006.5099	1005.5026	1	1004.4662	1.0364	0	14.22	6.10E+02	W	KVVDDVSNC
6	IRF5_HUMAN	Interferon re	8	56579	1	1	9	0	1006.5099	1005.5026	1	1005.5607	-0.0581	0	13.2	7.70E+02	L	RPPTLQPPT
7	SNPC4_HUM	snRNA-activ	7	160134	1	1	7	0	1006.5099	1005.5026	1	1005.5859	-0.0833	0	14.19	6.10E+02	A	PGPTVLNVPL
8	TRIM5_HUM/	Tripartite mc	7	57500	1	1	8	0	1006.5099	1005.5026	1	1004.539	0.9637	0	13.26	7.60E+02	D	ILKSLTNSE
9	CRSP2_HUM/	CRSP comple	7	162043	1	1	4	0	1006.5099	1005.5026	1	1004.4927	1.0099	0	14.75	5.40E+02	F	PKQPGTSGAY
10	DGKD_HUM/	Diacylglycer	6	136607	1	1	10	0	1006.5099	1005.5026	1	1004.539	0.9637	0	13.08	7.90E+02	H	LSKILTSDQ

Biotools-Score: 36

MASCOT-Score: 22

known O-glycosylation site

Protein AMBP

$_{20}$GPVP**T**PPDNI$_{29}$

Fraction 15

831.84++ → Pep [M+H]+ 1006.51+ [25.4-25.7 min] ETD

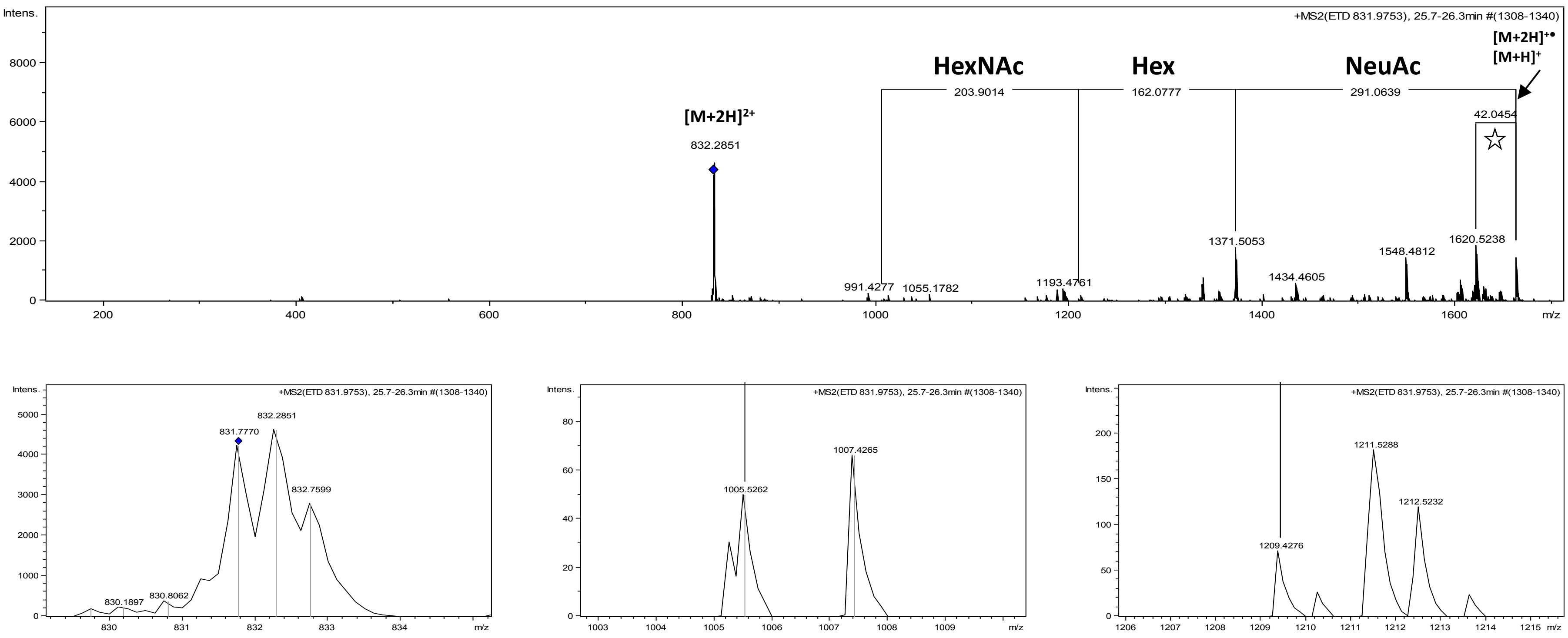

known O-glycosylation site

Protein AMBP

$_{20}$GPVP**T**PPDNI$_{29}$

Fraction 15

831.84++ → Pep [M+H]+ 1006.51+ [25.4-25.7 min]

ETD

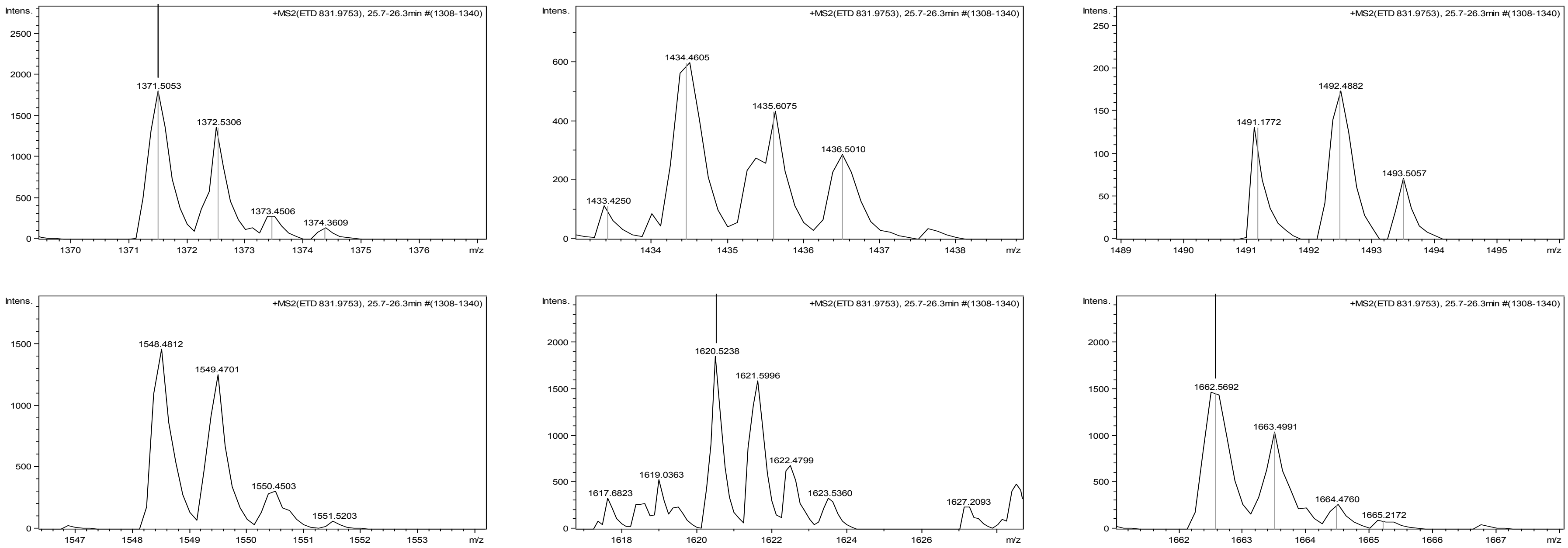

known O-glycosylation site

Protein AMBP

$_{20}$GPVP**T**PPDNI$_{29}$

Fraction 15

831.84++ → Pep [M+H]+ 1006.51+ [25.4-25.7 min] ETD

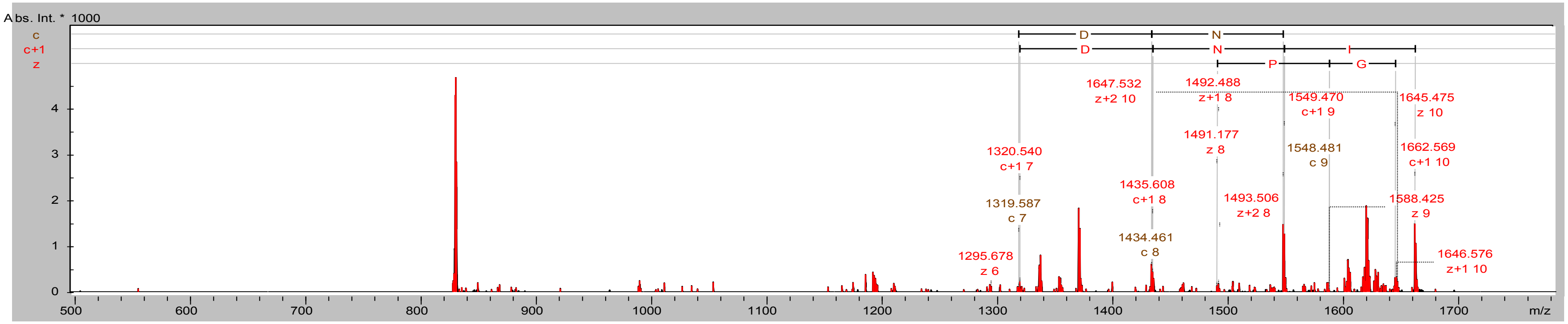

	G	P	V	P	T	P	P	D	N	I	Gly	Pro	Val	Pro	Thr	Pro	Pro	Asp	Asn	Ile
Ion	1	2	3	4	5	6	7	8	9	10	1	2	3	4	5	6	7	8	9	10
c	G	P	V	P	T*	P	P	D	N	I	75.055	172.108	271.176	368.229	1125.505	1222.557	1319.610	1434.637	1548.680	1661.764
c+1	G	P	V	P	T*	P	P	D	N	I	76.063	173.116	272.184	369.237	1126.512	1223.565	1320.618	1435.645	1549.688	1662.772
z	G	P	V	P	T*	P	P	D	N	I	115.075	229.118	344.145	441.198	538.251	1295.526	1392.579	1491.647	1588.700	1645.721
z+1	G	P	V	P	T*	P	P	D	N	I	116.083	230.126	345.153	442.206	539.259	1296.534	1393.587	1492.655	1589.708	1646.729
z+2	G	P	V	P	T*	P	P	D	N	I	117.091	231.134	346.161	443.214	540.266	1297.542	1394.594	1493.663	1590.716	1647.737
	10	9	8	7	6	5	4	3	2	1	Ile	Asn	Asp	Pro	Pro	Thr	Pro	Val	Pro	Gly

Biotools-Score: 33

known O-glycosylation site

Protein AMBP

$_{20}$GPVP**T**PPDNI$_{29}$

Fraction 15

680.61+++ → Pep [M+H]+ 1092.53+ [26.8-27.4 min] CID-MS Precursor

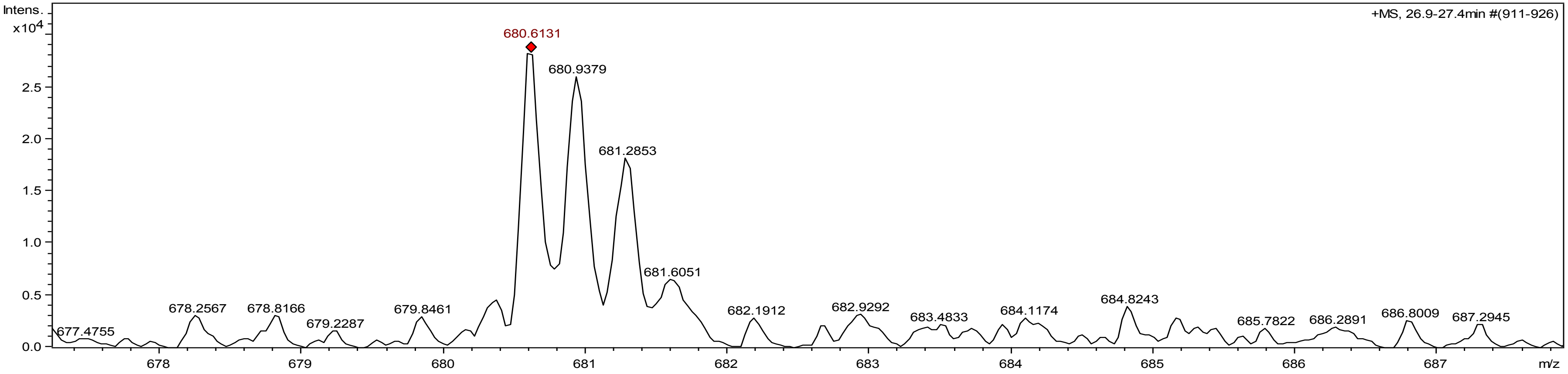

Fraction 15

680.61+++ → Pep [M+H]+ 1092.53+ [26.8-27.4 min] CID-MS2

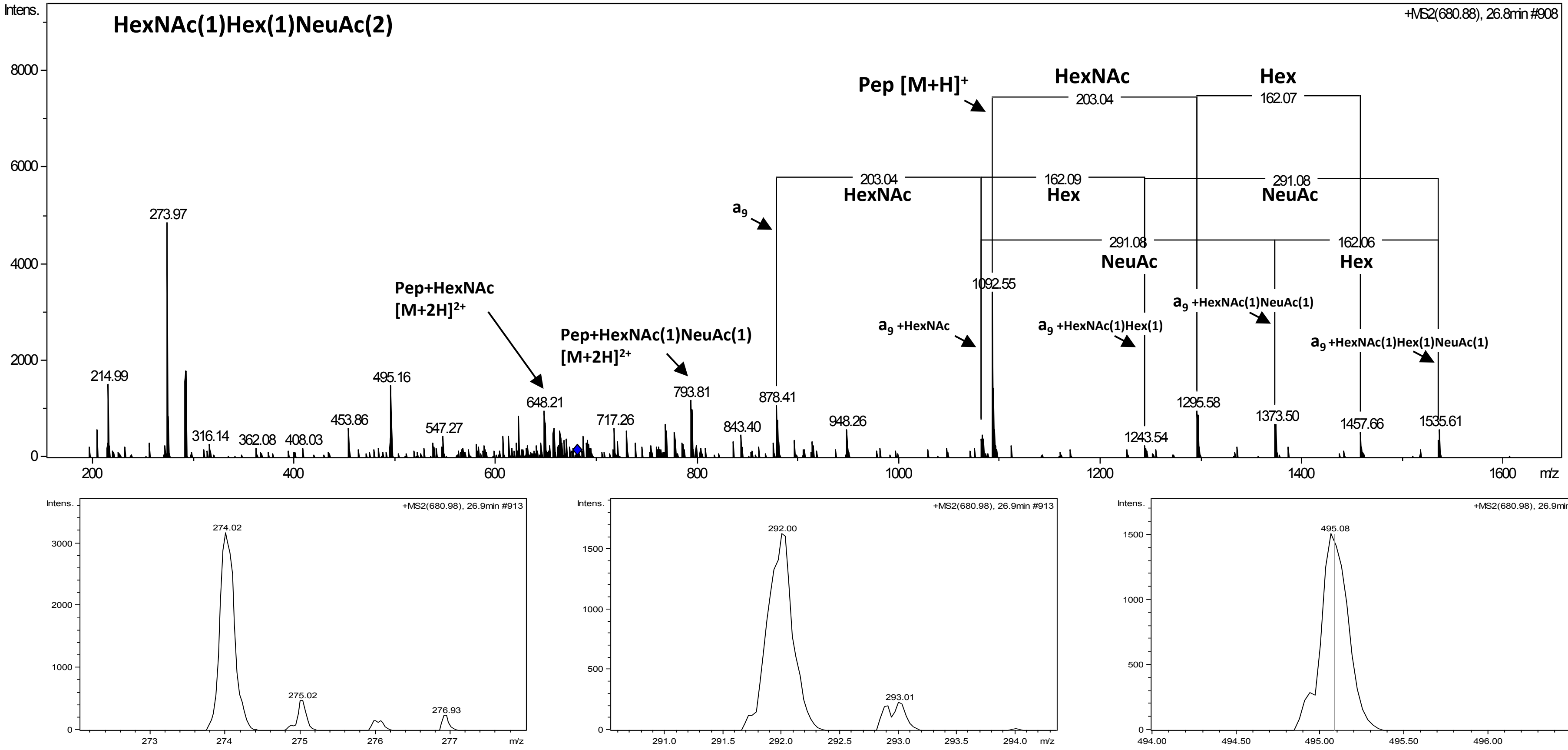

Fraction 15

680.61+++ → Pep [M+H]+ 1092.53+ [26.8-27.4 min] CID-MS2

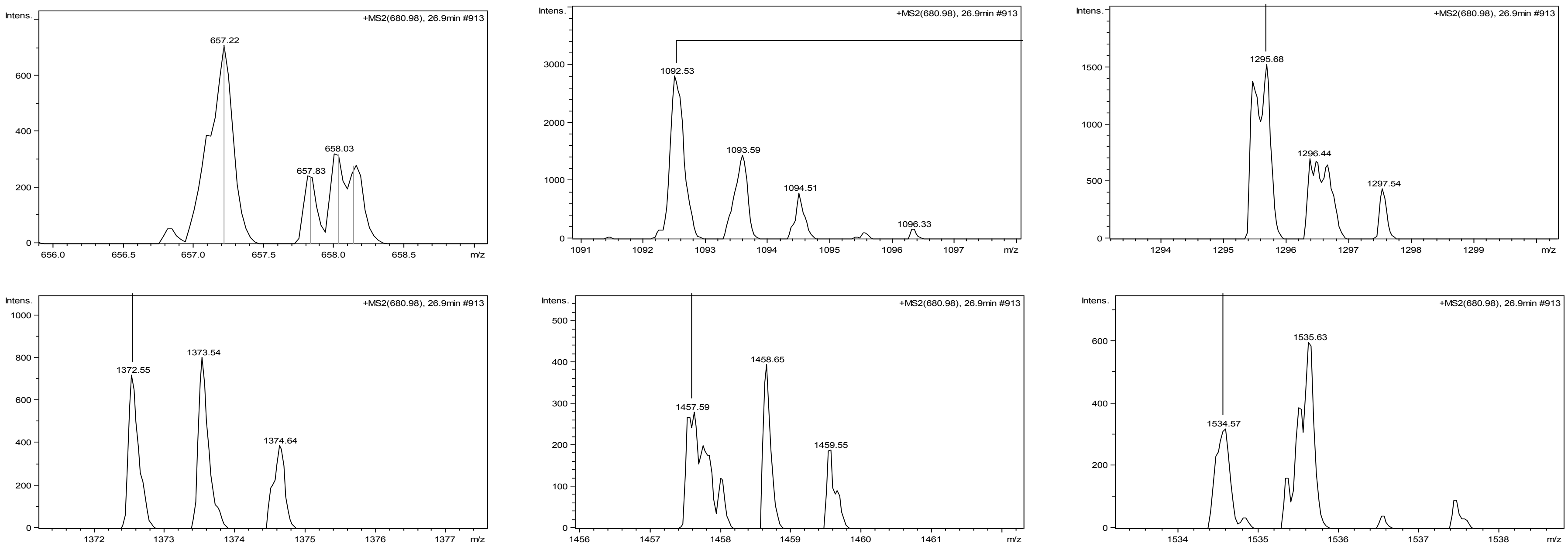

Fraction 15

680.61+++ → Pep [M+H]+ 1092.53+ [26.8-27.4 min] CID-MS3

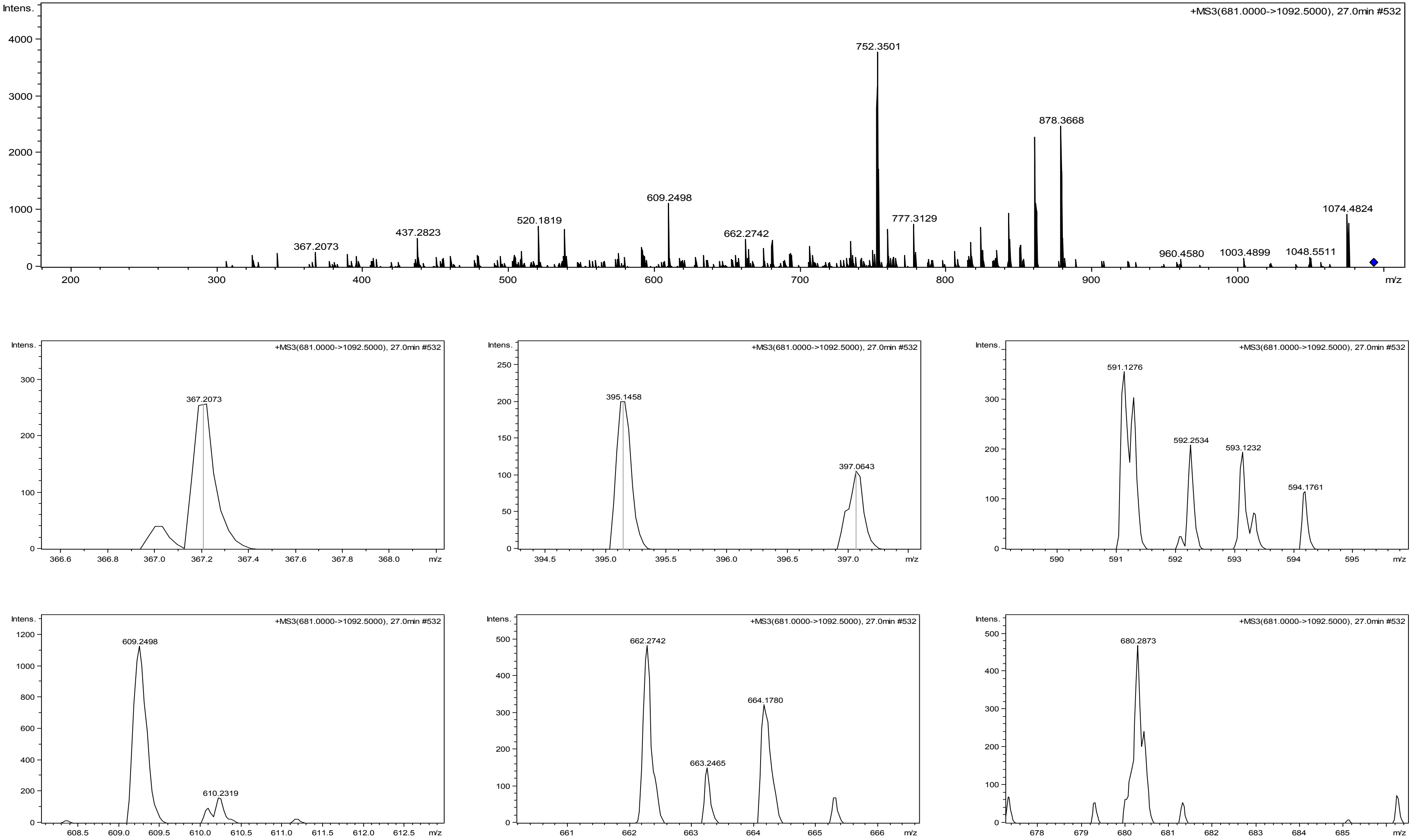

Fraction 15

680.61+++ → Pep [M+H]+ 1092.53+ [26.8-27.4 min] CID-MS3

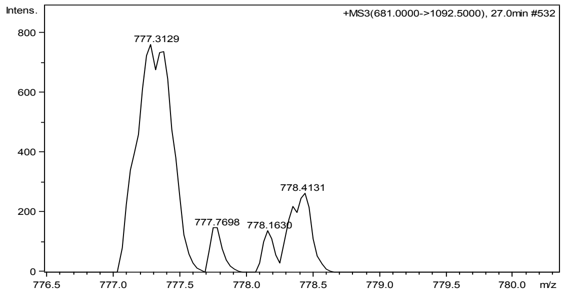

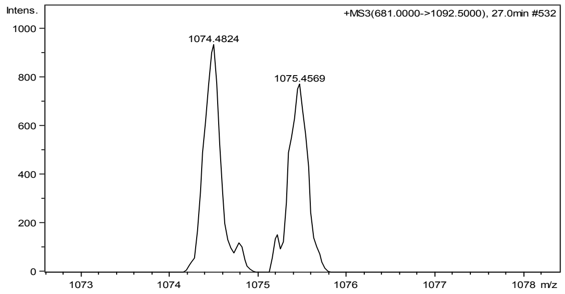

Fraction 15

680.61+++ → Pep [M+H]+ 1092.53+ [26.8-27.4 min] CID-MS3 MASCOT Search

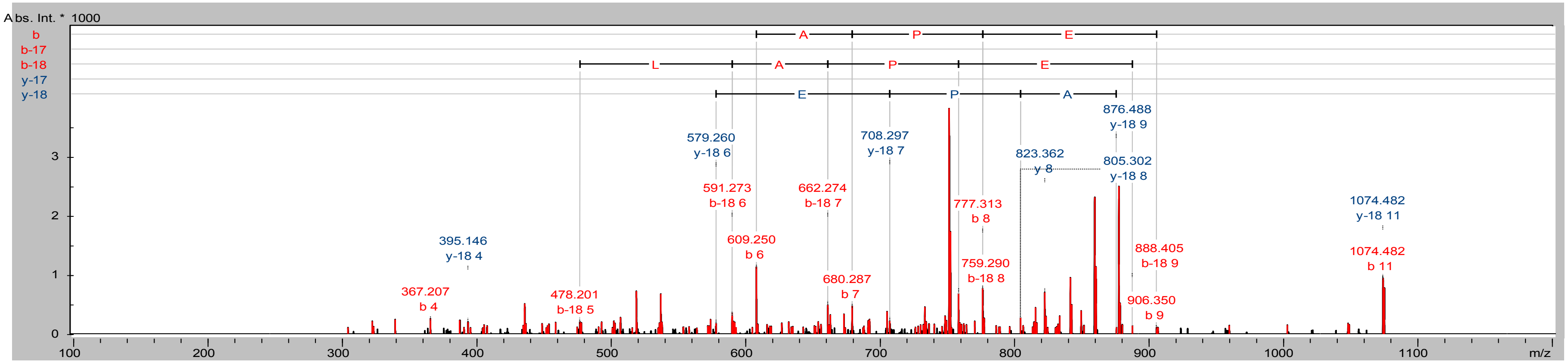

	T	P	A	P	E	L	A	P	E	A	P	Thr	Pro	Ala	Pro	Glu	Leu	Ala	Pro	Glu	Ala	Pro
Ion	1	2	3	4	5	6	7	8	9	10	11	1	2	3	4	5	6	7	8	9	10	11
b	T	P	A	P	E	L	A	P	E	A	P	102.055	199.108	270.145	367.198	496.240	609.324	680.361	777.414	906.457	977.494	1074.547
b-17	T	P	A	P	E	L	A	P	E	A	P	-	-	-	-	-	-	-	-	-	-	-
b-18	T	P	A	P	E	L	A	P	E	A	P	84.044	181.097	252.134	349.187	478.230	591.314	662.351	759.404	888.446	959.483	1056.536
y	T	P	A	P	E	L	A	P	E	A	P	116.071	187.108	316.150	413.203	484.240	597.324	726.367	823.420	894.457	991.509	1092.557
y-17	T	P	A	P	E	L	A	P	E	A	P	-	-	-	-	-	-	-	-	-	-	-
y-18	T	P	A	P	E	L	A	P	E	A	P	-	-	298.140	395.193	466.230	579.314	708.356	805.409	876.446	973.499	1074.547
	11	10	9	8	7	6	5	4	3	2	1	Pro	Ala	Glu	Pro	Ala	Leu	Glu	Pro	Ala	Pro	Thr

Most likely:

unknown O-glycosylation site

Tenascin-X precursor

$_{3586}$**T**PAPELAPEAP$_{3596}$

Fraction 15

680.61+++ → Pep [M+H]+ 1092.53+ [26.8-27.4 min] CID-MS3 MASCOT Search

[+] Internal Ions

[−] Theoretical Peak Table

70.0651	P	270.1448	b_3	393.2132	PELA-H_2O	551.3188	APELAP-28	749.4192	a_8		
74.0600	T	270.1448	PEA-28	393.2132	APEL-H_2O	561.3031	PAPELA-H_2O	751.3985	APELAPEA-28		
86.0964	L	270.1448	APE-28	395.1925	y_4-H_2O	561.3031	APELAP-H_2O	759.4036	b_8-H_2O		
102.0550	E	280.1292	PEA-H_2O	395.1925	PAPE	579.3137	PAPELA	761.3828	APELAPEA-H_2O		
116.0706	y_1	280.1292	APE-H_2O	411.2238	LAPE	579.3137	y_6-H_2O	777.4141	PAPELAPE-28		
126.0550	P	282.1812	LAP	411.2238	ELAP	579.3137	APELAP	777.4141	b_8		
141.1022	AP-28	286.1761	ELA-28	411.2238	PELA	581.3293	a_6	779.3934	APELAPEA		
141.1022	PA-28	296.1605	ELA-H_2O	411.2238	APEL	583.3086	ELAPEA-28	787.3985	PAPELAPE-H_2O		
157.1335	LA-28	298.1397	APE	413.2031	y_4	591.3137	b_6-H_2O	805.4090	y_8-H_2O		
169.0972	PA	298.1397	y_3-H_2O	454.2660	APELA-28	593.2930	ELAPEA-H_2O	805.4090	PAPELAPE		
169.0972	AP	298.1397	PEA	454.2660	LAPEA-28	597.3243	y_6	823.4196	y_8		
171.1128	a_2	312.1918	PEL-28	464.2504	APELA-H_2O	609.3243	b_6	848.4512	PAPELAPEA-28		
173.0921	EA-28	314.1710	ELA	464.2504	LAPEA-H_2O	609.3243	PELAPE-28	858.4356	PAPELAPEA-H_2O		
181.0972	b_2-H_2O	316.1503	y_3	466.2296	y_5-H_2O	611.3035	ELAPEA	876.4462	PAPELAPEA		
183.0764	EA-H_2O	322.1761	PEL-H_2O	468.2453	a_5	619.3086	PELAPE-H_2O	876.4462	y_9-H_2O		
185.1285	LA	339.2027	a_4	478.2296	b_5-H_2O	637.3192	PELAPE	878.4618	a_9		
187.1077	y_2	340.1867	PEL	480.2817	PELAP-28	648.3715	PAPELAP-28	888.4462	b_9-H_2O		
199.1077	b_2	341.1819	APEA-28	480.2817	PAPEL-28	652.3665	a_7	894.4567	y_9		
199.1077	PE-28	349.1870	b_4-H_2O	482.2609	LAPEA	658.3559	PAPELAP-H_2O	906.4567	b_9		
201.0870	EA	351.1663	APEA-H_2O	482.2609	APELA	662.3508	b_7-H_2O	949.4989	a_{10}		
209.0921	PE-H_2O	367.1976	PAPE-28	484.2402	y_5	676.3665	PAPELAP	959.4833	b_{10}-H_2O		
215.1390	EL-28	367.1976	b_4	490.2660	PELAP-H_2O	680.3614	b_7	973.4989	y_{10}-H_2O		
225.1234	EL-H_2O	369.1769	APEA	490.2660	PAPEL-H_2O	680.3614	APELAPE-28	977.4938	b_{10}		
227.1026	PE	377.1819	PAPE-H_2O	496.2402	b_5	680.3614	PELAPEA-28	991.5095	y_{10}		
238.1550	PAP-28	383.2289	APEL-28	508.2766	PAPEL	690.3457	APELAPE-H_2O	1074.5466	MH-H_2O		
242.1499	a_3	383.2289	PELA-28	508.2766	PELAP	690.3457	PELAPEA-H_2O	1092.5572	MH		
243.1339	EL	383.2289	ELAP-28	512.2715	ELAPE-28	708.3563	y_7-H_2O				
252.1343	b_3-H_2O	383.2289	LAPE-28	522.2558	ELAPE-H_2O	708.3563	PELAPEA				
254.1863	LAP-28	393.2132	LAPE-H_2O	540.2664	ELAPE	708.3563	APELAPE				
266.1499	PAP	393.2132	ELAP-H_2O	551.3188	PAPELA-28	726.3668	y_7				

MS-Product in ProteinProspector 5.12.4 Basic

© Copyright (1995-2014) The Regents of the University of California.

Most likely:

unknown O-glycosylation site

Tenascin-X precursor

$_{3586}$**T**PAPELAPEAP$_{3596}$

Fraction 15

680.61+++ → Pep [M+H]+ 1092.53+ [26.8-27.4 min]

CID-MS3 MASCOT Search

prot_hit_nur	prot_acc	prot_desc	prot_score	prot_mass	prot_matche	pep_query	pep_rank	pep_isbold	pep_exp_mz	pep_exp_mr	pep_exp_z	pep_calc_mr	pep_delta	pep_miss	pep_score	pep_expect	pep_res_bef	pep_seq
1	CNGB1_HUM	Cyclic nuclec	7	103075	1	1	2	1	1092.5286	1091.5213	1	1091.5975	-0.0761	0	13.33	7.70E+02	K	PSPAKKAPEF
2	NPAS2_HUM	Neuronal PA	7	92501	1	1	3	0	1092.5286	1091.5213	1	1091.4982	0.0231	0	13.26	7.80E+02	N	LTTPASTSQD
3	TENX_HUMA	Tenascin-X p	6	471178	1	1	1	0	1092.5286	1091.5213	1	1091.5499	-0.0285	0	15.11	5.10E+02	D	TPAPELAPEA
4	PBX3_HUMA	Pre-B-cell le	5	47445	1	1	6	0	1092.5286	1091.5213	1	1091.4771	0.0442	0	11.93	1.10E+03	G	WQDATTPSS
5	SP4_HUMAN	Transcriptior	5	82603	1	1	8	0	1092.5286	1091.5213	1	1091.4982	0.0231	0	11.69	1.10E+03	V	TITSVAGQQ(
6	CC020_HUM/	Uncharacteri	5	102228	1	1	7	0	1092.5286	1091.5213	1	1090.5295	0.9918	0	11.83	1.10E+03	R	GVGTPANSLI
7	DDFL1_HUM/	Developmer	5	100177	1	1	10	0	1092.5286	1091.5213	1	1091.5135	0.0078	0	11.21	1.30E+03	G	QPLPGPDAQ
8	WWTR1_HUI	WW domain-	5	44244	1	1	5	0	1092.5286	1091.5213	1	1091.4982	0.0231	0	11.99	1.00E+03	N	DVESALNKSE
9	NMD3A_HUI	Glutamate [I	5	126656	1	1	4	0	1092.5286	1091.5213	1	1091.5533	-0.0319	0	12.11	1.00E+03	A	DVTVSILTMN
10	SON_HUMAI	SON protein	4	264079	1	1	9	0	1092.5286	1091.5213	1	1091.5863	-0.065	0	11.63	1.10E+03	V	PELPGPSVTP

Biotools-Score: 49

MASCOT-Score: 15

Most likely:

unknown O-glycosylation site

Tenascin-X precursor

$_{3586}$**T**PAPELAPEAP$_{3596}$

Fraction 15

680.61+++ → Pep [M+H]+ 1092.53+ [26.8-27.4 min] ETD

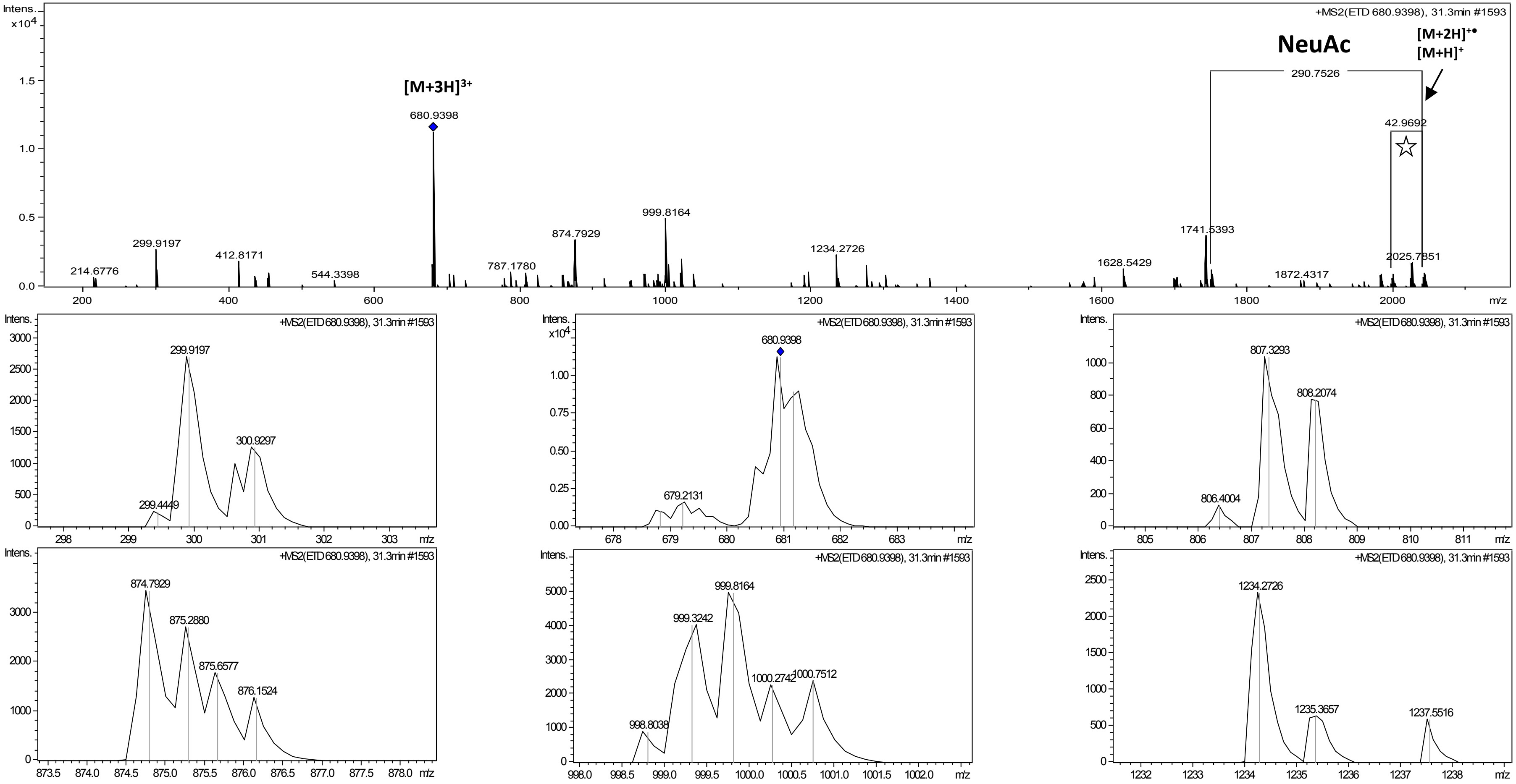

Most likely:

unknown O-glycosylation site

Tenascin-X precursor

$_{3586}$**T**PAPELAPEAP$_{3596}$

Fraction 15

680.61+++ → Pep [M+H]+ 1092.53+ [26.8-27.4 min]

ETD

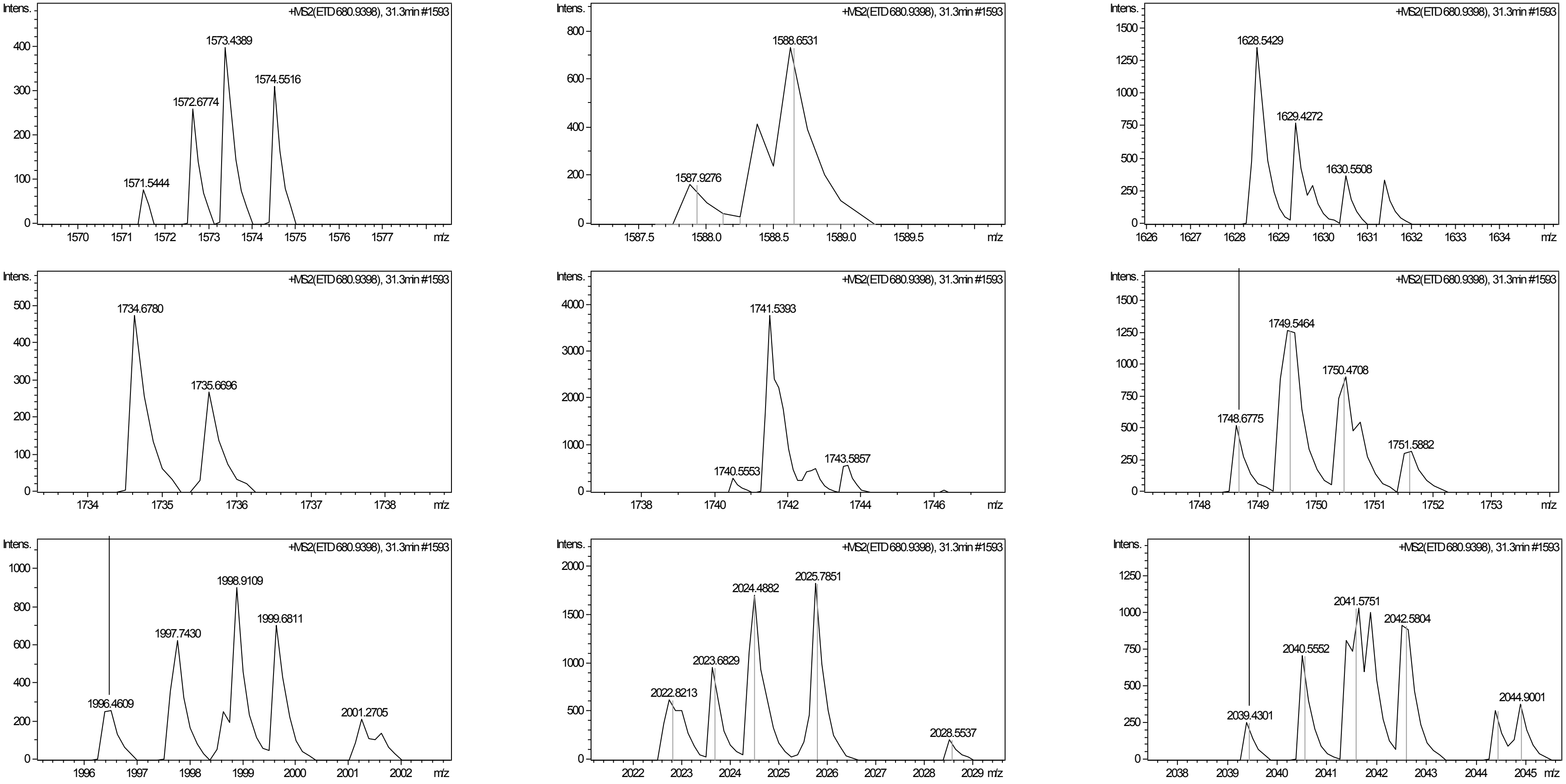

Most likely:

unknown O-glycosylation site

Tenascin-X precursor

$_{3586}$**T**PAPELAPEAP$_{3596}$

Fraction 15

680.61+++ → Pep [M+H]+ 1092.53+ [26.8-27.4 min] ETD

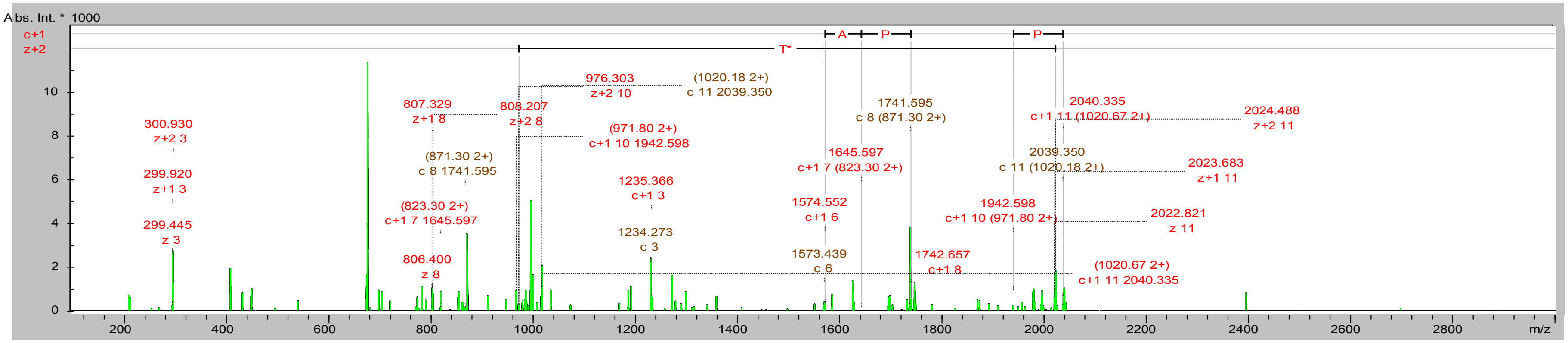

	T	P	A	P	E	L	A	P	E	A	P	Thr	Pro	Ala	Pro	Glu	Leu	Ala	Pro	Glu	Ala	Pro
Ion	1	2	3	4	5	6	7	8	9	10	11	1	2	3	4	5	6	7	8	9	10	11
c	T*	P	A	P	E	L	A	P	E	A	P	1066.405	1163.457	1234.494	1331.547	1460.590	1573.674	1644.711	1741.764	1870.806	1941.843	2038.896
c+1	T*	P	A	P	E	L	A	P	E	A	P	1067.412	1164.465	1235.502	1332.555	1461.598	1574.682	1645.719	1742.772	1871.814	1942.851	2039.904
z	T*	P	A	P	E	L	A	P	E	A	P	99.044	170.081	299.124	396.177	467.214	580.298	709.340	806.393	877.430	974.483	2022.854
z+1	T*	P	A	P	E	L	A	P	E	A	P	100.052	171.089	300.132	397.184	468.221	581.306	710.348	807.401	878.438	975.491	2023.861
z+2	T*	P	A	P	E	L	A	P	E	A	P	101.060	172.097	301.139	398.192	469.229	582.313	711.356	808.409	879.446	976.499	2024.869
	11	10	9	8	7	6	5	4	3	2	1	Pro	Ala	Glu	Pro	Ala	Leu	Glu	Pro	Ala	Pro	Thr

Biotools-Score: 26

Most likely:

unknown O-glycosylation site

Tenascin-X precursor

$_{3586}$**T**PAPELAPEAP$_{3596}$

Fraction 15

630.57+++ → Pep [M+H]+ 1233.58+ [27.5-28.5 min]

CID-MS Precursor

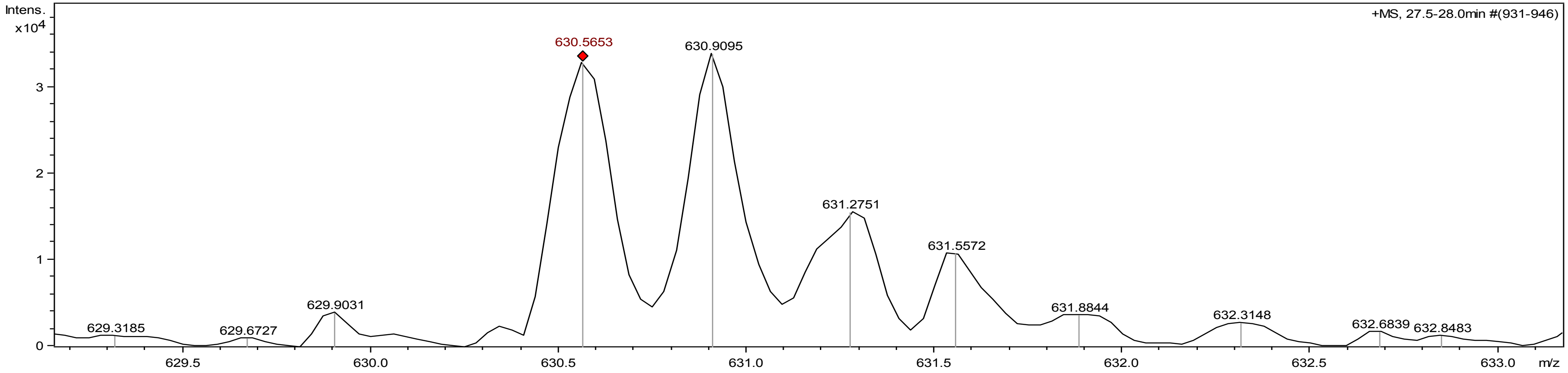

ETD spectrum of poor quality

Fraction 15

630.57+++ → Pep [M+H]+ 1233.58+ [27.5-28.5 min]   CID-MS2

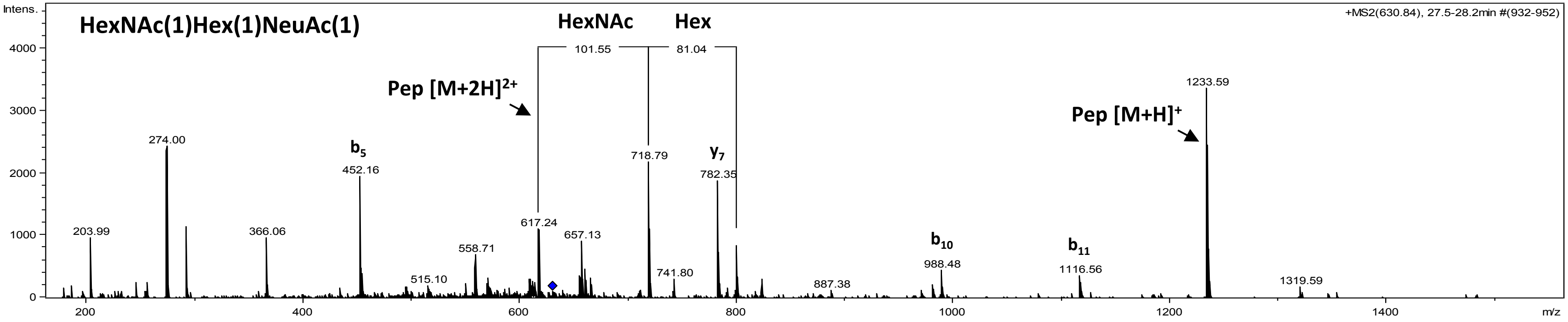

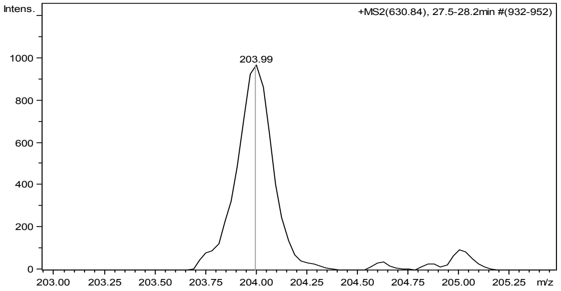

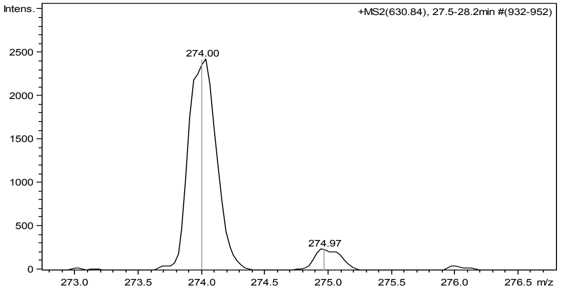

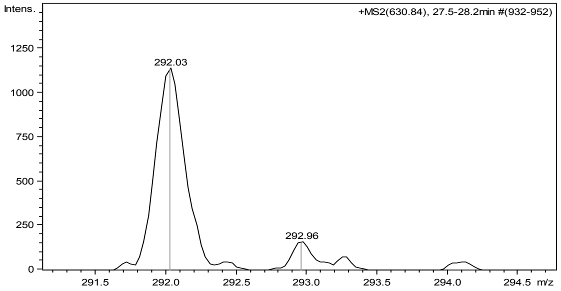

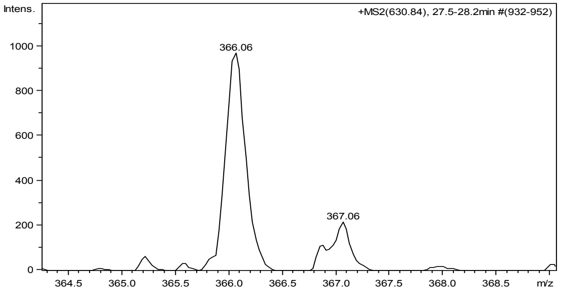

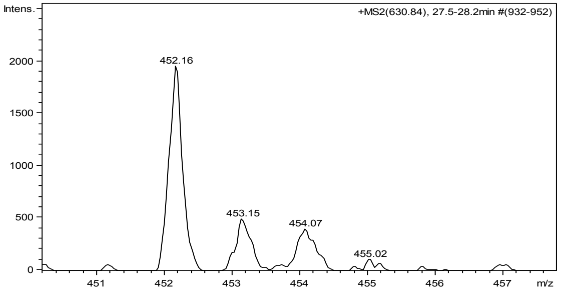

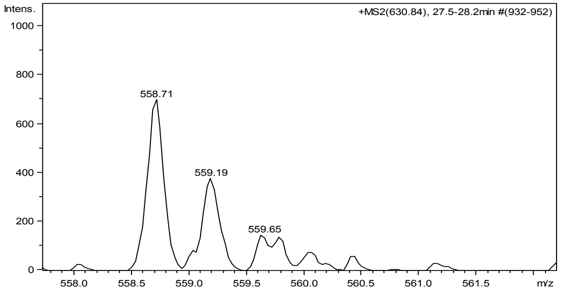

Fraction 15

630.57+++ → Pep [M+H]+ 1233.58+ [27.5-28.5 min]

CID-MS2

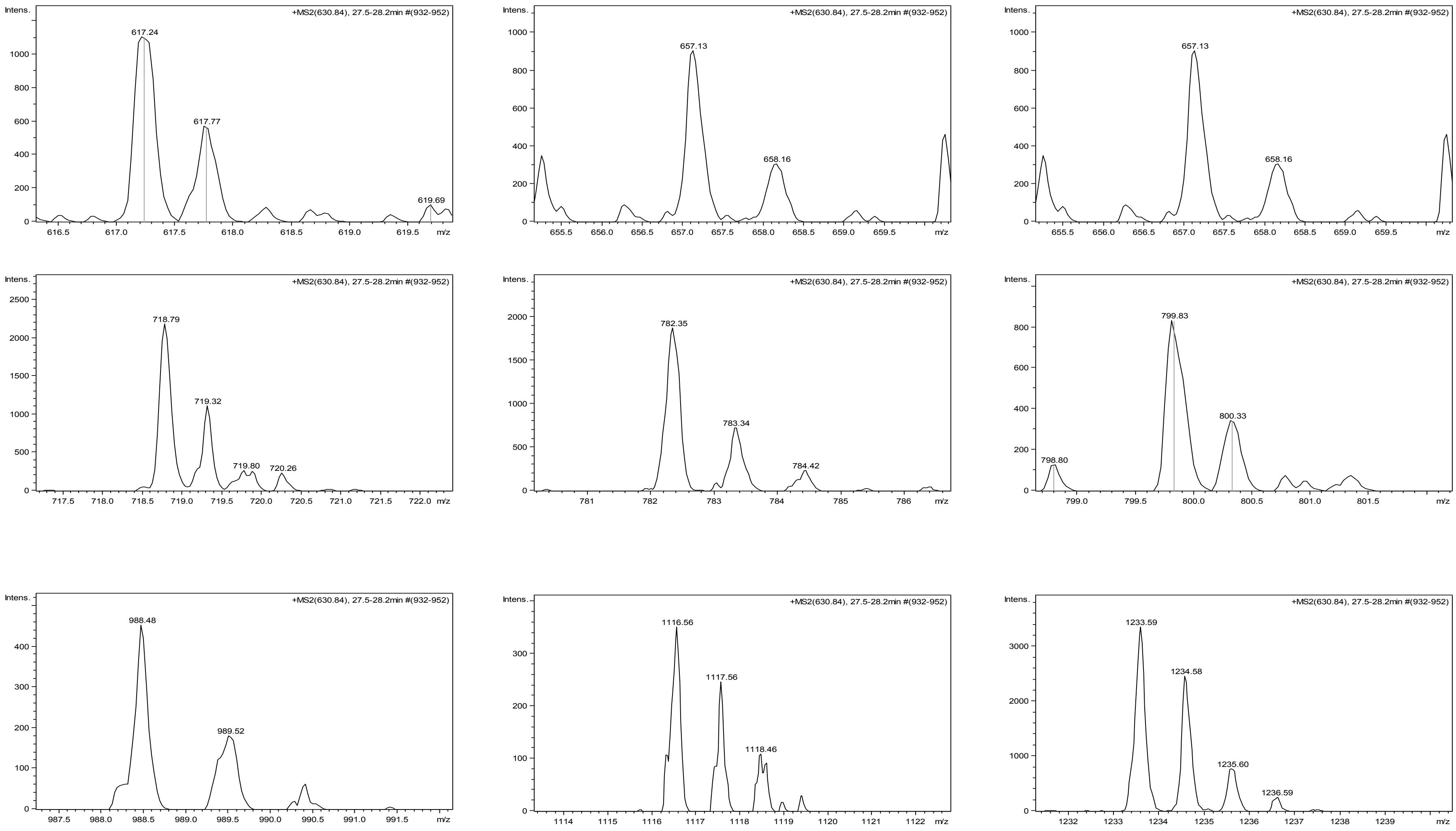

Fraction 15

630.57+++ → Pep [M+H]+ 1233.58+ [27.5-28.5 min] CID-MS3

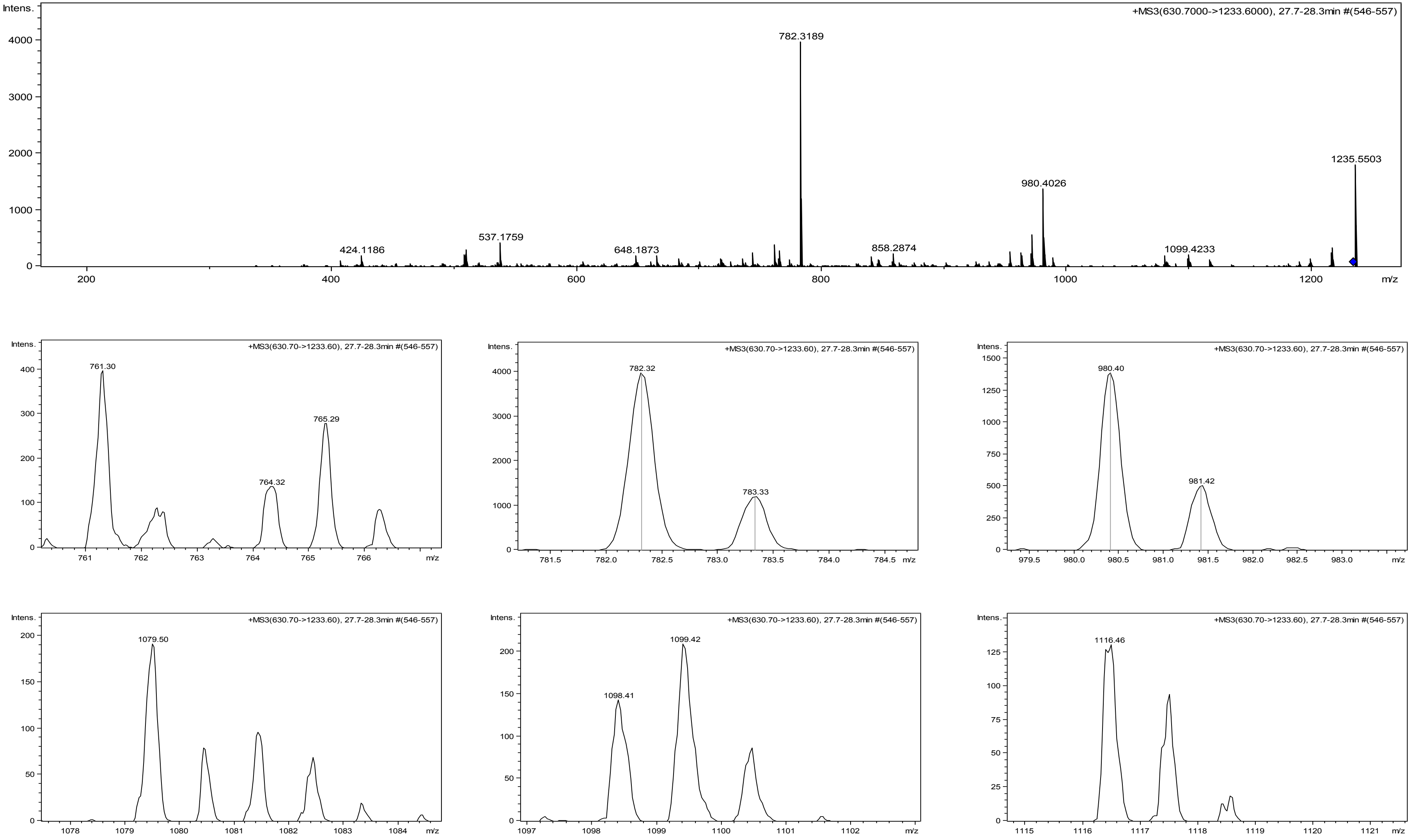

Fraction 15

630.57+++ → Pep [M+H]+ 1233.58+ [27.5-28.5 min] CID-MS3

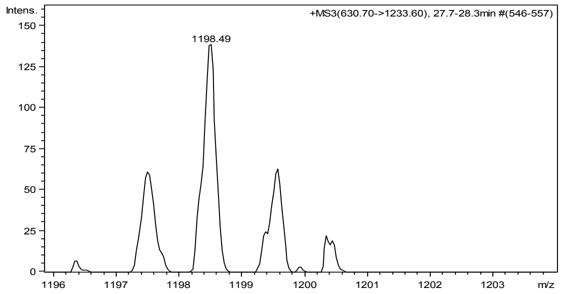

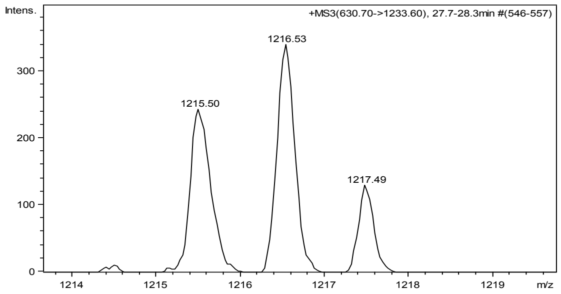

Fraction 15

630.57+++ → Pep [M+H]+ 1233.58+ [27.5-28.5 min]   CID-MS3   MASCOT Search

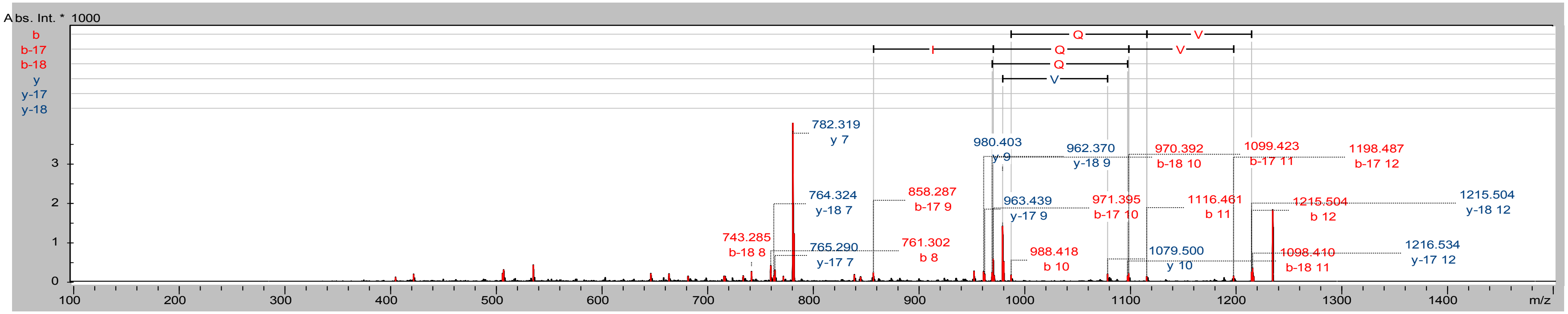

	G	P	V	P	T	P	P	D	N	I	Q	V	Gly	Pro	Val	Pro	Thr	Pro	Pro	Asp	Asn	Ile	Gln	Val
Ion	1	2	3	4	5	6	7	8	9	10	11	12	1	2	3	4	5	6	7	8	9	10	11	12
b	G	P	V	P	T	P	P	D	N	I	Q	V	58.029	155.082	254.150	351.203	452.250	549.303	646.356	761.383	875.426	988.510	1116.568	1215.637
b-17	G	P	V	P	T	P	P	D	N	I	Q	V	-	-	-	-	-	-	-	-	858.399	971.483	1099.542	1198.610
b-18	G	P	V	P	T	P	P	D	N	I	Q	V	-	-	-	-	434.240	531.293	628.345	743.372	857.415	970.499	1098.558	1197.626
y	G	P	V	P	T	P	P	D	N	I	Q	V	118.086	246.145	359.229	473.272	588.299	685.352	782.404	883.452	980.505	1079.573	1176.626	1233.647
y-17	G	P	V	P	T	P	P	D	N	I	Q	V	-	229.118	342.202	456.245	571.272	668.325	765.378	866.425	963.478	1062.547	1159.599	1216.621
y-18	G	P	V	P	T	P	P	D	N	I	Q	V	-	-	-	-	570.288	667.341	764.394	865.441	962.494	1061.563	1158.615	1215.637
	12	11	10	9	8	7	6	5	4	3	2	1	Val	Gln	Ile	Asn	Asp	Pro	Pro	Thr	Pro	Val	Pro	Gly

known O-glycosylation site

Protein AMBP

$_{20}$GPVP**T**PPDNIQV$_{31}$

Fraction 15

630.57+++ → Pep [M+H]+ 1233.58+ [27.5-28.5 min]

CID-MS3 MASCOT Search

prot_hit_nur	prot_acc	prot_desc	prot_score	prot_mass	prot_matche	pep_query	pep_rank	pep_isbold	pep_exp_mz	pep_exp_mr	pep_exp_z	pep_calc_mr	pep_delta	pep_miss	pep_score	pep_expect	pep_res_bef	pep_seq
1	AMBP_HUMA	AMBP protei	13	39886	1	1	1	1	1233.5807	1232.5734	1	1232.6401	-0.0667	0	18.26	2.70E+02	A	GPVPTPPDN
2	GAB2_HUMA	GRB2-associa	6	74925	1	1	2	0	1233.5807	1232.5734	1	1231.5681	1.0054	0	12.17	1.10E+03	G	HTKGSLTGSE
3	ERG25_HUMA	C-4 methylst	6	35592	1	1	4	0	1233.5807	1232.5734	1	1231.508	1.0654	0	9.41	2.10E+03	H	DFHHMNFIG
4	VACHT_HUM	Vesicular ace	4	57324	1	1	7	0	1233.5807	1232.5734	1	1232.7063	-0.1329	0	8.63	2.50E+03	L	PVGTPIHRLM
5	MUML1_HUM	MUM1-like p	3	80073	1	1	5	0	1233.5807	1232.5734	1	1232.5383	0.0351	0	9.39	2.10E+03	G	CGSFTGSLLEY
6	RABX5_HUM	Rab5 GDP/GT	2	80575	1	1	9	0	1233.5807	1232.5734	1	1232.6401	-0.0666	0	8.24	2.70E+03	P	PRLQSNIQYI
7	FA5_HUMAN	Coagulation	2	252654	1	1	3	0	1233.5807	1232.5734	1	1232.5747	-0.0013	0	10.25	1.70E+03	S	SSLMYTVNGY
8	ROBO2_HUM	Roundabout	1	151961	1	1	6	0	1233.5807	1232.5734	1	1231.6673	0.9061	0	9.18	2.20E+03	D	LPRGRYDIKD
9	YLPM1_HUM	YLP motif-co	0	220077	1	1	8	0	1233.5807	1232.5734	1	1232.6149	-0.0415	0	8.28	2.70E+03	K	PVGIGLPHSE

Biotools-Score: 9

MASCOT-Score: 18

known O-glycosylation site

Protein AMBP

$_{20}$GPVP**T**PPDNIQV$_{31}$

Fraction 15

775.67+++ → Pep [M+H]+ 1668.84+ [30.2-30.4 min] CID-MS Precursor

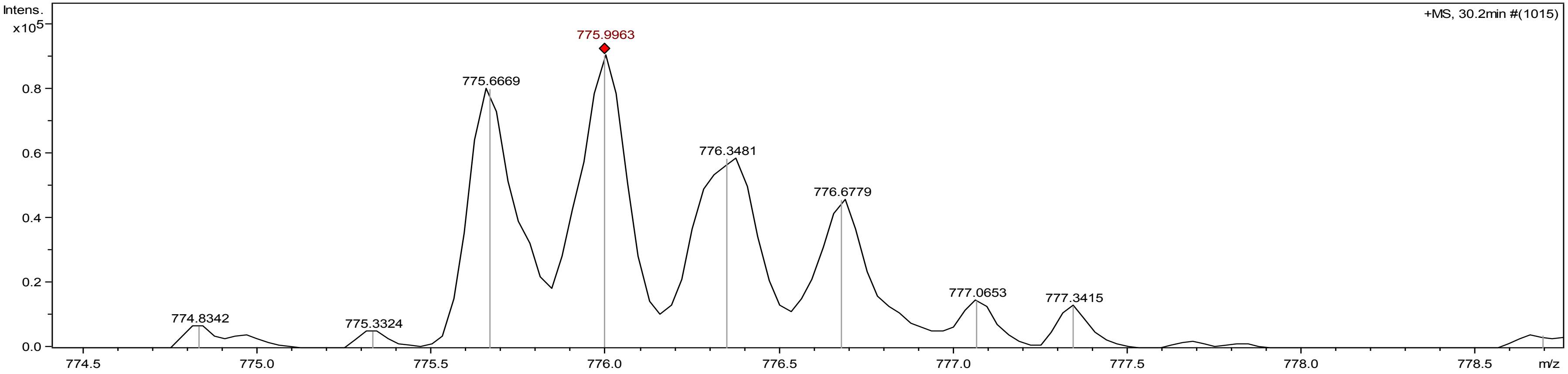

Fraction 15

775.67+++ → Pep [M+H]+ 1668.84+ [30.2-30.4 min] CID-MS2

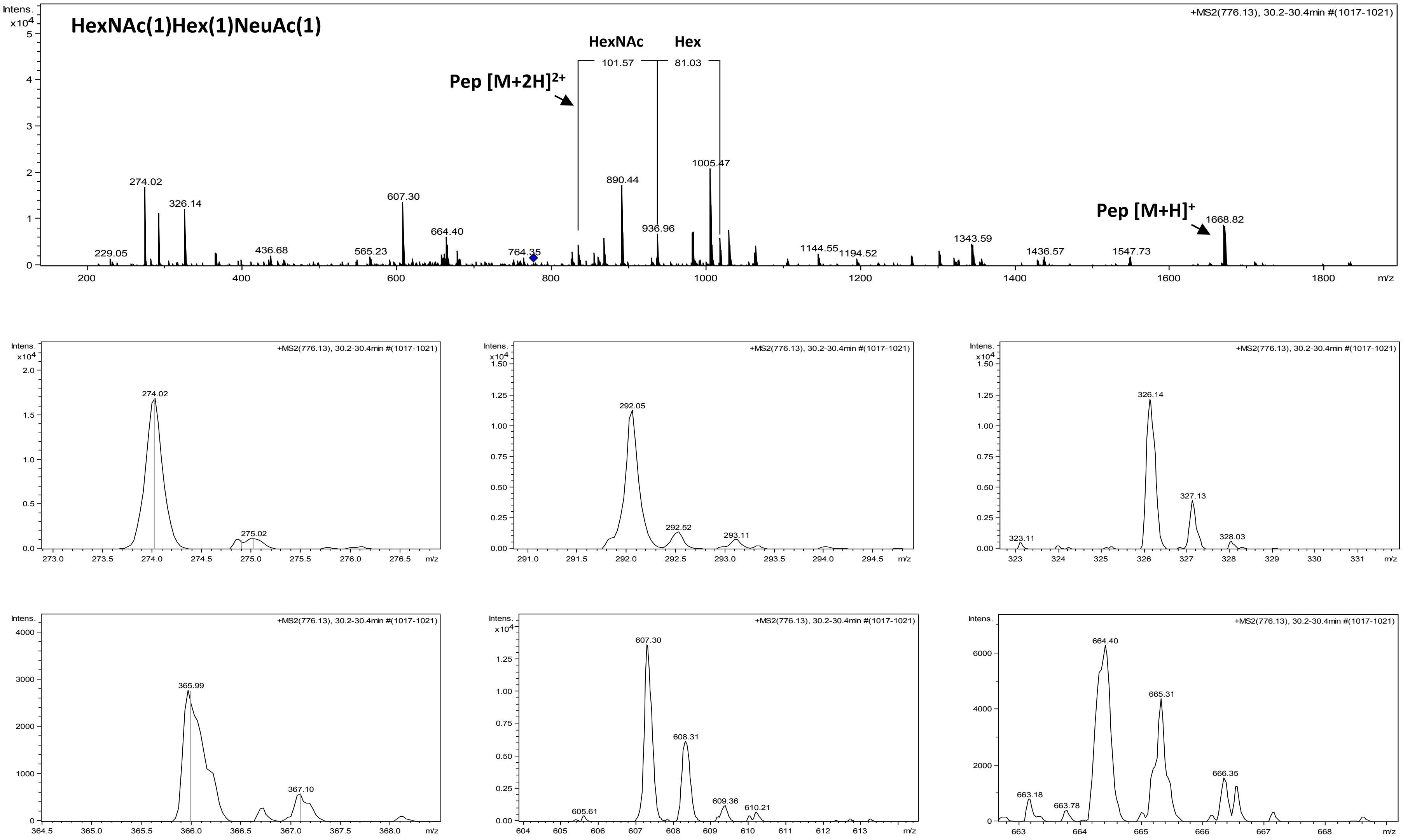

Fraction 15

775.67+++ → Pep [M+H]+ 1668.84+ [30.2-30.4 min] CID-MS2

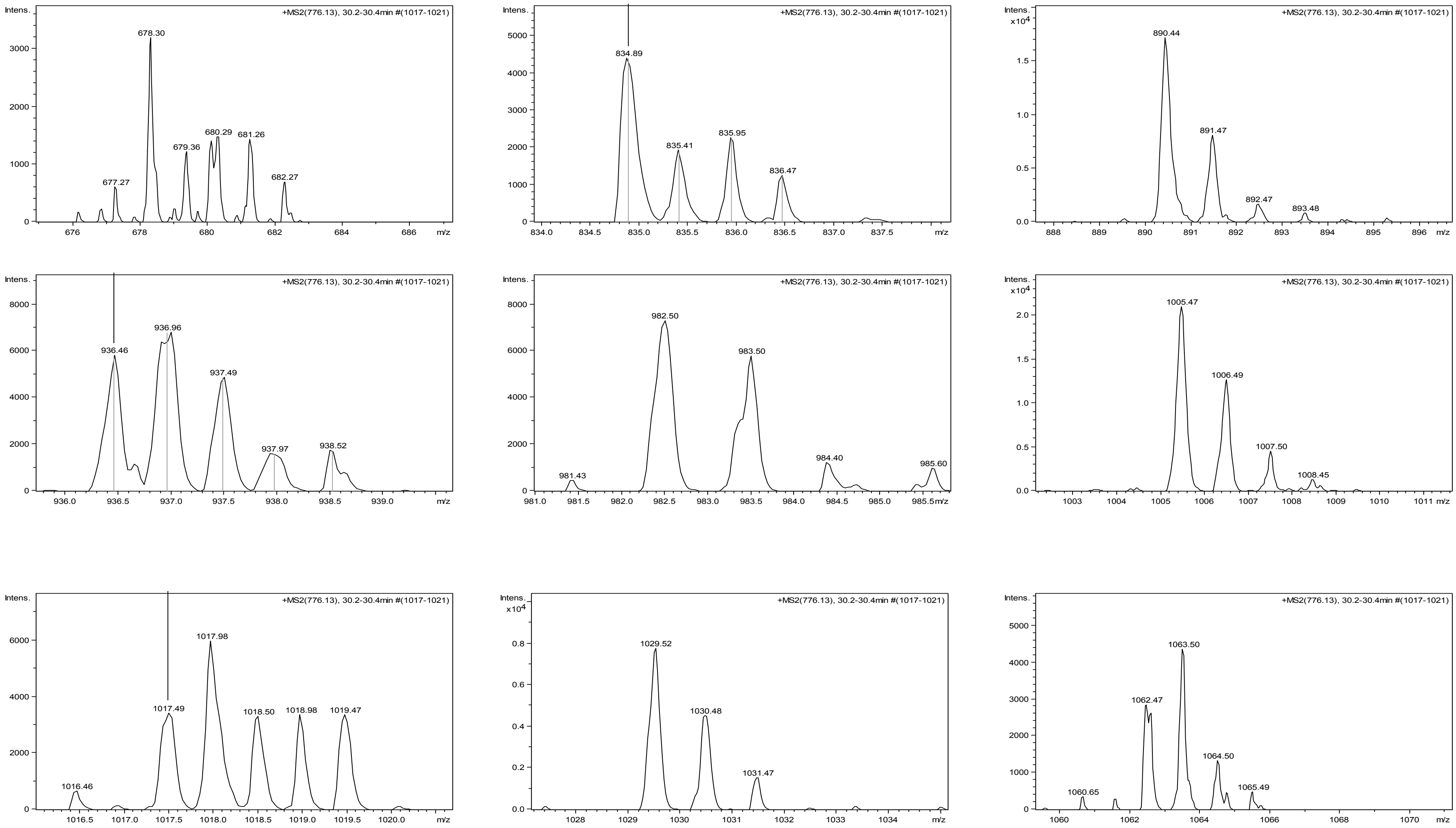

Fraction 15

775.67+++ → Pep [M+H]+ 1668.84+ [30.2-30.4 min]

CID-MS2

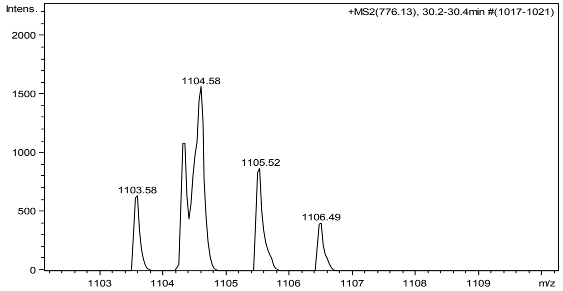

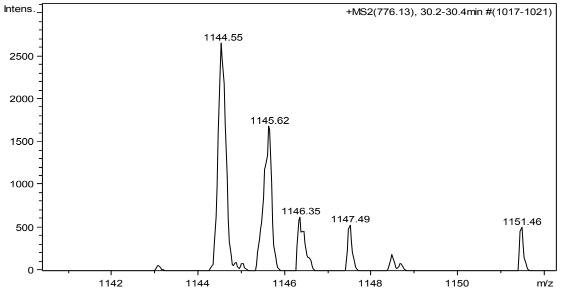

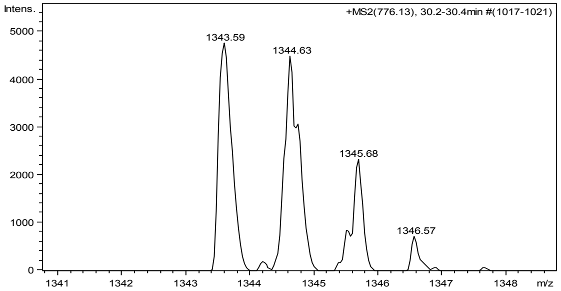

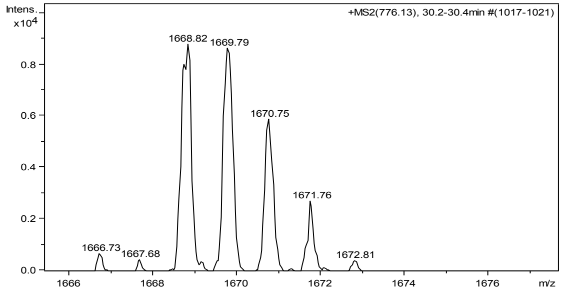

Fraction 15

775.67+++ → Pep [M+H]+ 1668.84+ [30.2-30.4 min]

CID-MS2 MASCOT Search

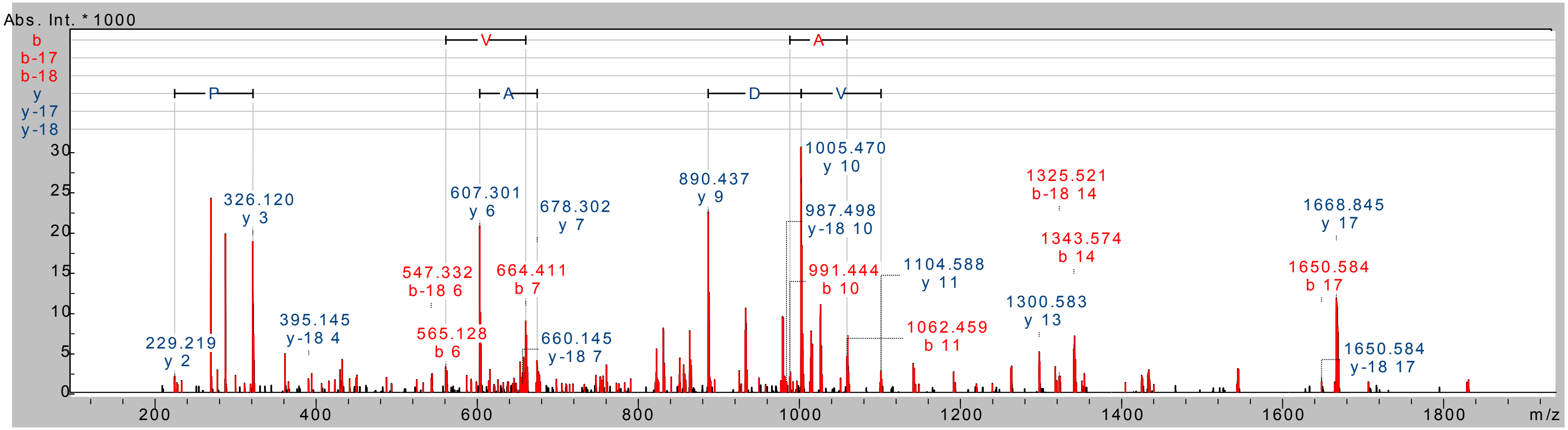

	A	V	P	T	P	V	V	D	P	D	A	P	P	S	P	P	L
Ion	1	2	3	4	5	6	7	8	9	10	11	12	13	14	15	16	17
b	A	V	P	T	P	V	V	D	P	D	A	P	P	S	P	P	L
b-17	A	V	P	T	P	V	V	D	P	D	A	P	P	S	P	P	L
b-18	A	V	P	T	P	V	V	D	P	D	A	P	P	S	P	P	L
y	A	V	P	T	P	V	V	D	P	D	A	P	P	S	P	P	L
y-17	A	V	P	T	P	V	V	D	P	D	A	P	P	S	P	P	L
y-18	A	V	P	T	P	V	V	D	P	D	A	P	P	S	P	P	L
	17	16	15	14	13	12	11	10	9	8	7	6	5	4	3	2	1

	Ala	Val	Pro	Thr	Pro	Val	Val	Asp	Pro	Asp	Ala	Pro	Pro	Ser	Pro	Pro	Leu
Ion	1	2	3	4	5	6	7	8	9	10	11	12	13	14	15	16	17
b	72.044	171.113	268.166	369.213	466.266	565.334	664.403	779.430	876.483	991.509	1062.547	1159.599	1256.652	1343.684	1440.737	1537.790	1650.874
b-17	-	-	-	-	-	-	-	-	-	-	-	-	-	-	-	-	-
b-18	-	-	-	351.203	448.255	547.324	646.392	761.419	858.472	973.499	1044.536	1141.589	1238.642	1325.674	1422.726	1519.779	1632.863
y	132.102	229.155	326.207	413.239	510.292	607.345	678.382	793.409	890.462	1005.489	1104.557	1203.626	1300.678	1401.726	1498.779	1597.847	1668.884
y-17	-	-	-	-	-	-	-	-	-	-	-	-	-	-	-	-	-
y-18	-	-	-	395.229	492.282	589.334	660.372	775.398	872.451	987.478	1086.547	1185.615	1282.668	1383.715	1480.768	1579.837	1650.874
	Leu	Pro	Pro	Ser	Pro	Pro	Ala	Asp	Pro	Asp	Val	Val	Pro	Thr	Pro	Val	Ala

For MASCOT search m/z of the unmodified peptide [M+H]$^{+}$ has to be given

known O-glycosylation site

Alpha-2-HS-glycoprotein precursor

$_{267}$AVP**T**PVVDPDAPPSPPL$_{283}$

Fraction 15

775.67+++ → Pep [M+H]+ 1668.84+ [30.2-30.4 min]

CID-MS2 MASCOT Search

prot_hit_nur	prot_acc	prot_desc	prot_score	prot_mass	prot	prot	prot	prot	pep	pep	pep	pep	pep_exp_mz	pep_exp_mr	pep_	pep_calc_mr	pep_delta	pep	pep_score	pep_expect	pep_	pep_seq
1	FDXA1_HUM	Ferredoxin-f	10	71625	1	0	1	0	1	1	1	1	1668,84	1667,8327	1	1666,9141	0,9186	0	16,7	5,80E+02	L	LDHLKGILDSLLTQT
2	FETUA_HUM	Alpha-2-HS-g	3	40098	1	0	1	0	1	5	0	1	1668,84	1667,8327	1	1667,8771	-0,0443	0	8,31	4,00E+03	E	AVPTPVVDPDAPPSPPL
3	MAP11_HUMAN		2	44100	1	0	1	0	1	7	0	1	1668,84	1667,8327	1	1667,9107	-0,078	0	5,75	7,20E+03	N	IIQKHAQANGFSVVR
4	CAPON_HUMAN		2	56457	1	0	1	0	1	4	0	1	1668,84	1667,8327	1	1667,7196	0,1131	0	8,51	3,80E+03	L	SGQNAMGSQDSLLEIT
5	USO1_HUMAN		1	108740	1	0	1	0	1	2	0	1	1668,84	1667,8327	1	1666,8712	0,9615	0	8,93	3,50E+03	L	SKKYRLEVGIQAME
6	TTC13_HUMAN		1	97607	1	0	1	0	1	3	0	1	1668,84	1667,8327	1	1667,7679	0,0649	0	8,52	3,80E+03	L	IEDYEEQPGLQPHI
7	FCN1_HUMAN		0	35512	1	0	1	0	1	8	0	1	1668,84	1667,8327	1	1667,8526	-0,0198	0	4,81	9,00E+03	K	AGPVGPKGDRGEKGMRG
8	ZSWM5_HUMAN		0	132318	1	0	1	0	1	9	0	1	1668,84	1667,8327	1	1667,7905	0,0422	0	4,8	9,00E+03	E	MLRMRDSNGARMLT
9	DYH11_HUMAN		0	524873	1	0	1	0	1	9	0	1	1668,84	1667,8327	1	1667,8916	-0,0589	0	4,8	9,00E+03	L	LGELPPGDRQKIMTI

BioTools-Score: 25

MASCOT-Score: 8

known O-glycosylation site

Alpha-2-HS-glycoprotein precursor

$_{267}$AVP**T**PVVDPDAPPSPPL$_{283}$

Fraction 15

775.67+++ → Pep [M+H]+ 1668.84+ [30.2-30.4 min]

CID-MS2 MASCOT Search

Internal glycopeptide fragmentation

$_{267}$AVPTPVVDPDAPPSPPL$_{283}$

HexNac-

b				y
---	1	A	17	---
171.11	2	V	16	1800.93
268.17	3	P	15	1701.86
572.29	4	T(HexNac)	14	1604.81
669.35	5	P	13	1300.68
768.41	6	V	12	1203.63
867.48	7	V	11	1104.56
982.51	8	D	10	1005.49
1079.56	9	P	9	890.46
1194.59	10	D	8	793.41
1265.63	11	A	7	678.38
1362.68	12	P	6	607.35
1459.73	13	P	5	510.29
1546.76	14	S	4	413.24
1643.82	15	P	3	326.21
1740.87	16	P	2	229.15
---	17	L	1	132.10

These fragment ions are not present in the MS³ peptide spectrum. This indicates that they are derived from glycopeptide fragmentation.

known O-glycosylation site

Alpha-2-HS-glycoprotein precursor

$_{267}$AVPTPVVDPDAPPSPPL$_{283}$

Fraction 15

775.67+++ → Pep [M+H]+ 1668.84+ [30.2-30.4 min] CID-MS3

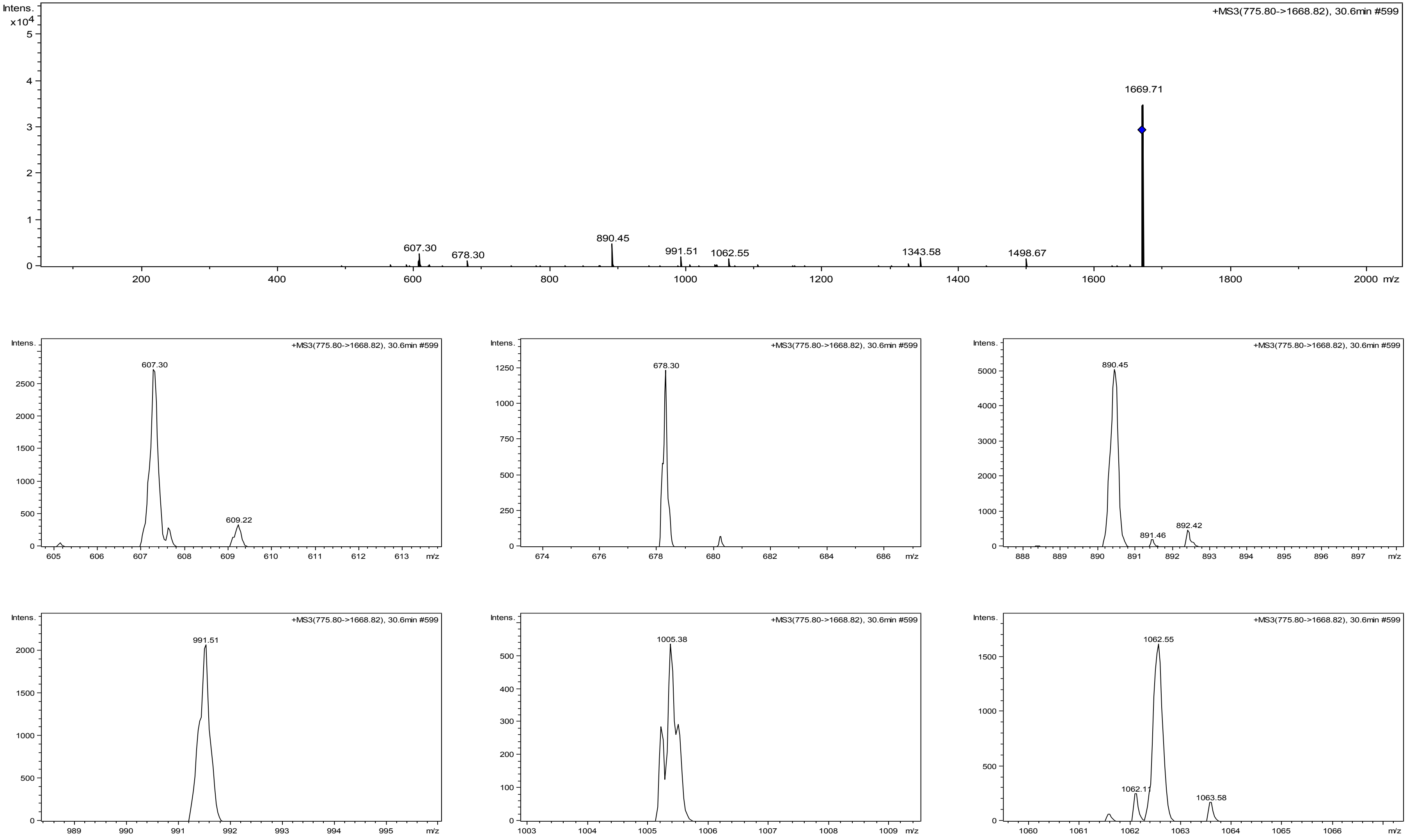

Fraction 15

775.67+++ → Pep [M+H]+ 1668.84+ [30.2-30.4 min] CID-MS3

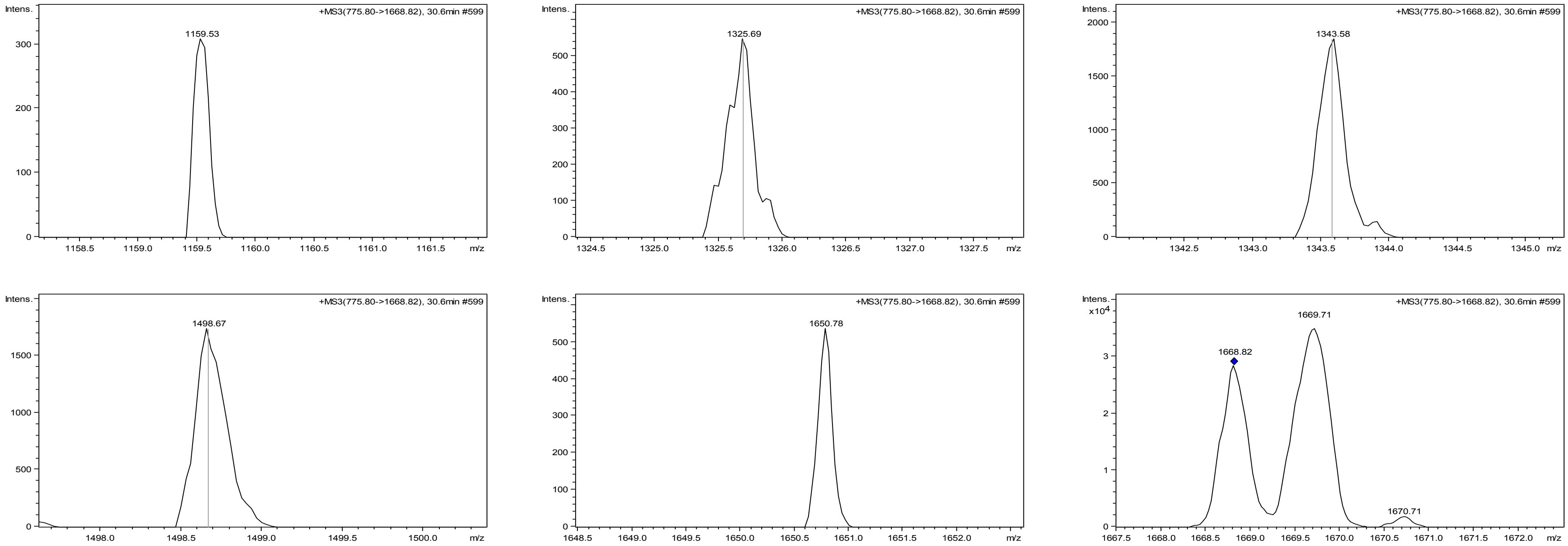

Fraction 15

775.67+++ → Pep [M+H]+ 1668.84+ [30.2-30.4 min]

CID-MS3 MASCOT Search

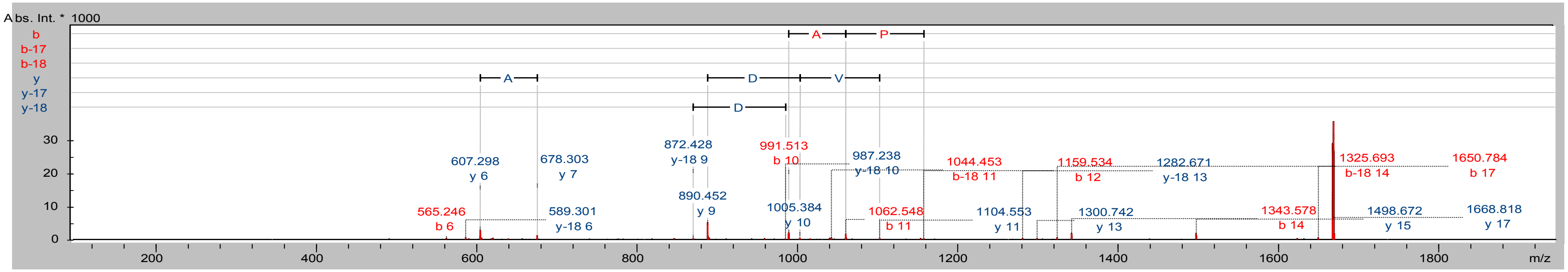

	A	V	P	T	P	V	V	D	P	D	A	P	P	S	P	P	L	Ala	Val	Pro	Thr	Pro	Val	Val	Asp	Pro	Asp	Ala	Pro	Pro	Ser	Pro	Pro	Leu
Ion	1	2	3	4	5	6	7	8	9	10	11	12	13	14	15	16	17	1	2	3	4	5	6	7	8	9	10	11	12	13	14	15	16	17
b	A	V	P	T	P	V	V	D	P	D	A	P	P	S	P	P	L	72.044	171.113	268.166	369.213	466.266	565.334	664.403	779.430	876.483	991.509	1062.547	1159.599	1256.652	1343.684	1440.737	1537.790	1650.874
b-17	A	V	P	T	P	V	V	D	P	D	A	P	P	S	P	P	L	-	-	-	-	-	-	-	-	-	-	-	-	-	-	-	-	-
b-18	A	V	P	T	P	V	V	D	P	D	A	P	P	S	P	P	L	-	-	-	351.203	448.255	547.324	646.392	761.419	858.472	973.499	1044.536	1141.589	1238.642	1325.674	1422.726	1519.779	1632.863
y	A	V	P	T	P	V	V	D	P	D	A	P	P	S	P	P	L	132.102	229.155	326.207	413.239	510.292	607.345	678.382	793.409	890.462	1005.489	1104.557	1203.626	1300.678	1401.726	1498.779	1597.847	1668.884
y-17	A	V	P	T	P	V	V	D	P	D	A	P	P	S	P	P	L	-	-	-	-	-	-	-	-	-	-	-	-	-	-	-	-	-
y-18	A	V	P	T	P	V	V	D	P	D	A	P	P	S	P	P	L	-	-	-	395.229	492.282	589.334	660.372	775.398	872.451	987.478	1086.547	1185.615	1282.668	1383.715	1480.768	1579.837	1650.874
	17	16	15	14	13	12	11	10	9	8	7	6	5	4	3	2	1	Leu	Pro	Pro	Ser	Pro	Pro	Ala	Asp	Pro	Asp	Val	Val	Pro	Thr	Pro	Val	Ala

known O-glycosylation site

Alpha-2-HS-glycoprotein precursor

$_{267}$AVP**T**PVVDPDAPPSPPL$_{283}$

Fraction 15

775.67+++ → Pep [M+H]+ 1668.84+ [30.2-30.4 min]

CID-MS3 MASCOT Search

prot_hit_nur	prot_acc	prot_desc	prot_score	prot_mass	prot_matche	pep_query	pep_rank	pep_isbold	pep_exp_mz	pep_exp_mr	pep_exp_z	pep_calc_mr	pep_delta	pep_miss	pep_score	pep_expect	pep_res_bef	pep_seq
1	FETUA_HUM	Alpha-2-HS-g	48	40098	1	1	1	1	1668.8449	1667.8376	1	1667.8771	-0.0394	0	53.68	0.089	E	AVPTPVVDP
2	TMM29_HUN	Transmembr	19	24567	1	1	3	0	1668.8449	1667.8376	1	1667.9471	-0.1095	0	22.85	1.10E+02	Q	AVPLPEGLLR
3	PI3R6_HUMA	Phosphoinos	18	85060	1	1	2	0	1668.8449	1667.8376	1	1667.8777	-0.0401	0	23.66	90	L	HTARVLVLGD
4	MYL4_HUMA	Myosin light	17	21665	1	1	5	0	1668.8449	1667.8376	1	1667.9028	-0.0652	0	20.91	1.70E+02	G	AELRHVLATL
5	YIPF1_HUMA	Protein YIPF	17	34426	1	1	4	0	1668.8449	1667.8376	1	1667.8745	-0.0369	0	20.94	1.70E+02	P	FWICATLVFA
6	GDF11_HUM	Growth/diffe	16	45803	1	1	10	0	1668.8449	1667.8376	1	1666.688	1.1497	0	20.79	1.70E+02	V	ISMAQETDPA
7	LR37A_HUM	Leucine-rich	12	181649	1	1	5	0	1668.8449	1667.8376	1	1667.6468	0.1908	0	20.91	1.70E+02	M	ENTNMPEGT

Biotools-Score: 20

MASCOT-Score: 54

known O-glycosylation site

Alpha-2-HS-glycoprotein precursor

$_{267}$AVPTPVVDPDAPPSPPL$_{283}$

Fraction 15

775.67+++ → Pep [M+H]+ 1668.84+ [30.2-30.4 min] ETD

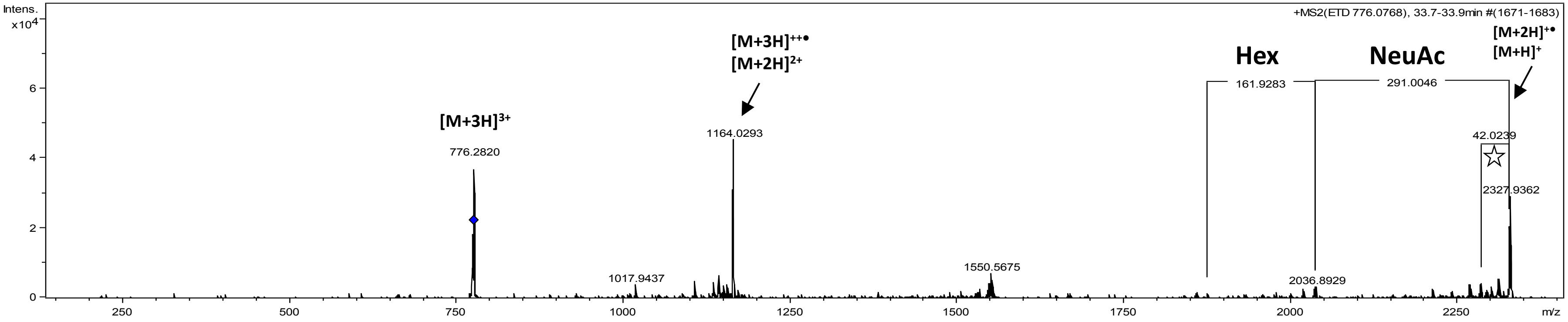

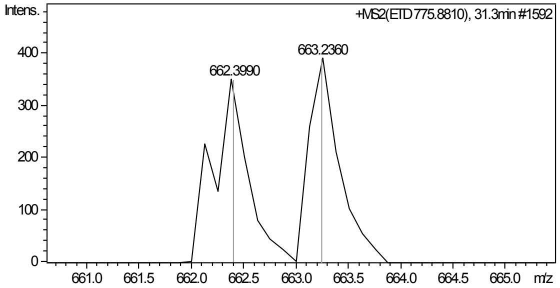

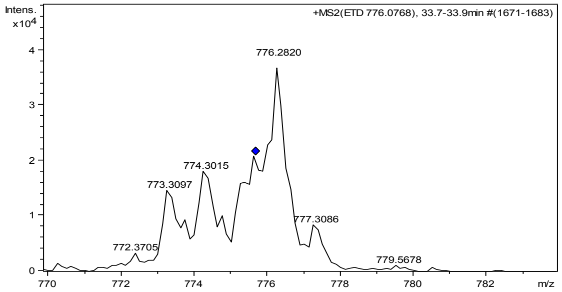

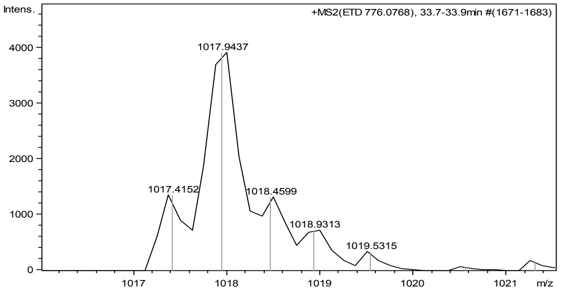

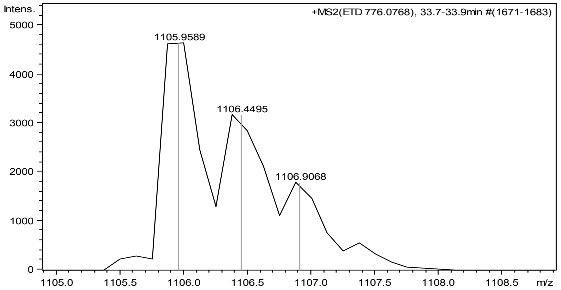

known O-glycosylation site

Alpha-2-HS-glycoprotein precursor $_{267}$AVPTPVVDPDAPPSPPL$_{283}$

Fraction 15

775.67+++ → Pep [M+H]+ 1668.84+ [30.2-30.4 min]

ETD

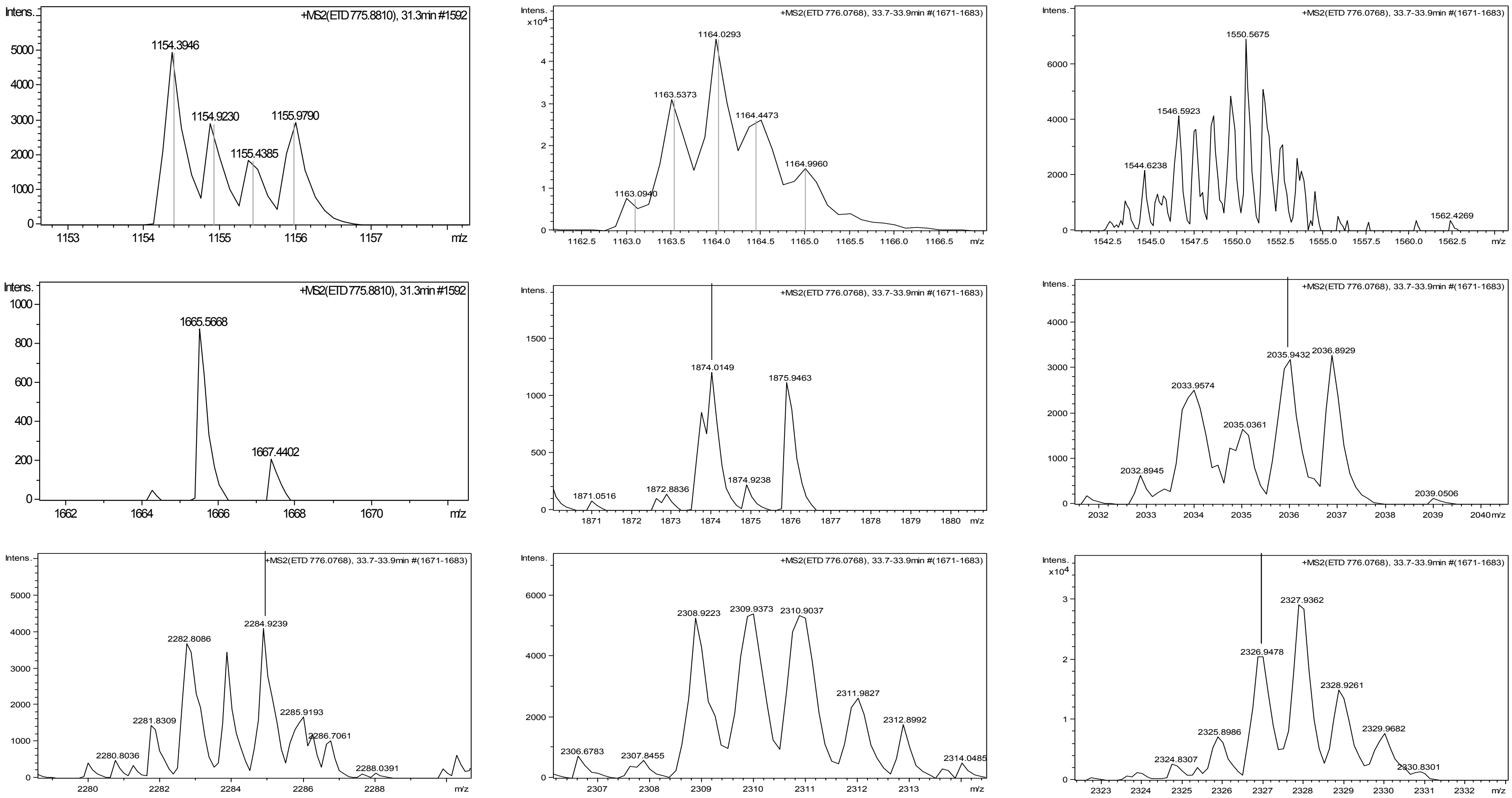

known O-glycosylation site

Alpha-2-HS-glycoprotein precursor

$_{267}$AVP**T**PVVDPDAPPSPPL$_{283}$

Fraction 15

775.67+++ → Pep [M+H]+ 1668.84+ [30.2-30.4 min] ETD

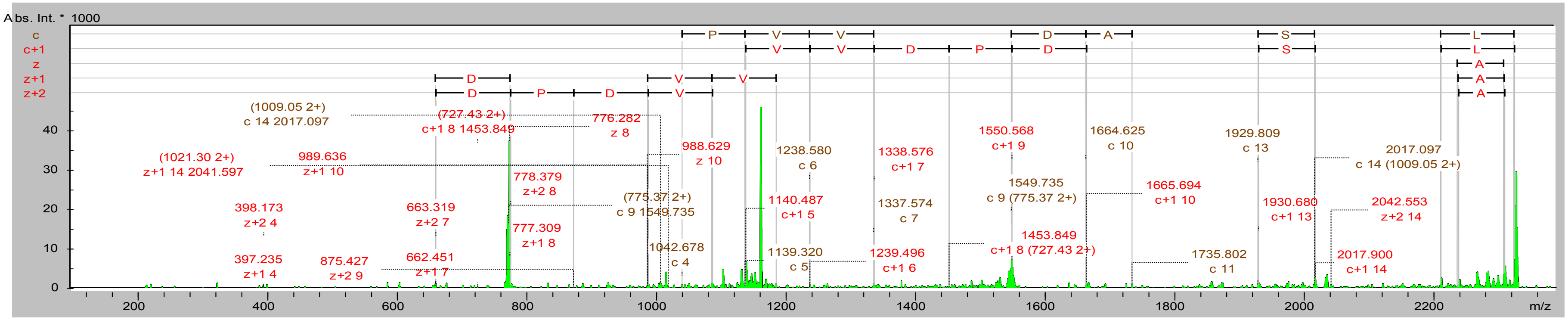

AVP**T**PVVDPDAPPSPPL

	A	V	P	T	P	V	V	D	P	D	A	P	P	S	P	P	L
Ion	1	2	3	4	5	6	7	8	9	10	11	12	13	14	15	16	17
c	A	V	P	T*	P	V	V	D	P	D	A	P	P	S	P	P	L
c+1	A	V	P	T*	P	V	V	D	P	D	A	P	P	S	P	P	L
z	A	V	P	T*	P	V	V	D	P	D	A	P	P	S	P	P	L
z+1	A	V	P	T*	P	V	V	D	P	D	A	P	P	S	P	P	L
z+2	A	V	P	T*	P	V	V	D	P	D	A	P	P	S	P	P	L
	17	16	15	14	13	12	11	10	9	8	7	6	5	4	3	2	1

	Ala	Val	Pro	Thr	Pro	Val	Val	Asp	Pro	Asp	Ala	Pro	Pro	Ser	Pro	Pro	Leu
Ion	1	2	3	4	5	6	7	8	9	10	11	12	13	14	15	16	17
c	89.071	188.139	285.192	1042.467	1139.520	1238.589	1337.657	1452.684	1549.737	1664.764	1735.801	1832.854	1929.906	2016.938	2113.991	2211.044	2324.128
c+1	90.079	189.147	286.200	1043.475	1140.528	1239.596	1338.665	1453.692	1550.745	1665.771	1736.809	1833.861	1930.914	2017.946	2114.999	2212.052	2325.136
z	115.075	212.128	309.181	396.213	493.266	590.318	661.356	776.382	873.435	988.462	1087.531	1186.599	1283.652	2040.927	2137.980	2237.048	2308.085
z+1	116.083	213.136	310.189	397.221	494.273	591.326	662.363	777.390	874.443	989.470	1088.538	1187.607	1284.660	2041.935	2138.988	2238.056	2309.093
z+2	117.091	214.144	311.197	398.229	495.281	592.334	663.371	778.398	875.451	990.478	1089.546	1188.615	1285.667	2042.943	2139.995	2239.064	2310.101
	Leu	Pro	Pro	Ser	Pro	Pro	Ala	Asp	Pro	Asp	Val	Val	Pro	Thr	Pro	Val	Ala

Biotools-Score: 153

known O-glycosylation site

Alpha-2-HS-glycoprotein precursor

$_{267}$AVP**T**PVVDPDAPPSPPL$_{283}$

Fraction 15

775.67+++ → Pep [M+H]+ 1668.84+ [30.2-30.4 min] ETD

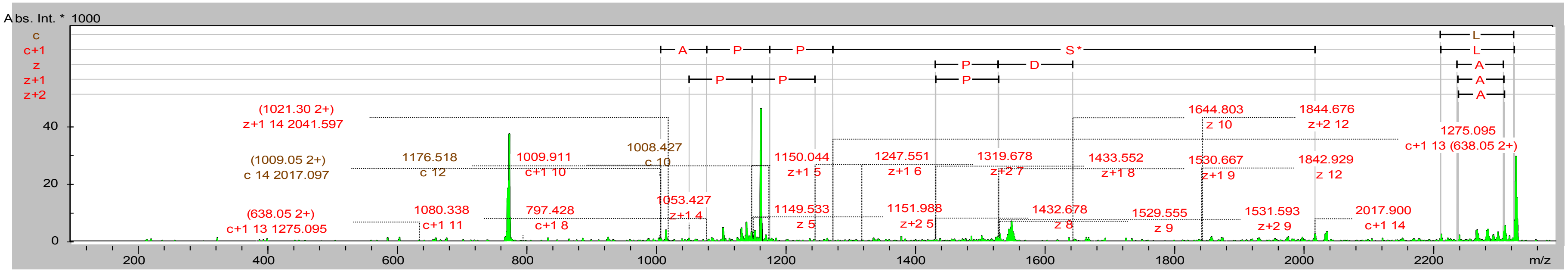

AVPTPVVDPDAPP**S**PPL

	A	V	P	T	P	V	V	D	P	D	A	P	P	S	P	P	L
Ion	1	2	3	4	5	6	7	8	9	10	11	12	13	14	15	16	17
c	A	V	P	T	P	V	V	D	P	D	A	P	P	S*	P	P	L
c+1	A	V	P	T	P	V	V	D	P	D	A	P	P	S*	P	P	L
z	A	V	P	T	P	V	V	D	P	D	A	P	P	S*	P	P	L
z+1	A	V	P	T	P	V	V	D	P	D	A	P	P	S*	P	P	L
z+2	A	V	P	T	P	V	V	D	P	D	A	P	P	S*	P	P	L
	17	16	15	14	13	12	11	10	9	8	7	6	5	4	3	2	1

	Ala	Val	Pro	Thr	Pro	Val	Val	Asp	Pro	Asp	Ala	Pro	Pro	Ser	Pro	Pro	Leu
Ion	1	2	3	4	5	6	7	8	9	10	11	12	13	14	15	16	17
c	89.071	188.139	285.192	386.240	483.293	582.361	681.429	796.456	893.509	1008.536	1079.573	1176.626	1273.679	2016.938	2113.991	2211.044	2324.128
c+1	90.079	189.147	286.200	387.248	484.300	583.369	682.437	797.464	894.517	1009.544	1080.581	1177.634	1274.687	2017.946	2114.999	2212.052	2325.136
z	115.075	212.128	309.181	1052.441	1149.493	1246.546	1317.583	1432.610	1529.663	1644.690	1743.758	1842.827	1939.879	2040.927	2137.980	2237.048	2308.085
z+1	116.083	213.136	310.189	1053.448	1150.501	1247.554	1318.591	1433.618	1530.671	1645.698	1744.766	1843.834	1940.887	2041.935	2138.988	2238.056	2309.093
z+2	117.091	214.144	311.197	1054.456	1151.509	1248.562	1319.599	1434.626	1531.679	1646.705	1745.774	1844.842	1941.895	2042.943	2139.995	2239.064	2310.101
	Leu	Pro	Pro	Ser	Pro	Pro	Ala	Asp	Pro	Asp	Val	Val	Pro	Thr	Pro	Val	Ala

Biotools-Score: 75

known O-glycosylation site

Alpha-2-HS-glycoprotein precursor

$_{267}$AVP**T**PVVDPDAPPSPPL$_{283}$

Fraction 15

550.86+++ → Pep [M+2H]++ 497.70++ [14.0-14.7 min]

CID-MS Precursor

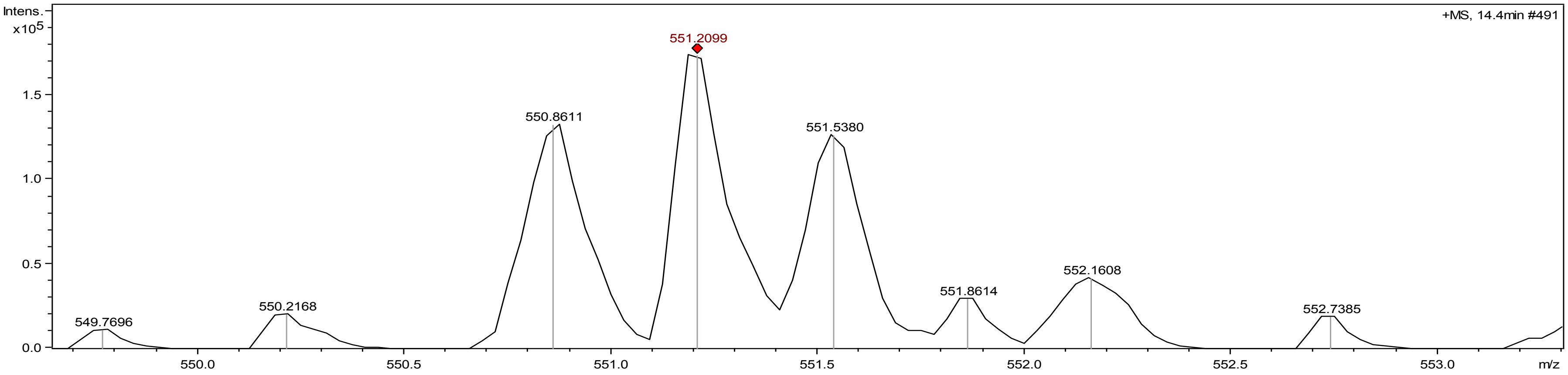

Fraction 15

550.86+++ → Pep [M+2H]++ 497.70++ [14.0-14.7 min] CID-MS2

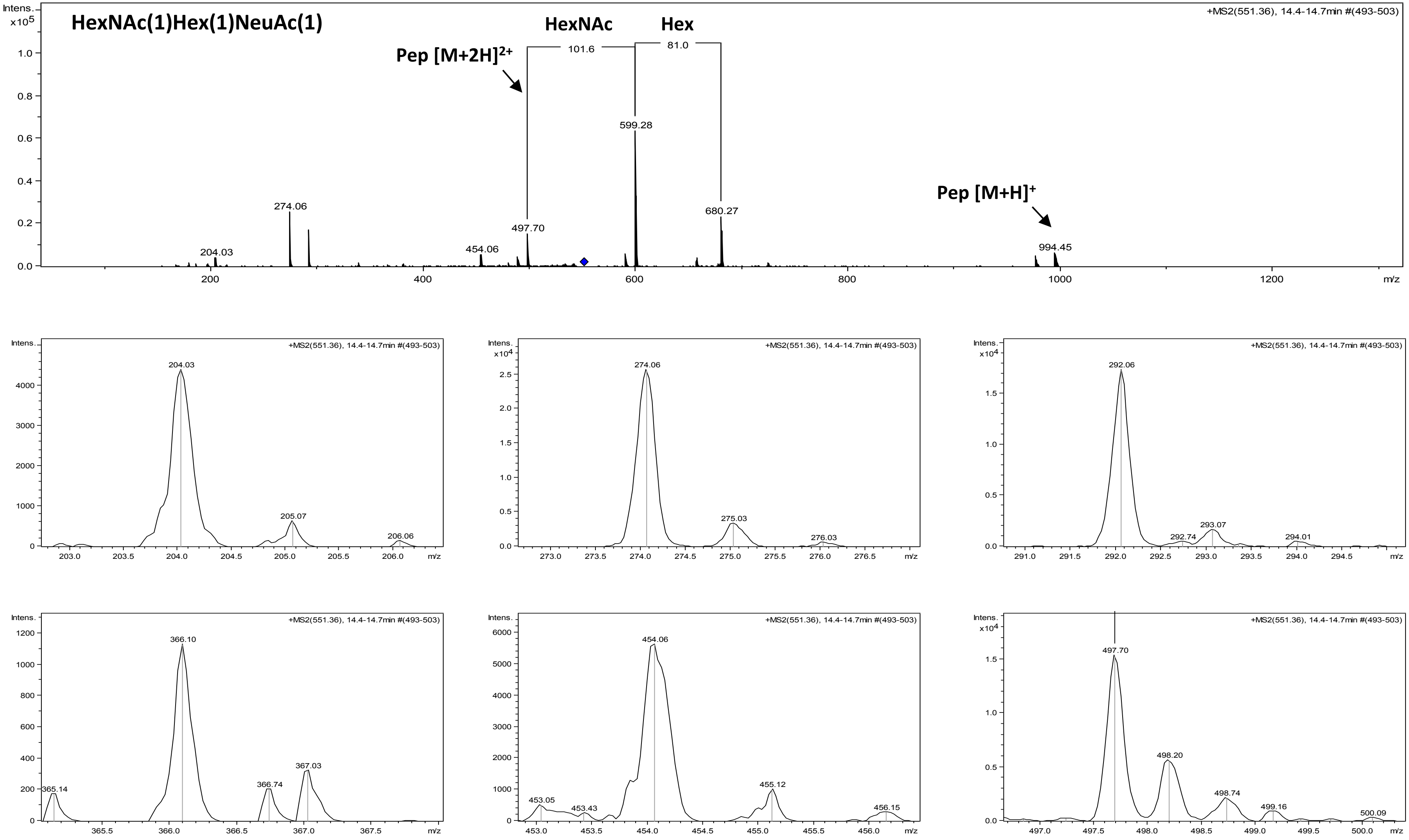

Fraction 15

550.86+++ → Pep [M+2H]++ 497.70++ [14.0-14.7 min] CID-MS2

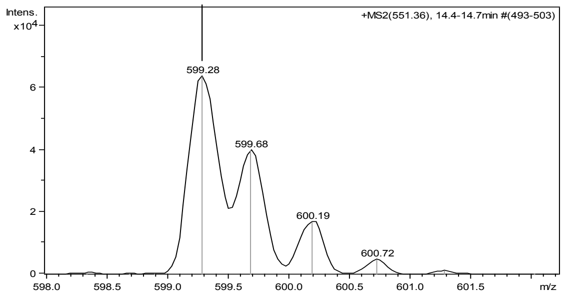

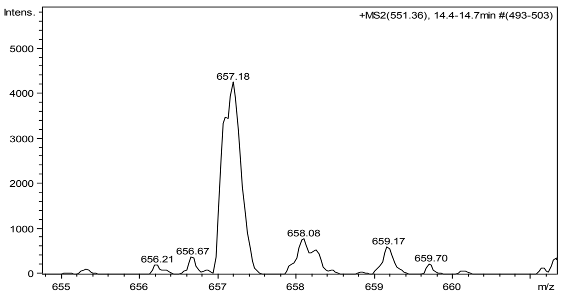

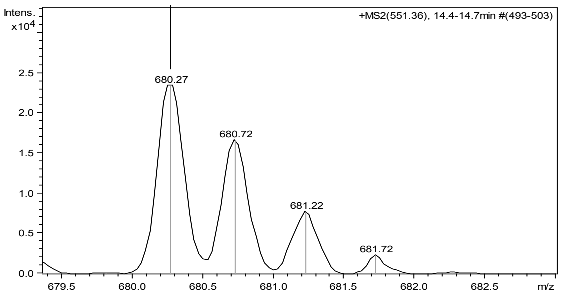

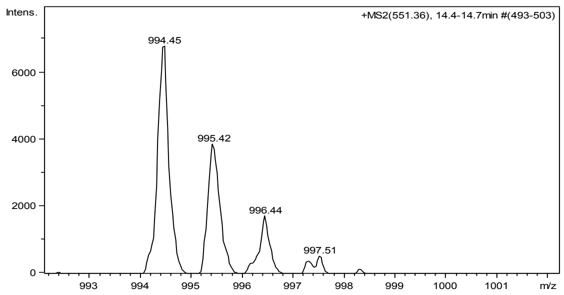

Fraction 15

550.86+++ → Pep [M+2H]++ 497.70++ [14.0-14.7 min] CID-MS3

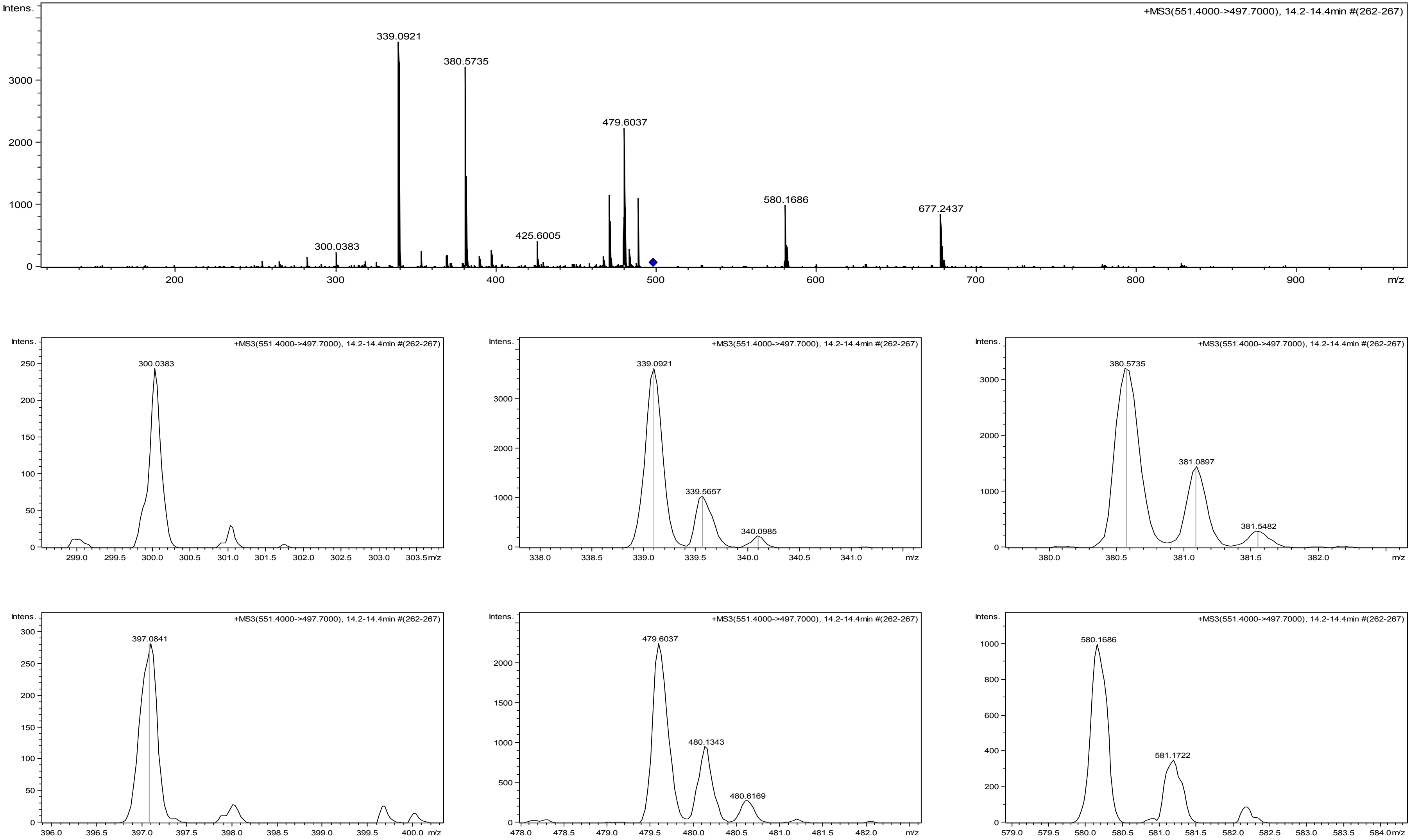

Fraction 15

550.86+++ → Pep [M+2H]++ 497.70++ [14.0-14.7 min] CID-MS3

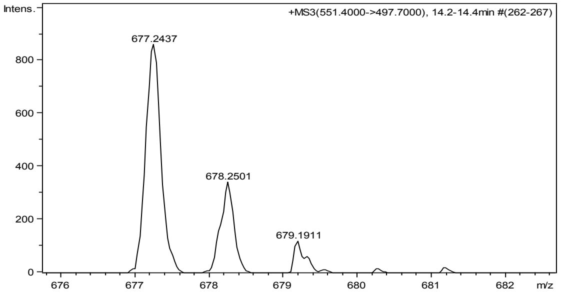

Fraction 15

550.86+++ → Pep [M+2H]++ 497.70++ [14.0-14.7 min]

CID-MS3 MASCOT Search

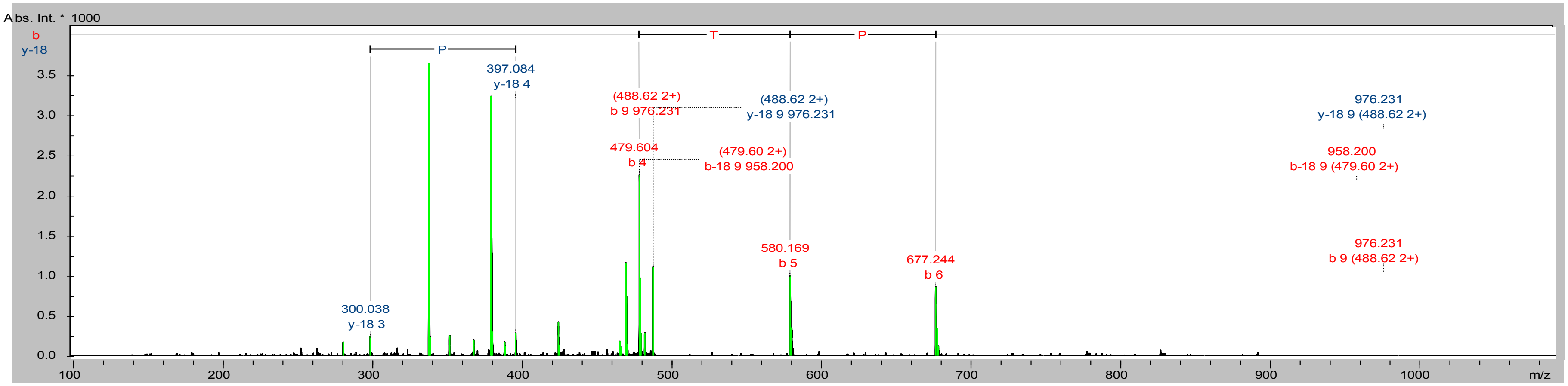

	H	L	D	L	T	P	S	P	D	His	Leu	Asp	Leu	Thr	Pro	Ser	Pro	Asp
Ion	1	2	3	4	5	6	7	8	9	1	2	3	4	5	6	7	8	9
b	H	L	D	L	T	P	S	P	D	138.066	251.150	366.177	479.261	580.309	677.362	764.394	861.446	976.473
b-17	H	L	D	L	T	P	S	P	D	-	-	-	-	-	-	-	-	-
b-18	H	L	D	L	T	P	S	P	D	-	-	348.167	461.251	562.298	659.351	746.383	843.436	958.463
y	H	L	D	L	T	P	S	P	D	134.045	231.098	318.130	415.182	516.230	629.314	744.341	857.425	994.484
y-17	H	L	D	L	T	P	S	P	D	-	-	-	-	-	-	-	-	-
y-18	H	L	D	L	T	P	S	P	D	116.034	213.087	300.119	397.172	498.219	611.304	726.330	839.415	976.473
	9	8	7	6	5	4	3	2	1	Asp	Pro	Ser	Pro	Thr	Leu	Asp	Leu	His

Most likely:

unknown O-glycosylation site

Legumain Precursor

Not sure

$_{301}$HLDL**TPS**PD$_{309}$

Fraction 15

550.86+++ → Pep [M+2H]++ 497.70++ [14.0-14.7 min]

CID-MS3 MASCOT Search

prot_hit_nur	prot_acc	prot_desc	prot_score	prot_mass	prot_matche	pep_query	pep_rank	pep_isbold	pep_exp_mz	pep_exp_mr	pep_exp_z	pep_calc_mr	pep_delta	pep_miss	pep_score	pep_expect	pep_res_bef	pep_seq
1	HEBP1_HUM	Heme-bindir	15	21198	1	1	2	1	497.6963	993.378	2	993.5317	-0.1537	0	17.5	2.70E+02	E	AMPKVAKYA
2	RM30_HUMA	39S ribosom	13	18648	1	1	3	0	497.6963	993.378	2	992.6382	0.7398	0	17.33	2.80E+02	I	KPLKLPQGL
3	LGMN_HUM	Legumain pr	12	49779	1	1	3	0	497.6963	993.378	2	993.4767	-0.0987	0	17.33	2.80E+02	T	HLDLTPSPD
4	SPAG7_HUM	Sperm-assoc	12	26132	1	1	8	0	497.6963	993.378	2	993.5495	-0.1715	0	14.6	5.20E+02	S	ILHDVVEVA
5	TPR_HUMAN	Nucleoprote	12	265840	1	1	1	0	497.6963	993.378	2	993.5859	-0.2078	0	20.45	1.40E+02	T	PLQVAAPVTV
6	NOL1_HUMA	Proliferating	11	94705	1	1	6	0	497.6963	993.378	2	992.6019	0.7762	0	16.74	3.20E+02	K	PSPGKLPKGI
7	WNK1_HUM	Serine/threc	9	251457	1	1	3	0	497.6963	993.378	2	992.5655	0.8126	0	17.33	2.80E+02	H	PQLLQPAVQ
8	ARHGC_HUN	Rho guanine	9	174094	1	1	6	0	497.6963	993.378	2	993.5131	-0.1351	0	16.74	3.20E+02	P	PGSPQIPLAD
9	CAC1A_HUM	Voltage-dep	6	283782	1	1	8	0	497.6963	993.378	2	992.5695	0.8085	0	14.6	5.20E+02	M	FIFAVVAVQ

Biotools-Score: 9

MASCOT-Score: 17

Most likely:

unknown O-glycosylation site

Legumain Precursor

Not sure

$_{301}$HLDL**T**P**S**PD$_{309}$

Fraction 15

550.86+++ → Pep [M+2H]++ 497.70++ [14.0-14.7 min] ETD

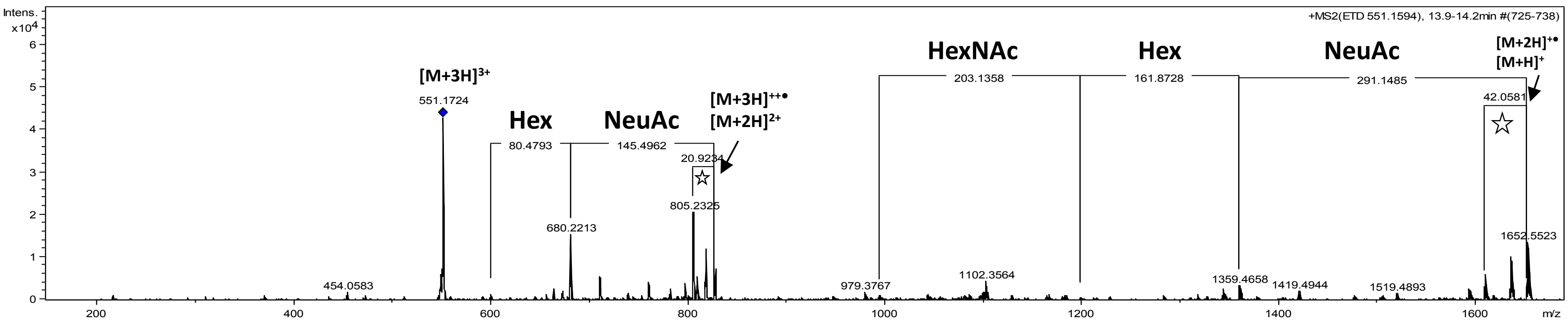

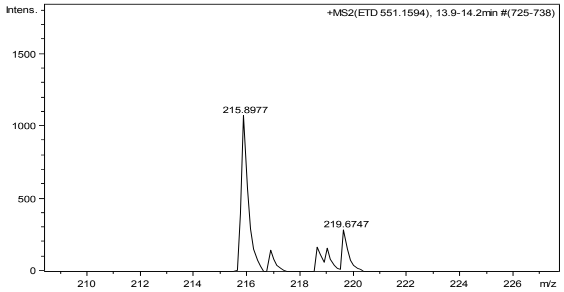

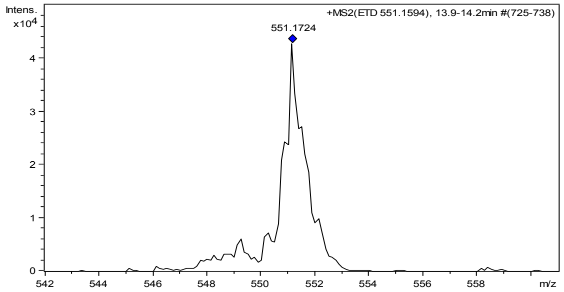

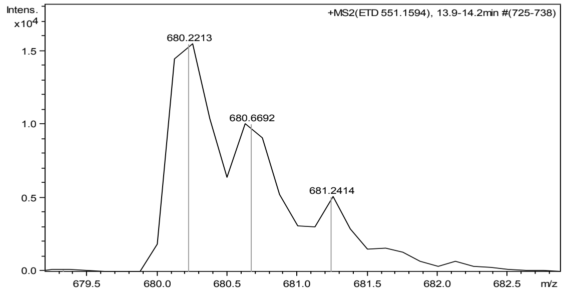

Fraction 15

550.86+++ → Pep [M+2H]++ 497.70++ [14.0-14.7 min]

ETD

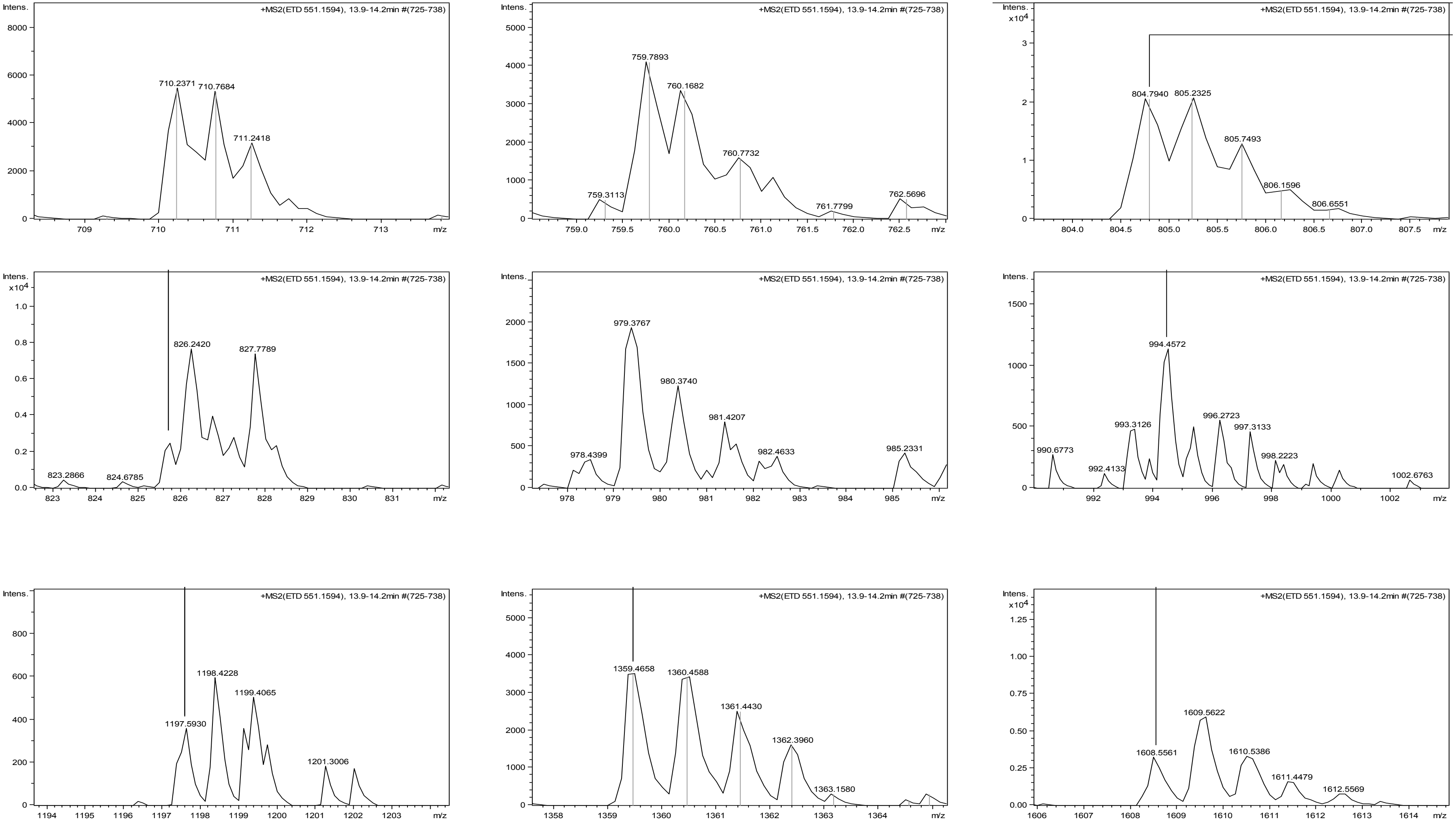

Fraction 15

550.86+++ → Pep [M+2H]++ 497.70++ [14.0-14.7 min] ETD

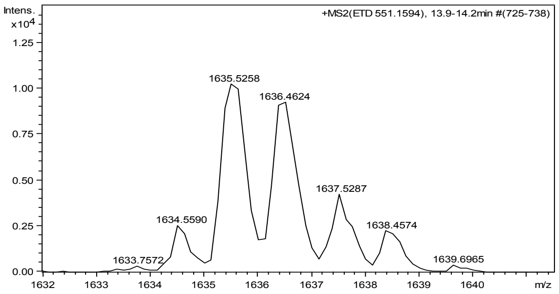

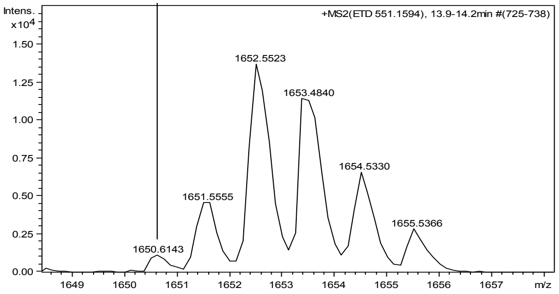

Fraction 15

550.86+++ → Pep [M+2H]++ 497.70++ [14.0-14.7 min] ETD

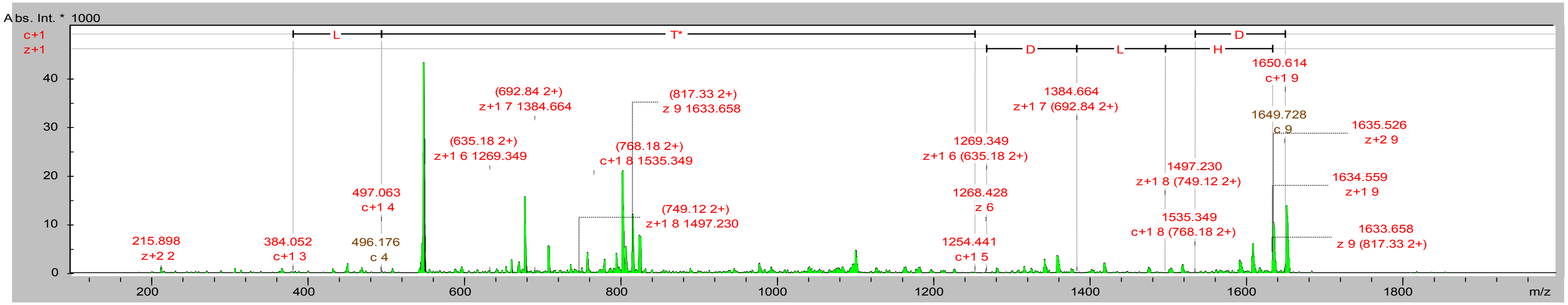

$_{301}$HLDLTPSPD$_{309}$

	H	L	D	L	T	P	S	P	D	His	Leu	Asp	Leu	Thr	Pro	Ser	Pro	Asp
Ion	1	2	3	4	5	6	7	8	9	1	2	3	4	5	6	7	8	9
c	H	L	D	L	T*	P	S	P	D	155.093	268.177	383.204	496.288	1253.563	1350.616	1437.648	1534.701	1649.728
c+1	H	L	D	L	T*	P	S	P	D	156.101	269.185	384.212	497.296	1254.571	1351.624	1438.656	1535.708	1650.735
z	H	L	D	L	T*	P	S	P	D	117.018	214.071	301.103	398.156	1155.431	1268.515	1383.542	1496.626	1633.685
z+1	H	L	D	L	T*	P	S	P	D	118.026	215.079	302.111	399.164	1156.439	1269.523	1384.550	1497.634	1634.693
z+2	H	L	D	L	T*	P	S	P	D	119.034	216.087	303.119	400.171	1157.447	1270.531	1385.558	1498.642	1635.701
	9	8	7	6	5	4	3	2	1	Asp	Pro	Ser	Pro	Thr	Leu	Asp	Leu	His

Biotools-Score: 31

Most likely:

unknown O-glycosylation site

Legumain Precursor

Not sure

$_{301}$HLDLTPSPD$_{309}$

Fraction 15

550.86+++ → Pep [M+2H]++ 497.70++ [14.0-14.7 min] ETD

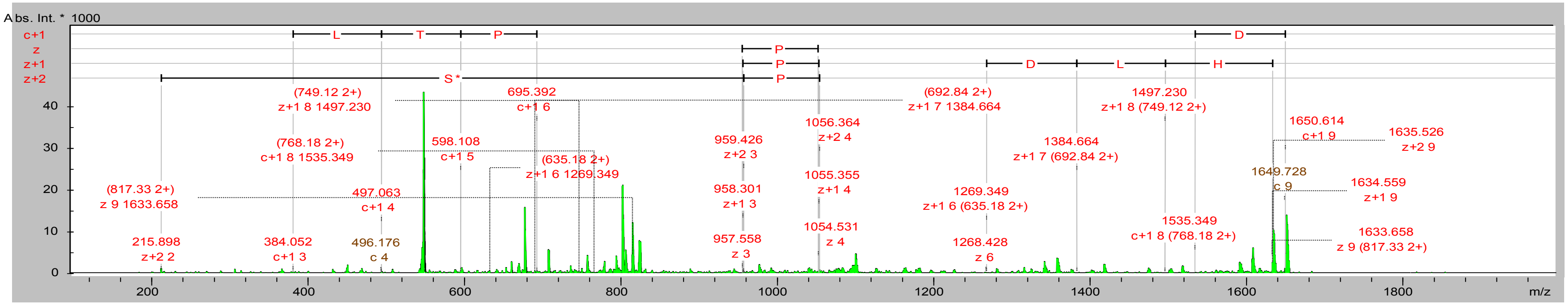

$_{301}$HLDLTPSPD$_{309}$ **Most likely**

	H	L	D	L	T	P	S	P	D	His	Leu	Asp	Leu	Thr	Pro	Ser	Pro	Asp
Ion	1	2	3	4	5	6	7	8	9	1	2	3	4	5	6	7	8	9
c	H	L	D	L	T	P	S*	P	D	155.093	268.177	383.204	496.288	597.335	694.388	1437.648	1534.701	1649.728
c+1	H	L	D	L	T	P	S*	P	D	156.101	269.185	384.212	497.296	598.343	695.396	1438.656	1535.708	1650.735
z	H	L	D	L	T	P	S*	P	D	117.018	214.071	957.331	1054.383	1155.431	1268.515	1383.542	1496.626	1633.685
z+1	H	L	D	L	T	P	S*	P	D	118.026	215.079	958.338	1055.391	1156.439	1269.523	1384.550	1497.634	1634.693
z+2	H	L	D	L	T	P	S*	P	D	119.034	216.087	959.346	1056.399	1157.447	1270.531	1385.558	1498.642	1635.701
	9	8	7	6	5	4	3	2	1	Asp	Pro	Ser	Pro	Thr	Leu	Asp	Leu	His

Biotools-Score: 51

Most likely:

unknown O-glycosylation site

Legumain Precursor

Not sure

$_{301}$HLDLTPSPD$_{309}$

Fraction 15

806.77++ → Pep [M+H]+ 956.51+ [15.3-15.8 min] CID-MS Precursor

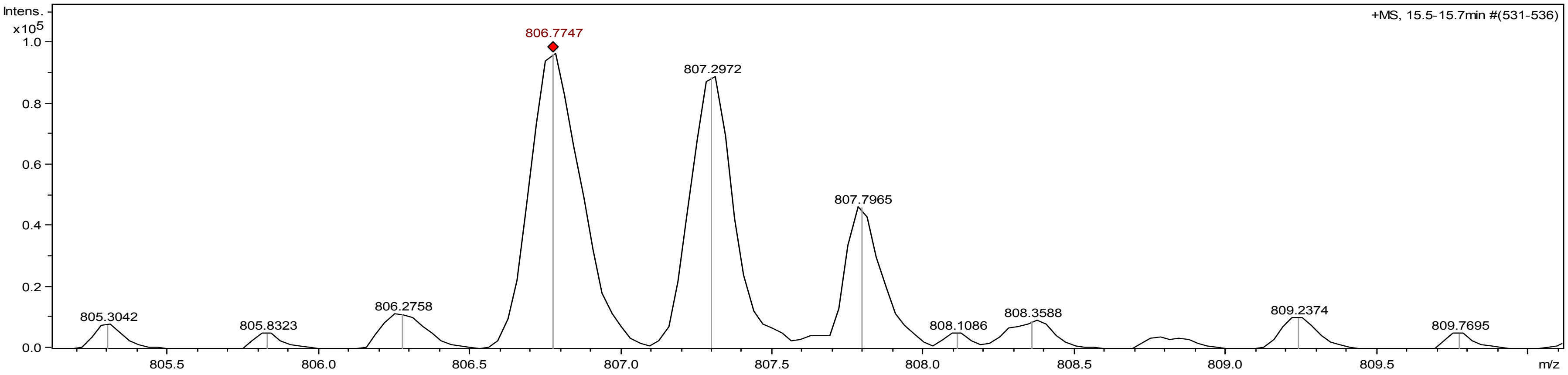

ETD spectrum not available

Fraction 15

806.77++ → Pep [M+H]+ 956.51+ [15.3-15.8 min] CID-MS2

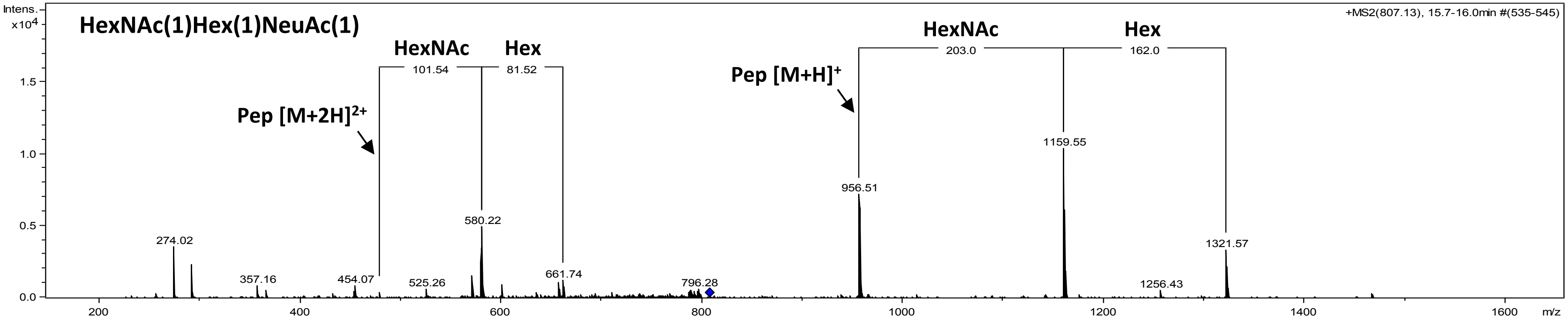

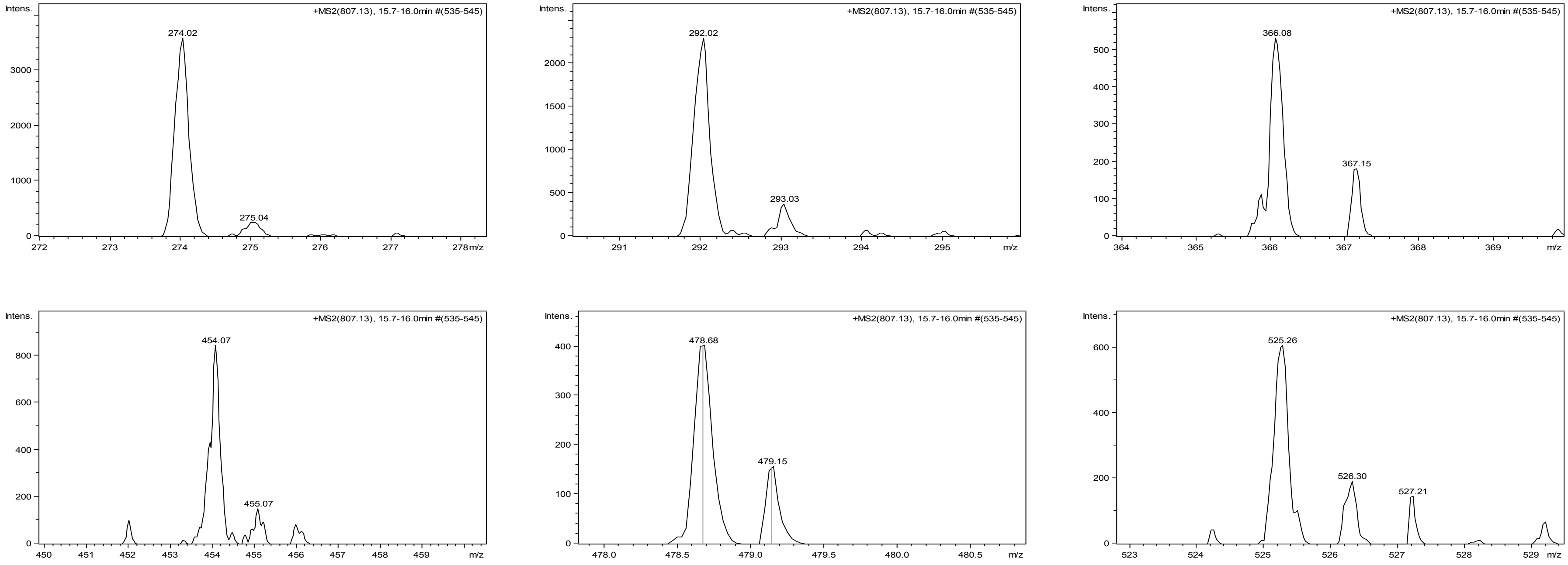

Fraction 15

806.77++ → Pep [M+H]+ 956.51+ [15.3-15.8 min]

CID-MS2

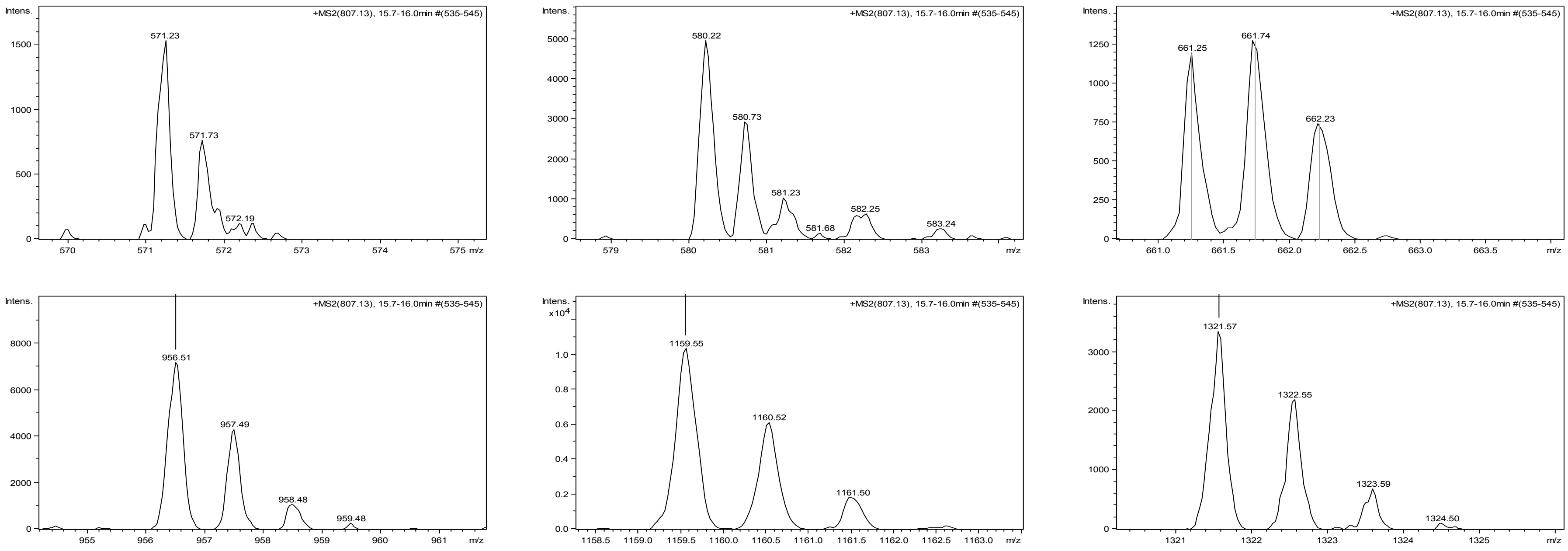

Fraction 15

806.77++ → Pep [M+H]+ 956.51+ [15.3-15.8 min] CID-MS3

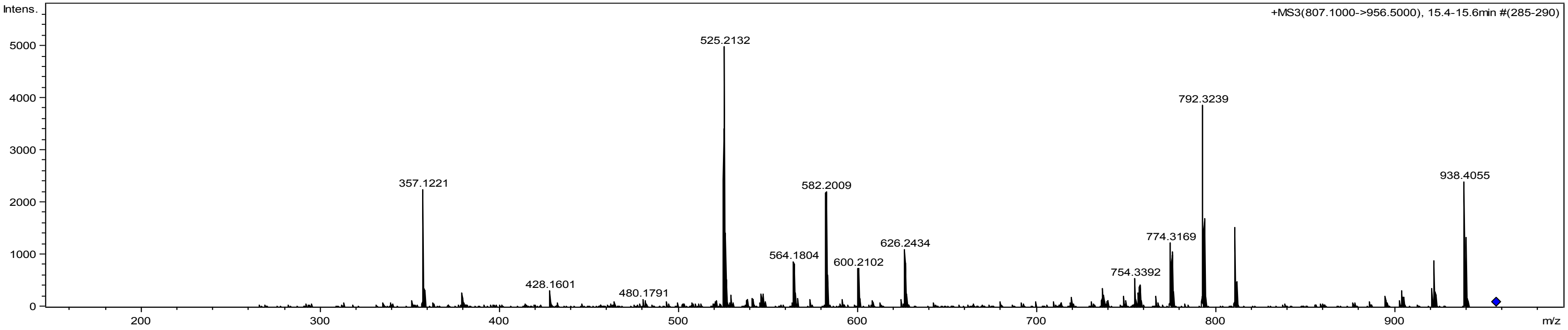

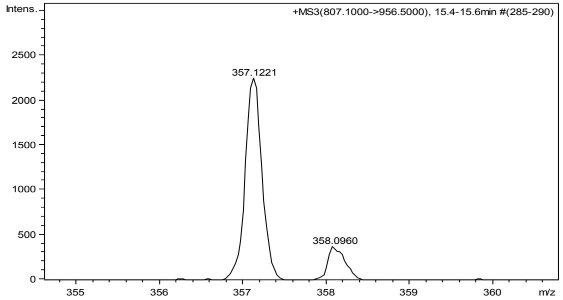

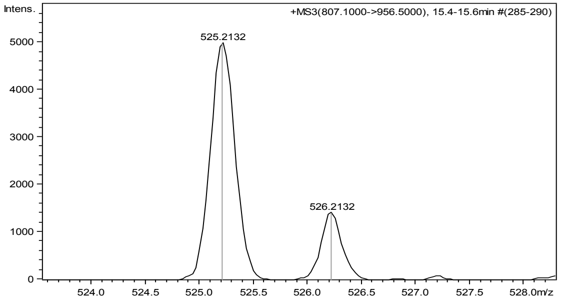

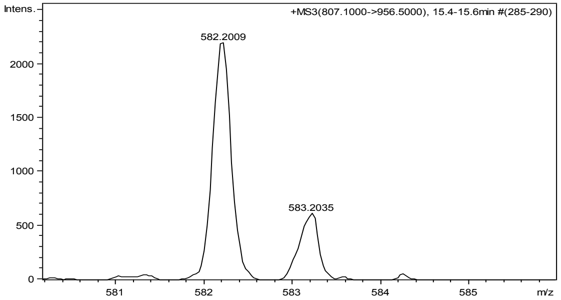

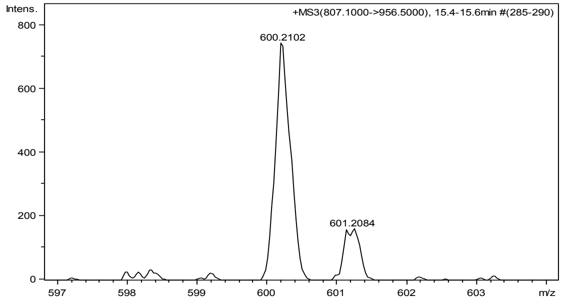

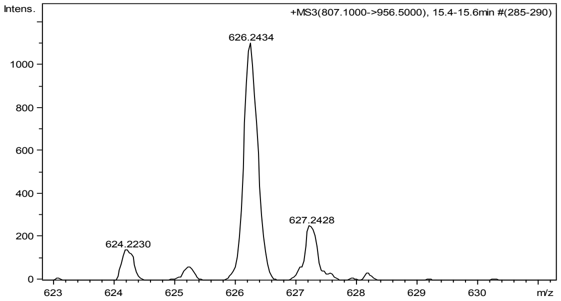

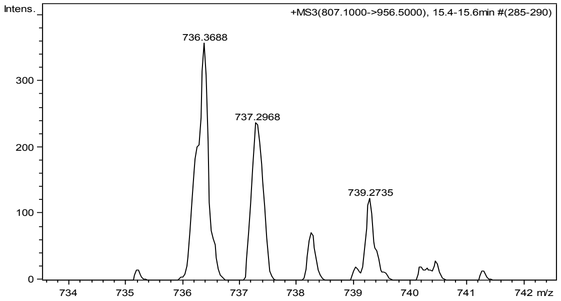

Fraction 15

806.77++ → Pep [M+H]+ 956.51+ [15.3-15.8 min]

CID-MS3

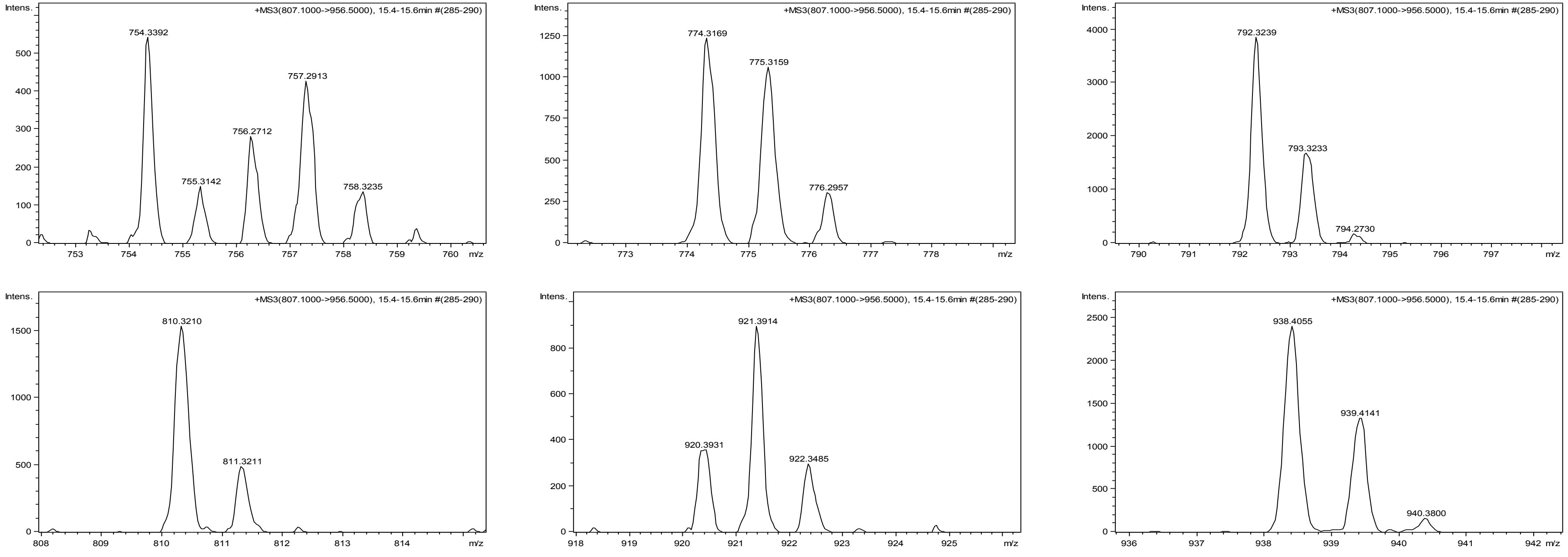

Fraction 15

806.77++ → Pep [M+H]+ 956.51+ [15.3-15.8 min]　　CID-MS3　MASCOT Search

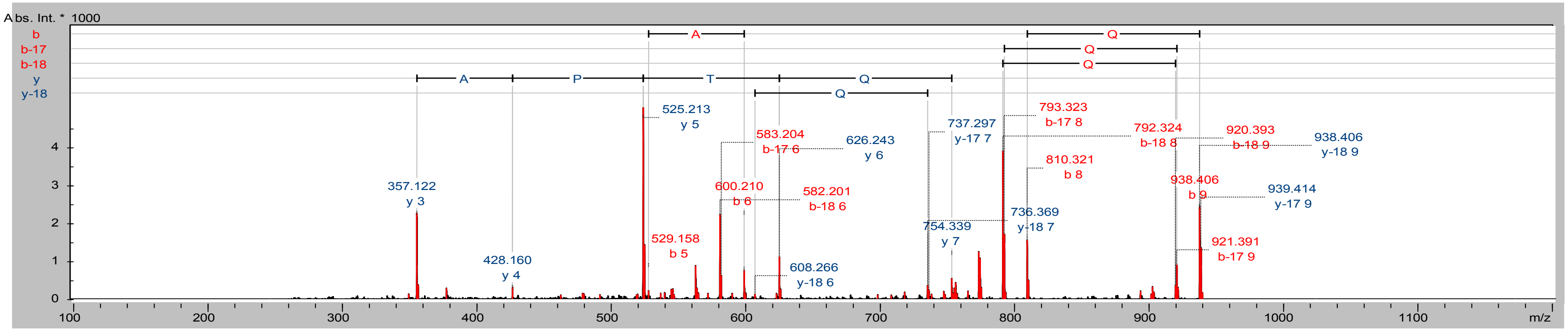

	T	T	Q	T	P	A	P	I	Q	Thr	Thr	Gln	Thr	Pro	Ala	Pro	Ile	Gln
Ion	1	2	3	4	5	6	7	8	9	1	2	3	4	5	6	7	8	9
b	T	T	Q	T	P	A	P	I	Q	102.055	203.103	331.161	432.209	529.262	600.299	697.352	810.436	938.494
b-17	T	T	Q	T	P	A	P	I	Q	-	-	314.135	415.182	512.235	583.272	680.325	793.409	921.468
b-18	T	T	Q	T	P	A	P	I	Q	84.044	185.092	313.151	414.198	511.251	582.288	679.341	792.425	920.484
y	T	T	Q	T	P	A	P	I	Q	147.076	260.160	357.213	428.250	525.303	626.351	754.409	855.457	956.505
y-17	T	T	Q	T	P	A	P	I	Q	130.050	243.134	340.187	411.224	508.277	609.324	737.383	838.431	939.478
y-18	T	T	Q	T	P	A	P	I	Q	-	-	-	-	-	608.340	736.399	837.446	938.494
	9	8	7	6	5	4	3	2	1	Gln	Ile	Pro	Ala	Pro	Thr	Gln	Thr	Thr

Glycosylation site cannot be exactly determined

known O-glycosylation region

Inter-alpha-trypsin inhibitor heavy chain H4 precursor

$_{722}$TTQTPAPIQ$_{730}$

Fraction 15

806.77++ → Pep [M+H]+ 956.51+ [15.3-15.8 min]

CID-MS3 MASCOT Search

prot_hit_nur	prot_acc	prot_desc	prot_score	prot_mass	prot_matche	pep_query	pep_rank	pep_isbold	pep_exp_mz	pep_exp_mr	pep_exp_z	pep_calc_mr	pep_delta	pep_miss	pep_score	pep_expect	pep_res_bef	pep_seq
1	CHD6_HUMA	Chromodom	30	308071	1	1	1	1	956.509	955.5017	1	955.5087	-0.0069	0	37.57	2.3	Q	EARAPTIAQ
2	ARPM1_HUN	Actin-relatec	27	41665	1	1	2	0	956.509	955.5017	1	955.4974	0.0043	0	32.06	8.3	M	NLEAPGIDK
3	ITIH4_HUMA	Inter-alpha-t	20	103489	1	1	3	0	956.509	955.5017	1	955.4975	0.0043	0	26.28	31	M	TTQTPAPIQ
4	DIDO1_HUM	Death-induc	15	245434	1	1	4	0	956.509	955.5017	1	955.5338	-0.0321	0	23.33	62	A	NVAAATPAIK
5	ADA15_HUM	ADAM 15 pre	15	90397	1	1	7	0	956.509	955.5017	1	955.4611	0.0406	0	20.33	1.20E+02	T	LEQGPGDLQ
6	MAPE_HUMA	Melanoma a	15	58650	1	1	9	0	956.509	955.5017	1	955.5855	-0.0837	0	19.25	1.60E+02	T	WKLPTLAK
7	ESPL1_HUMA	Separin (EC 3	14	236501	1	1	5	0	956.509	955.5017	1	955.4723	0.0294	0	22.32	78	R	RAQAPGNLE
8	LRC27_HUMA	Leucine-rich	13	60223	1	1	10	0	956.509	955.5017	1	955.5451	-0.0433	0	18.84	1.70E+02	T	RSLPATPSK
9	PRKDC_HUM	DNA-depenc	12	473749	1	1	6	0	956.509	955.5017	1	955.4974	0.0043	0	21.41	97	K	DSKPPGNLK
10	TENA_HUMA	Tenascin pre	11	246358	1	1	8	0	956.509	955.5017	1	955.5855	-0.0837	0	19.5	1.50E+02	L	LWKTPLAK

Biotools-Score: 4

MASCOT-Score: 26

Glycosylation site cannot be exactly determined

known O-glycosylation region

Inter-alpha-trypsin inhibitor heavy chain H4 precursor

$_{722}$TTQTPAPIQ$_{730}$

Fraction 15

664.69++ → Pep [M+H]+ 672.30+ [16.3-16.6 min] CID-MS Precursor

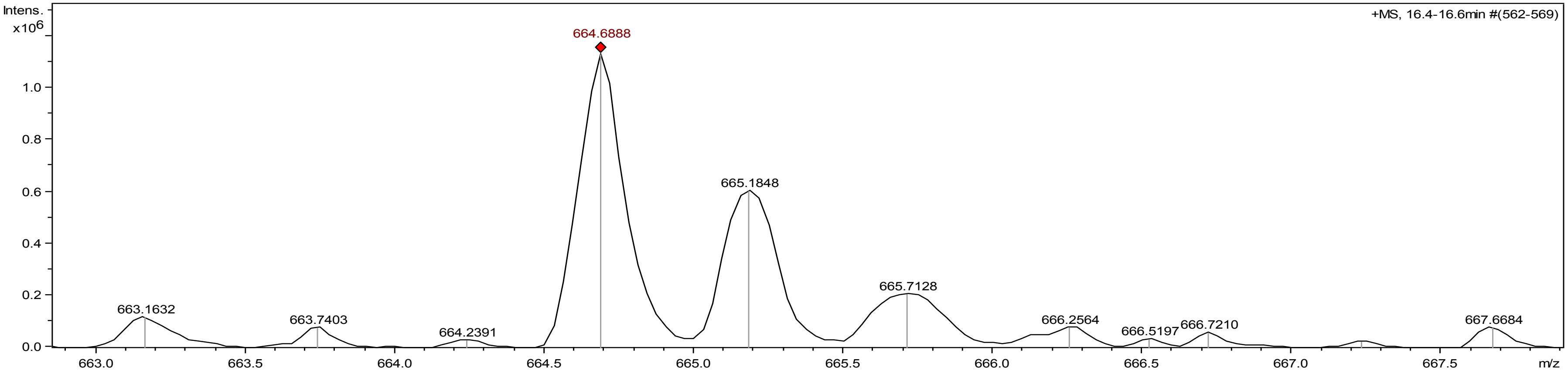

Fraction 15

664.69++ → Pep [M+H]+ 672.30+ [16.3-16.6 min] CID-MS2

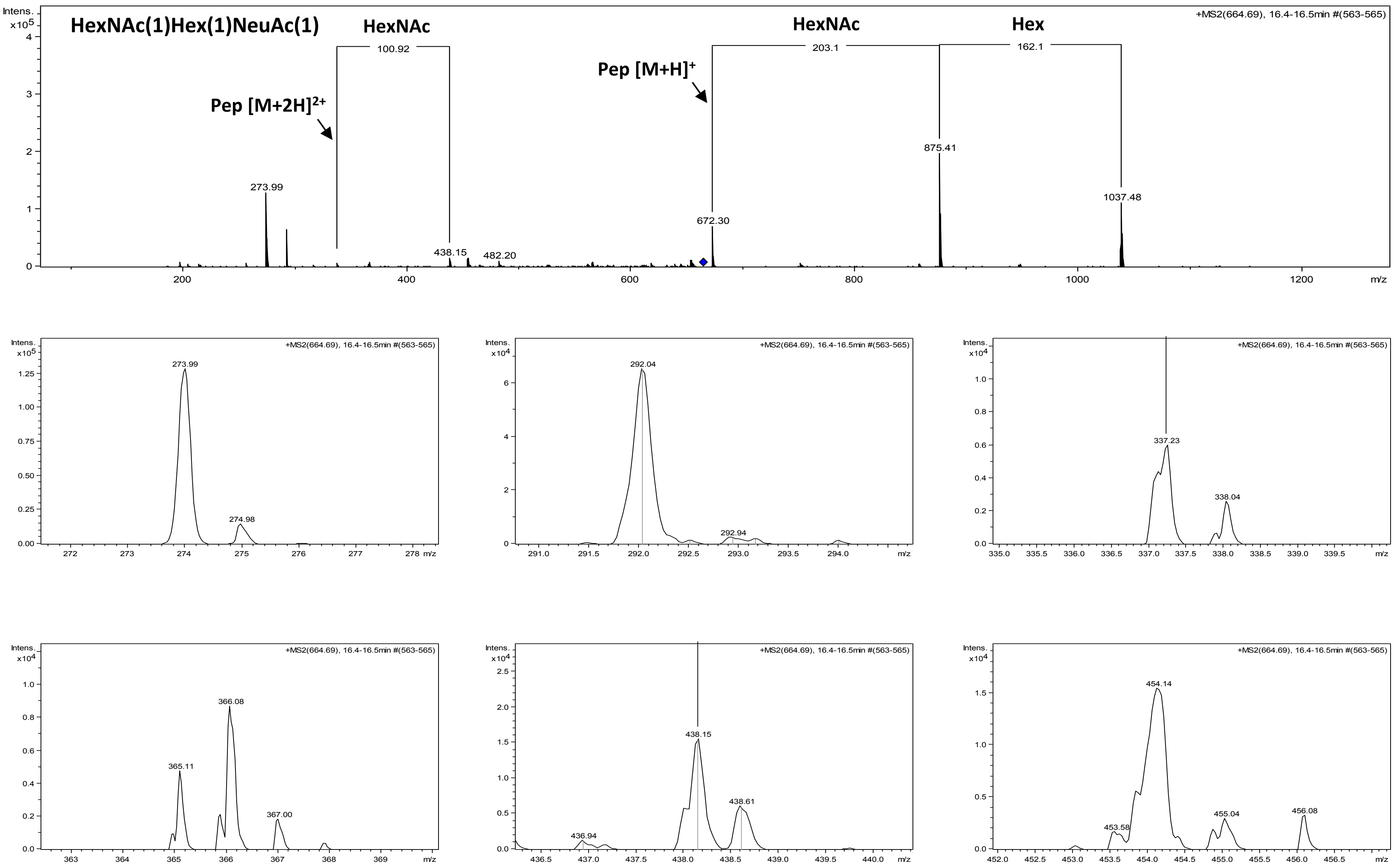

Fraction 15

664.69++ → Pep [M+H]+ 672.30+ [16.3-16.6 min] CID-MS2

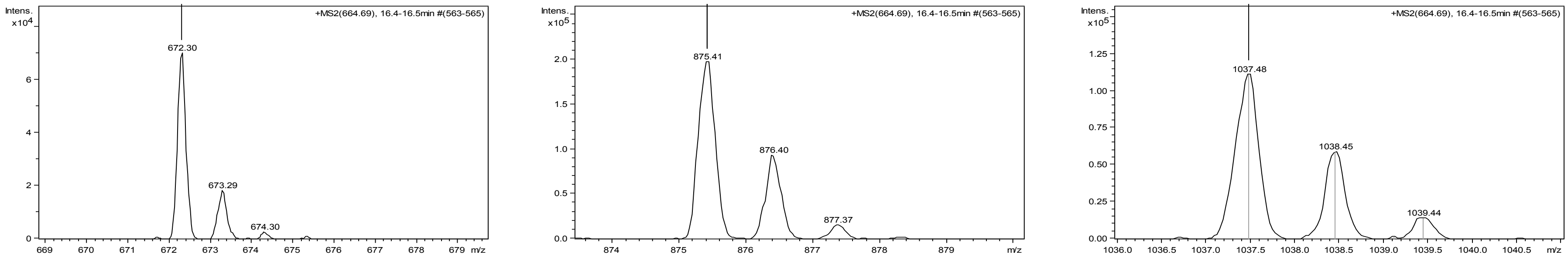

Fraction 15

664.69++ → Pep [M+H]+ 672.30+ [16.3-16.6 min]

CID-MS3

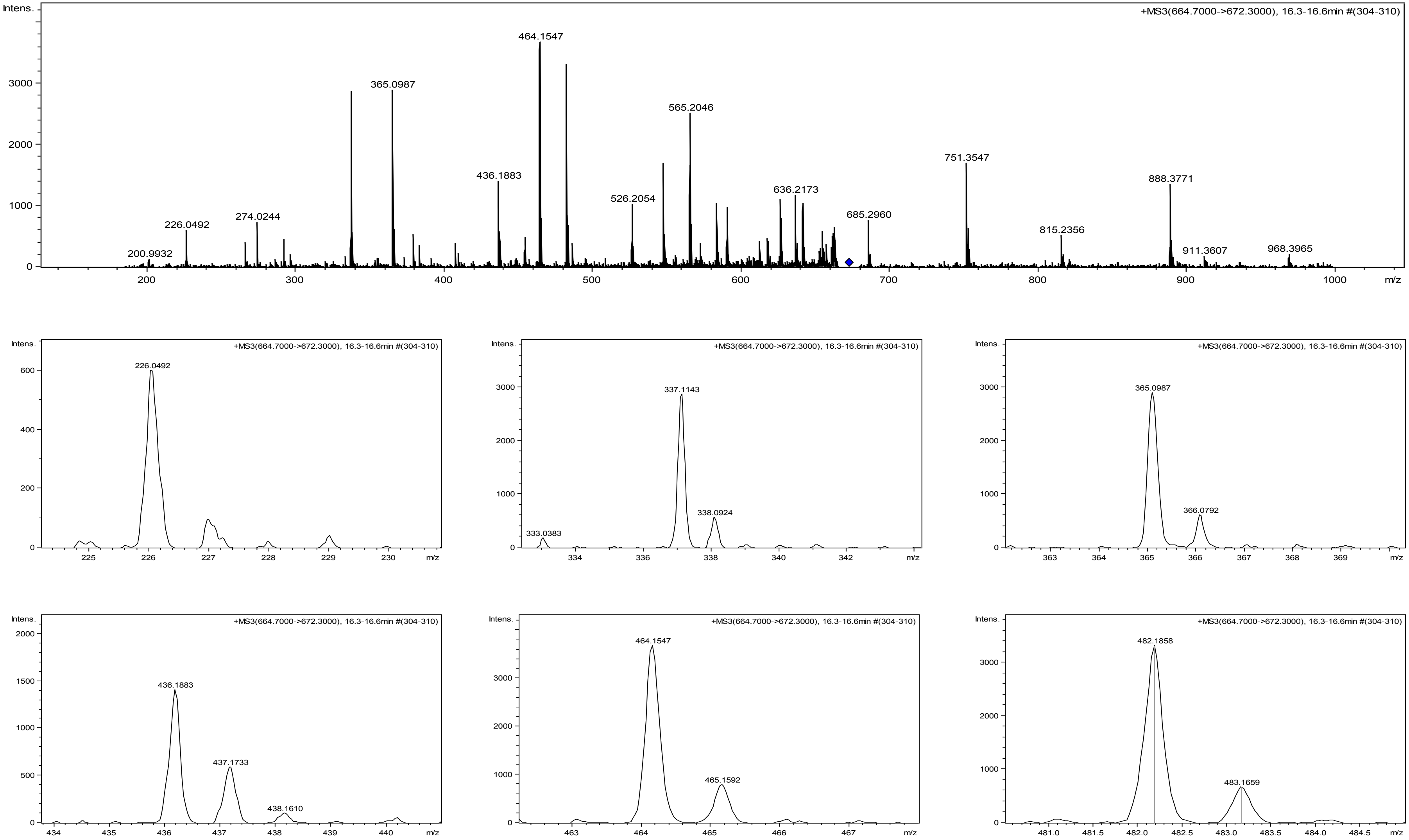

Fraction 15

664.69++ → Pep [M+H]+ 672.30+ [16.3-16.6 min]

CID-MS3

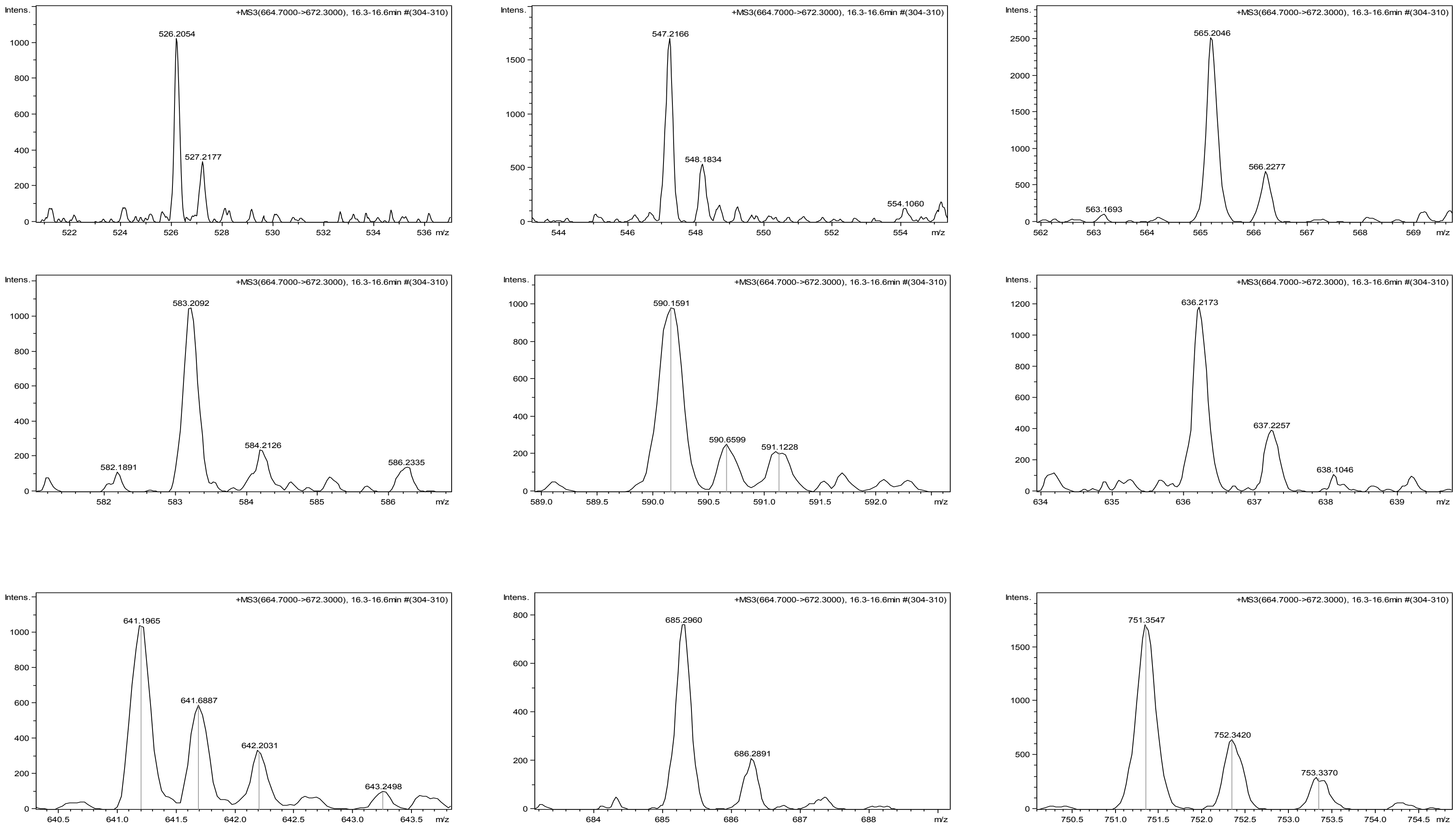

Fraction 15

664.69++ → Pep [M+H]+ 672.30+ [16.3-16.6 min]

CID-MS3

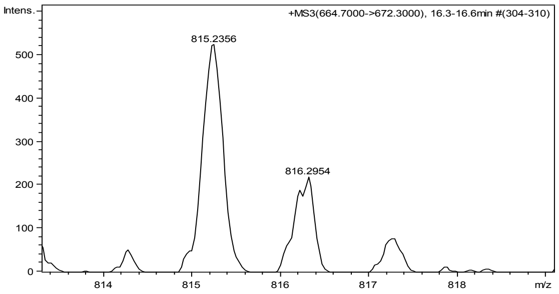

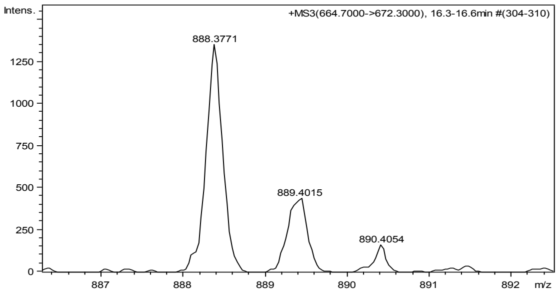

Fraction 15

664.69++ → Pep [M+H]+ 672.30+ [16.3-16.6 min]   CID-MS3   MASCOT Search

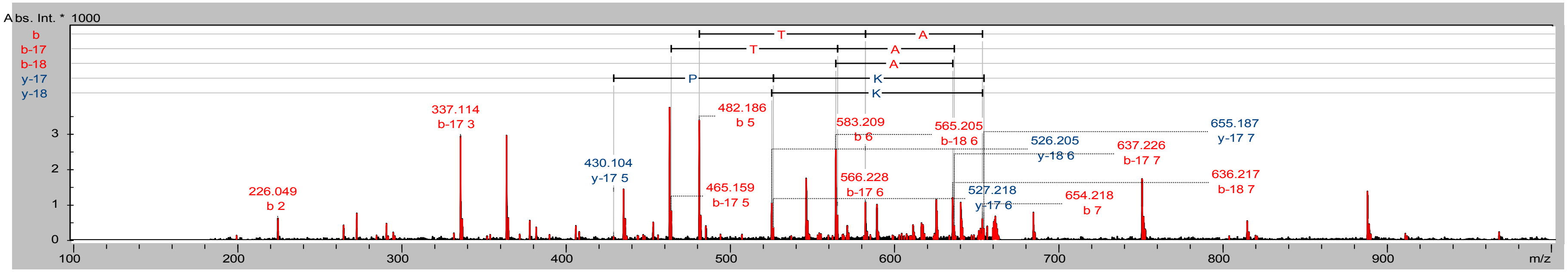

	K	P	Q	A	G	T	A	Lys	Pro	Gln	Ala	Gly	Thr	Ala
Ion	1	2	3	4	5	6	7	1	2	3	4	5	6	7
b	K	P	Q	A	G	T	A	129.102	226.155	354.214	425.251	482.272	583.320	654.357
b-17	K	P	Q	A	G	T	A	112.076	209.128	337.187	408.224	465.246	566.293	637.330
b-18	K	P	Q	A	G	T	A	-	-	-	-	-	565.309	636.346
y	K	P	Q	A	G	T	A	90.055	191.103	248.124	319.161	447.220	544.273	672.368
y-17	K	P	Q	A	G	T	A	-	-	-	-	430.193	527.246	655.341
y-18	K	P	Q	A	G	T	A	-	173.092	230.114	301.151	429.209	526.262	654.357
	7	6	5	4	3	2	1	Ala	Thr	Gly	Ala	Gln	Pro	Lys

known O-glycosylation site

Insulin-like growth factor-binding protein 6

$_{121}$KPQAG**T**A$_{127}$

Fraction 15

664.69++ → Pep [M+H]+ 672.30+ [16.3-16.6 min]

CID-MS3   MASCOT Search

prot_hit_nur	prot_acc	prot_desc	prot_score	prot_mass	prot_matche	pep_query	pep_rank	pep_isbold	pep_exp_mz	pep_exp_mr	pep_exp_z	pep_calc_mr	pep_delta	pep_miss	pep_score	pep_expect	pep_res_bef	pep_seq
1	FA96B_HUM/	Protein FAM	14	17766	1	1	1	1	672.2974	671.2901	1	671.3602	-0.0701	0	16.25	2.70E+02	G	ERPVTA
2	IBP6_HUMAI	Insulin-like g	7	26219	1	1	6	0	672.2974	671.2901	1	671.3602	-0.0701	0	10.27	1.10E+03	S	KPQAGTA
3	VAV2_HUMA	Protein vav-:	7	102446	1	1	4	0	672.2974	671.2901	1	671.3238	-0.0337	0	12.2	6.80E+02	R	ERPAEA
4	FA59A_HUM	Protein FAM	6	98550	1	1	3	0	672.2974	671.2901	1	671.349	-0.0589	0	12.68	6.10E+02	S	PTSPVTA
5	LSD1_HUMAI	Lysine-speci	6	93358	1	1	2	0	672.2974	671.2901	1	671.3239	-0.0337	0	12.71	6.00E+02	G	HVGSTTA
6	ELOV4_HUM	Elongation o	6	36976	1	1	6	0	672.2974	671.2901	1	671.3966	-0.1065	0	10.27	1.10E+03	K	PKAGKTA
7	K1219_HUM/	Protein KIAA	5	168460	1	1	4	0	672.2974	671.2901	1	671.3238	-0.0337	0	12.2	6.80E+02	S	ERPAQA
8	I20L2_HUMA	Interferon-st	5	39415	1	1	6	0	672.2974	671.2901	1	671.433	-0.1429	0	10.27	1.10E+03	F	PKKKTA
9	ODP2_HUMA	Dihydrolipoy	4	66195	1	1	9	0	672.2974	671.2901	1	671.3126	-0.0225	0	10	1.10E+03	P	PTPSAQA
10	MICA1_HUM	NEDD9-inter	3	118884	1	1	9	0	672.2974	671.2901	1	671.349	-0.0589	0	10	1.10E+03	T	PVVSAQA

Biotools-Score: 18

MASCOT-Score: 10

known O-glycosylation site

Insulin-like growth factor-binding protein 6

$_{121}$KPQAGTA$_{127}$

Fraction 15

664.69++ → Pep [M+H]+ 672.30+ [16.3-16.6 min]

ETD

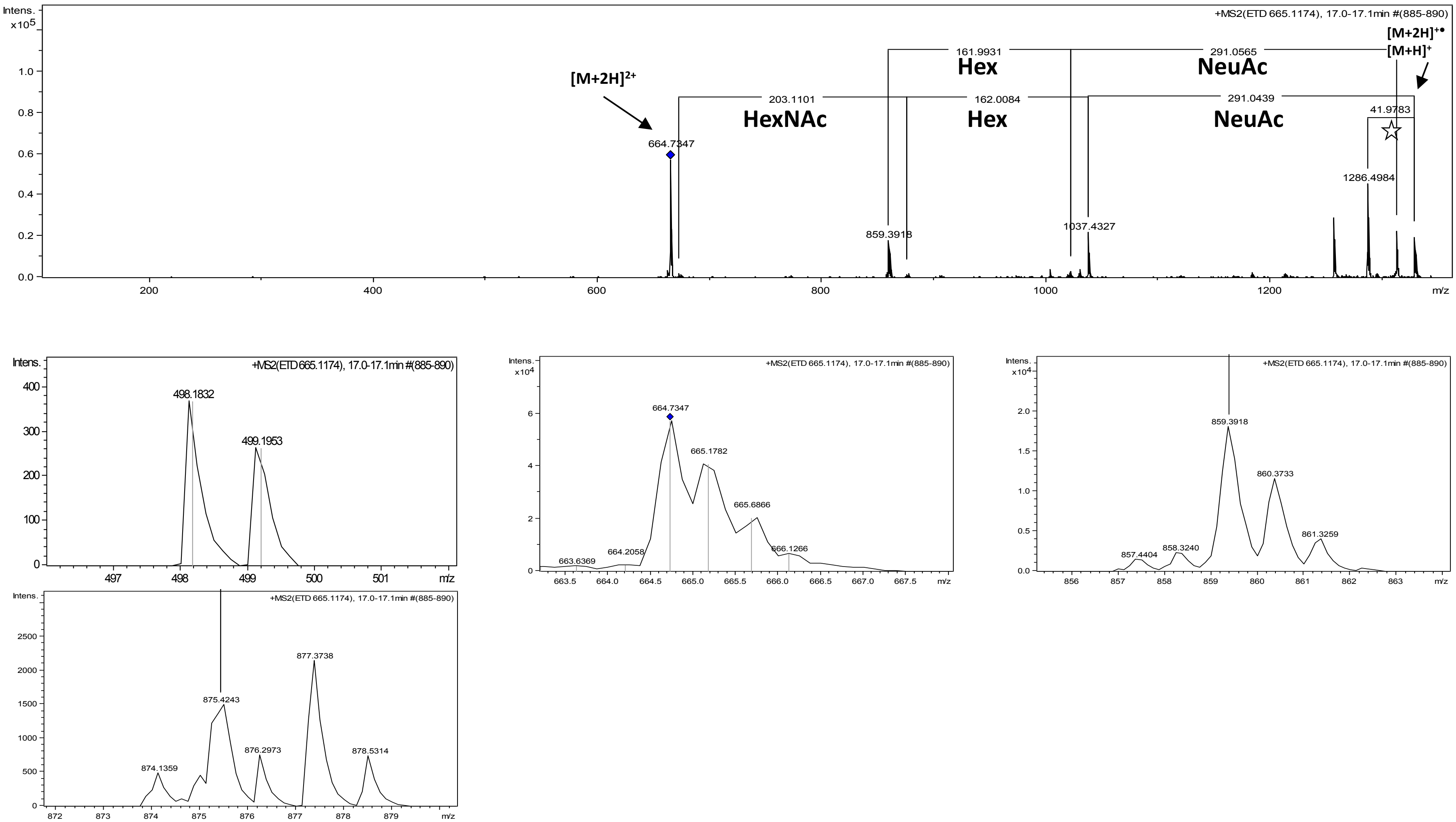

known O-glycosylation site

Insulin-like growth factor-binding protein 6

$_{121}$KPQAGTA$_{127}$

Fraction 15

664.69++ → Pep [M+H]+ 672.30+ [16.3-16.6 min]

ETD

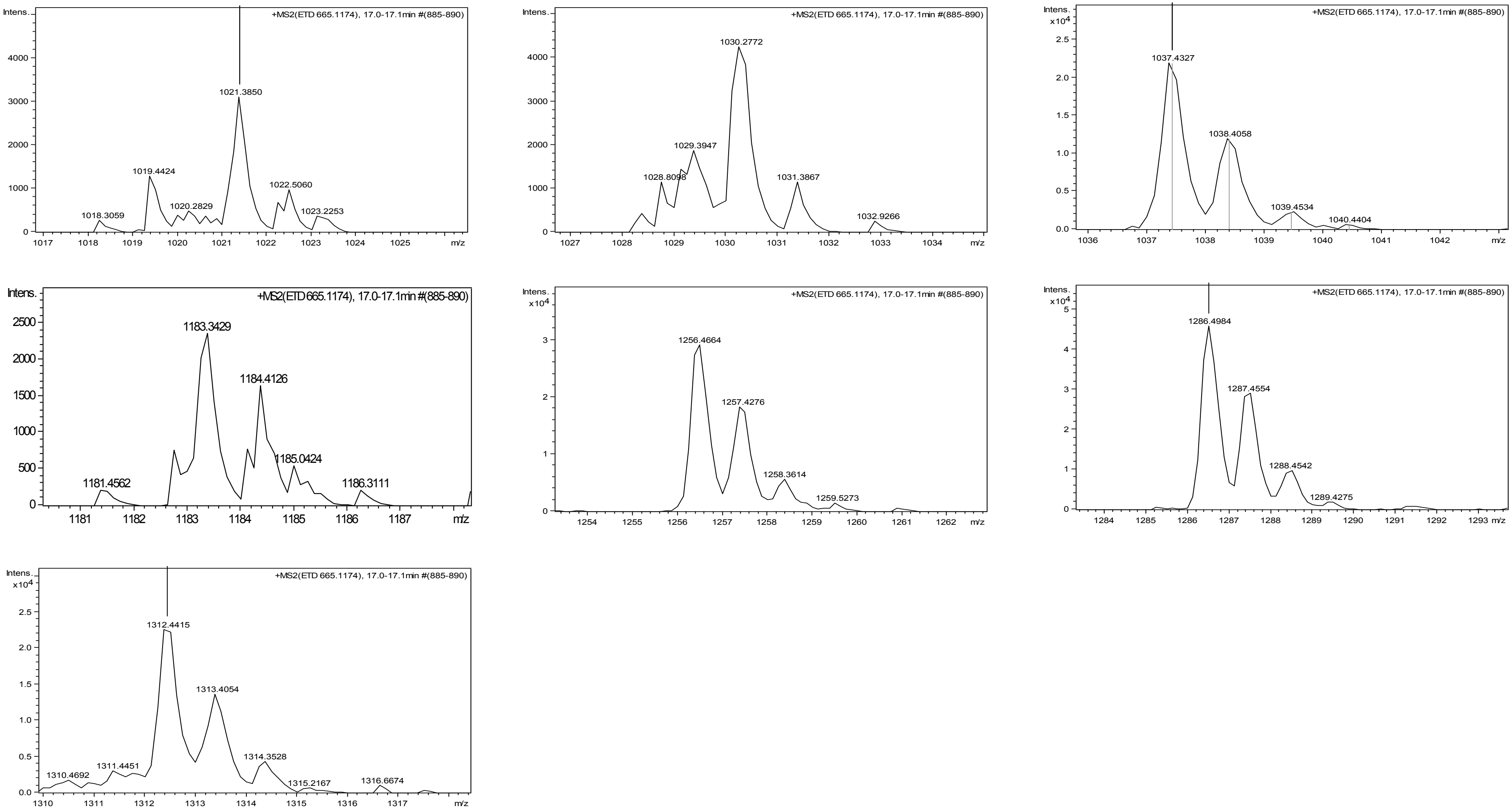

known O-glycosylation site

Insulin-like growth factor-binding protein 6

$_{121}$KPQAG**T**A$_{127}$

Fraction 15

664.69++ → Pep [M+H]+ 672.30+ [16.3-16.6 min] ETD

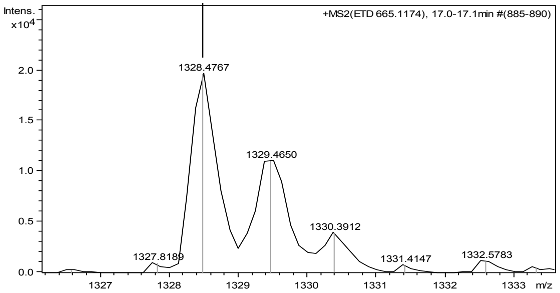

known O-glycosylation site

Insulin-like growth factor-binding protein 6

$_{121}$KPQAG**T**A$_{127}$

Fraction 15

664.69++ → Pep [M+H]+ 672.30+ [16.3-16.6 min] ETD

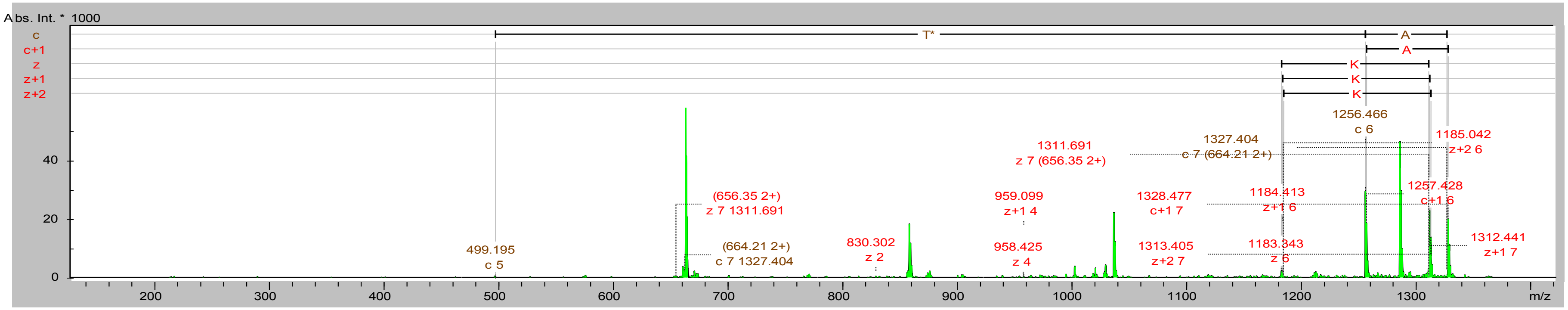

	K	P	Q	A	G	T	A	Lys	Pro	Gln	Ala	Gly	Thr	Ala
Ion	1	2	3	4	5	6	7	1	2	3	4	5	6	7
c	K	P	Q	A	G	T*	A	146.129	243.182	371.240	442.277	499.299	1256.574	1327.611
c+1	K	P	Q	A	G	T*	A	147.137	244.189	372.248	443.285	500.307	1257.582	1328.619
z	K	P	Q	A	G	T*	A	73.028	830.304	887.325	958.362	1086.421	1183.474	1311.569
z+1	K	P	Q	A	G	T*	A	74.036	831.312	888.333	959.370	1087.429	1184.481	1312.576
z+2	K	P	Q	A	G	T*	A	75.044	832.319	889.341	960.378	1088.437	1185.489	1313.584
	7	6	5	4	3	2	1	Ala	Thr	Gly	Ala	Gln	Pro	Lys

Biotools-Score: 22

known O-glycosylation site

Insulin-like growth factor-binding protein 6

$_{121}$KPQAG**T**A$_{127}$

Fraction 15

877.82++ → Pep [M+H]+ 1098.59+ [19.6-20.0 min] CID-MS Precursor

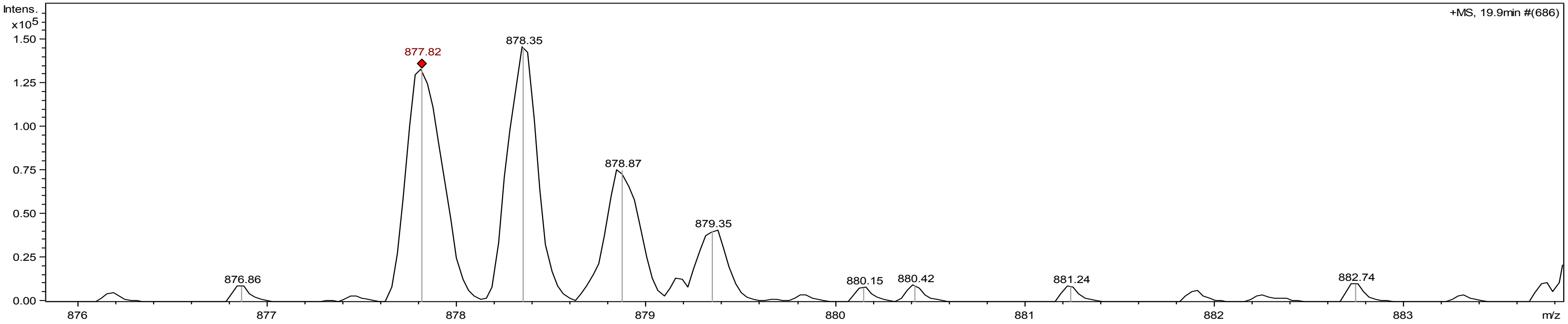

Fraction 15

877.82++ → Pep [M+H]+ 1098.59+ [19.6-20.0 min] CID-MS2

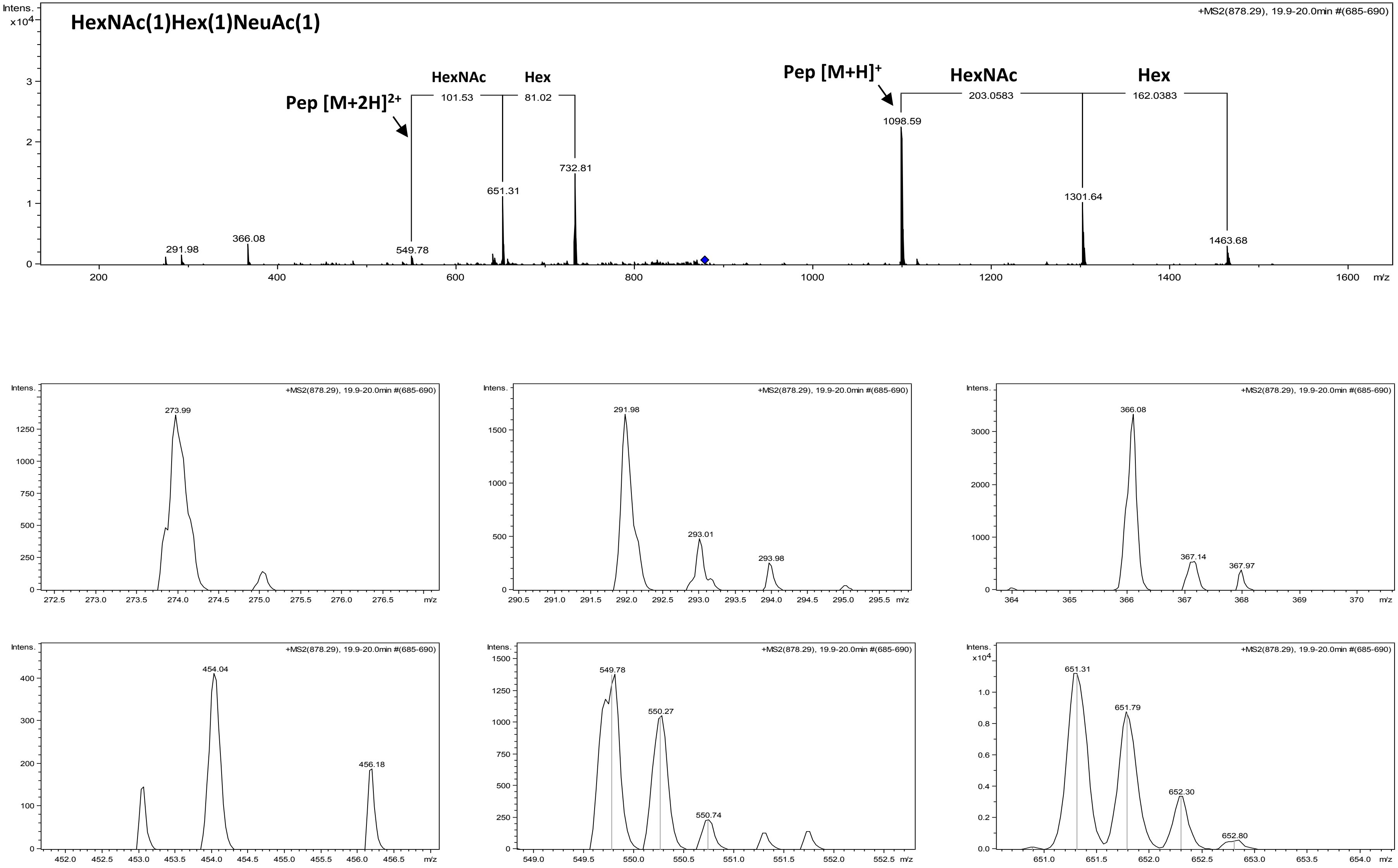

Fraction 15

877.82++ → Pep [M+H]+ 1098.59+ [19.6-20.0 min] CID-MS2

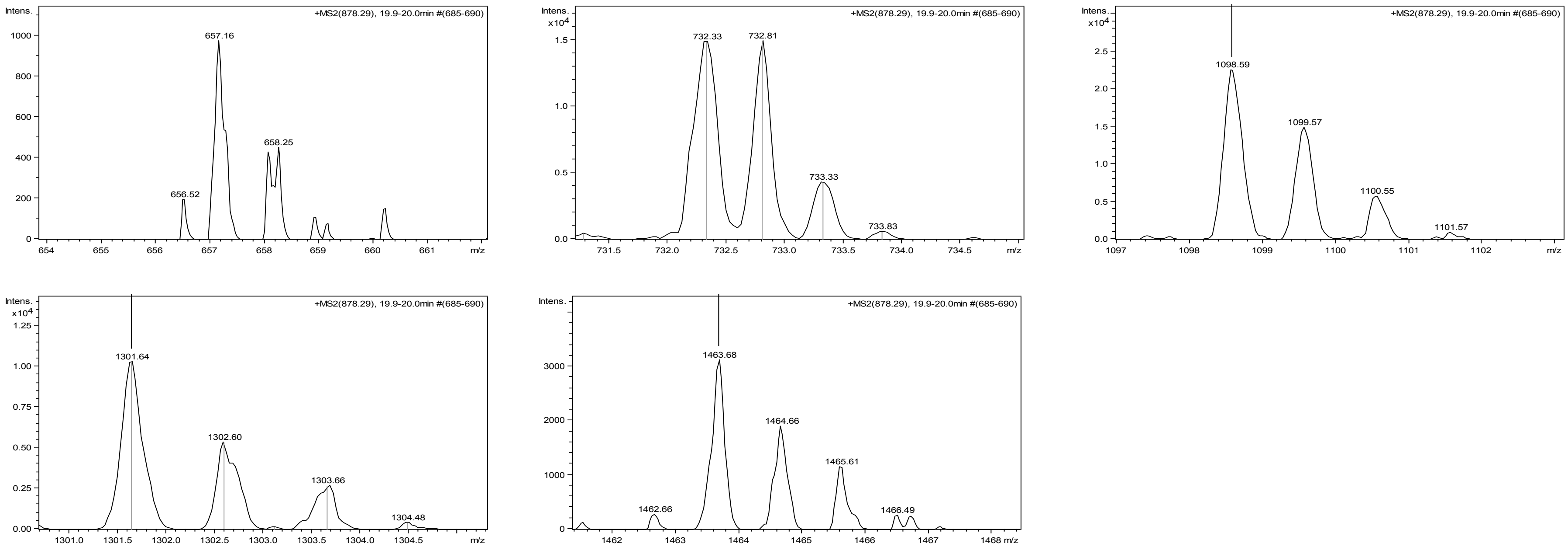

Fraction 15

877.82++ → Pep [M+H]+ 1098.59+ [19.6-20.0 min] CID-MS3

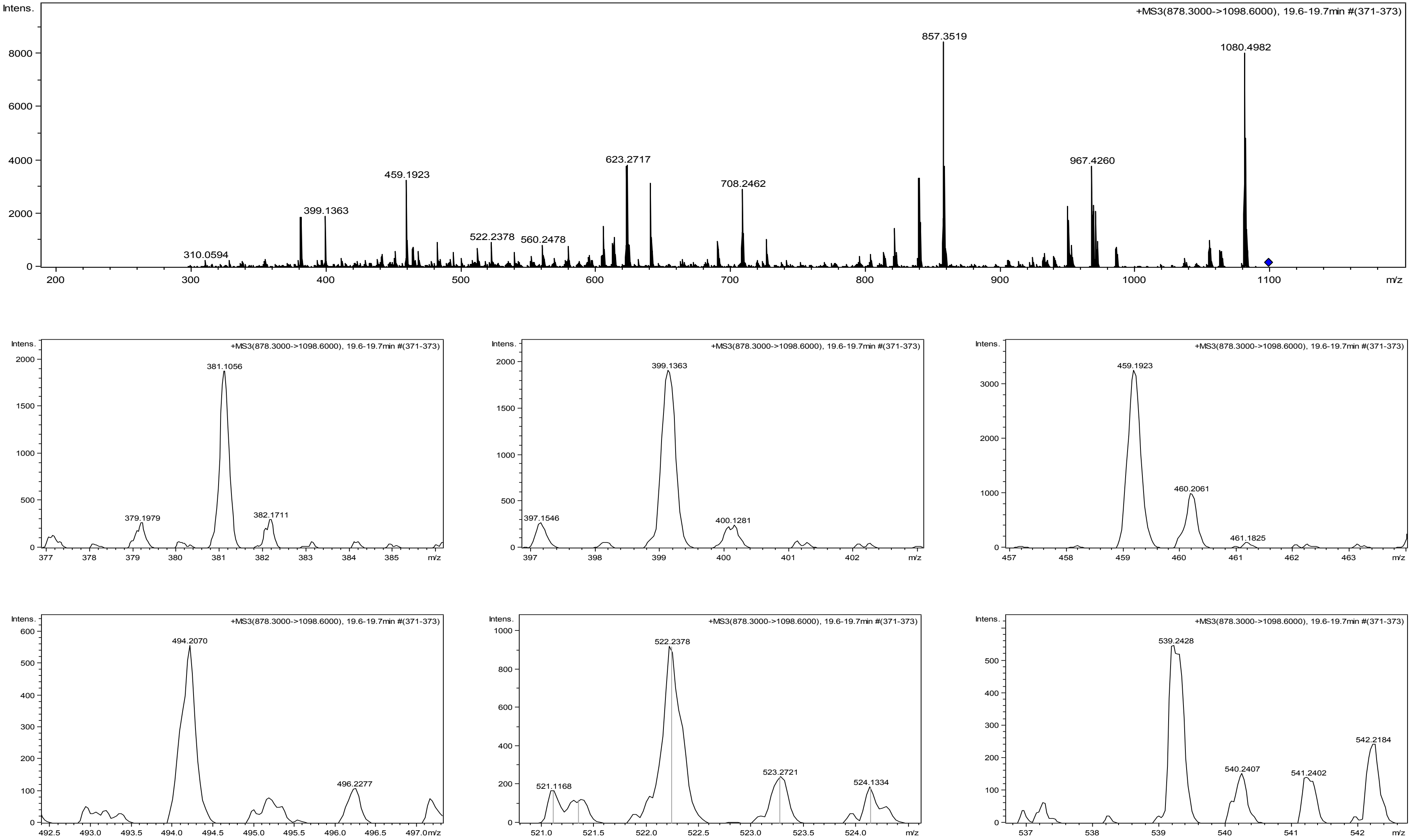

Fraction 15

877.82++ → Pep [M+H]+ 1098.59+ [19.6-20.0 min]   CID-MS3

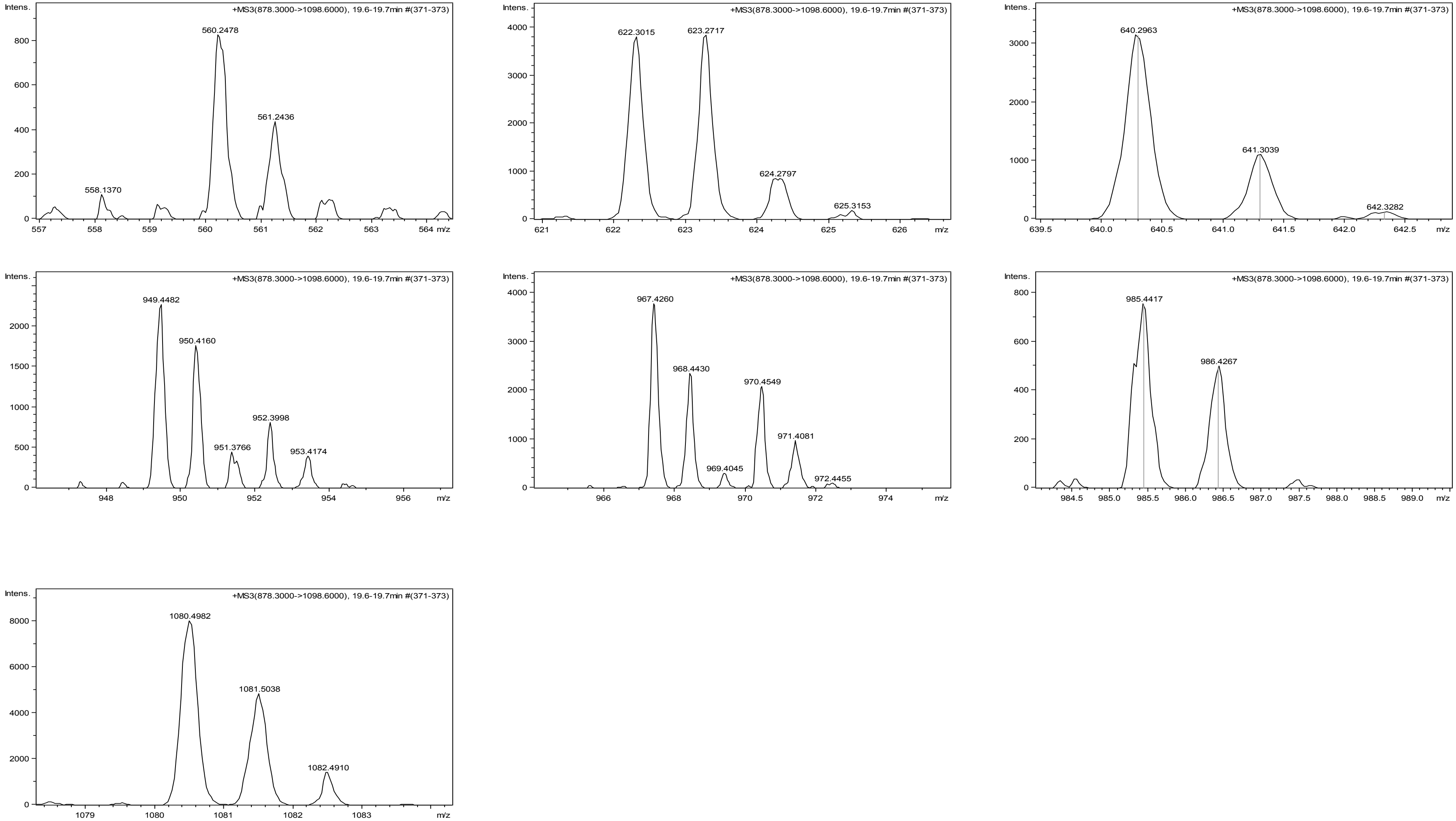

Fraction 15

877.82++ → Pep [M+H]+ 1098.59+ [19.6-20.0 min]

CID-MS3 MASCOT Search

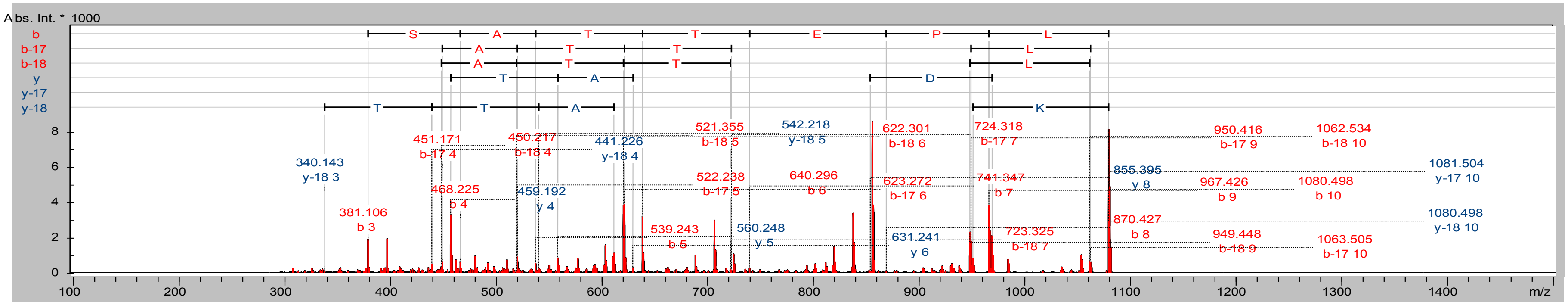

	K	D	H	S	A	T	T	E	P	L	Lys	Asp	His	Ser	Ala	Thr	Thr	Glu	Pro	Leu
Ion	1	2	3	4	5	6	7	8	9	10	1	2	3	4	5	6	7	8	9	10
b	K	D	H	S	A	T	T	E	P	L	129.102	244.129	381.188	468.220	539.257	640.305	741.353	870.395	967.448	1080.532
b-17	K	D	H	S	A	T	T	E	P	L	112.076	227.103	364.162	451.194	522.231	623.278	724.326	853.369	950.421	1063.505
b-18	K	D	H	S	A	T	T	E	P	L	-	226.119	363.178	450.210	521.247	622.294	723.342	852.385	949.437	1062.521
y	K	D	H	S	A	T	T	E	P	L	132.102	229.155	358.197	459.245	560.293	631.330	718.362	855.421	970.448	1098.543
y-17	K	D	H	S	A	T	T	E	P	L	-	-	-	-	-	-	-	-	-	1081.516
y-18	K	D	H	S	A	T	T	E	P	L	-	-	340.187	441.234	542.282	613.319	700.351	837.410	952.437	1080.532
	10	9	8	7	6	5	4	3	2	1	Leu	Pro	Glu	Thr	Thr	Ala	Ser	His	Asp	Lys

unknown O-glycosylation site

Tau-Tubulin Kinase 2

$_{814}$KDH**S**A**TT**EPL$_{823}$

Fraction 15

877.82++ → Pep [M+H]+ 1098.59+ [19.6-20.0 min]

CID-MS3 MASCOT Search

prot_hit_nur	prot_acc	prot_desc	prot_score	prot_mass	prot_matche	pep_query	pep_rank	pep_isbold	pep_exp_mz	pep_exp_mr	pep_exp_z	pep_calc_mr	pep_delta	pep_miss	pep_score	pep_expect	pep_res_bef	pep_seq
1	GRPR_HUMAN	Gastrin-releasing peptide recepto	13	43969	1	1	4	1	1098.5854	1097.5781	1	1097.706	-0.1278	0	17.52	2.60E+02	G	LIGNITLIKI
2	OR5R1_HUMAN	Olfactory receptor 5R1 (Olfactory	13	37424	1	1	4	0	1098.5854	1097.5781	1	1097.706	-0.1278	0	17.52	2.60E+02	G	NLGLITLIKI
3	TTBK2_HUMAN	Tau-tubulin kinase 2 (EC 2.7.11.1)	13	138240	1	1	1	0	1098.5854	1097.5781	1	1097.5353	0.0428	0	19.55	1.60E+02	E	KDHSATTEPL
4	OSBL9_HUMAN	Oxysterol-binding protein-relate	12	83817	1	1	2	0	1098.5854	1097.5781	1	1097.6696	-0.0914	0	17.73	2.50E+02	L	QILIEQLKL
5	STS2_HUMAN	Suppressor of T-cell receptor sign	12	75273	1	1	2	0	1098.5854	1097.5781	1	1097.7059	-0.1278	0	17.73	2.50E+02	A	KLILEELKL
6	DONS_HUMAN	Protein downstream neighbor of	11	63050	1	1	6	0	1098.5854	1097.5781	1	1096.6128	0.9653	0	17.39	2.70E+02	L	EQLSQIPLLG
7	AYTL1_HUMAN	Acyltransferase-like 1 (EC 2.3.1.-)	10	60797	1	1	10	0	1098.5854	1097.5781	1	1097.6233	-0.0452	0	15.45	4.20E+02	A	LKHPEYAKI
8	ARHGC_HUMAN	Rho guanine nucleotide exchange	10	174094	1	1	7	0	1098.5854	1097.5781	1	1097.5254	0.0527	0	17.27	2.80E+02	N	IKAYHSGEGH
9	SACS_HUMAN	Sacsin - Homo sapiens (Human)	8	526497	1	1	8	0	1098.5854	1097.5781	1	1097.6961	-0.118	0	17.22	2.80E+02	N	LKLFHSLKL
10	HUWE1_HUMAN	HECT, UBA and WWE domain-cont	7	485523	1	1	9	0	1098.5854	1097.5781	1	1097.6332	-0.0551	0	16.65	3.20E+02	E	QLLLELQQI

Biotools-Score: 313

MASCOT-Score: 22

unknown O-glycosylation site

Tau-Tubulin Kinase 2

$_{814}$KDHSATTEPL$_{823}$

Fraction 15

877.82++ → Pep [M+H]+ 1098.59+ [19.6-20.0 min] ETD

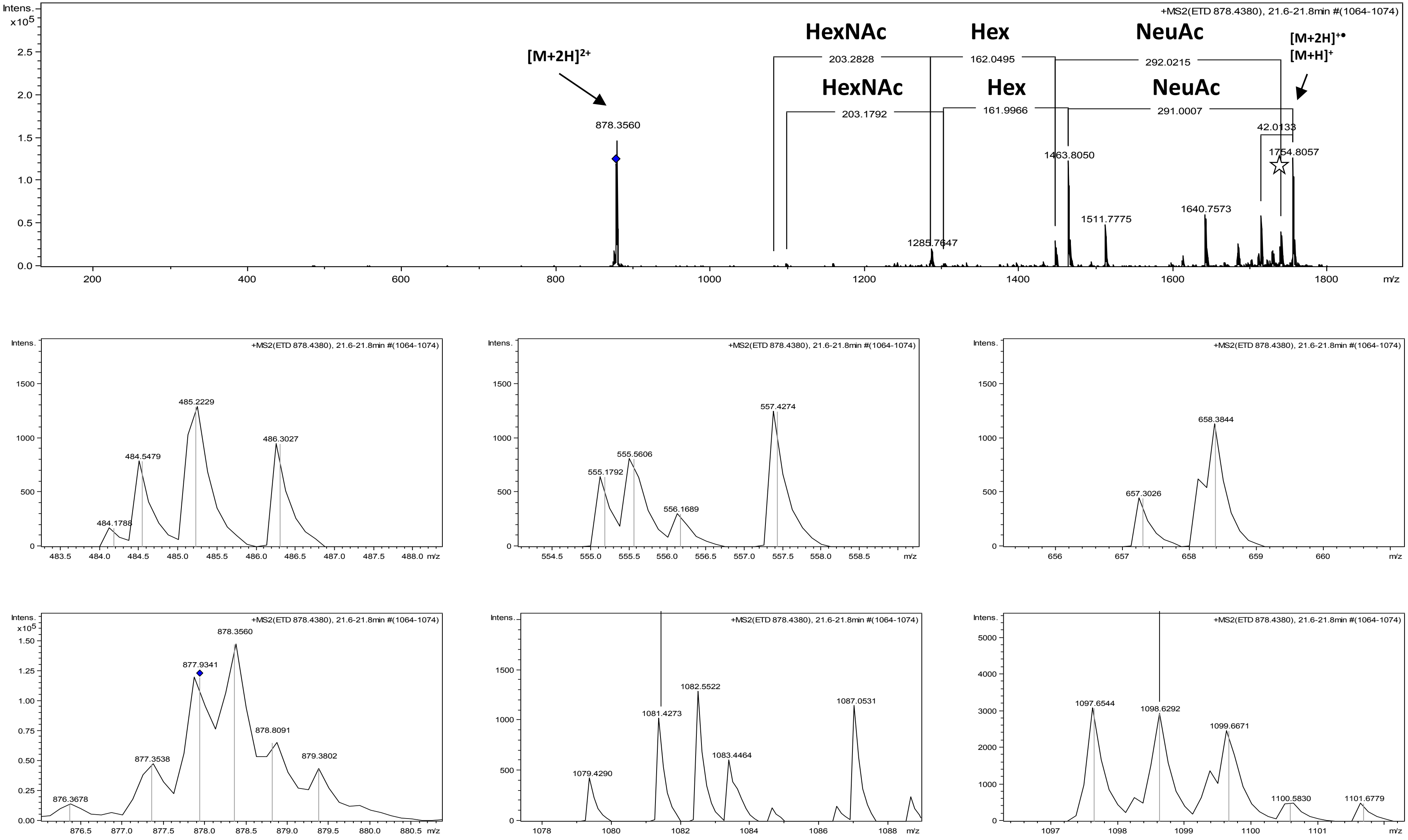

Fraction 15

877.82++ → Pep [M+H]+ 1098.59+ [19.6-20.0 min]

ETD

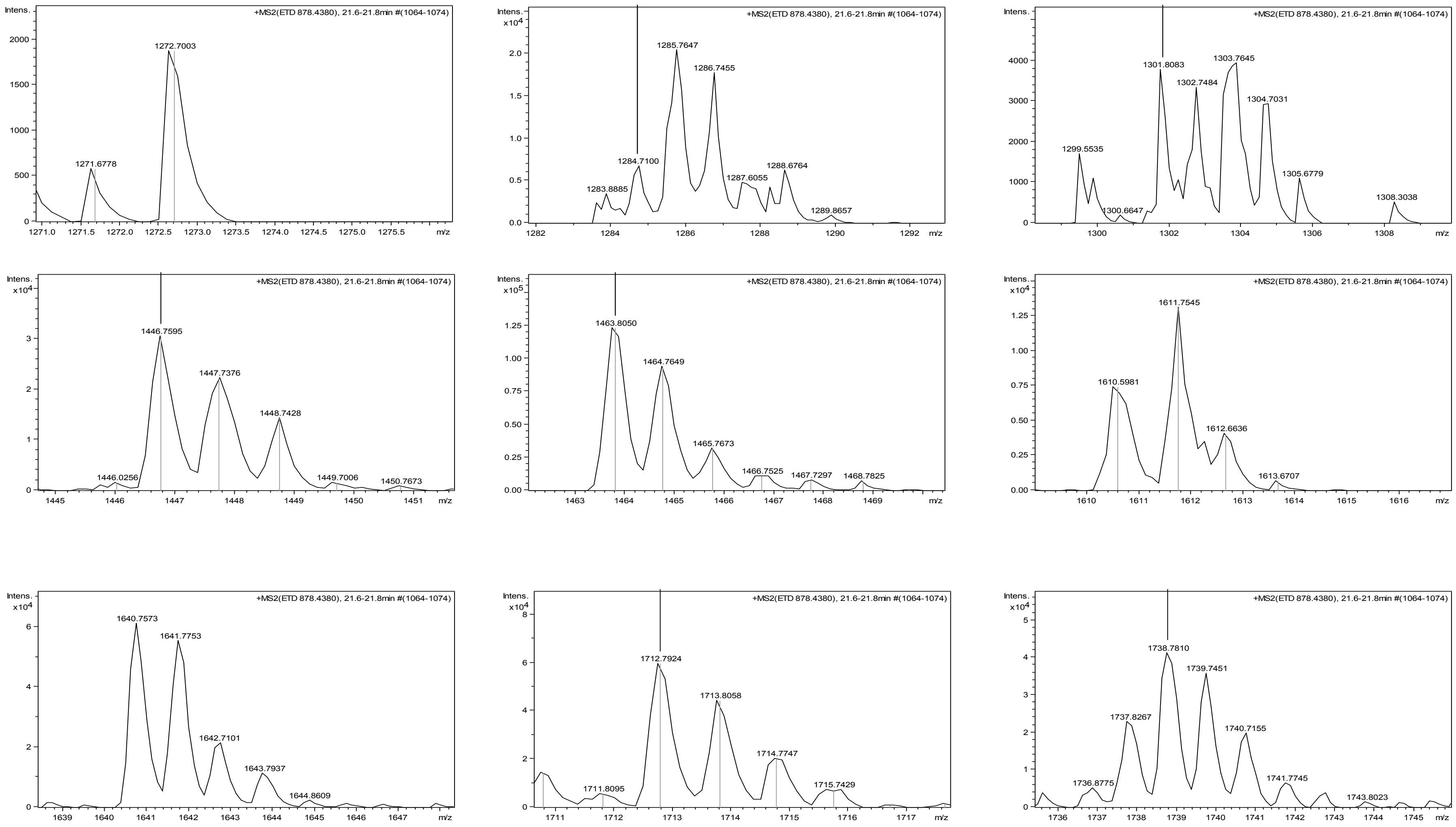

Fraction 15

877.82++ → Pep [M+H]+ 1098.59+ [19.6-20.0 min] ETD

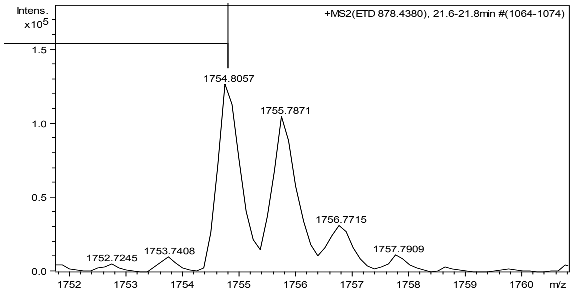

Fraction 15

877.82++ → Pep [M+H]+ 1098.59+ [19.6-20.0 min] ETD

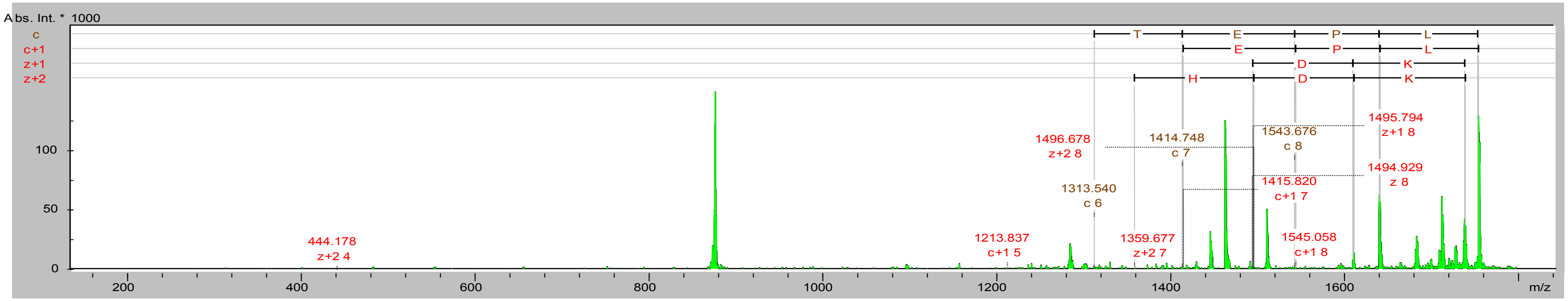

KDHSATTEPL

	K	D	H	S	A	T	T	E	P	L	Lys	Asp	His	Ser	Ala	Thr	Thr	Glu	Pro	Leu
Ion	1	2	3	4	5	6	7	8	9	10	1	2	3	4	5	6	7	8	9	10
c	K	D	H	S*	A	T	T	E	P	L	146.129	261.156	398.215	1141.474	1212.511	1313.559	1414.607	1543.649	1640.702	1753.786
c+1	K	D	H	S*	A	T	T	E	P	L	147.137	262.164	399.222	1142.482	1213.519	1314.567	1415.615	1544.657	1641.710	1754.794
z	K	D	H	S*	A	T	T	E	P	L	115.075	212.128	341.171	442.218	543.266	614.303	1357.563	1494.622	1609.649	1737.744
z+1	K	D	H	S*	A	T	T	E	P	L	116.083	213.136	342.179	443.226	544.274	615.311	1358.571	1495.630	1610.657	1738.751
z+2	K	D	H	S*	A	T	T	E	P	L	117.091	214.144	343.186	444.234	545.282	616.319	1359.578	1496.637	1611.664	1739.759
	10	9	8	7	6	5	4	3	2	1	Leu	Pro	Glu	Thr	Thr	Ala	Ser	His	Asp	Lys

Biotools-Score: 72

unknown O-glycosylation site

Tau-Tubulin Kinase 2

$_{814}$KDHSATTEPL$_{823}$

Fraction 15

877.82++ → Pep [M+H]+ 1098.59+ [19.6-20.0 min] ETD

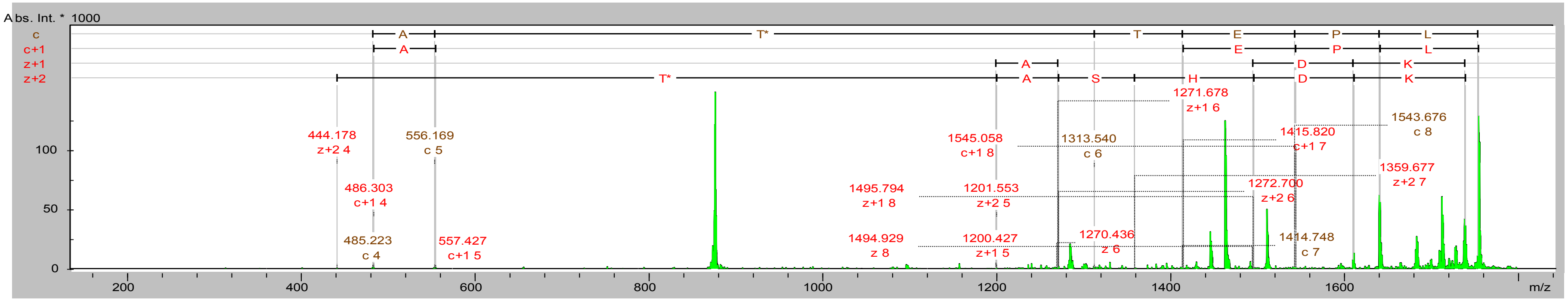

KDHSATTEPL

	K	D	H	S	A	T	T	E	P	L	Lys	Asp	His	Ser	Ala	Thr	Thr	Glu	Pro	Leu
Ion	1	2	3	4	5	6	7	8	9	10	1	2	3	4	5	6	7	8	9	10
c	K	D	H	S	A	T*	T	E	P	L	146.129	261.156	398.215	485.247	556.284	1313.559	1414.607	1543.649	1640.702	1753.786
c+1	K	D	H	S	A	T*	T	E	P	L	147.137	262.164	399.222	486.254	557.292	1314.567	1415.615	1544.657	1641.710	1754.794
z	K	D	H	S	A	T*	T	E	P	L	115.075	212.128	341.171	442.218	1199.494	1270.531	1357.563	1494.622	1609.649	1737.744
z+1	K	D	H	S	A	T*	T	E	P	L	116.083	213.136	342.179	443.226	1200.502	1271.539	1358.571	1495.630	1610.657	1738.751
z+2	K	D	H	S	A	T*	T	E	P	L	117.091	214.144	343.186	444.234	1201.509	1272.546	1359.578	1496.637	1611.664	1739.759
	10	9	8	7	6	5	4	3	2	1	Leu	Pro	Glu	Thr	Thr	Ala	Ser	His	Asp	Lys

Biotools-Score: 285

unknown O-glycosylation site

Tau-Tubulin Kinase 2

$_{814}$KDHSATTEPL$_{823}$

Fraction 15

877.82++ → Pep [M+H]+ 1098.59+ [19.6-20.0 min] ETD

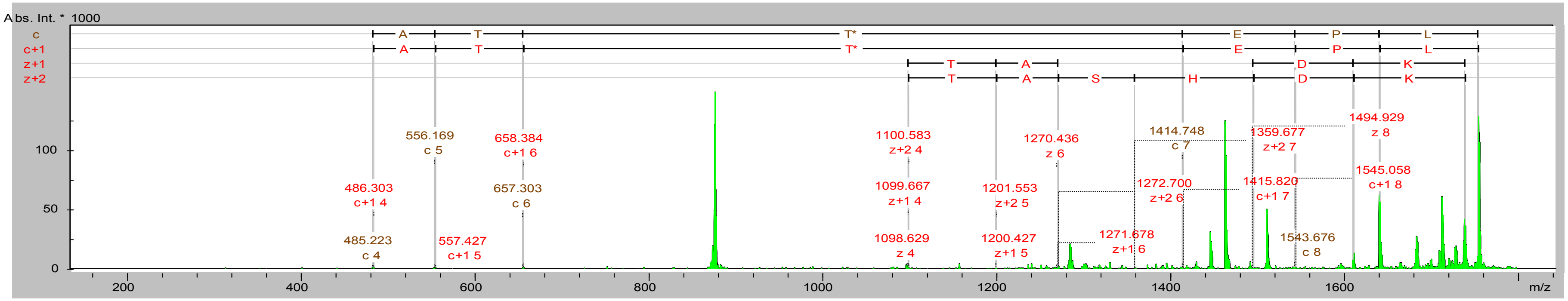

KDHSATTEPL **Most likely**

	K	D	H	S	A	T	T	E	P	L	Lys	Asp	His	Ser	Ala	Thr	Thr	Glu	Pro	Leu
Ion	1	2	3	4	5	6	7	8	9	10	1	2	3	4	5	6	7	8	9	10
c	K	D	H	S	A	T	T*	E	P	L	146.129	261.156	398.215	485.247	556.284	657.331	1414.607	1543.649	1640.702	1753.786
c+1	K	D	H	S	A	T	T*	E	P	L	147.137	262.164	399.222	486.254	557.292	658.339	1415.615	1544.657	1641.710	1754.794
z	K	D	H	S	A	T	T*	E	P	L	115.075	212.128	341.171	1098.446	1199.494	1270.531	1357.563	1494.622	1609.649	1737.744
z+1	K	D	H	S	A	T	T*	E	P	L	116.083	213.136	342.179	1099.454	1200.502	1271.539	1358.571	1495.630	1610.657	1738.751
z+2	K	D	H	S	A	T	T*	E	P	L	117.091	214.144	343.186	1100.462	1201.509	1272.546	1359.578	1496.637	1611.664	1739.759
	10	9	8	7	6	5	4	3	2	1	Leu	Pro	Glu	Thr	Thr	Ala	Ser	His	Asp	Lys

Biotools-Score: 399

unknown O-glycosylation site

Tau-Tubulin Kinase 2

$_{814}$KDH**SA**TTEPL$_{823}$

Fraction 15

741.80++ → Pep [M+H]+ 826.38+ [20.1-20.4 min] CID-MS Precursor

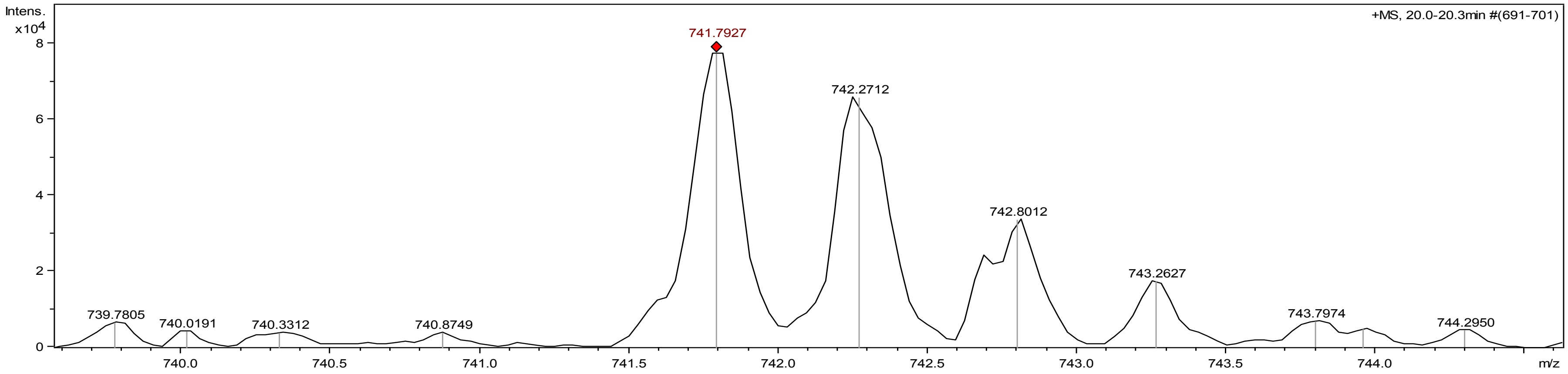

Fraction 15

741.80++ → Pep [M+H]+ 826.38+ [20.1-20.4 min] CID-MS2

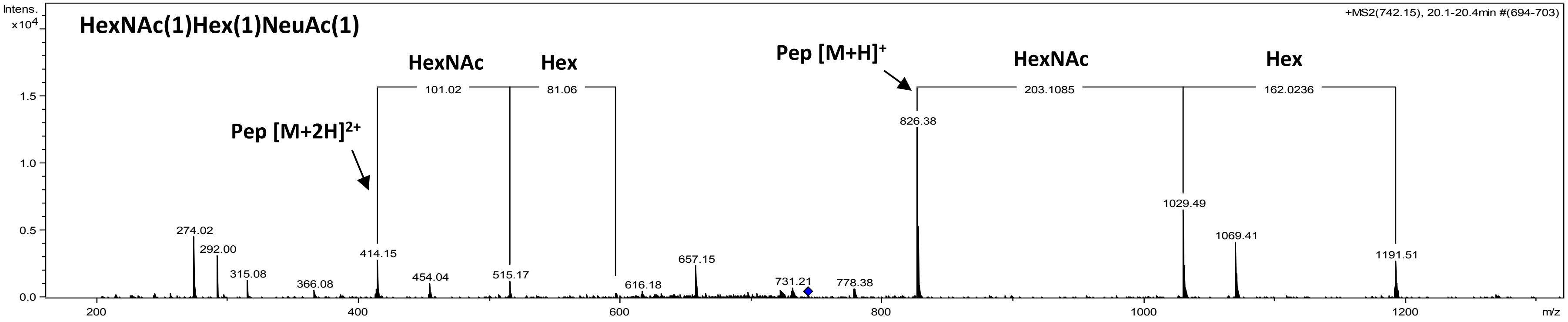

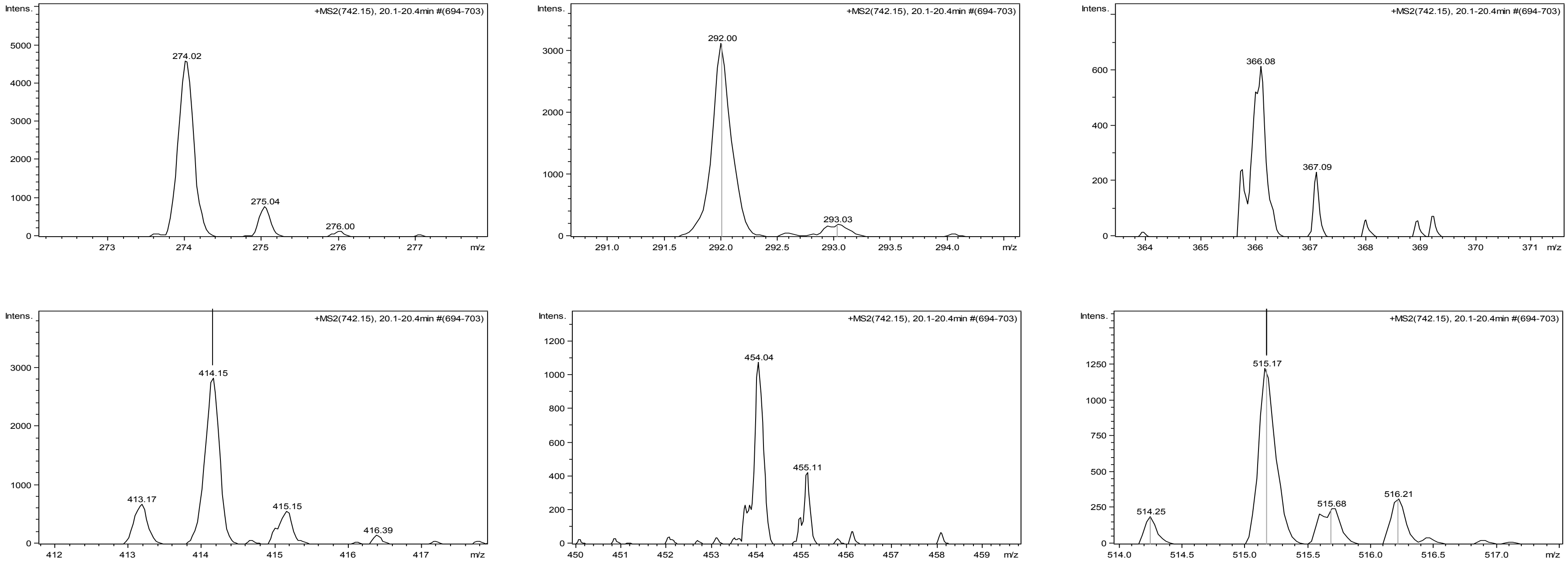

Fraction 15

741.80++ → Pep [M+H]+ 826.38+ [20.1-20.4 min]

CID-MS2

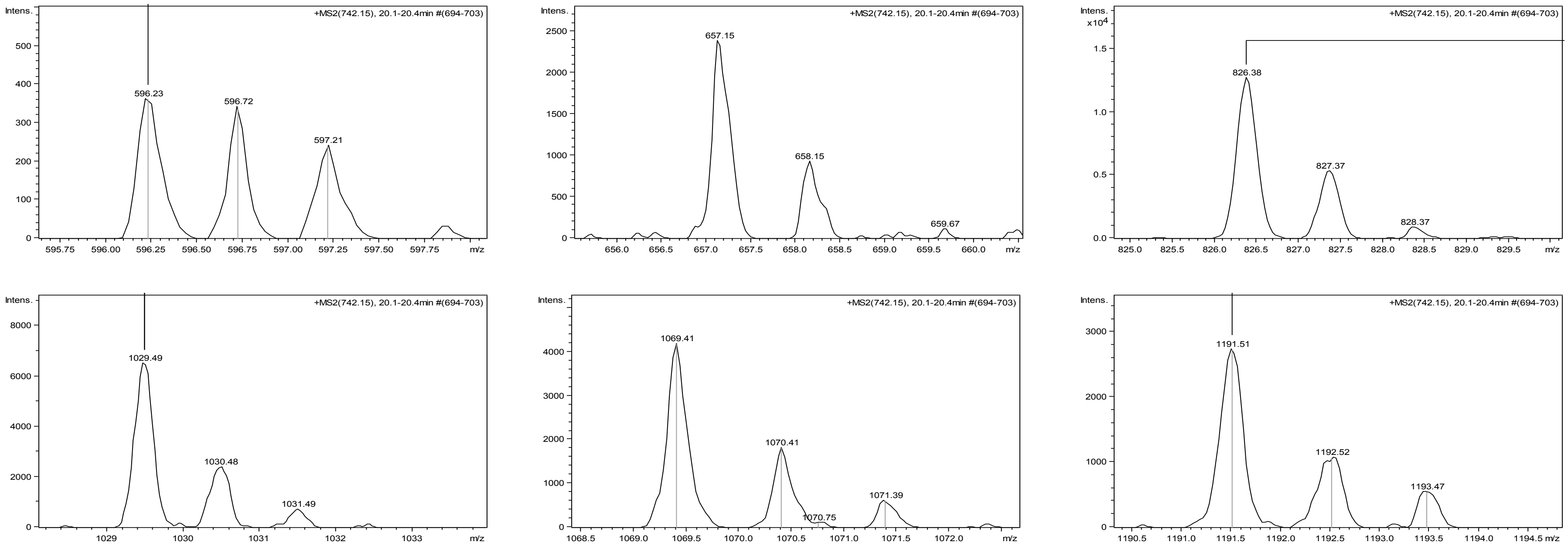

Fraction 15

741.80++ → Pep [M+H]+ 826.38+ [20.1-20.4 min] CID-MS3

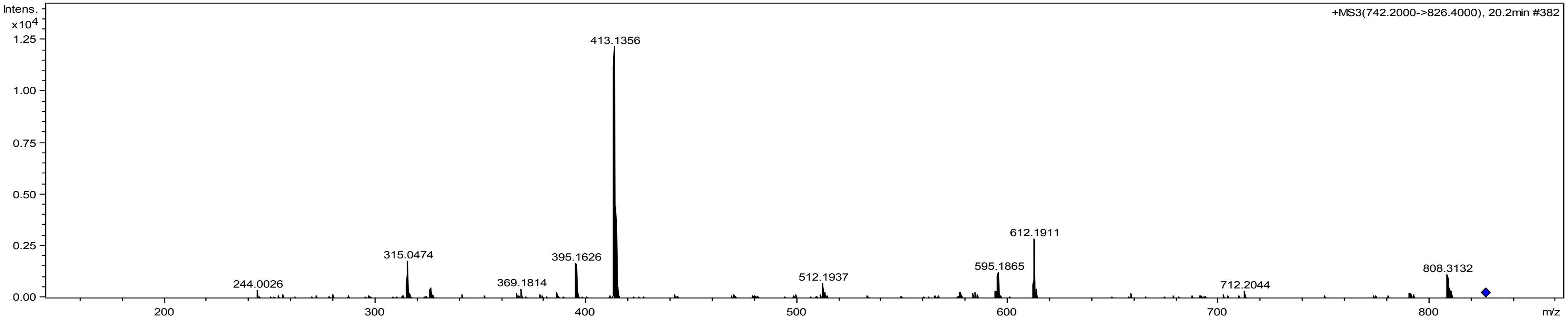

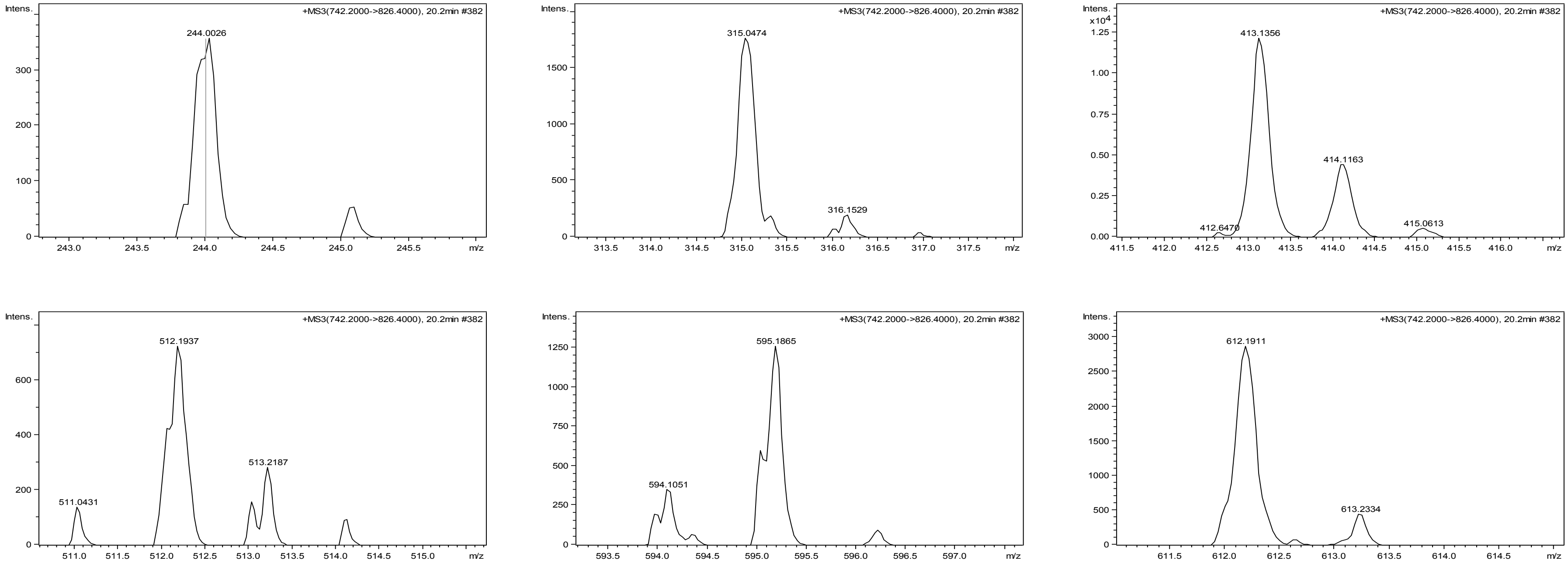

Fraction 15

741.80++ → Pep [M+H]+ 826.38+ [20.1-20.4 min] CID-MS3

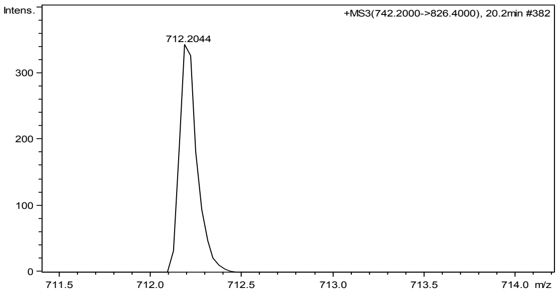

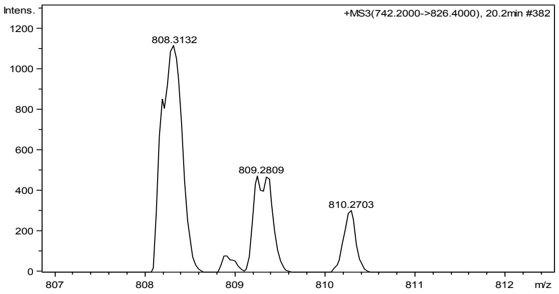

Fraction 15

741.80++ → Pep [M+H]+ 826.38+ [20.1-20.4 min] CID-MS3 MASCOT Search

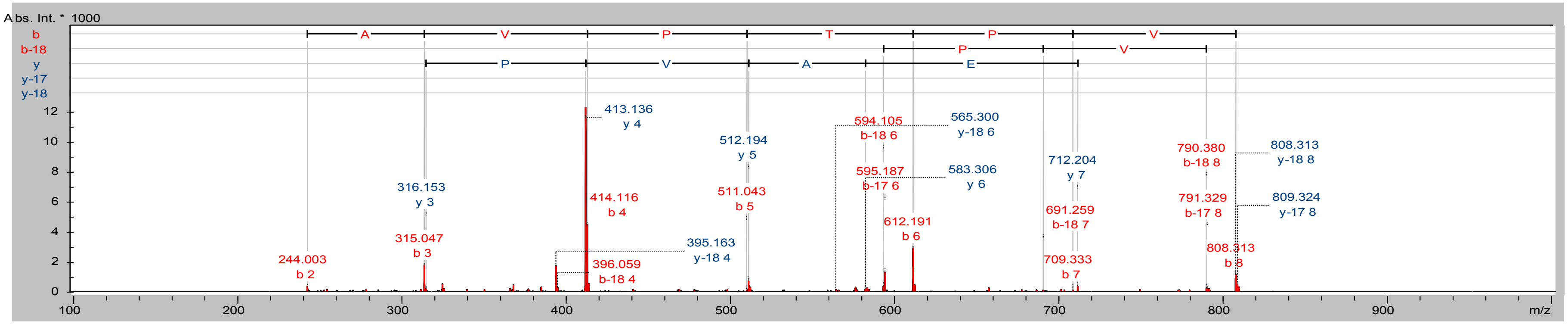

	N	E	A	V	P	T	P	V	Asn	Glu	Ala	Val	Pro	Thr	Pro	Val
Ion	1	2	3	4	5	6	7	8	1	2	3	4	5	6	7	8
b	N	E	A	V	P	T	P	V	115.050	244.093	315.130	414.198	511.251	612.299	709.352	808.420
b-17	N	E	A	V	P	T	P	V	98.024	227.066	298.103	397.172	494.225	595.272	692.325	791.393
b-18	N	E	A	V	P	T	P	V	-	226.082	297.119	396.188	493.241	594.288	691.341	790.409
y	N	E	A	V	P	T	P	V	118.086	215.139	316.187	413.239	512.308	583.345	712.388	826.431
y-17	N	E	A	V	P	T	P	V	-	-	-	-	-	-	-	809.404
y-18	N	E	A	V	P	T	P	V	-	-	298.176	395.229	494.297	565.334	694.377	808.420
	8	7	6	5	4	3	2	1	Val	Pro	Thr	Pro	Val	Ala	Glu	Asn

known O-glycosylation site

Alpha-2-HS-glycoprotein precursor $_{265}$NEAVP**T**PV$_{272}$

Fraction 15

741.80++ → Pep [M+H]+ 826.38+ [20.1-20.4 min]

CID-MS3 MASCOT Search

prot_hit_nur	prot_acc	prot_desc	prot_score	prot_mass	prot_matche	pep_query	pep_rank	pep_isbold	pep_exp_mz	pep_exp_mr	pep_exp_z	pep_calc_mr	pep_delta	pep_miss	pep_score	pep_expect	pep_res_bef	pep_seq
1	FETUA_HUM	Alpha-2-HS-g	39	40098	1	1	1	1	826.3784	825.3711	1	825.4232	-0.0521	0	43.28	0.54	A	NEAVPTPV
2	MARH1_HUN	E3 ubiquitin-	37	33200	1	1	2	0	826.3784	825.3711	1	825.496	-0.1249	0	40.58	1	I	KDAVVVPV
3	ARI4A_HUM	AT-rich inter	23	143548	1	1	3	0	826.3784	825.3711	1	825.4596	-0.0885	0	28.79	15	S	NSLVSIPP
4	SELS_HUMAI	Selenoprote	21	21274	1	1	5	0	826.3784	825.3711	1	825.3981	-0.0269	0	23.16	56	L	NAQVEKH
5	ZNF31_HUM	Zinc finger p	18	111813	1	1	5	0	826.3784	825.3711	1	825.3868	-0.0157	0	23.16	56	L	NAEVAPQP
6	ZBT16_HUM	Zinc finger ai	17	75651	1	1	5	0	826.3784	825.3711	1	825.3617	0.0094	0	23.16	56	D	NEAVEQH
7	RN123_HUM	E3 ubiquitin-	16	149960	1	1	5	0	826.3784	825.3711	1	825.3981	-0.0269	0	23.16	56	Y	NLSVHQQ
8	WNK1_HUM	Serine/threc	16	251457	1	1	4	0	826.3784	825.3711	1	825.4596	-0.0885	0	23.38	53	A	TQPSVVPV
9	K0100_HUM	UPF0378 fam	15	256105	1	1	5	0	826.3784	825.3711	1	825.4345	-0.0633	0	23.16	56	V	GGISVKEH

Biotools-Score: 158

MASCOT-Score: 43

known O-glycosylation site

Alpha-2-HS-glycoprotein precursor

$_{265}$NEAVPTPV$_{272}$

Fraction 15

741.80++ → Pep [M+H]+ 826.38+ [20.1-20.4 min] ETD

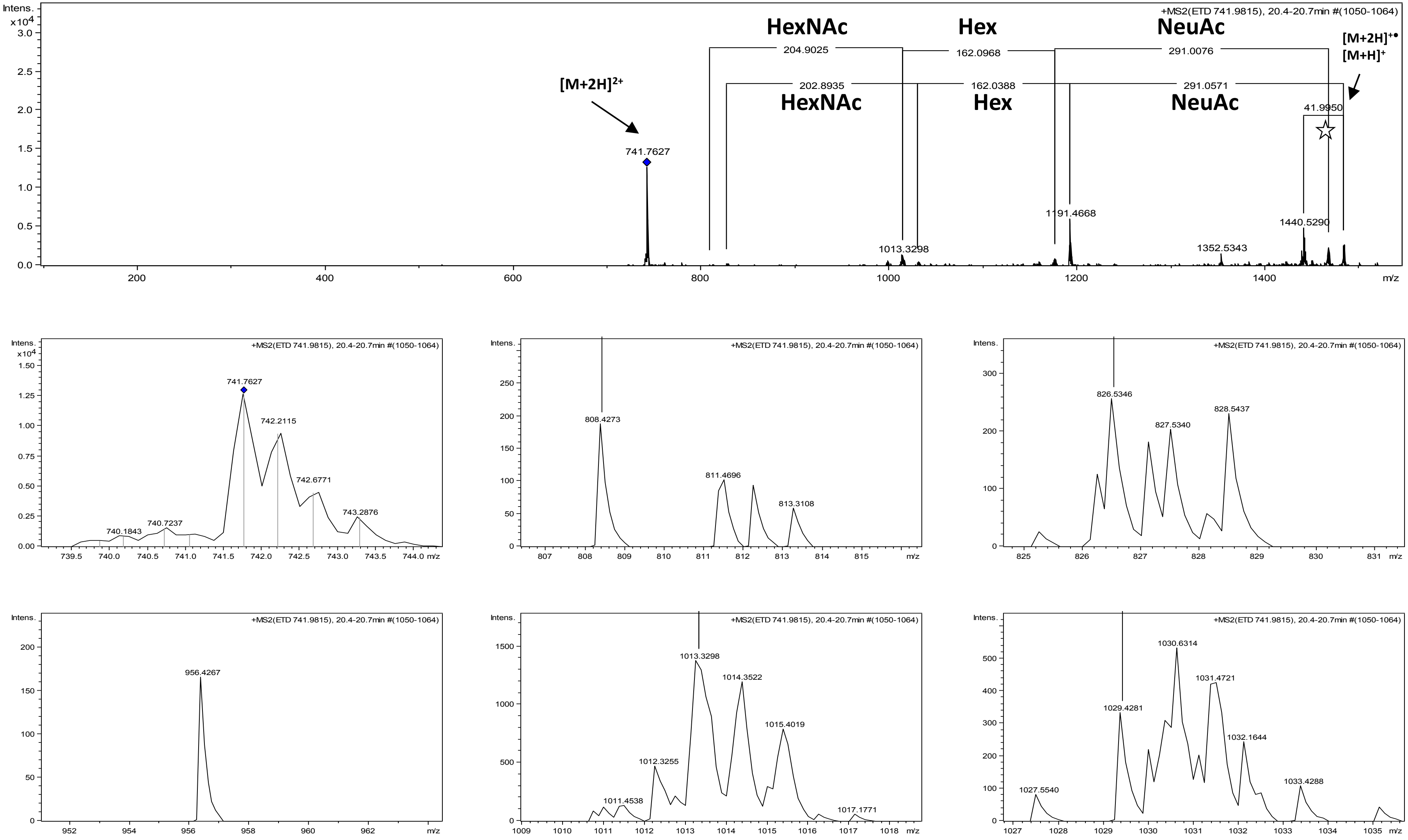

Fraction 15

741.80++ → Pep [M+H]+ 826.38+ [20.1-20.4 min] ETD

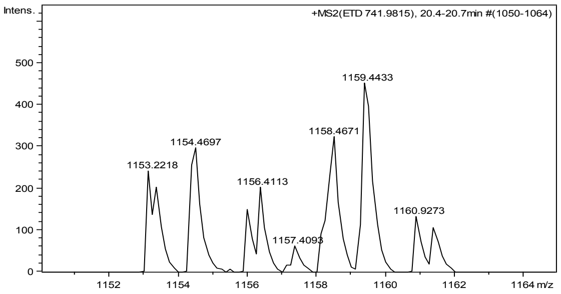

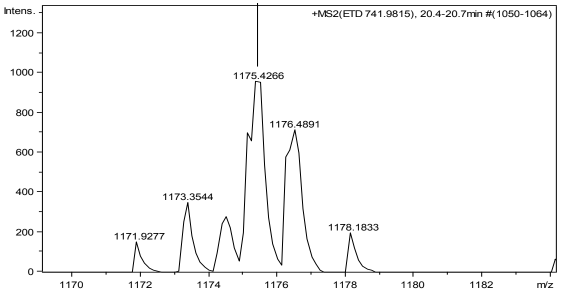

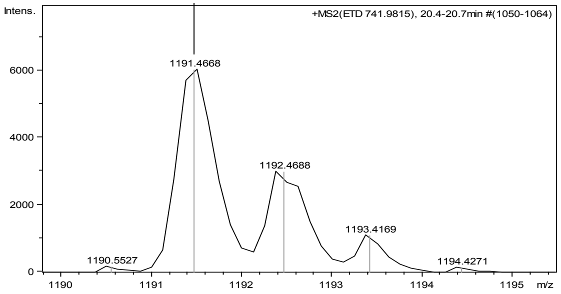

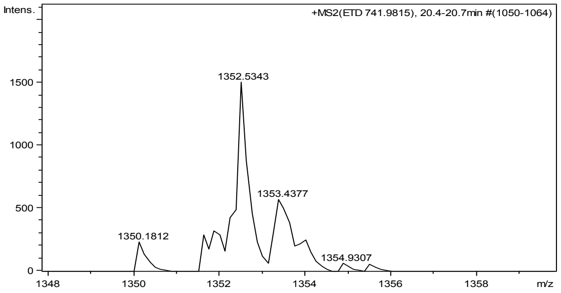

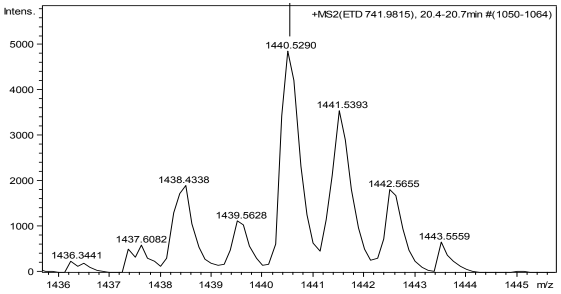

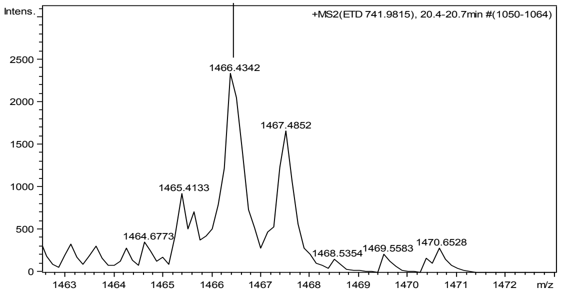

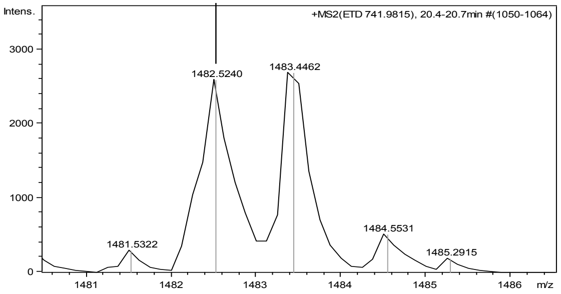

Fraction 15

741.80++ → Pep [M+H]+ 826.38+ [20.1-20.4 min] ETD

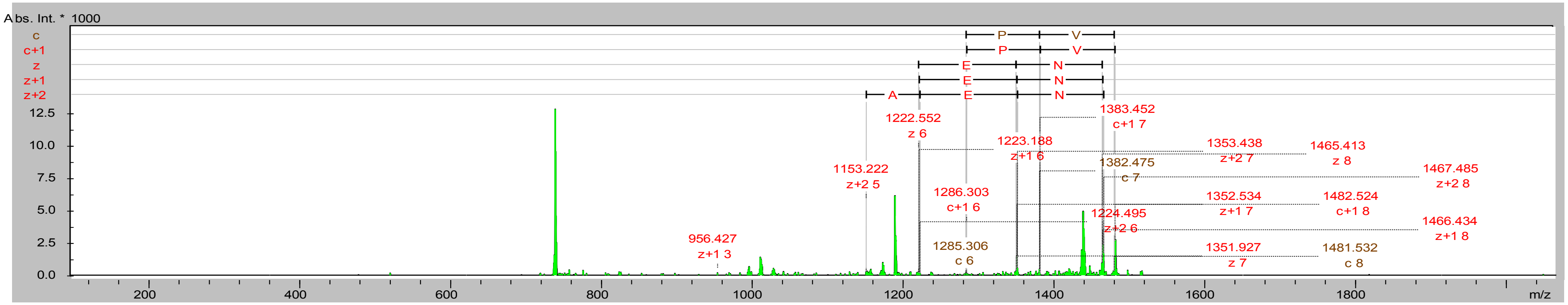

	N	E	A	V	P	T	P	V	Asn	Glu	Ala	Val	Pro	Thr	Pro	Val
Ion	1	2	3	4	5	6	7	8	1	2	3	4	5	6	7	8
c	N	E	A	V	P	T*	P	V	132.077	261.119	332.156	431.225	528.278	1285.553	1382.606	1481.674
c+1	N	E	A	V	P	T*	P	V	133.085	262.127	333.164	432.233	529.285	1286.561	1383.614	1482.682
z	N	E	A	V	P	T*	P	V	101.060	198.112	955.388	1052.441	1151.509	1222.546	1351.589	1465.632
z+1	N	E	A	V	P	T*	P	V	102.068	199.120	956.396	1053.448	1152.517	1223.554	1352.596	1466.639
z+2	N	E	A	V	P	T*	P	V	103.075	200.128	957.403	1054.456	1153.525	1224.562	1353.604	1467.647
	8	7	6	5	4	3	2	1	Val	Pro	Thr	Pro	Val	Ala	Glu	Asn

Biotools-Score: 44

known O-glycosylation site

Alpha-2-HS-glycoprotein precursor

$_{265}$NEAVP**T**PV$_{272}$

Fraction 15

597.58+++ → Pep [M+H]+ 1134.55+ [23.0-23.5 min] CID-MS Precursor

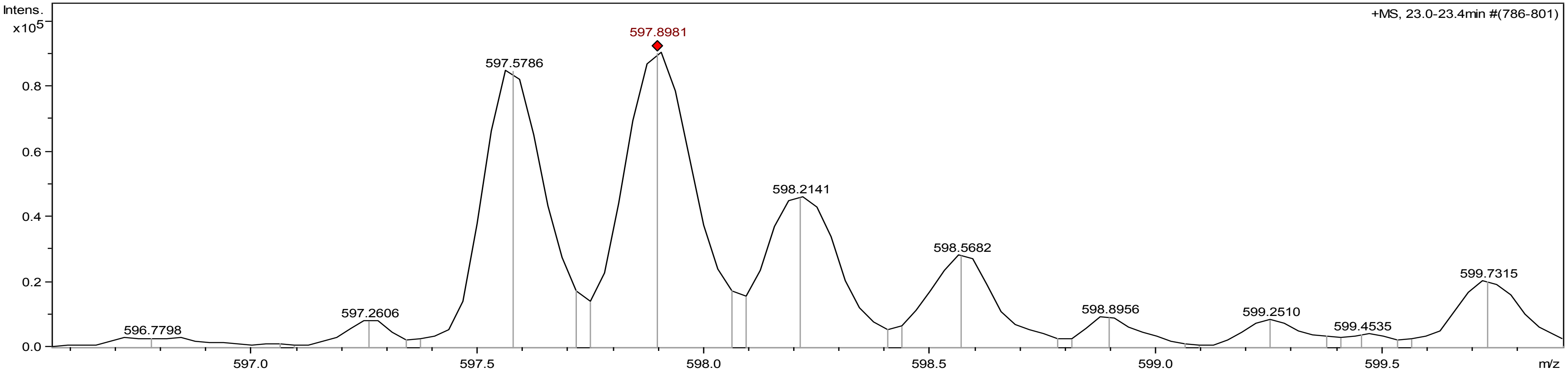

Fraction 15

597.58+++ → Pep [M+H]+ 1134.55+ [23.0-23.5 min] CID-MS2

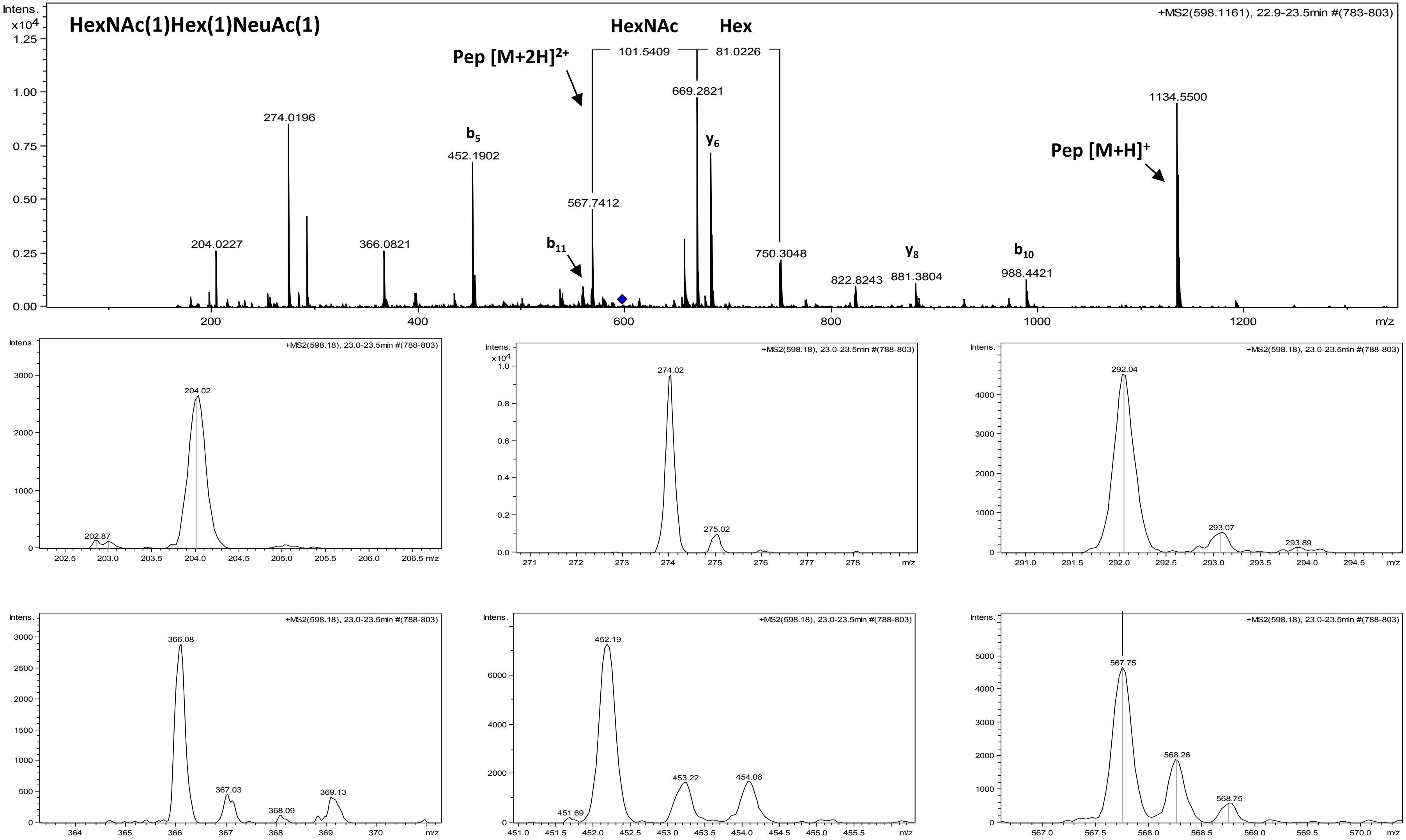

Fraction 15

597.58+++ → Pep [M+H]+ 1134.55+ [23.0-23.5 min]

CID-MS2

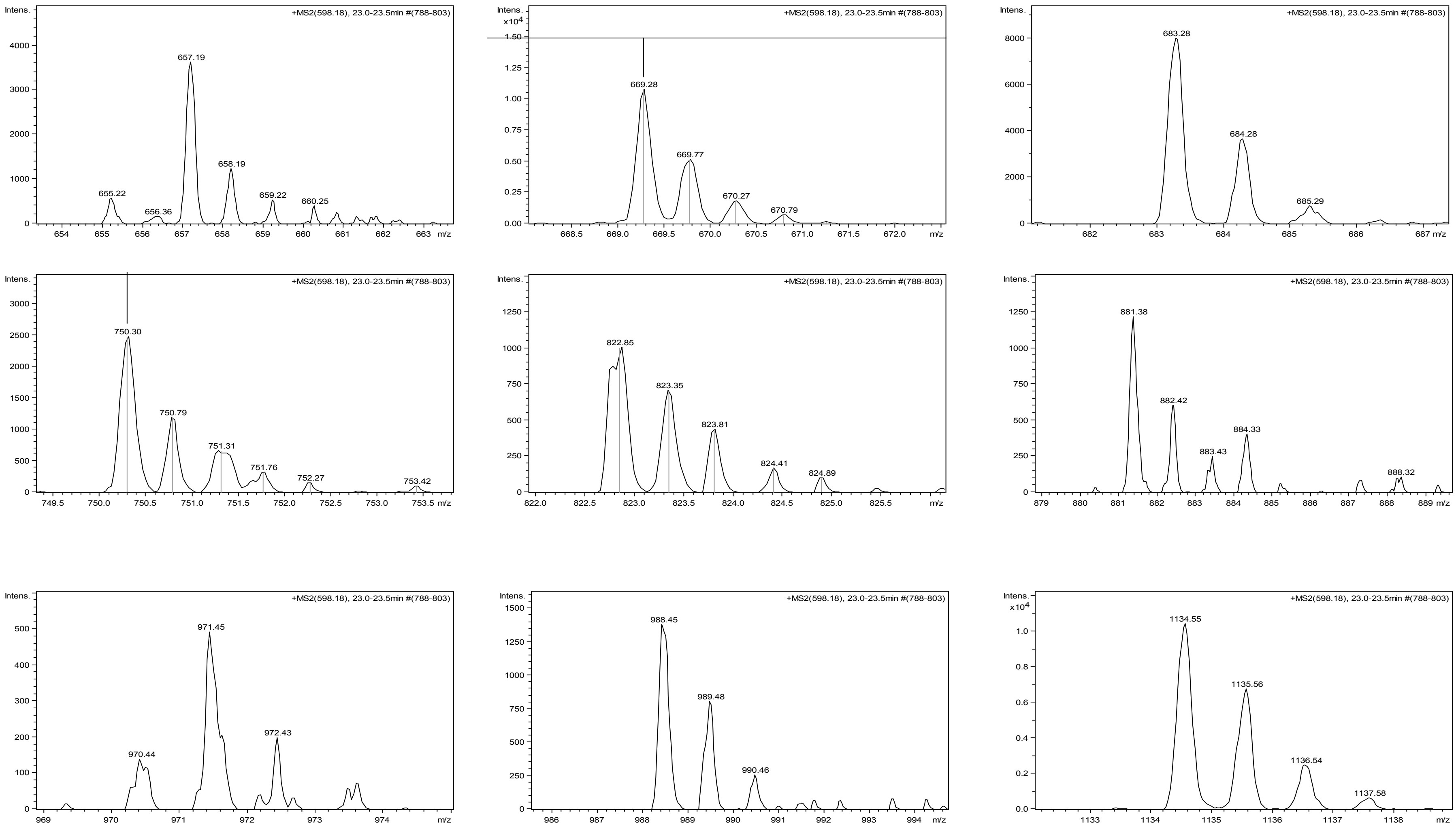

Fraction 15

597.58+++ → Pep [M+H]+ 1134.55+ [23.0-23.5 min] CID-MS3

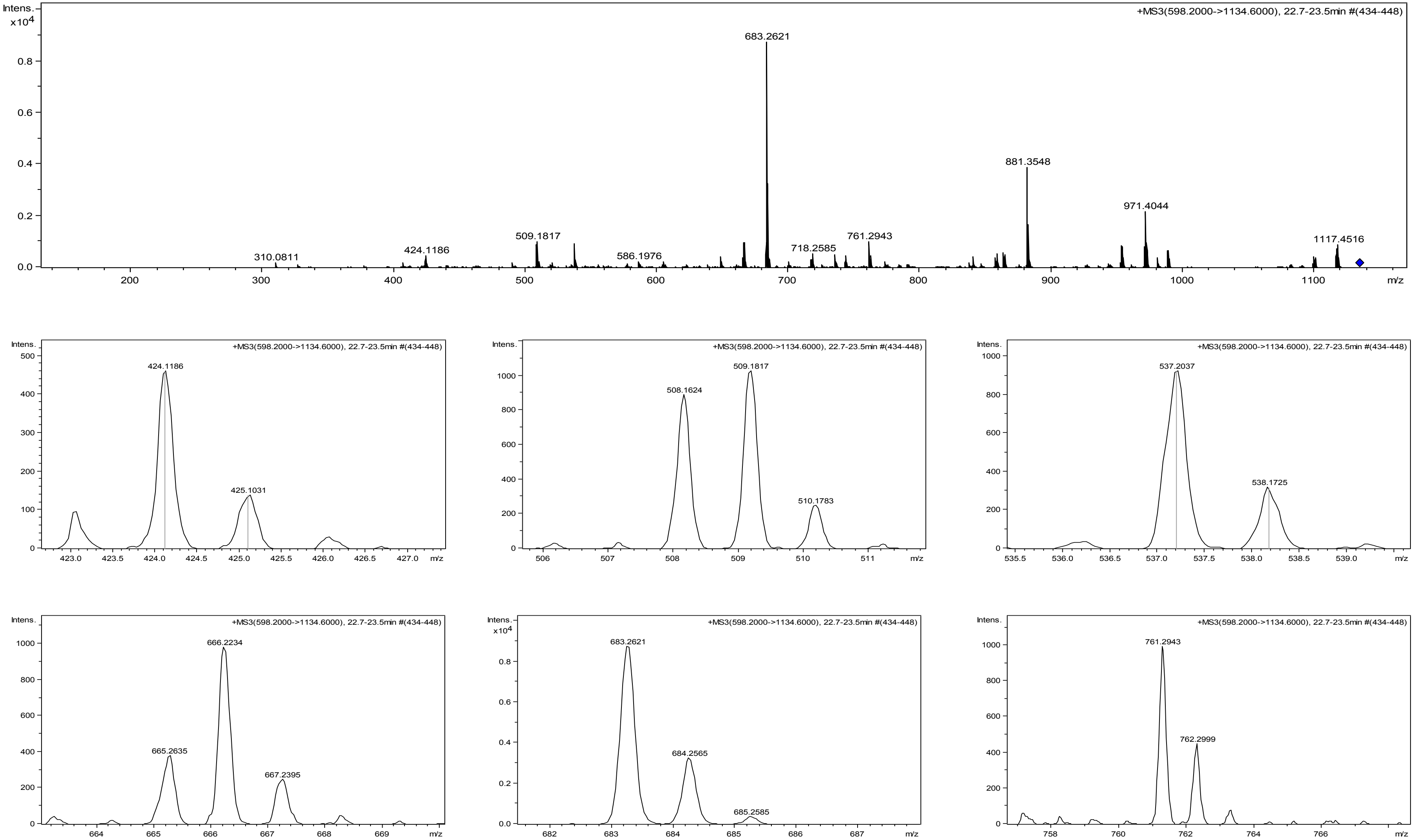

Fraction 15

597.58+++ → Pep [M+H]+ 1134.55+ [23.0-23.5 min]

CID-MS3

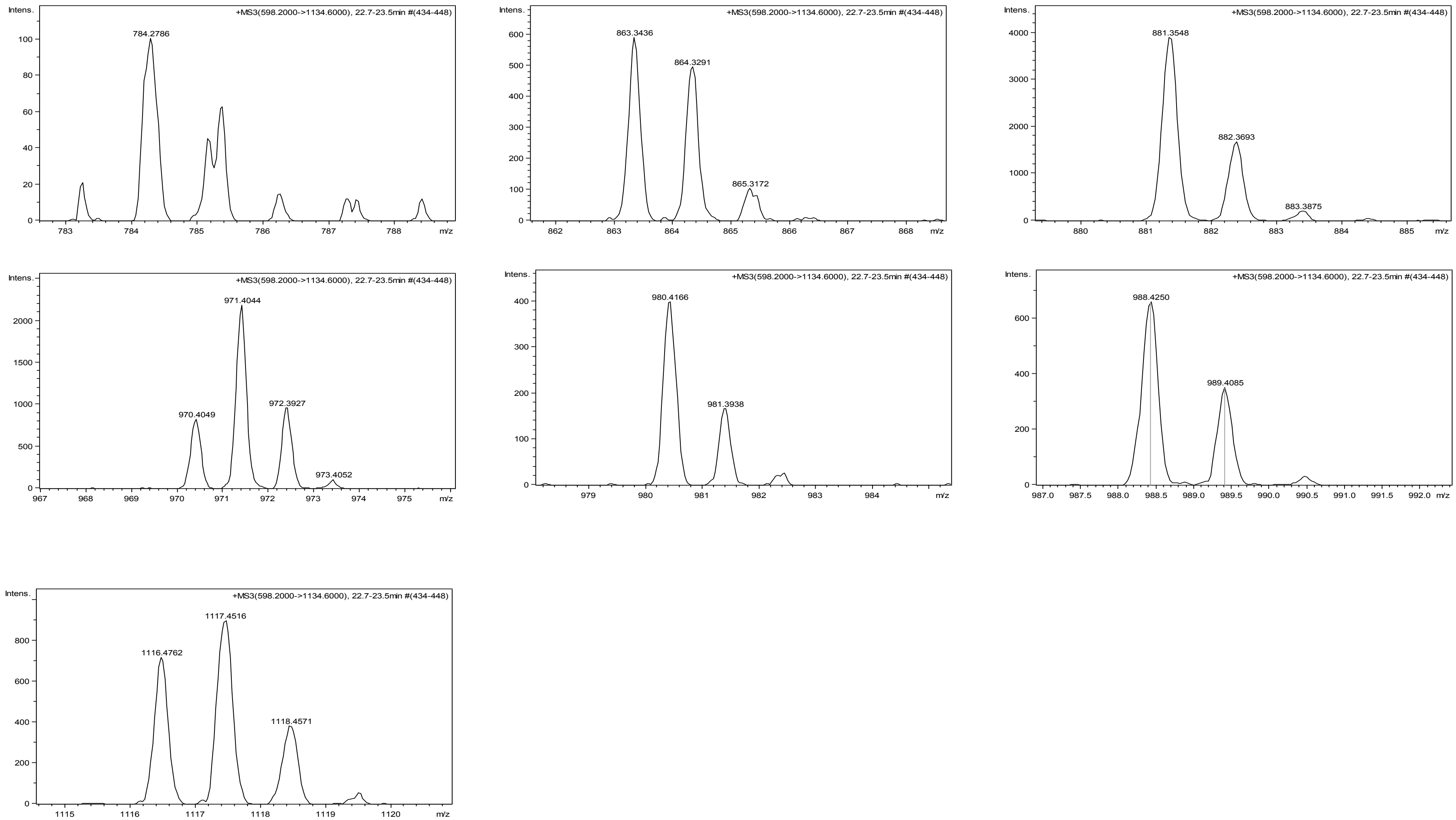

Fraction 15

597.58+++ → Pep [M+H]+ 1134.55+ [23.0-23.5 min] CID-MS3 MASCOT Search

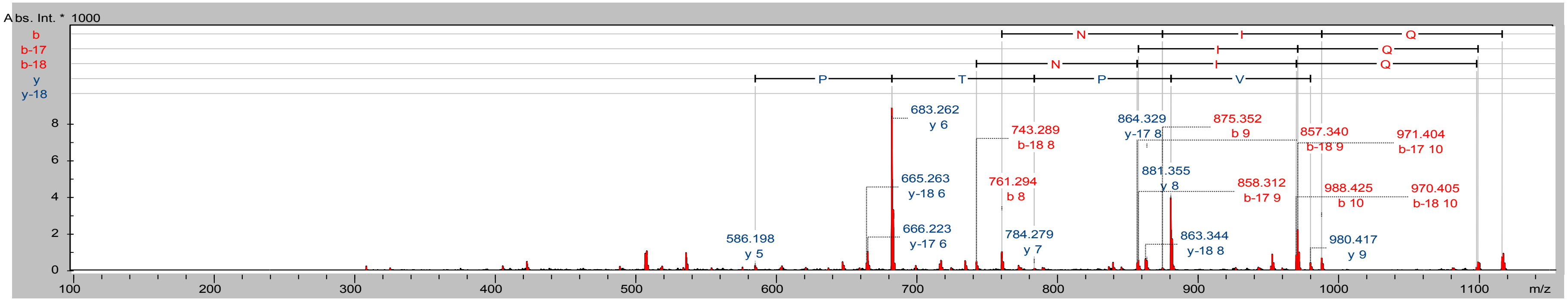

	G	P	V	P	T	P	P	D	N	I	Q	Gly	Pro	Val	Pro	Thr	Pro	Pro	Asp	Asn	Ile	Gln
Ion	1	2	3	4	5	6	7	8	9	10	11	1	2	3	4	5	6	7	8	9	10	11
b	G	P	V	P	T	P	P	D	N	I	Q	58.029	155.082	254.150	351.203	452.250	549.303	646.356	761.383	875.426	988.510	1116.568
b-17	G	P	V	P	T	P	P	D	N	I	Q	-	-	-	-	-	-	-	-	858.399	971.483	1099.542
b-18	G	P	V	P	T	P	P	D	N	I	Q	-	-	-	-	434.240	531.293	628.345	743.372	857.415	970.499	1098.558
y	G	P	V	P	T	P	P	D	N	I	Q	147.076	260.160	374.203	489.230	586.283	683.336	784.384	881.436	980.505	1077.557	1134.579
y-17	G	P	V	P	T	P	P	D	N	I	Q	130.050	243.134	357.177	472.204	569.257	666.309	767.357	864.410	963.478	1060.531	1117.552
y-18	G	P	V	P	T	P	P	D	N	I	Q	-	-	-	471.220	568.273	665.325	766.373	863.426	962.494	1059.547	1116.568
	11	10	9	8	7	6	5	4	3	2	1	Gln	Ile	Asn	Asp	Pro	Pro	Thr	Pro	Val	Pro	Gly

known O-glycosylation site

Protein AMBP

$_{20}$GPVP**T**PPDNIQ$_{30}$

Fraction 15

597.58+++ → Pep [M+H]+ 1134.55+ [23.0-23.5 min]

CID-MS3 MASCOT Search

prot_hit_nur	prot_acc	prot_desc	prot_score	prot_mass	prot_matche	pep_query	pep_rank	pep_isbold	pep_exp_mz	pep_exp_mr	pep_exp_z	pep_calc_mr	pep_delta	pep_miss	pep_score	pep_expect	pep_res_bef	pep_seq
1	AMBP_HUM	AMBP protei	6	39886	1	1	2	1	1134.55	1133.5427	1	1133.5717	-0.029	0	10.94	1.40E+03	A	GPVPTPPDN
2	K2C1_HUMA	Keratin, type	5	66149	1	1	3	0	1134.55	1133.5427	1	1133.4738	0.069	0	10.78	1.40E+03	S	YGSGSSSGGY
3	FOSL1_HUM	Fos-related a	5	29737	1	1	9	0	1134.55	1133.5427	1	1133.5869	-0.0442	0	8.24	2.50E+03	S	YPRPLTYPQ
4	TAF6_HUMA	Transcription	4	73250	1	1	4	0	1134.55	1133.5427	1	1133.6921	-0.1494	0	10.43	1.50E+03	G	PRTPGLLKVP
5	RT05_HUMA	Mitochondri	4	48489	1	1	8	0	1134.55	1133.5427	1	1133.5829	-0.0402	0	8.54	2.40E+03	L	VAVGNGKGA
6	CN101_HUM	Uncharacteri	4	80275	1	1	5	0	1134.55	1133.5427	1	1133.5393	0.0034	0	9.63	1.90E+03	L	YAQAYDLYK
7	CO8A1_HUM	Collagen alp	4	73431	1	1	1	0	1134.55	1133.5427	1	1132.5951	0.9477	0	11.03	1.30E+03	K	PLPPQIPPQ
8	DRBP1_HUM	Developmer	3	53868	1	1	7	0	1134.55	1133.5427	1	1132.4495	1.0932	0	8.64	2.30E+03	Q	QFMQFGGSS
9	MAGI1_HUM	Membrane-a	2	165510	1	1	6	0	1134.55	1133.5427	1	1133.5604	-0.0177	0	9.12	2.10E+03	Q	YLPLSAEDNL
10	KRR1_HUMA	KRR1 small s	1	43866	1	1	10	0	1134.55	1133.5427	1	1133.5717	-0.0289	0	7.33	3.10E+03	N	PRGLLEESSF

Biotools-Score: 45

MASCOT-Score: 11

known O-glycosylation site

Protein AMBP

$_{20}$GPVP**T**PPDNIQ$_{30}$

Fraction 15

597.58+++ → Pep [M+H]+ 1134.55+ [23.0-23.5 min] ETD

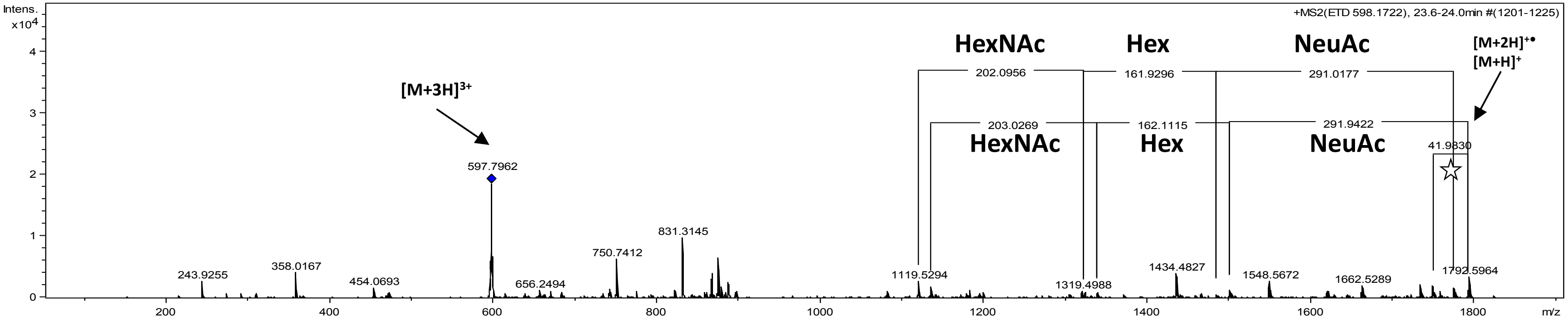

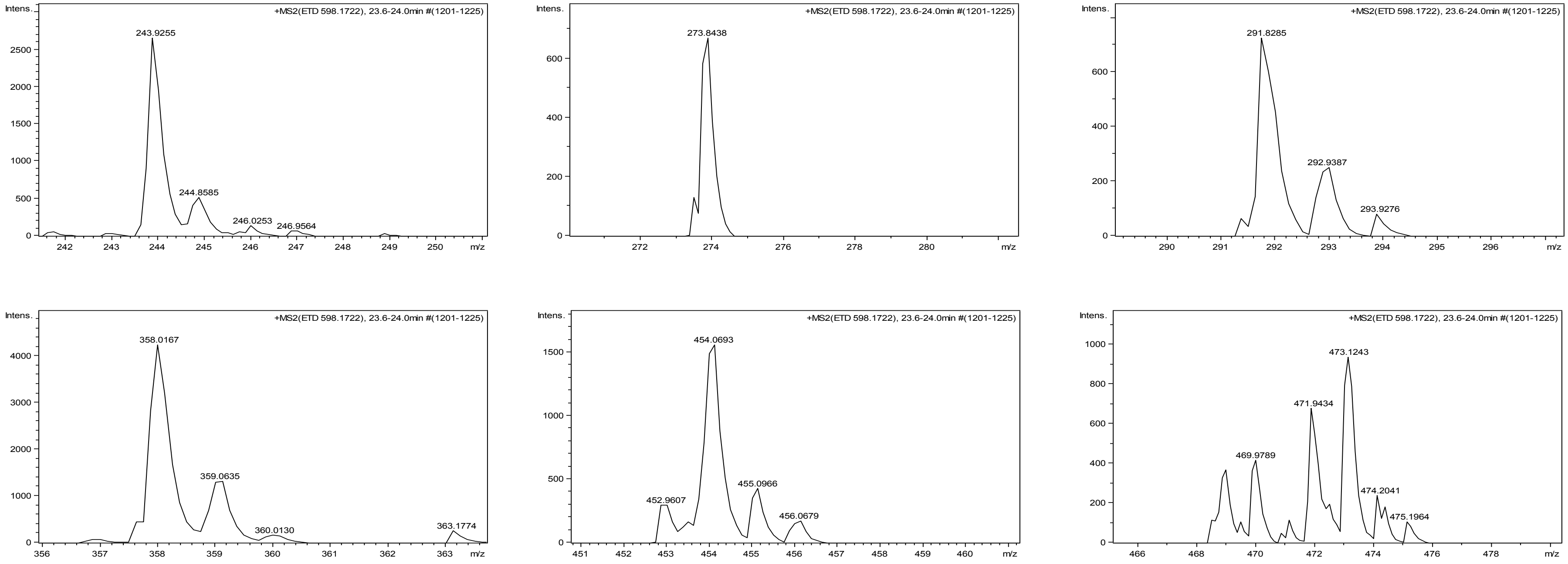

Fraction 15

597.58+++ → Pep [M+H]+ 1134.55+ [23.0-23.5 min] ETD

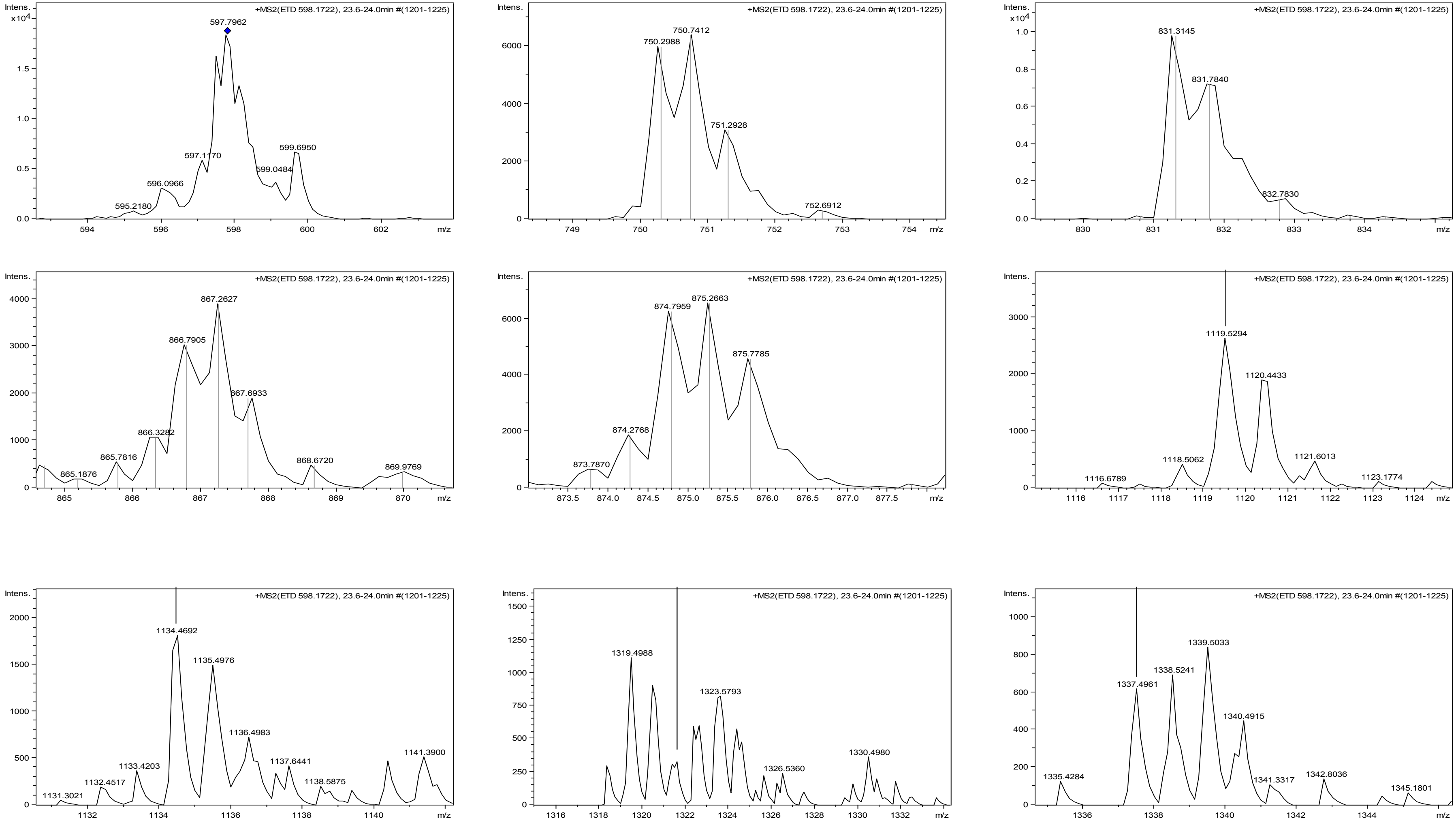

Fraction 15

597.58+++ → Pep [M+H]+ 1134.55+ [23.0-23.5 min]

ETD

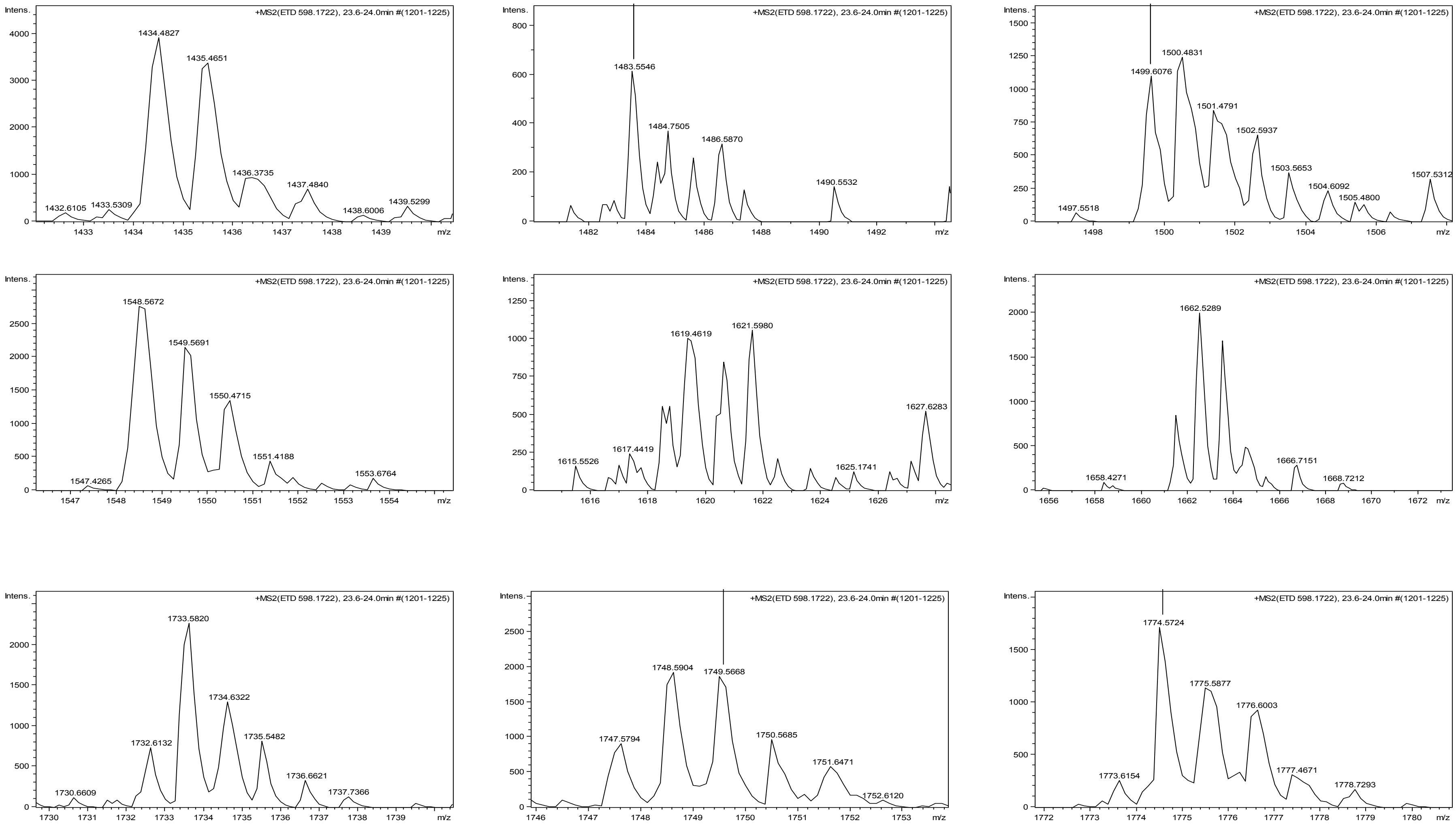

Fraction 15

597.58+++ → Pep [M+H]+ 1134.55+ [23.0-23.5 min] ETD

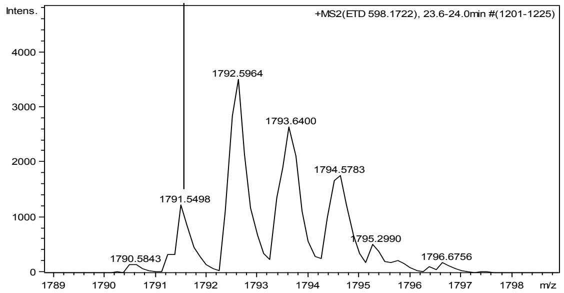

Fraction 15

597.58+++ → Pep [M+H]+ 1134.55+ [23.0-23.5 min] ETD

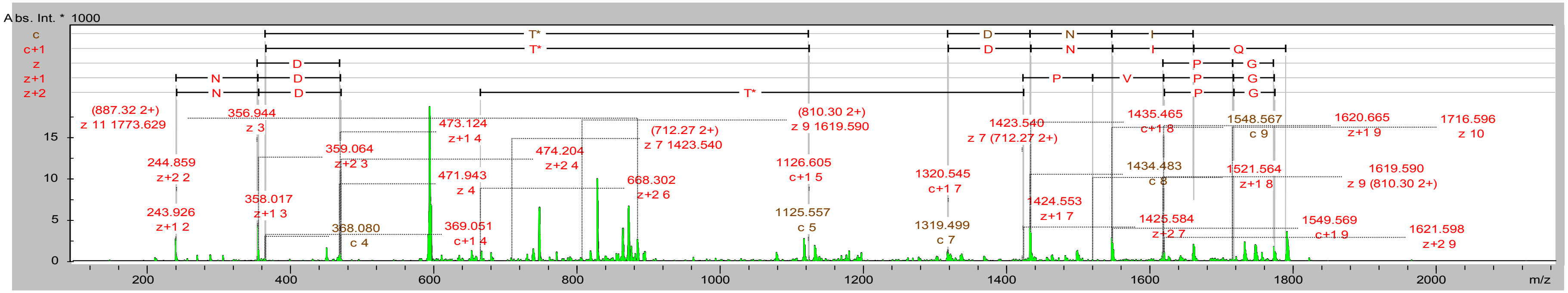

	G	P	V	P	T	P	P	D	N	I	Q	Gly	Pro	Val	Pro	Thr	Pro	Pro	Asp	Asn	Ile	Gln
Ion	1	2	3	4	5	6	7	8	9	10	11	1	2	3	4	5	6	7	8	9	10	11
c	G	P	V	P	T*	P	P	D	N	I	Q	75.055	172.108	271.176	368.229	1125.505	1222.557	1319.610	1434.637	1548.680	1661.764	1789.823
c+1	G	P	V	P	T*	P	P	D	N	I	Q	76.063	173.116	272.184	369.237	1126.512	1223.565	1320.618	1435.645	1549.688	1662.772	1790.830
z	G	P	V	P	T*	P	P	D	N	I	Q	130.050	243.134	357.177	472.204	569.257	666.309	1423.585	1520.637	1619.706	1716.759	1773.780
z+1	G	P	V	P	T*	P	P	D	N	I	Q	131.058	244.142	358.185	473.212	570.264	667.317	1424.592	1521.645	1620.714	1717.766	1774.788
z+2	G	P	V	P	T*	P	P	D	N	I	Q	132.066	245.150	359.193	474.219	571.272	668.325	1425.600	1522.653	1621.721	1718.774	1775.796
	11	10	9	8	7	6	5	4	3	2	1	Gln	Ile	Asn	Asp	Pro	Pro	Thr	Pro	Val	Pro	Gly

Biotools-Score: 117

known O-glycosylation site

Protein AMBP

$_{20}$GPVP**T**PPDNIQ$_{30}$

Fraction 15

805.73++ → Pep+HexNAc(1)NeuAc(1) [M+H]+ 1157.51+ [26.1-26.5 min]

CID-MS Precursor

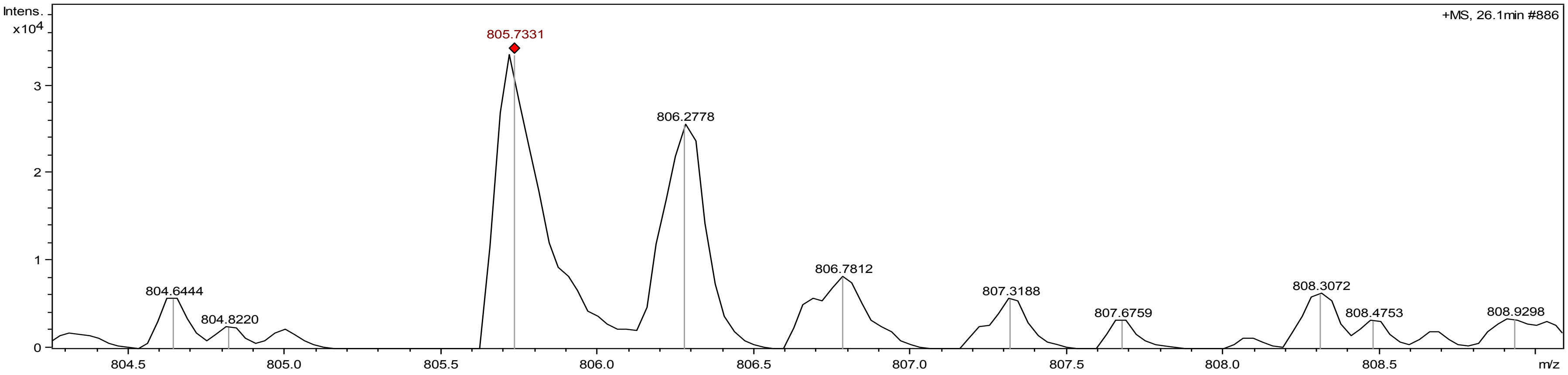

ETD spectrum not available

Fraction 15

805.73++ → Pep+HexNAc(1)NeuAc(1) [M+H]+ 1157.51+ [26.1-26.5 min]

CID-MS2

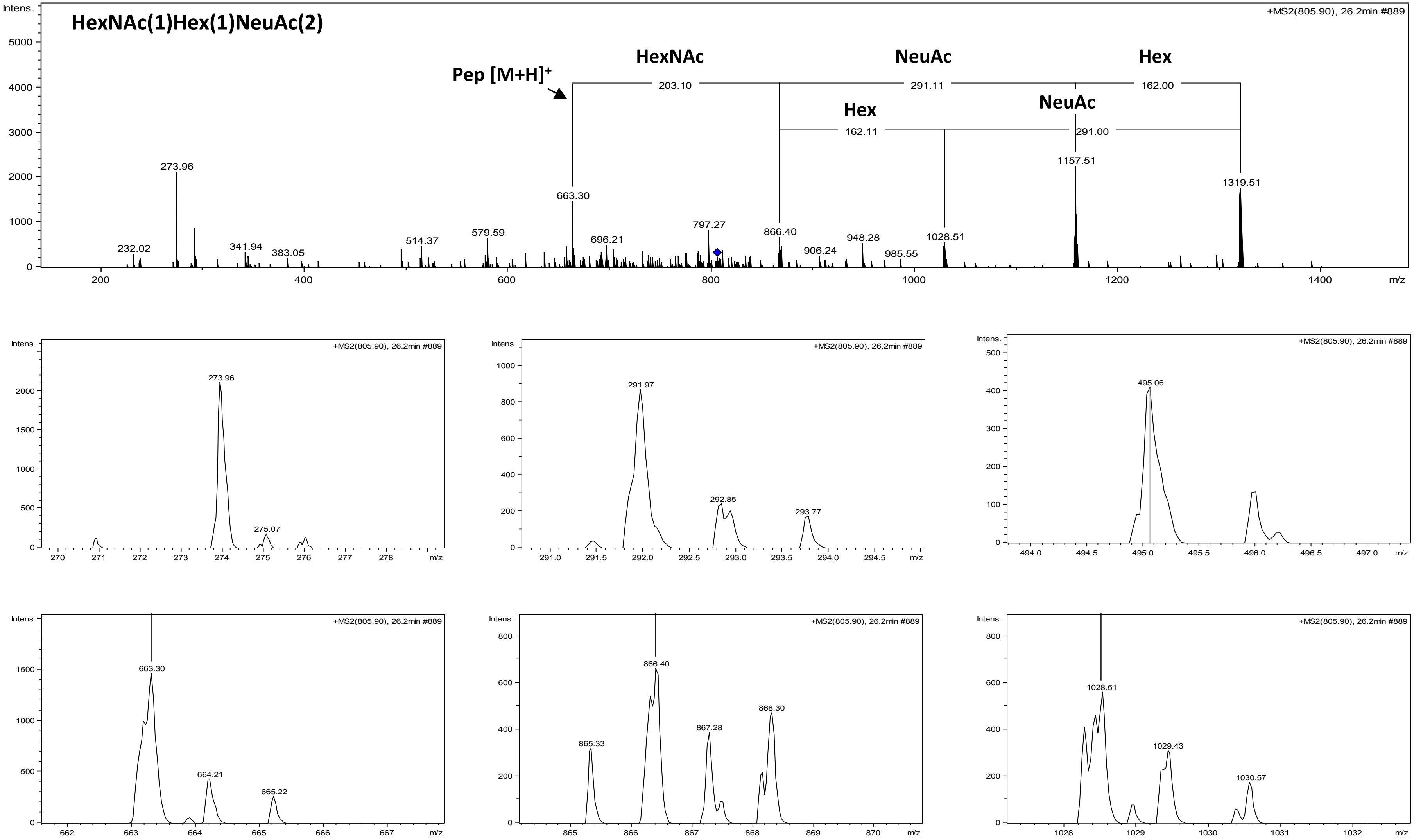

Fraction 15

805.73++ → Pep+HexNAc(1)NeuAc(1) [M+H]+ 1157.51+ [26.1-26.5 min]

CID-MS2

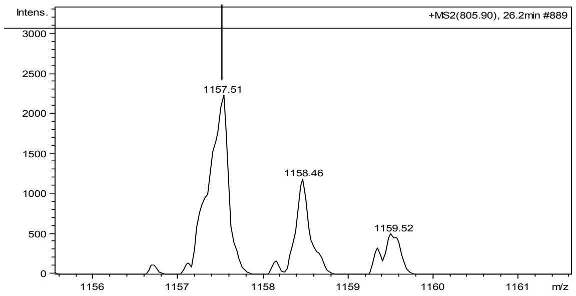

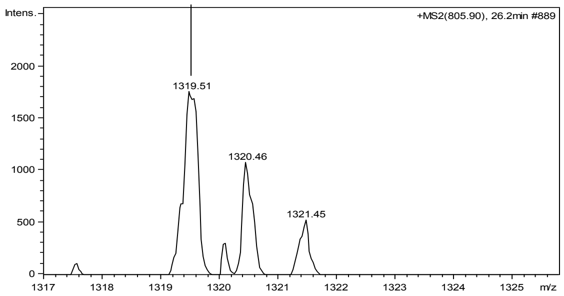

Fraction 15

805.73++ → Pep+HexNAc(1)NeuAc(1) [M+H]+ 1157.51+ [26.1-26.5 min]

CID-MS3

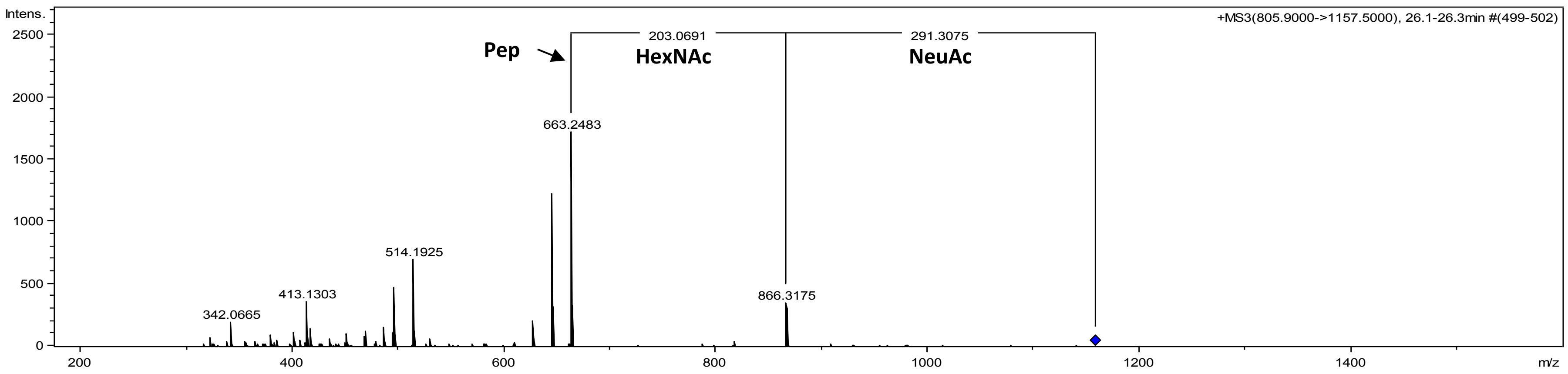

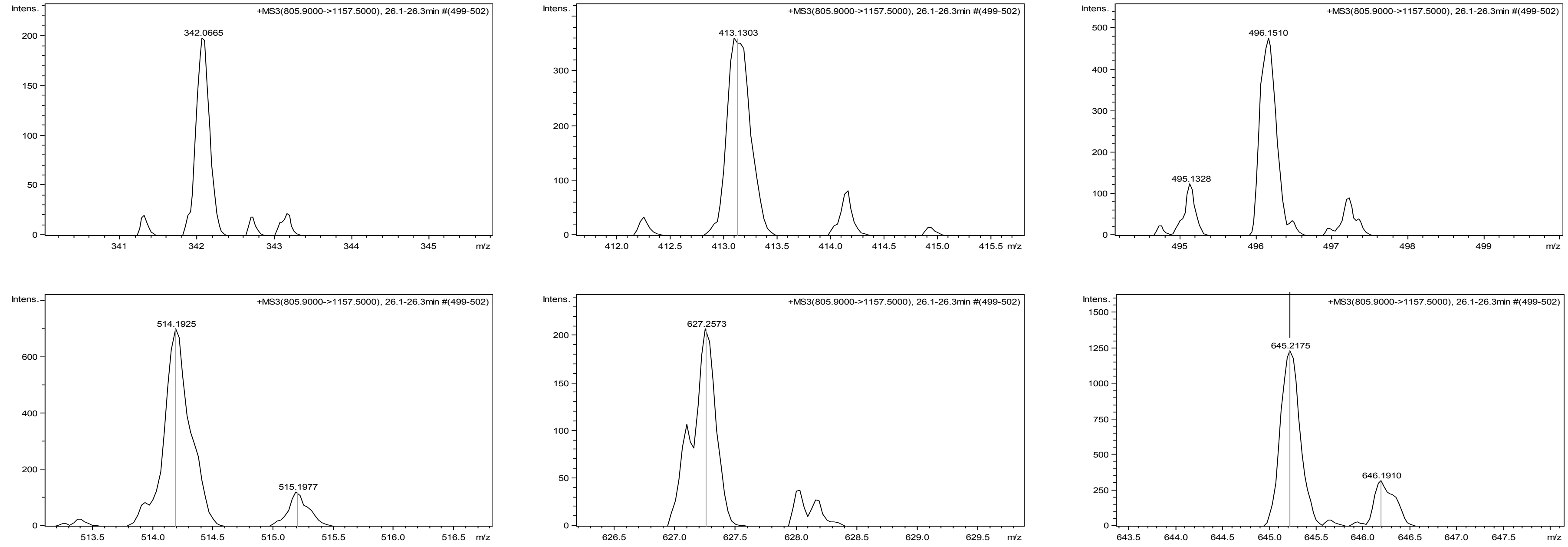

Fraction 15

805.73++ → Pep+HexNAc(1)NeuAc(1) [M+H]+ 1157.51+ [26.1-26.5 min]

CID-MS3

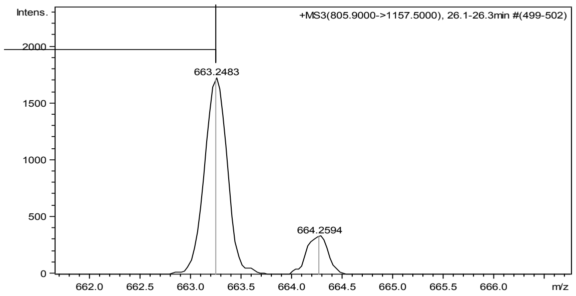

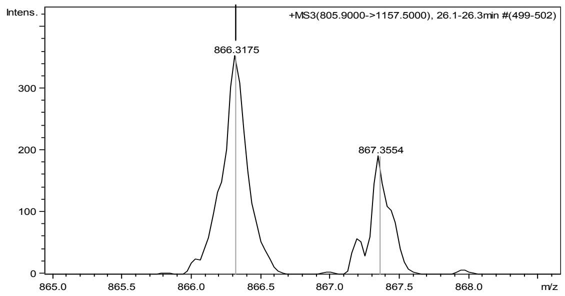

Fraction 15

805.73++ → Pep+HexNAc(1)NeuAc(1) [M+H]+ 1157.51+ [26.1-26.5 min] CID-MS3 MASCOT Search

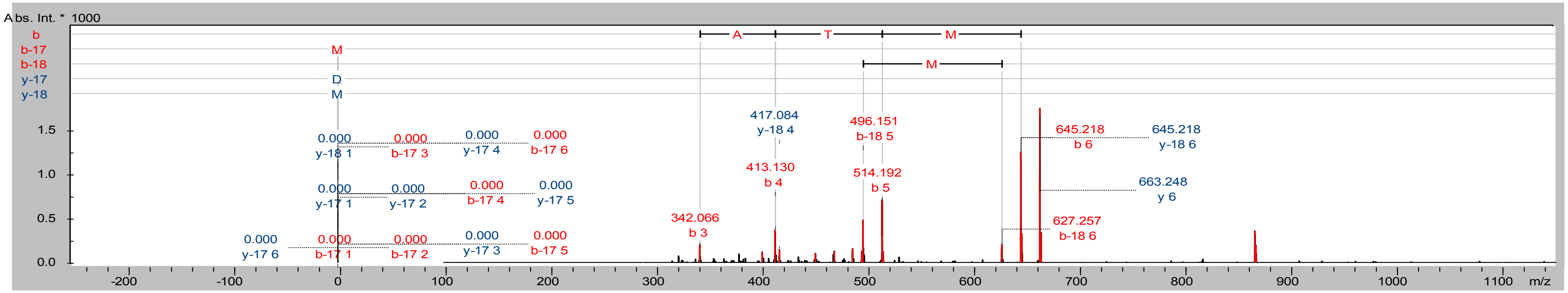

	D	L	I	A	T	M	Asp	Leu	Ile	Ala	Thr	Met
Ion	1	2	3	4	5	6	1	2	3	4	5	6
b	D	L	I	A	T	M	116.034	229.118	342.202	413.239	514.287	645.328
b-17	D	L	I	A	T	M	-	-	-	-	-	-
b-18	D	L	I	A	T	M	98.024	211.108	324.192	395.229	496.277	627.317
y	D	L	I	A	T	M	150.058	251.106	322.143	435.227	548.311	663.338
y-17	D	L	I	A	T	M	-	-	-	-	-	-
y-18	D	L	I	A	T	M	-	233.095	304.133	417.217	530.301	645.328
	6	5	4	3	2	1	Met	Thr	Ala	Ile	Leu	Asp

known O-glycosylation site

Kininogen-1 precursor

$_{567}$DLIA**T**M$_{572}$

Fraction 15

805.73++ → Pep+HexNAc(1)NeuAc(1) [M+H]+ 1157.51+ [26.1-26.5 min] CID-MS3 MASCOT Search

prot_hit_nur	prot_acc	prot_desc	prot_score	prot_mass	prot_matche	pep_query	pep_rank	pep_isbold	pep_exp_mz	pep_exp_mr	pep_exp_z	pep_calc_mr	pep_delta	pep_miss	pep_score	pep_expect	pep_res_bef	pep_seq
1	I20L2_HUMA	Interferon-st	24	39415	1	1	1	1	663.2483	662.241	1	662.2581	-0.0171	0	27.51	26	C	PENATM
2	CIDEA_HUM/	Cell death ac	24	24899	1	1	1	0	663.2483	662.241	1	662.3421	-0.1011	0	27.51	26	L	NVKATM
3	EDNRA_HUN	Endothelin-1	22	49887	1	1	1	0	663.2483	662.241	1	662.3309	-0.0899	0	27.51	26	G	INLATM
4	KNG1_HUMA	Kininogen-1	22	72996	1	1	1	0	663.2483	662.241	1	662.3309	-0.0899	0	27.51	26	S	DLIATM
5	LYPA3_HUM/	1-O-acylcera	22	46913	1	1	1	0	663.2483	662.241	1	662.3309	-0.0899	0	27.51	26	G	LVEATM
6	O13A1_HUM	Olfactory rec	22	34678	1	1	1	0	663.2483	662.241	1	662.3309	-0.0899	0	27.51	26	L	LNLATM
7	XPC_HUMAN	DNA-repair p	21	106771	1	1	1	0	663.2483	662.241	1	662.3534	-0.1123	0	27.51	26	L	KRGATM
8	K0355_HUM/	Uncharacteri	20	117229	1	1	1	0	663.2483	662.241	1	662.2581	-0.0171	0	27.51	26	K	EDPATM
9	NALP5_HUM	NACHT, LRR a	20	136886	1	1	1	0	663.2483	662.241	1	662.2581	-0.0171	0	27.51	26	E	DPEATM
10	CSMD2_HUN	CUB and sush	19	387494	1	1	1	0	663.2483	662.241	1	662.3421	-0.1011	0	27.51	26	S	KVNATM

Biotools-Score: 16

MASCOT-Score: 28

known O-glycosylation site

Kininogen-1 precursor

$_{567}$DLIATM$_{572}$

Fraction 15

945.37++ → Pep [M+H]+ 1233.63+ [27.6-28.1 min]

CID-MS Precursor

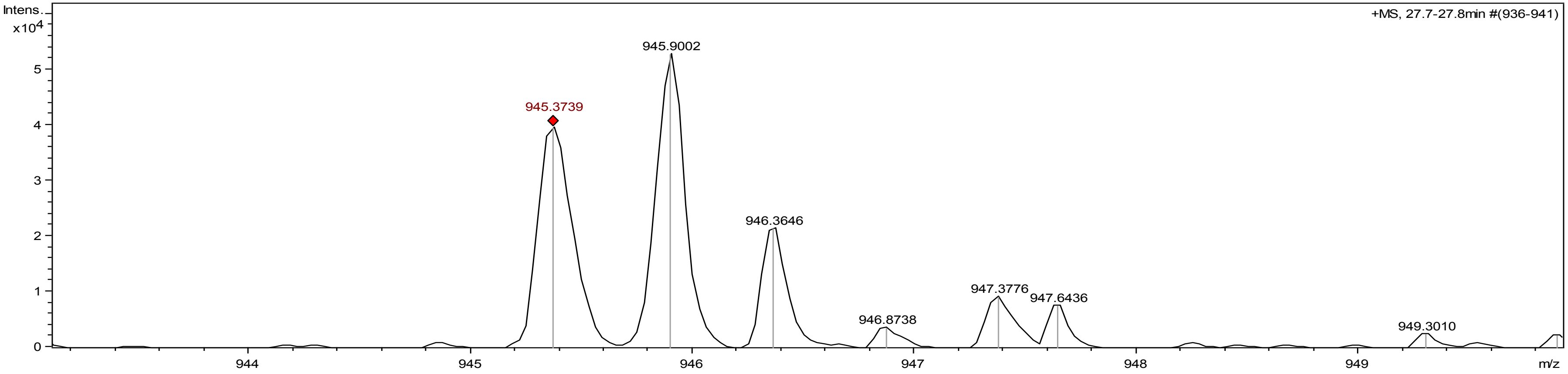

ETD spectrum not available

Fraction 15

945.37++ → Pep [M+H]+ 1233.63+ [27.6-28.1 min] CID-MS2

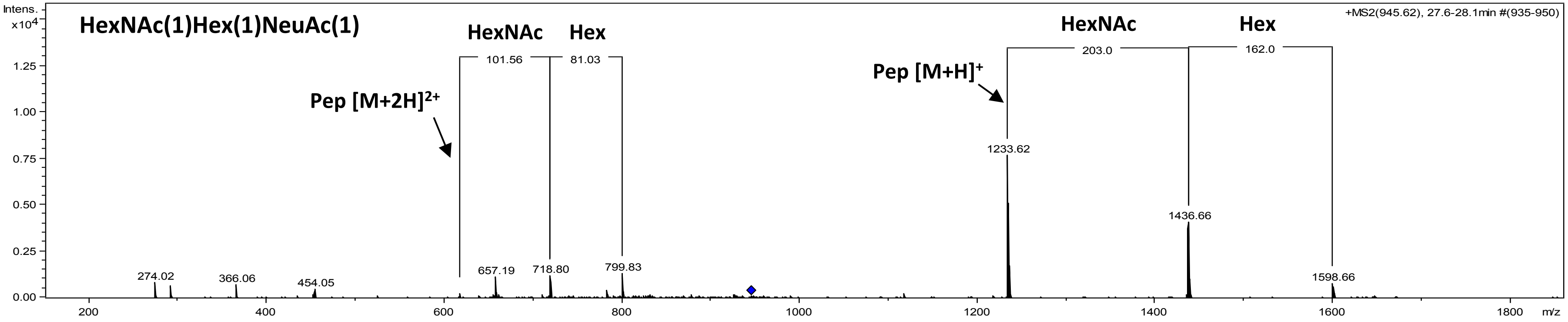

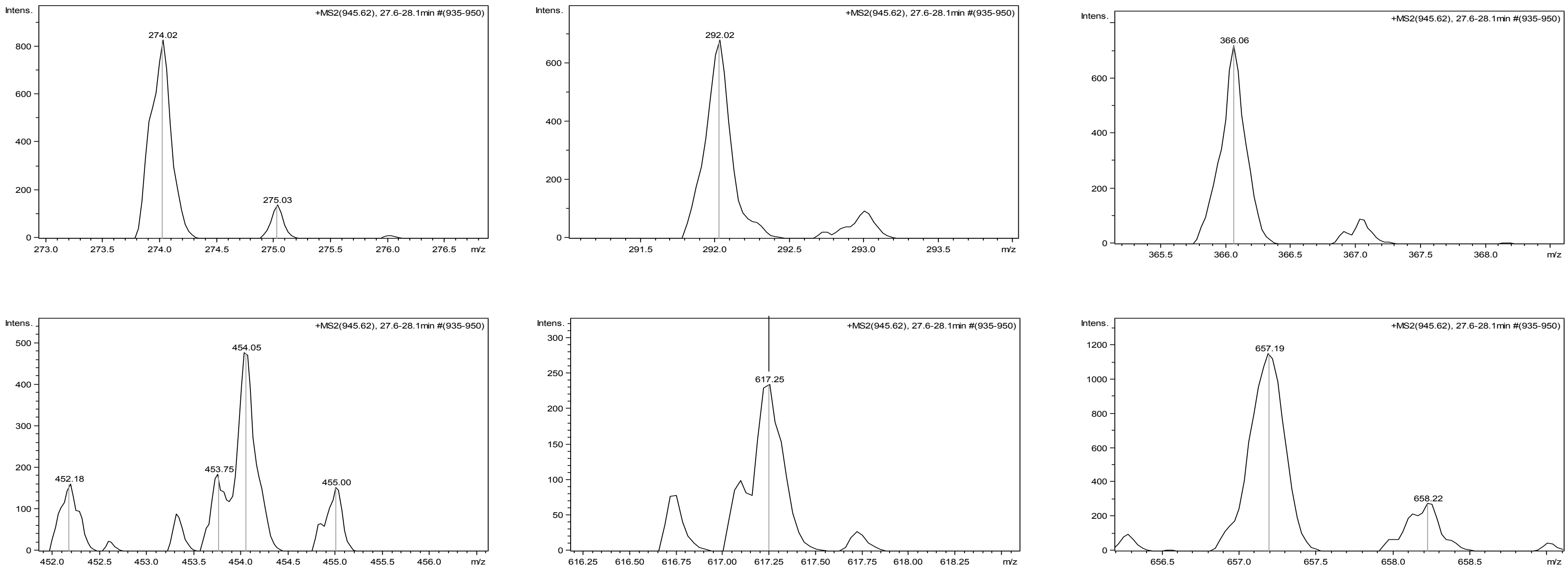

Fraction 15

945.37++ → Pep [M+H]+ 1233.63+ [27.6-28.1 min]

CID-MS2

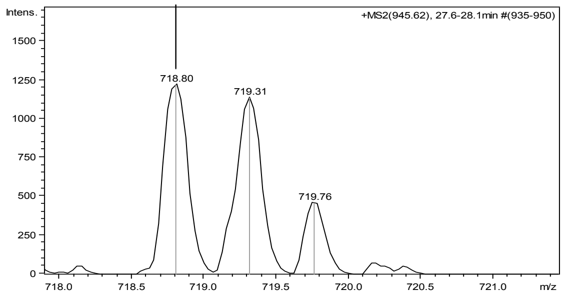

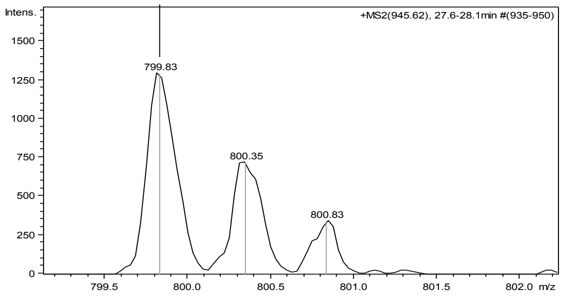

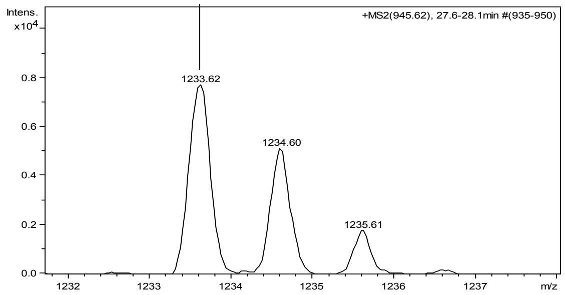

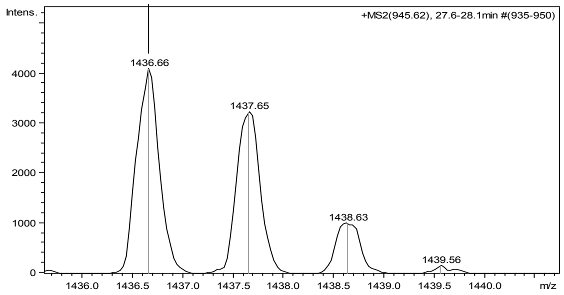

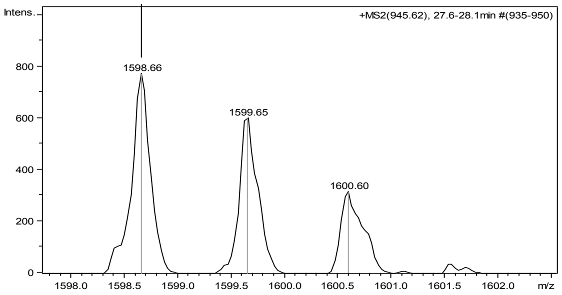

Fraction 15

945.37++ → Pep [M+H]+ 1233.63+ [27.6-28.1 min] CID-MS3

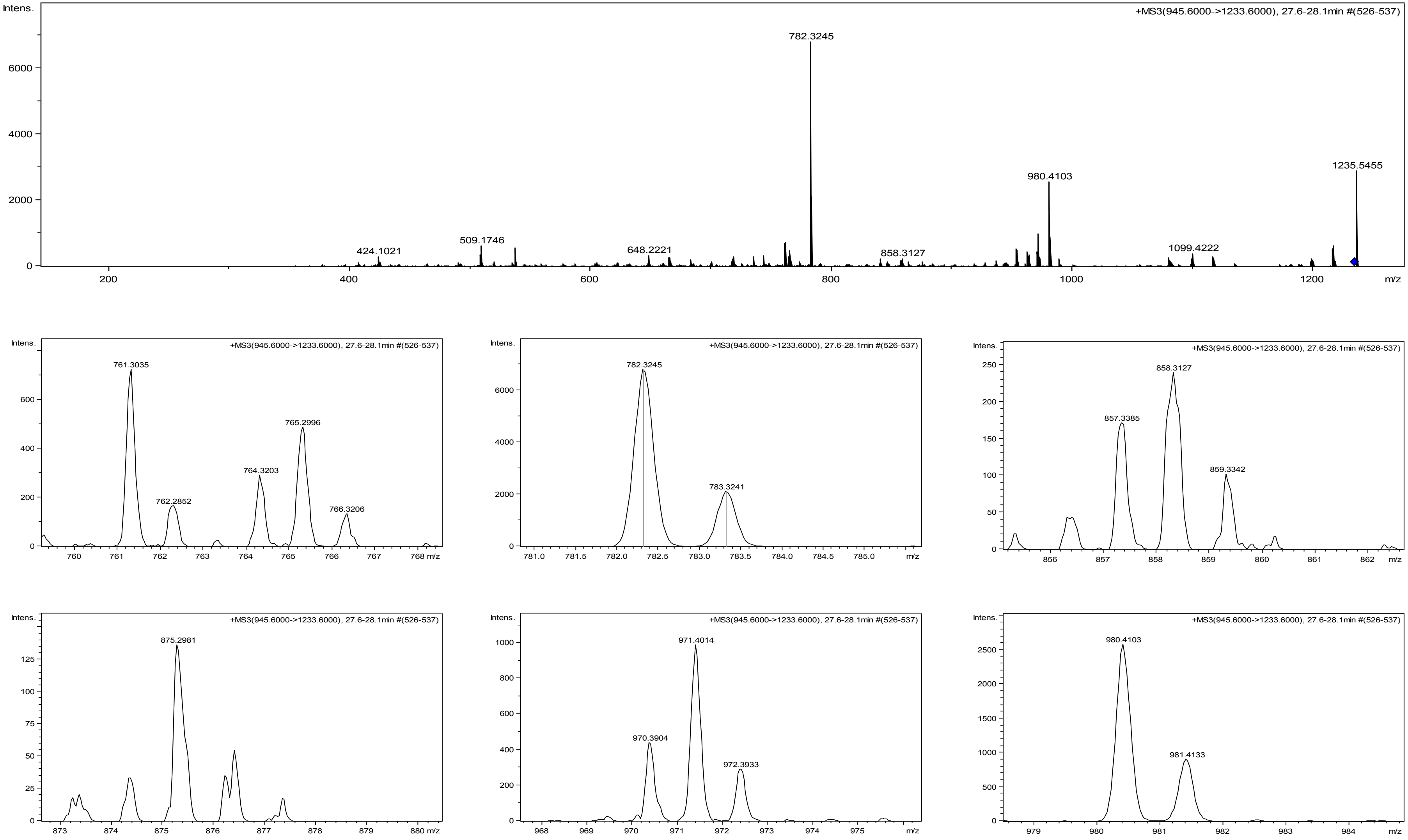

Fraction 15

945.37++ → Pep [M+H]+ 1233.63+ [27.6-28.1 min]

CID-MS3

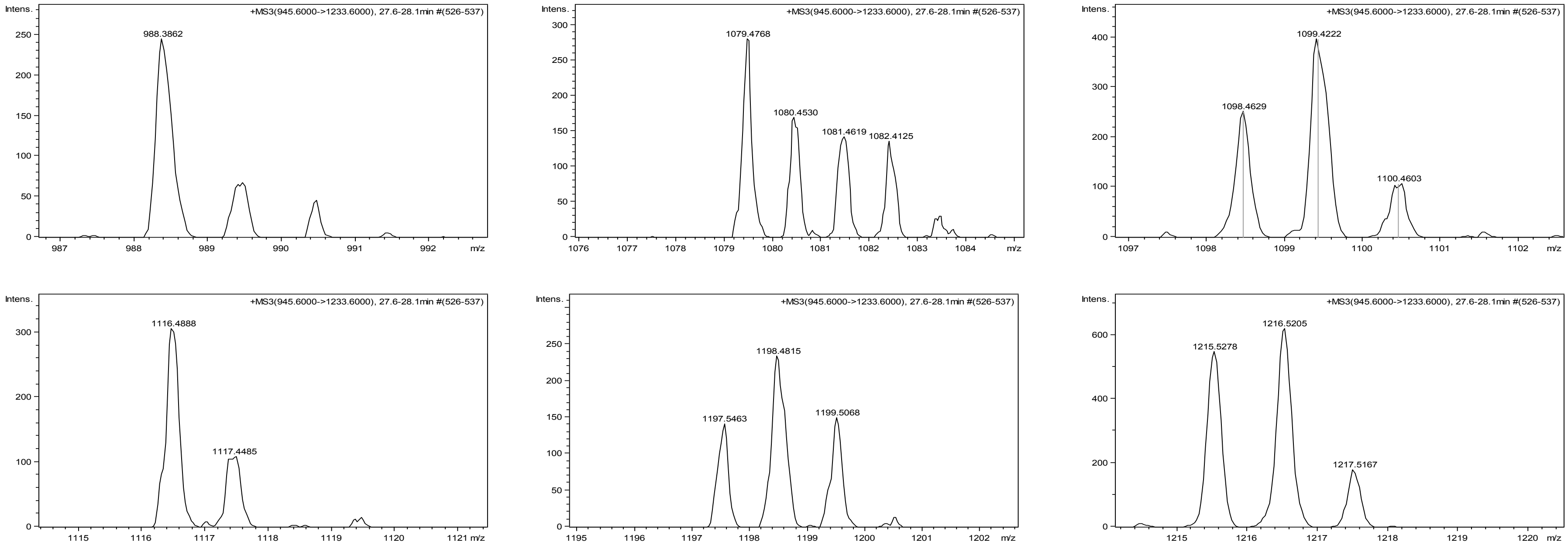

Fraction 15

945.37++ → Pep [M+H]+ 1233.63+ [27.6-28.1 min]   CID-MS3   MASCOT Search

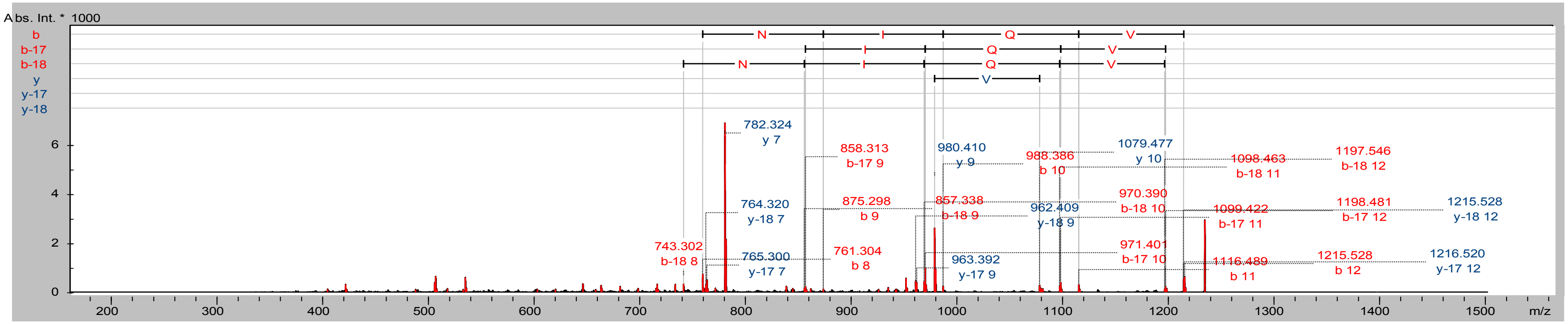

	G	P	V	P	T	P	P	D	N	I	Q	V	Gly	Pro	Val	Pro	Thr	Pro	Pro	Asp	Asn	Ile	Gln	Val
Ion	1	2	3	4	5	6	7	8	9	10	11	12	1	2	3	4	5	6	7	8	9	10	11	12
b	G	P	V	P	T	P	P	D	N	I	Q	V	58.029	155.082	254.150	351.203	452.250	549.303	646.356	761.383	875.426	988.510	1116.568	1215.637
b-17	G	P	V	P	T	P	P	D	N	I	Q	V	-	-	-	-	-	-	-	-	858.399	971.483	1099.542	1198.610
b-18	G	P	V	P	T	P	P	D	N	I	Q	V	-	-	-	-	434.240	531.293	628.345	743.372	857.415	970.499	1098.558	1197.626
y	G	P	V	P	T	P	P	D	N	I	Q	V	118.086	246.145	359.229	473.272	588.299	685.352	782.404	883.452	980.505	1079.573	1176.626	1233.647
y-17	G	P	V	P	T	P	P	D	N	I	Q	V	-	229.118	342.202	456.245	571.272	668.325	765.378	866.425	963.478	1062.547	1159.599	1216.621
y-18	G	P	V	P	T	P	P	D	N	I	Q	V	-	-	-	-	570.288	667.341	764.394	865.441	962.494	1061.563	1158.615	1215.637
	12	11	10	9	8	7	6	5	4	3	2	1	Val	Gln	Ile	Asn	Asp	Pro	Pro	Thr	Pro	Val	Pro	Gly

known O-glycosylation site

Protein AMBP

$_{20}$GPVP**T**PPDNIQV$_{31}$

Fraction 15

945.37++ → Pep [M+H]+ 1233.63+ [27.6-28.1 min]

CID-MS3 MASCOT Search

prot_hit_nur	prot_acc	prot_desc	prot_score	prot_mass	prot_matche	pep_query	pep_rank	pep_isbold	pep_exp_mz	pep_exp_mr	pep_exp_z	pep_calc_mr	pep_delta	pep_miss	pep_score	pep_expect	pep_res_bef	pep_seq
1	KBRS2_HUM/	NF-kappa-B i	8	21780	1	1	3	1	1233.625	1232.6177	1	1231.5391	1.0787	0	11.82	1.20E+03	G	NHVVGSEMI
2	AMBP_HUM/	AMBP protei	8	39886	1	1	2	0	1233.625	1232.6177	1	1232.6401	-0.0224	0	12.47	1.00E+03	A	GPVPTPPDN
3	ATL2_HUMAI	ADAMTS-like	7	108088	1	1	1	0	1233.625	1232.6177	1	1231.5932	1.0245	0	13.8	7.50E+02	S	DQLLGAGSDI
4	GAB2_HUMA	GRB2-associa	6	74925	1	1	5	0	1233.625	1232.6177	1	1231.5681	1.0497	0	11.68	1.20E+03	G	HTKGSLTGSE
5	DC1I2_HUM/	Cytoplasmic	5	71811	1	1	3	0	1233.625	1232.6177	1	1231.5332	1.0845	0	11.82	1.20E+03	V	YDVMWSPTH
6	HNRPL_HUM	Heterogene	5	60719	1	1	6	0	1233.625	1232.6177	1	1232.5536	0.0641	0	10.75	1.50E+03	V	FNVFCLYGN\
7	KAPCA_HUM	cAMP-deper	4	40678	1	1	9	0	1233.625	1232.6177	1	1231.5932	1.0246	0	8.78	2.40E+03	G	NLKNGVNDII
8	VACHT_HUM	Vesicular ace	3	57324	1	1	10	0	1233.625	1232.6177	1	1232.7063	-0.0886	0	8.11	2.80E+03	L	PVGTPIHRLM
9	PIGA_HUMA	Phosphatidy	3	54776	1	1	8	0	1233.625	1232.6177	1	1231.5438	1.0739	0	8.79	2.40E+03	T	RTHNICMVSI
10	BAZ2A_HUM	Bromodoma	1	210169	1	1	7	0	1233.625	1232.6177	1	1232.5594	0.0583	0	9.26	2.10E+03	L	MGAEDKLPLE

Biotools-Score: 32

MASCOT-Score: 12

known O-glycosylation site

Protein AMBP

$_{20}$GPVPTPPDNIQV$_{31}$

Fraction 15

718.30+++ → Pep [M+H]+ 1205.63+ [28.2-28.5 min] CID-MS Precursor

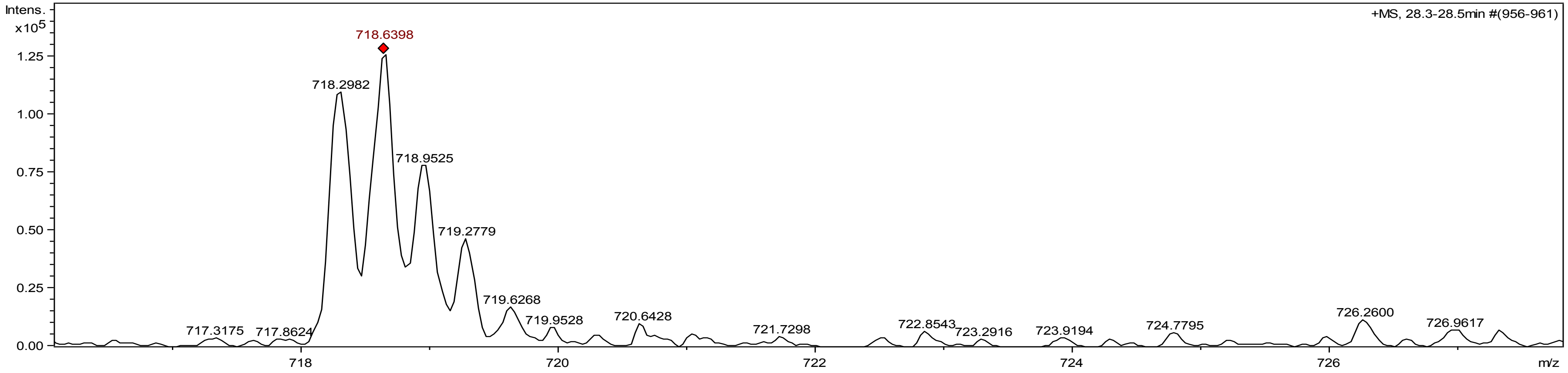

Fraction 15

718.30+++ → Pep [M+H]+ 1205.63+ [28.2-28.5 min] CID-MS2

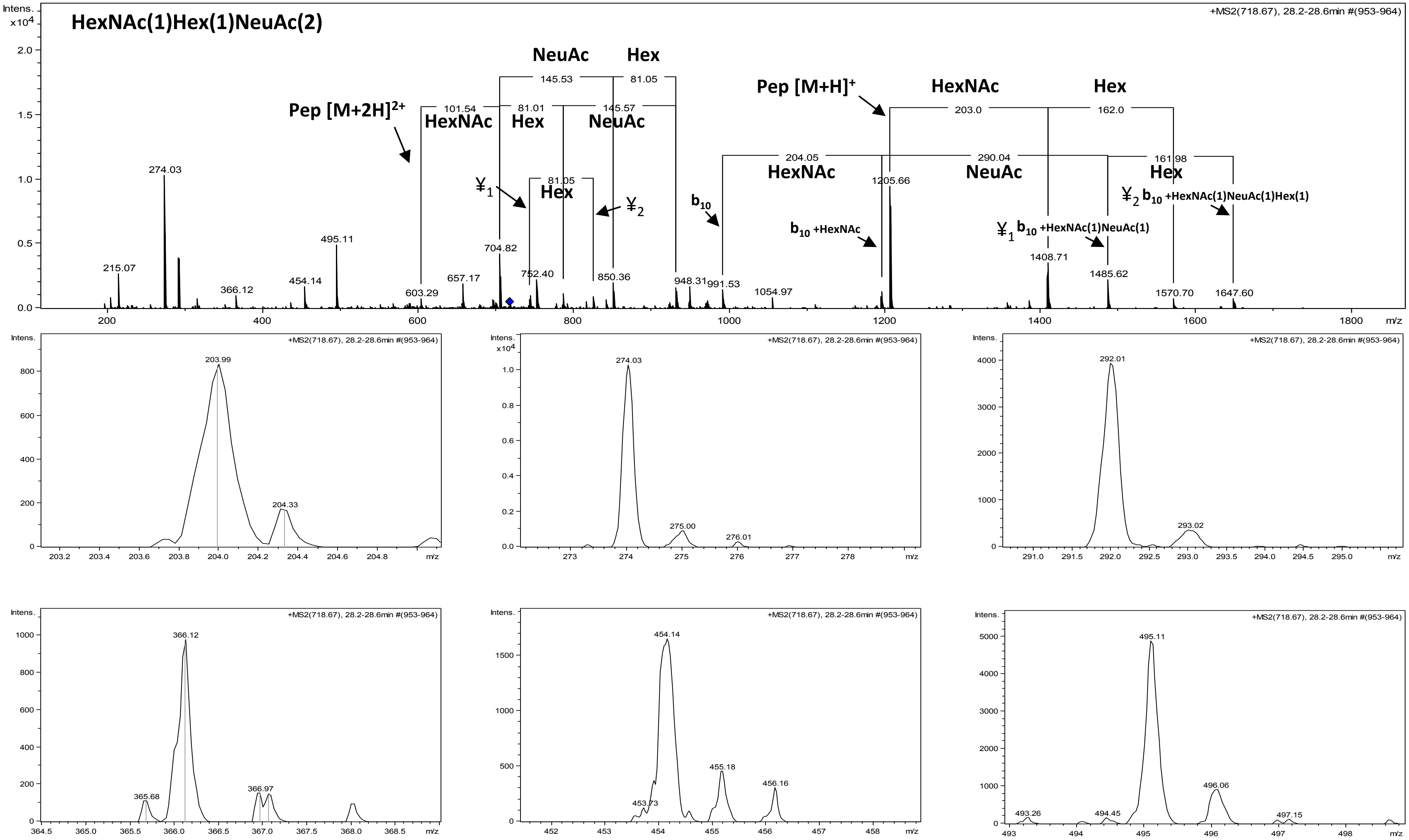

Fraction 15

718.30+++ → Pep [M+H]+ 1205.63+ [28.2-28.5 min]

CID-MS2

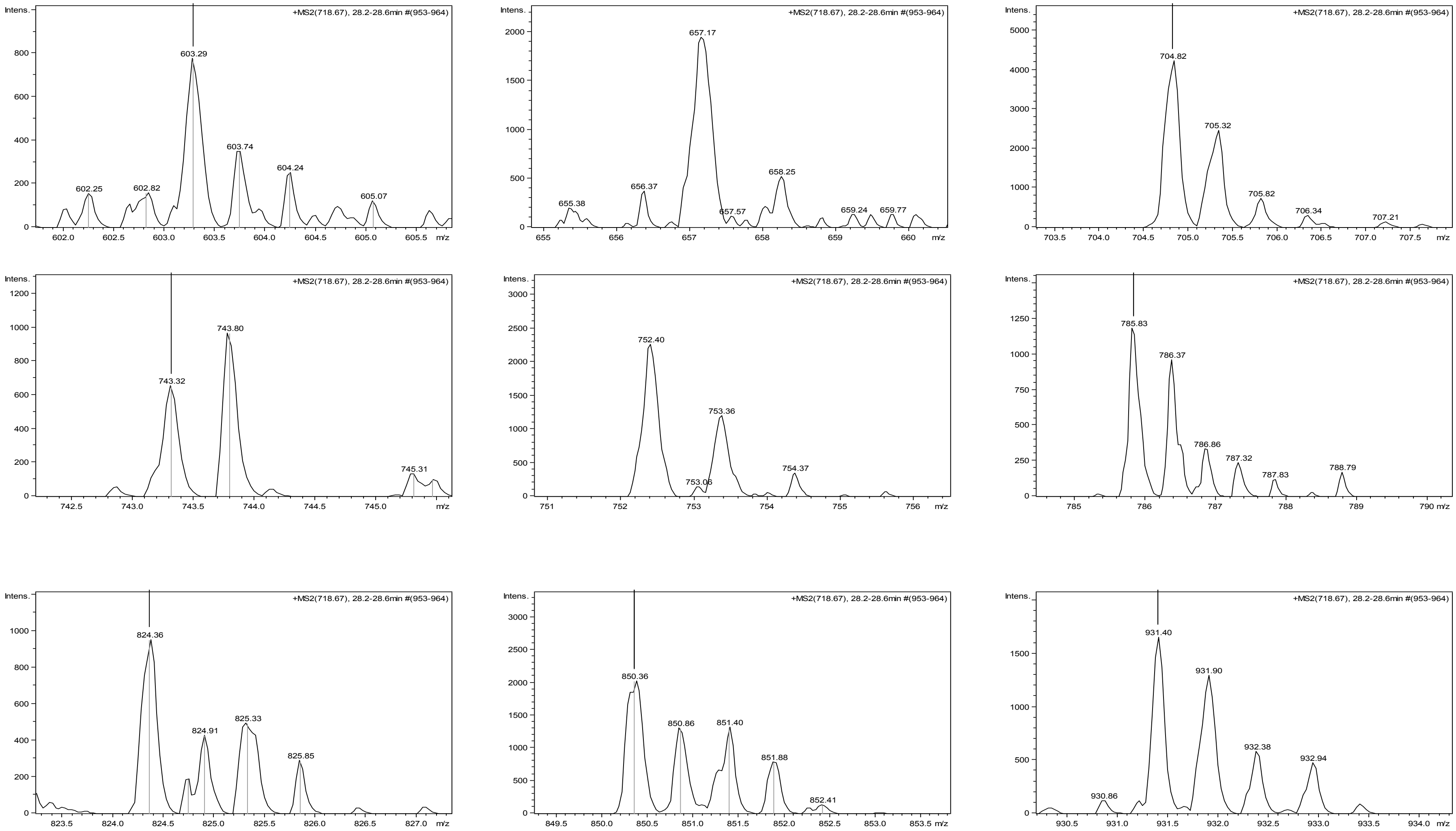

Fraction 15

718.30+++ → Pep [M+H]+ 1205.63+ [28.2-28.5 min]

CID-MS2

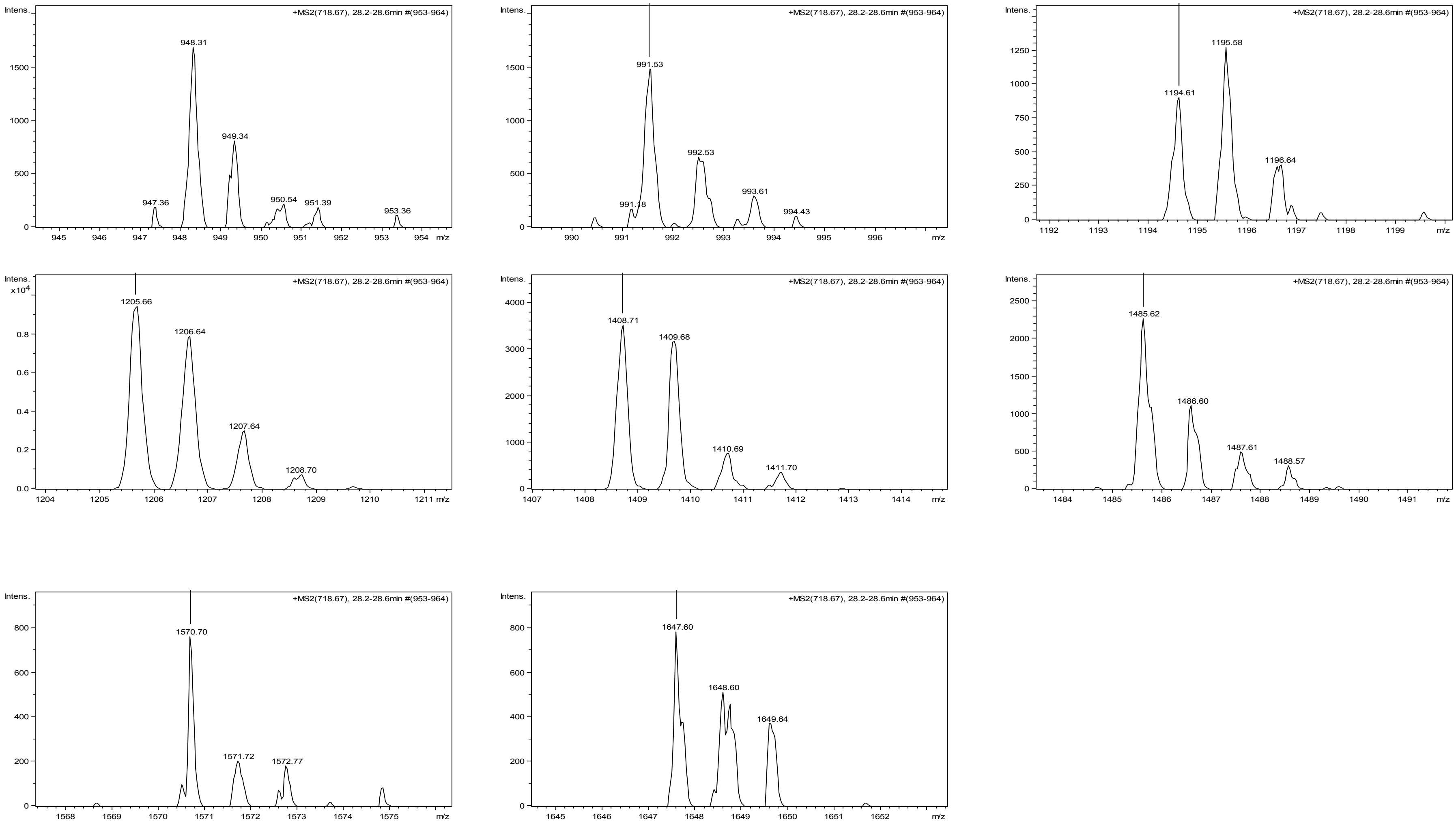

Fraction 15

718.30+++ → Pep [M+H]+ 1205.63+ [28.2-28.5 min] CID-MS3

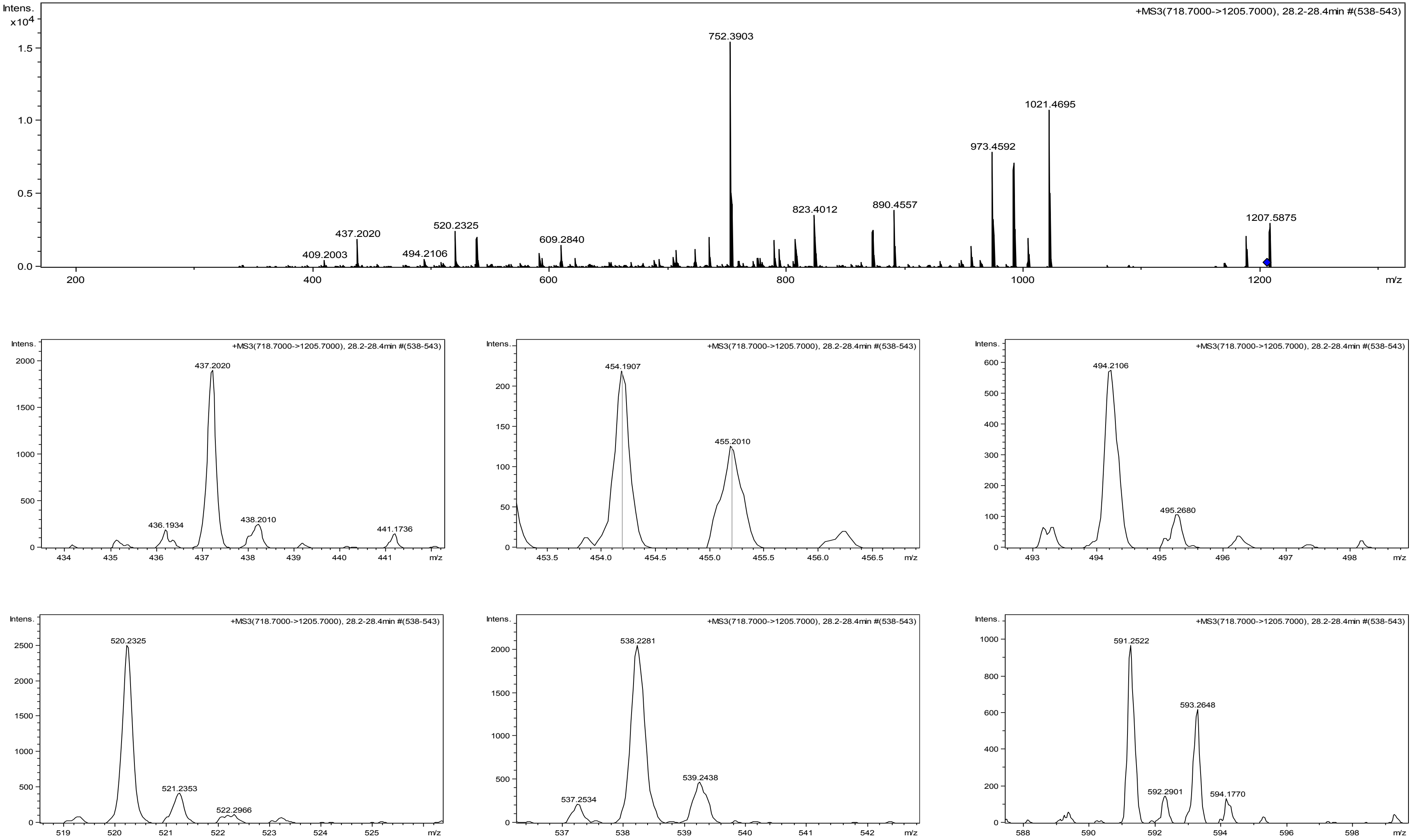

Fraction 15

718.30+++ → Pep [M+H]+ 1205.63+ [28.2-28.5 min] CID-MS3

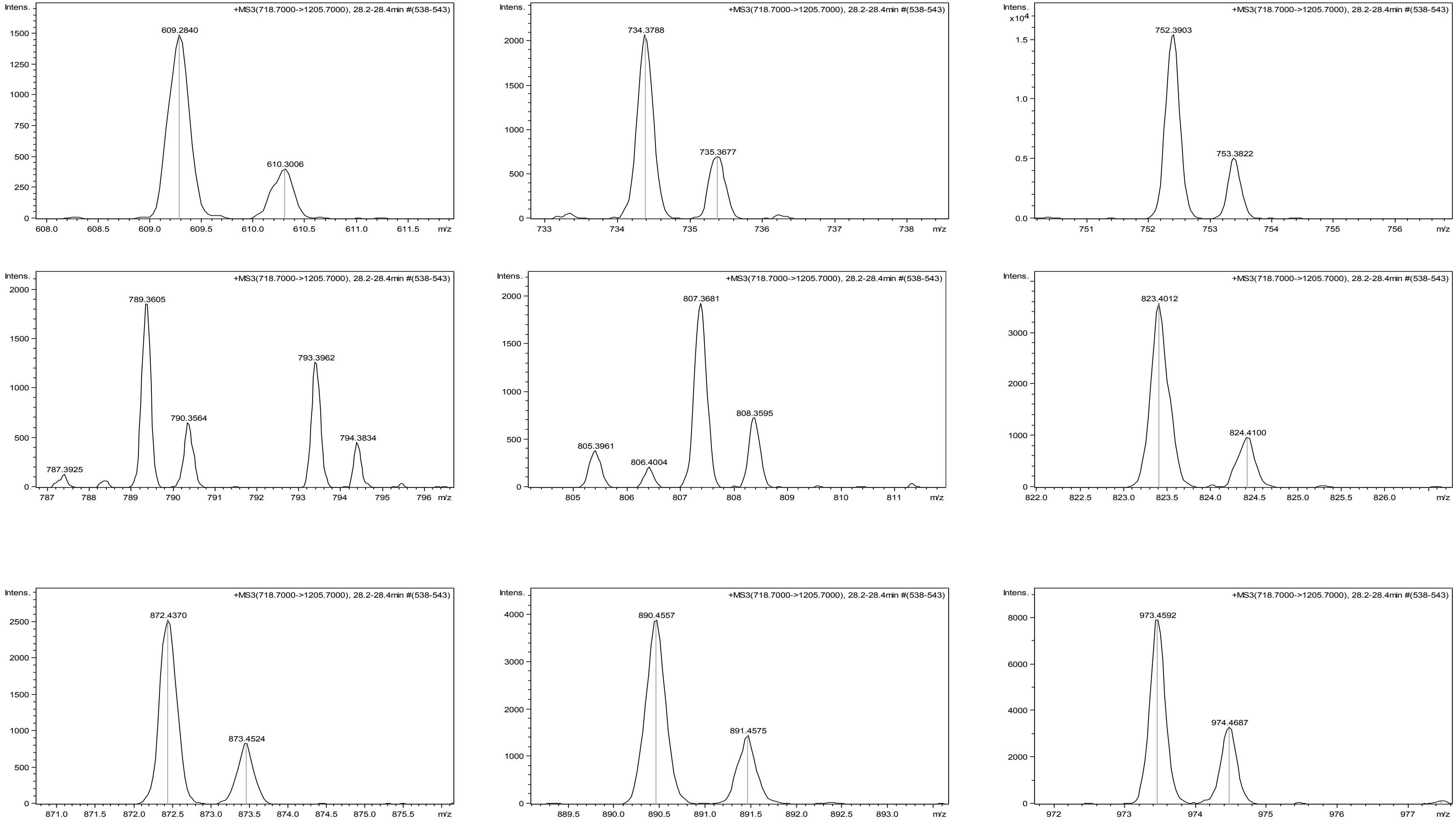

Fraction 15

718.30+++ → Pep [M+H]+ 1205.63+ [28.2-28.5 min] CID-MS3

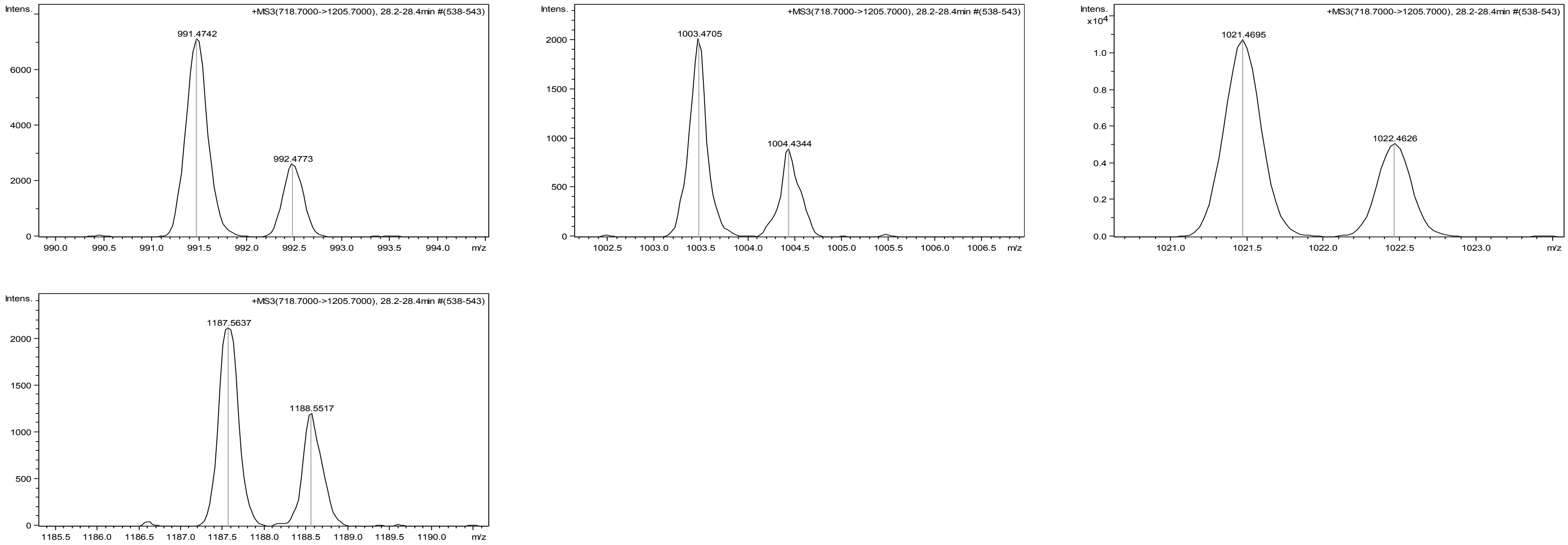

Fraction 15

718.30+++ → Pep [M+H]+ 1205.63+ [28.2-28.5 min]

CID-MS3 MASCOT Search

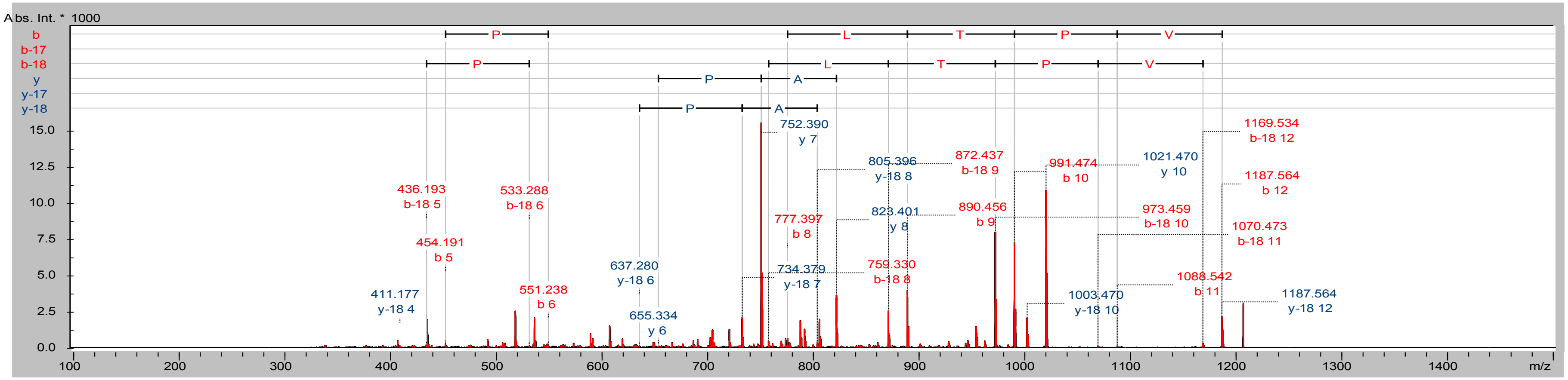

	L	A	P	T	A	P	P	E	L	T	P	V	Leu	Ala	Pro	Thr	Ala	Pro	Pro	Glu	Leu	Thr	Pro	Val
Ion	1	2	3	4	5	6	7	8	9	10	11	12	1	2	3	4	5	6	7	8	9	10	11	12
b	L	A	P	T	A	P	P	E	L	T	P	V	114.091	185.128	282.181	383.229	454.266	551.319	648.372	777.414	890.498	991.546	1088.599	1187.667
b-17	L	A	P	T	A	P	P	E	L	T	P	V	-	-	-	-	-	-	-	-	-	-	-	-
b-18	L	A	P	T	A	P	P	E	L	T	P	V	-	-	-	365.218	436.255	533.308	630.361	759.404	872.488	973.535	1070.588	1169.656
y	L	A	P	T	A	P	P	E	L	T	P	V	118.086	215.139	316.187	429.271	558.313	655.366	752.419	823.456	924.504	1021.556	1092.594	1205.678
y-17	L	A	P	T	A	P	P	E	L	T	P	V	-	-	-	-	-	-	-	-	-	-	-	-
y-18	L	A	P	T	A	P	P	E	L	T	P	V	-	-	298.176	411.260	540.303	637.356	734.408	805.445	906.493	1003.546	1074.583	1187.667
	12	11	10	9	8	7	6	5	4	3	2	1	Val	Pro	Thr	Leu	Glu	Pro	Pro	Ala	Thr	Pro	Ala	Leu

known O-glycosylation site

Plasminogen precursor

$_{362}$LAP**T**APPELTPV$_{373}$

Fraction 15

718.30+++ → Pep [M+H]+ 1205.63+ [28.2-28.5 min]

CID-MS3 MASCOT Search

prot_hit_nur	prot_acc	prot_desc	prot_score	prot_mass	prot_matche	pep_query	pep_rank	pep_isbold	pep_exp_mz	pep_exp_mr	pep_exp_z	pep_calc_mr	pep_delta	pep_miss	pep_score	pep_expect	pep_res_bef	pep_seq
1	PLMN_HUMA	Plasminoger	11	93247	1	1	1	1	1205.6667	1204.6594	1	1204.6703	-0.0109	0	16.49	4.00E+02	Q	LAPTAPPELTI
2	COPZ1_HUM	Coatomer su	10	20242	1	1	3	0	1205.6667	1204.6594	1	1204.7067	-0.0473	0	13.17	8.50E+02	I	ALLEGLTVVYI
3	MAP7_HUMA	Ensconsin (M	9	84116	1	1	2	0	1205.6667	1204.6594	1	1204.5571	0.1023	0	15.51	5.00E+02	K	PSRLDVTNSE
4	STABP_HUM	STAM-bindir	6	48617	1	1	7	0	1205.6667	1204.6594	1	1204.6928	-0.0334	0	11.35	1.30E+03	I	PTIDGLRHVV
5	TECT1_HUMA	Tectonic-1 pr	6	64613	1	1	6	0	1205.6667	1204.6594	1	1204.6048	0.0546	0	12.09	1.10E+03	K	TLTRREDTDV
6	MCAR6_HUM	Mitochondri	6	34573	1	1	10	0	1205.6667	1204.6594	1	1204.5434	0.116	0	10.8	1.50E+03	N	MPSLWASAC
7	ITA2_HUMAN	Integrin alph	6	130468	1	1	4	0	1205.6667	1204.6594	1	1203.4932	1.1663	0	13.15	8.50E+02	T	SQYGGDLTNT
8	G3BP1_HUM	Ras GTPase-a	5	52189	1	1	8	0	1205.6667	1204.6594	1	1204.5401	0.1194	0	11.26	1.30E+03	L	KDFFQSYGNV
9	RPGF2_HUM	Rap guanine	5	168168	1	1	5	0	1205.6667	1204.6594	1	1204.6816	-0.0221	0	12.56	9.80E+02	R	QTKHIPTALPY
10	WTAP_HUMA	Wilms~ tumc	5	44388	1	1	9	0	1205.6667	1204.6594	1	1204.6122	0.0473	0	11.14	1.40E+03	Q	PSVAQLRSTM

Biotools-Score: 47

MASCOT-Score: 16

known O-glycosylation site

Plasminogen precursor

$_{362}$LAP**T**APPELTPV$_{373}$

Fraction 15

718.30+++ → Pep [M+H]+ 1205.63+ [28.2-28.5 min] ETD

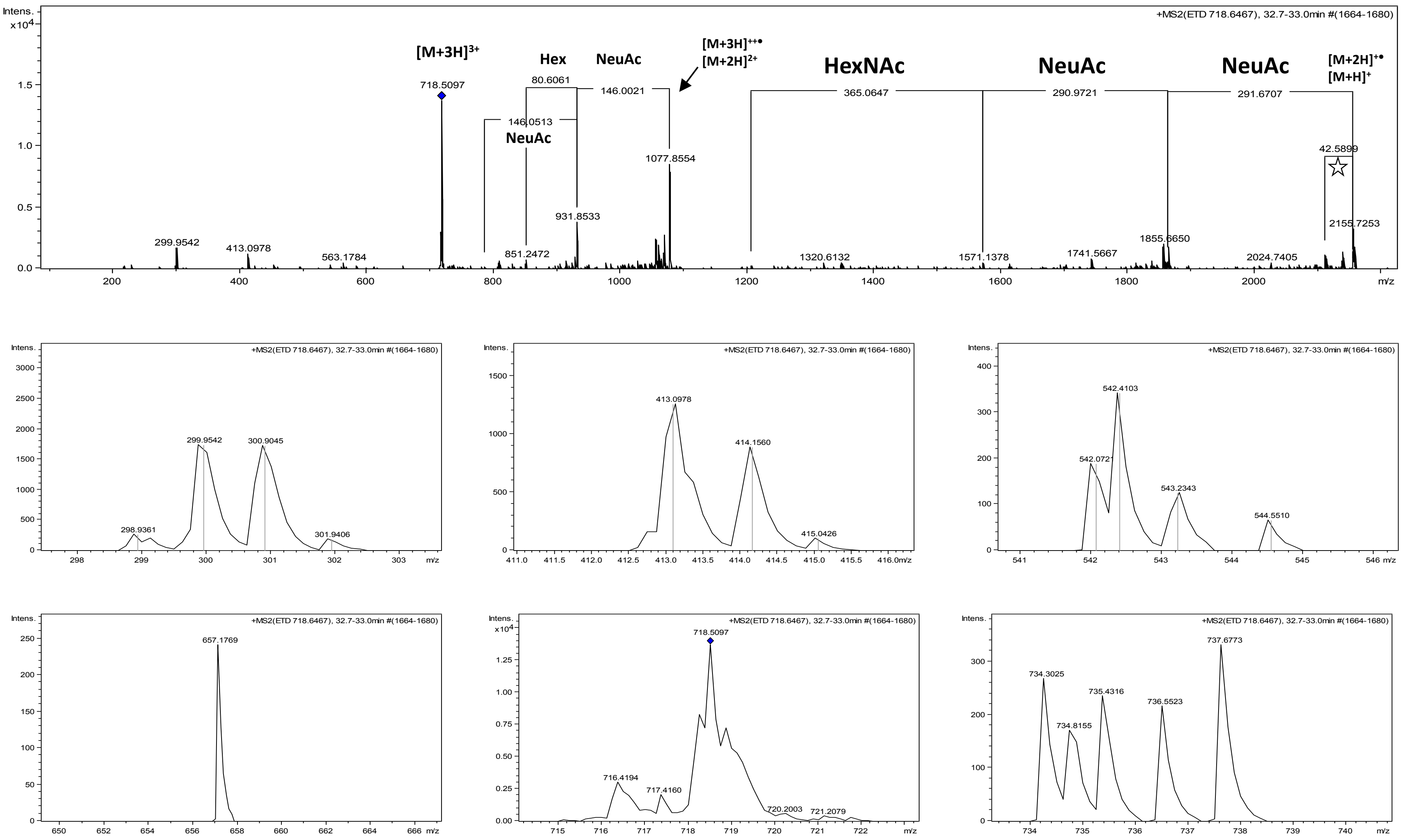

Fraction 15

718.30+++ → Pep [M+H]+ 1205.63+ [28.2-28.5 min]

ETD

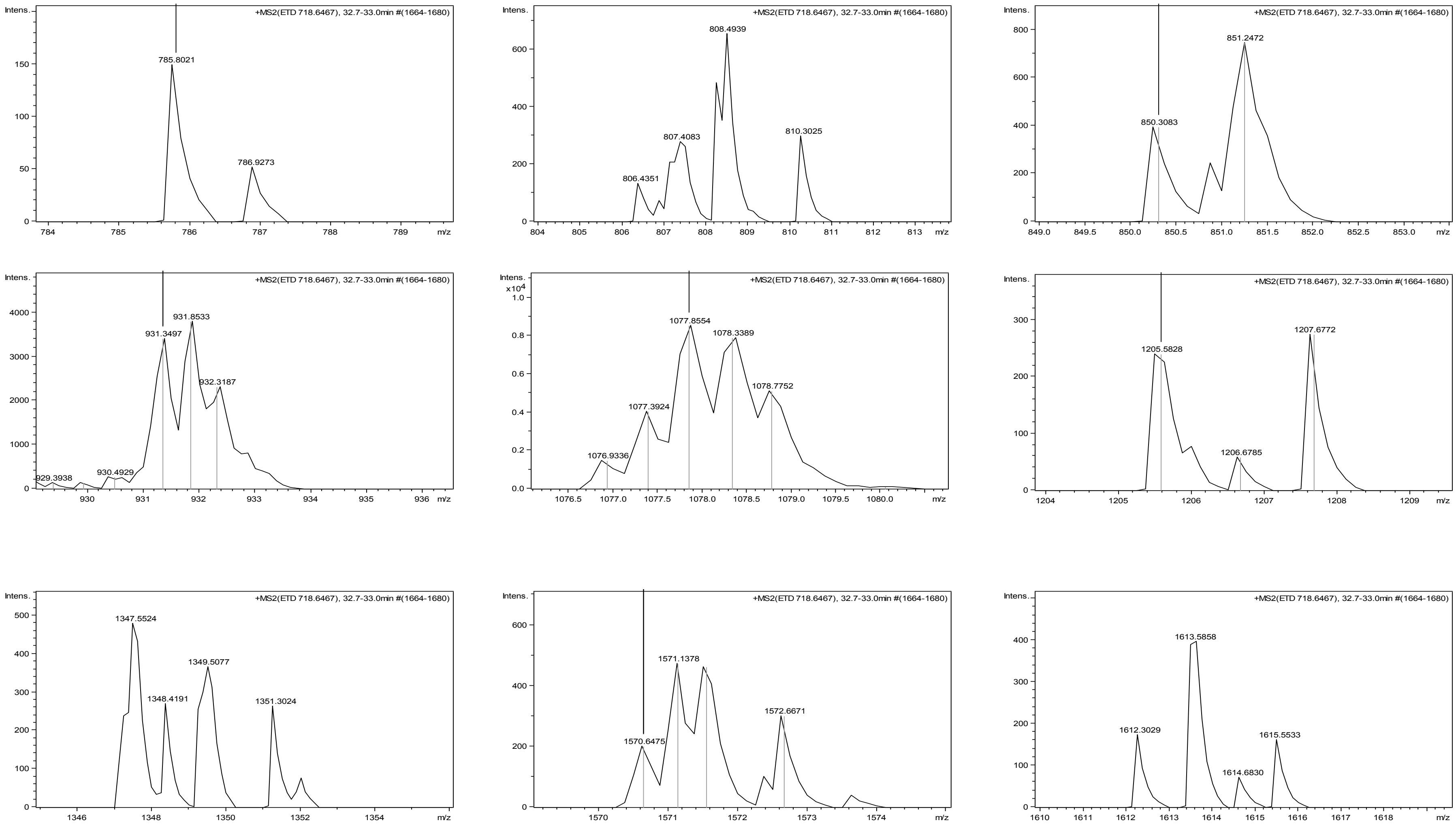

Fraction 15

718.30+++ → Pep [M+H]+ 1205.63+ [28.2-28.5 min]

ETD

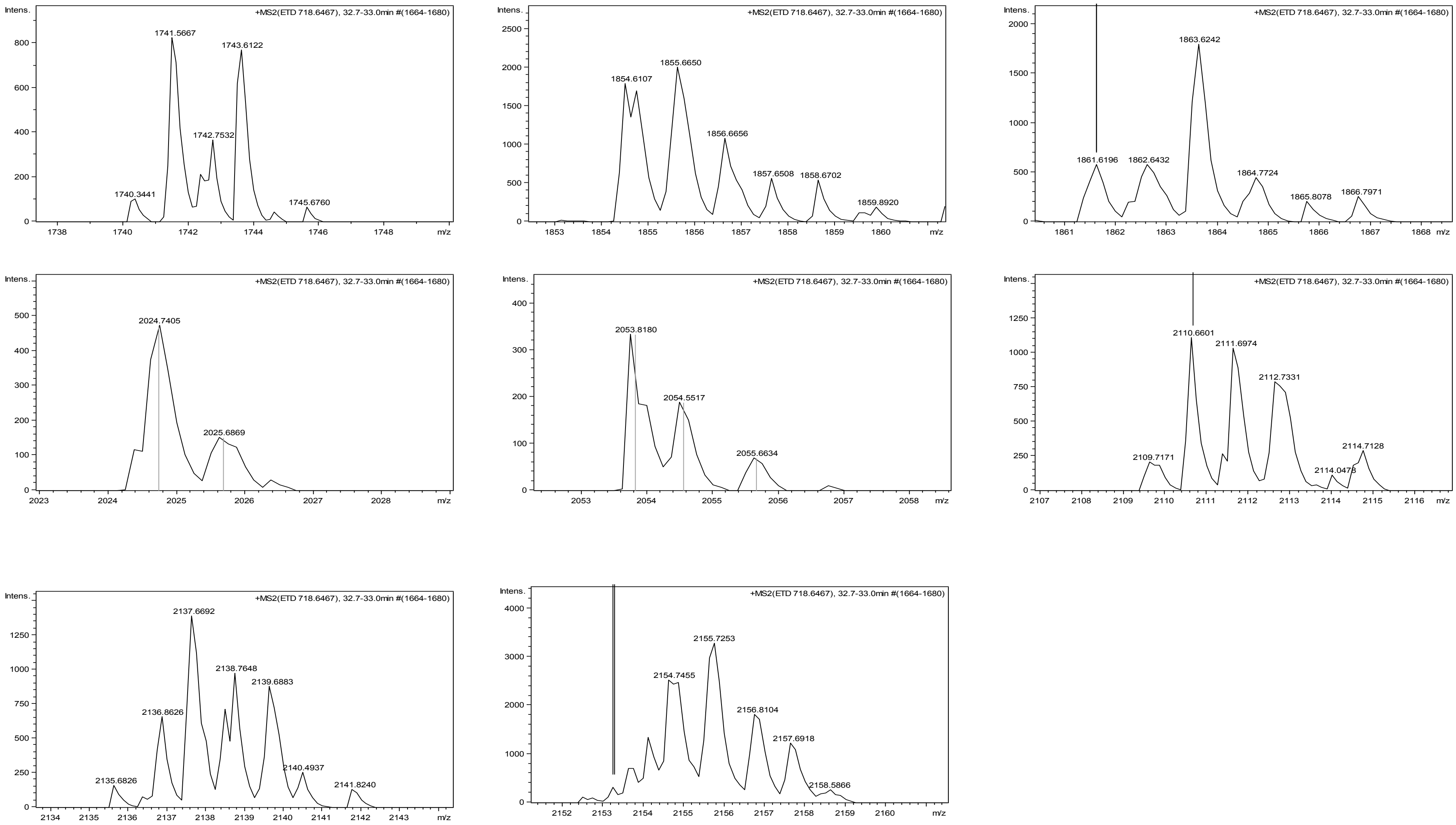

Fraction 15

718.30+++ → Pep [M+H]+ 1205.63+ [28.2-28.5 min] ETD

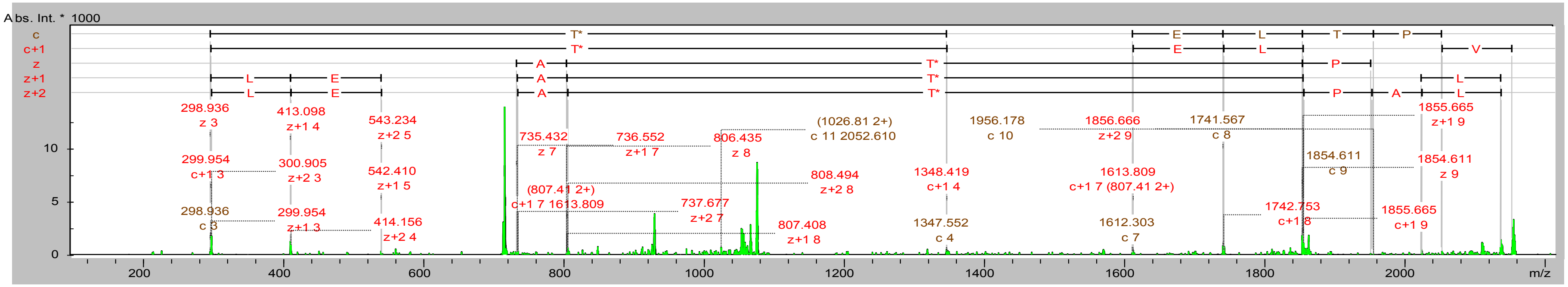

LAPTAPPELTPV **Most likely**

	L	A	P	T	A	P	P	E	L	T	P	V	Leu	Ala	Pro	Thr	Ala	Pro	Pro	Glu	Leu	Thr	Pro	Val
Ion	1	2	3	4	5	6	7	8	9	10	11	12	1	2	3	4	5	6	7	8	9	10	11	12
c	L	A	P	T*	A	P	P	E	L	T	P	V	131.118	202.155	299.208	1347.578	1418.616	1515.668	1612.721	1741.764	1854.848	1955.895	2052.948	2152.017
c+1	L	A	P	T*	A	P	P	E	L	T	P	V	132.126	203.163	300.216	1348.586	1419.623	1516.676	1613.729	1742.772	1855.856	1956.903	2053.956	2153.024
z	L	A	P	T*	A	P	P	E	L	T	P	V	101.060	198.112	299.160	412.244	541.287	638.340	735.392	806.429	1854.800	1951.853	2022.890	2135.974
z+1	L	A	P	T*	A	P	P	E	L	T	P	V	102.068	199.120	300.168	413.252	542.295	639.347	736.400	807.437	1855.808	1952.861	2023.898	2136.982
z+2	L	A	P	T*	A	P	P	E	L	T	P	V	103.075	200.128	301.176	414.260	543.302	640.355	737.408	808.445	1856.816	1953.869	2024.906	2137.990
	12	11	10	9	8	7	6	5	4	3	2	1	Val	Pro	Thr	Leu	Glu	Pro	Pro	Ala	Thr	Pro	Ala	Leu

Biotools-Score: 150

known O-glycosylation site

Plasminogen precursor

$_{362}$LAP**T**APPELTPV$_{373}$

Fraction 15

718.30+++ → Pep [M+H]+ 1205.63+ [28.2-28.5 min] ETD

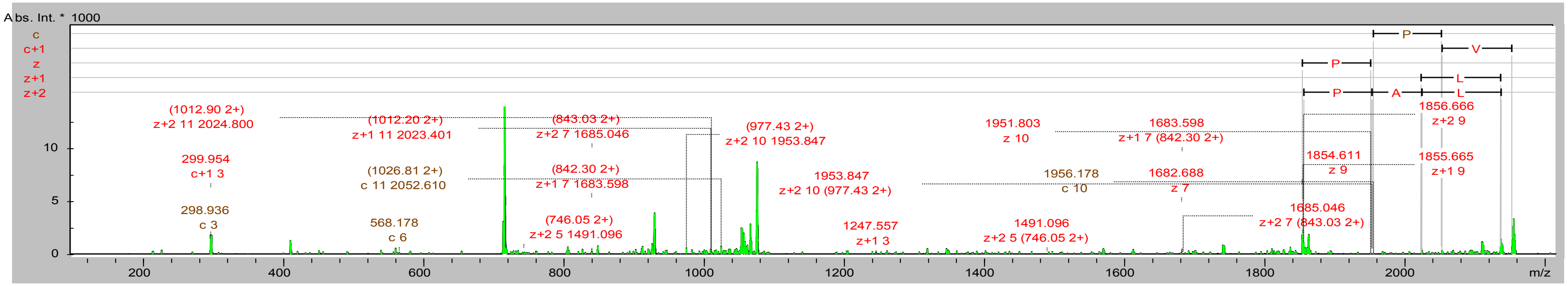

LAPTAPPELTPV

	L	A	P	T	A	P	P	E	L	T	P	V	Leu	Ala	Pro	Thr	Ala	Pro	Pro	Glu	Leu	Thr	Pro	Val
Ion	1	2	3	4	5	6	7	8	9	10	11	12	1	2	3	4	5	6	7	8	9	10	11	12
c	L	A	P	T	A	P	P	E	L	T*	P	V	131.118	202.155	299.208	400.255	471.293	568.345	665.398	794.441	907.525	1955.895	2052.948	2152.017
c+1	L	A	P	T	A	P	P	E	L	T*	P	V	132.126	203.163	300.216	401.263	472.300	569.353	666.406	795.449	908.533	1956.903	2053.956	2153.024
z	L	A	P	T	A	P	P	E	L	T*	P	V	101.060	198.112	1246.483	1359.567	1488.610	1585.663	1682.715	1753.752	1854.800	1951.853	2022.890	2135.974
z+1	L	A	P	T	A	P	P	E	L	T*	P	V	102.068	199.120	1247.491	1360.575	1489.618	1586.670	1683.723	1754.760	1855.808	1952.861	2023.898	2136.982
z+2	L	A	P	T	A	P	P	E	L	T*	P	V	103.075	200.128	1248.499	1361.583	1490.625	1587.678	1684.731	1755.768	1856.816	1953.869	2024.906	2137.990
	12	11	10	9	8	7	6	5	4	3	2	1	Val	Pro	Thr	Leu	Glu	Pro	Pro	Ala	Thr	Pro	Ala	Leu

Biotools-Score: 36

known O-glycosylation site

Plasminogen precursor

$_{362}$LAP**T**APPELTPV$_{373}$
