# Supplementary material for: Site-specific O-Glycosylation Analysis of Human Blood Plasma Proteins
Source: Mol Cell Proteomics. 2015 Nov 23;15(2):624–41. doi: 10.1074/mcp.M115.053546 (PMC4739677; doi:10.1074/mcp.M115.053546)
Supplement: Supplemental Data [file 10.1074_M115.053546_mcp.M115.053546-6.pdf]

Site-Specific *O*-Glycosylation Analysis of Human Blood Plasma Proteins

Proteinase K Digest

Fraction 16

Search Parameters For Protein Identification

Project: Blood Plasma Glycoprotomics (2013), ProtK-Digest
Glycopeptides measured on Bruker ESI-Ion Trap MS (CID-MS³)

Mascot version 2.2.07	
Database:	SwissProt
Fasta file:	SwissProt_51.6.fasta
Total sequences:	257964
Total residues:	93947433
Sequences after taxonomy filter:	15720
Number of queries:	1

Variable modifications ---

Identifier	*Name*	*Delta*	*Neutral loss(es)*
1	Deamidated (NQ)	0.984009	0
2	Oxidation (M)	15.994919	63.998285

Search Parameters ---

Taxonomy filter:	Homo sapiens (human)
Enzyme:	None
Maximum Missed Cleavages:	0
Fixed modifications	Carbamidomethyl (C)
ICAT experiment	0
Variable modifications	Deamidated (NQ), Oxidation (M)
Peptide Mass Tolerance	0.3
Peptide Mass Tolerance Units	Da
Fragment Mass Tolerance	0.35
Fragment Mass Tolerance Units	Da
Mass values	Monoisotopic
Instrument type	ESI-TRAP
Isotope error mode	1

Format parameters ---

Significance threshold	0.05
Max. number of hits	20
Use MudPIT protein scoring	0
Ions score cut-off	0
Include same-set proteins	0
Include sub-set proteins	0
Include unassigned	0
Require bold red	0

Extracted ion chromatograms of glycan-specific oxonium ions

Oxonium Ions	$[M+H]^+$ m/z
Fuc	147.08
Hex	163.06
HexNAc	204.09
NeuAc -H_2O	274.09
NeuAc	292.10
HexNAc(1)Hex(1)	366.14
Hex(1)NeuAc(1)	454.16
HexNAc(1)NeuAc(1)	495.18
HexNAc(1)Hex(1)Fuc(1)	512.21
HexNAc(1)Hex(2)	528.19
HexNAc(1)Hex(1)NeuAc(1)	657.24

Supplementary Figure 4: Human Blood Plasma O-Glycoproteomics, HILIC Fraction 16

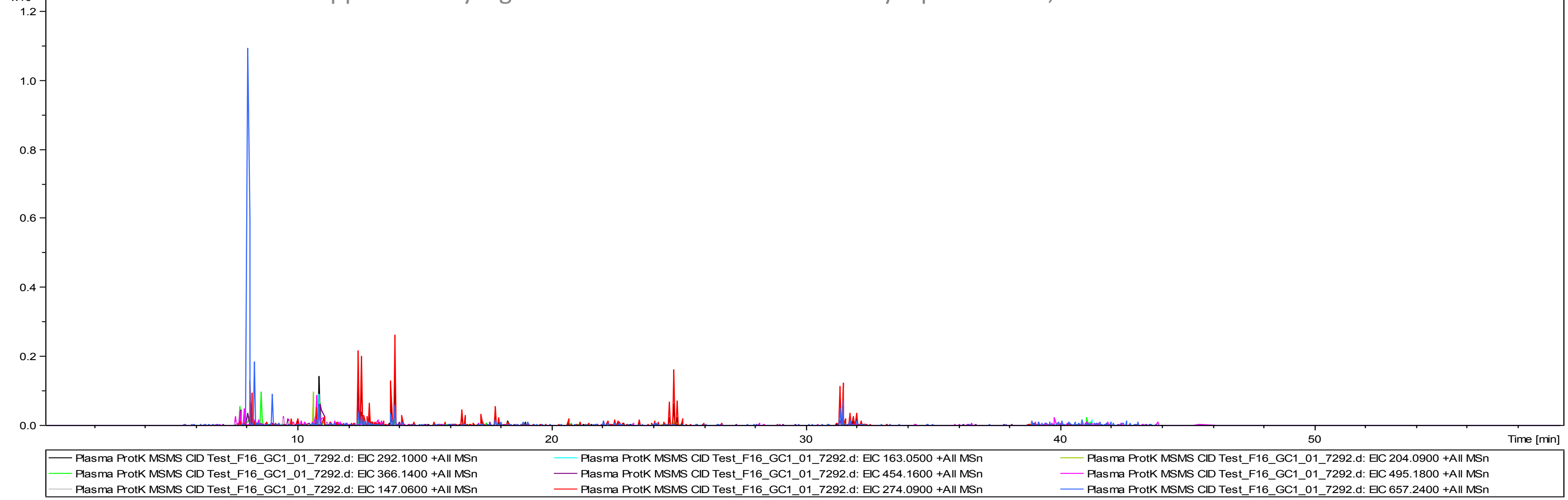

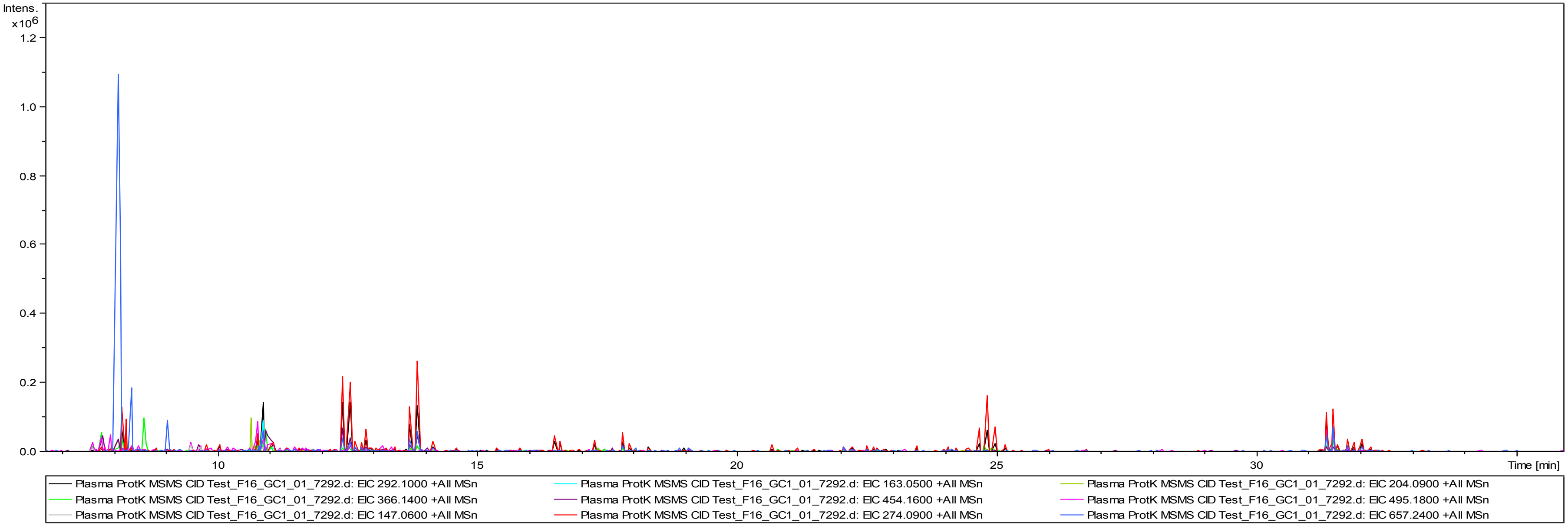

Extracted ion chromatograms of glycan-specific oxonium ions

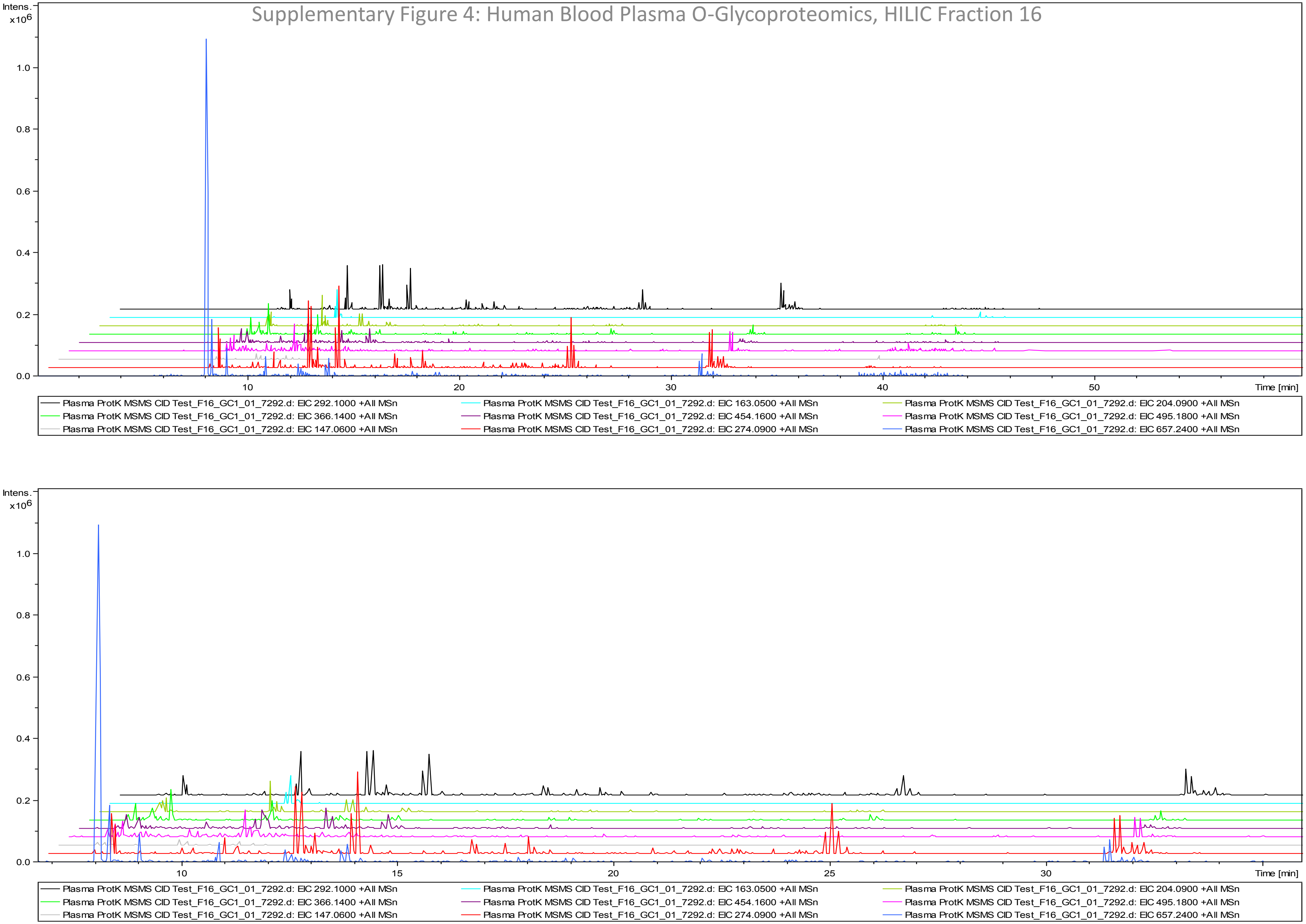

Extracted ion chromatograms of glycan-specific oxonium ions

Supplementary Figure 4: Human Blood Plasma O-Glycoproteomics, HILIC Fraction 16

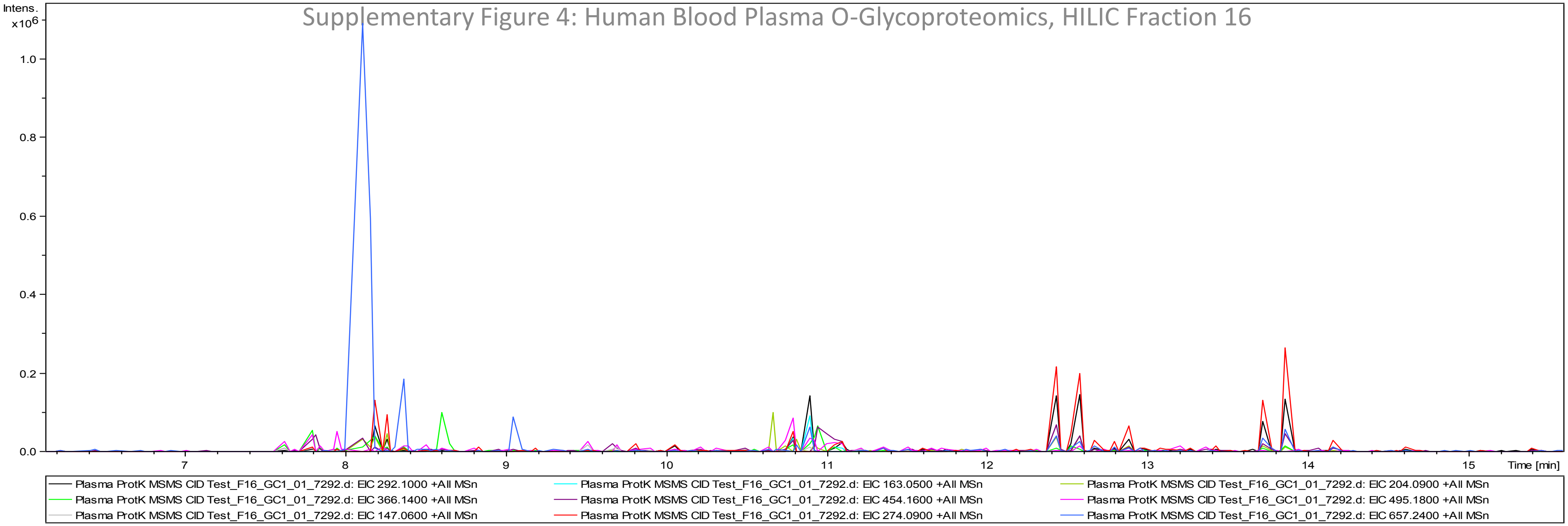

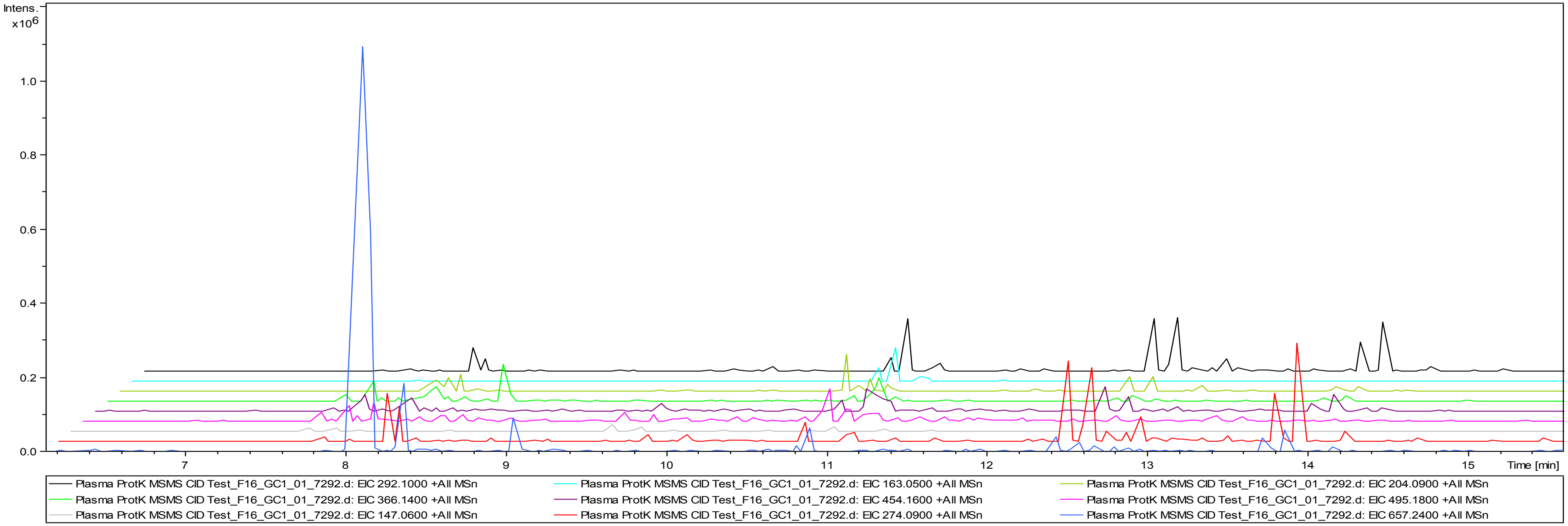

Extracted ion chromatograms of glycan-specific oxonium ions

Supplementary Figure 4: Human Blood Plasma O-Glycoproteomics, HILIC Fraction 16

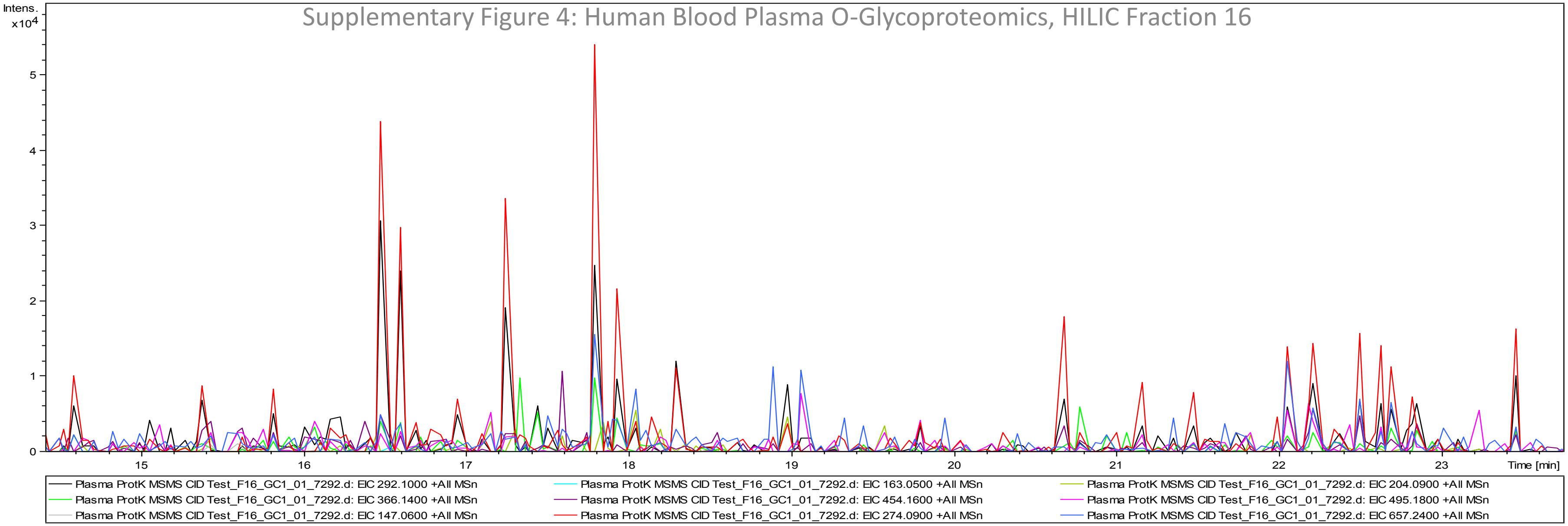

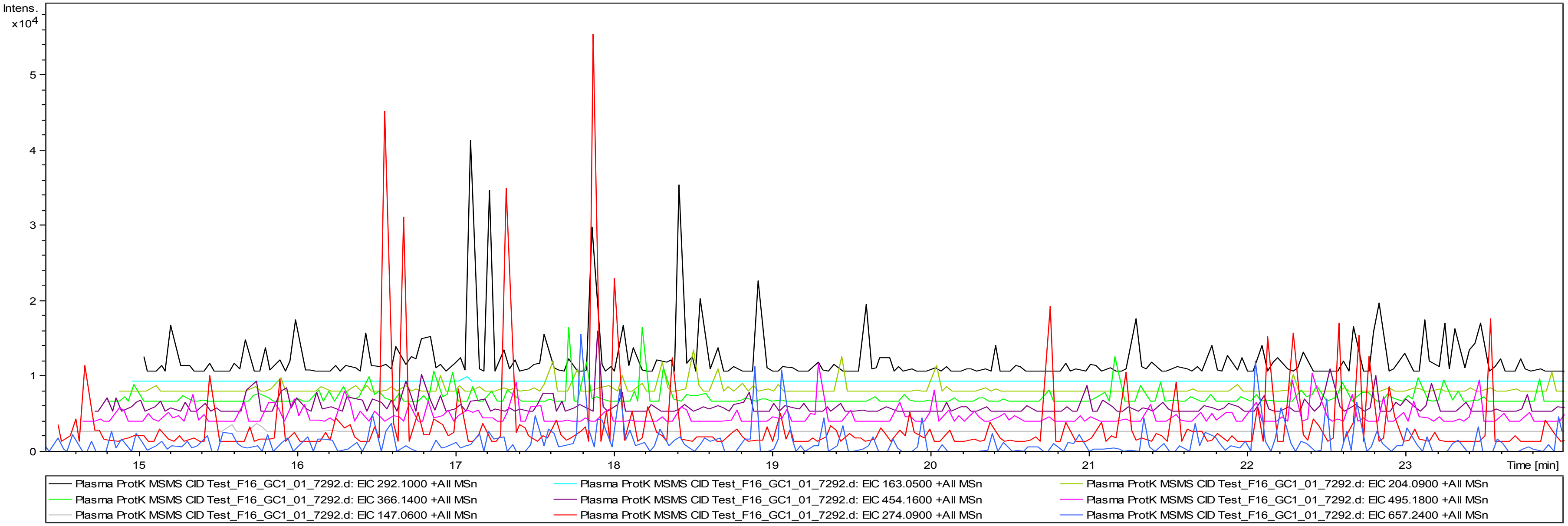

Extracted ion chromatograms of glycan-specific oxonium ions

Supplementary Figure 4: Human Blood Plasma O-Glycoproteomics, HILIC Fraction 16

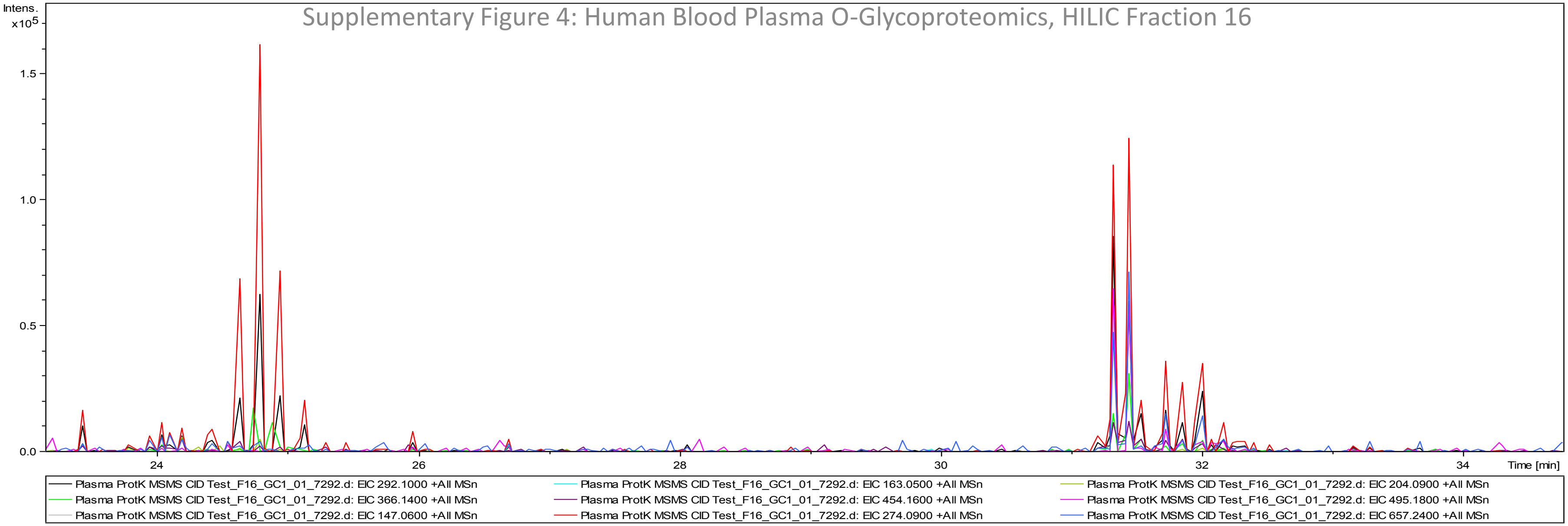

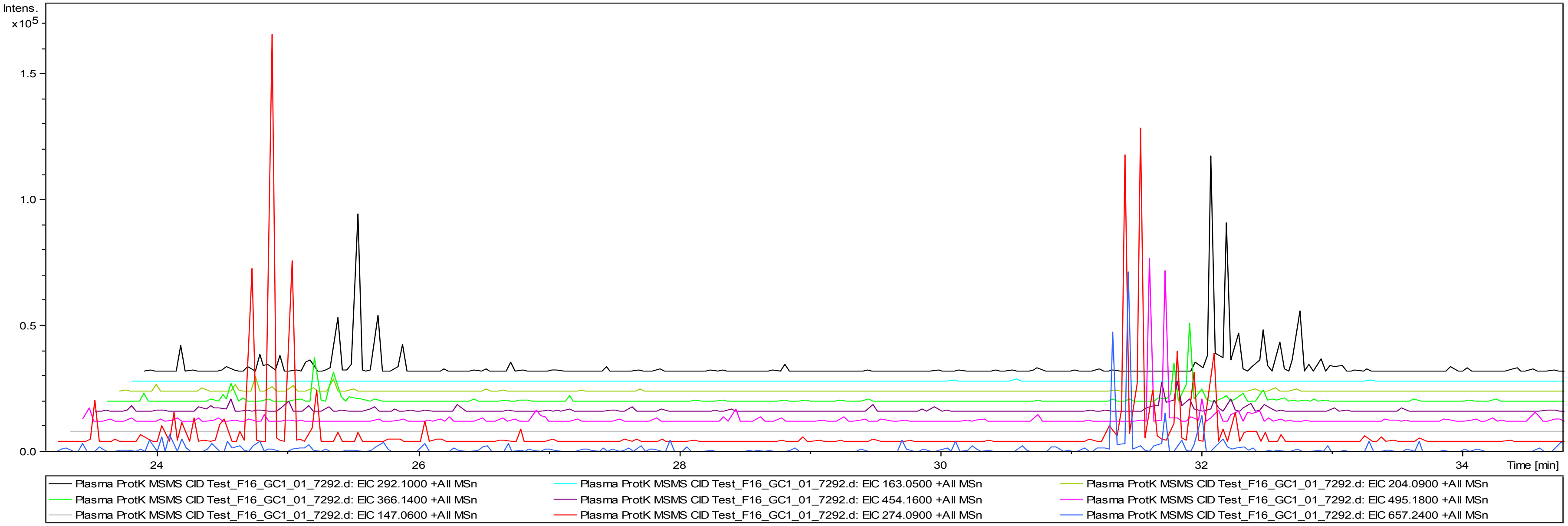

Extracted ion chromatograms of glycan-specific oxonium ions

Fraction 16

643.65++ → Pep [M+H]+ 630.30+ [8.2-8.3 min]

CID-MS Precursor

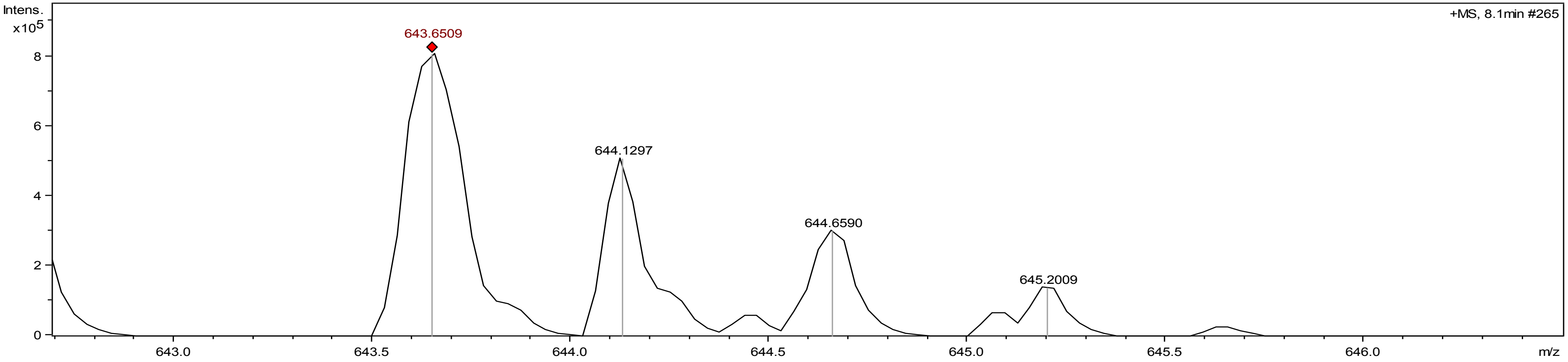

ETD spectrum not available

Fraction 16

643.65++ → Pep [M+H]+ 630.30+ [8.2-8.3 min] CID-MS2

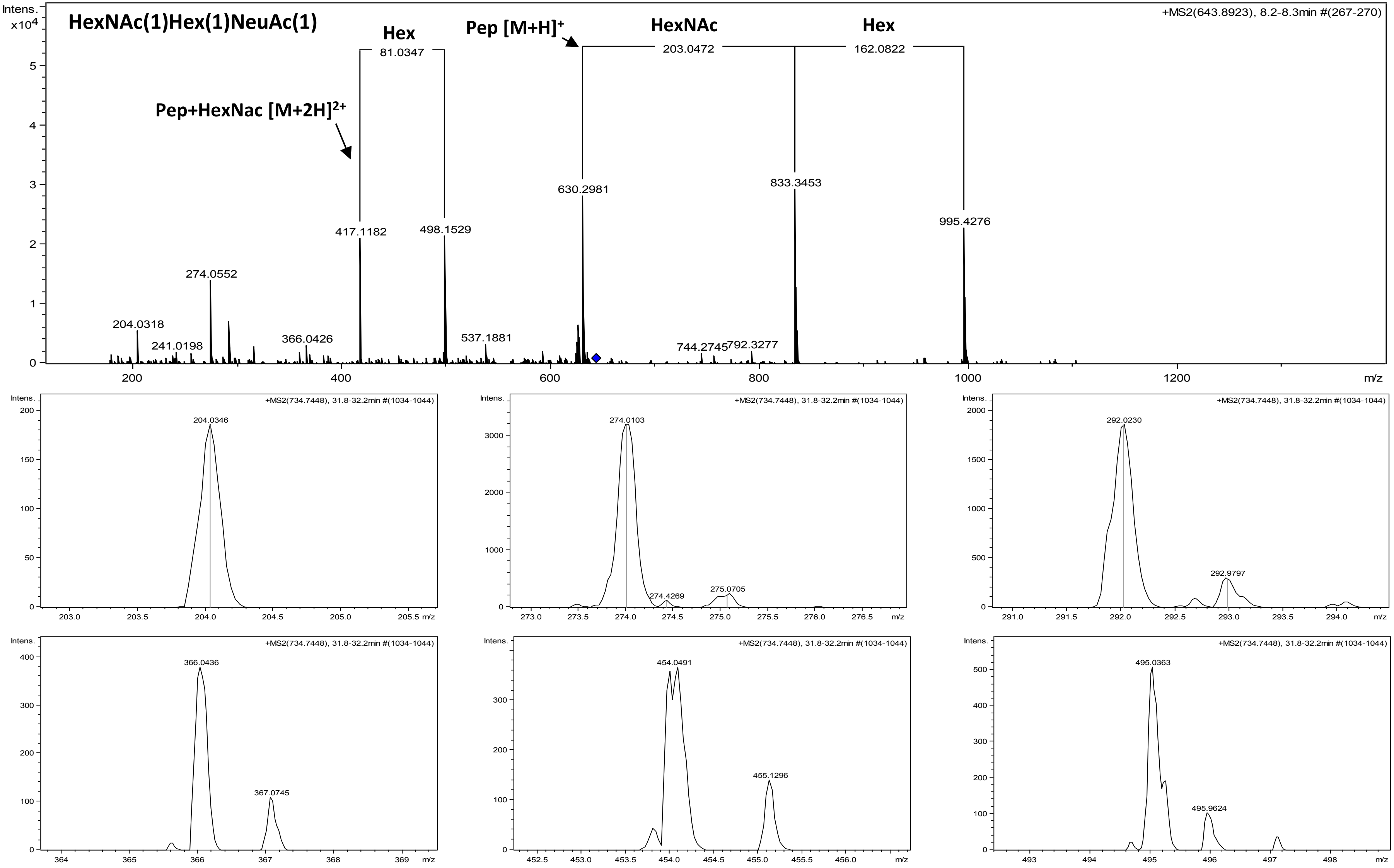

Fraction 16

643.65++ → Pep [M+H]+ 630.30+ [8.2-8.3 min]

CID-MS2

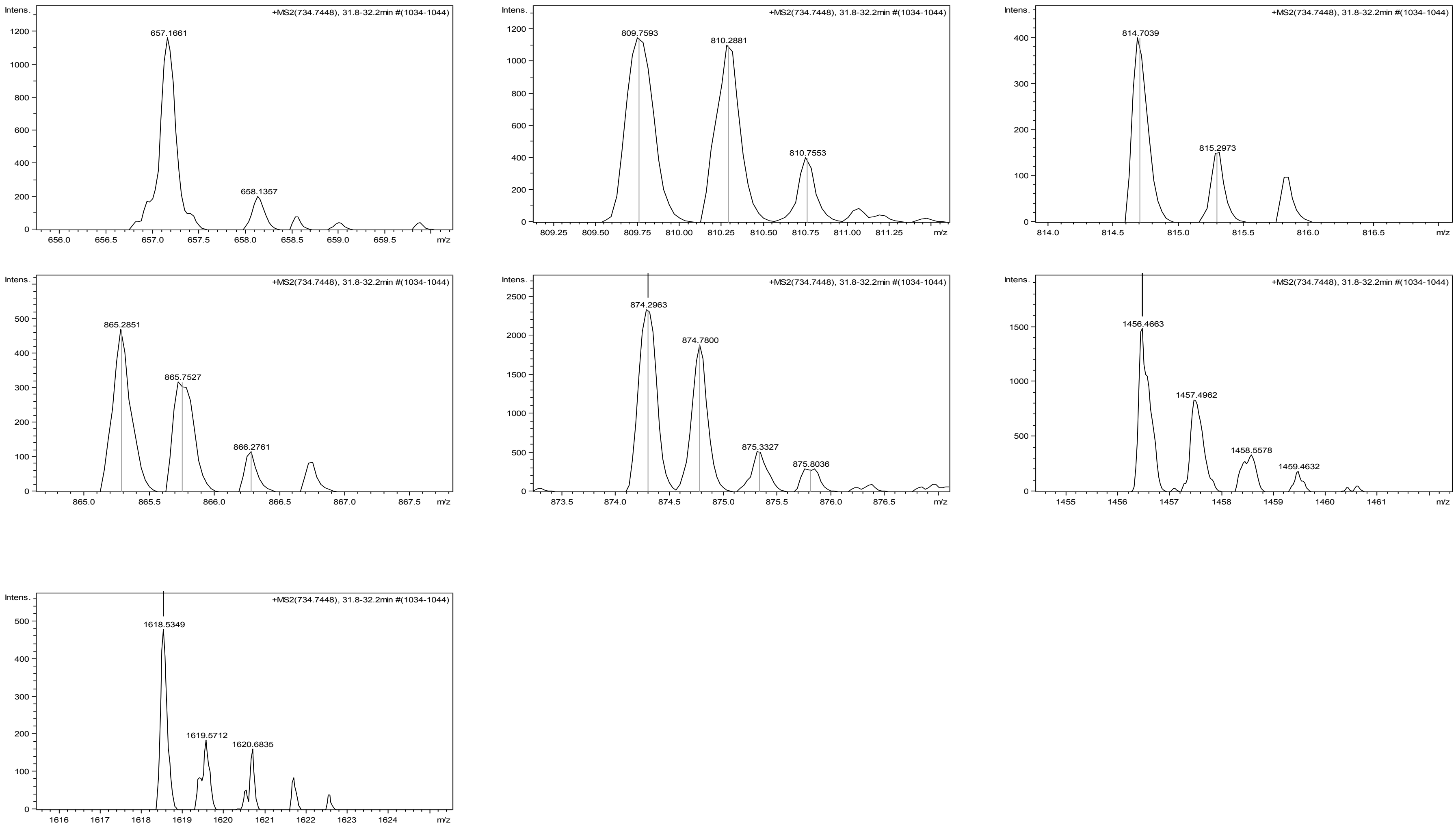

Fraction 16

643.65++ → Pep [M+H]+ 630.30+ [8.2-8.3 min] CID-MS3 MASCOT Search

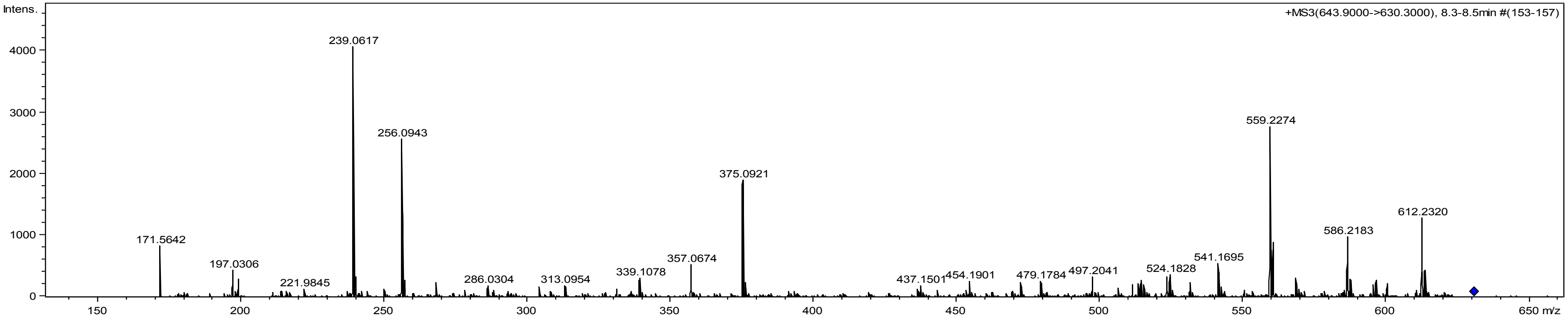

prot_hit_nur	prot_acc	prot_desc	prot_score	prot_mass	prot_matche	prot_matche	prot_sequen	prot_sequen	pep_query	pep_rank	pep_isbold	pep_isuniqu	pep_exp_mz	pep_exp_mr	pep_exp_z	pep_calc_mr	pep_delta	pep_miss	pep_score	pep_expect	pep_res_bef	pep_seq
1	MYOM3_HUI	Myomesin-3	17	163229	1	0	1	0	1	1	1	1	630.2981	629.2908	1	629.3384	-0.0476	0	23.82	63	Q	ALATPSA
2	M3K6_HUMA	Mitogen-acti	16	144217	1	0	1	0	1	1	0	1	630.2981	629.2908	1	629.302	-0.0112	0	23.82	63	S	ASPTPSA
3	GGN_HUMAI	Gametogene	15	67000	1	0	1	0	1	3	0	1	630.2981	629.2908	1	629.3384	-0.0476	0	20.83	1.30E+02	P	ALATPAS
4	PHRF1_HUM	PHD and RIN	14	180268	1	0	1	0	1	3	0	1	630.2981	629.2908	1	629.3384	-0.0476	0	20.83	1.30E+02	P	AALTPAS
5	ATX2_HUMA	Ataxin-2 OS=	13	140823	1	0	1	0	1	3	0	1	630.2981	629.2908	1	629.302	-0.0112	0	20.83	1.30E+02	A	ASPTPAS
6	NRG3_HUMA	Pro-neuregu	13	78878	1	0	1	0	1	8	0	1	630.2981	629.2908	1	629.302	-0.0112	0	18.72	2.00E+02	T	SPATPSA
7	TACC3_HUM	Transformin	13	91159	1	0	1	0	1	6	0	1	630.2981	629.2908	1	629.3497	-0.0588	0	18.97	1.90E+02	R	RVTPAS
8	HCN4_HUMA	Potassium/s	12	129645	1	0	1	0	1	8	0	1	630.2981	629.2908	1	629.302	-0.0112	0	18.72	2.00E+02	P	SAPTPSA
9	SUGP1_HUM	SURP and G-	10	72540	1	0	1	0	1	10	0	1	630.2981	629.2908	1	629.302	-0.0112	0	16.28	3.60E+02	A	PTSAPSA
10	MINT_HUMA	Msx2-interac	10	403030	1	0	1	0	1	6	0	1	630.2981	629.2908	1	629.3497	-0.0589	0	18.97	1.90E+02	S	VRTPGT

No unambiguous result

Fraction 16

507.15+++ → Pep [M+2H]++ 432.17++ [12.4-12.9 min]   CID-MS Precursor

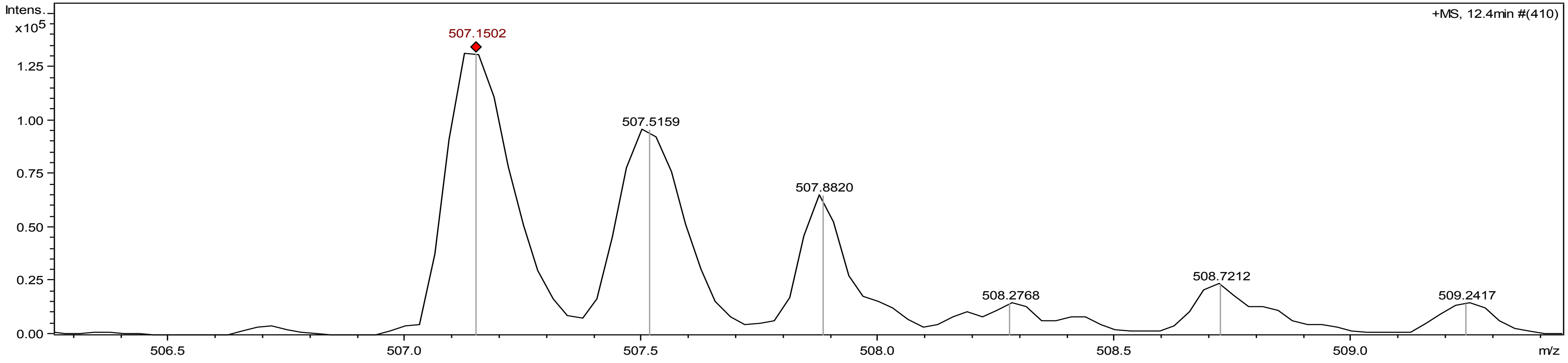

Not Sure

Fraction 16

507.15+++ → Pep [M+2H]++ 432.17++ [12.4-12.9 min] CID-MS2

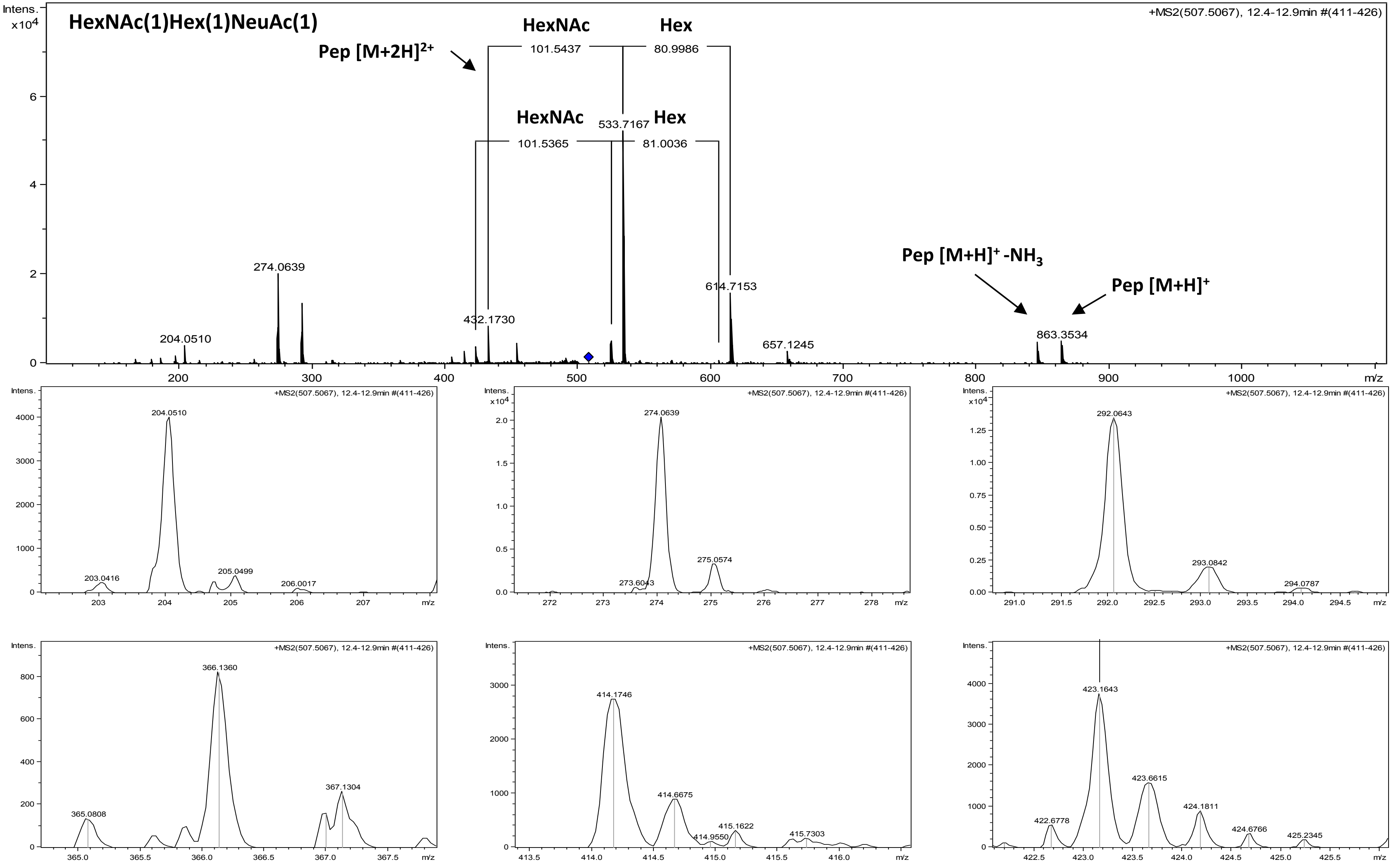

Fraction 16

507.15+++ → Pep [M+2H]++ 432.17++ [12.4-12.9 min]

CID-MS2

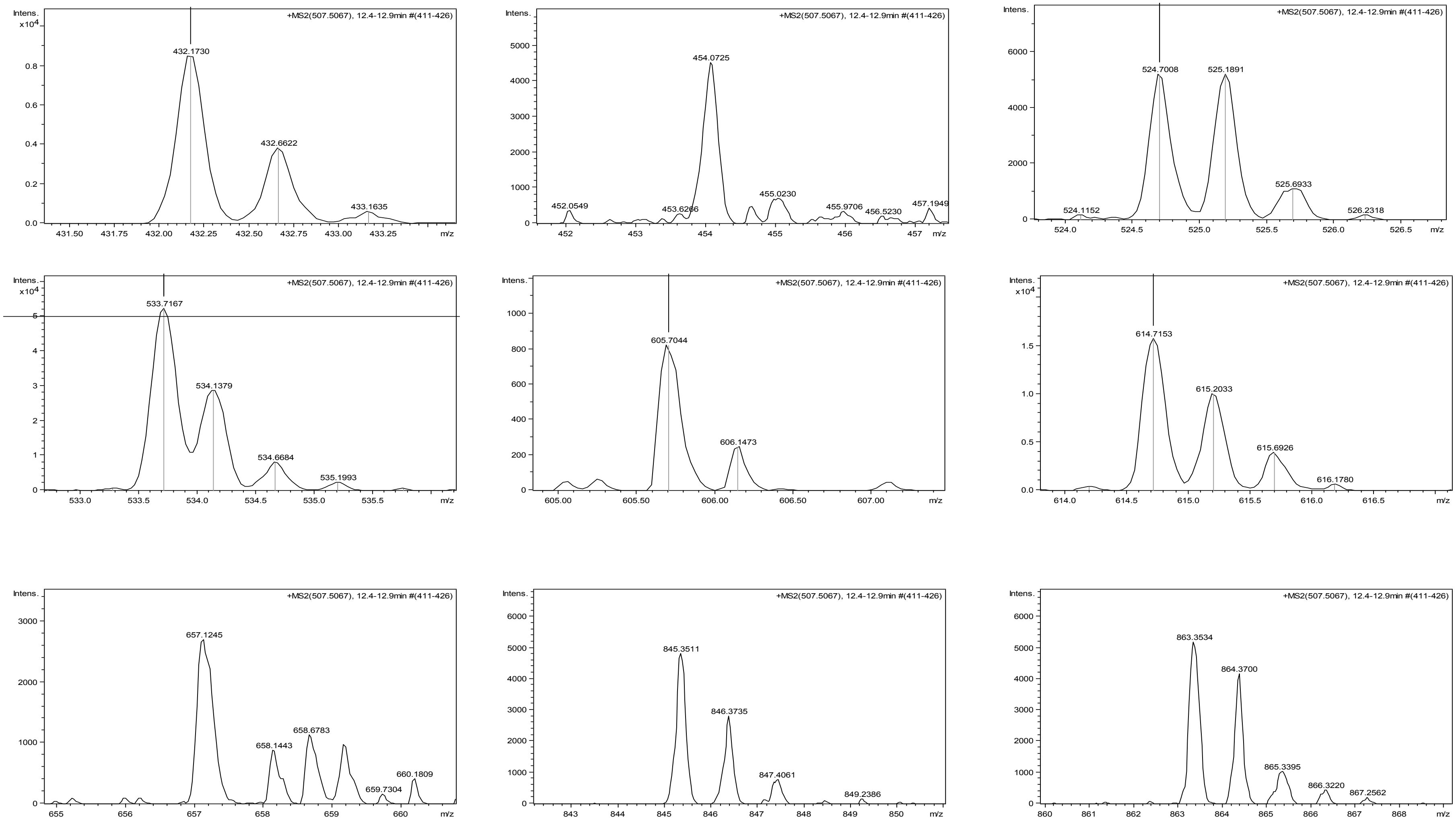

Fraction 16

507.15+++ → Pep [M+2H]++ 432.17++ [12.4-12.9 min] CID-MS3

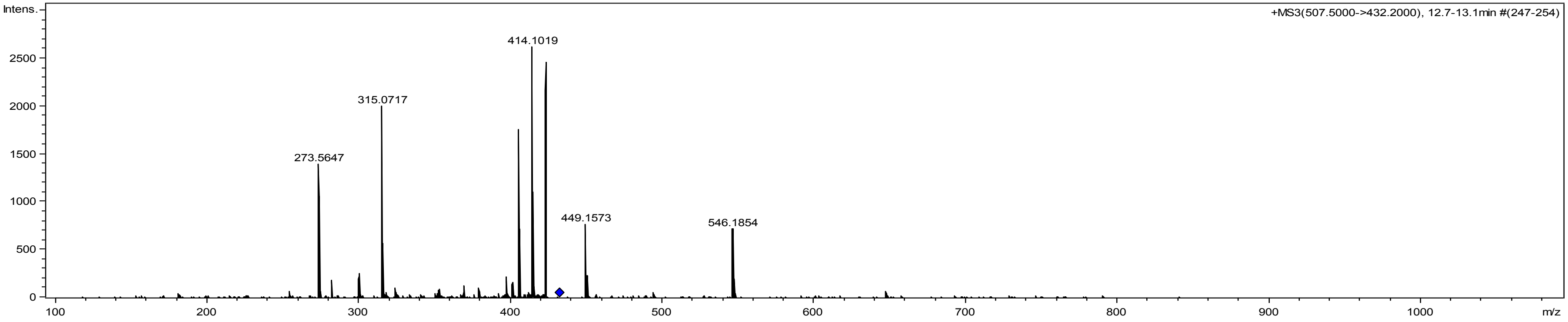

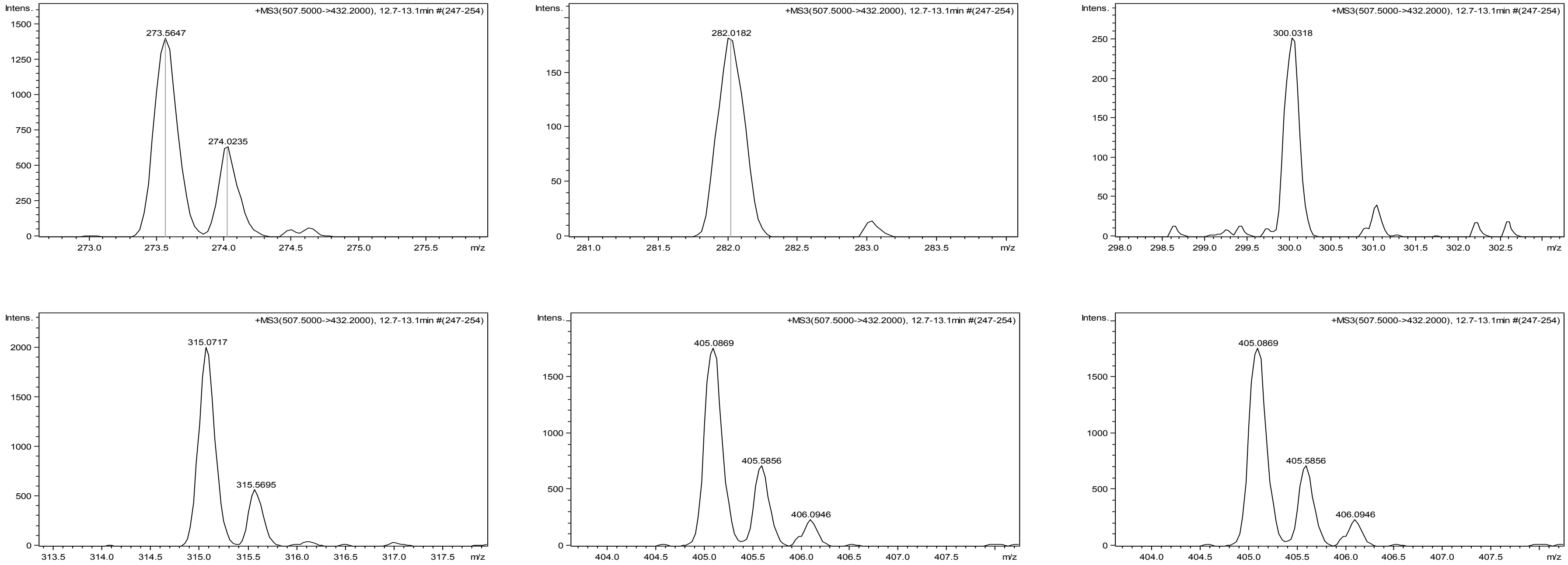

Fraction 16

507.15+++ → Pep [M+2H]++ 432.17++ [12.4-12.9 min] CID-MS3

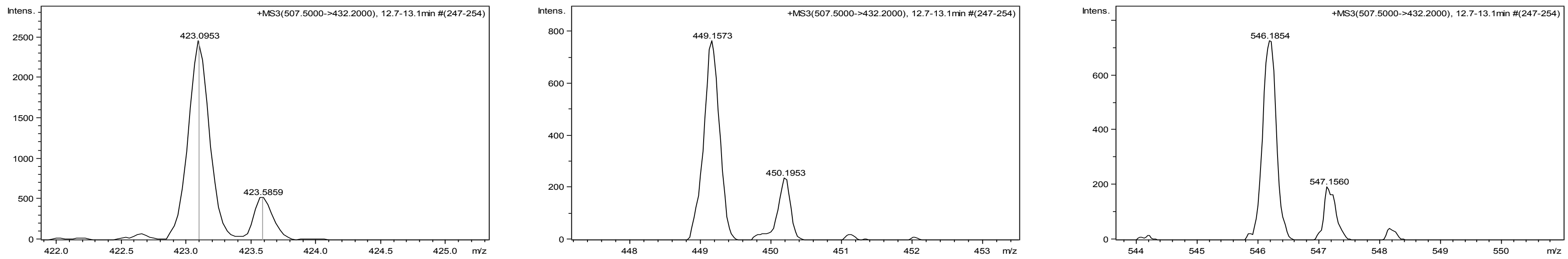

Fraction 16

507.15+++ → Pep [M+2H]++ 432.17++ [12.4-12.9 min]

CID-MS3 MASCOT Search

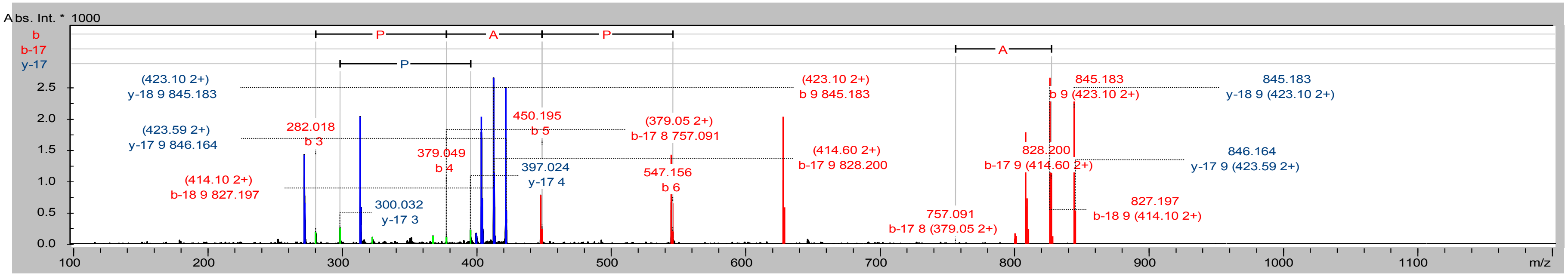

	G	S	H	P	A	P	A	R	A	Gly	Ser	His	Pro	Ala	Pro	Ala	Arg	Ala
Ion	1	2	3	4	5	6	7	8	9	1	2	3	4	5	6	7	8	9
b	G	S	H	P	A	P	A	R	A	58.029	145.061	282.120	379.172	450.210	547.262	618.299	774.401	845.438
b-17	G	S	H	P	A	P	A	R	A	-	-	-	-	-	-	-	757.374	828.411
b-18	G	S	H	P	A	P	A	R	A	-	127.050	264.109	361.162	432.199	529.252	600.289	756.390	827.427
y	G	S	H	P	A	P	A	R	A	90.055	246.156	317.193	414.246	485.283	582.336	719.395	806.427	863.448
y-17	G	S	H	P	A	P	A	R	A	-	229.130	300.167	397.219	468.257	565.309	702.368	789.400	846.422
y-18	G	S	H	P	A	P	A	R	A	-	-	-	-	-	-	-	788.416	845.438
	9	8	7	6	5	4	3	2	1	Ala	Arg	Ala	Pro	Ala	Pro	His	Ser	Gly

Fraction 16

507.15+++ → Pep [M+2H]++ 432.17++ [12.4-12.9 min]

CID-MS3 MASCOT Search

prot_hit_nur	prot_acc	prot_desc	prot_score	prot_mass	prot_matche	pep_query	pep_rank	pep_isbold	pep_exp_mz	pep_exp_mr	pep_exp_z	pep_calc_mr	pep_delta	pep_miss	pep_score	pep_expect	pep_res_bef	pep_seq
1	KISSR_HUMA	KiSS-1 recep	15	43414	1	1	1	1	432.173	862.3314	2	862.4409	-0.1095	0	19.86	1.60E+02	L	GSHPAPARA
2	PKR1_HUMA	Prokineticin	13	45481	1	1	2	0	432.173	862.3314	2	862.3742	-0.0428	0	17.94	2.50E+02	T	IGMPATEE
3	NPFF2_HUM	Neuropeptic	10	60858	1	1	3	0	432.173	862.3314	2	862.3709	-0.0394	0	16.27	3.60E+02	P	IWNVNDT
4	CK024_HUM	Uncharacteri	10	46171	1	1	3	0	432.173	862.3314	2	861.3902	0.9413	0	16.27	3.60E+02	N	KSTPMPSN
5	GPR81_HUM	Probable G-p	10	40067	1	1	3	0	432.173	862.3314	2	862.392	-0.0605	0	16.27	3.60E+02	R	IEGDTISQ
6	SNX17_HUM	Sorting nexir	10	53153	1	1	3	0	432.173	862.3314	2	862.4436	-0.1122	0	16.27	3.60E+02	L	FSLTPAEV
7	SPG16_HUM	Sperm-assoc	9	71716	1	1	3	0	432.173	862.3314	2	862.3709	-0.0394	0	16.27	3.60E+02	K	IWDVNSE
8	CTNA3_HUM	Catenin alph	9	100772	1	1	3	0	432.173	862.3314	2	862.3742	-0.0428	0	16.27	3.60E+02	Q	GIQNMTTP
9	SETD2_HUM	Histone-lysir	8	290393	1	1	3	0	432.173	862.3314	2	862.3709	-0.0394	0	16.27	3.60E+02	Q	AYVDPSNP
10	MYCB2_HUM	Probable E3	7	517856	1	1	3	0	432.173	862.3314	2	862.392	-0.0605	0	16.27	3.60E+02	L	ETSPITDT

Biotools-Score: 13

MASCOT-Score: 20

Not Sure

unknown O-glycosylation site

KiSS-Receptor-1

$_{367}$G**S**HPAPARA$_{375}$

Fraction 16

507.15+++ → Pep [M+2H]++ 432.17++ [12.4-12.9 min]

CID-MS3 Biotools

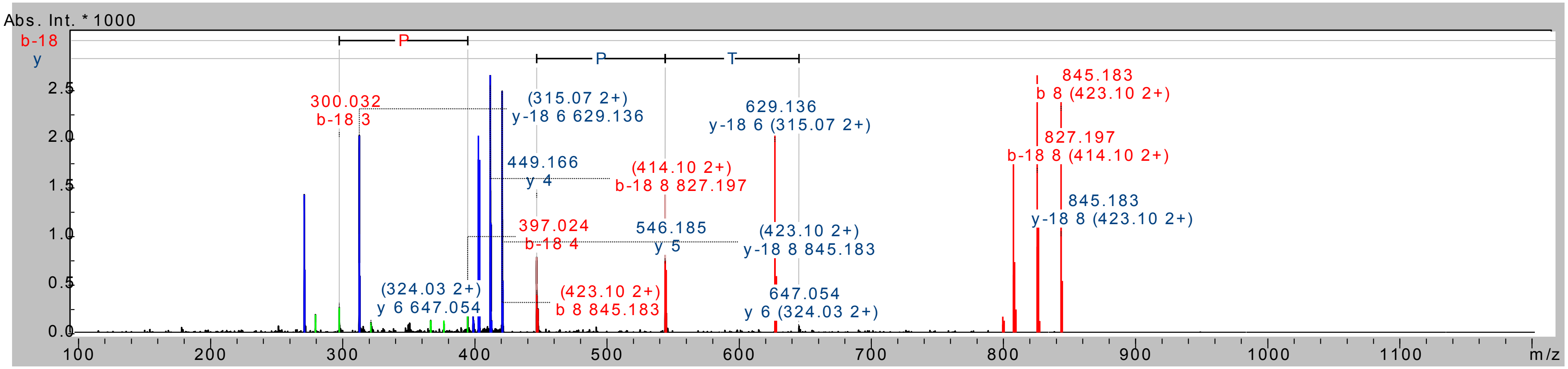

Theoretical fragment ions matched to spectrum using Biotools

Biotools-Score: 8

More likely

(Refer to subsequent glycopeptide 760.24 [M+2H]$^{2+}$)

known O-glycosylation region

Inter-alpha-trypsin inhibitor heavy chain H2

$_{689}$**ST**PPPHV$_{696}$ — printed as $_{689}$ESTPPPHV$_{696}$ with "ST" in red

Fraction 16

507.15+++ → Pep [M+2H]++ 432.17++ [12.4-12.9 min] ETD

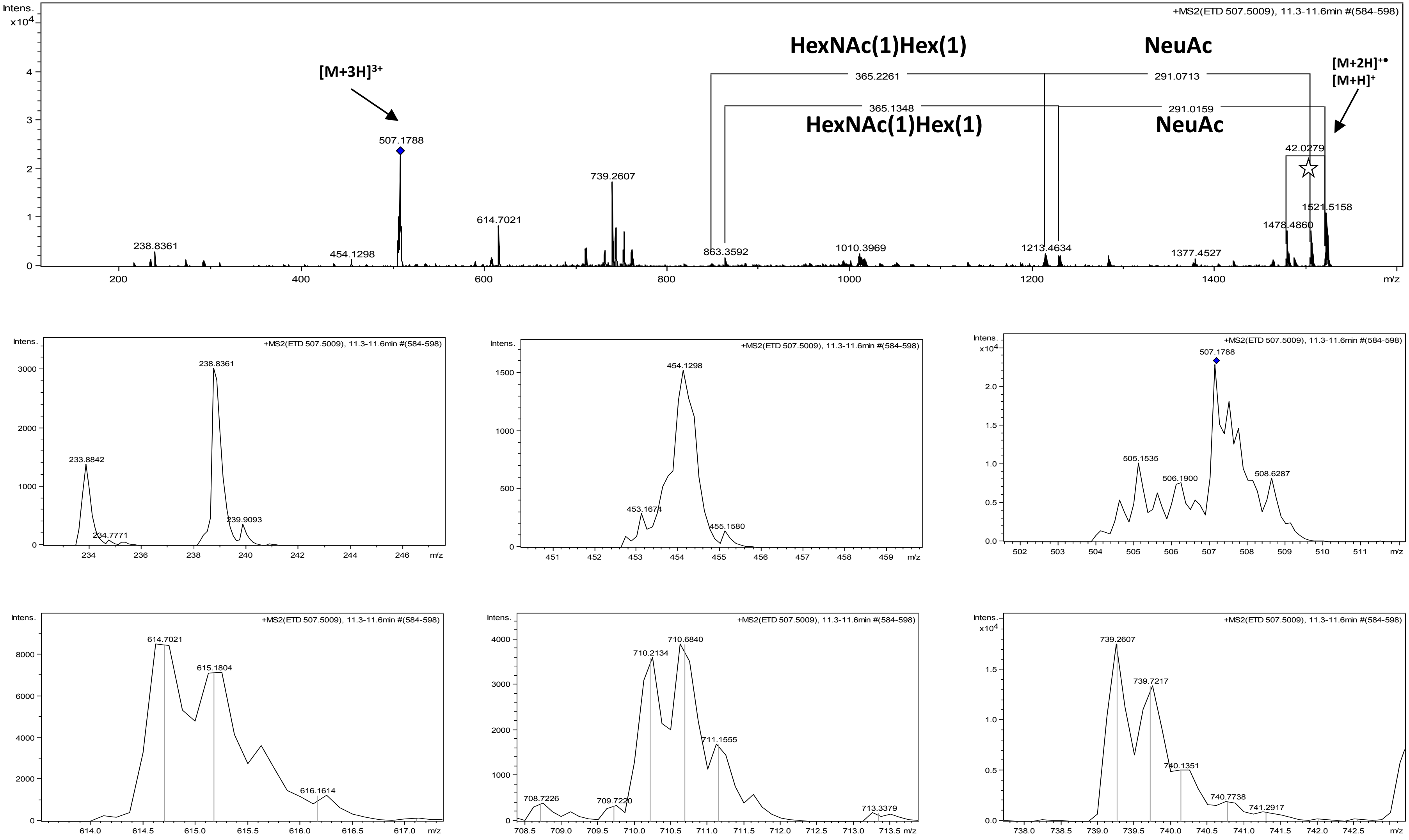

Fraction 16

507.15+++ → Pep [M+2H]++ 432.17++ [12.4-12.9 min] ETD

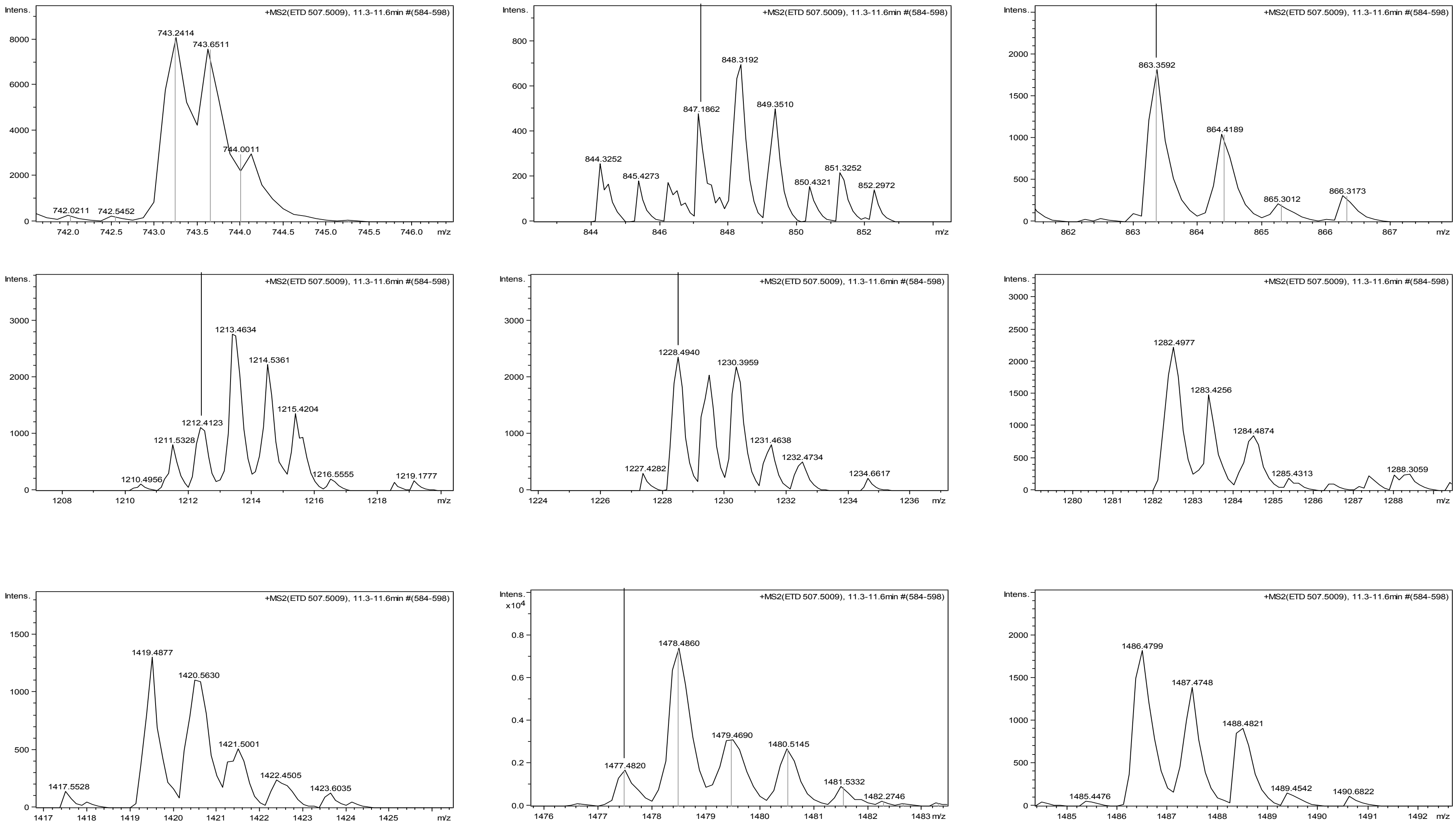

Fraction 16

507.15+++ → Pep [M+2H]++ 432.17++ [12.4-12.9 min] ETD

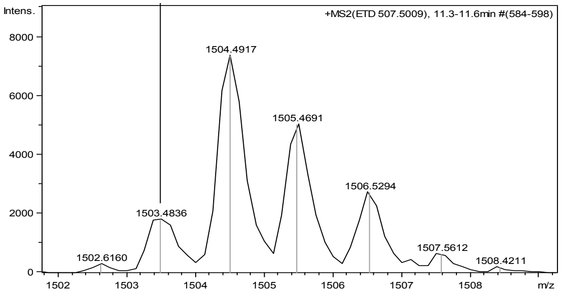

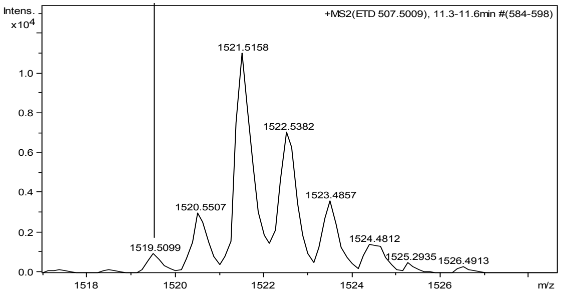

Fraction 16

507.15+++ → Pep [M+2H]++ 432.17++ [12.4-12.9 min] ETD

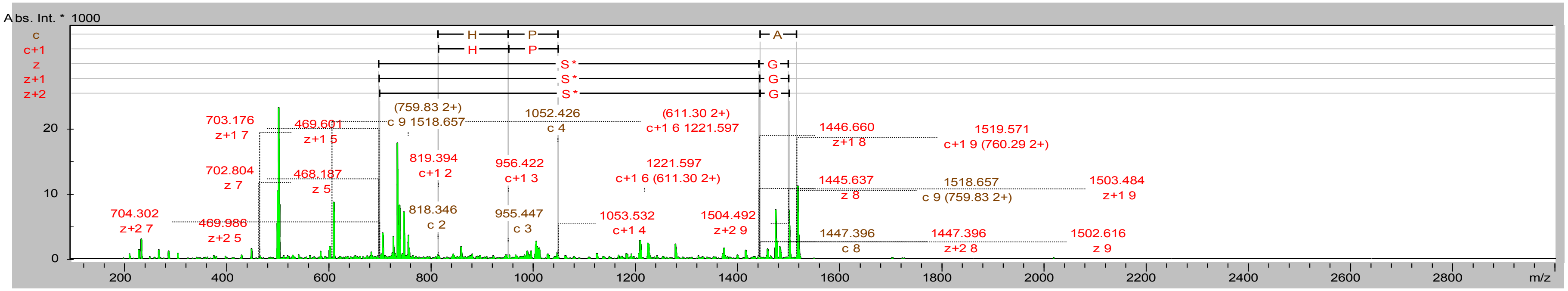

	G	S	H	P	A	P	A	R	A	Gly	Ser	His	Pro	Ala	Pro	Ala	Arg	Ala
Ion	1	2	3	4	5	6	7	8	9	1	2	3	4	5	6	7	8	9
c	G	S*	H	P	A	P	A	R	A	75.055	818.315	955.374	1052.427	1123.464	1220.516	1291.554	1447.655	1518.692
c+1	G	S*	H	P	A	P	A	R	A	76.063	819.323	956.382	1053.434	1124.472	1221.524	1292.561	1448.663	1519.700
z	G	S*	H	P	A	P	A	R	A	73.028	229.130	300.167	397.219	468.257	565.309	702.368	1445.628	1502.649
z+1	G	S*	H	P	A	P	A	R	A	74.036	230.137	301.174	398.227	469.264	566.317	703.376	1446.636	1503.657
z+2	G	S*	H	P	A	P	A	R	A	75.044	231.145	302.182	399.235	470.272	567.325	704.384	1447.643	1504.665
	9	8	7	6	5	4	3	2	1	Ala	Arg	Ala	Pro	Ala	Pro	His	Ser	Gly

Biotools-Score: 43

Not Sure

unknown O-glycosylation site

KiSS-Receptor-1

$_{367}$GSHPAPARA$_{375}$

Fraction 16

507.15+++ → Pep [M+2H]++ 432.17++ [12.4-12.9 min] ETD

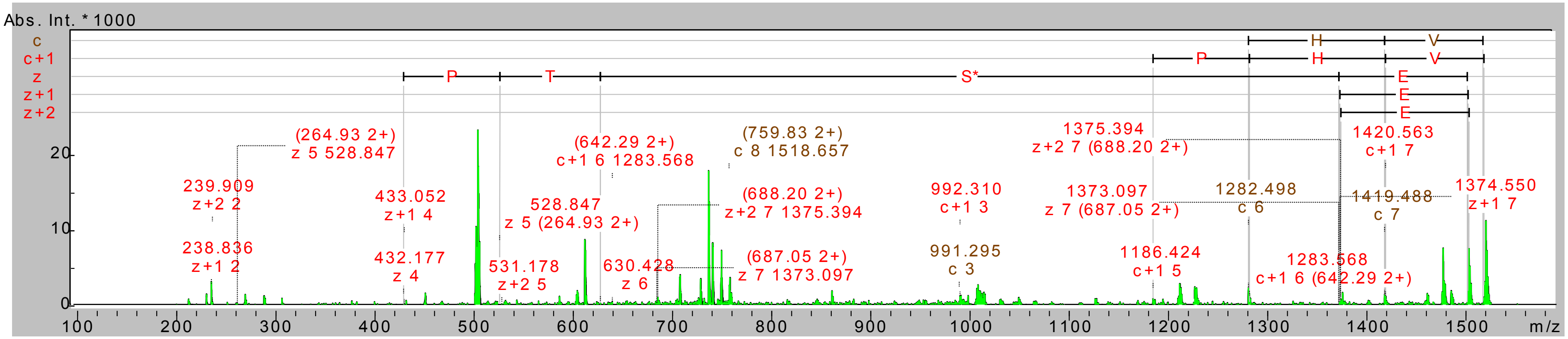

E**S**TPPPHV

	E	S	T	P	P	P	H	V	Glu	Ser	Thr	Pro	Pro	Pro	His	Val
Ion	1	2	3	4	5	6	7	8	1	2	3	4	5	6	7	8
c	E	S*	T	P	P	P	H	V	147.076	890.336	991.384	1088.437	1185.489	1282.542	1419.601	1518.669
c+1	E	S*	T	P	P	P	H	V	148.084	891.344	992.392	1089.444	1186.497	1283.550	1420.609	1519.677
z	E	S*	T	P	P	P	H	V	101.060	238.119	335.171	432.224	529.277	630.325	1373.584	1502.627
z+1	E	S*	T	P	P	P	H	V	102.068	239.126	336.179	433.232	530.285	631.332	1374.592	1503.635
z+2	E	S*	T	P	P	P	H	V	103.075	240.134	337.187	434.240	531.293	632.340	1375.600	1504.642
	8	7	6	5	4	3	2	1	Val	His	Pro	Pro	Pro	Thr	Ser	Glu

Biotools-Score: 65

More likely (Refer to subsequent glycopeptide 760.24 [M+2H]$^{2+}$)

known O-glycosylation region

Inter-alpha-trypsin inhibitor heavy chain H2 $_{689}$E**ST**PPPHV$_{696}$

Fraction 16

507.15+++ → Pep [M+2H]++ 432.17++ [12.4-12.9 min] ETD

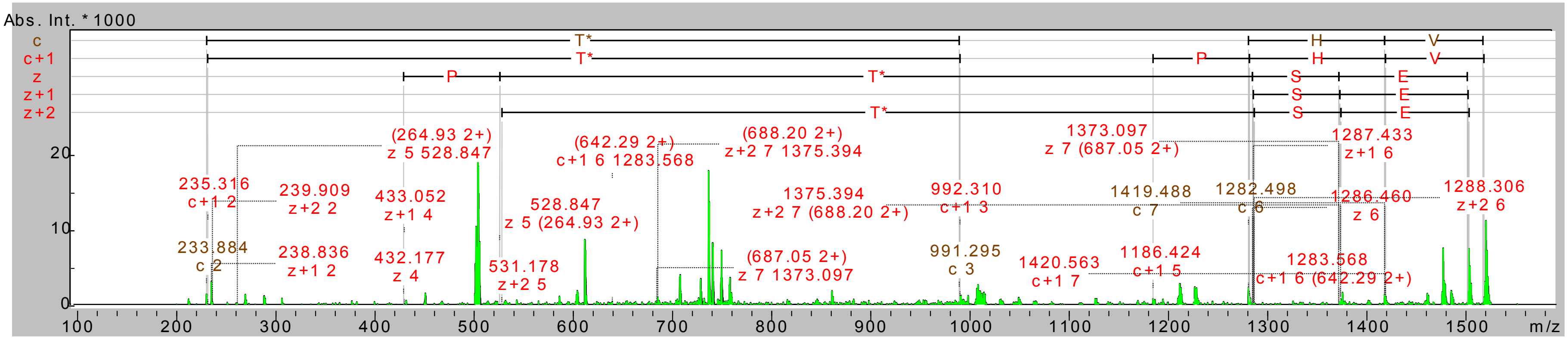

ESTPPPHV Most likely glycosylation site

	E	S	T	P	P	P	H	V	Glu	Ser	Thr	Pro	Pro	Pro	His	Val
Ion	1	2	3	4	5	6	7	8	1	2	3	4	5	6	7	8
c	E	S	T*	P	P	P	H	V	147.076	234.108	991.384	1088.437	1185.489	1282.542	1419.601	1518.669
c+1	E	S	T*	P	P	P	H	V	148.084	235.116	992.392	1089.444	1186.497	1283.550	1420.609	1519.677
z	E	S	T*	P	P	P	H	V	101.060	238.119	335.171	432.224	529.277	1286.552	1373.584	1502.627
z+1	E	S	T*	P	P	P	H	V	102.068	239.126	336.179	433.232	530.285	1287.560	1374.592	1503.635
z+2	E	S	T*	P	P	P	H	V	103.075	240.134	337.187	434.240	531.293	1288.568	1375.600	1504.642
	8	7	6	5	4	3	2	1	Val	His	Pro	Pro	Pro	Thr	Ser	Glu

Biotools-Score: 84

More likely (Refer to subsequent glycopeptide 760.24 [M+2H]$^{2+}$)

known O-glycosylation region

Inter-alpha-trypsin inhibitor heavy chain H2 $_{689}$ESTPPPHV$_{696}$

Fraction 16

760.24++ → Pep [M+H]+ 863.37+ [12.5-12.7 min] CID-MS Precursor

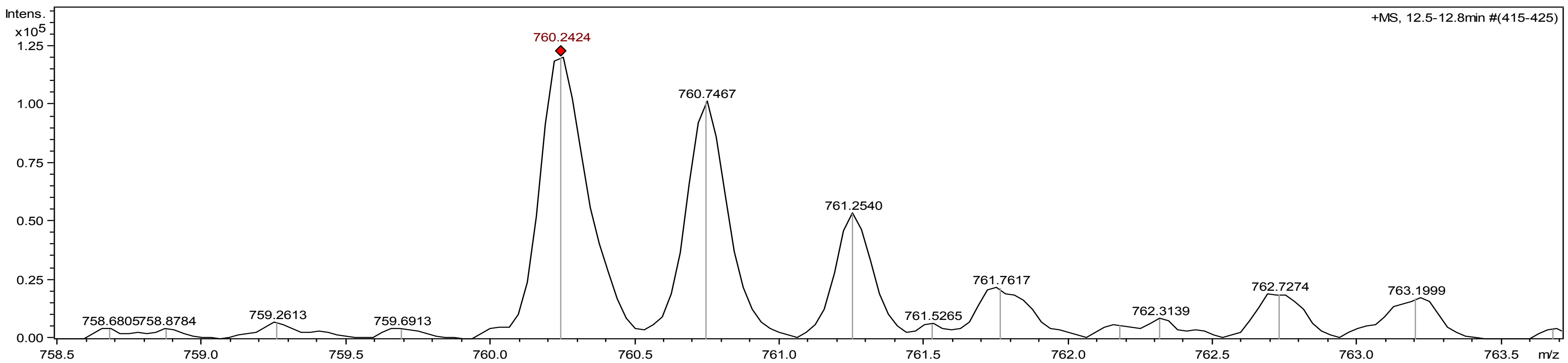

Fraction 16

760.24++ → Pep [M+H]+ 863.37+ [12.5-12.7 min] CID-MS2

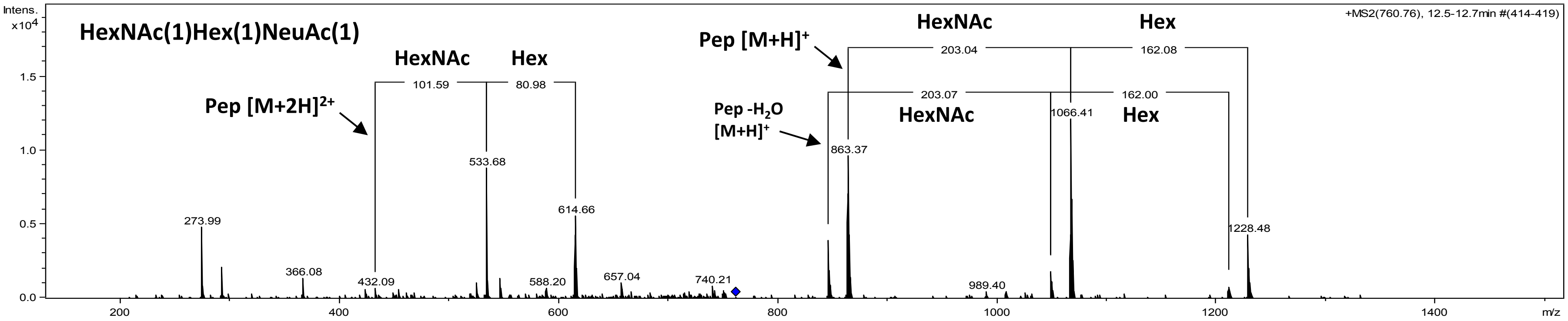

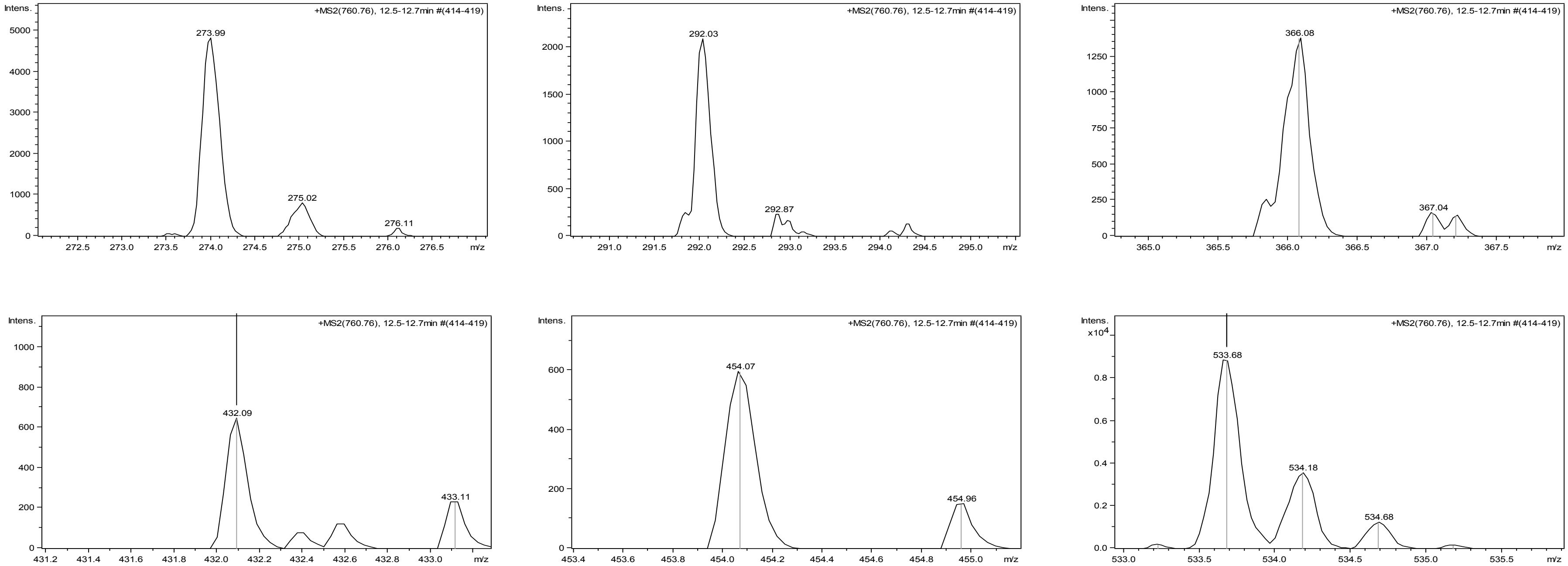

Fraction 16

760.24++ → Pep [M+H]+ 863.37+ [12.5-12.7 min]

CID-MS2

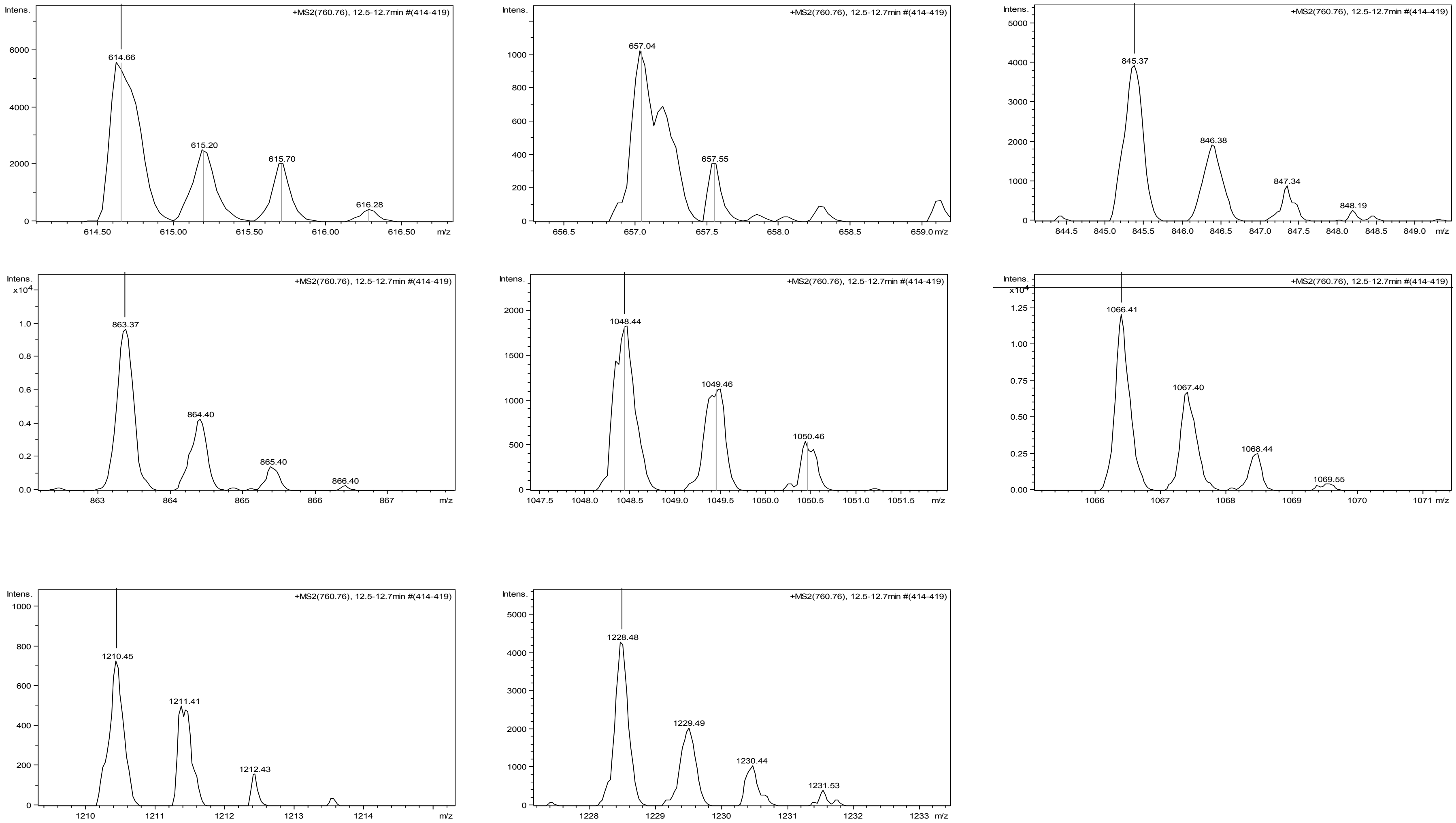

Fraction 16

760.24++ → Pep [M+H]+ 863.37+ [12.5-12.7 min] CID-MS3

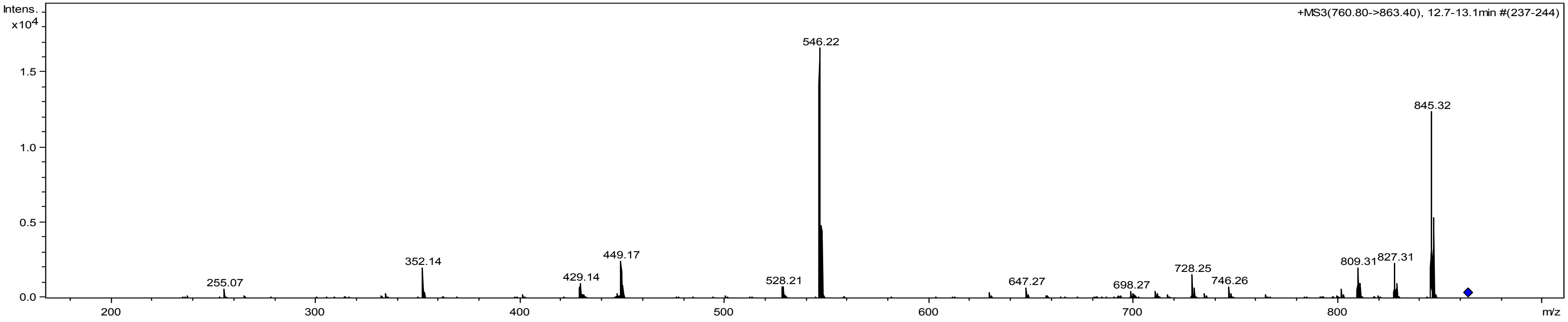

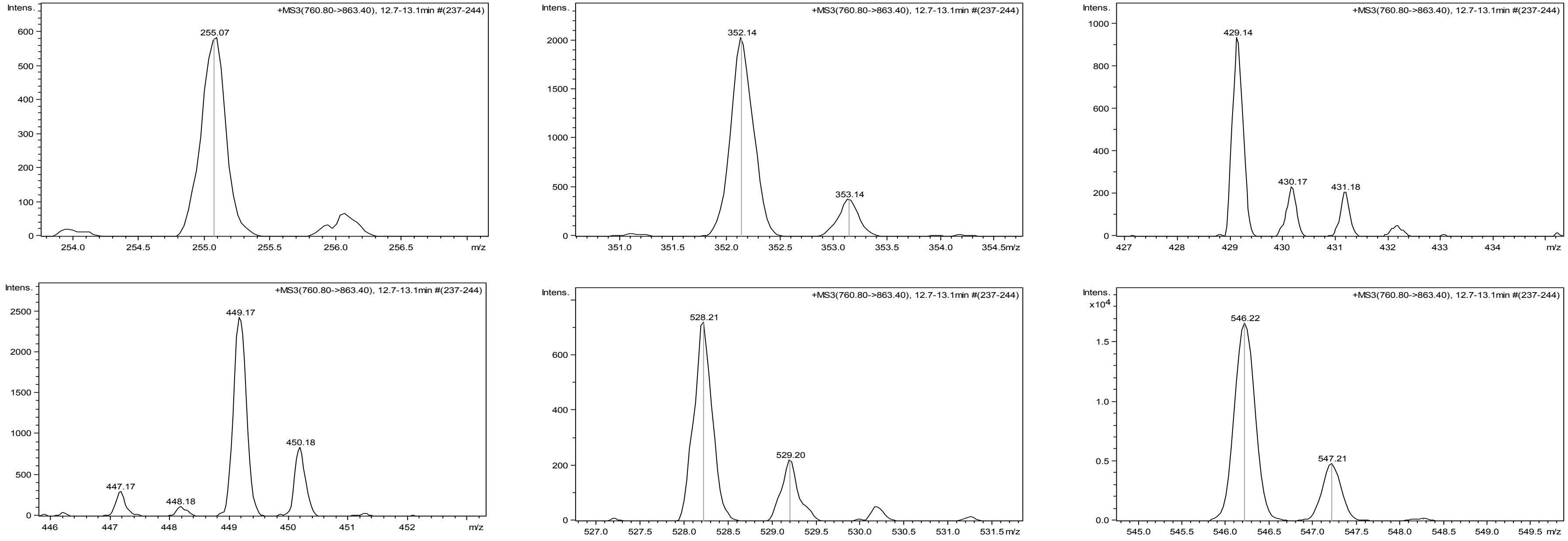

Fraction 16

760.24++ → Pep [M+H]+ 863.37+ [12.5-12.7 min]

CID-MS3

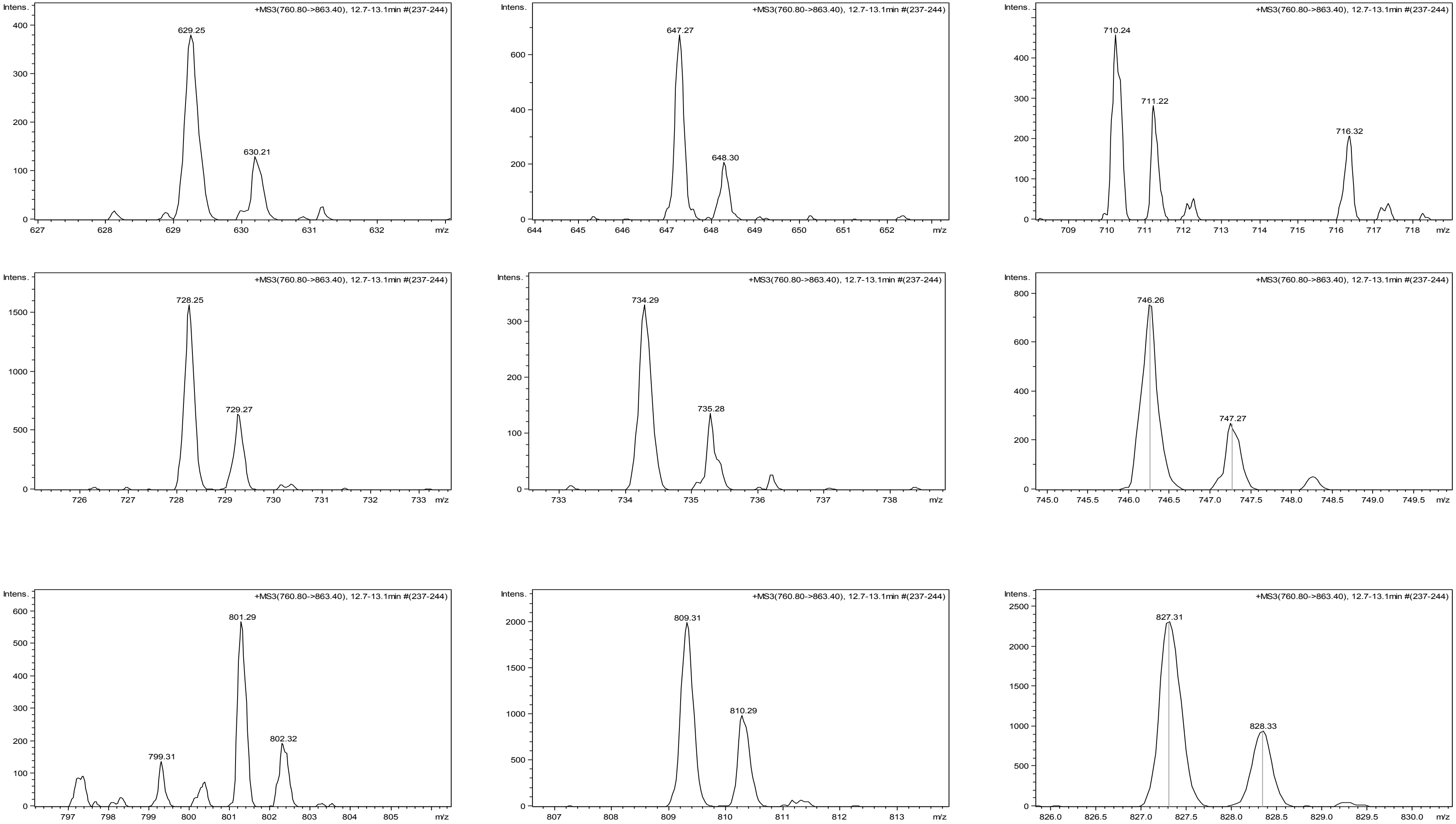

Fraction 16

760.24++ → Pep [M+H]+ 863.37+ [12.5-12.7 min] CID-MS3

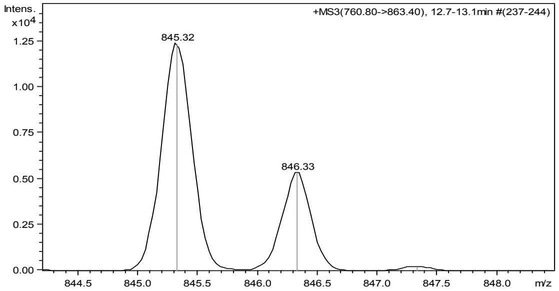

Fraction 16

760.24++ → Pep [M+H]+ 863.37+ [12.5-12.7 min]

CID-MS3 MASCOT Search

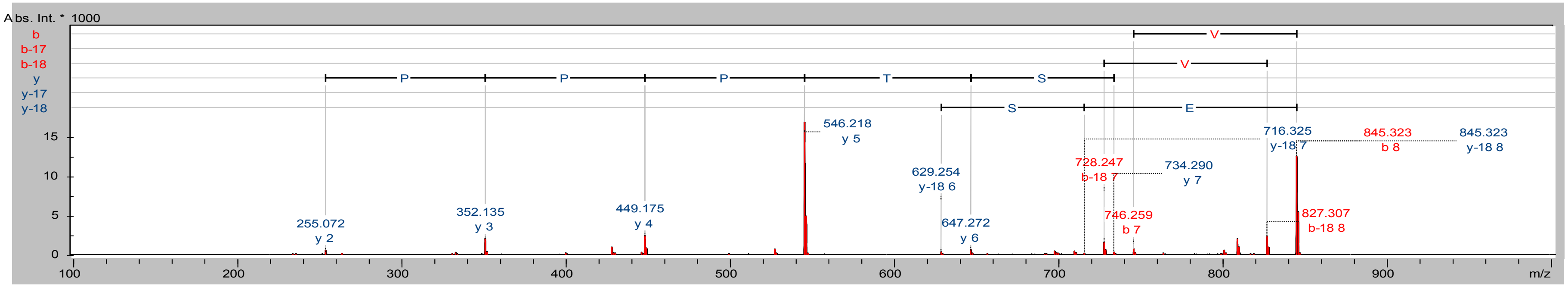

	E	S	T	P	P	P	H	V	Glu	Ser	Thr	Pro	Pro	Pro	His	Val
Ion	1	2	3	4	5	6	7	8	1	2	3	4	5	6	7	8
b	E	S	T	P	P	P	H	V	130.050	217.082	318.130	415.182	512.235	609.288	746.347	845.415
b-17	E	S	T	P	P	P	H	V	-	-	-	-	-	-	-	-
b-18	E	S	T	P	P	P	H	V	112.039	199.071	300.119	397.172	494.225	591.277	728.336	827.405
y	E	S	T	P	P	P	H	V	118.086	255.145	352.198	449.251	546.303	647.351	734.383	863.426
y-17	E	S	T	P	P	P	H	V	-	-	-	-	-	-	-	-
y-18	E	S	T	P	P	P	H	V	-	-	-	-	-	629.341	716.373	845.415
	8	7	6	5	4	3	2	1	Val	His	Pro	Pro	Pro	Thr	Ser	Glu

known O-glycosylation region

Inter-alpha-trypsin inhibitor heavy chain H2

$_{689}$**ES**TPPPHV$_{696}$

Fraction 16

760.24++ → Pep [M+H]+ 863.37+ [12.5-12.7 min]

CID-MS3 MASCOT Search

prot_hit_nur	prot_acc	prot_desc	prot_score	prot_mass	prot_matche	pep_query	pep_rank	pep_isbold	pep_exp_mz	pep_exp_mr	pep_exp_z	pep_calc_mr	pep_delta	pep_miss	pep_score	pep_expect	pep_res_bef	pep_seq
1	ITIH2_HUMA	Inter-alpha-t	26	106826	1	1	1	1	863.368	862.3607	1	862.4185	-0.0578	0	32	9.7	L	ESTPPPHV
2	NXT2_HUMA	NTF2-relatec	18	16389	1	1	4	0	863.368	862.3607	1	862.3556	0.0051	0	21.55	1.10E+02	A	QSTPNNTV
3	FLRT2_HUM/	Leucine-rich	17	74857	1	1	2	0	863.368	862.3607	1	862.4073	-0.0465	0	23.37	71	R	SYTPPTPT
4	GP101_HUM	Probable G-p	17	57478	1	1	3	0	863.368	862.3607	1	862.4072	-0.0465	0	21.74	1.00E+02	L	QSTPPLYG
5	PNMA1_HUN	Paraneoplas	15	39907	1	1	10	0	863.368	862.3607	1	862.3709	-0.0101	0	21.18	1.20E+02	L	GFQNPTPT
6	RNF12_HUM	RING finger p	15	68829	1	1	4	0	863.368	862.3607	1	862.3556	0.0051	0	21.55	1.10E+02	R	SQTPNNTV
7	TROP_HUMA	Trophinin (M	15	139803	1	1	4	0	863.368	862.3607	1	862.3192	0.0415	0	21.55	1.10E+02	E	SQTPNADQ
8	NPHP4_HUN	Nephrocystii	14	158754	1	1	8	0	863.368	862.3607	1	862.4185	-0.0578	0	21.28	1.10E+02	M	LGEDPPVH
9	K1024_HUM/	UPF0258 pro	14	104354	1	1	4	0	863.368	862.3607	1	862.3709	-0.0101	0	21.55	1.10E+02	S	SQTPNFPA
10	EP15_HUMAI	Epidermal gr	14	99222	1	1	8	0	863.368	862.3607	1	861.4709	0.8899	0	21.28	1.10E+02	I	KGIDPPHV

Biotools-Score: 65

MASCOT-Score: 32

known O-glycosylation region

Inter-alpha-trypsin inhibitor heavy chain H2

$_{689}$E**ST**PPPHV$_{696}$

Fraction 16

760.24++ → Pep [M+H]+ 863.37+ [12.5-12.7 min] ETD

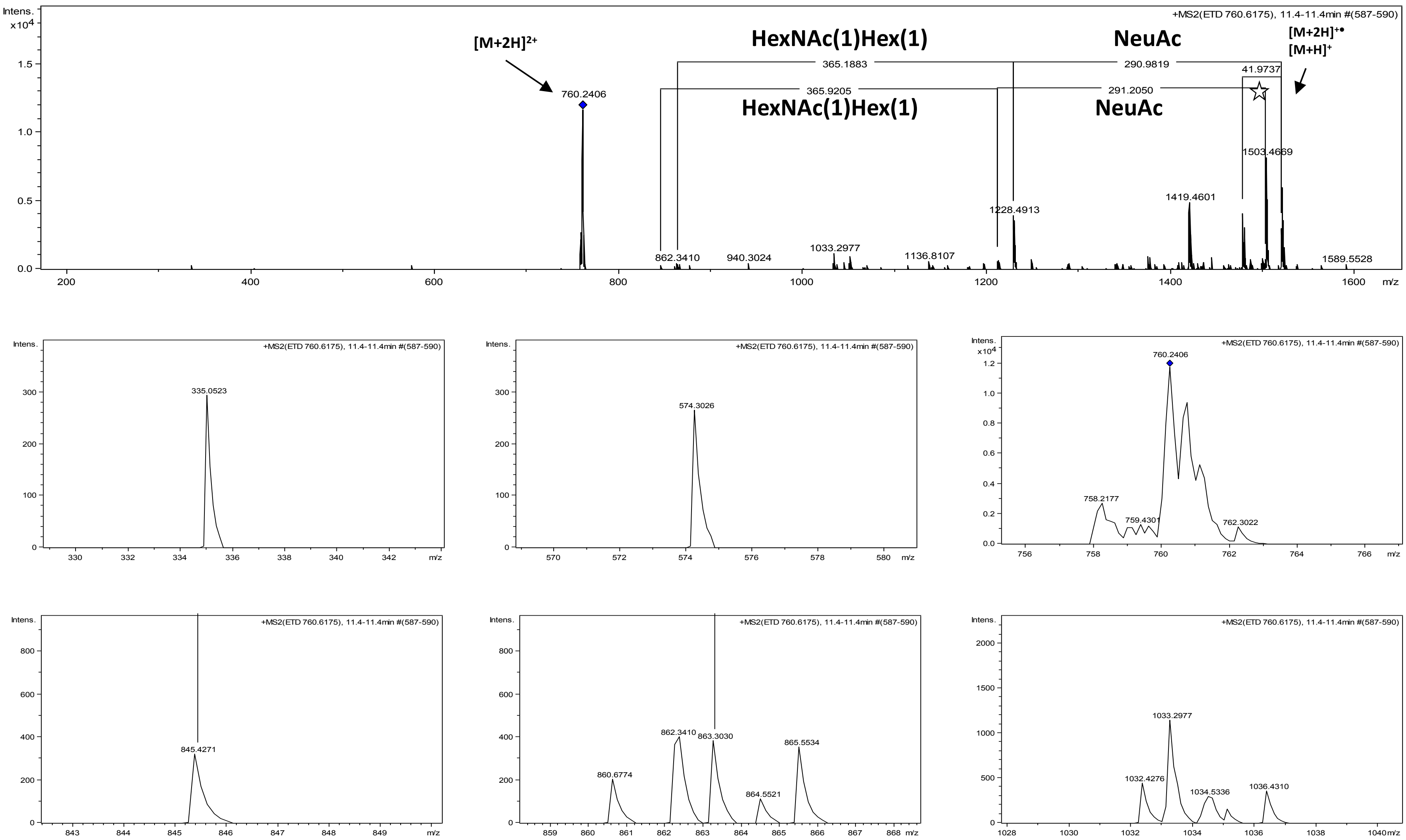

Fraction 16

760.24++ → Pep [M+H]+ 863.37+ [12.5-12.7 min]

ETD

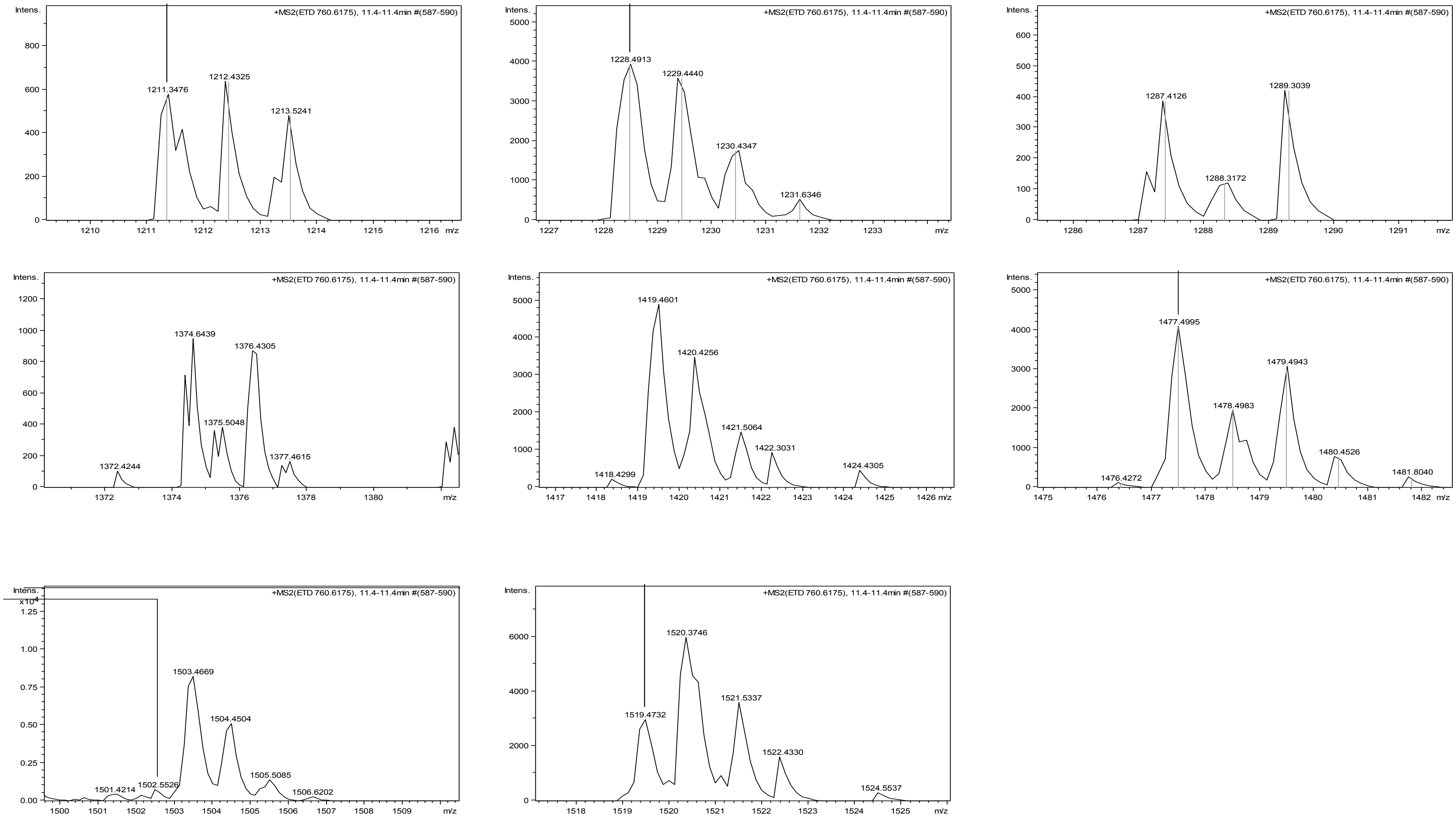

Fraction 16

760.24++ → Pep [M+H]+ 863.37+ [12.5-12.7 min] ETD

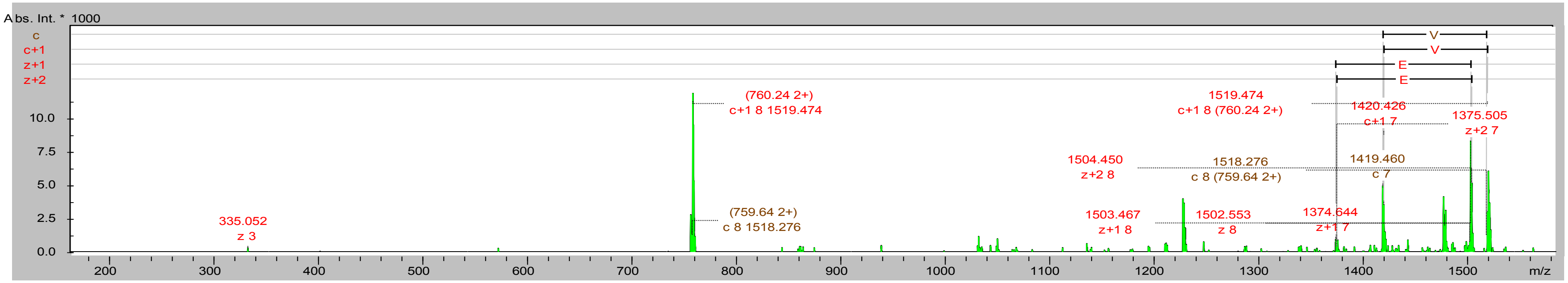

ESTPPPHV

	E	S	T	P	P	P	H	V	Glu	Ser	Thr	Pro	Pro	Pro	His	Val
Ion	1	2	3	4	5	6	7	8	1	2	3	4	5	6	7	8
c	E	S*	T	P	P	P	H	V	147.076	890.336	991.384	1088.437	1185.489	1282.542	1419.601	1518.669
c+1	E	S*	T	P	P	P	H	V	148.084	891.344	992.392	1089.444	1186.497	1283.550	1420.609	1519.677
z	E	S*	T	P	P	P	H	V	101.060	238.119	335.171	432.224	529.277	630.325	1373.584	1502.627
z+1	E	S*	T	P	P	P	H	V	102.068	239.126	336.179	433.232	530.285	631.332	1374.592	1503.635
z+2	E	S*	T	P	P	P	H	V	103.075	240.134	337.187	434.240	531.293	632.340	1375.600	1504.642
	8	7	6	5	4	3	2	1	Val	His	Pro	Pro	Pro	Thr	Ser	Glu

Biotools-Score: 14

known O-glycosylation region

Inter-alpha-trypsin inhibitor heavy chain H2 $_{689}$ESTPPPHV$_{696}$

Fraction 16

760.24++ → Pep [M+H]+ 863.37+ [12.5-12.7 min] ETD

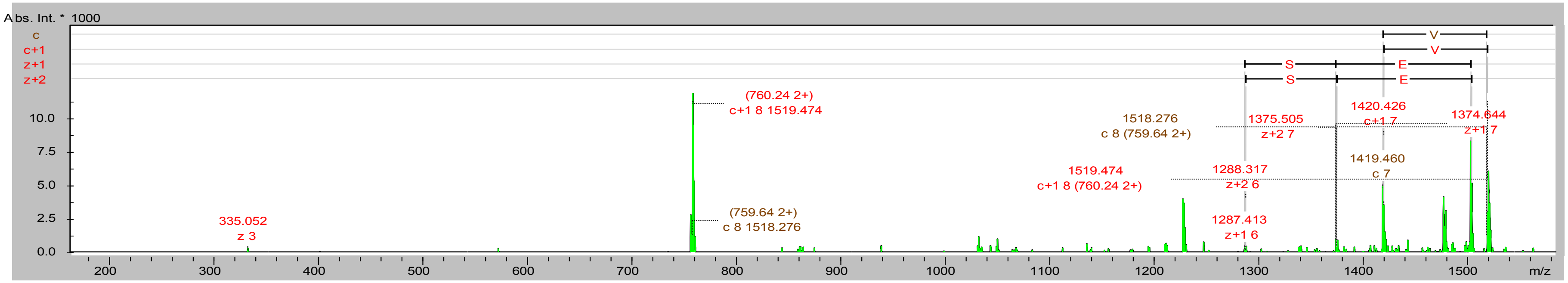

ES**T**PPPHV **Most likely**

	E	S	T	P	P	P	H	V	Glu	Ser	Thr	Pro	Pro	Pro	His	Val
Ion	1	2	3	4	5	6	7	8	1	2	3	4	5	6	7	8
c	E	S	T*	P	P	P	H	V	147.076	234.108	991.384	1088.437	1185.489	1282.542	1419.601	1518.669
c+1	E	S	T*	P	P	P	H	V	148.084	235.116	992.392	1089.444	1186.497	1283.550	1420.609	1519.677
z	E	S	T*	P	P	P	H	V	101.060	238.119	335.171	432.224	529.277	1286.552	1373.584	1502.627
z+1	E	S	T*	P	P	P	H	V	102.068	239.126	336.179	433.232	530.285	1287.560	1374.592	1503.635
z+2	E	S	T*	P	P	P	H	V	103.075	240.134	337.187	434.240	531.293	1288.568	1375.600	1504.642
	8	7	6	5	4	3	2	1	Val	His	Pro	Pro	Pro	Thr	Ser	Glu

Biotools-Score: 22

known O-glycosylation region

Inter-alpha-trypsin inhibitor heavy chain H2

Most likely **T**(691), highest ETD-Biotools-Score

$_{689}$**EST**PPPHV$_{696}$

Fraction 16

729.70++ → Pep [M+H]+ 802.34+ [12.8-12.9 min]

CID-MS Precursor

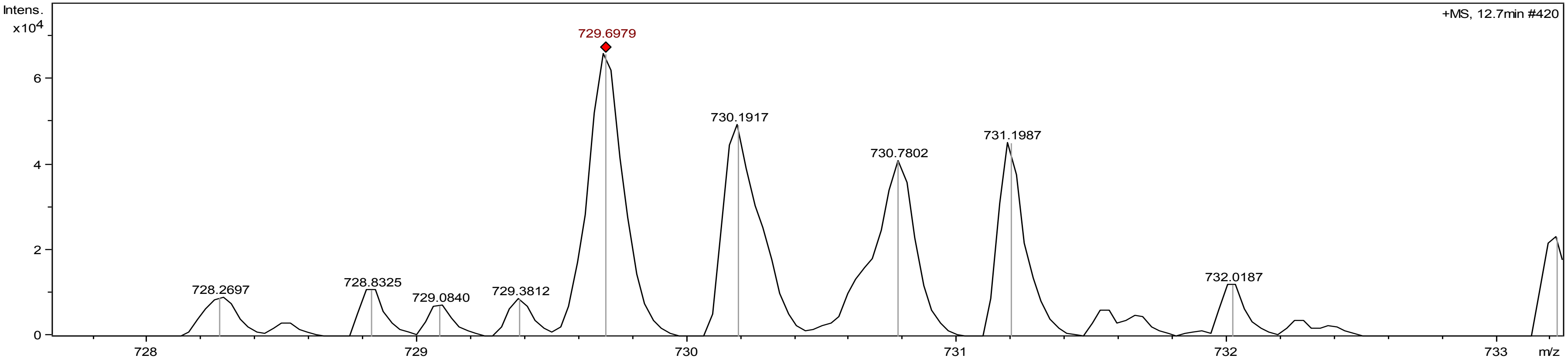

No reasonable result

Fraction 16

729.70++ → Pep [M+H]+ 802.34+ [12.8-12.9 min] CID-MS2

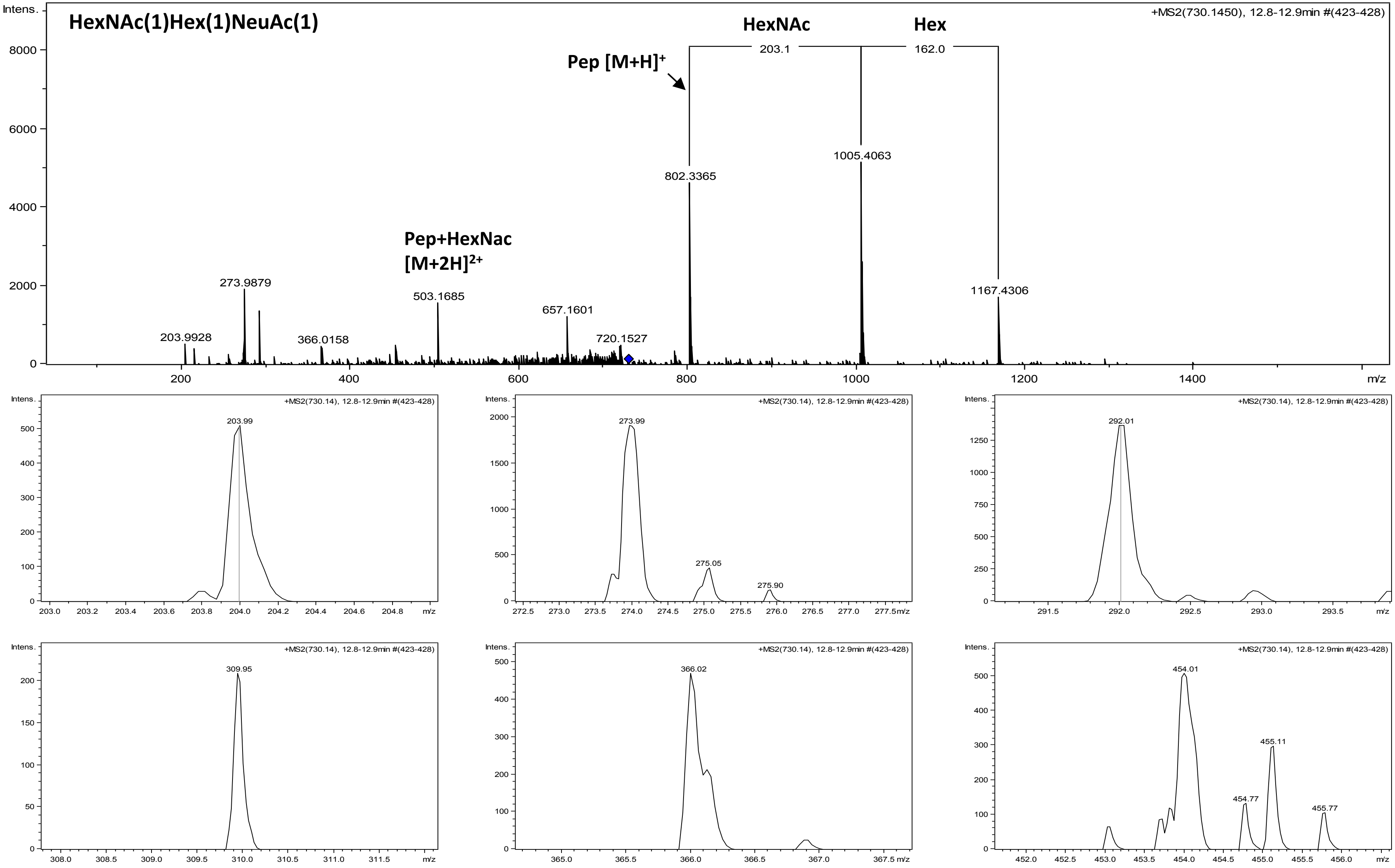

Fraction 16

729.70++ → Pep [M+H]+ 802.34+ [12.8-12.9 min]

CID-MS2

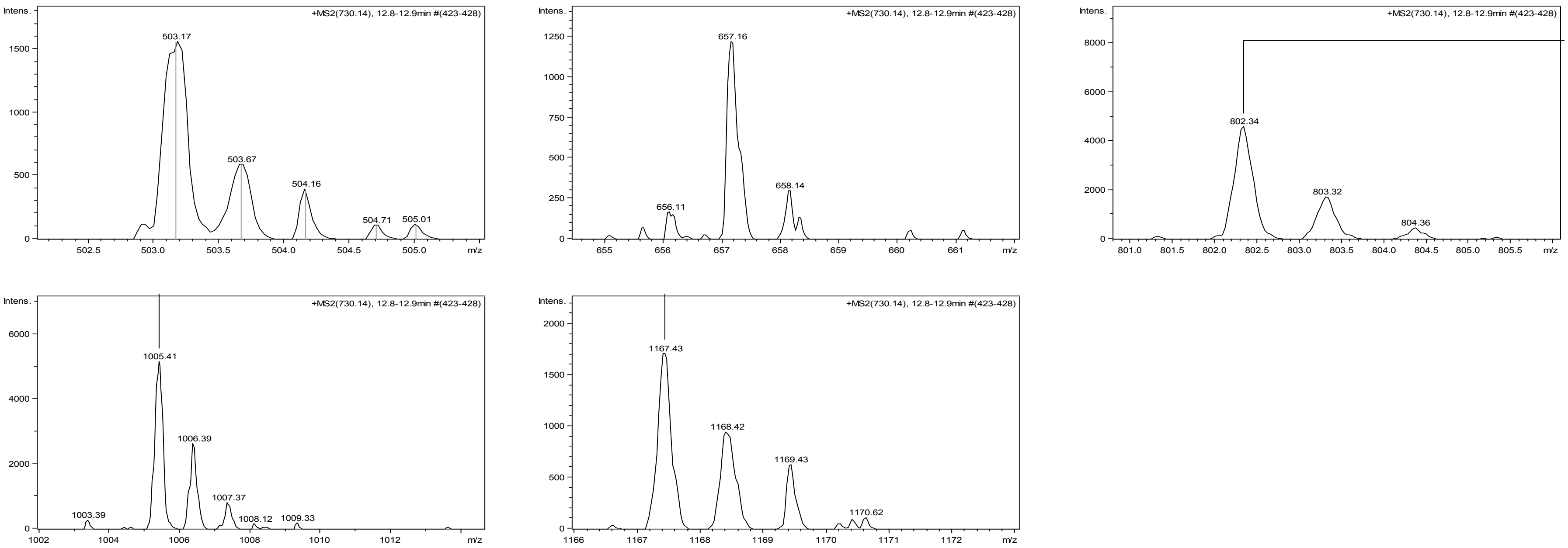

Fraction 16

729.70++ → Pep [M+H]+ 802.34+ [12.8-12.9 min] CID-MS3

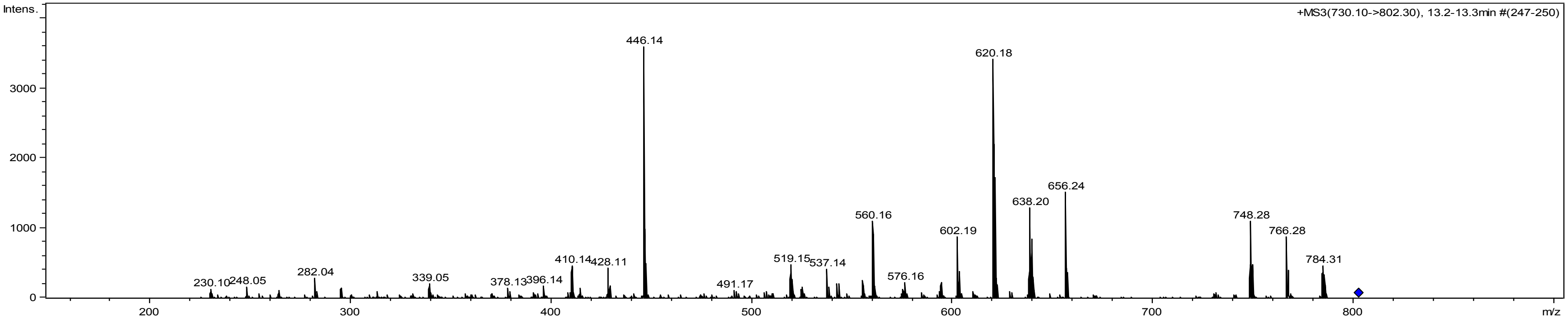

Fraction 16

729.70++ → Pep [M+H]+ 802.34+ [12.8-12.9 min] ETD

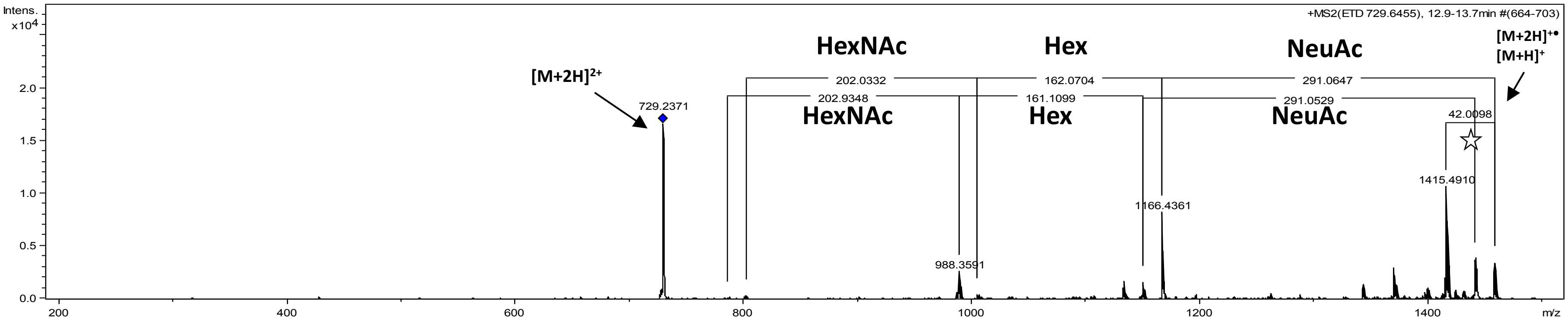

Fraction 16

781.76++ → Pep [M+H]+ 906.36+ [13.3 min]

CID-MS Precursor

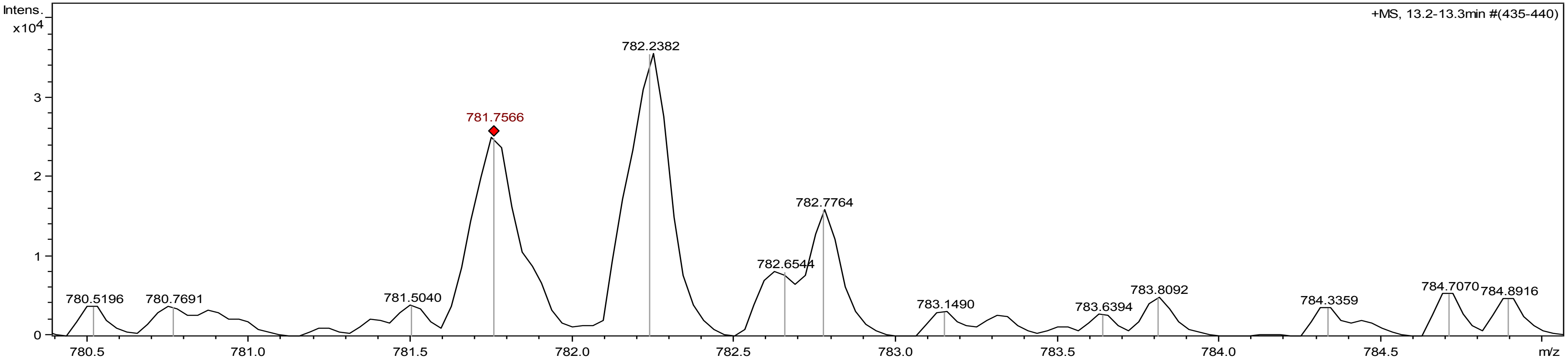

No reasonable result

Fraction 16

781.76++ → Pep [M+H]+ 906.36+ [13.3 min] CID-MS2

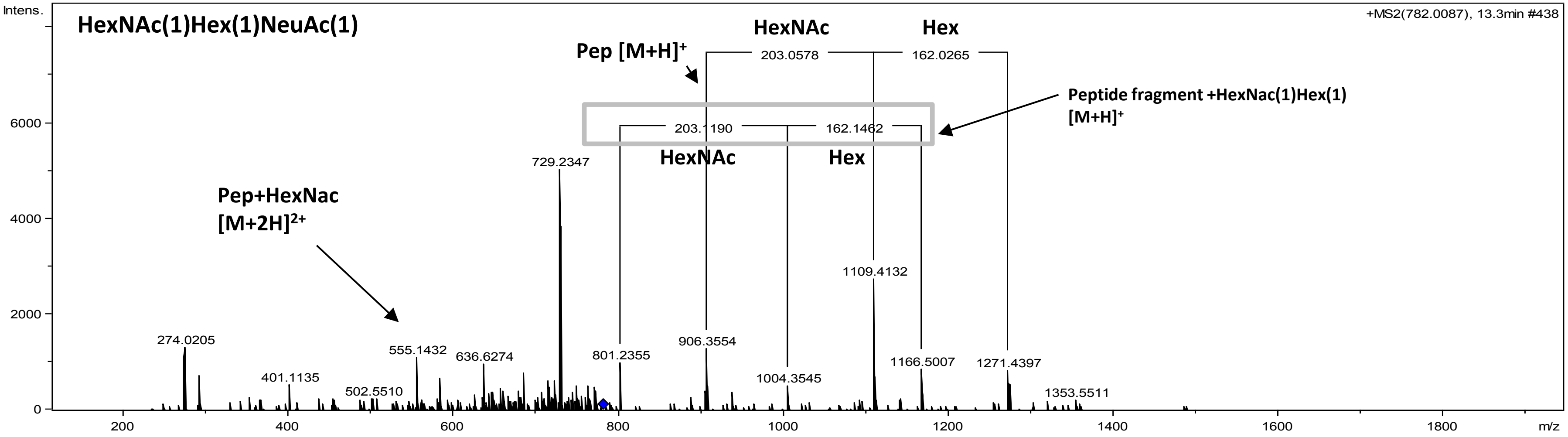

Fraction 16

781.76++ → Pep [M+H]+ 906.36+ [13.3 min]

CID-MS3 Manual DeNovo

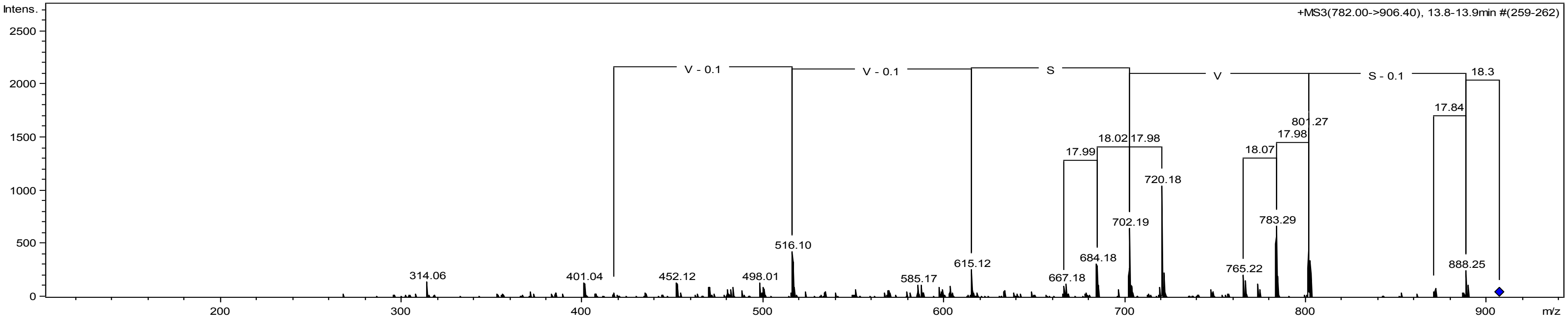

Fraction 16

781.76++ → Pep [M+H]+ 906.36+ [13.3 min]

CID-MS3 MASCOT Search

prot_hit_nur	prot_acc	prot_desc	prot_score	prot_mass	prot_matche	pep_query	pep_rank	pep_isbold	pep_exp_mz	pep_exp_mr	pep_exp_z	pep_calc_mr	pep_delta	pep_miss	pep_score	pep_expect	pep_res_bef	pep_seq
1	SPT4H_HUM/	Transcriptior	35	13470	1	1	1	1	906.3554	905.3481	1	905.3767	-0.0286	0	38.02	2.4	M	SPEDSWVS
2	ANTR2_HUM	Anthrax toxi	30	54259	1	1	2	0	906.3554	905.3481	1	905.4342	-0.0861	0	35.34	4.5	A	GETLDVSVS
3	GPX3_HUMA	Glutathione	29	25774	1	1	3	0	906.3554	905.3481	1	905.5222	-0.1741	0	32.55	8.6	L	SLLLAGFVS
4	KV111_HUM/	Ig kappa chai	26	12006	1	1	4	0	906.3554	905.3481	1	905.4342	-0.0861	0	31.76	10	T	QSPSTLSVS
5	WDR1_HUM/	WD repeat p	26	66836	1	1	5	0	906.3554	905.3481	1	905.4342	-0.0861	0	31.61	11	W	DVSVNSVVS
6	RBM14_HUM	RNA-binding	24	69620	1	1	6	0	906.3554	905.3481	1	905.3978	-0.0497	0	29.91	16	G	QLASPSSQS
7	DEND_HUMA	Dendrin - Ho	21	70212	1	1	7	0	906.3554	905.3481	1	905.3978	-0.0497	0	26.87	32	T	AELSDSVGE
8	APBA2_HUM	Amyloid bet	21	83315	1	1	7	0	906.3554	905.3481	1	905.3978	-0.0497	0	26.87	32	V	QALSNSVGE
9	MINT_HUMA	Msx2-interac	17	403030	1	1	9	0	906.3554	905.3481	1	905.4495	-0.1013	0	26.41	35	T	SFPSPVSVS
10	UBP35_HUM	Ubiquitin car	17	114987	1	1	10	0	906.3554	905.3481	1	905.4429	-0.0948	0	24.22	58	S	RMIDWVS

No reasonable result

Fraction 16

729.25++ → Pep [M+H]+ 801.34+ [13.7-14.1 min] CID-MS Precursor

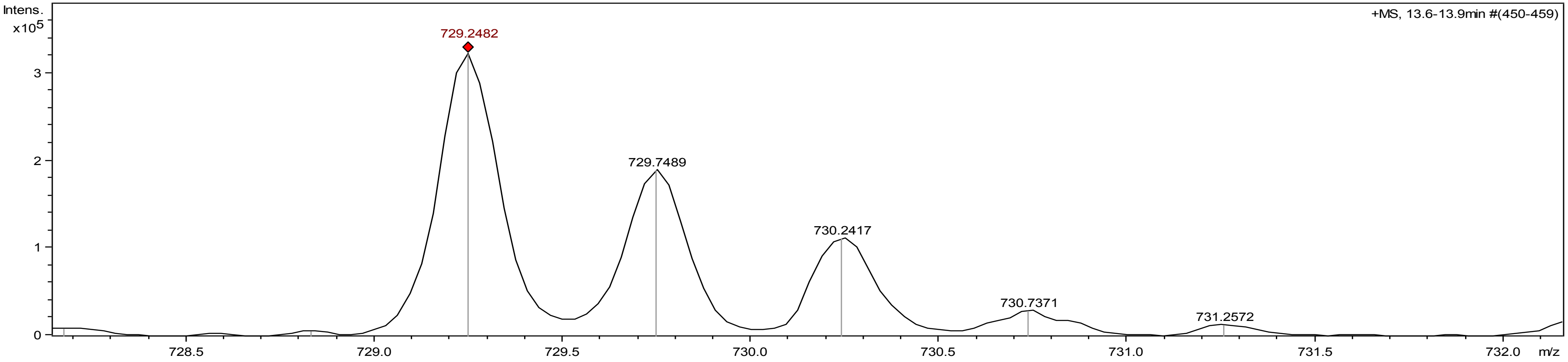

Fraction 16

729.25++ → Pep [M+H]+ 801.34+ [13.7-14.1 min] CID-MS2

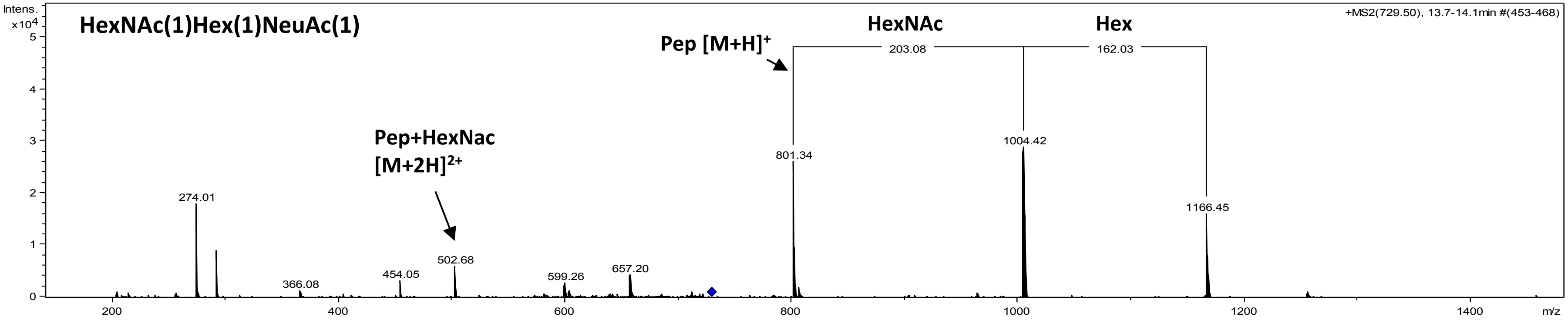

Fraction 16

729.25++ → Pep [M+H]+ 801.34+ [13.7-14.1 min]

CID-MS3

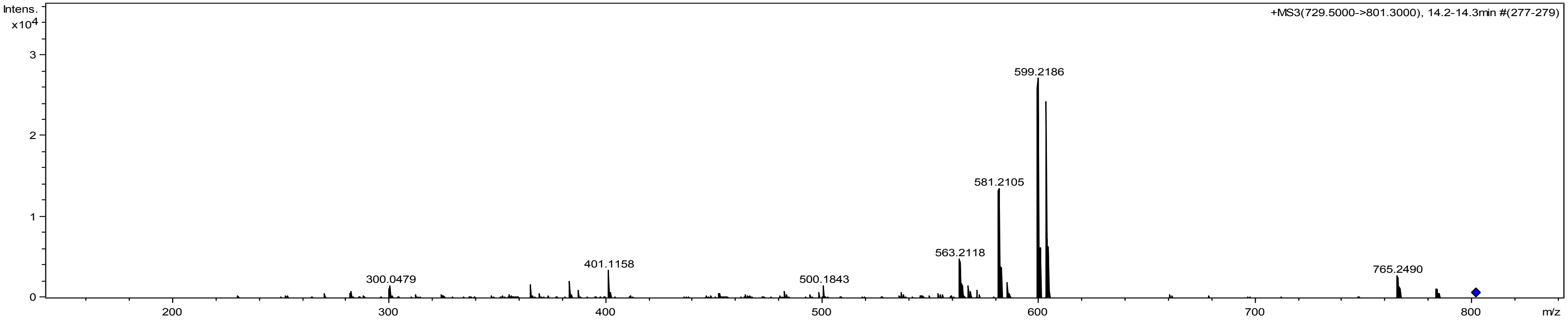

No reasonable result

Fraction 16

788.61+++ → Pep [M+2H]++ 854.33++ [14.6 min] CID-MS Precursor

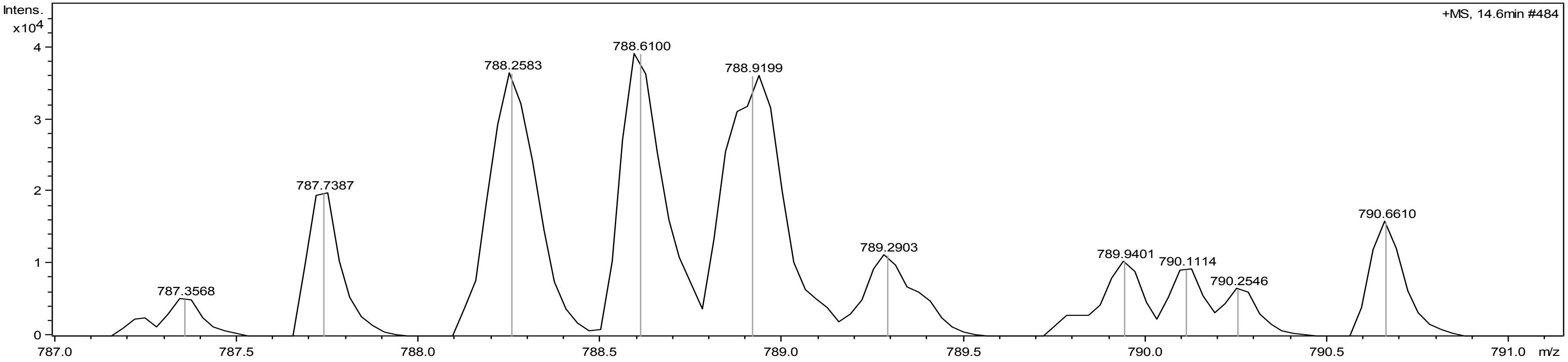

Fraction 16

788.26+++ → Pep [M+2H]++ 854.33++ [14.6 min] CID-MS2

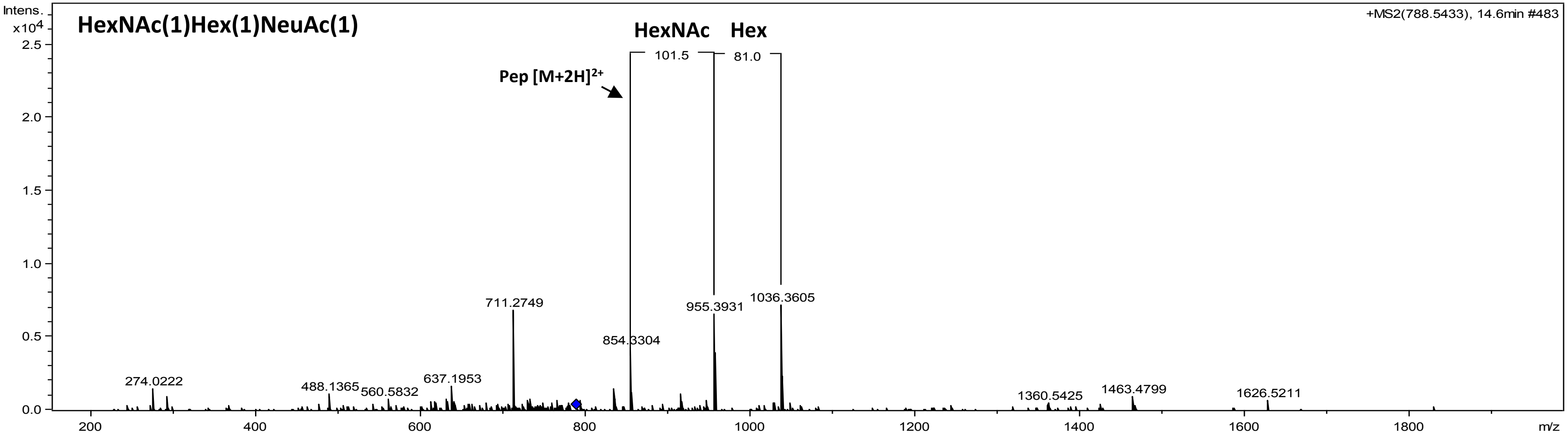

Fraction 16

788.26+++ → Pep [M+2H]++ 854.33++ [14.6 min]

CID-MS3

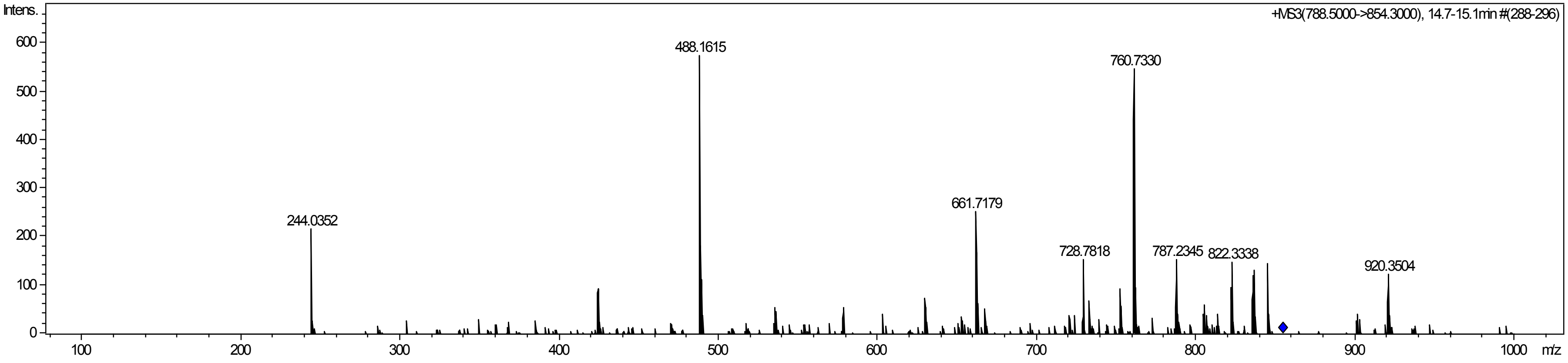

No reasonable result

Fraction 16

728.75++ → Pep [M+H]+ 800.36+ [15.4 min] CID-MS Precursor

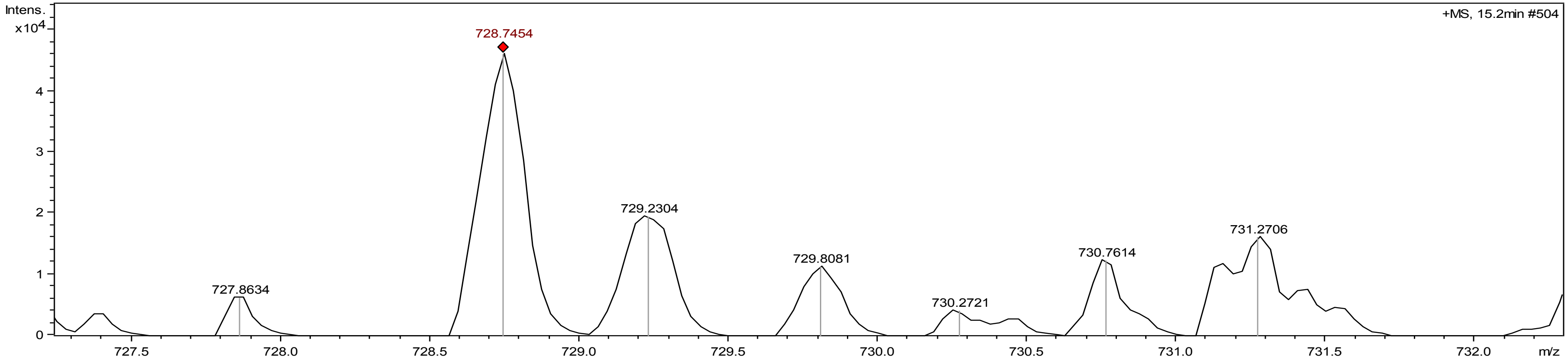

ETD spectrum not available

Fraction 16

728.75++ → Pep [M+H]+ 800.36+ [15.4 min] CID-MS2

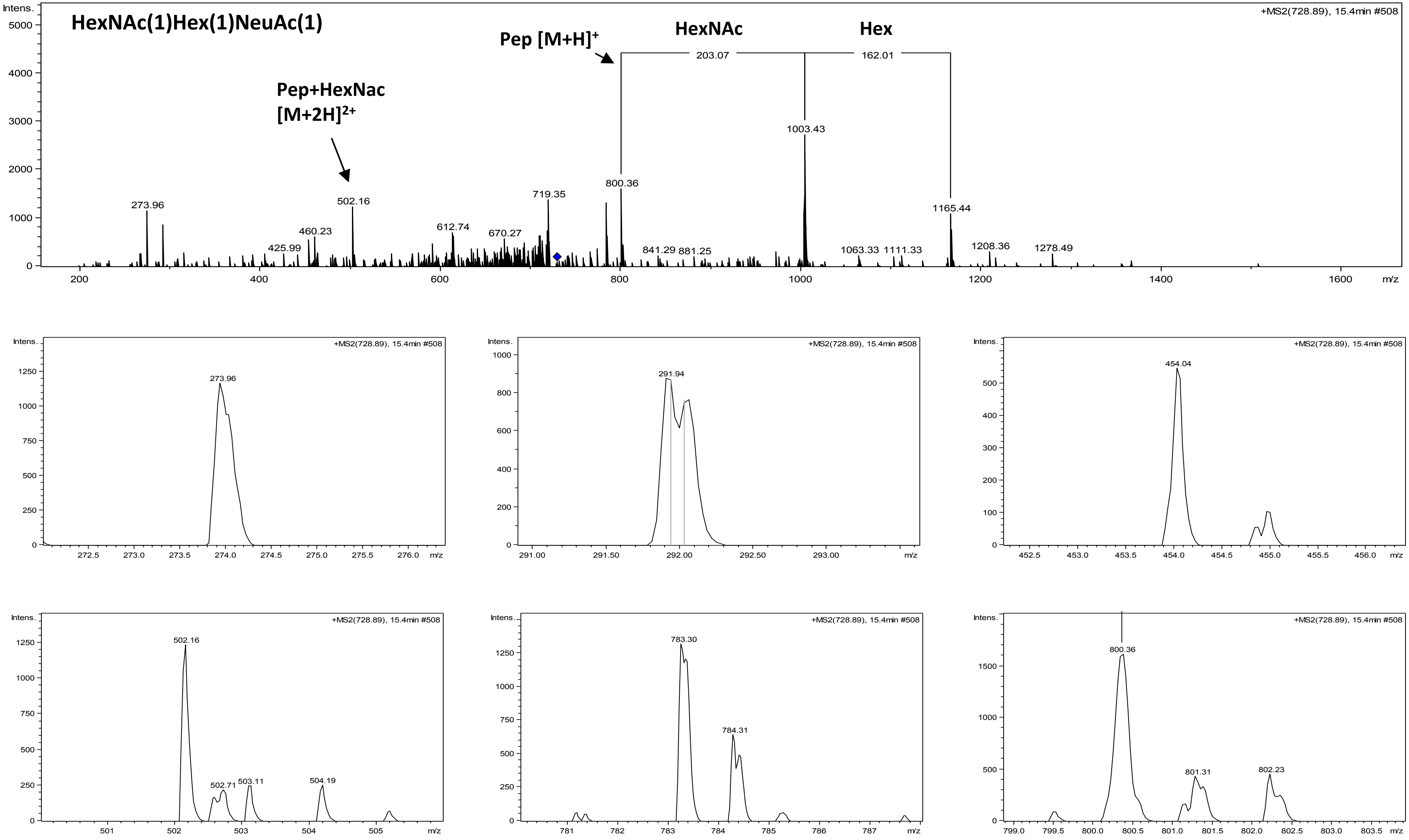

Fraction 16

728.75++ → Pep [M+H]+ 800.36+ [15.4 min] CID-MS2

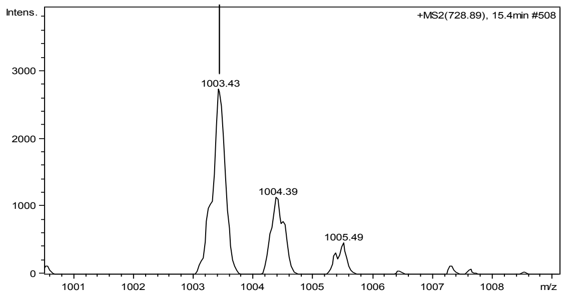

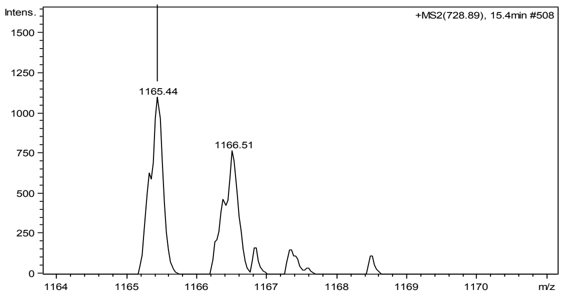

Fraction 16

728.75++ → Pep [M+H]+ 800.36+ [15.4 min] CID-MS3

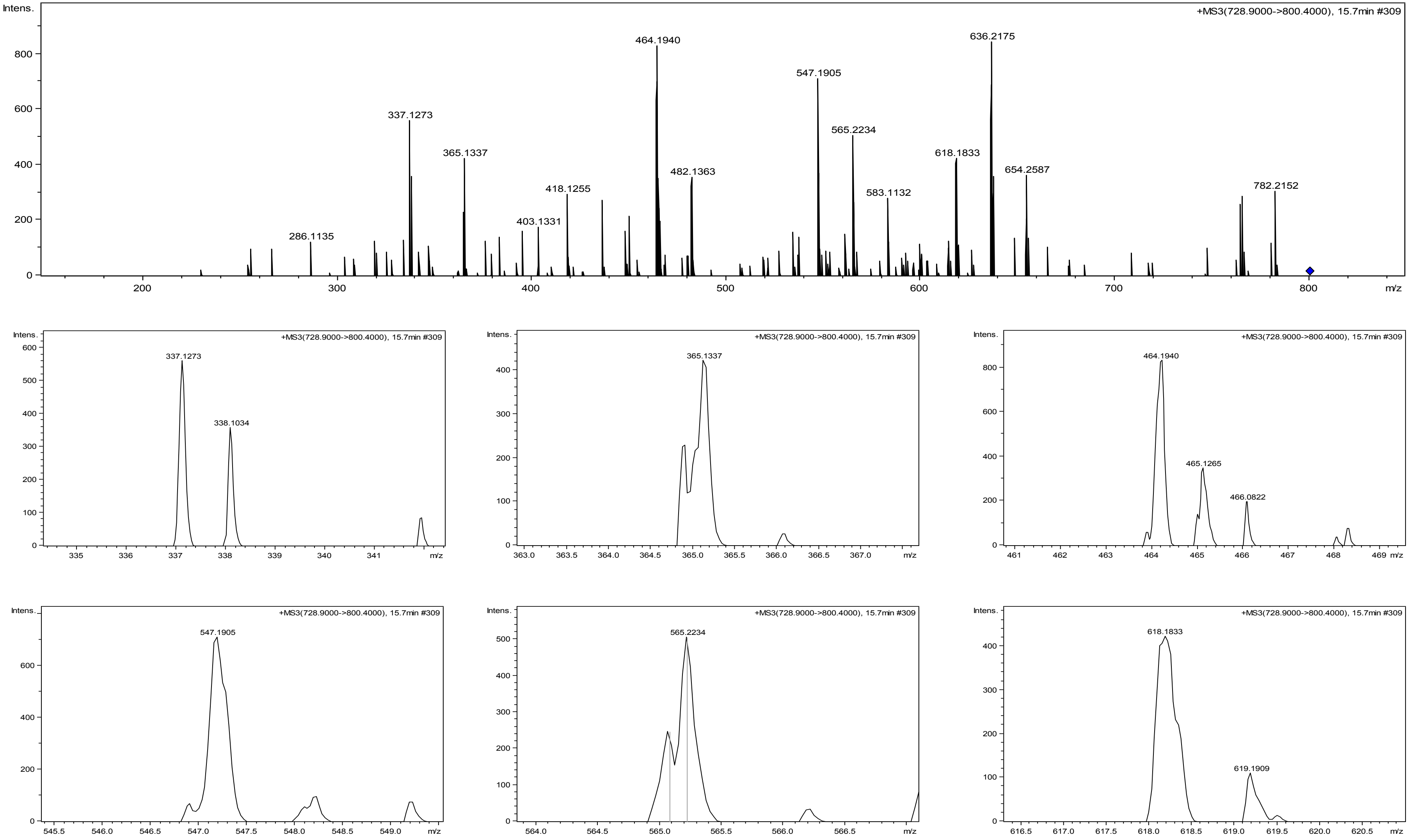

Fraction 16

728.75++ → Pep [M+H]+ 800.36+ [15.4 min]

CID-MS3

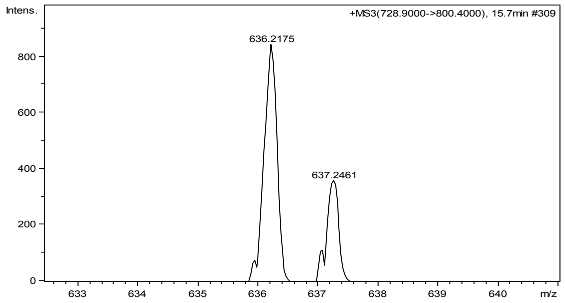

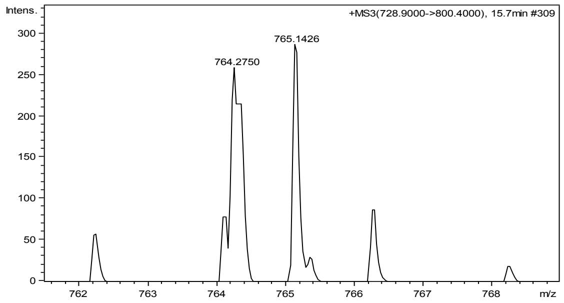

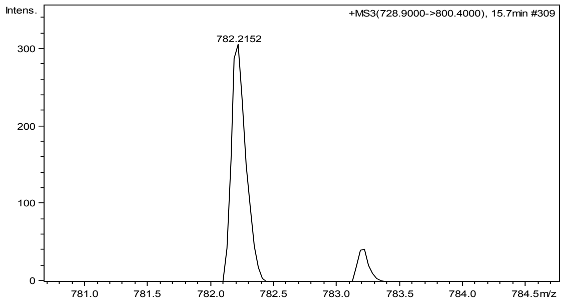

Fraction 16

728.75++ → Pep [M+H]+ 800.36+ [15.4 min] CID-MS3 MASCOT Search

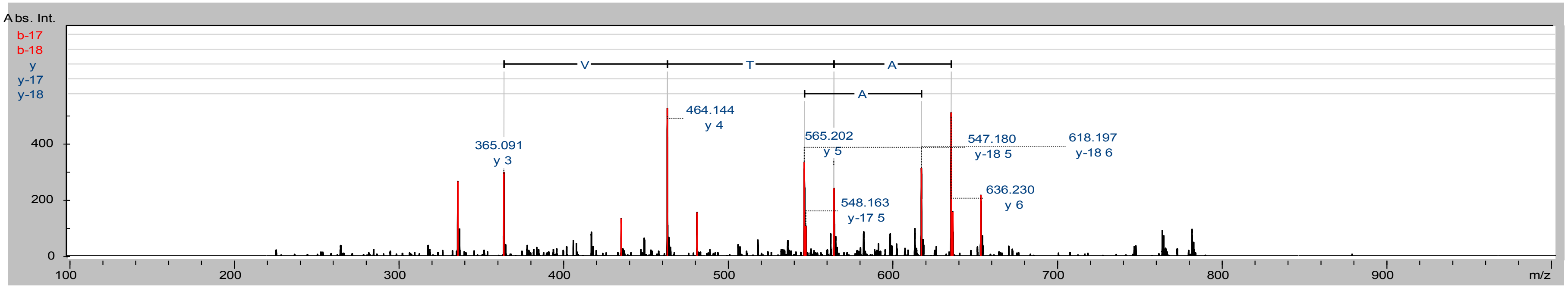

	Y	A	T	V	K	S	M	Tyr	Ala	Thr	Val	Lys	Ser	Met
Ion	1	2	3	4	5	6	7	1	2	3	4	5	6	7
b	Y	A	T	V	K	S	M	164.071	235.108	336.155	435.224	563.319	650.351	781.391
b-17	Y	A	T	V	K	S	M	-	-	-	-	546.292	633.324	764.365
b-18	Y	A	T	V	K	S	M	-	-	318.145	417.213	545.308	632.340	763.381
y	Y	A	T	V	K	S	M	150.058	237.090	365.185	464.254	565.301	636.339	799.402
y-17	Y	A	T	V	K	S	M	-	-	348.159	447.227	548.275	619.312	782.375
y-18	Y	A	T	V	K	S	M	-	219.080	347.175	446.243	547.291	618.328	781.391
	7	6	5	4	3	2	1	Met	Ser	Lys	Val	Thr	Ala	Tyr

Not Sure

unknown O-glycosylation region

GTP-binding protein 1

$_{117}$YA**T**VK**S**M$_{123}$

Fraction 16

728.75++ → Pep [M+H]+ 800.36+ [15.4 min]

CID-MS3 MASCOT Search

prot_hit_nur	prot_acc	prot_desc	prot_score	prot_mass	prot_matche	pep_query	pep_rank	pep_isbold	pep_exp_mz	pep_exp_mr	pep_exp_z	pep_calc_mr	pep_delta	pep_miss	pep_score	pep_expect	pep_res_bef	pep_seq
1	GTPB1_HUM	GTP-binding	23	72046	1	1	1	1	800.3614	799.3541	1	798.3946	0.9596	0	27.38	22	S	YATVKSM
2	IFRD1_HUMA	Interferon-re	16	51035	1	1	3	0	800.3614	799.3541	1	799.3864	-0.0323	0	18.86	1.60E+02	L	YNSAAFK
3	VPS72_HUM	Vacuolar pro	12	40798	1	1	8	0	800.3614	799.3541	1	798.4024	0.9517	0	15.83	3.20E+02	P	YATARAF
4	CPT1A_HUM	Carnitine O-|	12	88995	1	1	6	0	800.3614	799.3541	1	799.3977	-0.0436	0	17.45	2.20E+02	A	HQAVAFQ
5	SYNE1_HUM	Nesprin-1 (N	12	1017083	1	1	2	0	800.3614	799.3541	1	798.3396	1.0146	0	22.3	72	L	YDGVSATS
6	MYF5_HUMA	Myogenic fa	12	28990	1	1	8	0	800.3614	799.3541	1	798.3395	1.0146	0	15.83	3.20E+02	V	YATDKNS
7	SORC3_HUM	VPS10 doma	12	136582	1	1	4	0	800.3614	799.3541	1	798.4058	0.9483	0	17.75	2.00E+02	L	VAMKHTP
8	FHR3_HUMA	Complement	12	38496	1	1	8	0	800.3614	799.3541	1	798.3032	1.051	0	15.83	3.20E+02	G	YATADGNS
9	SYN3_HUMA	Synapsin-3 (!	12	63491	1	1	4	0	800.3614	799.3541	1	798.3946	0.9596	0	17.75	2.00E+02	V	VAMAKTY
10	SAPS1_HUM	SAPS domair	10	97291	1	1	7	0	800.3614	799.3541	1	799.3283	0.0258	0	17.15	2.30E+02	L	HAQVEGC

Biotools-Score: 9

MASCOT-Score: 27

Not Sure

unknown O-glycosylation region

GTP-binding protein 1

$_{117}$YA**T**VK**S**M$_{123}$

Fraction 16

765.20++ → Pep [M+H]+ 582.17+ [16.5-16.6 min] CID-MS Precursor

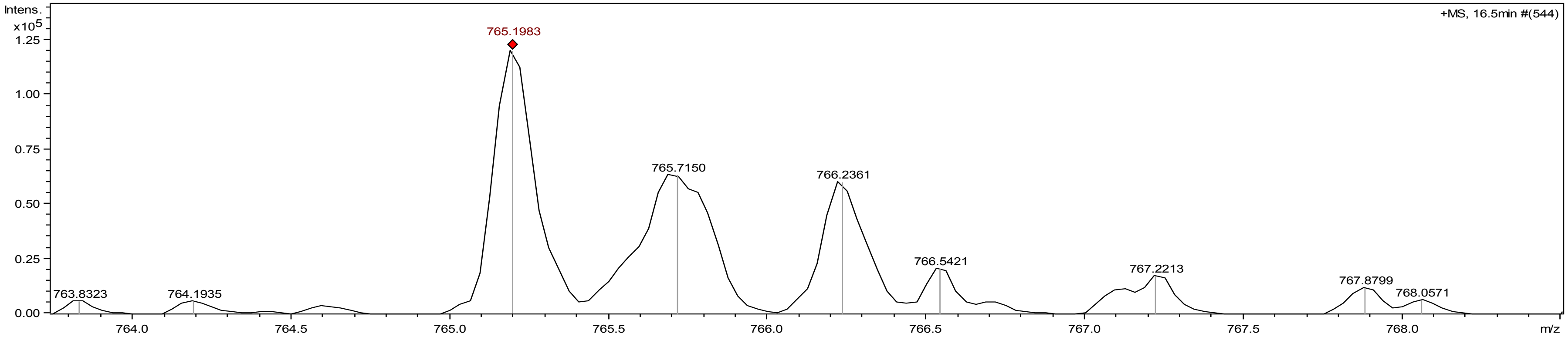

CID-MS3 spectrum of poor quality

ETD spectrum not available

Fraction 16

765.20++ → Pep [M+H]+ 582.17+ [16.5-16.6 min] CID-MS2

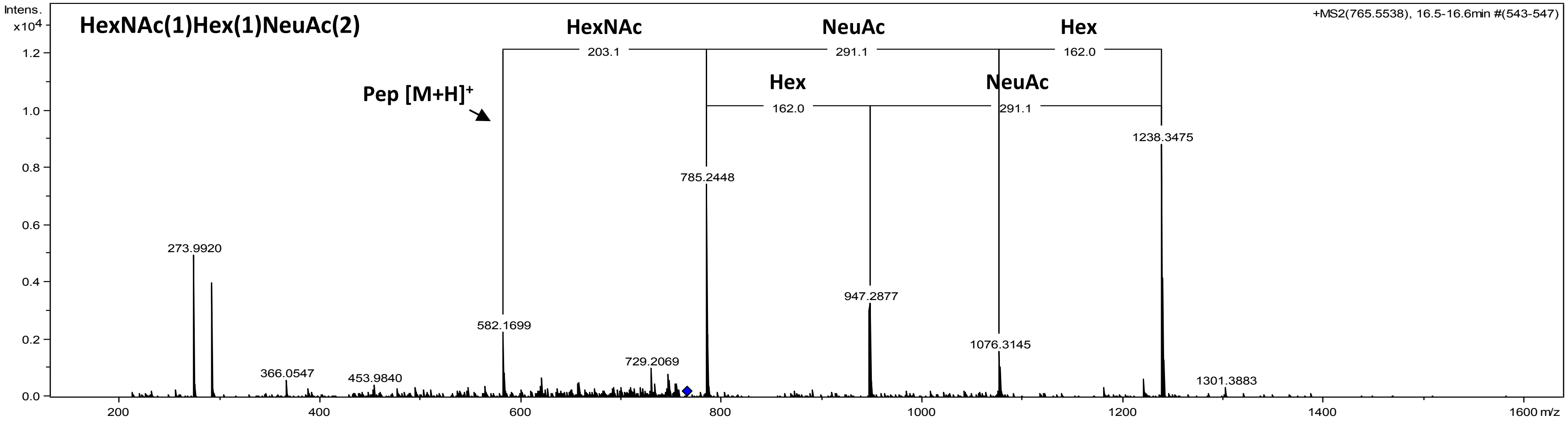

Fraction 16

734.23++ → Pep[M+H]+ 520.19+ [17.2 min]

CID-MS Precursor

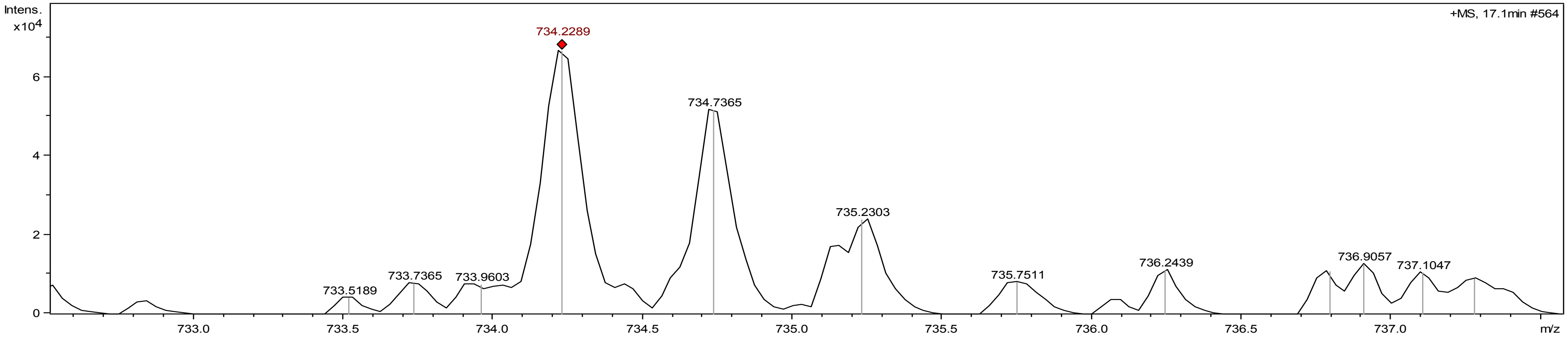

ETD spectrum of poor quality

CID-MS3 spectrum of poor quality

Fraction 16

734.23++ → Pep[M+H]+ 520.19+ [17.2 min] CID-MS2

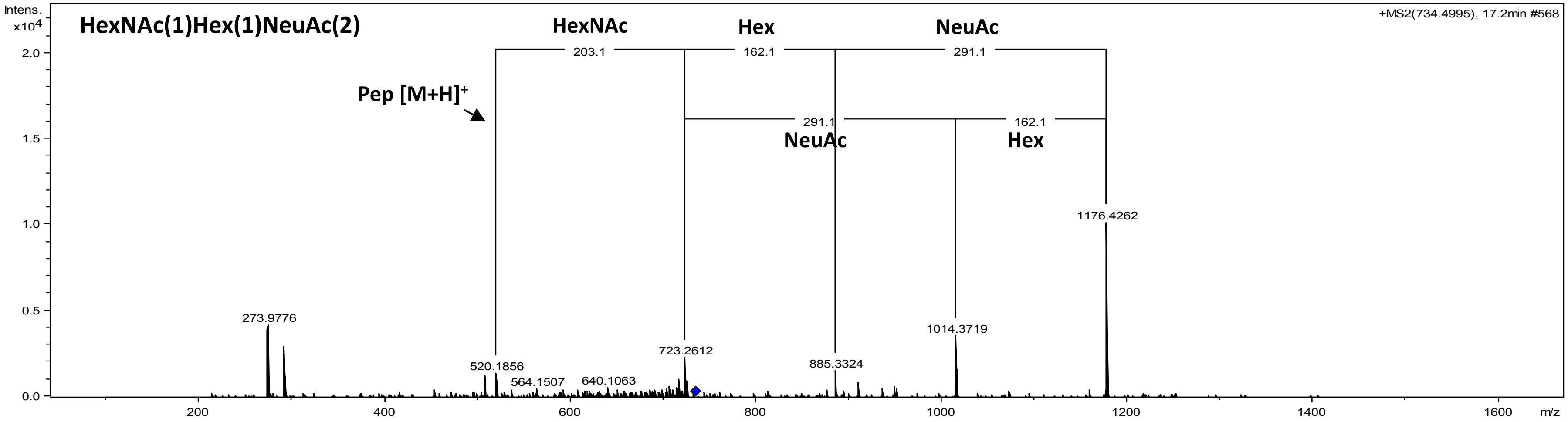

ETD spectrum of poor quality

CID spectrum of poor quality

Fraction 16

782.97+++ → Pep[M+2H]++ 845.80++ [17.8-18.1 min] CID-MS Precursor

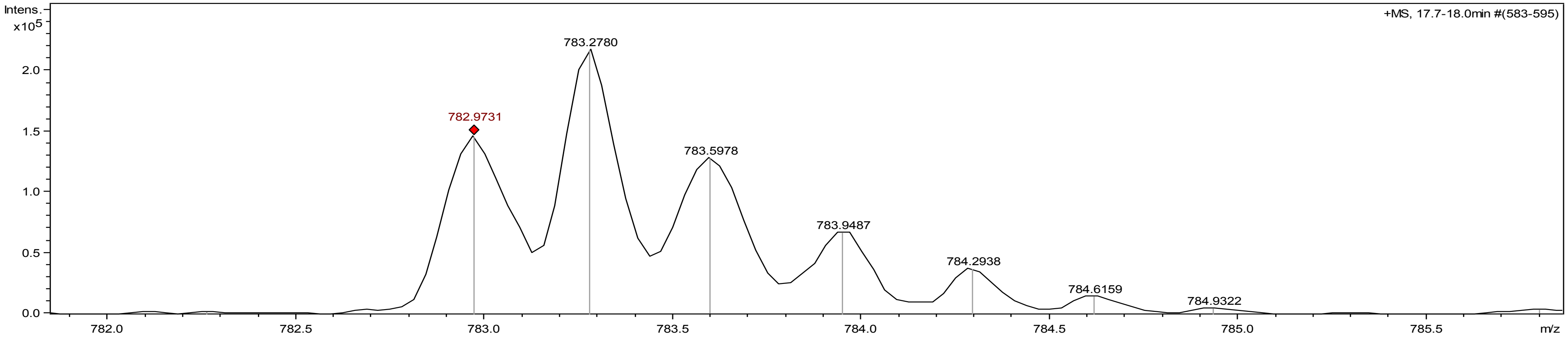

Many b- and y-ions in CID MS² spectrum

Fraction 16

782.97+++ → Pep[M+2H]++ 845.80++ [17.8-18.1 min] CID-MS2

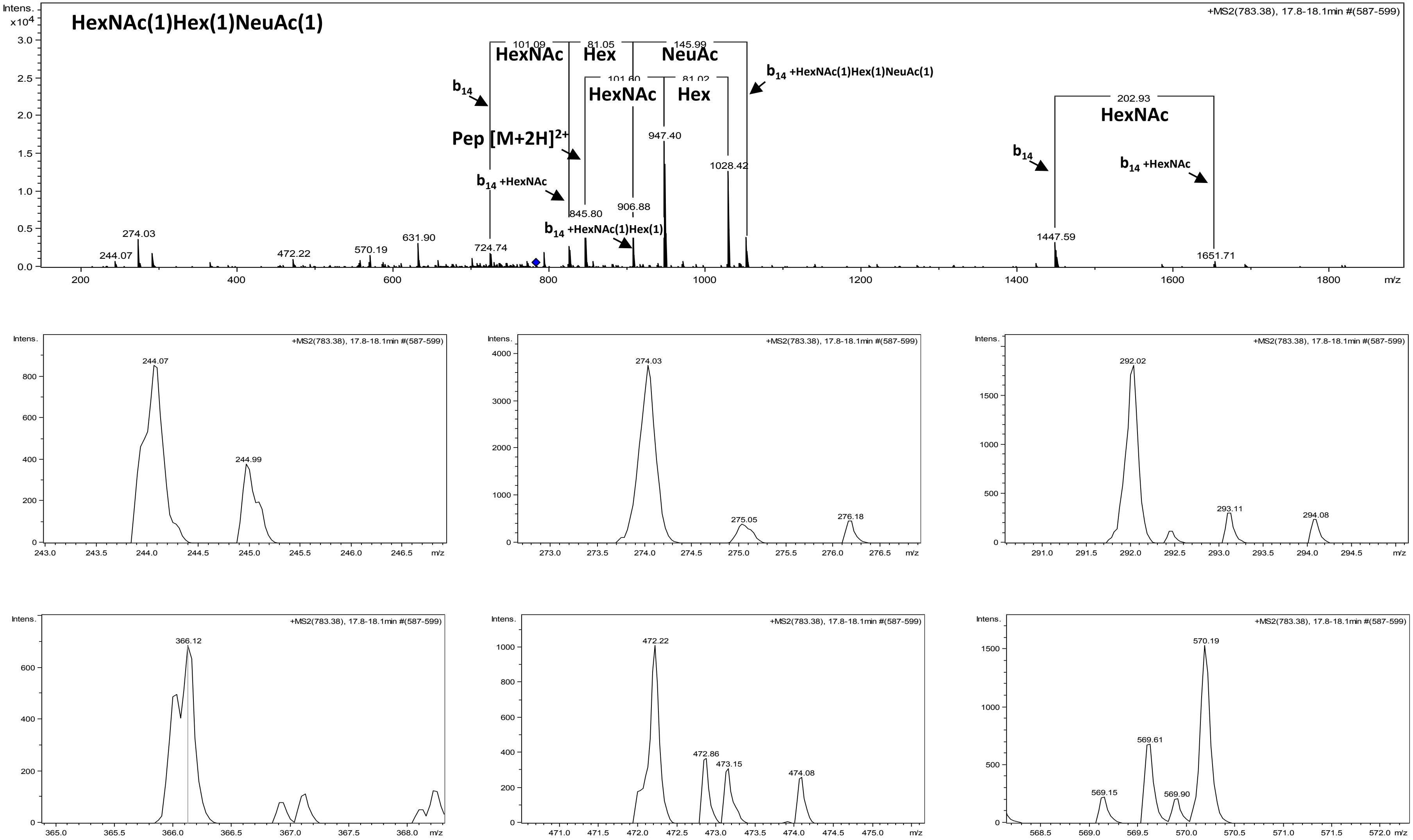

Fraction 16

782.97+++ → Pep[M+2H]++ 845.80++ [17.8-18.1 min]

CID-MS2

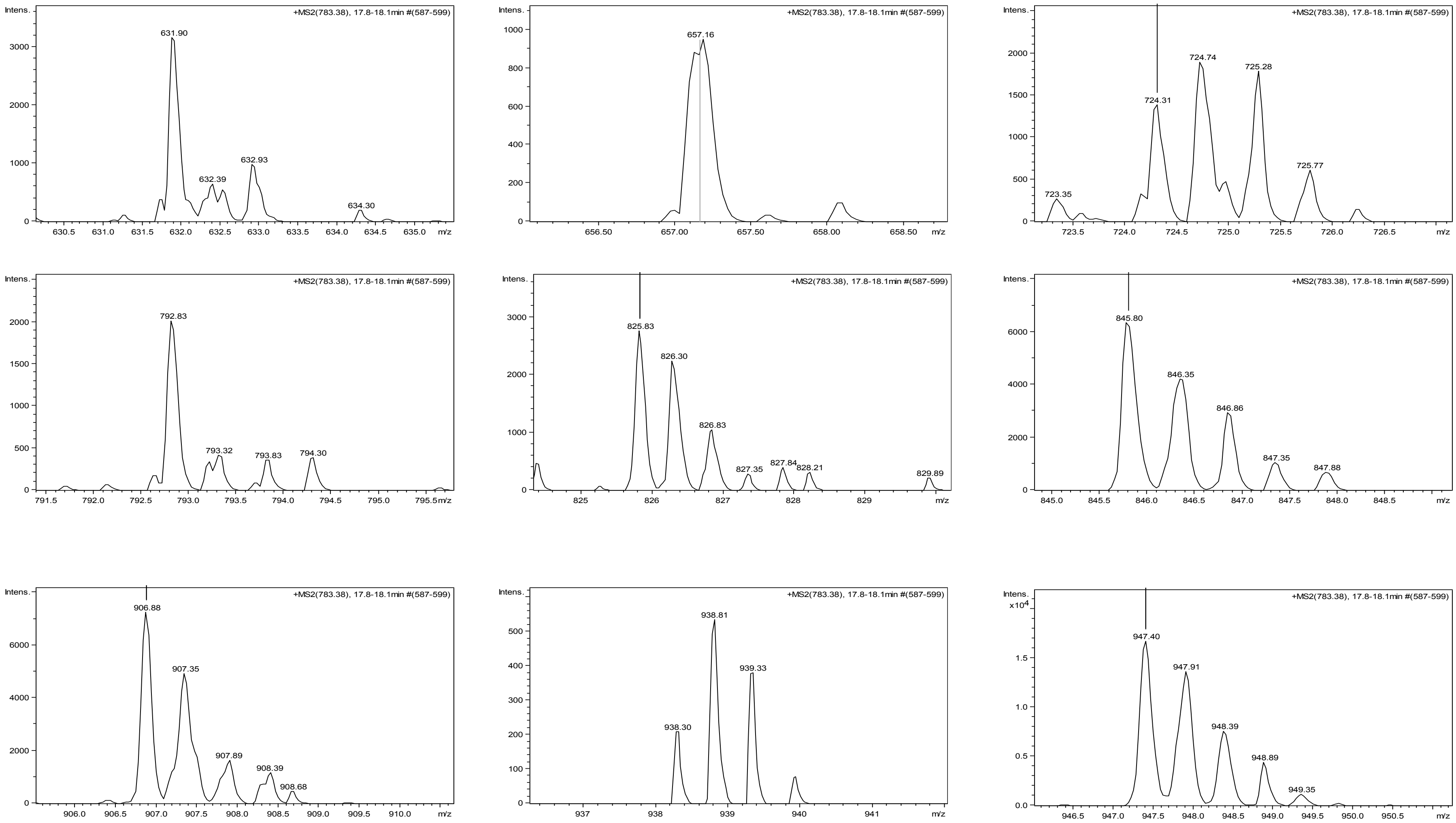

Fraction 16

782.97+++ → Pep[M+2H]++ 845.80++ [17.8-18.1 min] CID-MS2

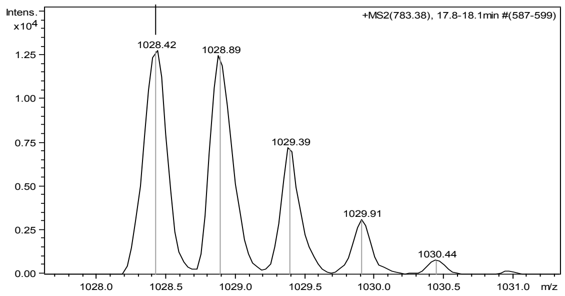

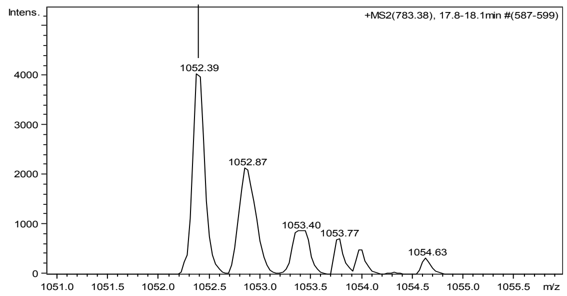

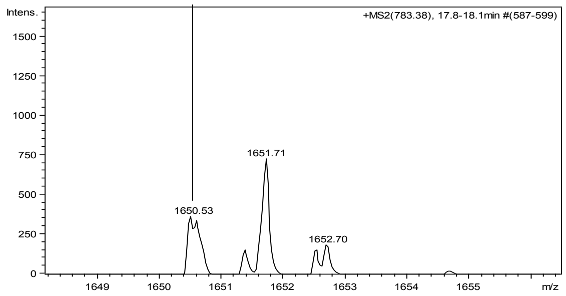

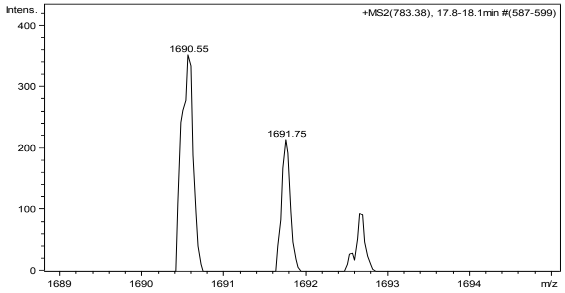

Fraction 16

782.97+++ → Pep[M+2H]++ 845.80++ [17.8-18.1 min] CID-MS3

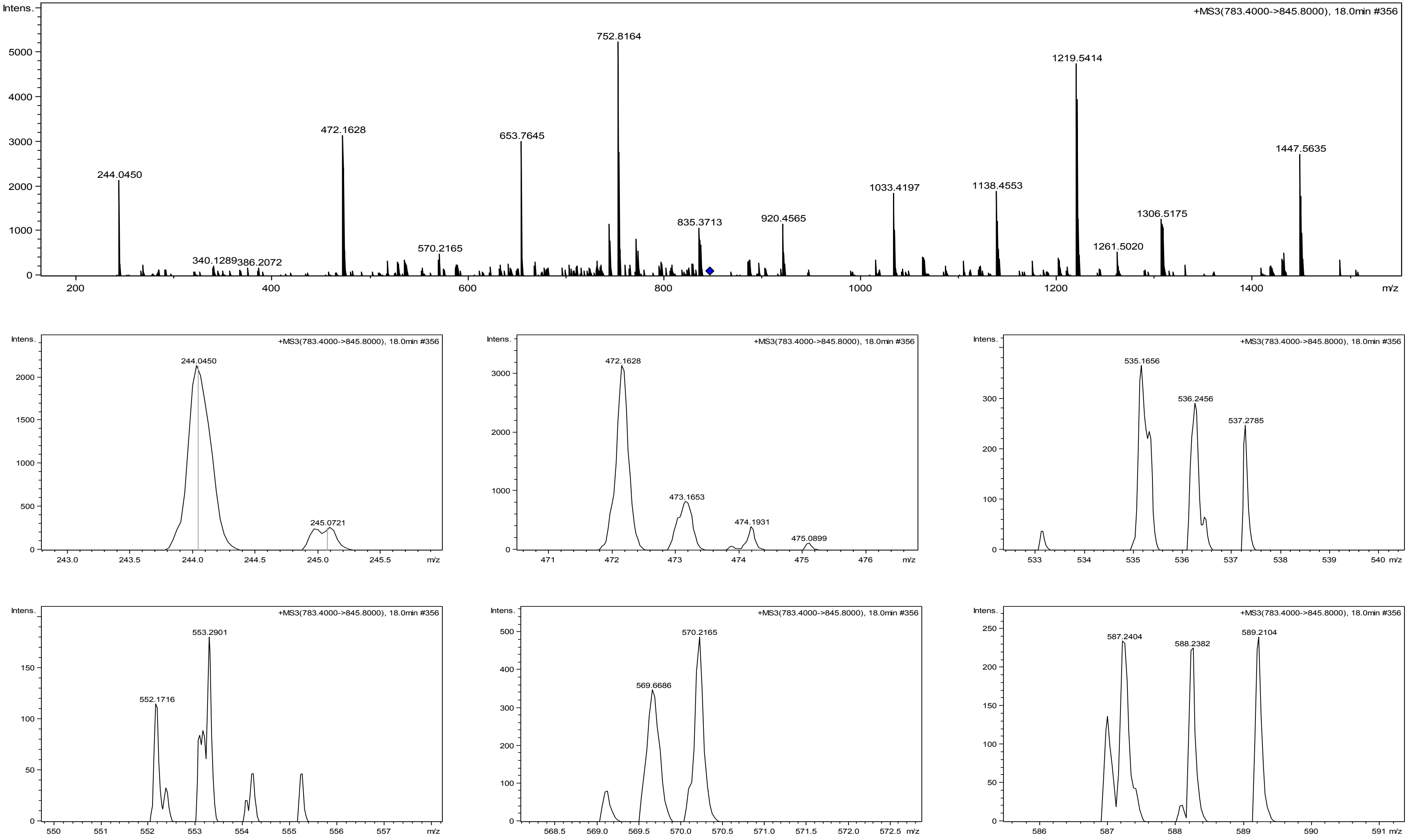

Fraction 16

782.97+++ → Pep[M+2H]++ 845.80++ [17.8-18.1 min]

CID-MS3

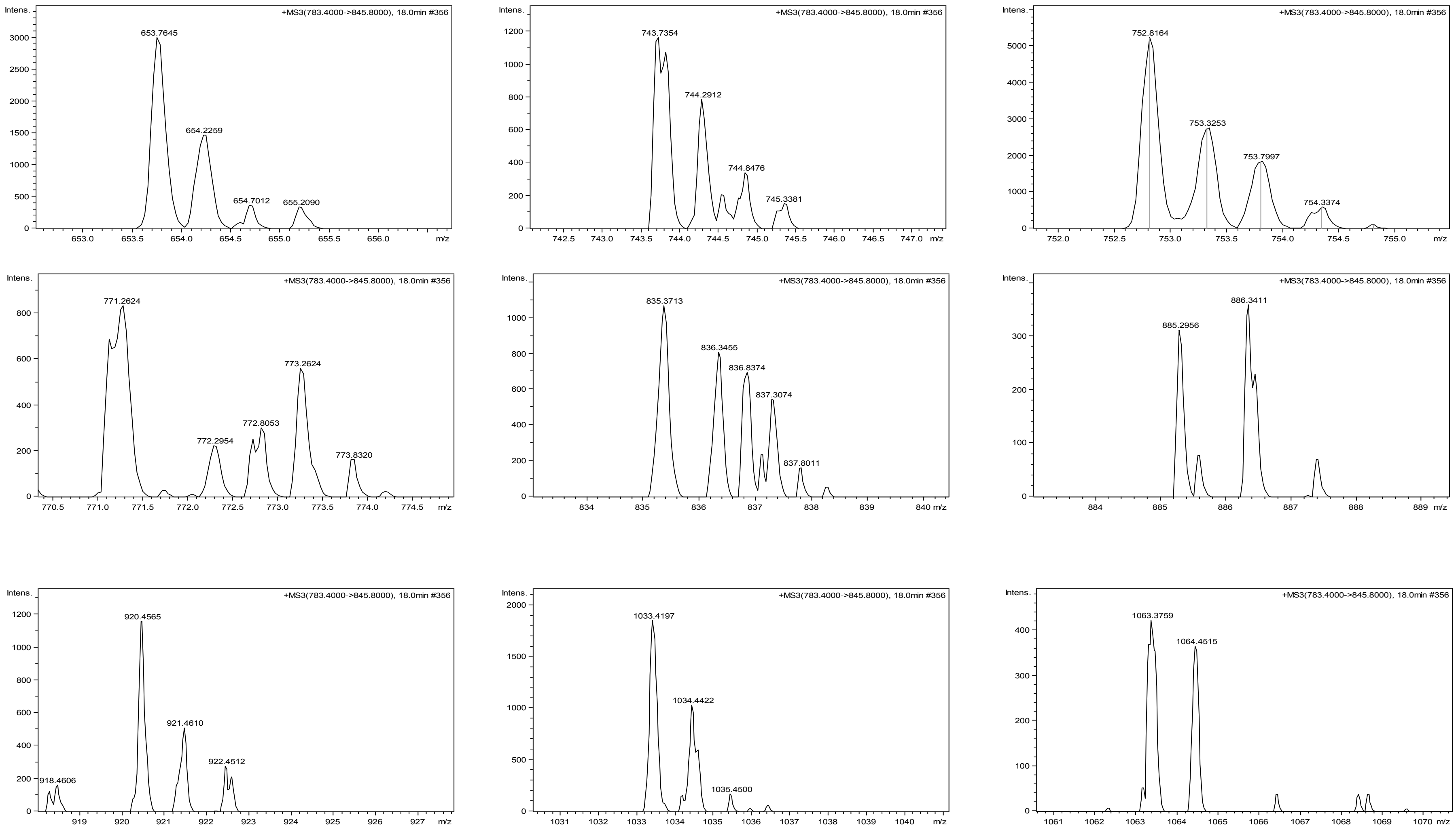

Fraction 16

782.97+++ → Pep[M+2H]++ 845.80++ [17.8-18.1 min] CID-MS3

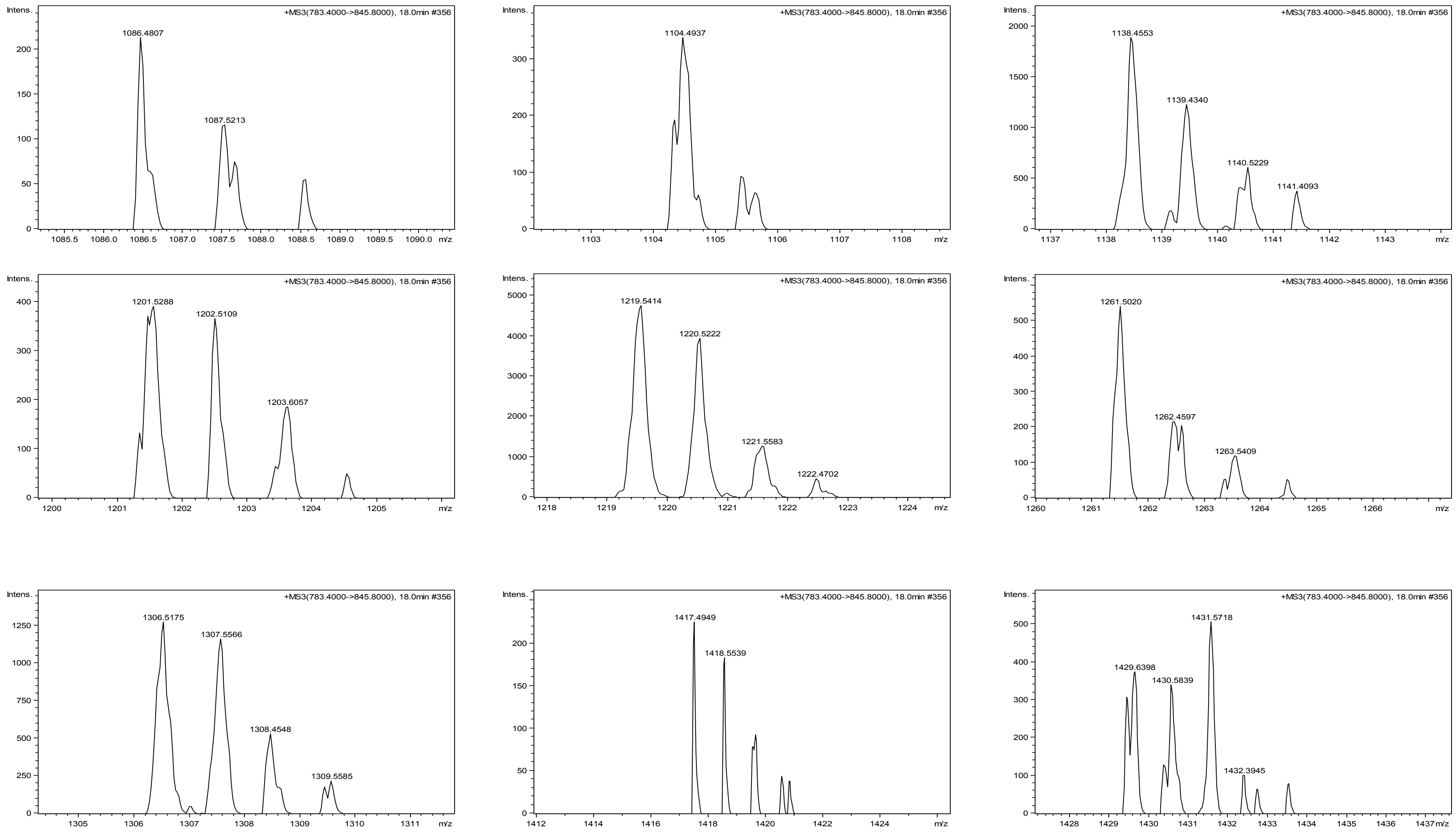

Fraction 16

782.97+++ → Pep[M+2H]++ 845.80++ [17.8-18.1 min] CID-MS3

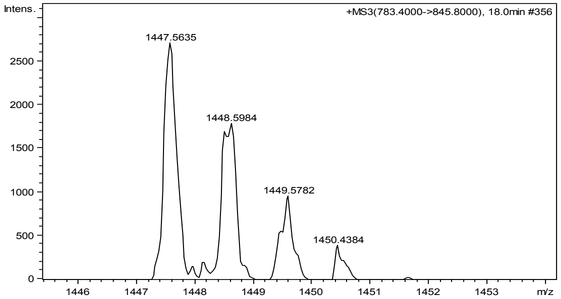

Fraction 16

782.97+++ → Pep[M+2H]++ 845.80++ [17.8-18.1 min]

CID-MS3 MASCOT Search

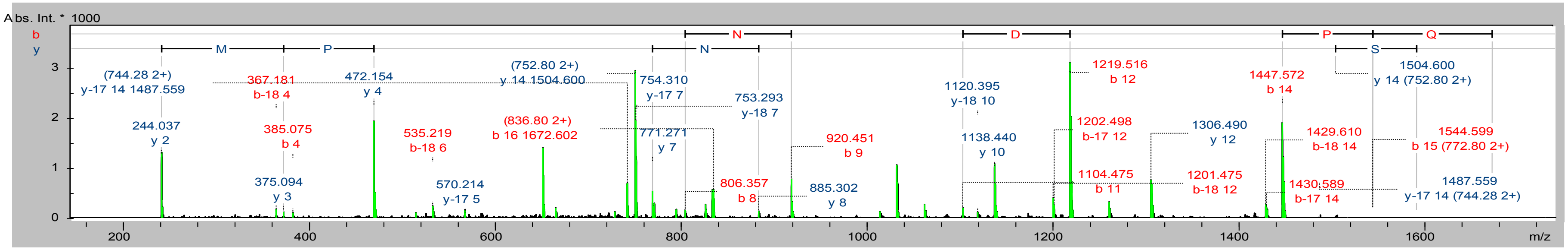

	V	S	P	T	P	A	P	R	N	P	S	D	P	M	P	Q	Val	Ser	Pro	Thr	Pro	Ala	Pro	Arg	Asn	Pro	Ser	Asp	Pro	Met	Pro	Gln
Ion	1	2	3	4	5	6	7	8	9	10	11	12	13	14	15	16	1	2	3	4	5	6	7	8	9	10	11	12	13	14	15	16
b	V	S	P	T	P	A	P	R	N	P	S	D	P	M	P	Q	100.076	187.108	284.160	385.208	482.261	553.298	650.351	806.452	920.495	1017.548	1104.580	1219.607	1316.659	1447.700	1544.753	1672.811
b-17	V	S	P	T	P	A	P	R	N	P	S	D	P	M	P	Q	-	-	-	-	-	-	-	789.425	903.468	1000.521	1087.553	1202.580	1299.633	1430.673	1527.726	1655.785
b-18	V	S	P	T	P	A	P	R	N	P	S	D	P	M	P	Q	-	169.097	266.150	367.198	464.250	535.287	632.340	788.441	902.484	999.537	1086.569	1201.596	1298.649	1429.689	1526.742	1654.801
y	V	S	P	T	P	A	P	R	N	P	S	D	P	M	P	Q	147.076	244.129	375.170	472.222	587.249	674.281	771.334	885.377	1041.478	1138.531	1209.568	1306.621	1407.669	1504.721	1591.753	1690.822
y-17	V	S	P	T	P	A	P	R	N	P	S	D	P	M	P	Q	130.050	227.103	358.143	455.196	570.223	657.255	754.308	868.351	1024.452	1121.504	1192.542	1289.594	1390.642	1487.695	1574.727	1673.795
y-18	V	S	P	T	P	A	P	R	N	P	S	D	P	M	P	Q	-	-	-	-	569.239	656.271	753.324	867.367	1023.468	1120.520	1191.558	1288.610	1389.658	1486.711	1573.743	1672.811
	16	15	14	13	12	11	10	9	8	7	6	5	4	3	2	1	Gln	Pro	Met	Pro	Asp	Ser	Pro	Asn	Arg	Pro	Ala	Pro	Thr	Pro	Ser	Val

unknown O-glycosylation region

Complement C4-B

$_{1241}$VSPTPAPRNPSDPMPQ$_{1256}$

Fraction 16

782.97+++ → Pep[M+2H]++ 845.80++ [17.8-18.1 min]

CID-MS3 MASCOT Search

prot_hit_nur	prot_acc	prot_desc	prot_score	prot_mass	prot_matche	pep_query	pep_rank	pep_isbold	pep_exp_mz	pep_exp_mr	pep_exp_z	pep_calc_mr	pep_delta	pep_miss	pep_score	pep_expect	pep_res_bef	pep_seq
1	CO4B_HUMA	Complemen	26	194212	1	1	1	1	845.8039	1689.5932	2	1689.8145	-0.2212	0	33.8	7.2	A	VSPTPAPRNI
2	ITAV_HUMAI	Integrin alph	10	117062	1	1	2	0	845.8039	1689.5932	2	1688.8508	0.7424	0	17.32	3.20E+02	Q	GEGAYEAELIV
3	PDE3A_HUM	cGMP-inhibi	10	126440	1	1	3	0	845.8039	1689.5932	2	1689.7888	-0.1956	0	17.26	3.20E+02	L	VCQMCIKLAI
4	RPKL1_HUM	Ribosomal p	9	60740	1	1	6	0	845.8039	1689.5932	2	1689.817	-0.2237	0	14.06	6.70E+02	E	GEPTARTSTS
5	CC45L_HUM	CDC45-relate	8	66211	1	1	5	0	845.8039	1689.5932	2	1689.8436	-0.2504	0	14.08	6.70E+02	S	KDLNDMLWV
6	XAB2_HUMA	XPA-binding	7	100745	1	1	7	0	845.8039	1689.5932	2	1689.8607	-0.2675	0	13.53	7.60E+02	Q	MLKVSGSATC
7	DYH8_HUMA	Ciliary dynei	6	517984	1	1	4	0	845.8039	1689.5932	2	1689.8308	-0.2376	0	16.17	4.10E+02	I	ENLNSVLDDN
8	PLXA2_HUM	Plexin-A2 pr	5	215770	1	1	8	0	845.8039	1689.5932	2	1688.8443	0.7489	0	12.89	8.80E+02	N	LELNWLLGKD
9	SCN8A_HUM	Sodium chan	4	227358	1	1	9	0	845.8039	1689.5932	2	1689.8243	-0.2311	0	12.2	1.00E+03	R	VLGDSGELDII

Biotools-Score: 30

MASCOT-Score: 34

unknown O-glycosylation region

Complement C4-B

$_{1241}$VSPTPAPRNPSDPMPQ$_{1256}$

Fraction 16

782.97+++ → Pep[M+2H]++ 845.80++ [17.8-18.1 min] ETD

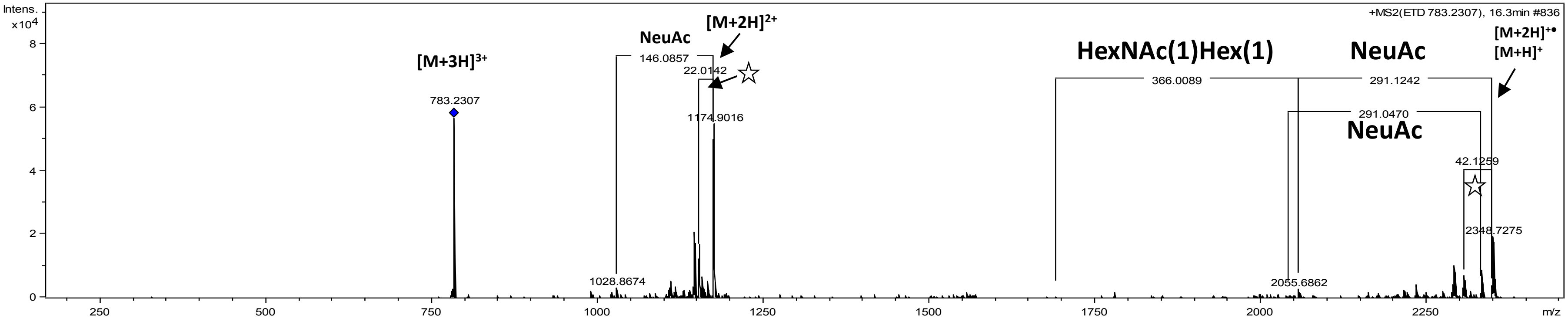

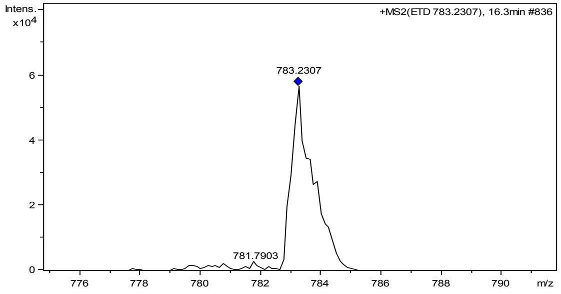

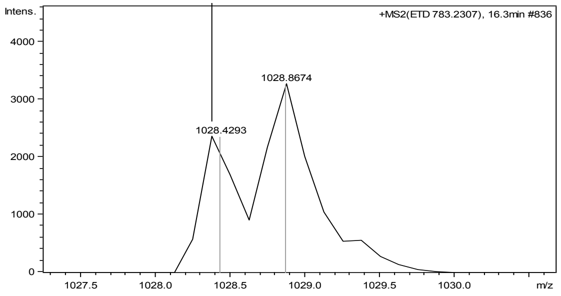

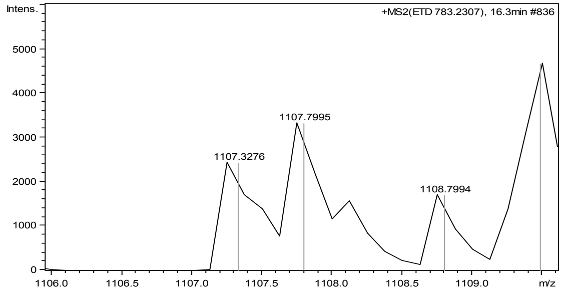

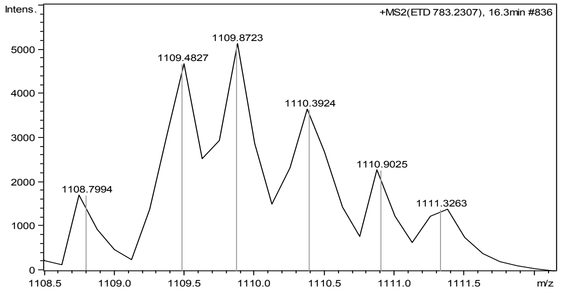

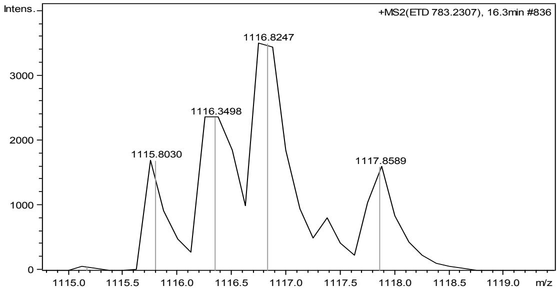

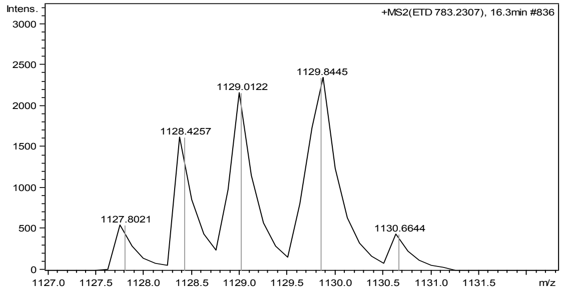

Fraction 16

782.97+++ → Pep[M+2H]++ 845.80++ [17.8-18.1 min] ETD

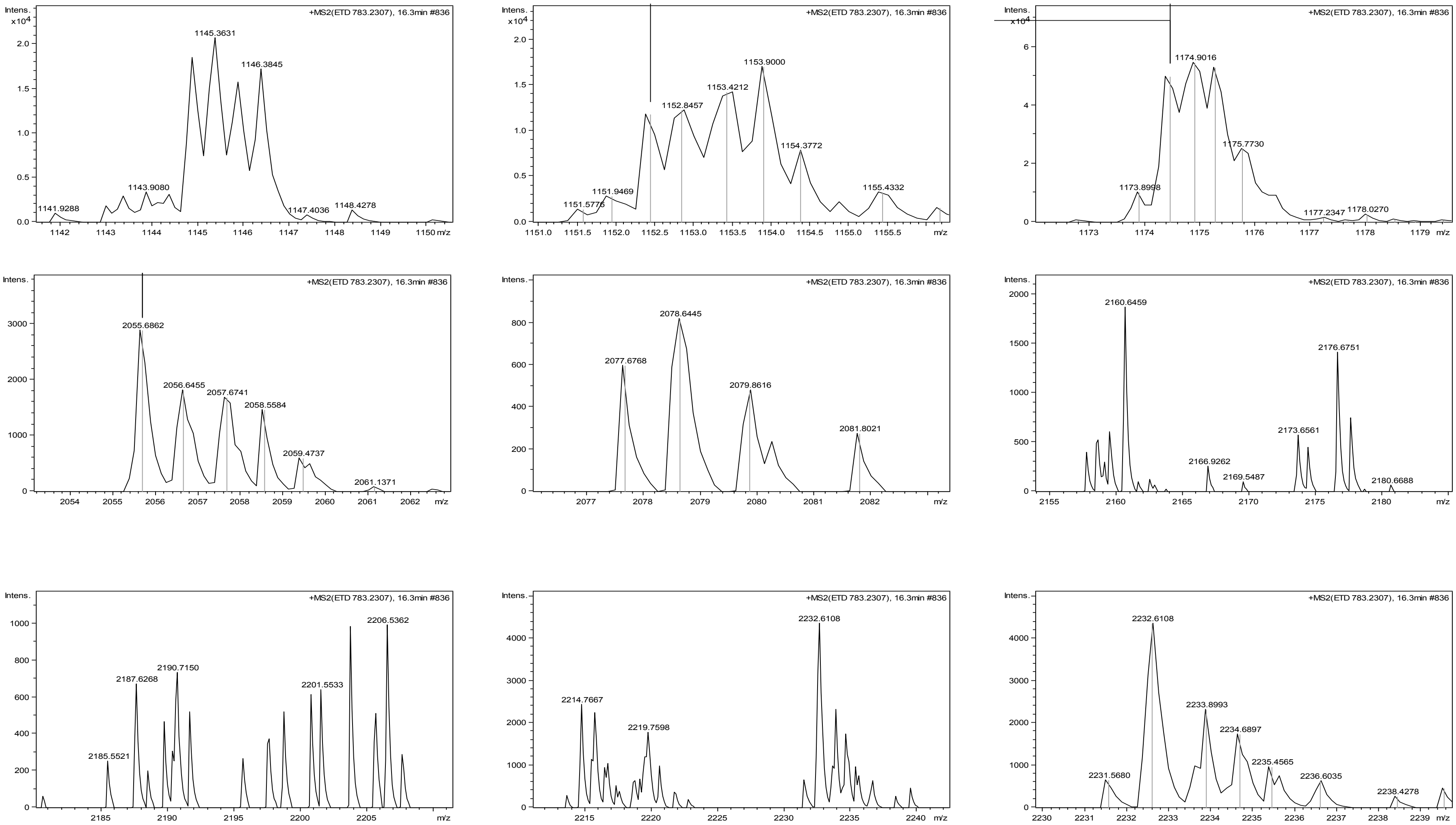

Fraction 16

782.97+++ → Pep[M+2H]++ 845.80++ [17.8-18.1 min] ETD

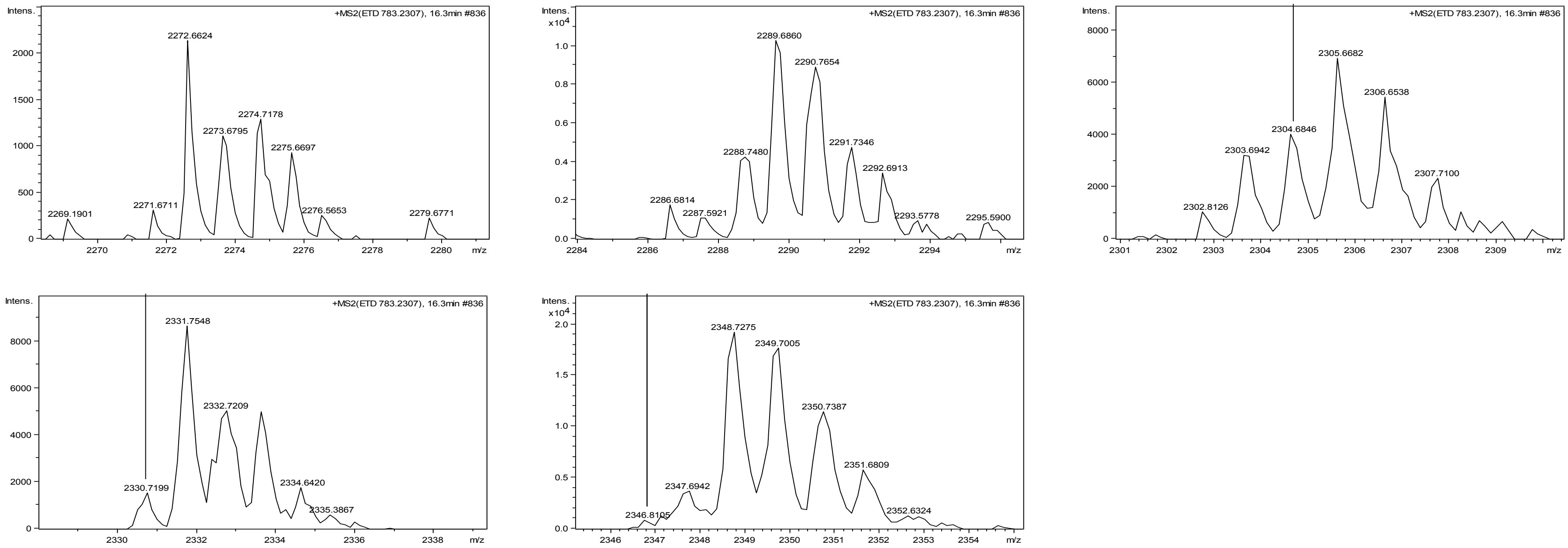

Fraction 16

782.97+++ → Pep[M+2H]++ 845.80++ [17.8-18.1 min] ETD

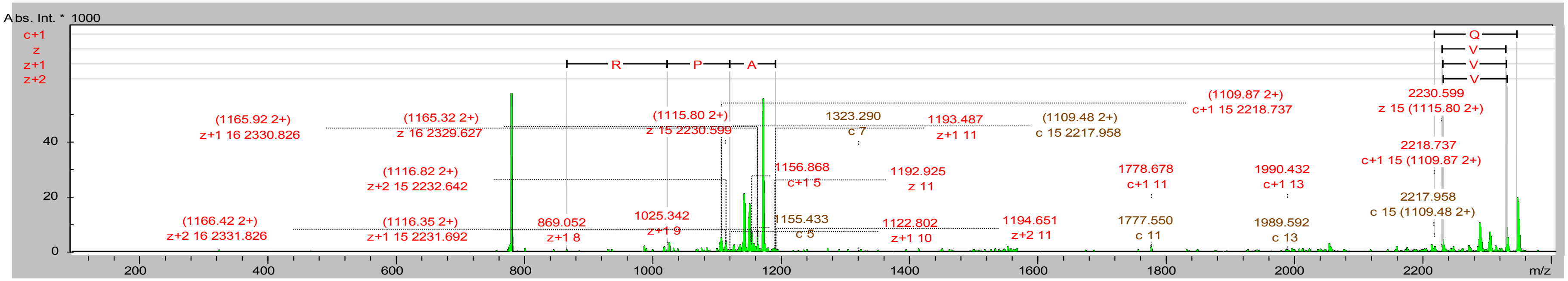

V<u>S</u>PTPAPRNPSDPMPQ

	V	S	P	T	P	A	P	R	N	P	S	D	P	M	P	Q
Ion	1	2	3	4	5	6	7	8	9	10	11	12	13	14	15	16
c	V	S*	P	T	P	A	P	R	N	P	S	D	P	M	P	Q
c+1	V	S*	P	T	P	A	P	R	N	P	S	D	P	M	P	Q
z	V	S*	P	T	P	A	P	R	N	P	S	D	P	M	P	Q
z+1	V	S*	P	T	P	A	P	R	N	P	S	D	P	M	P	Q
z+2	V	S*	P	T	P	A	P	R	N	P	S	D	P	M	P	Q
	16	15	14	13	12	11	10	9	8	7	6	5	4	3	2	1

	Val	Ser	Pro	Thr	Pro	Ala	Pro	Arg	Asn	Pro	Ser	Asp	Pro	Met	Pro	Gln
Ion	1	2	3	4	5	6	7	8	9	10	11	12	13	14	15	16
c	117.102	860.362	957.415	1058.462	1155.515	1226.552	1323.605	1479.706	1593.749	1690.802	1777.834	1892.861	1989.914	2120.954	2218.007	2346.065
c+1	118.110	861.370	958.422	1059.470	1156.523	1227.560	1324.613	1480.714	1594.757	1691.810	1778.842	1893.869	1990.921	2121.962	2219.015	2347.073
z	130.050	227.103	358.143	455.196	570.223	657.255	754.308	868.351	1024.452	1121.504	1192.542	1289.594	1390.642	1487.695	2230.954	2330.023
z+1	131.058	228.110	359.151	456.204	571.231	658.263	755.315	869.358	1025.459	1122.512	1193.549	1290.602	1391.650	1488.703	2231.962	2331.031
z+2	132.066	229.118	360.159	457.212	572.238	659.271	756.323	870.366	1026.467	1123.520	1194.557	1291.610	1392.658	1489.710	2232.970	2332.038
	Gln	Pro	Met	Pro	Asp	Ser	Pro	Asn	Arg	Pro	Ala	Pro	Thr	Pro	Ser	Val

Biotools-Score: 36

unknown O-glycosylation region

Complement C4-B

$_{1241}$VSPTPAPRNPSDPMPQ$_{1256}$

Fraction 16

782.97+++ → Pep[M+2H]++ 845.80++ [17.8-18.1 min] ETD

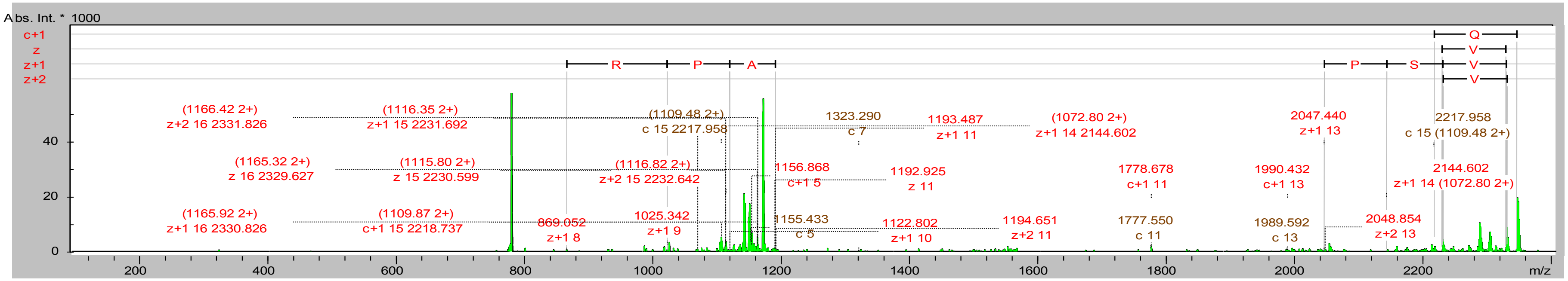

VSP**T**PAPRNPSDPMPQ

	V	S	P	T	P	A	P	R	N	P	S	D	P	M	P	Q
Ion	1	2	3	4	5	6	7	8	9	10	11	12	13	14	15	16
c	V	S	P	T*	P	A	P	R	N	P	S	D	P	M	P	Q
c+1	V	S	P	T*	P	A	P	R	N	P	S	D	P	M	P	Q
z	V	S	P	T*	P	A	P	R	N	P	S	D	P	M	P	Q
z+1	V	S	P	T*	P	A	P	R	N	P	S	D	P	M	P	Q
z+2	V	S	P	T*	P	A	P	R	N	P	S	D	P	M	P	Q
	16	15	14	13	12	11	10	9	8	7	6	5	4	3	2	1

	Val	Ser	Pro	Thr	Pro	Ala	Pro	Arg	Asn	Pro	Ser	Asp	Pro	Met	Pro	Gln
Ion	1	2	3	4	5	6	7	8	9	10	11	12	13	14	15	16
c	117.102	204.134	301.187	1058.462	1155.515	1226.552	1323.605	1479.706	1593.749	1690.802	1777.834	1892.861	1989.914	2120.954	2218.007	2346.065
c+1	118.110	205.142	302.195	1059.470	1156.523	1227.560	1324.613	1480.714	1594.757	1691.810	1778.842	1893.869	1990.921	2121.962	2219.015	2347.073
z	130.050	227.103	358.143	455.196	570.223	657.255	754.308	868.351	1024.452	1121.504	1192.542	1289.594	2046.870	2143.922	2230.954	2330.023
z+1	131.058	228.110	359.151	456.204	571.231	658.263	755.315	869.358	1025.459	1122.512	1193.549	1290.602	2047.877	2144.930	2231.962	2331.031
z+2	132.066	229.118	360.159	457.212	572.238	659.271	756.323	870.366	1026.467	1123.520	1194.557	1291.610	2048.885	2145.938	2232.970	2332.038
	Gln	Pro	Met	Pro	Asp	Ser	Pro	Asn	Arg	Pro	Ala	Pro	Thr	Pro	Ser	Val

Biotools-Score: 49

unknown O-glycosylation region

Complement C4-B $_{1241}$V**S**P**T**PAPRNP**S**DPMPQ$_{1256}$

Fraction 16

782.97+++ → Pep[M+2H]++ 845.80++ [17.8-18.1 min] ETD

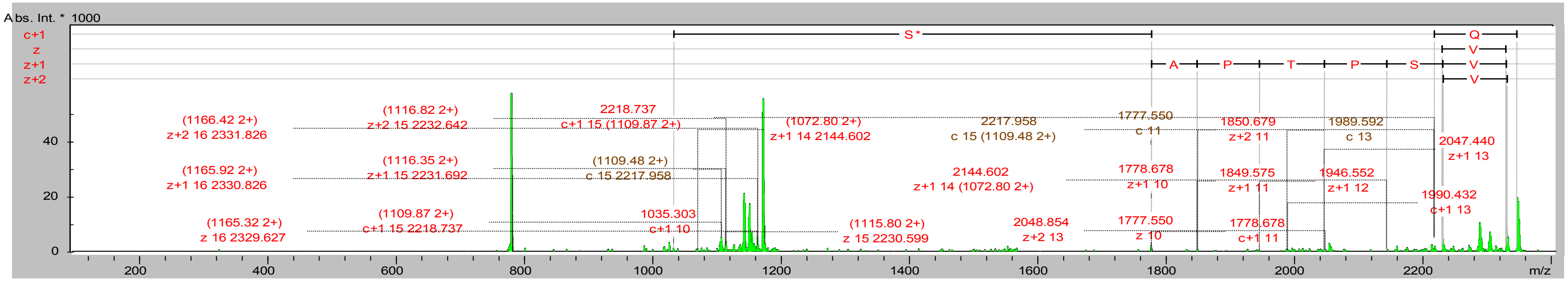

VSPTPAPRNP**S**DPMPQ **Most likely**

	V	S	P	T	P	A	P	R	N	P	S	D	P	M	P	Q	Val	Ser	Pro	Thr	Pro	Ala	Pro	Arg	Asn	Pro	Ser	Asp	Pro	Met	Pro	Gln
Ion	1	2	3	4	5	6	7	8	9	10	11	12	13	14	15	16	1	2	3	4	5	6	7	8	9	10	11	12	13	14	15	16
c	V	S	P	T	P	A	P	R	N	P	S*	D	P	M	P	Q	117.102	204.134	301.187	402.235	499.287	570.325	667.377	823.478	937.521	1034.574	1777.834	1892.861	1989.914	2120.954	2218.007	2346.065
c+1	V	S	P	T	P	A	P	R	N	P	S*	D	P	M	P	Q	118.110	205.142	302.195	403.243	500.295	571.332	668.385	824.486	938.529	1035.582	1778.842	1893.869	1990.921	2121.962	2219.015	2347.073
z	V	S	P	T	P	A	P	R	N	P	S*	D	P	M	P	Q	130.050	227.103	358.143	455.196	570.223	1313.482	1410.535	1524.578	1680.679	1777.732	1848.769	1945.822	2046.870	2143.922	2230.954	2330.023
z+1	V	S	P	T	P	A	P	R	N	P	S*	D	P	M	P	Q	131.058	228.110	359.151	456.204	571.231	1314.490	1411.543	1525.586	1681.687	1778.740	1849.777	1946.830	2047.877	2144.930	2231.962	2331.031
z+2	V	S	P	T	P	A	P	R	N	P	S*	D	P	M	P	Q	132.066	229.118	360.159	457.212	572.238	1315.498	1412.551	1526.594	1682.695	1779.748	1850.785	1947.838	2048.885	2145.938	2232.970	2332.038
	16	15	14	13	12	11	10	9	8	7	6	5	4	3	2	1	Gln	Pro	Met	Pro	Asp	Ser	Pro	Asn	Arg	Pro	Ala	Pro	Thr	Pro	Ser	Val

Biotools-Score: 145

unknown O-glycosylation region

Complement C4-B

$_{1241}$V**S**P**T**PAPRNP**S**DPMPQ$_{1256}$

Fraction 16

790.27++ → Pep [M+H]+ 632.19+ [18.2 min] CID-MS Precursor

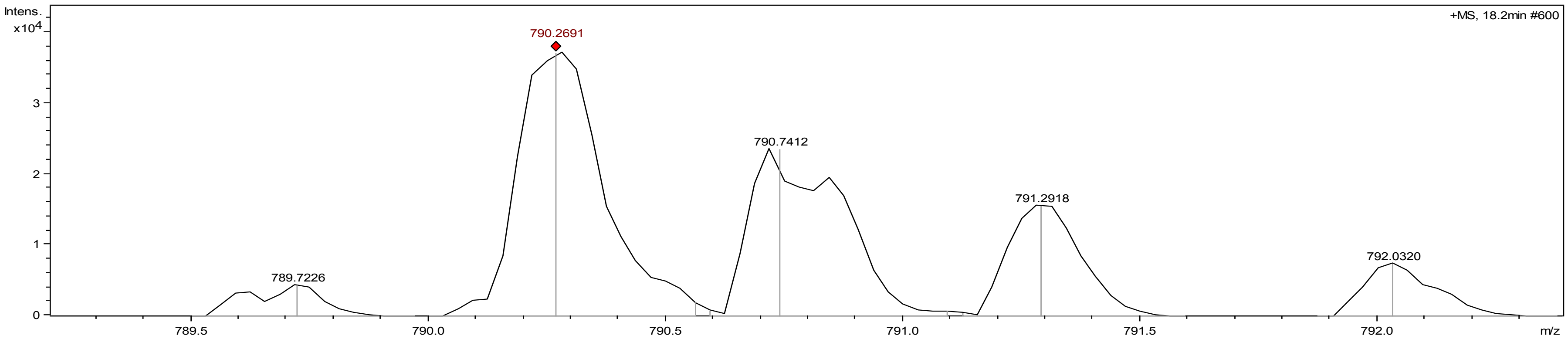

ETD spectrum of poor quality

CID-MS3 spectrum of poor quality

Fraction 16

790.27++ → Pep [M+H]+ 632.19+ [18.2 min] CID-MS2

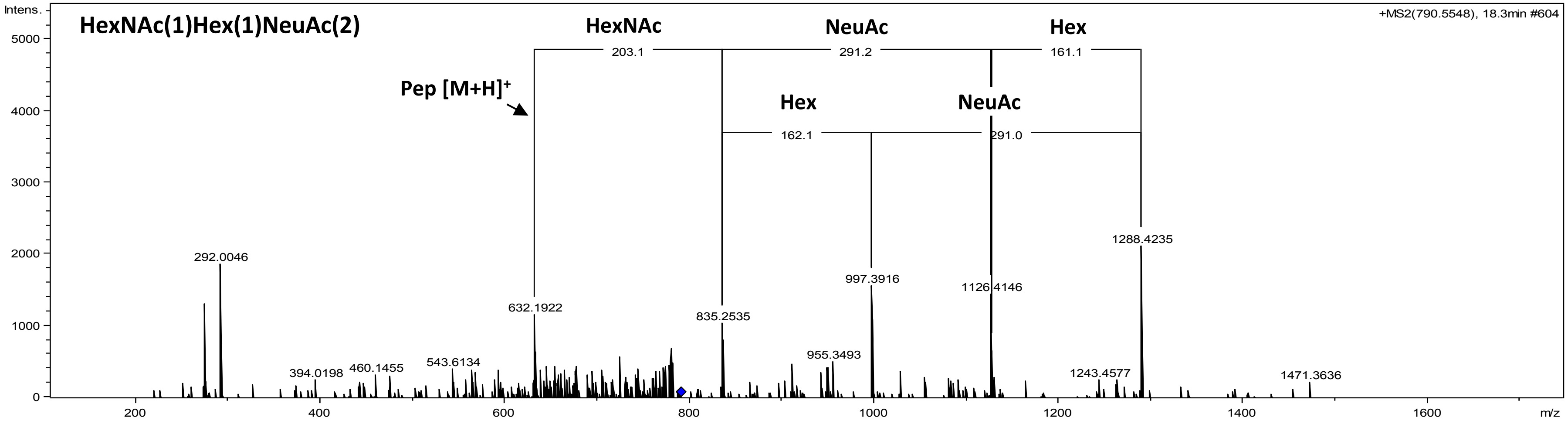

Fraction 16

899.77++ → Pep [M+H]+ 851.33+ [19.8 min]

CID-MS Precursor

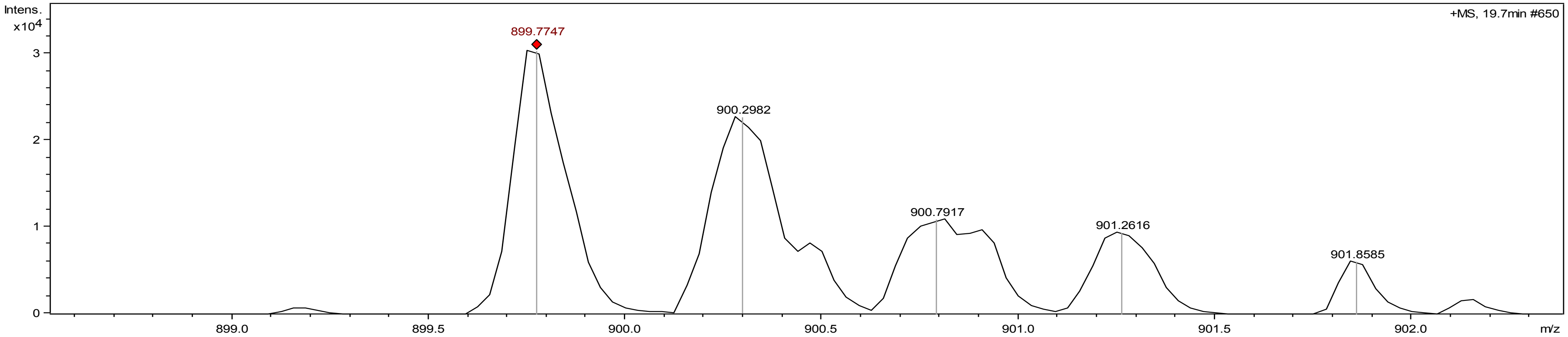

ETD spectrum of poor quality

CID-MS3 spectrum of poor quality

Fraction 16

899.77++ → Pep [M+H]+ 851.33+ [19.8 min] CID-MS2

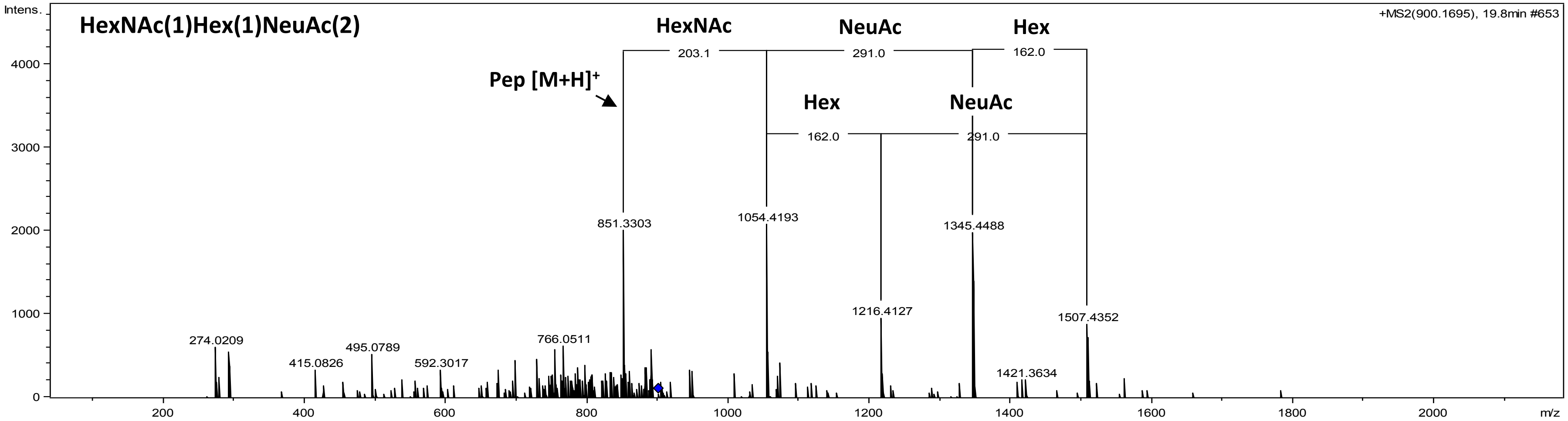

Fraction 16

585.61+++ → Pep+HexNAc [M+2H]++ 651.27++ [20.7 min]   CID-MS Precursor

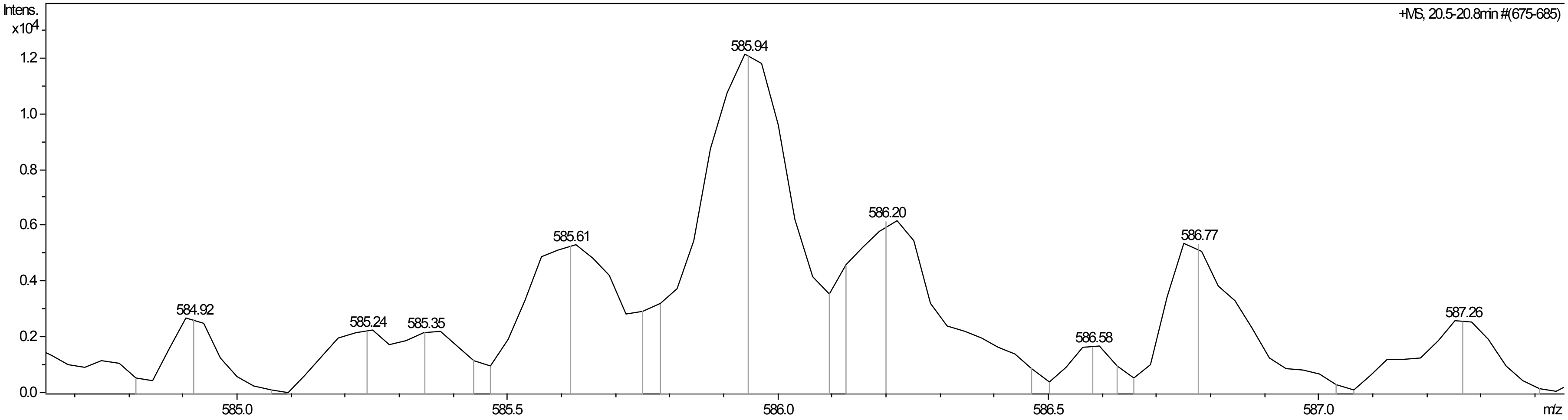

ETD spectrum of poor quality

CID-MS3 spectrum of poor quality

Fraction 16

585.61+++ → Pep+HexNAc [M+2H]++ 651.27++ [20.7 min] CID-MS2

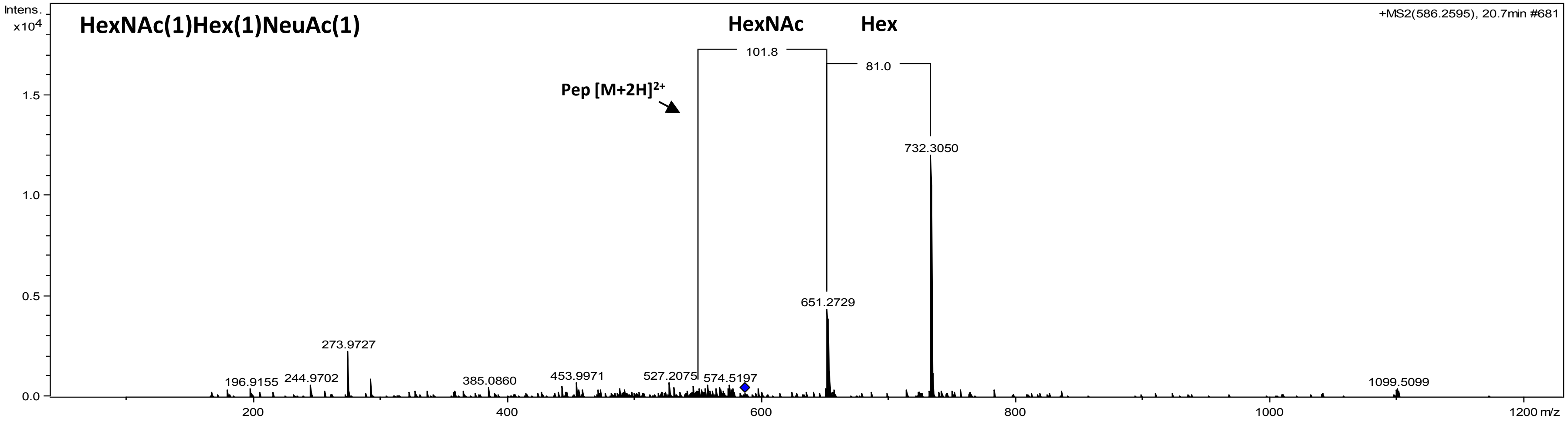

Fraction 16

877.86++ → Pep [M+H]+ 1098.51+ [20.6-20.8 min]　　CID-MS Precursor

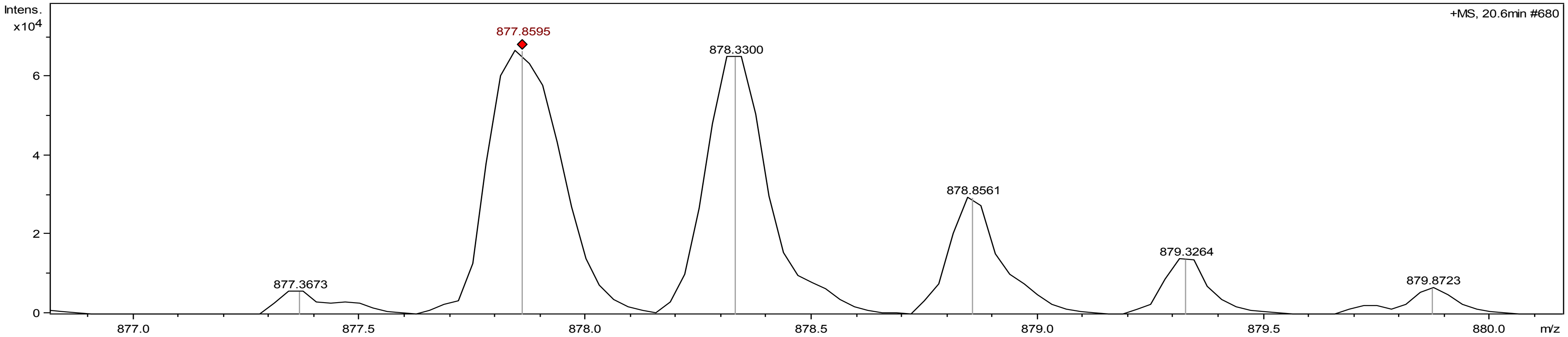

ETD spectrum of poor quality

CID-MS3 spectrum of poor quality

Fraction 16

877.86++ → Pep [M+H]+ 1098.51+ [20.6-20.8 min] CID-MS2

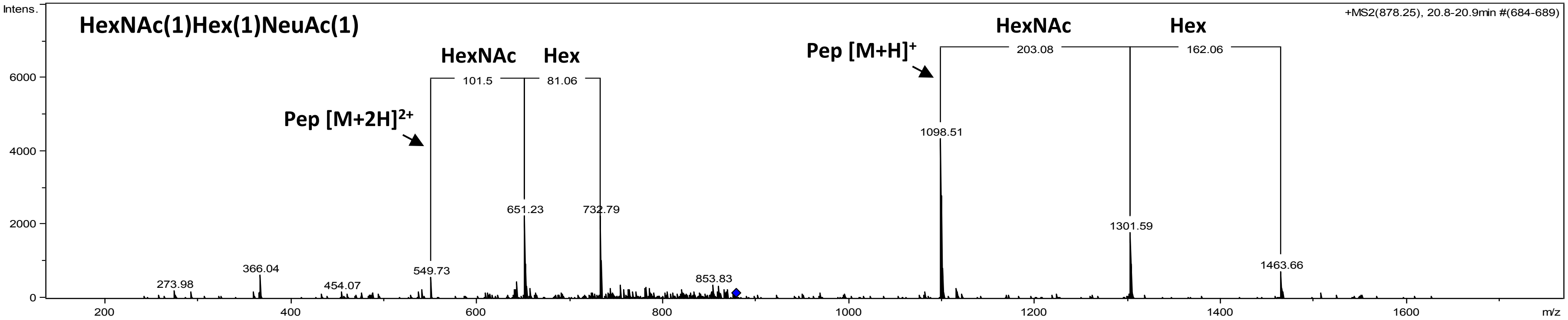

Fraction 16

609.20+++ → Pep+HexNAc [M+2H]++ 686.80++ [21.1 min]

CID-MS Precursor

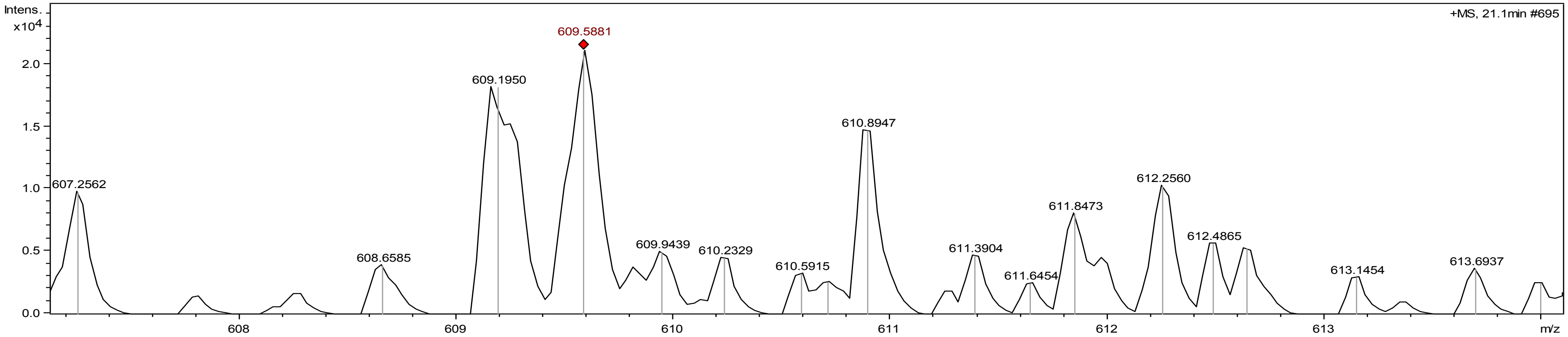

ETD spectrum of poor quality

CID spectrum of poor quality

Fraction 16

609.20+++ → Pep+HexNAc [M+2H]++ 686.80++ [21.1 min]

CID-MS2

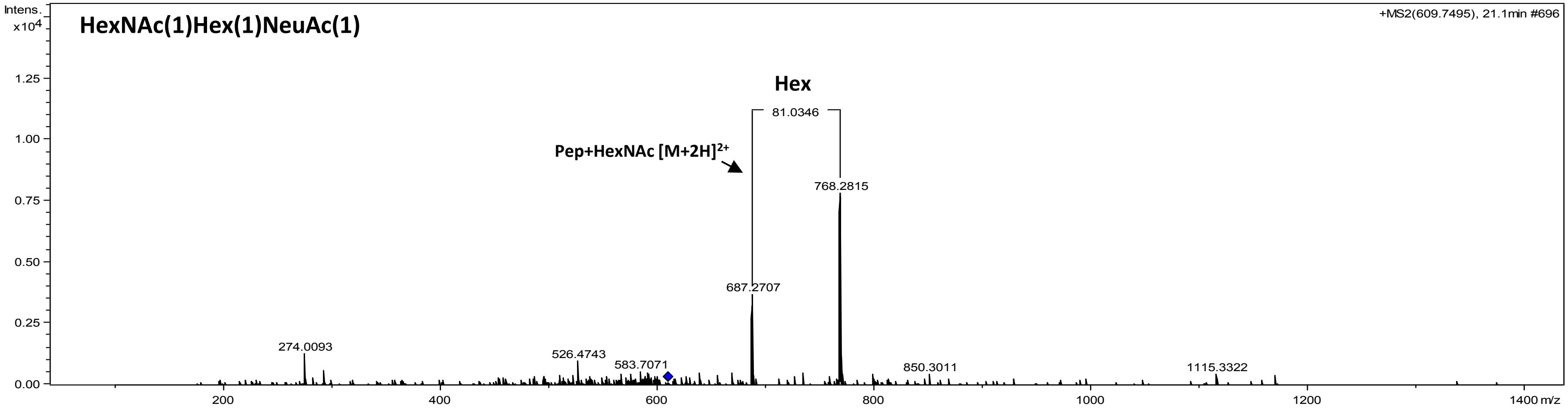

ETD spectrum of poor quality

CID spectrum of poor quality

Fraction 16

572.52+++ → Pep+HexNAc [M+2H]++ 631.65++ [21.5]

CID-MS Precursor

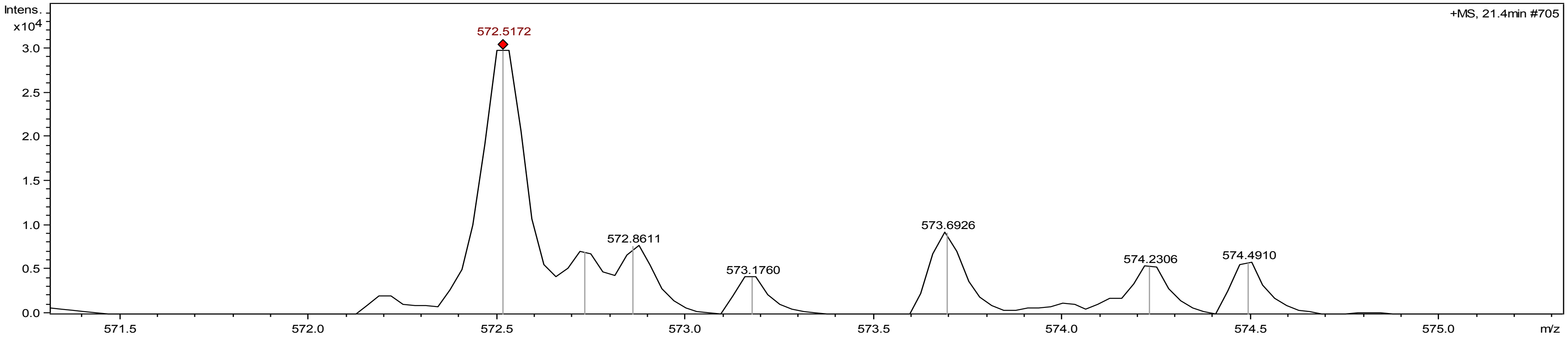

ETD spectrum of poor quality

CID spectrum of poor quality

Fraction 16

572.52+++ → Pep+HexNAc [M+2H]++ 631.65++ [21.5]

CID-MS2

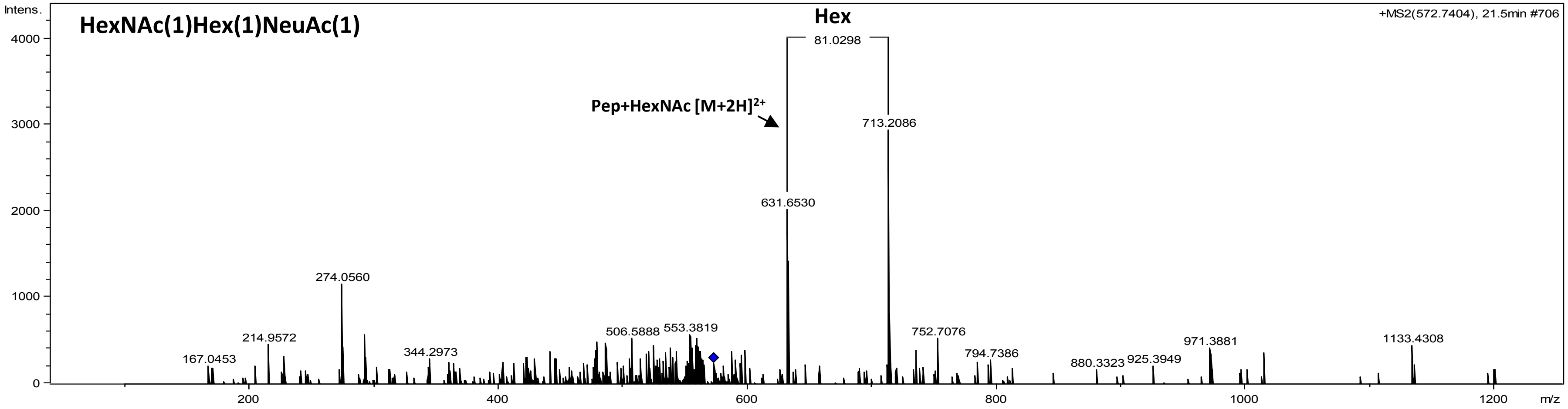

ETD spectrum of poor quality

CID spectrum of poor quality

Fraction 16

572.52+++ → Pep+HexNAc [M+2H]++ 631.65++ [21.5]

CID-MS3

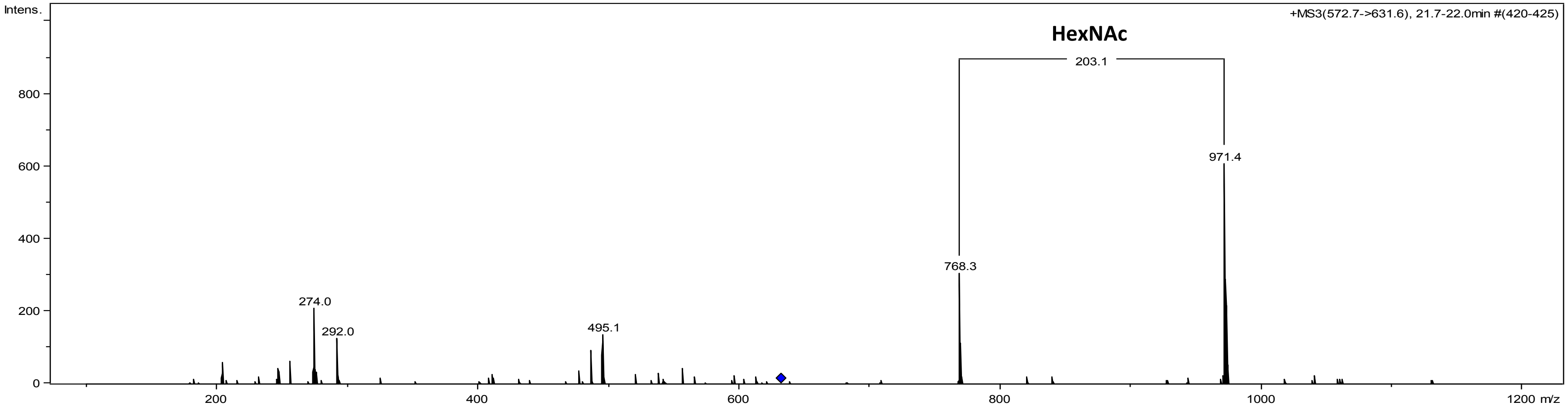

ETD spectrum of poor quality

CID spectrum of poor quality

Fraction 16

830.34++ → Pep [M+H]+ 712.32+ [21.9-22.2 min] CID-MS Precursor

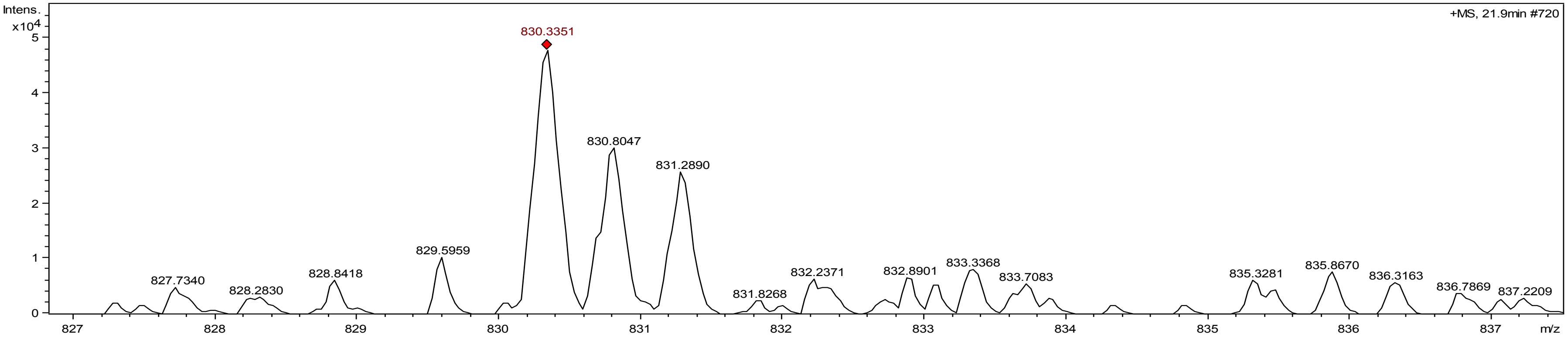

Fraction 16

830.34++ → Pep [M+H]+ 712.32+ [21.9-22.2 min] CID-MS2

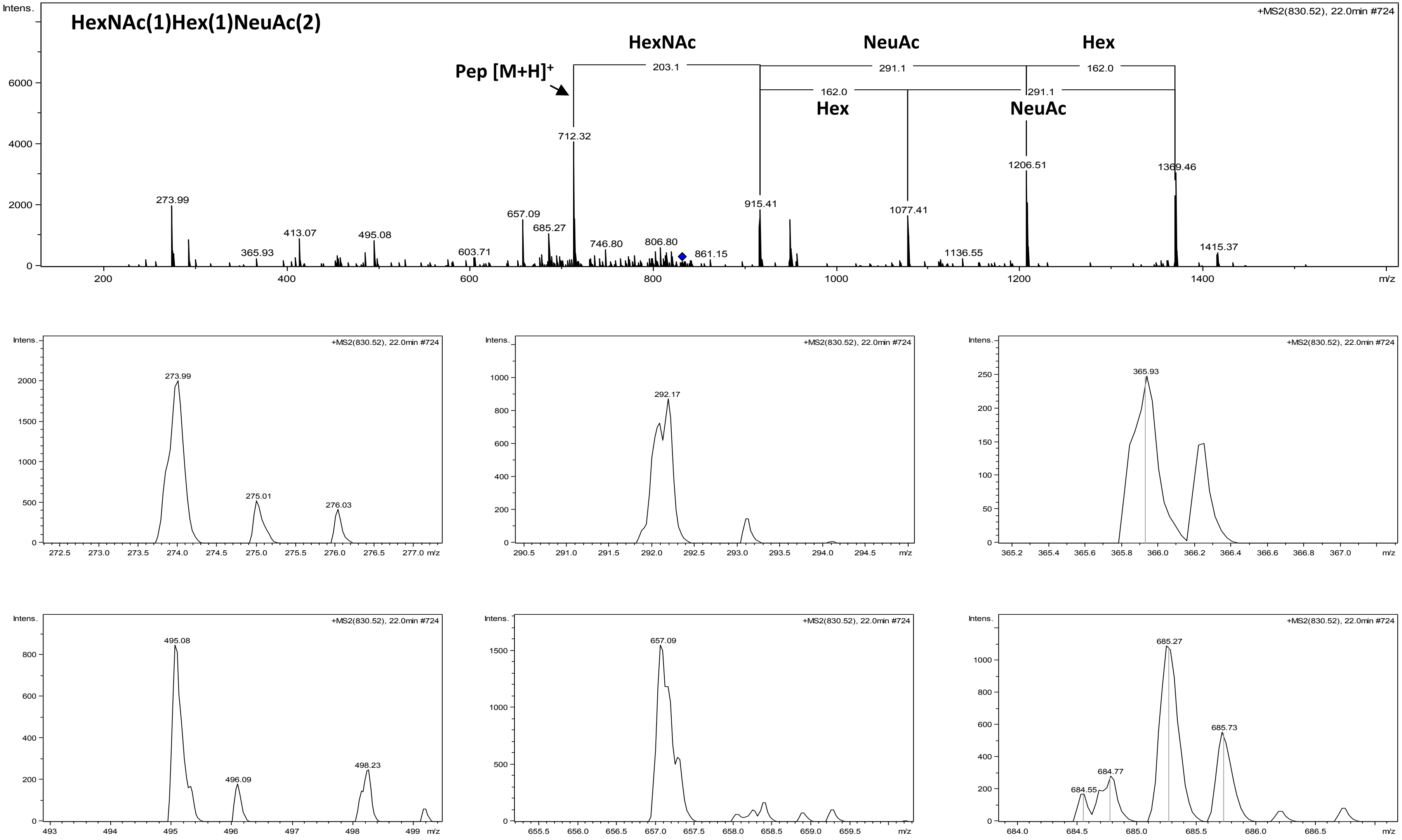

Fraction 16

830.34++ → Pep [M+H]+ 712.32+ [21.9-22.2 min]

CID-MS2

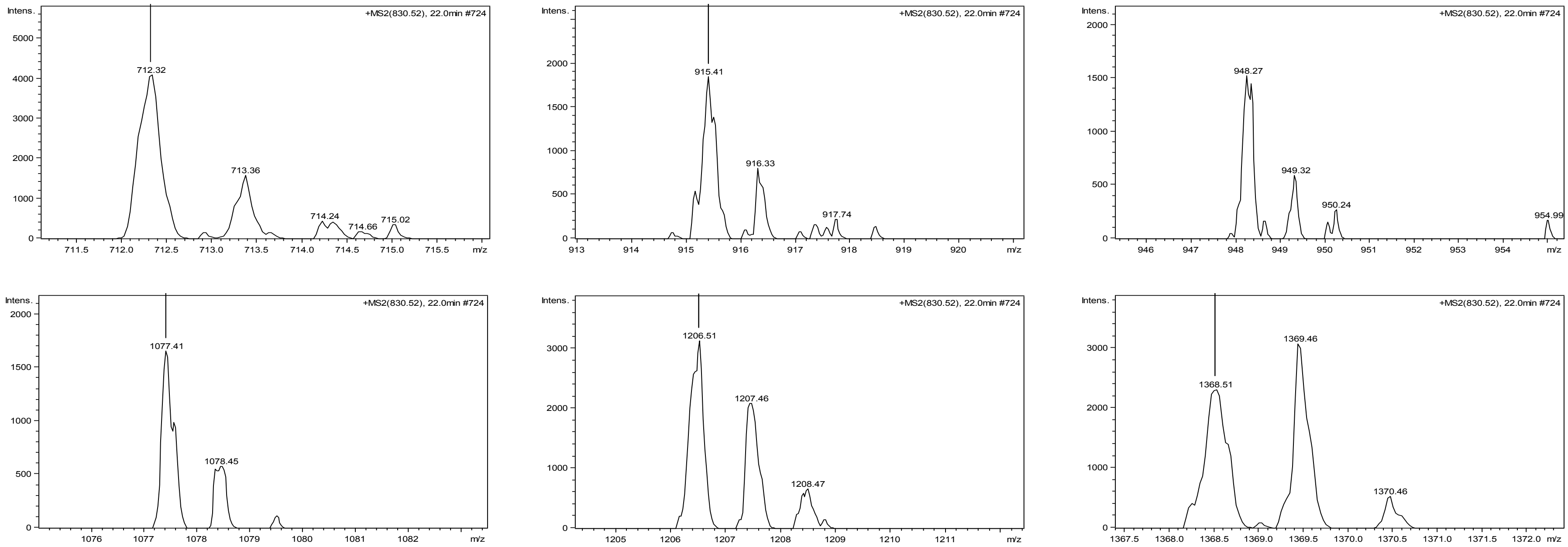

Fraction 16

830.34++ → Pep [M+H]+ 712.32+ [21.9-22.2 min]

CID-MS3

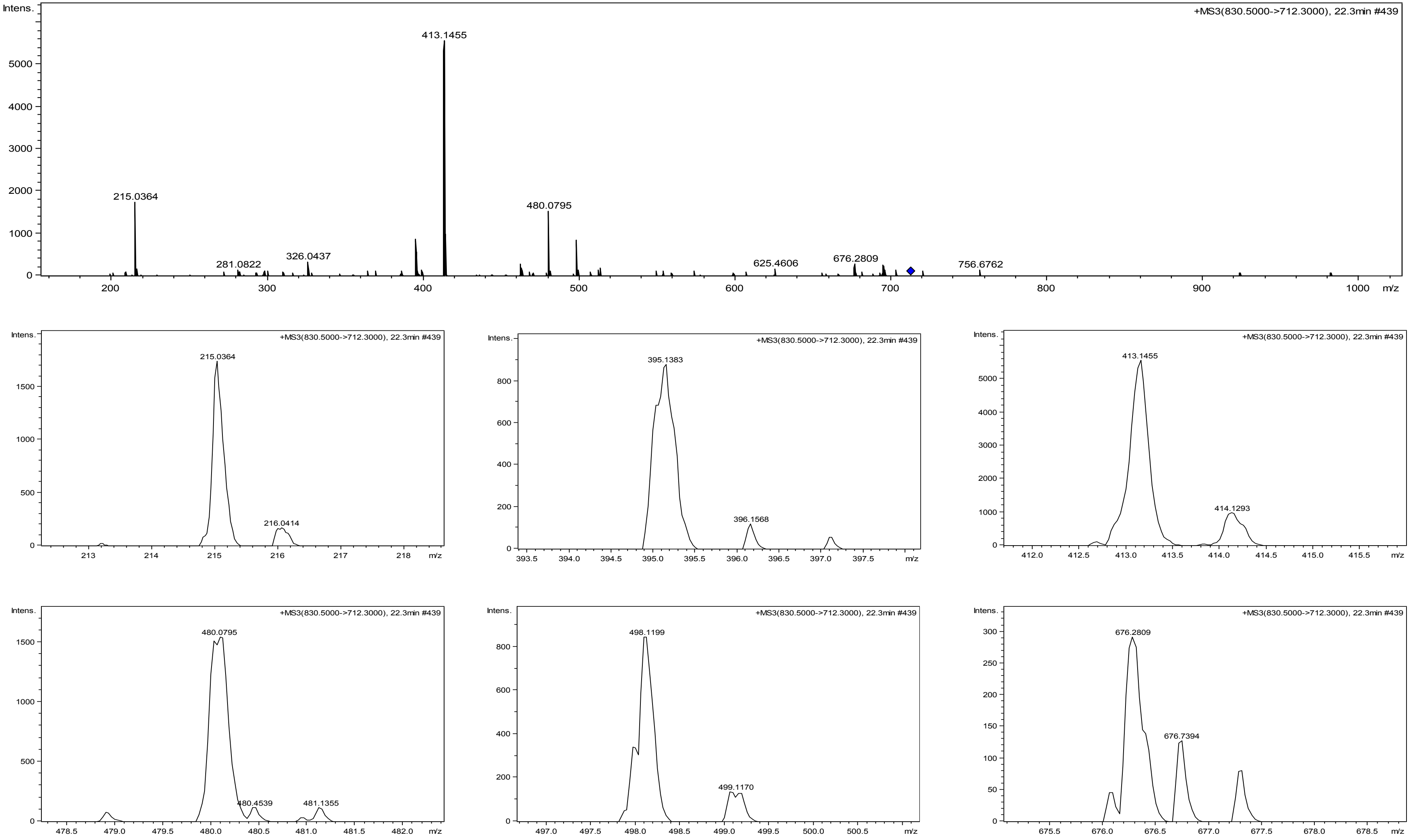

Fraction 16

830.34++ → Pep [M+H]+ 712.32+ [21.9-22.2 min] CID-MS3

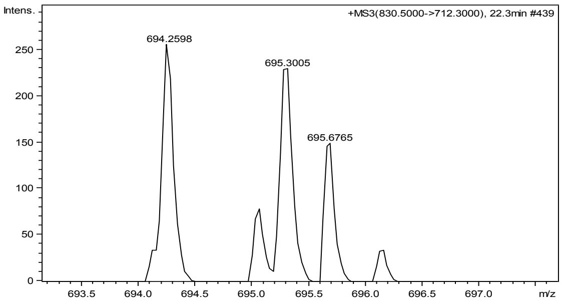

Fraction 16

830.34++ → Pep [M+H]+ 712.32+ [21.9-22.2 min] CID-MS3 MASCOT Search

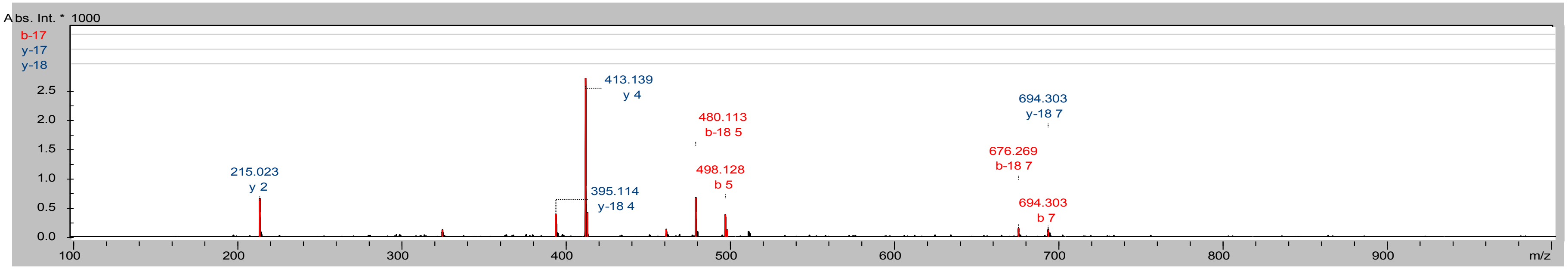

	E A V P T P V	Glu	Ala	Val	Pro	Thr	Pro	Val
Ion	1 2 3 4 5 6 7	1	2	3	4	5	6	7
b	E A V P T P V	130.050	201.087	300.155	397.208	498.256	595.309	694.377
b-17	E A V P T P V	-	-	-	-	-	-	-
b-18	E A V P T P V	112.039	183.076	282.145	379.198	480.245	577.298	676.366
y	E A V P T P V	118.086	215.139	316.187	413.239	512.308	583.345	712.388
y-17	E A V P T P V	-	-	-	-	-	-	-
y-18	E A V P T P V	-	-	298.176	395.229	494.297	565.334	694.377
	7 6 5 4 3 2 1	Val	Pro	Thr	Pro	Val	Ala	Glu

known O-glycosylation site

Alpha-2-HS-glycoprotein

$_{266}$EAVP**T**PV$_{272}$

Fraction 16

830.34++ → Pep [M+H]+ 712.32+ [21.9-22.2 min] CID-MS3 MASCOT Search

prot_hit_nur	prot_acc	prot_desc	prot_score	prot_mass	prot_matche	pep_query	pep_rank	pep_isbold	pep_exp_mz	pep_exp_mr	pep_exp_z	pep_calc_mr	pep_delta	pep_miss	pep_score	pep_expect	pep_res_bef	pep_seq
1	FETUA_HUM	Alpha-2-HS-g	12	40098	1	1	1	1	712.3221	711.3148	1	711.3803	-0.0655	0	16.09	2.40E+02	N	EAVPTPV
2	SRR_HUMAN	Serine racem	11	36999	1	1	1	0	712.3221	711.3148	1	711.4167	-0.1019	0	16.09	2.40E+02	V	DALVVPV
3	CT2NL_HUM	CTTNBP2 N-t	11	70571	1	1	1	0	712.3221	711.3148	1	711.3803	-0.0655	0	16.09	2.40E+02	V	GIETPVP
4	VGFR2_HUM	Vascular end	11	153312	1	1	1	0	712.3221	711.3148	1	711.3803	-0.0655	0	16.09	2.40E+02	V	GELPTPV
5	TBX22_HUM	T-box transc	11	58387	1	1	1	0	712.3221	711.3148	1	711.4167	-0.1019	0	16.09	2.40E+02	V	AIDVVPV
6	PACS2_HUM	Phosphofuri	11	98440	1	1	1	0	712.3221	711.3148	1	711.3803	-0.0655	0	16.09	2.40E+02	G	ALNTPVP
7	USBP1_HUM	USH1C-bindi	10	76364	1	1	1	0	712.3221	711.3148	1	711.3625	-0.0477	0	16.09	2.40E+02	L	APMPTVP
8	ACINU_HUM	Apoptotic ch	10	152196	1	1	1	0	712.3221	711.3148	1	711.3803	-0.0655	0	16.09	2.40E+02	Q	EVATPPV
9	CN043_HUM	Uncharacteri	10	115431	1	1	1	0	712.3221	711.3148	1	711.3803	-0.0655	0	16.09	2.40E+02	S	AEVTPPV
10	WNK2_HUM	Serine/threc	8	244293	1	1	1	0	712.3221	711.3148	1	711.3803	-0.0655	0	16.09	2.40E+02	A	AQVPTVP

Biotools-Score: 2

MASCOT-Score: 16

known O-glycosylation site

Alpha-2-HS-glycoprotein

$_{266}$EAVP**T**PV$_{272}$

Fraction 16

830.34++ → Pep [M+H]+ 712.32+ [21.9-22.2 min] ETD

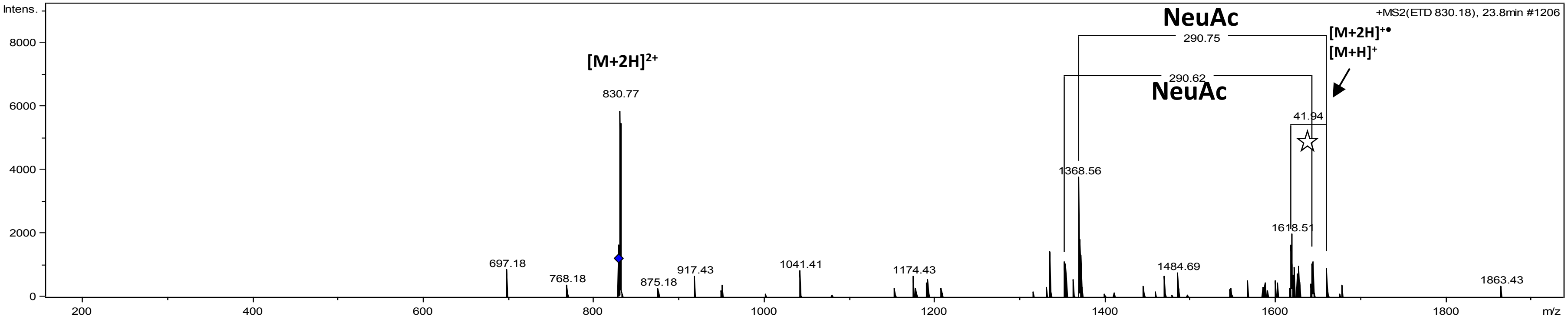

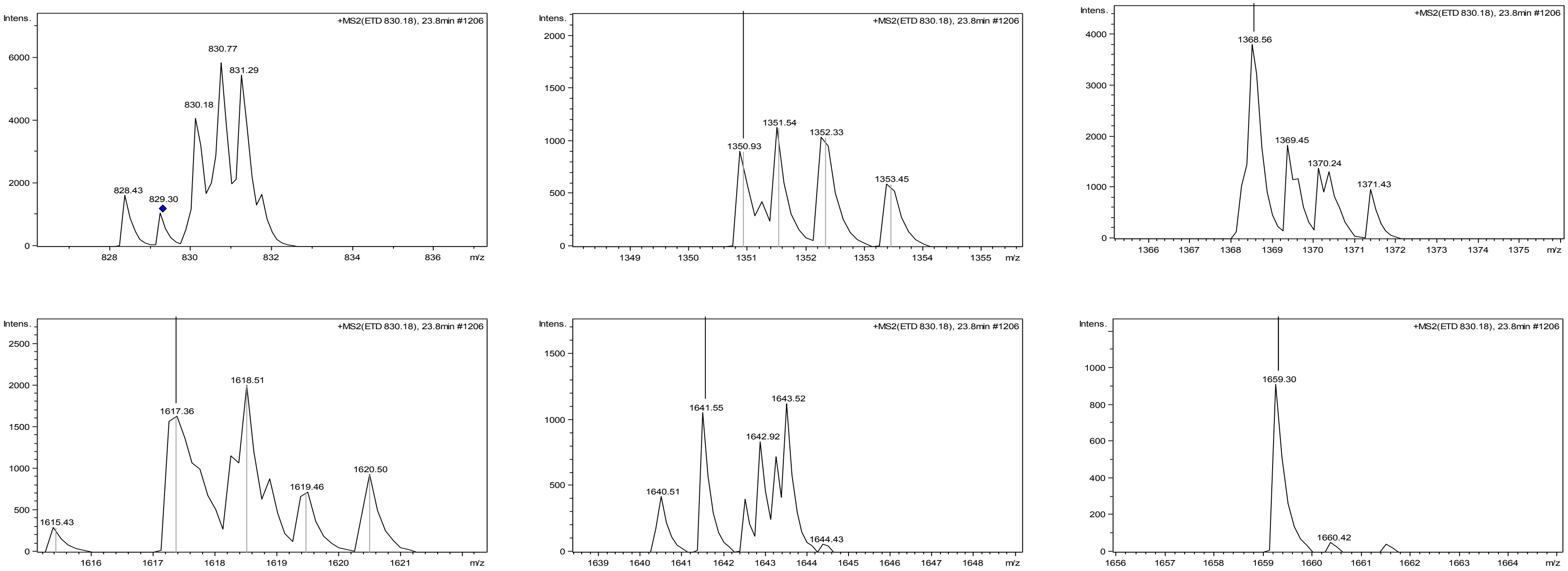

Fraction 16

830.34++ → Pep [M+H]+ 712.32+ [21.9-22.2 min] ETD

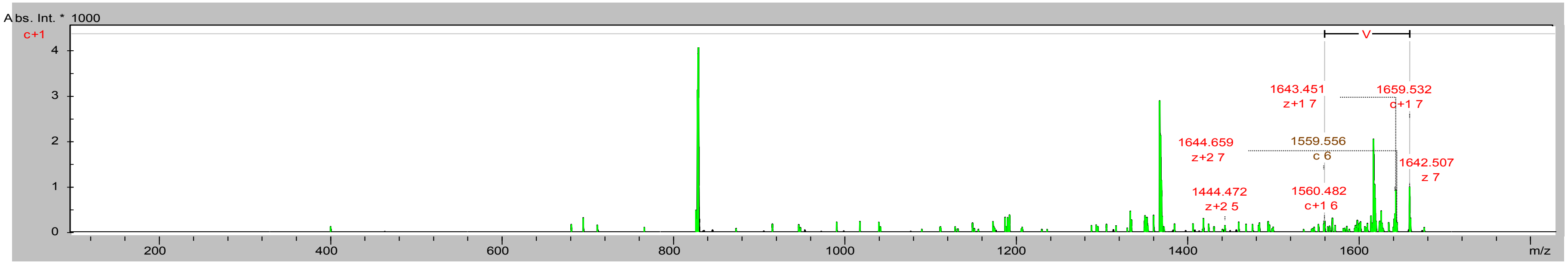

	E	A	V	P	T	P	V	Glu	Ala	Val	Pro	Thr	Pro	Val
Ion	1	2	3	4	5	6	7	1	2	3	4	5	6	7
c	E	A	V	P	T*	P	V	147.076	218.114	317.182	414.235	1462.605	1559.658	1658.727
c+1	E	A	V	P	T*	P	V	148.084	219.121	318.190	415.243	1463.613	1560.666	1659.734
z	E	A	V	P	T*	P	V	101.060	198.112	1246.483	1343.536	1442.604	1513.641	1642.684
z+1	E	A	V	P	T*	P	V	102.068	199.120	1247.491	1344.544	1443.612	1514.649	1643.692
z+2	E	A	V	P	T*	P	V	103.075	200.128	1248.499	1345.552	1444.620	1515.657	1644.700
	7	6	5	4	3	2	1	Val	Pro	Thr	Pro	Val	Ala	Glu

Biotools-Score: 7

known O-glycosylation site

Alpha-2-HS-glycoprotein

$_{266}$EAVP**T**PV$_{272}$

Fraction 16

833.30++ → Pep [M+H]+ 1009.43+ [22.1-22.2 min] CID-MS Precursor

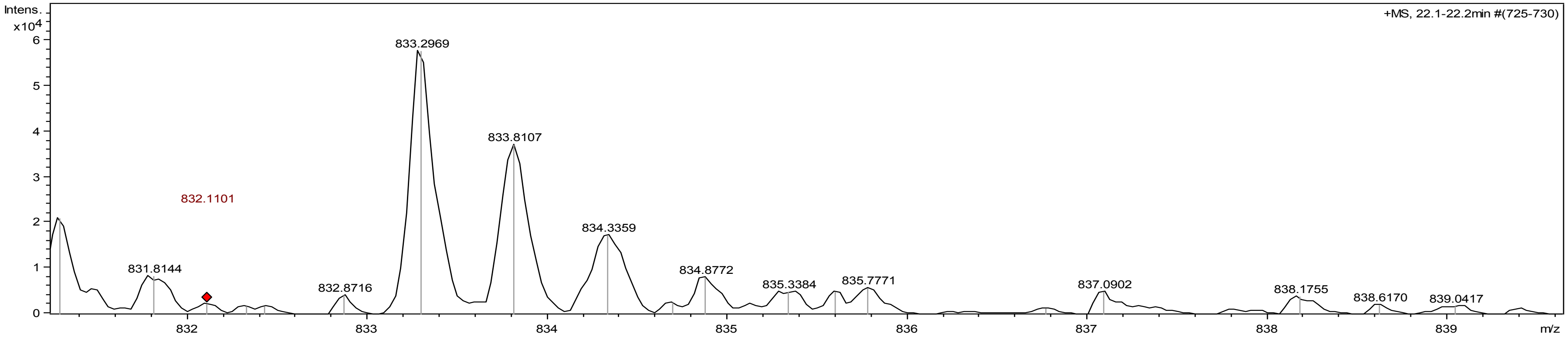

ETD spectrum of poor quality

Fraction 16

833.30++ → Pep [M+H]+ 1009.43+ [22.1-22.2 min] CID-MS2

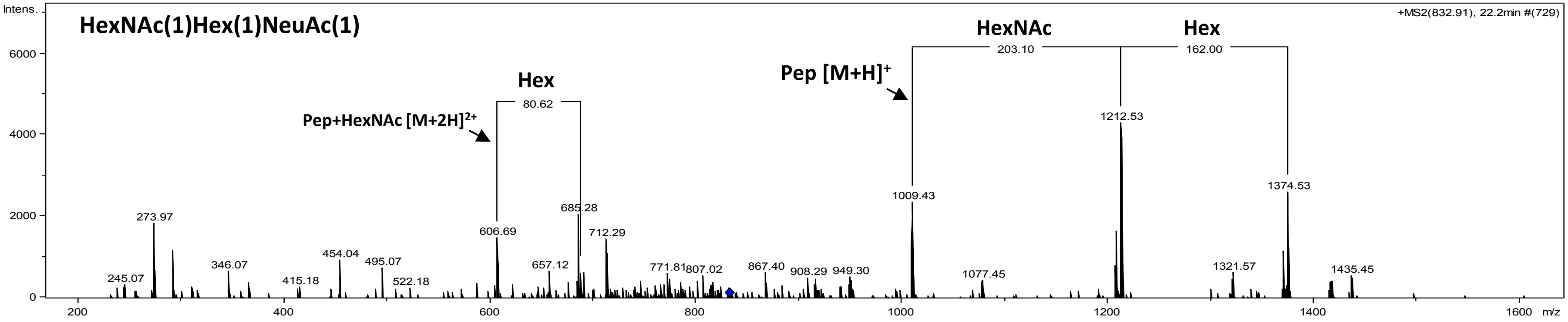

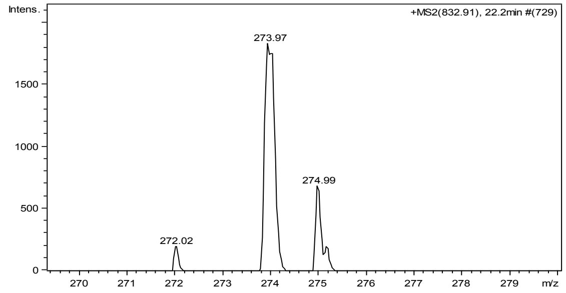

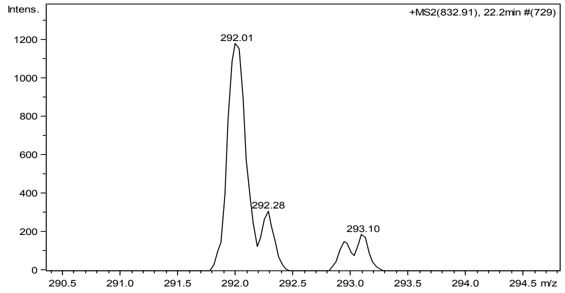

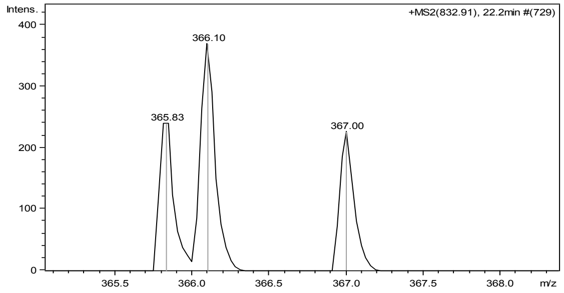

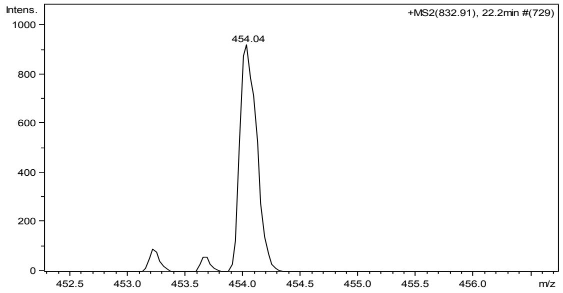

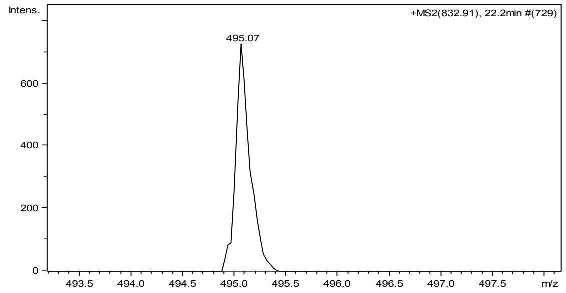

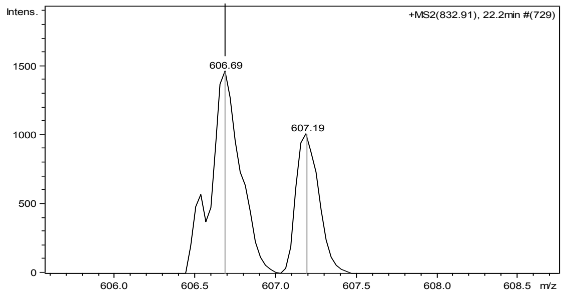

Fraction 16

833.30++ → Pep [M+H]+ 1009.43+ [22.1-22.2 min]

CID-MS2

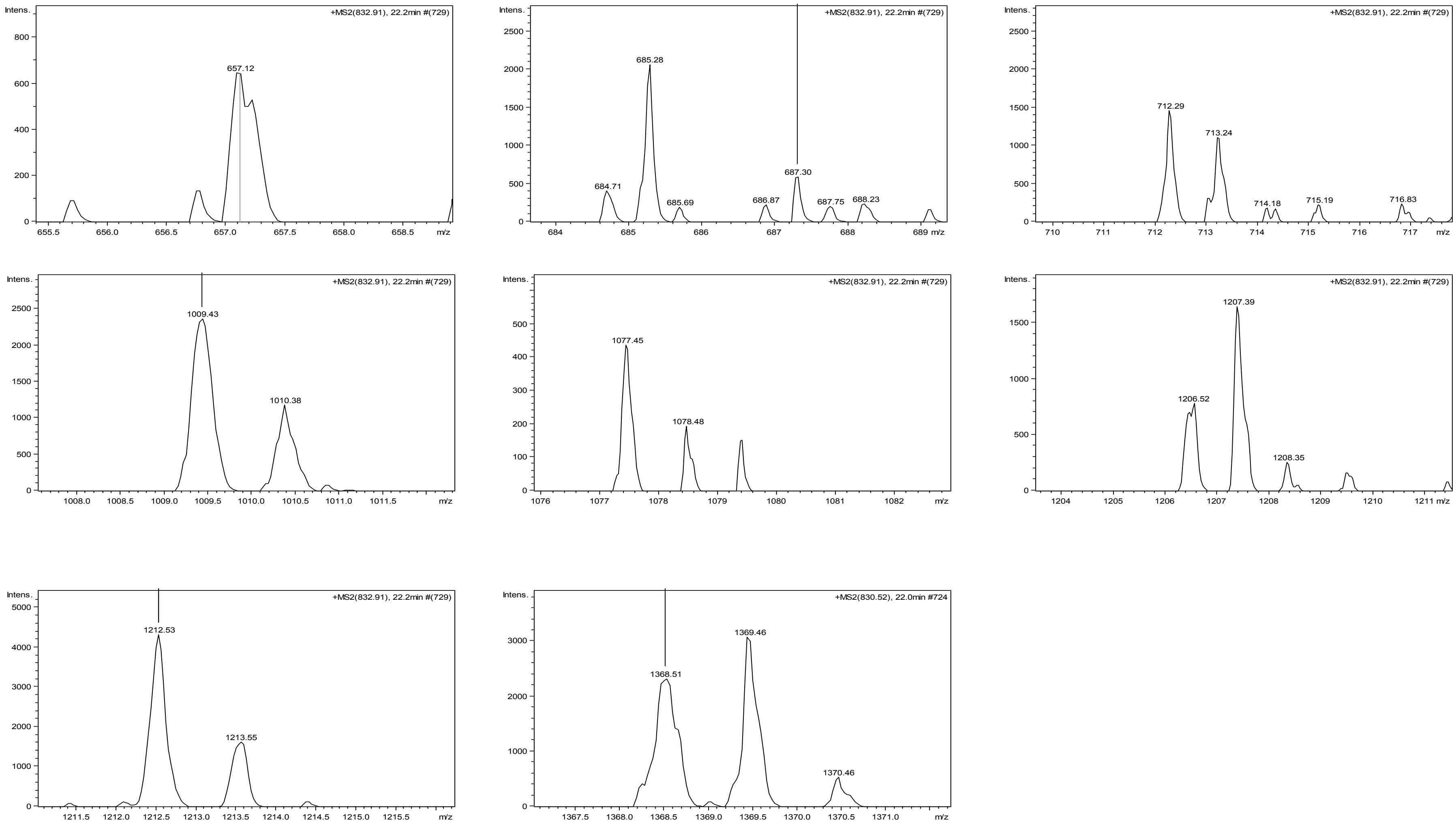

Fraction 16

833.30++ → Pep [M+H]+ 1009.43+ [22.1-22.2 min] CID-MS3

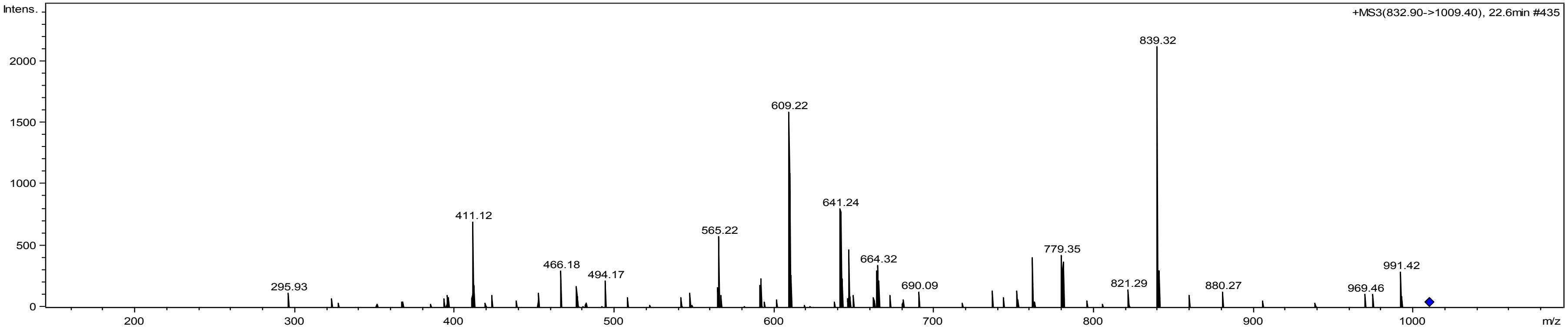

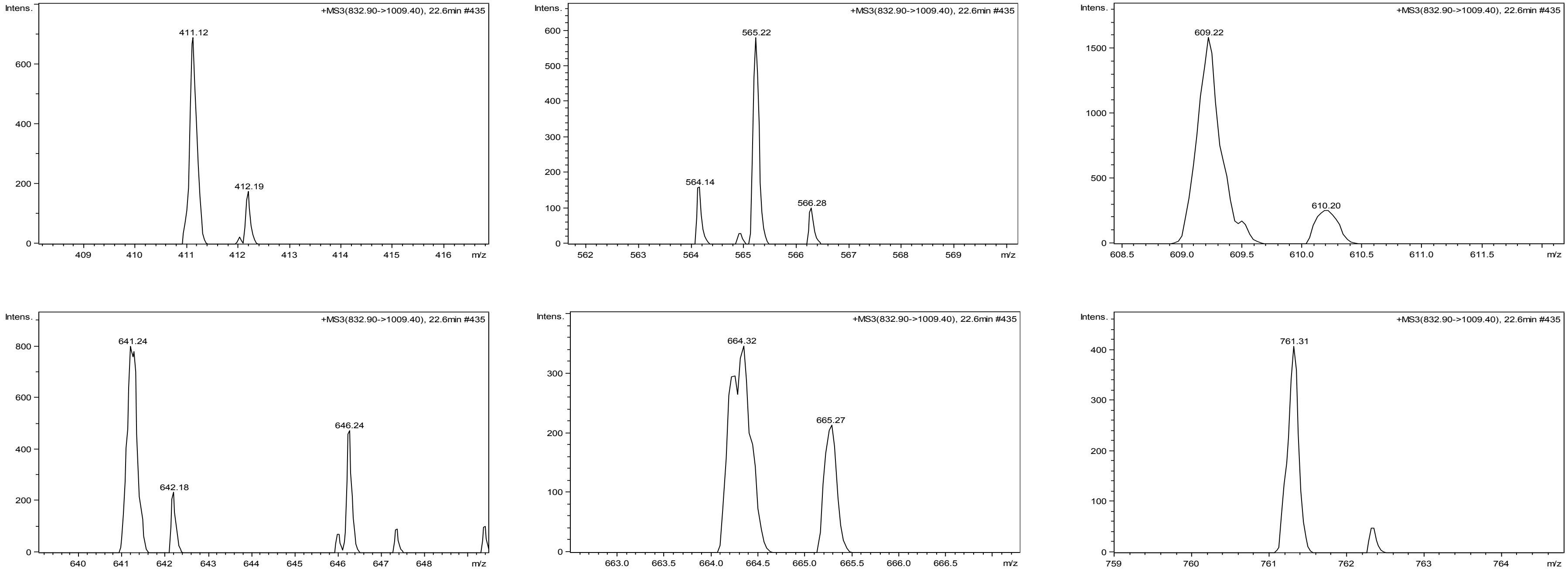

Fraction 16

833.30++ → Pep [M+H]+ 1009.43+ [22.1-22.2 min]

CID-MS3

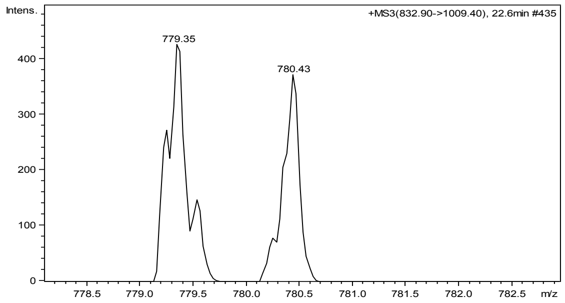

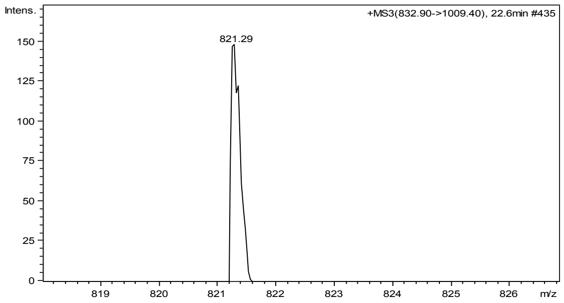

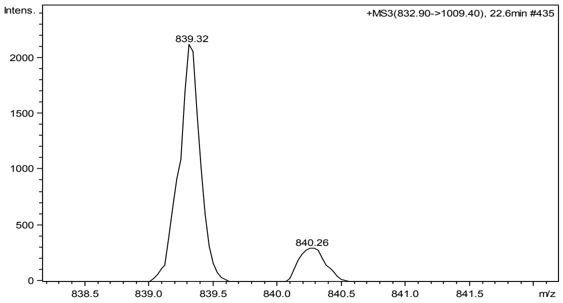

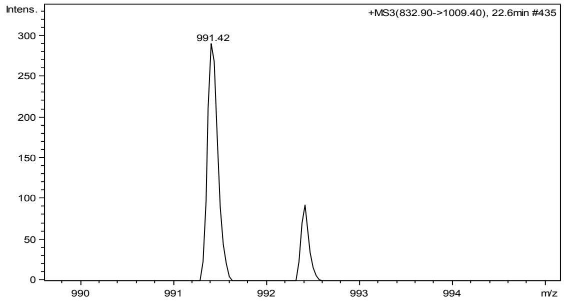

Fraction 16

833.30++ → Pep [M+H]+ 1009.43+ [22.1-22.2 min]

CID-MS3 MASCOT Search

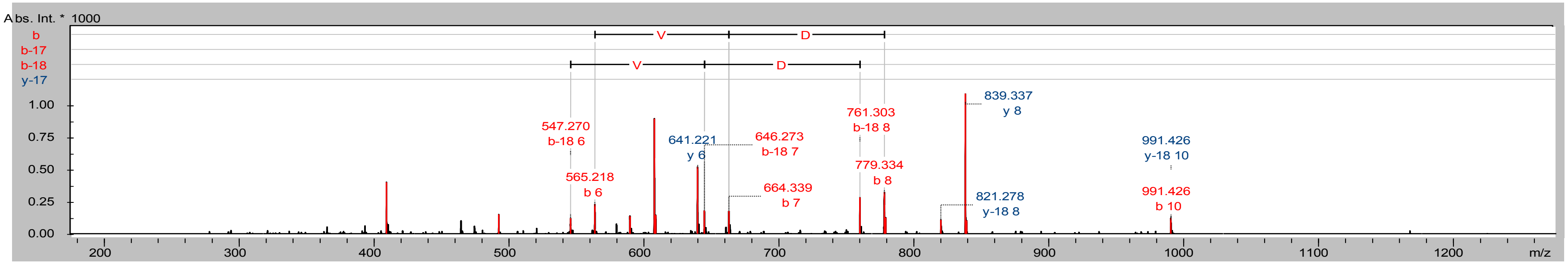

	A	V	P	T	P	V	V	D	P	D	Ala	Val	Pro	Thr	Pro	Val	Val	Asp	Pro	Asp
Ion	1	2	3	4	5	6	7	8	9	10	1	2	3	4	5	6	7	8	9	10
b	A	V	P	T	P	V	V	D	P	D	72.044	171.113	268.166	369.213	466.266	565.334	664.403	779.430	876.483	991.509
b-17	A	V	P	T	P	V	V	D	P	D	-	-	-	-	-	-	-	-	-	-
b-18	A	V	P	T	P	V	V	D	P	D	-	-	-	351.203	448.255	547.324	646.392	761.419	858.472	973.499
y	A	V	P	T	P	V	V	D	P	D	134.045	231.098	346.124	445.193	544.261	641.314	742.362	839.415	938.483	1009.520
y-17	A	V	P	T	P	V	V	D	P	D	-	-	-	-	-	-	-	-	-	-
y-18	A	V	P	T	P	V	V	D	P	D	116.034	213.087	328.114	427.182	526.251	623.304	724.351	821.404	920.472	991.509
	10	9	8	7	6	5	4	3	2	1	Asp	Pro	Asp	Val	Val	Pro	Thr	Pro	Val	Ala

known O-glycosylation site

Alpha-2-HS-glycoprotein

$_{267}$AVP**T**PVVDPD$_{276}$

Fraction 16

833.30++ → Pep [M+H]+ 1009.43+ [22.1-22.2 min]

CID-MS3 MASCOT Search

prot_hit_nur	prot_acc	prot_desc	prot_score	prot_mass	prot_matche	pep_query	pep_rank	pep_isbold	pep_exp_mz	pep_exp_mr	pep_exp_z	pep_calc_mr	pep_delta	pep_miss	pep_score	pep_expect	pep_res_bef	pep_seq
1	SYLM_HUMA	Probable leu	20	102823	1	1	1	1	1009.4283	1008.421	1	1007.4668	0.9543	0	26.41	34	C	LTPVMAVNN
2	FETUA_HUM	Alpha-2-HS-g	16	40098	1	1	3	0	1009.4283	1008.421	1	1008.5128	-0.0918	0	19.46	1.70E+02	E	AVPTPVVDP
3	ZN416_HUM	Zinc finger p	14	69425	1	1	2	0	1009.4283	1008.421	1	1007.4019	1.0192	0	19.89	1.50E+02	F	HESGMPFTS
4	TACD2_HUM	Tumor-assoc	13	36371	1	1	4	0	1009.4283	1008.421	1	1008.6947	-0.2737	0	17.52	2.60E+02	A	VIVVVVVALV
5	SL9A1_HUM	Sodium/hyd	12	91218	1	1	4	0	1009.4283	1008.421	1	1008.6583	-0.2373	0	17.52	2.60E+02	F	PSLLVVVALV
6	EPHA5_HUM	Ephrin type-	11	116706	1	1	4	0	1009.4283	1008.421	1	1008.6947	-0.2737	0	17.52	2.60E+02	V	ILLAVVIGVL
7	TENR_HUMA	Tenascin-R p	9	151791	1	1	7	0	1009.4283	1008.421	1	1007.3866	1.0344	0	17.05	2.90E+02	R	DNDVAVTNC
8	MY18B_HUM	Myosin-XVIII	9	287175	1	1	7	0	1009.4283	1008.421	1	1008.4434	-0.0224	0	17.05	2.90E+02	K	DDDVASIMK
9	BSN_HUMAN	Protein bass	7	418354	1	1	10	0	1009.4283	1008.421	1	1007.4117	1.0093	0	16.04	3.70E+02	G	QTPMPTTQS

Biotools-Score: 8

MASCOT-Score: 19

known O-glycosylation site

Alpha-2-HS-glycoprotein

$_{267}$AVP**T**PVVDPD$_{276}$

Fraction 16

833.30++ → Pep [M+H]+ 1009.43+ [22.1-22.2 min] ETD

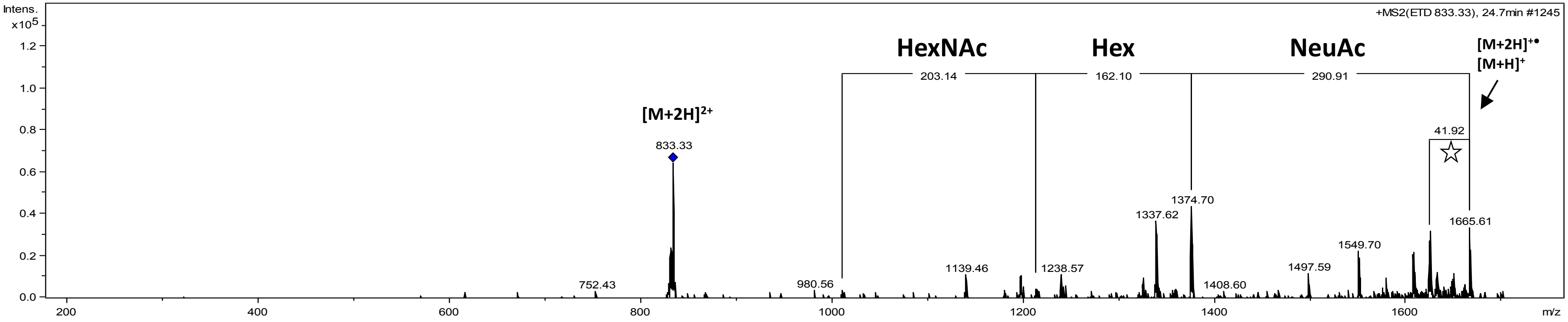

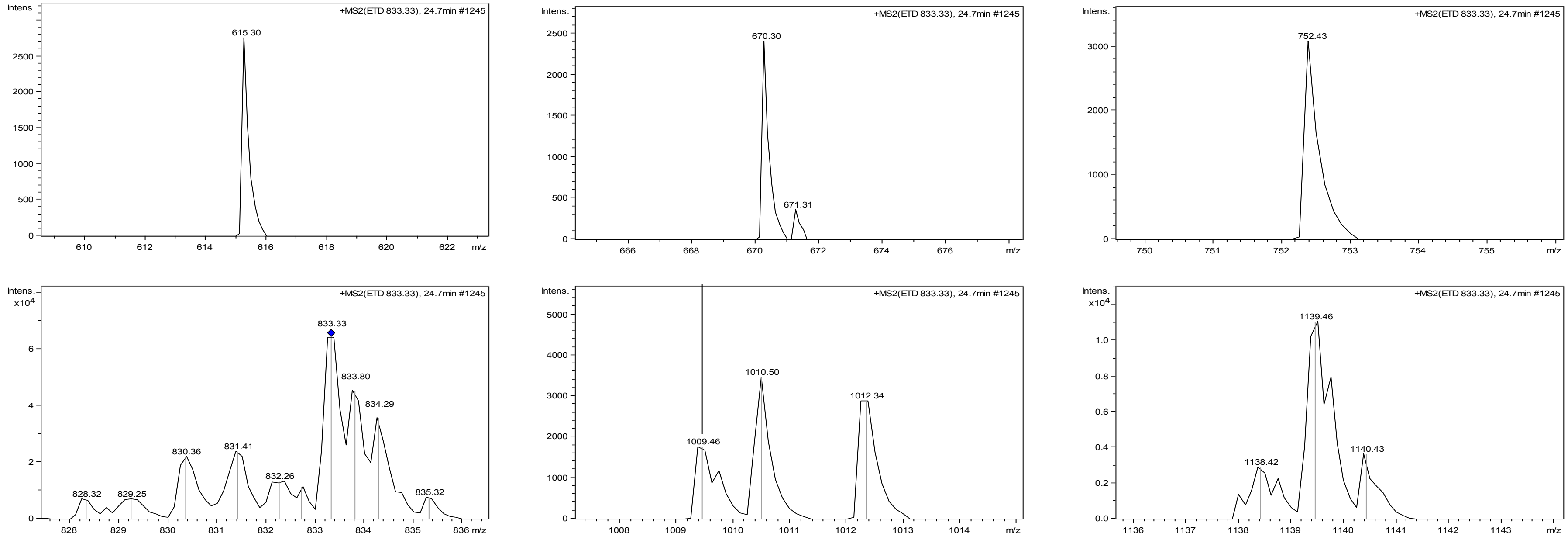

Fraction 16

833.30++ → Pep [M+H]+ 1009.43+ [22.1-22.2 min] ETD

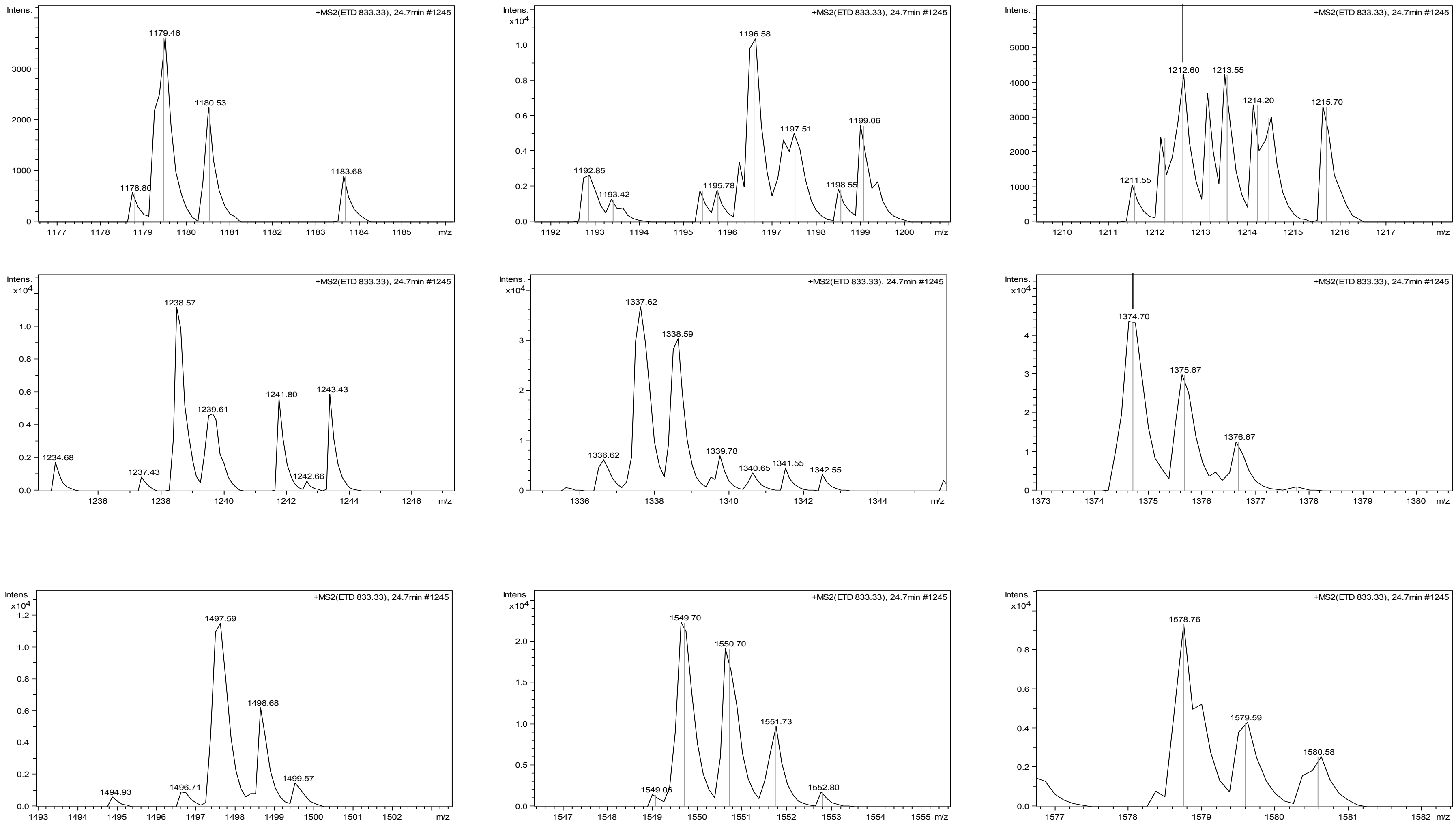

Fraction 16

833.30++ → Pep [M+H]+ 1009.43+ [22.1-22.2 min] ETD

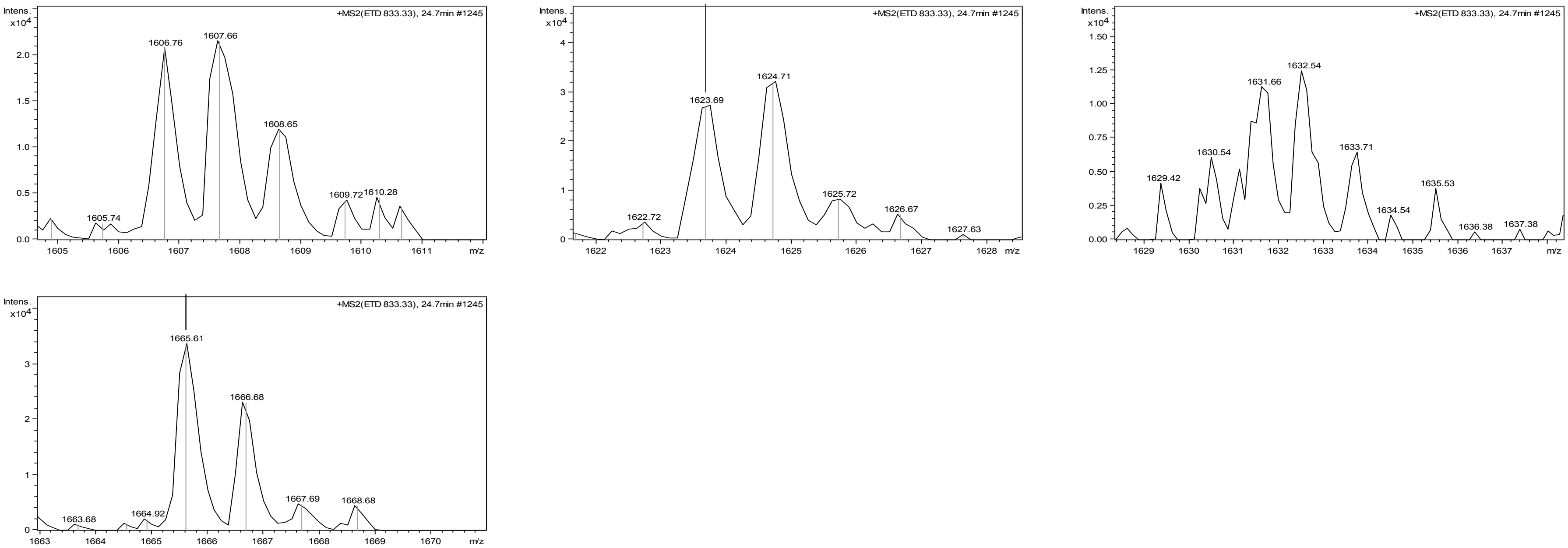

Fraction 16

833.30++ → Pep [M+H]+ 1009.43+ [22.1-22.2 min] ETD

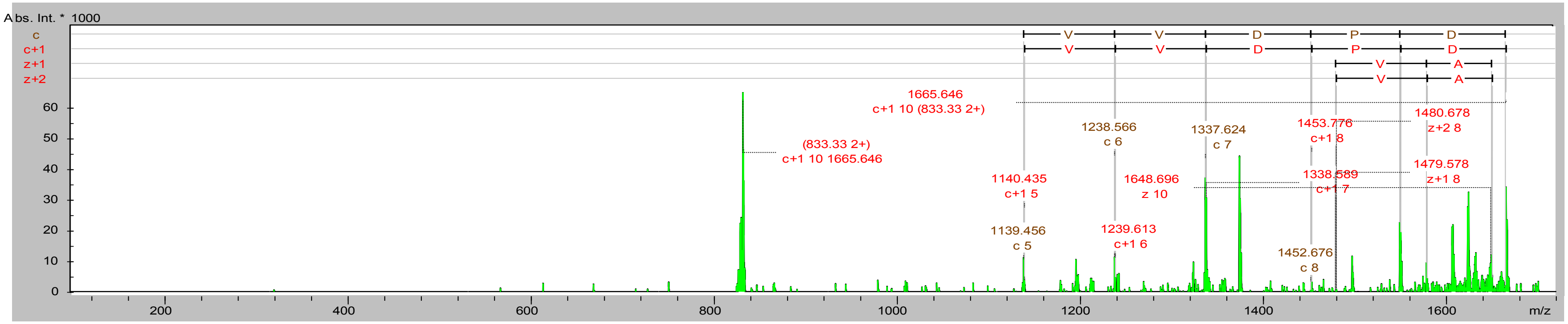

	A	V	P	T	P	V	V	D	P	D	Ala	Val	Pro	Thr	Pro	Val	Val	Asp	Pro	Asp
Ion	1	2	3	4	5	6	7	8	9	10	1	2	3	4	5	6	7	8	9	10
c	A	V	P	T*	P	V	V	D	P	D	89.071	188.139	285.192	1042.467	1139.520	1238.589	1337.657	1452.684	1549.737	1664.764
c+1	A	V	P	T*	P	V	V	D	P	D	90.079	189.147	286.200	1043.475	1140.528	1239.596	1338.665	1453.692	1550.745	1665.771
z	A	V	P	T*	P	V	V	D	P	D	117.018	214.071	329.098	428.166	527.235	624.288	1381.563	1478.616	1577.684	1648.721
z+1	A	V	P	T*	P	V	V	D	P	D	118.026	215.079	330.106	429.174	528.243	625.295	1382.571	1479.623	1578.692	1649.729
z+2	A	V	P	T*	P	V	V	D	P	D	119.034	216.087	331.114	430.182	529.250	626.303	1383.578	1480.631	1579.700	1650.737
	10	9	8	7	6	5	4	3	2	1	Asp	Pro	Asp	Val	Val	Pro	Thr	Pro	Val	Ala

Biotools-Score: 141

known O-glycosylation site

Alpha-2-HS-glycoprotein

$_{267}$AVPTPVVDPD$_{276}$

Fraction 16

706.27+++ → Pep [M+2H]++ 731.36++ [23.3-23.5 min]

CID-MS Precursor

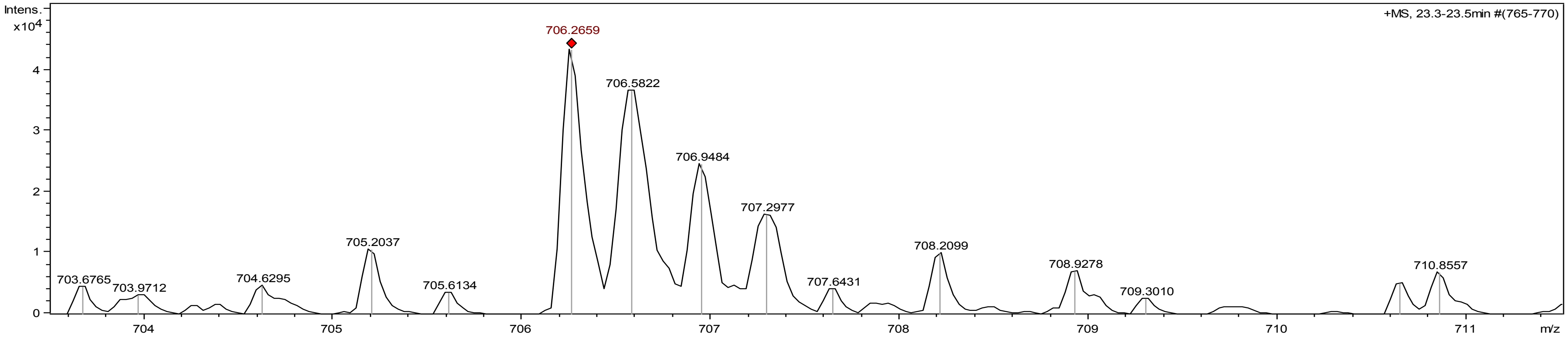

Fraction 16

706.27+++ → Pep [M+2H]++ 731.36++ [23.3-23.5 min] CID-MS2

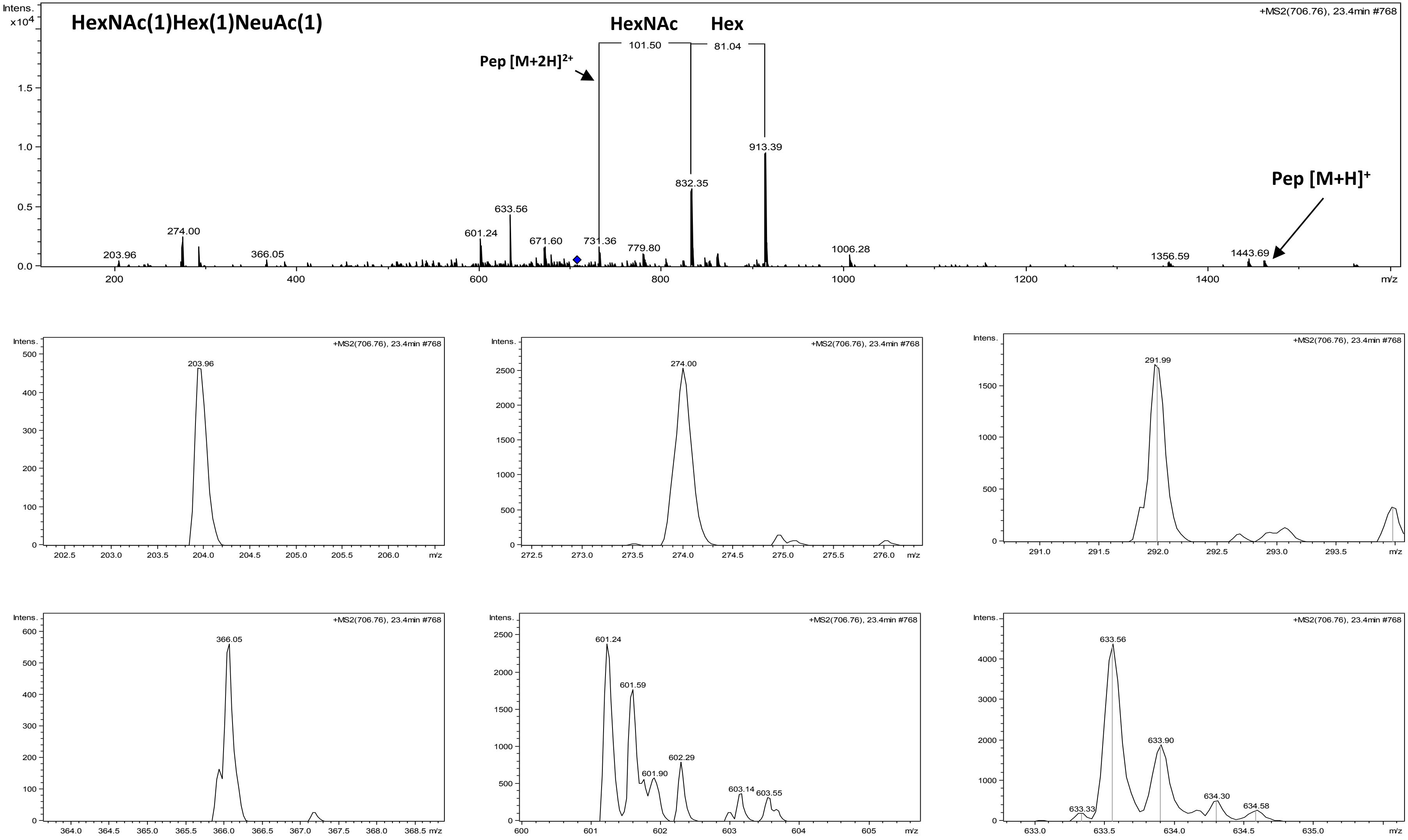

Fraction 16

706.27+++ → Pep [M+2H]++ 731.36++ [23.3-23.5 min] CID-MS2

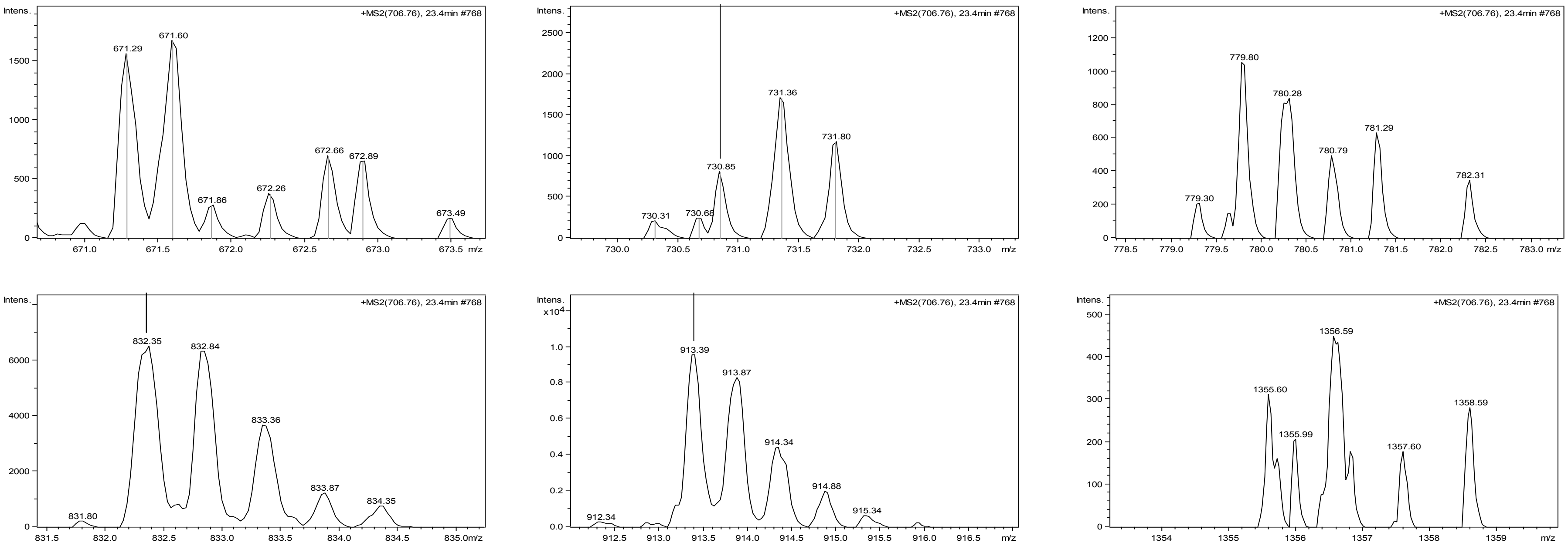

Fraction 16

706.27+++ → Pep [M+2H]++ 731.36++ [23.3-23.5 min]

CID-MS3 MASCOT Search

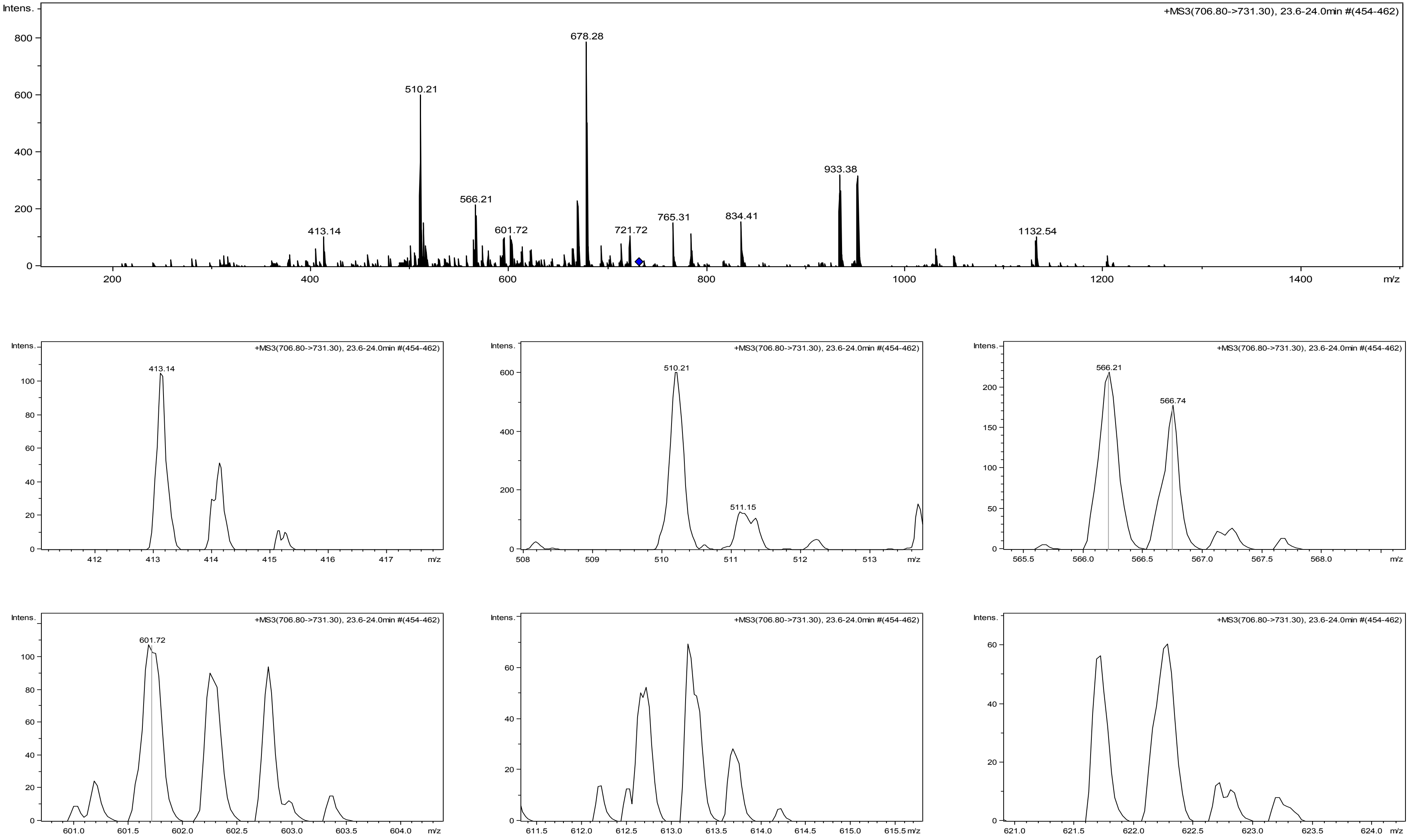

Fraction 16

706.27+++ → Pep [M+2H]++ 731.36++ [23.3-23.5 min]

CID-MS3 MASCOT Search

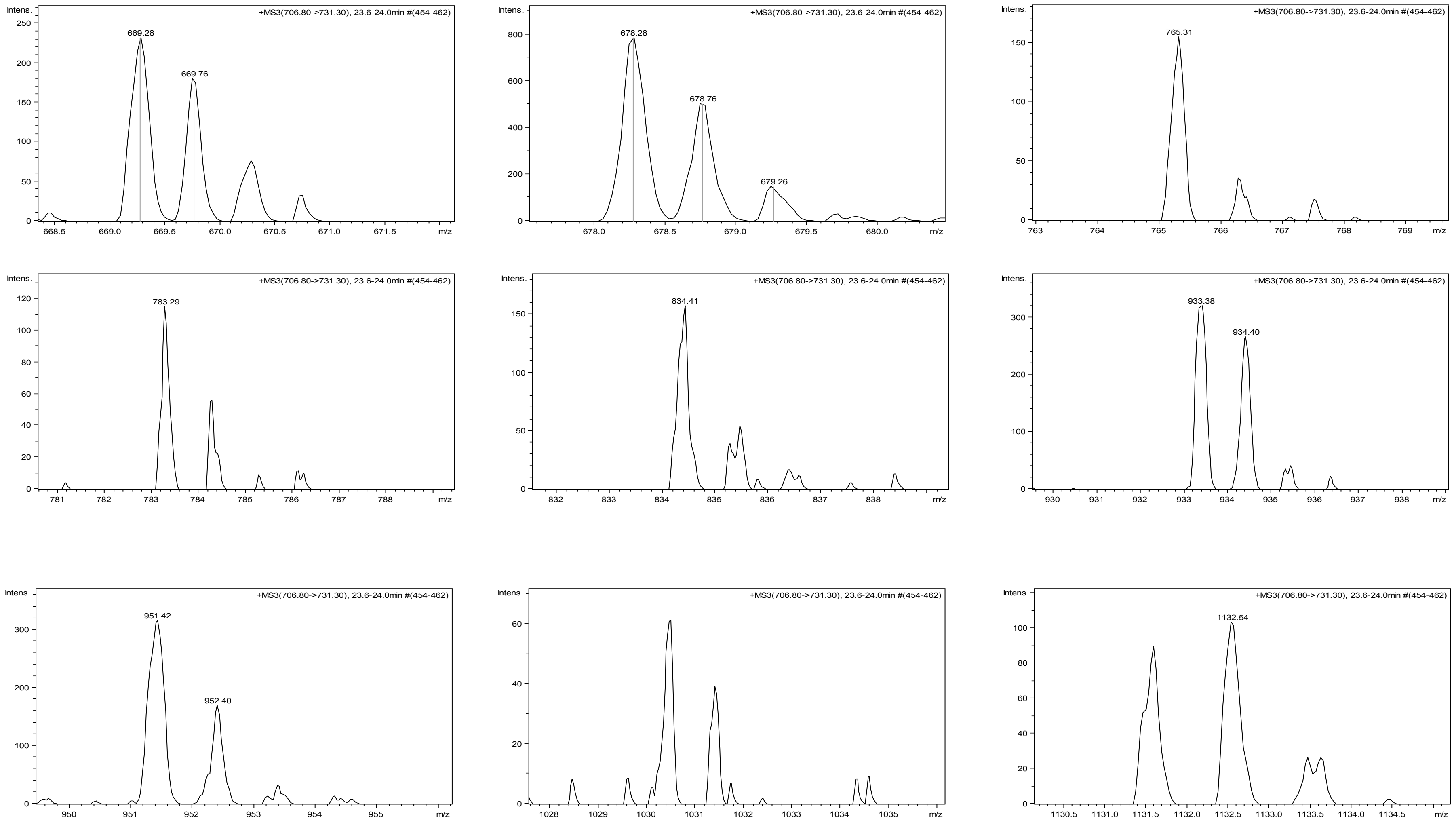

Fraction 16

706.27+++ → Pep [M+2H]++ 731.36++ [23.3-23.5 min]

CID-MS3 MASCOT Search

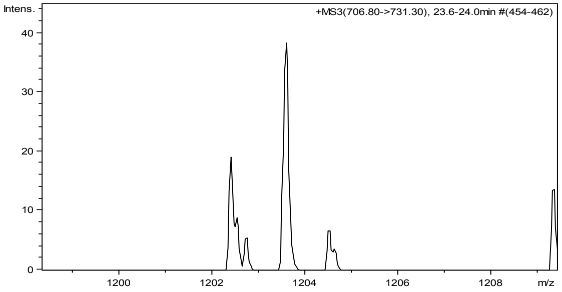

Fraction 16

706.27+++ → Pep [M+2H]++ 731.36++ [23.3-23.5 min]

CID-MS3 MASCOT Search

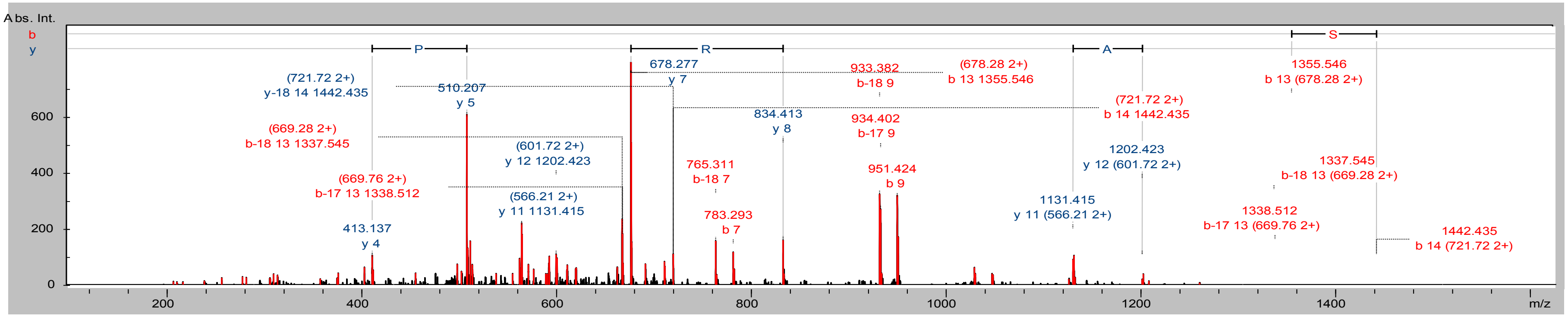

	E	E	A	P	S	L	R	P	A	P	P	P	I	S
Ion	1	2	3	4	5	6	7	8	9	10	11	12	13	14
b	E	E	A	P	S	L	R	P	A	P	P	P	I	S
b-17	E	E	A	P	S	L	R	P	A	P	P	P	I	S
b-18	E	E	A	P	S	L	R	P	A	P	P	P	I	S
y	E	E	A	P	S	L	R	P	A	P	P	P	I	S
y-17	E	E	A	P	S	L	R	P	A	P	P	P	I	S
y-18	E	E	A	P	S	L	R	P	A	P	P	P	I	S
	14	13	12	11	10	9	8	7	6	5	4	3	2	1

	Glu	Glu	Ala	Pro	Ser	Leu	Arg	Pro	Ala	Pro	Pro	Pro	Ile	Ser
Ion	1	2	3	4	5	6	7	8	9	10	11	12	13	14
b	130.050	259.092	330.130	427.182	514.214	627.298	783.400	880.452	951.489	1048.542	1145.595	1242.648	1355.732	1442.764
b-17	-	-	-	-	-	-	766.373	863.426	934.463	1031.516	1128.568	1225.621	1338.705	1425.737
b-18	112.039	241.082	312.119	409.172	496.204	609.288	765.389	862.442	933.479	1030.532	1127.584	1224.637	1337.721	1424.753
y	106.050	219.134	316.187	413.239	510.292	581.329	678.382	834.483	947.567	1034.599	1131.652	1202.689	1331.732	1460.774
y-17	-	-	-	-	-	-	-	817.457	930.541	1017.573	1114.626	1185.663	1314.705	1443.748
y-18	88.039	201.123	298.176	395.229	492.282	563.319	660.372	816.473	929.557	1016.589	1113.642	1184.679	1313.721	1442.764
	Ser	Ile	Pro	Pro	Pro	Ala	Pro	Arg	Leu	Ser	Pro	Ala	Glu	Glu

known O-glycosylation region

Also found in Zauner et al., 2012

Fibrinogen beta chain precursor

$_{54}$EEAP**S**LRPAPPPI**S**$_{67}$

Fraction 16

706.27+++ → Pep [M+2H]++ 731.36++ [23.3-23.5 min]

CID-MS3 MASCOT Search

prot_hit_nur	prot_acc	prot_desc	prot_score	prot_mass	prot_	prot_	prot_	prot_	pep_	pep_	pep_	pep_	pep_exp_mz	pep_exp_mr	pep_e	pep_calc_mr	pep_delta	pep_n	pep_s	pep_expect	pep	pep_seq
1	FIBB_HUMAN	Fibrinogen b	15	56577	1	0	1	0	1	1	1	1	730,84	1459,6654	2	1459,7671	-0,1016	0	21,71	1,70E+02	R	EEAPSLRPAPPPIS
2	CENPI_HUMA	Centromere	14	87748	1	0	1	0	1	3	0	1	730,84	1459,6654	2	1459,7341	-0,0686	0	20,02	2,60E+02	T	TLGGSMNSVSKLIH
3	PPRC1_HUMA	Peroxisome	13	178917	1	0	1	0	1	2	0	1	730,84	1459,6654	2	1459,7559	-0,0904	0	21,02	2,00E+02	P	SPPVQSVSPAVPTPP
4	PRR22_HUMA	Proline-rich	10	44351	1	0	1	0	1	10	0	1	730,84	1459,6654	2	1458,7692	0,8963	0	13,47	1,20E+03	A	RQPAGPASATPPGPR
5	NPNT_HUMA	Nephronecti	9	64033	1	0	1	0	1	7	0	1	730,84	1459,6654	2	1459,865	-0,1995	0	15,58	7,10E+02	L	LALVLVSSLYLQAA
6	STOX1_HUMA	Storkhead-b	8	111862	1	0	1	0	1	6	0	1	730,84	1459,6654	2	1458,6297	1,0358	0	15,69	6,90E+02	E	YNSTMERVESQV
7	HD_HUMAN	Huntingtin O	7	351374	1	0	1	0	1	4	0	1	730,84	1459,6654	2	1458,7831	0,8824	0	16,63	5,60E+02	H	DVLKATHANYKVT
8	RIM3C_HUMA	RIMS-binding	7	183232	1	0	1	0	1	8	0	1	730,84	1459,6654	2	1459,5521	0,1133	0	15,54	7,20E+02	T	SSQSNSSSEGSMWA
9	EMSY_HUMA	Protein EMSY	6	141780	1	0	1	0	1	9	0	1	730,84	1459,6654	2	1459,6501	0,0154	0	13,84	1,10E+03	G	STVYVKSVSCSDE

Biotools-Score: 14

MASCOT-Score: 22

known O-glycosylation region

Also found in Zauner et al., 2012

Fibrinogen beta chain precursor

$_{54}$EEAP**S**LRPAPPPI**S**$_{67}$

Fraction 16

706.27+++ → Pep [M+2H]++ 731.36++ [23.3-23.5 min] ETD

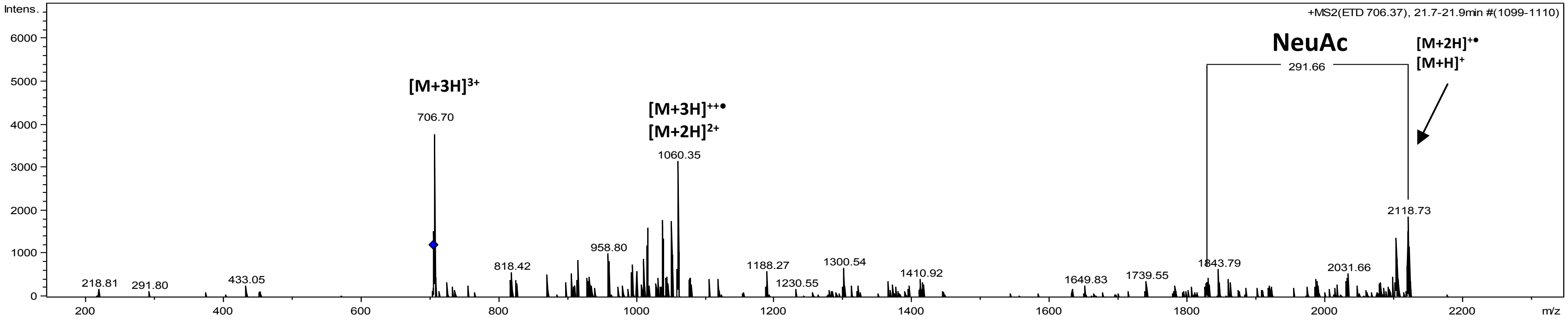

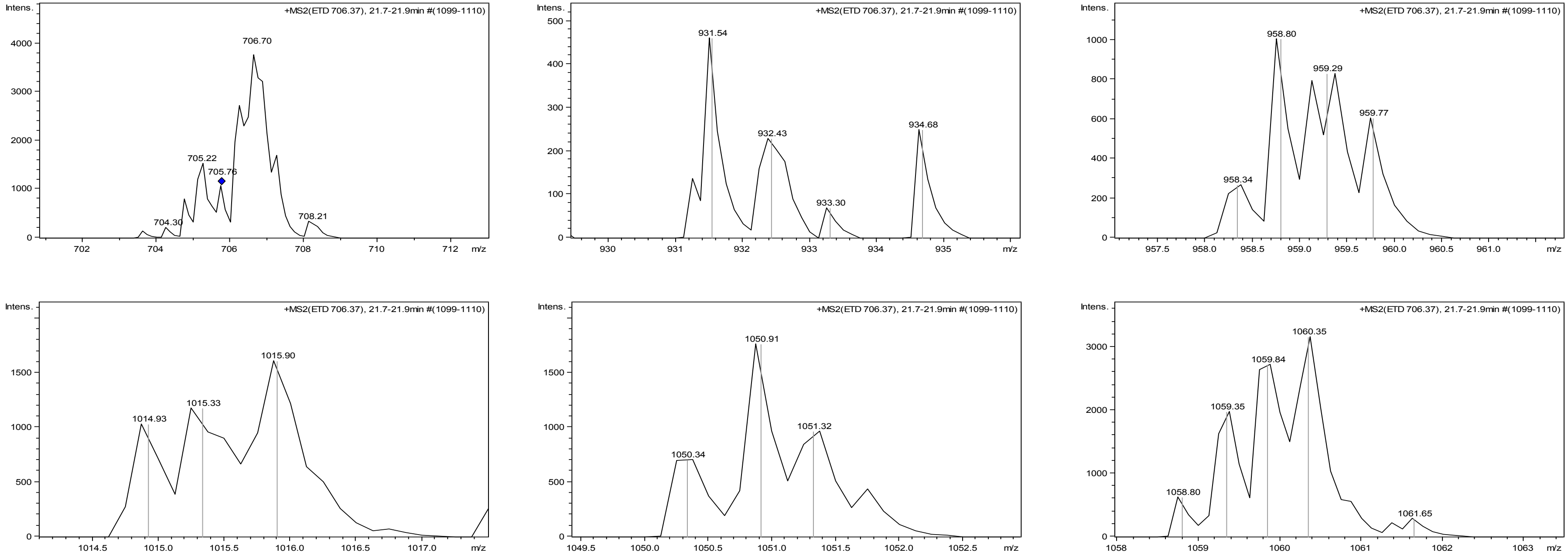

Fraction 16

706.27+++ → Pep [M+2H]++ 731.36++ [23.3-23.5 min]

ETD

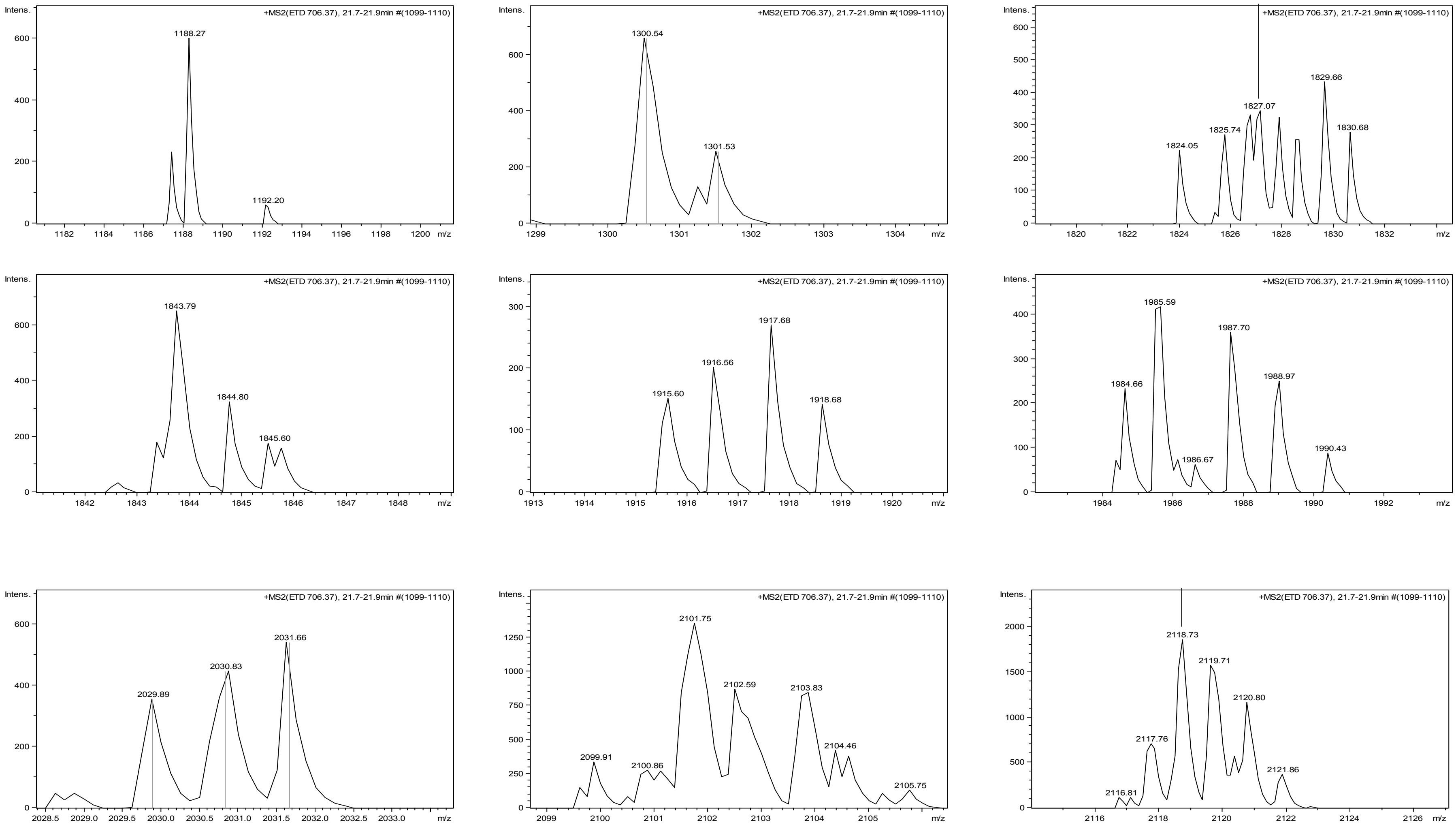

Fraction 16

706.27+++ → Pep [M+2H]++ 731.36++ [23.3-23.5 min] ETD

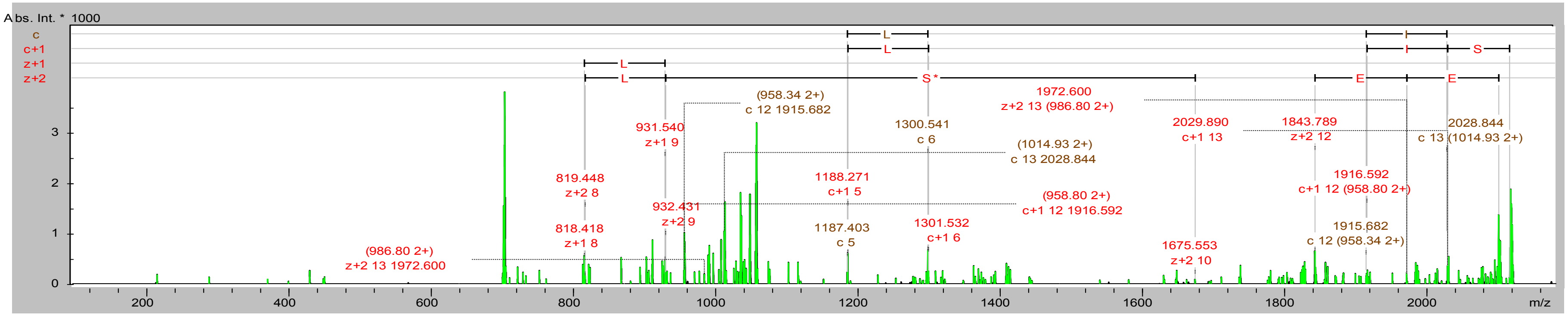

EEAPSLRPAPPPIS **Most likely**

	E	E	A	P	S	L	R	P	A	P	P	P	I	S	Glu	Glu	Ala	Pro	Ser	Leu	Arg	Pro	Ala	Pro	Pro	Pro	Ile	Ser
Ion	1	2	3	4	5	6	7	8	9	10	11	12	13	14	1	2	3	4	5	6	7	8	9	10	11	12	13	14
c	E	E	A	P	S*	L	R	P	A	P	P	P	I	S	147.076	276.119	347.156	444.209	1187.469	1300.553	1456.654	1553.706	1624.744	1721.796	1818.849	1915.902	2028.986	2116.018
c+1	E	E	A	P	S*	L	R	P	A	P	P	P	I	S	148.084	277.127	348.164	445.217	1188.476	1301.560	1457.662	1554.714	1625.751	1722.804	1819.857	1916.910	2029.994	2117.026
z	E	E	A	P	S*	L	R	P	A	P	P	P	I	S	89.023	202.107	299.160	396.213	493.266	564.303	661.356	817.457	930.541	1673.800	1770.853	1841.890	1970.933	2099.975
z+1	E	E	A	P	S*	L	R	P	A	P	P	P	I	S	90.031	203.115	300.168	397.221	494.273	565.311	662.363	818.464	931.549	1674.808	1771.861	1842.898	1971.941	2100.983
z+2	E	E	A	P	S*	L	R	P	A	P	P	P	I	S	91.039	204.123	301.176	398.229	495.281	566.318	663.371	819.472	932.556	1675.816	1772.869	1843.906	1972.948	2101.991
	14	13	12	11	10	9	8	7	6	5	4	3	2	1	Ser	Ile	Pro	Pro	Pro	Ala	Pro	Arg	Leu	Ser	Pro	Ala	Glu	Glu

Biotools-Score: 34

known O-glycosylation region

Also found in Zauner et al., 2012

Fibrinogen beta chain precursor

$_{54}$EEAP**S**LRPAPPPI**S**$_{67}$

Fraction 16

706.27+++ → Pep [M+2H]++ 731.36++ [23.3-23.5 min] ETD

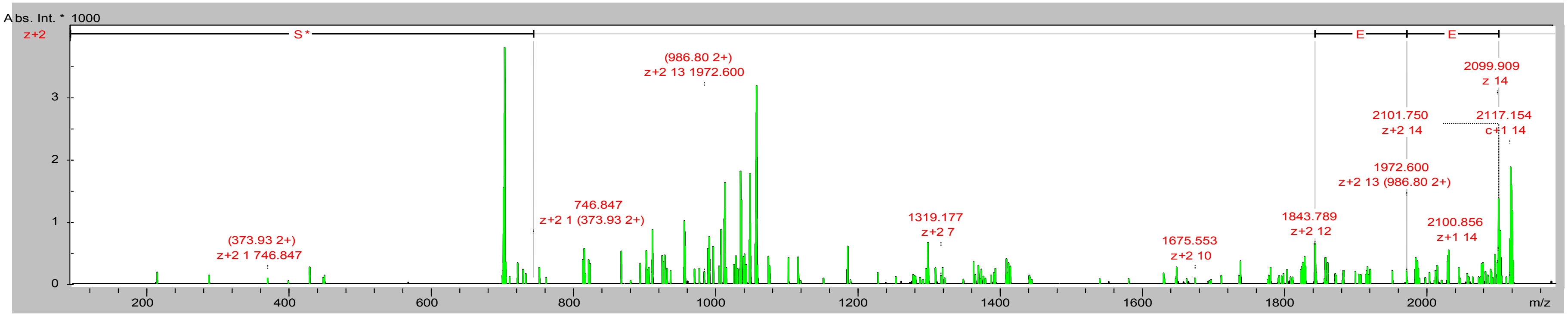

EEAPSLRPAPPPIS

	E	E	A	P	S	L	R	P	A	P	P	P	I	S	Glu	Glu	Ala	Pro	Ser	Leu	Arg	Pro	Ala	Pro	Pro	Pro	Ile	Ser
Ion	1	2	3	4	5	6	7	8	9	10	11	12	13	14	1	2	3	4	5	6	7	8	9	10	11	12	13	14
c	E	E	A	P	S	L	R	P	A	P	P	P	I	S*	147.076	276.119	347.156	444.209	531.241	644.325	800.426	897.479	968.516	1065.569	1162.621	1259.674	1372.758	2116.018
c+1	E	E	A	P	S	L	R	P	A	P	P	P	I	S*	148.084	277.127	348.164	445.217	532.249	645.333	801.434	898.487	969.524	1066.577	1163.629	1260.682	1373.766	2117.026
z	E	E	A	P	S	L	R	P	A	P	P	P	I	S*	745.251	858.335	955.388	1052.441	1149.493	1220.530	1317.583	1473.684	1586.768	1673.800	1770.853	1841.890	1970.933	2099.975
z+1	E	E	A	P	S	L	R	P	A	P	P	P	I	S*	746.259	859.343	956.396	1053.448	1150.501	1221.538	1318.591	1474.692	1587.776	1674.808	1771.861	1842.898	1971.941	2100.983
z+2	E	E	A	P	S	L	R	P	A	P	P	P	I	S*	747.267	860.351	957.403	1054.456	1151.509	1222.546	1319.599	1475.700	1588.784	1675.816	1772.869	1843.906	1972.948	2101.991
	14	13	12	11	10	9	8	7	6	5	4	3	2	1	Ser	Ile	Pro	Pro	Pro	Ala	Pro	Arg	Leu	Ser	Pro	Ala	Glu	Glu

Biotools-Score: 13

known O-glycosylation region

Also found in Zauner et al., 2012

Fibrinogen beta chain precursor

$_{54}$EEAP**S**LRPAPPPI**S**$_{67}$

Fraction 16

895.85++ → Pep [M+H]+ 1134.48+ [24.1 min] CID-MS Precursor

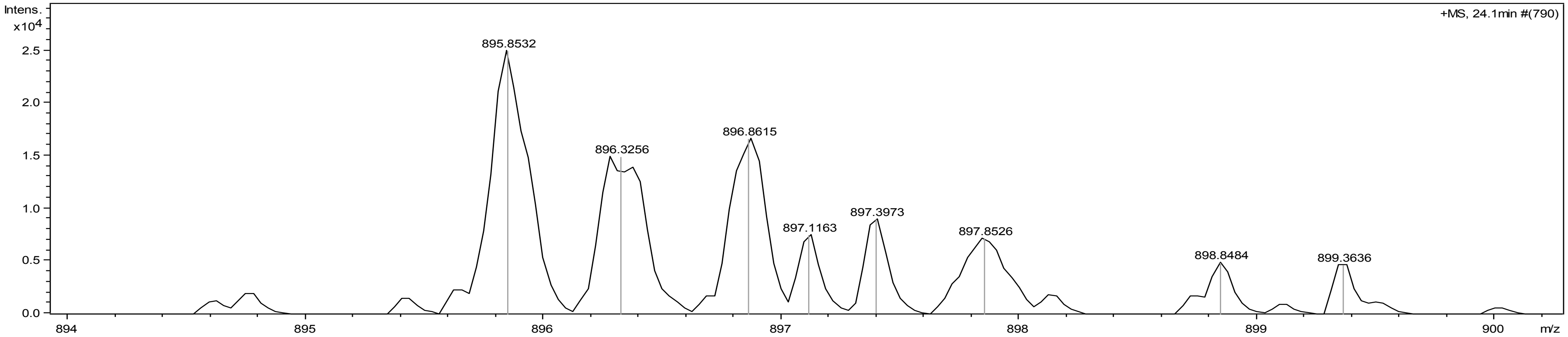

Fraction 16

895.85++ → Pep [M+H]+ 1134.48+ [24.1 min] CID-MS2

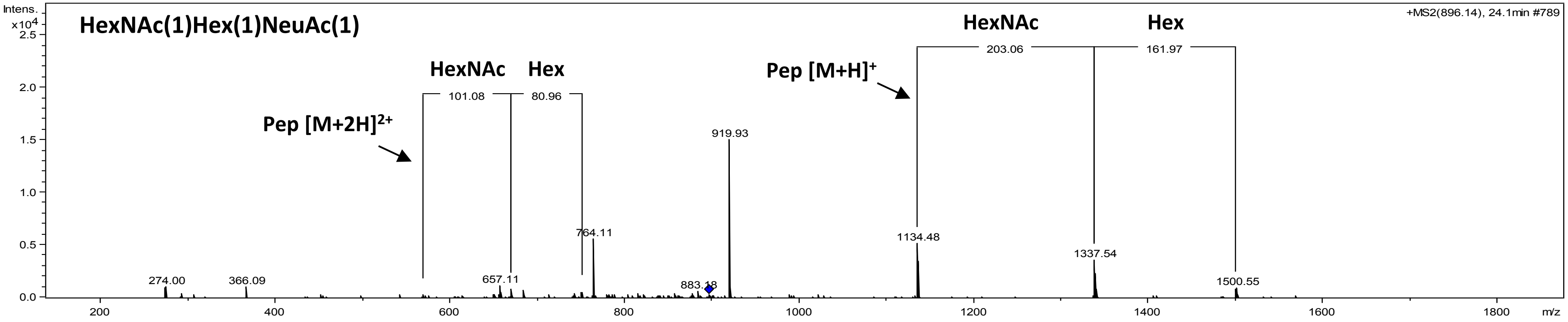

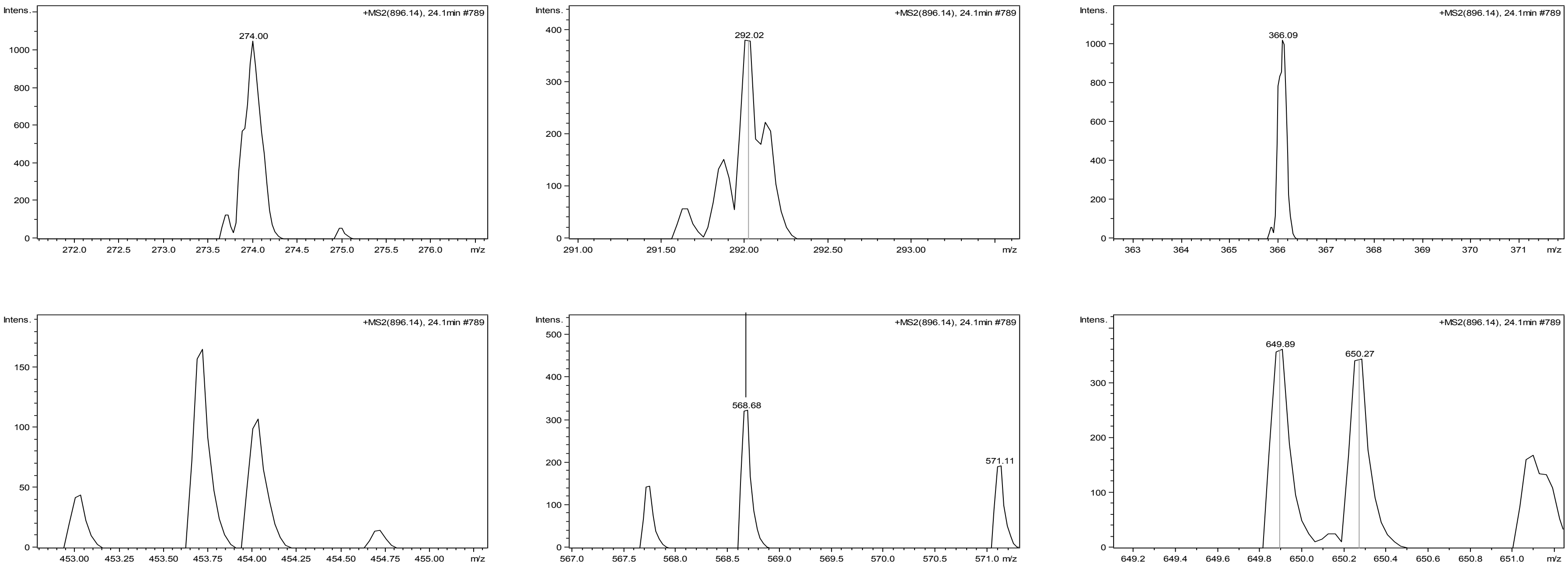

Fraction 16

895.85++ → Pep [M+H]+ 1134.48+ [24.1 min]

CID-MS2

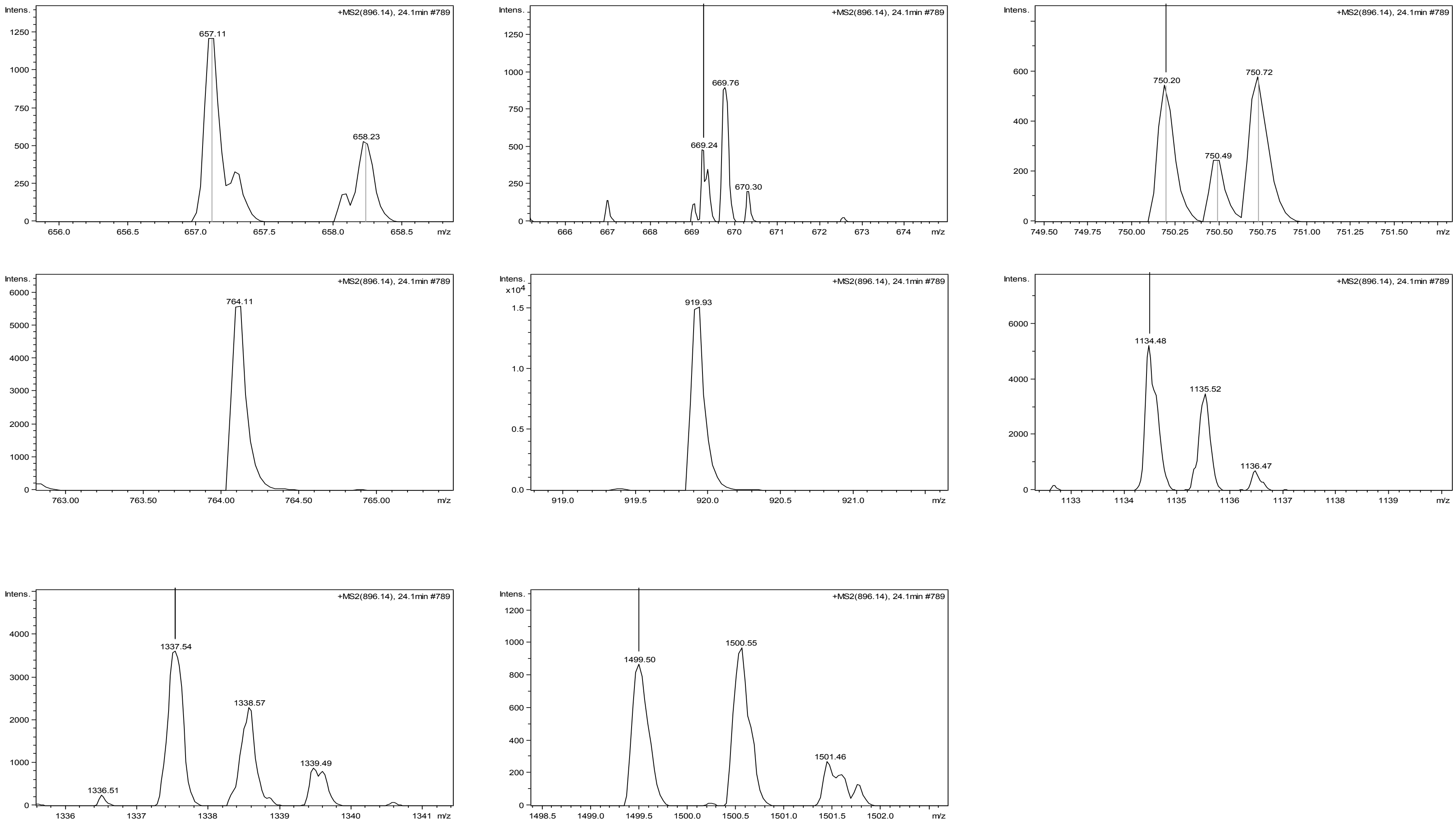

Fraction 16

895.85++ → Pep [M+H]+ 1134.48+ [24.1 min] CID-MS3

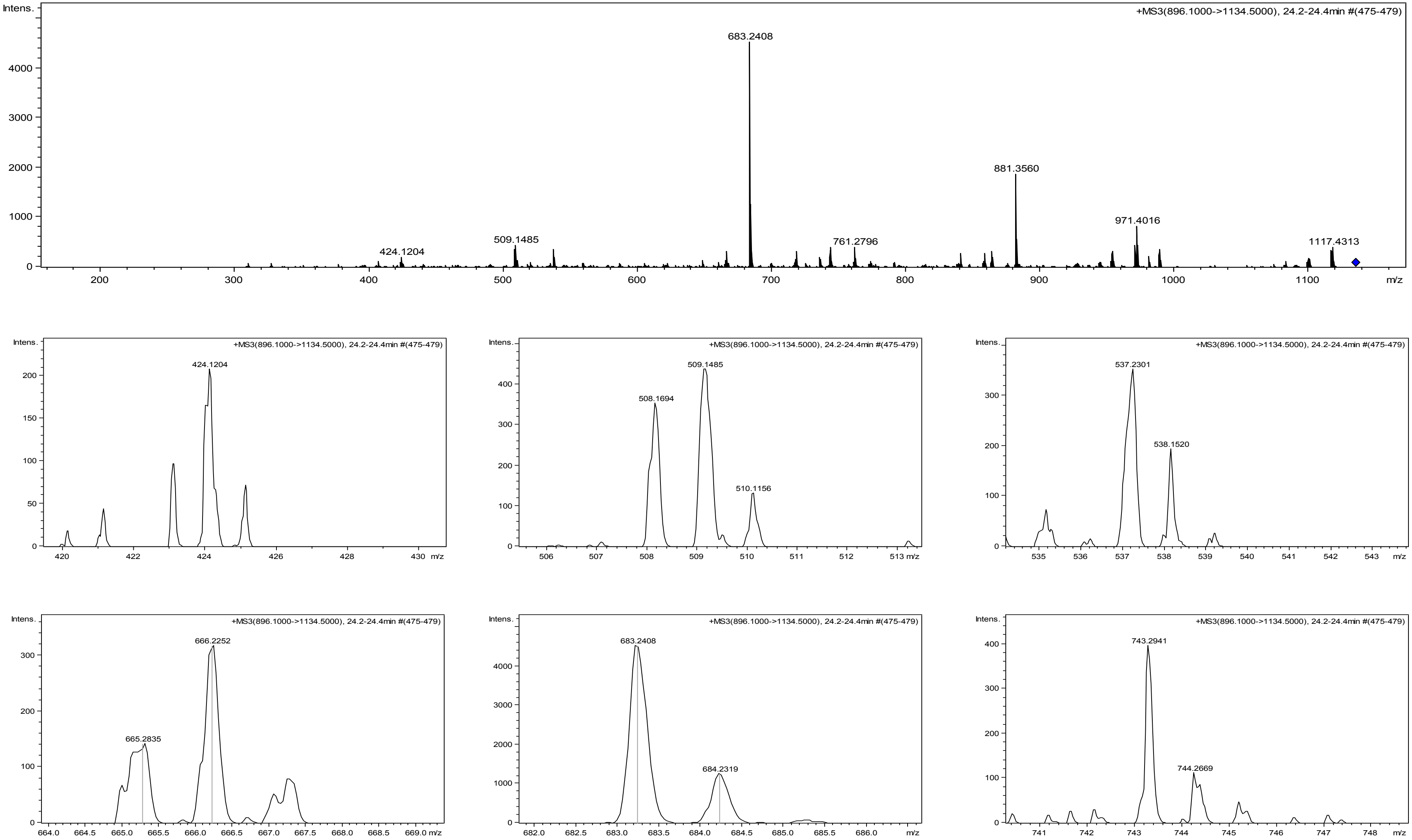

Fraction 16

895.85++ → Pep [M+H]+ 1134.48+ [24.1 min] CID-MS3

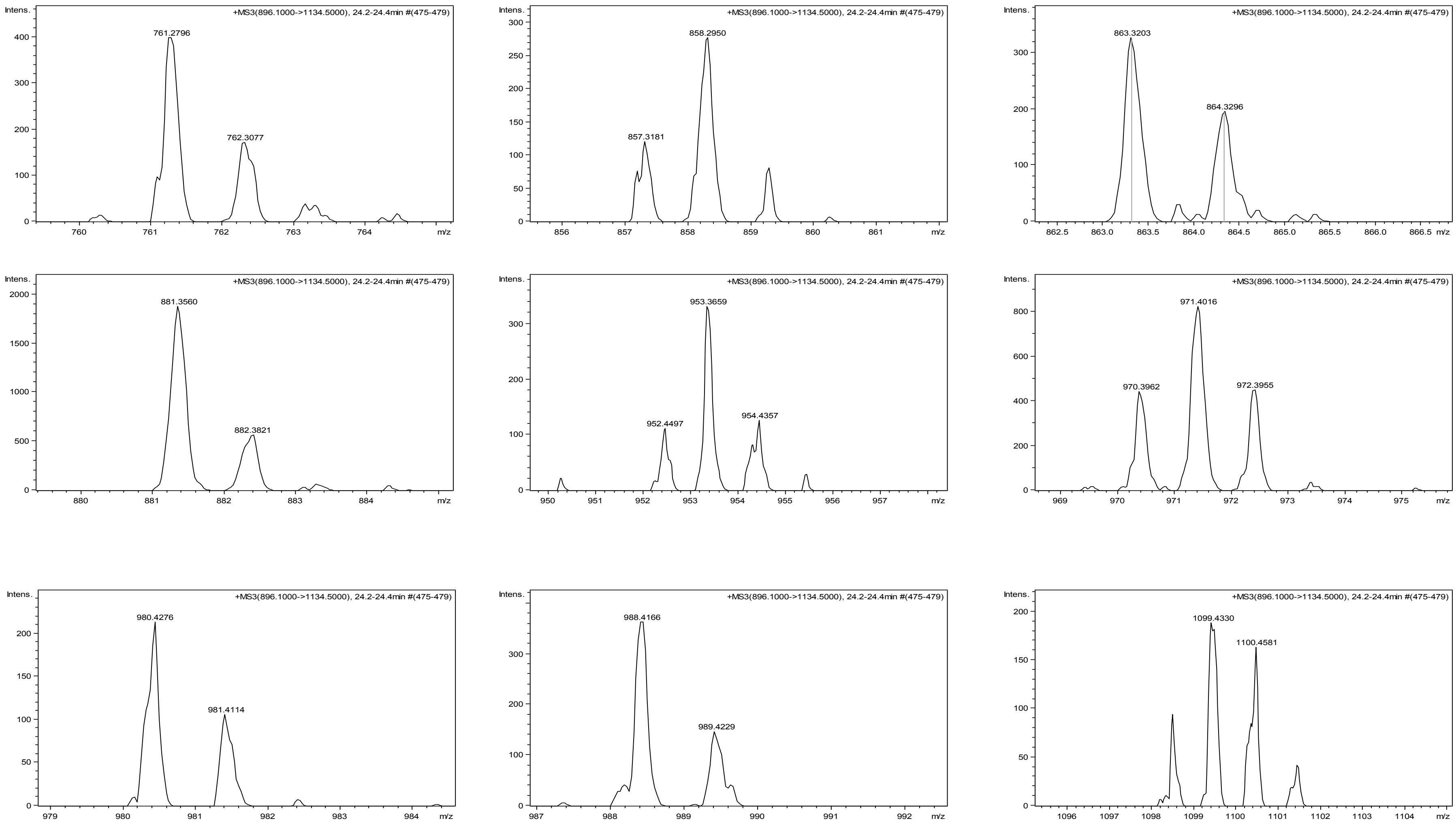

Fraction 16

895.85++ → Pep [M+H]+ 1134.48+ [24.1 min] CID-MS3

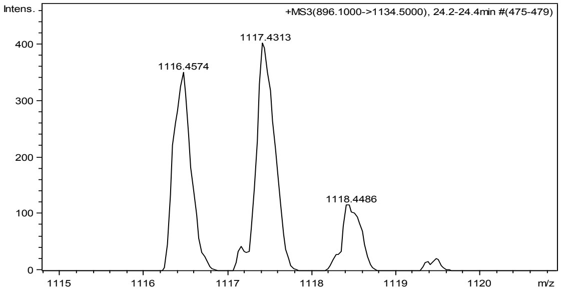

Fraction 16

895.85++ → Pep [M+H]+ 1134.48+ [24.1 min]

CID-MS3 MASCOT Search

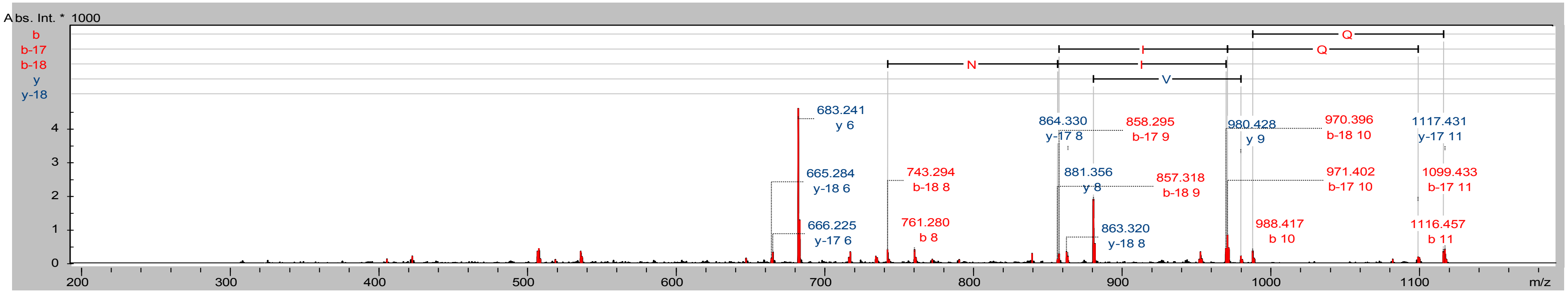

	G	P	V	P	T	P	P	D	N	I	Q	Gly	Pro	Val	Pro	Thr	Pro	Pro	Asp	Asn	Ile	Gln
Ion	1	2	3	4	5	6	7	8	9	10	11	1	2	3	4	5	6	7	8	9	10	11
b	G	P	V	P	T	P	P	D	N	I	Q	58.029	155.082	254.150	351.203	452.250	549.303	646.356	761.383	875.426	988.510	1116.568
b-17	G	P	V	P	T	P	P	D	N	I	Q	-	-	-	-	-	-	-	-	858.399	971.483	1099.542
b-18	G	P	V	P	T	P	P	D	N	I	Q	-	-	-	-	434.240	531.293	628.345	743.372	857.415	970.499	1098.558
y	G	P	V	P	T	P	P	D	N	I	Q	147.076	260.160	374.203	489.230	586.283	683.336	784.384	881.436	980.505	1077.557	1134.579
y-17	G	P	V	P	T	P	P	D	N	I	Q	130.050	243.134	357.177	472.204	569.257	666.309	767.357	864.410	963.478	1060.531	1117.552
y-18	G	P	V	P	T	P	P	D	N	I	Q	-	-	-	471.220	568.273	665.325	766.373	863.426	962.494	1059.547	1116.568
	11	10	9	8	7	6	5	4	3	2	1	Gln	Ile	Asn	Asp	Pro	Pro	Thr	Pro	Val	Pro	Gly

[24] "Alpha1-microglobulin chromophores are located to three lysine residues semiburied in the lipocalin pocket and associated with a novel lipophilic compound."
Berggaard T., Cohen A., Persson P., Lindqvist A., Cedervall T., Silow M., Thoegersen I.B., Joensson J.A., Enghild J.J., Aakerstroem B.
Protein Sci. 8:2611-2620(1999) [PubMed] [Europe PMC] [Abstract]
Cited for: BINDING TO CHROMOPHORE.

known O-glycosylation site

Protein AMBP

$_{20}$GPVP**T**PPDNIQ$_{30}$

Fraction 16

895.85++ → Pep [M+H]+ 1134.48+ [24.1 min]

CID-MS3 MASCOT Search

prot_hit_nur	prot_acc	prot_desc	prot_score	prot_mass	prot_matche	pep_query	pep_rank	pep_isbold	pep_exp_mz	pep_exp_mr	pep_exp_z	pep_calc_mr	pep_delta	pep_miss	pep_score	pep_expect	pep_res_bef	pep_seq
1	K2C1_HUMA	Keratin, type	10	66149	1	1	1	1	1134.4755	1133.4682	1	1133.4738	-0.0055	0	14.99	5.40E+02	S	YGSGSSSGGY
2	AMBP_HUM/	AMBP protei	5	39886	1	1	2	0	1134.4755	1133.4682	1	1133.5717	-0.1035	0	10.15	1.60E+03	A	GPVPTPPDN
3	HAX1_HUMA	HS1-associat	4	31601	1	1	3	0	1134.4755	1133.4682	1	1133.6445	-0.1762	0	9.14	2.10E+03	E	GLGPVLQPQ
4	KRR1_HUMA	KRR1 small s	3	43866	1	1	3	0	1134.4755	1133.4682	1	1133.5717	-0.1034	0	9.14	2.10E+03	N	PRGLLEESSF
5	TAF6_HUMA	Transcriptior	2	73250	1	1	8	0	1134.4755	1133.4682	1	1133.6921	-0.2239	0	8.21	2.60E+03	G	PRTPGLLKVP
6	CN145_HUM	Uncharacteri	2	74039	1	1	7	0	1134.4755	1133.4682	1	1133.5274	-0.0592	0	8.27	2.50E+03	H	EQNIQELMK
7	LDB3_HUMA	LIM domain-	1	78226	1	1	10	0	1134.4755	1133.4682	1	1133.52	-0.0518	0	7.66	2.90E+03	S	KAAQSQLSQ
8	TFP11_HUM/	Tuftelin-inte	1	97158	1	1	9	0	1134.4755	1133.4682	1	1133.5216	-0.0533	0	8.14	2.60E+03	W	YLGWKSMFS
9	CO8A1_HUM	Collagen alp	1	73431	1	1	6	0	1134.4755	1133.4682	1	1132.5951	0.8732	0	8.31	2.50E+03	K	PLPPQIPPQN
10	PLEC1_HUM/	Plectin-1 (PL	0	533408	1	1	5	0	1134.4755	1133.4682	1	1133.4989	-0.0307	0	8.43	2.40E+03	G	YLNKDTHDQ

Biotools-Score: 7

MASCOT-Score: 10

known O-glycosylation site

Protein AMBP

$_{20}$GPVP**T**PPDNIQ$_{30}$

Fraction 16

895.85++ → Pep [M+H]+ 1134.48+ [24.1 min]

ETD

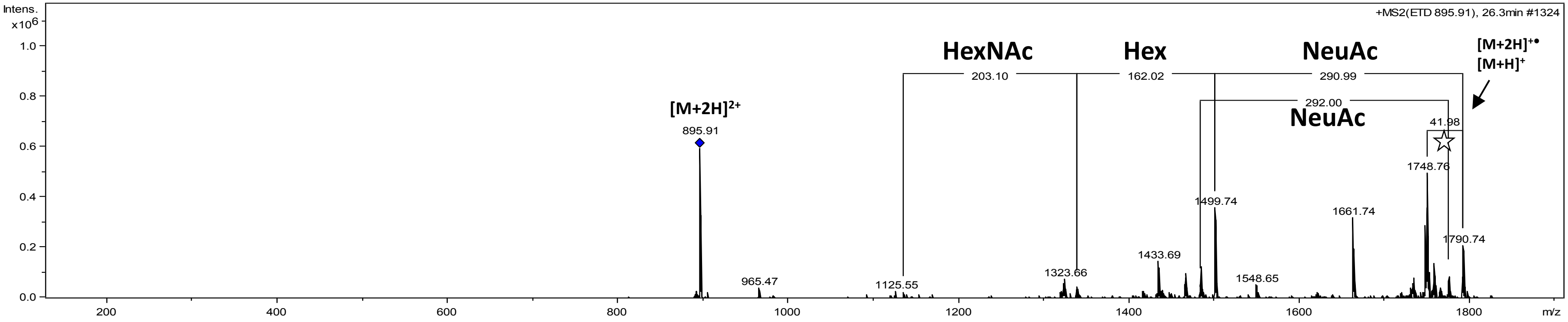

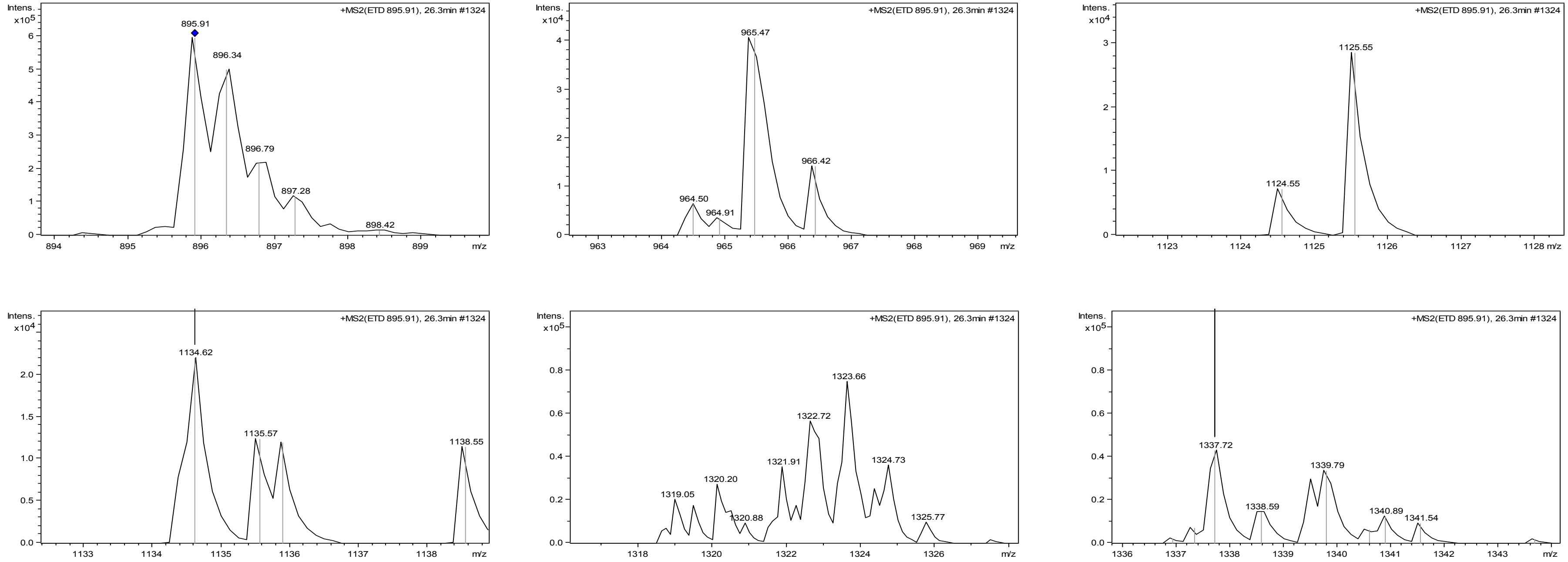

Fraction 16

895.85++ → Pep [M+H]+ 1134.48+ [24.1 min] ETD

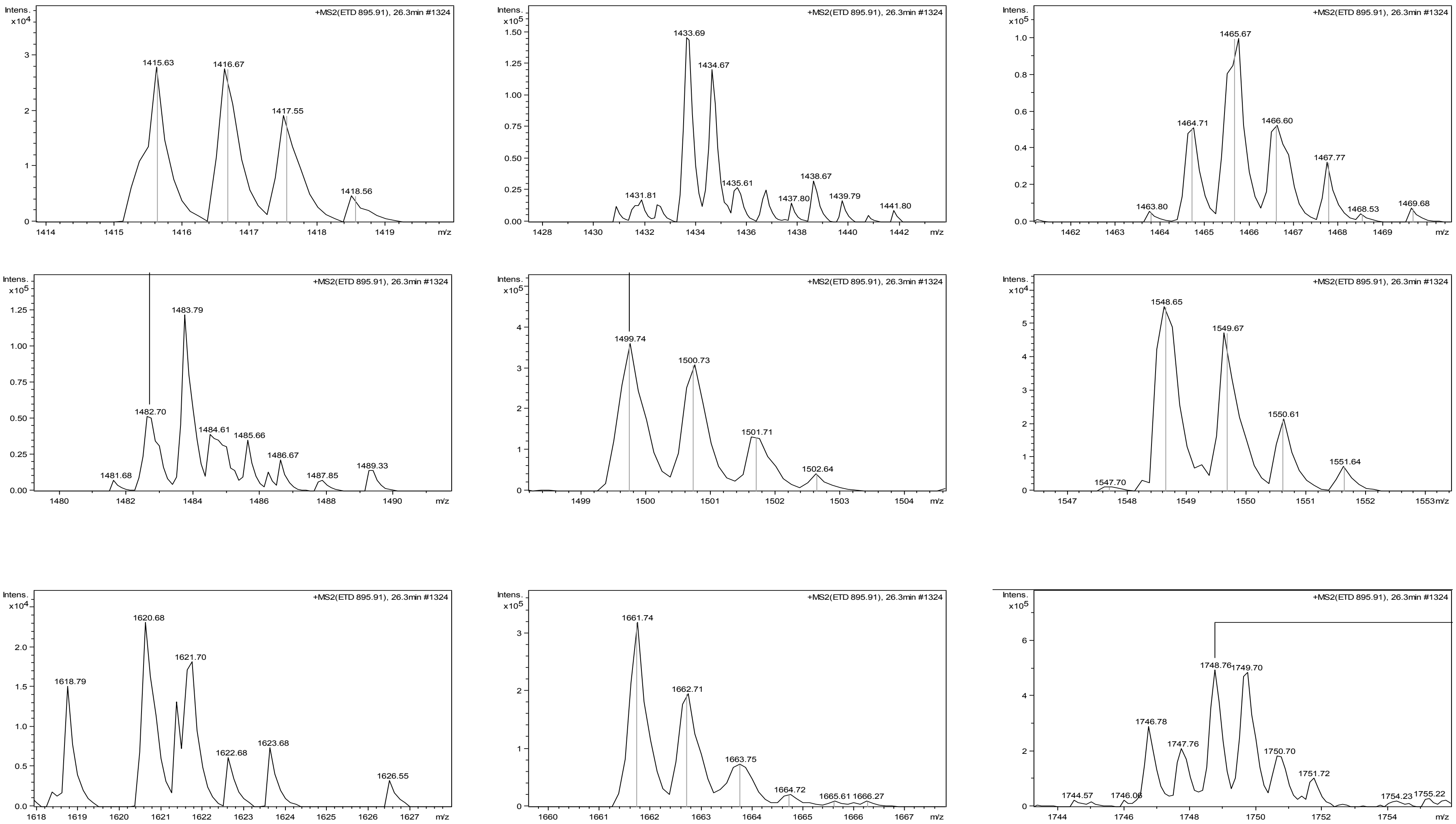

Fraction 16

895.85++ → Pep [M+H]+ 1134.48+ [24.1 min] ETD

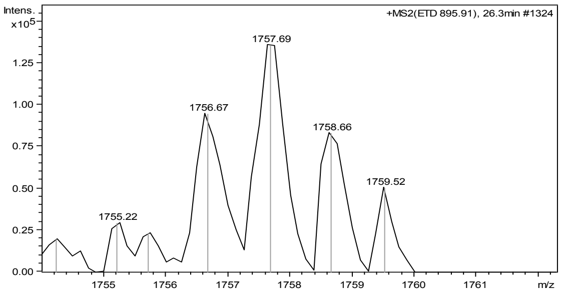

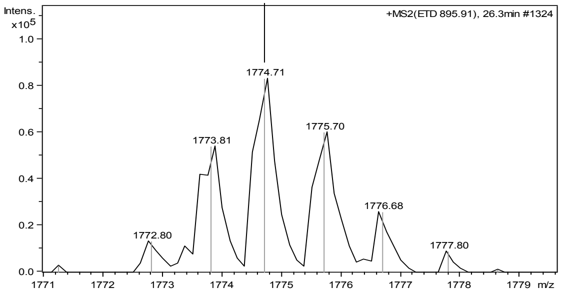

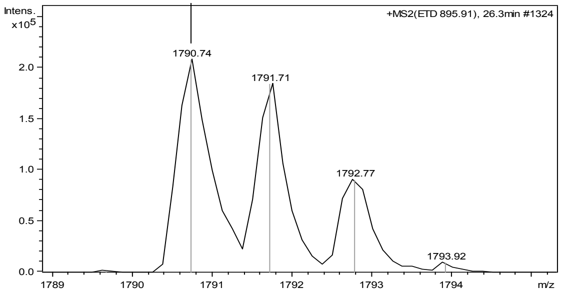

Fraction 16

895.85++ → Pep [M+H]+ 1134.48+ [24.1 min] ETD

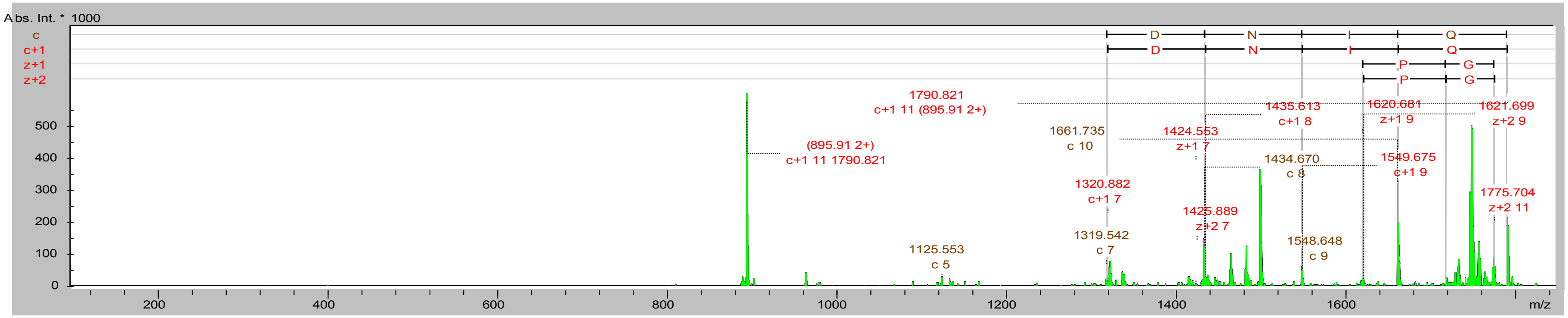

	G	P	V	P	T	P	P	D	N	I	Q	Gly	Pro	Val	Pro	Thr	Pro	Pro	Asp	Asn	Ile	Gln
Ion	1	2	3	4	5	6	7	8	9	10	11	1	2	3	4	5	6	7	8	9	10	11
c	G	P	V	P	T*	P	P	D	N	I	Q	75.055	172.108	271.176	368.229	1125.505	1222.557	1319.610	1434.637	1548.680	1661.764	1789.823
c+1	G	P	V	P	T*	P	P	D	N	I	Q	76.063	173.116	272.184	369.237	1126.512	1223.565	1320.618	1435.645	1549.688	1662.772	1790.830
z	G	P	V	P	T*	P	P	D	N	I	Q	130.050	243.134	357.177	472.204	569.257	666.309	1423.585	1520.637	1619.706	1716.759	1773.780
z+1	G	P	V	P	T*	P	P	D	N	I	Q	131.058	244.142	358.185	473.212	570.264	667.317	1424.592	1521.645	1620.714	1717.766	1774.788
z+2	G	P	V	P	T*	P	P	D	N	I	Q	132.066	245.150	359.193	474.219	571.272	668.325	1425.600	1522.653	1621.721	1718.774	1775.796
	11	10	9	8	7	6	5	4	3	2	1	Gln	Ile	Asn	Asp	Pro	Pro	Thr	Pro	Val	Pro	Gly

Biotools-Score: 80

known O-glycosylation site

Protein AMBP

$_{20}$GPVP**T**PPDNIQ$_{30}$

Fraction 16

720.91+++ → Pep [M+2H]++ 752.72++ [24.3 min] CID-MS Precursor

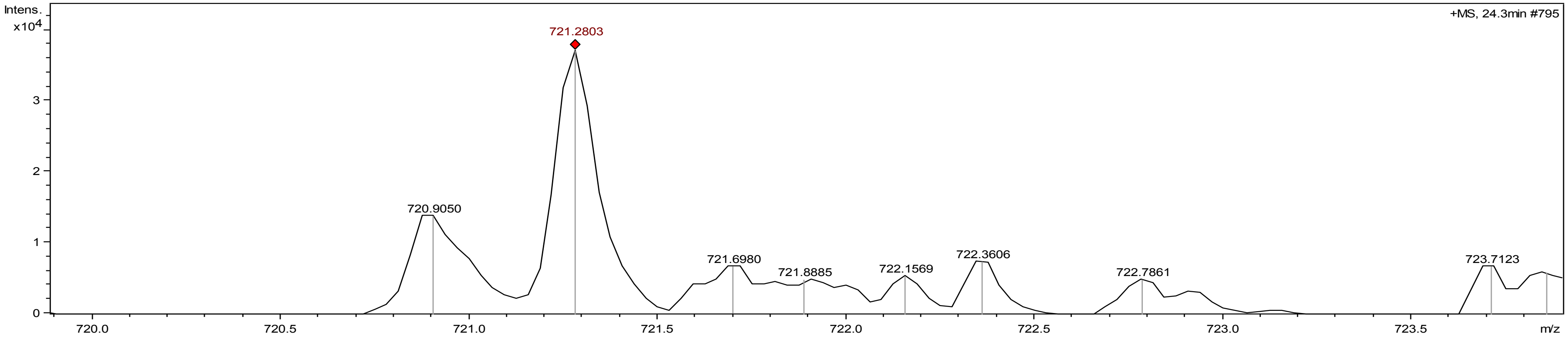

ETD spectrum of poor quality

CID spectrum of poor quality

Fraction 16

720.91+++ → Pep [M+2H]++ 752.72++ [24.3 min] CID-MS2

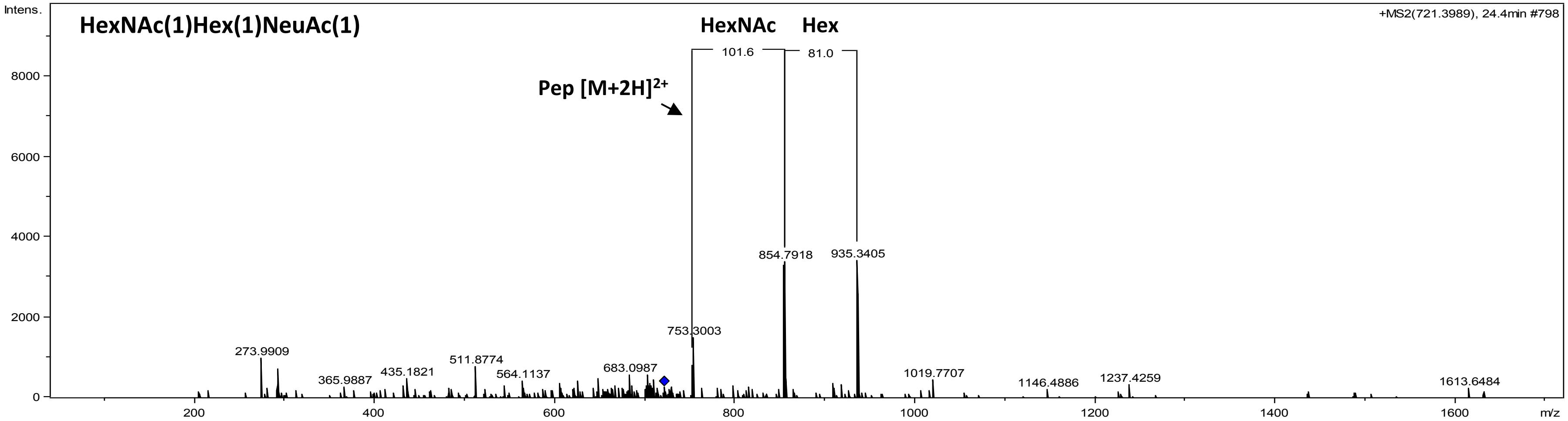

Fraction 16

883.82++ → Pep [M+H]+ 819.35+ [24.3-24.5 min] CID-MS Precursor

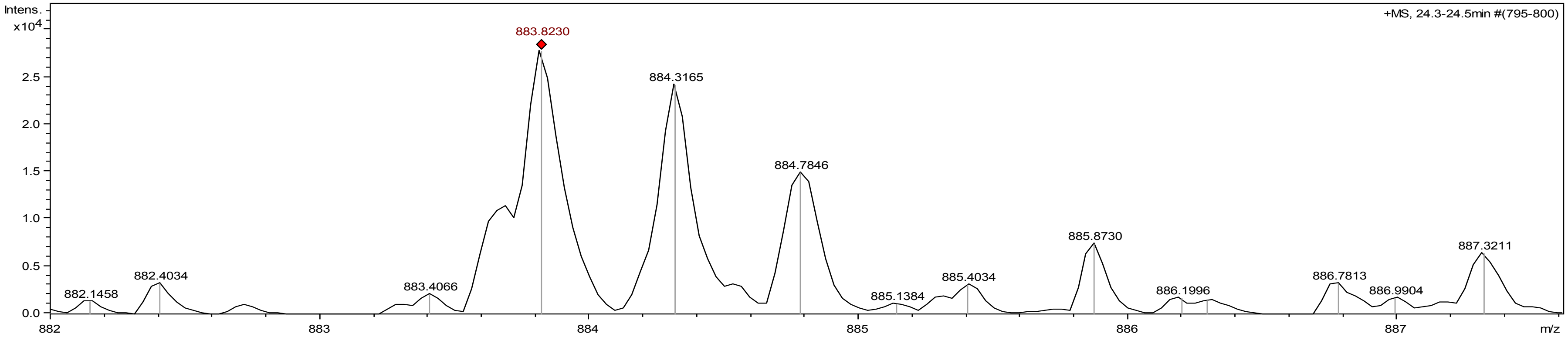

ETD spectrum of poor quality

Fraction 16

883.82++ → Pep [M+H]+ 819.35+ [24.3-24.5 min] CID-MS2

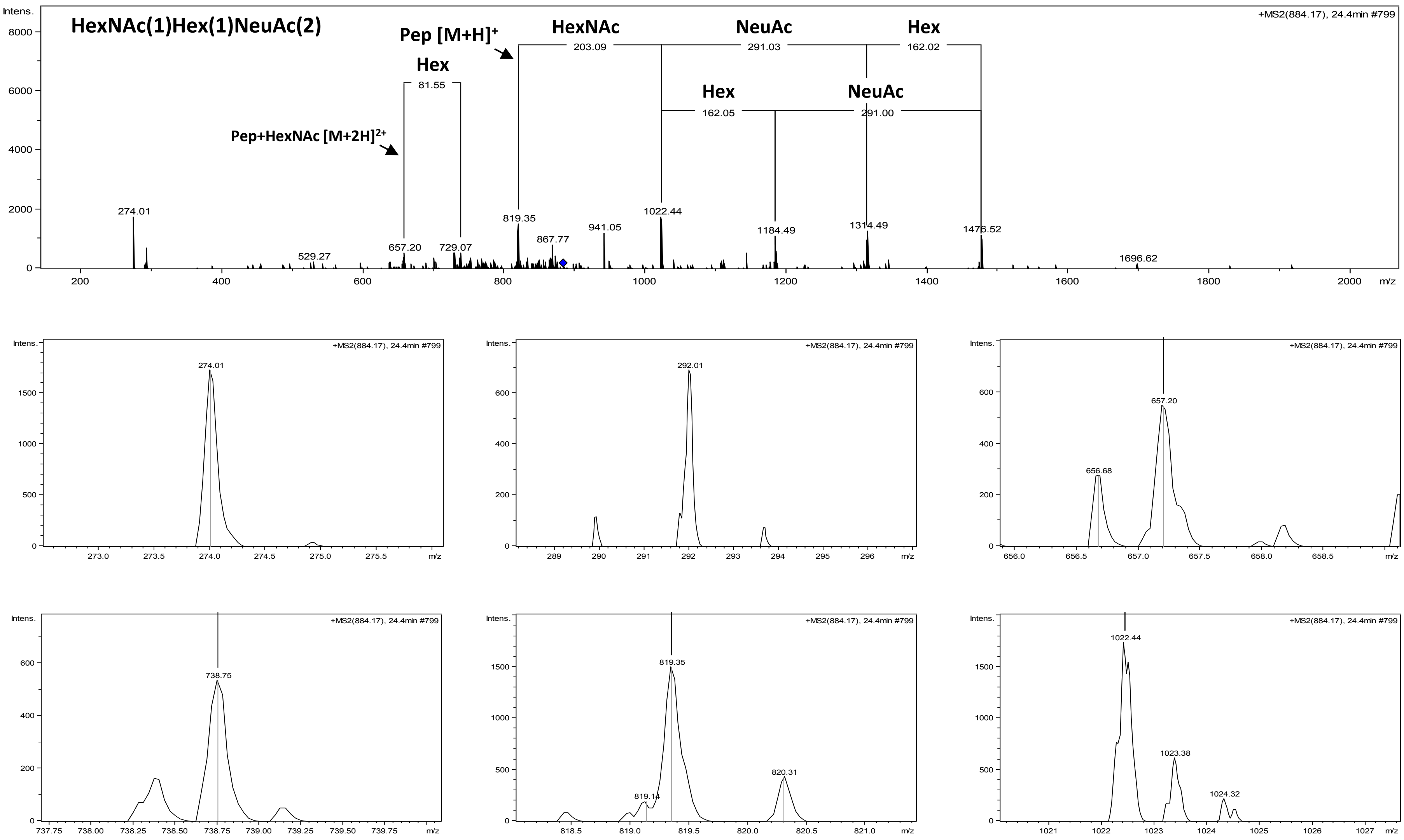

Fraction 16

883.82++ → Pep [M+H]+ 819.35+ [24.3-24.5 min]

CID-MS2

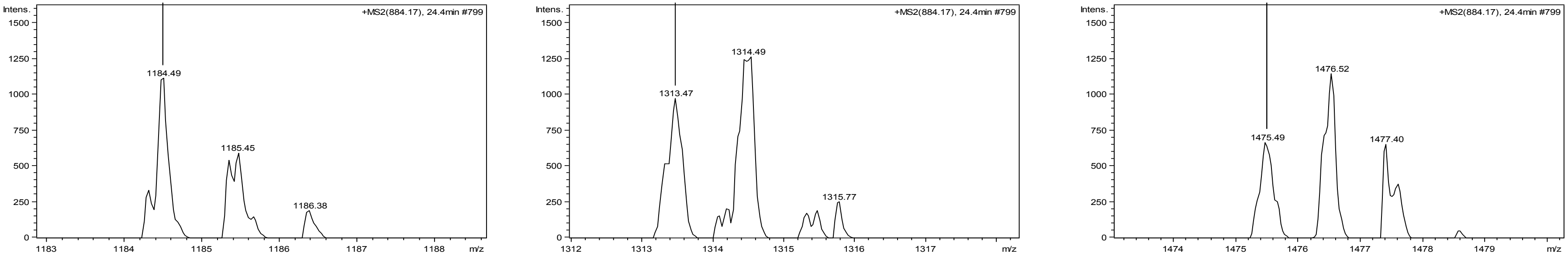

Fraction 16

883.82++ → Pep [M+H]+ 819.35+ [24.3-24.5 min] CID-MS3

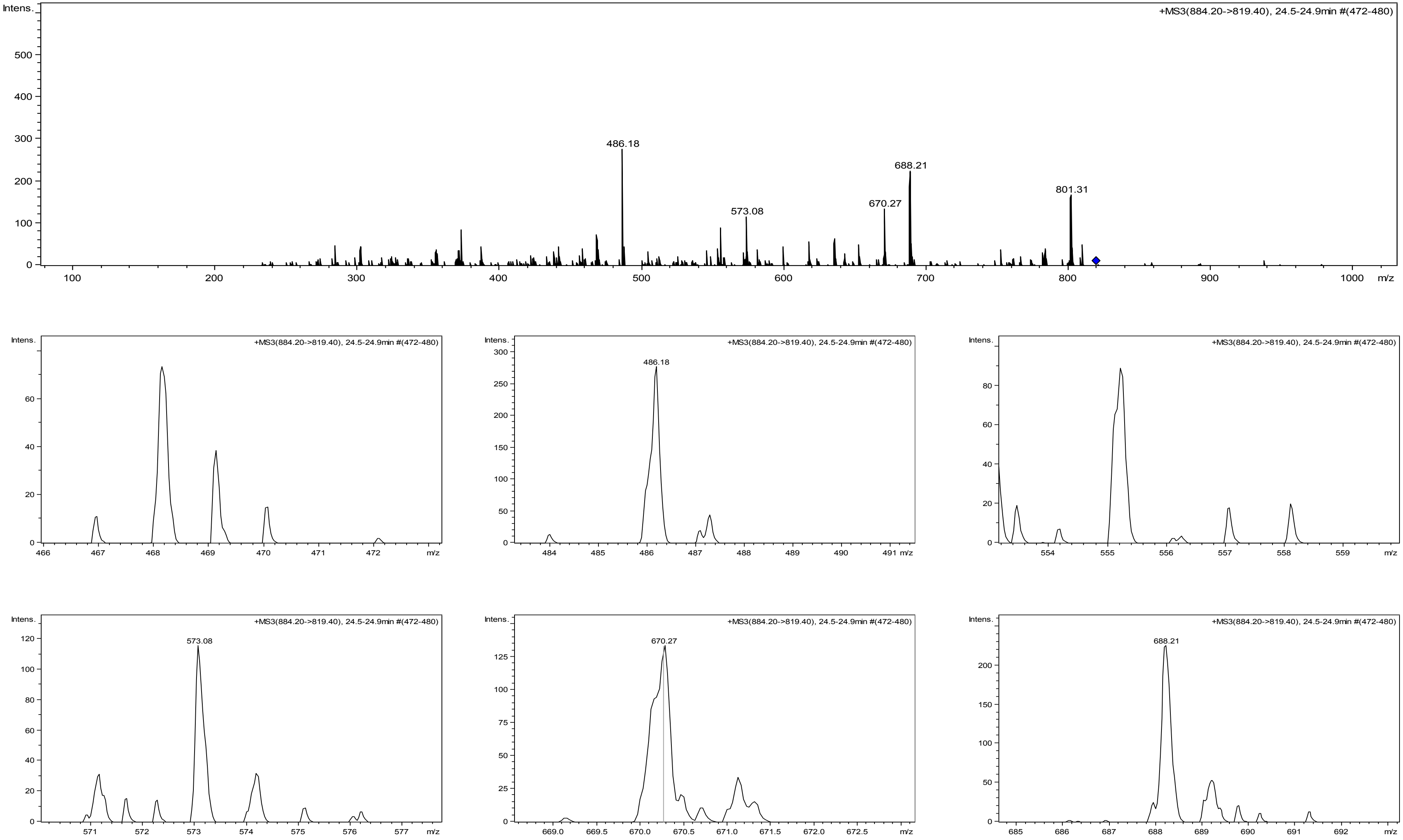

Fraction 16

883.82++ → Pep [M+H]+ 819.35+ [24.3-24.5 min] CID-MS3

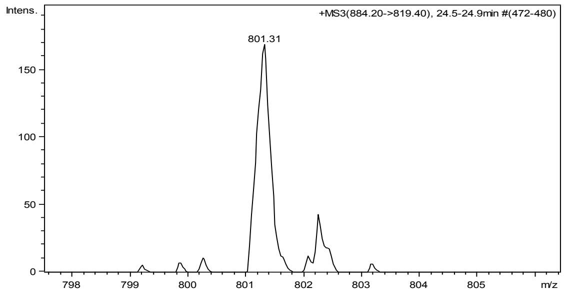

Fraction 16

883.82++ → Pep [M+H]+ 819.35+ [24.3-24.5 min] CID-MS3 MASCOT Search

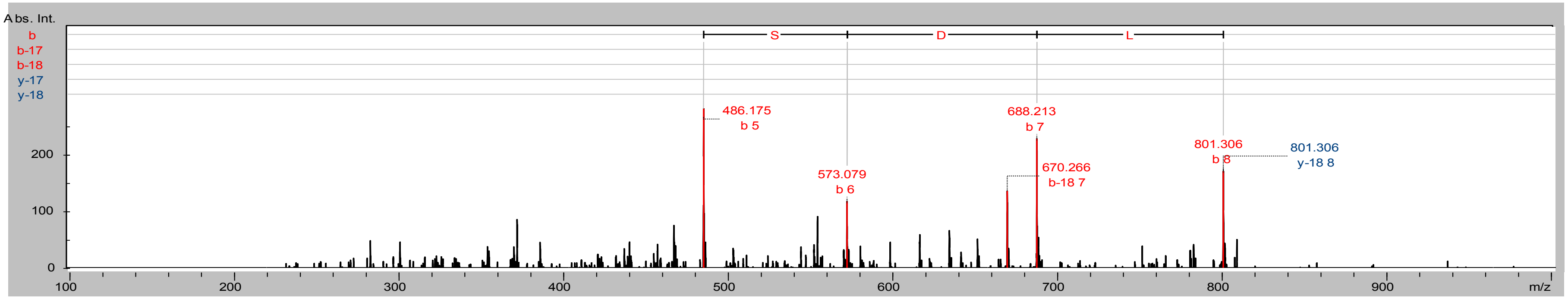

	A	V	T	I	T	S	D	L	Ala	Val	Thr	Ile	Thr	Ser	Asp	Leu
Ion	1	2	3	4	5	6	7	8	1	2	3	4	5	6	7	8
b	A	V	T	I	T	S	D	L	72.044	171.113	272.160	385.245	486.292	573.324	688.351	801.435
b-17	A	V	T	I	T	S	D	L	-	-	-	-	-	-	-	-
b-18	A	V	T	I	T	S	D	L	-	-	254.150	367.234	468.282	555.314	670.341	783.425
y	A	V	T	I	T	S	D	L	132.102	247.129	334.161	435.209	548.293	649.340	748.409	819.446
y-17	A	V	T	I	T	S	D	L	-	-	-	-	-	-	-	-
y-18	A	V	T	I	T	S	D	L	-	229.118	316.150	417.198	530.282	631.330	730.398	801.435
	8	7	6	5	4	3	2	1	Leu	Asp	Ser	Thr	Ile	Thr	Val	Ala

Not sure

unknown O-glycosylation region

Apolipoprotein-L domain-containing protein 1

$_{131}$AV**TITS**DL$_{138}$

Fraction 16

883.82++ → Pep [M+H]+ 819.35+ [24.3-24.5 min]   CID-MS3 MASCOT Search

prot_hit_nur	prot_acc	prot_desc	prot_score	prot_mass	prot_matche	pep_query	pep_rank	pep_isbold	pep_exp_mz	pep_exp_mr	pep_exp_z	pep_calc_mr	pep_delta	pep_miss	pep_score	pep_expect	pep_res_bef	pep_seq
1	APLD1_HUM	Apolipoprot	26	31154	1	1	1	1	819.3526	818.3453	1	818.4386	-0.0932	0	30.75	13	G	AVTITSDL
2	IRX2_HUMAI	Iroquois-clas	26	49498	1	1	1	0	819.3526	818.3453	1	818.4021	-0.0568	0	30.75	13	L	AEIATSDL
3	SLAF6_HUM	SLAM family	26	37593	1	1	1	0	819.3526	818.3453	1	818.3657	-0.0204	0	30.75	13	I	AENAVSNL
4	APOF_HUMA	Apolipoprot	25	33727	1	1	1	0	819.3526	818.3453	1	818.4022	-0.0568	0	30.75	13	R	AISDVSDL
5	TRI22_HUMA	Tripartite mc	25	58051	1	1	1	0	819.3526	818.3453	1	818.4385	-0.0932	0	30.75	13	D	ASTLISDL
6	DEOC_HUMA	Putative dec	25	35494	1	1	1	0	819.3526	818.3453	1	818.4385	-0.0932	0	30.75	13	G	ASTLLSDI
7	HCN1_HUMA	Potassium/s	25	99493	1	1	1	0	819.3526	818.3453	1	818.3844	-0.0391	0	30.75	13	Q	APVSMSDL
8	AL2S4_HUM	Amyotrophic	24	48442	1	1	1	0	819.3526	818.3453	1	818.3658	-0.0204	0	30.75	13	L	AGDQLSNL
9	NRAP_HUMA	Nebulin-rela	23	197920	1	1	1	0	819.3526	818.3453	1	818.4021	-0.0568	0	30.75	13	K	AQTLASDL
10	ZN318_HUM	Zinc finger p	23	233392	1	1	1	0	819.3526	818.3453	1	818.3406	0.0047	0	30.75	13	L	ANGENSNL

Biotools-Score: 10

MASCOT-Score: 31

Not sure

unknown O-glycosylation region

Apolipoprotein-L domain-containing protein 1

$_{131}$AV**T**I**TS**DL$_{138}$

Fraction 16

883.82++ → Pep [M+H]+ 819.35+ [24.3-24.5 min] ETD

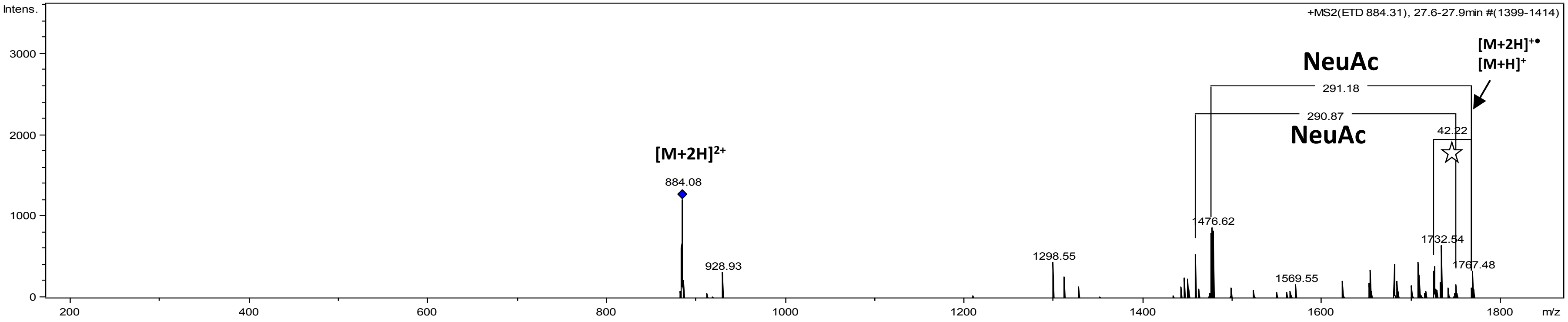

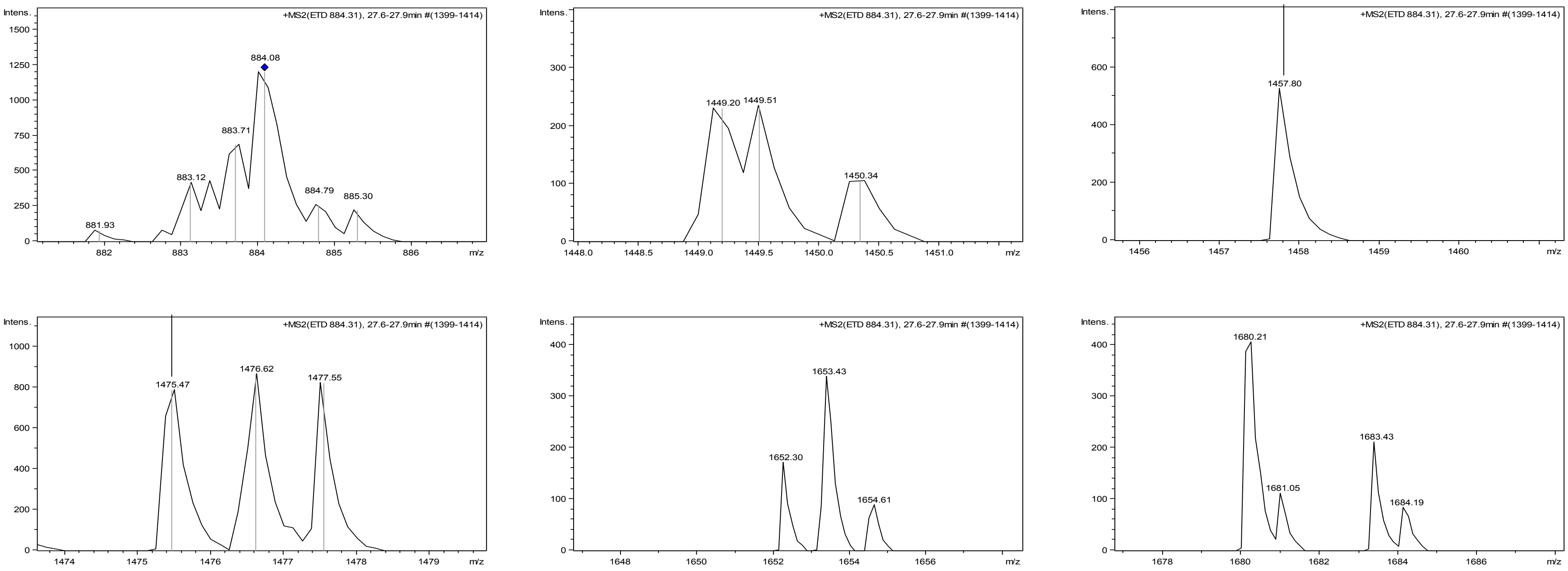

Fraction 16

883.82++ → Pep [M+H]+ 819.35+ [24.3-24.5 min]

ETD

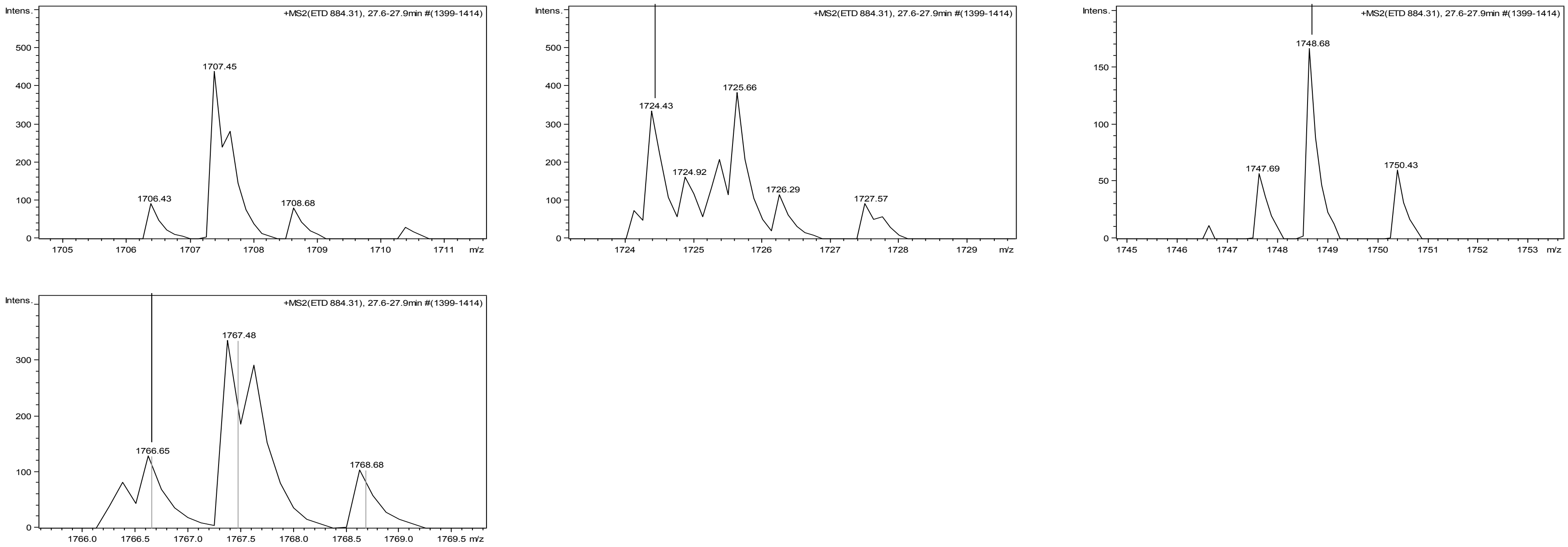

Fraction 16

883.82++ → Pep [M+H]+ 819.35+ [24.3-24.5 min] ETD

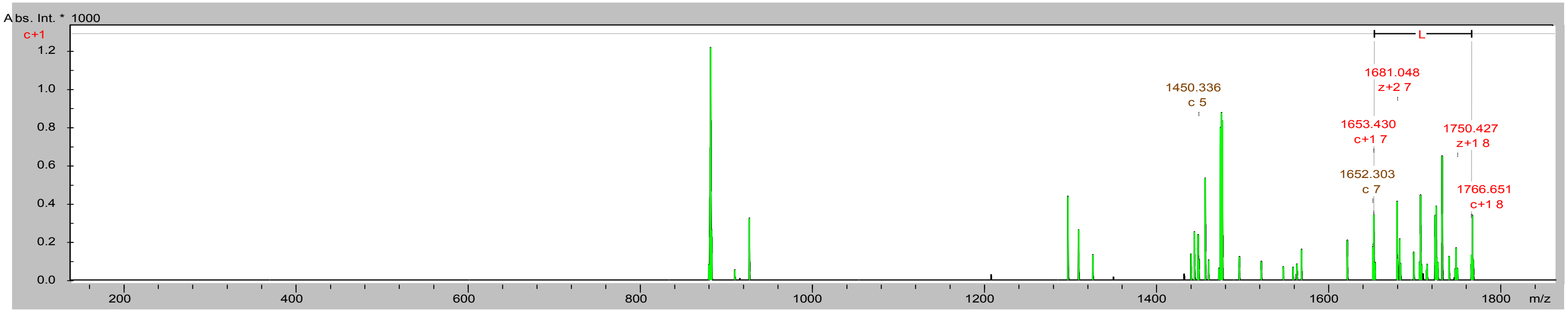

AVTITSDL

	A	V	T	I	T	S	D	L	Ala	Val	Thr	Ile	Thr	Ser	Asp	Leu
Ion	1	2	3	4	5	6	7	8	1	2	3	4	5	6	7	8
c	A	V	T*	I	T	S	D	L	89.071	188.139	1236.510	1349.594	1450.642	1537.674	1652.701	1765.785
c+1	A	V	T*	I	T	S	D	L	90.079	189.147	1237.518	1350.602	1451.650	1538.682	1653.709	1766.793
z	A	V	T*	I	T	S	D	L	115.075	230.102	317.134	418.182	531.266	1579.637	1678.705	1749.742
z+1	A	V	T*	I	T	S	D	L	116.083	231.110	318.142	419.190	532.274	1580.645	1679.713	1750.750
z+2	A	V	T*	I	T	S	D	L	117.091	232.118	319.150	420.198	533.282	1581.652	1680.721	1751.758
	8	7	6	5	4	3	2	1	Leu	Asp	Ser	Thr	Ile	Thr	Val	Ala

Biotools-Score: 4

Not sure

unknown O-glycosylation region

Apolipoprotein-L domain-containing protein 1 $_{131}$AVTITSDL$_{138}$

Fraction 16

883.82++ → Pep [M+H]+ 819.35+ [24.3-24.5 min] ETD

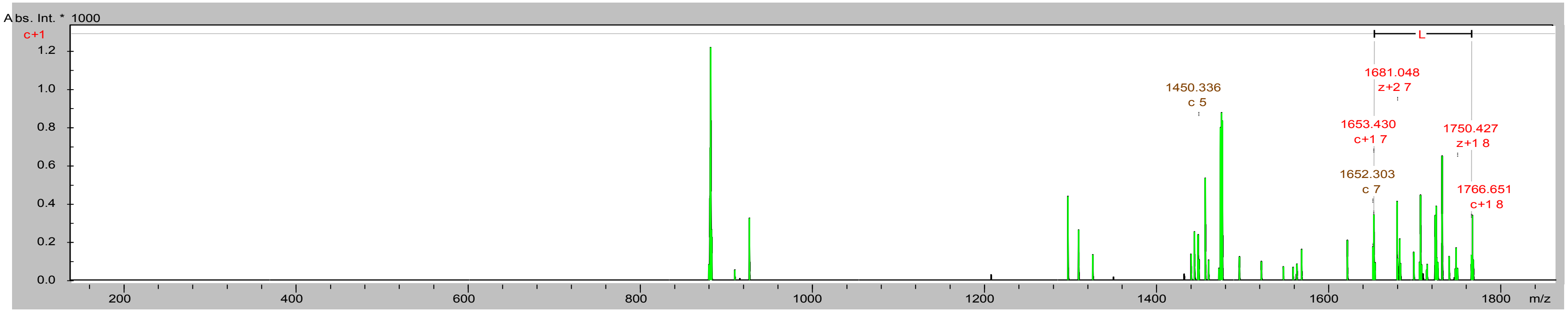

AVTI**T**SDL

	A	V	T	I	T	S	D	L	Ala	Val	Thr	Ile	Thr	Ser	Asp	Leu
Ion	1	2	3	4	5	6	7	8	1	2	3	4	5	6	7	8
c	A	V	T	I	T*	S	D	L	89.071	188.139	289.187	402.271	1450.642	1537.674	1652.701	1765.785
c+1	A	V	T	I	T*	S	D	L	90.079	189.147	290.195	403.279	1451.650	1538.682	1653.709	1766.793
z	A	V	T	I	T*	S	D	L	115.075	230.102	317.134	1365.505	1478.589	1579.637	1678.705	1749.742
z+1	A	V	T	I	T*	S	D	L	116.083	231.110	318.142	1366.513	1479.597	1580.645	1679.713	1750.750
z+2	A	V	T	I	T*	S	D	L	117.091	232.118	319.150	1367.521	1480.605	1581.652	1680.721	1751.758
	8	7	6	5	4	3	2	1	Leu	Asp	Ser	Thr	Ile	Thr	Val	Ala

Biotools-Score: 4

Not sure

unknown O-glycosylation region

Apolipoprotein-L domain-containing protein 1 ${}_{131}$AVT**IT**SDL${}_{138}$

Fraction 16

883.82++ → Pep [M+H]+ 819.35+ [24.3-24.5 min] ETD

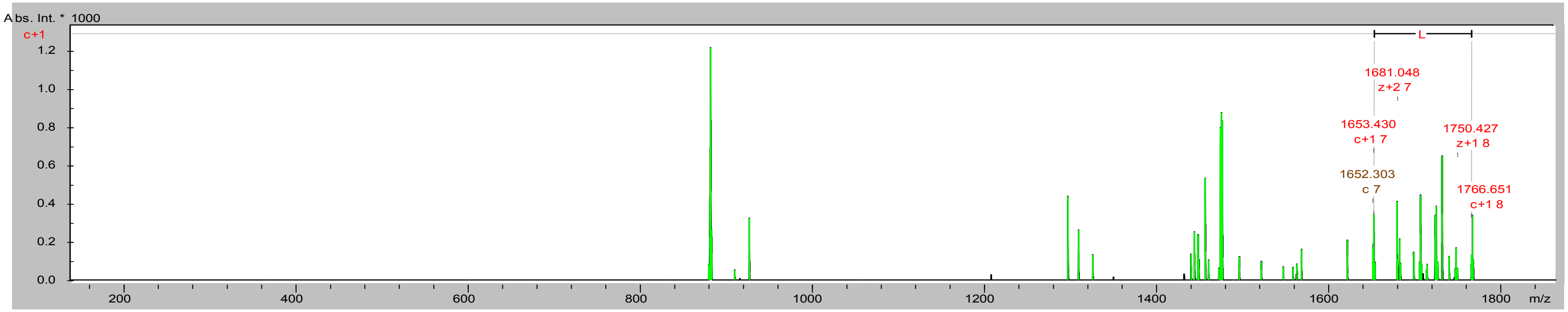

AVTITSDL

	A	V	T	I	T	S	D	L	Ala	Val	Thr	Ile	Thr	Ser	Asp	Leu
Ion	1	2	3	4	5	6	7	8	1	2	3	4	5	6	7	8
c	A	V	T	I	T	S*	D	L	89.071	188.139	289.187	402.271	503.319	1537.674	1652.701	1765.785
c+1	A	V	T	I	T	S*	D	L	90.079	189.147	290.195	403.279	504.327	1538.682	1653.709	1766.793
z	A	V	T	I	T	S*	D	L	115.075	230.102	1264.457	1365.505	1478.589	1579.637	1678.705	1749.742
z+1	A	V	T	I	T	S*	D	L	116.083	231.110	1265.465	1366.513	1479.597	1580.645	1679.713	1750.750
z+2	A	V	T	I	T	S*	D	L	117.091	232.118	1266.473	1367.521	1480.605	1581.652	1680.721	1751.758
	8	7	6	5	4	3	2	1	Leu	Asp	Ser	Thr	Ile	Thr	Val	Ala

Biotools-Score: 3

Not sure

unknown O-glycosylation region

Apolipoprotein-L domain-containing protein 1 $_{131}$AVTITSDL$_{138}$

Fraction 16

681.35+++ → Pep [M+2H]++ 693.36++ [24.6-25.1 min]

CID-MS Precursor

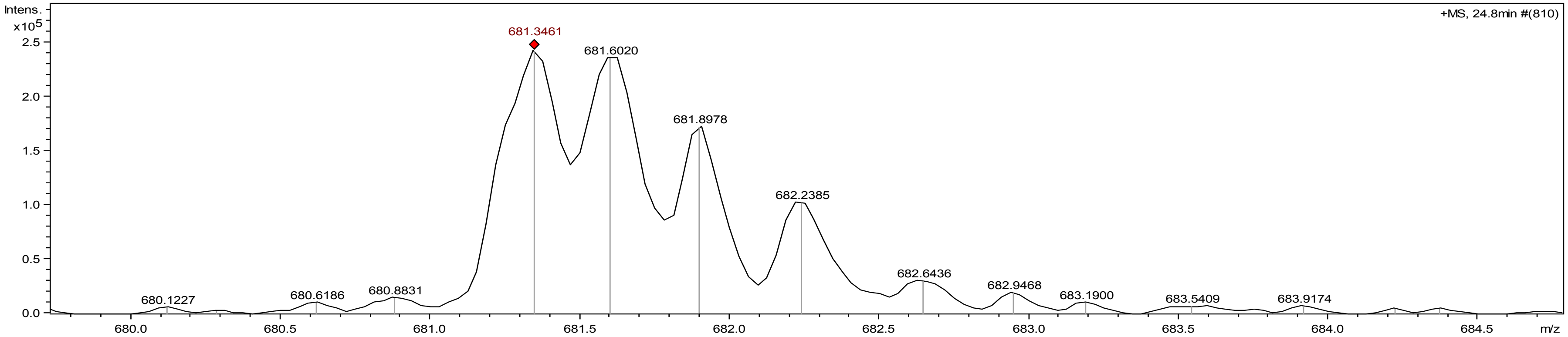

Fraction 16

681.35+++ → Pep [M+2H]++ 693.36++ [24.6-25.1 min] CID-MS2

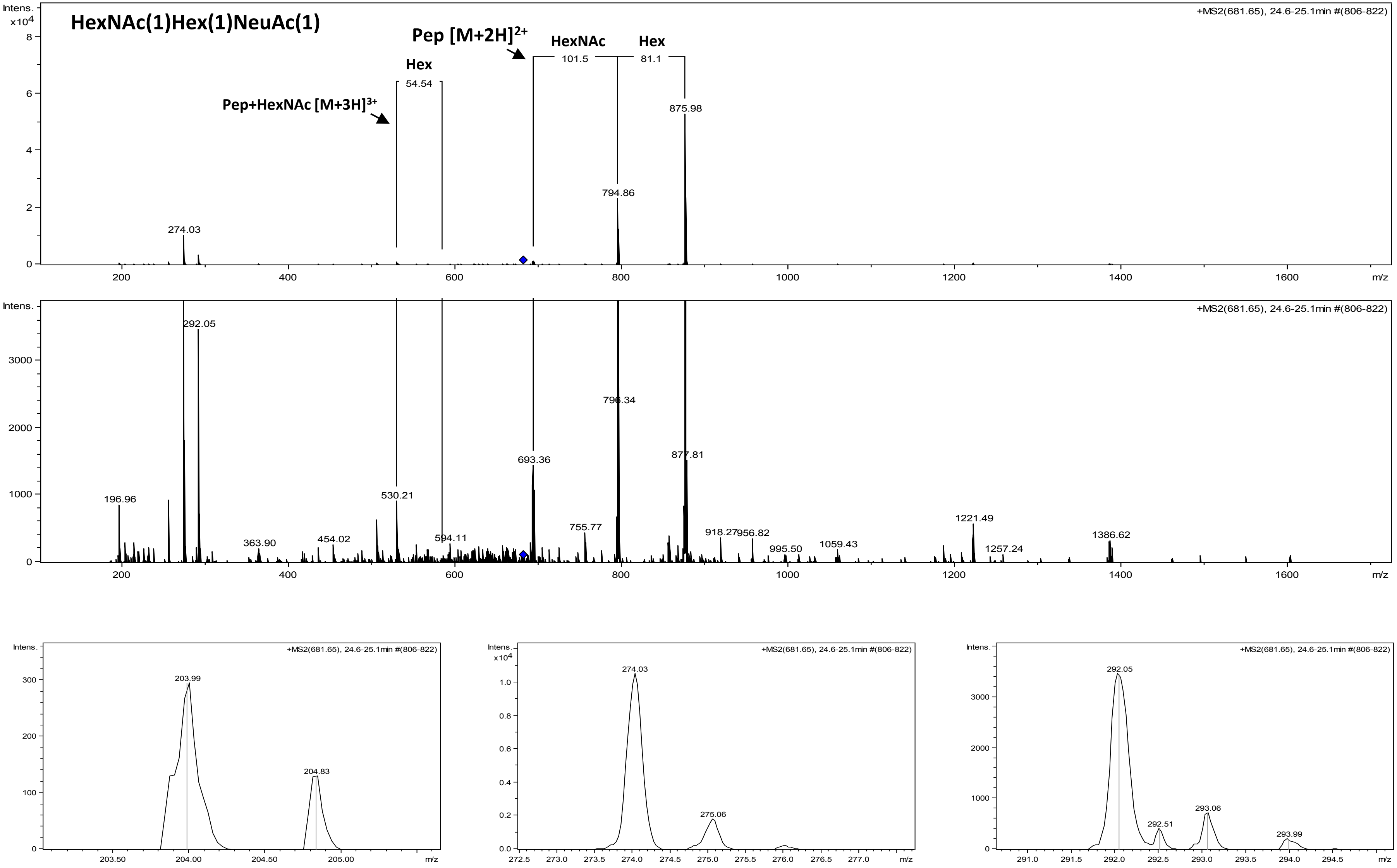

Fraction 16

681.35+++ → Pep [M+2H]++ 693.36++ [24.6-25.1 min]

CID-MS2

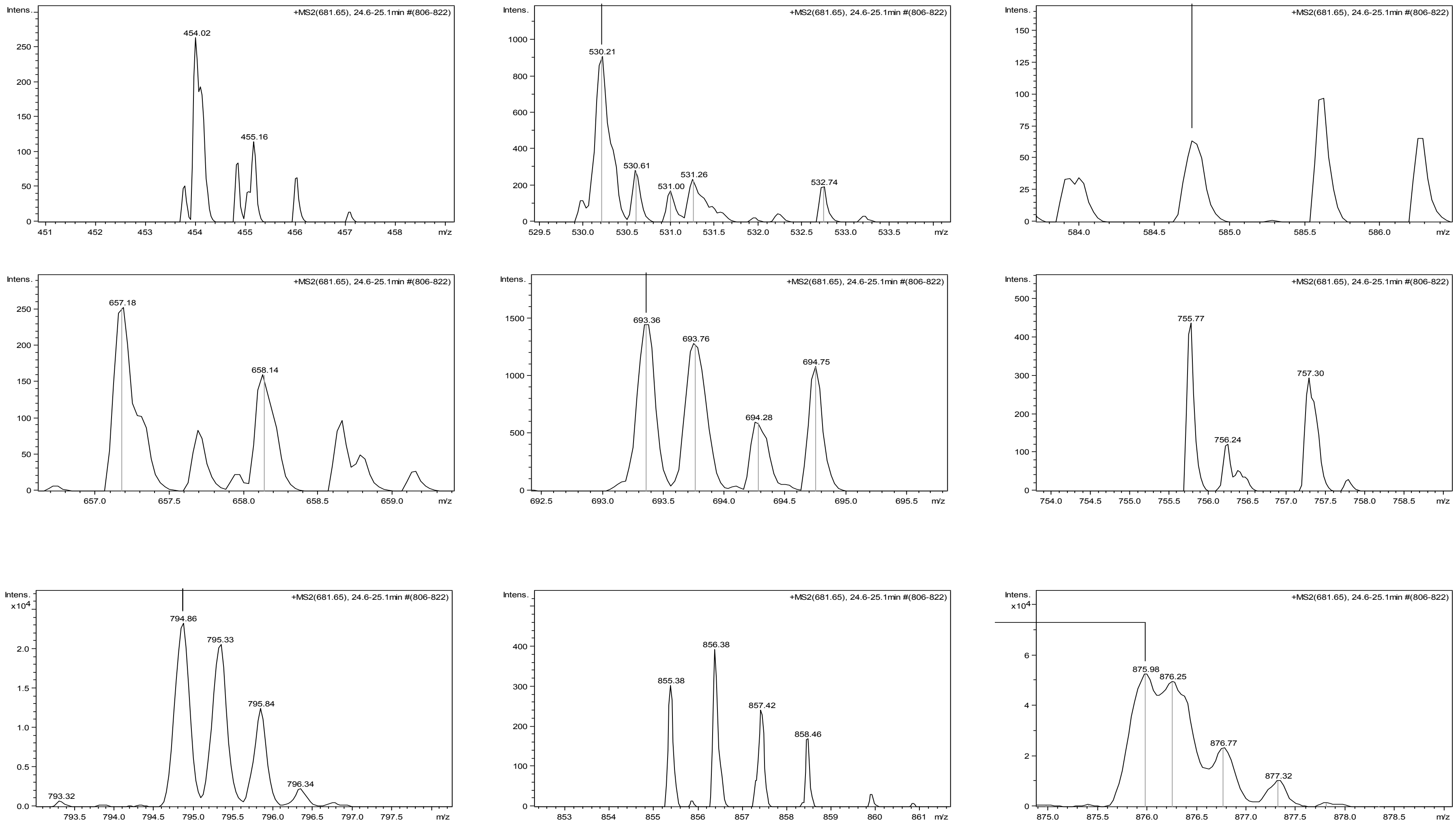

Fraction 16

681.35+++ → Pep [M+2H]++ 693.36++ [24.6-25.1 min] CID-MS2

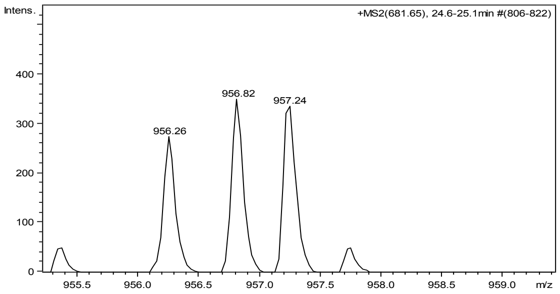

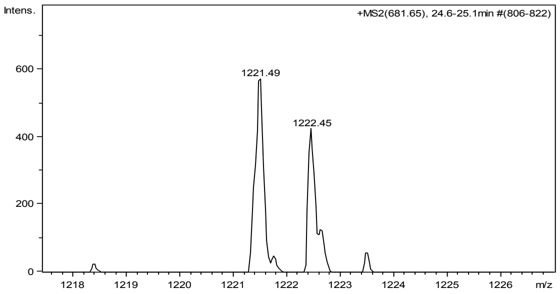

Fraction 16

681.35+++ → Pep [M+2H]++ 693.36++ [24.6-25.1 min] CID-MS3

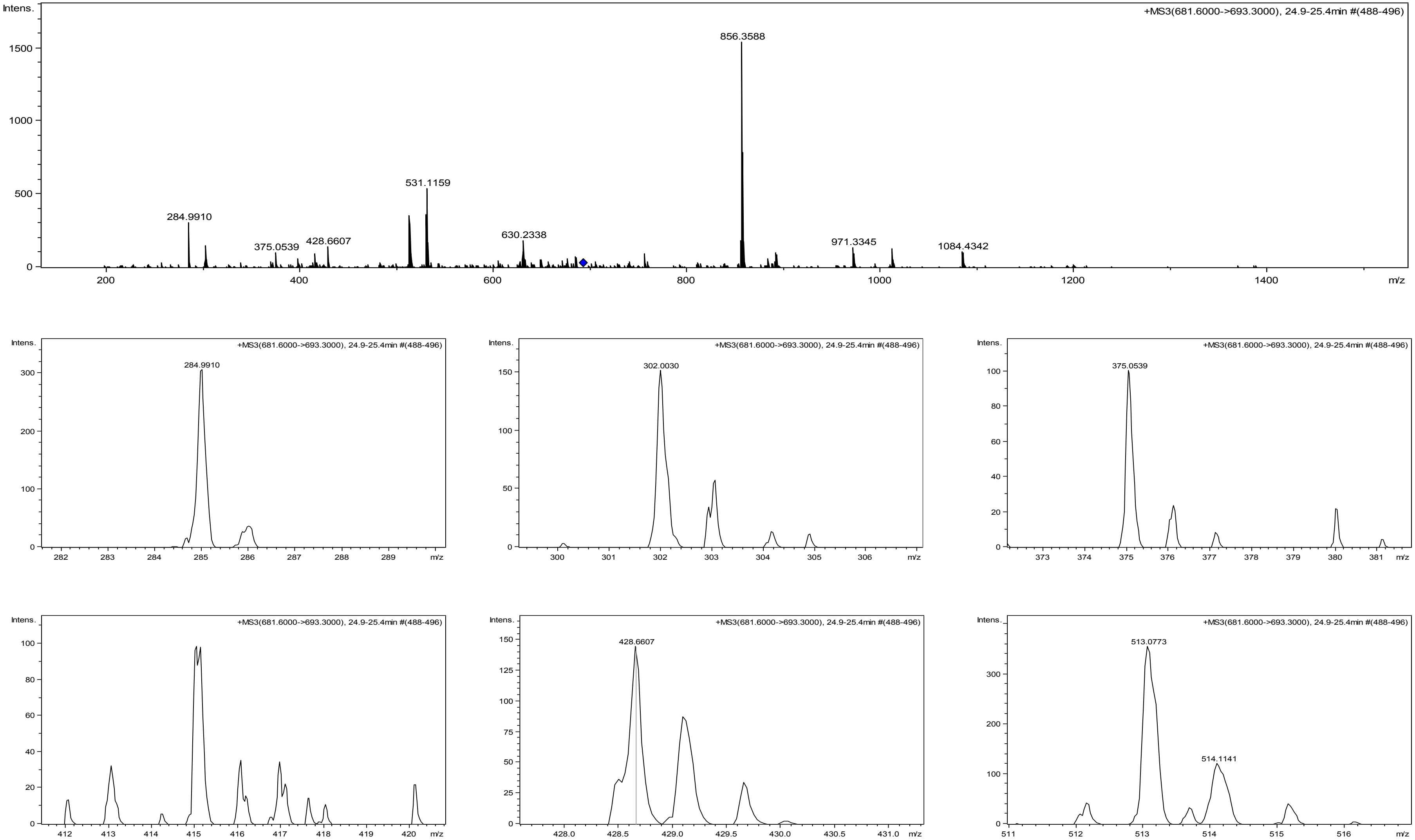

Fraction 16

681.35+++ → Pep [M+2H]++ 693.36++ [24.6-25.1 min] CID-MS3

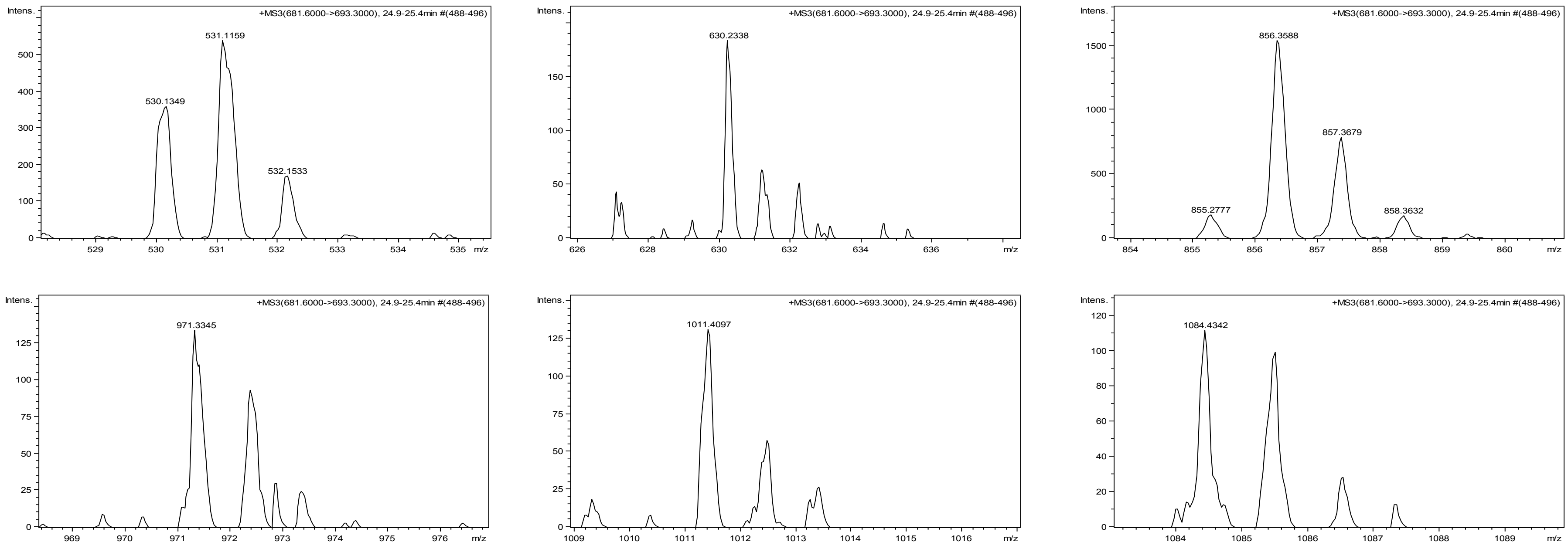

Fraction 16

681.35+++ → Pep [M+2H]++ 693.36++ [24.6-25.1 min]

CID-MS3 MASCOT Search

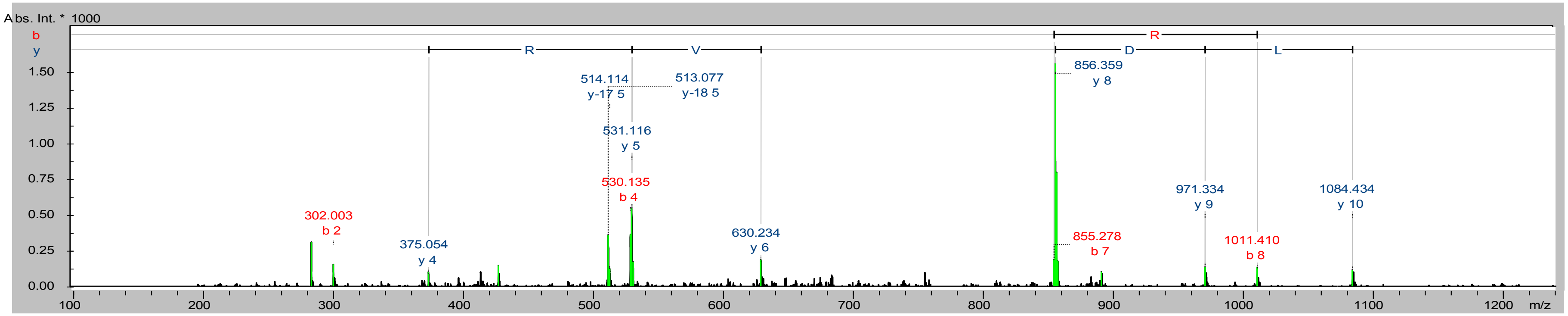

	W	D	L	D	P	E	V	R	P	T	S	A	Trp	Asp	Leu	Asp	Pro	Glu	Val	Arg	Pro	Thr	Ser	Ala
Ion	1	2	3	4	5	6	7	8	9	10	11	12	1	2	3	4	5	6	7	8	9	10	11	12
b	W	D	L	D	P	E	V	R	P	T	S	A	187.087	302.114	415.198	530.225	627.277	756.320	855.388	1011.489	1108.542	1209.590	1296.622	1367.659
b-17	W	D	L	D	P	E	V	R	P	T	S	A	-	-	-	-	-	-	-	994.463	1091.516	1192.563	1279.595	1350.632
b-18	W	D	L	D	P	E	V	R	P	T	S	A	-	284.103	397.187	512.214	609.267	738.309	837.378	993.479	1090.532	1191.579	1278.611	1349.648
y	W	D	L	D	P	E	V	R	P	T	S	A	90.055	177.087	278.135	375.187	531.289	630.357	759.400	856.452	971.479	1084.563	1199.590	1385.670
y-17	W	D	L	D	P	E	V	R	P	T	S	A	-	-	-	-	514.262	613.330	742.373	839.426	954.453	1067.537	1182.564	1368.643
y-18	W	D	L	D	P	E	V	R	P	T	S	A	-	159.076	260.124	357.177	513.278	612.346	741.389	838.442	953.469	1066.553	1181.580	1367.659
	12	11	10	9	8	7	6	5	4	3	2	1	Ala	Ser	Thr	Pro	Arg	Val	Glu	Pro	Asp	Leu	Asp	Trp

Enrichment of glycopeptides for glycan structure and attachment site identification
Nilsson et al., Nature Methods 6, 809 - 811 (2009)

known O-glycosylation site

Apolipoprotein C-III precursor (Apo-CIII)

$_{85}$WDLDPEVRP**T**SA$_{96}$

Fraction 16

681.35+++ → Pep [M+2H]++ 693.36++ [24.6-25.1 min]

CID-MS3 MASCOT Search

prot_hit_nur	prot_acc	prot_desc	prot_score	prot_mass	prot_matche	pep_query	pep_rank	pep_isbold	pep_exp_mz	pep_exp_mr	pep_exp_z	pep_calc_mr	pep_delta	pep_miss	pep_score	pep_expect	pep_res_bef	pep_seq
1	APOC3_HUM	Apolipoprot	45	10846	1	1	1	1	693.3595	1384.7044	2	1384.6623	0.0422	0	46.03	0.46	F	WDLDPEVRP
2	NMUR2_HUN	Neuromedin	25	48208	1	1	2	0	693.3595	1384.7044	2	1384.6511	0.0534	0	30.35	17	C	HFVELTEDIGI
3	CEBPD_HUM	CCAAT/enha	18	28632	1	1	3	0	693.3595	1384.7044	2	1384.7423	-0.0378	0	21.82	1.20E+02	H	QRVEQLTRDI
4	5HT1E_HUM	5-hydroxytry	15	42339	1	1	7	0	693.3595	1384.7044	2	1384.8541	-0.1497	0	20.36	1.70E+02	V	ITTLTTLLNLA
5	MPP4_HUM	MAGUK p55	15	73561	1	1	4	0	693.3595	1384.7044	2	1384.7133	-0.0088	0	21.69	1.20E+02	I	KRHEMTGDIL
6	DCJ11_HUM	DnaJ homolo	15	63524	1	1	8	0	693.3595	1384.7044	2	1383.7146	0.9898	0	19.99	1.80E+02	D	YYSLLNVRRE
7	LA_HUMAN	Lupus La pro	14	46979	1	1	10	0	693.3595	1384.7044	2	1384.7059	-0.0014	0	19.56	2.00E+02	D	ENGATGPVK
8	NRK_HUMAN	Nik-related	13	179509	1	1	6	0	693.3595	1384.7044	2	1383.7333	0.9712	0	20.6	1.60E+02	N	MLQHPFVRD
9	TCF20_HUM	Transcription	11	213123	1	1	9	0	693.3595	1384.7044	2	1384.7674	-0.063	0	19.66	2.00E+02	E	KRKGEVASDI

Biotools-Score: 15

MASCOT-Score: 46

Enrichment of glycopeptides for glycan structure and attachment site identification
Nilsson et al., Nature Methods 6, 809 - 811 (2009)

known O-glycosylation site

Apolipoprotein C-III precursor (Apo-CIII)

$_{85}$WDLDPEVRPTSA$_{96}$

Fraction 16

681.35+++ → Pep [M+2H]++ 693.36++ [24.6-25.1 min] ETD

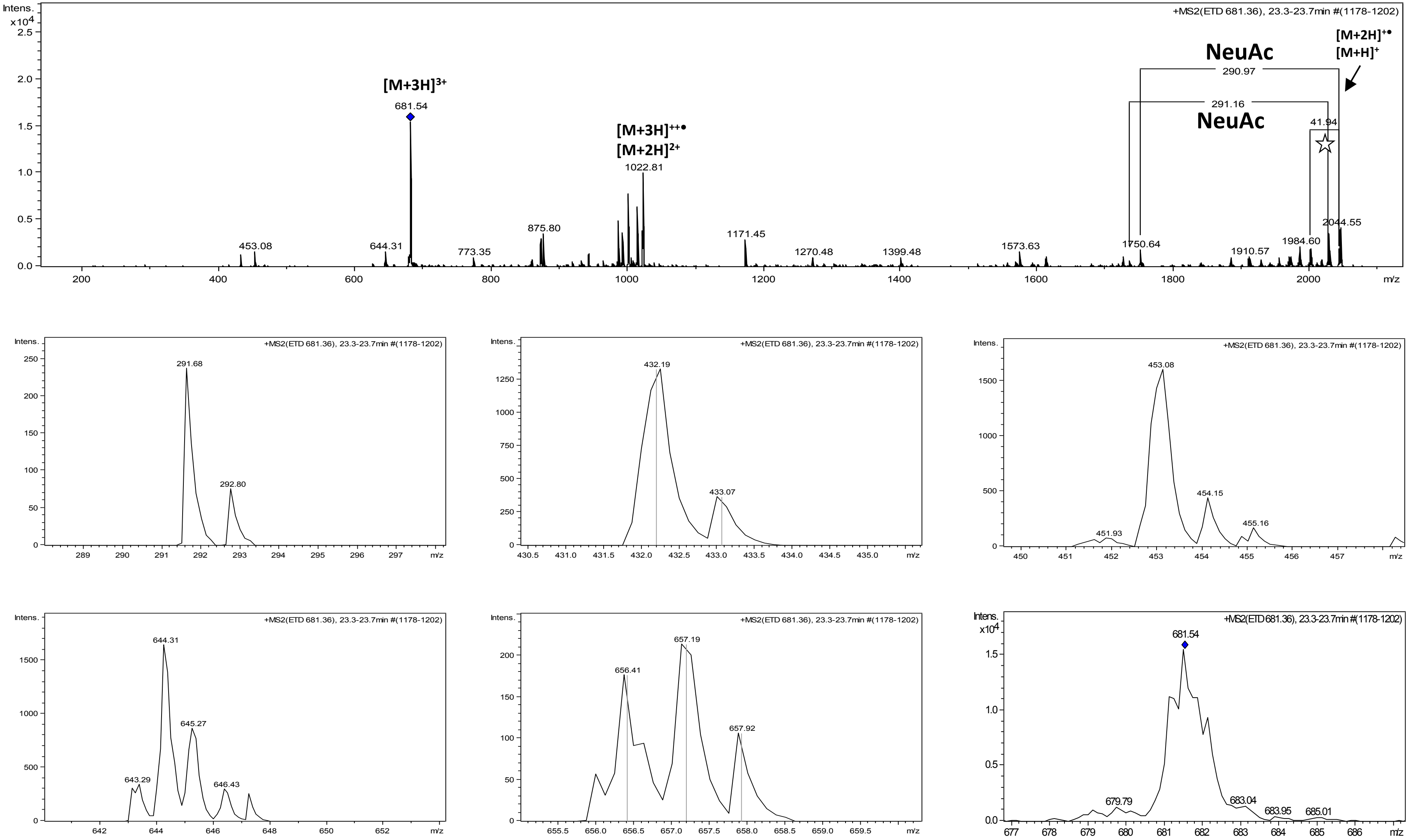

Fraction 16

681.35+++ → Pep [M+2H]++ 693.36++ [24.6-25.1 min]

ETD

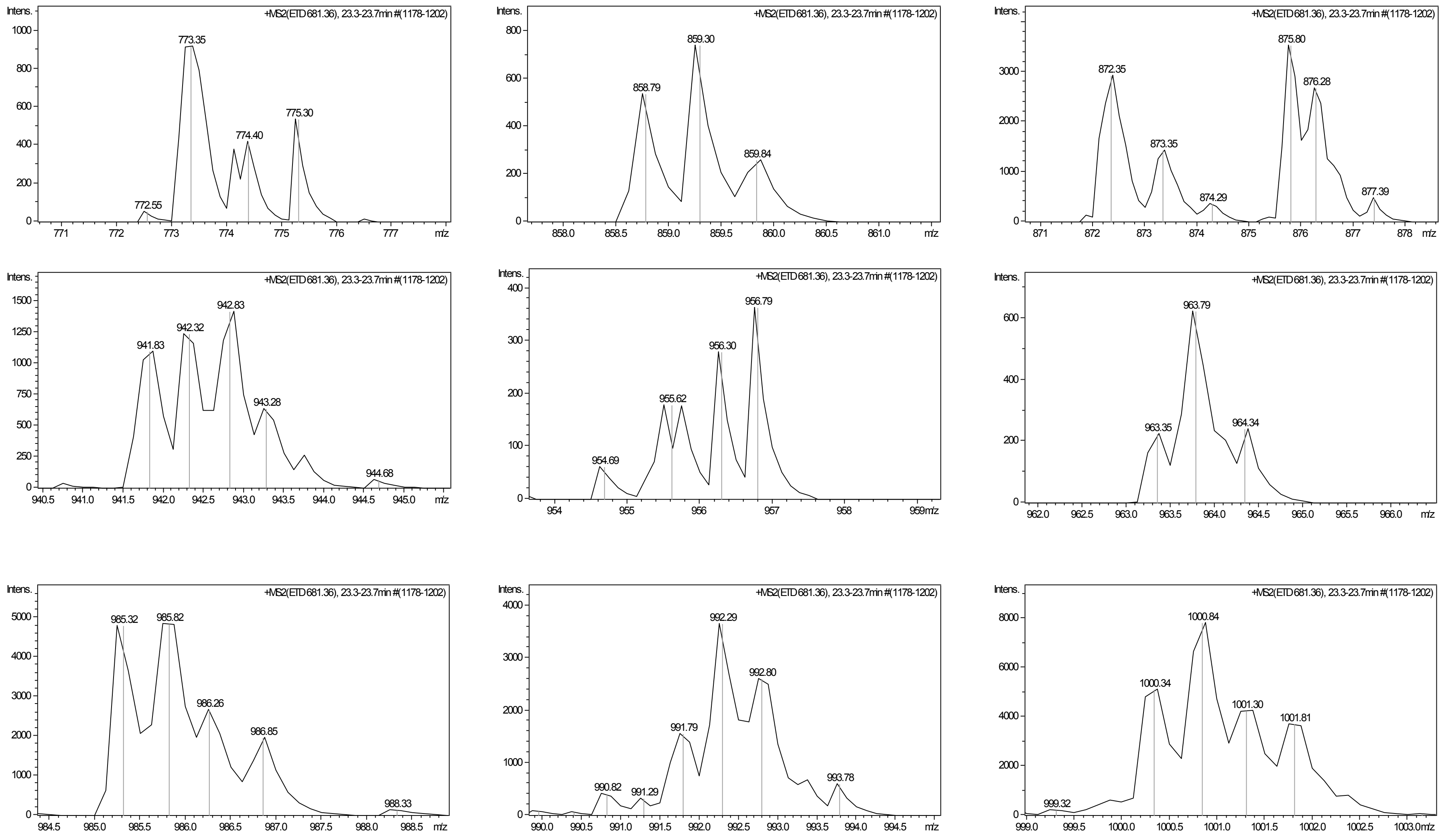

Fraction 16

681.35+++ → Pep [M+2H]++ 693.36++ [24.6-25.1 min] ETD

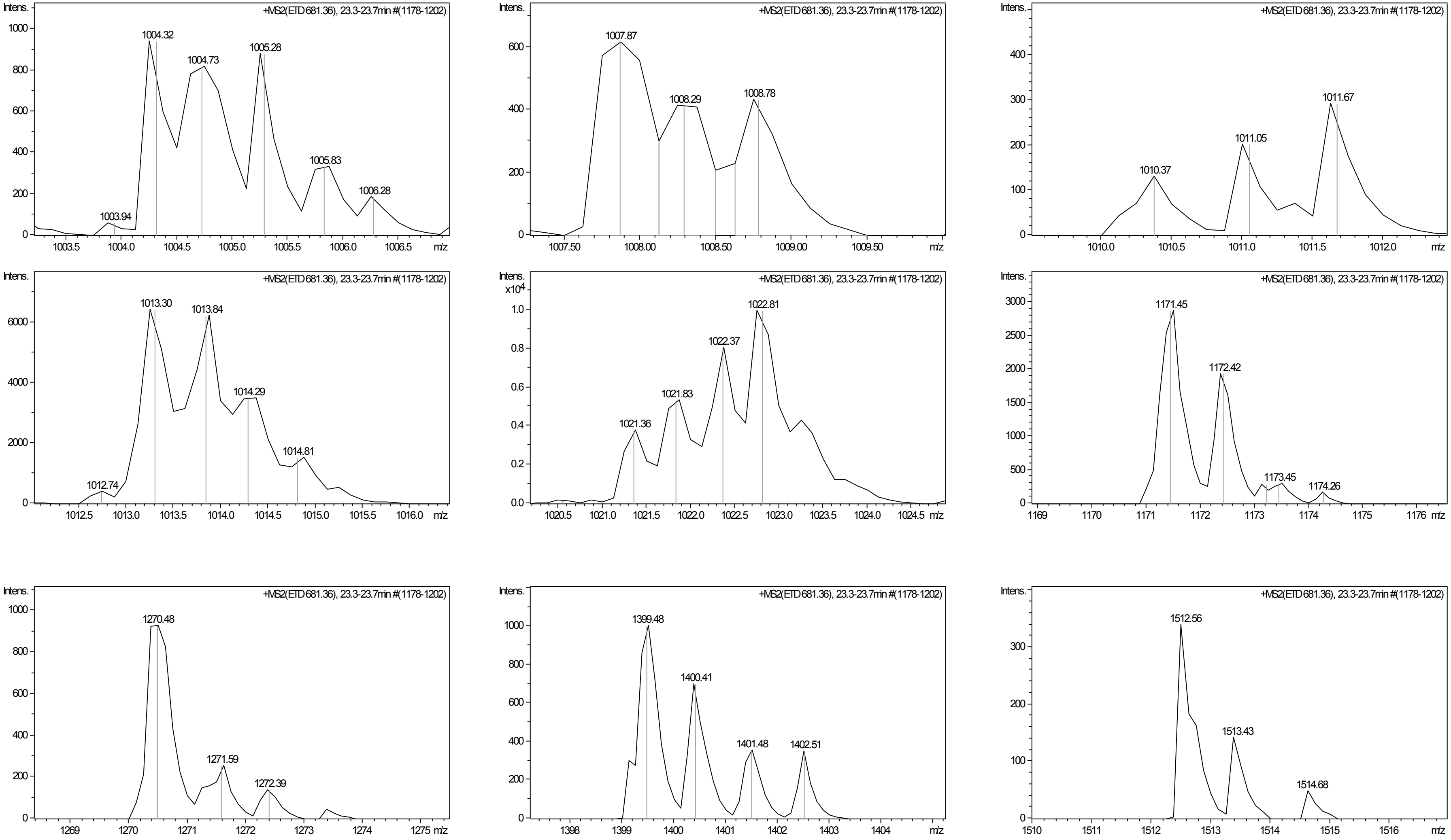

Fraction 16

681.35+++ → Pep [M+2H]++ 693.36++ [24.6-25.1 min] ETD

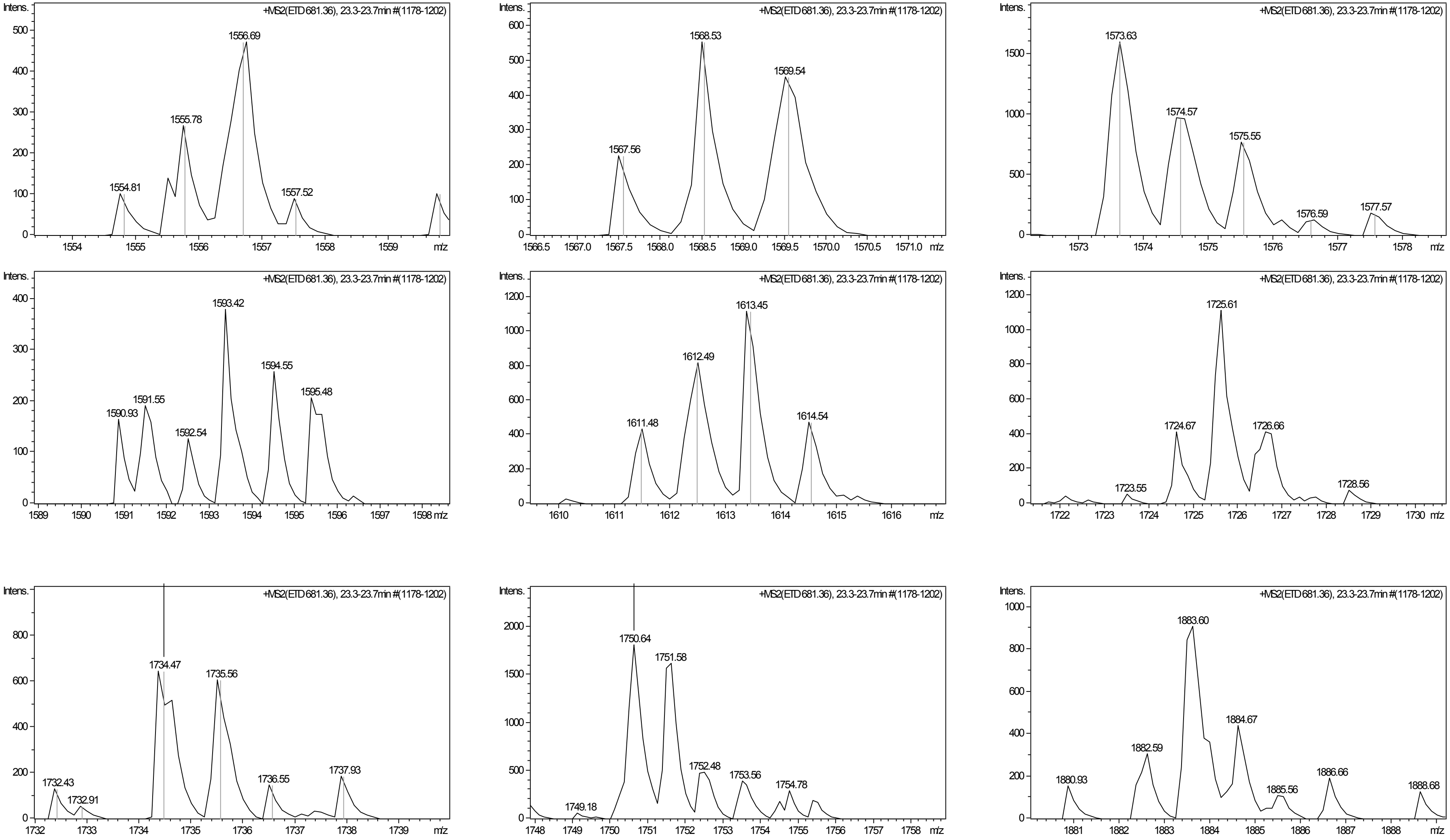

Fraction 16

681.35+++ → Pep [M+2H]++ 693.36++ [24.6-25.1 min] ETD

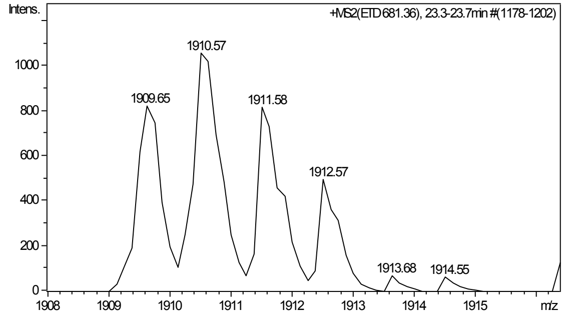

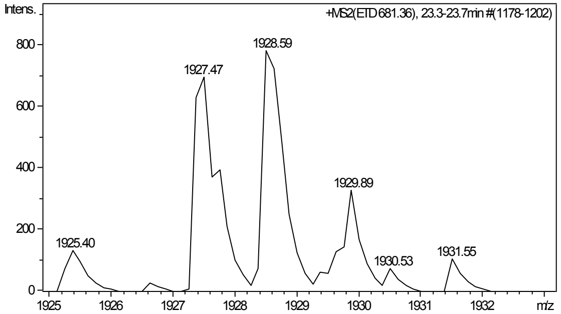

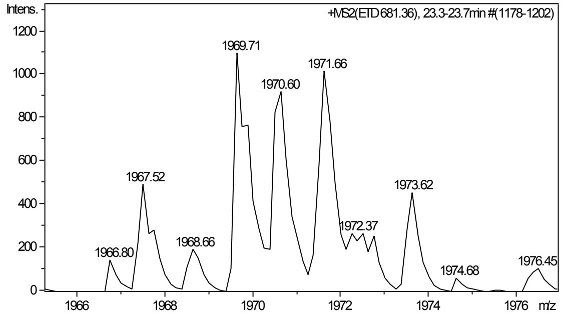

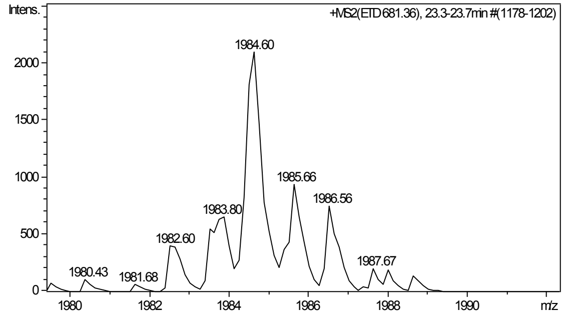

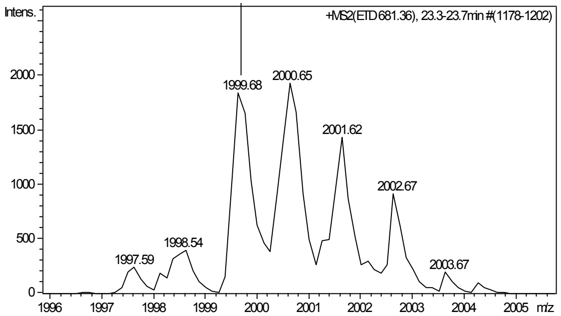

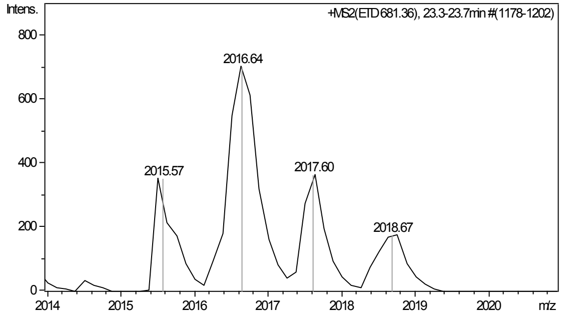

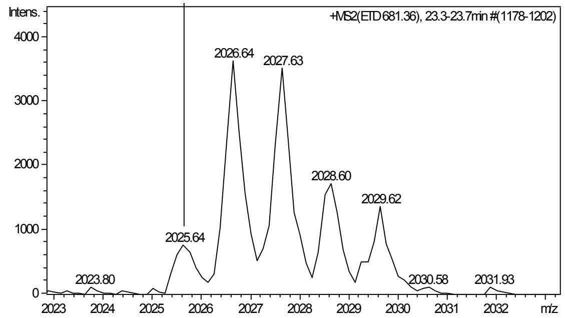

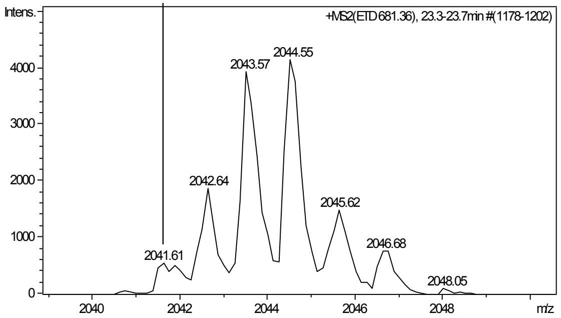

Fraction 16

681.35+++ → Pep [M+2H]++ 693.36++ [24.6-25.1 min] ETD

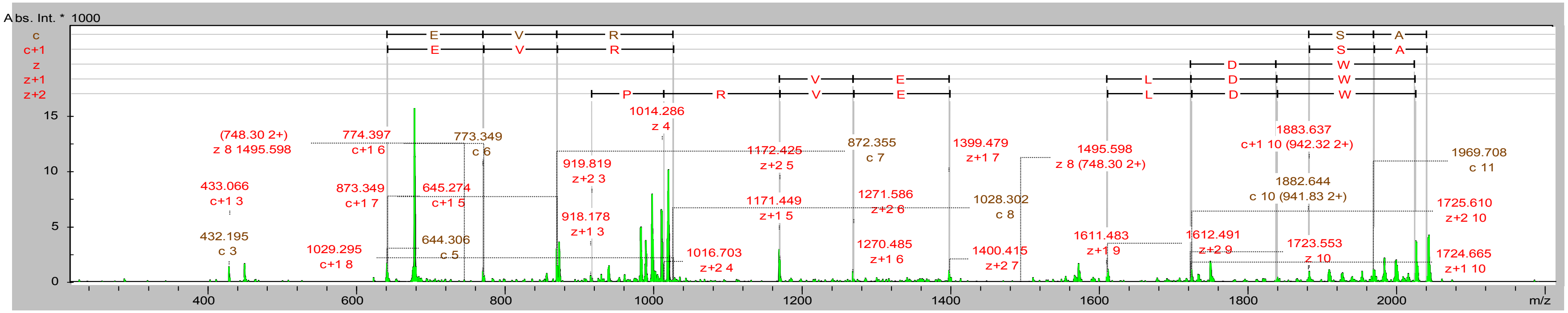

WDLDPEVRP<u>T</u>SA **Known O-Glycosylation site**

	W	D	L	D	P	E	V	R	P	T	S	A	Trp	Asp	Leu	Asp	Pro	Glu	Val	Arg	Pro	Thr	Ser	Ala
Ion	1	2	3	4	5	6	7	8	9	10	11	12	1	2	3	4	5	6	7	8	9	10	11	12
c	W	D	L	D	P	E	V	R	P	T*	S	A	204.113	319.140	432.224	547.251	644.304	773.346	872.415	1028.516	1125.569	1882.844	1969.876	2040.913
c+1	W	D	L	D	P	E	V	R	P	T*	S	A	205.121	320.148	433.232	548.259	645.312	774.354	873.423	1029.524	1126.577	1883.852	1970.884	2041.921
z	W	D	L	D	P	E	V	R	P	T*	S	A	73.028	160.060	917.336	1014.388	1170.490	1269.558	1398.601	1495.653	1610.680	1723.764	1838.791	2024.871
z+1	W	D	L	D	P	E	V	R	P	T*	S	A	74.036	161.068	918.344	1015.396	1171.497	1270.566	1399.608	1496.661	1611.688	1724.772	1839.799	2025.878
z+2	W	D	L	D	P	E	V	R	P	T*	S	A	75.044	162.076	919.351	1016.404	1172.505	1271.574	1400.616	1497.669	1612.696	1725.780	1840.807	2026.886
	12	11	10	9	8	7	6	5	4	3	2	1	Ala	Ser	Thr	Pro	Arg	Val	Glu	Pro	Asp	Leu	Asp	Trp

Biotools-Score: 124

Enrichment of glycopeptides for glycan structure and attachment site identification
Nilsson et al., Nature Methods 6, 809 - 811 (2009)

known O-glycosylation site

Apolipoprotein C-III precursor (Apo-CIII) $_{85}$WDLDPEVRP**T**SA$_{96}$

Fraction 16

681.35+++ → Pep [M+2H]++ 693.36++ [24.6-25.1 min] ETD

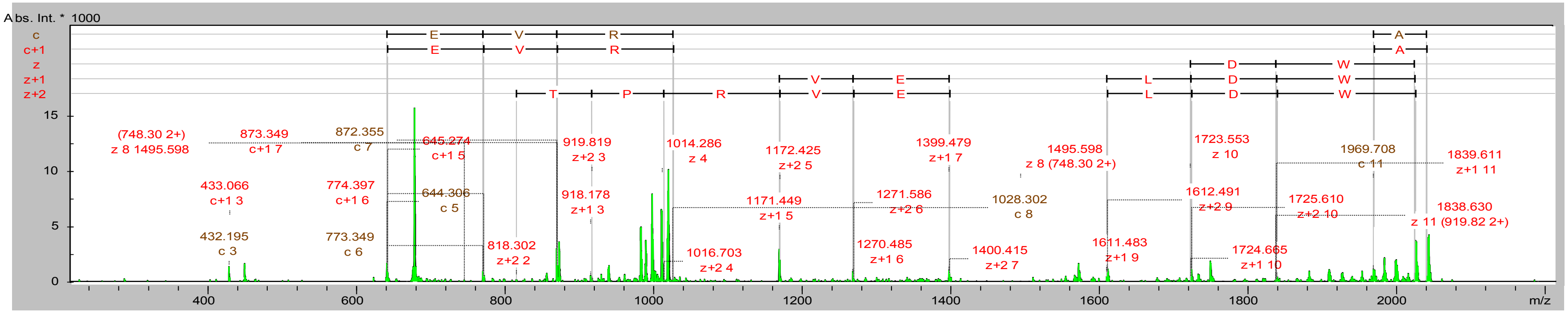

WDLDPEVRPT**S**A

	W	D	L	D	P	E	V	R	P	T	S	A	Trp	Asp	Leu	Asp	Pro	Glu	Val	Arg	Pro	Thr	Ser	Ala
Ion	1	2	3	4	5	6	7	8	9	10	11	12	1	2	3	4	5	6	7	8	9	10	11	12
c	W	D	L	D	P	E	V	R	P	T	S*	A	204.113	319.140	432.224	547.251	644.304	773.346	872.415	1028.516	1125.569	1226.616	1969.876	2040.913
c+1	W	D	L	D	P	E	V	R	P	T	S*	A	205.121	320.148	433.232	548.259	645.312	774.354	873.423	1029.524	1126.577	1227.624	1970.884	2041.921
z	W	D	L	D	P	E	V	R	P	T	S*	A	73.028	816.288	917.336	1014.388	1170.490	1269.558	1398.601	1495.653	1610.680	1723.764	1838.791	2024.871
z+1	W	D	L	D	P	E	V	R	P	T	S*	A	74.036	817.296	918.344	1015.396	1171.497	1270.566	1399.608	1496.661	1611.688	1724.772	1839.799	2025.878
z+2	W	D	L	D	P	E	V	R	P	T	S*	A	75.044	818.304	919.351	1016.404	1172.505	1271.574	1400.616	1497.669	1612.696	1725.780	1840.807	2026.886
	12	11	10	9	8	7	6	5	4	3	2	1	Ala	Ser	Thr	Pro	Arg	Val	Glu	Pro	Asp	Leu	Asp	Trp

Biotools-Score: 148

O-glycosylation of Ser(95) unlikely:

- 818.304 m/z, signal too low
- 1882.84 m/z, detected, confirms presence of unmodiefed Ser(95)

Enrichment of glycopeptides for glycan structure and attachment site identification
Nilsson et al., Nature Methods 6, 809 - 811 (2009)

known O-glycosylation site

Apolipoprotein C-III precursor (Apo-CIII)

$_{85}$WDLDPEVRP**T**SA$_{96}$

Fraction 16

685.28+++ → Pep+HexNAc [M+2H]++ 800.82++ [26.6-26.9 min]

CID-MS Precursor

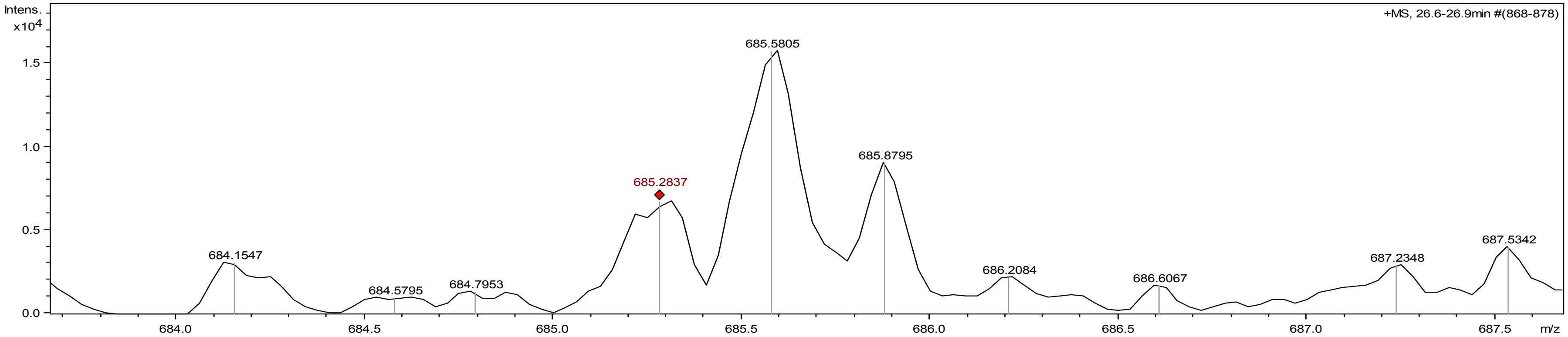

Fraction 16

685.28+++ → Pep+HexNAc [M+2H]++ 800.82++ [26.6-26.9 min]

CID-MS2

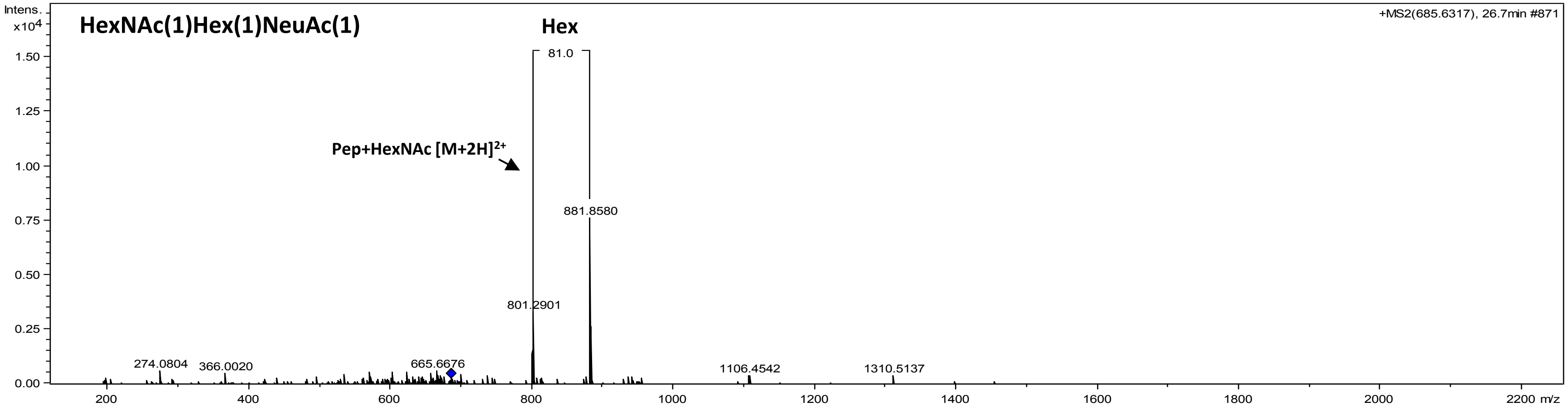

No reasonable result

Fraction 16

685.28+++ → Pep+HexNAc [M+2H]++ 800.82++ [26.6-26.9 min]

CID-MS3

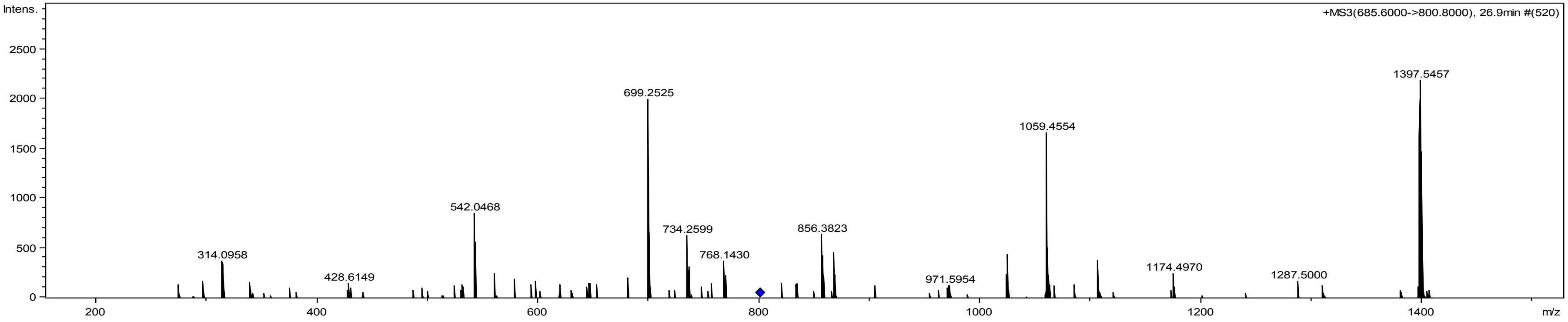

No reasonable result

Fraction 16

872.73+++ → Pep [M+H]+ 1668.77+ [31.3-31.7 min] CID-MS Precursor

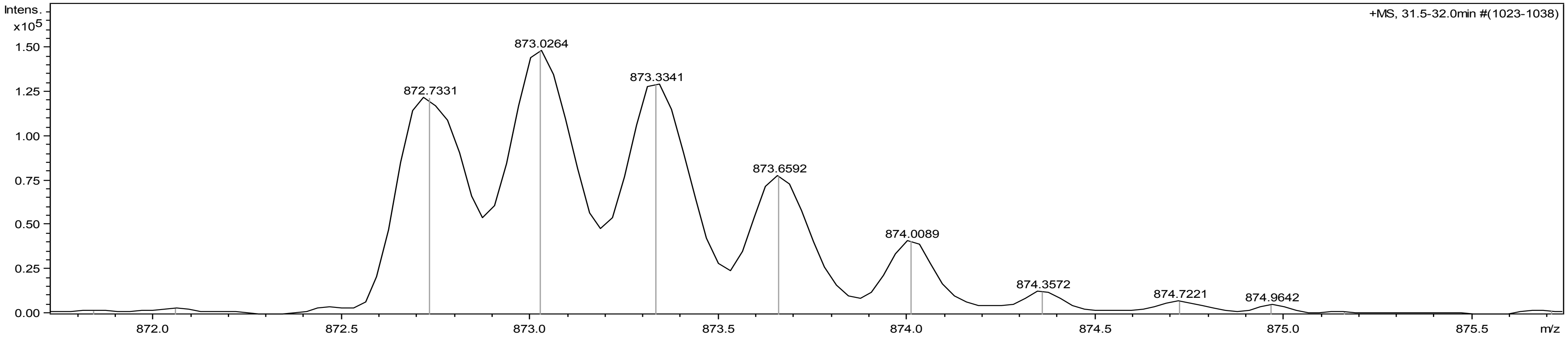

MASCOT Search also on CID-MS^2 spectrum, For MASCOT search m/z of the unmodified peptide [M+H]$^+$ has to be given

Many b- and y-ions in CID MS^2 spectrum

Fraction 16

872.73+++ → Pep [M+H]+ 1668.77+ [31.3-31.7 min] CID-MS2

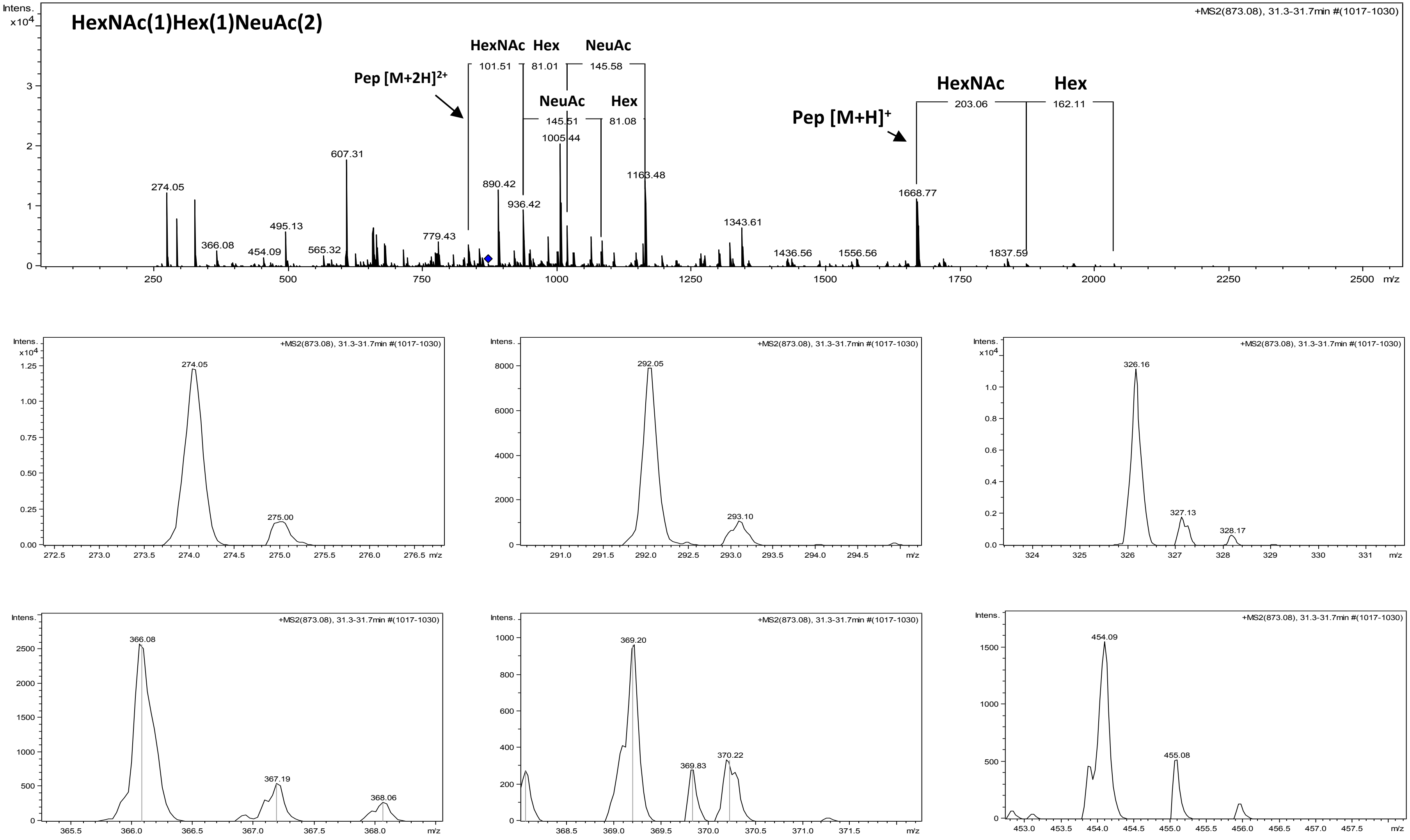

Fraction 16

872.73+++ → Pep [M+H]+ 1668.77+ [31.3-31.7 min] CID-MS2

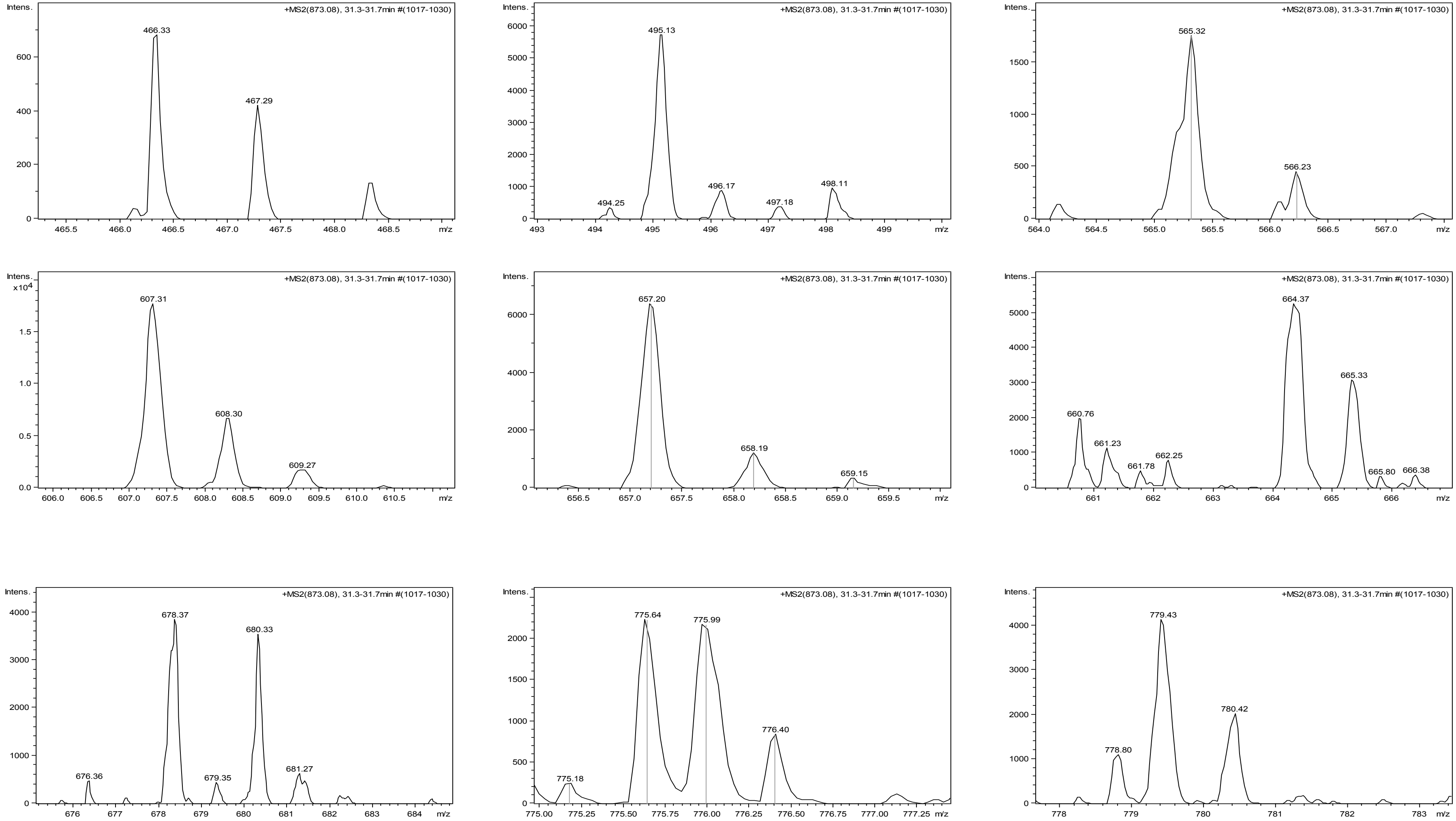

Fraction 16

872.73+++ → Pep [M+H]+ 1668.77+ [31.3-31.7 min]

CID-MS2

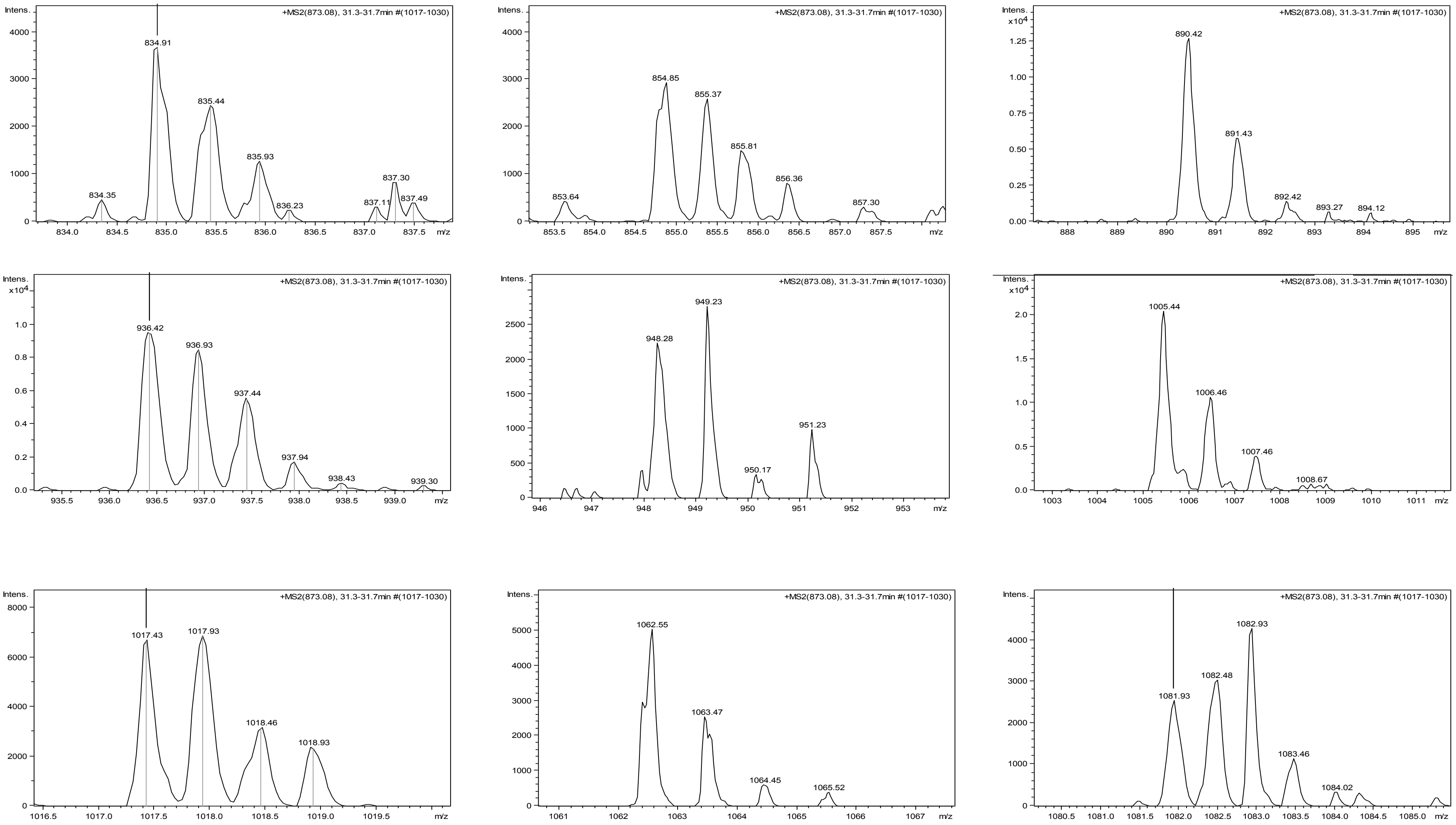

Fraction 16

872.73+++ → Pep [M+H]+ 1668.77+ [31.3-31.7 min]

CID-MS2

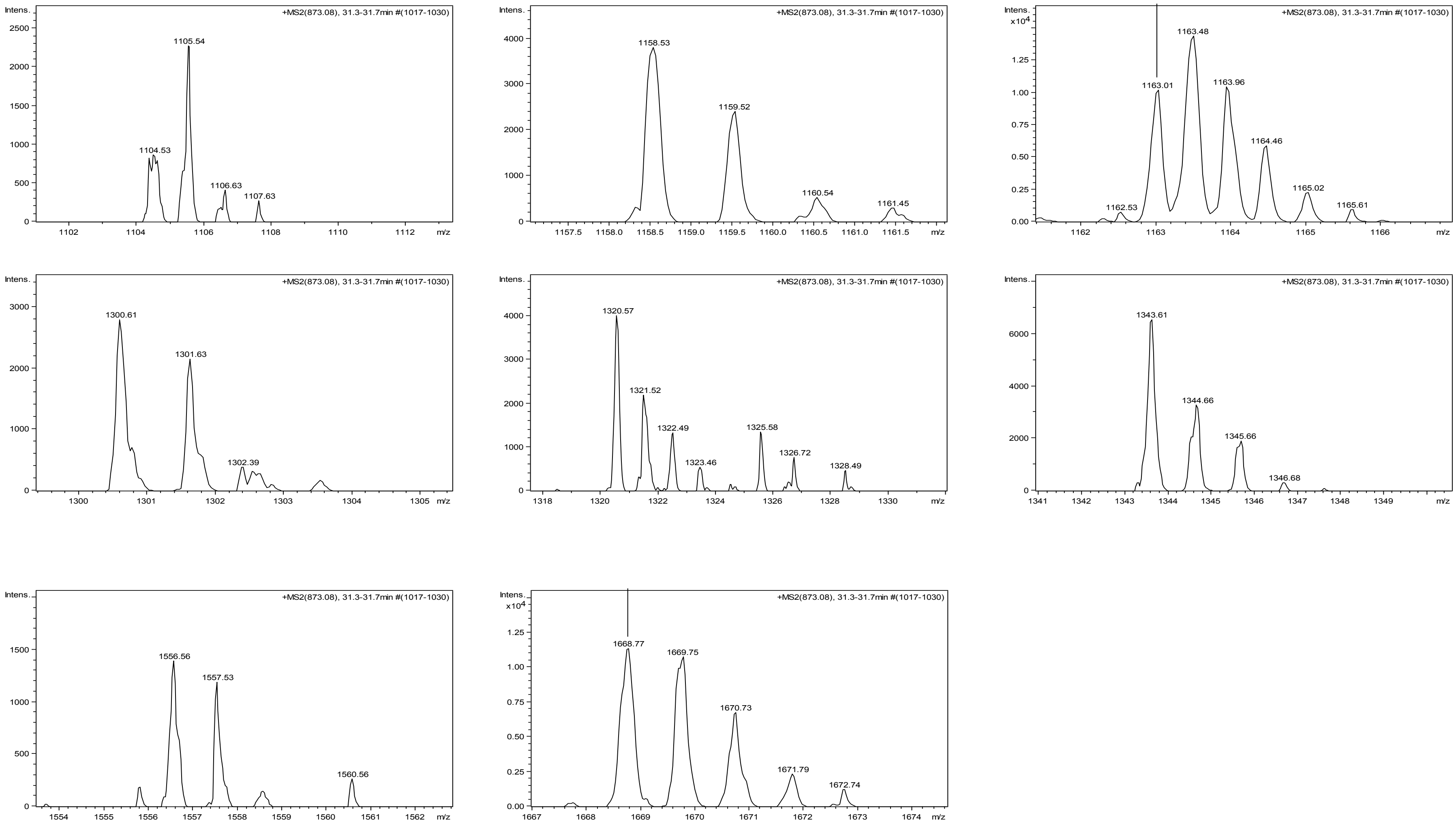

Fraction 16

872.73+++ → Pep [M+H]+ 1668.77+ [31.3-31.7 min]

CID-MS2 MASCOT Search

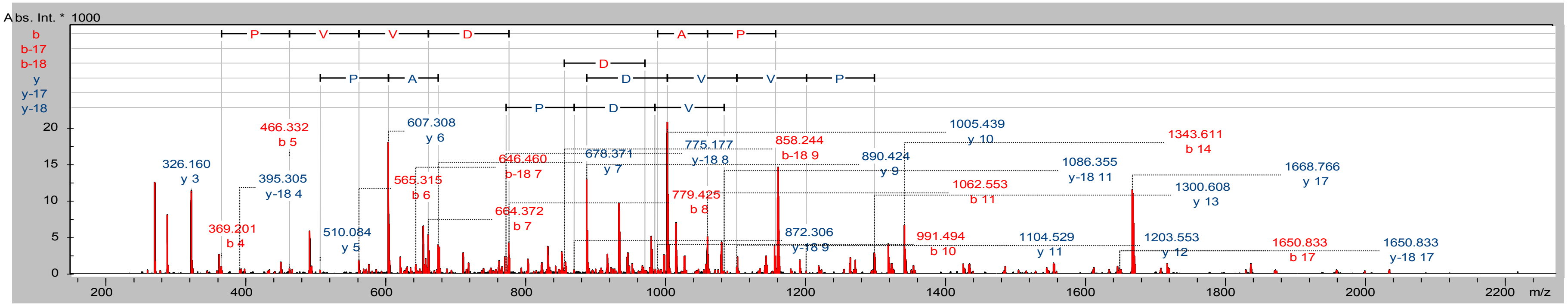

	A	V	P	T	P	V	V	D	P	D	A	P	P	S	P	P	L
Ion	1	2	3	4	5	6	7	8	9	10	11	12	13	14	15	16	17
b	A	V	P	T	P	V	V	D	P	D	A	P	P	S	P	P	L
b-17	A	V	P	T	P	V	V	D	P	D	A	P	P	S	P	P	L
b-18	A	V	P	T	P	V	V	D	P	D	A	P	P	S	P	P	L
y	A	V	P	T	P	V	V	D	P	D	A	P	P	S	P	P	L
y-17	A	V	P	T	P	V	V	D	P	D	A	P	P	S	P	P	L
y-18	A	V	P	T	P	V	V	D	P	D	A	P	P	S	P	P	L
	17	16	15	14	13	12	11	10	9	8	7	6	5	4	3	2	1

	Ala	Val	Pro	Thr	Pro	Val	Val	Asp	Pro	Asp	Ala	Pro	Pro	Ser	Pro	Pro	Leu
Ion	1	2	3	4	5	6	7	8	9	10	11	12	13	14	15	16	17
b	72.044	171.113	268.166	369.213	466.266	565.334	664.403	779.430	876.483	991.509	1062.547	1159.599	1256.652	1343.684	1440.737	1537.790	1650.874
b-17	-	-	-	-	-	-	-	-	-	-	-	-	-	-	-	-	-
b-18	-	-	-	351.203	448.255	547.324	646.392	761.419	858.472	973.499	1044.536	1141.589	1238.642	1325.674	1422.726	1519.779	1632.863
y	132.102	229.155	326.207	413.239	510.292	607.345	678.382	793.409	890.462	1005.489	1104.557	1203.626	1300.678	1401.726	1498.779	1597.847	1668.884
y-17	-	-	-	-	-	-	-	-	-	-	-	-	-	-	-	-	-
y-18	-	-	-	395.229	492.282	589.334	660.372	775.398	872.451	987.478	1086.547	1185.615	1282.668	1383.715	1480.768	1579.837	1650.874
	Leu	Pro	Pro	Ser	Pro	Pro	Ala	Asp	Pro	Asp	Val	Val	Pro	Thr	Pro	Val	Ala

For MASCOT search m/z of the unmodified peptide $[M+H]^+$ has to be given

known O-glycosylation site

Alpha-2-HS-glycoprotein

$_{267}$AVP**T**PVVDPDAPPSPPL$_{283}$

Fraction 16

872.73+++ → Pep [M+H]+ 1668.77+ [31.3-31.7 min]

CID-MS2 MASCOT Search

prot_hit_nur	prot_acc	prot_desc	prot_scoı	prot_mass	pr	pr	pr	pr	pe	pe	pe	pe	pep_exp_mz	pep_exp_mr	pep	pep_calc_mr	pep_delta	pep_ı	pep_score	pep_expect	pep_res_bef	pep_seq
1	FDXA1_HUM	Ferredoxin-f	8	71625	1	0	1	0	1	1	1	1	1668.77	1667.7627	1	1666.9141	0.8486	0	14.22	1.00E+03	L	LDHLKGILDSLLTQT
2	MAP11_HUM	Methionine a	7	44100	1	0	1	0	1	3	0	1	1668.77	1667.7627	1	1667.9107	-0.148	0	10.62	2.30E+03	N	IIQKHAQANGFSVVR
3	SCRB2_HUM	Lysosome m	5	54712	1	0	1	0	1	2	0	1	1668.77	1667.7627	1	1667.6556	0.1071	0	10.74	2.30E+03	S	DNAGFCIPEGNCLGSG
4	FETUA_HUM	Alpha-2-HS-g	3	40098	1	0	1	0	1	6	0	1	1668.77	1667.7627	1	1667.8771	-0.1143	0	8.71	3.60E+03	E	AVPTPVVDPDAPPSPPL
5	TTC13_HUM	Tetratricope	2	97607	1	0	1	0	1	4	0	1	1668.77	1667.7627	1	1667.7679	-0.0051	0	9.99	2.70E+03	L	IEDYEEQPGLQPHI
6	CBPB1_HUM	Carboxypept	2	47737	1	0	1	0	1	#	0	1	1668.77	1667.7627	1	1666.7006	1.0622	0	7.48	4.80E+03	T	GSSCIGTDPNRNFDAG
7	ADAT1_HUMAN		2	56156	1	0	1	0	1	7	0	1	1668.77	1667.7627	1	1667.8155	-0.0528	0	8.2	4.10E+03	A	VPEQPLDVTANGFPQG
8	C144B_HUMAN		1	83698	1	0	1	0	1	8	0	1	1668.77	1667.7627	1	1667.7374	0.0253	0	7.8	4.50E+03	N	STELSGTLTDGTTVGND
9	DYH11_HUMAN		0	524873	1	0	1	0	1	5	0	1	1668.77	1667.7627	1	1667.8916	-0.1289	0	9.9	2.80E+03	L	LGELPPGDRQKIMTI

Biotools-Score: 558

MASCOT-Score: 9

known O-glycosylation site

Alpha-2-HS-glycoprotein

$_{267}$AVP**T**PVVDPDAPPSPPL$_{283}$

Fraction 16

872.73+++ → Pep [M+H]+ 1668.77+ [31.3-31.7 min]

CID-MS2

Internal glycopeptide fragmentation

AVP**T**PVVDPDAPPSPPL
HexNac-

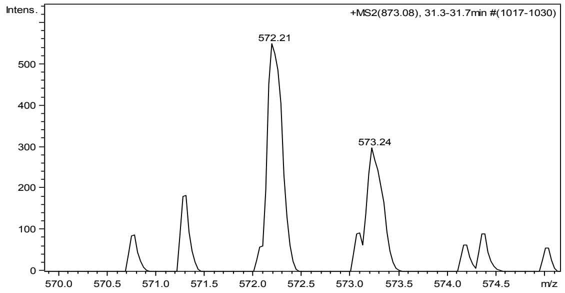

AVP**T**
HexNac-

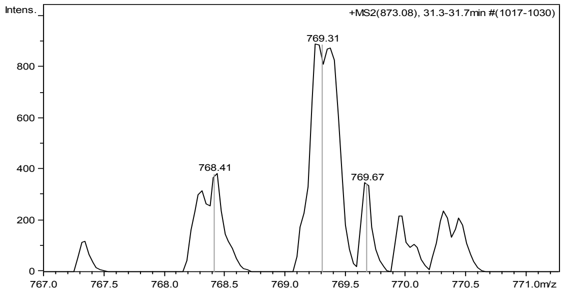

AVP**T**PV
HexNac-

b				y
---	1	**A**	17	---
171.1128	2	**V**	16	1800.9266
268.1656	3	**P**	15	1701.8582
572.2926	4	**T(HexNAc)**	14	1604.8054
669.3454	5	**P**	13	1300.6783
768.4138	6	**V**	12	1203.6256
867.4822	7	**V**	11	1104.5572
982.5092	8	**D**	10	1005.4888
1079.5619	9	**P**	9	890.4618
1194.5889	10	**D**	8	793.4090
1265.6260	11	**A**	7	678.3821
1362.6787	12	**P**	6	607.3450
1459.7315	13	**P**	5	510.2922
1546.7635	14	**S**	4	413.2395
1643.8163	15	**P**	3	326.2074
1740.8691	16	**P**	2	229.1547
---	17	**L**	1	132.1019

These fragment ions are not present in the MS^3 peptide spectrum. This indicates that they are derived from glycopeptide fragmentation.

Fraction 16

872.73+++ → Pep [M+H]+ 1668.77+ [31.3-31.7 min]

CID-MS2

Internal glycopeptide fragmentation

AVP**T**PVVDPDAPPSPPL
HexNac-

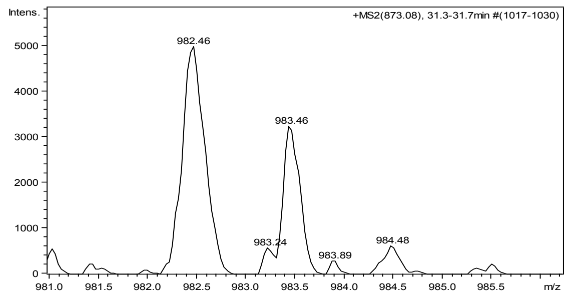

AVP**T**PVVD
HexNac-

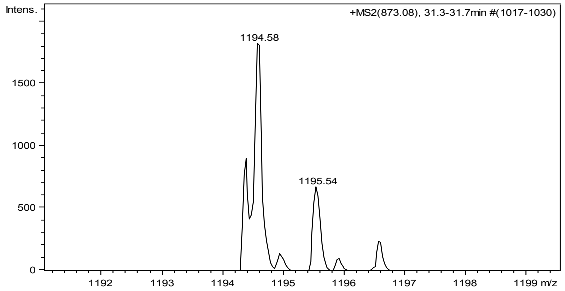

AVP**T**PVVDPD
HexNac-

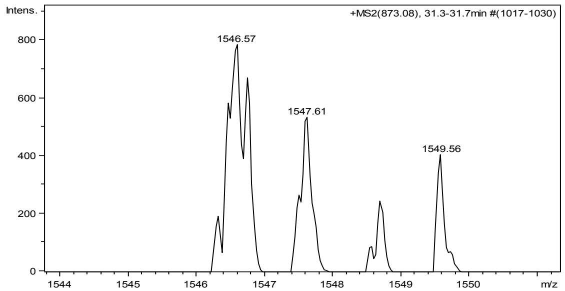

AVP**T**PVVDPDAPPS
HexNac-

Fraction 16

872.73+++ → Pep [M+H]+ 1668.77+ [31.3-31.7 min] CID-MS2

Internal glycopeptide fragmentation

AVPTPVVDPDAPPSPPL

HexNac(1)NeuAc(1)-

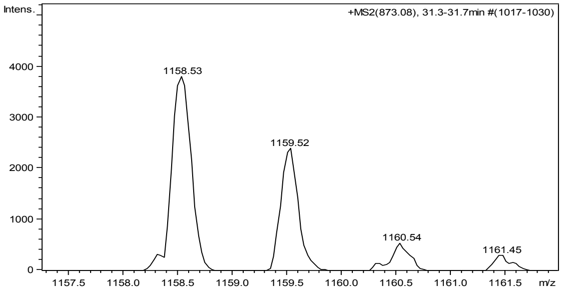

AVPTPVV

HexNac(1)NeuAc(1)-

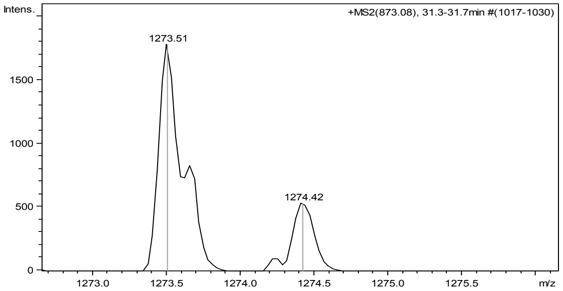

AVPTPVVD

HexNac(1)NeuAc(1)-

b				y
---	1	**A**	17	---
171.1128	2	**V**	16	2092.0220
268.1656	3	**P**	15	1992.9536
863.3880	4	**T(HexNAc(1)NeuAc(1))**	14	1895.9008
960.4408	5	**P**	13	1300.6783
1059.5092	6	**V**	12	1203.6256
1158.5776	7	**V**	11	1104.5572
1273.6046	8	**D**	10	1005.4888
1370.6573	9	**P**	9	890.4618
1485.6843	10	**D**	8	793.4090
1556.7214	11	**A**	7	678.3821
1653.7742	12	**P**	6	607.3450
1750.8269	13	**P**	5	510.2922
1837.8589	14	**S**	4	413.2395
1934.9117	15	**P**	3	326.2074
2031.9645	16	**P**	2	229.1547
---	17	**L**	1	132.1019

These fragment ions are not present in the MS³ peptide spectrum. This indicates that they are derived from glycopeptide fragmentation.

Fraction 16

872.73+++ → Pep [M+H]+ 1668.77+ [31.3-31.7 min]

CID-MS2

Internal glycopeptide fragmentation

AVPTPVVDPDAPPSPPL

HexNac(1)NeuAc(1)-

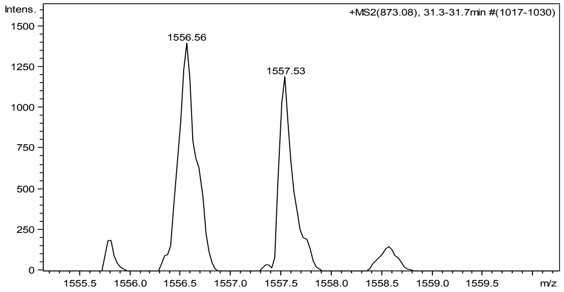

AVPTPVVDPDA

HexNac(1)NeuAc(1)-

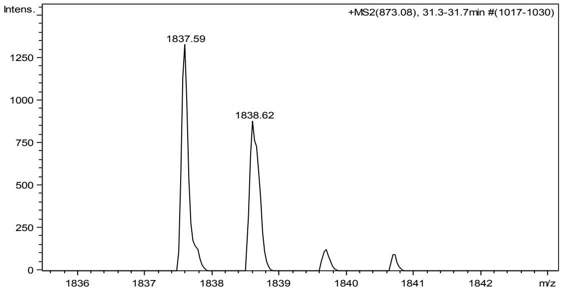

AVPTPVVDPDAPPSPPL

HexNac(1)NeuAc(1)-

Fraction 16

872.73+++ → Pep [M+H]+ 1668.77+ [31.3-31.7 min]

CID-MS2

Internal glycopeptide fragmentation

AVPTPVVDPDAPPSPPL

HexNac(1)Hex(1)-

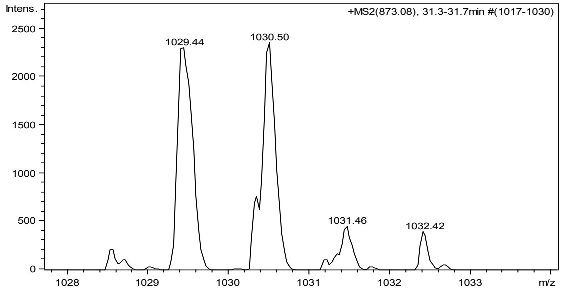

AVPTPVV

HexNac(1)Hex(1)-

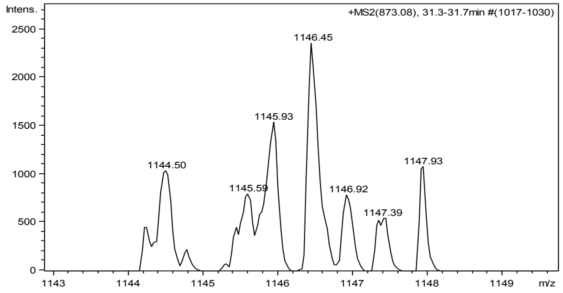

AVPTPVVD

HexNac(1)Hex(1)-

b				y
---	1	A	17	---
171.1128	2	V	16	1962.9794
268.1656	3	P	15	1863.9110
734.3454	4	T(HexNAc(1)Hex(1))	14	1766.8582
831.3982	5	P	13	1300.6783
930.4666	6	V	12	1203.6256
1029.5350	7	V	11	1104.5572
1144.5620	8	D	10	1005.4888
1241.6147	9	P	9	890.4618
1356.6417	10	D	8	793.4090
1427.6788	11	A	7	678.3821
1524.7316	12	P	6	607.3450
1621.7843	13	P	5	510.2922
1708.8164	14	S	4	413.2395
1805.8691	15	P	3	326.2074
1902.9219	16	P	2	229.1547
---	17	L	1	132.1019

These fragment ions are not present in the MS^3 peptide spectrum. This indicates that they are derived from glycopeptide fragmentation.

Fraction 16

872.73+++ → Pep [M+H]+ 1668.77+ [31.3-31.7 min]

CID-MS2

Internal glycopeptide fragmentation

AVP**T**PVVDPDAPPSPPL
HexNac(1)Hex(1)-

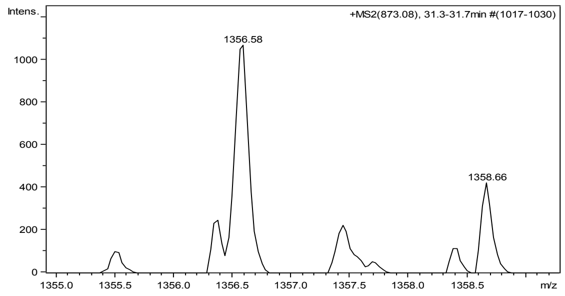

AVP**T**PVVDPD
HexNac(1)Hex(1)-

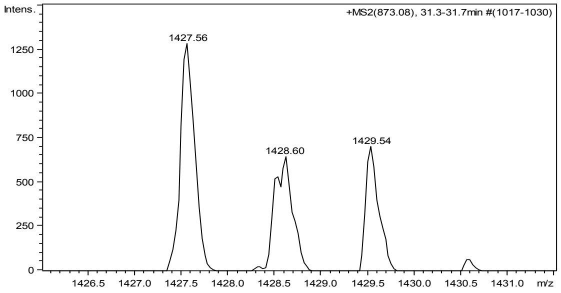

AVP**T**PVVDPDA
HexNac(1)Hex(1)-

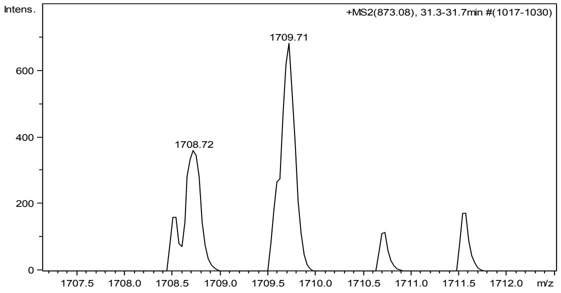

AVP**T**PVVDPDAPPS
HexNac(1)Hex(1)-

These fragment ions are not present in the MS^3 peptide spectrum. This indicates that they are derived from glycopeptide fragmentation.

Fraction 16

872.73+++ → Pep [M+H]+ 1668.77+ [31.3-31.7 min] CID-MS3

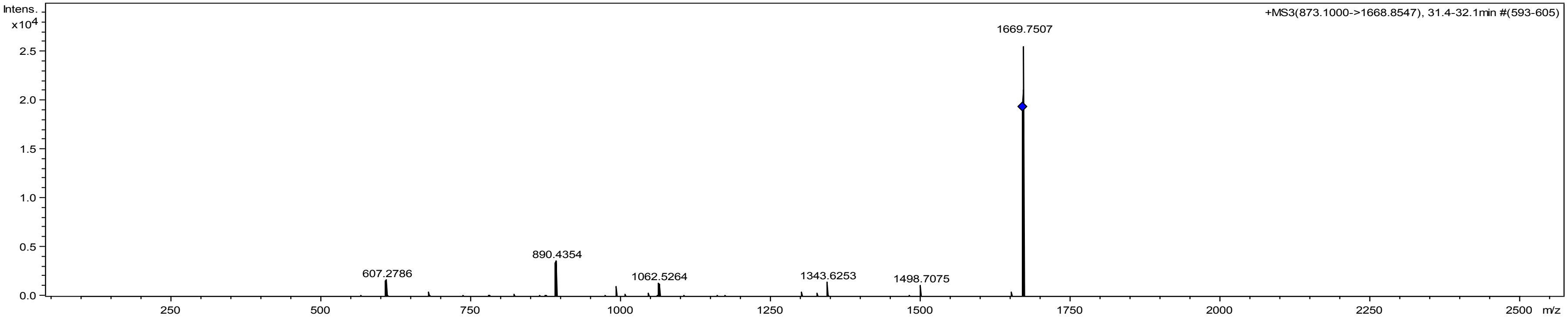

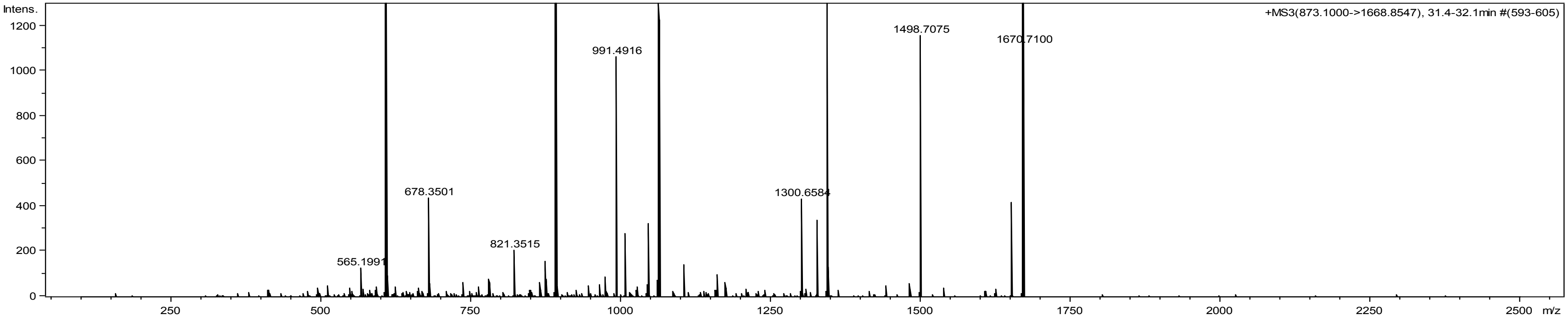

Fraction 16

872.73+++ → Pep [M+H]+ 1668.77+ [31.3-31.7 min] CID-MS3

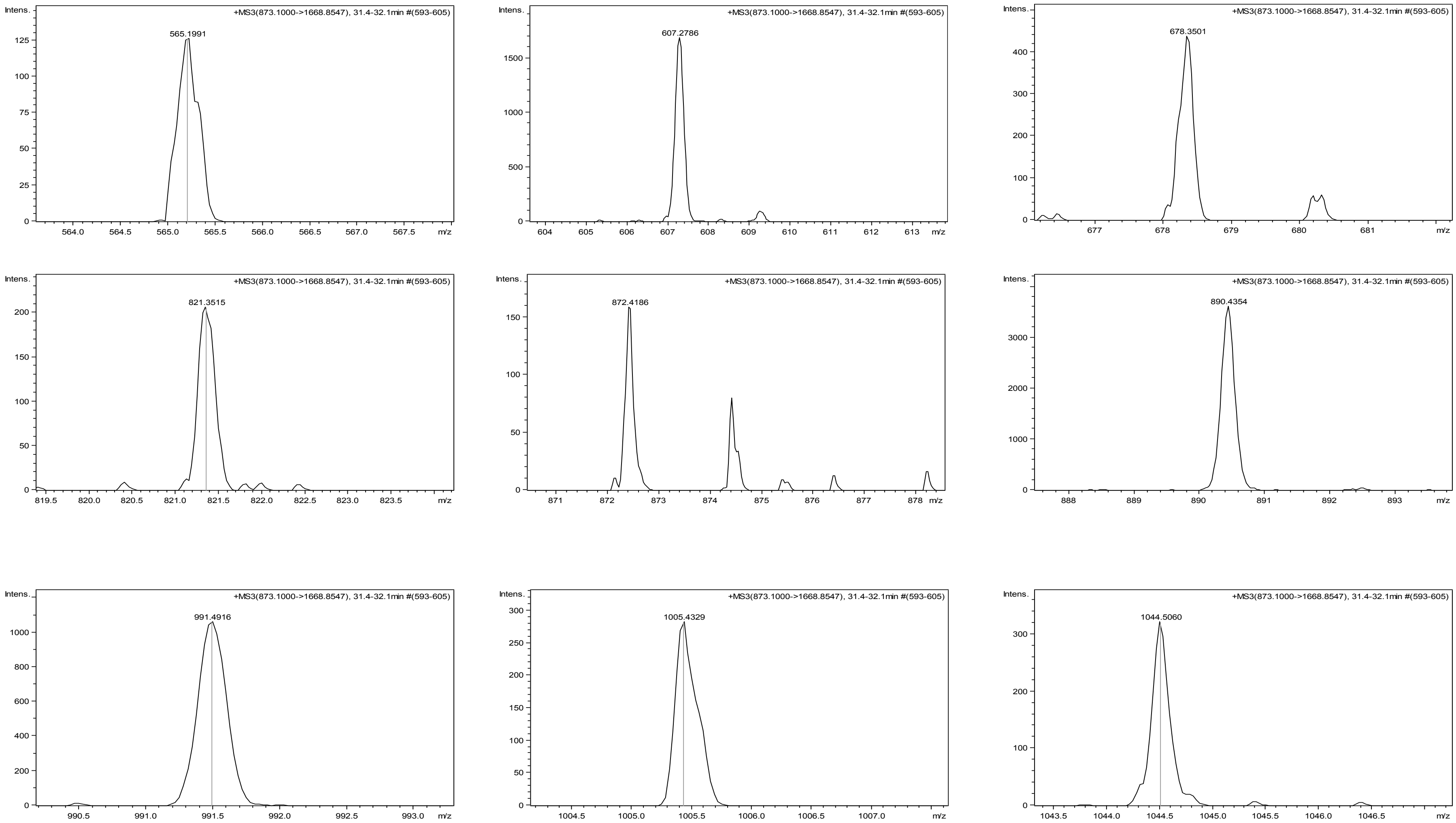

Fraction 16

872.73+++ → Pep [M+H]+ 1668.77+ [31.3-31.7 min] CID-MS3

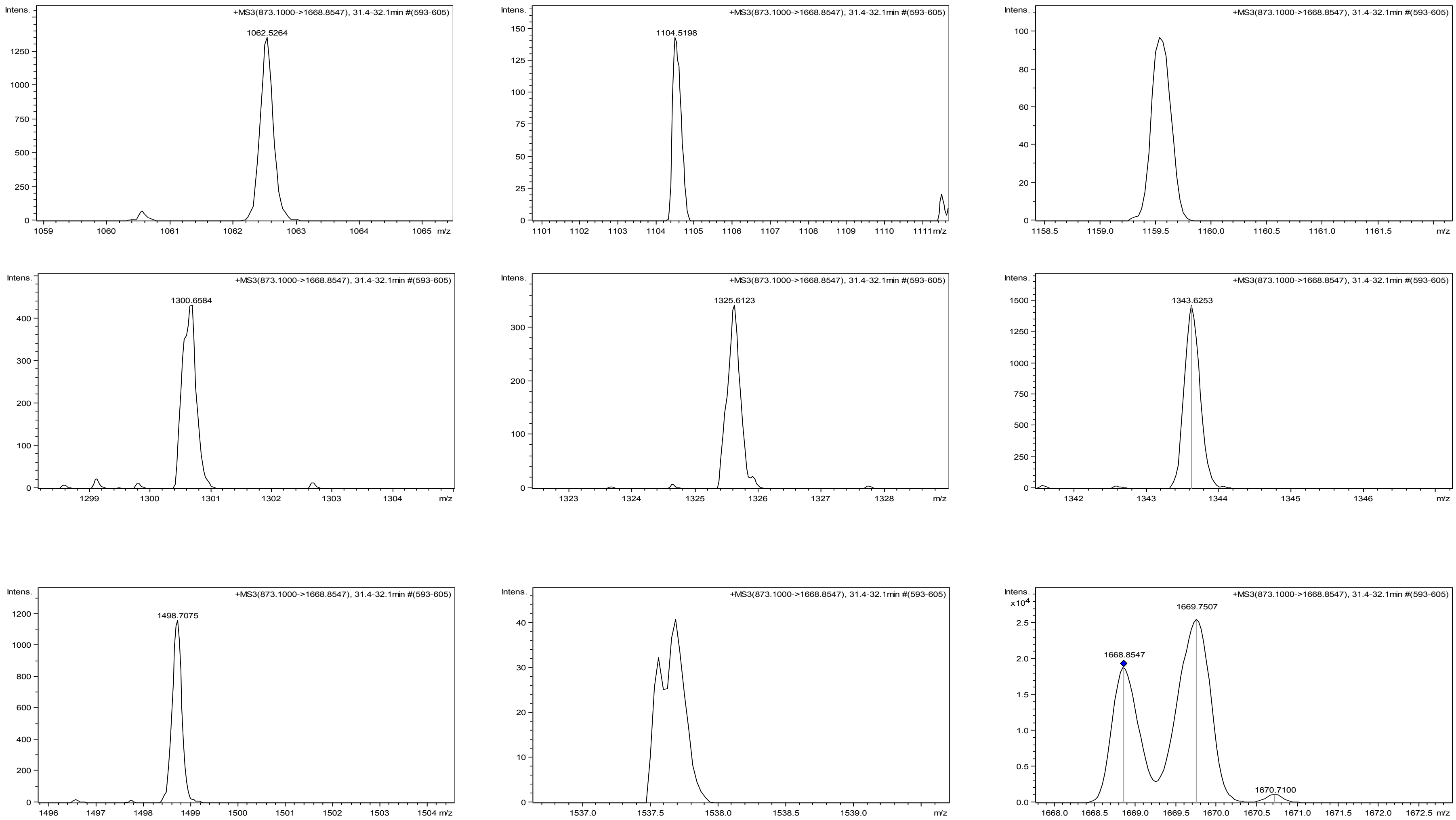

Fraction 16

872.73+++ → Pep [M+H]+ 1668.77+ [31.3-31.7 min]

CID-MS3 MASCOT Search

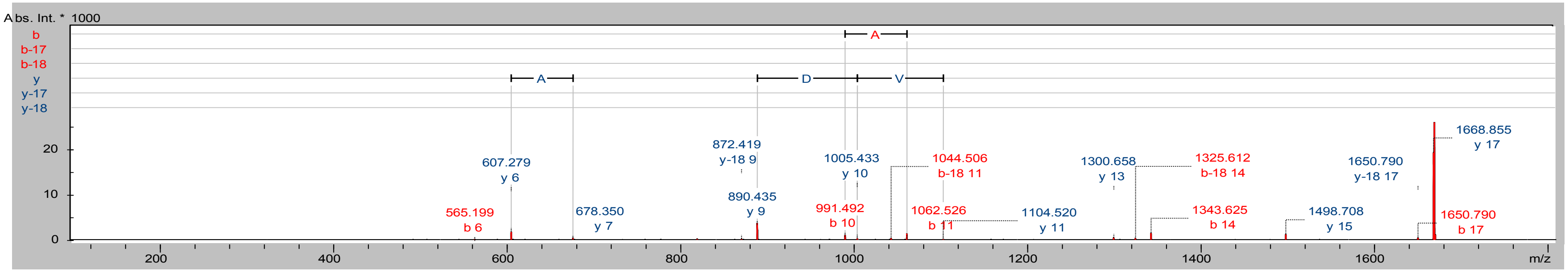

	A	V	P	T	P	V	V	D	P	D	A	P	P	S	P	P	L
Ion	1	2	3	4	5	6	7	8	9	10	11	12	13	14	15	16	17
b	A	V	P	T	P	V	V	D	P	D	A	P	P	S	P	P	L
b-17	A	V	P	T	P	V	V	D	P	D	A	P	P	S	P	P	L
b-18	A	V	P	T	P	V	V	D	P	D	A	P	P	S	P	P	L
y	A	V	P	T	P	V	V	D	P	D	A	P	P	S	P	P	L
y-17	A	V	P	T	P	V	V	D	P	D	A	P	P	S	P	P	L
y-18	A	V	P	T	P	V	V	D	P	D	A	P	P	S	P	P	L
	17	16	15	14	13	12	11	10	9	8	7	6	5	4	3	2	1

	Ala	Val	Pro	Thr	Pro	Val	Val	Asp	Pro	Asp	Ala	Pro	Pro	Ser	Pro	Pro	Leu
Ion	1	2	3	4	5	6	7	8	9	10	11	12	13	14	15	16	17
b	72.044	171.113	268.166	369.213	466.266	565.334	664.403	779.430	876.483	991.509	1062.547	1159.599	1256.652	1343.684	1440.737	1537.790	1650.874
b-17	-	-	-	-	-	-	-	-	-	-	-	-	-	-	-	-	-
b-18	-	-	-	351.203	448.255	547.324	646.392	761.419	858.472	973.499	1044.536	1141.589	1238.642	1325.674	1422.726	1519.779	1632.863
y	132.102	229.155	326.207	413.239	510.292	607.345	678.382	793.409	890.462	1005.489	1104.557	1203.626	1300.678	1401.726	1498.779	1597.847	1668.884
y-17	-	-	-	-	-	-	-	-	-	-	-	-	-	-	-	-	-
y-18	-	-	-	395.229	492.282	589.334	660.372	775.398	872.451	987.478	1086.547	1185.615	1282.668	1383.715	1480.768	1579.837	1650.874
	Leu	Pro	Pro	Ser	Pro	Pro	Ala	Asp	Pro	Asp	Val	Val	Pro	Thr	Pro	Val	Ala

known O-glycosylation site

Alpha-2-HS-glycoprotein

$_{267}$AVP**T**PVVDPDAPPSPPL$_{283}$

Fraction 16

872.73+++ → Pep [M+H]+ 1668.77+ [31.3-31.7 min]

CID-MS3 MASCOT Search

prot_hit_nur	prot_acc	prot_desc	prot_score	prot_mass	prot_matche	pep_query	pep_rank	pep_isbold	pep_exp_mz	pep_exp_mr	pep_exp_z	pep_calc_mr	pep_delta	pep_miss	pep_score	pep_expect	pep_res_bef	pep_seq
1	FETUA_HUM	Alpha-2-HS-g	60	40098	1	1	1	1	1668.7659	1667.7586	1	1667.8771	-0.1184	0	65.13	0.0064	E	AVPTPVVDP
2	MYL4_HUMA	Myosin light	20	21665	1	1	2	0	1668.7659	1667.7586	1	1667.9028	-0.1442	0	23.94	84	G	AELRHVLATL
3	PI3R6_HUMA	Phosphoinos	18	85060	1	1	2	0	1668.7659	1667.7586	1	1667.8777	-0.1191	0	23.94	84	L	HTARVLVLGD
4	YIPF1_HUMA	Protein YIPF	16	34426	1	1	8	0	1668.7659	1667.7586	1	1667.8745	-0.1159	0	20.05	2.10E+02	P	FWICATLVFA
5	LR37A_HUM	Leucine-rich	15	181649	1	1	2	0	1668.7659	1667.7586	1	1667.6468	0.1118	0	23.94	84	M	ENTNMPEGT
6	FA22A_HUM	Protein FAM	13	60343	1	1	9	0	1668.7659	1667.7586	1	1667.7726	-0.014	0	19.81	2.20E+02	S	GEPGHSLGLT
7	AOC2_HUMA	Retina-speci	13	84077	1	1	10	0	1668.7659	1667.7586	1	1667.8043	-0.0456	0	19.74	2.20E+02	S	QYSVQGNLV

Biotools-Score: 15

MASCOT-Score: 65

known O-glycosylation site

Alpha-2-HS-glycoprotein

$_{267}$AVP**T**PVVDPDAPPSPPL$_{283}$

Fraction 16

872.73+++ → Pep [M+H]+ 1668.77+ [31.3-31.7 min] ETD

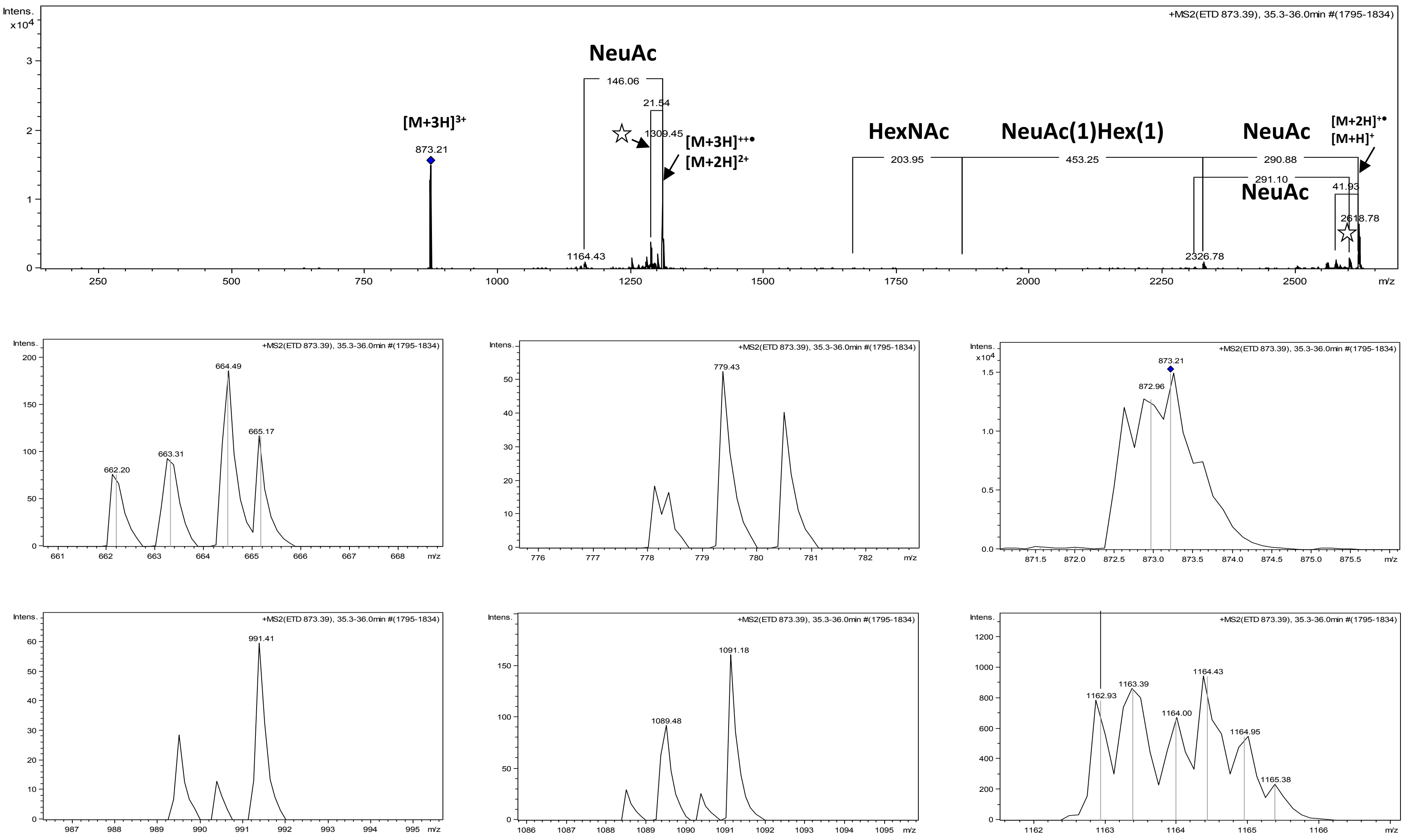

Fraction 16

872.73+++ → Pep [M+H]+ 1668.77+ [31.3-31.7 min]

ETD

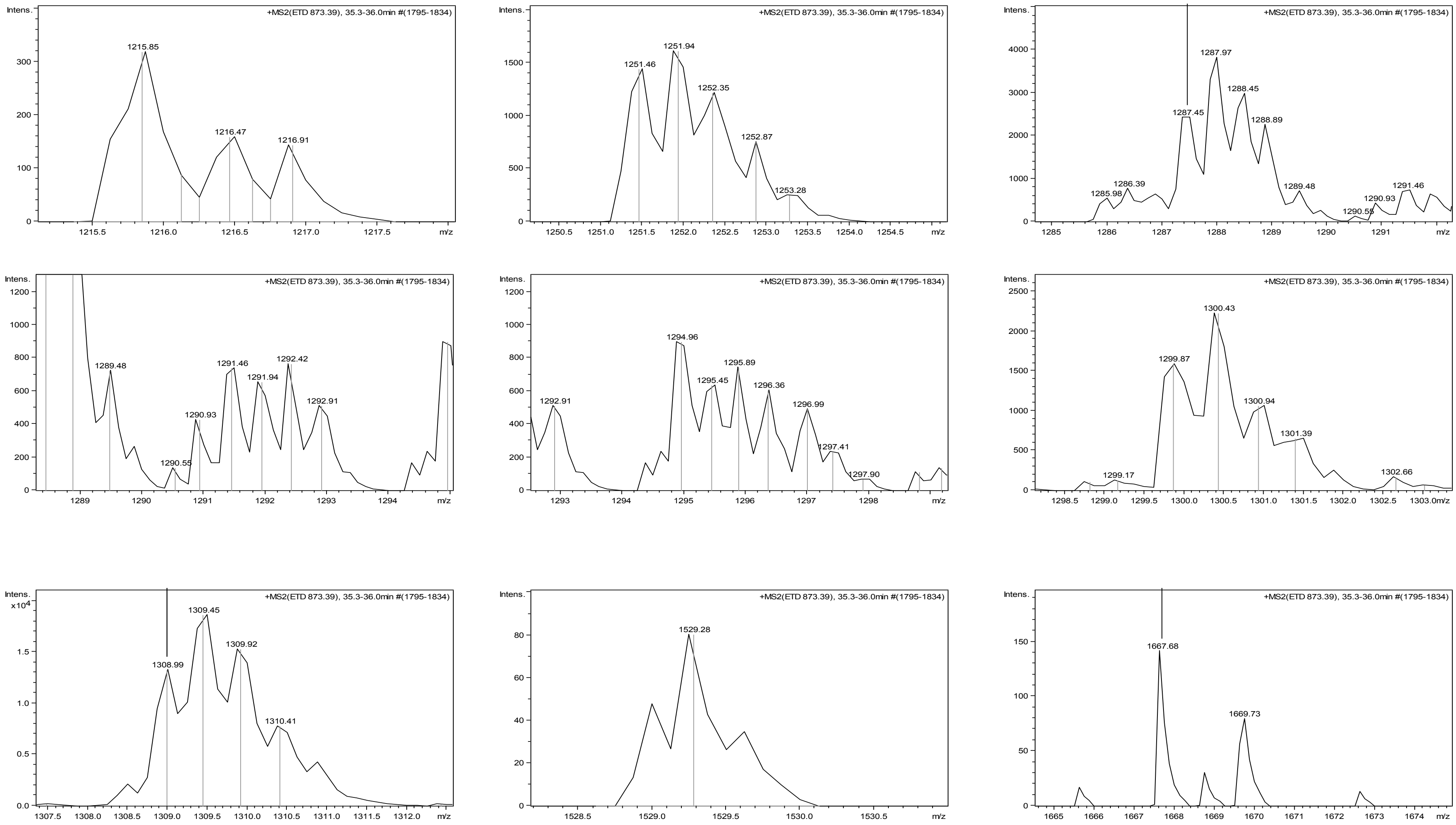

Fraction 16

872.73+++ → Pep [M+H]+ 1668.77+ [31.3-31.7 min]

ETD

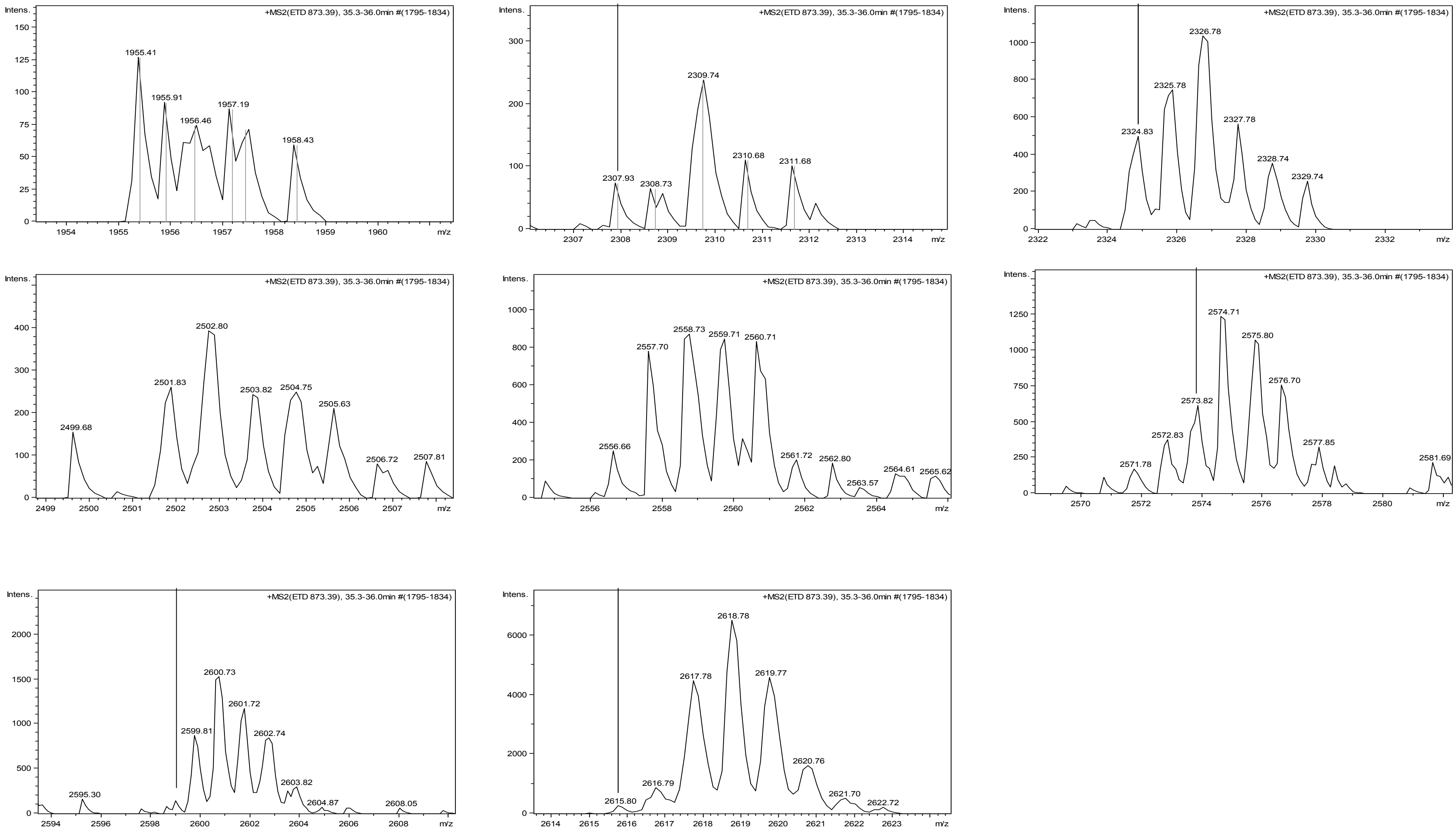

Fraction 16

872.73+++ → Pep [M+H]+ 1668.77+ [31.3-31.7 min] ETD

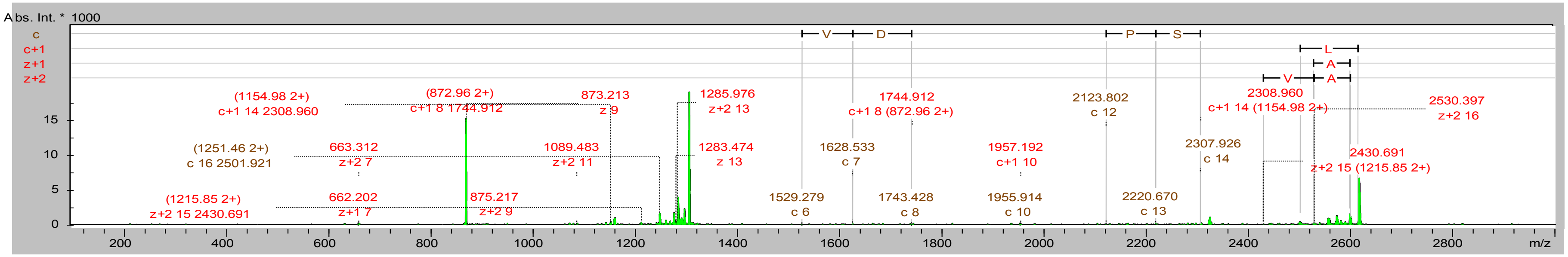

AVP**T**PVVDPDAPPSPPL

	A	V	P	T	P	V	V	D	P	D	A	P	P	S	P	P	L	Ala	Val	Pro	Thr	Pro	Val	Val	Asp	Pro	Asp	Ala	Pro	Pro	Ser	Pro	Pro	Leu
Ion	1	2	3	4	5	6	7	8	9	10	11	12	13	14	15	16	17	1	2	3	4	5	6	7	8	9	10	11	12	13	14	15	16	17
c	A	V	P	T*	P	V	V	D	P	D	A	P	P	S	P	P	L	89.071	188.139	285.192	1333.563	1430.616	1529.684	1628.752	1743.779	1840.832	1955.859	2026.896	2123.949	2221.002	2308.034	2405.086	2502.139	2615.223
c+1	A	V	P	T*	P	V	V	D	P	D	A	P	P	S	P	P	L	90.079	189.147	286.200	1334.571	1431.623	1530.692	1629.760	1744.787	1841.840	1956.867	2027.904	2124.957	2222.010	2309.042	2406.094	2503.147	2616.231
z	A	V	P	T*	P	V	V	D	P	D	A	P	P	S	P	P	L	115.075	212.128	309.181	396.213	493.266	590.318	661.356	776.382	873.435	988.462	1087.531	1186.599	1283.652	2332.023	2429.075	2528.144	2599.181
z+1	A	V	P	T*	P	V	V	D	P	D	A	P	P	S	P	P	L	116.083	213.136	310.189	397.221	494.273	591.326	662.363	777.390	874.443	989.470	1088.538	1187.607	1284.660	2333.030	2430.083	2529.152	2600.189
z+2	A	V	P	T*	P	V	V	D	P	D	A	P	P	S	P	P	L	117.091	214.144	311.197	398.229	495.281	592.334	663.371	778.398	875.451	990.478	1089.546	1188.615	1285.667	2334.038	2431.091	2530.159	2601.196
	17	16	15	14	13	12	11	10	9	8	7	6	5	4	3	2	1	Leu	Pro	Pro	Ser	Pro	Pro	Ala	Asp	Pro	Asp	Val	Val	Pro	Thr	Pro	Val	Ala

Biotools-Score: 39

known O-glycosylation site

Alpha-2-HS-glycoprotein $_{267}$AVP**T**PVVDPDAPPSPPL$_{283}$

Fraction 16

872.73+++ → Pep [M+H]+ 1668.77+ [31.3-31.7 min] ETD

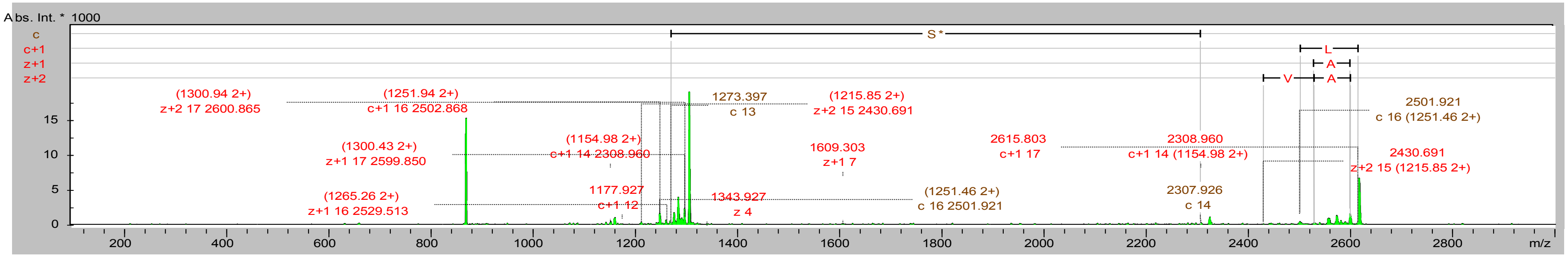

AVPTPVVDPDAPP**S**PPL

	A	V	P	T	P	V	V	D	P	D	A	P	P	S	P	P	L
Ion	1	2	3	4	5	6	7	8	9	10	11	12	13	14	15	16	17
c	A	V	P	T	P	V	V	D	P	D	A	P	P	S*	P	P	L
c+1	A	V	P	T	P	V	V	D	P	D	A	P	P	S*	P	P	L
z	A	V	P	T	P	V	V	D	P	D	A	P	P	S*	P	P	L
z+1	A	V	P	T	P	V	V	D	P	D	A	P	P	S*	P	P	L
z+2	A	V	P	T	P	V	V	D	P	D	A	P	P	S*	P	P	L
	17	16	15	14	13	12	11	10	9	8	7	6	5	4	3	2	1

	Ala	Val	Pro	Thr	Pro	Val	Val	Asp	Pro	Asp	Ala	Pro	Pro	Ser	Pro	Pro	Leu
Ion	1	2	3	4	5	6	7	8	9	10	11	12	13	14	15	16	17
c	89.071	188.139	285.192	386.240	483.293	582.361	681.429	796.456	893.509	1008.536	1079.573	1176.626	1273.679	2308.034	2405.086	2502.139	2615.223
c+1	90.079	189.147	286.200	387.248	484.300	583.369	682.437	797.464	894.517	1009.544	1080.581	1177.634	1274.687	2309.042	2406.094	2503.147	2616.231
z	115.075	212.128	309.181	1343.536	1440.589	1537.641	1608.679	1723.706	1820.758	1935.785	2034.854	2133.922	2230.975	2332.023	2429.075	2528.144	2599.181
z+1	116.083	213.136	310.189	1344.544	1441.597	1538.649	1609.686	1724.713	1821.766	1936.793	2035.861	2134.930	2231.983	2333.030	2430.083	2529.152	2600.189
z+2	117.091	214.144	311.197	1345.552	1442.604	1539.657	1610.694	1725.721	1822.774	1937.801	2036.869	2135.938	2232.990	2334.038	2431.091	2530.159	2601.196
	Leu	Pro	Pro	Ser	Pro	Pro	Ala	Asp	Pro	Asp	Val	Val	Pro	Thr	Pro	Val	Ala

Biotools-Score: 21

known O-glycosylation site

Alpha-2-HS-glycoprotein $_{267}$AVP**T**PVVDPDAPPSPPL$_{283}$

Fraction 16

818.68+++ → Pep [M+H]+ 1797.75+ [31.5-31.7 min]

CID-MS Precursor

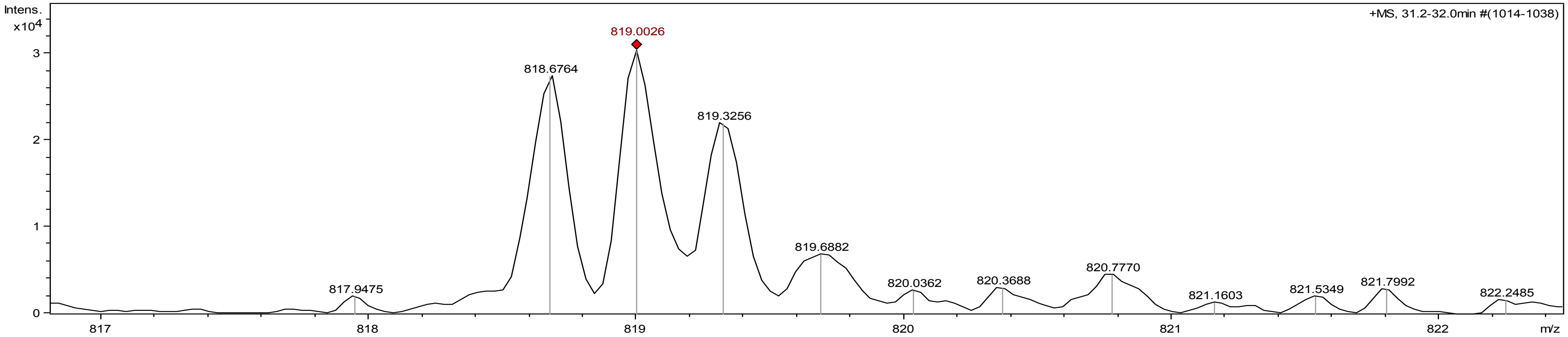

MASCOT Search only on CID-MS² spectrum possible,
For MASCOT search m/z of the unmodified peptide [M+H]⁺ has to be given

Many b- and y-ions in CID MS² spectrum

ETD Spectrum of poor quality

Fraction 16

818.68+++ → Pep [M+H]+ 1797.75+ [31.5-31.7 min] CID-MS2

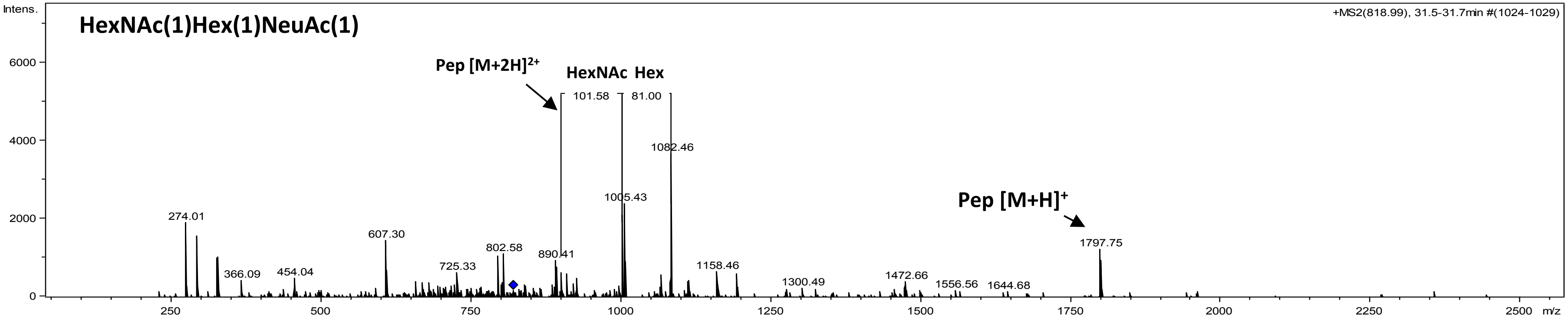

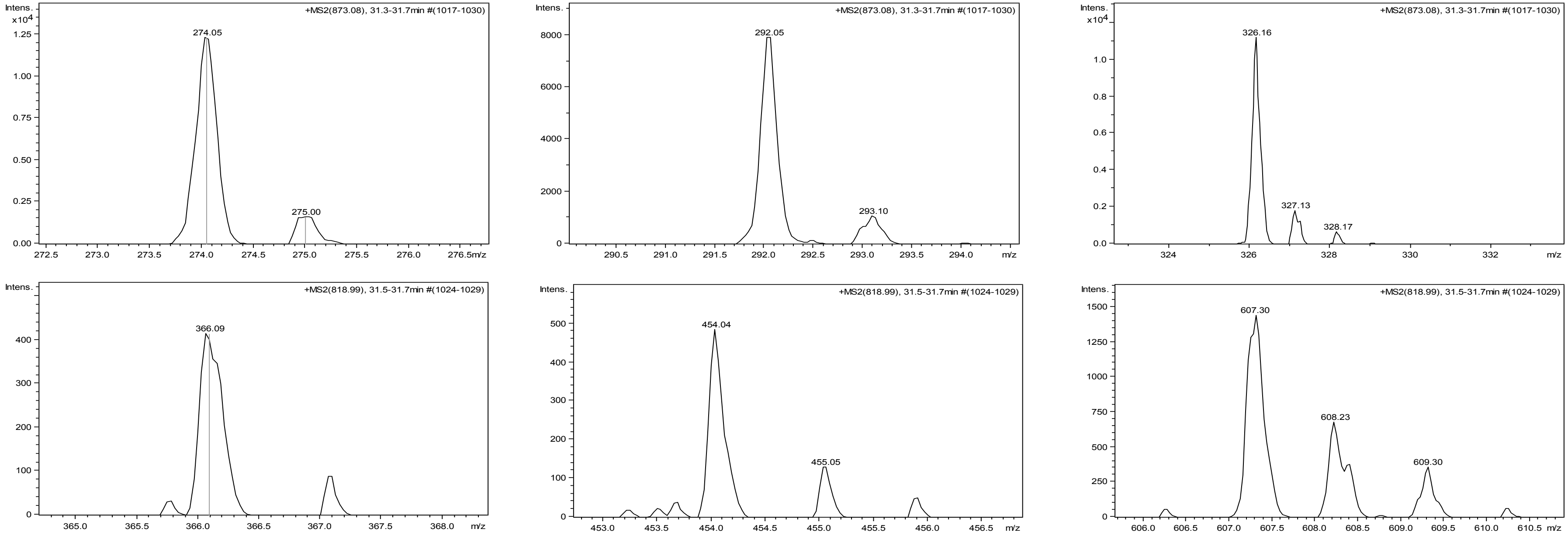

Fraction 16

818.68+++ → Pep [M+H]+ 1797.75+ [31.5-31.7 min] CID-MS2

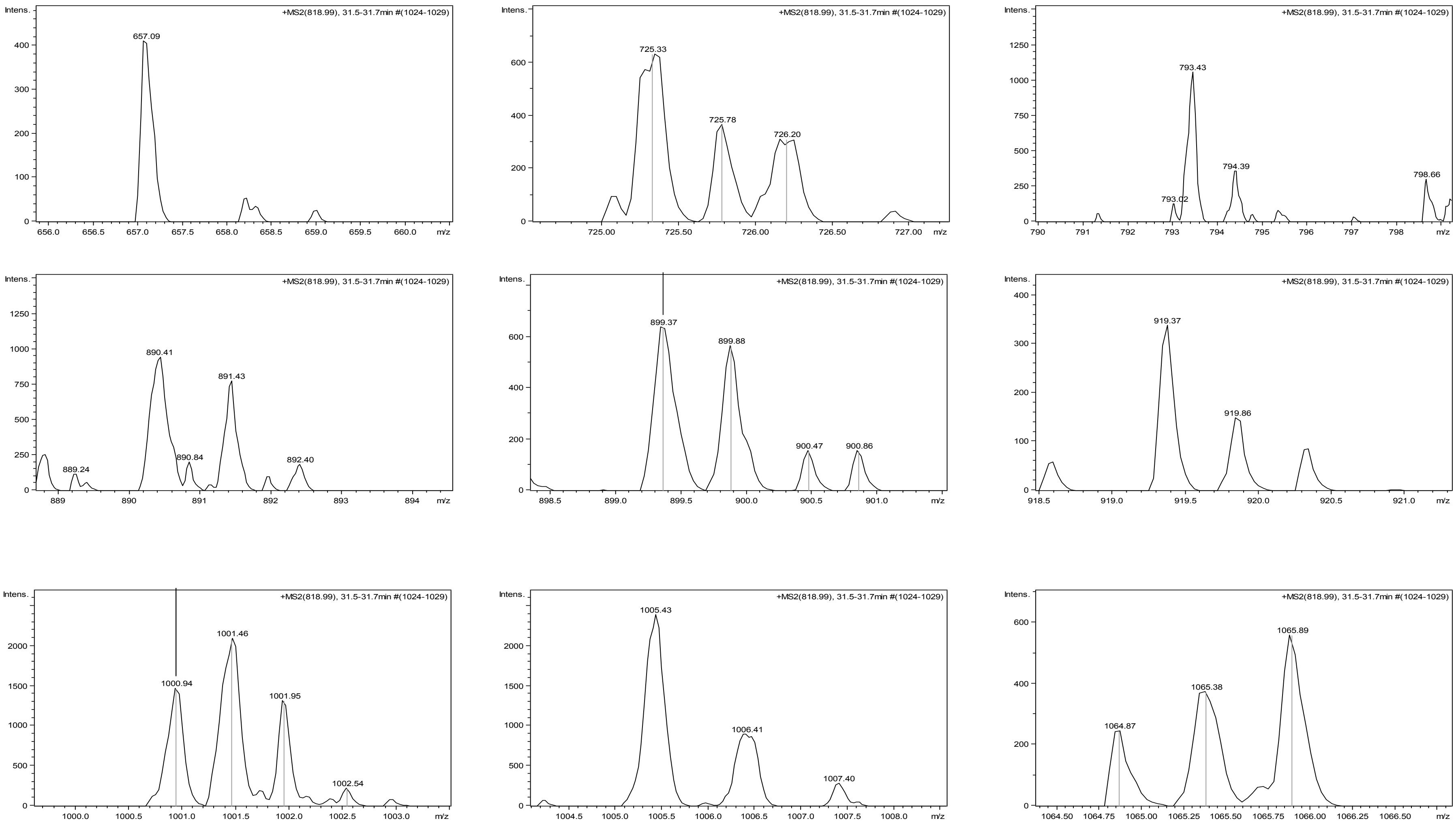

Fraction 16

818.68+++ → Pep [M+H]+ 1797.75+ [31.5-31.7 min]

CID-MS2

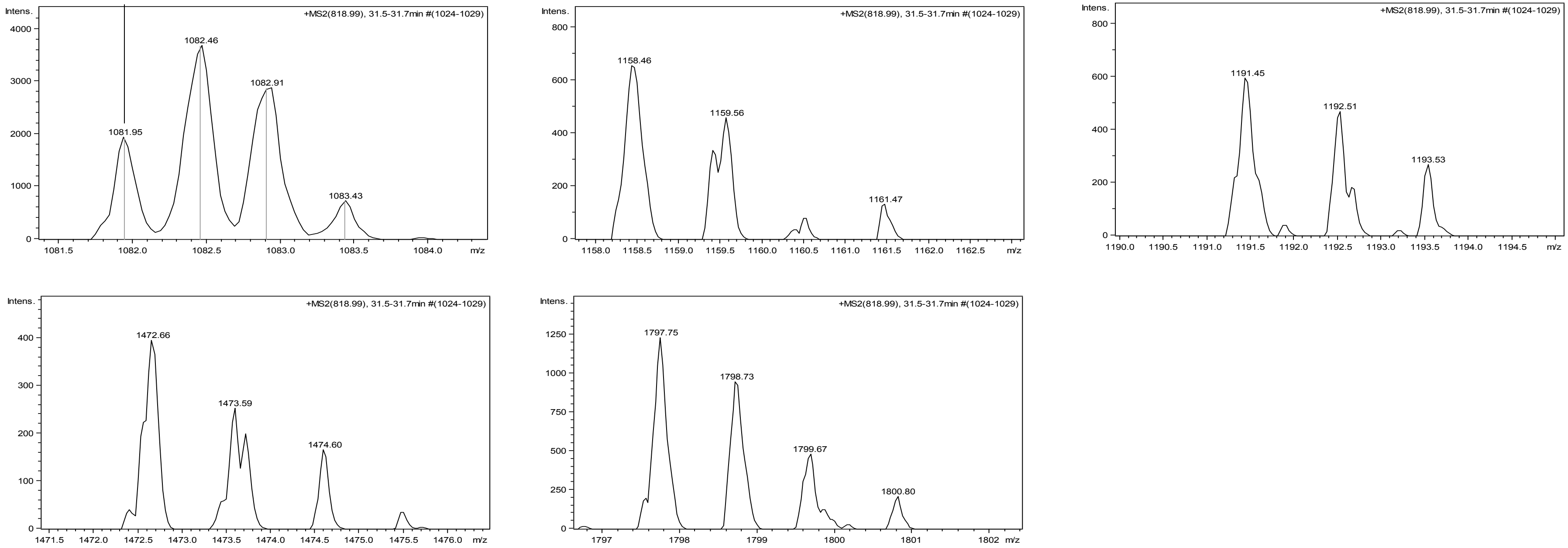

Fraction 16

818.68+++ → Pep [M+H]+ 1797.75+ [31.5-31.7 min]

CID-MS2 MASCOT Search

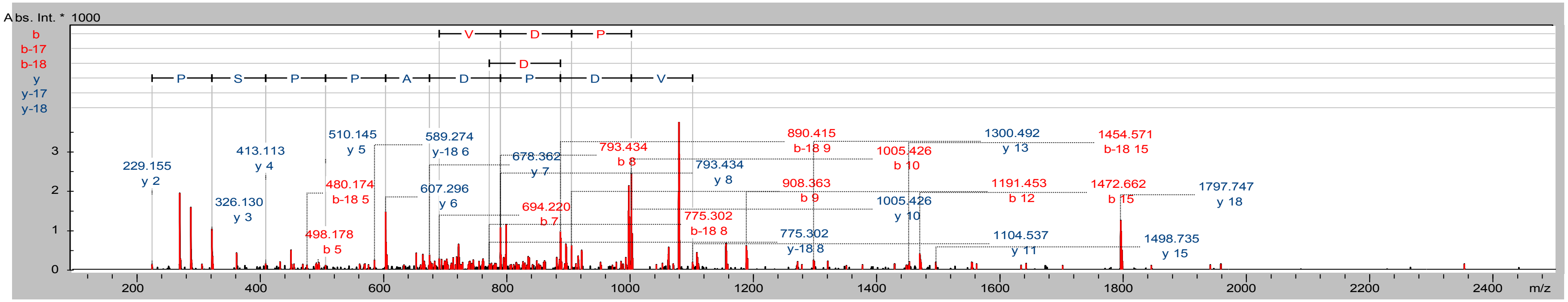

	E	A	V	P	T	P	V	V	D	P	D	A	P	P	S	P	P	L	Glu	Ala	Val	Pro	Thr	Pro	Val	Val	Asp	Pro	Asp	Ala	Pro	Pro	Ser	Pro	Pro	Leu
Ion	1	2	3	4	5	6	7	8	9	10	11	12	13	14	15	16	17	18	1	2	3	4	5	6	7	8	9	10	11	12	13	14	15	16	17	18
b	E	A	V	P	T	P	V	V	D	P	D	A	P	P	S	P	P	L	130.050	201.087	300.155	397.208	498.256	595.309	694.377	793.445	908.472	1005.525	1120.552	1191.589	1288.642	1385.695	1472.727	1569.780	1666.832	1779.916
b-17	E	A	V	P	T	P	V	V	D	P	D	A	P	P	S	P	P	L	-	-	-	-	-	-	-	-	-	-	-	-	-	-	-	-	-	-
b-18	E	A	V	P	T	P	V	V	D	P	D	A	P	P	S	P	P	L	112.039	183.076	282.145	379.198	480.245	577.298	676.366	775.435	890.462	987.515	1102.542	1173.579	1270.631	1367.684	1454.716	1551.769	1648.822	1761.906
y	E	A	V	P	T	P	V	V	D	P	D	A	P	P	S	P	P	L	132.102	229.155	326.207	413.239	510.292	607.345	678.382	793.409	890.462	1005.489	1104.557	1203.626	1300.678	1401.726	1498.779	1597.847	1668.884	1797.927
y-17	E	A	V	P	T	P	V	V	D	P	D	A	P	P	S	P	P	L	-	-	-	-	-	-	-	-	-	-	-	-	-	-	-	-	-	-
y-18	E	A	V	P	T	P	V	V	D	P	D	A	P	P	S	P	P	L	-	-	-	395.229	492.282	589.334	660.372	775.398	872.451	987.478	1086.547	1185.615	1282.668	1383.715	1480.768	1579.837	1650.874	1779.916
	18	17	16	15	14	13	12	11	10	9	8	7	6	5	4	3	2	1	Leu	Pro	Pro	Ser	Pro	Pro	Ala	Asp	Pro	Asp	Val	Val	Pro	Thr	Pro	Val	Ala	Glu

For MASCOT search m/z of the unmodified peptide [M+H]$^{+}$ has to be given

known O-glycosylation site

Alpha-2-HS-glycoprotein

$_{266}$EAVP**T**PVVDPDAPPSPPL$_{283}$

Fraction 16

818.68+++ → Pep [M+H]+ 1797.75+ [31.5-31.7 min]

CID-MS2 MASCOT Search

prot_hit_nur	prot_acc	prot_desc	prot_score	prot_mass	pro	pro	pro	pro	pep	pep	pep	pep	pep_exp_mz	pep_exp_mr	pep_e	pep_calc_mr	pep_delta	pep_n	pep_score	pep_expect	pep_re	pep_seq
1	FETUA_HUM	Alpha-2-HS-g	6	40098	1	0	1	0	1	1	1	1	1797.75	1796.7427	1	1796.9196	-0.1769	0	10.71	2.40E+03	N	EAVPTPVVDI
2	VOPP1_HUMAN		1	19839	1	0	1	0	1	4	0	1	1797.75	1796.7427	1	1795.7215	1.0212	0	6.67	6.00E+03	D	PGGPGMNPV
3	CO6A1_HUMAN		1	109602	1	0	1	0	1	3	0	1	1797.75	1796.7427	1	1795.7802	0.9625	0	7.16	5.40E+03	G	NGTEGFPGFF
4	CO8A1_HUMAN		0	73431	1	0	1	0	1	2	0	1	1797.75	1796.7427	1	1796.8992	-0.1565	0	7.29	5.20E+03	L	PGIGKPGFPG
5	MGLL_HUMAN		0	33468	1	0	1	0	1	8	0	1	1797.75	1796.7427	1	1796.9632	-0.2205	0	1.83	1.80E+04	L	PNLSLGPIDSS
6	DDB1_HUMAN		0	128142	1	0	1	0	1	10	0	1	1797.75	1796.7427	1	1796.8866	-0.1439	0	1.6	1.90E+04	T	QGSVLFGTVN
7	JAM1_HUMAN		0	32962	1	0	1	0	1	9	0	1	1797.75	1796.7427	1	1796.9309	-0.1881	0	1.65	1.90E+04	N	KITASYEDRV
8	NCKP1_HUMAN		0	130018	1	0	1	0	1	5	0	1	1797.75	1796.7427	1	1795.9403	0.8024	0	5.9	7.20E+03	I	IWLLRHADNI
9	TRIM2_HUMAN		0	82506	1	0	1	0	1	6	0	1	1797.75	1796.7427	1	1796.7853	-0.0426	0	3.82	1.20E+04	M	LKFGSNGEGN

Biotools-Score: 12

MASCOT-Score: 11

$_{266}$EAVP**T**PVVDPDAPPSPPL$_{283}$

Fraction 16

818.68+++ → Pep [M+H]+ 1797.75+ [31.5-31.7 min]

CID-MS2

Internal glycopeptide fragmentation

EAVP**T**PVVDPDAPPSPPL
HexNac-

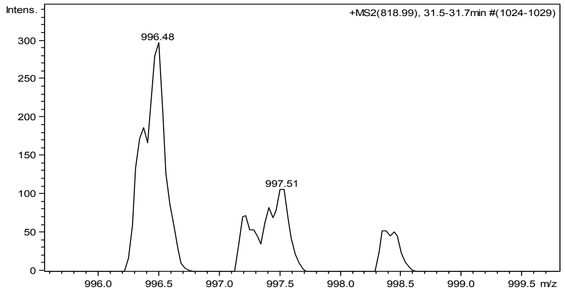

EAVP**T**PVV
HexNac-

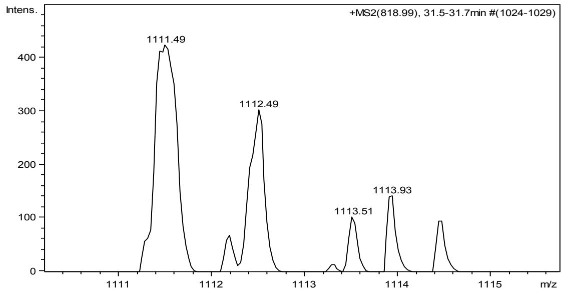

EAVP**T**PVVD
HexNac-

b				y
---	1	E	18	---
201.0870	2	A	17	1871.9637
300.1554	3	V	16	1800.9266
397.2082	4	P	15	1701.8582
701.3352	5	T(HexNAc)	14	1604.8054
798.3880	6	P	13	1300.6783
897.4564	7	V	12	1203.6256
996.5248	8	V	11	1104.5572
1111.5517	9	D	10	1005.4888
1208.6045	10	P	9	890.4618
1323.6315	11	D	8	793.4090
1394.6686	12	A	7	678.3821
1491.7213	13	P	6	607.3450
1588.7741	14	P	5	510.2922
1675.8061	15	S	4	413.2395
1772.8589	16	P	3	326.2074
1869.9117	17	P	2	229.1547
---	18	L	1	132.1019

Fraction 16

818.68+++ → Pep [M+H]+ 1797.75+ [31.5-31.7 min]

CID-MS2

Internal glycopeptide fragmentation

EAVP**T**PVVDPDAPPSPPL

HexNac-

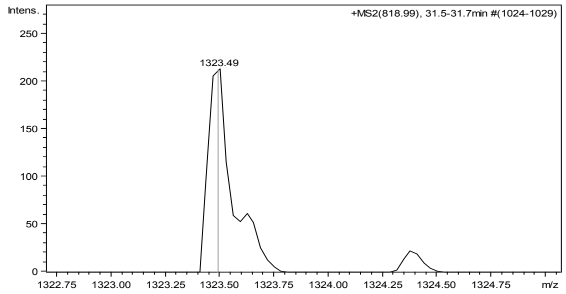

EAVP**T**PVVDPD

HexNac-

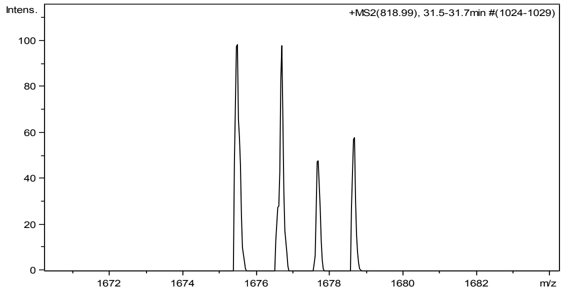

EAVP**T**PVVDPDAPPS

HexNac-

Fraction 16

818.68+++ → Pep [M+H]+ 1797.75+ [31.5-31.7 min]

CID-MS2

Internal glycopeptide fragmentation

EAVPTPVVDPDAPPSPPL

HexNac(1)Hex(1)-

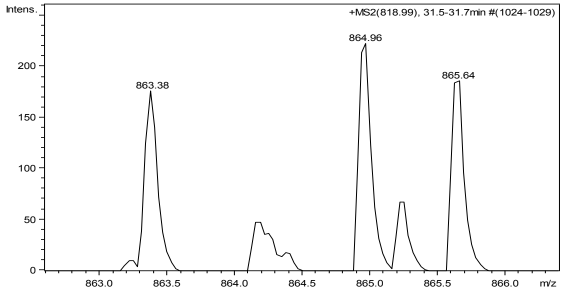

EAVPT

HexNac(1)Hex(1)-

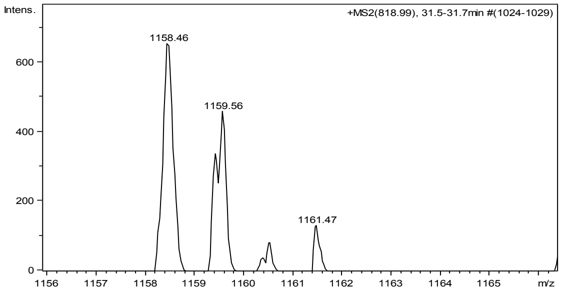

EAVPTPVV

HexNac(1)Hex(1)-

b				y
---	1	E	18	---
201.0870	2	A	17	2034.0165
300.1554	3	V	16	1962.9794
397.2082	4	P	15	1863.9110
863.3880	5	T(HexNAc(1)Hex(1))	14	1766.8582
960.4408	6	P	13	1300.6783
1059.5092	7	V	12	1203.6256
1158.5776	8	V	11	1104.5572
1273.6046	9	D	10	1005.4888
1370.6573	10	P	9	890.4618
1485.6843	11	D	8	793.4090
1556.7214	12	A	7	678.3821
1653.7742	13	P	6	607.3450
1750.8269	14	P	5	510.2922
1837.8589	15	S	4	413.2395
1934.9117	16	P	3	326.2074
2031.9645	17	P	2	229.1547
---	18	L	1	132.1019

Fraction 16

818.68+++ → Pep [M+H]+ 1797.75+ [31.5-31.7 min]

CID-MS2

Internal glycopeptide fragmentation

EAVP**T**PVVDPDAPPSPPL

HexNac(1)Hex(1)-

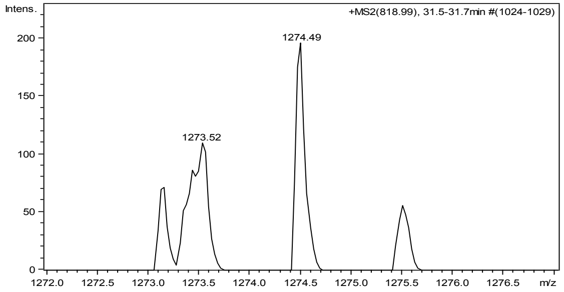

EAVP**T**PVVD

HexNac(1)Hex(1)-

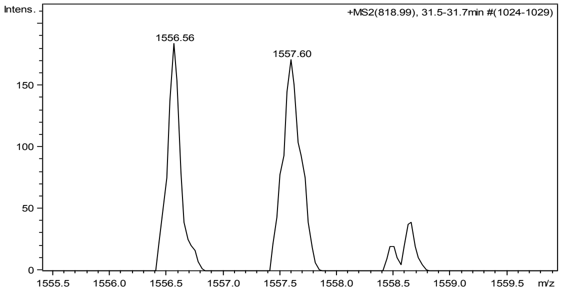

EAVP**T**PVVDPDA

HexNac(1)Hex(1)-

Fraction 16

818.68+++ → Pep [M+H]+ 1797.75+ [31.5-31.7 min]

CID-MS3

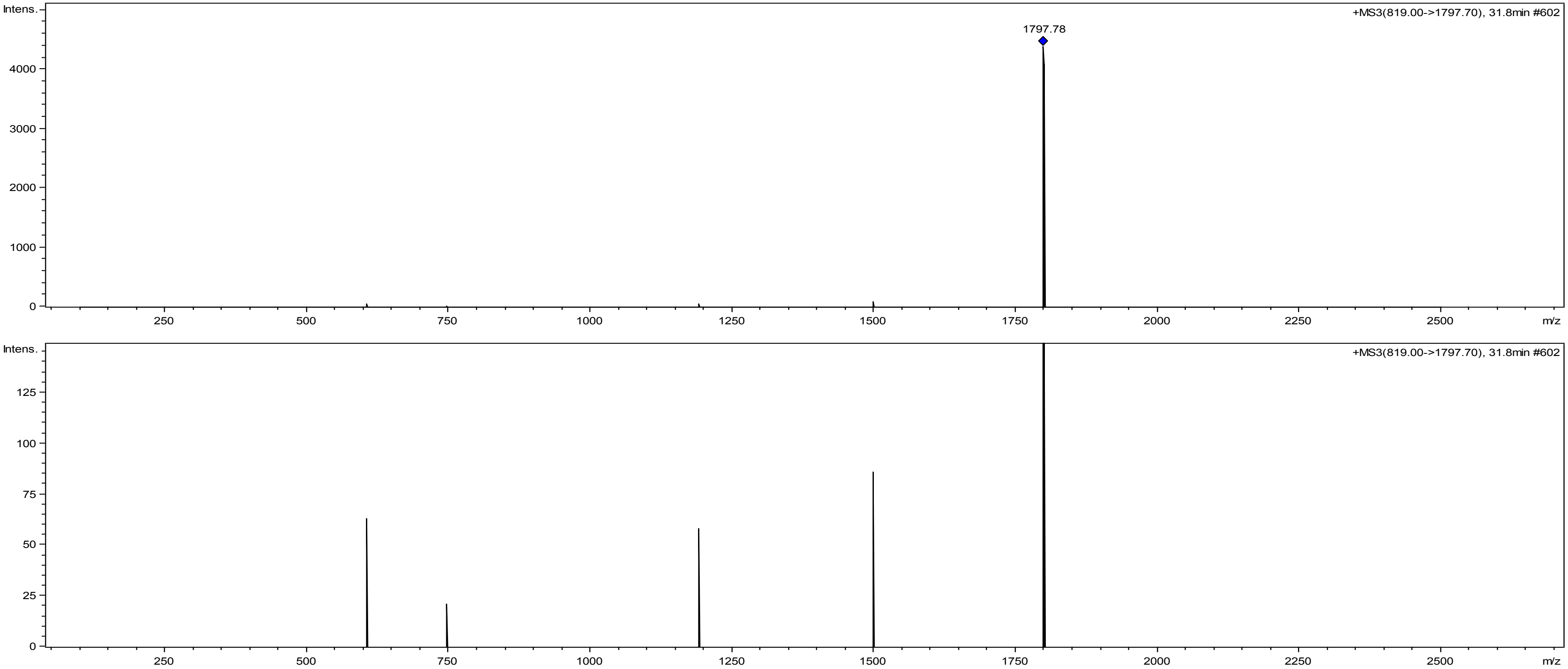

Poor spectrum quality

Fraction 16

734.26+++ → Pep [M+H]+ 1253.49+ [31.8-32.2] CID-MS Precursor

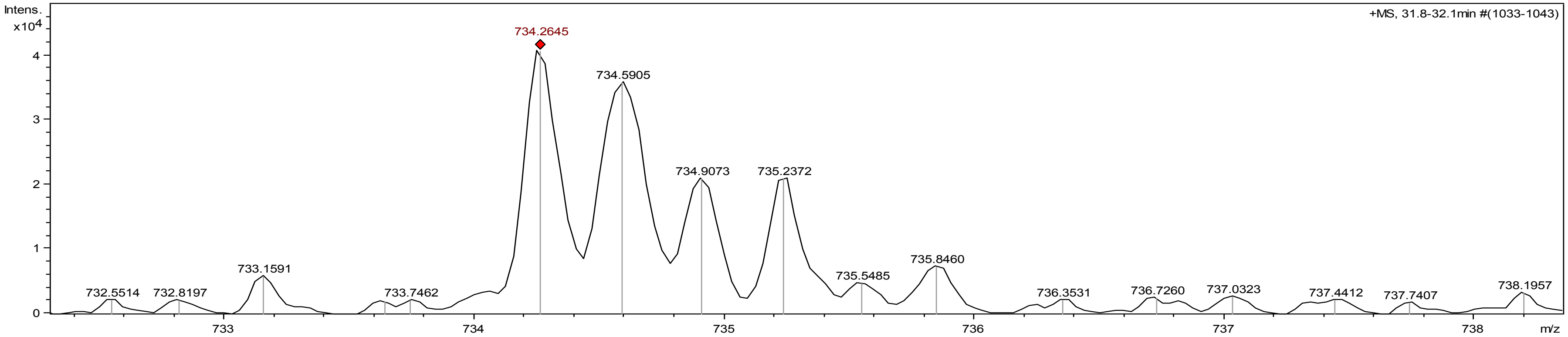

Fraction 16

734.26+++ → Pep [M+H]+ 1253.49+ [31.8-32.2] CID-MS2

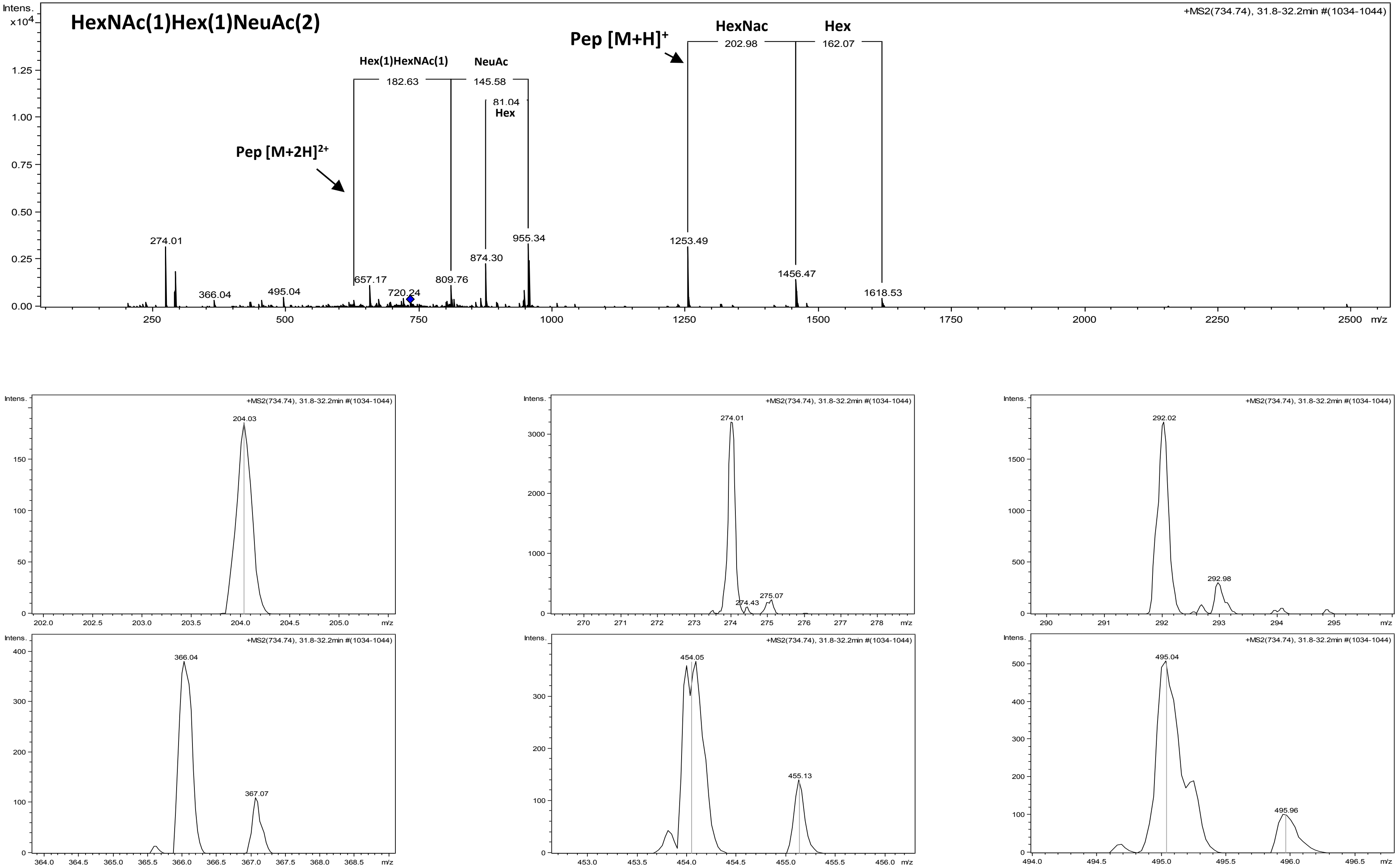

Fraction 16

734.26+++ → Pep [M+H]+ 1253.49+ [31.8-32.2] CID-MS2

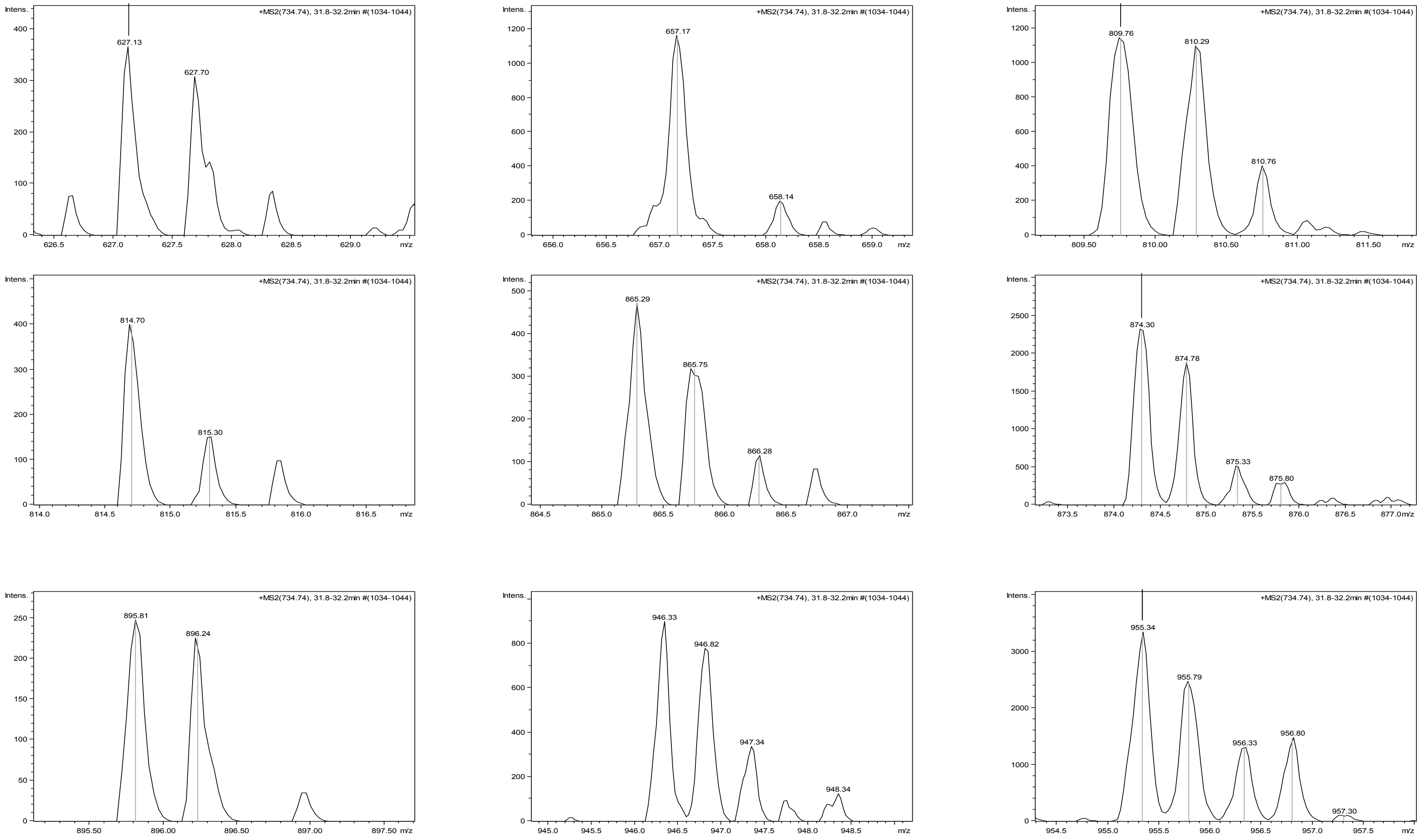

Fraction 16

734.26+++ → Pep [M+H]+ 1253.49+ [31.8-32.2]

CID-MS2

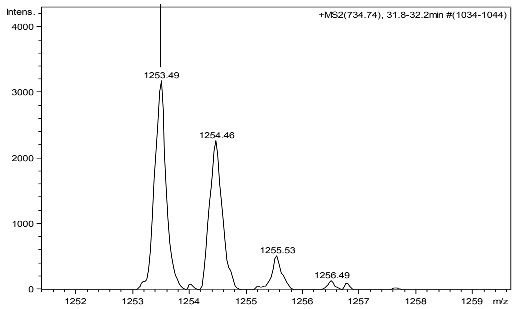

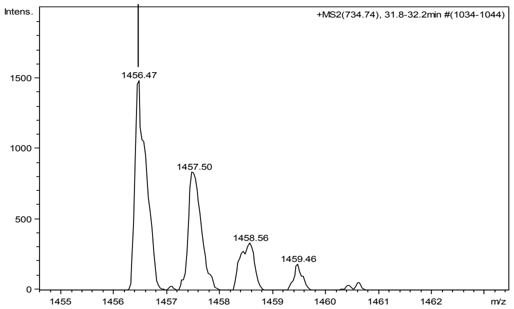

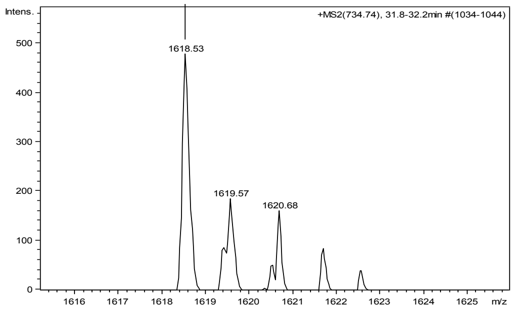

Fraction 16

734.26+++ → Pep [M+H]+ 1253.49+ [31.8-32.2] CID-MS3

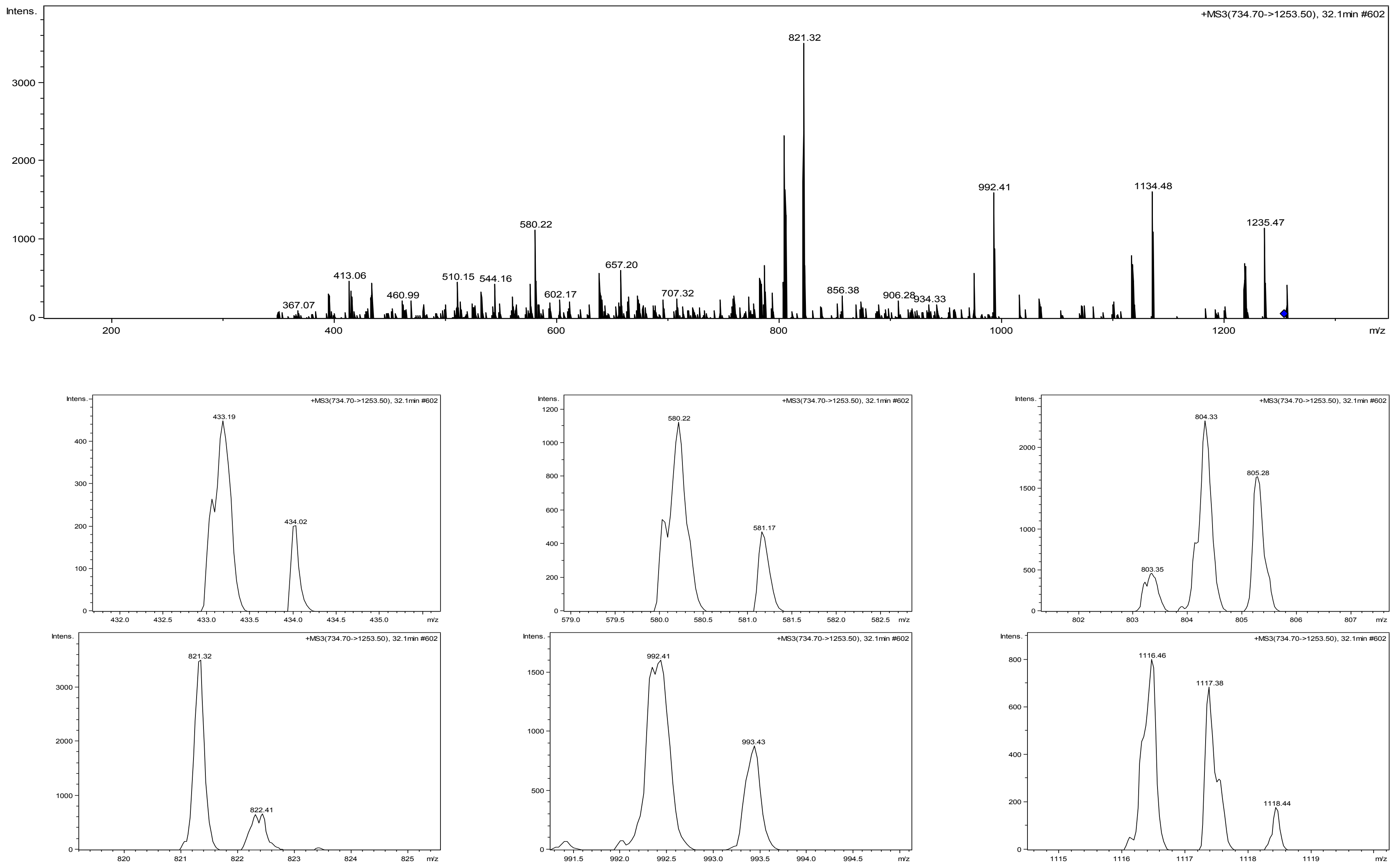

Fraction 16

734.26+++ → Pep [M+H]+ 1253.49+ [31.8-32.2] CID-MS3

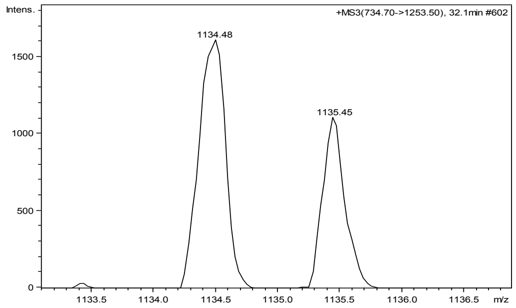

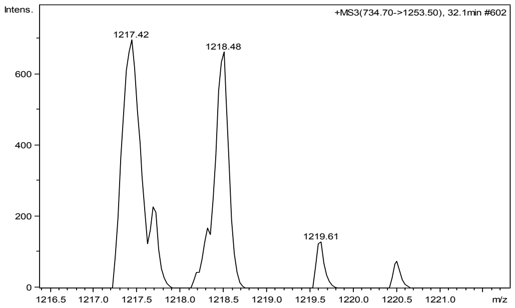

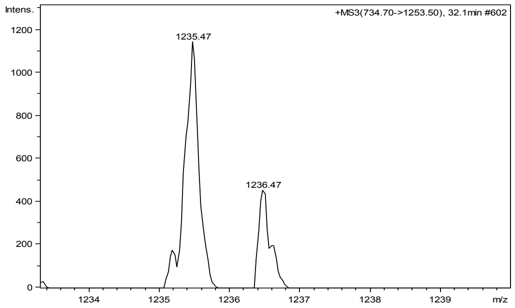

Fraction 16

734.26+++ → Pep [M+H]+ 1253.49+ [31.8-32.2] CID-MS3 MASCOT Search

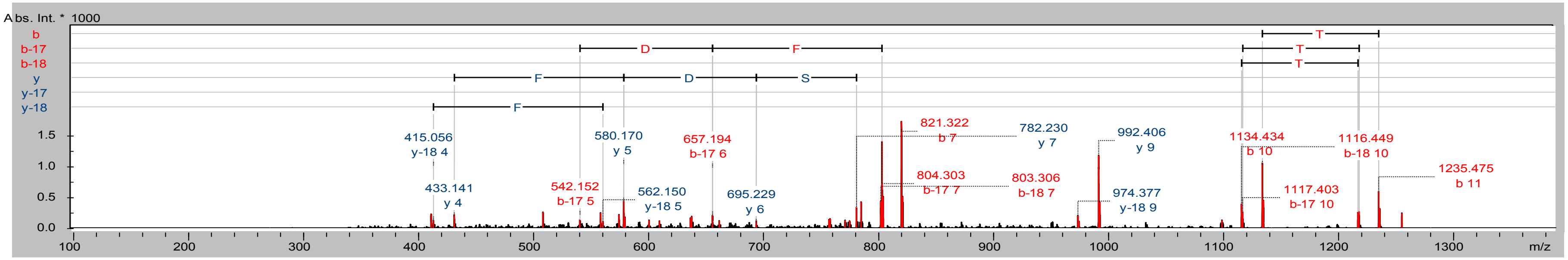

	F	N	P	I	S	D	F	P	D	T	T	Phe	Asn	Pro	Ile	Ser	Asp	Phe	Pro	Asp	Thr	Thr
Ion	1	2	3	4	5	6	7	8	9	10	11	1	2	3	4	5	6	7	8	9	10	11
b	F	N	P	I	S	D	F	P	D	T	T	148.076	262.119	359.171	472.255	559.287	674.314	821.383	918.436	1033.463	1134.510	1235.558
b-17	F	N	P	I	S	D	F	P	D	T	T	-	245.092	342.145	455.229	542.261	657.288	804.356	901.409	1016.436	1117.484	1218.531
b-18	F	N	P	I	S	D	F	P	D	T	T	-	-	-	-	541.277	656.304	803.372	900.425	1015.452	1116.500	1217.547
y	F	N	P	I	S	D	F	P	D	T	T	120.066	221.113	336.140	433.193	580.261	695.288	782.320	895.404	992.457	1106.500	1253.568
y-17	F	N	P	I	S	D	F	P	D	T	T	-	-	-	-	-	-	-	-	-	1089.473	1236.542
y-18	F	N	P	I	S	D	F	P	D	T	T	102.055	203.103	318.130	415.182	562.251	677.278	764.310	877.394	974.447	1088.489	1235.558
	11	10	9	8	7	6	5	4	3	2	1	Thr	Thr	Asp	Pro	Phe	Asp	Ser	Ile	Pro	Asn	Phe

unknown O-glycosylation site

Kininogen-1 precursor

$_{600}$FNPI**S**DFPD**TT**$_{610}$

Fraction 16

734.26+++ → Pep [M+H]+ 1253.49+ [31.8-32.2]

CID-MS3 MASCOT Search

prot_hit_nur	prot_acc	prot_desc	prot_score	prot_mass	prot_matche	pep_query	pep_rank	pep_isbold	pep_exp_mz	pep_exp_mr	pep_exp_z	pep_calc_mr	pep_delta	pep_miss	pep_score	pep_expect	pep_res_bef	pep_seq
1	KNG1_HUMA	Kininogen-1	18	72996	1	1	1	1	1253.4884	1252.4811	1	1252.5612	-0.0801	0	24.42	59	S	FNPISDFPDT
2	DPYS_HUMA	Dihydropyrir	14	57107	1	1	2	0	1253.4884	1252.4811	1	1252.5481	-0.067	0	18.65	2.20E+02	T	HMQFPFMGS
3	ARHG4_HUM	Rho guanine	9	76159	1	1	5	0	1253.4884	1252.4811	1	1252.7251	-0.244	0	14.03	6.50E+02	L	VLAEPRRKPS
4	RUVB1_HUM	RuvB-like 1 (	8	50538	1	1	8	0	1253.4884	1252.4811	1	1252.7139	-0.2328	0	13.59	7.20E+02	T	PANLLAKING
5	CCD99_HUM	Coiled-coil d	8	70703	1	1	6	0	1253.4884	1252.4811	1	1252.6155	-0.1344	0	13.78	6.90E+02	Q	MQRMKLQIA
6	DUS8_HUMA	Dual specific	8	66598	1	1	7	0	1253.4884	1252.4811	1	1251.6023	0.8788	0	13.77	6.90E+02	S	ISPNFNFLGQ
7	RECQ5_HUM	ATP-depend	8	110500	1	1	3	0	1253.4884	1252.4811	1	1252.503	-0.0219	0	14.81	5.40E+02	K	TCIGPSQGNC
8	CREM_HUMA	cAMP-respoi	8	35680	1	1	9	0	1253.4884	1252.4811	1	1252.6663	-0.1852	0	13.49	7.40E+02	E	IHTVQVAAIA
9	MAP2_HUMA	Microtubule-	7	199873	1	1	4	0	1253.4884	1252.4811	1	1252.5434	-0.0623	0	14.57	5.70E+02	S	PMPSPFQGG
10	TIE2_HUMAN	Angiopoietir	7	127897	1	1	9	0	1253.4884	1252.4811	1	1252.6775	-0.1964	0	13.49	7.40E+02	D	QHVDVKIKN.

Biotools-Score: 21

MASCOT-Score: 24

unknown O-glycosylation site

Kininogen-1 precursor

$_{600}$FNPI**S**DFPD**TT**$_{610}$

Fraction 16

734.26+++ → Pep [M+H]+ 1253.49+ [31.8-32.2] ETD

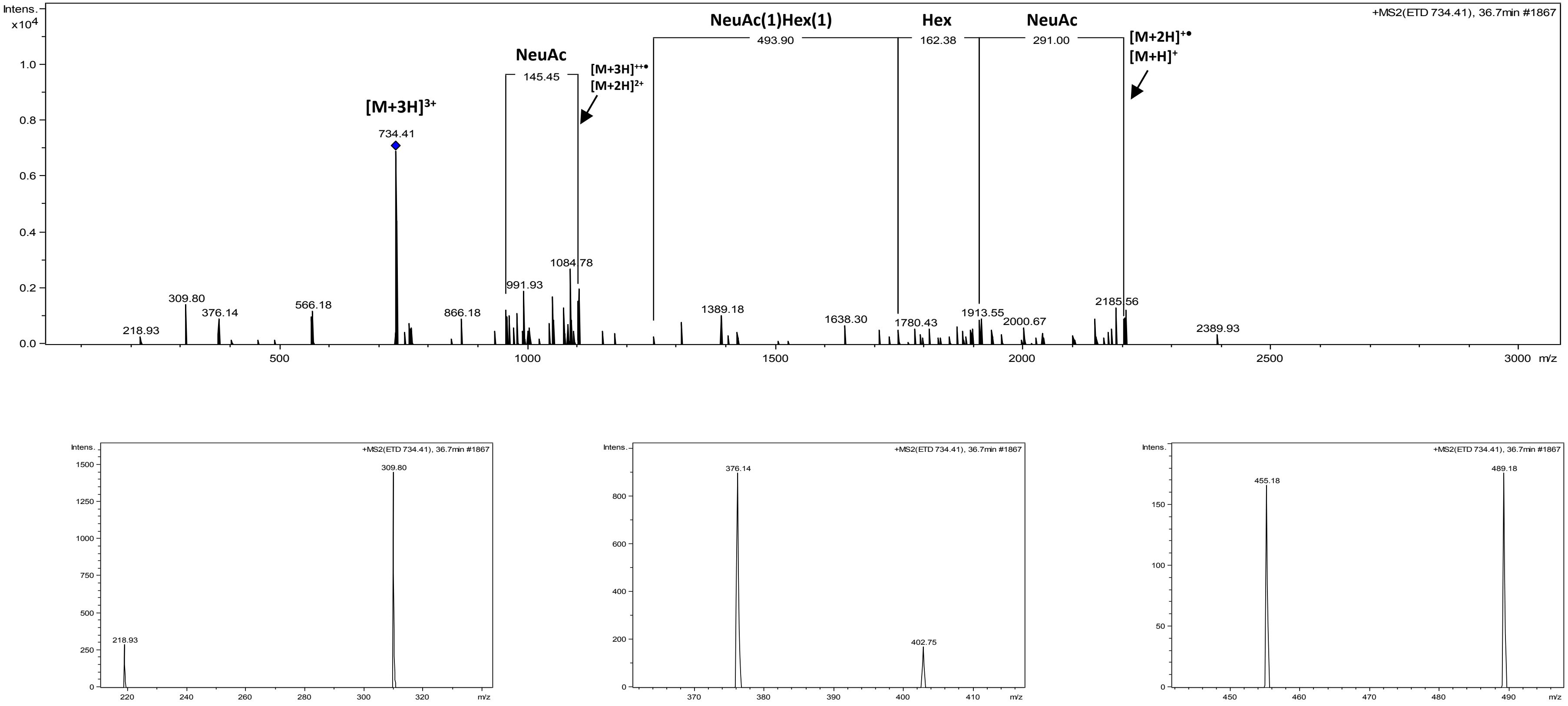

unknown O-glycosylation site

Kininogen-1 precursor

$_{600}$FNPI**S**DFPDTT$_{610}$

Fraction 16

734.26+++ → Pep [M+H]+ 1253.49+ [31.8-32.2]

ETD

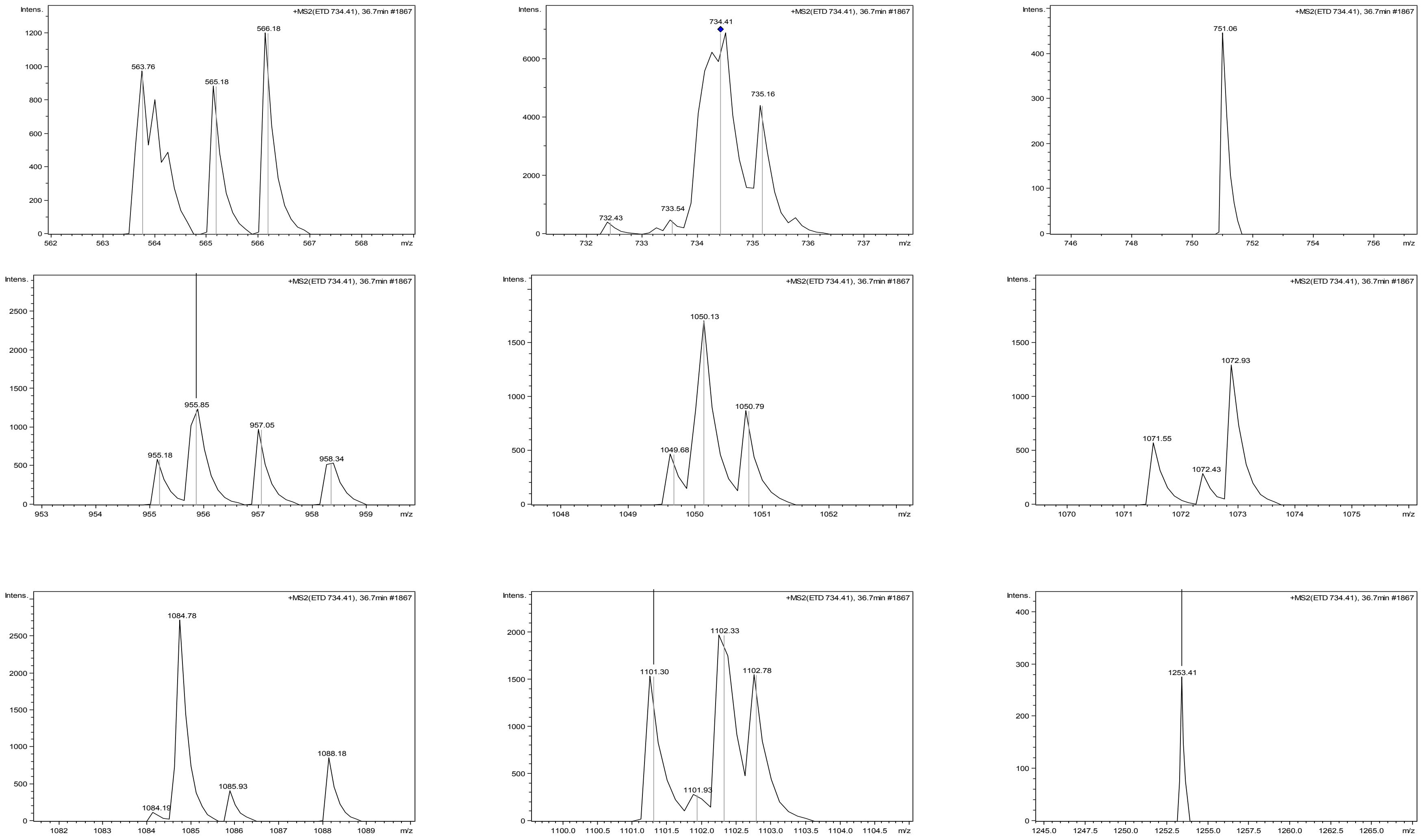

unknown O-glycosylation site

Kininogen-1 precursor

$_{600}$FN PI**S**DFPDTT$_{610}$

Fraction 16

734.26+++ → Pep [M+H]+ 1253.49+ [31.8-32.2]

ETD

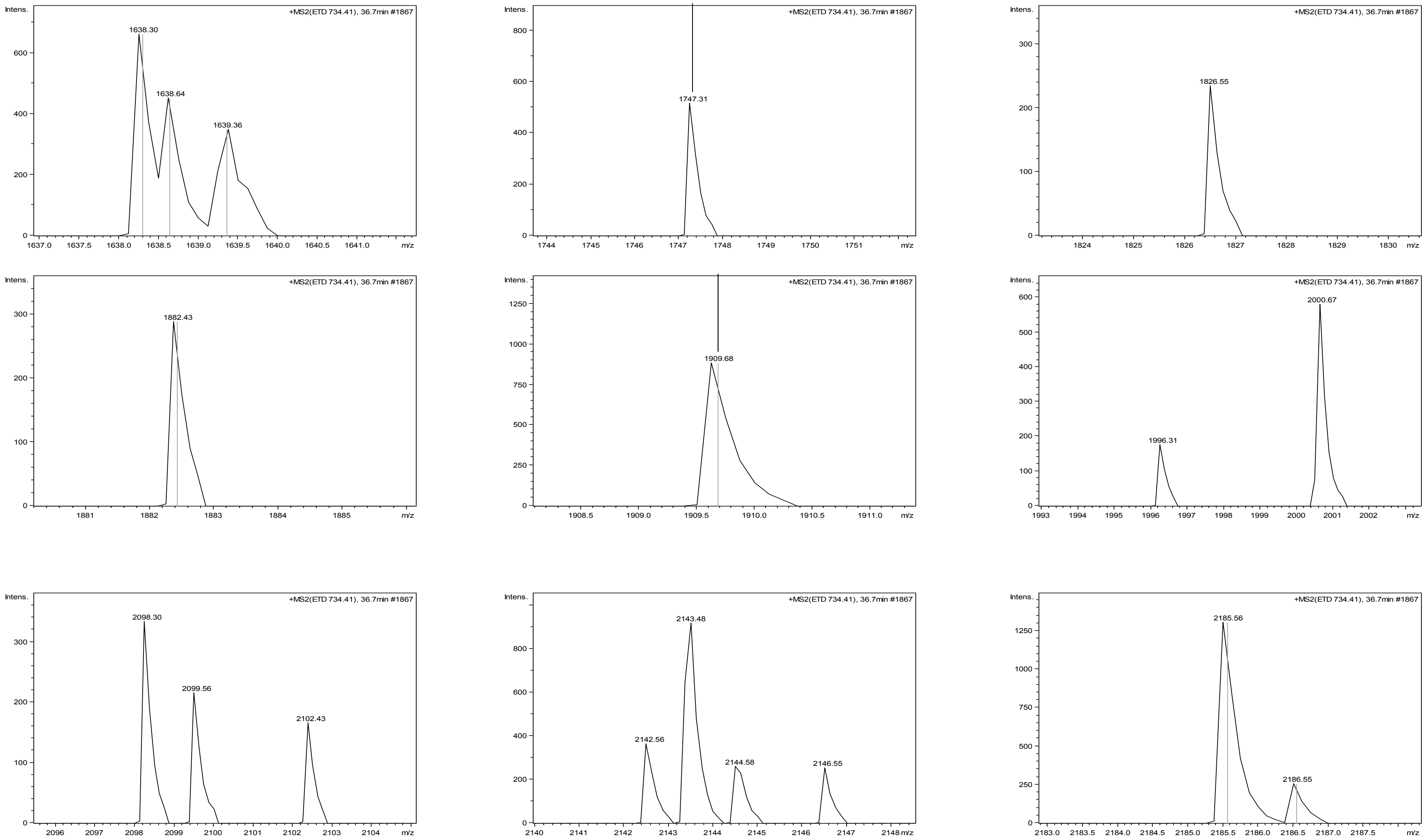

unknown O-glycosylation site

Kininogen-1 precursor

$_{600}$FNPI**S**DFPDTT$_{610}$

Fraction 16

734.26+++ → Pep [M+H]+ 1253.49+ [31.8-32.2] ETD

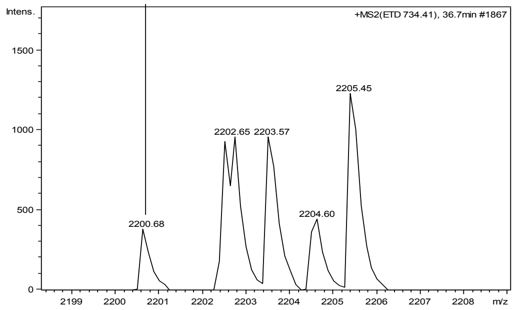

unknown O-glycosylation site

Kininogen-1 precursor

$_{600}$FNPI**S**DFPDTT$_{610}$

Fraction 16

734.26+++ → Pep [M+H]+ 1253.49+ [31.8-32.2] ETD

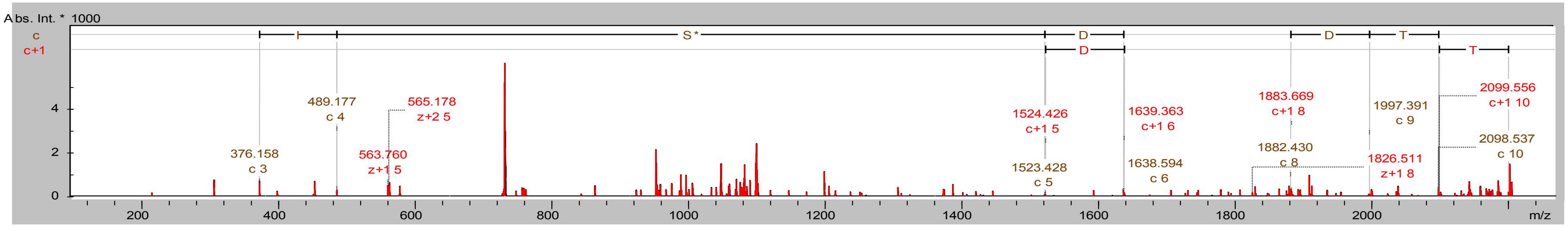

$_{600}$FNPI**S**DFPDTT$_{610}$

	F	N	P	I	S	D	F	P	D	T	T	Phe	Asn	Pro	Ile	Ser	Asp	Phe	Pro	Asp	Thr	Thr
Ion	1	2	3	4	5	6	7	8	9	10	11	1	2	3	4	5	6	7	8	9	10	11
c	F	N	P	I	S*	D	F	P	D	T	T	165.102	279.145	376.198	489.282	1523.637	1638.664	1785.732	1882.785	1997.812	2098.860	2199.907
c+1	F	N	P	I	S*	D	F	P	D	T	T	166.110	280.153	377.206	490.290	1524.645	1639.672	1786.740	1883.793	1998.820	2099.868	2200.915
z	F	N	P	I	S*	D	F	P	D	T	T	103.039	204.087	319.114	416.166	563.235	678.262	1712.617	1825.701	1922.754	2036.797	2183.865
z+1	F	N	P	I	S*	D	F	P	D	T	T	104.047	205.094	320.121	417.174	564.243	679.270	1713.625	1826.709	1923.761	2037.804	2184.873
z+2	F	N	P	I	S*	D	F	P	D	T	T	105.055	206.102	321.129	418.182	565.250	680.277	1714.632	1827.716	1924.769	2038.812	2185.881
	11	10	9	8	7	6	5	4	3	2	1	Thr	Thr	Asp	Pro	Phe	Asp	Ser	Ile	Pro	Asn	Phe

Biotools-Score: 44

unknown O-glycosylation site

Kininogen-1 precursor

Most likely **S**(604), highest ETD-Biotools-Score

$_{600}$FNPI**S**DFPDTT$_{610}$

Fraction 16

734.26+++ → Pep [M+H]+ 1253.49+ [31.8-32.2] ETD

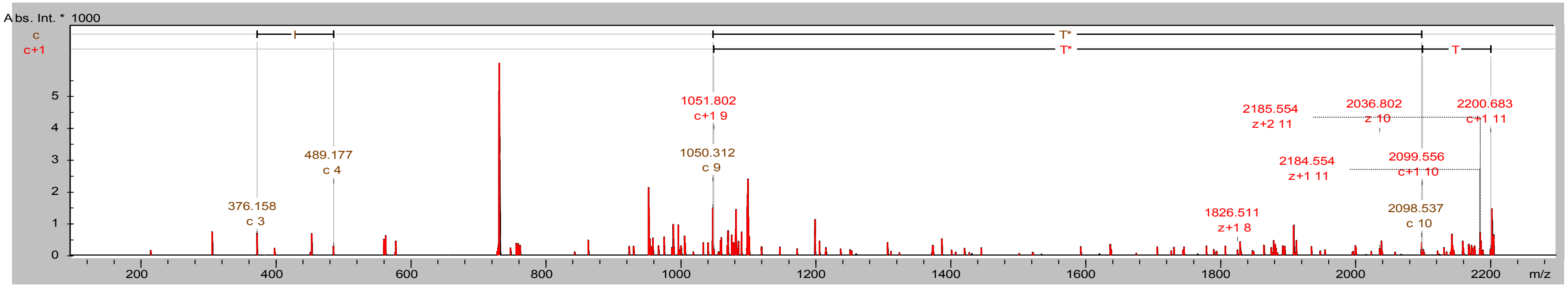

$_{600}$FNPISDFPDTT$_{610}$

	F	N	P	I	S	D	F	P	D	T	T	Phe	Asn	Pro	Ile	Ser	Asp	Phe	Pro	Asp	Thr	Thr
Ion	1	2	3	4	5	6	7	8	9	10	11	1	2	3	4	5	6	7	8	9	10	11
c	F	N	P	I	S	D	F	P	D	T*	T	165.102	279.145	376.198	489.282	576.314	691.341	838.409	935.462	1050.489	2098.860	2199.907
c+1	F	N	P	I	S	D	F	P	D	T*	T	166.110	280.153	377.206	490.290	577.322	692.349	839.417	936.470	1051.497	2099.868	2200.915
z	F	N	P	I	S	D	F	P	D	T*	T	103.039	1151.410	1266.437	1363.489	1510.558	1625.585	1712.617	1825.701	1922.754	2036.797	2183.865
z+1	F	N	P	I	S	D	F	P	D	T*	T	104.047	1152.418	1267.444	1364.497	1511.566	1626.593	1713.625	1826.709	1923.761	2037.804	2184.873
z+2	F	N	P	I	S	D	F	P	D	T*	T	105.055	1153.425	1268.452	1365.505	1512.573	1627.600	1714.632	1827.716	1924.769	2038.812	2185.881
	11	10	9	8	7	6	5	4	3	2	1	Thr	Thr	Asp	Pro	Phe	Asp	Ser	Ile	Pro	Asn	Phe

Biotools-Score: 22

unknown O-glycosylation site

Most likely **S**(604), highest ETD-Biotools-Score

Kininogen-1 precursor

$_{600}$FNPI**S**DFPDTT$_{610}$

Fraction 16

734.26+++ → Pep [M+H]+ 1253.49+ [31.8-32.2] ETD

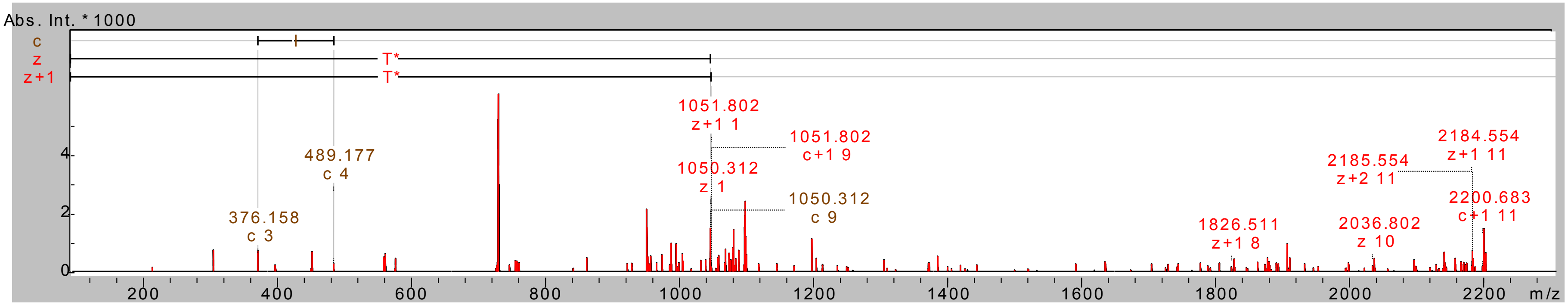

$_{600}$FNPISDFPDT**T**$_{610}$

	F	N	P	I	S	D	F	P	D	T	T	Phe	Asn	Pro	Ile	Ser	Asp	Phe	Pro	Asp	Thr	Thr
Ion	1	2	3	4	5	6	7	8	9	10	11	1	2	3	4	5	6	7	8	9	10	11
c	F	N	P	I	S	D	F	P	D	T	T*	165.102	279.145	376.198	489.282	576.314	691.341	838.409	935.462	1050.489	1151.537	2199.907
c+1	F	N	P	I	S	D	F	P	D	T	T*	166.110	280.153	377.206	490.290	577.322	692.349	839.417	936.470	1051.497	1152.545	2200.915
z	F	N	P	I	S	D	F	P	D	T	T*	1050.362	1151.410	1266.437	1363.489	1510.558	1625.585	1712.617	1825.701	1922.754	2036.797	2183.865
z+1	F	N	P	I	S	D	F	P	D	T	T*	1051.370	1152.418	1267.444	1364.497	1511.566	1626.593	1713.625	1826.709	1923.761	2037.804	2184.873
z+2	F	N	P	I	S	D	F	P	D	T	T*	1052.378	1153.425	1268.452	1365.505	1512.573	1627.600	1714.632	1827.716	1924.769	2038.812	2185.881
	11	10	9	8	7	6	5	4	3	2	1	Thr	Thr	Asp	Pro	Phe	Asp	Ser	Ile	Pro	Asn	Phe

Biotools-Score: 16

unknown O-glycosylation site

Most likely **S**(604), highest ETD-Biotools-Score

Kininogen-1 precursor

$_{600}$FNPI**S**DFPDTT$_{610}$
