# Supplementary material for: Site-specific O-Glycosylation Analysis of Human Blood Plasma Proteins
Source: Mol Cell Proteomics. 2015 Nov 23;15(2):624–41. doi: 10.1074/mcp.M115.053546 (PMC4739677; doi:10.1074/mcp.M115.053546)
Supplement: Supplemental Data [file 10.1074_M115.053546_mcp.M115.053546-7.pdf]

Site-Specific *O*-Glycosylation Analysis of Human Blood Plasma Proteins

Proteinase K Digest

Fraction 17

Search Parameters For Protein Identification

Project: Blood Plasma Glycoprotomics (2013), ProtK-Digest
Glycopeptides measured on Bruker ESI-Ion Trap MS (CID-MS^3)

Mascot version 2.2.07	
Database:	SwissProt
Fasta file:	SwissProt_51.6.fasta
Total sequences:	257964
Total residues:	93947433
Sequences after taxonomy filter:	15720
Number of queries:	1

Variable modifications ---

Identifier	*Name*	*Delta*	*Neutral loss(es)*
1	Deamidated (NQ)	0.984009	0
2	Oxidation (M)	15.994919	63.998285

Search Parameters ---

Taxonomy filter:	Homo sapiens (human)
Enzyme:	None
Maximum Missed Cleavages:	0
Fixed modifications	Carbamidomethyl (C)
ICAT experiment	0
Variable modifications	Deamidated (NQ), Oxidation (M)
Peptide Mass Tolerance	0.3
Peptide Mass Tolerance Units	Da
Fragment Mass Tolerance	0.35
Fragment Mass Tolerance Units	Da
Mass values	Monoisotopic
Instrument type	ESI-TRAP
Isotope error mode	1

Format parameters ---

Significance threshold	0.05
Max. number of hits	20
Use MudPIT protein scoring	0
Ions score cut-off	0
Include same-set proteins	0
Include sub-set proteins	0
Include unassigned	0
Require bold red	0

Extracted ion chromatograms of glycan-specific oxonium ions

**Oxonium Ions**	**$[M+H]^+$ m/z**
Fuc	147.08
Hex	163.06
HexNAc	204.09
NeuAc -H_2O	274.09
NeuAc	292.10
HexNAc(1)Hex(1)	366.14
Hex(1)NeuAc(1)	454.16
HexNAc(1)NeuAc(1)	495.18
HexNAc(1)Hex(1)Fuc(1)	512.21
HexNAc(1)Hex(2)	528.19
HexNAc(1)Hex(1)NeuAc(1)	657.24

Supplementary Figure 7: Human Blood Plasma O-Glycoproteomics, HILIC Fraction 17

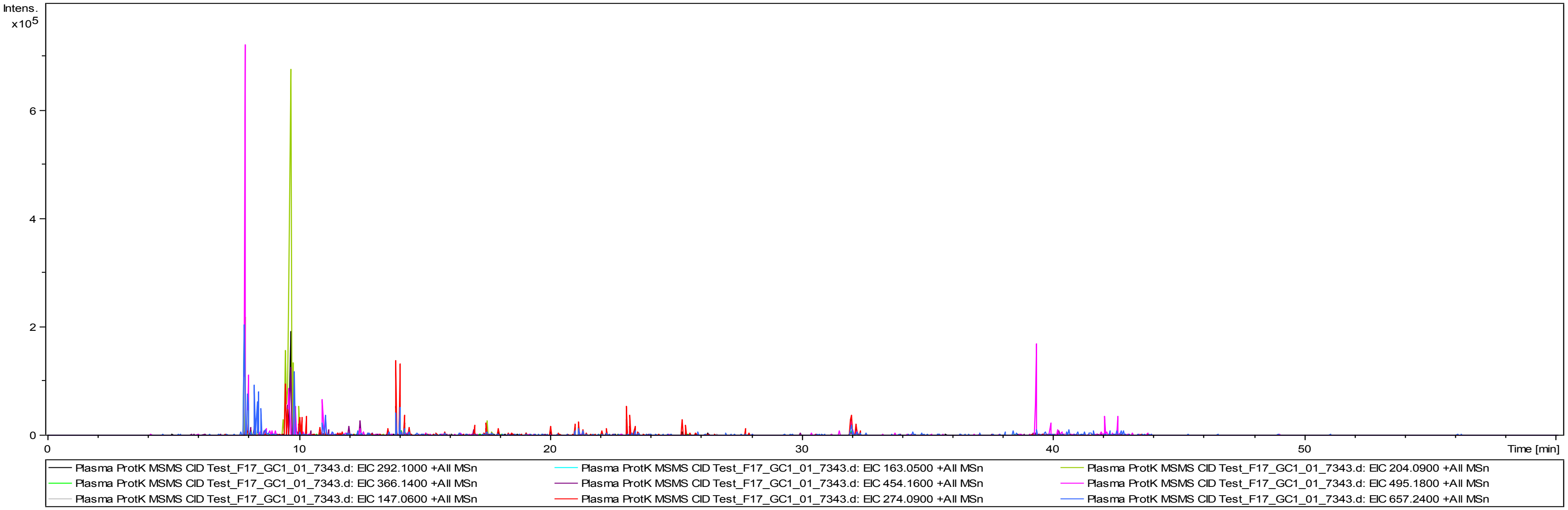

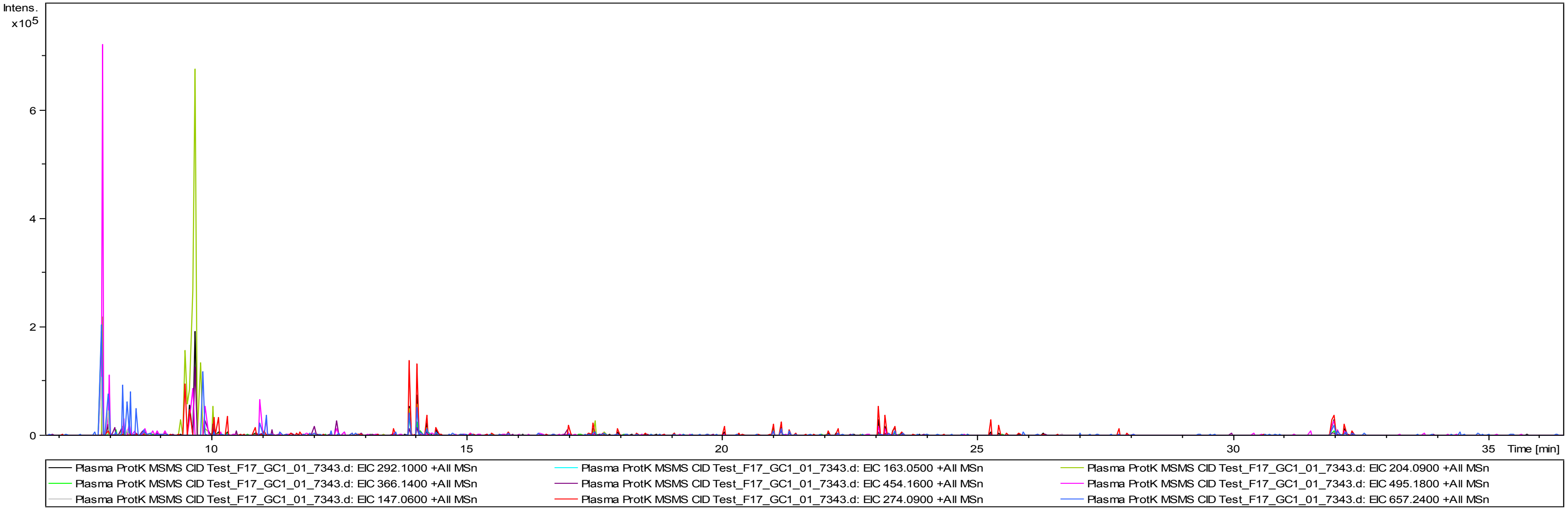

Extracted ion chromatograms of glycan-specific oxonium ions

Supplementary Figure 7: Human Blood Plasma O-Glycoproteomics, HILIC Fraction 17

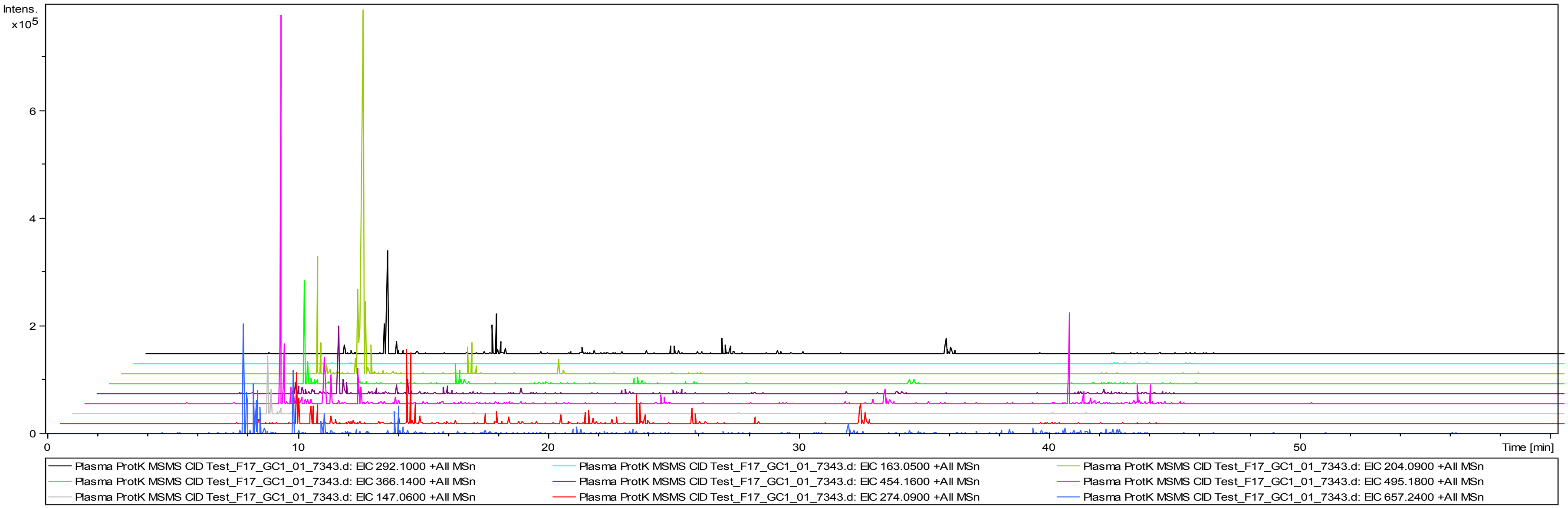

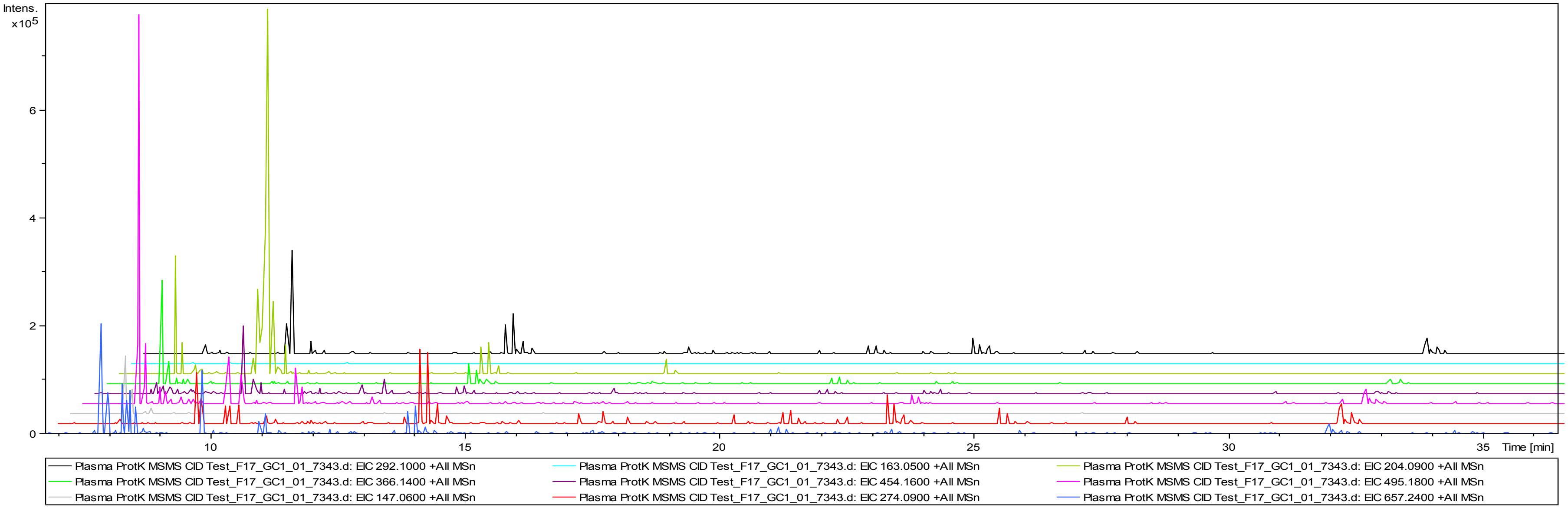

Extracted ion chromatograms of glycan-specific oxonium ions

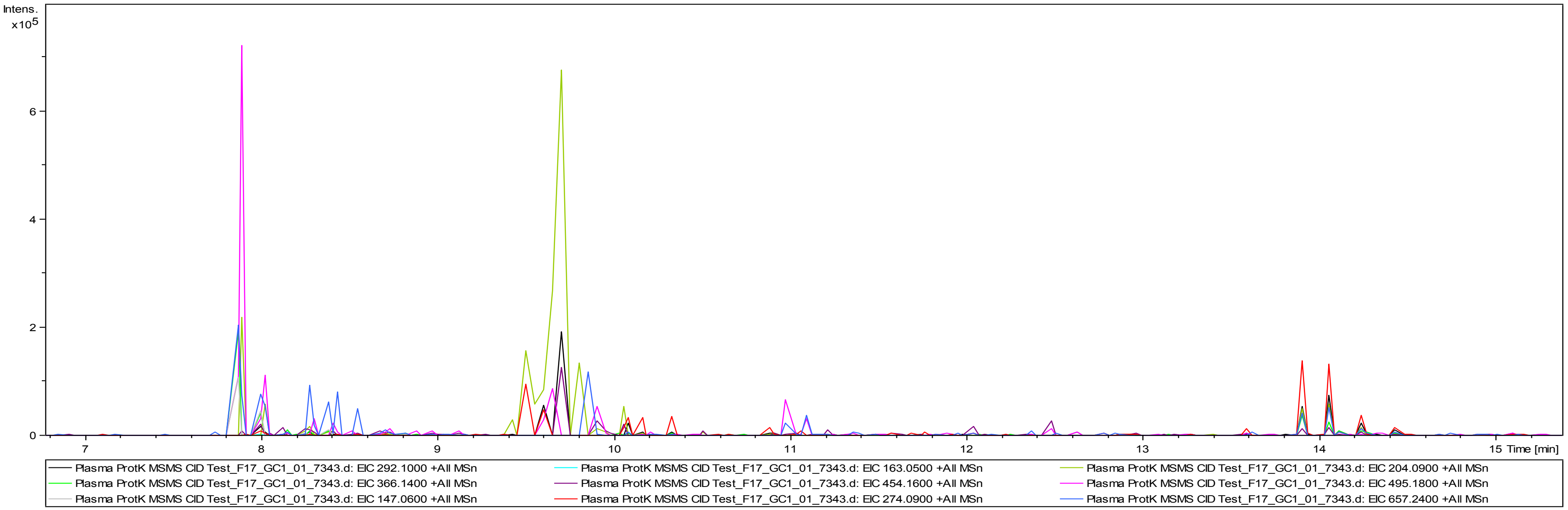

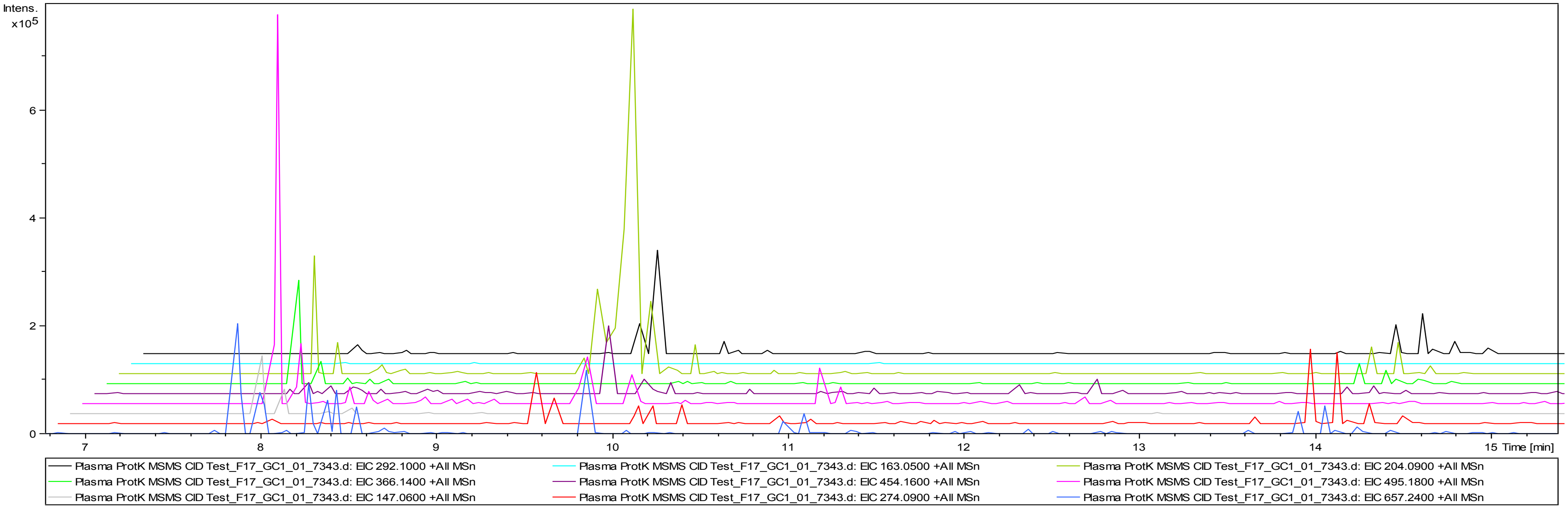

Extracted ion chromatograms of glycan-specific oxonium ions

Supplementary Figure 7: Human Blood Plasma O-Glycoproteomics, HILIC Fraction 17

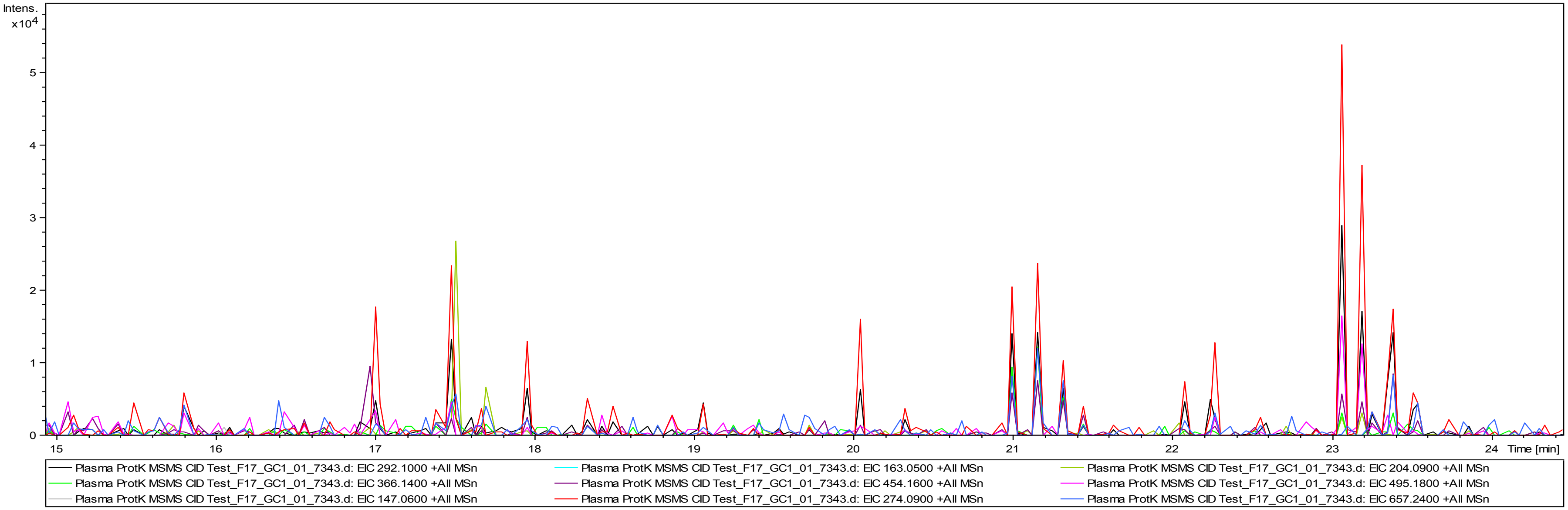

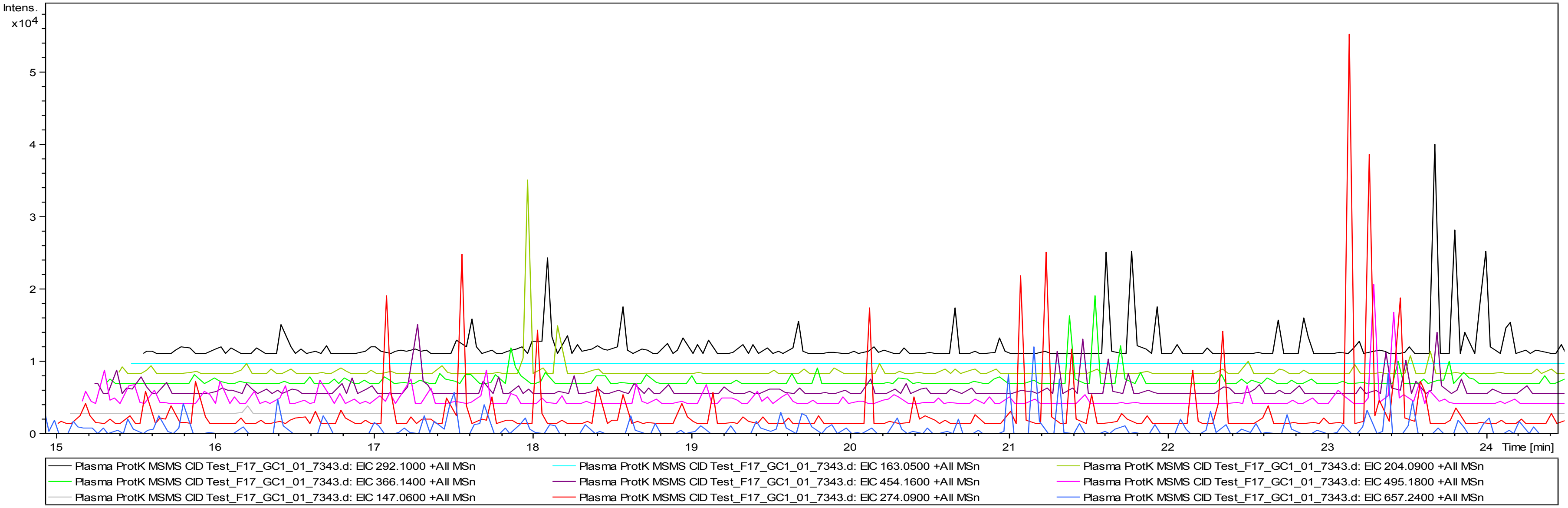

Extracted ion chromatograms of glycan-specific oxonium ions

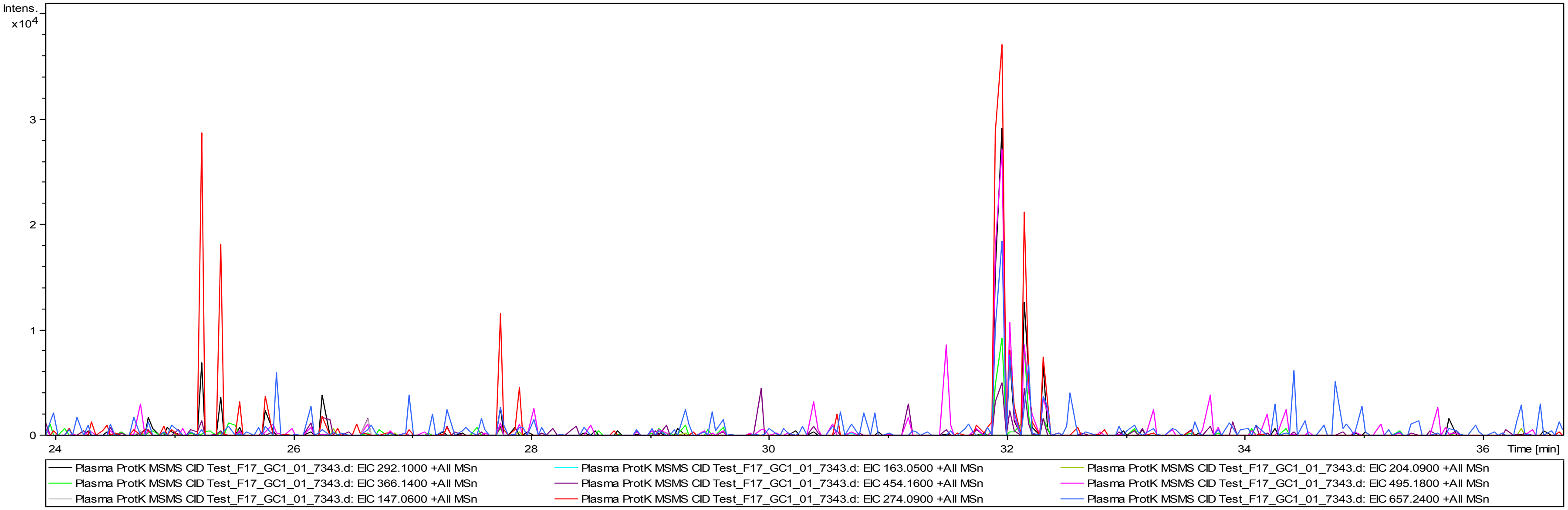

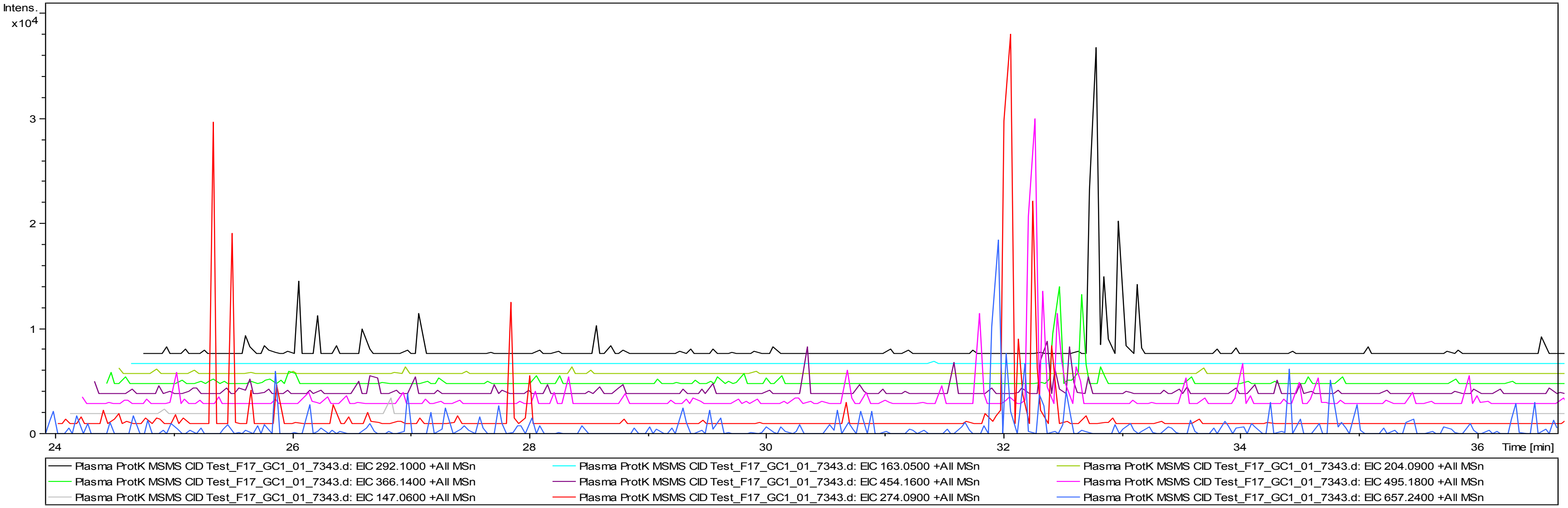

Extracted ion chromatograms of glycan-specific oxonium ions

Fraction 17

789.21++ → Pep [M+H]+ 630.29+ [10.1 min] CID-MS Precursor

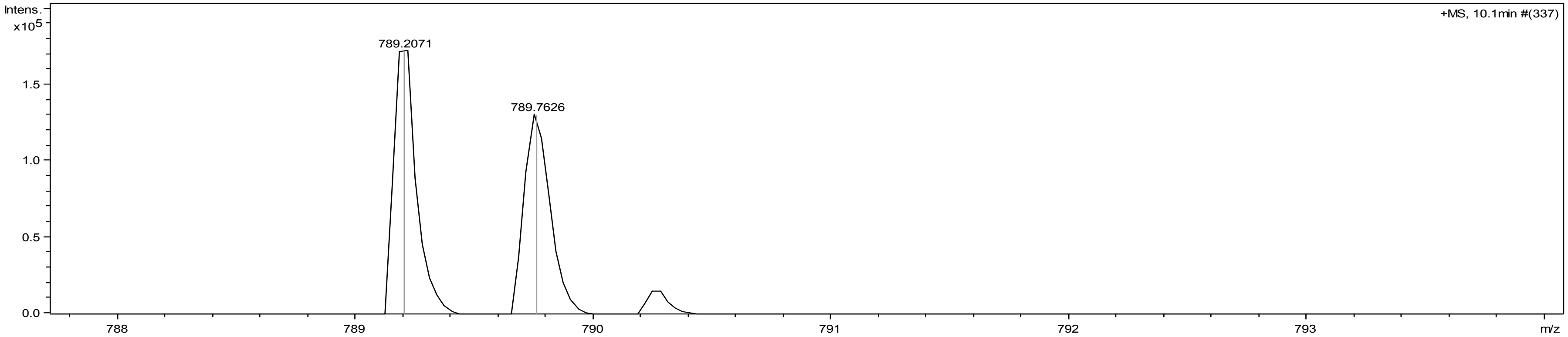

CID MS³ spectrum of poor quality

ETD spectrum of poor quality

Fraction 17

789.21++ → Pep [M+H]+ 630.29+ [10.1 min] CID-MS2

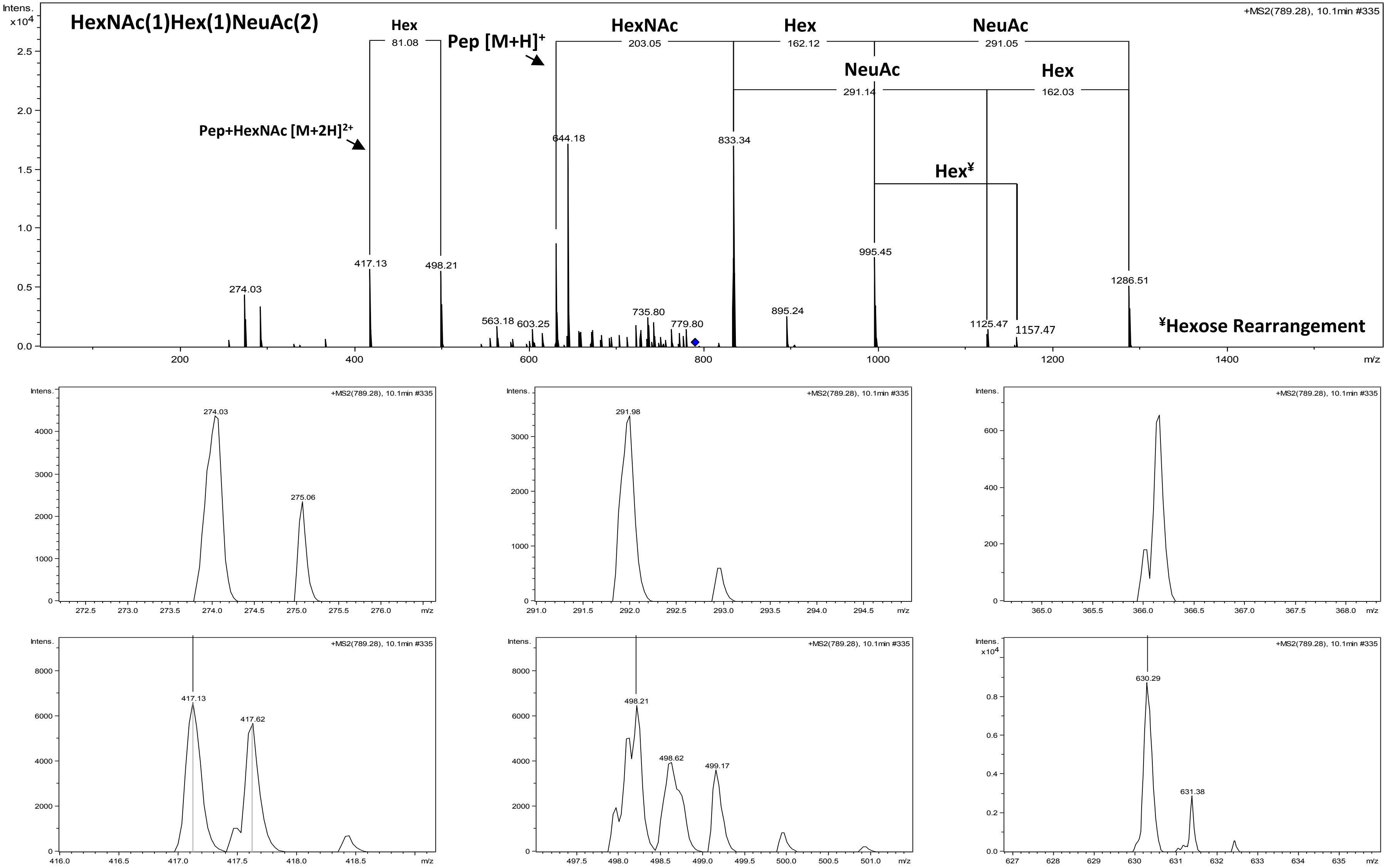

Fraction 17

789.21++ → Pep [M+H]+ 630.29+ [10.1 min]

CID-MS2

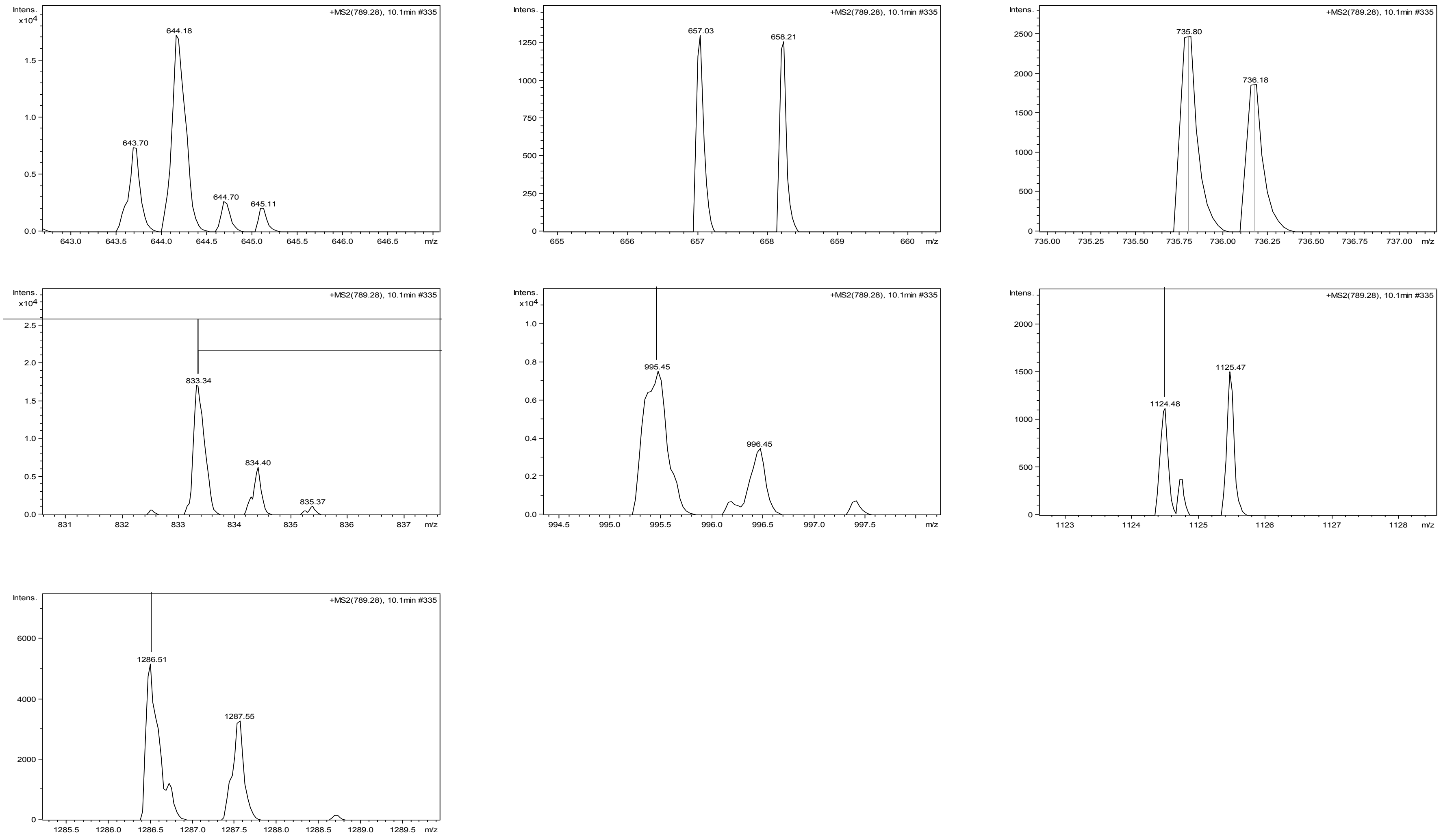

Fraction 17

570.23+++ → Pep [M+2H]++ 526.72++ [10.2-10.3 min] CID-MS Precursor

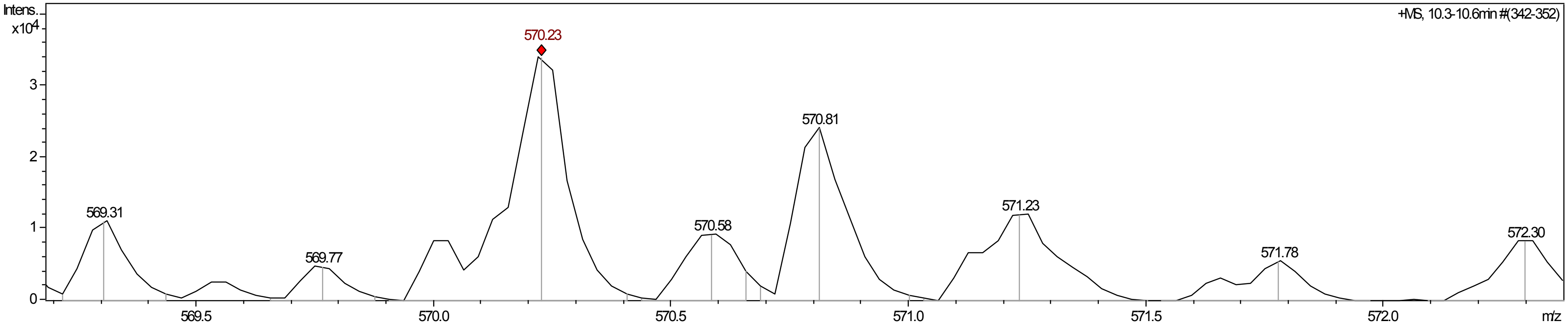

Precursor spectrum of poor quality

CID MS^3 spectrum of poor quality

ETD spectrum of poor quality

Fraction 17

570.23+++ → Pep [M+2H]++ 526.72++ [10.2-10.3 min] CID-MS2

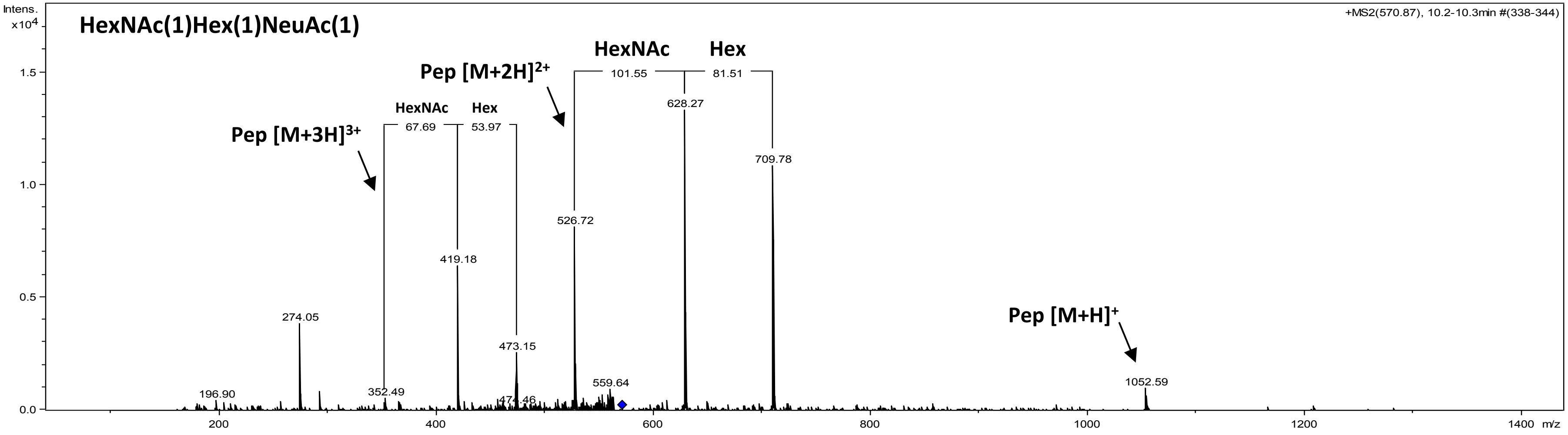

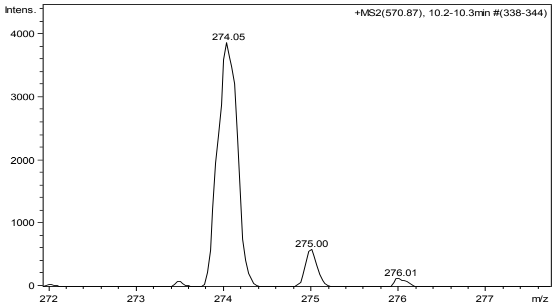

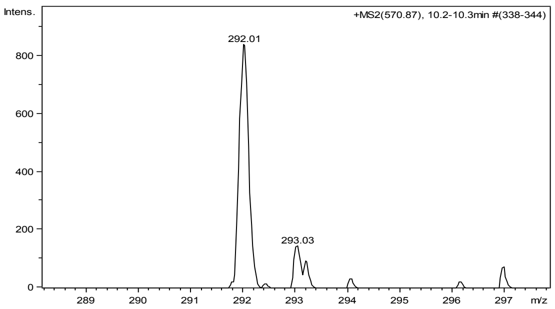

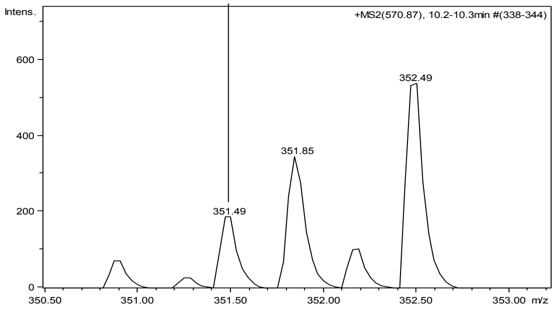

Fraction 17

570.23+++ → Pep [M+2H]++ 526.72++ [10.2-10.3 min] CID-MS2

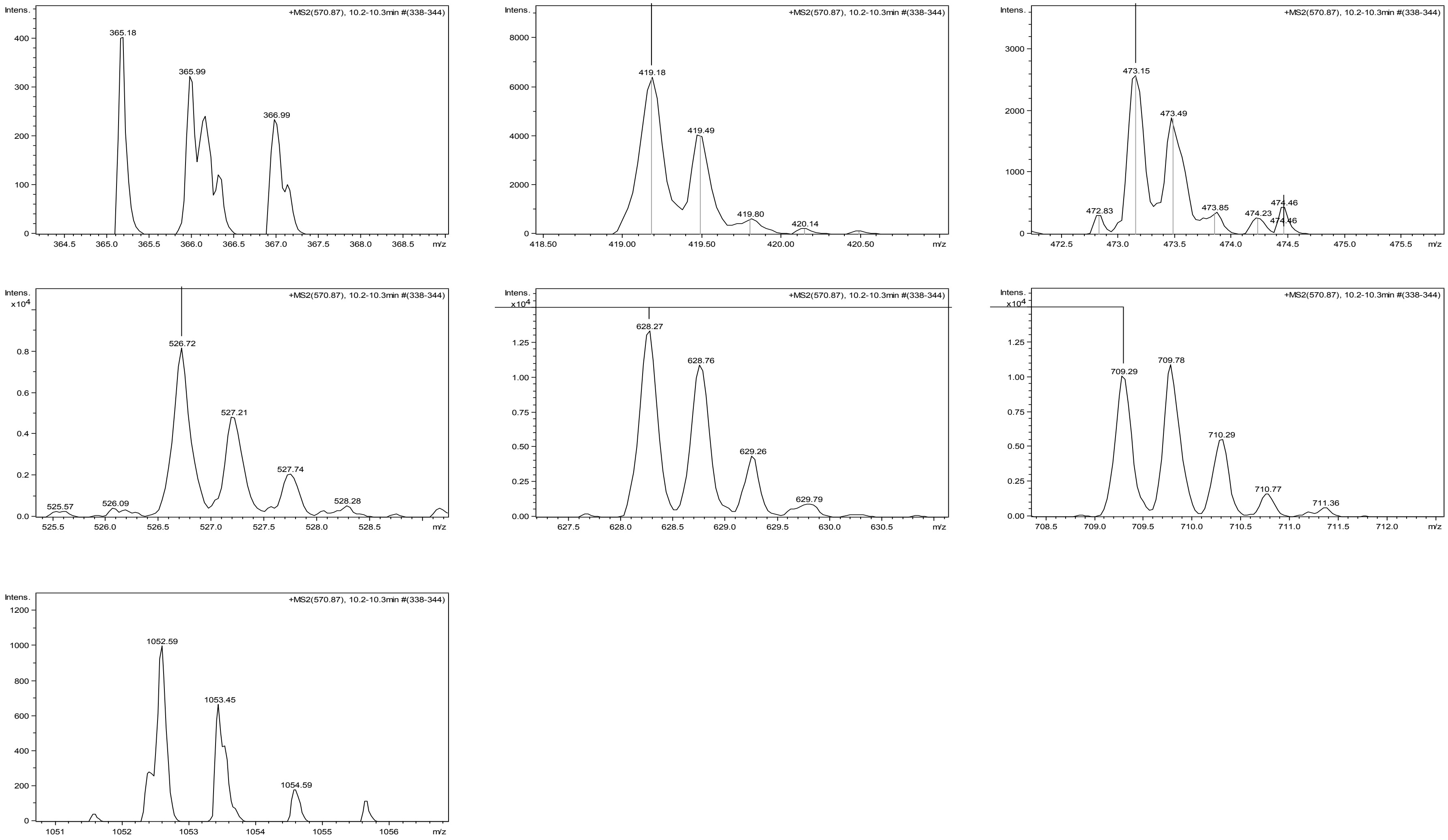

Fraction 17

599.21+++ → Pep [M+2H]++ 570.25++ [10.9 min] CID-MS Precursor

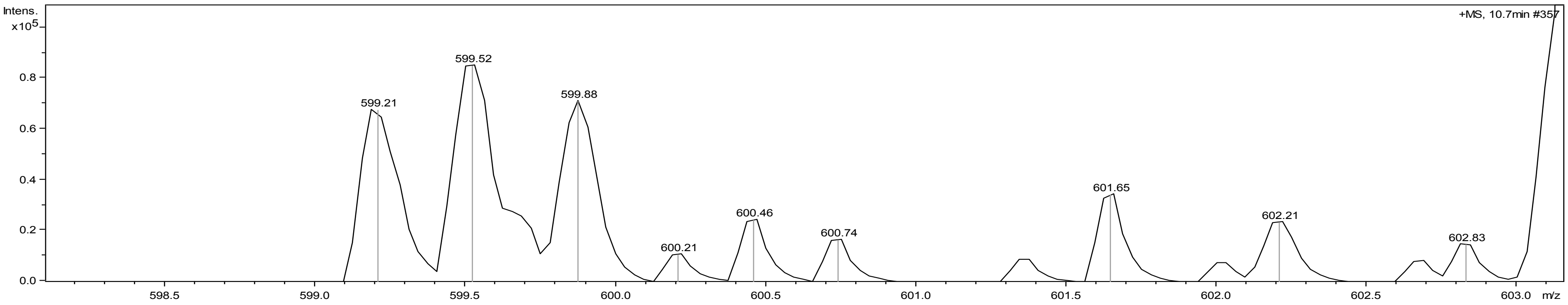

CID MS³ spectrum of poor quality

ETD spectrum of poor quality

Fraction 17

599.21+++ → Pep [M+2H]++ 570.25++ [10.9 min] CID-MS2

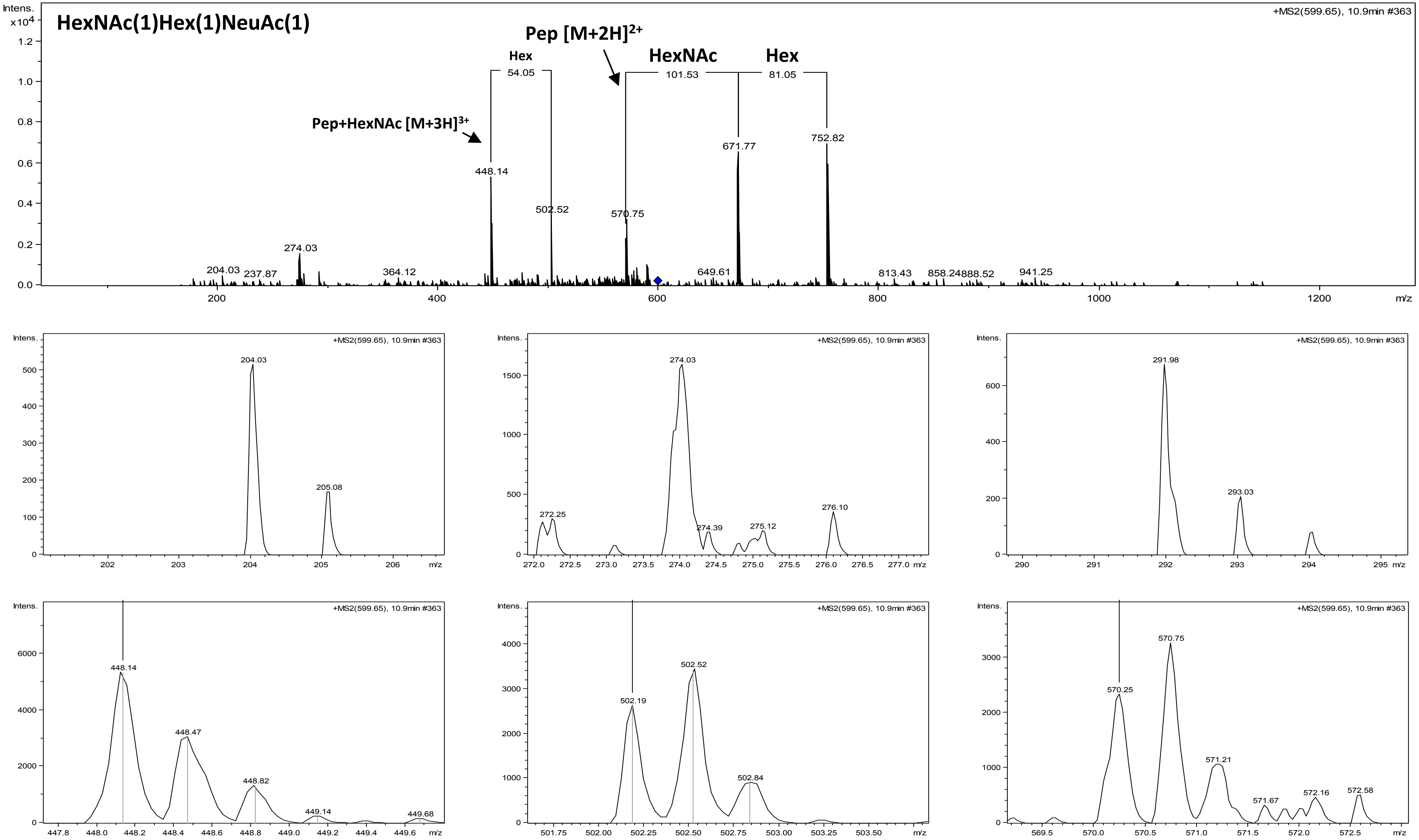

Fraction 17

599.21+++ → Pep [M+2H]++ 570.25++ [10.9 min] CID-MS2

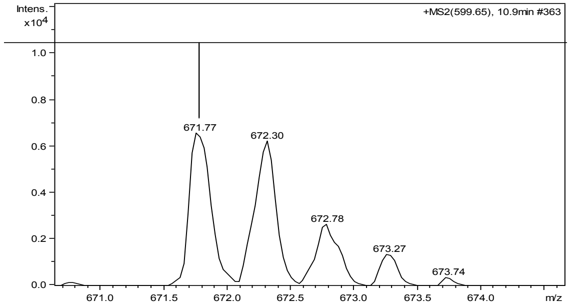

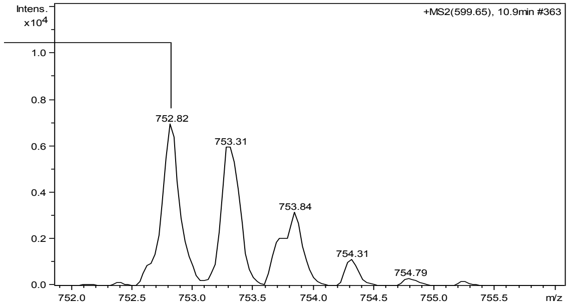

Fraction 17

653.18+++ → Pep+HexNAc(1)NeuAc(1) [M+2H]++ 752.76++ [13.5-13.6 min]

CID-MS Precursor

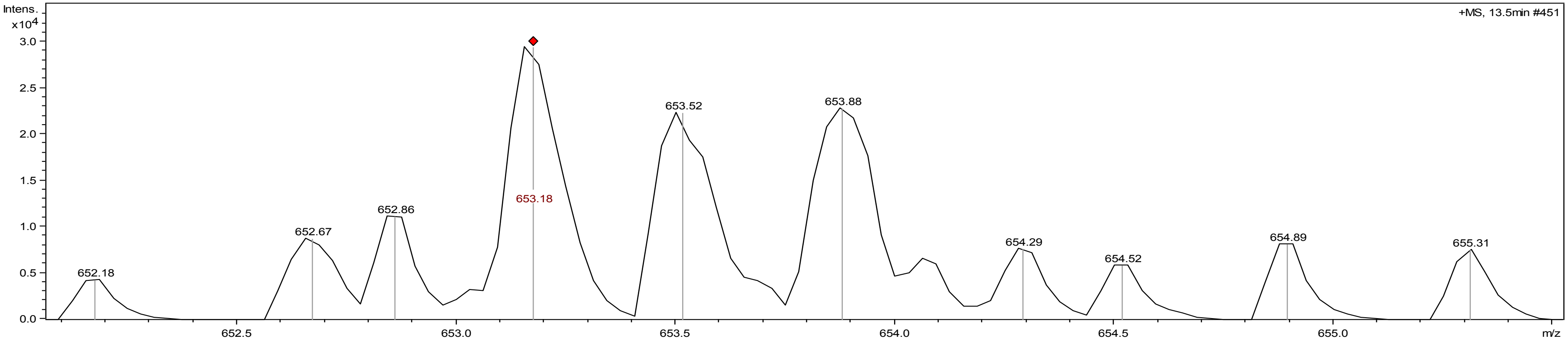

CID MS³ spectrum of poor quality

ETD spectrum of poor quality

Fraction 17

653.18+++ → Pep+HexNAc(1)NeuAc(1) [M+2H]++ 752.76++ [13.5-13.6 min]

CID-MS2

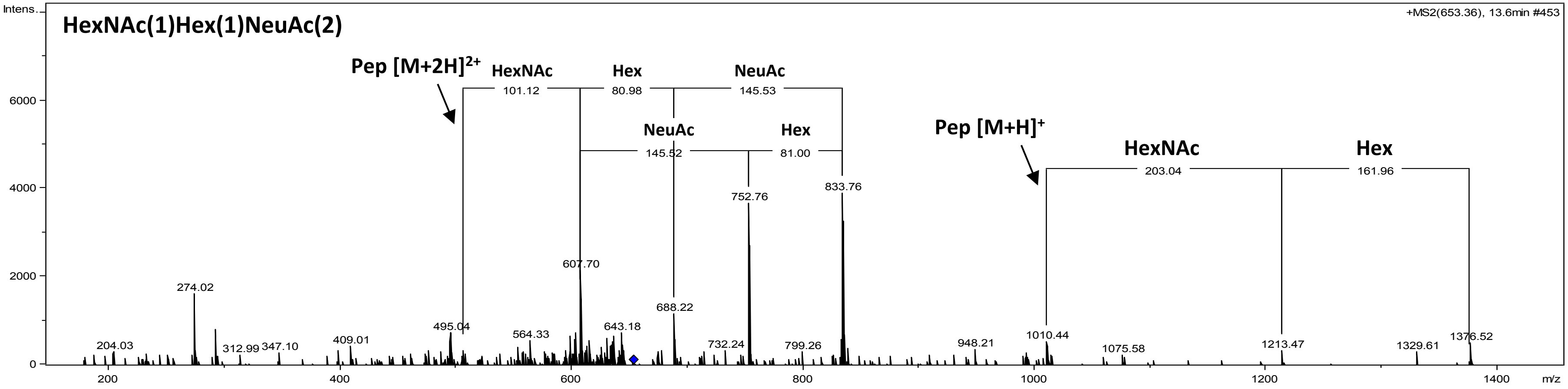

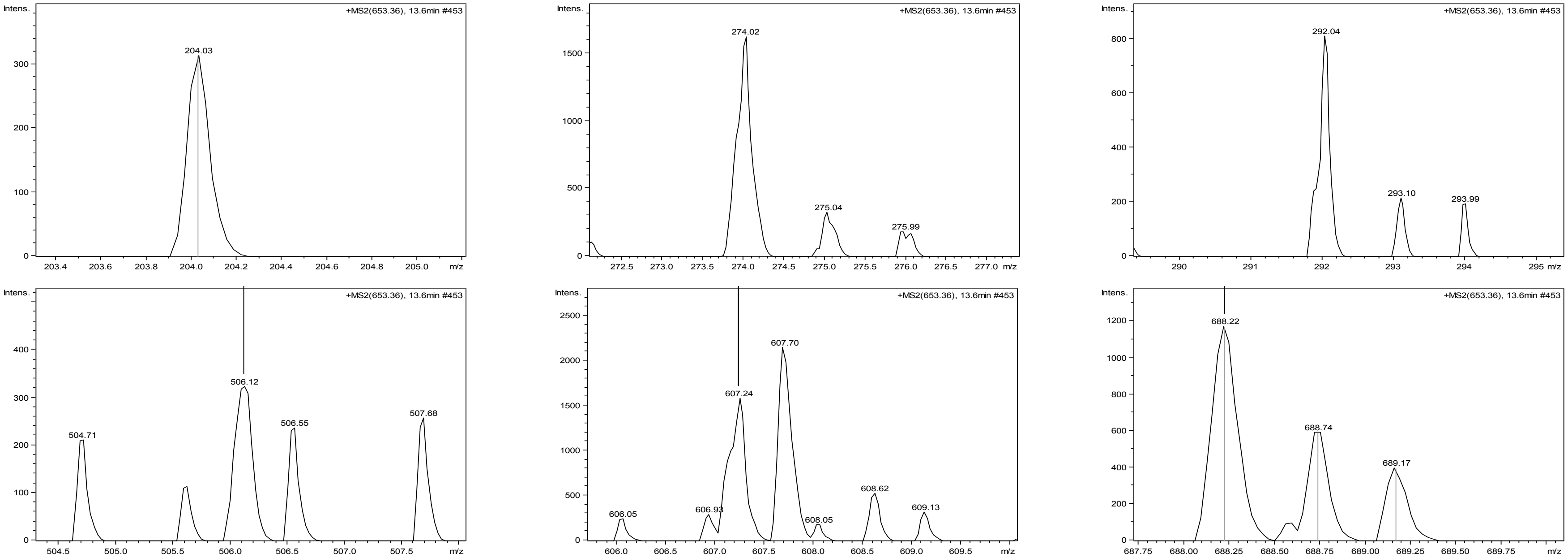

Fraction 17

653.18+++ → Pep+HexNAc(1)NeuAc(1) [M+2H]++ 752.76++ [13.5-13.6 min]

CID-MS2

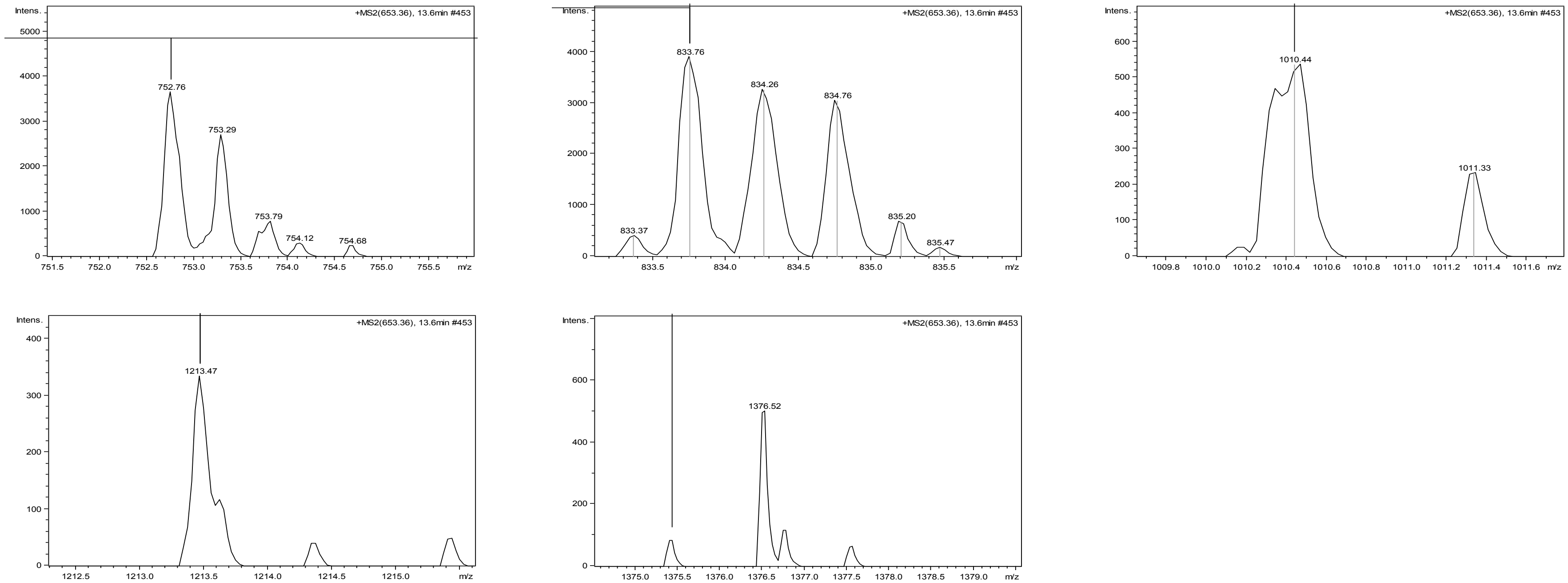

Fraction 17

623.23+++ → Pep [M+2H]++ 606.25++ [13.9-14.0 min] CID-MS Precursor

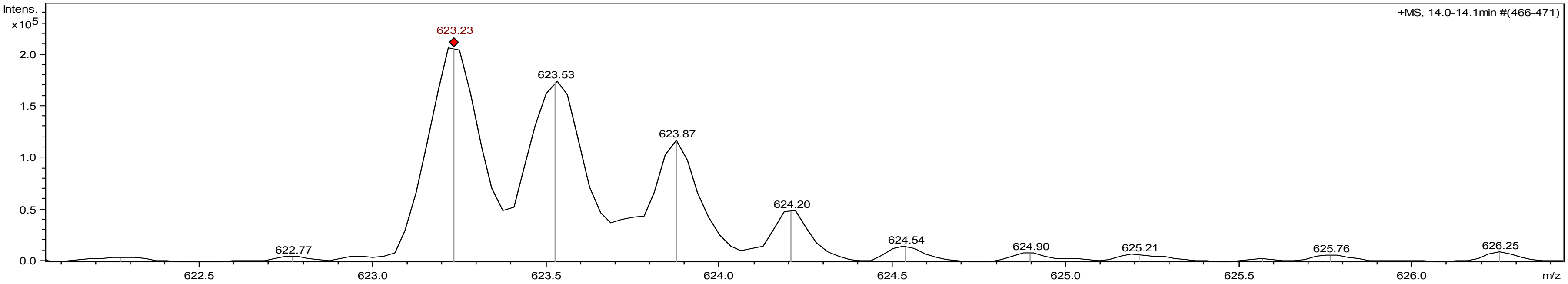

Fraction 17

623.23+++ → Pep [M+2H]++ 606.25++ [13.9-14.0 min] CID-MS2

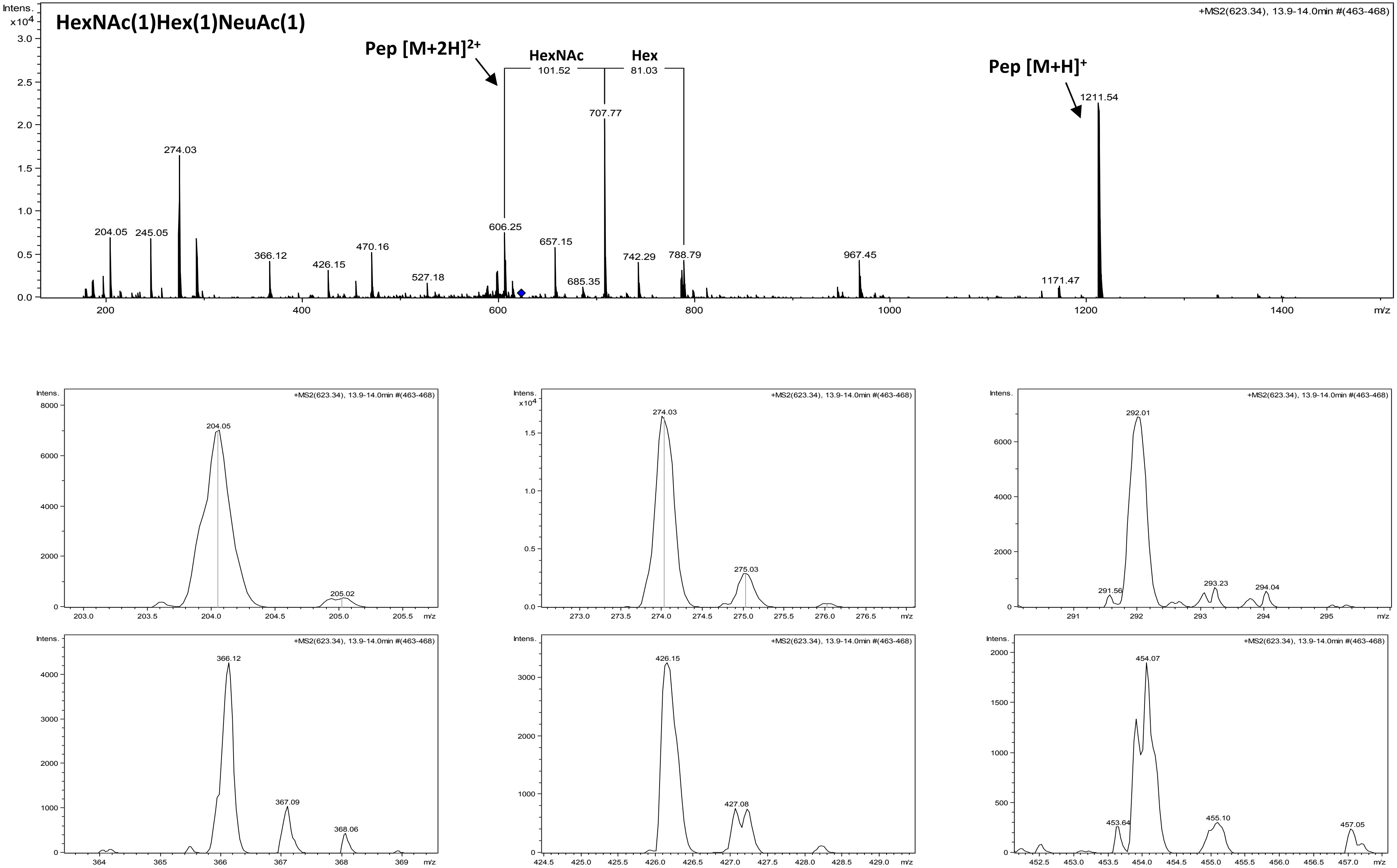

Fraction 17

623.23+++ → Pep [M+2H]++ 606.25++ [13.9-14.0 min] CID-MS2

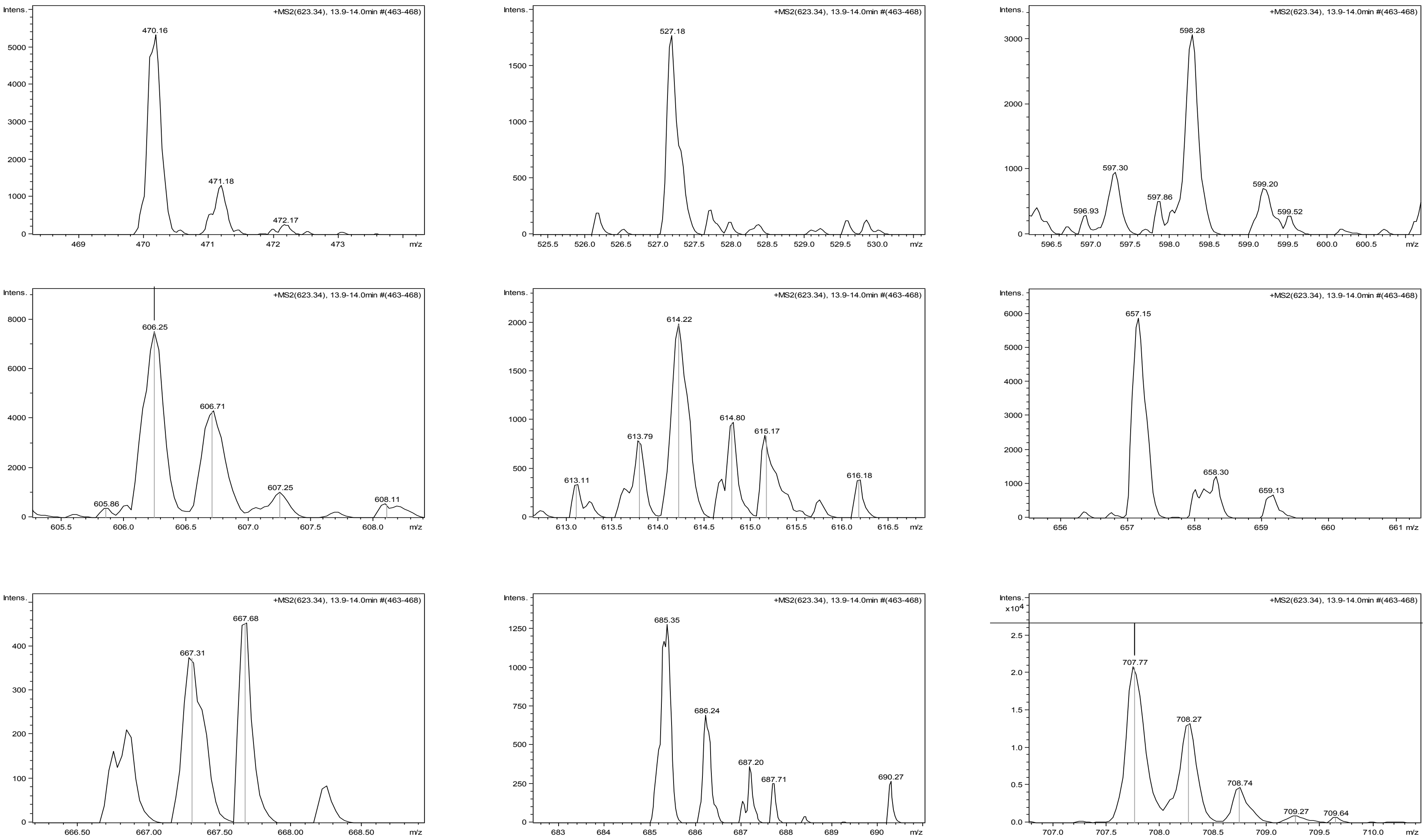

Fraction 17

623.23+++ → Pep [M+2H]++ 606.25++ [13.9-14.0 min] CID-MS2

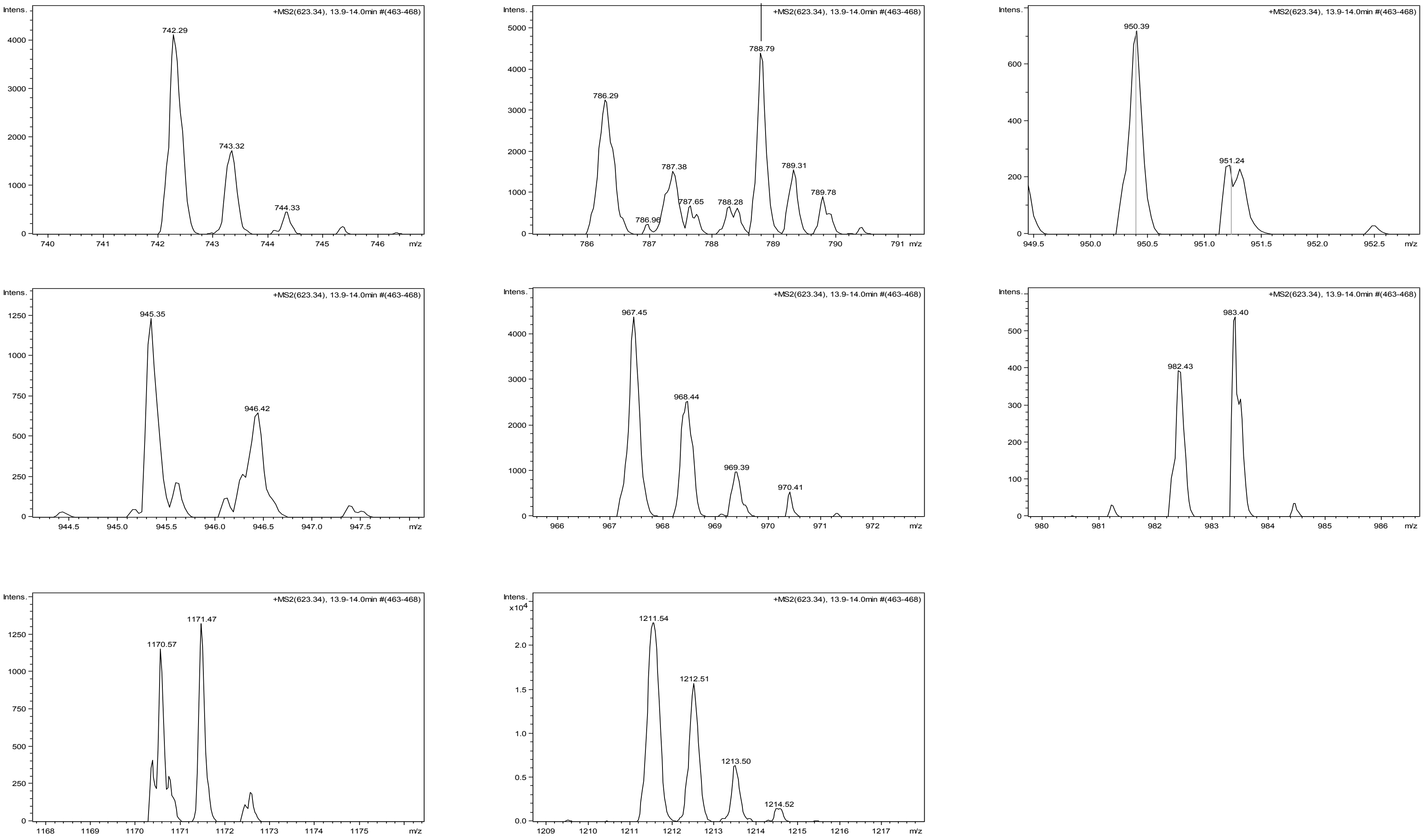

Fraction 17

623.23+++ → Pep [M+2H]++ 606.25++ [13.9-14.0 min]

CID-MS2 MASCOT Search

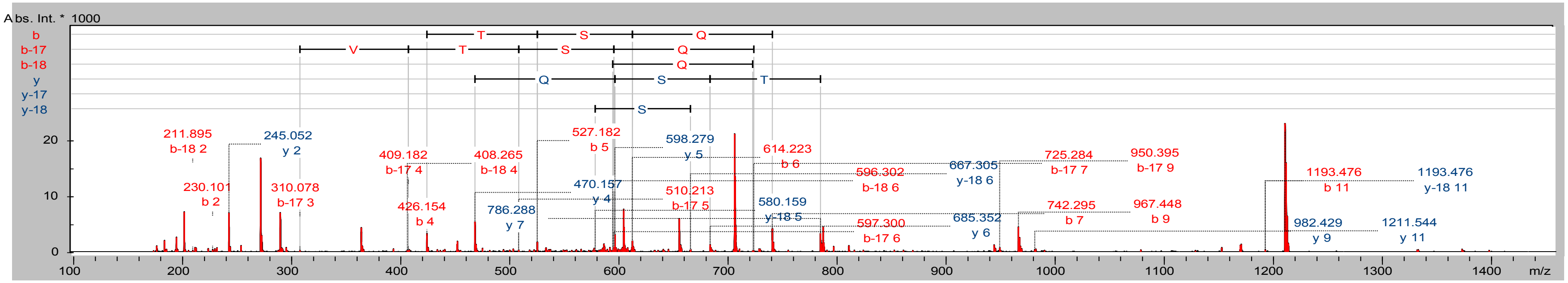

	T	Q	P	V	T	S	Q	P	Q	P	E	Thr	Gln	Pro	Val	Thr	Ser	Gln	Pro	Gln	Pro	Glu
Ion	1	2	3	4	5	6	7	8	9	10	11	1	2	3	4	5	6	7	8	9	10	11
b	T	Q	P	V	T	S	Q	P	Q	P	E	102.055	230.114	327.166	426.235	527.282	614.314	742.373	839.426	967.484	1064.537	1193.580
b-17	T	Q	P	V	T	S	Q	P	Q	P	E	-	213.087	310.140	409.208	510.256	597.288	725.346	822.399	950.458	1047.511	1176.553
b-18	T	Q	P	V	T	S	Q	P	Q	P	E	84.044	212.103	309.156	408.224	509.272	596.304	724.362	821.415	949.474	1046.527	1175.569
y	T	Q	P	V	T	S	Q	P	Q	P	E	148.060	245.113	373.172	470.225	598.283	685.315	786.363	885.431	982.484	1110.543	1211.590
y-17	T	Q	P	V	T	S	Q	P	Q	P	E	-	-	356.145	453.198	581.257	668.289	769.336	868.405	965.457	1093.516	1194.564
y-18	T	Q	P	V	T	S	Q	P	Q	P	E	130.050	227.103	355.161	452.214	580.273	667.305	768.352	867.421	964.473	1092.532	1193.580
	11	10	9	8	7	6	5	4	3	2	1	Glu	Pro	Gln	Pro	Gln	Ser	Thr	Val	Pro	Gln	Thr

For MASCOT search m/z of the unmodified peptide $[M+H]^+$ has to be given

known O-glycosylation site

Alpha-2-HS-glycoprotein precursor

$_{252}$TQPV**T**SQPQPE$_{262}$

Fraction 17

623.23+++ → Pep [M+2H]++ 606.25++ [13.9-14.0 min]

CID-MS2 MASCOT Search

prot_hit_nur	prot_acc	prot_desc	prot_score	prot_mass	prot_matche	prot_matche	prot_sequen	prot_sequen	pep_query	pep_rank	pep_isbold	pep_isuniqu	pep_exp_mz	pep_exp_mr	pep_exp_z	pep_calc_mr	pep_delta	pep_miss	pep_score	pep_expect	pep_res_bef	pep_seq
1	FETUA_HUM	Alpha-2-HS-g	5	40098	1	0	1	0	1	1	1	1	1211.54	1210.5327	1	1210.583	-0.0503	0	9.46	2.40E+03	Q	TQPVTSQPQ
2	SPA9_HUMA	Serpin A9 OS	3	46698	1	0	1	0	1	2	0	1	1211.54	1210.5327	1	1210.5573	-0.0246	0	8.54	2.90E+03	F	LMMITNKATI
3	PTHD1_HUMAN		2	102531	1	0	1	0	1	4	0	1	1211.54	1210.5327	1	1210.671	-0.1383	0	7.99	3.30E+03	D	VLEYTKGFVR
4	COPB_HUMAN		1	108214	1	0	1	0	1	5	0	1	1211.54	1210.5327	1	1209.5513	0.9814	0	7.67	3.60E+03	G	NIVYDVSGA
5	CLASR_HUMAN		1	77286	1	0	1	0	1	8	0	1	1211.54	1210.5327	1	1209.4795	1.0533	0	6.45	4.70E+03	P	VNMMPWQ
6	PCF11_HUMAN		0	173629	1	0	1	0	1	7	0	1	1211.54	1210.5327	1	1209.6142	0.9185	0	7.63	3.60E+03	L	RFEGPPGPV
7	FRYL_HUMAN		0	342177	1	0	1	0	1	2	0	1	1211.54	1210.5327	1	1209.5295	1.0032	0	8.54	2.90E+03	L	SRTQMLNSD
8	MLXPL_HUMAN		0	93698	1	0	1	0	1	10	0	1	1211.54	1210.5327	1	1210.6445	-0.1118	0	5.39	6.00E+03	F	SSVVPVLLGD
9	TRM6_HUMAN		0	56049	1	0	1	0	1	9	0	1	1211.54	1210.5327	1	1210.5102	0.0225	0	5.41	6.00E+03	M	EGSGEQPGP

BioTools-Score: 93

MASCOT-Score: 9

known O-glycosylation site

Alpha-2-HS-glycoprotein precursor

$_{252}$TQPV**T**SQPQPE$_{262}$

Fraction 17

623.23+++ → Pep [M+2H]++ 606.25++ [13.9-14.0 min]

CID-MS2

Internal glycopeptide fragmentation

$_{252}$TQPV**T**SQPQPE$_{262}$
(T: HexNac-)

b				y
---	1	**T**	11	---
230.11	2	**Q**	10	1313.62
327.17	3	**P**	9	1185.56
426.23	4	**V**	8	1088.51
730.36	5	**T(HexNAc)**	7	989.44
817.39	6	**S**	6	685.32
945.45	7	**Q**	5	598.28
1042.51	8	**P**	4	470.22
1170.56	9	**Q**	3	373.17
1267.62	10	**P**	2	245.11
---	11	**E**	1	148.06

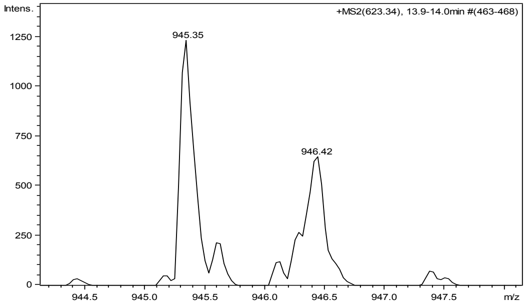

TQPV**T**SQ
(T: HexNac-)

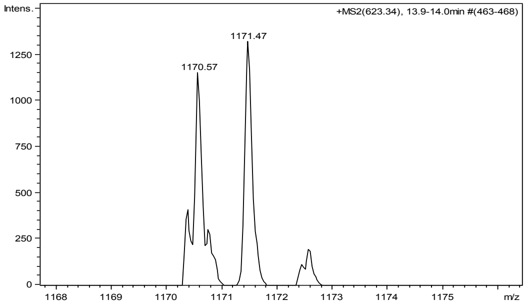

TQPV**T**SQPQ
(T: HexNac-)

These fragment ions are not present in the MS³ peptide spectrum. This indicates that they are derived from glycopeptide fragmentation.

Fraction 17

623.23+++ → Pep [M+2H]++ 606.25++ [13.9-14.0 min] CID-MS3

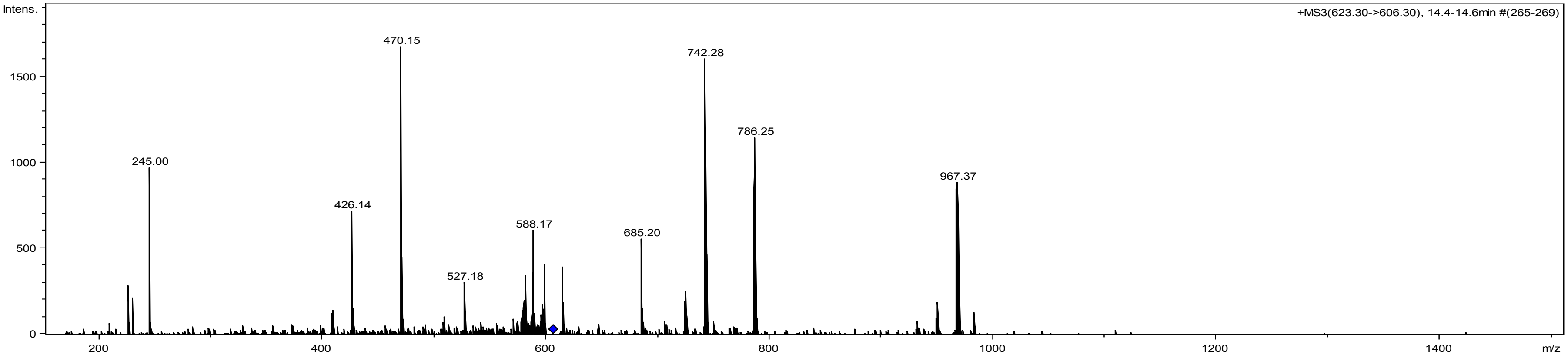

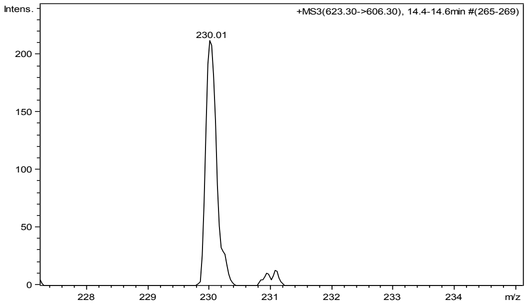

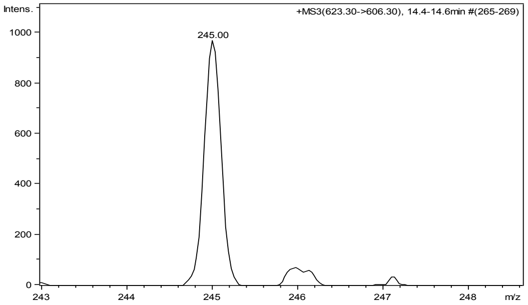

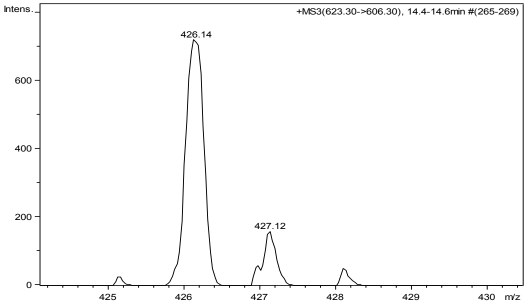

known O-glycosylation site

Alpha-2-HS-glycoprotein precursor $_{252}$TQPV**T**SQPQPE$_{262}$

Fraction 17

623.23+++ → Pep [M+2H]++ 606.25++ [13.9-14.0 min]

CID-MS3

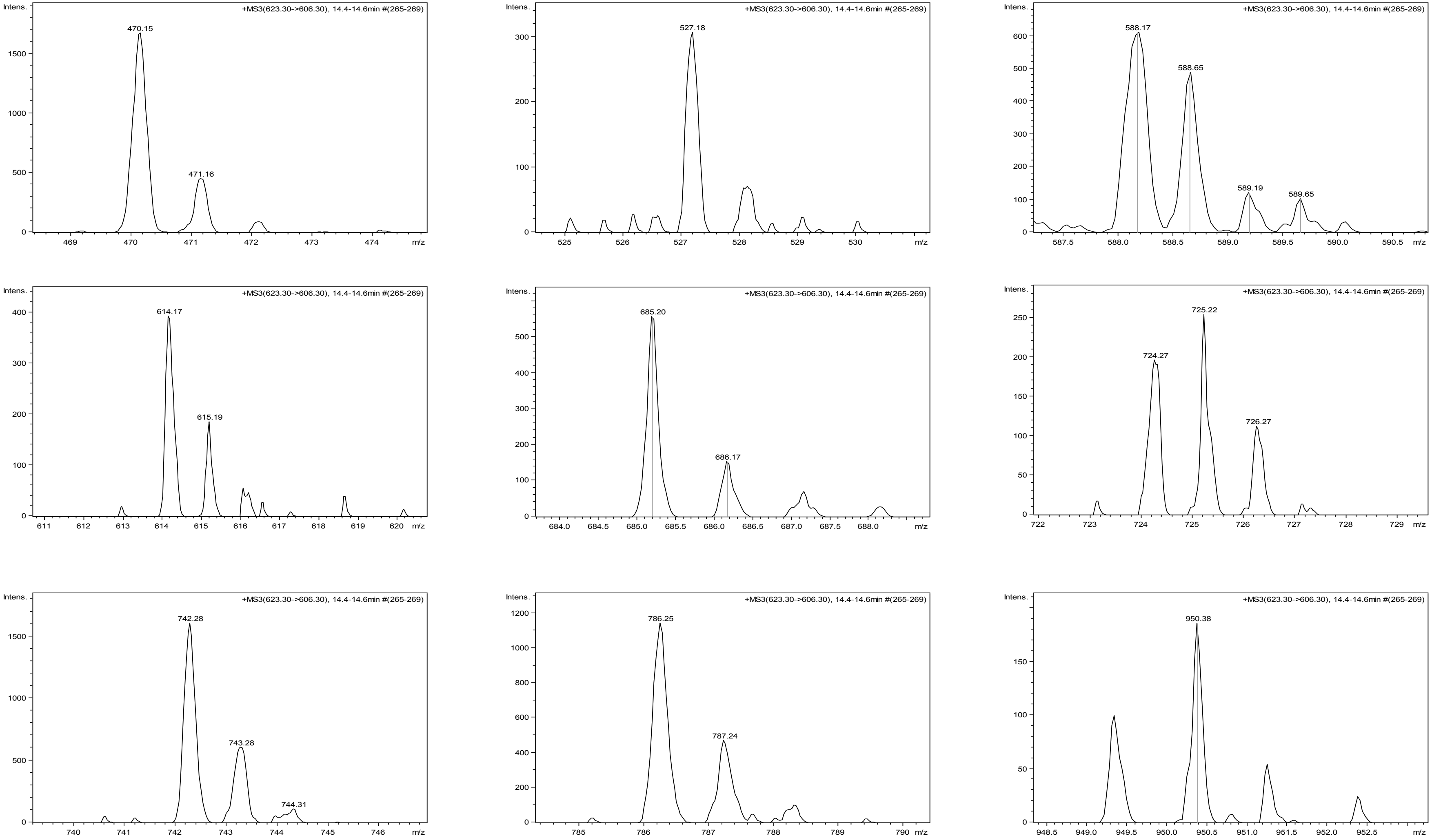

known O-glycosylation site

Alpha-2-HS-glycoprotein precursor

$_{252}$TQPV**T**SQPQPE$_{262}$

Fraction 17

623.23+++ → Pep [M+2H]++ 606.25++ [13.9-14.0 min] CID-MS3

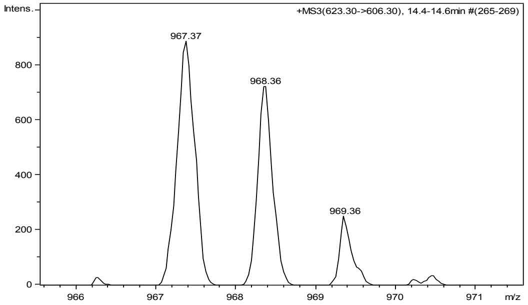

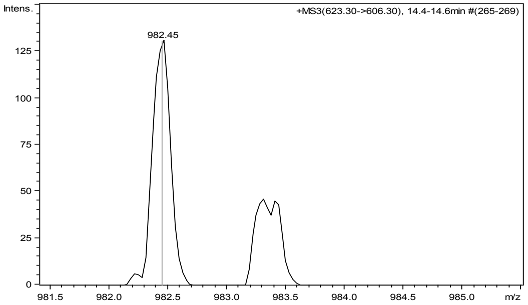

known O-glycosylation site

Alpha-2-HS-glycoprotein precursor

$_{252}$TQPV**T**SQPQPE$_{262}$

Fraction 17

623.23+++ → Pep [M+2H]++ 606.25++ [13.9-14.0 min]

CID-MS3 MASCOT Search

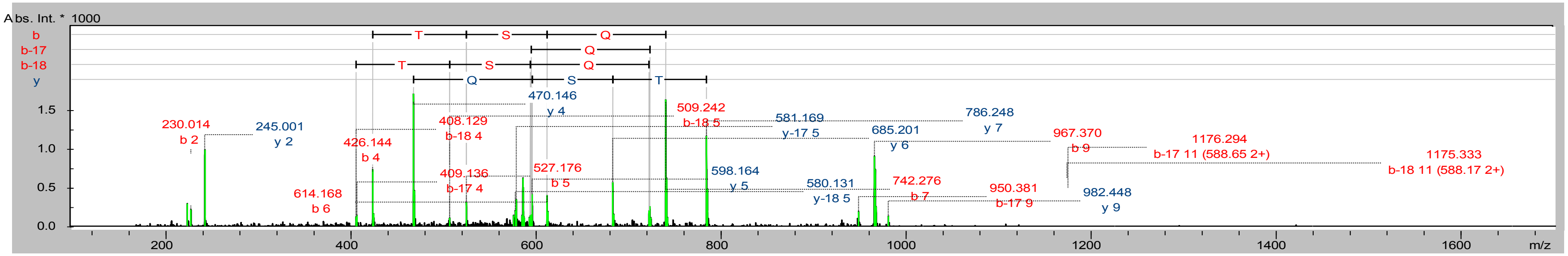

	T	Q	P	V	T	S	Q	P	Q	P	E	Thr	Gln	Pro	Val	Thr	Ser	Gln	Pro	Gln	Pro	Glu
Ion	1	2	3	4	5	6	7	8	9	10	11	1	2	3	4	5	6	7	8	9	10	11
b	T	Q	P	V	T	S	Q	P	Q	P	E	102.055	230.114	327.166	426.235	527.282	614.314	742.373	839.426	967.484	1064.537	1193.580
b-17	T	Q	P	V	T	S	Q	P	Q	P	E	-	213.087	310.140	409.208	510.256	597.288	725.346	822.399	950.458	1047.511	1176.553
b-18	T	Q	P	V	T	S	Q	P	Q	P	E	84.044	212.103	309.156	408.224	509.272	596.304	724.362	821.415	949.474	1046.527	1175.569
y	T	Q	P	V	T	S	Q	P	Q	P	E	148.060	245.113	373.172	470.225	598.283	685.315	786.363	885.431	982.484	1110.543	1211.590
y-17	T	Q	P	V	T	S	Q	P	Q	P	E	-	-	356.145	453.198	581.257	668.289	769.336	868.405	965.457	1093.516	1194.564
y-18	T	Q	P	V	T	S	Q	P	Q	P	E	130.050	227.103	355.161	452.214	580.273	667.305	768.352	867.421	964.473	1092.532	1193.580
	11	10	9	8	7	6	5	4	3	2	1	Glu	Pro	Gln	Pro	Gln	Ser	Thr	Val	Pro	Gln	Thr

known O-glycosylation site

Alpha-2-HS-glycoprotein precursor

$_{252}$TQPV**T**SQPQPE$_{262}$

Fraction 17

623.23+++ → Pep [M+2H]++ 606.25++ [13.9-14.0 min]

CID-MS3 MASCOT Search

prot_hit_nur	prot_acc	prot_desc	prot_score	prot_mass	prot_matche	pep_query	pep_rank	pep_isbold	pep_exp_mz	pep_exp_mr	pep_exp_z	pep_calc_mr	pep_delta	pep_miss	pep_score	pep_expect	pep_res_bef	pep_seq
1	FETUA_HUM	Alpha-2-HS-g	37	40098	1	1	1	1	606.2462	1210.4778	2	1210.583	-0.1051	0	41.61	1.1	Q	TQPVTSQPQ
2	HNRU2_HUM	Heterogene	26	24411	1	1	7	0	606.2462	1210.4778	2	1210.5578	-0.08	0	28.74	21	Q	GAAAAAGSG
3	IRTF_HUMAN	Transcription	26	44125	1	1	2	0	606.2462	1210.4778	2	1210.6234	-0.1455	0	31.57	11	L	FPKPGPLEPT
4	NETO1_HUM	Neuropilin a	25	61377	1	1	2	0	606.2462	1210.4778	2	1210.6267	-0.1489	0	31.57	11	T	EMPTQPGKP
5	TRM6_HUMA	tRNA (adeni	25	56049	1	1	5	0	606.2462	1210.4778	2	1210.5102	-0.0324	0	30.82	13	M	EGSGEQPGP
6	LR37A_HUM	Leucine-rich	24	181649	1	1	2	0	606.2462	1210.4778	2	1210.5466	-0.0688	0	31.57	11	P	TQQETPGQP
7	CO3A1_HUM	Collagen alp	22	139733	1	1	6	0	606.2462	1210.4778	2	1210.5401	-0.0622	0	30.15	15	G	ENGAPGPM
8	PANK2_HUM	Pantothenat	20	63268	1	1	8	0	606.2462	1210.4778	2	1210.569	-0.0912	0	25.85	41	A	AGDPEGRRQ
9	ACSL3_HUM	Long-chain-f	19	81338	1	1	8	0	606.2462	1210.4778	2	1210.751	-0.2731	0	25.85	41	V	RSLLGGNIRL
10	IF4G3_HUMA	Eukaryotic tr	18	177682	1	1	8	0	606.2462	1210.4778	2	1210.6167	-0.1388	0	25.85	41	R	RSQPGQRRE

BioTools-Score: 46

MASCOT-Score: 42

known O-glycosylation site

Alpha-2-HS-glycoprotein precursor

$_{252}$TQPV**T**SQPQPE$_{262}$

Fraction 17

623.23+++ → Pep [M+2H]++ 606.25++ [13.9-14.0 min] ETD

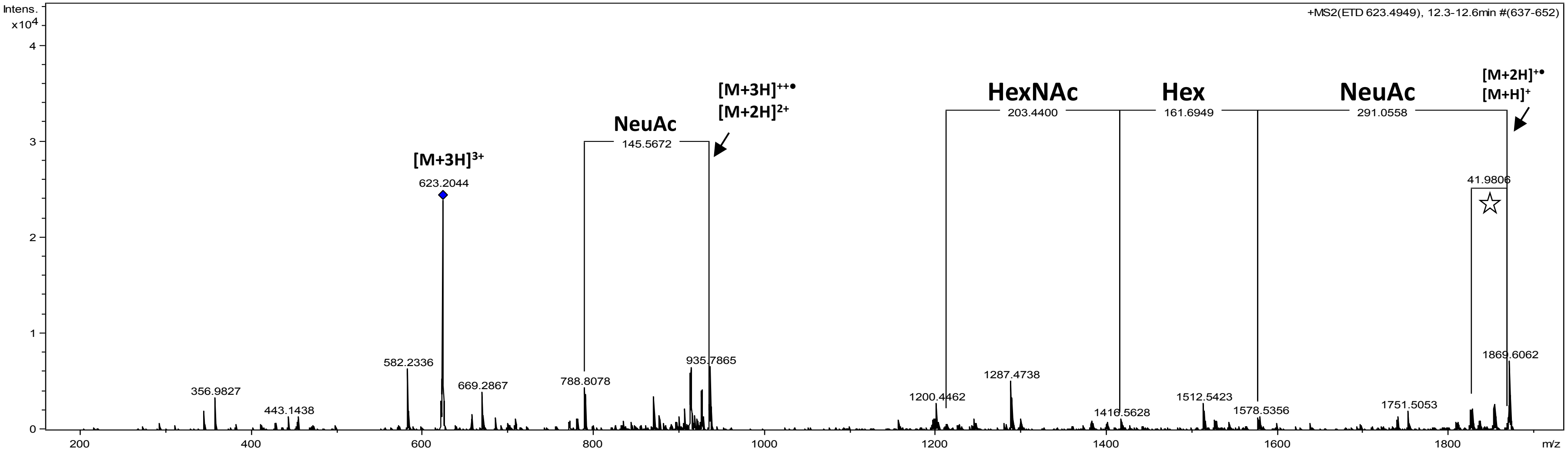

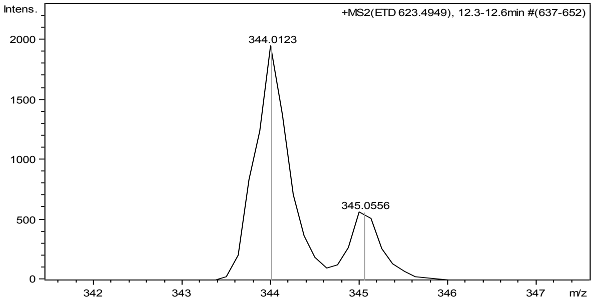

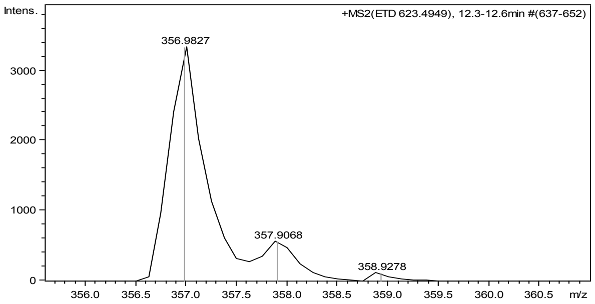

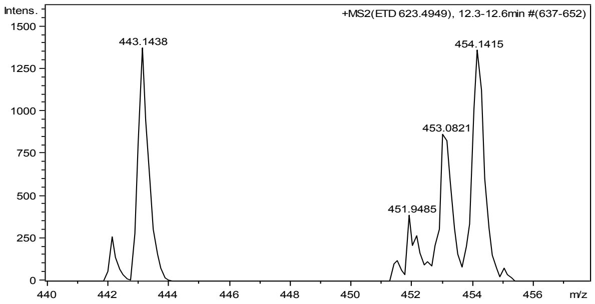

known O-glycosylation site

Alpha-2-HS-glycoprotein precursor

$_{252}$TQPV**T**SQPQPE$_{262}$

Fraction 17

623.23+++ → Pep [M+2H]++ 606.25++ [13.9-14.0 min] ETD

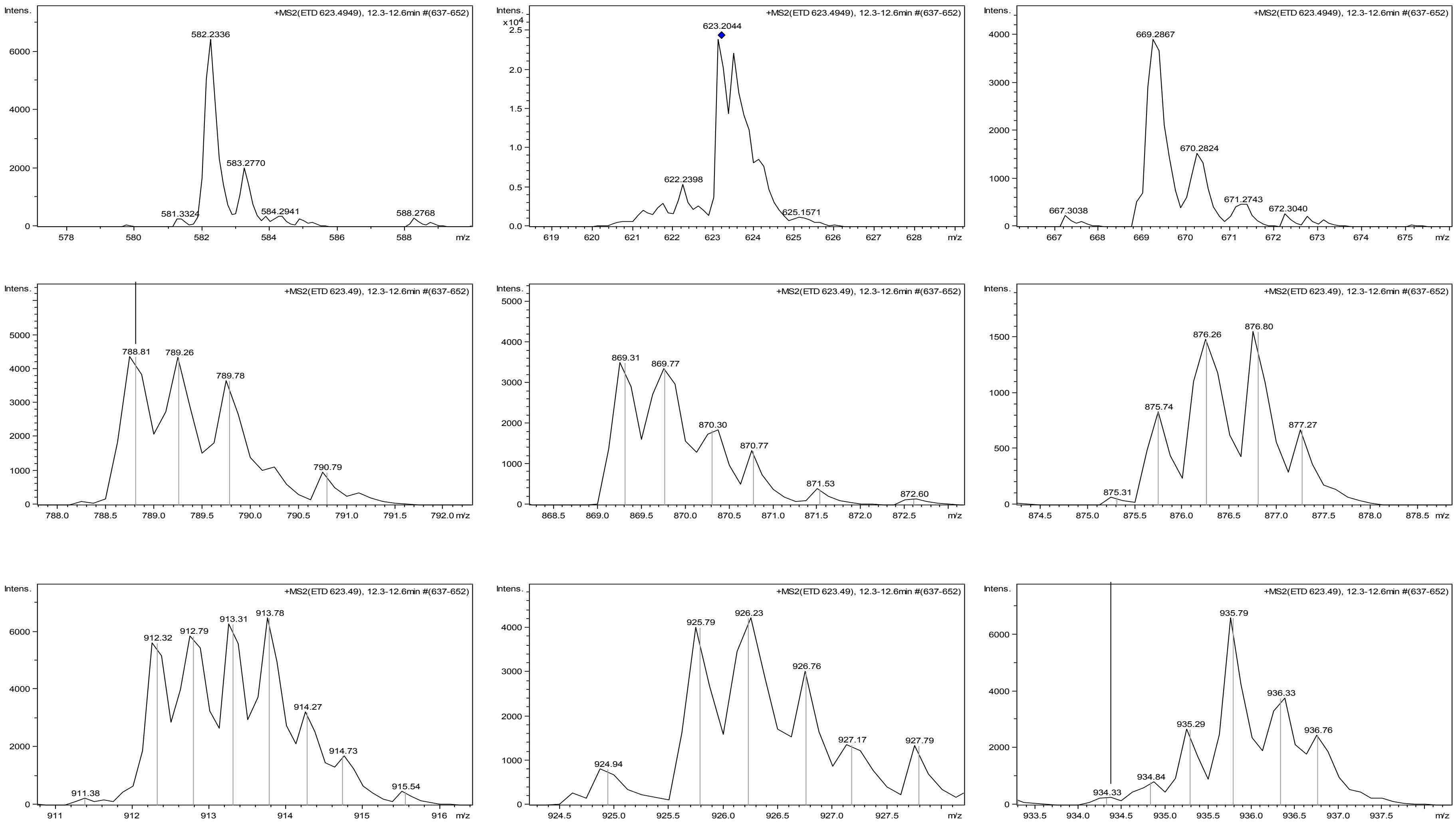

known O-glycosylation site

Alpha-2-HS-glycoprotein precursor

$_{252}$TQPV**T**SQPQPE$_{262}$

Fraction 17

623.23+++ → Pep [M+2H]++ 606.25++ [13.9-14.0 min]

ETD

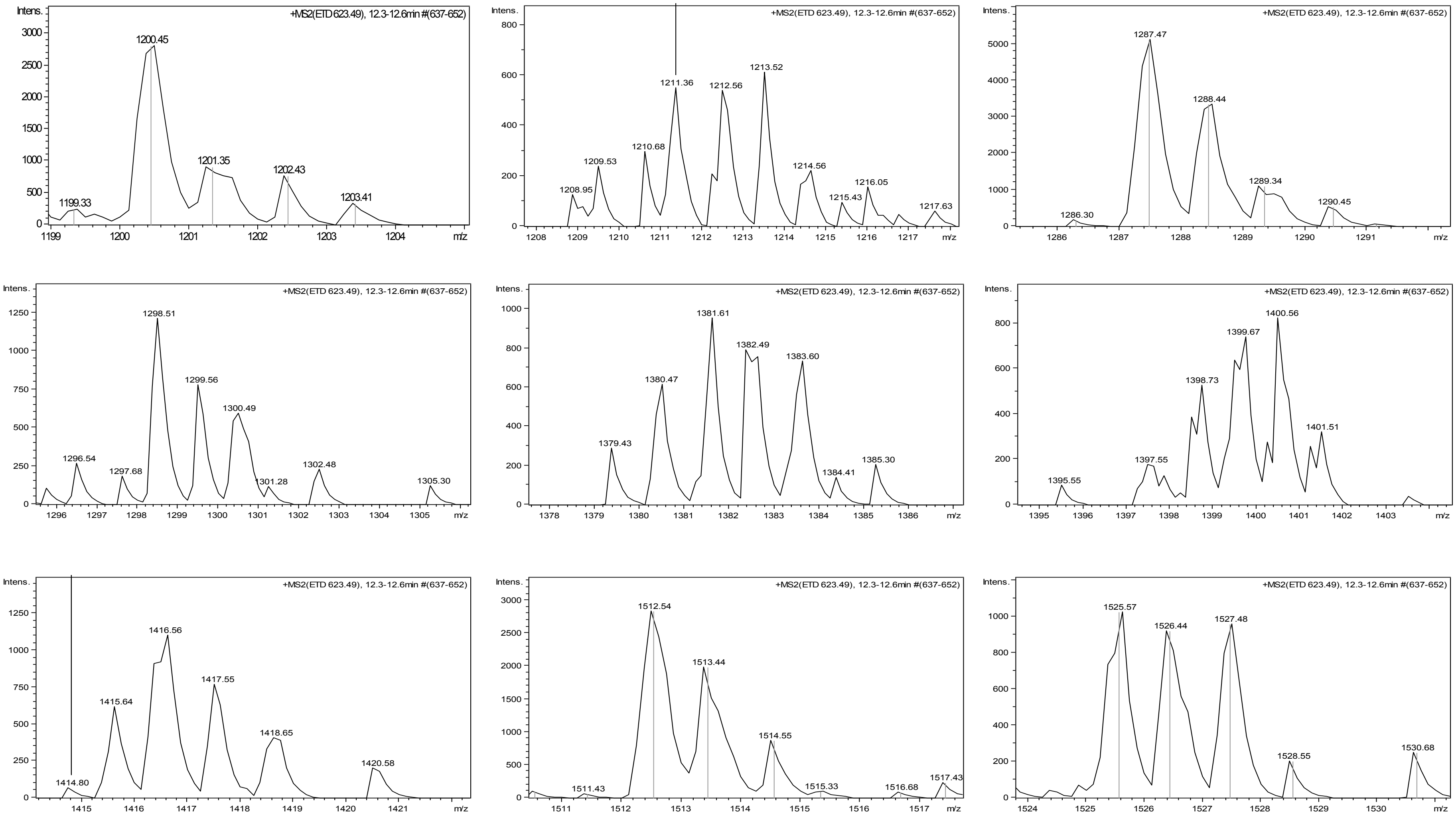

known O-glycosylation site

$_{252}$TQPV**T**SQPQPE$_{262}$

Alpha-2-HS-glycoprotein precursor

Fraction 17

623.23+++ → Pep [M+2H]++ 606.25++ [13.9-14.0 min] ETD

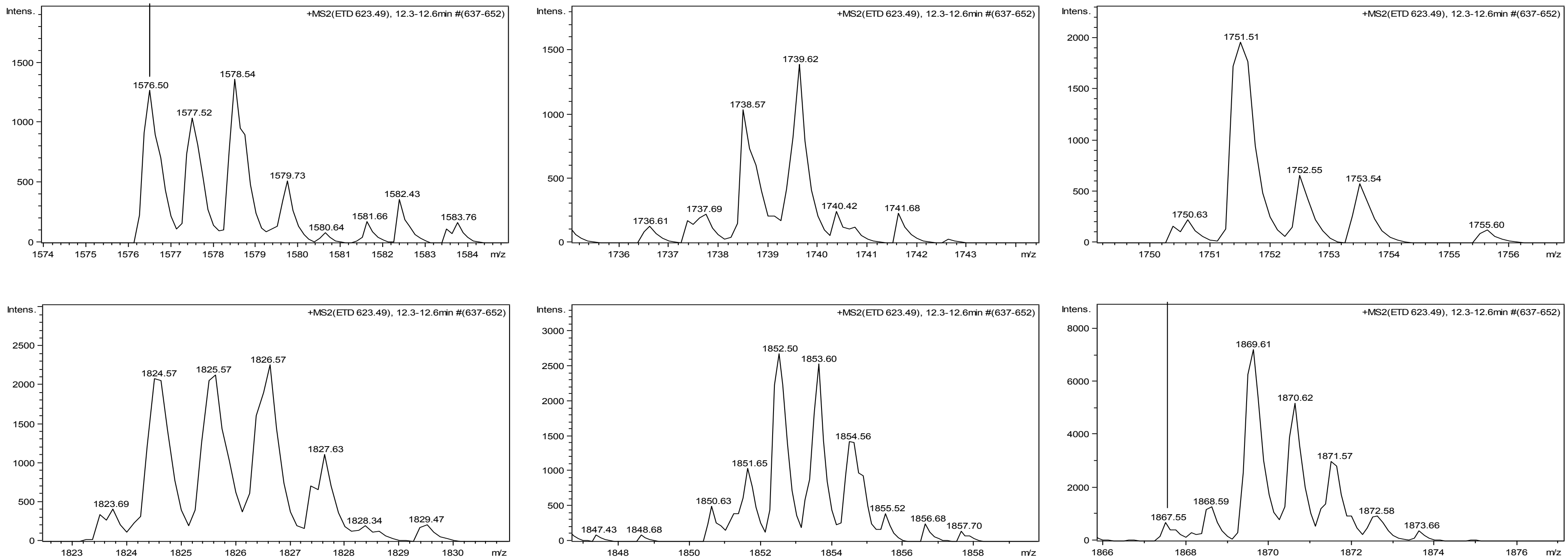

known O-glycosylation site

Alpha-2-HS-glycoprotein precursor

$_{252}$TQPV**T**SQPQPE$_{262}$

Fraction 17

623.23+++ → Pep [M+2H]++ 606.25++ [13.9-14.0 min] ETD

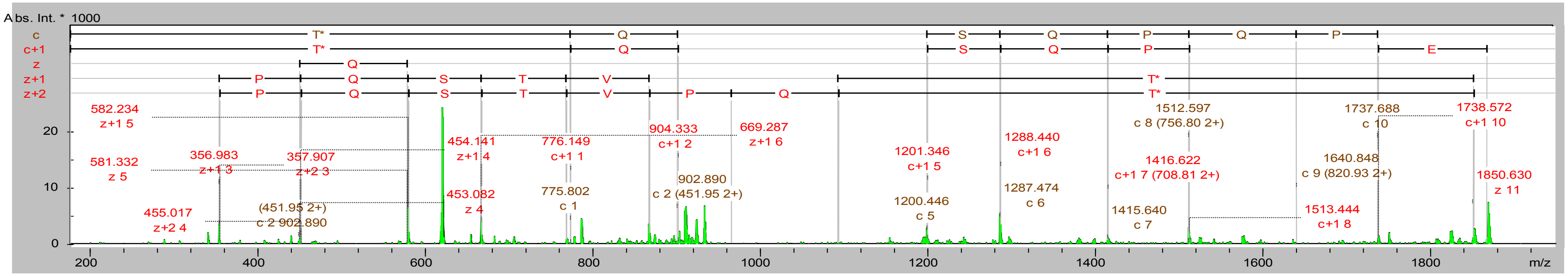

TQPVTSQPQPE

	T	Q	P	V	T	S	Q	P	Q	P	E	Thr	Gln	Pro	Val	Thr	Ser	Gln	Pro	Gln	Pro	Glu
Ion	1	2	3	4	5	6	7	8	9	10	11	1	2	3	4	5	6	7	8	9	10	11
c	T*	Q	P	V	T	S	Q	P	Q	P	E	775.309	903.368	1000.420	1099.489	1200.537	1287.569	1415.627	1512.680	1640.738	1737.791	1866.834
c+1	T*	Q	P	V	T	S	Q	P	Q	P	E	776.317	904.376	1001.428	1100.497	1201.544	1288.576	1416.635	1513.688	1641.746	1738.799	1867.842
z	T*	Q	P	V	T	S	Q	P	Q	P	E	131.034	228.087	356.145	453.198	581.257	668.289	769.336	868.405	965.457	1093.516	1850.791
z+1	T*	Q	P	V	T	S	Q	P	Q	P	E	132.042	229.094	357.153	454.206	582.264	669.296	770.344	869.413	966.465	1094.524	1851.799
z+2	T*	Q	P	V	T	S	Q	P	Q	P	E	133.050	230.102	358.161	455.214	583.272	670.304	771.352	870.420	967.473	1095.532	1852.807
	11	10	9	8	7	6	5	4	3	2	1	Glu	Pro	Gln	Pro	Gln	Ser	Thr	Val	Pro	Gln	Thr

BioTools-Score: 669

known O-glycosylation site

Alpha-2-HS-glycoprotein precursor

$_{252}$TQPVTSQPQPE$_{262}$

Fraction 17

623.23+++ → Pep [M+2H]++ 606.25++ [13.9-14.0 min] ETD

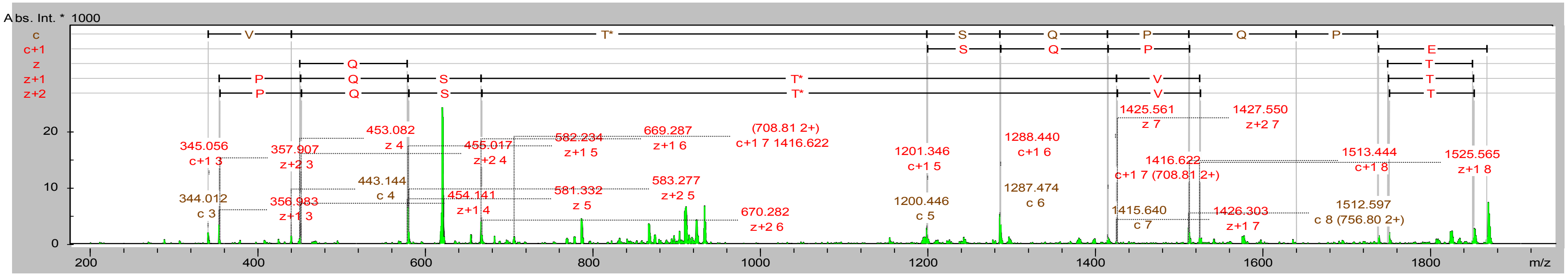

TQPVTSQPQPE

	T	Q	P	V	T	S	Q	P	Q	P	E	Thr	Gln	Pro	Val	Thr	Ser	Gln	Pro	Gln	Pro	Glu
Ion	1	2	3	4	5	6	7	8	9	10	11	1	2	3	4	5	6	7	8	9	10	11
c	T	Q	P	V	T*	S	Q	P	Q	P	E	119.082	247.140	344.193	443.261	1200.537	1287.569	1415.627	1512.680	1640.738	1737.791	1866.834
c+1	T	Q	P	V	T*	S	Q	P	Q	P	E	120.089	248.148	345.201	444.269	1201.544	1288.576	1416.635	1513.688	1641.746	1738.799	1867.842
z	T	Q	P	V	T*	S	Q	P	Q	P	E	131.034	228.087	356.145	453.198	581.257	668.289	1425.564	1524.632	1621.685	1749.744	1850.791
z+1	T	Q	P	V	T*	S	Q	P	Q	P	E	132.042	229.094	357.153	454.206	582.264	669.296	1426.572	1525.640	1622.693	1750.751	1851.799
z+2	T	Q	P	V	T*	S	Q	P	Q	P	E	133.050	230.102	358.161	455.214	583.272	670.304	1427.580	1526.648	1623.701	1751.759	1852.807
	11	10	9	8	7	6	5	4	3	2	1	Glu	Pro	Gln	Pro	Gln	Ser	Thr	Val	Pro	Gln	Thr

BioTools-Score: 412

Although the BioTools-Score is lower, this annotation is more reasonable since most of the major peaks were allocated to glycopeptide fragment ions.

known O-glycosylation site

Alpha-2-HS-glycoprotein precursor $_{252}$TQPVTSQPQPE$_{262}$

Fraction 17

623.23+++ → Pep [M+2H]++ 606.25++ [13.9-14.0 min] ETD

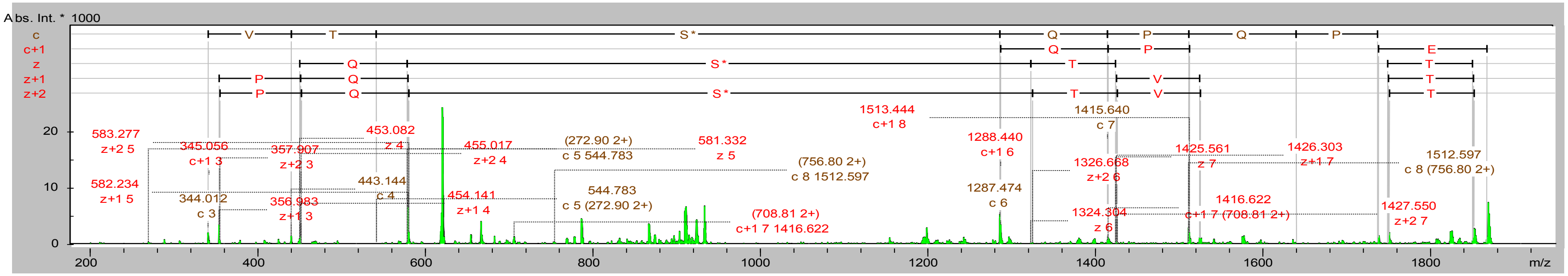

TQPVT**S**QPQPE

	T	Q	P	V	T	S	Q	P	Q	P	E	Thr	Gln	Pro	Val	Thr	Ser	Gln	Pro	Gln	Pro	Glu
Ion	1	2	3	4	5	6	7	8	9	10	11	1	2	3	4	5	6	7	8	9	10	11
c	T	Q	P	V	T	S*	Q	P	Q	P	E	119.082	247.140	344.193	443.261	544.309	1287.569	1415.627	1512.680	1640.738	1737.791	1866.834
c+1	T	Q	P	V	T	S*	Q	P	Q	P	E	120.089	248.148	345.201	444.269	545.317	1288.576	1416.635	1513.688	1641.746	1738.799	1867.842
z	T	Q	P	V	T	S*	Q	P	Q	P	E	131.034	228.087	356.145	453.198	581.257	1324.516	1425.564	1524.632	1621.685	1749.744	1850.791
z+1	T	Q	P	V	T	S*	Q	P	Q	P	E	132.042	229.094	357.153	454.206	582.264	1325.524	1426.572	1525.640	1622.693	1750.751	1851.799
z+2	T	Q	P	V	T	S*	Q	P	Q	P	E	133.050	230.102	358.161	455.214	583.272	1326.532	1427.580	1526.648	1623.701	1751.759	1852.807
	11	10	9	8	7	6	5	4	3	2	1	Glu	Pro	Gln	Pro	Gln	Ser	Thr	Val	Pro	Gln	Thr

BioTools-Score: 362

known O-glycosylation site

Alpha-2-HS-glycoprotein precursor

$_{252}$TQPV**T**SQPQPE$_{262}$

Fraction 17

934.36++ → Pep [M+H]+ 1211.55+ [14.1-14.3 min] CID-MS Precursor

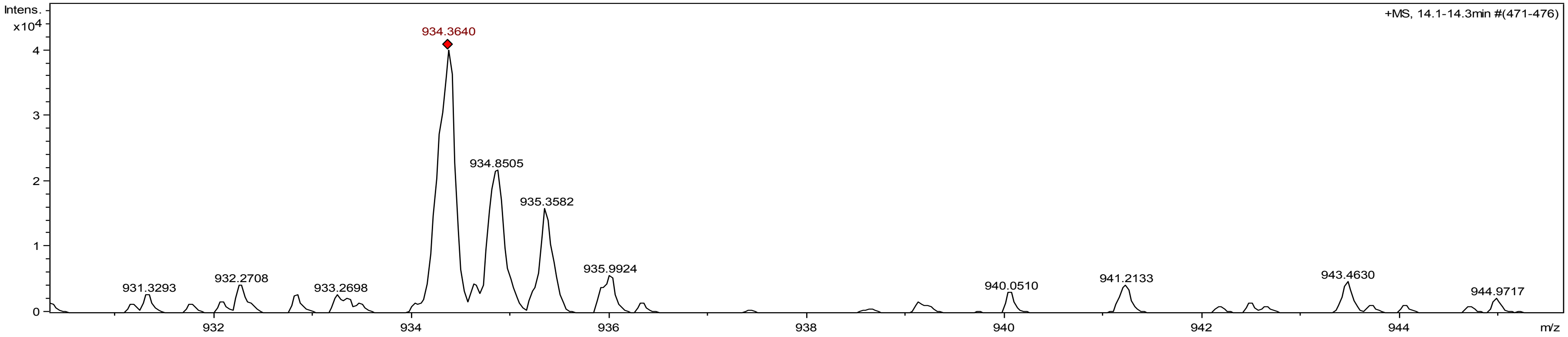

Fraction 17

934.36++ → Pep [M+H]+ 1211.55+ [14.1-14.3 min] CID-MS2

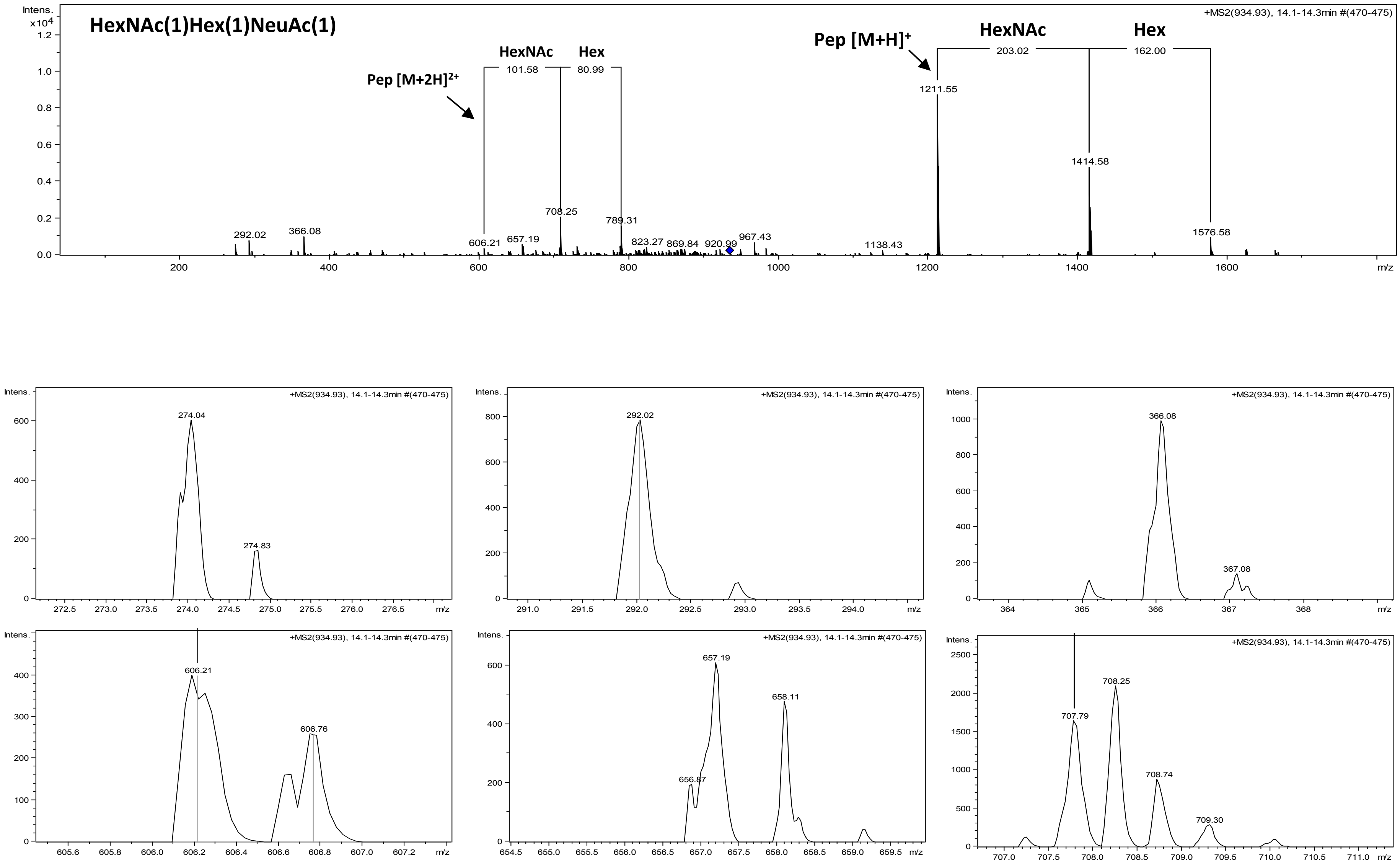

Fraction 17

934.36++ → Pep [M+H]+ 1211.55+ [14.1-14.3 min] CID-MS2

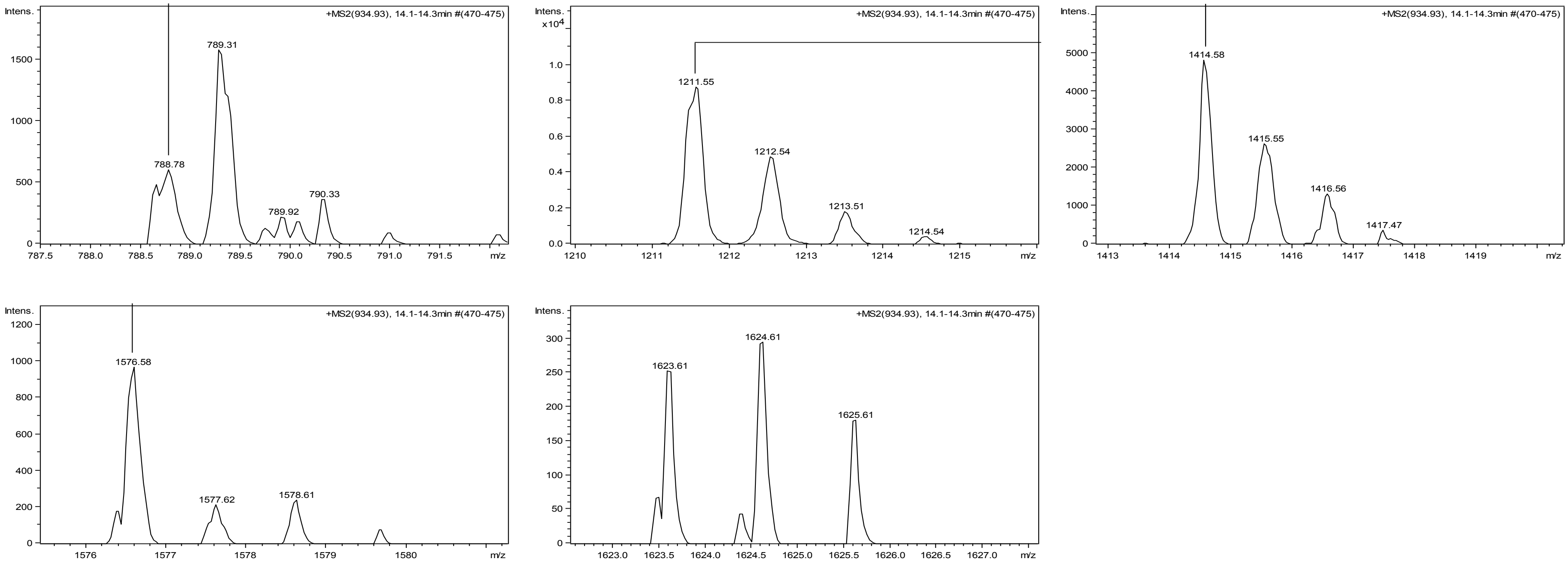

Fraction 17

934.36++ → Pep [M+H]+ 1211.55+ [14.1-14.3 min] CID-MS3

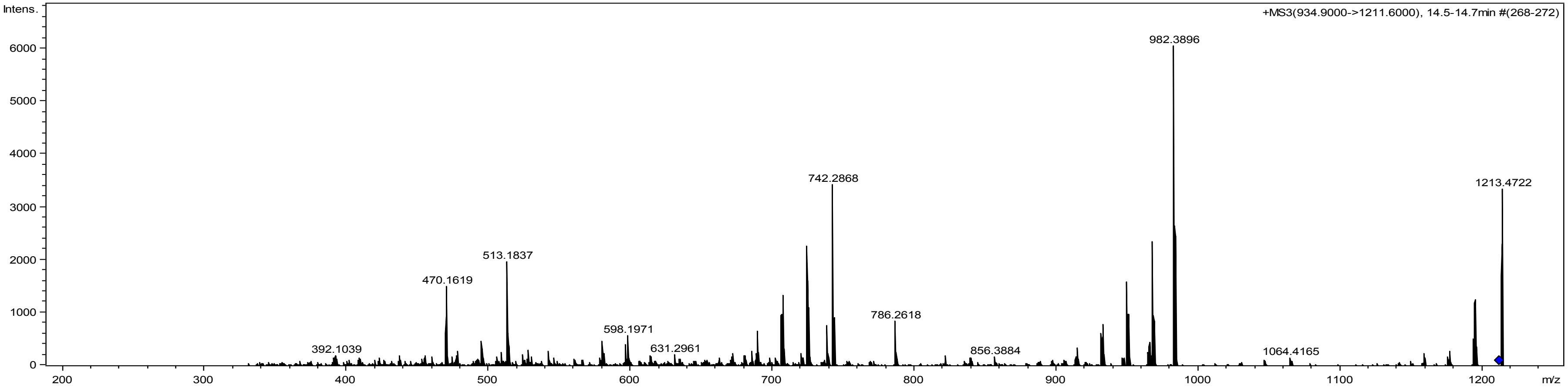

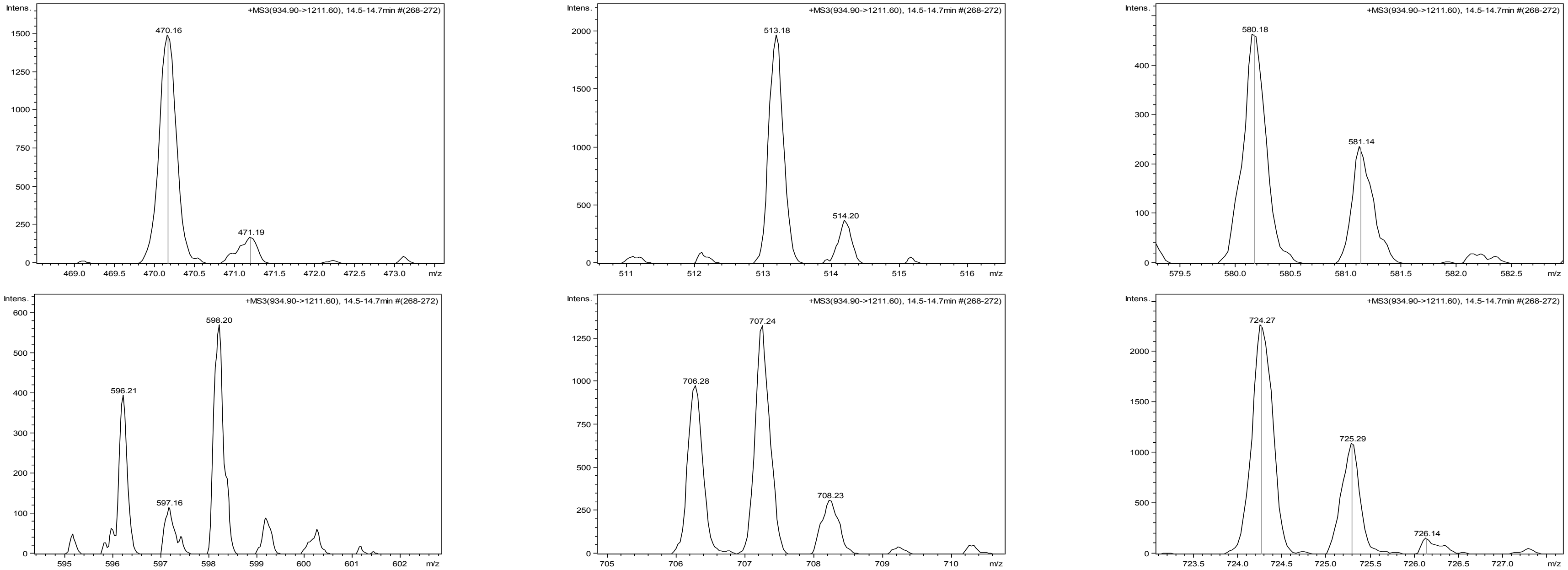

Fraction 17

934.36++ → Pep [M+H]+ 1211.55+ [14.1-14.3 min]

CID-MS3

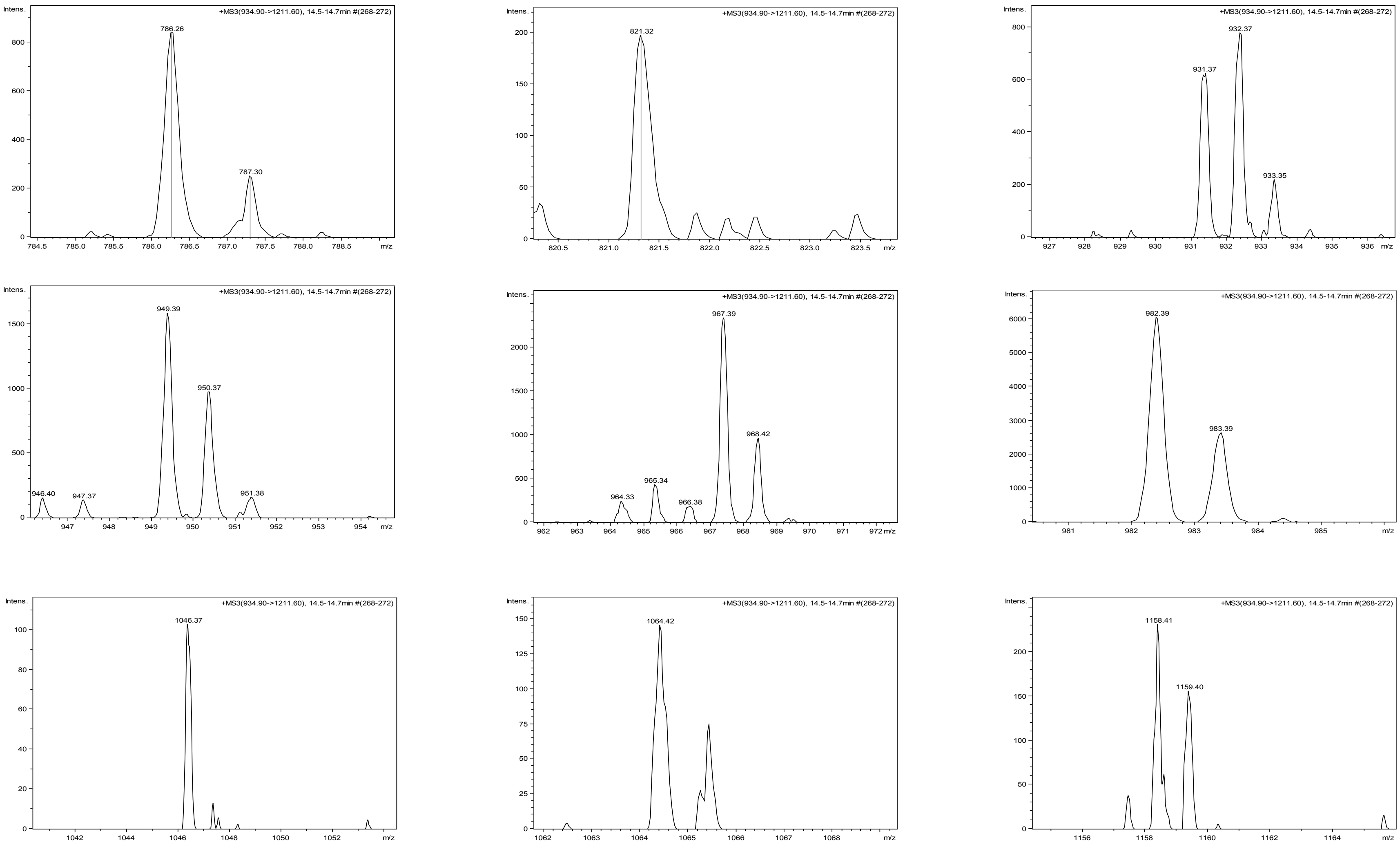

Fraction 17

934.36++ → Pep [M+H]+ 1211.55+ [14.1-14.3 min] CID-MS3

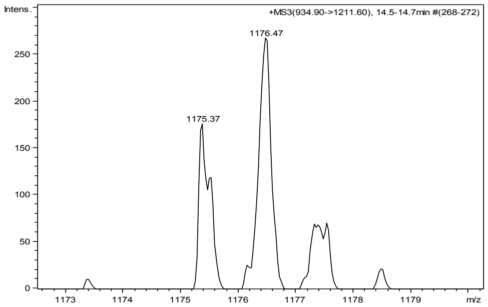

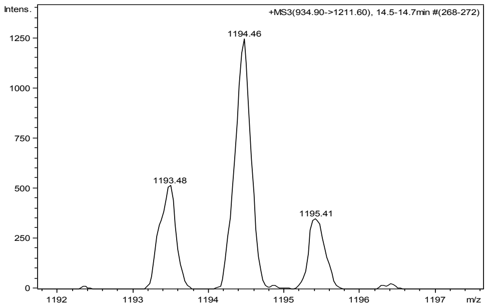

Fraction 17

934.36++ → Pep [M+H]+ 1211.55+ [14.1-14.3 min] CID-MS3 MASCOT Search

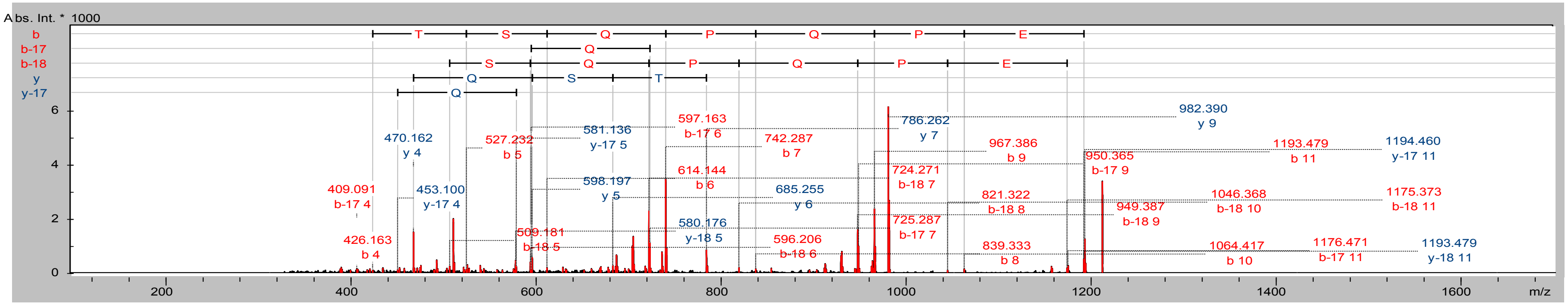

	T	Q	P	V	T	S	Q	P	Q	P	E	Thr	Gln	Pro	Val	Thr	Ser	Gln	Pro	Gln	Pro	Glu
Ion	1	2	3	4	5	6	7	8	9	10	11	1	2	3	4	5	6	7	8	9	10	11
b	T	Q	P	V	T	S	Q	P	Q	P	E	102.055	230.114	327.166	426.235	527.282	614.314	742.373	839.426	967.484	1064.537	1193.580
b-17	T	Q	P	V	T	S	Q	P	Q	P	E	-	213.087	310.140	409.208	510.256	597.288	725.346	822.399	950.458	1047.511	1176.553
b-18	T	Q	P	V	T	S	Q	P	Q	P	E	84.044	212.103	309.156	408.224	509.272	596.304	724.362	821.415	949.474	1046.527	1175.569
y	T	Q	P	V	T	S	Q	P	Q	P	E	148.060	245.113	373.172	470.225	598.283	685.315	786.363	885.431	982.484	1110.543	1211.590
y-17	T	Q	P	V	T	S	Q	P	Q	P	E	-	-	356.145	453.198	581.257	668.289	769.336	868.405	965.457	1093.516	1194.564
y-18	T	Q	P	V	T	S	Q	P	Q	P	E	130.050	227.103	355.161	452.214	580.273	667.305	768.352	867.421	964.473	1092.532	1193.580
	11	10	9	8	7	6	5	4	3	2	1	Glu	Pro	Gln	Pro	Gln	Ser	Thr	Val	Pro	Gln	Thr

known O-glycosylation site

Alpha-2-HS-glycoprotein precursor

$_{252}$TQPV**T**SQPQPE$_{262}$

Fraction 17

934.36++ → Pep [M+H]+ 1211.55+ [14.1-14.3 min]   CID-MS3 MASCOT Search

prot_hit_nur	prot_acc	prot_desc	prot_score	prot_mass	prot_matche	pep_query	pep_rank	pep_isbold	pep_exp_mz	pep_exp_mr	pep_exp_z	pep_calc_mr	pep_delta	pep_miss	pep_score	pep_expect	pep_res_bef	pep_seq
1	FETUA_HUM	Alpha-2-HS-g	23	40098	1	1	1	1	1211.5543	1210.547	1	1210.583	-0.036	0	28.32	24	Q	TQPVTSQPQ
2	ZYX_HUMAN	Zyxin (Zyxin-	17	62436	1	1	2	0	1211.5543	1210.547	1	1210.5619	-0.0148	0	23.71	68	Q	TQFHVQPQP
3	IRTF_HUMAN	Transcription	15	44125	1	1	4	0	1211.5543	1210.547	1	1210.6234	-0.0764	0	20.06	1.60E+02	L	FPKPGPLEPT
4	CU125_HUM	Putative unc	14	17275	1	1	5	0	1211.5543	1210.547	1	1210.6194	-0.0723	0	18.58	2.20E+02	S	LATATVGAG
5	ISCA1_HUM	Iron-sulfur cl	14	14342	1	1	10	0	1211.5543	1210.547	1	1210.7285	-0.1815	0	17.17	3.10E+02	K	IKQLLKDKPE
6	R51A1_HUM	RAD51-assoc	13	38662	1	1	7	0	1211.5543	1210.547	1	1209.7081	0.8389	0	17.84	2.60E+02	A	PKELKQDKPK
7	ZN343_HUM	Zinc finger p	13	71115	1	1	8	0	1211.5543	1210.547	1	1210.6445	-0.0974	0	17.76	2.70E+02	R	NLLSLAEPKP
8	HRX_HUMAN	Zinc finger p	13	436044	1	1	3	0	1211.5543	1210.547	1	1210.5903	-0.0433	0	21.8	1.10E+02	R	NLMLPDGPK
9	XYLT2_HUM	Xylosyltransf	11	97449	1	1	9	0	1211.5543	1210.547	1	1210.6445	-0.0975	0	17.59	2.80E+02	G	LSSILNLPQPE
10	LR37A_HUM	Leucine-rich	11	181649	1	1	6	0	1211.5543	1210.547	1	1210.5466	0.0004	0	18.44	2.30E+02	P	TQQETPGQP

BioTools-Score: 402

MASCOT-Score: 28

known O-glycosylation site

Alpha-2-HS-glycoprotein precursor

$_{252}$TQPV**T**SQPQPE$_{262}$

Fraction 17

934.36++ → Pep [M+H]+ 1211.55+ [14.1-14.3 min] ETD

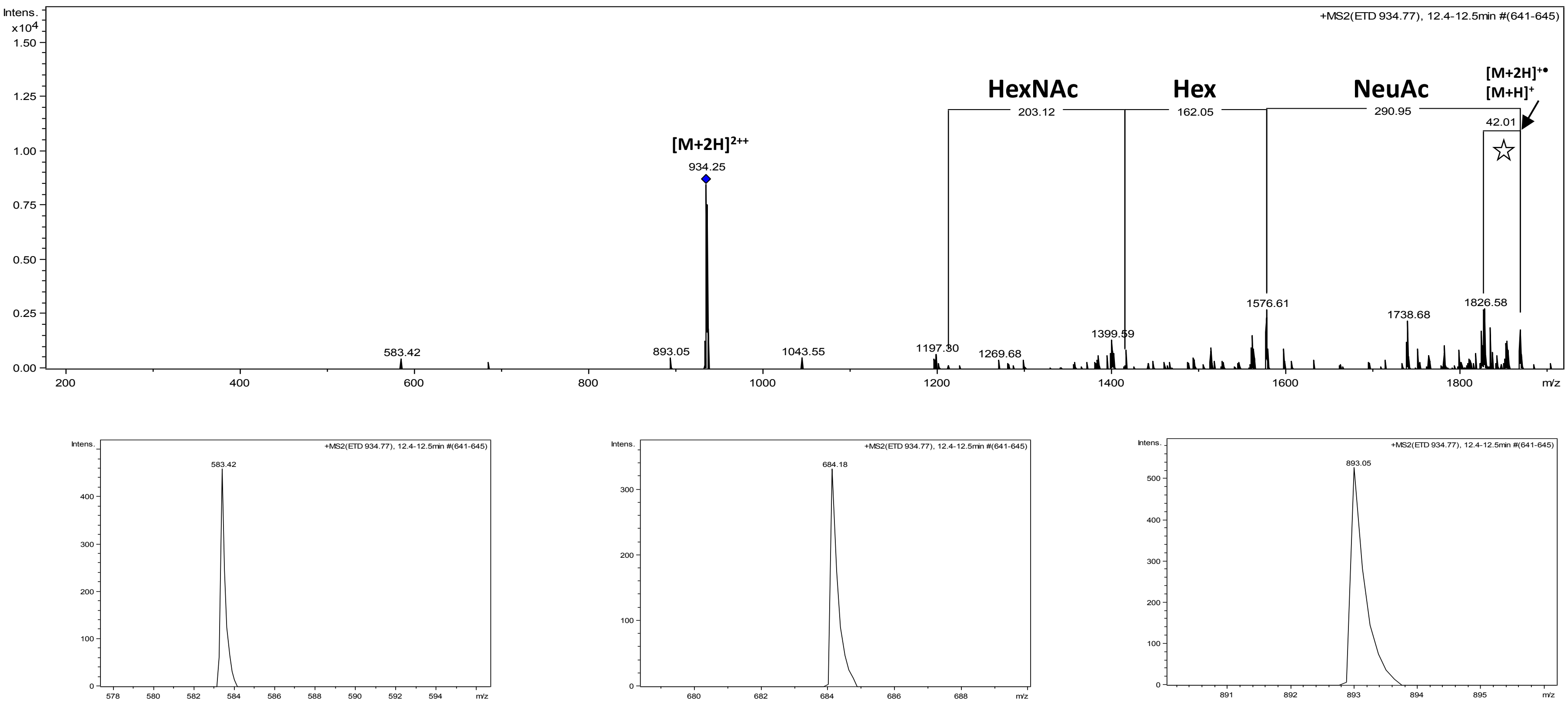

known O-glycosylation site

Alpha-2-HS-glycoprotein precursor

$_{252}$TQPV**T**SQPQPE$_{262}$

Fraction 17

934.36++ → Pep [M+H]+ 1211.55+ [14.1-14.3 min]

ETD

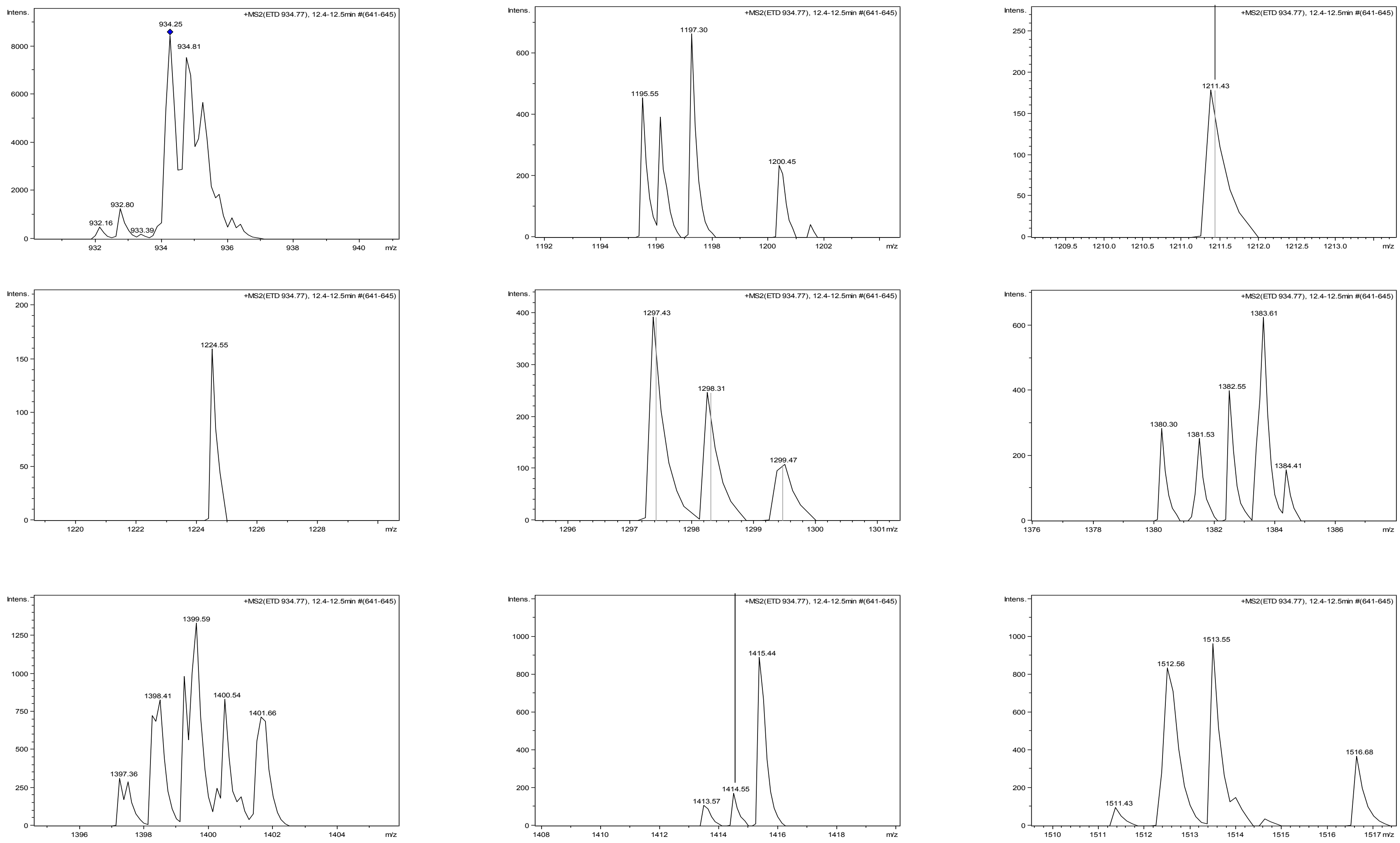

known O-glycosylation site

$_{252}$TQPV**T**SQPQPE$_{262}$

Alpha-2-HS-glycoprotein precursor

Fraction 17

934.36++ → Pep [M+H]+ 1211.55+ [14.1-14.3 min]

ETD

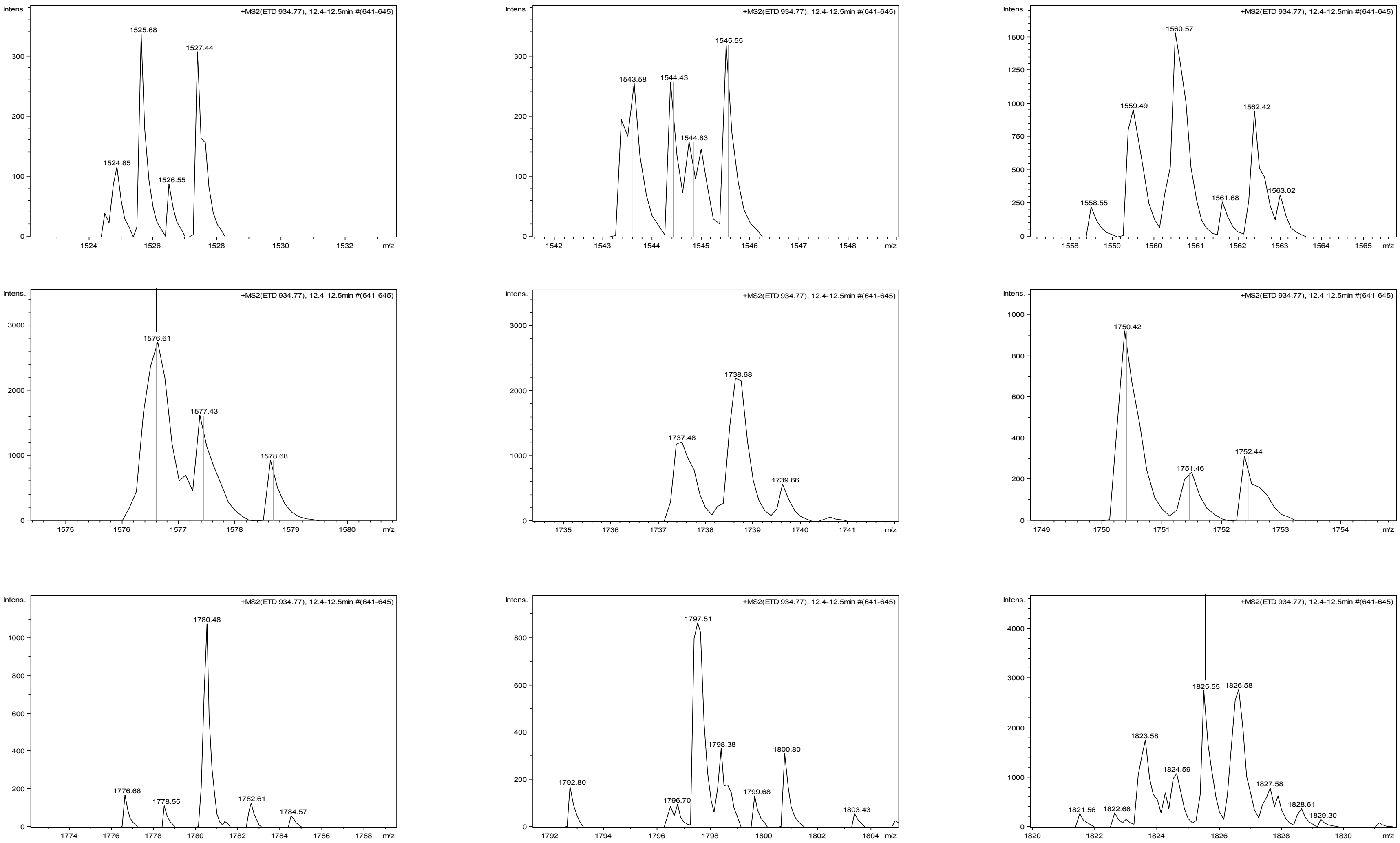

known O-glycosylation site

$_{252}$TQPV**T**SQPQPE$_{262}$

Alpha-2-HS-glycoprotein precursor

Fraction 17

934.36++ → Pep [M+H]+ 1211.55+ [14.1-14.3 min] ETD

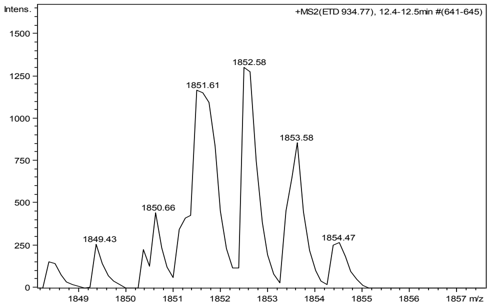

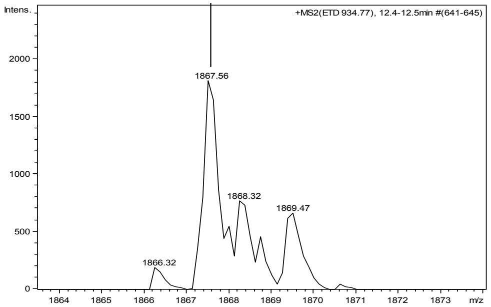

known O-glycosylation site $_{252}$TQPV**T**SQPQPE$_{262}$

Alpha-2-HS-glycoprotein precursor

Fraction 17

934.36++ → Pep [M+H]+ 1211.55+ [14.1-14.3 min] ETD

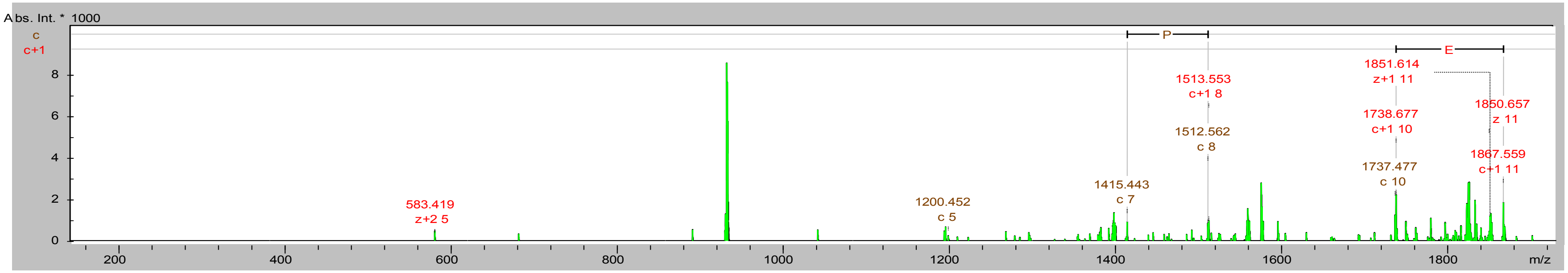

TQPVTSQPQPE

	T	Q	P	V	T	S	Q	P	Q	P	E	Thr	Gln	Pro	Val	Thr	Ser	Gln	Pro	Gln	Pro	Glu
Ion	1	2	3	4	5	6	7	8	9	10	11	1	2	3	4	5	6	7	8	9	10	11
c	T*	Q	P	V	T	S	Q	P	Q	P	E	775.309	903.368	1000.420	1099.489	1200.537	1287.569	1415.627	1512.680	1640.738	1737.791	1866.834
c+1	T*	Q	P	V	T	S	Q	P	Q	P	E	776.317	904.376	1001.428	1100.497	1201.544	1288.576	1416.635	1513.688	1641.746	1738.799	1867.842
z	T*	Q	P	V	T	S	Q	P	Q	P	E	131.034	228.087	356.145	453.198	581.257	668.289	769.336	868.405	965.457	1093.516	1850.791
z+1	T*	Q	P	V	T	S	Q	P	Q	P	E	132.042	229.094	357.153	454.206	582.264	669.296	770.344	869.413	966.465	1094.524	1851.799
z+2	T*	Q	P	V	T	S	Q	P	Q	P	E	133.050	230.102	358.161	455.214	583.272	670.304	771.352	870.420	967.473	1095.532	1852.807
	11	10	9	8	7	6	5	4	3	2	1	Glu	Pro	Gln	Pro	Gln	Ser	Thr	Val	Pro	Gln	Thr

BioTools-Score: 12

known O-glycosylation site

Alpha-2-HS-glycoprotein precursor $_{252}$TQPVTSQPQPE$_{262}$

Fraction 17

934.36++ → Pep [M+H]+ 1211.55+ [14.1-14.3 min] ETD

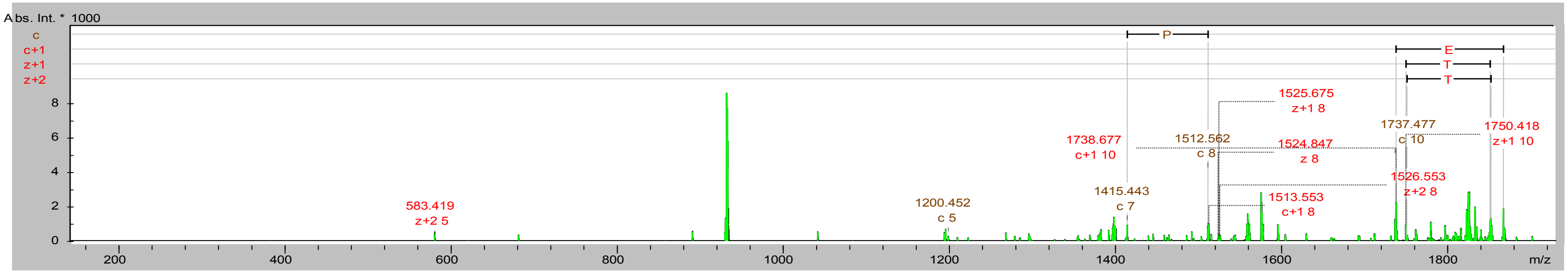

TQPVTSQPQPE

	T	Q	P	V	T	S	Q	P	Q	P	E	Thr	Gln	Pro	Val	Thr	Ser	Gln	Pro	Gln	Pro	Glu
Ion	1	2	3	4	5	6	7	8	9	10	11	1	2	3	4	5	6	7	8	9	10	11
c	T	Q	P	V	T*	S	Q	P	Q	P	E	119.082	247.140	344.193	443.261	1200.537	1287.569	1415.627	1512.680	1640.738	1737.791	1866.834
c+1	T	Q	P	V	T*	S	Q	P	Q	P	E	120.089	248.148	345.201	444.269	1201.544	1288.576	1416.635	1513.688	1641.746	1738.799	1867.842
z	T	Q	P	V	T*	S	Q	P	Q	P	E	131.034	228.087	356.145	453.198	581.257	668.289	1425.564	1524.632	1621.685	1749.744	1850.791
z+1	T	Q	P	V	T*	S	Q	P	Q	P	E	132.042	229.094	357.153	454.206	582.264	669.296	1426.572	1525.640	1622.693	1750.751	1851.799
z+2	T	Q	P	V	T*	S	Q	P	Q	P	E	133.050	230.102	358.161	455.214	583.272	670.304	1427.580	1526.648	1623.701	1751.759	1852.807
	11	10	9	8	7	6	5	4	3	2	1	Glu	Pro	Gln	Pro	Gln	Ser	Thr	Val	Pro	Gln	Thr

BioTools-Score: 19

known O-glycosylation site

Alpha-2-HS-glycoprotein precursor

$_{252}$TQPVTSQPQPE$_{262}$

Fraction 17

934.36++ → Pep [M+H]+ 1211.55+ [14.1-14.3 min] ETD

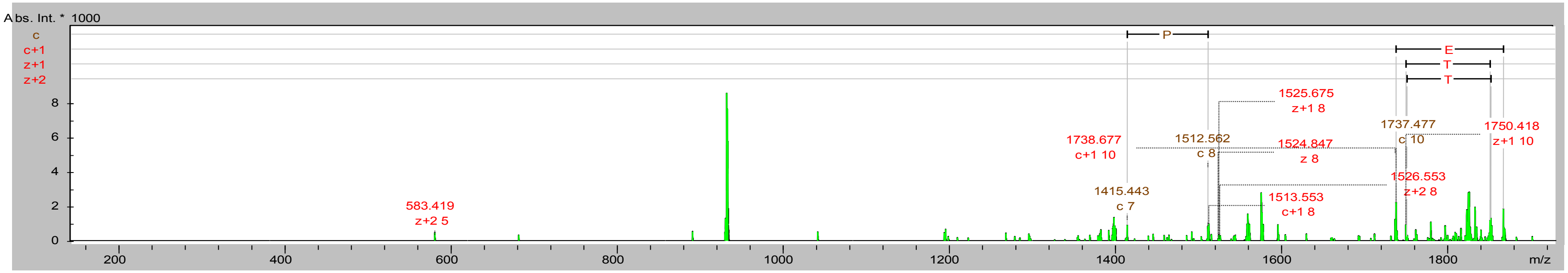

TQPVT**S**QPQPE

	T	Q	P	V	T	S	Q	P	Q	P	E	Thr	Gln	Pro	Val	Thr	Ser	Gln	Pro	Gln	Pro	Glu
Ion	1	2	3	4	5	6	7	8	9	10	11	1	2	3	4	5	6	7	8	9	10	11
c	T	Q	P	V	T	S*	Q	P	Q	P	E	119.082	247.140	344.193	443.261	544.309	1287.569	1415.627	1512.680	1640.738	1737.791	1866.834
c+1	T	Q	P	V	T	S*	Q	P	Q	P	E	120.089	248.148	345.201	444.269	545.317	1288.576	1416.635	1513.688	1641.746	1738.799	1867.842
z	T	Q	P	V	T	S*	Q	P	Q	P	E	131.034	228.087	356.145	453.198	581.257	1324.516	1425.564	1524.632	1621.685	1749.744	1850.791
z+1	T	Q	P	V	T	S*	Q	P	Q	P	E	132.042	229.094	357.153	454.206	582.264	1325.524	1426.572	1525.640	1622.693	1750.751	1851.799
z+2	T	Q	P	V	T	S*	Q	P	Q	P	E	133.050	230.102	358.161	455.214	583.272	1326.532	1427.580	1526.648	1623.701	1751.759	1852.807
	11	10	9	8	7	6	5	4	3	2	1	Glu	Pro	Gln	Pro	Gln	Ser	Thr	Val	Pro	Gln	Thr

BioTools-Score: 18

known O-glycosylation site

Alpha-2-HS-glycoprotein precursor

$_{252}$TQPV**T**SQPQPE$_{262}$

Fraction 17

887.82++ → Pep [M+H]+ 827.33+ [15.8 min] CID-MS Precursor

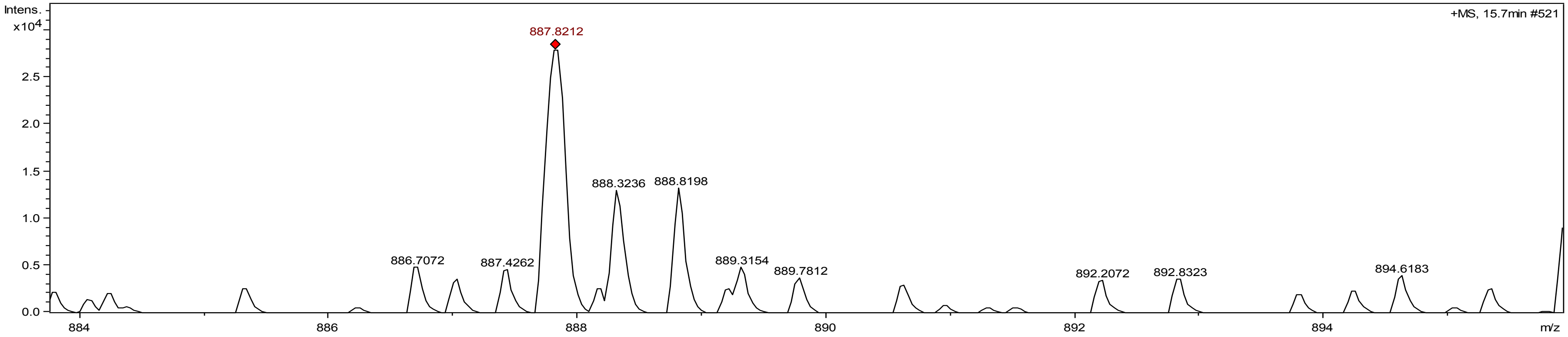

CID-MS³ spectrum of poor quality

ETD spectrum of poor quality

Fraction 17

887.82++ → Pep [M+H]+ 827.33+ [15.8 min] CID-MS2

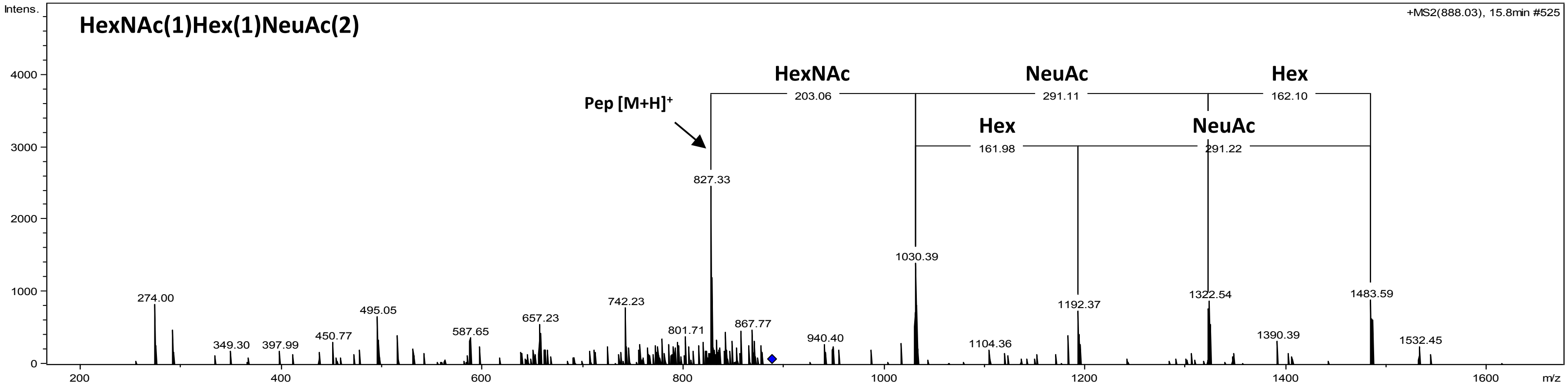

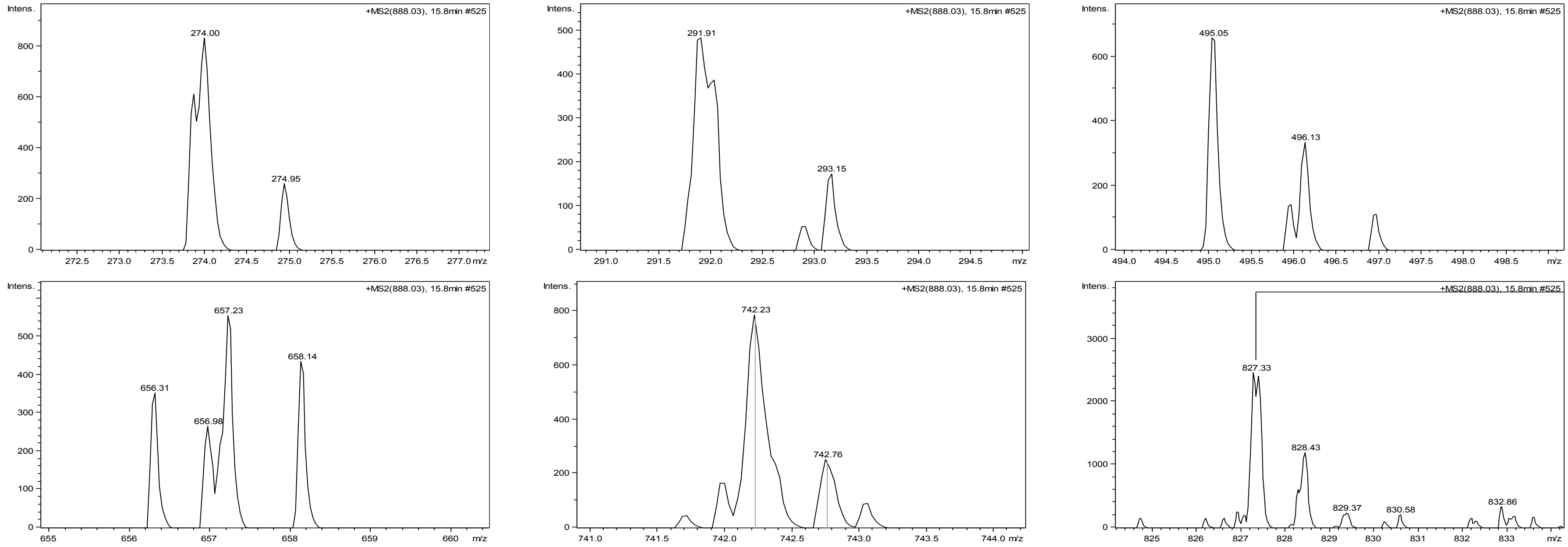

Fraction 17

887.82++ → Pep [M+H]+ 827.33+ [15.8 min]

CID-MS2

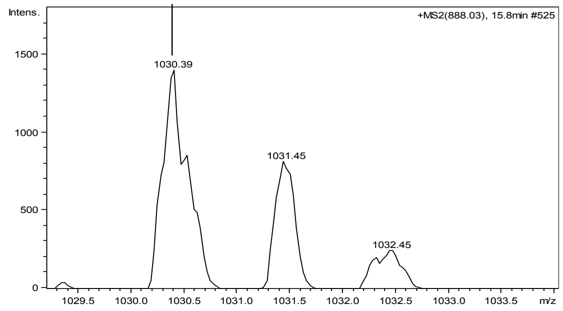

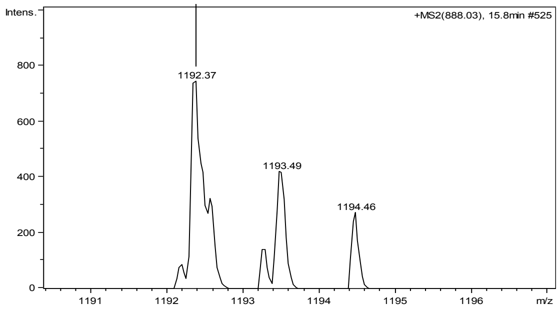

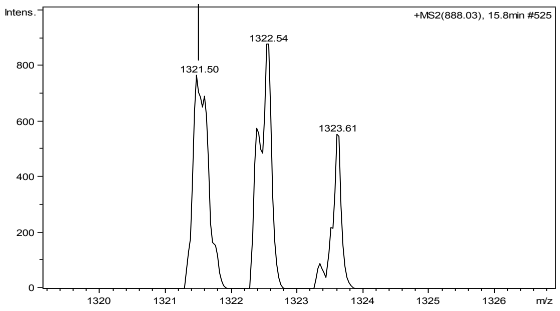

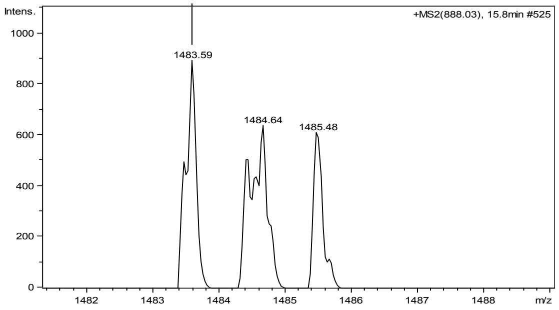

Fraction 17

887.82++ → Pep [M+H]+ 827.33+ [15.8 min]

CID-MS3 MASCOT Search

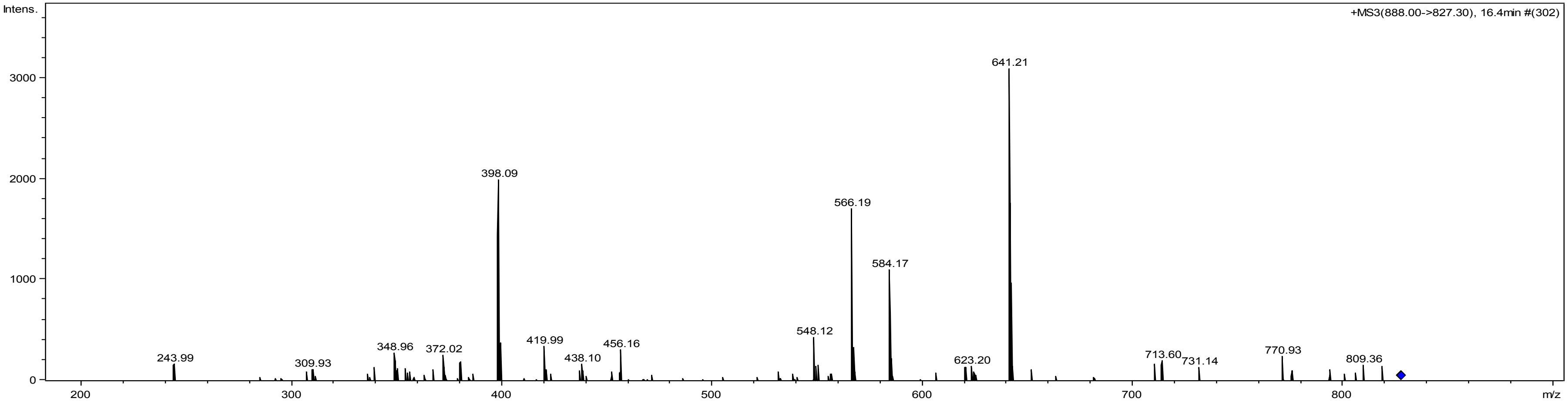

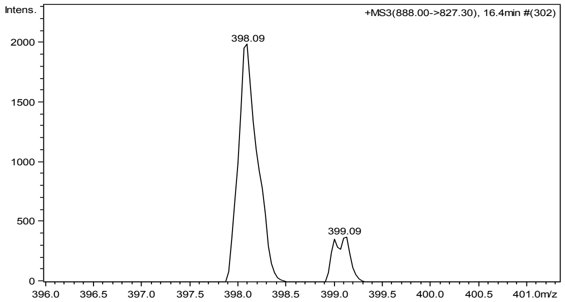

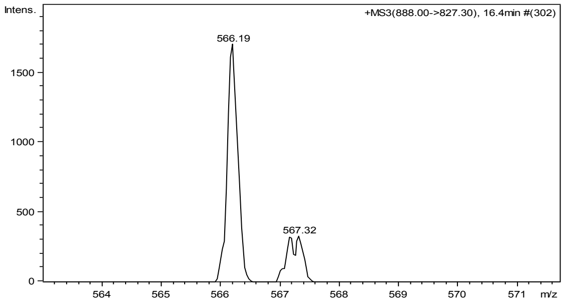

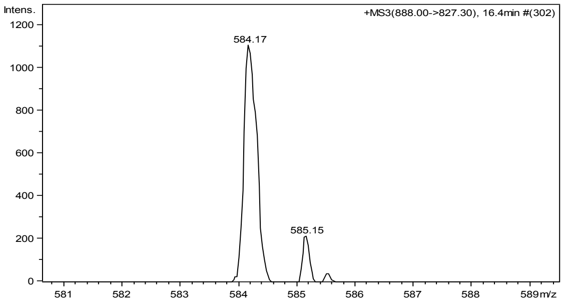

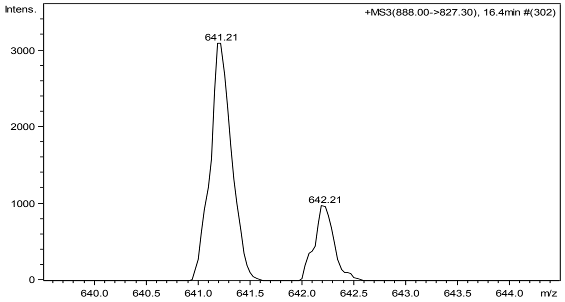

No unambiguous result

Fraction 17

651.99+++ → Pep+HexNAc [M+2H]++ 750.81++ [17.0 min]

CID-MS Precursor

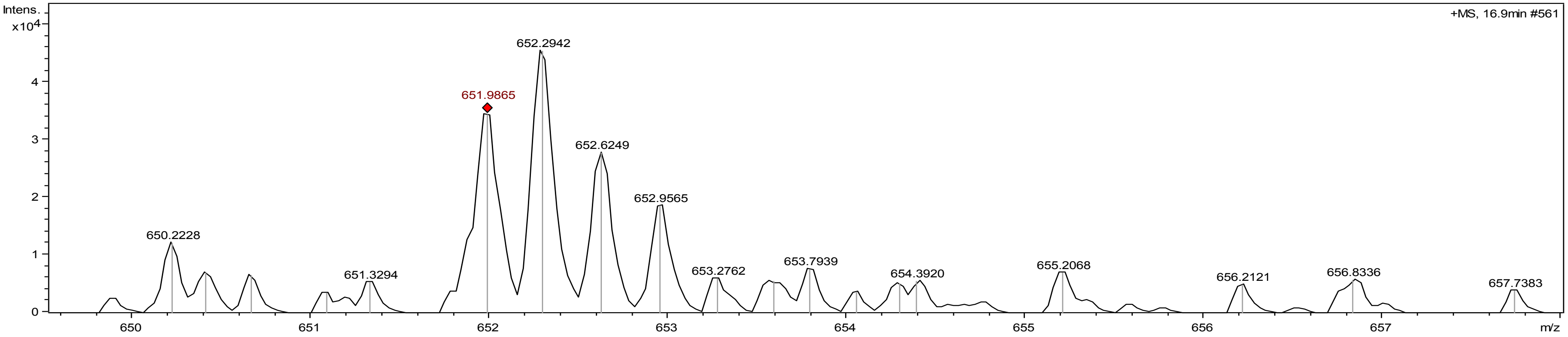

CID MS³ spectrum of poor quality

ETD spectrum of poor quality

Fraction 17

651.99+++ → Pep+HexNAc [M+2H]++ 750.81++ [17.0 min]

CID-MS2

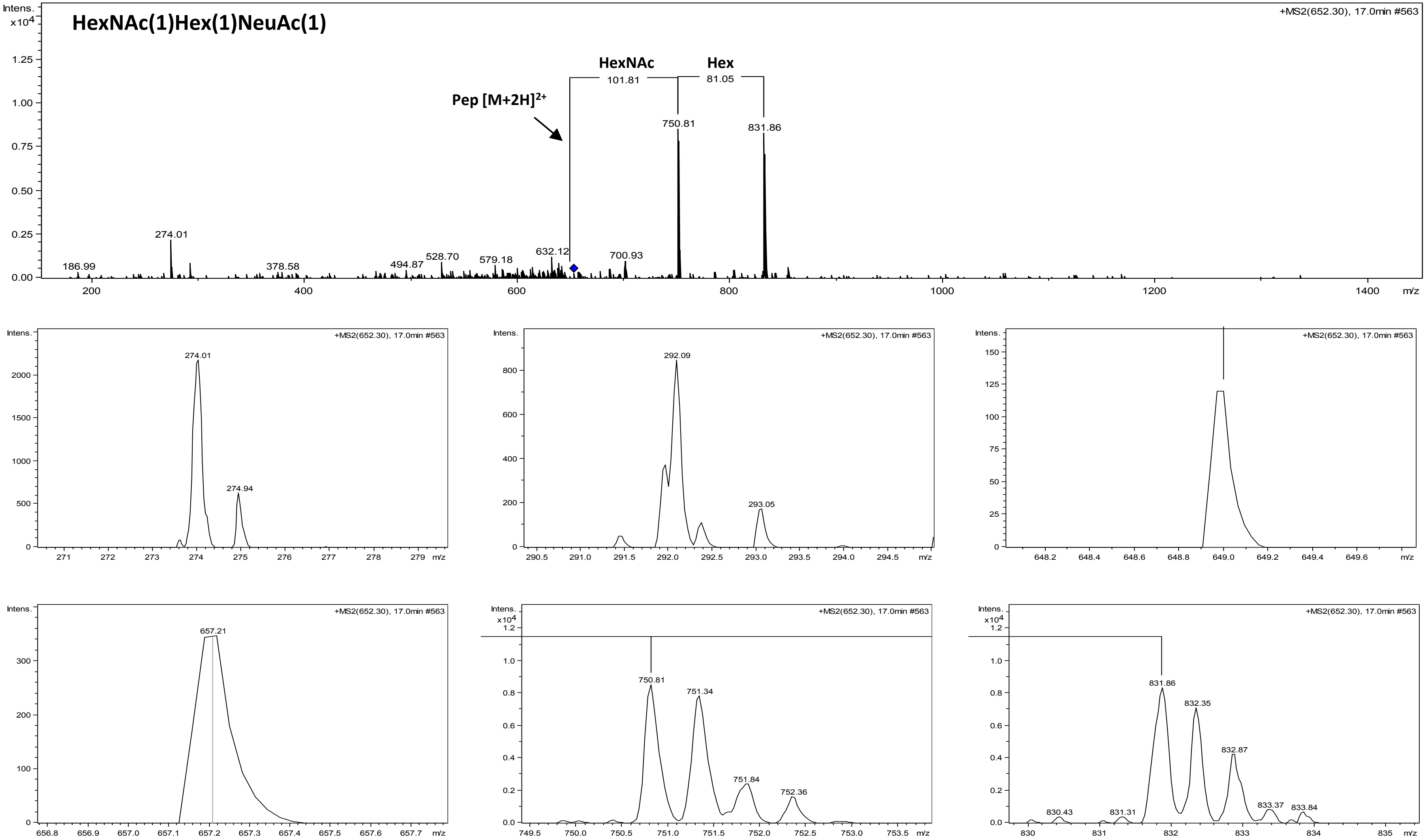

Fraction 17

874.28++ → Pep [M+H]+ 800.25+ [17.4 min]

CID-MS Precursor

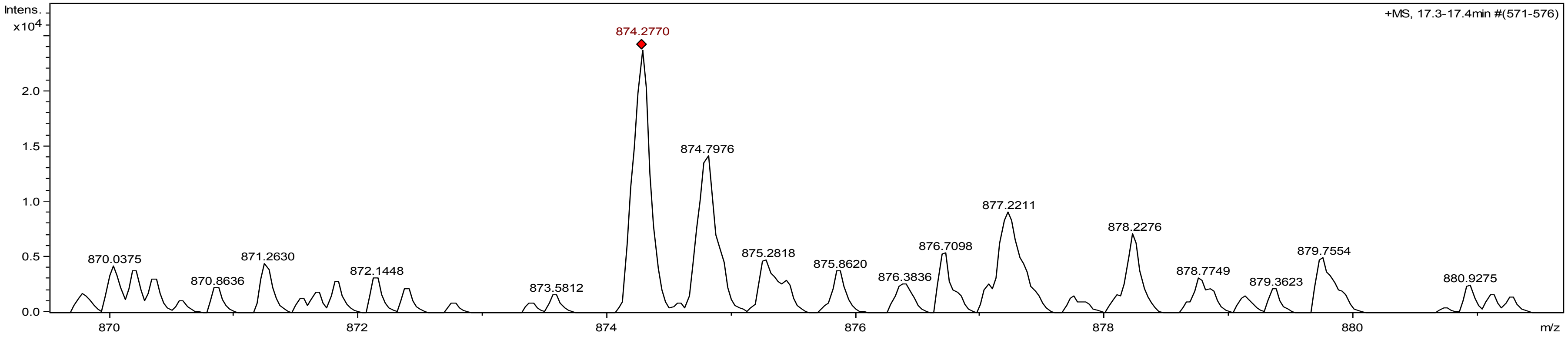

ETD spectrum of poor quality

Fraction 17

874.28++ → Pep [M+H]+ 800.25+ [17.4 min] CID-MS2

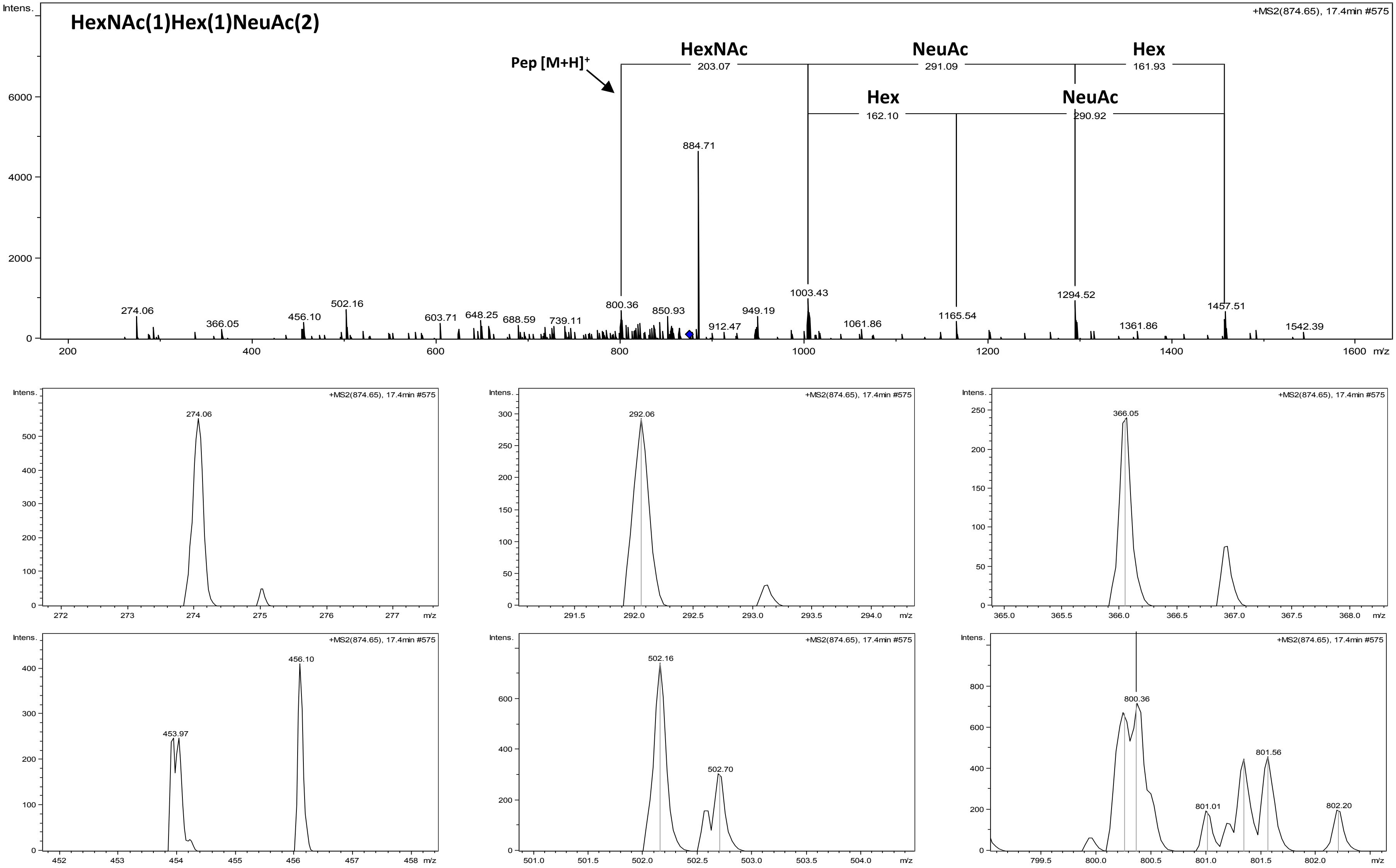

Fraction 17

874.28++ → Pep [M+H]+ 800.25+ [17.4 min]

CID-MS2

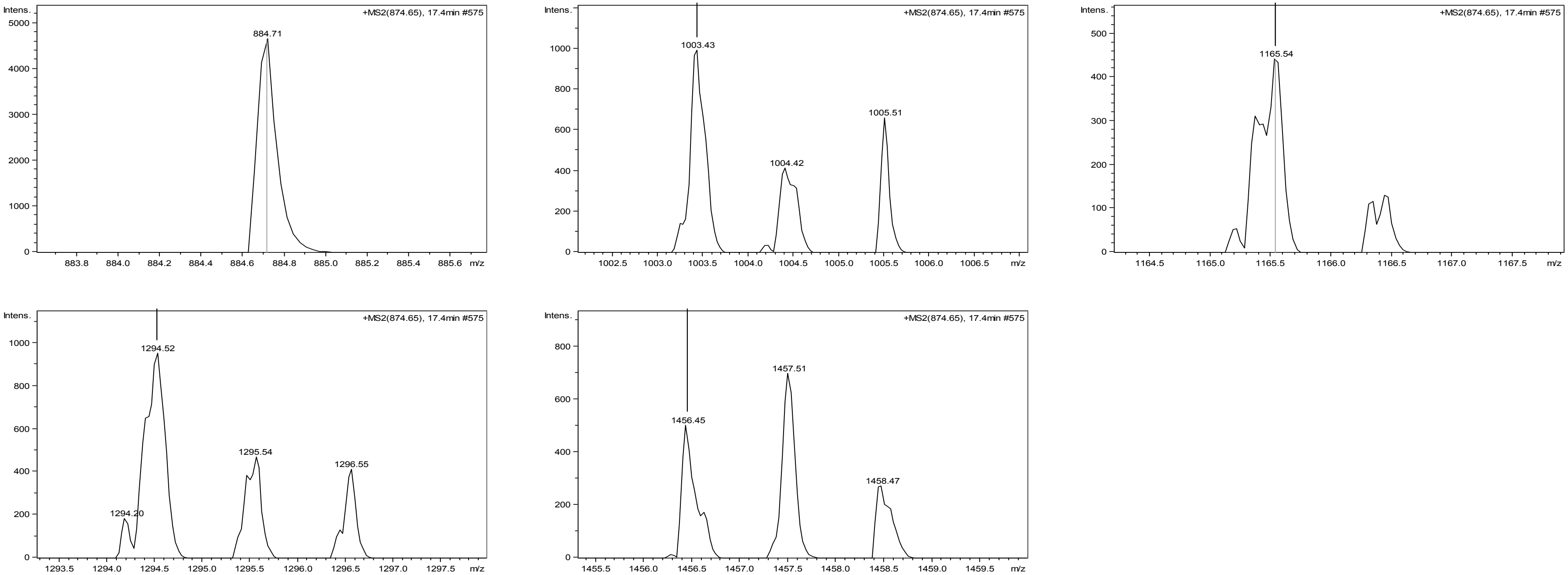

Fraction 17

874.28++ → Pep [M+H]+ 800.25+ [17.4 min] CID-MS3

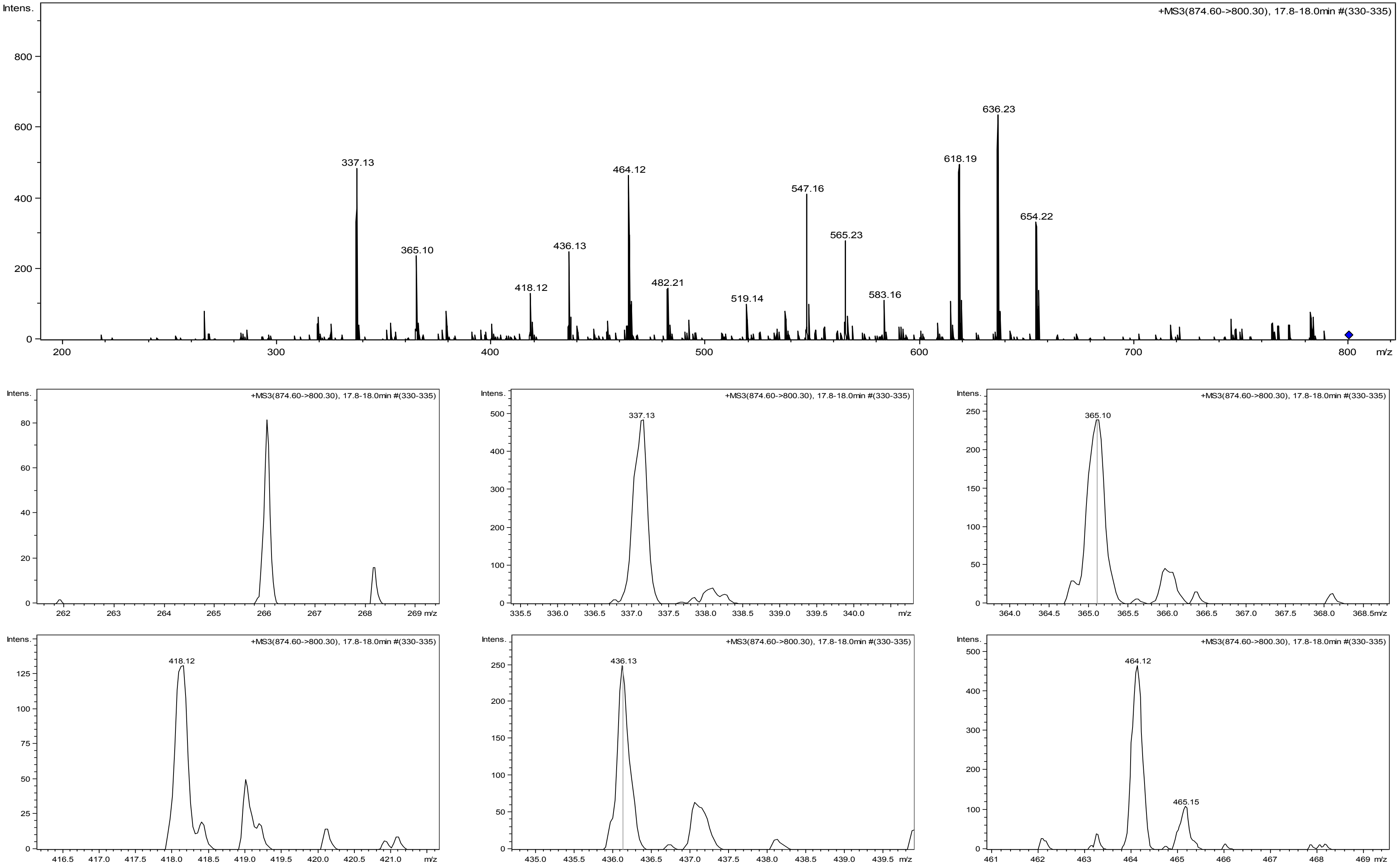

Fraction 17

874.28++ → Pep [M+H]+ 800.25+ [17.4 min]

CID-MS3

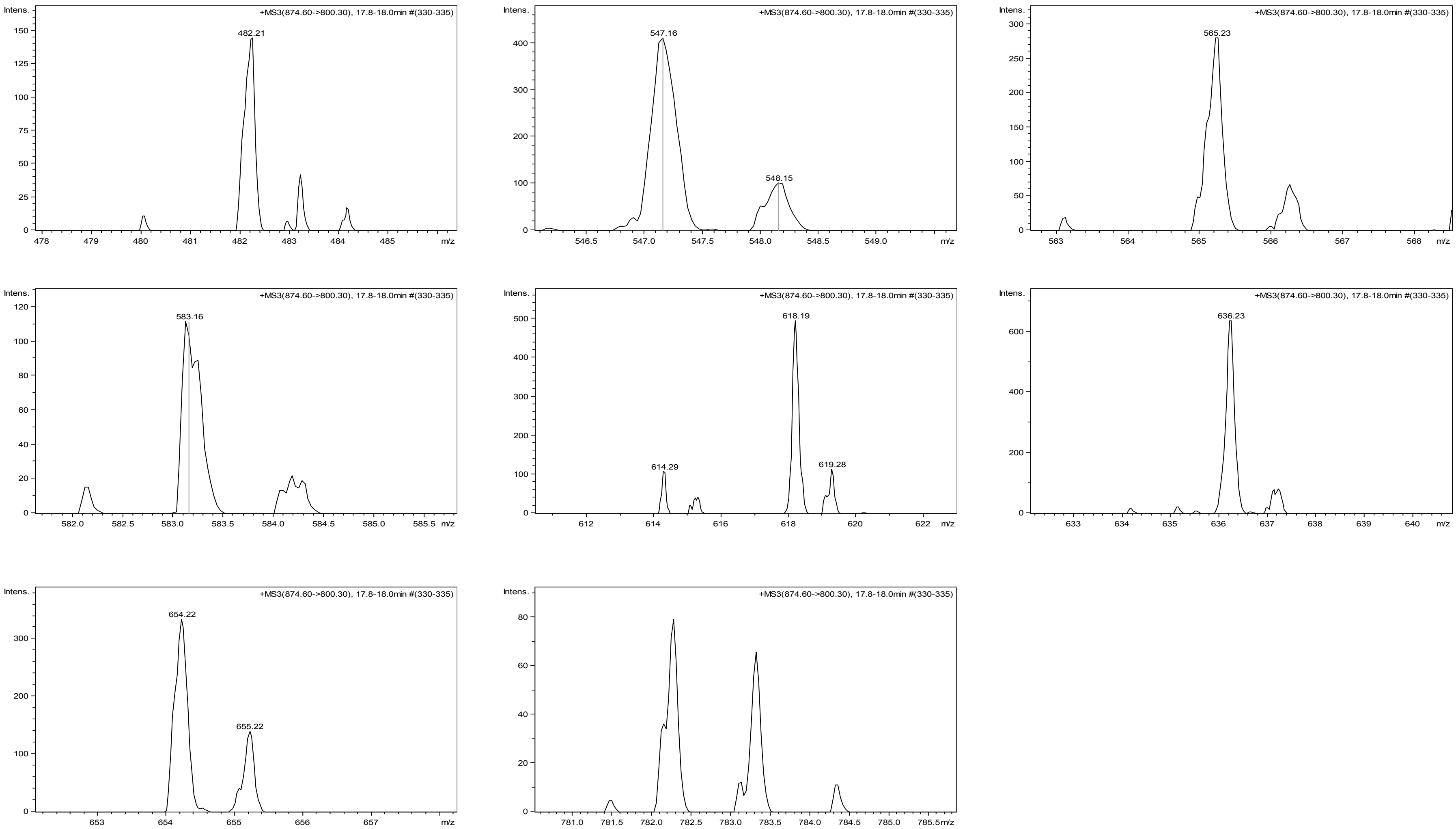

Fraction 17

874.28++ → Pep [M+H]+ 800.25+ [17.4 min]

CID-MS3 MASCOT Search

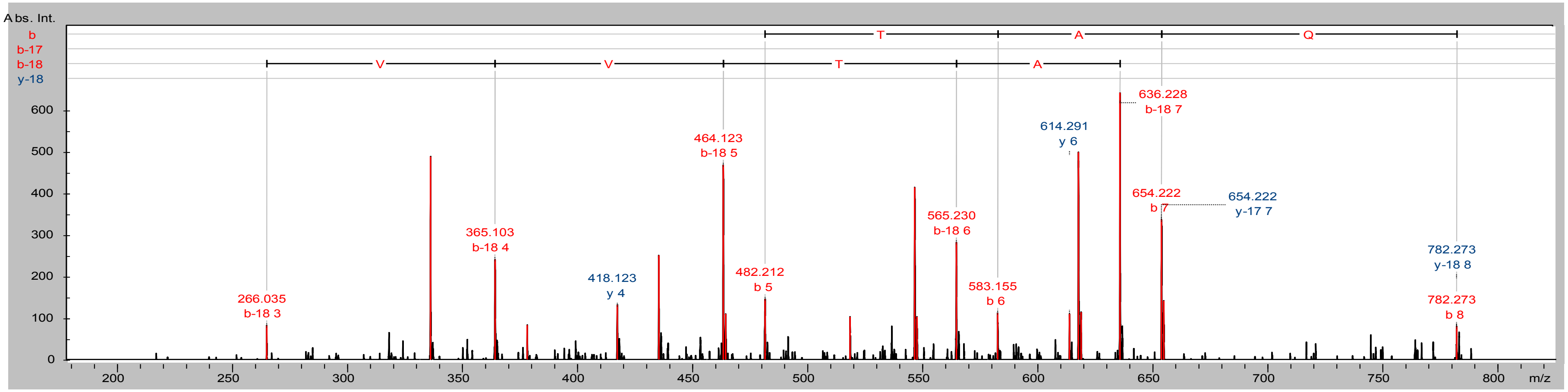

	E	G	P	V	V	T	A	Q	Glu	Gly	Pro	Val	Val	Thr	Ala	Gln
Ion	1	2	3	4	5	6	7	8	1	2	3	4	5	6	7	8
b	E	G	P	V	V	T	A	Q	130.050	187.071	284.124	383.193	482.261	583.309	654.346	782.404
b-17	E	G	P	V	V	T	A	Q	-	-	-	-	-	-	-	765.378
b-18	E	G	P	V	V	T	A	Q	112.039	169.061	266.114	365.182	464.250	565.298	636.335	764.394
y	E	G	P	V	V	T	A	Q	147.076	218.114	319.161	418.230	517.298	614.351	671.372	800.415
y-17	E	G	P	V	V	T	A	Q	130.050	201.087	302.135	401.203	500.271	597.324	654.346	783.388
y-18	E	G	P	V	V	T	A	Q	-	-	301.151	400.219	499.287	596.340	653.362	782.404
	8	7	6	5	4	3	2	1	Gln	Ala	Thr	Val	Val	Pro	Gly	Glu

known O-glycosylation site

Kininogen-1 precursor

$_{132}$EGPVV**T**AQ$_{139}$

Fraction 17

874.28++ → Pep [M+H]+ 800.25+ [17.4 min]

CID-MS3 MASCOT Search

prot_hit_nur	prot_acc	prot_desc	prot_score	prot_mass	prot_matche	pep_query	pep_rank	pep_isbold	pep_exp_mz	pep_exp_mr	pep_exp_z	pep_calc_mr	pep_delta	pep_miss	pep_score	pep_expect	pep_res_bef	pep_seq
1	GTPB1_HUM	GTP-binding	18	72046	1	1	1	1	800.25	799.2427	1	798.3946	0.8482	0	22.01	76	S	YATVKSM
2	KNG1_HUMA	Kininogen-1	13	72996	1	1	3	0	800.25	799.2427	1	799.4076	-0.1649	0	18.76	1.60E+02	A	EGPVVTAQ
3	TCF8_HUMAI	Transcriptior	13	125822	1	1	2	0	800.25	799.2427	1	799.444	-0.2012	0	19.8	1.30E+02	I	AIPTVTAQ
4	MUTYH_HUN	A/G-specific	11	60773	1	1	5	0	800.25	799.2427	1	799.346	-0.1033	0	15.54	3.40E+02	K	HAKNNSQ
5	BCL6B_HUM	B-cell CLL/lyı	11	52669	1	1	5	0	800.25	799.2427	1	799.3824	-0.1397	0	15.54	3.40E+02	K	HGAATNTK
6	GRIA3_HUM	Glutamate re	10	101662	1	1	5	0	800.25	799.2427	1	799.3712	-0.1285	0	15.54	3.40E+02	K	PAPATNTQ
7	TROAP_HUN	Trophinin-as	9	85382	1	1	5	0	800.25	799.2427	1	799.346	-0.1033	0	15.54	3.40E+02	R	HQAETSQ
8	BRD3_HUMA	Bromodoma	9	79777	1	1	5	0	800.25	799.2427	1	799.4262	-0.1835	0	15.54	3.40E+02	A	PAAPMVSK
9	LPIN1_HUM	Lipin-1 - Hon	9	99287	1	1	5	0	800.25	799.2427	1	799.346	-0.1033	0	15.54	3.40E+02	A	KHASDNGA
10	ISK5_HUMAN	Serine prote	8	124444	1	1	4	0	800.25	799.2427	1	798.3218	0.9209	0	15.58	3.30E+02	Y	YEAVCGT

BioTools-Score: 19

MASCOT-Score: 19

Fraction 17

647.89+++ → Pep+HexNac(1)NeuAc(1) [M+2H]++ 744.74++ [18.0 min]

CID-MS Precursor

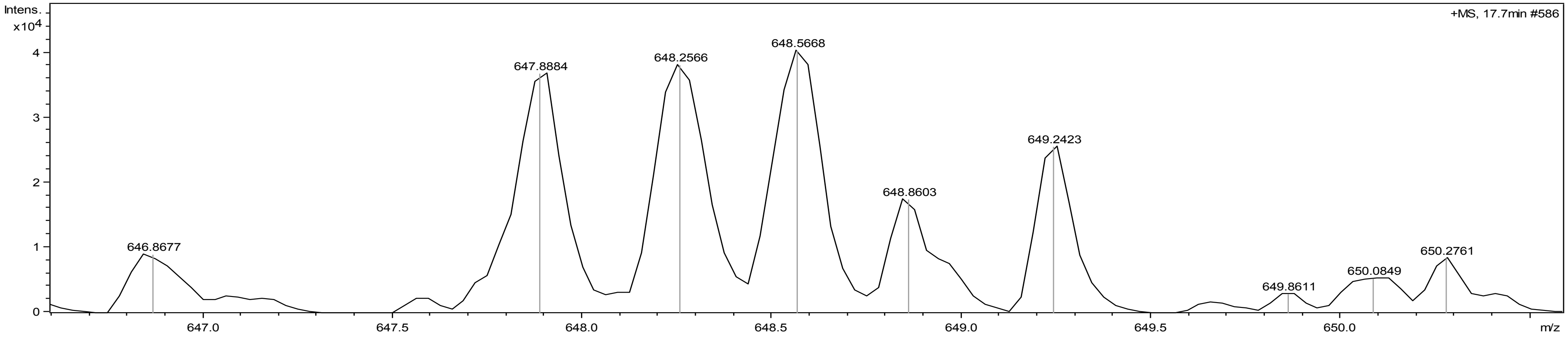

CID MS³ spectrum of poor quality

ETD spectrum of poor quality

Fraction 17

647.89+++ → Pep+HexNac(1)NeuAc(1) [M+2H]++ 744.74++ [18.0 min]

CID-MS2

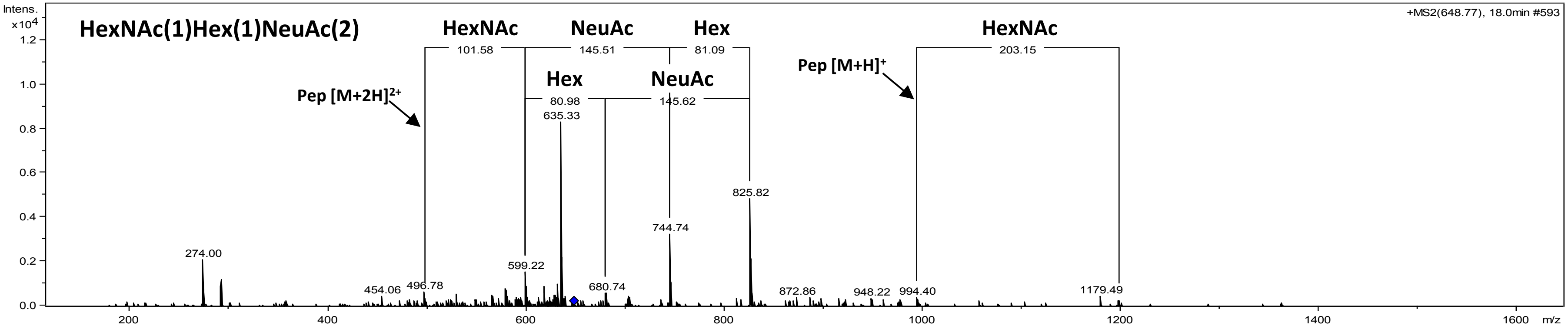

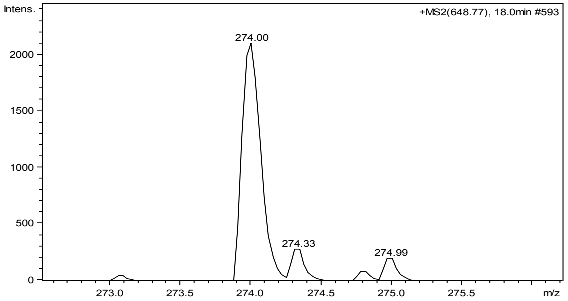

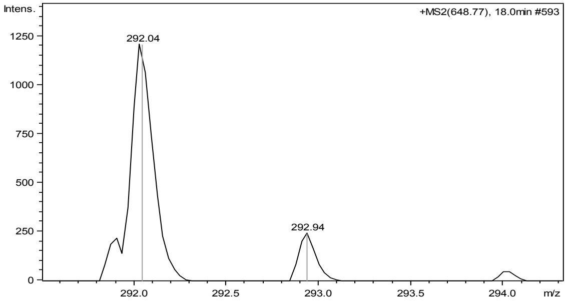

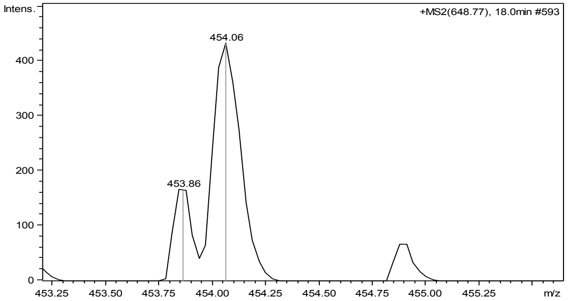

Fraction 17

647.89+++ → Pep+HexNac(1)NeuAc(1) [M+2H]++ 744.74++ [18.0 min]

CID-MS2

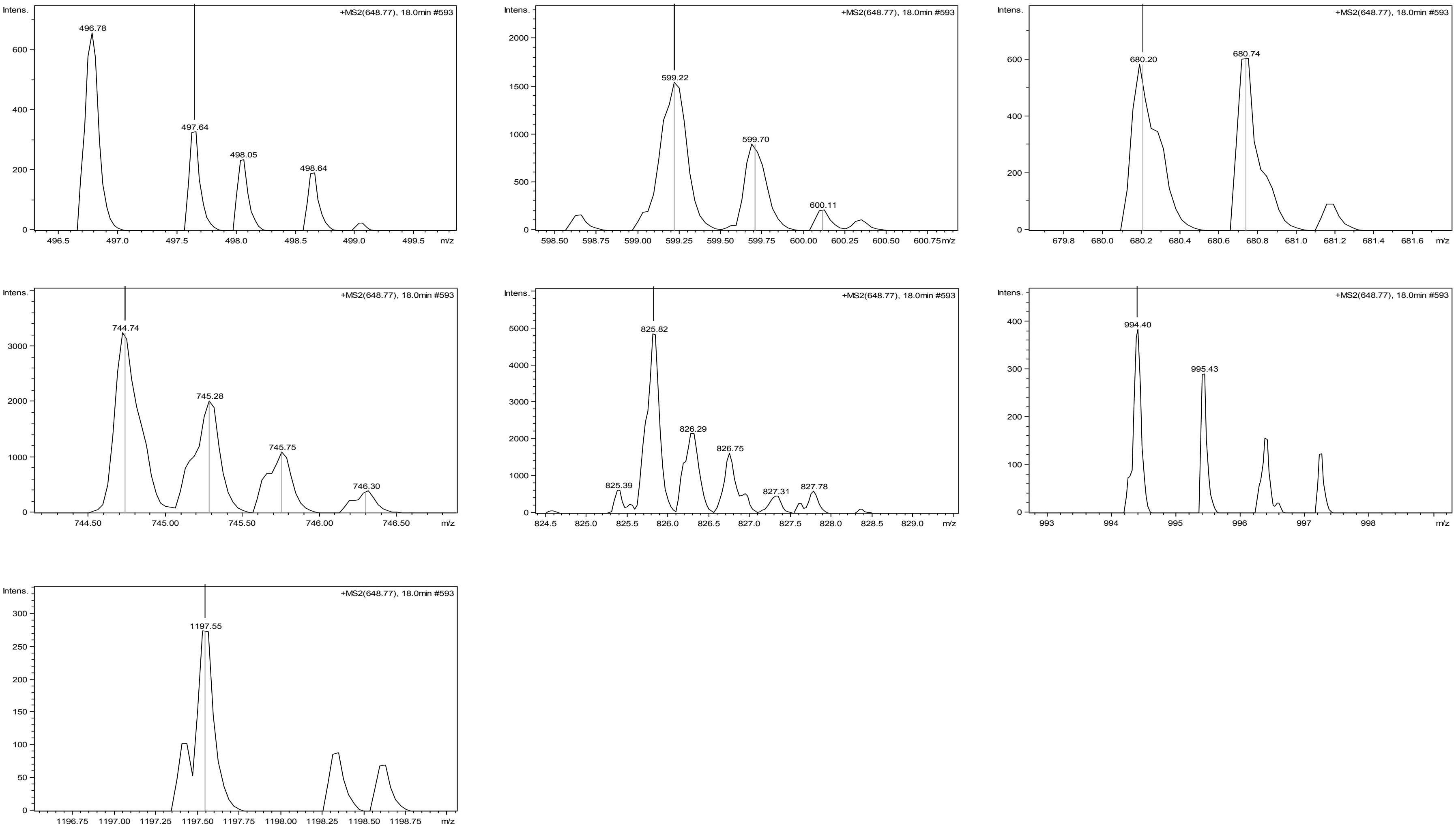

Fraction 17

760.92+++ → Pep [M+H]+ 968.40+ [21.0-21.1 min] CID-MS Precursor

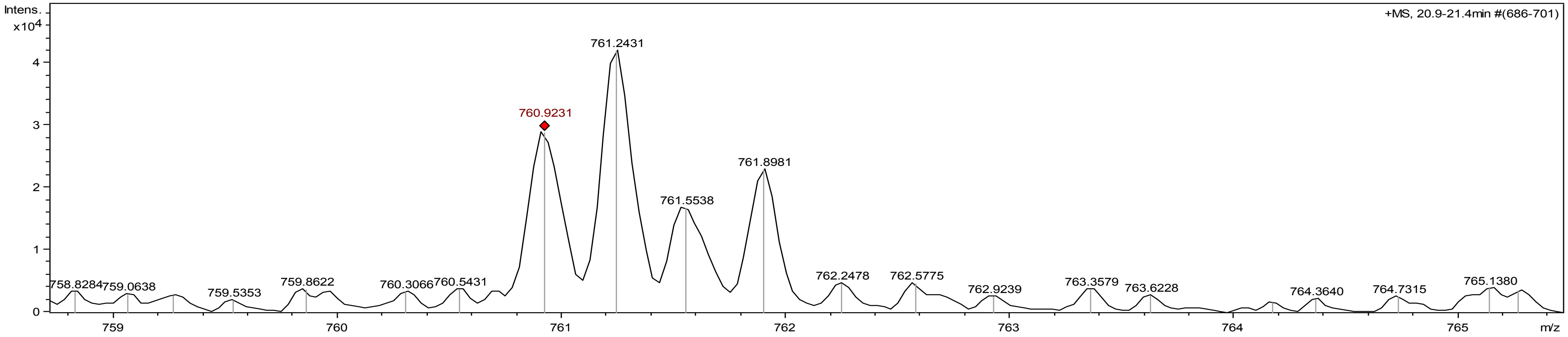

2 occupied glycosylation sites

Hexose rearrangement

ETD spectrum of poor quality

Fraction 17

760.92+++ → Pep [M+H]+ 968.40+ [21.0-21.1 min] CID-MS2

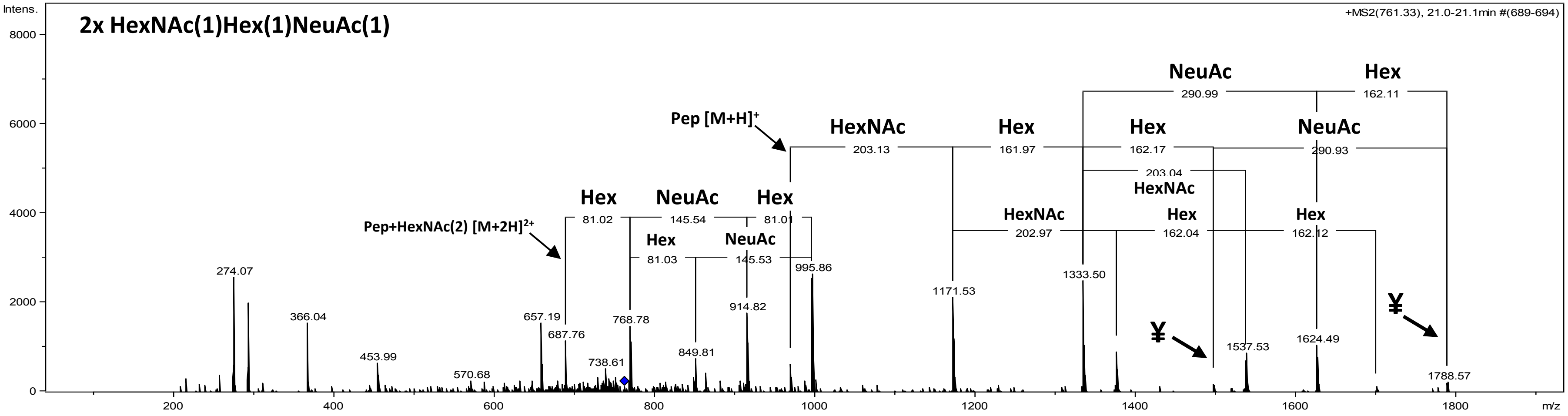

¥ Hexose rearrangement

Additional Hexose has been transferred either to the glycan or to the peptide backbone

Fraction 17

760.92+++ → Pep [M+H]+ 968.40+ [21.0-21.1 min] CID-MS2

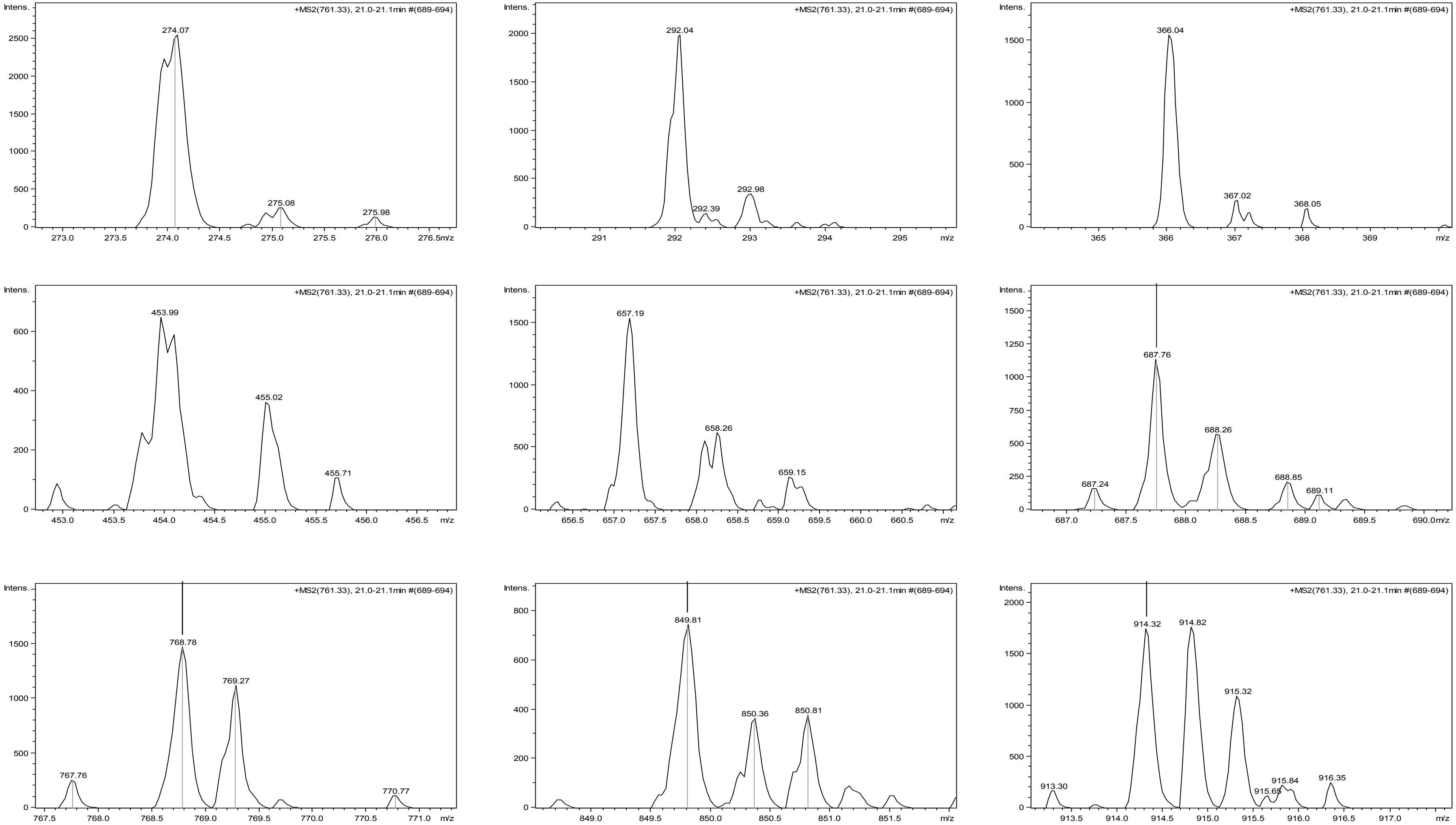

Fraction 17

760.92+++ → Pep [M+H]+ 968.40+ [21.0-21.1 min] CID-MS2

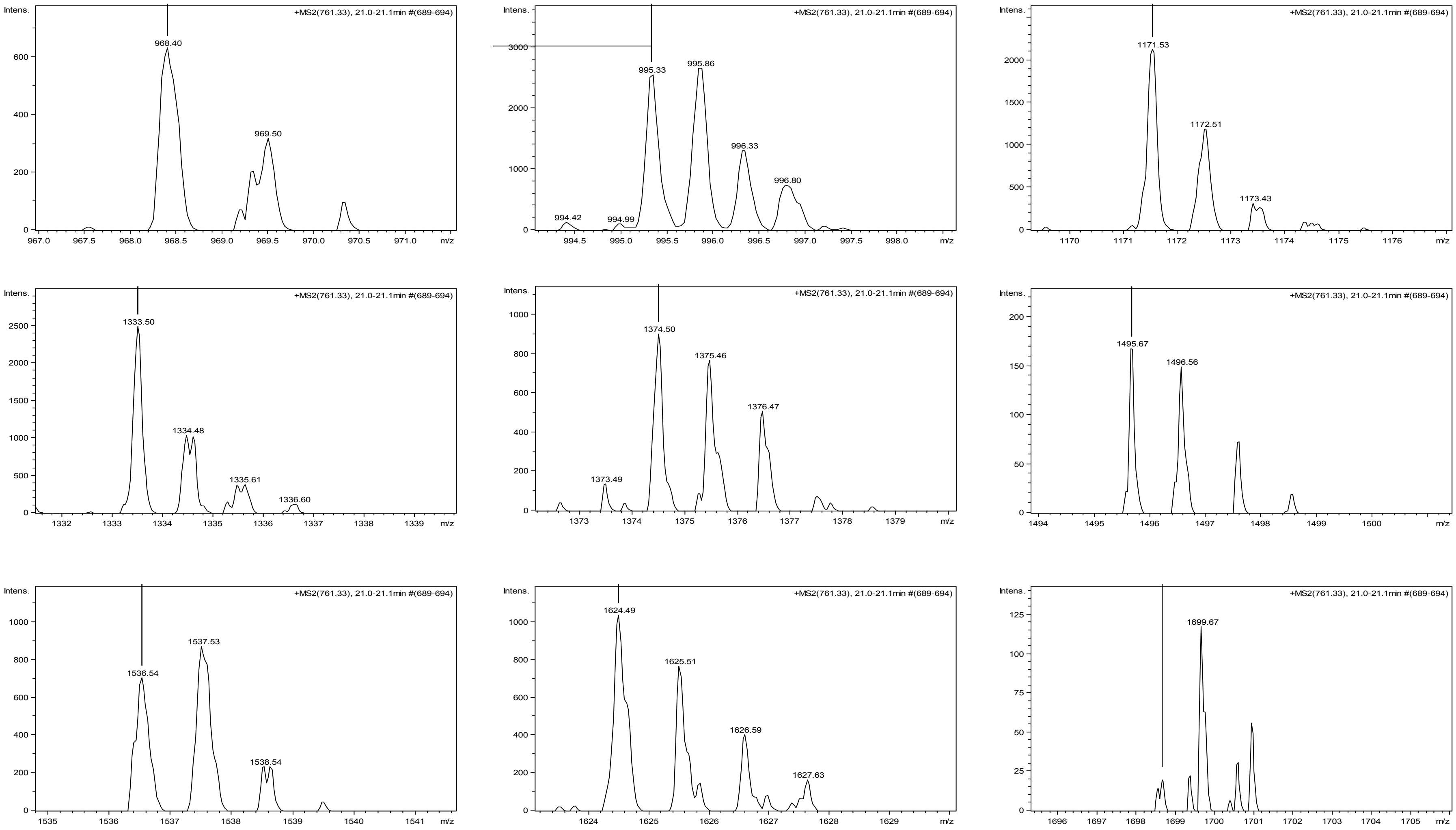

Fraction 17

760.92+++ → Pep [M+H]+ 968.40+ [21.0-21.1 min] CID-MS2

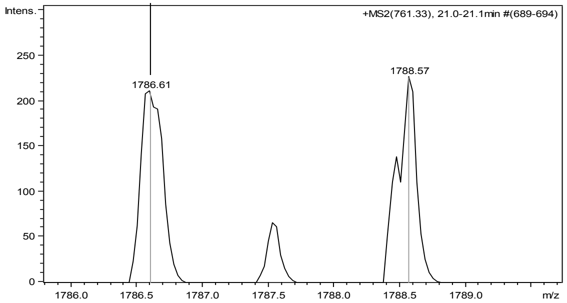

Fraction 17

760.92+++ → Pep [M+H]+ 968.40+ [21.0-21.1 min] CID-MS3

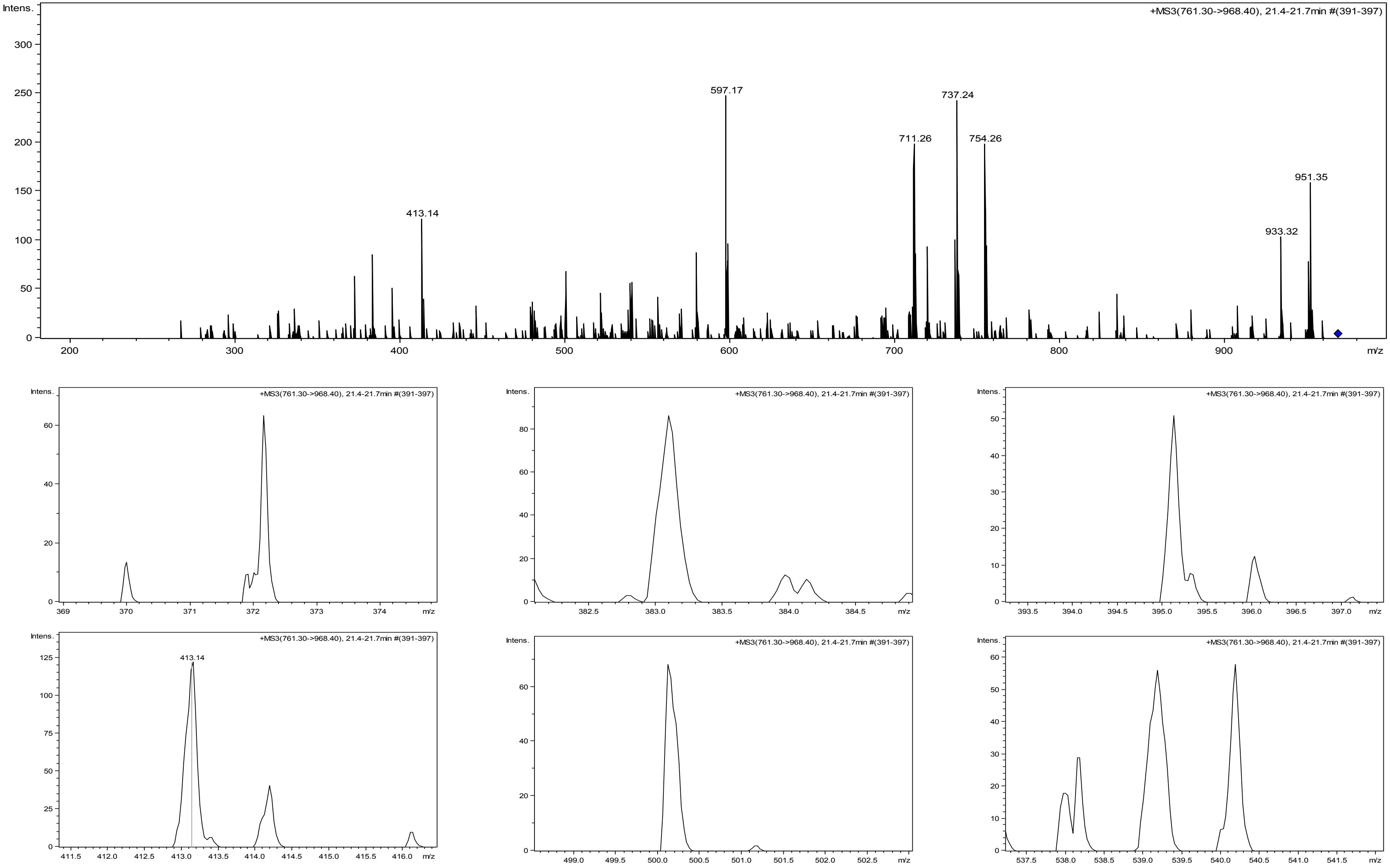

Fraction 17

760.92+++ → Pep [M+H]+ 968.40+ [21.0-21.1 min]

CID-MS3

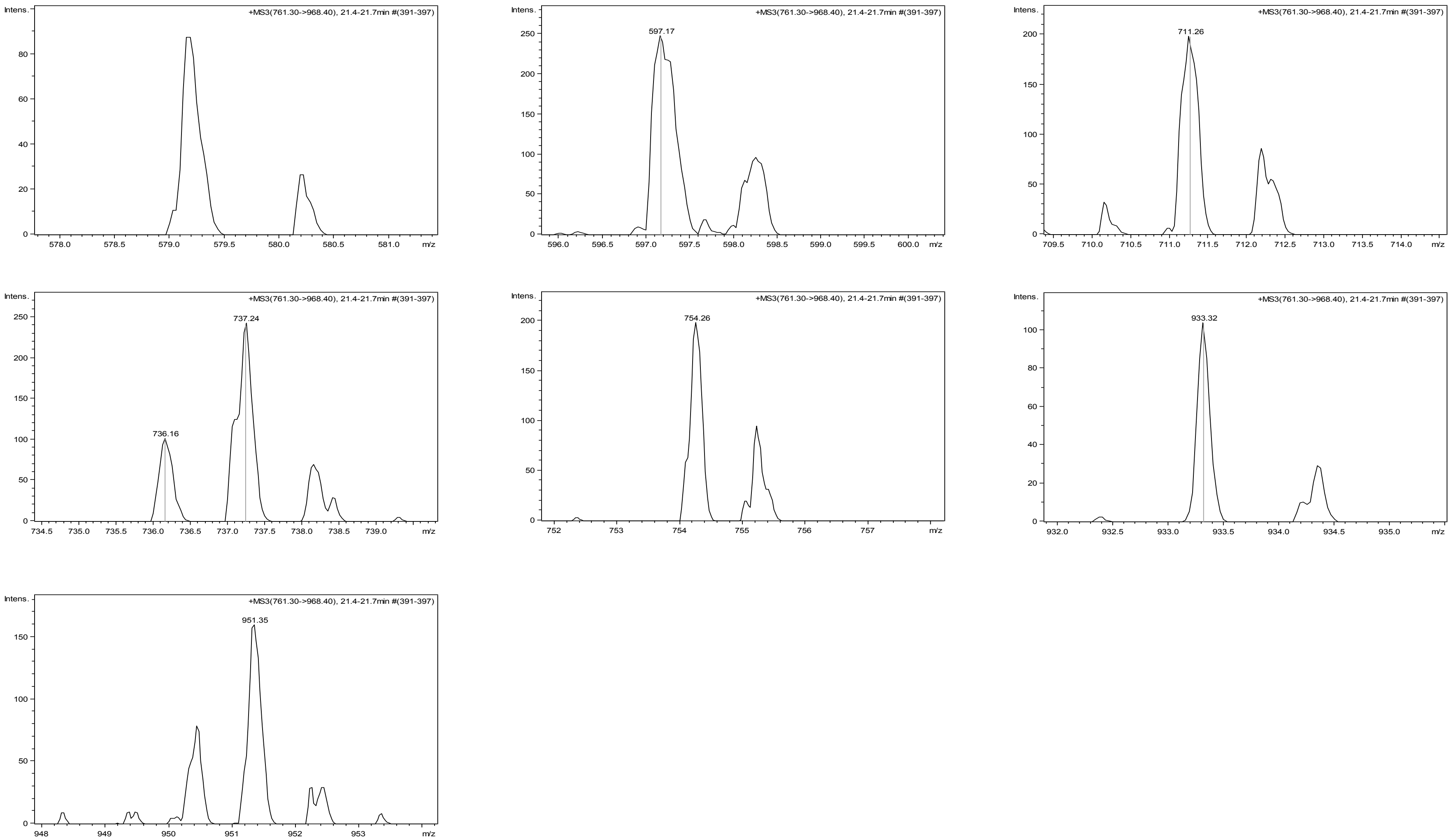

Fraction 17

760.92+++ → Pep [M+H]+ 968.40+ [21.0-21.1 min] CID-MS3 MASCOT Search

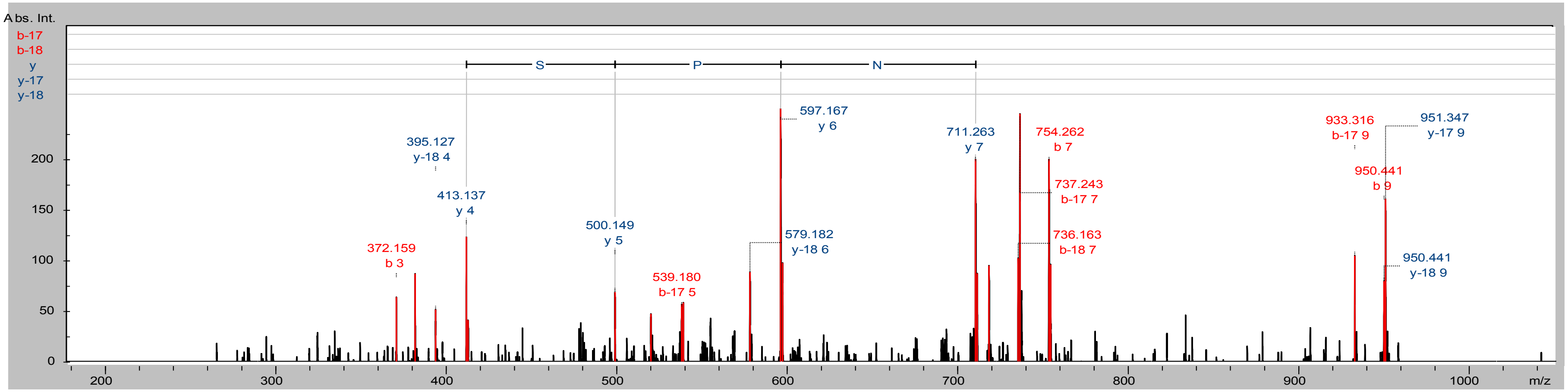

	W	A	N	P	S	P	T	P	V	Trp	Ala	Asn	Pro	Ser	Pro	Thr	Pro	Val
Ion	1	2	3	4	5	6	7	8	9	1	2	3	4	5	6	7	8	9
b	W	A	N	P	S	P	T	P	V	187.087	258.124	372.167	469.219	556.251	653.304	754.352	851.405	950.473
b-17	W	A	N	P	S	P	T	P	V	-	-	355.140	452.193	539.225	636.278	737.325	834.378	933.446
b-18	W	A	N	P	S	P	T	P	V	-	-	-	-	538.241	635.294	736.341	833.394	932.462
y	W	A	N	P	S	P	T	P	V	118.086	215.139	316.187	413.239	500.271	597.324	711.367	782.404	968.484
y-17	W	A	N	P	S	P	T	P	V	-	-	-	-	-	-	694.341	765.378	951.457
y-18	W	A	N	P	S	P	T	P	V	-	-	298.176	395.229	482.261	579.314	693.357	764.394	950.473
	9	8	7	6	5	4	3	2	1	Val	Pro	Thr	Pro	Ser	Pro	Asn	Ala	Trp

known O-glycosylation site

Inter-alpha-trypsin inhibitor heavy chain H2

$_{669}$WANP**S**P**T**PV$_{677}$

Fraction 17

760.92+++ → Pep [M+H]+ 968.40+ [21.0-21.1 min] CID-MS3 MASCOT Search

prot_hit_nur	prot_acc	prot_desc	prot_score	prot_mass	prot_matche	pep_query	pep_rank	pep_isbold	pep_exp_mz	pep_exp_mr	pep_exp_z	pep_calc_mr	pep_delta	pep_miss	pep_score	pep_expect	pep_res_bef	pep_seq
1	ITIH2_HUMA	Inter-alpha-t	24	106826	1	1	1	1	968.4039	967.3966	1	967.4763	-0.0797	0	29.98	14	S	WANPSPTPV
2	NUD22_HUM	Nucleoside c	19	32857	1	1	2	0	968.4039	967.3966	1	967.3818	0.0148	0	23.04	67	L	CPGGSPQHQ
3	PHLP_HUMA	Phosducin-li	18	34659	1	1	2	0	968.4039	967.3966	1	967.5814	-0.1848	0	23.04	67	F	TRNALPALL
4	RD23B_HUM	UV excision r	17	43202	1	1	2	0	968.4039	967.3966	1	967.4974	-0.1008	0	23.04	67	I	QQNPSLLPA
5	PMS1_HUMA	PMS1 protei	16	106619	1	1	2	0	968.4039	967.3966	1	967.4875	-0.0909	0	23.04	67	S	AWNLAQKH
6	KI67_HUMAN	Antigen KI-6	15	360698	1	1	2	0	968.4039	967.3966	1	967.4359	-0.0393	0	23.04	67	Q	QQNSPQKH

BioTools-Score: 1

MASCOT-Score: 30

known O-glycosylation site

Inter-alpha-trypsin inhibitor heavy chain H2

$_{669}$WANP**S**P**T**PV$_{677}$

Fraction 17

652.93+++ → Pep [M+H]+ 1009.47+ [23.0-23.2 min] CID-MS Precursor

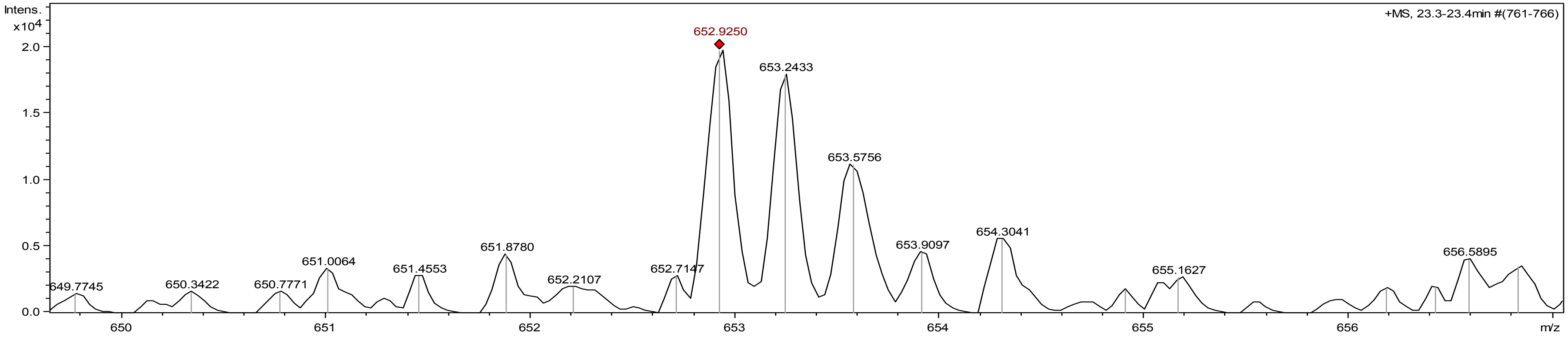

Fraction 17

652.93+++ → Pep [M+H]+ 1009.47+ [23.0-23.2 min] CID-MS2

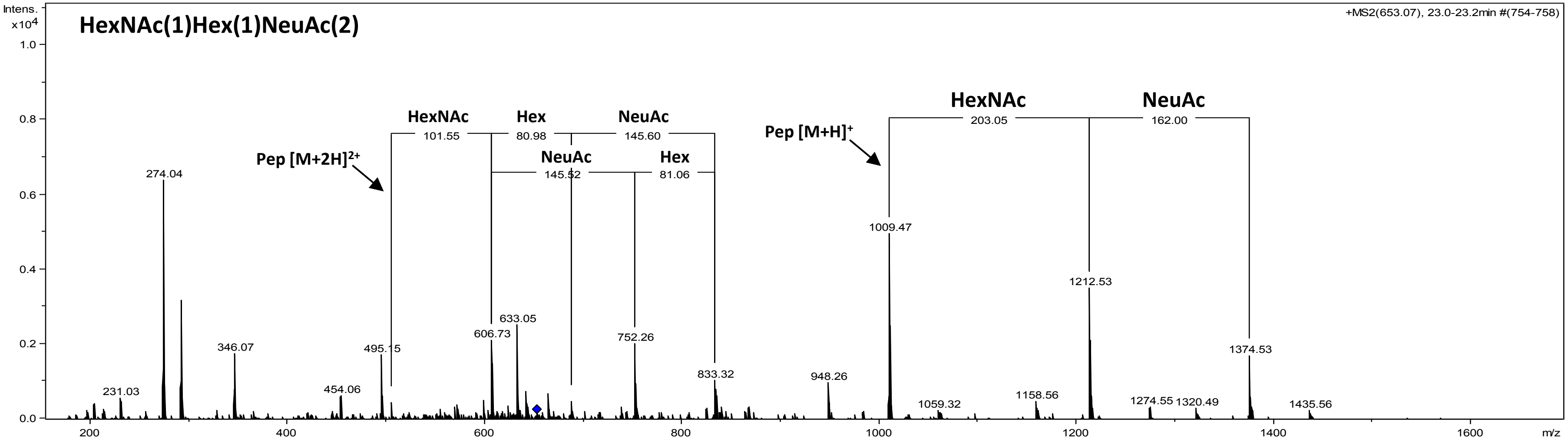

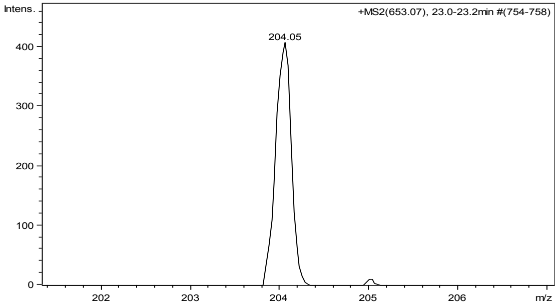

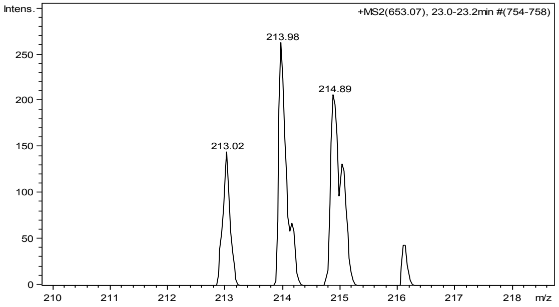

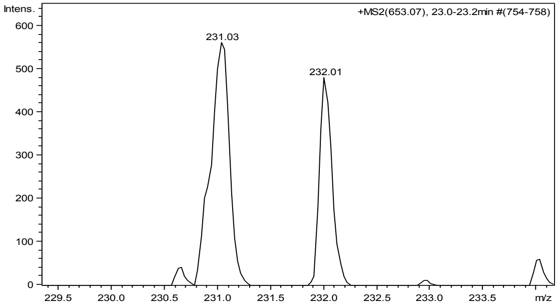

Fraction 17

652.93+++ → Pep [M+H]+ 1009.47+ [23.0-23.2 min] CID-MS2

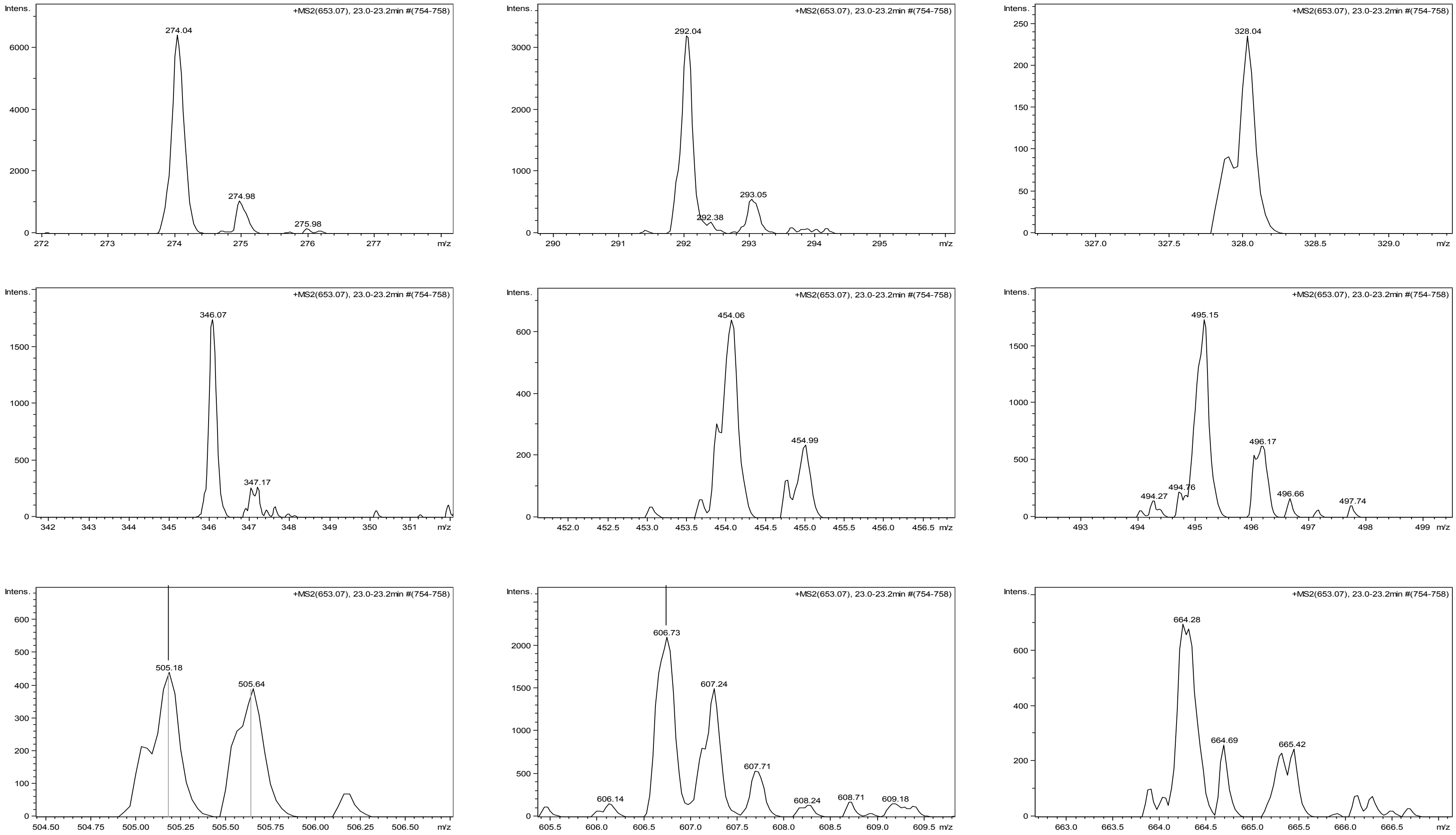

Fraction 17

652.93+++ → Pep [M+H]+ 1009.47+ [23.0-23.2 min]

CID-MS2

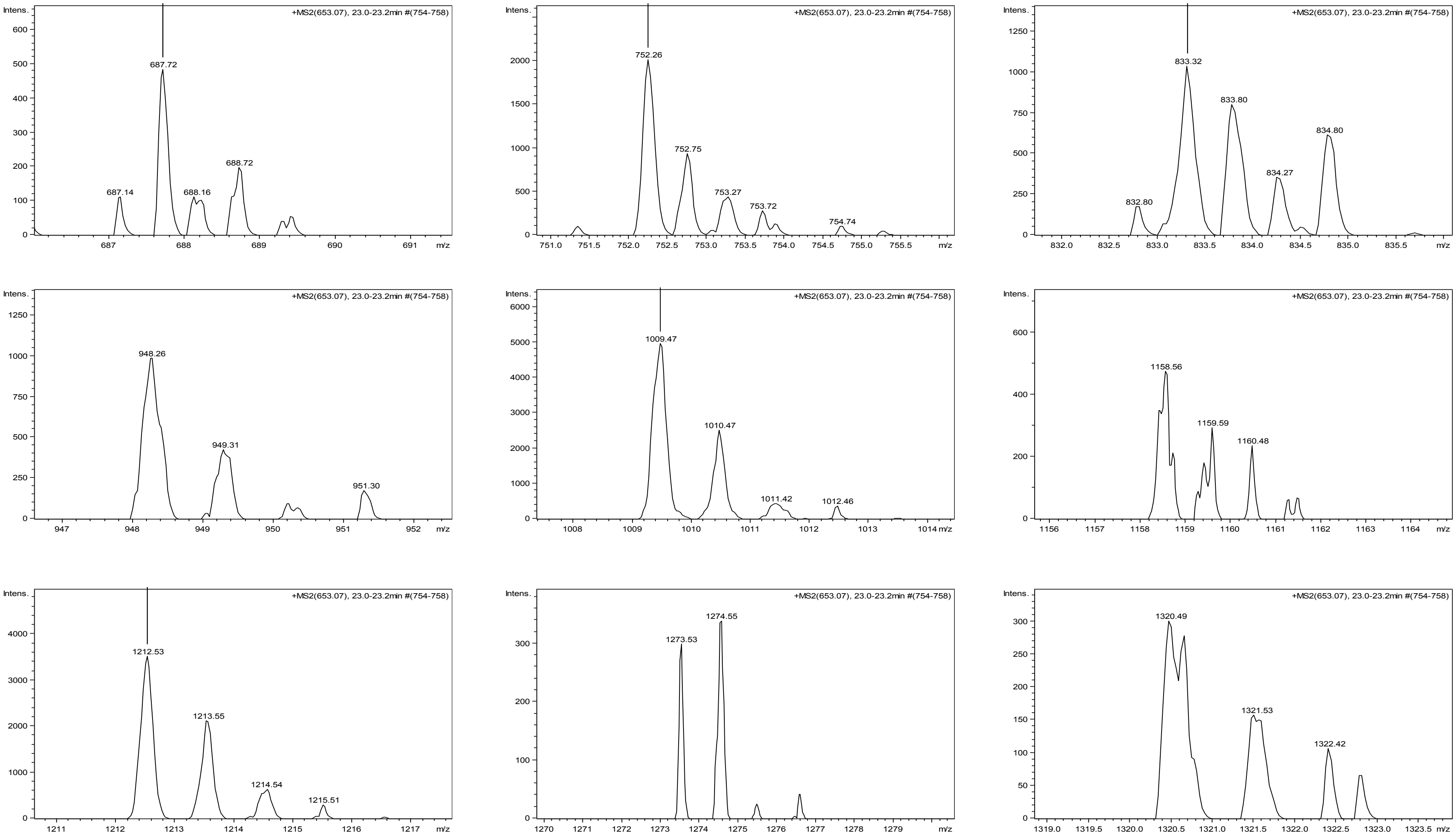

Fraction 17

652.93+++ → Pep [M+H]+ 1009.47+ [23.0-23.2 min] CID-MS2

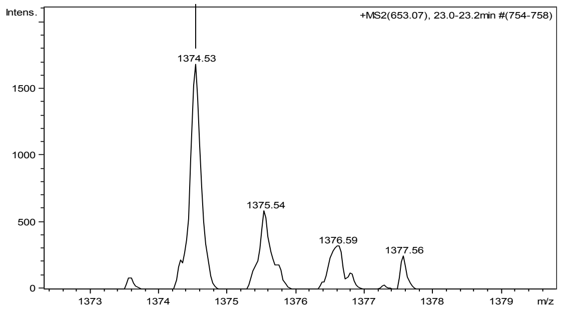

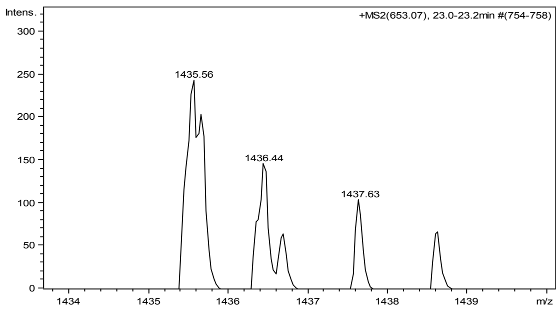

Fraction 17

652.93+++ → Pep [M+H]+ 1009.47+ [23.0-23.2 min] CID-MS3

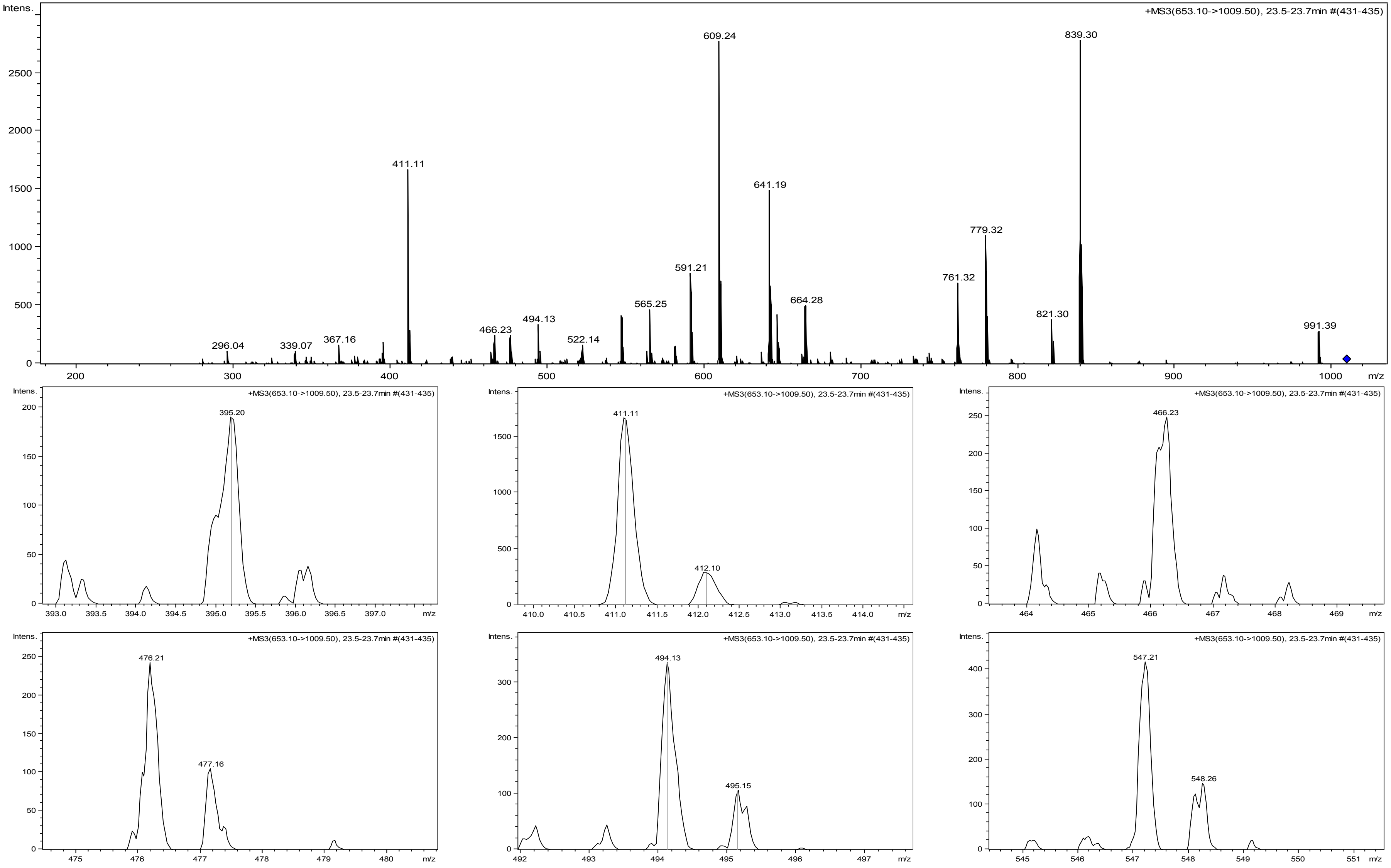

Fraction 17

652.93+++ → Pep [M+H]+ 1009.47+ [23.0-23.2 min] CID-MS3

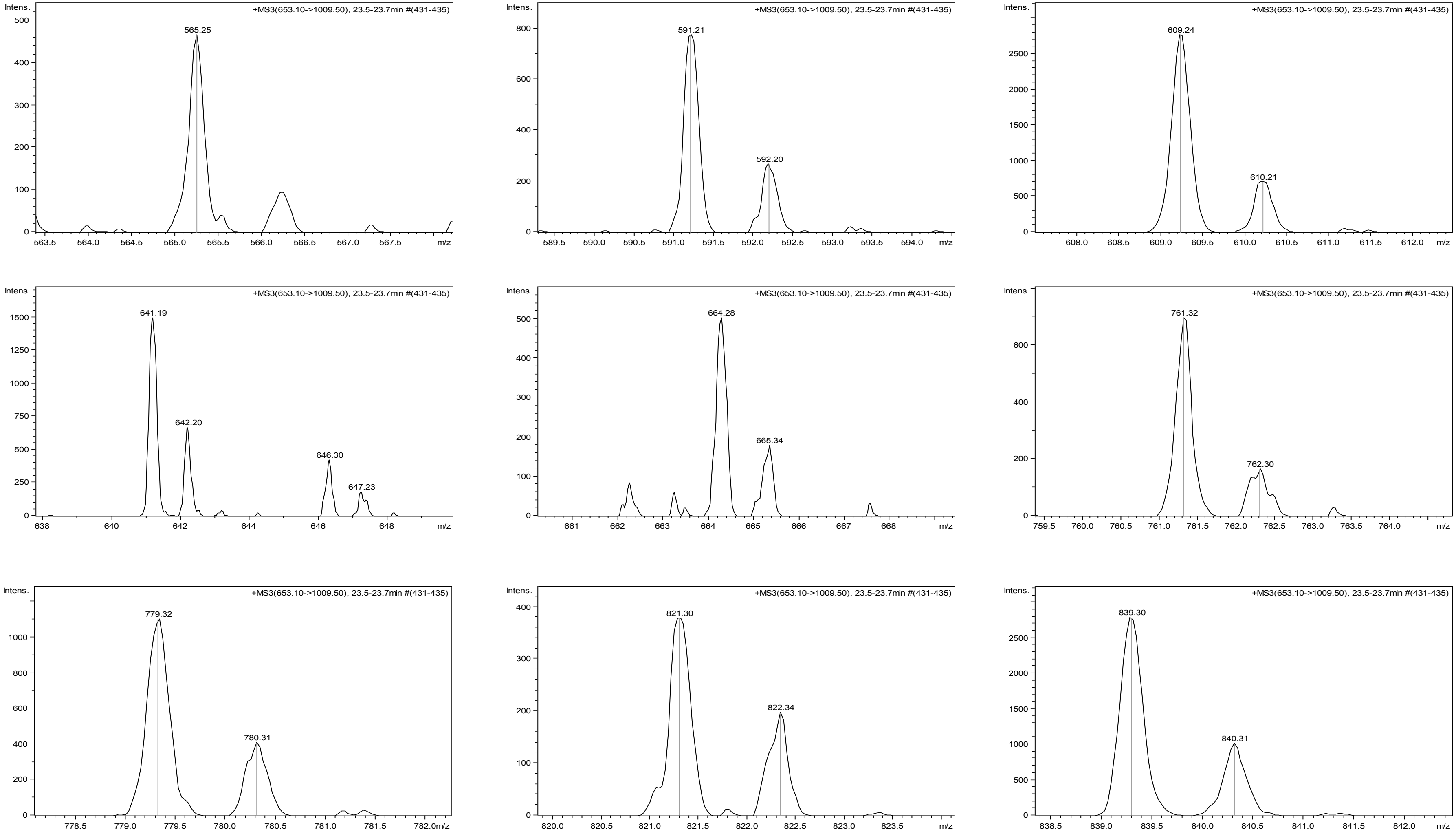

Fraction 17

652.93+++ → Pep [M+H]+ 1009.47+ [23.0-23.2 min] CID-MS3

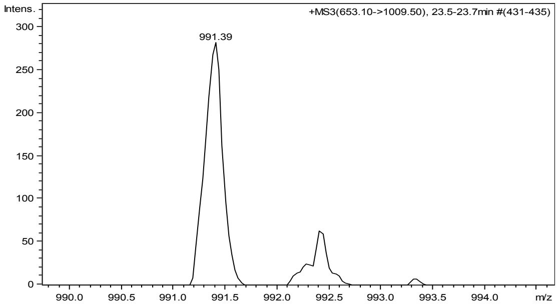

Fraction 17

652.93+++ → Pep [M+H]+ 1009.47+ [23.0-23.2 min] CID-MS3 MASCOT Search

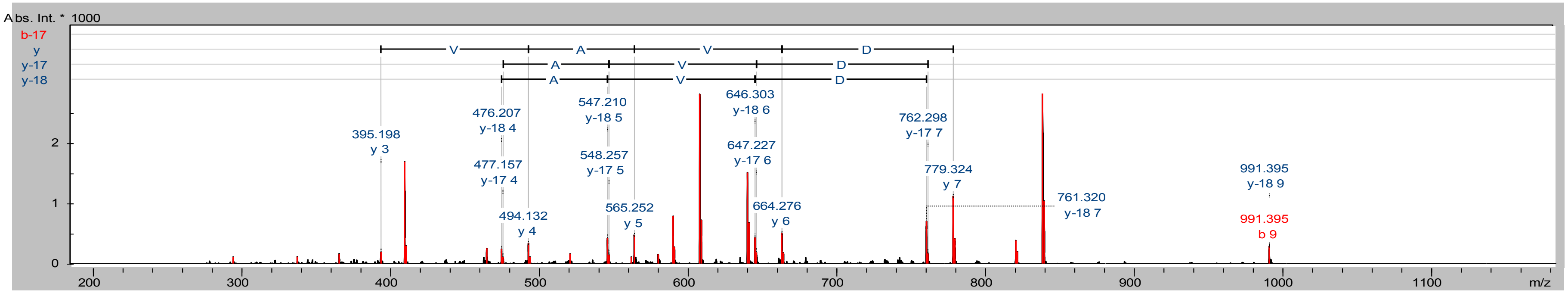

	D	N	D	V	A	V	T	N	C	Asp	Asn	Asp	Val	Ala	Val	Thr	Asn	Cys
Ion	1	2	3	4	5	6	7	8	9	1	2	3	4	5	6	7	8	9
b	D	N*	D	V	A	V	T	N*	C*	116.034	231.061	346.088	445.157	516.194	615.262	716.310	831.337	991.367
b-17	D	N*	D	V	A	V	T	N*	C*	-	214.035	329.062	428.130	499.167	598.235	699.283	814.310	974.341
b-18	D	N*	D	V	A	V	T	N*	C*	98.024	213.051	328.078	427.146	498.183	597.251	698.299	813.326	973.357
y	D	N*	D	V	A	V	T	N*	C*	179.048	294.075	395.123	494.192	565.229	664.297	779.324	894.351	1009.378
y-17	D	N*	D	V	A	V	T	N*	C*	-	277.049	378.097	477.165	548.202	647.271	762.297	877.324	992.351
y-18	D	N*	D	V	A	V	T	N*	C*	-	-	377.113	476.181	547.218	646.286	761.313	876.340	991.367
	9	8	7	6	5	4	3	2	1	Cys	Asn	Thr	Val	Ala	Val	Asp	Asn	Asp

N* deamidated (NQ)
C* Carbamidomethyl (C)

Some peptide fragment ions are also present in CID-MS²

There are major peaks that cannot be assigned to peptide fragment ions

unknown O-glycosylation site

Tenascin-R

$_{1282}$DNDVAV**T**NC$_{1290}$

Fraction 17

652.93+++ → Pep [M+H]+ 1009.47+ [23.0-23.2 min] CID-MS3 MASCOT Search

prot_hit_nur	prot_acc	prot_desc	prot_score	prot_mass	prot_matche	pep_query	pep_rank	pep_isbold	pep_exp_mz	pep_exp_mr	pep_exp_z	pep_calc_mr	pep_delta	pep_miss	pep_score	pep_expect	pep_res_bef	pep_seq
1	ZN426_HUM/	Zinc finger p	12	65004	1	1	4	1	1009.4748	1008.4675	1	1008.44	0.0275	0	16.72	3.10E+02	F	DDVAVDFTQ
2	SL9A1_HUM/	Sodium/hyd	12	91218	1	1	1	0	1009.4748	1008.4675	1	1008.6583	-0.1908	0	17.82	2.40E+02	F	PSLLVVVALV
3	TIF1G_HUMA	Transcriptior	10	124610	1	1	4	0	1009.4748	1008.4675	1	1008.5855	-0.118	0	16.72	3.10E+02	E	AEPPLTAVLV
4	TENR_HUMA	Tenascin-R p	10	151791	1	1	2	0	1009.4748	1008.4675	1	1007.3866	1.0809	0	17.67	2.50E+02	R	DNDVAVTNC
5	SYLM_HUMA	Probable leu	10	102823	1	1	8	0	1009.4748	1008.4675	1	1007.4668	1.0008	0	15.82	3.90E+02	C	LTPVMAVNN
6	PLXD1_HUM/	Plexin-D1 pr	9	215381	1	1	4	0	1009.4748	1008.4675	1	1008.5492	-0.0816	0	16.72	3.10E+02	V	EPAPDVVAV
7	EPHA5_HUM	Ephrin type-.	8	116706	1	1	10	0	1009.4748	1008.4675	1	1008.6947	-0.2272	0	14.73	5.00E+02	V	ILLAVVIGVL
8	NUP98_HUM	Nuclear pore	8	188643	1	1	9	0	1009.4748	1008.4675	1	1008.5128	-0.0452	0	15.11	4.60E+02	A	PQAPVALTDI
9	BSN_HUMAN	Protein bass	7	418354	1	1	7	0	1009.4748	1008.4675	1	1007.4117	1.0558	0	16.1	3.60E+02	G	QTPMPTTQS

BioTools-Score: 27

MASCOT-Score: 18

unknown O-glycosylation site

Tenascin-R (C-terminal domain of fibrinogen)

$_{1282}$DNDVAV**T**NC$_{1290}$

Fraction 17

652.93+++ → Pep [M+H]+ 1009.47+ [23.0-23.2 min] CID-MS2

Peptide fragment ions present in CID-MS²

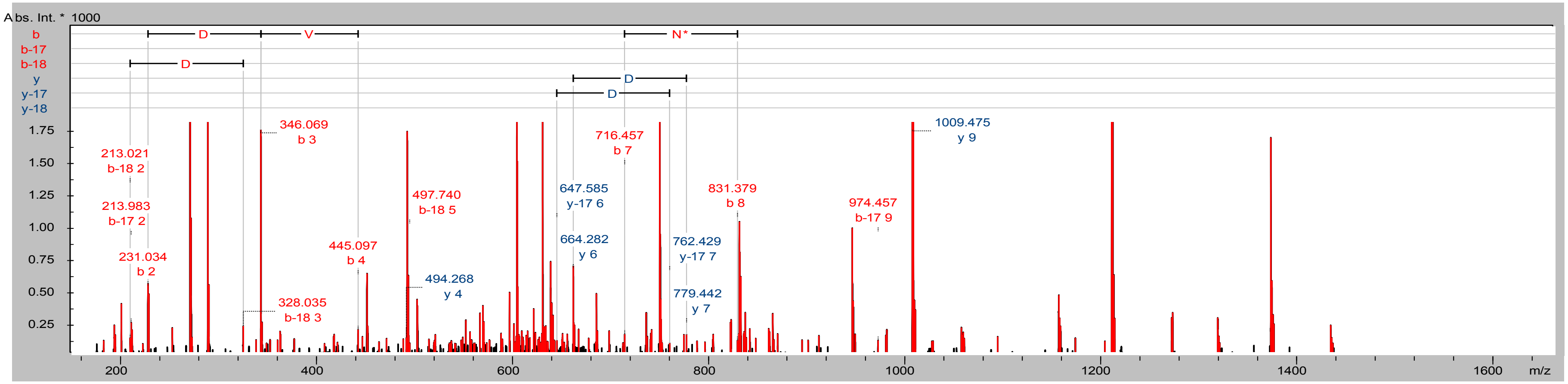

	D	N	D	V	A	V	T	N	C	Asp	Asn	Asp	Val	Ala	Val	Thr	Asn	Cys
Ion	1	2	3	4	5	6	7	8	9	1	2	3	4	5	6	7	8	9
b	D	N*	D	V	A	V	T	N*	C*	116.034	231.061	346.088	445.157	516.194	615.262	716.310	831.337	991.367
b-17	D	N*	D	V	A	V	T	N*	C*	-	214.035	329.062	428.130	499.167	598.235	699.283	814.310	974.341
b-18	D	N*	D	V	A	V	T	N*	C*	98.024	213.051	328.078	427.146	498.183	597.251	698.299	813.326	973.357
y	D	N*	D	V	A	V	T	N*	C*	179.048	294.075	395.123	494.192	565.229	664.297	779.324	894.351	1009.378
y-17	D	N*	D	V	A	V	T	N*	C*	-	277.049	378.097	477.165	548.202	647.271	762.297	877.324	992.351
y-18	D	N*	D	V	A	V	T	N*	C*	-	-	377.113	476.181	547.218	646.286	761.313	876.340	991.367
	9	8	7	6	5	4	3	2	1	Cys	Asn	Thr	Val	Ala	Val	Asp	Asn	Asp

Sequence matched to spectrum using BioTools

BioTools-Score: 36

Fraction 17

652.93+++ → Pep [M+H]+ 1009.47+ [23.0-23.2 min] ETD

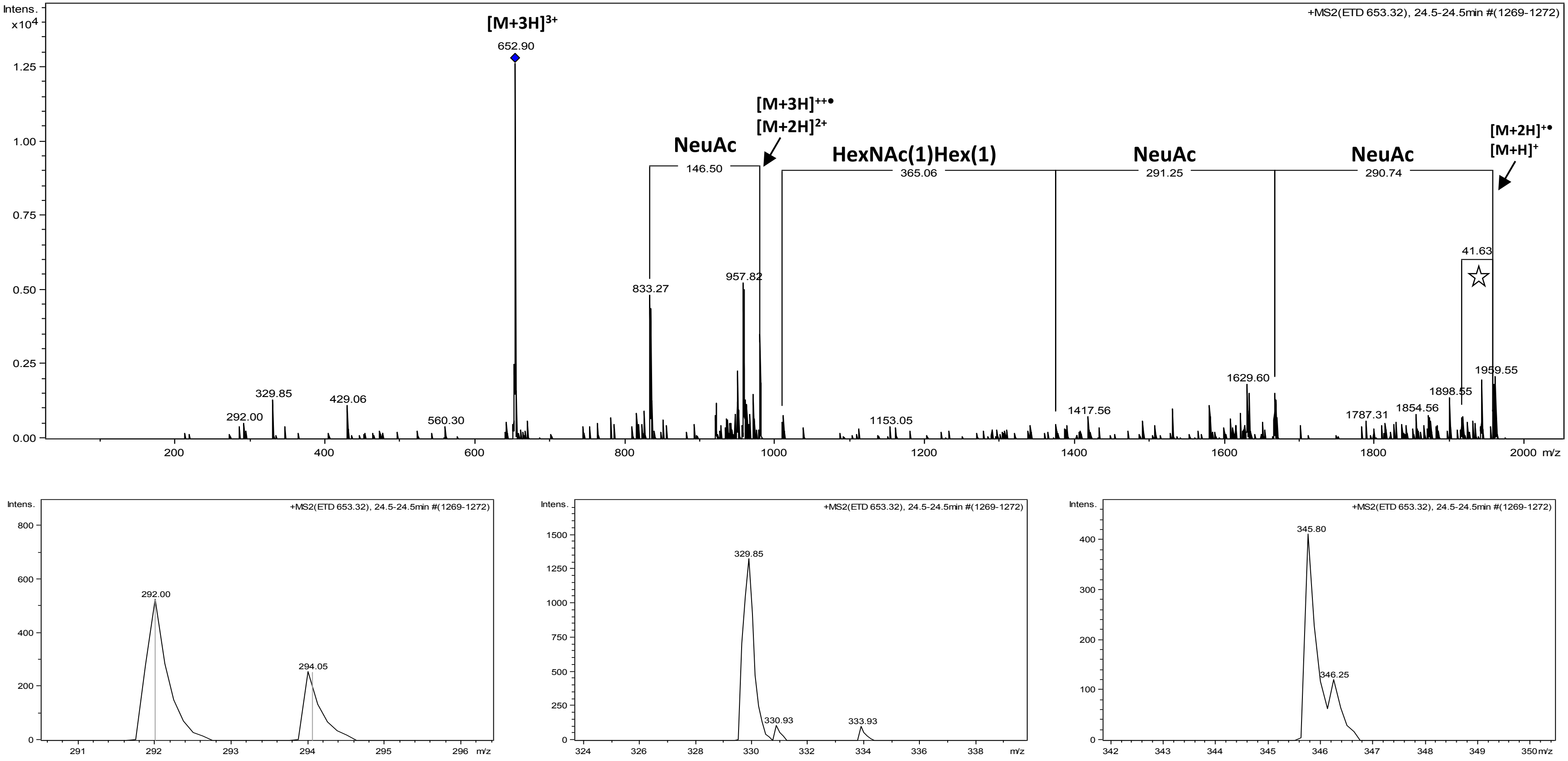

unknown O-glycosylation site

$_{1282}$DNDVAV**T**NC$_{1290}$

Tenascin-R (C-terminal domain of fibrinogen)

Fraction 17

652.93+++ → Pep [M+H]+ 1009.47+ [23.0-23.2 min]

ETD

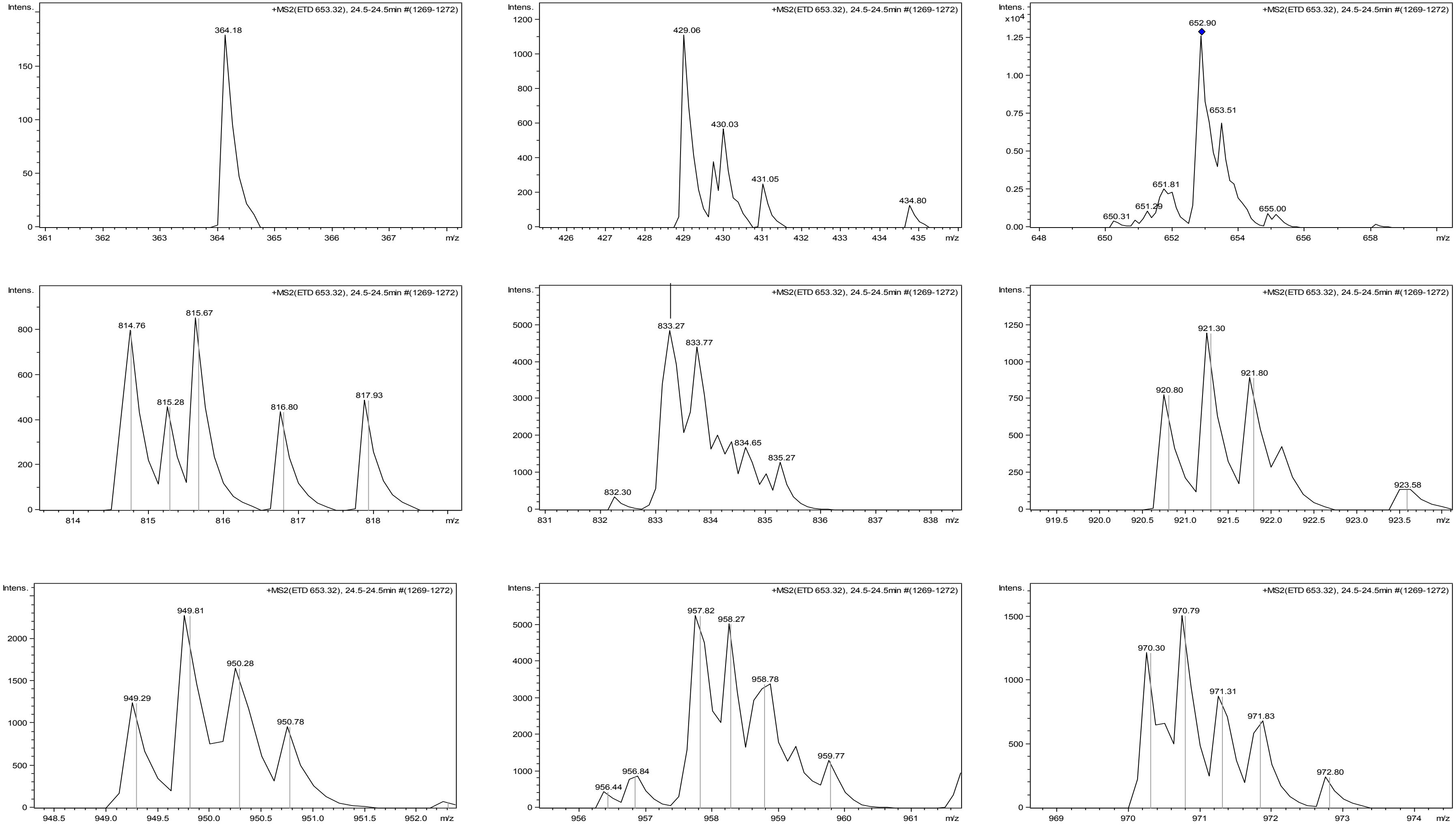

unknown O-glycosylation site

Tenascin-R (C-terminal domain of fibrinogen)

$_{1282}$DNDVAVTNC$_{1290}$

Fraction 17

652.93+++ → Pep [M+H]+ 1009.47+ [23.0-23.2 min]

ETD

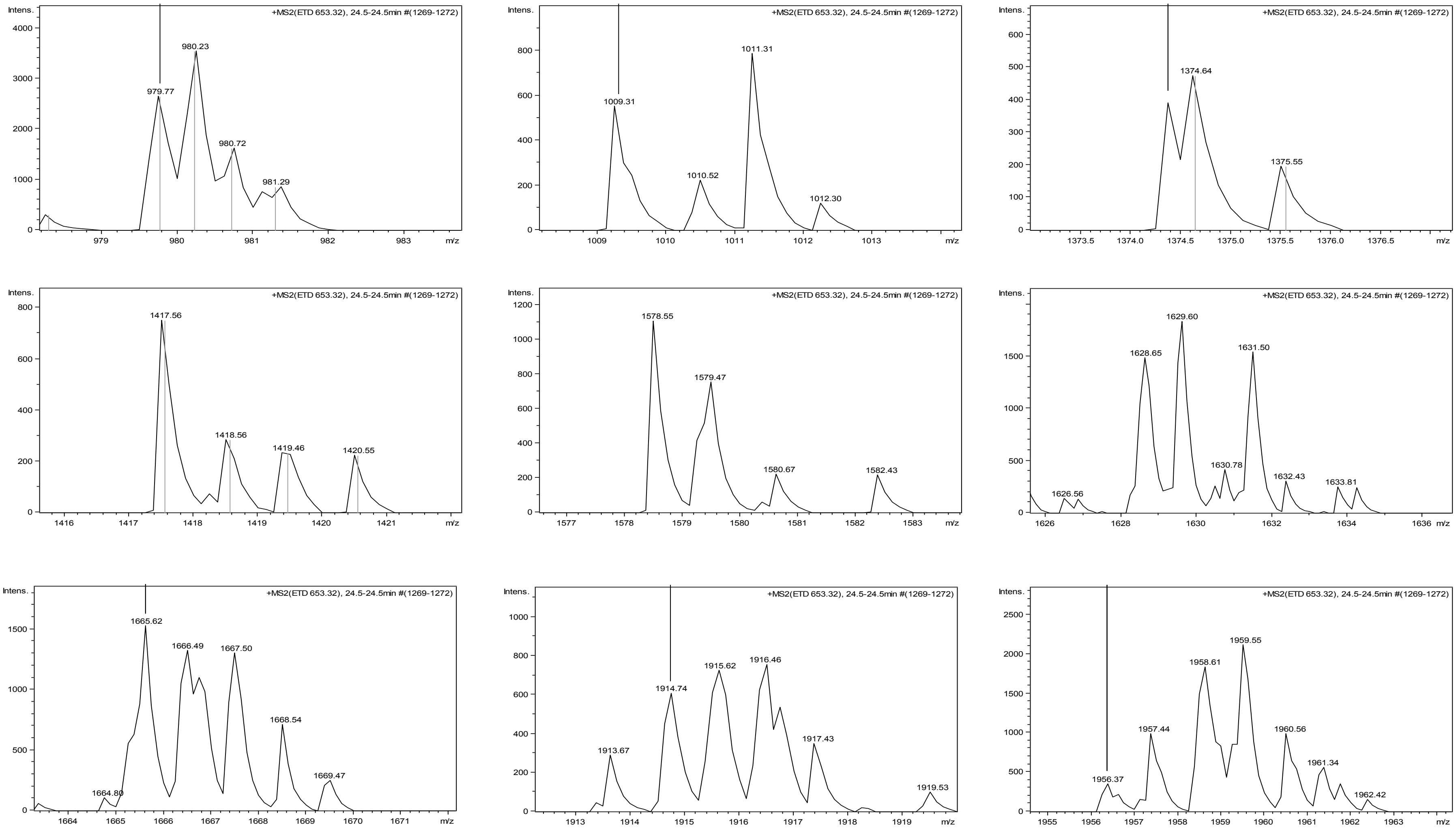

unknown O-glycosylation site
Tenascin-R (C-terminal domain of fibrinogen)

$_{1282}$DNDVAV**T**NC$_{1290}$

Fraction 17

652.93+++ → Pep [M+H]+ 1009.47+ [23.0-23.2 min] ETD

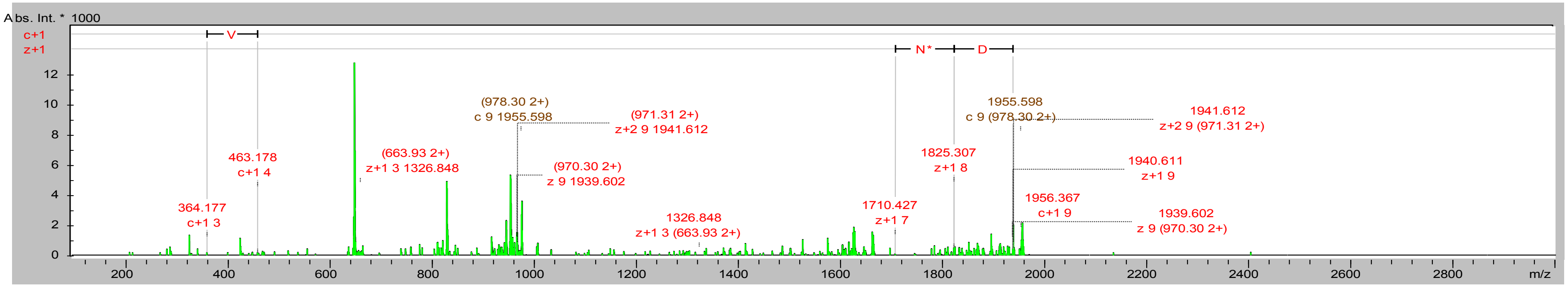

	D	N	D	V	A	V	T	N	C	Asp	Asn	Asp	Val	Ala	Val	Thr	Asn	Cys
Ion	1	2	3	4	5	6	7	8	9	1	2	3	4	5	6	7	8	9
c	D	N*	D	V	A	V	T*	N*	C*	133.061	248.088	363.115	462.183	533.220	632.289	1680.659	1795.686	1955.717
c+1	D	N*	D	V	A	V	T*	N*	C*	134.069	249.096	364.122	463.191	534.228	633.296	1681.667	1796.694	1956.725
z	D	N*	D	V	A	V	T*	N*	C*	162.022	277.049	1325.420	1424.488	1495.525	1594.594	1709.620	1824.647	1939.674
z+1	D	N*	D	V	A	V	T*	N*	C*	163.030	278.057	1326.427	1425.496	1496.533	1595.601	1710.628	1825.655	1940.682
z+2	D	N*	D	V	A	V	T*	N*	C*	164.038	279.065	1327.435	1426.504	1497.541	1596.609	1711.636	1826.663	1941.690
	9	8	7	6	5	4	3	2	1	Cys	Asn	Thr	Val	Ala	Val	Asp	Asn	Asp

N* deamidated (NQ)
C* Carbamidomethyl (C)

BioTools-Score: 15

unknown O-glycosylation site

Tenascin-R (C-terminal domain of fibrinogen) $_{1282}$DNDVAV**T**NC$_{1290}$

Fraction 17

681.25+++ → Pep+HexNAc [M+2H]++ 794.83++ [25.2-25.4 min]

CID-MS Precursor

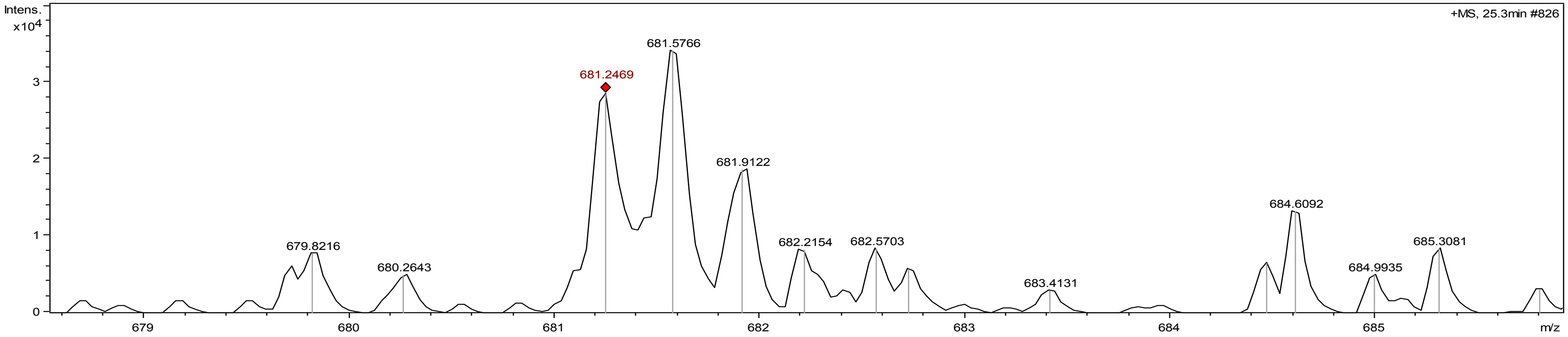

Fraction 17

681.25+++ → Pep+HexNAc [M+2H]++ 794.83++ [25.2-25.4 min]

CID-MS2

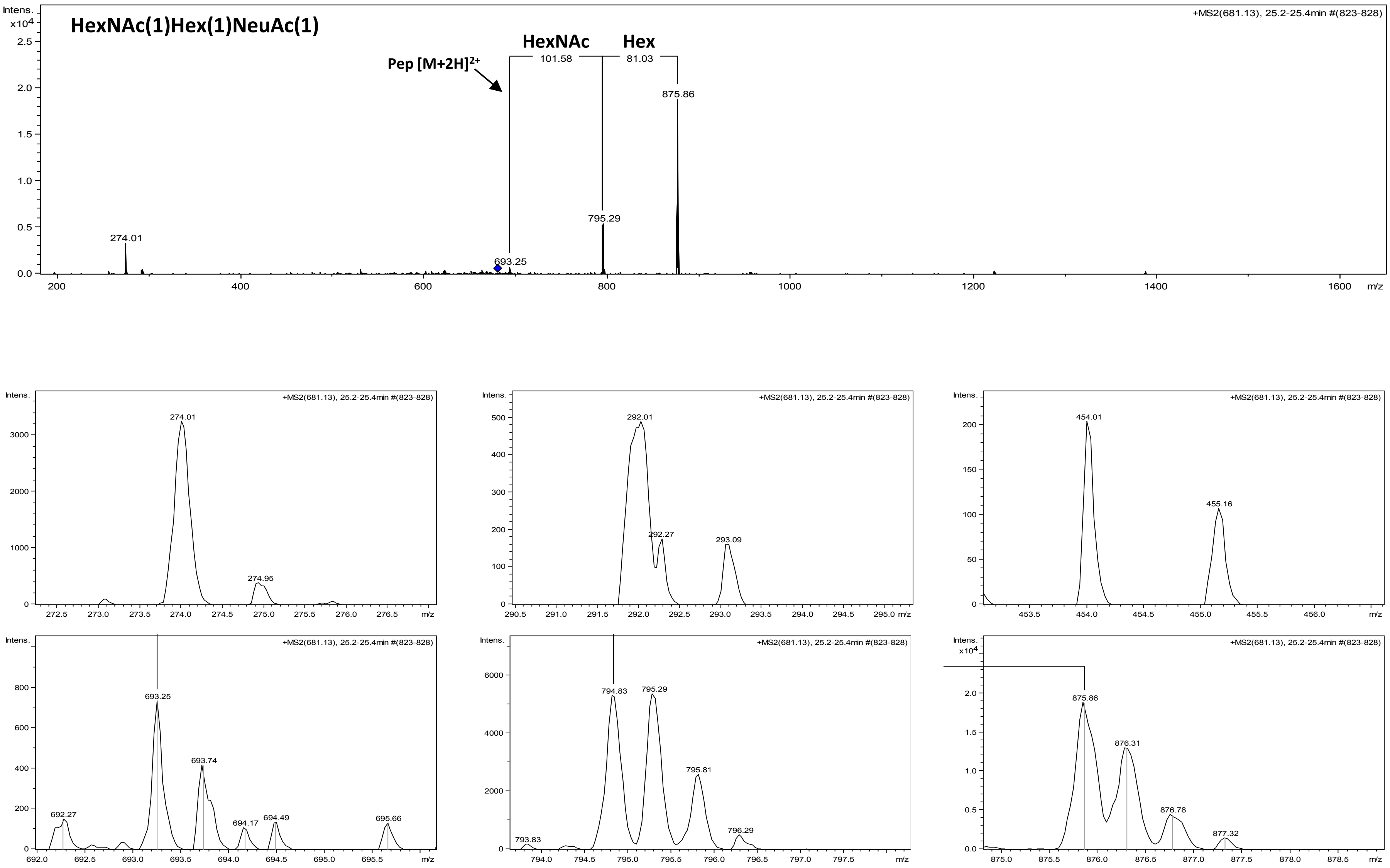

Fraction 17

681.25+++ → Pep+HexNAc [M+2H]++ 794.83++ [25.2-25.4 min]

CID-MS2

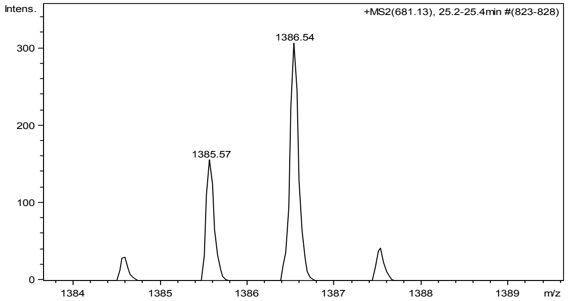

Fraction 17

681.25+++ → Pep+HexNAc [M+2H]++ 794.83++ [25.2-25.4 min]

CID-MS3

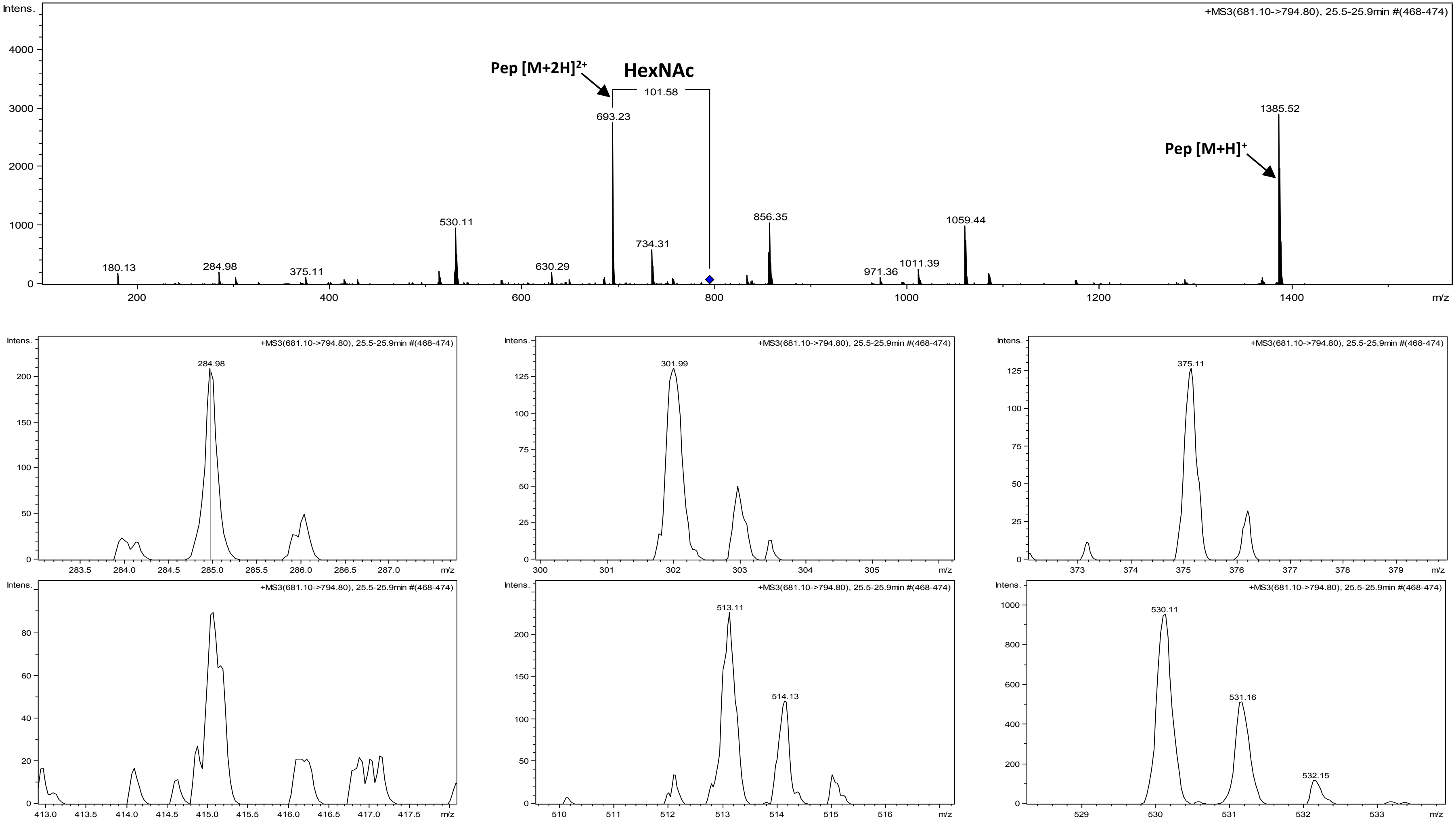

Fraction 17

681.25+++ → Pep+HexNAc [M+2H]++ 794.83++ [25.2-25.4 min]

CID-MS3

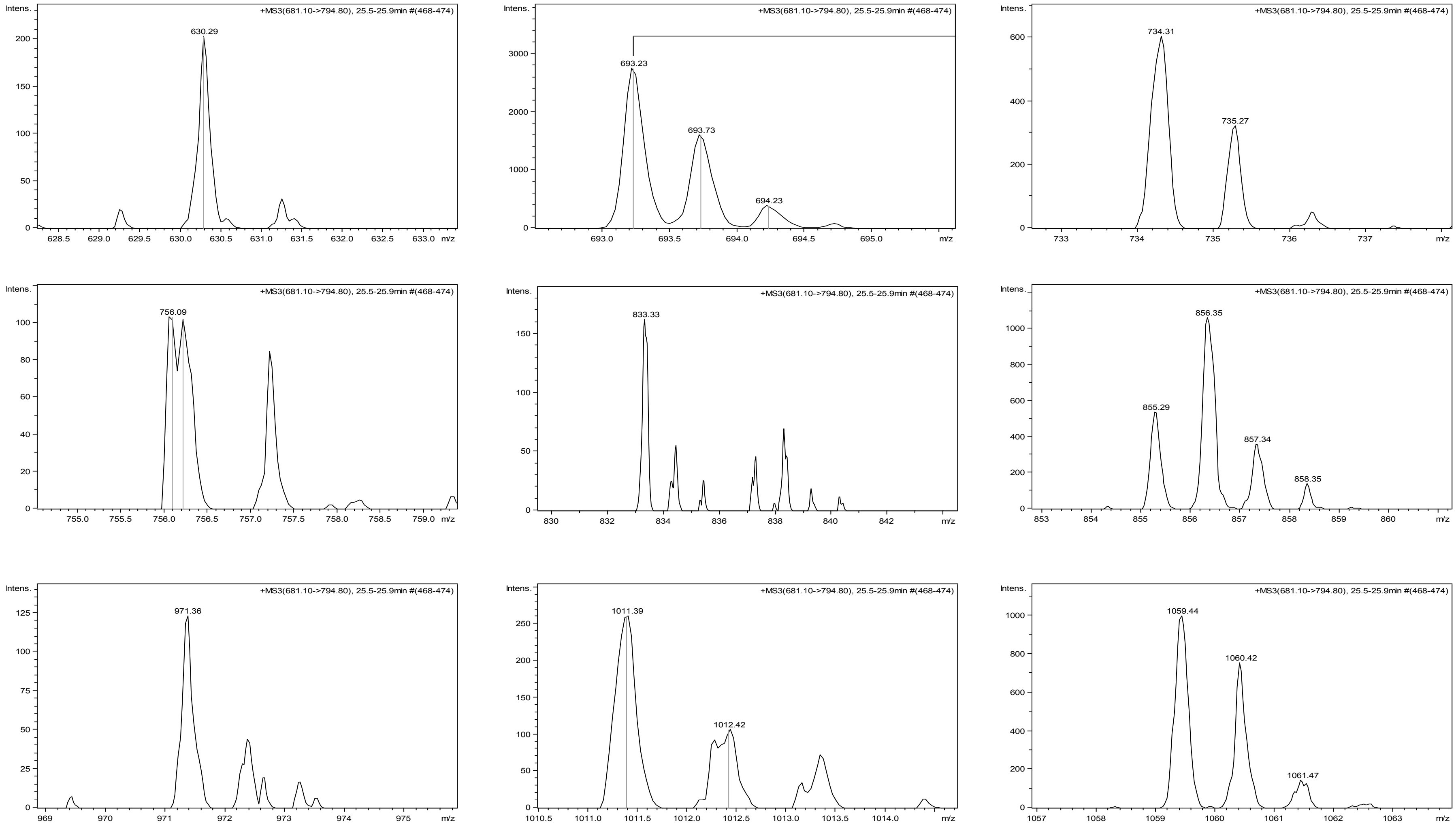

Fraction 17

681.25+++ → Pep+HexNAc [M+2H]++ 794.83++ [25.2-25.4 min]

CID-MS3

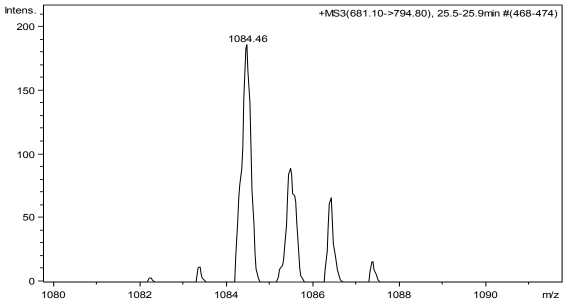

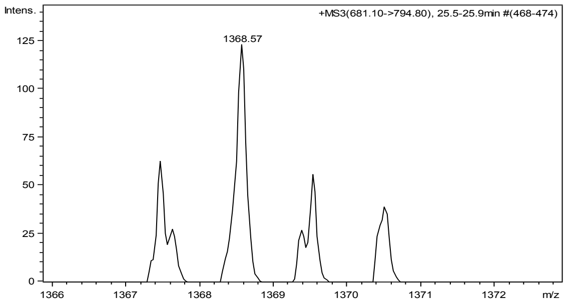

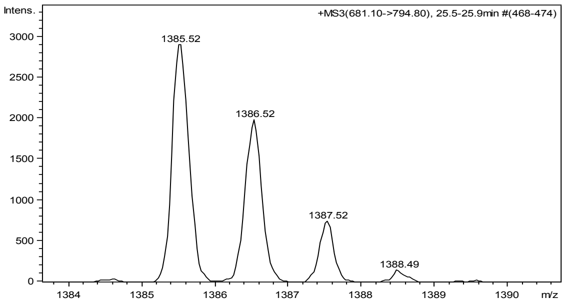

Fraction 17

681.25+++ → Pep+HexNAc [M+2H]++ 794.83++ [25.2-25.4 min]

CID-MS3 MASCOT Search

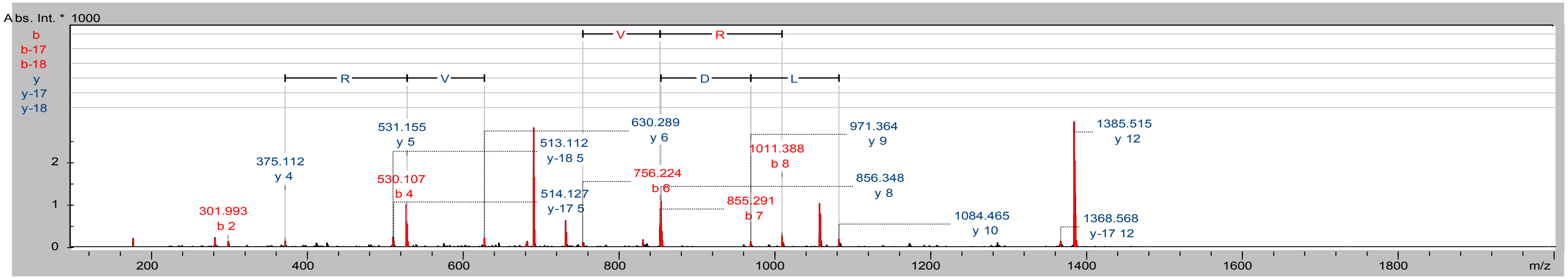

	W	D	L	D	P	E	V	R	P	T	S	A	Trp	Asp	Leu	Asp	Pro	Glu	Val	Arg	Pro	Thr	Ser	Ala
Ion	1	2	3	4	5	6	7	8	9	10	11	12	1	2	3	4	5	6	7	8	9	10	11	12
b	W	D	L	D	P	E	V	R	P	T	S	A	187.087	302.114	415.198	530.225	627.277	756.320	855.388	1011.489	1108.542	1209.590	1296.622	1367.659
b-17	W	D	L	D	P	E	V	R	P	T	S	A	-	-	-	-	-	-	-	994.463	1091.516	1192.563	1279.595	1350.632
b-18	W	D	L	D	P	E	V	R	P	T	S	A	-	284.103	397.187	512.214	609.267	738.309	837.378	993.479	1090.532	1191.579	1278.611	1349.648
y	W	D	L	D	P	E	V	R	P	T	S	A	90.055	177.087	278.135	375.187	531.289	630.357	759.400	856.452	971.479	1084.563	1199.590	1385.670
y-17	W	D	L	D	P	E	V	R	P	T	S	A	-	-	-	-	514.262	613.330	742.373	839.426	954.453	1067.537	1182.564	1368.643
y-18	W	D	L	D	P	E	V	R	P	T	S	A	-	159.076	260.124	357.177	513.278	612.346	741.389	838.442	953.469	1066.553	1181.580	1367.659
	12	11	10	9	8	7	6	5	4	3	2	1	Ala	Ser	Thr	Pro	Arg	Val	Glu	Pro	Asp	Leu	Asp	Trp

known O-glycosylation site

Apolipoprotein C-III

$_{85}$WDLDPEVRP**T**SA$_{96}$

Fraction 17

681.25+++ → Pep+HexNAc [M+2H]++ 794.83++ [25.2-25.4 min]

CID-MS3 MASCOT Search

prot_hit_nur	prot_acc	prot_desc	prot_score	prot_mass	prot_matche	pep_query	pep_rank	pep_isbold	pep_exp_mz	pep_exp_mr	pep_exp_z	pep_calc_mr	pep_delta	pep_miss	pep_score	pep_expect	pep_res_bef	pep_seq
1	APOC3_HUM	Apolipoprot	35	10846	1	1	1	1	1385.5025	1384.4952	1	1384.6623	-0.1671	0	34.93	5.5	F	WDLDPEVRP
2	RIMS1_HUM	Regulating sy	21	190154	1	1	2	0	1385.5025	1384.4952	1	1383.6347	0.8606	0	27.84	28	E	KGDLDYYWLI
3	NMUR2_HUN	Neuromedin	14	48208	1	1	3	0	1385.5025	1384.4952	1	1384.6511	-0.1558	0	19.84	1.80E+02	C	HFVELTEDIGF
4	DPOE1_HUM	DNA polyme	11	264031	1	1	4	0	1385.5025	1384.4952	1	1384.6663	-0.1711	0	19.33	2.00E+02	I	LDWDYYIERL
5	PCD17_HUM	Protocadher	9	127405	1	1	5	0	1385.5025	1384.4952	1	1383.5864	0.9089	0	16.1	4.20E+02	V	KGNLDYEENC
6	KCNA6_HUM	Potassium vo	9	59148	1	1	8	0	1385.5025	1384.4952	1	1384.7748	-0.2796	0	14.18	6.50E+02	F	RNIMNIIDLV
7	VPP3_HUMA	Vacuolar pro	8	93680	1	1	8	0	1385.5025	1384.4952	1	1384.6722	-0.1769	0	14.18	6.50E+02	Q	EENKAGLLDL
8	RIPK5_HUM	Receptor-int	8	106617	1	1	8	0	1385.5025	1384.4952	1	1384.7748	-0.2796	0	14.18	6.50E+02	C	NVKCQLLNLL
9	MPP4_HUM	MAGUK p55 s	7	73561	1	1	6	0	1385.5025	1384.4952	1	1384.7133	-0.218	0	14.19	6.50E+02	I	KRHEMTGDIL
10	TCF20_HUM	Transcriptior	5	213123	1	1	6	0	1385.5025	1384.4952	1	1384.7674	-0.2722	0	14.19	6.50E+02	E	KRKGEVASDI

BioTools-Score: 21

MASCOT-Score: 35

known O-glycosylation site

Apolipoprotein C-III

$_{85}$WDLDPEVRP**T**SA$_{96}$

Fraction 17

681.25+++ → Pep+HexNAc [M+2H]++ 794.83++ [25.2-25.4 min]

ETD

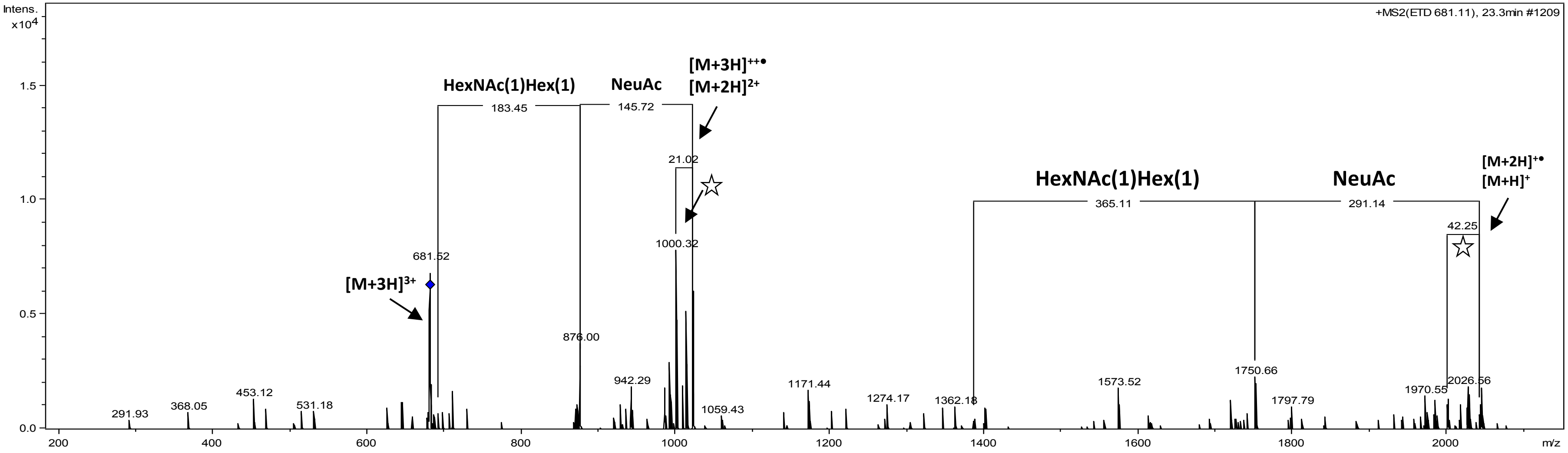

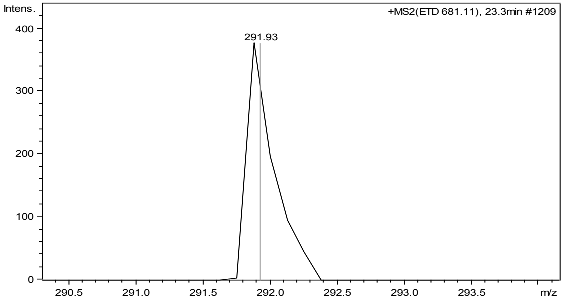

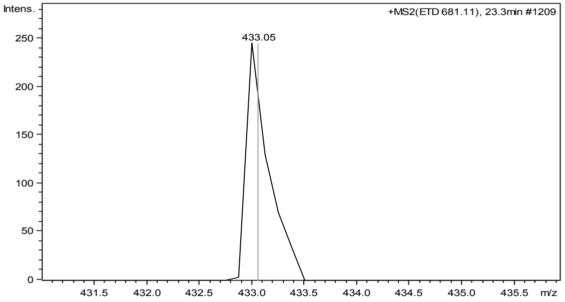

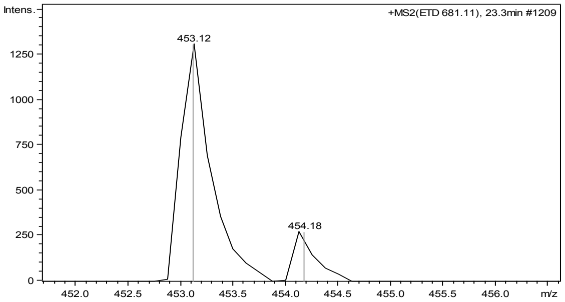

known O-glycosylation site

Apolipoprotein C-III

$_{85}$WDLDPEVRPTSA$_{96}$

Fraction 17

681.25+++ → Pep+HexNAc [M+2H]++ 794.83++ [25.2-25.4 min]

ETD

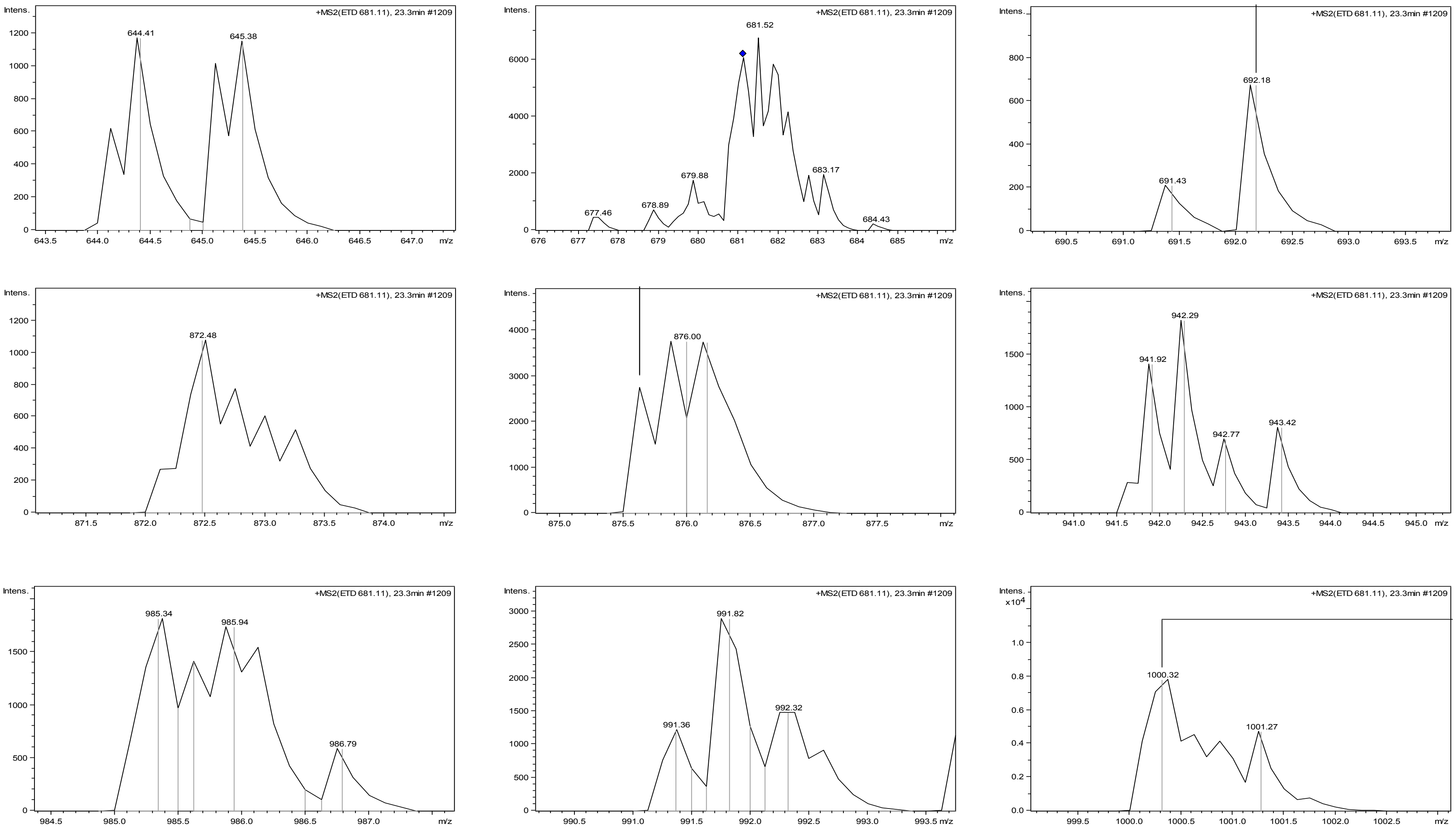

known O-glycosylation site
Apolipoprotein C-III

$_{85}$WDLDPEVRP**T**SA$_{96}$

Fraction 17

681.25+++ → Pep+HexNAc [M+2H]++ 794.83++ [25.2-25.4 min]

ETD

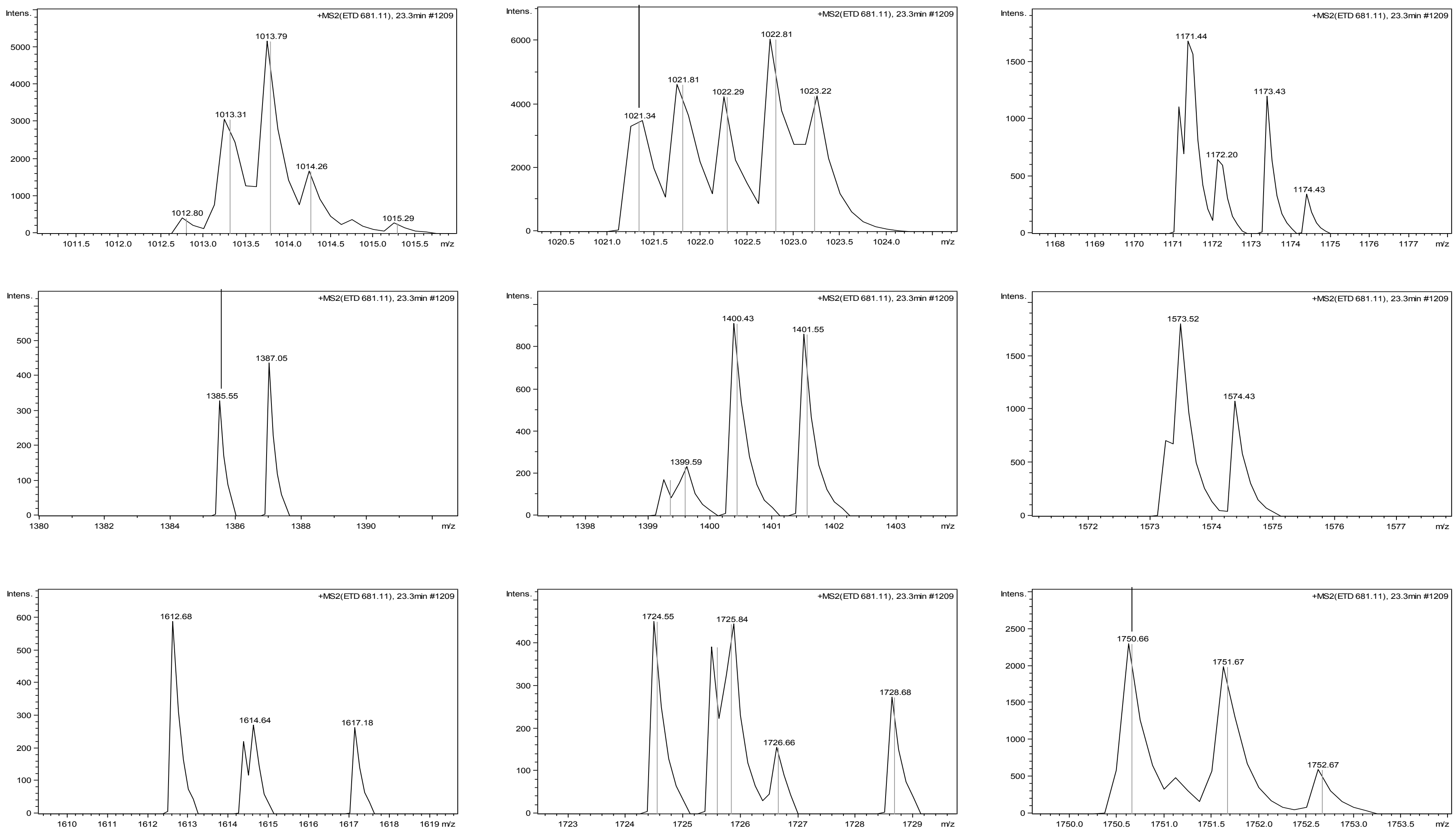

known O-glycosylation site
Apolipoprotein C-III

$_{85}$WDLDPEVRP**T**SA$_{96}$

Fraction 17

681.25+++ → Pep+HexNAc [M+2H]++ 794.83++ [25.2-25.4 min]

ETD

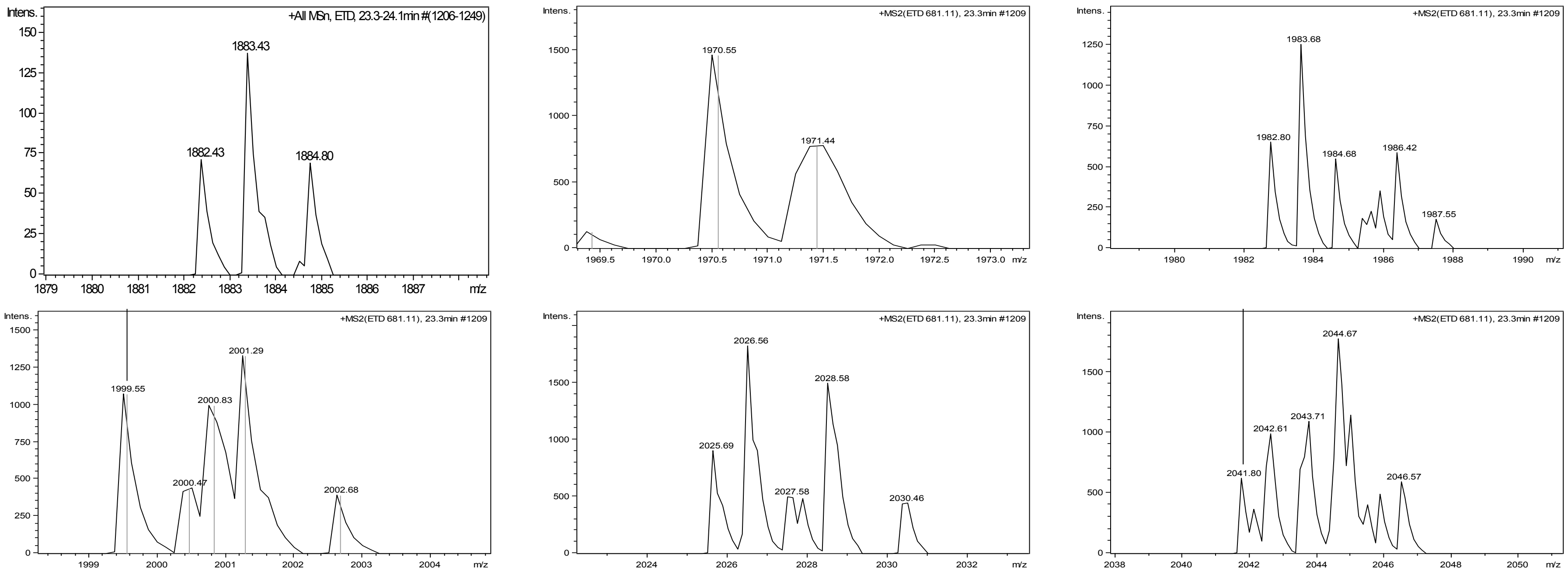

known O-glycosylation site
Apolipoprotein C-III

$_{85}$WDLDPEVRPTSA$_{96}$

Fraction 17

681.25+++ → Pep+HexNAc [M+2H]++ 794.83++ [25.2-25.4 min]

ETD

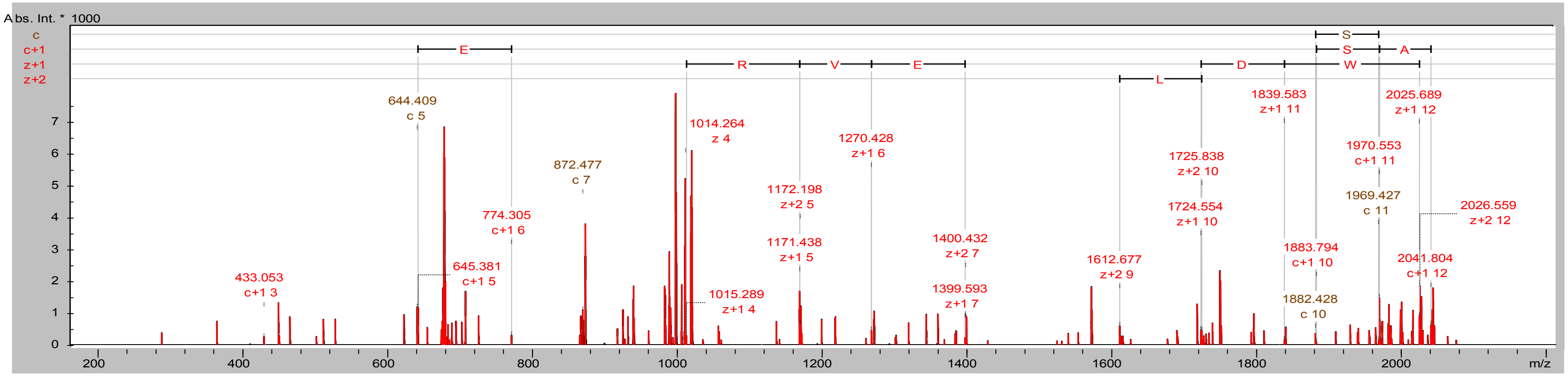

WDLDPEVRP**T**SA

	W	D	L	D	P	E	V	R	P	T	S	A	Trp	Asp	Leu	Asp	Pro	Glu	Val	Arg	Pro	Thr	Ser	Ala
Ion	1	2	3	4	5	6	7	8	9	10	11	12	1	2	3	4	5	6	7	8	9	10	11	12
c	W	D	L	D	P	E	V	R	P	T*	S	A	204.113	319.140	432.224	547.251	644.304	773.346	872.415	1028.516	1125.569	1882.844	1969.876	2040.913
c+1	W	D	L	D	P	E	V	R	P	T*	S	A	205.121	320.148	433.232	548.259	645.312	774.354	873.423	1029.524	1126.577	1883.852	1970.884	2041.921
z	W	D	L	D	P	E	V	R	P	T*	S	A	73.028	160.060	917.336	1014.388	1170.490	1269.558	1398.601	1495.653	1610.680	1723.764	1838.791	2024.871
z+1	W	D	L	D	P	E	V	R	P	T*	S	A	74.036	161.068	918.344	1015.396	1171.497	1270.566	1399.608	1496.661	1611.688	1724.772	1839.799	2025.878
z+2	W	D	L	D	P	E	V	R	P	T*	S	A	75.044	162.076	919.351	1016.404	1172.505	1271.574	1400.616	1497.669	1612.696	1725.780	1840.807	2026.886
	12	11	10	9	8	7	6	5	4	3	2	1	Ala	Ser	Thr	Pro	Arg	Val	Glu	Pro	Asp	Leu	Asp	Trp

BioTools-Score: 46

known O-glycosylation site

Apolipoprotein C-III

$_{85}$WDLDPEVRP**T**SA$_{96}$

Fraction 17

681.25+++ → Pep+HexNAc [M+2H]++ 794.83++ [25.2-25.4 min]

ETD

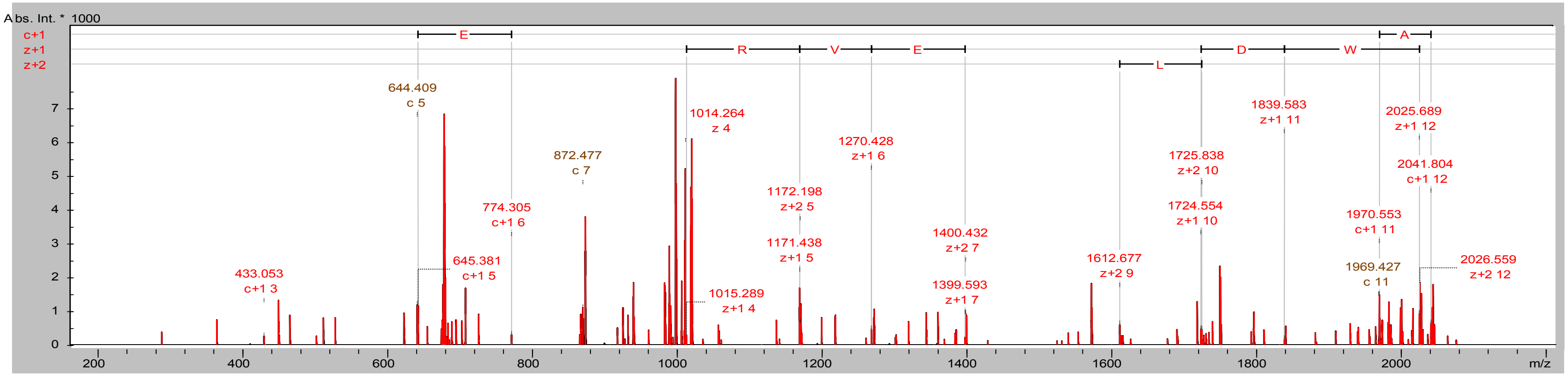

WDLDPEVRPTSA

	W	D	L	D	P	E	V	R	P	T	S	A	Trp	Asp	Leu	Asp	Pro	Glu	Val	Arg	Pro	Thr	Ser	Ala
Ion	1	2	3	4	5	6	7	8	9	10	11	12	1	2	3	4	5	6	7	8	9	10	11	12
c	W	D	L	D	P	E	V	R	P	T	S*	A	204.113	319.140	432.224	547.251	644.304	773.346	872.415	1028.516	1125.569	1226.616	1969.876	2040.913
c+1	W	D	L	D	P	E	V	R	P	T	S*	A	205.121	320.148	433.232	548.259	645.312	774.354	873.423	1029.524	1126.577	1227.624	1970.884	2041.921
z	W	D	L	D	P	E	V	R	P	T	S*	A	73.028	816.288	917.336	1014.388	1170.490	1269.558	1398.601	1495.653	1610.680	1723.764	1838.791	2024.871
z+1	W	D	L	D	P	E	V	R	P	T	S*	A	74.036	817.296	918.344	1015.396	1171.497	1270.566	1399.608	1496.661	1611.688	1724.772	1839.799	2025.878
z+2	W	D	L	D	P	E	V	R	P	T	S*	A	75.044	818.304	919.351	1016.404	1172.505	1271.574	1400.616	1497.669	1612.696	1725.780	1840.807	2026.886
	12	11	10	9	8	7	6	5	4	3	2	1	Ala	Ser	Thr	Pro	Arg	Val	Glu	Pro	Asp	Leu	Asp	Trp

BioTools-Score: 40

known O-glycosylation site

Apolipoprotein C-III

$_{85}$WDLDPEVRPTSA$_{96}$

Fraction 17

872.72+++ → Pep [M+H]+ 1668.75+ [31.9-32.0 min]

CID-MS Precursor

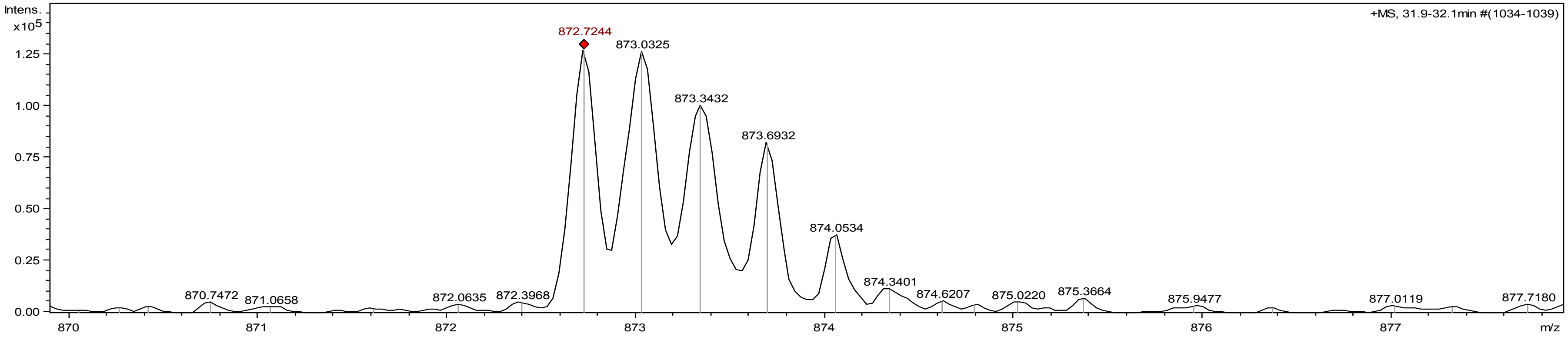

ETD spectrum of poor quality

Fraction 17

872.72+++ → Pep [M+H]+ 1668.75+ [31.9-32.0 min]

CID-MS2

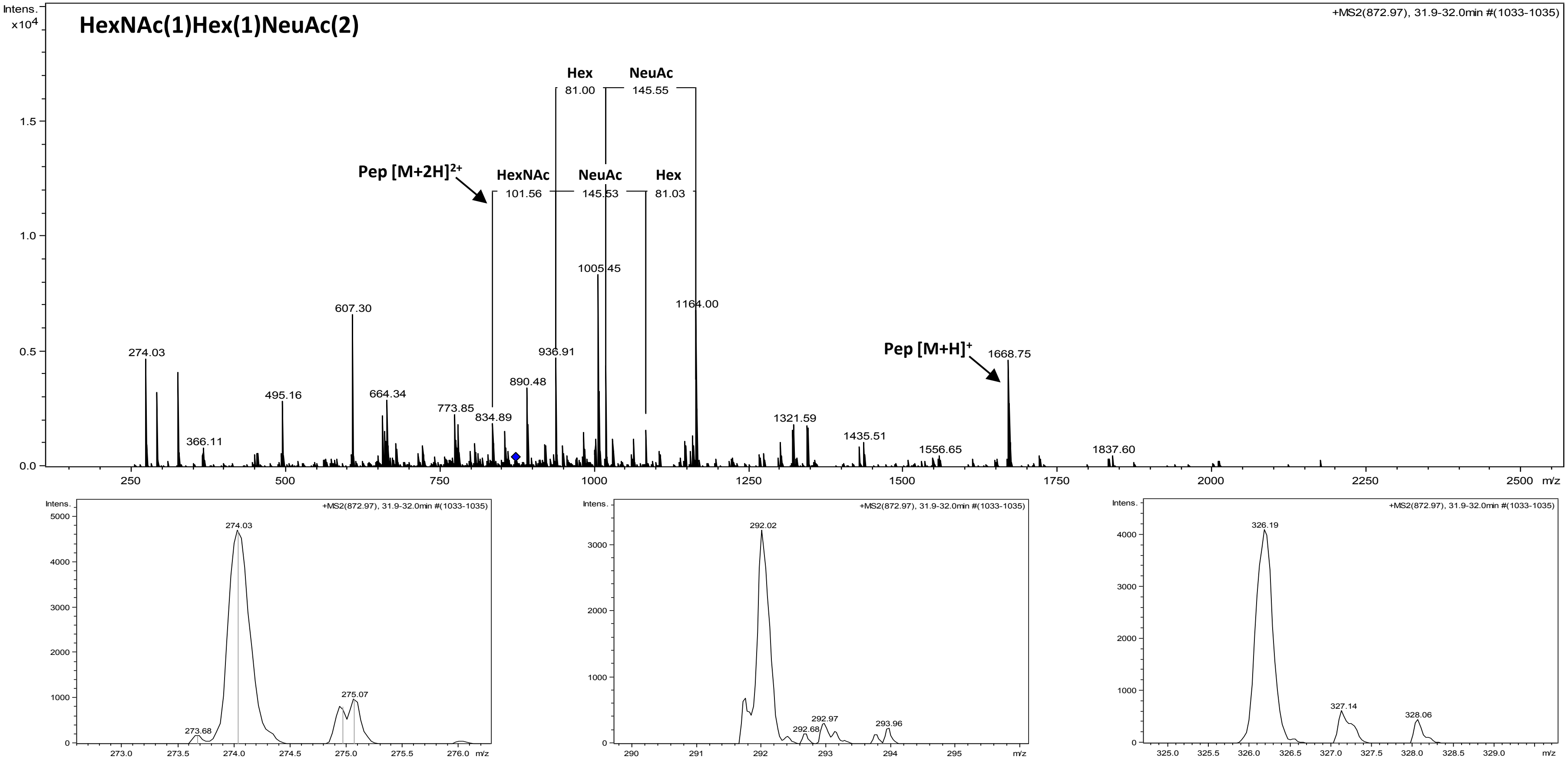

Fraction 17

872.72+++ → Pep [M+H]+ 1668.75+ [31.9-32.0 min]

CID-MS2

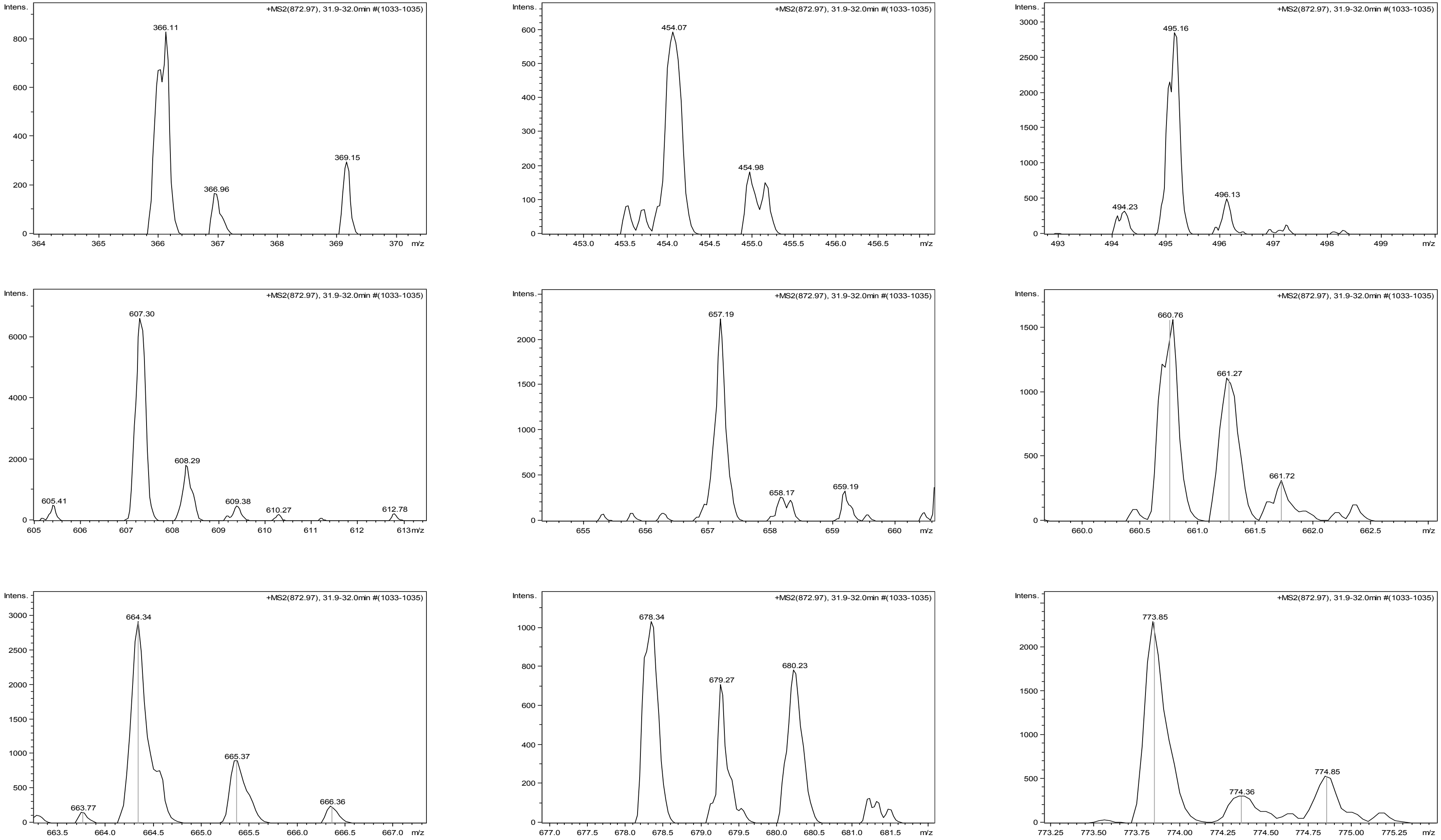

Fraction 17

872.72+++ → Pep [M+H]+ 1668.75+ [31.9-32.0 min]

CID-MS2

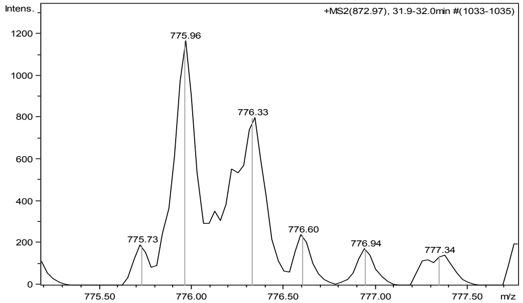

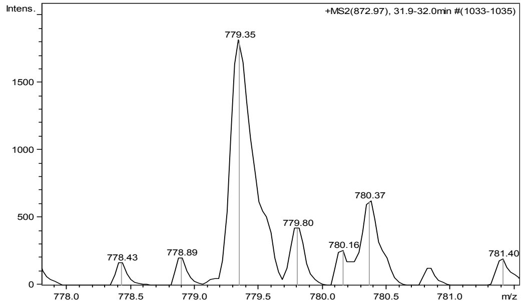

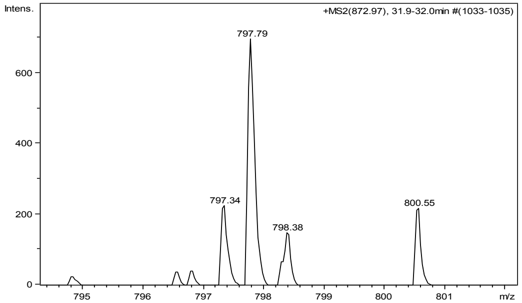

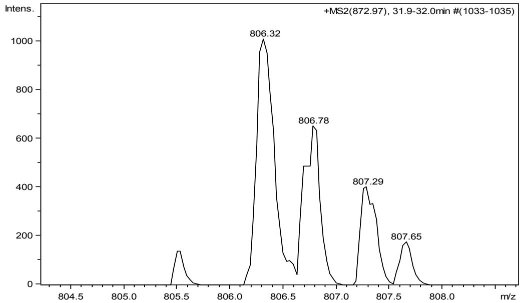

Fraction 17

872.72+++ → Pep [M+H]+ 1668.75+ [31.9-32.0 min]

CID-MS2 MASCOT Search

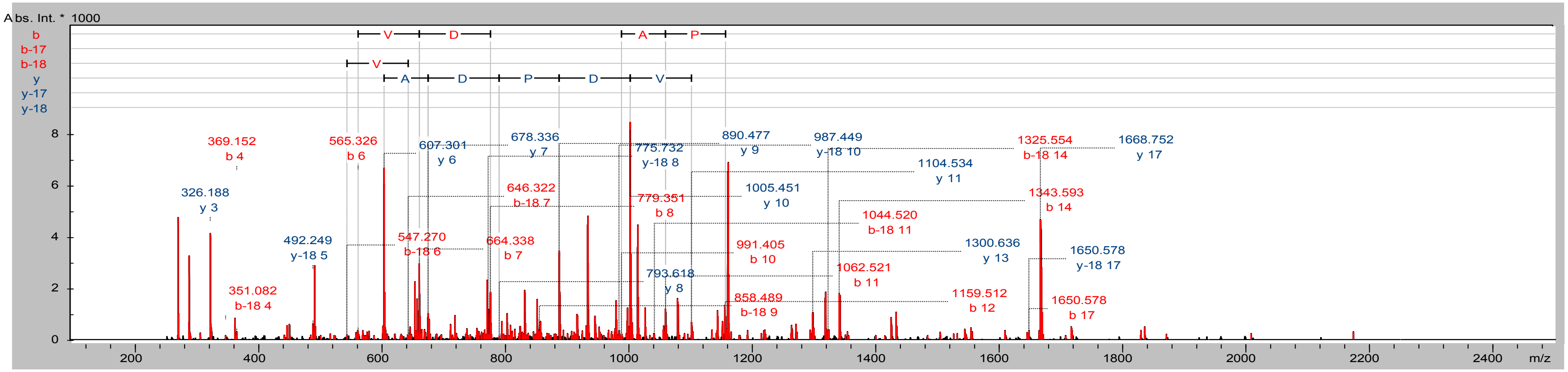

	A	V	P	T	P	V	V	D	P	D	A	P	P	S	P	P	L
Ion	1	2	3	4	5	6	7	8	9	10	11	12	13	14	15	16	17
b	A	V	P	T	P	V	V	D	P	D	A	P	P	S	P	P	L
b-17	A	V	P	T	P	V	V	D	P	D	A	P	P	S	P	P	L
b-18	A	V	P	T	P	V	V	D	P	D	A	P	P	S	P	P	L
y	A	V	P	T	P	V	V	D	P	D	A	P	P	S	P	P	L
y-17	A	V	P	T	P	V	V	D	P	D	A	P	P	S	P	P	L
y-18	A	V	P	T	P	V	V	D	P	D	A	P	P	S	P	P	L
	17	16	15	14	13	12	11	10	9	8	7	6	5	4	3	2	1

	Ala	Val	Pro	Thr	Pro	Val	Val	Asp	Pro	Asp	Ala	Pro	Pro	Ser	Pro	Pro	Leu
Ion	1	2	3	4	5	6	7	8	9	10	11	12	13	14	15	16	17
b	72.044	171.113	268.166	369.213	466.266	565.334	664.403	779.430	876.483	991.509	1062.547	1159.599	1256.652	1343.684	1440.737	1537.790	1650.874
b-17	-	-	-	-	-	-	-	-	-	-	-	-	-	-	-	-	-
b-18	-	-	-	351.203	448.255	547.324	646.392	761.419	858.472	973.499	1044.536	1141.589	1238.642	1325.674	1422.726	1519.779	1632.863
y	132.102	229.155	326.207	413.239	510.292	607.345	678.382	793.409	890.462	1005.489	1104.557	1203.626	1300.678	1401.726	1498.779	1597.847	1668.884
y-17	-	-	-	-	-	-	-	-	-	-	-	-	-	-	-	-	-
y-18	-	-	-	395.229	492.282	589.334	660.372	775.398	872.451	987.478	1086.547	1185.615	1282.668	1383.715	1480.768	1579.837	1650.874
	Leu	Pro	Pro	Ser	Pro	Pro	Ala	Asp	Pro	Asp	Val	Val	Pro	Thr	Pro	Val	Ala

For MASCOT search m/z of the unmodified peptide $[M+H]^+$ has to be given

known O-glycosylation site

Alpha-2-HS-glycoprotein precursor

$_{267}$AVP**T**PVVDPDAPPSPPL$_{283}$

Fraction 17

872.72+++ → Pep [M+H]+ 1668.75+ [31.9-32.0 min]

CID-MS2 MASCOT Search

prot_hit_nur	prot_acc	prot_desc	prot_score	prot_mass	prot_matche	prot_matche	prot_sequen	prot_sequen	pep_query	pep_rank	pep_isbold	pep_isuniqu	pep_exp_mz	pep_exp_mr	pep_exp_z	pep_calc_mr	pep_delta	pep_miss	pep_score	pep_expect	pep_res_bef	pep_seq
1	TGON2_HUMAN		2	51082	1	0	1	0	1	2	1	1	1668.75	1667.7427	1	1667.6911	0.0517	0	7.19	5.20E+03	T	GSEKDDLYPN
2	SENP3_HUMAN		1	65596	1	0	1	0	1	3	0	1	1668.75	1667.7427	1	1667.7759	-0.0332	0	7.1	5.30E+03	L	IQSYQRMPGI
3	AOC2_HUMA	Retina-speci	1	84077	1	0	1	0	1	1	0	1	1668.75	1667.7427	1	1667.8043	-0.0615	0	7.64	4.70E+03	S	QYSVQGNLV
4	FETUA_HUMAN		0	40098	1	0	1	0	1	7	0	1	1668.75	1667.7427	1	1667.8771	-0.1343	0	4.8	9.00E+03	E	AVPTPVVDP
5	QRFPR_HUMAN		0	50082	1	0	1	0	1	10	0	1	1668.75	1667.7427	1	1667.7348	0.0079	0	1.75	1.80E+04	K	GEAFSDGNIE
6	LRIG2_HUMAN		0	120315	1	0	1	0	1	8	0	1	1668.75	1667.7427	1	1667.7131	0.0297	0	4.55	9.50E+03	S	CMAQNTAG
7	MYO6_HUMAN		0	150965	1	0	1	0	1	9	0	1	1668.75	1667.7427	1	1667.9107	-0.168	0	2.23	1.60E+04	A	IKSYQGKSLG
8	AKAP9_HUMAN		0	455725	1	0	1	0	1	6	0	1	1668.75	1667.7427	1	1667.7494	-0.0067	0	5.2	8.20E+03	I	SVSSMDASR
9	SIG10_HUMAN		0	77456	1	0	1	0	1	4	0	1	1668.75	1667.7427	1	1667.7726	-0.0299	0	6.07	6.70E+03	G	GSQAMDGRI

BioTools-Score: 93

MASCOT-Score: 5

known O-glycosylation site

Alpha-2-HS-glycoprotein precursor

$_{267}$AVP**T**PVVDPDAPPSPPL$_{283}$

Fraction 17

872.72+++ → Pep [M+H]+ 1668.75+ [31.9-32.0 min]

Internal glycopeptide fragmentation

$_{267}$AVP**T**PVVDPDAPPSPPL$_{283}$

HexNac-

CID-MS2 MASCOT Search

b				y
---	1	**A**	17	---
171.11	2	**V**	16	1800.93
268.17	3	**P**	15	1701.86
572.29	4	**T(HexNac)**	14	1604.81
669.35	5	**P**	13	1300.68
768.41	6	**V**	12	1203.63
867.48	7	**V**	11	1104.56
**982.51**	8	**D**	10	1005.49
1079.56	9	**P**	9	890.46
1194.59	10	**D**	8	793.41
1265.63	11	**A**	7	678.38
1362.68	12	**P**	6	607.35
1459.73	13	**P**	5	510.29
**1546.76**	14	**S**	4	413.24
1643.82	15	**P**	3	326.21
1740.87	16	**P**	2	229.15
---	17	**L**	1	132.10

These fragment ions are not present in the MS³ peptide spectrum. This indicates that they are derived from glycopeptide fragmentation.

known O-glycosylation site

Alpha-2-HS-glycoprotein precursor

$_{267}$AVP**T**PVVDPDAPPSPPL$_{283}$

Fraction 17

872.72+++ → Pep [M+H]+ 1668.75+ [31.9-32.0 min]

CID-MS2 MASCOT Search

Internal glycopeptide fragmentation

$_{267}$AVPTPVVDPDAPPSPPL$_{283}$

HexNac(1)NeuAc(1)-

b				y
---	1	A	17	---
171.11	2	V	16	2092.02
268.17	3	P	15	1992.95
863.39	4	T(HexNac(1)NeuAc(1))	14	1895.9
960.44	5	P	13	1300.68
1059.51	6	V	12	1203.63
1158.58	7	V	11	1104.56
1273.6	8	D	10	1005.49
1370.66	9	P	9	890.46
1485.68	10	D	8	793.41
1556.72	11	A	7	678.38
1653.77	12	P	6	607.35
1750.83	13	P	5	510.29
1837.86	14	S	4	413.24
1934.91	15	P	3	326.21
2031.96	16	P	2	229.15
---	17	L	1	132.1

known O-glycosylation site

Alpha-2-HS-glycoprotein precursor

These fragment ions are not present in the MS³ peptide spectrum. This indicates that they are derived from glycopeptide fragmentation.

$_{267}$AVPTPVVDPDAPPSPPL$_{283}$

Fraction 17

872.72+++ → Pep [M+H]+ 1668.75+ [31.9-32.0 min]

CID-MS2 MASCOT Search

Internal glycopeptide fragmentation

$_{267}$AVP**T**PVVDPDAPPSPPL$_{283}$

HexNac(1)Hex(1)NeuAc(1)-

b				y
---	1	**A**	17	---
171.11	2	**V**	16	2254.07
268.17	3	**P**	15	2155.01
1025.44	4	**T(HexNac(1)Hex(1)NeuAc(1))**	14	2057.95
1122.49	5	**P**	13	1300.68
**1221.56**	6	**V**	12	1203.63
**1320.63**	7	**V**	11	1104.56
**1435.66**	8	**D**	10	1005.49
1532.71	9	**P**	9	890.46
1647.74	10	**D**	8	793.41
**1718.77**	11	**A**	7	678.38
1815.83	12	**P**	6	607.35
1912.88	13	**P**	5	510.29
**1999.91**	14	**S**	4	413.24
2096.96	15	**P**	3	326.21
2194.02	16	**P**	2	229.15
---	17	**L**	1	132.10

These fragment ions are not present in the MS³ peptide spectrum. This indicates that they are derived from glycopeptide fragmentation.

known O-glycosylation site

$_{267}$AVP**T**PVVDPDAPPSPPL$_{283}$

Alpha-2-HS-glycoprotein precursor

Fraction 17

872.72+++ → Pep [M+H]+ 1668.75+ [31.9-32.0 min]

CID-MS2 MASCOT Search

Internal glycopeptide fragmentation

$_{267}$AVP**T**PVVDPDAPPSPPL$_{283}$

HexNac(1)Hex(1)NeuAc(2)-

b				y
---	1	**A**	17	---
171.11	2	**V**	16	2545.17
268.17	3	**P**	15	2446.10
1316.54	4	**T(HexNAc(1)Hex(1)NeuAc(2))**	14	2349.05
1413.59	5	**P**	13	1300.68
1512.66	6	**V**	12	1203.63
**1611.73**	7	**V**	11	1104.56
**1726.75**	8	**D**	10	1005.49
1823.81	9	**P**	9	890.46
1938.83	10	**D**	8	793.41
**2009.87**	11	**A**	7	678.38
2106.92	12	**P**	6	607.35
2203.98	13	**P**	5	510.29
2291.01	14	**S**	4	413.24
2388.06	15	**P**	3	326.21
2485.11	16	**P**	2	229.15
---	17	**L**	1	132.10

These fragment ions are not present in the MS^3 peptide spectrum. This indicates that they are derived from glycopeptide fragmentation.

known O-glycosylation site

Alpha-2-HS-glycoprotein precursor

$_{267}$AVP**T**PVVDPDAPPSPPL$_{283}$

Fraction 17

872.72+++ → Pep [M+H]+ 1668.75+ [31.9-32.0 min]

CID-MS3

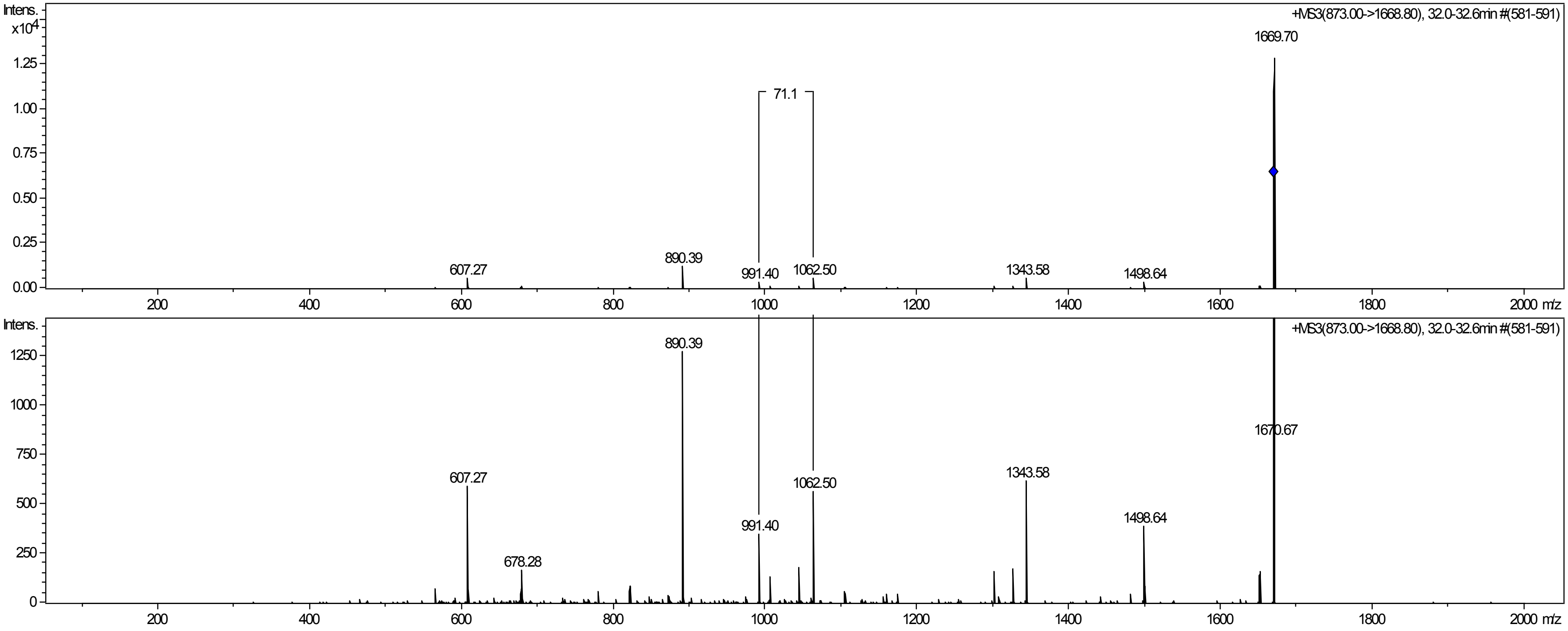

known O-glycosylation site

Alpha-2-HS-glycoprotein precursor

$_{267}$AVP**T**PVVDPDAPPSPPL$_{283}$

Fraction 17

872.72+++ → Pep [M+H]+ 1668.75+ [31.9-32.0 min]

CID-MS3

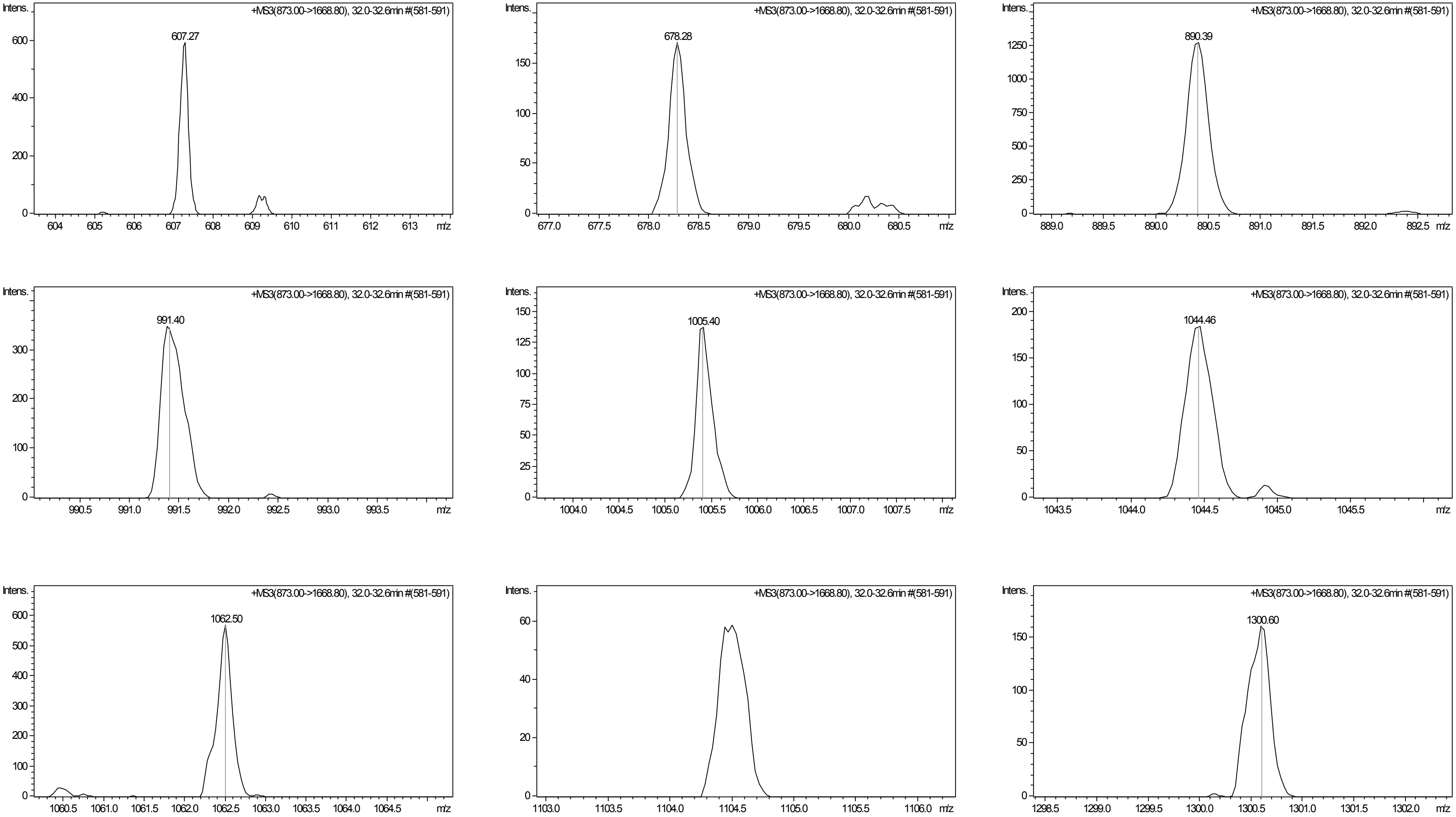

known O-glycosylation site

Alpha-2-HS-glycoprotein precursor

$_{267}$AVP**T**PVVDPDAPPSPPL$_{283}$

Fraction 17

872.72+++ → Pep [M+H]+ 1668.75+ [31.9-32.0 min]

CID-MS3

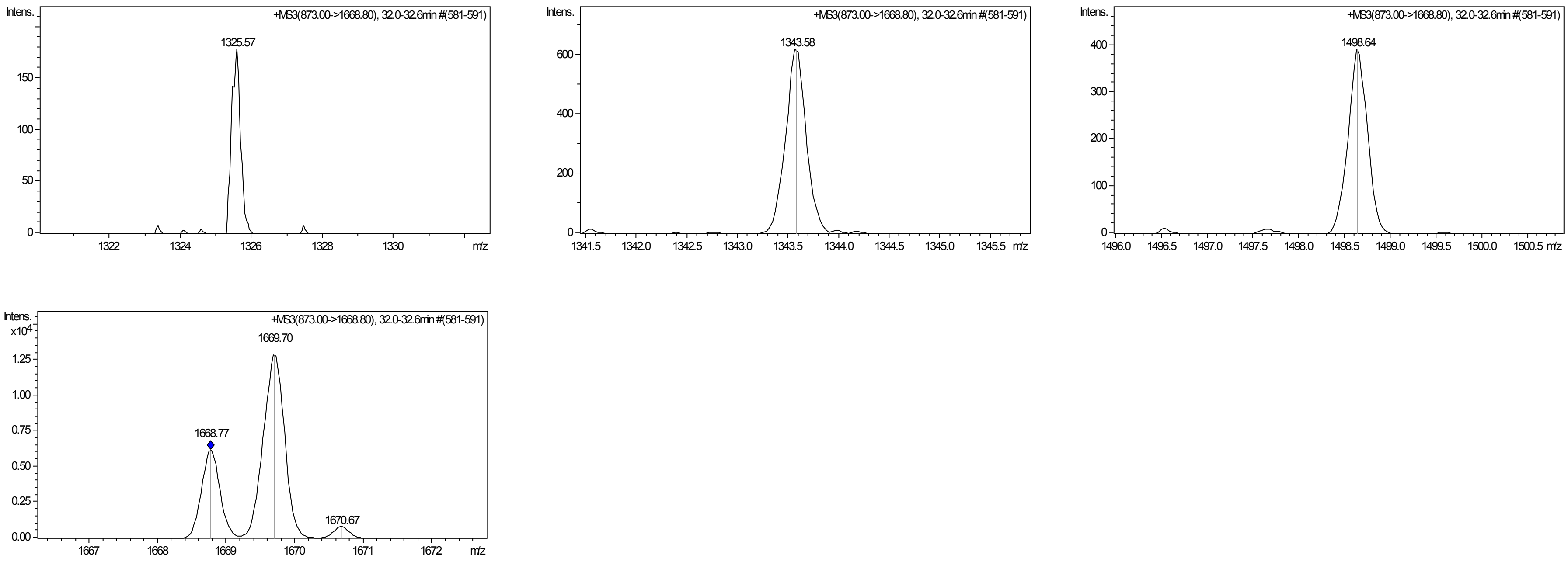

known O-glycosylation site

Alpha-2-HS-glycoprotein precursor

$_{267}$AVP**T**PVVDPDAPPSPPL$_{283}$

Fraction 17

872.72+++ → Pep [M+H]+ 1668.75+ [31.9-32.0 min]   CID-MS3 MASCOT Search

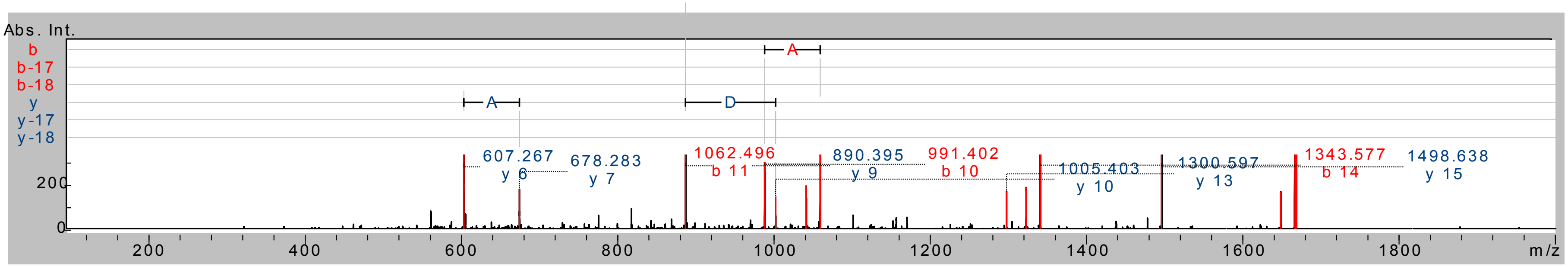

	A	V	P	T	P	V	V	D	P	D	A	P	P	S	P	P	L
Ion	1	2	3	4	5	6	7	8	9	10	11	12	13	14	15	16	17
b	A	V	P	T	P	V	V	D	P	D	A	P	P	S	P	P	L
b-17	A	V	P	T	P	V	V	D	P	D	A	P	P	S	P	P	L
b-18	A	V	P	T	P	V	V	D	P	D	A	P	P	S	P	P	L
y	A	V	P	T	P	V	V	D	P	D	A	P	P	S	P	P	L
y-17	A	V	P	T	P	V	V	D	P	D	A	P	P	S	P	P	L
y-18	A	V	P	T	P	V	V	D	P	D	A	P	P	S	P	P	L
	17	16	15	14	13	12	11	10	9	8	7	6	5	4	3	2	1

	Ala	Val	Pro	Thr	Pro	Val	Val	Asp	Pro	Asp	Ala	Pro	Pro	Ser	Pro	Pro	Leu
Ion	1	2	3	4	5	6	7	8	9	10	11	12	13	14	15	16	17
b	72.044	171.113	268.166	369.213	466.266	565.334	664.403	779.430	876.483	991.509	1062.547	1159.599	1256.652	1343.684	1440.737	1537.790	1650.874
b-17	-	-	-	-	-	-	-	-	-	-	-	-	-	-	-	-	-
b-18	-	-	-	351.203	448.255	547.324	646.392	761.419	858.472	973.499	1044.536	1141.589	1238.642	1325.674	1422.726	1519.779	1632.863
y	132.102	229.155	326.207	413.239	510.292	607.345	678.382	793.409	890.462	1005.489	1104.557	1203.626	1300.678	1401.726	1498.779	1597.847	1668.884
y-17	-	-	-	-	-	-	-	-	-	-	-	-	-	-	-	-	-
y-18	-	-	-	395.229	492.282	589.334	660.372	775.398	872.451	987.478	1086.547	1185.615	1282.668	1383.715	1480.768	1579.837	1650.874
	Leu	Pro	Pro	Ser	Pro	Pro	Ala	Asp	Pro	Asp	Val	Val	Pro	Thr	Pro	Val	Ala

known O-glycosylation site

Alpha-2-HS-glycoprotein precursor

$_{267}$AVP**T**PVVDPDAPPSPPL$_{283}$

Fraction 17

872.72+++ → Pep [M+H]+ 1668.75+ [31.9-32.0 min] CID-MS3 MASCOT Search

prot_hit_nur	prot_acc	prot_desc	prot_score	prot_mass	prot_matche	pep_query	pep_rank	pep_isbold	pep_exp_mz	pep_exp_mr	pep_exp_z	pep_calc_mr	pep_delta	pep_miss	pep_score	pep_expect	pep_res_bef	pep_seq
1	FETUA_HUM	Alpha-2-HS-g	53	40098	1	1	1	1	1668.7516	1667.7443	1	1667.8771	-0.1327	0	58.63	0.029	E	AVPTPVVDP
2	YIPF1_HUMA	Protein YIPF1	17	34426	1	1	4	0	1668.7516	1667.7443	1	1667.8745	-0.1302	0	21.72	1.40E+02	P	FWICATLVFA
3	AOC2_HUMA	Retina-speci	17	84077	1	1	2	0	1668.7516	1667.7443	1	1667.8043	-0.0599	0	23.92	84	S	QYSVQGNLV
4	MMP24_HUN	Matrix metal	13	73641	1	1	10	0	1668.7516	1667.7443	1	1667.8267	-0.0824	0	18.93	2.70E+02	T	LGNANHDGN
5	ABCG4_HUM	ATP-binding	13	72932	1	1	7	0	1668.7516	1667.7443	1	1667.7977	-0.0534	0	19.81	2.20E+02	E	VASGEYGDLN
6	TTC28_HUMA	Tetratricope	12	187184	1	1	5	0	1668.7516	1667.7443	1	1666.7733	0.971	0	21.22	1.60E+02	P	DHKQPQPGT
7	NCOA6_HUN	Nuclear rece	11	219350	1	1	6	0	1668.7516	1667.7443	1	1666.7443	1	0	20.9	1.70E+02	P	QMSNPGQFT
8	DYH11_HUM	Ciliary dynei	10	524811	1	1	7	0	1668.7516	1667.7443	1	1667.8916	-0.1473	0	19.81	2.20E+02	L	LGELPPGDRC
9	MUC16_HUM	Mucin-16 (O	6	2359682	1	1	9	0	1668.7516	1667.7443	1	1667.7672	-0.0229	0	19.22	2.50E+02	P	ATSSMEATSI

BioTools-Score: 10

MASCOT-Score: 59

known O-glycosylation site

Alpha-2-HS-glycoprotein precursor

$_{267}$AVP**T**PVVDPDAPPSPPL$_{283}$

Fraction 17

872.72+++ → Pep [M+H]+ 1668.75+ [31.9-32.0 min] ETD

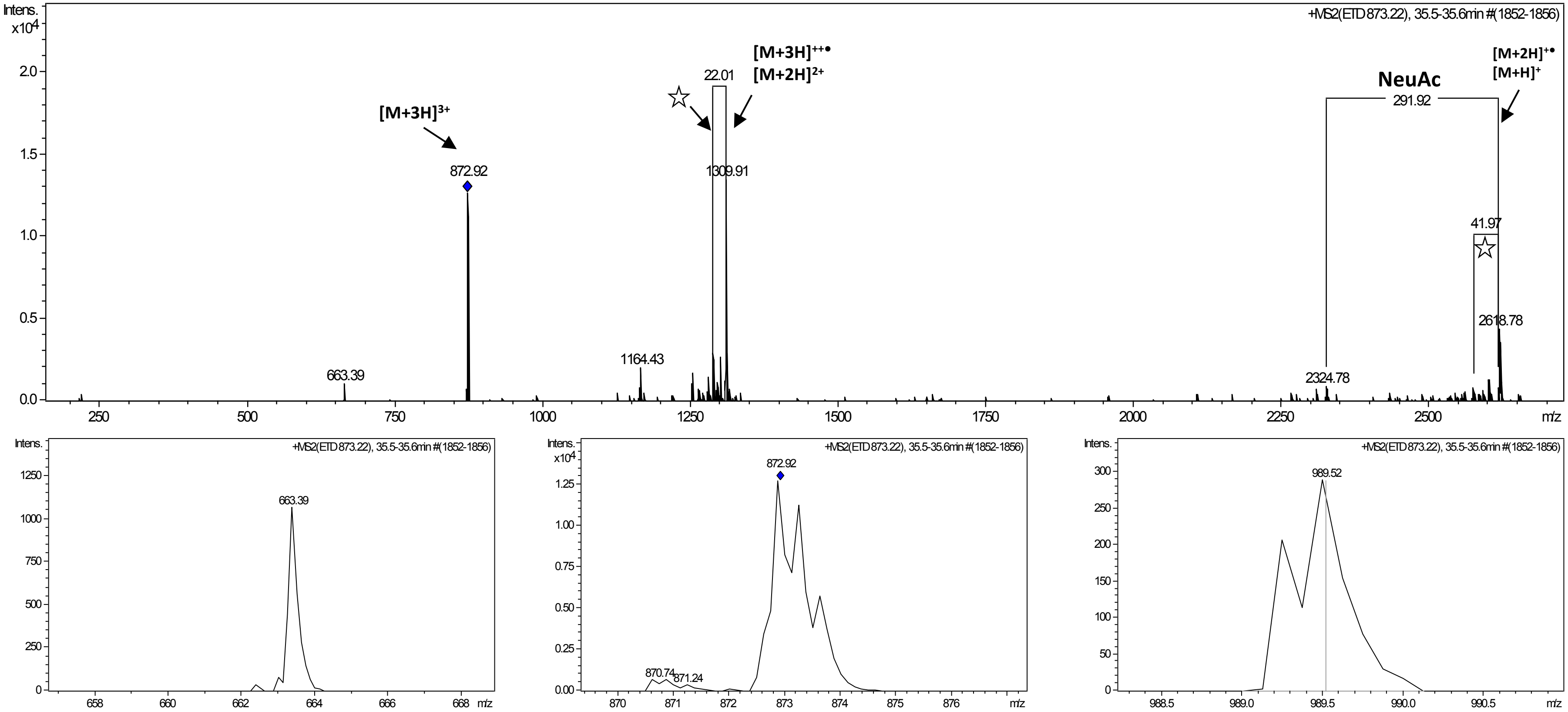

known O-glycosylation site

Alpha-2-HS-glycoprotein precursor

$_{267}$AVPTPVVDPDAPPSPPL$_{283}$

Fraction 17

872.72+++ → Pep [M+H]+ 1668.75+ [31.9-32.0 min]

ETD

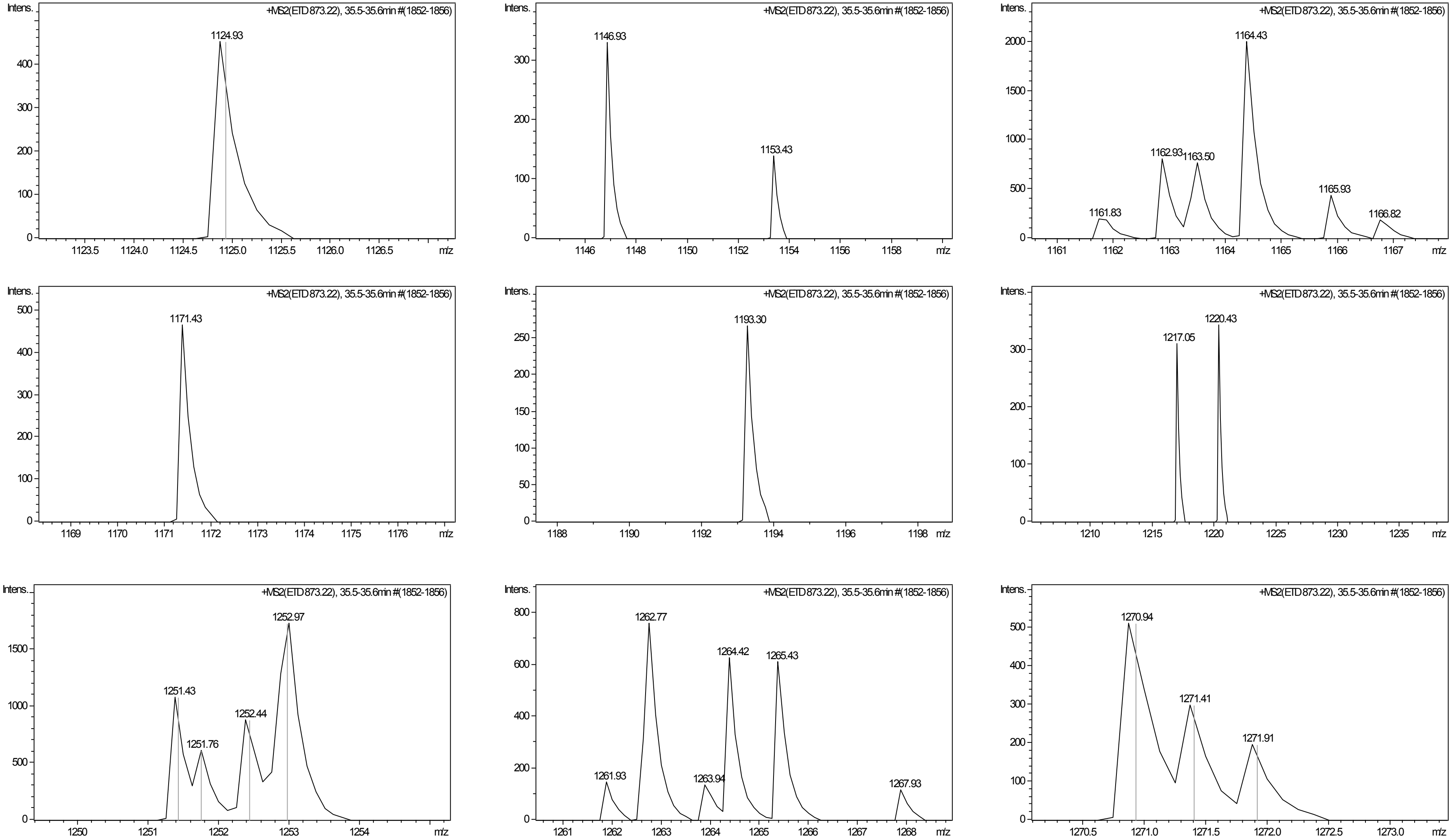

known O-glycosylation site
Alpha-2-HS-glycoprotein precursor

$_{267}$AVP**T**PVVDPDAPPSPPL$_{283}$

Fraction 17

872.72+++ → Pep [M+H]+ 1668.75+ [31.9-32.0 min]

ETD

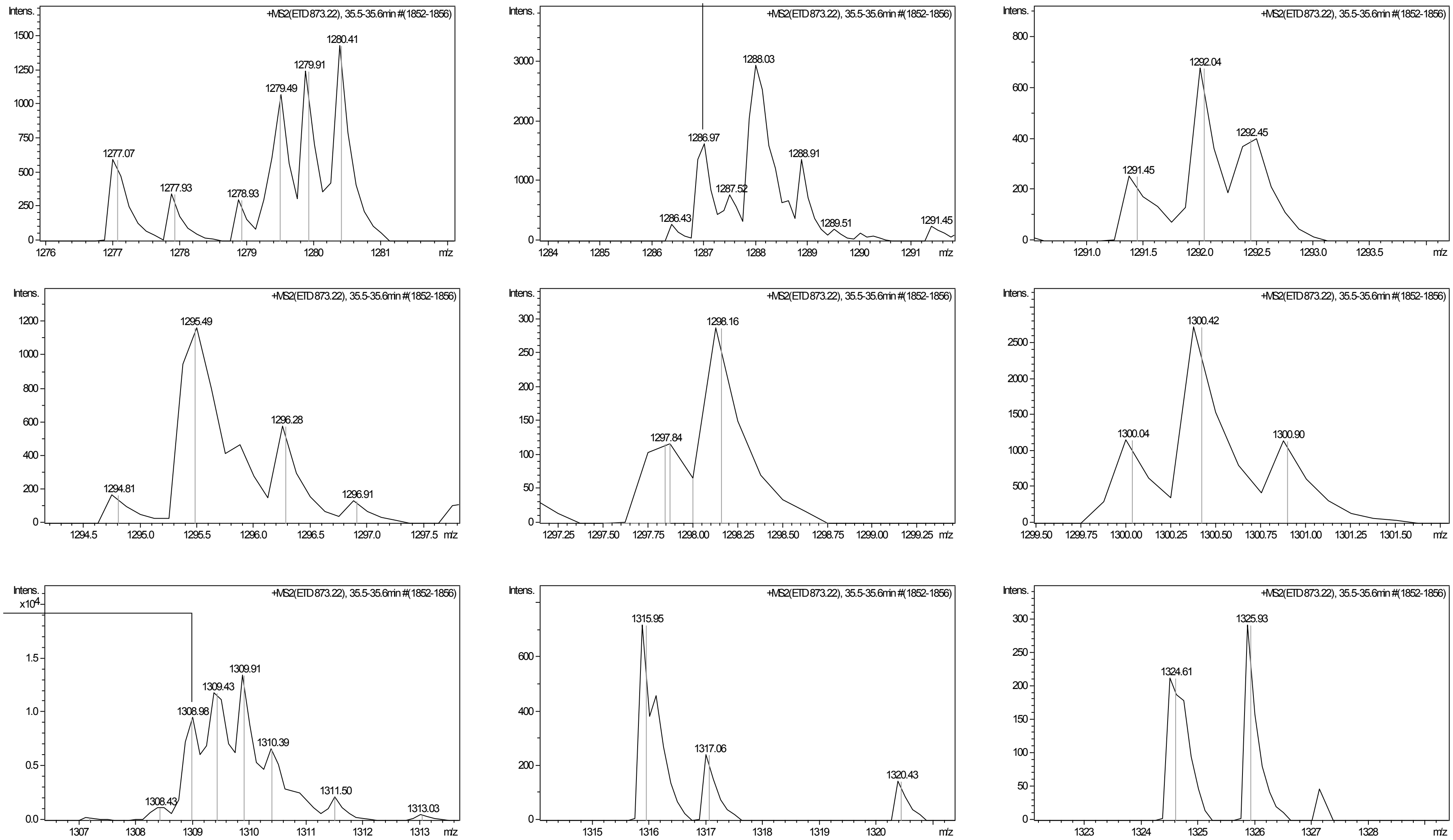

known O-glycosylation site
Alpha-2-HS-glycoprotein precursor

$_{267}$AVP**T**PVVDPDAPPSPPL$_{283}$

Fraction 17

872.72+++ → Pep [M+H]+ 1668.75+ [31.9-32.0 min]

ETD

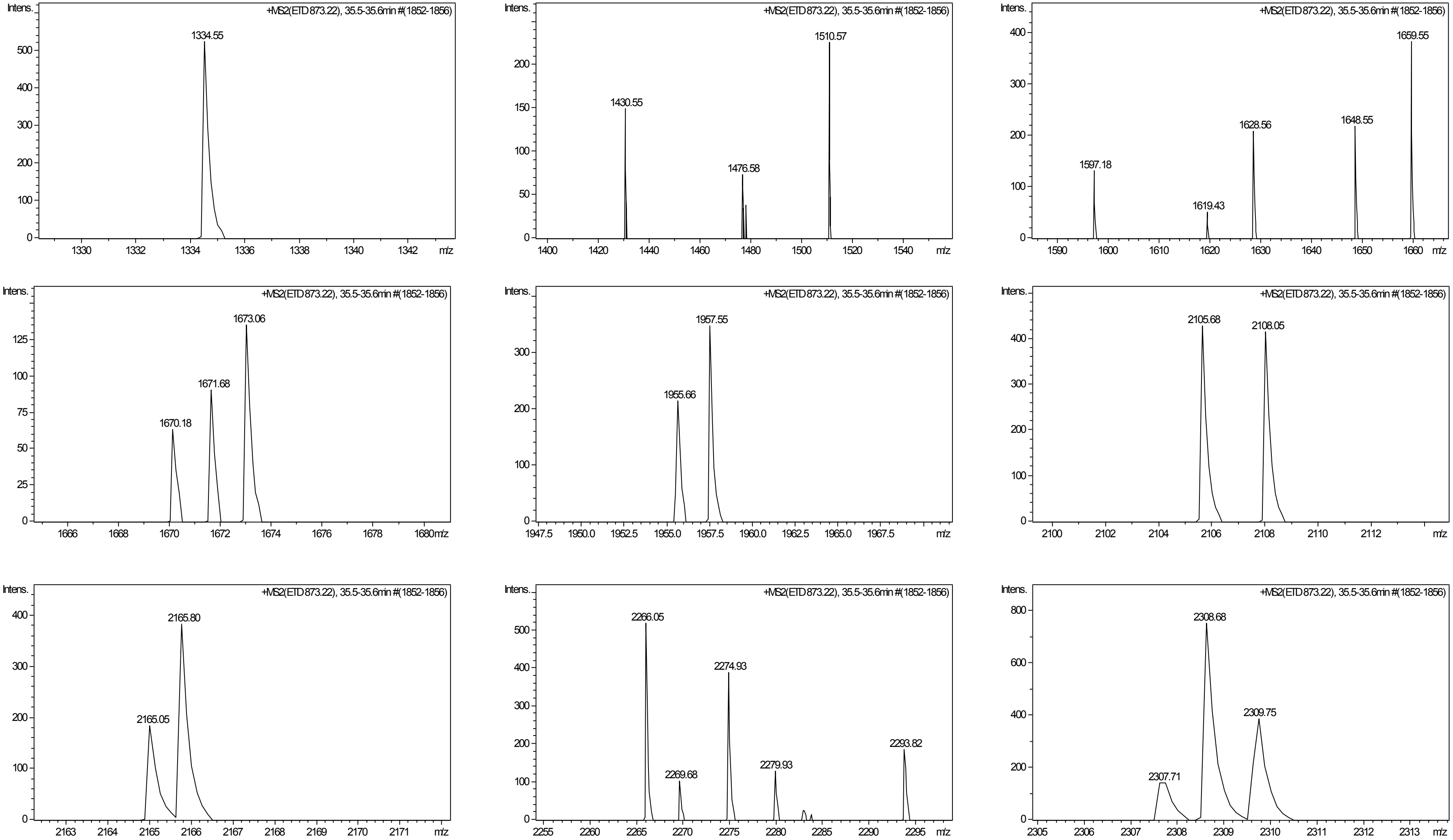

known O-glycosylation site

Alpha-2-HS-glycoprotein precursor

$_{267}$AVP**T**PVVDPDAPPSPPL$_{283}$

Fraction 17

872.72+++ → Pep [M+H]+ 1668.75+ [31.9-32.0 min]

ETD

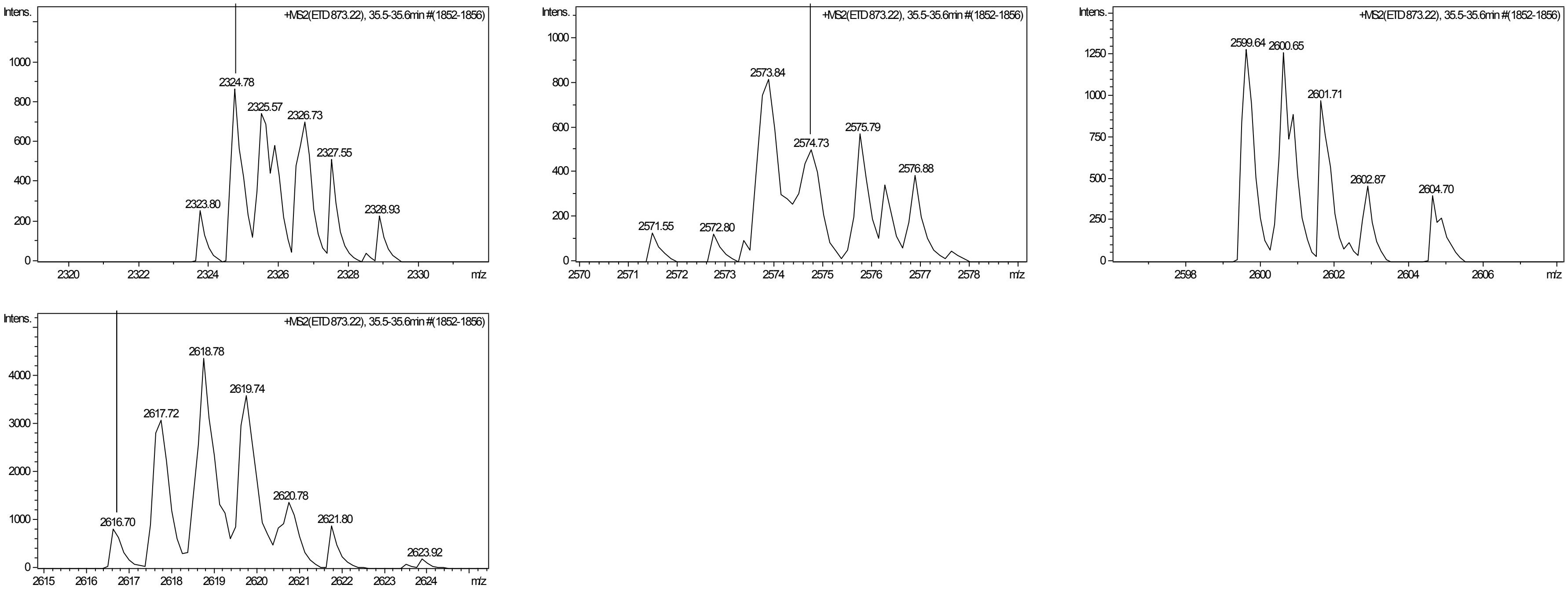

known O-glycosylation site

Alpha-2-HS-glycoprotein precursor

$_{267}$AVP**T**PVVDPDAPPSPPL$_{283}$

Fraction 17

872.72+++ → Pep [M+H]+ 1668.75+ [31.9-32.0 min] ETD

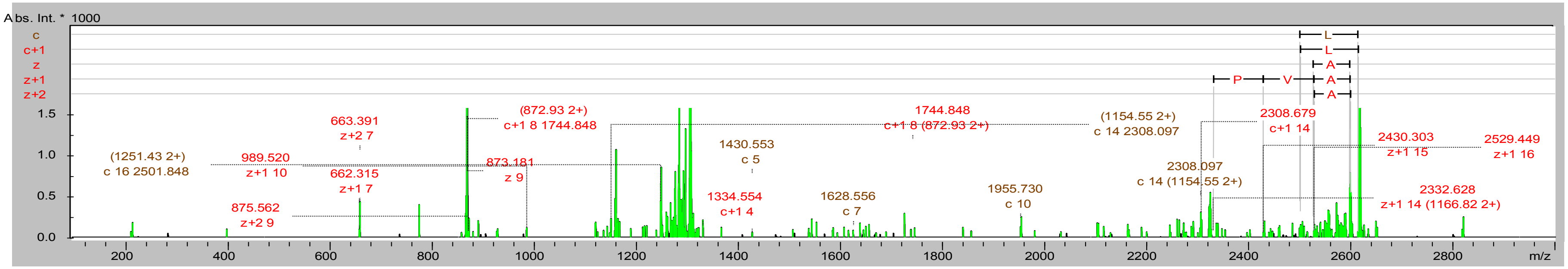

AVPTPVVDPDAPPSPPL

	A	V	P	T	P	V	V	D	P	D	A	P	P	S	P	P	L
Ion	1	2	3	4	5	6	7	8	9	10	11	12	13	14	15	16	17
c	A	V	P	T*	P	V	V	D	P	D	A	P	P	S	P	P	L
c+1	A	V	P	T*	P	V	V	D	P	D	A	P	P	S	P	P	L
z	A	V	P	T*	P	V	V	D	P	D	A	P	P	S	P	P	L
z+1	A	V	P	T*	P	V	V	D	P	D	A	P	P	S	P	P	L
z+2	A	V	P	T*	P	V	V	D	P	D	A	P	P	S	P	P	L
	17	16	15	14	13	12	11	10	9	8	7	6	5	4	3	2	1

	Ala	Val	Pro	Thr	Pro	Val	Val	Asp	Pro	Asp	Ala	Pro	Pro	Ser	Pro	Pro	Leu
Ion	1	2	3	4	5	6	7	8	9	10	11	12	13	14	15	16	17
c	89.071	188.139	285.192	1333.563	1430.616	1529.684	1628.752	1743.779	1840.832	1955.859	2026.896	2123.949	2221.002	2308.034	2405.086	2502.139	2615.223
c+1	90.079	189.147	286.200	1334.571	1431.623	1530.692	1629.760	1744.787	1841.840	1956.867	2027.904	2124.957	2222.010	2309.042	2406.094	2503.147	2616.231
z	115.075	212.128	309.181	396.213	493.266	590.318	661.356	776.382	873.435	988.462	1087.531	1186.599	1283.652	2332.023	2429.075	2528.144	2599.181
z+1	116.083	213.136	310.189	397.221	494.273	591.326	662.363	777.390	874.443	989.470	1088.538	1187.607	1284.660	2333.030	2430.083	2529.152	2600.189
z+2	117.091	214.144	311.197	398.229	495.281	592.334	663.371	778.398	875.451	990.478	1089.546	1188.615	1285.667	2334.038	2431.091	2530.159	2601.196
	Leu	Pro	Pro	Ser	Pro	Pro	Ala	Asp	Pro	Asp	Val	Val	Pro	Thr	Pro	Val	Ala

BioTools-Score: 39

known O-glycosylation site

Alpha-2-HS-glycoprotein precursor

$_{267}$AVPTPVVDPDAPPSPPL$_{283}$

Fraction 17

872.72+++ → Pep [M+H]+ 1668.75+ [31.9-32.0 min]

ETD

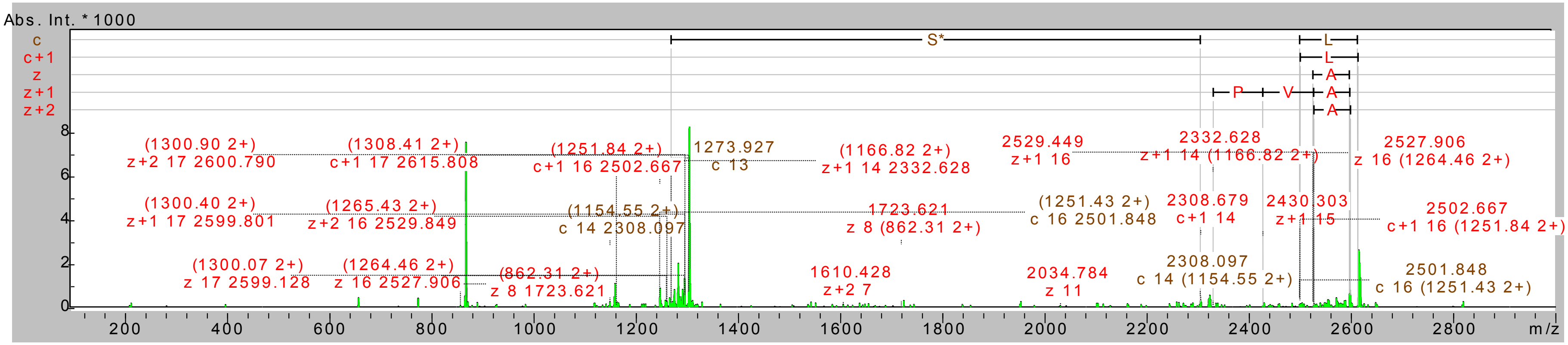

AVPTPVVDPDAPP**S**PPL

	A	V	P	T	P	V	V	D	P	D	A	P	P	S	P	P	L
Ion	1	2	3	4	5	6	7	8	9	10	11	12	13	14	15	16	17
c	A	V	P	T	P	V	V	D	P	D	A	P	P	S*	P	P	L
c+1	A	V	P	T	P	V	V	D	P	D	A	P	P	S*	P	P	L
z	A	V	P	T	P	V	V	D	P	D	A	P	P	S*	P	P	L
z+1	A	V	P	T	P	V	V	D	P	D	A	P	P	S*	P	P	L
z+2	A	V	P	T	P	V	V	D	P	D	A	P	P	S*	P	P	L
	17	16	15	14	13	12	11	10	9	8	7	6	5	4	3	2	1

	Ala	Val	Pro	Thr	Pro	Val	Val	Asp	Pro	Asp	Ala	Pro	Pro	Ser	Pro	Pro	Leu
Ion	1	2	3	4	5	6	7	8	9	10	11	12	13	14	15	16	17
c	89.071	188.139	285.192	386.240	483.293	582.361	681.429	796.456	893.509	1008.536	1079.573	1176.626	1273.679	2308.034	2405.086	2502.139	2615.223
c+1	90.079	189.147	286.200	387.248	484.300	583.369	682.437	797.464	894.517	1009.544	1080.581	1177.634	1274.687	2309.042	2406.094	2503.147	2616.231
z	115.075	212.128	309.181	1343.536	1440.589	1537.641	1608.679	1723.706	1820.758	1935.785	2034.854	2133.922	2230.975	2332.023	2429.075	2528.144	2599.181
z+1	116.083	213.136	310.189	1344.544	1441.597	1538.649	1609.686	1724.713	1821.766	1936.793	2035.861	2134.930	2231.983	2333.030	2430.083	2529.152	2600.189
z+2	117.091	214.144	311.197	1345.552	1442.604	1539.657	1610.694	1725.721	1822.774	1937.801	2036.869	2135.938	2232.990	2334.038	2431.091	2530.159	2601.196
	Leu	Pro	Pro	Ser	Pro	Pro	Ala	Asp	Pro	Asp	Val	Val	Pro	Thr	Pro	Val	Ala

BioTools-Score: 34

known O-glycosylation site

Alpha-2-HS-glycoprotein precursor

$_{267}$AVP**T**PVVDPDAPPSPPL$_{283}$

Fraction 17

915.71+++ → Pep+HexNac [M+H]+ 1000.91++ [32.0 min] CID-MS Precursor

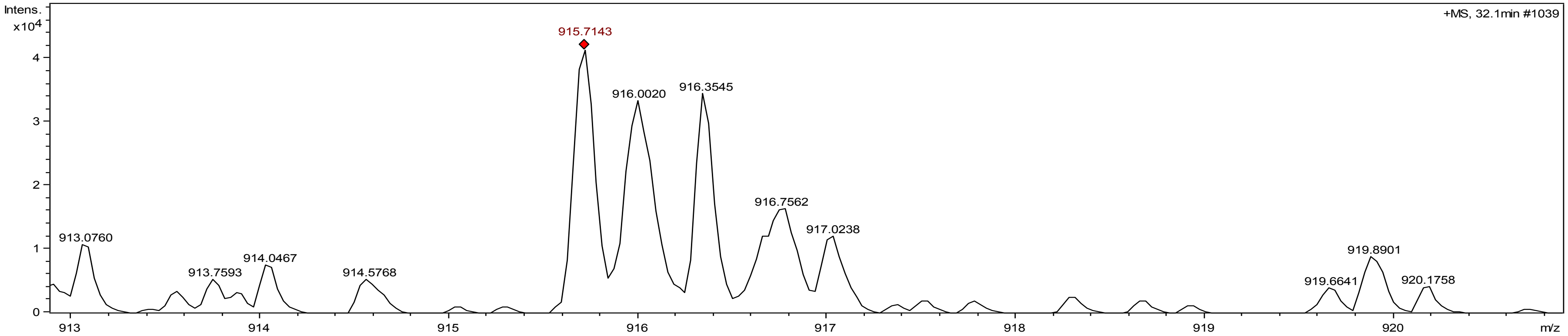

ETD spectrum of poor quality

Fraction 17

915.71+++ → Pep+HexNac [M+H]+ 1000.91++ [32.0 min] CID-MS2

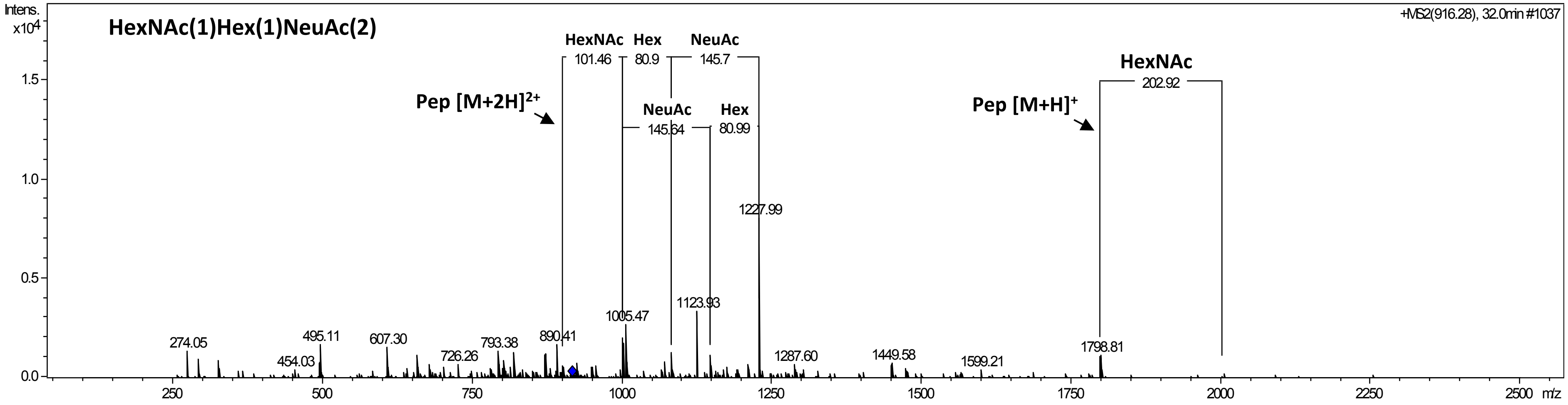

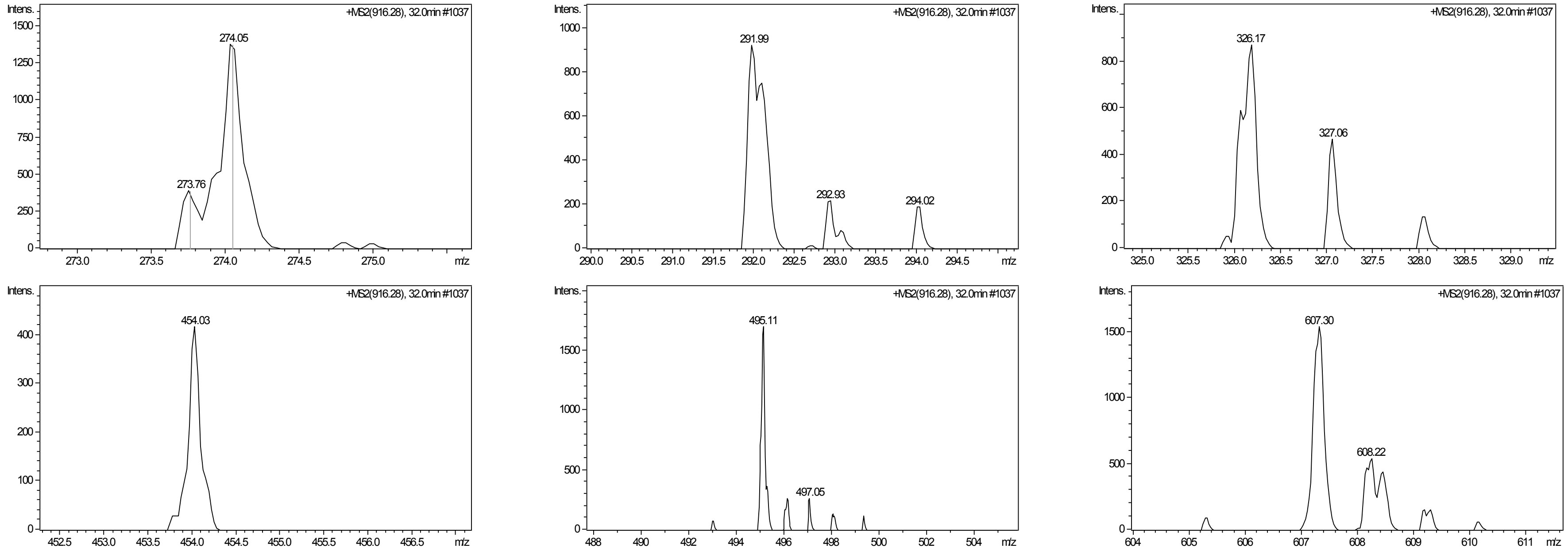

Fraction 17

915.71+++ → Pep+HexNac [M+H]+ 1000.91++ [32.0 min] CID-MS2

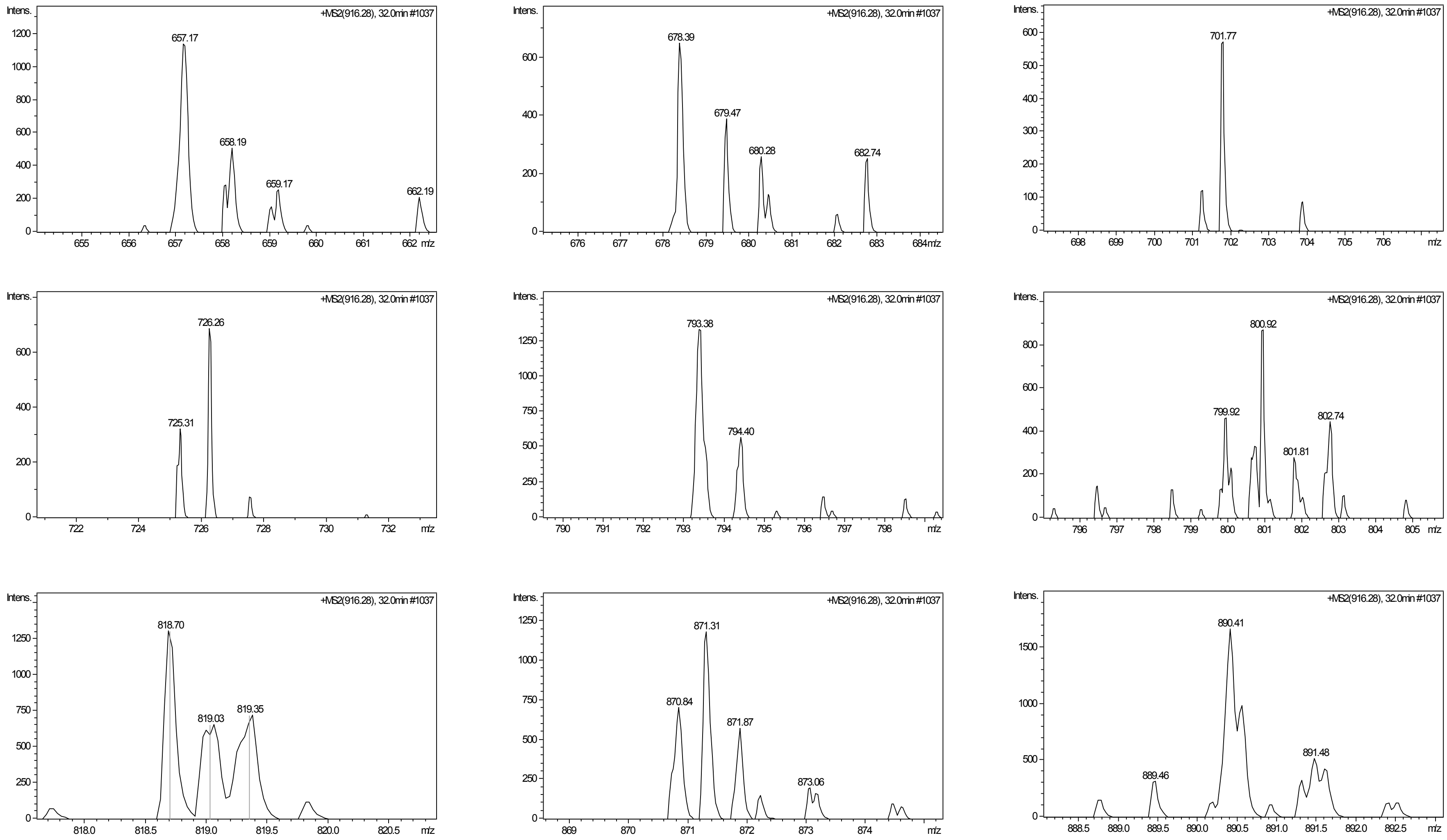

Fraction 17

915.71+++ → Pep+HexNac [M+H]+ 1000.91++ [32.0 min] CID-MS2

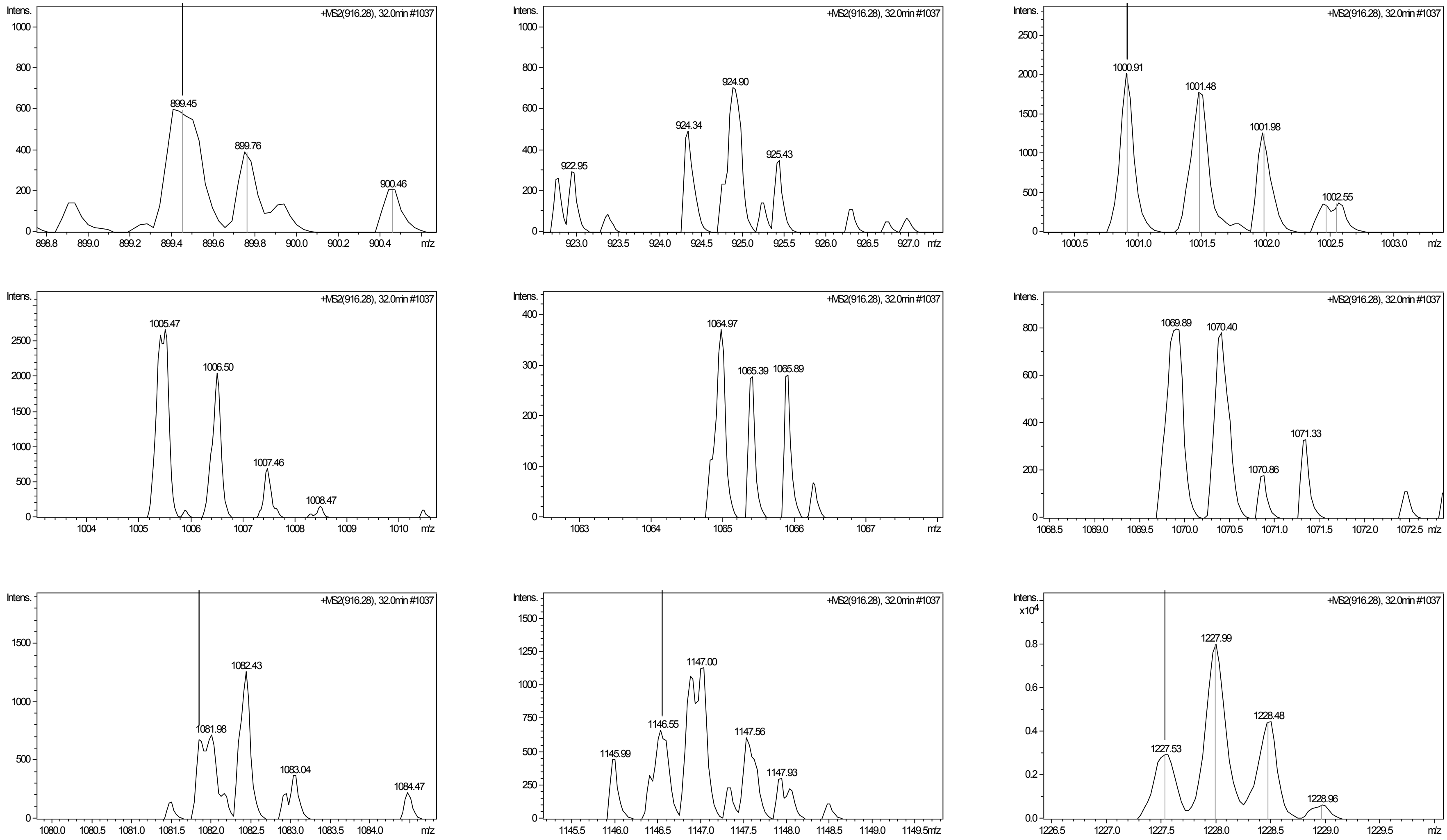

Fraction 17

915.71+++ → Pep+HexNac [M+H]+ 1000.91++ [32.0 min] CID-MS2

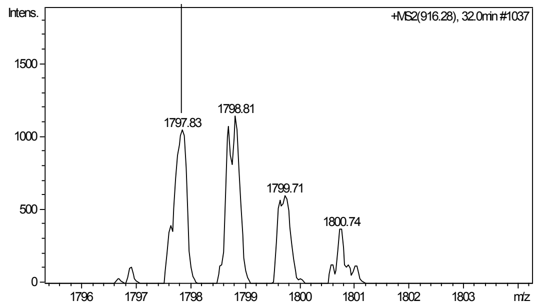

Fraction 17

915.71+++ → Pep+HexNac [M+H]+ 1000.91++ [32.0 min]

CID-MS2 MASCOT Search

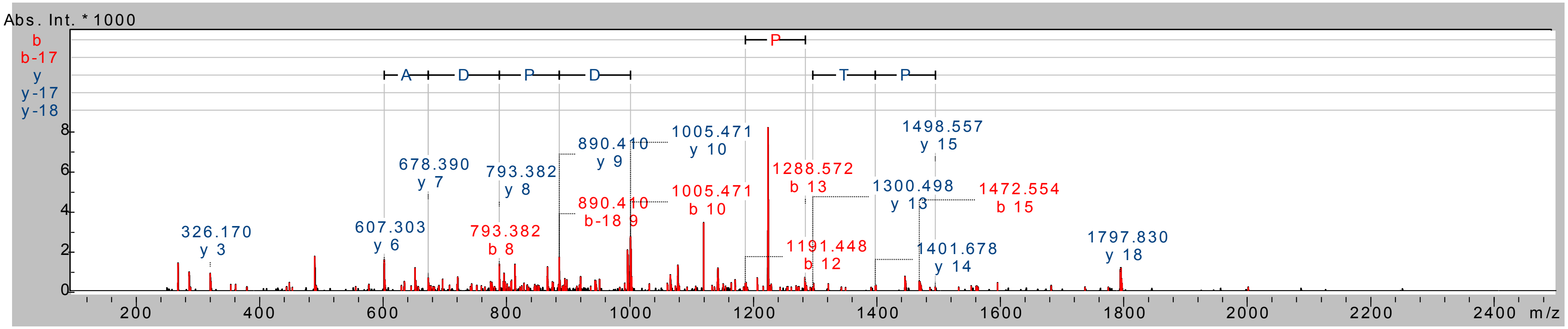

	E	A	V	P	T	P	V	V	D	P	D	A	P	P	S	P	P	L
Ion	1	2	3	4	5	6	7	8	9	10	11	12	13	14	15	16	17	18
b	E	A	V	P	T	P	V	V	D	P	D	A	P	P	S	P	P	L
b-17	E	A	V	P	T	P	V	V	D	P	D	A	P	P	S	P	P	L
b-18	E	A	V	P	T	P	V	V	D	P	D	A	P	P	S	P	P	L
y	E	A	V	P	T	P	V	V	D	P	D	A	P	P	S	P	P	L
y-17	E	A	V	P	T	P	V	V	D	P	D	A	P	P	S	P	P	L
y-18	E	A	V	P	T	P	V	V	D	P	D	A	P	P	S	P	P	L
	18	17	16	15	14	13	12	11	10	9	8	7	6	5	4	3	2	1

	Glu	Ala	Val	Pro	Thr	Pro	Val	Val	Asp	Pro	Asp	Ala	Pro	Pro	Ser	Pro	Pro	Leu
Ion	1	2	3	4	5	6	7	8	9	10	11	12	13	14	15	16	17	18
b	130.050	201.087	300.155	397.208	498.256	595.309	694.377	793.445	908.472	1005.525	1120.552	1191.589	1288.642	1385.695	1472.727	1569.780	1666.832	1779.916
b-17	-	-	-	-	-	-	-	-	-	-	-	-	-	-	-	-	-	-
b-18	112.039	183.076	282.145	379.198	480.245	577.298	676.366	775.435	890.462	987.515	1102.542	1173.579	1270.631	1367.684	1454.716	1551.769	1648.822	1761.906
y	132.102	229.155	326.207	413.239	510.292	607.345	678.382	793.409	890.462	1005.489	1104.557	1203.626	1300.678	1401.726	1498.779	1597.847	1668.884	1797.927
y-17	-	-	-	-	-	-	-	-	-	-	-	-	-	-	-	-	-	-
y-18	-	-	-	395.229	492.282	589.334	660.372	775.398	872.451	987.478	1086.547	1185.615	1282.668	1383.715	1480.768	1579.837	1650.874	1779.916
	Leu	Pro	Pro	Ser	Pro	Pro	Ala	Asp	Pro	Asp	Val	Val	Pro	Thr	Pro	Val	Ala	Glu

For MASCOT search m/z of the unmodified peptide [M+H]$^+$ has to be given

known O-glycosylation site

Alpha-2-HS-glycoprotein precursor

$_{266}$EAVP**T**PVVDPDAPPSPPL$_{283}$

Fraction 17

915.71+++ → Pep+HexNac [M+H]+ 1000.91++ [32.0 min]

CID-MS2 MASCOT Search

prot_hit_nur	prot_acc	prot_desc	prot_score	prot_mass	prot	prot	prot	prot	pep	pep	pep	pep	pep_exp_mz	pep_exp_mr	pep_exp_z	pep_calc_mr	pep_delta	pep	pep_score	pep_expect	pep_res_bef
1	FETUA_HUMAN		0	40098	1	0	1	0	1	5	1	1	1797,83	1796,8227	1	1796,9196	-0,0969	0	4,04	1,10E+04	N
2	ROCK1_HUMAN		0	159102	1	0	1	0	1	3	0	1	1797,83	1796,8227	1	1796,7925	0,0302	0	4,93	9,30E+03	R
3	OR2B8_HUMAN		0	35509	1	0	1	0	1	6	0	1	1797,83	1796,8227	1	1796,8217	0,001	0	2	1,80E+04	L
4	TRIM2_HUMAN		0	82506	1	0	1	0	1	7	0	1	1797,83	1796,8227	1	1796,7853	0,0374	0	0,98	2,30E+04	M
5	MAGD4_HUMAN		0	81555	1	0	1	0	1	7	0	1	1797,83	1796,8227	1	1796,8468	-0,0241	0	0,98	2,30E+04	T
6	CD19_HUMA	B-lymphocyt	0	61546	1	0	1	0	1	1	0	1	1797,83	1796,8227	1	1796,5955	0,2272	0	5,52	8,10E+03	G
7	MMS19_HUMAN		0	114928	1	0	1	0	1	4	0	1	1797,83	1796,8227	1	1796,7808	0,0419	0	4,1	1,10E+04	R
8	CD021_HUMAN		0	238905	1	0	1	0	1	2	0	1	1797,83	1796,8227	1	1795,8013	1,0215	0	5,14	8,80E+03	R
9	RNF43_HUMAN		0	87150	1	0	1	0	1	9	0	1	1797,83	1796,8227	1	1795,7869	1,0358	0	0,67	2,50E+04	L

BioTools-Score: 45

MASCOT-Score: 4

known O-glycosylation site

Alpha-2-HS-glycoprotein precursor

$_{266}$EAVP**T**PVVDPDAPPSPPL$_{283}$

Fraction 17

915.71+++ → Pep+HexNac [M+H]+ 1000.91++ [32.0 min]

CID-MS3

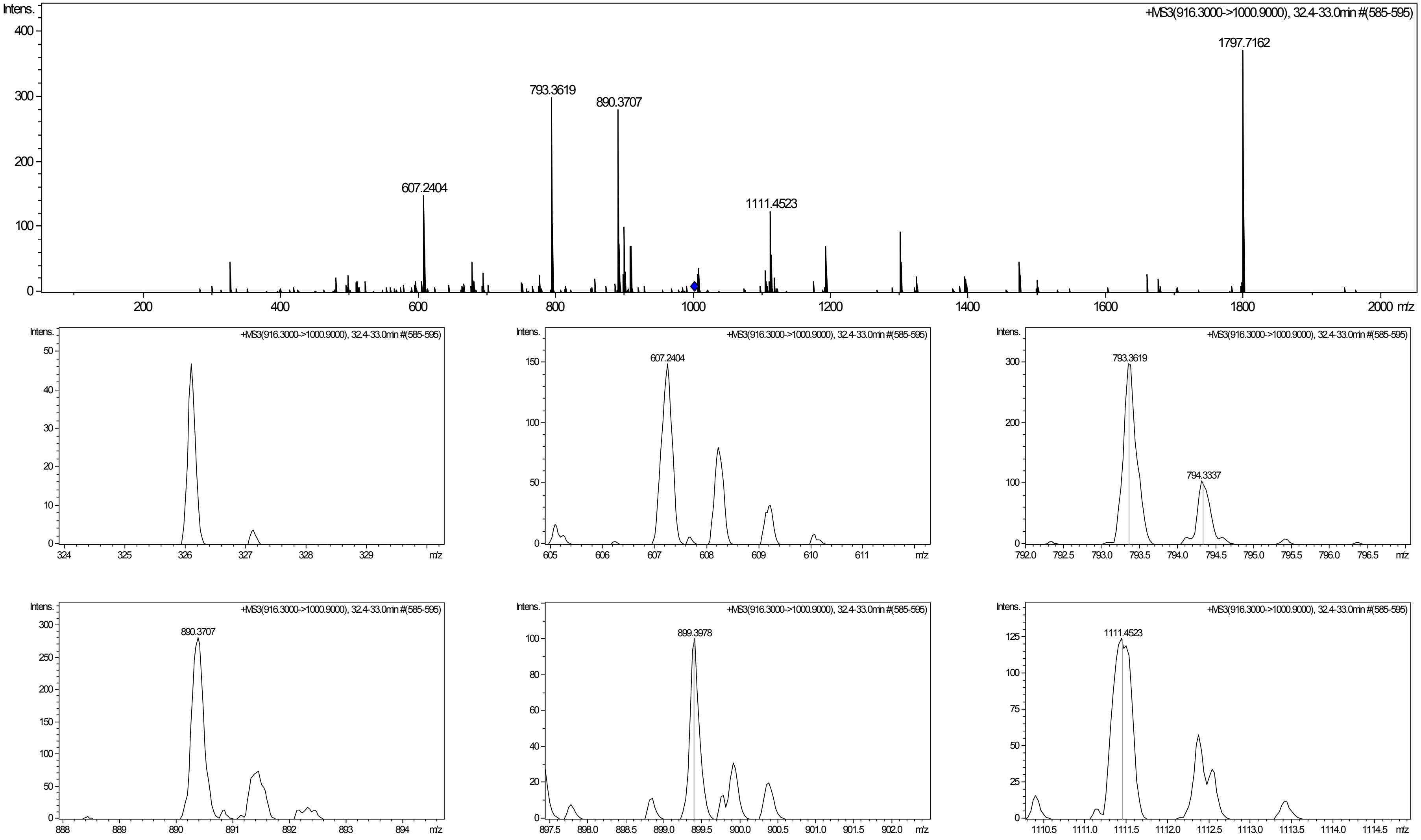

known O-glycosylation site
Alpha-2-HS-glycoprotein precursor

$_{266}$EAVP**T**PVVDPDAPPSPPL$_{283}$

Fraction 17

915.71+++ → Pep+HexNac [M+H]+ 1000.91++ [32.0 min]

CID-MS3

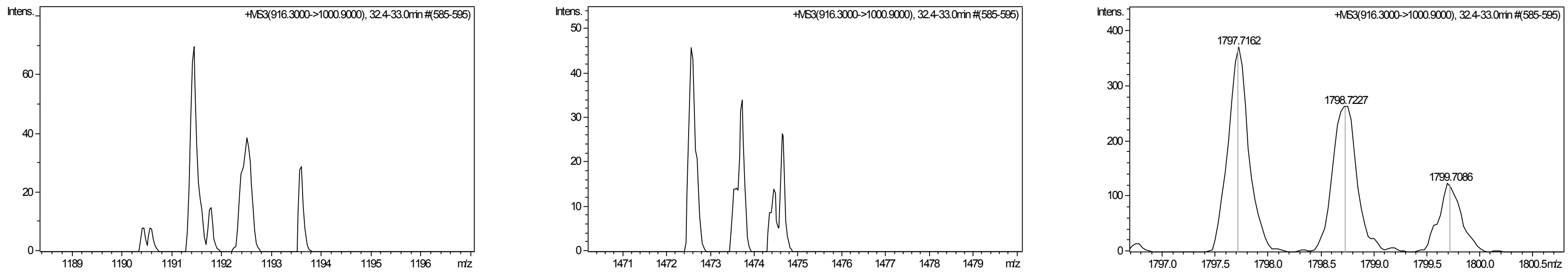

known O-glycosylation site
Alpha-2-HS-glycoprotein precursor

$_{266}$EAVP**T**PVVDPDAPPSPPL$_{283}$

Fraction 17

915.71+++ → Pep+HexNac [M+H]+ 1000.91++ [32.0 min]   CID-MS3 MASCOT Search

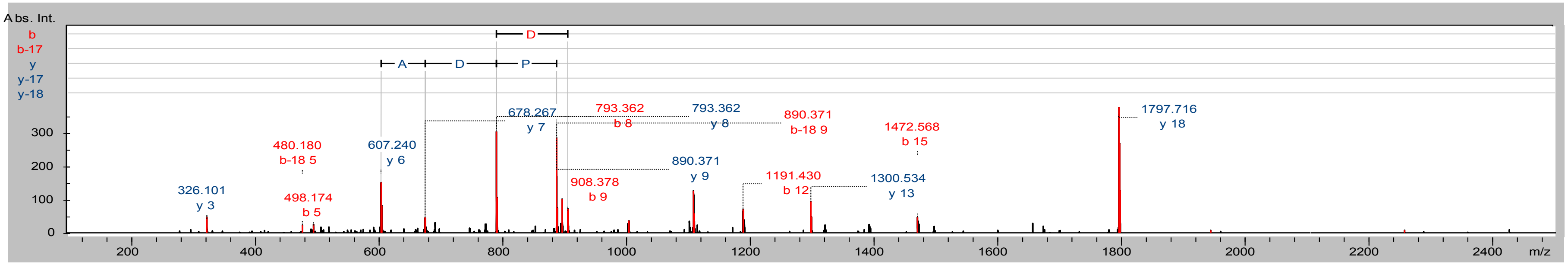

	E	A	V	P	T	P	V	V	D	P	D	A	P	P	S	P	P	L	Glu	Ala	Val	Pro	Thr	Pro	Val	Val	Asp	Pro	Asp	Ala	Pro	Pro	Ser	Pro	Pro	Leu
Ion	1	2	3	4	5	6	7	8	9	10	11	12	13	14	15	16	17	18	1	2	3	4	5	6	7	8	9	10	11	12	13	14	15	16	17	18
b	E	A	V	P	T	P	V	V	D	P	D	A	P	P	S	P	P	L	130.050	201.087	300.155	397.208	498.256	595.309	694.377	793.445	908.472	1005.525	1120.552	1191.589	1288.642	1385.695	1472.727	1569.780	1666.832	1779.916
b-17	E	A	V	P	T	P	V	V	D	P	D	A	P	P	S	P	P	L	-	-	-	-	-	-	-	-	-	-	-	-	-	-	-	-	-	-
b-18	E	A	V	P	T	P	V	V	D	P	D	A	P	P	S	P	P	L	112.039	183.076	282.145	379.198	480.245	577.298	676.366	775.435	890.462	987.515	1102.542	1173.579	1270.631	1367.684	1454.716	1551.769	1648.822	1761.906
y	E	A	V	P	T	P	V	V	D	P	D	A	P	P	S	P	P	L	132.102	229.155	326.207	413.239	510.292	607.345	678.382	793.409	890.462	1005.489	1104.557	1203.626	1300.678	1401.726	1498.779	1597.847	1668.884	1797.927
y-17	E	A	V	P	T	P	V	V	D	P	D	A	P	P	S	P	P	L	-	-	-	-	-	-	-	-	-	-	-	-	-	-	-	-	-	-
y-18	E	A	V	P	T	P	V	V	D	P	D	A	P	P	S	P	P	L	-	-	-	395.229	492.282	589.334	660.372	775.398	872.451	987.478	1086.547	1185.615	1282.668	1383.715	1480.768	1579.837	1650.874	1779.916
	18	17	16	15	14	13	12	11	10	9	8	7	6	5	4	3	2	1	Leu	Pro	Pro	Ser	Pro	Pro	Ala	Asp	Pro	Asp	Val	Val	Pro	Thr	Pro	Val	Ala	Glu

known O-glycosylation site

Alpha-2-HS-glycoprotein precursor

$_{266}$EAVP**T**PVVDPDAPPSPPL$_{283}$

Fraction 17

915.71+++ → Pep+HexNac [M+H]+ 1000.91++ [32.0 min]

CID-MS3 MASCOT Search

prot_hit_nur	prot_acc	prot_desc	prot_score	prot_mass	prot_matche	pep_query	pep_rank	pep_isbold	pep_exp_mz	pep_exp_mr	pep_exp_z	pep_calc_mr	pep_delta	pep_miss	pep_score	pep_expect	pep_res_bef	pep_seq
1	FETUA_HUM	Alpha-2-HS-g	41	40098	1	1	1	1	1797.7162	1796.7089	1	1796.9196	-0.2107	0	46.12	0.5	N	EAVPTPVVDI
2	TACD2_HUM	Tumor-assoc	14	36371	1	1	2	0	1797.7162	1796.7089	1	1796.8329	-0.124	0	18.12	3.20E+02	V	RPSEHALVDN
3	MMS19_HUN	MMS19-like	10	114914	1	1	2	0	1797.7162	1796.7089	1	1796.7808	-0.0719	0	18.12	3.20E+02	R	GNMVAQSSD
4	ROCK1_HUM	Rho-associat	9	159102	1	1	4	0	1797.7162	1796.7089	1	1796.7925	-0.0836	0	17.39	3.80E+02	R	RYLSSANPND
5	ACBD4_HUM	Acyl-CoA-bir	9	30972	1	1	9	0	1797.7162	1796.7089	1	1795.8815	0.8275	0	14.12	8.00E+02	F	EPLYQVIPDM
6	ARHG4_HUN	Rho guanine	9	76159	1	1	8	0	1797.7162	1796.7089	1	1795.6665	1.0424	0	14.87	6.70E+02	Y	SEYCNNHPN

BioTools-Score: 23

MASCOT-Score: 46

known O-glycosylation site

Alpha-2-HS-glycoprotein precursor

$_{266}$EAVP**T**PVVDPDAPPSPPL$_{283}$
